# Transcriptome and proteome combined analysis of wool fiber diameter regulation mechanism

**DOI:** 10.5713/ab.25.0378

**Published:** 2025-09-30

**Authors:** Lin Yue, Zengkui Lu, Tingting Guo, Bowen Chen, Jianbin Liu, Chao Yuan

**Affiliations:** 1 Lanzhou Institute of Husbandry and Pharmaceutical Sciences, Chinese Academy of Agricultural Sciences, Lanzhou, China; 2 Sheep Breeding Engineering Technology Research Center of Chinese Academy of Agricultural Sciences, Lanzhou, China; 3 Key Laboratory of Animal Genetics and Breeding on Tibetan Plateau, Ministry of Agriculture and Rural Affairs, Lanzhou, China

**Keywords:** Conjoint Analysis, Proteomics, Whole Transcriptome, Wool Fiber Diameter

## Abstract

**Objective:**

The diameter of wool fiber is a crucial phenotypic trait and a key determinant affecting its economic value. Understanding the regulatory mechanisms that influence wool fiber diameter is a fundamental approach to optimizing wool fineness.

**Methods:**

This study involved the selection of fine-wool Alpine Merino sheep with three distinct fiber diameter ranges for detailed whole-transcriptome and proteome analyses of skin tissues.

**Results:**

This led to the identification of key microRNAs (oar-miR-23a, oar-miR-23b, oar-miR-150, and oar-miR-218a), critical circular RNAs (circRNA3051, circRNA0315, and circRNA_1477), and multiple pivotal genes (*LOC10112037*, *LOC105614079*, *IGFBP1*, *IGFBP4*, and *MAPK9*). Correlation analysis was utilized to develop a comprehensive regulatory network, revealing a close regulation of wool fiber diameter and both energy metabolism and lipid metabolism.

**Conclusion:**

This study found that the triglyceride and energy metabolic pathways as significant factors influencing of wool fiber diameter, thus offering a theoretical basis for promoting wool industry through the refinement of wool diameter and quality.

## INTRODUCTION

Wool was one of the earliest natural fibers to be utilized by humans, possessing numerous excellent properties. Notably, Merino sheep, renowned for their high-quality wool characterized by exceptionally fine fibers, have become a primary source of raw material for many premium textiles. Therefore, making the analysis of regulatory mechanisms governing wool fiber diameter crucial for the high-quality development of the wool industry. Zhang et al [1] selected ultra-fine-haired Merino sheep and coarse-haired small-tail Han sheep breeds for skin transcriptome analyses, discovering high expression of keratin-related genes and hair follicle stem cell marker genes in the Merino sheep. Ma et al [2] compared the skin transcriptomes of Chinese Merino sheep and Super Merino sheep, identifying several differentially expressed genes (DEGs) and speculating that the *COL1A1* and *LOC101116863* genes play significant roles in the regulation of wool fineness. He et al [3] analyzed the mRNA data from Merino sheep skin tissues collected at the embryonic stage and 2 days after birth, identifying *LAMAS*, *WNT10A*, and *KRT25* as key genes influencing the morphological development of hair follicles. Subsequently, Pu et al [4] employed Weighted Gene Co-expression Network Analysis (WGCNA) to integrate transcriptome data from Ordos fine-wool sheep skin with coarse-wool and fine-wool skin data from other species. Their findings indicate that the mechanisms regulating wool fineness in different sheep breeds are highly similar, however, the analysis focused solely on the regulatory mechanisms of wool fineness from a transcriptomic perspective.

Total transcriptomics offers comprehensive data on microRNA (miRNA), long noncoding RNA (lncRNA), circular RNA (circRNA), and mRNA, encompassing most of the RNA types involved in phenotypic regulation, with the objective to develop a deeper biological insight. Fu et al [5] previously conducted a joint analysis of lncRNA and mRNA, discovering that specific lncRNAs may regulate the quality of Tibetan cashmere through target genes. Wang et al [6] utilized lncRNA and miRNA sequencing data to investigate the cyclical development of cashmere goat hair follicles, constructing a competing endogenous RNA (ceRNA) network of lncRNAs and miRNAs to elucidate the underlying mechanisms. Some scholars have integrated lncRNA, miRNA, and mRNA sequencing data to analyze the dynamic changes in these three RNA types during the development of Aohan fine wool sheep follicles, identifying an RNA regulatory network primarily governed by miR-21 [7]. Additionally, some researchers have employed multiomics approaches to investigate the regulatory mechanisms underlying hair fineness. For instance, using transcriptomics and proteomics, Zhao et al [8] identified key genes and their encoded proteins in the nuclear family YB, high mobility group, and cold shock domain families that may regulate the fineness of cashmere in Tibetan cashmere goats. This method has also been employed to investigate the regulatory mechanisms underlying rabbit hair thickness, resulting in the identification of several valuable proteins, including keratin intermediate filament (KRT)77 and KRT82 [9]. Researchers have also ingeniously merged the methylome with the whole transcriptome to investigate the developmental mechanism of Merino sheep hair follicles, obtaining the RNA expression profile of the hair follicle development stage and elucidating the intricate interplay between methylation modifications. Furthermore, their use of genome-wide association studies enabled a systematic analysis of the relationship between genes expressed at specific stages of hair follicle development and wool traits [10]. Such findings demonstrate the superiority of multiomics analysis to investigate biological mechanisms.

The heritability of wool fiber diameter is classified as medium to high. A significant number of scholars have used GWAS to identify a large number of SNP loci related to wool fiber diameter, offering a reference for understanding the genetic mechanisms underlying this trait [11,12]. The Alpine Merino sheep selected for this study display the excellent wool characteristics of traditional Merino sheep alongside the cold resistance, drought tolerance, and adaptability to rough feeding traits of local breeds [13]. Here, we established three distinct gradients of wool fiber diameter and integrated whole transcriptome analysis with proteomics to comprehensively elucidate the regulatory mechanisms governing wool fiber diameter from both epigenetic and molecular genetic perspectives. The study also analyzed the biological mechanisms involved in regulating wool fiber diameter, providing theoretical guidance for fine-wool sheep breeding and ensuring a high-quality developmental trajectory for the fine-wool sheep industry.

## MATERIALS AND METHODS

### Experimental animals and traits

The Alpine Merino sheep used in this experiment originated from the Gansu Sheep Breeding and Promotion Station. All experimental animals were adult ewes from the same population. According to the difference of wool fib re diameter, coefficient of variation (CV), and mean fibre diameter (MFD): SF group (MFD = 17.68±0.26 μm, CV<20%), EF group (MFD = 19.15±0.37 μm, CV<22%), and M group (MFD = 22.51±0.43 μm, CV<23.6%). Comparisons between the EF and SF (EF–SF), M and EF (M–EF), and M and SF (M–SF) groups were set up by collecting skin tissue from the left shoulders of three sheep from each group. The skin tissue samples (collection area: ~ 2 cm×3 cm) were placed in a freezing tube, immediately frozen in liquid nitrogen, and stored at −80°C.

### RNA extraction and sequencing

Total RNA was extracted using the TRIzol reagent (Invitrogen) in accordance with the manufacturer’s protocol. RNA purity and quantification were evaluated using the NanoDrop 2000 spectrophotometer (Thermo Fisher Scientific). RNA integrity was assessed using the Agilent 2100 Bioanalyzer (Agilent). The libraries were sequenced on an illumina Novaseq 6000 platform.

### Analysis of mRNA and micro RNA

The fastp tool was used to process raw reads and obtain clean reads for each sample [14]. Which were retained for subsequent analyses. The clean reads were mapped to the reference genome (Oar_v4.0) using HISAT2 [15]. The fragments per kilobase of transcript per million mapped fragments (FPKM) [16] of each gene were calculated, and the read counts for each gene were obtained using HTSeq-count [17]. Differential expression analysis was performed using the DESeq2 tool [18]. A p value<0.05 and a fold change (|FC|)>1.5 were set as the thresholds for identifying significant DEGs. Using the hypergeometric distribution to analyze GO and KEGG significantly enriched items, and screen for a threshold of p<0.05.

Small RNA libraries were constructed, purified, and sequenced using the NEBNext Small RNA Library Prep Set for Illumina kit (NEB#E7330S; NEB). Filtering of low quality reads, The length distribution of the clean sequences in the reference genome was determined, then the sequences were aligned and subjected to the Bowtie [19] search against Rfam v.10.1, ribosomal RNA, small cytoplasmic RNA, cis-regulatory RNA, small nuclear RNA, transfer RNA, and other RNAs were annotated and filtered. Next, cDNA sequences and species-specific repeat sequences from the Repbase database [20] were identified with Bowtie software. Mature miRNAs were identified by aligning against the miRBase v22 database, and the expression patterns in different samples were analyzed. Unannotated reads were analyzed by miRDeep2 [21] to predict novel miRNAs. Based on the hairpin structure of each pre-miRNA and the miRBase database, the corresponding miRNA star and miRNA mature sequence were also identified. Differentially expressed miRNAs (DEmiRNAs) were calculated and filtered with the threshold of p<0.05 and |FC|>1.5. The targets of DEmiRNAs in animals were predicted using miRanda software [22], with the following parameter score (S)≥150, a change in free energy (ΔG)≤−30 kcal/mol, and strict 5′ seed pairing requirements. GO enrichment and KEGG pathway enrichment analysis of DEmiRNAs target Genes were performed using R, based on the hypergeometric distribution.

### Long noncoding RNA and circular RNA analysis

StringTie was used to count reads for determining the original expression levels of lncRNA, which were normalized using FPKM. We screened for lncRNAs long than 200 bp and coverage exceeding 3. the selected lncRNAs were using PLEK, CNCI, and Pfamscan software to identify those with high credibility. To identify circRNA, we utilized find_circ. The 20 bp sequences at both ends of the unaligned reads from the HISAT2 alignment results were extracted and employed as anchor sequences. These sequences were realigned to the genome using Bowtie2 for circRNA detection. The read count values obtained from the circRNA identification results of find_circ were used as the original expression level of circRNAs, which were normalized using transcripts per million.

DESeq was used to normalize lncRNAs and circRNAs and to calculate FCs, distinguishing differentially expressed lncRNAs (DELs) from differentially expressed circRNAs (DECs) based on the criteria of |FC|>1.5 and p-value<0.05. The k-means clustering algorithm was employed to analyze the DELs and DECs, which were functionally analyzed using the GO and KEGG databases.

### Treatment and mass spectrometry analysis of skin tissue proteins

Skin samples from different groups were ground to a powder in lysis solution (Biyuntian) containing phosphatase inhibitor (Roche) and protease inhibitor PMSF (Biyuntian) The samples were then centrifuged to determine the protein concentration and molecular mass in the supernatant, and adjusted to the same concentration and volume. The samples were incubated in DTT, to which an appropriate volume of iodoacetamide was added and mixed well. Acetone was added to precipitate the protein, and the mixture was centrifuged to collect the precipitate, which was redissolved in NH_4_HCO_3_ (Source Leaf Bio). Trypsin Trypsin-TPCK (Wallis) was added at a concentration of 1/50th of the sample mass, incubated overnight at 37°C for digestion, and terminated by adjusting the pH to ~3 with phosphoric acid. The digested peptides were desalted using SOLA SPE 96-well plates, separated on an 1100 HPLC System (Agilent) using a Nano Chrom-C18 column (5 μm, 150 mm×2.1 mm) and lyophilized for MS. Peptides were separated in 90 min at a flow rate of 300 nl/min on a 25 cm×75 m column (1.6 μm C18, ionopticks). Mobile phases A and B were 0.1 vol% formic acid solution and 80:20:0.1 vol% ACN: water: formic acid, respectively. The total run was 60 min (45 min, 5%–27% B; 45–50 min, 27%–46% B; 50–55 min, 46%–100% B; 55–60 min, 100% B). Liquid chromatography was coupled online to a hybrid TIMS quadrupole TOF mass spectrometer (Bruker timsTOF Pro) via a Captive Spray nano-electrospray ion source. The conditions of DDA mass spectrometry are shown in the attached Materials and Methods Supplement 1. To perform data independent acquisition (DIA), we used the instrument control software (Bruker otofControl v6) to define quadrupole isolation windows as a function of the TIMS scan time (diaPASEF). The conditions of DIA mass spectrometry are shown in the attached Materials and Methods Supplement 2. The mass and concentration of proteins are shown in Supplement 3.

### Proteome data processing analysis

Spectronaut (ver. 15.3.210906.50606) was used to search all raw data against the sample protein database, performing the database search with trypsin digestion specificity and considering alkylation on cysteine as a fixed modification. The false discovery rates (FDRs) for proteins, peptides, and peptide-spectrum matches were all set to 0.01. For DIA data, the quantification FDR was also set to 0.05, and the quantification was performed at the MS2 level. Proteins with a p-value<0.05 in the three group comparisons (EF–SF, M–EF, M–SF) were considered differentially expressed proteins (DEPs), and were subjected to GO and KEGG analyses.

### Construction of regulatory networks

The cor function in R was used to analyze correlation coefficients (CCs) and p-values between the target gene, DELs, DECs, and DEPs, employing screening conditions of |CC|≥0.9 and p<0.01. The igraph package was employed to construct the ceRNA competitive regulatory network.

### Quantitative real time polymerase chain reaction validation

Seven RNAs were randomly selected for real time polymerase chain reaction (RT-PCR) analysis, with the ARPC5 gene as an internal reference. The reactions were performed using the PerfectStart Green qPCR SuperMix kit on the LightCycler 480 II Real-Time PCR System (Roche). The expression levels of the genes were analyzed using the 2^−ΔΔCt^ method. The relationship between the expression levels of these seven genes in different groups and the sequencing data was analyzed using the cor function in R language.

## RESULTS

### Analysis of micro RNA and mRNA

**Figure 1:**
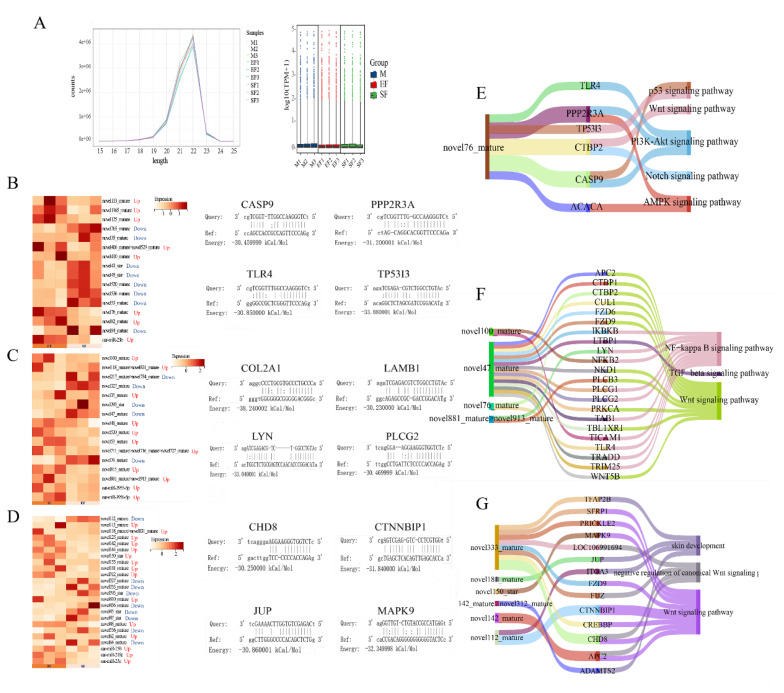
Analysis of miRNAs and their target genes. (A) Overall expression trends of miRNAs in the three groups. (B–G) Expression of DEmiRNAs, putative binding sequences in target genes, and functional analyses in the EF–SF (B, E), M–EF (C, F), and M–SF (D, G) group comparisons. miRNA, micro RNA; DEmiRNA, differentially expressed micro RNA.

A total of 18,931 mRNAs were identified in this study (Supplement 4), Counting of the miRNAs in the group samples showed that most were between 20–23 bp, with similar expression levels in each sample (Figure 1A). Differential analysis identified 16 DEmiRNAs in the EF–SF comparison, of which eight were significantly upregulated and eight were significantly downregulated, and 16 DEmiRNAs in the M–EF comparison, of which 11 were upregulated. The highest number of DEmiRNAs (24) was identified in the M–SF comparison, of which 15 were upregulated. Only a few of these DEmiRNAs have been previously assigned official names. The prediction of target genes and binding free energy of these DEmiRNAs revealed that target genes could be paired with the seed region sequence of the miRNAs (Figures 1B–1D). Because most of the DEmiRNAs did not have official names, we analyzed their functions on the basis of genomic location. In the EF–SF comparison, the novel76mature miRNA and its target genes were mainly enriched in the PI3K Akt, p53, and Wnt signaling pathways (Figure 1E). In the M–EF comparison, several DEmiRNAs and their target genes were enriched in valuable pathways. Among them, novel47mature and its target genes were found to be involved in the nuclear factor (NF)-kappa B, transforming growth factor (TGF)-beta, and Wnt signaling pathways. Novel76mature was also identified in the M–EF comparison, where it participated in different regulatory pathways (Figure 1F). In the M–SF comparison, six DEmiRNAs were enriched in pathways related to hair follicle development. Multiple target genes of novel333mature showed involvement in multiple pathways, namely negative regulation of the Wnt signaling pathway, the Wnt signaling pathway, and skin development. Other miRNAs, such as novel112mature, novel150star, and novel181_mature and their target genes were only enriched in one hair follicle-related pathway (Figure 1G).

### Differentially expressed long noncoding RNAs and differentially expressed circular RNAs analysis

**Figure 2:**
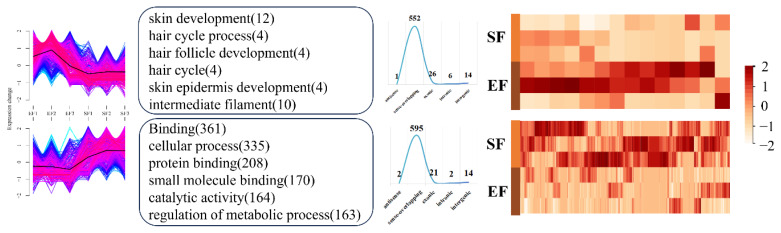
DECs analysis in the EF–SF group comparison. From left to right are the expression patterns, functional analysis, location distribution, and expression heat maps of the main pathways of DECs in Cluster 1 (top) and Cluster 2 (bottom). DEC, differentially expressed circular RNA.

**Figure 3:**
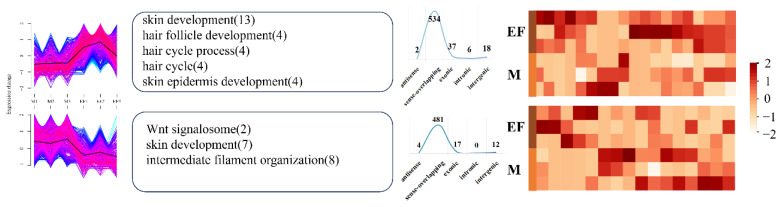
DECs analysis in the M–EF group comparison. From left to right are the expression patterns, functional analysis, location distribution, and expression heat maps of the main pathways of DECs in Cluster 1 (top) and Cluster 2 (bottom). DEC, differentially expressed circular RNA.

**Figure 4:**
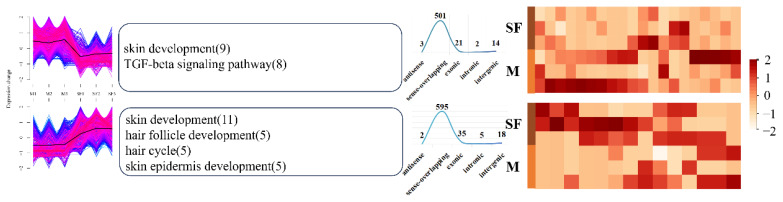
DECs analysis in the M–SF group comparison. From left to right are the expression patterns, functional analysis, location distribution, and expression heat maps of the main pathways of DECs in Cluster 1 (top) and Cluster 2 (bottom). DEC, differentially expressed circular RNA.

Sequencing data revealed 373,461 and 377 DELs in the EF–SF, M–EF, and M–SF comparisons, respectively. There were large numbers of DECs in all three pairings, with 1,233 DECs in the EF–SF comparison and 1,111 DECs in the M–EF comparison. Additionally, the total number of DECs in the three comparisons was higher than that in the upregulated group (Supplement 5). Next, we applied clustering algorithms to partition DECs and DELs within each group comparison into two distinct clusters, enabling the investigation of expression patterns across experimental groups. In the EF–SF comparison, Cluster 1 DECs were predominantly upregulated in the EF group, while Cluster 2 DECs were predominantly upregulated in the SF group. Functional enrichment analysis revealed that Cluster 1 DECs were significantly enriched in pathways associated with skin development (e.g., epidermal differentiation and hair follicle morphogenesis), with pathway-related DECs demonstrating consistently higher expression levels in the EF group than in the SF group (Figure 2). In contrast, DECs in Cluster 2 were enriched in a broader spectrum of biological processes, with key DECs involved in representative pathways (e.g., extracellular matrix organization and immune response) showing elevated expression in the SF group. For DELs in the EF–SF comparison, Clusters 1 and 2 displayed divergent expression profiles and functional annotations. DELs in Cluster 1 were predominantly associated with the regulation of genetic material synthesis (e.g., DNA replication and RNA processing), while DELs in Cluster 2 were enriched in metabolic processes and cellular communication pathways, including signal transduction and intercellular transport (Supplement 6). In the M–EF comparison, the numbers of DECs and DELs in Clusters 1 and 2 were comparable. DECs in Cluster 1 showed significant enrichment in pathways associated with skin and hair follicle development (e.g., epidermal morphogenesis and Wnt signaling). Notably, DECs within these pathways exhibited consistently higher expression levels in the EF group than in the M group (Figure 3). In contrast, DECs in Cluster 2 were enriched in biologically relevant pathways; however, pathways enriched by DECs in the M-EF comparison group lacked directional specificity and exhibited a broad distribution. For DELs in the M–EF comparison, Clusters 1 and 2 displayed divergent expression patterns between the two groups. Functionally, DELs in Cluster 1 were predominantly involved in biological binding processes (e.g., protein–DNA interaction), while DELs in Cluster 2 participated in both binding processes and metabolic regulation (e.g., lipid catabolism) (Supplement 7). In the M–SF comparison, DECs in Cluster 1 were enriched in pathways involved in TGF-beta signaling and skin development, with pathway-related DECs demonstrating overall higher expression in the M group than in the SF group. DECs in Cluster 2 were enriched in more functionally significant pathways, including hair cycle and hair follicle development, with pathway-associated DECs exhibiting elevated expression in the SF group (Figure 4). DELs in the M–SF comparison retained consistent expression patterns with prior groups, though neither cluster showed a distinct functional orientation (Supplement 8).

### RNA interaction network analysis

**Figure 5:**
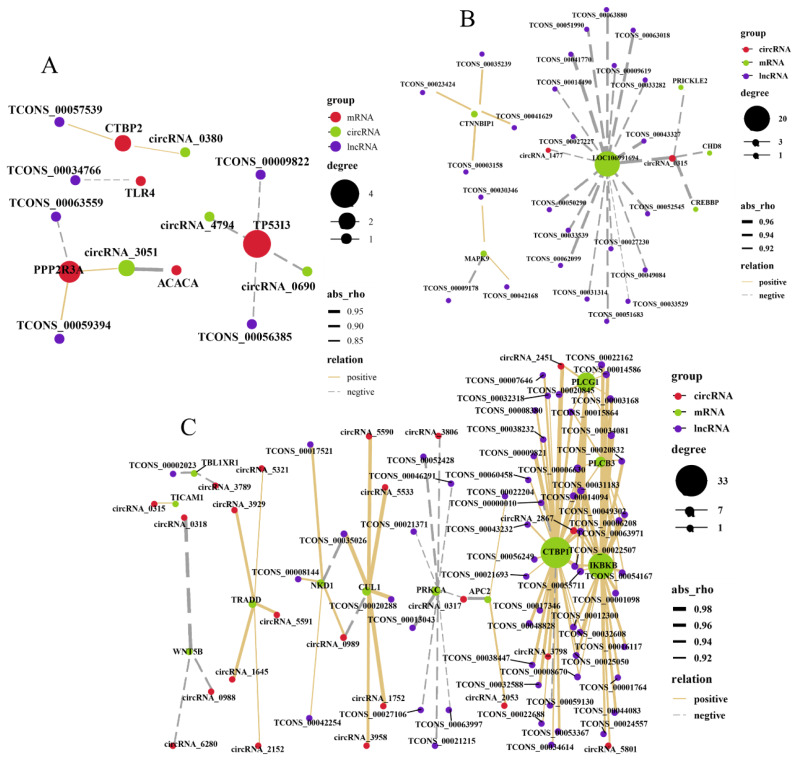
Regulatory networks based on the expression relationships among the DECs, DELs, and DEmiRNAs associated with the skin follicle development-related pathways in the three groups. (A–C) The results depict comparisons between EF–SF (A), M–SF (B), and M–EF (C). DEC, differentially expressed circular RNA; DEL, differentially expressed long noncoding RNA; DEmiRNA, differentially expressed micro RNA.

Next, we compiled the target genes enriched in the DECs, DELs, and DEmiRNAs associated with the skin follicle development-related pathways in each group to construct a ceRNA regulatory network based on the expression relationships among these RNA types. The EF–SF comparison was mainly dominated by the target genes *TP53I3* and *PPP2R3A*. Furthermore, *TP53I3* was negatively correlated with circ_4794, circ_ 0690, TCONS_00009822, and TCONS_00056385, suggestive of competitive relationships (Figure 5A). The target genes in the M–SF comparison were negatively correlated with most of the DELs and DECs, suggesting that multiple circRNAs or lncRNAs may compete for binding to the same miRNA (Figure 5B). DELs were positively correlated with most of the mRNAs in the M–EF comparison; however, *PRKCA* was negatively correlated with both DECs and DELs, suggesting that these DECs and DELs may share an miRNA with *PRKCA* (Figure 5C).

### Correlation analysis between differentially expressed genes and differentially expressed proteins

**Figure 6:**
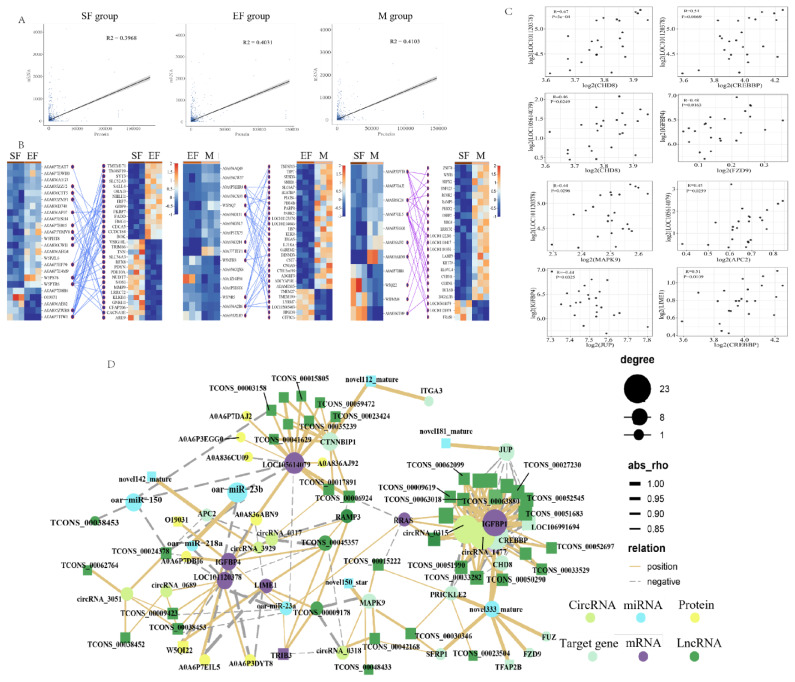
Correlation analysis of RNA–protein interactions among the three groups. (A) Correlation of protein expression with transcription in the three group comparisons. (B) The correlation and heat map of specific high expression proteins and genes in each group. (C) Correlation analysis of skin development-related miRNAs in association with specific target DEGs. (D) Integrated regulatory network of group-specific DEGs, miRNAs, circRNAs, lncRNAs, proteins, and target genes. DEG, differentially expressed gene; miRNA, micro RNA; circRNA, circular RNA; lncRNA, long noncoding RNA.

Correlation analysis of the transcriptomes and proteomes in the EF–SF, M–EF, and M–SF group comparisons revealed that the CC between mRNA and protein expression in M-SF pairing was the highest (0.41) (Figure 6A), We selected genes and proteins enriched in wool fiber diameter-related regulatory pathways and calculated their CCs. The results showed that the differences in specific DEPs were greatest between the M and SF groups, with very obvious differences in DEGs observed in all three group comparisons (Figure 6B). Analysis of the target genes of skin development-related miRNAs in association with specific DEGs showed that *LOC10112037* and *LOC105614079* had positive correlations with most of the target genes, suggesting that they may act as key genes to regulate skin development (Figure 6C). Therefore, we constructed the main regulatory network comprising group-specific DEGs, miRNAs, circRNAs, lncRNAs, proteins, and target genes. Key nodes in this network were *LOC10112037*, *LOC105614079*, *IGFBP1*, and *IGFBP4*. The majority of RNAs exhibited positive correlations with one other, while some target genes, circRNAs, and lncRNAs showed negative correlations, suggesting a complex regulatory relationship among these RNAs (Figure 6D).

### Validation of expression levels by real time polymerase chain reaction

**Figure 7:**
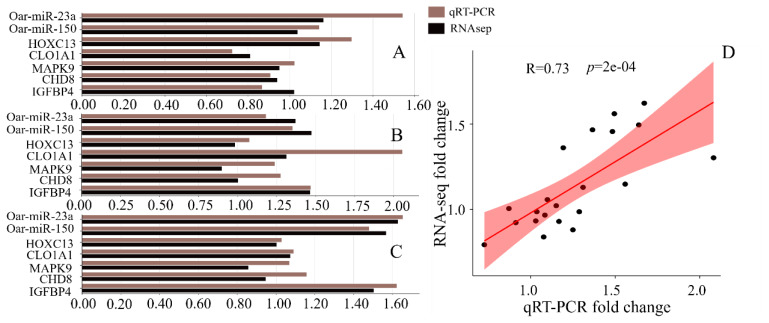
qRT-PCR validation of differentially expressed genes. (A) qRT-PCR expression levels in the EFSF group. (B) qRT-PCR expression levels in the MEF group. (C) qRT-PCR expression levels in the MSF group. (D) Correlation between qRT-PCR and RNA-Seq mRNA expression levels. qRT-PCR, quantitative real time polymerase chain reaction.

We selected seven RNAs, namely oar-miR-23a, oar-miR-150, *HOXC13*, *COL1A1*, *MAPK9*, *CHD8* and *IGFBP4*, and analyzed their expression levels across different groups with varying fiber diameters. The expression levels measured by qRT-PCR showed an extremely strong correlation with the RNA-Seq data (Figure 6), thereby confirming the authenticity and reliability of the RNA-Seq data in this study (Figure 7).

## DISCUSSION

Wool fineness is a key factor influencing the economic value and quality of wool products. In this study, we sought to resolve the regulatory mechanisms underlying wool fiber diameter by analyzing skin tissues from sheep with three different levels of wool fineness. Through DEmiRNA mining, we identified oar-miR-23b in the EF–SF comparison. This miRNA and its target genes, *TGFβ2* and *NOTCH1*, are differentially expressed at various stages of hair follicle development, inhibiting dermal fibroblast proliferation and migration while promoting apoptosis [23]. Dermal fibroblasts are essential for hair follicle development and regeneration [24,25], and the regulatory effects of oar-miR-23b on these cells directly inhibit hair follicle development. In this study, oar-miR-23b was significantly upregulated in the EF group and, combined with the analysis of individual wool phenotypes in the EF and SF groups, it is possible that oar-miR-23b affects wool fineness through regulating dermal fibroblasts, leading to the coarsening of wool. In addition, a family member of oar-miR-23b, namely oar-miR-23a, was identified in the M–SF comparison. Typically, members of an miRNA family share homology and can exhibit synergistic or antagonistic effects on function. For example, miR-23a and miR-23b have been shown to achieve functional complementarity or antagonism through common promoters or by synergistically regulating transcription in both humans and mice [26]. Therefore, oar-miR-23a and oar-miR-23b are likely to exhibit a similar relationship. We found that oar-miR-23a expression was significantly higher in group M than in group SF, suggesting that its influence on wool fineness may functionally synergize with that of oar-miR-23b, particularly given the notable differences in individual wool fineness between the two groups. However, because previous studies on oar-miR-23a only reported its interaction with circRNA2440 to regulate genes related to fat differentiation [27], its involvement in regulating wool fineness requires further study. The M–SF comparison also showed strong upregulation of oar-miR-150 and oar-miR-218a in the M group, where oar-miR-150 may interact with lncRNA XLOC_012081 to deregulate the inhibition of Notch1 and promote the proliferation of follicular stem cells [28]. Collectively, the miRNAs mined in this study appear to affect wool fiber diameter by inhibiting hair follicle development.

This study also unearthed many previously unnamed miRNAs linked to pathways related to hair follicle development, with the highest number identified in the M–SF comparison and the second highest in the M–SF comparison. This may be related to the large phenotypic differences, including the diameter of individual wool fibers, between the M and SF groups. These DEmiRNAs and their target genes were found to be mainly focused on the skin development and Wnt-related pathways, with most showing upregulation in the M group. However, transcriptome data revealed that the target genes of these DEmiRNAs were not differentially expressed between the M and SF groups. Coincidentally, the target genes of DEmiRNAs identified in the EF–SF and M–EF comparisons, such as novel76_mature, novel00_mature, and novel00_mature, were enriched in hair follicle and skin development-related pathways and did not show between-group differences, indicating that these miRNAs may not affect wool traits by acting on target genes. In our analysis of the expression relationships among these target genes, DECs, and DELs, most showed negative correlations in the EF–SF and M–EF comparisons, suggesting that the DECs may act as sponges, releasing the target genes by competitively binding to miRNAs [29]. However, this hypothesis requires validation through double luciferase assays and other tests.

CircRNAs and lncRNAs generally influence biological traits through gene regulatory networks, exhibiting both similarities and notable differences in their mechanisms of action, functional localization, and regulatory patterns. In this study, the expression patterns of circRNAs and lncRNAs across different groups were analyzed through clustering. In the EF–SF comparison, DELs did not show obvious expression patterns between the EF and SF groups, and there was little functional overlap among lncRNAs across different clusters. By contrast, circRNAs appeared to be more closely associated with the regulation of skin and hair. In Cluster 1, DECs enriched in skin development-related functions were more highly expressed overall in the EF group than in the SF group, suggesting that these DECs may regulate hair growth toward coarseness. This pattern did not continue in the M–EF comparison, where DECs involved in skin development-related pathways did not differ significantly between the two groups. Further analysis showed a completely different expression pattern of DECs in the M–SF pairing compared with those in the other group comparisons. Additionally, DECs enriched in skin- and hair-related functions were more highly expressed overall in the SF group than in the M group. This led us to deduce that the phenotypic differences between the groups might be attributable to different sets of dominant genes.

Considering that proteins are the executors of biological functions, we compiled the highly expressed DEPs identified in each group comparison. In the EF–SF pairing, the highly expressed DEPs were found to be mainly focused on energy metabolism in the SF group and regulation of signaling in the EF group. In the M–EF comparison, the biological functions of the highly expressed DEPs in the EF group tended to be in the area of membrane system and signaling, while those in the M group tended to be involved in regulation of gene expression. The M–SF comparison revealed DEPs involved in biological processes related to gene expression regulation and energy metabolism. The connections between each group were weak, making it challenging to observe the effects of highly expressed DEPs on trait development. Therefore, we further analyzed the highly expressed DEGs in each group to investigate the link between these DEPs and DEGs. In the EF–SF comparison, the highly expressed DEGs in the EF group also showed involvement in signaling regulation, while those in the SF group were more related to developmental and differentiation regulation, and cell structure and motility. Combined with the highly expressed DEPs reported in the EF–SF comparisons in previous studies [30–32], the phenotypic differences between the EF and SF groups appear to involve multiple regulatory processes, with key biological processes, including energy metabolism, signaling regulation, and cell structure and motility, playing essential roles in hair follicle development. Notably, in the M–SF comparison, we found that the highly expressed DEGs in the M group were mainly involved in lipid-related biological processes, particularly the regulation of triglyceride and acyltransferase activities. Triglycerides affect the integrity of the cell membrane, and acyltransferases influence the synthesis of triglycerides; when triglyceride synthesis is blocked, cell membrane fluidity decreases and follicular keratinocytes are blocked, leading to thickening of the hair diameter and roughness of the skin surface [33–35]. The wool fiber diameter phenotype of group M is probably affected by this phenomenon. Likewise, the high expression of DEGs *LOC10112037* and *LOC105614079* in group SF appears to influence the development of the cutaneous epidermis and the establishment of the skin barrier, which have not been previously reported for these genes.

To further elucidate the roles of the newly identified DEGs in regulating wool fiber diameter, we constructed an integrated regulatory network in which mRNAs serve as the main targets, linking proteins with various RNAs. Among these, *IGFBP1* and *IGFBP4* belong to the insulin-like growth factor (IGF) system and encode IGF binding proteins, which play important roles in the regulation of hair growth and the hair follicle cycle [36,37], IGFBP4 has been associated with dermal papillae and epithelial matrix, suggesting its involvement in the hair follicle growth cycle [38,39], while another family member, IGFBP3, is reportedly linked to the proliferation of hair follicle keratinocytes, influencing the diameter of wool fibers [40], This leads us to speculate that *IGFBP1* may also be involved in the hair growth and hair follicle cycle. The key nodes of this network, oar-mir-23a and oar-miR-218a, are closely related to genes involved in hair follicle development. Their target genes, such as *CTNNBIP1*, *MAPK9*, *CREBBP*, and *CHD8*, are also deeply involved in this regulatory network, and may influence hair follicle development and morphology through signaling pathways such as Wnt/β-catenin and JNK [41–45]. The proteins and lncRNAs in this regulatory network are mainly involved in energy and fat metabolism, with related circRNAs showing enrichment in the Notch, NF-kappa B, and other pathways. Collectively, our findings lead us to speculate that wool fiber diameter may be directly regulated by multiple types of RNAs involved in various signaling pathways, with the effects mediated by proteins. This regulation appears to primarily involve energy and fat metabolism, and the entire biological process contributes to the overall regulation of wool phenotype. Further research will be necessary to refine our understanding of the specific role of energy and fat metabolism in this process.

## CONCLUSION

Through comprehensive transcriptomic and proteomic analyses of Alpine Merino sheep exhibiting a range of wool fiber diameters, we identified numerous key RNAs that may be involved in regulating this important trait. Our findings highlight the potential importance of energy and fat metabolism on wool fiber diameter, offering valuable insights for elucidating the underlying regulatory mechanisms. This research provides a theoretical foundation for advancing the fine-wool sheep industry through improved practices and targeted breeding strategies.

## SUPPLEMENTARY MATERIAL

**Supplement 1**. DDA mass spectrometry conditions. **Supplement 2**. DIA mass spectrometry conditions.

**Supplement 3**. Protein mass and concentration.

**Supplement 4**. mRNA assembly table.

**Supplement 5**. Summary of differential circRNA and differential lncRNA.

**Supplement 6**. Analysis of differential lncRNA in EF/SF group.

**Supplement 7**. Analysis of differential lncRNA in M/EF group.

**Supplement 8**. Analysis of differential lncRNA in M/SF group.
