# Supplementary material for: Transcriptome and proteome combined analysis of wool fiber diameter regulation mechanism
Source: Anim Biosci. 2025 Sep 30;39(2):250378. doi: 10.5713/ab.25.0378 (PMC12877398; doi:10.5713/ab.25.0378)
Supplement: Supplementary file 1 [file ab-25-0378-Supplementary-1,2.pdf]

Supplement 1. DDA mass spectrometry conditions

Items	Value
Ion Mobility	0.85-1.3
Capillary	1.4KV
Mass Range	100-1700m/z
Dry Temperature	180℃
Collision Energy	20-59eV
Dry Gas	3.0L/min

Supplement 2. DIA mass spectrometry conditions

Items	Value
Ion Mobility	0.7-1.3
Capillary	1.4KV
Mass Range	100-1700m/z
Dry Temperature	180℃
Collision Energy	20-59eV
Dry Gas	3.0L/min
