# Supplementary material for: Transcriptome and proteome combined analysis of wool fiber diameter regulation mechanism
Source: Anim Biosci. 2025 Sep 30;39(2):250378. doi: 10.5713/ab.25.0378 (PMC12877398; doi:10.5713/ab.25.0378)
Supplement: Supplementary file 2 [file ab-25-0378-Supplementary-3.pdf]

Supplement 3. Protein mass and concentration

Sample	absorbance1	absorbance1	absorbance1	mean absorbance	determination concentration(μg/μl)	actual concentration(μg/μl)
M1	0.205	0.206	0.215	0.2087	0.1986	1.9864
M2	0.23	0.244	0.24	0.238	0.2264	2.2641
M3	0.244	0.247	0.241	0.244	0.2321	2.3209
EF1	0.378	0.382	0.378	0.3793	0.3602	3.6012
EF2	0.243	0.252	0.237	0.244	0.2321	2.3209
EF3	0.282	0.299	0.279	0.2867	0.2725	2.7249
SF1	0.285	0.307	0.298	0.2967	0.282	2.8195
SF2	0.233	0.245	0.244	0.2407	0.2289	2.2894
SF3	0.256	0.246	0.238	0.2467	2346	2.3462
