# Supplementary material for: Transcriptome and proteome combined analysis of wool fiber diameter regulation mechanism
Source: Anim Biosci. 2025 Sep 30;39(2):250378. doi: 10.5713/ab.25.0378 (PMC12877398; doi:10.5713/ab.25.0378)
Supplement: Supplementary file 3 [file ab-25-0378-Supplementary-4.pdf]

Supplement 4 mRNA assembly table

id	EF1	EF2	EF3	M1	M2	M3	SF1	SF2
A1BG	0.110139	0.111585	0.122363	0.225735	0.275384	0.09703	0.209609	0.139634
A1CF	0.006163	0.003122	0	0.009473	0.003082	0	0.012511	0.006251
A2ML1	0.098294	0.099584	0.048535	0.081842	0.110595	0.18556	0.043649	0.143309
A3GALT2	0.31378	0.413267	0.485463	0.26796	0.31382	0.315923	0.286638	0.243989
A4GALT	1.052224	1.539086	1.279408	1.860047	1.420686	1.410343	1.355035	1.307324
A4GNT	0	0.027638	0.05388	0.027956	0.027283	0	0	0
AAAS	11.31633	10.79395	10.50685	10.01323	11.46497	11.20103	11.02305	11.4625
AACS	24.79934	22.37628	25.36278	25.96644	26.31165	23.35507	21.02901	20.60877
AADACL3	1.792061	1.057098	1.938555	2.108294	1.314741	1.103949	0.981271	0.645716
AAGAB	4.94368	5.671299	5.067409	5.747483	4.34907	5.290568	5.300837	5.046686
AAK1	1.92578	1.661927	1.510241	1.529225	1.823627	1.478329	1.58098	1.8847
AAMDC	39.51411	43.83789	46.23672	39.70202	40.80566	41.54533	39.21905	39.92007
AAMP	46.93663	44.64506	42.75579	45.11085	48.03833	44.44545	45.65173	42.75355
AANAT	0	0	0	0	0.02919	0	0	0
AAR2	5.304544	4.866703	4.469666	5.02778	5.118128	4.927616	5.071724	4.535407
AARD	0.181748	0.184133	0.358966	1.055415	0.424133	0.243986	0.430439	0.49156
AARS	50.26996	45.44728	50.35231	54.2118	50.33421	49.2679	49.29921	41.65691
AARS2	2.585133	1.559441	1.831868	2.271678	2.197318	2.013357	1.876129	2.168266
AASDH	5.287071	5.321631	4.379285	5.199622	5.487163	5.212435	5.945583	6.519876
AASDHPPT	20.42008	22.58017	23.04993	21.89546	22.75134	22.92037	23.08127	23.25581
AATF	14.17651	14.33525	15.54357	13.67147	15.95737	13.33717	14.51225	12.64253
AATK	2.028816	2.093107	1.479051	2.318545	2.390276	2.315389	1.816667	2.025378
ABAT	1.401555	1.862924	1.591113	0.932963	1.365794	1.519678	1.440816	1.81637
ABCA1	16.32565	11.77173	12.24986	15.7368	13.80648	11.67985	13.66413	16.90071
ABCA10	10.60501	10.67165	9.120674	9.349888	9.944039	9.87552	10.78225	13.28386
ABCA12	8.664181	6.274759	6.206339	6.773546	7.838587	5.789063	7.930461	6.768641
ABCA2	3.410841	3.094466	1.980971	3.033344	3.467204	2.895798	3.295288	3.36724
ABCA3	0.371854	0.601326	0.331911	0.586254	0.457726	0.514792	0.500823	0.736163
ABCA4	0.502226	0.575766	0.691745	0.771999	0.426286	0.688624	0.657321	0.489268
ABCA5	2.611347	3.096971	2.493075	2.80714	2.78077	2.516474	2.49515	2.611447
ABCA6	6.970905	5.305704	5.911247	6.283464	7.414142	4.781909	5.856264	9.838047
ABCA7	0.558006	0.30607	0.221123	0.415213	0.423002	0.347111	0.411254	0.40013
ABCB1	4.2646	2.986058	3.967111	3.974975	3.486361	3.458434	4.985979	3.653631
ABCB10	10.6429	11.15871	11.69846	13.42179	11.88198	11.0894	10.32103	12.02865
ABCB11	0.020133	0.016318	0	0.04539	0.096652	0.036487	0.012261	0.012252
ABCB4	0.053376	0.081115	0.026356	0.047861	0.020019	0.067176	0.033861	0.020301
ABCB6	4.939264	4.950852	4.393408	5.321872	5.885842	5.16699	5.20891	4.503291
ABCB7	7.292037	6.814188	7.180661	7.036226	6.934564	6.578384	7.042944	8.054306
ABCB8	6.077579	6.696714	6.592585	7.012768	6.269809	5.830007	6.071597	6.176231
ABCB9	2.95768	2.555595	1.870284	2.634525	3.030604	2.661435	2.445808	2.329533
ABCC1	8.253626	7.648517	6.773505	7.1179	6.905588	6.026484	7.10109	7.652271
ABCC10	1.794488	1.606753	1.120752	1.247496	1.343615	1.284255	1.471893	1.834791
ABCC11	1.63432	2.466285	6.156736	9.691605	8.338397	7.801671	8.253575	3.888952
ABCC12	0	0	0.00677	0	0	0	0.013916	0.006953
ABCC2	0.009032	0.02135	0.020811	0.012341	0.003011	0.006062	0.012223	0.01832
ABCC3	4.263745	4.481894	3.251504	5.184171	3.864018	4.11557	3.536907	3.87522
ABCC5	4.343471	3.18796	2.68516	3.530531	3.477916	3.355	2.83626	4.015236
ABCC6	0	0.005462	0.005324	0.044195	0.021566	0	0	0.02187
ABCC8	0.207327	0.186709	0.199056	0.218364	0.178555	0.29572	0.263046	0.262848
ABCD1	3.192562	3.736902	4.262378	3.644865	4.239217	4.1584	3.602249	3.898188
ABCD2	0.047058	0.476758	0.051635	0.230403	0.151652	0.168461	0.116757	0.111366
ABCD3	27.82982	33.55478	30.89778	29.6459	29.0207	33.32231	35.21811	27.72499
ABCD4	7.034252	4.666957	3.242773	3.85144	5.47871	4.543958	3.743638	5.816421
ABCE1	33.36233	37.07912	37.66327	35.9839	37.31847	40.09158	34.62023	35.01643
ABCF1	30.5954	27.23505	31.14535	30.24893	30.71064	31.0049	27.77888	29.96173
ABCF2	14.16502	14.00558	13.06281	12.32298	14.04374	12.01193	14.43516	12.24432

ABCF3	16.6744	17.39925	15.51809	15.67004	17.17608	16.34346	16.79777	16.16165
ABCG1	10.1049	7.131102	8.20415	11.3265	8.262118	8.093253	11.5497	11.75113
ABCG2	8.364672	12.98249	9.966144	12.5923	11.4916	13.28279	15.30229	11.88726
ABCG4	0.063483	0.021439	0.062692	0.014457	0.028219	0.007102	0.014319	0.007154
ABCG5	0.049626	0.025139	0.024504	0.076283	0.049633	0.012491	0.012593	0.025166
ABCG8	0.418999	0.328986	0.237923	0.515254	0.366672	0.495688	0.510341	0.499333
ABHD1	0.822031	0.60938	0.277196	0.554747	0.380991	0.383544	0.488407	0.589714
ABHD11	7.931783	6.719658	7.85546	8.221684	8.685057	8.330192	7.611207	6.588329
ABHD12	39.36254	35.18306	39.26694	37.62108	39.01216	42.13637	42.11747	39.66755
ABHD13	11.85242	11.51414	11.76554	10.87357	11.26896	12.24852	11.95209	12.3438
ABHD14A	1.78135	2.513435	2.385703	2.07554	2.782195	2.145672	2.460297	2.631691
ABHD14B	44.08463	48.6283	35.90577	39.86377	29.77198	31.71145	45.21021	30.89644
ABHD15	0.495932	0.900206	0.704019	0.645858	0.44433	0.509723	0.492884	0.513471
ABHD16A	20.07918	21.8193	21.36612	25.07178	21.6102	21.48433	22.10387	21.19742
ABHD16B	0.035508	0.044967	0	0.081872	0.017756	0	0	0
ABHD17A	27.0128	22.43973	23.55149	21.9936	24.79206	22.26395	23.74758	22.17094
ABHD17B	6.612606	6.302936	5.711479	6.470547	6.892062	7.365628	5.8501	5.623706
ABHD17C	12.91717	13.93834	11.63376	11.24731	13.68852	11.20539	11.63279	12.77683
ABHD18	2.353007	2.920998	2.495813	2.99923	3.145012	2.566505	3.066462	3.196736
ABHD2	17.87656	16.79641	15.64099	19.12803	19.02342	19.03794	17.23368	17.34208
ABHD3	7.807067	6.927848	6.260786	9.149451	7.503506	6.187975	6.912576	8.900961
ABHD4	12.05013	11.13173	11.17517	12.71907	12.19412	13.20231	11.84172	12.08834
ABHD5	70.22379	62.23841	90.24606	86.0474	102.3057	100.5312	67.95575	75.95842
ABHD6	3.822859	4.55899	5.133073	4.440605	4.6672	4.562137	5.296952	5.113362
ABHD8	3.355386	3.469369	3.531755	3.396049	3.701068	3.169771	3.531854	3.655237
ABI1	38.84724	37.03034	38.4193	34.42894	37.6186	37.62558	40.15479	39.00518
ABI2	2.338916	2.303213	1.674686	2.02746	2.183544	1.859995	2.515359	2.26835
ABI3	1.549459	1.285895	0.797633	1.250001	1.994774	1.294505	0.853274	1.15356
ABI3BP	18.2355	16.75086	16.81726	19.26246	17.6501	16.25825	18.50013	24.69571
ABITRAM	16.76295	19.16868	18.72233	17.13905	17.24248	18.16538	17.67327	18.29897
ABL1	13.03427	12.67054	11.17967	11.69269	13.11311	10.06938	12.00984	14.9907
ABL2	2.034075	1.668243	1.151389	1.224207	1.897998	1.275016	1.682256	1.699181
ABLIM1	26.39407	26.63291	25.67907	26.58349	27.31685	26.96285	26.77159	30.82744
ABLIM2	0.840155	0.967915	0.640044	0.821608	0.566578	0.667048	1.101275	0.676823
ABLIM3	7.063691	5.798153	5.351974	5.560225	5.761732	5.431057	6.348058	6.022586
ABO	0.471469	0.431056	0.283899	0.129625	0.172511	0.254712	0.163404	0.151618
ABR	22.50205	22.49956	20.02999	22.83853	24.46	20.27823	21.57652	22.55406
ABRA	0	0.010553	0	0.021349	0	0	0	0
ABRACL	69.1698	80.01448	88.25358	68.71184	83.2264	89.28946	70.41548	70.29313
ABRAXAS1	6.580883	5.955856	6.724652	7.312857	7.055357	8.163101	6.157494	5.540055
ABRAXAS2	2.631057	2.83756	2.412017	2.077739	2.206978	2.772463	1.990927	2.687641
ABT1	4.539266	4.034546	4.041495	4.132815	4.161532	4.201416	4.528752	4.132622
ABTB2	6.354173	4.675036	5.492033	5.669858	6.537544	5.742893	5.465274	5.56529
ACAA1	27.64489	28.2693	25.14156	29.82906	26.42615	27.79909	28.19934	26.13545
ACAA2	74.93302	101.0562	114.9082	137.3239	139.2416	135.9079	80.64892	74.2998
ACACA	10.80722	9.863128	8.205375	9.954487	10.82742	9.028322	9.985005	8.233741
ACACB	0.302277	0.502703	0.278798	0.403279	0.365061	0.315827	0.257606	0.251627
ACAD11	7.310886	6.283614	7.263586	5.343067	5.981255	7.18861	5.980166	6.547527
ACAD8	5.88046	6.472248	5.768602	5.959386	5.360181	5.599358	6.57677	4.722462
ACAD9	44.66086	49.38606	41.79892	54.89455	57.30526	43.43303	44.82853	37.9488
ACADL	5.438607	5.976437	7.701048	7.421269	6.206762	6.963005	7.301834	5.778698
ACADS	3.765866	4.647195	3.481266	4.134802	4.828295	3.36397	4.943195	4.479987
ACADSB	1.469985	2.365323	1.585094	1.456553	2.172832	2.04592	2.08446	1.704682
ACADVL	63.97343	70.30261	75.9363	75.00672	73.70843	74.444	72.0481	63.19317
ACAN	0.025488	0.006456	0.050341	0.026119	0.035051	0.009623	0.022637	0.019388
ACAP1	11.6104	11.71784	9.72452	10.13659	10.92428	11.84603	11.94214	11.48326
ACAP2	11.41244	10.60139	8.263791	9.057191	11.20668	9.885598	10.28937	11.27583
ACAP3	7.009509	6.273936	4.784846	6.725248	6.354061	5.15949	5.633614	6.387035

ACAT1	42.22039	49.73547	53.73361	51.3274	43.02564	50.34674	45.37158	44.31115
ACAT2	47.51349	43.45758	46.17639	54.15913	46.4706	48.96826	54.89419	41.91885
ACBD3	20.60994	22.51582	21.12172	19.65827	21.04504	20.4315	24.18861	20.90334
ACBD4	3.179555	3.267301	2.808001	3.174528	2.916483	3.640308	3.660621	3.20639
ACBD5	7.867225	8.182144	6.581051	7.796315	7.457537	7.819419	7.963296	8.096131
ACBD6	13.39089	14.56745	16.62038	13.1227	14.65504	14.62432	14.79868	14.28657
ACBD7	0.138732	0.281105	0.034251	0.17771	0.104062	0.06984	0.352032	0.175884
ACCS	2.964017	3.595598	2.439238	3.410456	3.029409	3.481644	2.982088	2.927101
ACCSL	0.026158	0.026501	0	0.026806	0	0	0.013275	0.02653
ACD	10.49947	9.160667	7.839369	8.772948	9.425275	7.778809	8.976555	10.26145
ACE2	0.762283	0.654381	0.551659	0.727497	0.663446	0.744056	0.608342	0.613786
ACER1	25.525	26.09551	33.81589	26.91959	35.01423	28.99957	27.08765	17.87193
ACER2	7.269719	4.567286	4.245568	4.528005	5.031236	5.215248	4.090896	4.239216
ACER3	14.72526	20.89724	18.08071	14.98509	15.54637	15.50998	15.8909	16.64362
ACHE	0.885033	0.818191	1.005106	1.031662	1.493686	0.868802	0.763562	0.617122
ACIN1	46.64128	41.84393	36.8557	41.46279	42.41264	38.90995	40.98375	46.13962
ACKR1	1.541732	1.195061	2.145836	2.555443	5.422631	2.448203	2.48907	2.854503
ACKR3	9.832162	10.43754	9.299962	9.678915	12.35057	7.908326	10.38669	13.3663
ACKR4	0.237379	0.55751	0.586054	0.453347	0.593525	0.488865	0.536639	0.853598
ACLY	40.36571	41.46426	42.03062	44.3392	47.23506	44.36329	41.25932	35.29737
ACMSD	0.011173	0.02264	0	0	0	0.01125	0	0
ACO1	20.27518	21.21276	20.1566	22.85596	27.72124	21.28201	18.82046	21.44662
ACO2	50.44454	48.10567	49.19342	54.00138	49.62038	52.02841	43.98977	45.80382
ACOD1	0.095485	0.024185	0.188591	0.110082	0.322305	0.024034	0.048459	0.41159
ACOT11	0.137963	0.139773	0.153274	0.106035	0.224218	0.27781	0.096272	0.087454
ACOT12	0.033301	0.008434	0.008221	0.025594	0.008326	0	0.05915	0
ACOT13	6.546667	4.718778	7.547357	7.226031	10.38372	3.949027	6.688776	5.528344
ACOT6	0.729295	0.838713	1.2263	0.828156	1.478492	0.873195	0.680216	0.899607
ACOT7	14.10033	14.57109	14.81582	13.38033	14.15857	14.79292	13.79661	14.00074
ACOT8	10.38651	10.81046	8.715025	9.98916	9.867278	10.67186	12.29536	9.550522
ACOT9	3.656464	4.551657	3.562326	4.368741	4.230895	3.896056	4.343731	8.431469
ACOX1	20.63162	19.33482	18.9013	20.17712	21.71057	19.27984	19.09873	18.69554
ACOX2	0.099645	0.168255	0.164006	0.045384	0.088585	0.089178	0.235993	0.168439
ACOX3	10.76812	10.44007	10.82349	12.24888	12.67585	10.97495	12.1321	11.16306
ACOXL	0	0.100082	0	0.016872	0	0	0	0.016699
ACP1	48.2223	48.98103	47.56886	42.94185	44.6784	45.90703	44.46	46.20862
ACP2	6.126384	6.085084	5.456906	7.27662	6.741228	6.410128	6.760177	7.066443
ACP4	0.102964	0	0.118628	0.035172	0.137304	0.034556	0.104509	0
ACP5	29.00533	26.29749	25.35172	27.38536	28.47419	26.52904	23.88528	31.44334
ACP6	13.94912	15.06644	13.63423	15.89182	12.48309	15.05133	13.11692	12.1059
ACP7	6.784095	3.752496	3.607458	4.199979	4.328125	4.562171	4.663779	4.234259
ACPP	30.18599	30.89539	42.03914	35.83641	28.17181	35.42207	32.02619	30.10407
ACR	0	0	0	0	0	0	0	0
ACRBP	0.333204	0.715664	0.25008	0.204875	0.466546	0.134192	0.135281	0.270358
ACRV1	0	0	0	0	0	0	0	0
ACSBG1	21.52686	16.71238	22.57417	19.19184	22.8903	24.19232	15.58539	14.96278
ACSBG2	0.02525	0.025581	0.24935	0.038813	0.075759	0.050844	0.012814	0.128045
ACSF2	21.65743	24.86964	21.0323	26.20009	28.35259	20.48933	24.73312	25.89427
ACSF3	3.40694	2.657467	3.394263	3.038179	2.814355	3.926027	3.141821	3.79181
ACSL1	36.29833	46.24888	34.90969	49.45074	43.64326	35.93417	34.0144	37.62257
ACSL3	37.199	40.73887	43.96589	42.99079	38.84175	46.3573	42.11807	39.07042
ACSL4	10.19881	12.01408	10.40481	12.53623	12.7274	10.18224	11.18319	13.53678
ACSL5	29.19279	35.02462	35.37596	40.61555	34.54672	34.19155	24.49186	26.36269
ACSL6	1.113357	0.997645	1.366689	1.709129	0.944923	0.897663	1.03551	1.318157
ACSM1	5.755518	19.17059	24.44814	13.42555	23.47188	7.951364	11.93712	42.50145
ACSM3	16.18981	24.39823	36.45386	30.93207	32.18778	26.10171	28.05091	19.77325
ACSM4	0	0	0	0	0	0	0	0.016287
ACSM5	1.067597	2.938708	2.615844	2.322258	0.392846	2.464135	1.809425	0.909139

ACSS1	9.346471	11.42914	8.336786	11.48329	9.724602	8.836933	11.61182	11.50114
ACSS2	24.40587	26.13638	29.42367	35.6025	34.4078	33.16118	28.61901	24.49576
ACSS3	25.21387	33.52128	33.91231	35.25805	32.048	32.09762	28.6221	26.69739
ACTA1	0.215081	0.261484	0.368161	0.073469	0.860434	0.750707	0.30563	0.261771
ACTA2	235.6361	297.7731	279.8034	292.5692	282.7409	254.9898	338.4524	260.7949
ACTB	806.2675	761.1382	744.0066	737.0255	892.6181	766.6625	928.7535	782.0099
ACTBL2	86.09576	97.90637	142.7233	97.81456	102.1204	139.3072	96.02795	99.49468
ACTC1	1.427048	3.007215	0.807979	1.852369	1.560441	2.088145	1.197388	1.022803
ACTG1	1448.807	1351.702	1450.619	1412.496	1431.265	1316.393	1491.444	1280.955
ACTG2	42.05626	65.42353	70.66617	70.14572	45.04538	47.30798	62.51663	40.32259
ACTL10	0	0	0	0	0	0	0	0
ACTL6A	22.11009	23.23383	21.77551	21.1401	20.81131	19.73078	24.20307	20.40691
ACTL6B	0	0	0	0	0	0	0	0
ACTL8	0	0	0	0	0	0	0	0
ACTN1	70.57497	51.76405	57.7733	56.73988	67.64966	52.79783	59.56124	59.7617
ACTN2	0.308355	0.726936	0.772995	0.729215	0.569345	0.662716	0.397245	0.44506
ACTN3	1.646421	15.9735	7.863142	1.315447	1.023332	1.077017	1.491732	1.066068
ACTN4	166.8631	141.7553	142.7934	148.2934	147.3742	135.2901	158.7994	157.45
ACTR10	28.42161	30.16985	31.15182	30.4305	29.42246	30.17598	29.10399	29.02115
ACTR1A	65.05253	59.13994	60.85433	59.98671	63.35511	63.43237	63.88865	60.22527
ACTR1B	12.49816	15.52451	13.01629	14.64793	15.02607	15.86201	15.06722	14.64305
ACTR2	65.18025	70.20263	63.99806	61.98163	63.91627	68.39794	65.14082	67.75199
ACTR3	76.62638	81.28485	79.87456	73.48692	78.23481	80.67708	83.34062	76.52967
ACTR3B	3.655463	4.229369	3.684677	3.956584	3.547772	4.192212	4.346973	3.883004
ACTR5	6.171401	5.345436	4.654981	4.911659	6.05335	4.594366	5.70914	6.23515
ACTR6	9.314912	11.73269	12.98661	9.242128	12.24869	13.70252	10.50682	10.45385
ACTR8	11.31422	11.78909	10.00247	10.56444	9.51136	11.13203	11.3793	10.97863
ACTRT2	0	0	0	0.050586	0	0	0	0
ACTRT3	0.165494	0.104791	0.122574	0.021199	0.165515	0.083312	0.167976	0.16785
ACVR1	27.80078	27.40219	30.49305	32.08391	26.27912	33.16613	29.21028	21.57276
ACVR1B	17.93307	16.45477	19.08737	19.55393	17.16364	17.55213	16.28474	19.23395
ACVR1C	0.099752	0.285348	0.069536	0.07817	0.246477	0.041355	0.047646	0.077367
ACVR2A	21.23691	19.78702	15.97121	16.25712	18.18986	17.2518	16.91275	18.18869
ACVR2B	0.861205	0.722075	0.630524	0.897754	1.009819	0.478394	0.738483	1.084301
ACVRL1	5.879865	6.824437	6.701837	7.780264	8.021324	6.82642	6.172536	9.947794
ACY1	6.0597	6.083076	7.042376	8.48168	7.889715	8.482013	8.719595	5.508882
ACYP1	5.146768	4.628436	5.767938	5.363147	5.292024	5.036368	5.282666	5.161383
ACYP2	3.56867	4.519382	5.245327	1.530865	3.652135	5.890931	4.696225	5.513383
ADA	4.658771	4.701403	3.590367	5.597949	4.732461	3.255826	3.690203	4.465675
ADA2	0.469092	0.402133	0.368222	1.010727	0.661626	0.593398	0.451712	0.670958
ADAD2	0	0	0.015179	0	0	0	0	0
ADAL	4.404995	3.512803	2.982624	3.329714	3.631319	3.326316	3.463973	3.372895
ADAM10	22.24993	21.7775	21.65	21.03543	25.68134	21.43288	23.6812	25.89895
ADAM11	0.421789	0.784491	0.230026	0.909633	0.566656	0.31058	0.30032	0.249014
ADAM12	3.391677	1.399146	1.769179	2.06759	2.891126	2.38514	3.121248	2.903679
ADAM15	17.54025	16.17921	16.01212	20.02761	21.60599	16.57112	19.28299	16.93145
ADAM17	19.23047	16.72756	17.22013	18.21087	17.18633	19.74438	18.36876	19.07831
ADAM18	0	0	0.018803	0	0	0	0.009663	0.057934
ADAM19	1.440053	1.168894	1.219554	1.410039	1.440239	0.9164	1.370572	1.646915
ADAM21	0.0113	0	0	0.01158	0	0	0	0
ADAM22	0.237436	0.469649	0.167484	0.243317	0.29966	0.108145	0.504947	0.183479
ADAM23	0.121146	0.231834	0.243704	0.321862	0.33656	0.316227	0.209492	0.250292
ADAM33	15.72829	18.77036	14.36431	18.33118	17.22181	16.17598	16.06115	20.34073
ADAM7	0	0	0	0	0	0	0	0
ADAM8	0.544644	0.327334	0.446695	0.340557	0.655504	0.250947	0.33731	1.310773
ADAM9	7.257679	8.293888	8.33995	6.859589	7.96192	7.367374	9.554959	11.39811
ADAMDEC	0.029359	0.049574	0.057987	0.010029	0.146815	0.02956	0.0298	0.079406
ADAMTS1	17.7991	20.86268	12.21475	15.53204	20.85211	15.997	17.92432	16.99857

ADAMTS1(	3.118624	3.106563	2.279156	3.195874	3.347887	2.764718	3.079135	3.16302
ADAMTS1:	1.022366	1.028053	0.930514	0.891318	1.632944	0.929488	1.788873	1.946159
ADAMTS1:	0.127964	0.047143	0.068929	0.143055	0.104711	0.128838	0.141691	0.224175
ADAMTS1	0.107572	0.129742	0.070821	0.073491	0.16394	0.149566	0.062392	0.072735
ADAMTS1!	1.037284	1.241567	0.726128	1.403849	1.694012	1.141316	1.203884	2.366004
ADAMTS1(	0.104502	0.129401	0.183467	0.297474	0.214838	0.187051	0.300531	0.459289
ADAMTS1	3.086446	3.227087	2.835028	2.799188	2.848704	2.849701	2.84546	3.422005
ADAMTS1	3.521683	3.320622	2.917025	3.215866	3.088721	2.773402	2.982971	2.743277
ADAMTS1!	0.766826	0.519919	0.664069	0.785821	0.61944	0.706736	1.101635	0.807656
ADAMTS2(	0.063138	0.102347	0.096645	0.119699	0.009472	0.117604	0.099332	0.105661
ADAMTS3	0.070942	0.093987	0.129337	0.078291	0.147359	0.07692	0.04985	0.143903
ADAMTS4	0.206766	0.195514	0.292671	0.409649	0.530767	0.131846	0.279823	0.433399
ADAMTS5	2.032438	1.719164	1.685218	1.827347	1.500555	1.317562	2.374309	2.012754
ADAMTS6	0.206995	0.237673	0.143091	0.339396	0.28983	0.218829	0.283635	0.335907
ADAMTS7	0.763619	0.655015	0.638474	0.949474	0.692437	0.615068	0.56322	0.779652
ADAMTS8	0.741943	0.45792	0.488466	0.664189	0.400872	0.343454	0.571296	0.467072
ADAMTS9	2.425219	2.449321	1.483695	1.891323	3.554397	1.693126	2.12487	2.711133
ADAMTSL:	0.813826	1.300323	0.821388	1.587143	1.387627	0.87353	1.197203	1.065399
ADAMTSL:	4.214325	3.733803	2.503721	3.64768	3.753082	3.059777	3.468046	3.763444
ADAMTSL:	4.129018	3.142527	3.96974	3.336134	3.429834	2.874634	3.509565	3.707903
ADAMTSL	8.013336	7.302768	9.406396	8.161312	9.767345	6.06071	7.705455	16.09372
ADAMTSL!	3.970556	3.541264	2.764041	4.202315	4.342136	3.42752	4.281171	4.522212
ADAP1	1.868983	2.678626	1.656624	2.644019	1.896579	2.203026	1.637913	3.106917
ADAP2	3.871906	3.365434	2.292786	3.470007	4.308231	3.286991	3.045375	3.629962
ADAR	12.42799	11.4071	10.87085	11.18625	12.40446	10.21022	11.24116	12.52419
ADARB1	9.213894	7.873649	7.04017	7.983305	8.310766	7.324414	7.956493	7.889868
ADARB2	0.088976	0	0	0.052103	0	0.012798	0	0
ADAT1	0.930341	0.856864	0.723863	0.832046	0.857152	0.857219	0.778329	0.857804
ADAT2	2.821796	3.786017	2.818913	3.003341	3.258016	3.597961	2.808831	2.928266
ADAT3	0.910786	0.825142	0.596742	0.897449	0.910904	0.599582	0.773336	0.905987
ADCK1	1.993294	1.562672	0.984228	1.665747	1.447698	1.935235	1.348795	0.938632
ADCK2	8.55197	6.831201	5.7214	7.466965	7.059995	6.748956	7.616543	7.931684
ADCK5	4.745494	4.226556	3.083341	4.394348	4.51343	3.737154	3.665505	4.585134
ADCY1	0.314791	0.228519	0.234988	0.190505	0.176008	0.067381	0.143404	0.183519
ADCY10	0	0	0.007906	0.004102	0.004004	0.008061	0	0.00812
ADCY2	2.674034	2.008615	1.81854	3.188547	1.771159	1.946413	1.647106	2.239809
ADCY3	3.678411	2.965241	3.461296	3.991699	4.640167	3.259697	3.557544	3.672138
ADCY4	3.03418	3.007607	2.284492	3.801048	4.384728	3.615744	3.092996	3.403057
ADCY5	0.321019	0.674032	0.330803	0.719922	0.614203	0.337265	0.325835	0.311433
ADCY6	4.316436	5.20296	3.566278	4.930654	4.719486	4.094921	4.392027	5.150875
ADCY7	19.0395	17.69538	16.44542	16.18597	16.70412	17.49084	16.5294	15.30253
ADCY8	0.0656	0.029538	0.043188	0.044816	0.043739	0.007339	0.051787	0.022178
ADCY9	1.730872	1.531194	1.450169	1.538454	2.026341	1.35402	1.659518	1.883465
ADCYAP1	0	0	0	0	0	0	0	0.04575
ADCYAP1F	0.926617	0.727665	0.599832	0.972287	1.081932	0.763321	0.904516	1.25008
ADD1	44.88984	43.78064	37.95977	43.92487	42.73812	38.90884	45.36794	45.92605
ADD2	0.053898	0.115636	0.115847	0.087724	0.228305	0.076612	0.199519	0.209016
ADD3	27.20342	31.13666	34.96495	32.8366	33.82709	28.70778	33.16398	34.00234
ADGRA1	0.034597	0.011684	0.045555	0.070908	0.046136	0.034834	0.081938	0.07018
ADGRA2	11.5943	13.24956	11.13904	13.37116	13.44933	11.51256	12.41721	12.69665
ADGRA3	36.77287	38.24494	32.94508	34.54818	28.11643	32.19458	35.53635	35.89829
ADGRB1	0.330891	0.31927	0.202285	0.683557	0.430739	0.290846	1.007563	0.261023
ADGRB2	0.795208	0.646708	0.422032	0.637511	0.438233	0.615458	0.521619	0.438927
ADGRB3	0.055378	0.018702	0	0.004729	0	0.004646	0.009368	0
ADGRD1	2.19037	2.686035	2.862897	2.732147	2.438465	1.841104	2.761427	2.905106
ADGRE2	0.059371	0.094522	0.050255	0.026075	0.135723	0.051237	0.06887	0.197853
ADGRE3	1.019553	0.442686	0.587329	0.398022	0.728346	0.549921	0.591341	0.910964
ADGRE5	6.134366	6.957759	5.430944	6.918614	10.03447	6.869529	7.297392	12.47966

ADGRF1	0	0.242149	0.048835	0.498311	0	0.041491	0.209136	0.050155
ADGRF2	4.460748	3.426444	4.629429	4.421638	3.62584	3.780792	3.951411	4.055373
ADGRF3	0.062697	0.042347	0.025798	0.048188	0.078382	0.052605	0.015909	0.06359
ADGRF4	23.09975	21.49702	21.70731	22.76014	20.41617	25.23692	20.62512	19.72436
ADGRF5	12.04234	11.31975	11.33085	12.33582	13.35496	14.35274	9.881392	12.61883
ADGRG1	80.29287	70.87109	74.06909	85.05301	78.40206	86.62322	68.93563	70.19056
ADGRG2	1.552858	2.108266	1.608012	2.02942	1.477606	1.253305	1.56977	1.906535
ADGRG3	0.115658	0.068352	0.076144	0.138276	0.260263	0.029112	0.088044	1.104614
ADGRG5	0.068322	0.069218	0.052477	0.101132	0.083515	0.107004	0.107873	0.284877
ADGRG6	7.222175	6.261376	5.425829	5.630385	5.566195	5.473182	7.63923	7.777876
ADGRG7	0.008068	0.065394	0.023904	0.057878	0.072625	0.016247	0.073705	0.049099
ADGRL1	4.232579	4.462129	2.909215	4.787869	4.474769	3.977199	4.402435	4.744554
ADGRL2	23.39653	21.88528	20.73621	20.07388	22.40992	19.2965	19.57426	21.25523
ADGRL3	3.576322	2.635491	3.241855	3.177425	3.396766	2.899588	2.773042	3.542474
ADGRL4	3.988108	3.289918	2.904306	3.327737	4.194739	4.183594	3.137711	3.688977
ADH5	50.24177	54.62376	50.20817	53.77242	55.10435	56.81375	49.45412	45.95636
ADH7	0	0	0	0	0	0	0	0
ADHFE1	2.937849	2.16999	1.815063	2.669516	2.286897	1.850521	2.438415	2.040268
ADI1	87.33781	92.58542	95.6607	83.48682	86.24489	84.3071	81.70783	83.80729
ADIG	0.354053	0.941587	0.39959	0.69973	0.663935	0.636557	0.404284	0.37833
ADIPOQ	7.010645	109.3673	0.870357	25.22032	42.46997	5.525827	7.929039	14.16672
ADIPOR1	12.80566	11.97247	11.48677	11.71728	11.92673	12.00063	11.79402	11.3599
ADIPOR2	18.27386	21.40645	19.41987	24.82613	24.12666	20.69116	18.05008	17.49443
ADIRF	30.99019	53.76565	35.80553	35.17839	52.31036	41.12978	49.42047	42.76776
ADK	32.01173	36.03697	36.80759	31.27686	32.466	38.0081	33.2628	28.25922
ADM	7.320243	8.46934	5.894667	6.527734	6.652927	7.833695	8.03289	8.373215
ADM2	1.696313	2.313467	3.157064	4.479546	5.220099	4.401131	1.787979	1.323432
ADM5	0.385101	0.343337	0.410727	0.410426	0.508399	0.527316	0.484689	0.828037
ADNP	17.96957	18.58975	15.77541	17.32819	18.18193	16.1552	17.90561	19.56522
ADNP2	7.142803	7.872033	6.395299	7.881453	7.714546	7.709349	8.133246	8.04115
ADO	15.63941	19.83195	21.04124	17.28959	18.08998	16.55515	18.79975	19.28111
ADORA1	0.175591	0.231264	0.164733	0.377874	0.333666	0.309383	0.338627	0.427417
ADORA2A	0.169029	0.070513	0.078552	0.132459	0.139218	0.080086	0.131196	0.272279
ADORA2B	5.965415	5.317829	5.137395	5.554534	5.810411	5.802303	4.995684	7.3931
ADORA3	2.207797	1.683592	1.969292	1.605634	1.353341	0.908273	1.927668	3.87651
ADPGK	9.572702	10.00242	9.167598	8.788193	11.36436	9.055279	10.2712	9.795954
ADPRH	2.776012	3.601414	2.705232	2.23451	3.49108	2.88722	2.696763	2.072156
ADPRHL1	0	0	0	0.060005	0.019521	0	0.019811	0
ADPRHL2	8.081978	5.972964	6.519441	7.410363	6.789739	6.47549	6.156723	6.74745
ADPRM	4.338972	4.37705	2.979206	3.56861	4.48853	3.674886	4.574171	4.532951
ADRA1A	0.67009	0.957402	0.639117	0.704296	0.876385	0.542041	0.778966	0.789997
ADRA1B	0.496582	0.304909	0.282348	0.339255	0.436447	0.499974	0.931693	0.35103
ADRA1D	0.192498	0.448555	0.503763	0.591798	0.279158	0.261647	0.429847	0.712619
ADRA2A	2.477257	2.879424	1.270606	2.243431	3.812396	2.010812	1.978397	2.697553
ADRA2B	0.762501	0.733492	0.676939	0.481461	0.770302	1.108914	0.828661	1.21862
ADRA2C	0.057621	0.45242	0.142256	0.221429	1.094931	0.24656	0.336288	0.452916
ADRB1	1.663022	2.256675	1.881181	1.828159	1.79428	1.948373	2.046021	1.932034
ADRB2	2.71653	3.293594	2.902573	3.331456	3.050924	2.264294	3.5483	4.471556
ADRB3	0.142077	0.086365	0.098215	0.218394	0.184724	0.085829	0.072104	0.1441
ADRM1	44.32262	41.24225	42.58685	40.66651	43.11043	41.56994	44.88936	42.99311
ADSL	20.00845	18.9262	18.95982	21.03491	17.85774	19.11832	18.81336	19.94849
ADSS	7.073705	7.387729	6.349534	8.166225	8.177299	7.770485	7.866767	7.340477
ADSSL1	4.103007	6.778295	4.307407	7.083493	3.22368	5.694133	6.145529	4.408852
ADTRP	12.81182	14.76427	22.98537	26.76263	26.70625	19.23602	11.48803	12.74903
AEBP1	42.42176	39.52454	45.50503	43.33042	43.52242	43.18198	41.83548	46.90545
AEBP2	11.7292	12.76945	12.319	12.45139	12.8217	13.38065	12.02025	12.02763
AEN	7.070473	6.843884	7.160268	6.676423	7.221522	6.242153	4.243447	5.816072
AES	146.8554	148.9604	147.7273	159.5148	154.2234	147.0641	135.4946	123.4043

AFAP1	3.271641	2.893233	2.176734	2.791533	3.674139	1.807508	2.968945	3.226823
AFAP1L1	2.309785	2.01185	1.950724	2.099239	2.236914	1.915171	1.803409	1.727849
AFAP1L2	34.48888	39.76308	43.03085	38.51925	38.23942	43.32427	30.79443	37.39328
AFDN	48.11507	37.13305	39.81268	41.84803	41.72366	41.59596	41.29506	44.32519
AFF1	10.62947	10.53447	9.313108	9.977435	10.39639	9.50998	10.15339	12.00875
AFF2	0.423734	0.498289	0.261534	0.418722	0.257301	0.331399	0.506892	0.514185
AFF4	11.7115	11.45736	11.24199	11.36602	11.94474	11.40976	11.40969	11.61948
AFG1L	1.214178	1.597549	1.167905	1.244253	1.308958	1.285978	0.992314	1.887176
AFG3L2	37.44169	37.1473	31.88174	37.15706	36.23451	35.51098	39.88281	35.27064
AFM	0	0	0	0	0	0	0	0
AFMID	1.038608	0.930827	1.144013	1.159852	1.38499	1.783059	1.770494	0.688757
AFP	0	0	0	0	0.030361	0.030564	0	0
AFTPH	21.36467	21.97924	20.45377	20.71081	21.07191	22.66607	21.37127	20.6373
AGA	7.706758	11.76823	10.67426	10.07878	7.63819	9.398127	12.9196	8.62069
AGAP1	11.16041	9.229724	8.107977	9.136178	9.454639	9.489977	9.237398	9.804404
AGAP2	1.179408	1.102663	0.805137	0.949052	1.128103	0.872667	0.940004	0.955352
AGAP3	17.01699	14.00442	12.59937	14.89577	15.17999	13.42133	14.27148	17.10753
AGBL1	0	0	0	0	0.004677	0.004709	0	0
AGBL2	0.087576	0.301665	0.322875	0.173507	0.256922	0.241009	0.290372	0.225017
AGBL5	2.158533	2.410582	1.85905	2.291203	1.866186	2.084347	1.809883	2.13327
AGER	0.490279	0.452886	0.156643	0.265989	0.432655	0.217777	0.204908	0.263254
AGFG1	14.38206	15.99252	16.22987	15.94742	17.07916	17.23579	15.75286	15.64186
AGFG2	8.50755	7.857824	7.821143	10.7226	8.190659	9.671543	8.400459	7.397391
AGGF1	13.58909	13.33094	13.46982	11.92073	12.93183	13.20221	11.85852	12.80578
AGK	5.502143	6.18444	5.034417	6.678205	6.567125	5.290568	5.676777	6.074095
AGL	3.053487	4.450447	2.702454	2.951015	2.999348	2.875338	4.233851	2.872288
AGMAT	0.017077	0.069204	0	0.0175	0	0	0	0
AGMO	2.370958	2.57022	2.481901	2.526875	1.553179	2.076825	1.636436	1.923768
AGO1	15.84714	13.33001	12.72028	13.67348	13.71222	11.78251	13.73922	15.71539
AGO2	7.928779	6.180493	6.30776	6.239374	7.818981	6.239726	6.965176	7.367332
AGO3	3.107177	2.675016	2.41336	2.605055	3.107579	2.726016	2.43558	2.99359
AGO4	10.00856	10.71745	9.591637	10.27878	9.66604	9.643191	10.4767	11.43096
AGPAT1	5.548608	4.571511	4.328143	5.473652	5.665926	4.803967	4.492321	5.307896
AGPAT2	35.69504	29.72817	29.31483	32.18349	30.24537	32.98327	27.50998	26.77059
AGPAT3	15.66089	16.91896	21.05017	21.85629	22.74465	21.25629	15.98265	14.47479
AGPAT4	0.170406	0.187029	0.042071	0.451119	0.298249	0.185868	0.28827	0.302455
AGPAT5	12.57393	14.31343	11.60339	12.53953	12.15951	12.46227	12.06944	12.25937
AGPS	4.645566	4.884354	4.38485	4.354989	5.325912	4.605865	4.33242	5.204079
AGR2	0.052562	0.532516	0.674789	0.727161	0.315412	0.502748	0.693553	0.15993
AGRN	16.21828	15.30824	11.5688	16.03055	15.00315	12.41278	12.62741	15.4033
AGRP	0	0	0	0	0.022593	0.022744	0	0
AGT	2.286477	0.694945	0.265646	0.289443	2.246417	0.23021	0.286684	0.695708
AGTPBP1	2.749724	3.935917	3.574943	3.44474	3.109608	2.863756	4.173652	3.31338
AGTR1	6.272593	4.948196	6.699613	7.95071	6.175953	7.983213	7.825456	8.659579
AGTR2	0.018697	0	0	0.01916	0.046748	0	0.009489	0
AGTRAP	13.83658	14.84142	12.05925	15.54405	14.67364	11.54486	13.28808	13.39254
AGXT	0.249599	0.034483	0.089632	0.011626	0.068081	0.034269	0.069094	0
AGXT2	0	0	0	0.015184	0.029638	0	0.015039	0
AHCTF1	11.49838	10.97013	9.550281	10.21404	10.86119	9.98906	10.06652	11.53683
AHCY	227.6271	343.9093	376.2189	227.9215	295.8978	403.15	237.6611	238.2958
AHCYL1	31.45578	32.31144	25.11006	30.45871	26.71148	28.63563	32.47836	29.41182
AHCYL2	20.04658	24.56314	18.10708	22.25326	18.38017	17.98759	20.90474	17.81456
AHDC1	3.217949	2.912901	2.065605	2.899789	3.291828	2.5356	2.446114	3.423399
AHI1	2.931145	2.92377	2.16476	2.467918	2.066649	2.232359	2.536245	2.376257
AHNAK	198.8201	176.5902	172.1834	167.0465	178.8717	151.0453	194.5703	213.8181
AHNAK2	9.655993	7.830802	7.641108	9.684533	8.433566	8.353755	7.621766	7.87745
AHR	12.32004	15.08016	14.79545	17.48874	19.06074	16.5716	14.30038	14.32445
AHRR	0.266021	0.452395	0.4785	0.457595	0.361075	0.229575	0.395373	0.423983

AHSA1	80.5394	74.52315	78.09088	72.40234	84.04106	71.00865	69.9935	84.2724
AHSA2P	4.359968	3.073498	3.522798	3.780586	4.634971	3.990685	4.255161	5.086886
AHSG	0.866595	0.504365	0.145668	0.623532	0.516336	0.705438	0.149718	0.336612
AHSP	0.096737	0	0	0	0.03225	0.032466	0.065459	0.065409
AICDA	0	0	0	0	0.046193	0	0.04688	0
AIDA	12.22972	12.33986	10.9678	11.97225	11.59488	9.720477	11.62599	12.23239
AIF1	7.581252	8.469582	8.741332	9.196867	9.426559	7.880602	11.48007	13.79895
AIF1L	5.970325	6.921594	6.317072	10.20297	9.200364	8.13165	6.528064	6.13476
AIFM1	7.71703	8.459748	7.278761	8.630447	7.586606	8.010298	9.251416	7.633032
AIFM2	2.466894	3.536702	2.865165	3.656855	3.11893	3.499115	3.511756	3.509111
AIFM3	0.246146	0.104577	0.211714	0.244106	0.357355	0.159889	0.209542	0.185224
AIG1	6.797736	7.526079	7.768304	5.950226	7.764327	7.427486	6.703688	6.724755
AIMP1	20.51437	23.42842	23.80274	22.95952	21.76629	24.21449	19.91649	21.52364
AIMP2	26.59083	26.18577	23.54224	24.98448	28.26346	25.02399	24.54025	22.89766
AIP	11.60891	12.51872	11.18235	11.17209	14.80531	12.57784	12.23836	11.51222
AIPL1	0	0	0	0	0	0	0	0.027348
AIRE	0	0	0.02942	0	0	0	0	0
AJAP1	0	0.068356	0.011105	0.138284	0.044986	0.022644	0.114138	0.102647
AJM1	0.009504	0.009629	0.028157	0.024348	0.061784	0.009569	0.019293	0.033737
AJUBA	49.89845	42.80973	43.81939	44.90397	44.75805	42.57663	37.58848	41.81061
AK1	13.26822	14.61978	14.20277	15.92919	16.0789	16.1379	15.72817	12.62217
AK2	72.84287	79.32621	79.63807	74.88016	82.37267	84.82722	77.36766	72.30915
AK3	13.64932	16.26069	16.07949	16.51375	15.98112	16.46508	16.06153	16.08217
AK4	12.98282	15.58229	18.0771	25.31933	25.63738	17.91662	19.96052	14.0817
AK5	0.076427	0.068826	0.008386	0.043511	0.059451	0.085499	0.11205	0.025838
AK6	7.942871	11.27234	8.995585	6.104714	7.628664	8.25101	5.918542	6.105893
AK7	0.069944	0.094482	0.046048	0.095568	0.069953	0.105632	0.047329	0.059116
AK8	1.301457	1.127905	1.223301	1.397969	1.317307	1.610307	1.846187	1.956121
AK9	0.042902	0	0	0.026379	0.004291	0.004319	0	0.017405
AKAP1	12.41049	13.70791	11.41593	13.48054	13.90778	12.90203	13.08584	12.98253
AKAP10	6.216228	6.673847	6.014468	5.665713	6.888445	6.482916	6.02774	6.53059
AKAP11	12.44576	12.7091	10.74252	13.06047	11.96829	11.62758	13.93783	13.839
AKAP12	8.371901	9.349962	7.320652	7.684785	9.319245	6.956889	8.811123	10.29075
AKAP17A	10.7092	9.848098	9.020504	10.72342	10.92935	11.31972	10.59454	10.51557
AKAP2	10.64835	8.981896	9.817821	8.860326	11.19249	8.933085	10.75417	10.71094
AKAP3	0.021901	0	0.010814	0.022443	0	0	0.011115	0.011106
AKAP4	1.389015	1.052788	1.139651	0.915056	1.253408	1.671895	1.054737	0.958611
AKAP5	0.303656	0.367832	0.306391	0.669709	0.369716	0.319023	0.428815	0.301284
AKAP6	0.26141	0.214521	0.216848	0.30271	0.316347	0.247404	0.209612	0.156427
AKAP7	1.280261	1.306009	0.776024	1.339118	1.32458	1.297897	1.236733	1.07461
AKAP8	15.0283	14.5694	12.17621	14.21786	14.36592	14.06929	13.94746	13.36432
AKAP8L	20.45696	18.23545	16.01888	20.85998	20.99459	18.55887	17.3741	20.98278
AKAP9	6.91697	7.26013	7.251528	7.126479	7.704884	6.600893	6.666261	7.2374
AKIP1	8.408363	10.5425	8.46416	8.259598	9.989126	8.512578	9.265343	10.01223
AKIRIN1	22.39133	29.59214	30.48166	26.05458	30.02578	28.14889	21.48927	27.34091
AKIRIN2	65.26232	57.91767	63.41036	57.92733	57.76942	59.03144	65.35927	59.65109
AKNA	1.990818	2.836168	1.612231	2.632766	2.467091	1.684948	2.540136	3.451775
AKNAD1	0.077546	0.093294	0.134014	0.1341	0.096945	0.136632	0.088548	0.103228
AKR1A1	26.52002	36.56194	31.67673	36.5962	34.11503	33.43338	33.72374	30.0305
AKR1B1	4.323039	6.111313	4.964155	5.316147	6.67644	5.891156	5.102191	4.710875
AKR1D1	0	0	0	0	0	0	0	0
AKR1E2	4.479757	4.636721	2.29662	3.338013	2.847734	2.011276	2.088118	4.573769
AKR7A2	11.46185	11.69278	17.71419	13.61857	14.54274	8.660115	9.536876	10.47662
AKT1	49.22582	50.03148	48.42352	54.07804	55.63664	49.55077	53.17407	54.58365
AKT1S1	6.026437	5.57261	6.348958	6.974298	6.754639	6.008762	5.549972	5.249402
AKT2	15.31846	12.99943	12.43592	13.20051	13.19463	12.36062	13.48381	14.52832
AKT3	9.941055	9.184335	9.61051	9.566466	10.26733	9.787162	9.59265	11.25561
AKTIP	42.43775	39.98197	43.73597	45.90823	38.44751	44.06469	45.14796	43.67501

ALAD	5.908134	6.409029	5.242077	8.004803	7.166814	7.510601	6.483888	6.009025
ALAS1	39.7044	39.0487	47.91745	39.51038	38.74771	43.88644	44.1791	40.02893
ALAS2	0	0	0	0.014435	0	0	0	0.028573
ALB	0.353949	0.648227	2.98452	0.864938	0.748834	1.439173	0.842874	3.424186
ALCAM	25.86628	32.96277	33.69429	34.16186	27.15828	33.79816	34.03204	26.65476
ALDH16A1	5.687997	5.403088	5.730792	6.910135	5.602394	5.70754	6.454821	5.875982
ALDH18A1	13.91824	15.25698	12.24495	13.45597	11.58128	12.41943	13.4174	13.63551
ALDH1A1	17.42087	36.62202	35.97641	32.33848	19.87366	31.60973	39.28614	23.1443
ALDH1A2	0.557325	0.323837	0.526099	0.470342	0.32788	0.412597	0.183016	0.382381
ALDH1A3	26.10033	24.43811	25.36329	20.35341	22.13744	23.75944	19.66139	20.64176
ALDH1L1	0.012028	0.56055	0	0.172563	0.096236	0	0.097667	0.085395
ALDH1L2	6.143087	6.35716	6.906162	5.734857	5.876408	5.341089	6.389189	6.748737
ALDH2	242.3132	259.4432	244.9866	219.6049	210.2669	223.9164	231.168	222.1964
ALDH3A1	0.725108	1.046839	0.769778	1.337524	0.543901	0.492792	0.717585	0.625116
ALDH3A2	58.65867	86.54612	85.39742	84.93352	82.12534	75.06475	85.27715	74.15104
ALDH3B1	1.261296	2.026064	1.401278	2.568071	2.381835	1.22814	1.827677	2.802571
ALDH4A1	12.79601	9.406557	12.06976	10.26722	13.09313	12.78995	16.38058	10.07606
ALDH5A1	11.20584	14.86812	15.29626	13.406	12.4337	14.90664	16.18046	13.29111
ALDH6A1	20.7823	21.5304	25.16303	22.79083	20.59728	23.34927	22.87833	22.01087
ALDH7A1	32.20349	39.86409	33.78365	38.59314	34.32348	34.22385	33.01034	32.38095
ALDH8A1	0.215117	0.048431	0.070812	0.061235	0.011953	0.024065	0.036391	0
ALDH9A1	42.52559	39.94139	35.52538	34.01814	37.26142	39.96923	43.4922	34.96969
ALDOA	432.2314	392.8111	410.8194	433.114	435.7971	355.4002	413.2945	364.2295
ALDOB	0.115717	0.1612	0.199982	0.163053	0.057866	0.24758	0.058727	0.2494
ALDOC	4.078972	4.448077	3.969556	5.563213	4.687716	3.509479	5.841385	4.046776
ALG1	5.605525	5.66935	4.69061	6.020263	4.981196	6.127852	4.879595	4.329817
ALG11	6.925872	7.383842	6.591864	6.647592	6.81527	5.91388	6.527635	6.572187
ALG12	7.224797	6.397648	5.954763	6.957748	6.489704	7.505206	7.630351	6.356513
ALG14	19.33047	16.63548	23.71231	19.80929	22.94729	22.12428	19.62043	19.28586
ALG2	4.352382	5.169294	3.967524	4.295508	4.339551	4.624817	5.6818	5.663939
ALG3	6.658556	6.726333	5.409569	6.387105	7.975814	5.437292	6.385149	6.9104
ALG5	13.4101	18.47976	13.65685	15.79909	17.4045	17.12141	17.7976	15.56956
ALG6	4.186809	5.167444	4.328809	4.468301	4.684743	4.692847	4.226133	3.835846
ALG8	3.539863	4.657304	4.654623	4.442502	3.821606	3.466401	3.48468	3.698455
ALG9	4.209731	4.618165	3.938198	5.163818	4.770236	4.509711	4.24071	5.142877
ALK	0.442903	0.163169	0.090885	0.335984	0.132313	0.104243	0.157633	0.169182
ALKAL1	0.102713	0	0	0.210514	0	0	0	0
ALKAL2	1.129246	0.354116	0.531036	0.661267	1.290733	0.622621	0.982444	2.399721
ALKBH1	4.332842	4.080661	3.448904	3.604447	4.476604	3.654172	3.936568	4.205104
ALKBH2	0.992817	1.324365	1.127513	1.373503	1.357025	1.074569	1.066492	1.384557
ALKBH3	11.13243	10.58732	9.153217	13.59092	8.637488	8.410551	12.71818	11.34154
ALKBH4	6.754581	6.131699	5.40569	5.8635	6.09437	5.719264	5.031853	6.033676
ALKBH5	17.6225	17.78618	17.31232	17.04197	19.12618	17.9864	17.5059	17.26434
ALKBH6	6.046153	5.729637	5.219388	7.249646	7.013621	5.901107	6.053351	5.610776
ALKBH7	15.86416	15.15289	19.61	17.70798	20.29568	14.76637	11.86472	14.06494
ALKBH8	2.868432	3.462087	3.540864	2.632038	3.300587	3.241664	3.097138	3.310032
ALMS1	3.610107	3.653303	2.568357	3.64665	3.199765	2.928182	2.970794	3.552638
ALOX12	5.721625	5.758479	7.387177	7.54191	6.794365	5.145122	7.983328	5.841355
ALOX12B	2.366602	2.041573	2.394963	3.061544	2.437212	3.066931	2.069136	1.414033
ALOX15	0.304748	0.356989	0.931065	0.975926	0.771492	0.086296	0.734632	0.598854
ALOX5	2.468432	2.779846	1.732562	2.895424	1.948477	1.0886	1.863564	4.220898
ALOX5AP	3.897614	5.689954	6.910104	6.258564	6.752645	3.677044	5.233237	15.84352
ALOXE3	7.081377	6.225719	4.443652	4.955853	4.19119	4.951472	4.613349	4.79427
ALPK1	1.807451	2.666755	2.010212	3.410726	2.340045	2.343947	2.368901	2.26033
ALPK2	0.117119	0.142388	0.113347	0.153626	0.067938	0.169804	0.159294	0.035636
ALPK3	0.335839	0.319626	0.256279	0.461485	0.480923	0.302271	0.382195	0.438678
ALPL	0.736292	0.817485	0.38846	1.178307	1.028924	1.198301	1.371822	1.452627
ALS2	10.06271	9.894296	10.66955	11.05443	9.703826	8.467763	10.35127	10.56693

ALS2CL	3.683218	3.731557	2.856736	4.185086	4.067984	4.089719	3.682752	3.796891
ALS2CR12	0	0.009503	0.009263	0.009612	0	0.018888	0	0.009513
ALX1	0	0	0	0.083331	0	0.049123	0	0
ALX3	0.870053	0.552787	1.092219	1.16362	0.575194	0.579049	1.062718	0.493567
ALX4	0.679804	0.522481	0.462989	0.624576	0.281334	0.212415	0.356897	0.404179
ALYREF	36.62842	43.12337	41.41073	42.4283	37.82057	45.50062	45.61337	37.55978
AMACR	5.921626	5.585594	4.522715	4.857635	4.857528	4.992875	5.211031	4.497047
AMBN	12.03911	10.69336	13.28508	24.89194	13.38375	19.5697	9.182708	20.38269
AMBP	0.308771	0	0.035873	0.037226	0.490464	0.292593	0.055306	0.092108
AMBRA1	9.508918	8.809251	8.932603	8.748784	8.775182	8.441698	8.214832	9.314137
AMD1	20.31175	21.56458	21.57605	22.21691	24.74564	22.15118	20.87813	23.27474
AMDHD1	0.828674	0.926399	0.437393	1.347007	0.342944	0.863105	1.015125	1.637468
AMDHD2	12.40342	11.53682	10.46928	14.45911	11.88735	14.12879	13.40671	10.28565
AMELX	0.445194	0.098051	0.07646	0.178522	0.367817	0.409259	0.11788	0.21595
AMER1	3.509272	3.627674	2.800645	3.613621	3.387293	3.064201	3.192604	3.717877
AMER2	0.168673	0.080103	0.161366	0.097229	0.089619	0.05307	0.107002	0.160382
AMER3	0	0	0	0.005268	0	0	0	0
AMFR	41.44831	44.1075	42.00461	49.20942	42.81748	44.55493	45.76094	45.85931
AMH	0.015575	0.01578	0.030763	0.031923	0.031155	0.031364	0.015809	0
AMHR2	0.223794	0.332539	0.27994	0.443386	0.462567	0.375538	0.575449	0.30264
AMIGO1	3.214458	3.357305	2.810792	2.898792	2.612816	2.959851	3.191489	2.62003
AMIGO2	4.894631	5.402784	4.865304	4.523046	3.762298	5.498347	4.837883	5.78808
AMIGO3	0.262071	0.51332	0.293313	0.617694	0.541683	0.439769	0.46107	0.478443
AMMECR1	9.225362	9.869794	9.429895	9.175822	9.231773	10.44418	9.861589	9.711272
AMMECR1	19.60602	16.1472	16.97803	18.80032	19.60856	18.04235	18.41091	17.30498
AMN	0.055351	0.07477	0.036441	0.018907	0	0.018576	0.037454	0.037426
AMN1	4.255509	5.841624	3.956042	4.360919	4.991674	4.906109	4.719281	5.221935
AMOT	6.346129	7.045386	5.641771	6.116222	6.328959	5.958447	6.105379	6.586071
AMOTL1	29.21048	24.89435	22.30542	25.7256	25.5647	23.86311	25.12744	27.90238
AMOTL2	22.81725	23.05113	22.76946	22.46705	21.05933	18.35895	20.78115	19.09795
AMPD1	0.011721	0	0	0	0	0.035402	0.011897	0.011888
AMPD2	5.565132	5.178036	4.556901	5.665749	6.256284	4.109384	5.372018	6.442796
AMPD3	3.496308	2.689794	1.747913	2.631302	3.004347	3.2441	2.561934	3.533439
AMT	4.870688	5.014685	4.019714	5.841787	5.958224	4.605546	4.582744	4.899944
AMTN	0	0	0	0.026983	0	0.02651	0	0.05341
AMZ1	0.192881	0.065137	0.179896	0.175697	0.10717	0.226565	0.152269	0.086945
AMZ2	7.824206	8.641331	6.566049	8.551401	6.297117	5.89979	9.06634	8.173996
ANAPC1	10.09669	9.284278	8.637087	8.19177	9.288394	7.104891	8.805808	9.892381
ANAPC10	2.913438	3.128032	3.69142	3.370542	2.620601	4.141731	3.208218	2.806238
ANAPC11	24.00117	27.05914	25.25575	23.73333	24.99225	26.92791	25.58667	25.67873
ANAPC13	12.59106	11.43739	12.63504	13.44491	14.46429	12.90235	16.3104	14.66831
ANAPC15	42.95717	42.70124	43.96998	46.32723	42.45698	45.18533	44.57673	33.542
ANAPC16	32.27783	35.21694	37.35543	37.22167	34.03155	37.80109	35.91219	33.42412
ANAPC2	11.63582	13.45526	11.24338	12.92888	12.86862	10.98036	12.81667	12.29872
ANAPC4	8.346583	9.209726	8.49883	9.368778	7.811336	9.814364	8.787828	9.711088
ANAPC5	11.45106	11.66497	11.78387	11.71326	11.2013	9.906339	12.51526	10.95588
ANAPC7	13.85719	13.60953	12.82391	14.55444	13.25437	12.91633	15.97757	13.4015
ANGEL1	2.54116	2.685162	2.372876	3.230881	2.31574	2.243287	2.194972	1.949617
ANGEL2	12.01013	12.34457	11.18384	11.57897	12.33493	11.22008	10.72063	12.81704
ANGPT1	0.895552	0.894527	0.678866	0.749699	1.116431	0.768325	0.812966	0.652442
ANGPT2	1.848791	1.235829	1.562243	1.943415	1.725126	1.573572	1.876522	2.136078
ANGPT4	4.067854	3.773428	2.603483	3.92965	5.759219	4.154339	3.491132	3.179727
ANGPTL1	3.146901	3.035864	3.033445	3.79718	5.102292	3.579318	2.703543	4.912821
ANGPTL2	13.42199	9.306123	15.07371	12.37572	10.21196	10.54647	15.63105	13.28816
ANGPTL4	12.39604	13.09526	9.549211	10.09012	11.94646	9.103821	11.20832	15.09895
ANGPTL5	2.23857	2.182366	1.821375	1.558202	2.182536	1.956183	2.900919	4.355242
ANGPTL7	0.138789	0.04687	0.106602	0.110621	0.123384	0.031053	0.070436	0.156406
ANGPTL8	0	0	0.013569	0.014081	0	0	0	0

ANHX	0	0	0	0	0.024555	0.02472	0	0
ANK1	0.073395	0.107819	0.105097	0.17299	0.099096	0.184741	0.145267	0.119104
ANK2	1.61499	2.182737	1.835027	1.946329	2.531563	1.795005	2.291077	2.344935
ANK3	2.836798	2.655219	2.478141	2.975571	3.027451	2.871709	3.195991	2.793734
ANKAR	0	0	0	0	0	0.042967	0	0
ANKDD1A	0	0	0	0	0	0	0	0
ANKDD1B	0.012693	0	0	0.013008	0	0	0	0.012874
ANKEF1	0.011679	0.14199	0.011534	0.017953	0.058403	0.035277	0.035563	0.023691
ANKFY1	11.12031	10.07684	8.958225	9.627837	10.39919	9.067383	10.77507	11.74605
ANKH	21.33582	23.54282	20.78785	21.45599	19.14306	25.59925	20.80122	18.36845
ANKIB1	7.608316	7.915643	6.946374	7.88802	8.517607	7.897856	7.062712	7.24252
ANKLE1	0.424408	0.352802	0.300907	0.457224	0.359161	0.361568	0.463911	0.496673
ANKLE2	13.70474	15.13954	11.89496	13.88789	13.79806	13.08572	13.97844	14.51252
ANKMY1	0.101074	0.170668	0.116451	0.198524	0.193751	0.161128	0.042746	0.111056
ANKMY2	13.56064	13.35393	16.47712	16.16446	16.01448	15.18245	15.669	14.5349
ANKRA2	11.70279	10.96715	10.01604	10.63635	9.168368	10.32392	12.26013	11.20526
ANKRD1	0.354497	0.048975	0.127302	0.082563	0.14504	0.178459	0.016355	0.049029
ANKRD10	17.57701	16.76569	15.61193	17.75186	17.50994	15.55622	15.70589	18.28434
ANKRD11	25.32191	22.01574	19.81771	23.60222	22.20938	21.23486	22.05337	23.90816
ANKRD12	13.1362	12.25608	12.13435	12.24448	13.85446	12.03846	12.30395	14.0331
ANKRD13/	36.89448	39.62873	40.08539	41.01096	38.10028	41.30548	42.27941	36.6713
ANKRD13E	3.466344	3.087527	2.815961	3.360442	3.030101	2.359579	2.677193	3.669331
ANKRD13(	12.30919	12.8185	14.90833	12.80404	13.00425	13.87246	12.86314	13.02056
ANKRD13[	5.438409	5.197171	4.87548	5.625821	5.37482	5.475562	4.188925	4.876879
ANKRD16	2.271776	2.95706	2.954991	3.420499	3.617665	3.101544	3.343113	2.729148
ANKRD17	13.68613	12.56188	12.12386	12.18037	13.14273	11.42636	12.39891	13.2418
ANKRD2	0.232188	0.21385	0.270984	0.324462	0.189996	0.170017	0.192821	0.214085
ANKRD22	15.17071	17.41822	20.81514	23.41453	17.023	15.93961	19.42221	18.36215
ANKRD23	0.392416	0.613113	0.424878	0.557176	0.628892	0.442698	0.374308	0.733666
ANKRD24	0.276404	0.546728	0.350948	0.330459	0.289603	0.417438	0.313949	0.120145
ANKRD27	14.21643	15.5114	12.05254	12.22041	14.38847	12.74695	13.96699	12.72973
ANKRD28	4.498705	5.252811	4.266215	4.811536	4.43496	4.284798	4.968765	4.588116
ANKRD29	4.246632	5.123725	3.621847	5.143063	4.903563	5.610162	5.185459	4.946622
ANKRD31	0.21831	0.207352	0.157201	0.680474	0.218338	0.174009	0.115409	0.184515
ANKRD33	0.153291	0.228387	0.151381	0.351138	0.270549	0.226968	0.183048	0.393256
ANKRD33E	1.890206	2.340154	2.229716	2.340416	2.161576	2.220929	3.558193	2.399218
ANKRD34/	0.009079	0	0.017931	0.018607	0.027239	0.054843	0.018429	0.073662
ANKRD34E	0	0.013373	0	0	0	0	0	0.006694
ANKRD34(	0	0	0	0	0	0	0	0
ANKRD35	13.55766	13.76627	15.85372	15.13033	13.32145	15.78014	13.88397	14.09289
ANKRD37	1.628375	1.578017	1.584779	2.104026	1.085723	0.997956	0.814423	1.819105
ANKRD39	4.008261	3.9913	4.534689	4.670466	4.738429	4.735616	3.589239	4.300361
ANKRD40	14.74365	16.0213	13.21354	15.03575	14.90412	13.70312	14.28898	13.84408
ANKRD40(	0.065548	0	0	0	0	0	0	0
ANKRD42	1.599003	1.860979	1.19736	1.709708	1.63886	1.523443	1.291013	1.477684
ANKRD44	3.006913	2.950938	2.51175	2.92694	3.278638	2.50391	3.151456	4.249733
ANKRD45	1.643252	2.156076	2.500673	3.257489	2.50561	2.712259	1.722586	1.693967
ANKRD46	9.681366	12.18274	11.51904	11.95331	11.86622	12.47681	12.25274	10.74016
ANKRD49	5.561109	6.245338	6.761151	5.743662	6.165233	5.748761	5.387149	5.480644
ANKRD50	5.799338	6.219206	5.050086	6.63486	7.164407	6.103888	7.481787	7.058275
ANKRD52	7.439209	5.872534	5.09198	6.343299	6.971646	5.128564	5.652003	6.423797
ANKRD53	0.273697	0.160536	0.085354	0.132858	0.244919	0.130532	0.146212	0.02922
ANKRD54	12.65061	11.29263	11.56757	11.64988	10.88882	10.50247	11.08827	10.20453
ANKRD55	0.331934	0.252218	0.234142	0.425195	0.699523	0.262587	0.22862	0.517013
ANKRD6	2.414107	1.766033	1.961787	1.570626	2.40784	1.62923	1.922865	2.475159
ANKRD60	0.062968	0.159485	0.015546	0.080659	0	0.095097	0.07989	0.063864
ANKRD63	0.115293	0.185516	0.093764	0.145949	0.196702	0.157051	0.068837	0.254505
ANKRD65	0.142948	0.144824	0.056467	0.307626	0.057186	0.143924	0.130583	0.101488

ANKRD66	0.139713	0.21232	0.14947	0.286347	0.442482	0.386835	0.04727	0.247978
ANKRD7	0.136279	0.170941	0.160216	0.27266	0.142787	0.254819	0.072455	0.250111
ANKRD9	1.731805	1.972582	1.472969	2.159392	2.212591	1.375759	1.981315	1.599087
ANKS1A	9.377444	8.779312	7.4344	8.546054	8.905348	8.024079	8.303114	10.71695
ANKS1B	0.009887	0.120197	0.068344	0.010132	0.019776	0.069679	0.010035	0.090247
ANKS3	2.605177	1.870944	1.807414	3.252651	2.424117	2.324155	2.167285	2.709156
ANKS4B	0.74411	0.93696	0.745336	0.762542	0.83989	1.573306	0.712113	1.315339
ANKS6	0.504141	0.501299	0.654593	0.65057	0.597578	0.554584	0.625415	0.473442
ANKUB1	0	0	0	0	0	0	0.012728	0
ANKZF1	7.476272	6.572208	5.408801	7.469392	8.018766	7.03461	6.468121	7.867845
ANLN	12.45491	11.1645	9.955509	11.13353	9.06581	11.17944	12.13801	13.15375
ANO1	2.352281	2.25042	2.170067	2.532601	1.870156	2.044576	2.151831	2.470326
ANO10	27.80097	26.39686	24.81132	26.50744	24.47931	27.054	29.7886	25.10029
ANO2	0.46472	0.586458	0.201285	0.300777	0.46478	0.344764	0.248258	0.471335
ANO3	2.078105	2.374907	1.809317	2.175876	2.148657	2.648227	2.282502	2.296057
ANO4	0.210287	0.014203	0.034611	0.014366	0.028042	0	0.028459	0.014219
ANO5	0	0	0	0	0	0	0.009551	0
ANO6	15.09144	14.35371	12.79402	14.02572	15.20887	15.27204	13.38417	16.79934
ANO8	3.639127	3.205693	2.913342	3.934927	3.726573	3.381106	3.17089	3.358477
ANO9	6.744997	6.499667	4.759277	6.795993	6.611982	5.816479	5.706886	6.496355
ANOS1	2.609159	1.926617	1.671995	1.429094	2.04506	1.79936	0.84264	1.924584
ANP32A	73.84322	84.57378	82.07878	80.49346	78.00588	87.28211	79.24214	76.02688
ANP32B	102.9846	120.979	126.1448	99.29858	99.14958	107.6181	109.9558	99.00952
ANP32E	32.47224	38.71298	37.51252	36.14407	34.66561	39.57824	35.70021	33.66689
ANPEP	31.83677	36.10141	30.52587	35.72338	33.18628	31.13676	36.99777	38.13622
ANTXR1	25.74624	24.35586	21.97819	23.64847	25.25985	21.22275	23.92801	22.62838
ANTXR2	5.455586	7.176016	7.783937	6.199528	7.870616	5.880389	6.475894	8.108927
ANXA1	339.7256	366.5366	298.0687	390.7803	432.6563	364.1702	453.8193	432.2412
ANXA10	0	0	0	0	0	0	0	0
ANXA11	21.68469	25.39749	24.71925	25.84254	25.10161	24.08907	24.93654	26.97088
ANXA13	0.944162	1.487972	1.388237	1.37607	1.321998	1.056236	1.235174	1.106563
ANXA2	685.3932	590.4322	719.6015	660.1093	722.4204	627.7932	700.2579	774.845
ANXA3	22.79963	24.60656	22.02566	24.1269	20.16931	23.53167	29.04806	26.68474
ANXA4	60.70818	63.97735	61.36793	58.9892	61.65961	66.37903	61.33791	59.46725
ANXA5	71.339	75.88519	92.6072	82.46048	95.31146	74.04479	78.59441	91.34348
ANXA6	18.4628	25.74442	22.88264	24.09238	27.11869	21.56976	24.11771	27.61199
ANXA7	25.01722	25.34039	25.97696	26.13233	28.02841	23.53317	25.46996	27.3192
ANXA9	22.45783	20.39463	23.87941	22.90571	23.01998	22.76341	19.0825	18.10247
AOC1	0.96005	0.851069	2.844264	0.633814	0.775526	0.250947	3.523016	3.47355
AOC2	0.137123	0.114407	0.095586	0.082658	0.161342	0.07309	0.106431	0.147256
AOPEP	76.57788	73.50744	76.7951	69.84509	69.33722	70.64751	67.20498	66.43702
AOX1	34.98646	33.33445	31.95853	34.37695	29.03482	27.29185	35.51703	44.12899
AP1AR	5.278959	5.077737	4.602969	5.284641	5.272012	4.861863	4.18121	4.131639
AP1B1	16.23154	15.55881	14.72528	15.92921	17.22865	16.02675	15.17758	16.1018
AP1G1	25.21462	24.68126	21.94785	23.26652	25.02287	22.5852	23.5972	22.91197
AP1G2	6.546584	6.244755	6.256143	8.483982	6.617941	6.692712	6.010973	6.272037
AP1M1	22.06248	20.62403	17.18286	20.23239	21.48854	18.45747	21.75823	21.44316
AP1M2	35.95146	35.68697	37.62478	37.68608	36.08533	36.48976	38.68739	32.26981
AP1S1	35.83978	39.20631	39.52242	42.03993	42.24521	39.24203	37.04863	36.47981
AP1S2	13.56375	15.66079	16.73243	16.48785	18.03038	15.11479	17.08461	16.09234
AP1S3	5.65491	7.4318	6.409069	6.390731	6.089064	7.438852	6.340529	6.721688
AP2A1	23.18634	21.05771	19.71656	20.70345	22.94343	19.03722	21.58751	20.88131
AP2A2	23.1278	23.50057	21.94527	24.91767	26.16419	21.84925	22.66823	24.40156
AP2B1	32.40305	30.3423	30.16116	30.34199	30.8765	30.3676	30.4937	30.90639
AP2M1	83.93911	89.51277	84.32289	87.16735	83.21181	80.89711	86.73966	87.7252
AP2S1	64.96776	60.24678	64.94237	58.09144	55.90048	61.07175	63.61084	63.24656
AP3B1	19.79807	21.47787	20.3582	19.26359	17.99737	19.88369	20.62405	20.5228
AP3B2	0.065518	0.086291	0.01941	0.21485	0.170368	0.006597	0.073151	0.106321

AP3D1	36.79267	35.9155	31.87353	34.30324	35.35112	33.3257	34.54928	37.27554
AP3M1	32.11377	29.89096	31.26656	27.98373	31.70429	28.76559	30.55896	31.51093
AP3M2	0.286895	0.196623	0.174992	0.12106	0.185662	0.127436	0.402539	0.273863
AP3S1	28.1506	32.24094	34.07722	26.80063	31.70632	31.55308	32.21872	32.13305
AP4B1	6.936364	5.469973	5.470692	5.552065	4.799834	4.6055	5.394476	5.590058
AP4E1	13.2098	11.4105	12.01466	11.13739	10.46653	11.44641	11.22926	11.70268
AP4M1	12.96592	13.91165	11.6704	12.37187	12.07438	13.56833	12.22154	11.905
AP4S1	13.52112	14.41251	13.85283	15.66139	17.09079	18.77953	15.46737	14.96436
AP5B1	4.840101	4.639513	5.749481	5.797046	6.196477	5.888045	5.071461	5.852025
AP5M1	9.423111	8.159538	8.237958	7.86109	9.325665	7.218911	7.625933	8.608733
AP5S1	6.557375	5.790149	5.39144	6.257432	6.678557	6.026754	5.571889	5.32363
AP5Z1	0.13856	0.640478	0.590095	0.319483	0.441718	0.444678	0.263697	0.175666
APAF1	2.533165	3.034339	2.452482	2.308287	3.077145	2.414541	3.076019	3.333148
APBA1	1.616639	1.678352	1.793864	2.525987	2.385294	1.802077	1.09092	1.770284
APBA2	0.423366	1.056418	0.634878	0.482058	0.329327	0.307853	0.326266	0.453248
APBA3	9.464262	7.698978	7.037241	8.7003	9.270608	8.029515	7.896883	8.752022
APBB1	6.279851	6.191958	6.05931	7.955047	7.121287	6.75797	7.056548	5.827315
APBB1IP	3.150579	4.887224	4.776718	5.264927	5.445583	3.093128	4.896274	6.927849
APBB2	15.60227	19.65808	14.44936	15.16937	15.19804	17.03049	18.19721	16.48355
APBB3	5.269292	5.389654	3.494048	5.192627	5.105681	4.936336	4.617264	4.434362
APC	7.897748	7.508936	6.108439	6.744856	7.60533	6.159428	6.620544	7.490527
APC2	0.820868	0.499606	0.381122	0.533598	0.768898	0.684618	0.606233	0.739358
APCDD1	47.02718	47.20531	54.97831	56.97443	49.37221	57.95558	55.94586	50.8264
APCDD1L	6.29371	9.934222	10.83671	8.585624	5.742941	7.341106	7.03022	4.746349
APEH	15.69924	14.77766	13.27064	15.98006	14.13114	14.70947	14.16288	13.2016
APELA	0.031413	0.190953	0	0.160957	0.094252	0.094883	0.095653	0.063721
APEX1	86.30866	80.18409	75.64019	76.03897	75.90552	77.22337	81.34338	81.37072
APEX2	13.39849	13.20375	10.59594	11.28695	10.81231	11.34853	11.42685	11.17092
APH1A	32.28518	35.42913	34.90406	36.7369	31.05246	34.61926	36.08934	32.89332
APH1B	2.756215	3.932939	3.354419	3.69968	3.688369	2.990012	2.12772	4.567216
API5	32.25517	35.81833	33.3652	32.40677	33.96894	33.36598	34.54191	34.47067
APIP	10.69	17.45879	14.89341	14.93216	13.88548	12.63874	16.0279	15.00359
APLF	3.416418	4.708167	3.164293	4.729671	4.265769	2.969729	4.436887	4.594962
APLNR	2.429691	2.595441	2.341425	3.520429	4.669134	4.234816	2.548093	1.392206
APLP1	3.880028	4.514176	3.763462	4.507076	4.387186	3.431259	4.031718	3.911908
APLP2	104.7666	103.0546	98.65284	110.0502	93.41514	102.3232	110.7353	110.2608
APMAP	27.22549	31.39536	22.86104	29.26705	28.37927	27.65234	27.49379	28.37828
APOA5	0	0	0	0	0	0	0	0
APOB	0.005994	0.002024	0	0.002047	0.001998	0.002012	0.004056	0.002026
APOBEC2	0	0	0	0	0.090299	0.022726	0.045821	0
APOBEC3F	4.706437	5.020936	5.091223	6.101202	5.405618	6.329282	5.570393	6.224021
APOBEC3Z	0.083054	0.016829	0.032808	0.017022	0	0.016724	0	0.016847
APOBR	1.877151	2.221117	1.846845	2.167694	2.27669	1.459794	2.11148	3.829059
APOD	559.8642	428.1612	407.4698	373.3971	430.0587	452.8678	478.3277	518.3884
APOE	42.0678	42.52055	37.67009	44.73201	43.40422	46.55826	52.59504	58.12498
APOF	0	0.033069	0.145052	0.250867	0.081612	0.082159	0.13252	0.049658
APOH	0.046688	0	0	0.023922	0	0	0	0
APOLD1	0.879422	1.059335	0.786405	1.518567	1.572084	1.338603	2.164763	1.685558
APOM	2.245381	2.215506	2.120995	2.120922	2.118742	1.995332	2.031338	2.099119
APOO	16.53574	17.61502	19.40352	15.20096	16.87836	17.50562	15.62372	16.59849
APOOL	10.68677	11.76851	15.01104	15.57696	15.40156	18.24484	16.64116	16.98768
APOPT1	25.28816	22.19947	30.60784	28.99386	18.77441	32.29355	26.76409	18.83376
APP	269.4805	250.8629	242.2483	254.3781	240.298	245.8579	270.7919	280.2535
APPBP2	15.73385	16.69701	15.99827	15.92061	17.53934	15.5796	16.02011	18.3559
APPL1	14.16313	13.84874	13.45214	13.43867	14.48312	13.41854	15.23821	18.14977
APPL2	19.28261	18.16475	21.09076	20.71816	18.75979	18.56284	21.04471	18.25692
APRT	7.021786	6.683794	7.901869	10.30828	10.09308	8.582354	9.149224	9.440453
APTX	5.068904	5.805266	5.829671	6.21077	4.077689	5.689963	5.28874	5.236859

AQP1	99.27781	107.9901	116.5352	135.2173	185.6274	140.7986	107.7391	127.5189
AQP10	0.04601	0.326294	0.227181	0.204313	0.10737	0.138972	0.171232	0.326652
AQP11	0.102471	0.133477	0.216844	0.135012	0.161046	0.073693	0.074291	0.178165
AQP3	63.67612	59.47377	49.92911	64.55921	78.35823	59.22839	62.92797	58.83636
AQP4	0.28575	0.14475	0.042328	0.087848	0.342944	0.02877	0.449555	0.1594
AQP5	0.103205	0.230031	0.020384	0.084609	0.206436	0.20782	0.335209	0.251218
AQP6	0	0	0	0	0	0	0	0
AQP7	0.170468	1.381639	0	0.723717	0.535825	0.057211	0.205982	0.156428
AQP8	1.149583	3.882235	4.77755	4.254102	2.331401	3.970652	2.43089	0.777299
AQP9	22.03429	14.53351	15.53908	18.73834	11.19711	15.88115	18.76677	8.859003
AQR	7.873222	8.019865	6.554914	6.826133	6.557234	6.887436	7.043124	7.365211
AR	2.970449	3.772389	2.148433	2.550982	2.740698	2.200934	3.025622	3.564363
ARAF	16.83769	17.40031	15.0317	17.02835	16.13045	16.09805	15.49689	15.27293
ARAP1	9.012307	7.914814	7.8109	8.483757	9.51395	7.743366	8.348627	9.332777
ARAP2	17.19847	14.9752	14.69939	15.16631	14.56099	14.38276	16.99806	16.04664
ARAP3	3.035418	3.146005	2.395319	3.024731	4.874991	3.346771	2.745447	3.834121
ARC	1.786042	0.619033	0.603401	1.300464	1.480727	1.159394	1.407331	1.453941
ARCN1	62.53387	64.86577	61.65648	65.39978	67.4076	64.83257	65.4362	60.8716
AREG	12.69517	11.74336	11.21969	16.96908	10.9487	14.2407	13.11903	10.26339
AREL1	8.581868	7.078931	6.2643	7.593327	7.445959	7.075482	6.775476	7.459145
ARF1	85.24231	85.50769	83.86545	88.38025	94.25793	87.66973	88.9702	85.80931
ARF3	41.9915	36.93414	38.21421	39.91709	39.12786	40.41269	40.29199	40.60765
ARF4	141.6468	144.3936	153.1167	141.8805	153.5737	145.0018	144.9749	133.1551
ARF5	80.05004	68.51514	80.5288	76.80351	63.76993	85.26769	78.00704	74.44559
ARF6	47.44253	47.18474	44.11151	47.06088	48.57533	47.51387	49.08197	49.92641
ARFGAP1	8.309527	7.755824	7.298485	8.483434	8.349544	8.679931	8.141701	7.701146
ARFGAP2	34.56864	33.4556	32.02266	32.58029	32.69027	33.66365	36.04258	33.20006
ARFGAP3	8.518891	10.67563	9.629168	9.875509	9.60699	10.15077	9.592241	9.301561
ARFGEF1	13.80418	15.43211	13.98943	13.69734	15.9159	14.29339	15.01232	15.54173
ARFGEF2	8.232475	8.245595	7.537734	8.049141	8.795768	7.43343	8.289393	8.197627
ARFGEF3	1.935613	1.84112	2.178906	2.306273	2.146501	1.962973	1.887283	2.449382
ARFIP1	10.76851	12.51378	13.20811	12.41274	12.48771	11.90222	12.66581	11.73981
ARFIP2	14.87952	15.82061	15.65311	17.39888	17.04317	18.89205	17.43966	15.139
ARFRP1	28.83587	24.45586	24.04337	26.97985	27.44954	25.15645	26.91721	25.77893
ARG1	0.014639	0.133477	0	0.93008	0.278171	0.176864	0.312024	0.371177
ARG2	0.839285	0.692104	1.657654	1.40012	1.639745	1.513172	1.426393	1.346135
ARGLU1	61.91304	56.37807	51.90739	60.2848	58.7565	58.40533	53.60641	62.53895
ARHGAP1	21.89829	21.23242	19.73607	21.11652	19.89984	18.17009	18.71862	18.06831
ARHGAP1(	26.76369	26.80681	31.58905	29.6083	27.38444	28.25242	31.74109	32.72423
ARHGAP1:	5.018913	5.420108	3.940141	4.582041	4.231117	3.659631	5.08198	5.670197
ARHGAP1:	11.85483	11.8615	11.82994	13.1623	11.38143	11.62846	11.76297	11.34702
ARHGAP1!	0.34732	1.0435	0.733293	1.202773	1.161874	0.566743	1.069744	1.166115
ARHGAP1;	19.31117	19.09225	16.92437	16.4142	18.98275	16.53572	17.11257	20.11997
ARHGAP1{	9.844189	10.98612	14.58624	14.24289	12.57276	15.95009	10.43828	11.50915
ARHGAP1!	5.640873	5.054221	4.32123	4.691307	4.278487	4.162711	4.92458	5.456612
ARHGAP2(	0.486202	0.989045	0.699422	0.698328	0.70451	0.659122	0.7655	0.706681
ARHGAP2:	15.4722	11.67338	14.14947	12.67391	13.54631	12.18422	13.69964	17.70668
ARHGAP2:	0.537087	0.50903	0.316526	0.426109	0.623795	0.357597	0.615484	0.492017
ARHGAP2:	15.63359	13.31149	13.94471	15.08875	14.45573	14.51634	13.2813	13.60011
ARHGAP2	4.926517	4.361787	4.299197	4.826471	4.233595	4.863199	5.288806	4.06863
ARHGAP2!	3.426667	4.131536	3.30007	4.691736	3.332699	3.412059	3.583463	4.509465
ARHGAP2(	2.096437	1.942444	2.008237	2.315855	2.253883	1.765822	2.07684	2.489694
ARHGAP2;	7.291766	7.456918	7.668661	8.200464	8.065611	6.237206	7.280953	8.400608
ARHGAP2{	3.651133	4.575027	4.501765	4.588142	5.363963	4.520754	4.001329	5.465667
ARHGAP2!	17.65429	18.35724	17.30232	16.4753	17.42664	16.18151	18.06563	19.06064
ARHGAP3(	4.430494	5.874263	4.57186	6.573476	5.670481	3.465171	5.71569	7.359027
ARHGAP3:	7.81864	6.32723	6.301395	7.456329	8.374322	6.919153	7.030356	8.189791
ARHGAP3:	10.04649	8.928367	7.703311	8.329165	9.652423	9.44585	8.022297	7.809401

ARHGAP33	0.62643	0.49071	0.573982	0.668419	0.477957	0.578692	0.544058	0.445399
ARHGAP35	14.90196	12.86086	10.92761	12.84886	12.4979	12.16489	13.88634	14.02496
ARHGAP36	0	0.018534	0.009033	0	0	0.009209	0	0.009277
ARHGAP39	6.869795	7.005543	4.433922	6.486603	5.410538	6.378087	5.785327	5.851965
ARHGAP4	1.615366	1.860097	1.058304	1.962229	1.647098	0.848902	1.679586	1.718281
ARHGAP42	7.013598	6.336996	5.70234	5.414242	5.881471	7.040773	5.571691	6.392696
ARHGAP44	0.868202	1.221661	1.122764	0.748484	1.709062	1.505451	1.475701	0.642949
ARHGAP45	1.98687	1.842696	1.322718	2.096864	2.076103	1.433153	1.896275	2.436231
ARHGAP5	19.79972	19.69913	19.21519	18.43572	20.45574	19.85576	20.75295	20.06076
ARHGAP6	1.605294	1.722697	1.651576	1.851406	1.980305	2.004839	1.731564	2.116023
ARHGAP8	2.580684	1.807376	2.001193	1.561916	2.771563	1.848465	2.549075	1.703959
ARHGAP9	0.375031	0.929885	0.389851	0.7383	0.848864	0.457086	0.691192	1.101071
ARHGDIA	104.0078	98.67093	105.4427	95.46005	101.8955	94.859	104.0379	96.41448
ARHGDIB	25.15434	32.09013	26.29665	34.99609	37.1032	25.63063	32.09201	41.23008
ARHGDIG	0.518318	0.068494	0.178038	0.323313	0.247923	0.181516	0.160115	0.297133
ARHGEF1	18.18488	15.99097	14.55359	19.35298	18.33346	16.41079	17.49688	17.19491
ARHGEF10	11.39377	10.57655	9.925352	11.55377	11.57159	10.84503	9.269641	10.909
ARHGEF10	10.99151	9.565167	9.26506	10.36093	10.40833	10.40132	9.89242	10.2802
ARHGEF11	6.816982	5.810396	5.084429	6.273455	5.86623	5.446222	5.596247	6.204556
ARHGEF12	27.59569	25.56912	22.80448	25.28656	27.63307	25.40102	27.74753	28.7368
ARHGEF15	2.616897	2.120993	2.802519	3.104834	3.448934	2.898252	2.86274	2.164607
ARHGEF16	3.04582	2.983275	2.218428	2.99683	3.764756	2.771172	3.019612	3.232862
ARHGEF17	5.580152	5.56382	4.103283	6.059012	6.164425	5.098458	5.042912	4.942277
ARHGEF18	6.165628	5.509841	5.297603	5.631187	6.101103	5.677284	6.059232	5.997258
ARHGEF19	7.905116	6.904444	4.904979	8.034346	6.801445	6.788875	6.880591	7.959074
ARHGEF2	6.936688	6.026463	4.94618	6.466453	7.534367	6.030337	6.295026	6.256243
ARHGEF25	6.084214	6.516037	6.034795	7.248211	6.984832	7.462149	6.690853	6.794233
ARHGEF26	6.130491	8.9923	7.56628	9.258193	6.043772	7.692064	8.609293	10.07266
ARHGEF28	5.780883	5.280621	5.130656	5.199947	6.14172	6.186266	4.381913	4.897357
ARHGEF3	5.68003	5.968102	5.447951	6.204095	6.318401	5.692308	6.257254	5.616599
ARHGEF33	0	0	0	0	0	0	0	0
ARHGEF37	24.80635	21.34755	24.88376	25.91036	22.12138	24.50064	23.68877	21.23671
ARHGEF38	0.068819	0.082026	0.047973	0.103712	0.056682	0.028531	0.032871	0.069799
ARHGEF39	4.914881	4.859399	3.645104	5.400718	4.856294	4.133651	3.886704	5.645491
ARHGEF40	8.6396	6.695214	6.274205	7.742506	7.330625	6.228826	6.832104	7.389237
ARHGEF5	16.75592	13.28979	12.46448	15.42712	14.04338	13.78011	12.68391	14.31028
ARHGEF6	8.260859	9.593648	7.01221	8.641618	8.740022	7.230313	7.507042	10.43221
ARHGEF9	1.247985	1.267581	0.912566	1.314694	1.270378	1.045494	1.437533	1.00165
ARID1A	22.13707	19.29222	16.06187	19.61529	19.10272	17.12716	16.90979	19.42401
ARID1B	11.48857	12.37695	9.799203	10.64394	11.52379	10.96422	11.34991	11.56659
ARID2	9.680819	8.980555	8.087597	8.016537	8.711496	8.106574	8.344519	8.698331
ARID3A	6.850024	6.542057	7.202353	7.468135	7.698155	7.207488	6.18404	7.19221
ARID3B	1.00971	1.432146	0.624867	1.255461	0.989644	0.772622	0.840382	1.174283
ARID3C	0.01428	0.05787	0	0.029268	0.085692	0.014378	0.014494	0.014483
ARID4A	7.864446	8.021198	6.931453	7.443853	8.701498	7.299036	7.387511	7.323477
ARID4B	8.800593	8.130212	6.969333	7.883581	8.513805	7.403387	7.8003	8.901543
ARID5A	1.342978	0.762955	0.694109	1.337658	2.171637	1.276331	1.146552	1.285717
ARID5B	14.9431	15.58659	13.85684	13.66245	12.39413	13.95681	14.22422	15.76492
ARIH1	20.99462	19.64168	20.15876	17.66934	21.42529	18.42053	20.65806	20.41437
ARIH2	18.08541	17.35197	17.3373	18.62953	16.35872	18.10776	18.88719	17.32344
ARIH2OS	2.778405	3.306054	3.075249	3.458712	2.57362	2.290477	2.611887	2.53427
ARL1	44.38778	52.3419	46.97607	48.01643	48.72682	48.34471	50.08223	45.56665
ARL10	2.845887	2.862242	2.660356	2.704023	2.687362	2.427184	2.657209	2.220847
ARL13A	0.017884	0.036238	0.052985	0	0	0	0.018153	0
ARL13B	2.771425	3.297092	2.883643	3.23601	3.52232	3.037226	3.740607	3.300709
ARL14	0	0	0	0	0.027417	0	0	0
ARL14EP	13.11917	14.32404	13.18071	15.07857	14.85992	14.42825	13.76632	13.35458
ARL14EPL	0.162444	0.674763	0.385009	0.432817	0.454903	0.310753	0.34625	0.576649

ARL15	31.10286	29.71339	29.33372	26.99526	26.20111	29.40001	32.39198	29.03652
ARL16	3.179257	4.050928	2.658103	2.51846	2.848046	2.454736	2.732016	3.60038
ARL2	57.13824	58.39905	56.82471	57.62333	57.49869	52.60336	62.9093	52.42756
ARL2BP	63.87711	64.77342	68.05534	67.10172	57.98914	69.32718	60.07139	54.04676
ARL3	57.59174	67.51546	68.75467	63.54712	63.56256	79.17343	66.35887	59.40765
ARL4A	17.29595	16.85944	18.02405	18.06544	20.1329	19.92191	18.42717	16.59483
ARL4C	17.64429	17.53303	15.03753	17.53643	17.35649	16.69407	21.44168	17.30713
ARL4D	8.815613	7.2865	5.611453	7.536634	8.199742	8.892183	8.865454	8.167201
ARL5A	43.17588	54.34995	45.76024	38.56198	66.24959	52.81211	51.76751	50.25658
ARL5B	10.03642	9.385971	8.469494	7.751685	9.157565	9.243098	9.058237	9.027073
ARL5C	0.076242	0.085825	0.033463	0.034725	0.008472	0.034117	0.060189	0.060143
ARL6	2.878885	2.997686	2.400767	2.319182	2.823272	2.98712	3.011354	2.449445
ARL6IP1	105.874	117.318	108.7099	109.3402	106.8025	103.6504	113.9145	118.8847
ARL6IP4	13.17349	13.69375	12.40344	14.12856	13.03081	12.91828	11.75924	12.62892
ARL6IP5	31.45293	31.44993	33.54136	32.14791	28.99428	31.78349	32.52456	31.33459
ARL6IP6	7.804383	6.954107	6.932285	7.678529	7.691576	7.688846	8.249736	7.763613
ARL8A	24.7961	22.34148	20.98898	26.94413	24.79931	20.19342	20.81301	22.87201
ARL8B	111.807	113.6282	134.0634	108.1344	114.0668	117.594	113.6866	110.7673
ARL9	0	0	0	0	0	0	0.019352	0.019338
ARMC1	19.50512	22.17481	21.02216	19.70871	20.65354	22.7329	21.4775	20.0318
ARMC10	17.53684	20.73042	17.9941	19.31873	21.14891	18.95353	20.56452	19.82778
ARMC12	0.125448	0.167038	0.198215	0.194669	0.261682	0.147957	0.192813	0.319901
ARMC2	0.778507	0.659952	0.711277	0.824926	0.672674	0.82115	0.811686	0.956101
ARMC3	0	0.008567	0	0	0	0	0	0
ARMC4	0.193517	0.148238	0.069917	0.072553	0.122734	0.04277	0.06707	0.114891
ARMC5	0.897817	0.965209	0.933091	0.968269	1.019488	0.911846	0.779964	0.874811
ARMC6	5.222734	3.800219	4.012941	4.244311	4.650267	3.055387	3.741158	4.028953
ARMC7	4.356428	4.63787	2.920563	4.573225	3.799084	3.886948	4.170129	4.122085
ARMC8	8.425503	8.99229	7.982985	8.784318	8.372333	7.461599	7.869057	8.622482
ARMC9	1.686443	1.774572	1.222268	1.683713	1.672183	1.574078	1.447265	1.431493
ARMCX1	8.892713	9.874024	9.392611	10.16497	9.784486	10.59722	9.641234	8.730789
ARMCX2	5.026092	5.385462	6.005978	5.638843	6.381532	5.069834	5.566116	5.599825
ARMCX3	13.56846	12.94294	12.81609	13.75323	14.3002	13.31283	13.85763	14.12106
ARMCX4	0.070232	0.142307	0.142363	0.219702	0.181147	0.219578	0.165081	0.11622
ARMCX5	3.765067	3.978897	4.337846	5.077916	4.187556	5.195995	4.17295	3.632122
ARMH1	0.269288	0.630902	0.382279	0.896866	0.8753	0.864221	0.495407	0.938857
ARMH2	0	0	0	0	0	0	0.036752	0.036724
ARMH3	7.538629	7.534156	6.904704	7.971917	7.196894	7.99386	7.444505	6.921282
ARMH4	6.174983	7.011367	6.539804	5.303245	5.754706	6.190674	7.727676	6.414123
ARMT1	5.619872	5.941715	6.625961	6.235864	7.249225	6.533507	7.096037	6.059997
ARNT	30.01305	26.22162	22.09117	27.19405	30.27888	22.05525	25.12221	30.09014
ARNT2	0.695407	0.792103	0.40739	0.833418	0.730862	0.205696	1.335909	0.673429
ARNTL	4.86328	4.726896	5.231069	4.865202	5.360423	4.687832	4.765002	5.025394
ARNTL2	0.700102	0.641379	0.522211	0.45031	0.58101	0.592403	0.476255	0.38525
ARPC1A	139.384	139.8506	118.0266	133.2583	128.0789	132.9483	149.4929	131.8021
ARPC1B	79.31499	84.73146	84.69682	94.55716	95.8243	84.44781	93.37343	98.98063
ARPC2	196.0542	213.0503	200.8977	206.11	202.7897	208.6957	202.9376	202.8225
ARPC3	156.6202	174.8703	180.5139	167.566	164.916	184.4189	170.8676	155.4166
ARPC4	43.47283	45.46817	48.70432	50.32854	47.72563	48.80896	48.40726	42.81914
ARPC5	63.98329	66.07948	76.4913	65.92923	64.76679	73.0618	66.00158	69.09652
ARPC5L	48.20072	46.65602	47.32221	47.87402	47.84872	46.26321	45.62579	44.57944
ARPIN	20.77473	22.50202	18.46779	21.57997	21.0788	18.36454	21.89598	22.34693
ARPP19	26.68573	28.06434	28.53461	27.68363	28.57536	26.3222	27.75922	24.52109
ARPP21	0.011782	0.014921	0.029089	0.006037	0	0.005931	0.008969	0.029875
ARRB1	5.442534	7.216066	6.354158	7.628161	9.135602	6.627541	8.053397	9.242977
ARRB2	6.450922	7.161656	5.075596	7.861807	6.331162	5.220274	5.798068	8.117297
ARRDC1	12.45544	13.03822	11.76378	12.33982	12.4053	11.14713	12.66852	13.31489
ARRDC2	13.23786	14.46938	13.76345	13.64429	15.74953	14.35058	13.31976	14.97101

ARRDC3	56.68952	62.47048	51.51317	41.97816	45.86136	47.70119	60.87932	61.99972
ARRDC4	34.88186	37.07676	30.83725	35.88647	25.37939	33.24884	43.59156	38.72429
ARRDC5	1.134696	2.001134	1.36957	1.421203	1.450077	1.269386	1.364997	1.172161
ARSA	6.411273	5.551771	5.503555	6.395092	6.474204	6.033069	7.563129	5.982968
ARSB	14.8836	9.769261	10.08155	14.82234	9.517413	7.516107	8.949207	11.06326
ARSE	0.547254	0.663864	0.156442	0.47964	0.288066	0.420494	0.40198	0.525832
ARSG	2.044152	2.973715	1.91516	2.880332	1.906812	2.453909	2.527018	1.860611
ARSH	13.93736	11.06689	12.20649	8.639925	9.424194	12.02243	9.732116	11.90448
ARSI	0.553181	0.442929	0.466988	0.182866	1.151122	0.512044	0.253571	0.190035
ARSJ	7.172503	7.432816	4.903707	4.553743	5.861035	5.434894	6.76549	6.881386
ARSK	2.057651	2.628479	2.069876	2.357463	2.390251	2.238989	2.607402	2.009169
ART3	0.315828	0.484283	0.092725	0.306156	0.247573	0.146101	0.372549	0.43287
ART4	0.514743	0.554092	0.254165	0.461557	1.3192	0.340108	0.636753	0.603645
ARTN	0.01666	0.008439	0	0.025609	0.008331	0.025161	0.008455	0.050692
ARV1	15.6534	18.51436	18.02872	15.75939	19.59678	21.73967	19.06957	21.41619
ARVCF	0.587741	0.627641	0.352956	0.765083	0.627534	0.471805	0.540126	0.62833
ARX	0.009855	0.009985	0	0	0	0.009923	0.010003	0.019991
AS3MT	1.698595	1.958251	2.173222	2.186556	2.083768	1.844993	2.301596	1.892507
ASAH1	39.04901	41.52727	45.85978	52.60938	53.10705	60.10511	41.94882	48.23105
ASAH2	5.317941	6.204401	4.577253	4.898965	10.25656	3.764898	4.82953	6.392889
ASAP1	3.495365	3.824891	3.6635	3.738852	3.775832	3.193306	4.213839	4.401455
ASAP2	6.379772	6.151327	5.586945	6.449802	7.118179	6.158894	5.491074	6.055613
ASAP3	13.84647	11.21558	11.59904	10.42678	13.64157	14.28786	11.78872	14.14839
ASB1	11.58129	10.21396	9.495559	10.78767	10.06194	10.3789	10.97475	11.73763
ASB10	0.039491	0.040009	0.019499	0.010117	0	0.00994	0	0
ASB11	1.560184	1.635165	1.102429	1.833142	1.587289	1.70626	1.242297	2.032558
ASB12	0.42139	0.831371	0.372335	0.886384	0.465807	0.401939	0.697844	0.787295
ASB13	10.77307	11.55772	11.71814	12.05325	10.12904	14.27358	8.768863	8.688361
ASB14	0	0	0	0.024348	0.007921	0	0	0
ASB15	0	0.005579	0	0	0	0	0	0
ASB16	0.09807	0.079486	0.096848	0.070349	0.039233	0.039496	0.059725	0.05968
ASB18	0	0	0.009152	0	0	0	0	0
ASB2	0.858503	1.907565	1.416222	1.609577	1.297679	1.109928	1.217953	1.602925
ASB3	8.919624	8.864479	8.576682	8.692662	8.232695	8.410106	8.412614	8.307767
ASB4	0.297109	0.401344	0.225697	0.320082	0.342862	0.283798	0.502609	0.193166
ASB5	0	0.101833	0.015271	0.04754	0.023198	0.007785	0.031391	0.031368
ASB6	10.0761	12.01806	8.907046	11.21517	10.75678	10.24626	10.34215	9.760234
ASB7	6.461328	5.978997	5.06937	6.035523	5.818925	5.6001	5.718067	6.311713
ASB8	18.47334	17.49656	16.82194	16.38816	17.61957	17.5128	17.12599	15.6157
ASB9	1.781893	2.554639	2.871947	2.101658	2.118373	2.149494	1.569747	2.386946
ASCC1	14.15997	18.9344	18.56933	19.67356	16.27399	20.28987	15.79282	17.03256
ASCC2	18.11424	19.04587	17.47464	22.28181	18.22905	21.51095	20.68939	18.7661
ASCC3	13.27834	13.35861	12.99561	11.9187	11.6284	13.49983	14.35517	13.47489
ASCL1	0	0.056713	0.033168	0.034419	0.055986	0.045089	0.022727	0
ASCL2	2.115569	1.839853	2.07072	2.033673	2.321809	1.809576	1.767249	2.297592
ASCL3	0	0	0	0.052532	0.563957	0	0	0
ASCL4	0.159029	0.214822	0	0.434583	0.371116	0.053372	0.10761	0.161293
ASDURF	1.84262	1.547792	1.842694	1.876311	1.901176	2.442299	1.751888	2.164555
ASF1A	12.31246	15.07427	13.18657	12.11985	12.08858	13.33941	13.95808	12.52291
ASF1B	7.149406	6.580556	5.693325	7.279735	6.237522	6.600946	7.642639	6.263787
ASGR1	4.146543	14.29077	0.51186	2.466085	7.072251	6.635061	4.114796	4.449645
ASH1L	16.43889	15.34597	13.61742	14.36386	15.62614	13.8123	14.84996	16.24069
ASH2L	19.59135	16.78989	16.31341	18.64959	17.86095	16.12906	18.56889	18.64116
ASIC1	0.013027	0.013198	0.038595	0.020025	0	0.006558	0.019834	0.013213
ASIC2	0.008846	0.017924	0	0.018131	0.09732	0	0.008979	0
ASIC3	0	0.030295	0.014765	0.015322	0.014953	0	0	0.015164
ASIC4	1.134884	0.592524	0.680697	1.098781	0.800789	0.532763	0.75616	0.699098
ASIP	110.4921	153.6197	165.3925	122.5008	177.5914	214.7279	112.5459	110.8437

ASL	36.33653	27.98529	34.405	28.12743	30.71718	41.12378	29.86422	24.52612
ASMT	0.029744	0.331479	1.174938	0.152404	0.267731	0.299473	0.513234	0.784355
ASMTL	16.01854	15.23049	14.0569	15.84222	16.38017	15.9939	16.90755	14.67999
ASNA1	41.86158	43.97869	46.83589	41.25713	44.02551	45.08586	47.57337	43.06326
ASNS	18.07276	19.96262	29.37854	28.46251	28.57239	27.8238	24.43305	14.84689
ASPA	3.922472	4.613824	5.252335	3.389436	4.554645	3.765192	3.896959	7.096902
ASPG	0.866114	0.819752	0.934102	1.19121	1.230953	1.686692	1.017912	0.439222
ASPH	10.8366	13.01171	11.99364	14.74557	13.72207	13.12288	12.02641	13.47526
ASPHD1	0.007478	0	0	0.007663	0	0	0	0
ASPHD2	1.133831	0.416871	0.64112	1.068213	1.152267	0.929833	0.733195	0.49152
ASPM	3.792788	4.116504	3.096015	3.502306	3.237807	2.948025	3.941162	4.23467
ASPN	12.09587	11.17487	18.42354	12.54394	14.73631	13.42865	12.31932	17.56778
ASPRV1	270.9706	208.9718	305.7173	227.0296	256.9205	262.7028	274.6258	202.1001
ASPSCR1	17.3663	16.57866	17.39418	18.27673	17.55082	19.51655	17.05861	15.23688
ASRGL1	9.702396	11.47019	7.931708	13.2877	9.793618	11.19193	11.86969	14.79336
ASS1	9.07073	8.109701	9.36812	11.12857	14.92707	12.20725	11.97616	9.163205
ASTE1	2.300822	2.10988	1.629025	1.74911	1.825149	1.923845	2.110155	2.377126
ASTN1	0.016487	0.013363	0.006513	0.027033	0.046171	0.01992	0.023429	0.070233
ASTN2	0.959035	0.909735	0.633401	1.201884	0.788099	0.879484	1.035422	0.749651
ASXL1	7.850091	7.899802	5.795867	6.88671	6.928539	6.376581	7.141524	8.005795
ASXL2	7.646355	6.65018	6.125246	6.136139	6.518867	6.205197	6.484591	7.410777
ASXL3	0.00244	0.034602	0.019273	0.0175	0.00488	0.017194	0.022285	0.064332
ASZ1	0	0	0.019356	0	0.039206	0	0	0
ATAD1	25.74548	30.71342	27.33409	26.92359	29.20267	31.95927	28.87077	27.49624
ATAD2	6.201651	5.936284	5.524047	5.507201	5.037577	5.313073	5.957646	6.22258
ATAD2B	9.238793	8.389129	9.045696	9.320514	9.178962	9.818681	8.885555	9.352112
ATAD5	2.348615	2.946842	1.712751	1.893033	2.028201	2.04634	2.406766	2.65232
ATAT1	4.526721	3.447672	3.195333	3.952823	4.43963	3.771291	2.734124	3.815191
ATCAY	0.00623	0.012623	0.030759	0.025535	0.012461	0.006272	0.012646	0.044227
ATE1	7.463134	7.443702	6.945858	6.39147	7.246838	6.45456	7.183097	8.122636
ATF1	10.07171	11.00591	10.96019	10.61838	10.73932	11.46628	10.06368	10.89079
ATF2	13.1847	13.52657	10.20964	12.90335	13.47307	12.06676	12.42852	14.67718
ATF3	3.254148	3.762779	3.213601	3.638998	3.865892	2.959877	4.325164	2.69824
ATF4	430.1702	413.4795	497.6583	505.4048	468.3444	532.4357	422.447	381.7452
ATF5	17.16305	13.84373	15.95845	17.29537	15.80627	11.89739	11.51054	11.45036
ATF6	15.65819	13.96115	16.22489	14.04179	14.86142	15.34544	12.87184	15.27652
ATF6B	24.61813	25.35062	24.74195	25.41319	24.02572	26.47799	24.56645	24.41852
ATF7	11.15805	8.922928	8.285934	9.186098	10.54196	9.006359	9.407102	10.49038
ATF7IP	10.64645	10.79401	9.627675	10.01178	9.946399	9.478263	9.570863	10.62803
ATF7IP2	0	0.012121	0.005907	0	0.005983	0	0	0.006067
ATG10	3.591337	5.406147	3.776327	5.661993	3.373676	4.596695	2.671184	4.910688
ATG101	12.52507	11.51225	11.05279	11.94227	13.44952	11.14828	12.71294	10.86632
ATG12	14.78385	15.94846	16.98633	13.76669	17.12666	16.01382	15.3592	13.94537
ATG13	21.44447	21.69951	19.92965	21.06807	20.04695	21.23035	20.17317	18.03122
ATG14	6.458529	5.860163	5.437994	6.130759	5.434593	6.043406	5.49527	5.960459
ATG16L2	1.514592	1.509519	1.349796	1.829722	1.61331	1.078615	1.281092	1.723489
ATG2A	10.85901	9.849629	8.587739	9.339324	10.84709	9.569343	8.912186	9.567642
ATG2B	10.11609	9.64405	11.819	10.93788	9.498772	8.621029	9.118038	10.01208
ATG3	36.57005	40.61321	42.16982	40.92912	39.06098	39.71208	38.0718	39.64927
ATG4A	7.514406	7.947107	9.562143	8.069184	8.514763	10.21174	7.880684	7.043694
ATG4B	32.26855	32.10311	33.35659	31.35679	33.21282	31.45542	29.97821	29.52913
ATG4C	4.478266	5.359689	4.1491	4.412687	4.448084	4.50886	4.863871	4.960033
ATG4D	13.67003	11.49516	10.2049	15.87114	12.82786	14.27318	13.61155	15.06281
ATG5	7.054586	7.273818	7.349379	7.024364	6.90547	7.753064	6.699398	7.298705
ATG7	7.151874	8.365581	6.66288	8.20941	7.650813	6.79855	6.576004	6.077113
ATG9A	10.15158	8.114814	8.324771	8.20104	8.727081	8.251635	9.00364	9.541701
ATG9B	21.44691	15.74456	19.52659	20.47249	19.36612	18.34779	18.0218	19.12772
ATIC	13.9485	13.5609	13.3297	16.25655	12.02471	13.21889	14.62549	11.91386

ATL1	2.30499	2.645017	1.800111	2.253622	2.74047	2.746994	1.981468	2.540572
ATL2	48.48817	50.34781	52.58618	47.59905	48.12603	54.21786	48.35693	49.61432
ATL3	21.12878	18.76956	17.10075	18.85482	20.07006	18.80259	18.37342	18.85475
ATM	2.297691	2.68311	1.666077	1.867099	2.293083	1.802319	2.506384	2.86015
ATMIN	15.80259	12.11514	14.05201	13.88616	14.26921	14.67142	12.92313	11.06679
ATN1	19.93675	17.00168	16.38049	19.53126	21.27934	16.70048	16.43479	17.21058
ATOH1	0	0.023396	0	0	0.023096	0.069753	0.140639	0
ATOH7	0	0	0	0	0	0	0	0
ATOH8	3.26647	3.698673	3.023368	3.793699	4.176498	4.075514	3.029427	3.131082
ATOX1	35.04308	39.48601	48.69826	41.53155	46.53598	46.31029	40.24882	33.11341
ATP10A	4.988545	3.902118	3.696133	3.679383	3.618115	3.834064	2.987225	3.619489
ATP10B	1.240605	0.757355	0.589013	0.831258	1.348139	0.740641	1.166388	0.84691
ATP10D	8.349371	7.584977	4.767111	8.034022	6.911026	6.133799	6.600942	6.360239
ATP11A	8.004181	7.460489	6.262478	7.810967	7.524902	6.886663	7.555546	9.05171
ATP11B	14.47243	16.02331	13.59911	14.18714	14.62469	13.24235	14.76074	15.75282
ATP11C	4.886925	6.13913	5.6146	6.050719	6.612576	6.395024	5.289058	5.618284
ATP12A	6.304797	2.966891	3.939882	1.948882	2.708312	0.922696	6.539246	3.83673
ATP13A1	10.54242	9.070532	7.1999	9.137446	10.72607	8.493017	9.028128	10.21185
ATP13A2	10.82174	8.963947	9.348459	9.529958	9.140926	8.579374	8.705416	10.24266
ATP13A3	7.32783	7.638567	6.674812	6.917153	7.930935	6.509724	7.07538	9.251826
ATP13A4	5.408252	4.091334	4.96258	4.377885	4.04535	4.327397	4.731539	3.404406
ATP13A5	0.421423	0.477681	0.869429	1.227172	0.67186	0.806594	0.90631	0.60093
ATP1A1	111.2543	110.0423	89.94572	114.9443	111.2313	101.5727	104.3003	113.8308
ATP1A2	0.679085	0.53939	0.466754	0.72931	0.874774	0.661845	0.882267	0.920173
ATP1A3	0.015602	0	0	0.015988	0	0.023562	0	0
ATP1B1	37.10046	44.363	36.13489	44.38268	36.59554	44.2236	39.82117	37.11171
ATP1B2	11.55594	11.0017	10.21202	9.508577	8.906022	9.0427	10.54773	10.70352
ATP1B3	34.12182	37.98325	35.87972	49.76499	44.07526	32.4882	32.48036	32.61863
ATP1B4	0.014427	0	0.010686	0.011089	0.018037	0	0.007322	0.007316
ATP2A1	0.10023	0.031733	0.037118	0.044937	0.043856	0.189215	0.057225	0.031768
ATP2A2	43.21626	39.78575	39.03539	39.16387	41.00027	39.57569	39.74273	41.38732
ATP2A3	4.043083	3.655499	2.794894	5.040932	4.974861	4.638135	3.755535	5.007751
ATP2B1	13.54882	13.35062	11.22998	11.6063	14.30062	12.67828	13.31708	14.45963
ATP2B2	1.000123	1.890433	1.822941	3.569569	1.926201	2.131869	3.170091	1.23752
ATP2B3	0.082219	0.179749	0.106835	0.128601	0.125509	0.074067	0.096629	0.079001
ATP2B4	31.26853	30.81165	21.00862	26.53267	26.12378	25.27918	22.27197	26.85298
ATP2C1	36.17091	36.12921	38.0693	33.4573	35.74423	34.03621	35.45985	37.23595
ATP2C2	9.311917	8.451029	5.828752	7.946065	5.262002	6.75827	7.328149	8.144282
ATP4A	0.252021	0.240309	0.197641	0.129132	0.185334	0.276132	0.195613	0.18043
ATP4B	0	0.022831	0	0.023094	0.022538	0	0	0
ATP5F1A	271.1849	283.9406	273.7382	276.4278	265.39	271.7532	294.0716	274.6542
ATP5F1B	272.5351	281.1755	287.2475	278.6297	274.6479	270.2611	280.7608	261.92
ATP5F1C	156.998	177.2162	181.3118	183.1339	179.1773	179.9518	170.9792	165.1491
ATP5F1D	95.98293	93.54084	94.972	94.64014	97.79905	98.52519	96.35288	92.83579
ATP5F1E	320.9605	354.4218	405.2366	336.6891	323.2387	409.673	330.8651	308.552
ATP5IF1	107.6747	116.9705	144.2355	116.3887	131.0856	131.228	124.5014	120.5944
ATP5MC1	0.267291	0	0	0.195651	0.229136	0.461343	0.155029	0.19364
ATP5MC2	332.6875	374.3388	362.307	350.5031	331.9552	325.6764	344.925	330.2646
ATP5MC3	150.818	161.6396	168.4802	170.8611	179.7709	169.9155	168.3539	162.6564
ATP5MD	117.1785	153.9856	205.0341	142.2707	158.3472	184.5744	144.5891	127.603
ATP5ME	214.0439	242.7878	262.4697	239.3366	237.823	283.434	245.507	253.8462
ATP5MF	179.2699	222.0671	244.1488	198.7904	204.8495	251.0206	184.7847	186.9145
ATP5MG	134.3023	158.6408	162.4617	143.9651	140.6902	141.7792	149.3947	139.4128
ATP5PB	146.7928	163.8349	177.2185	155.8098	141.4214	191.9992	161.1096	161.2463
ATP5PD	197.6794	241.9434	266.4255	219.8827	220.1941	244.1662	224.7682	213.725
ATP5PF	92.73721	120.5406	125.228	108.5664	127.7318	108.053	103.8821	106.2917
ATP5PO	265.3885	320.3177	320.227	280.3779	298.3887	325.0415	301.6019	272.8343
ATP6AP1	54.90488	53.94716	60.62046	61.14037	53.29497	57.00121	55.39217	50.7536

ATP6AP1L	0.13256	0.038371	0.074805	0.038813	0.094698	0.0572	0.115327	0.115241
ATP6AP2	68.34053	67.99545	82.99771	85.62424	73.58914	85.9313	75.53468	71.66922
ATP6V0A1	20.53171	19.37854	18.80797	20.8588	20.07256	19.17539	20.04359	21.7093
ATP6V0A2	9.803336	8.826226	9.098566	7.839437	9.484995	9.548558	9.44139	9.43428
ATP6V0A4	4.878933	4.253474	3.294132	4.616698	4.27561	4.178802	4.04731	3.276241
ATP6V0B	65.27161	64.90979	63.0277	71.70616	64.54195	72.34981	66.02874	63.8444
ATP6V0C	142.0988	114.3348	120.3657	131.6452	141.4593	134.2783	137.4316	131.8083
ATP6V0D1	76.3138	70.20449	75.37987	78.7317	84.3731	78.753	76.15215	74.58056
ATP6V0D2	1.618124	0.369225	0.561446	1.434123	3.484518	0.9687	0.399502	0.665335
ATP6V0E1	209.8391	252.0792	256.3106	253.8676	258.3043	240.0942	265.3123	220.6418
ATP6V0E2	2.619564	2.166718	2.542225	2.290191	2.223805	2.341244	2.762226	2.421583
ATP6V1A	35.88799	31.47688	33.40676	32.73299	36.98359	32.39257	30.46706	30.56126
ATP6V1B1	71.062	53.81715	64.41211	85.56215	48.45136	60.79887	40.77404	66.01363
ATP6V1B2	60.59374	56.84659	57.73884	54.89871	53.23245	54.71772	59.0934	52.7294
ATP6V1C1	11.95378	13.6209	13.29095	12.97003	11.90563	13.0646	13.27146	11.92236
ATP6V1C2	47.901	34.95276	37.5871	39.98238	32.20333	41.37722	40.47072	35.69863
ATP6V1D	88.34807	86.50302	93.3534	81.91585	81.53576	98.14342	86.82059	78.16422
ATP6V1E1	111.9795	113.4723	131.0887	117.1669	110.1873	115.3001	108.6691	95.78499
ATP6V1E2	0.086977	0.158613	0.12025	0.356525	0.208772	0.350284	0.211876	0.211716
ATP6V1F	95.24615	89.23506	99.38629	91.57515	91.7122	90.162	92.69298	87.76438
ATP6V1FN	0	0	0	0.051867	0	0	0	0
ATP6V1G1	158.2588	175.5295	186.2245	170.0153	168.3527	184.8543	166.4853	158.0269
ATP6V1G2	0.301973	0.401542	0.167744	0.096704	0.169882	0.266032	0.402285	0.325414
ATP6V1G3	0.075851	0.102462	0.049938	0.07773	0	0.127283	0	0.025644
ATP6V1H	31.88863	29.69173	33.08799	31.13301	32.4363	31.43616	31.77395	29.93101
ATP7A	6.134837	6.103865	5.384638	6.439036	6.922532	5.556303	6.042567	7.098297
ATP7B	0.176217	0.36131	0.236172	0.292371	0.386049	0.295702	0.344945	0.293621
ATP8A1	5.114909	5.636844	6.592726	5.78877	6.013516	5.766191	5.764646	7.443555
ATP8B1	9.53269	8.10778	7.665541	8.368778	8.67418	7.521635	9.608163	10.54026
ATP8B2	14.64783	14.92072	11.30681	16.21997	14.74808	11.53241	14.86755	14.78986
ATP8B3	0.431651	0.241675	0.252398	0.448164	0.306739	0.268766	0.380478	0.316826
ATP8B4	1.554954	1.480296	1.257586	1.584312	1.787534	1.435112	2.217753	2.551441
ATP9A	13.0453	12.30969	10.65648	11.64789	11.40117	11.08296	12.10677	14.15574
ATP9B	13.9127	9.161218	11.97447	14.41785	12.01318	13.63497	10.91264	11.8721
ATPAF1	22.22966	28.8391	26.25768	27.42511	22.75226	26.52356	25.90362	24.3176
ATPAF2	9.07235	8.778562	8.366256	9.20916	9.45963	8.270562	8.119968	8.418394
ATPSCKMT	6.025376	6.33614	7.413326	4.45266	6.135541	6.707229	6.600173	6.47419
ATR	2.7812	2.80754	2.386722	2.548639	2.774873	2.4771	2.507375	2.888599
ATRAID	36.71759	36.49204	36.7198	40.01234	38.87563	26.77428	38.21336	36.35502
ATRIP	4.337885	3.93104	4.133146	4.154937	4.011427	3.357942	4.203824	3.614777
ATRN	8.512281	7.997348	7.442892	7.589461	7.267986	7.667849	7.973438	8.401317
ATRNL1	2.624503	3.079667	2.732874	2.886971	2.658068	2.458466	2.650383	3.48401
ATRX	8.013515	8.759246	7.480663	7.879362	8.57179	7.812163	8.753336	9.093175
ATXN1	2.220983	2.078905	2.167911	2.221404	2.331507	2.197315	2.297184	2.682998
ATXN10	49.16146	58.88666	50.86992	51.00882	51.39163	51.64765	52.36363	56.1224
ATXN1L	6.939491	6.334961	5.787536	6.045216	6.66011	6.299142	6.453359	6.910375
ATXN2	11.6684	12.0116	10.30649	11.57295	12.54547	10.92965	11.13892	12.3292
ATXN2L	21.49868	18.51313	17.6699	20.02943	19.44558	17.12319	17.64251	20.37066
ATXN3	15.29615	13.73688	14.15482	13.29403	13.17339	13.39705	12.28291	13.0919
ATXN7	5.35584	4.885085	4.354085	4.676863	5.415877	4.602786	4.270908	5.136407
ATXN7L1	3.016692	3.3029	2.687203	2.650422	3.117072	3.186102	2.894286	3.346202
ATXN7L2	0.905253	0.666522	0.58732	0.701151	0.663236	0.67298	0.608993	0.635225
ATXN7L3	17.82203	18.74463	15.25039	18.93739	18.09776	17.23704	17.86437	18.06825
ATXN7L3B	49.51929	49.25772	45.44722	45.78163	47.00327	44.72473	46.80461	46.87855
AUH	6.446887	6.771551	6.415306	6.009659	6.516838	6.490929	7.505588	8.8317
AUNIP	2.075601	1.932915	1.511423	2.019589	2.32749	2.723047	1.744972	1.190791
AUP1	53.65534	60.80765	58.99233	59.86158	59.29106	63.39633	58.35155	58.30761
AURKA	4.302791	4.941442	3.017331	4.13648	3.966929	4.205183	3.826751	5.216952

AURKAIP1	26.42371	27.53615	28.4667	29.78883	30.28727	29.51194	30.62803	26.14288
AURKB	11.4325	11.77885	10.16317	10.89934	10.16353	10.79525	12.93702	12.62157
AURKC	0.018222	0.036923	0.017995	0.112041	0.091122	0.05504	0.129468	0
AUTS2	4.305278	4.588238	3.162332	4.845146	3.887886	4.222164	3.935831	4.405993
AVEN	12.80834	15.23867	14.14944	13.09382	14.6555	15.12841	13.99202	13.27376
AVIL	0.229243	0.188705	0.290058	0.278969	0.236438	0.512108	0.138155	0.167114
AVL9	5.371485	5.537529	4.462092	5.16627	5.475078	4.911819	4.694948	5.100117
AVP	0.111733	0.1132	0	0	0	0.112497	0	0
AVPI1	10.61027	9.711636	10.60453	9.917009	11.80088	9.946014	9.841027	6.790761
AVPR1A	2.239133	0.654381	1.275711	2.051899	2.627015	1.170569	1.114507	1.069994
AVPR1B	0	0	0	0	0	0	0	0
AVPR2	0.527475	0.356265	0.368973	0.720722	0.846268	0.674913	0.423848	0.646439
AWAT1	12.45389	13.7555	16.30848	15.88719	22.71203	31.12122	12.68957	6.348146
AWAT2	1.020357	3.896434	2.526858	2.203869	7.221918	6.954214	1.354327	1.958315
AXDND1	0.077063	0.163955	0.281579	0.2764	0.39307	0.294839	0.031287	0.07816
AXIN1	9.737123	7.675751	7.795331	9.452412	9.508767	8.396324	8.265683	8.266307
AXIN2	7.922954	8.755331	9.024138	9.585381	9.369277	9.048412	8.129368	8.77223
AXL	18.76004	17.31844	20.0463	17.7402	18.71417	16.57884	19.05982	19.8046
AZGP1	1.873291	3.014273	4.483406	3.071508	7.031258	3.03993	2.885639	2.19054
AZI2	23.74163	25.60718	28.74085	29.75722	26.44564	24.84097	25.77435	25.74164
AZIN1	20.1579	25.53463	22.48462	25.05816	25.02572	21.93256	23.22819	22.2778
AZIN2	0.424316	0.445807	0.294872	0.354303	0.377219	0.253165	0.239267	0.382539
AZU1	0	0	0	0	0	0.029233	0	0
B2M	216.087	270.0183	275.461	269.3433	328.8555	269.9974	266.9638	253.4704
B3GALNT1	1.313902	3.335603	1.674719	1.980531	2.207946	2.068919	1.938387	1.960171
B3GALNT2	14.59311	13.4841	13.72688	13.5019	13.85462	14.17741	12.8456	13.81839
B3GALT1	0	0.014159	0	0.009548	0	0	0	0
B3GALT2	0.454932	0.907174	0.677461	0.495801	0.881094	0.7634	0.447097	0.55662
B3GALT4	6.878383	7.895337	6.702349	7.498684	6.787792	9.356621	8.80122	7.495813
B3GALT5	0	0.017233	0	0.017431	0	0	0	0
B3GALT6	3.532066	3.236261	3.34909	3.201361	3.870294	2.55026	3.527915	3.268357
B3GAT1	0.388738	0.496989	0.411316	0.758795	0.259192	0.270248	0.450937	0.469372
B3GAT2	0.043461	0.11008	0	0.022269	0.043467	0.021879	0.088227	0.06612
B3GAT3	7.756624	9.066137	7.047177	8.802204	8.835528	9.272888	8.071869	7.5358
B3GNT2	18.17124	16.94636	17.25656	17.18257	18.89083	17.10552	18.15675	15.56145
B3GNT3	0.315535	0.142078	0.334686	0.371255	0.455832	0.329457	0.498195	0.474115
B3GNT4	0	0	0.016346	0.016962	0	0.016665	0.0168	0.016787
B3GNT5	1.610625	1.757283	1.626542	1.654255	1.993497	2.025201	1.35369	1.944002
B3GNT6	0.084262	0	0.097081	0.043175	0.056182	0.084838	0.014254	0.028487
B3GNT7	0.145253	0.237719	0.231716	0.366403	0.100573	0.31499	0.170114	0.385301
B3GNT8	1.788644	1.28534	1.247747	1.577189	1.627669	1.53911	1.599095	1.466051
B3GNT9	3.496733	3.682158	3.551393	4.67325	4.109383	4.722408	4.162719	3.399063
B3GNTL1	1.662192	1.553632	1.419086	0.912124	1.640956	1.22007	1.153776	1.587966
B4GALNT1	0.396398	0.244453	0.567332	0.518074	0.517108	0.289207	0.460655	0.559362
B4GALNT2	0	0	0	0	0	0	0	0
B4GALNT3	14.8581	12.28801	11.20603	12.25518	12.56645	13.19797	13.24182	13.12274
B4GALNT4	5.896256	5.445357	4.832517	6.239608	5.174934	5.209613	5.626432	5.850012
B4GALT1	40.87012	41.16205	34.18393	39.23868	43.80833	32.55319	43.51777	46.27105
B4GALT2	14.42546	13.07782	11.16964	13.7709	13.18839	12.01145	13.43004	13.22873
B4GALT3	10.84456	9.51103	9.811855	11.17698	11.53083	10.89357	11.66437	9.774025
B4GALT4	5.698077	5.318513	5.014873	4.906567	4.551136	5.29876	5.234646	6.100259
B4GALT5	1.987589	2.191934	2.245985	2.484255	2.607011	2.145514	3.624711	2.392623
B4GALT6	0.527124	1.155157	1.629568	1.409169	1.919669	1.153751	1.035169	1.121558
B4GALT7	11.11741	12.87236	11.16073	11.34561	13.0986	11.86542	12.84947	11.43909
B4GAT1	5.481155	5.264439	5.654026	5.742054	5.224053	4.767305	5.040085	4.939967
B9D1	4.39723	4.568441	3.411259	4.534853	4.397799	4.596465	3.761966	3.844353
B9D2	2.265789	2.781637	3.211553	2.51313	3.252495	1.637146	2.218608	2.892832
BAALC	0.834069	0.81731	0.688647	0.798681	0.943577	0.839767	1.09639	0.568584

BABAM1	23.73538	24.3185	22.71597	24.10353	23.07706	23.69959	24.90776	22.13363
BABAM2	19.41799	19.75567	19.10876	19.80123	18.889	20.36011	20.98172	18.68551
BAC5	0	0	0.044084	0.045746	0	0	0	0
BACE1	6.287956	6.84209	5.662345	6.042399	6.148362	5.252203	6.502272	6.130165
BACE2	6.550351	5.274279	4.971602	6.272915	6.85158	5.457517	6.053426	5.976339
BACH1	13.61561	11.78379	13.89052	12.60183	13.3463	11.95573	13.27898	13.29228
BACH2	4.09214	3.505154	2.957343	2.887105	3.225946	2.914049	3.161839	3.454702
BAD	9.209182	11.65034	9.808657	10.72196	10.3677	12.01489	12.1613	9.633693
BAG1	84.39757	75.37041	77.90112	85.91518	77.40598	72.26958	89.09461	86.50631
BAG2	15.60844	15.54473	17.00001	14.86055	15.5013	15.41678	14.18076	16.52649
BAG3	31.10969	26.05528	24.40155	26.95303	26.45355	26.57739	23.78729	25.90087
BAG4	5.751296	6.008409	5.049351	5.485898	4.715669	4.797811	5.983141	5.934996
BAG5	18.73586	22.65293	18.21312	21.954	23.0821	21.92875	21.53784	19.68724
BAG6	47.88695	46.22299	46.34908	47.8174	47.4133	46.03382	47.83531	45.76737
BAHCC1	1.862983	2.030889	1.54091	2.26478	2.118688	1.646487	1.721843	1.568485
BAHD1	14.29497	13.15857	14.03123	13.93185	14.17293	15.29685	13.11378	13.10391
BAIAP2	25.68569	23.15454	19.77402	22.90641	21.51905	20.44535	20.01039	24.41987
BAIAP2L1	25.0612	25.42617	29.74091	25.6455	24.90423	29.30938	23.89247	23.08919
BAIAP2L2	0.050137	0.063494	0.024756	0.141293	0.025072	0.08834	0.089056	0.13984
BAIAP3	0.11112	0.099334	0.167831	0.087079	0.228806	0.164528	0.07298	0.086184
BAK1	3.796026	3.369836	5.133325	4.340965	4.214208	4.272484	4.95057	5.067865
BAMBI	166.3264	141.0944	182.4691	159.3837	161.1998	171.7095	165.0842	162.0233
BANF1	187.5467	206.1374	216.749	199.5602	204.5849	201.787	179.1852	185.2357
BANF2	0	0	0	0	0	0	0	0
BANK1	0.560718	0.986244	0.599875	0.582588	0.716565	0.690004	0.687697	1.405953
BANP	7.986982	5.652535	5.281171	3.950067	6.008376	3.974155	5.275286	8.276784
BAP1	17.51908	16.87848	15.23444	18.70312	17.68049	16.61344	16.97434	16.72755
BARD1	1.959025	1.897012	1.646026	1.752448	1.840206	1.754463	2.263053	1.635636
BARHL2	0	0	0	0	0	0	0	0
BARX1	0.022108	0.067195	0.043666	0.045312	0.309557	0.178075	0	0
BARX2	61.33948	49.93722	57.03381	53.52468	62.04678	52.33498	61.7136	58.88474
BASP1	7.490434	9.411655	12.74092	10.06618	11.33041	8.82985	9.496724	11.86872
BATF	0.59326	0.515182	0.313858	0.303978	0.317859	0.40532	0.494631	0.279363
BATF2	0.030961	0.031368	0.168166	0.126914	0.139345	0.26497	0.078565	0.015701
BATF3	1.135975	1.822232	1.152982	1.325796	1.956654	0.69895	1.601409	1.952248
BAX	14.78419	14.53568	15.16407	14.11742	18.51623	15.35881	15.75625	14.34716
BAZ1A	21.31441	21.07624	23.27968	21.54802	23.13823	22.92076	21.19433	20.96604
BAZ1B	21.85553	21.74946	18.3433	19.62869	20.49416	19.25816	19.6723	19.91518
BAZ2A	21.77144	20.06757	18.06757	18.99305	19.22033	17.40664	18.1433	19.47552
BAZ2B	4.900222	5.230643	3.801603	5.232886	5.392353	4.95076	4.747256	5.537159
BBC3	1.232414	1.113199	1.173068	1.323806	1.098921	1.270733	1.567393	1.73187
BBIP1	13.93473	20.08484	18.67915	18.13203	15.73247	14.82543	17.15719	11.77758
BBOF1	0.059028	0.039869	0.009715	0.080654	0.039357	0.049526	0.059914	0.019956
BBOX1	2.450433	3.611968	3.153804	3.715239	3.66608	2.709846	4.250647	2.933488
BBS1	4.771314	5.621076	4.344704	5.436578	5.019823	4.439177	4.194585	5.404861
BBS10	1.196053	2.71679	2.392393	2.326449	1.80574	3.18314	1.492362	2.549785
BBS12	4.149958	3.421933	3.62012	3.780226	2.790055	3.0757	4.142004	3.636139
BBS2	8.047536	9.156454	7.195773	7.712752	7.933895	7.945081	8.982958	7.189413
BBS4	10.46398	11.62505	10.38169	12.53844	10.84888	12.31283	11.41797	9.878246
BBS5	5.790862	6.285922	7.333171	6.802246	6.461388	7.297943	6.417516	5.533686
BBS7	2.499764	3.196852	3.666503	4.023104	3.270606	3.06147	2.736914	3.183735
BBS9	3.140965	3.457927	2.949279	2.796635	3.089874	3.229078	3.016357	2.670897
BBX	7.6893	7.655306	6.55547	7.911704	8.038822	7.25119	7.266884	7.534397
BCAM	65.4	75.1661	65.03627	71.22474	67.1413	71.49674	64.02743	54.03
BCAN	0.125437	0.101667	0.107357	0.085696	0.108726	0.033678	0.084879	0.203556
BCAP29	5.770962	10.7992	12.55468	12.26267	12.05268	11.99674	10.31958	9.86422
BCAP31	105.9882	113.8805	110.0031	119.1472	107.7186	117.8666	109.8745	103.501
BCAR1	1.873226	2.15786	1.591009	2.249825	2.113796	1.842034	1.79046	2.392868

BCAR3	2.586901	2.516659	2.144515	2.432092	2.057129	1.979317	2.103776	2.262664
BCAS1	6.188381	6.6816	8.363714	7.899908	5.526055	9.248076	6.442724	4.698391
BCAS2	33.33442	30.91542	30.45376	37.26012	29.43208	33.47343	31.33039	31.69403
BCAS3	7.395055	6.606677	7.583226	6.759172	6.947768	7.407971	6.383875	5.826013
BCAS4	8.117951	7.864496	8.151763	7.030565	8.091664	10.26492	8.128748	7.734514
BCAT1	0.070369	0.095056	0.092656	0.168261	0.093837	0.029521	0.124992	0.166531
BCAT2	15.6344	12.13761	17.9537	16.48206	16.14565	14.56514	11.20248	7.776595
BCCIP	31.83623	30.97043	35.62542	31.79859	31.86339	33.88603	29.08708	28.34088
BCDIN3D	2.870075	2.747536	3.162251	2.673789	2.174581	2.085666	2.423593	1.667972
BCHE	3.338897	6.357174	5.18471	5.651543	3.512052	3.454443	5.036726	3.655007
BCKDHA	40.65093	44.43361	40.24724	37.43233	37.78193	43.61248	35.34215	29.84501
BCKDHB	23.24268	34.99718	25.68736	23.6254	35.33948	27.91488	20.2266	15.26359
BCKDK	10.51942	9.311696	9.831825	10.29874	10.23897	9.402464	9.819219	10.23369
BCL10	11.9257	10.79777	11.24053	11.4787	12.58212	11.68455	12.47224	10.35745
BCL11A	2.549498	2.406846	1.955923	2.704416	2.017735	2.277872	2.119915	2.620518
BCL11B	4.089946	4.321965	3.79487	4.152874	4.225326	3.865233	3.333962	3.513788
BCL2	4.686227	4.141635	3.629123	5.196416	4.872026	3.198078	3.397514	3.799461
BCL2A1	4.389847	6.876213	5.810944	7.912404	8.376038	4.075026	4.613698	5.936452
BCL2L1	25.66259	25.20841	27.0159	24.85014	25.16618	26.19949	24.97773	27.38197
BCL2L10	0.135083	0	0.02668	0	0.1351	0.027201	0.027422	0
BCL2L11	3.504114	3.067004	2.548897	2.86466	2.905159	2.34763	2.828467	2.888455
BCL2L12	7.012147	6.652638	6.86609	8.738713	7.339933	6.371995	5.488788	6.50926
BCL2L13	17.74237	15.92541	15.29519	17.75483	17.93547	17.44909	16.4441	17.50102
BCL2L14	0.161243	0.149746	0.026539	0.289166	0.161264	0.189402	0.190939	0.218052
BCL2L15	0.375869	0.859019	0.517934	0.582249	0.874228	0.756874	0.47023	0.895424
BCL3	4.980883	4.759533	6.386082	7.04736	7.019424	5.385329	5.170496	5.281415
BCL6	9.261402	7.872367	8.056134	8.959953	10.12759	8.095593	8.008106	10.06484
BCL6B	2.927397	3.245921	2.425161	3.208236	3.529595	3.184824	3.268438	2.45773
BCL7A	6.501151	5.646648	4.835759	6.085576	5.368896	6.56853	5.379265	6.601411
BCL7B	11.79601	10.25765	9.982596	12.80319	12.70629	11.10878	12.02236	11.30567
BCL7C	36.55861	44.26782	38.96687	42.50615	40.70196	40.77789	43.6222	42.00309
BCL9	3.363827	3.296017	2.894124	3.506037	3.4571	2.999617	3.598234	3.564142
BCL9L	11.98604	10.75327	9.147354	11.34735	11.82323	9.44686	8.848358	10.45024
BCLAF1	28.67124	34.28166	29.74712	31.07185	30.64886	30.41104	31.14385	31.36571
BCLAF3	3.770601	4.675095	3.715973	3.943343	3.833037	3.843133	4.267244	4.66452
BCO1	0.232637	0.412458	0.186663	0.3874	0.407168	0.219587	0.280401	0.339177
BCO2	14.46866	21.78198	10.50652	22.20904	21.49028	20.32012	17.79781	12.82625
BCOR	7.935174	6.327975	6.439732	7.415172	5.967865	5.497645	6.168242	6.880758
BCORL1	1.786542	1.533463	1.340714	1.551091	1.886039	1.495387	1.381593	1.740071
BCR	14.05198	13.71998	11.75001	13.24711	13.52563	13.51456	11.8297	14.40572
BDH1	11.93874	12.36644	15.9972	15.9708	14.71479	18.42448	12.93238	11.64646
BDH2	12.46537	13.96698	9.953295	15.52842	12.69871	10.05442	11.00614	12.80732
BDKRB1	0.398625	0.245199	0.449895	0.466856	0.45563	0.387014	0.592456	0.721964
BDKRB2	0.952316	1.017578	1.175563	1.311373	1.205431	1.086165	1.034565	1.207341
BDNF	2.394309	2.517848	1.795804	2.018798	1.379179	1.525738	1.86112	3.043176
BDP1	9.526869	9.104908	8.264254	9.207059	9.336655	8.697494	8.571274	10.0682
BEAN1	0.185114	0.168789	0.082263	0.170729	0.166624	0.288886	0.225468	0.253461
BECN1	53.32784	52.14802	54.46942	53.18207	51.04176	57.2147	55.36527	53.28053
BECN2	0.043193	0	0	0	0	0	0	0
BEGAIN	0.020267	0.051333	0.020015	0.103847	0.091215	0.020406	0.051428	0.133613
BEND2	0.03716	0.00753	0.007339	0.030465	0.007433	0.014966	0.007544	0.007538
BEND3	1.791404	1.566863	1.390282	1.802324	1.648795	1.594108	1.399948	1.514778
BEND4	0	0	0.015263	0	0	0.015561	0	0
BEND5	2.579923	3.160852	1.955271	3.197188	2.460245	2.416324	2.801316	2.129831
BEND6	0.383906	0.704962	0.438361	0.209002	0.443949	0.229502	0.426196	0.328532
BEND7	1.949311	1.884844	2.76949	2.311684	1.83921	1.996573	2.012771	2.511738
BEST1	0.019144	0.019396	0.075624	0.049047	0.02872	0.048188	0.009716	0.029125
BEST2	0.014435	0	0.042765	0.029585	0.014437	0	0.043954	0.029281

BEST3	0	0	0	0	0.011897	0	0	0
BEST4	0	0	0	0	0	0	0.015257	0.060983
BET1	17.95565	22.43289	20.60111	20.15725	20.35839	20.622	17.78539	17.69879
BET1L	20.14035	23.06778	20.40126	21.43597	20.75391	21.02345	20.81824	22.54862
BEX2	27.85444	33.95272	33.72729	35.79268	28.49807	33.84358	33.7421	24.18583
BEX4	1.551856	1.393347	1.459106	1.476023	1.7844	1.234996	1.056377	1.140405
BEX5	2.196618	7.017335	4.723362	9.966249	9.886059	4.744291	5.573918	1.832618
BFAR	11.87109	10.20212	10.43969	12.69999	11.18688	11.84915	11.82064	11.87401
BFSP1	0.066124	0.143553	0.083957	0.212965	0.113369	0.133151	0.134231	0.13413
BFSP2	0.072652	0.036803	0.017937	0.037226	0.308811	0.109722	0.165919	0.073686
BGLAP	0.538253	1.772281	0.465103	1.103172	2.01871	0.880637	1.843854	8.120494
BGN	181.5964	135.1478	139.126	155.127	146.0265	130.7987	136.3996	157.1977
BHLHA15	0.151679	0.271181	0.449366	0.356588	0.223086	0.296446	0.389413	0.199084
BHLHE22	6.021137	7.070638	5.883048	6.45075	5.948923	5.841825	7.157809	7.679829
BHLHE23	0	0	0	0.014363	0	0	0.028452	0.014215
BHLHE40	39.44236	39.41072	41.51639	43.82236	41.52602	37.0962	43.38162	39.43105
BHLHE41	11.73111	13.24125	13.8122	13.45997	13.31483	12.12164	10.94364	11.90634
BHMG1	0.011513	0.046658	0.02274	0.070792	0.080604	0.011592	0.058431	0.023355
BHMT2	0.03352	0.01698	0.016551	0.017175	0.134097	0.016874	0	0
BICC1	6.81527	7.266007	5.11624	6.400387	7.247114	5.919325	6.620891	6.570694
BICD1	0.112029	0.104297	0.062792	0.105496	0.133241	0.079261	0.101417	0.138192
BICD2	14.16614	11.65262	9.875034	11.55695	11.7728	10.39931	12.0438	11.60859
BICDL1	3.02511	2.021471	2.415358	3.194269	2.427758	2.110751	2.977159	2.890985
BICDL2	24.49787	25.33148	26.93228	26.28523	24.66563	27.90816	27.89595	24.65585
BICRA	2.478136	2.701283	1.613377	2.342002	2.326995	2.2548	1.816611	2.443596
BICRAL	3.818378	3.62386	3.229228	3.831179	3.55282	3.640497	3.697033	4.013864
BID	30.38943	32.52221	29.39242	33.30246	28.11803	29.10501	29.57417	28.55696
BIIIB4	1419.397	1388.928	1633.048	1763.917	1660.452	1790.715	1585.872	1658.026
BIN1	7.000051	7.472244	8.210348	7.467624	9.419268	7.859378	8.057622	9.283364
BIN2	2.392236	3.406549	2.874288	3.486568	3.974284	2.368436	4.006398	4.367324
BIN3	13.16965	13.44604	13.56788	14.48337	13.02533	13.64183	11.98898	12.46863
BIRC2	15.62789	15.67867	14.33	15.06682	15.38728	14.74059	14.7342	15.64437
BIRC3	16.91228	16.31638	15.82462	15.74758	18.61572	13.96827	18.47112	17.6209
BIRC5	8.571412	10.92315	10.33854	9.601334	8.7127	10.6382	12.12526	10.79298
BIRC6	10.05517	9.281451	8.144989	8.651999	9.573957	7.84693	9.055594	10.64625
BIVM	10.29521	10.20986	10.16693	8.689326	9.305912	9.821578	9.238638	9.977521
BLCAP	3.765988	3.968459	3.975551	4.253077	4.166713	3.906455	3.833238	4.160971
BLK	1.300535	1.850251	0.505534	0.921582	0.816399	1.392999	0.617892	1.417276
BLM	2.436747	2.714983	1.81829	2.391243	1.902245	2.098538	2.504137	2.570046
BLMH	115.5872	104.5888	113.0244	108.8935	109.0129	119.8104	108.6794	102.7154
BLNK	2.053869	2.013429	1.88868	2.062138	1.987604	2.360923	1.527638	2.091541
BLOC1S1	0	0	0	0	0	0	0	0
BLOC1S2	17.06656	14.27256	21.48108	19.17464	19.14806	23.99393	21.16502	22.21913
BLOC1S3	4.295624	4.720814	4.005857	4.263466	5.44044	4.209777	3.906106	3.808222
BLOC1S4	8.858956	8.715069	7.861035	8.771396	9.116915	7.325174	9.38279	7.596073
BLOC1S5	4.391427	3.665807	3.630939	3.638855	3.870529	3.769753	3.599599	3.574094
BLOC1S6	17.01805	18.81766	19.59381	16.93827	17.29296	16.29455	16.54071	17.83783
BLVRA	27.95556	31.20462	30.43874	33.50305	38.20864	29.09378	25.51012	23.29851
BLVRB	20.34096	23.67437	21.72665	25.51405	21.77578	20.38162	22.8263	19.80531
BLZF1	33.81391	34.45268	36.98394	32.78293	36.11809	36.53351	34.61929	35.42543
BMF	6.196903	5.942879	5.776729	5.766521	5.551884	5.714748	4.918426	6.252098
BMI1	21.1545	24.24406	23.8117	22.12292	21.29603	22.23342	21.48062	21.73715
BMP1	8.695283	7.165067	6.25675	8.169226	8.246593	7.114002	7.456205	8.766178
BMP2	11.69564	12.60651	13.91546	12.98207	13.96423	13.3699	11.13679	10.54956
BMP2K	14.45074	15.3777	13.28033	14.6945	13.2163	13.22073	16.77396	16.28456
BMP3	1.990317	1.990114	2.97137	2.561505	2.063337	4.405472	1.561282	2.245288
BMP4	41.43663	38.69845	37.89182	40.01381	41.47079	47.01078	37.01646	38.40505
BMP5	0.00708	0.107601	0	0.007256	0.021244	0.014258	0.02156	0.064632

BMP6	1.805412	1.604479	1.36586	1.655382	2.745426	1.403172	1.939657	1.627656
BMP7	26.55826	22.46217	20.18682	18.62749	20.44417	21.67356	21.68979	19.04202
BMP8A	8.509061	5.438584	6.103324	6.658546	8.605353	5.558133	6.395407	6.345541
BMP8B	7.636259	5.115289	4.366721	2.828075	4.124427	5.620287	5.252084	6.584017
BMPER	0.404205	0.300865	0.382876	0.304323	0.932268	0.523243	0.594471	0.426693
BMPR1A	27.03649	29.44047	28.06016	28.9378	26.58068	28.71161	27.1693	28.07048
BMPR1B	1.460103	1.498832	1.308399	1.733773	1.390754	1.687867	1.478084	1.10479
BMPR2	27.24417	19.96627	19.73582	20.36446	20.57656	20.4964	22.87844	24.14396
BMS1	19.0032	19.68582	16.69851	18.33977	19.37043	18.55688	19.09039	19.13979
BMT2	4.684288	5.505972	5.38132	4.74806	4.881615	4.826311	5.087297	5.792786
BMX	0.982542	1.085116	1.127645	1.306222	2.142395	1.657669	0.988295	1.607015
BNC1	25.23857	20.00269	18.29235	20.04217	17.80063	18.54366	17.60629	19.44658
BNC2	11.54989	8.229702	7.914482	8.287916	8.113046	8.828852	9.826347	9.782931
BNIP1	4.138933	5.784308	5.123695	5.106291	5.937641	4.403645	4.759662	4.823065
BNIP2	8.385149	8.600127	7.515607	8.210759	8.096196	8.505	8.414212	8.403673
BNIP3	36.61285	35.25402	36.12345	33.81602	33.84353	40.28186	35.35342	33.34905
BNIP3L	59.14749	61.08508	66.26799	62.21444	55.49511	59.99593	63.4194	53.076
BNIPL	28.41305	25.80584	25.16686	25.39168	24.61412	25.64556	26.46639	26.68096
BOC	15.56312	15.15281	11.69007	15.27134	15.21652	16.32575	15.13952	14.96743
BOD1	17.29519	17.71325	18.55111	18.36142	18.14626	18.95149	18.14807	17.27359
BOD1L1	4.90167	4.868735	3.518708	4.300683	4.669122	3.811144	4.574284	5.321981
BOK	19.08854	15.53832	16.45349	15.26465	18.66799	17.10819	12.33794	13.46639
BOLA1	7.744713	10.41373	10.71312	9.687262	10.25168	11.6391	8.554491	7.999357
BOLA2B	29.06689	34.61869	39.15986	34.30202	39.05675	37.37171	34.71496	33.43501
BOLA3	15.38815	12.16983	16.08804	14.64294	17.51021	14.74233	16.53539	16.44331
BOLL	0	0.007168	0	0	0.007076	0	0	0.007176
BOP1	8.000392	7.198377	5.907445	7.206063	7.089768	6.94881	7.120837	6.867837
BORA	3.365938	3.495671	2.895095	4.191102	3.921403	4.117736	3.379692	3.438327
BORCS5	7.407747	7.663382	6.852198	8.372396	8.131982	8.442305	7.796605	6.482366
BORCS6	2.120825	2.520319	2.479316	2.684392	2.820489	2.418099	2.449353	1.871966
BORCS7	12.38127	16.0218	18.13671	16.41744	14.65306	17.24442	16.10859	13.87035
BORCS8	6.74392	5.98797	6.388631	6.12475	5.664868	6.322703	5.604889	4.774498
BPGM	11.27185	15.03341	12.05474	13.25444	13.41195	15.3933	13.91892	14.23332
BPHL	12.98321	19.39679	12.60465	19.3466	15.94883	11.05113	8.371504	11.87986
BPI	0.044404	0.059982	0.27772	0.091007	0.103622	0.208633	0.450698	0.07506
BPIFB1	0	0	0	0.015935	0	0	0.031566	0.047313
BPIFB2	0.078291	0.145418	0.206176	2.420292	0.247955	0.748849	0.9271	0.436732
BPIFB3	0	0.017333	0	0	0.034221	0	0	0.017352
BPIFB4	0	0	0	0	0	0	0	0.015262
BPIFB6	0.798719	0.378775	1.074066	0.696599	0.220951	0.461973	1.121181	0.775618
BPIFC	21.33243	21.93965	18.25127	18.29173	18.84913	22.28413	21.05374	15.98138
BPNT1	20.71639	20.57606	22.11568	19.38803	19.58308	20.00448	22.40371	18.01951
BPTF	10.43746	10.14743	8.530428	8.925596	9.827352	8.417272	8.934521	10.39579
BRAF	18.31056	16.45689	16.25907	17.7066	17.80129	16.94671	16.24565	18.98869
BRAP	15.13003	14.98984	14.37742	14.41976	14.03123	14.71439	13.70957	13.35306
BRAT1	3.236101	3.042136	2.704121	3.284374	2.847515	3.446182	2.54244	3.376847
BRCA1	2.540921	2.883467	2.298987	2.240193	2.257311	2.651178	2.784349	3.289752
BRCA2	1.411387	1.419733	1.378921	1.482378	1.353801	1.443786	1.521774	1.808452
BRCC3	11.95381	12.7393	13.83457	13.3527	13.25464	11.98666	12.18232	13.63824
BRD1	14.82655	14.39099	11.89614	13.83187	13.26534	12.24629	11.99491	13.17746
BRD2	58.0015	52.51652	48.72539	53.75815	52.30388	46.23004	52.60769	54.51684
BRD3	14.08711	12.73455	11.28717	12.09959	12.66746	12.73744	13.18641	14.74564
BRD3OS	2.369929	2.507407	2.414463	3.612208	3.180316	2.476732	2.938337	2.434093
BRD4	14.34248	12.83275	12.26969	12.46875	12.76498	12.3449	12.36522	12.69035
BRD7	39.51554	44.72738	46.99591	44.2323	42.11078	42.89489	44.7485	42.77872
BRD8	21.2785	21.21651	18.65296	20.0067	19.27298	19.61083	19.71076	21.53543
BRD9	23.18649	23.03176	20.31364	22.85543	21.02574	20.15165	22.59154	22.76983
BRF1	5.905871	6.005357	4.841491	6.952483	5.179831	5.067116	5.719231	6.495982

BRF2	3.616641	3.525838	3.032471	3.461476	3.309996	3.21767	2.805115	3.645057
BRI3	62.40087	59.19002	59.06472	59.60867	56.82498	59.66744	55.85499	54.70259
BRI3BP	10.09835	10.60812	10.75225	10.8358	10.17593	9.977632	9.27994	10.45596
BRICD5	0	0	0.056123	0	0.028419	0	0.028842	0
BRINP1	0.160725	0.449734	0.42326	0.423531	0.153092	0.077059	0.349578	0.248401
BRINP2	0.028998	0.047006	0.011455	0.03566	0.063805	0.023357	0.03532	0.035293
BRINP3	0	0	0.010514	0.005455	0.005324	0	0	0.021596
BRIP1	1.499962	1.919554	1.319352	1.599348	1.372612	1.528551	1.873798	1.619861
BRIX1	28.62083	34.64568	32.35112	34.68681	31.19507	35.13226	28.49743	28.71898
BRK1	53.77831	58.52087	61.87108	53.7577	59.04155	54.63793	56.39621	60.14702
BRMS1	18.57781	17.38608	18.00082	19.86261	17.96787	18.24679	17.77338	20.1552
BRMS1L	7.172091	8.285849	7.501203	8.098834	7.766355	6.741104	7.967854	7.113019
BROX	20.34093	21.67605	21.42779	17.87207	22.71464	18.22194	18.04459	21.19175
BRPF1	8.433084	7.546295	6.638098	8.143514	7.555004	7.747243	7.631648	7.542687
BRPF3	5.336403	4.855782	4.440388	4.442077	4.856395	4.662809	4.673283	5.243803
BRSK1	0.077694	0.265658	0.220586	0.208998	0.135982	0.224895	0.147861	0.1182
BRSK2	0.512726	0.623346	0.466956	1.325243	0.615351	0.975099	0.572459	0.456467
BRWD1	8.221958	7.252454	6.438072	6.941644	8.146647	6.484106	7.087897	8.551279
BRWD3	5.222103	4.896184	4.336964	4.319111	4.844276	4.397015	4.675879	5.089335
BSCL2	5.505867	5.866227	6.33949	6.398682	5.82124	5.555664	5.88323	5.234464
BSDC1	17.70781	15.48265	15.94852	16.73144	16.78634	16.18493	15.01889	17.2502
BSG	190.0416	189.681	210.136	198.2022	206.2343	223.9952	212.8243	186.1326
BSN	0.072865	0.028808	0.035101	0.0601	0.0551	0.030419	0.045097	0.045063
BSND	0.020974	0.095619	0.300327	0.343889	0.083905	0.179493	0.095797	0.212721
BSP5L	0	0	0	0	0	0	0	0
BSPH1	0	0	0	0	0	0	0	0.038834
BSPRY	1.363679	1.436291	2.093365	2.476682	1.404365	1.631281	2.343434	1.903462
BST-2A	1.893385	1.195421	1.436218	2.052765	1.26242	0.828835	1.169782	0.83493
BST-2B	1.137331	1.349787	1.080372	1.165503	1.689967	0.981515	1.978955	1.417183
BST1	18.40797	21.80877	26.80847	21.776	22.84945	19.33128	20.86645	18.75929
BTAF1	12.73177	10.87673	10.51488	10.88769	12.94839	11.3734	11.73449	12.40303
BTBD1	21.63336	21.79756	24.6063	22.61036	22.11734	24.73856	22.4976	19.13338
BTBD10	5.430108	5.989911	5.928369	5.614745	5.619525	5.368695	5.894602	4.869485
BTBD11	2.967916	2.974031	2.58343	3.24546	3.0285	2.410133	2.467283	3.259057
BTBD16	0	0.017902	0	0.018108	0	0	0	0.035843
BTBD17	0.011765	0.035759	0.034856	0.072339	0.011767	0.011845	0	0.011933
BTBD18	0	0	0.012656	0	0	0	0	0.025996
BTBD19	1.419379	1.400165	2.299269	2.42423	2.029726	1.980649	0.960447	1.414329
BTBD2	29.13253	31.66613	25.56652	28.39552	27.67773	27.58129	29.2261	28.36394
BTBD3	23.21982	18.39581	23.67401	18.57125	22.70715	19.79174	16.75281	21.209
BTBD6	17.1629	13.7896	15.15312	16.49606	18.07453	16.75087	16.84351	15.59157
BTBD7	7.63143	7.164707	6.940802	6.817023	7.483188	6.866697	7.692264	8.112096
BTBD9	15.31662	14.69711	12.15002	16.03777	13.35315	12.77111	14.50672	13.98845
BTC	3.185147	3.421751	4.350806	4.043645	3.687221	3.855021	3.360205	3.417027
BTD	6.867978	7.700506	7.151319	9.423397	6.969456	8.448326	9.056463	9.938577
BTF3	346.0013	374.4772	359.4573	363.3569	347.3599	381.3878	359.4179	343.8808
BTF3L4	36.02783	36.94488	39.16327	33.97462	32.25626	39.58496	35.7276	33.13123
BTG1	213.2276	224.6786	200.9776	195.4887	181.9834	200.3191	213.201	203.4137
BTG2	18.35168	19.4964	17.92836	20.18829	18.31255	20.0229	21.41731	21.30649
BTG3	34.25996	39.97801	40.41413	42.25122	39.39968	41.44561	40.87259	33.39588
BTG4	0	0	0	0	0	0	0	0
BTK	1.174879	1.86445	1.232114	1.981775	1.705355	1.21431	1.203056	3.62754
BTLA	0.121165	0.122755	0.049856	0.082778	0.121181	0.132159	0.040994	0.133131
BTN1A1	0	0	0	0	0	0.010772	0	0
BTNL2	0	0	0.00818	0	0	0	0	0
BTNL9	2.502769	2.633452	1.502024	1.22053	1.666045	2.511764	2.27076	2.30986
BTRC	7.709132	7.474472	6.677179	7.407968	7.421105	7.090021	7.540075	7.198194
BUB1	8.578646	9.097985	7.355134	7.212599	6.072205	7.647309	9.056617	9.249241

BUB1B	7.659477	9.961853	8.122958	7.130017	6.483095	8.684326	7.399055	8.235391
BUB3	31.6438	33.91541	28.05121	32.10284	30.23141	30.92263	33.9179	33.9928
BUD13	7.836478	8.63185	7.714725	8.468395	8.460095	7.631928	8.238982	7.650912
BUD23	21.55657	24.74975	18.91186	23.65045	21.21558	23.13757	23.74893	24.22907
BUD31	65.78741	70.84774	76.39268	70.80434	71.0553	79.72193	70.91358	71.66565
BVES	0.077318	0.137082	0.209976	0.019808	0.057996	0.11677	0.078478	0.137233
BYSL	13.57683	14.24507	12.2786	14.20604	15.6786	14.93139	14.51693	13.02307
BZW1	98.7819	102.4001	106.8592	109.2616	113.0692	111.4858	110.2053	99.28369
BZW2	65.79856	64.25849	72.24469	61.35032	60.95639	68.00178	73.17147	70.3903
C10H13orf	0	0	0	0	0	0	0	0.018837
C11H17orf	0.486567	0.845061	0.686434	0.688569	0.301247	0.139969	0.44683	0.657991
C11H17orf	0.14796	0.074951	0.024353	0.176896	0.147979	0	0.02503	0.075033
C11H17orf	0.14757	0.205572	0.151804	0.201634	0.221384	0.167151	0.18723	0.137198
C11H17orf	0	0	0	0	0	0	0	0
C11H17orf	39.66192	47.56157	50.16107	47.45644	43.80234	41.72617	44.5182	40.22653
C11H17orf	0	0	0	0	0	0	0	0
C11H17orf	0.542062	0.464687	0.463247	0.373887	0.625537	0.356846	0.33858	0.496915
C11H17orf	2.408302	2.395276	2.798846	2.467956	5.140333	2.350829	2.623287	3.083019
C11H17orf	0.032283	0	0	0	0	0	0	0
C11H17orf	14.11759	17.14044	7.400679	0.038786	0.094634	7.869164	0.019208	0.095969
C11H17orf	6.54796	8.07779	6.771315	7.107734	7.483219	7.334072	6.654216	6.994514
C11H17orf	5.310557	4.611645	4.395295	5.136307	5.427585	4.924182	4.604784	4.86293
C11H17orf	0	0	0	0	0	0	0	0.128306
C12H1orf1	0	0	0.014998	0	0	0	0	0
C12H1orf1	0.206019	0.268358	0.37784	0.090481	0.323786	0.148162	0.746819	0.447754
C12H1orf1	3.413903	3.205232	3.514828	3.184184	3.083403	3.518484	2.867114	4.002751
C12H1orf1	15.49471	18.48771	15.65251	20.23752	23.44247	19.74415	17.70053	16.64619
C12H1orf1	20.59885	17.30121	21.23259	19.65602	20.16991	20.98786	18.80376	21.22036
C12H1orf1	0.054989	0.139276	0.054303	0.028175	0.054996	0.055364	0.027907	0
C12H1orf1	0.863441	0.95561	0.757002	1.217734	0.706802	0.74023	0.749128	1.101169
C12H1orf1	0	0.012984	0	0.013133	0	0.006452	0	0.006499
C12H1orf1	17.40556	16.64356	15.02698	17.59714	16.43008	16.64718	18.24906	16.14451
C12H1orf2	29.27413	27.73896	30.82907	31.58616	32.63902	27.8984	33.00728	28.50457
C12H1orf5	3.868068	4.315119	3.734031	6.190781	4.694442	4.463351	3.617295	4.319853
C12H1orf7	3.005079	2.902912	2.846859	2.739337	2.114311	2.673793	2.748686	2.941537
C13H10orf	0	0	0	0	0	0.022016	0.022195	0
C13H10orf	0.042819	0.101222	0.035238	0.153578	0.021412	0.043111	0.108652	0.07238
C13H20orf	3.844285	4.290812	3.864752	4.657301	4.451638	4.063255	4.311158	4.832461
C13H20orf	0.257118	0.390739	0.399009	0.526974	0.514303	0.51775	0.242334	0.335287
C13H20orf	0.07131	0.036123	0	0.109615	0.03566	0	0	0
C13H20orf	10.77491	12.43608	11.86912	13.9663	13.83173	12.15624	12.96045	12.98779
C13H20orf	0.269868	0.214822	0.184016	0.164615	0.179935	0.155264	0.195654	0.175956
C14H16orf	0.558387	0.592234	0.465269	0.339755	0.55846	0.377729	0.416217	0.407054
C14H16orf	13.43519	12.61725	14.92505	13.29531	11.75854	13.70483	12.43143	11.21768
C14H16orf	3.603083	4.576188	2.862021	3.398016	3.472985	2.970506	3.816135	5.031386
C14H16orf	2.189156	3.245688	2.646984	2.429419	2.24284	3.010494	1.961858	2.22032
C14H16orf	0.058069	0.029415	0.057345	0.148768	0.058076	0.058466	0	0.029448
C14H19orf	3.057165	3.188889	2.801431	3.636703	2.786279	3.885967	2.735937	3.57066
C14H19orf	0.117932	0.219046	0.038821	0.100711	0.294868	0.158317	0.01995	0
C14H19orf	3.98926	6.192797	7.942648	6.066177	3.603669	8.097808	5.747108	2.28406
C14H19orf	2.012583	2.003675	1.79345	1.643452	2.158655	2.016759	1.820801	1.497976
C14H19orf	4.454779	4.219764	3.958571	3.369688	4.760731	4.106846	4.346776	4.732591
C14H19orf	1.896899	1.80706	1.632814	2.054136	1.512052	1.727423	1.425335	1.418519
C14H19orf	0.051641	0	0.101994	0	0.051647	0	0.10483	0
C14H19orf	0.044783	0.04537	0	0.22946	0.089577	0	0.045454	0
C14HXorf5	0.017942	0.018177	0	0	0.017944	0	0	0
C15H11orf	0.228297	0.393198	0.383268	0.233952	0.319657	0.551655	0.185377	0.289433
C15H11orf	0.015715	0	0.062078	0.016105	0.047152	0.031646	0.031902	0.047817

C15H11orf	7.420801	9.872373	5.83306	7.589254	7.256833	7.803564	8.064687	6.386071
C15H11orf	2.140208	1.951466	3.804373	3.996537	2.045352	3.351956	2.992962	2.26715
C15H11orf	0	0	0	0	0	0	0	0
C15H11orf	38.68101	43.20201	46.60225	39.40267	39.83034	43.14666	49.06921	44.01981
C15H11orf	0.162492	0.041156	0.040117	0.104073	0.040628	0.061351	0.041232	0.061802
C15H11orf	7.629445	4.37273	6.630255	6.612157	4.578259	7.110935	5.221592	3.227874
C15H11orf	20.92929	15.82688	30.90386	21.26813	19.63	23.89534	22.76786	22.7761
C15H11orf	0.015278	0.041276	0.025146	0.031313	0.03056	0.041019	0.072366	0.051651
C15H11orf	0	0	0	0	0	0	0	0
C15H11orf	0	0	0.039713	0	0.04022	0	0	0
C15H11orf	1.063916	0.66331	1.616399	0.251601	2.209957	1.153583	0.581471	0.166009
C15H11orf	13.70415	22.53698	18.4836	18.32062	31.88724	19.84106	18.62635	18.9178
C15H11orf	0.044747	0	0	0	0	0.045053	0	0
C16H5orf2	7.989509	8.173034	7.480975	7.860269	7.015454	7.540248	7.040949	8.006986
C16H5orf3	3.093539	3.722826	2.747878	2.85986	2.701058	2.842757	2.799367	3.120977
C16H5orf4	0.276816	0.31161	0.394863	0.724941	0.645987	0.433544	0.312187	0.218366
C16H5orf5	16.90981	17.35331	17.30287	16.19788	16.8772	17.12042	17.41571	16.53045
C16H5orf5	0	0	0	0	0	0	0	0
C17H12orf	7.135771	6.816782	6.435487	7.429393	8.000266	6.052711	7.077446	7.749586
C17H12orf	14.91076	15.61696	15.3583	16.17203	16.72237	14.09019	14.06501	13.84535
C17H12orf	4.481411	4.08222	5.201988	4.149289	5.425567	5.006766	4.109729	5.14326
C17H22orf	0.4088	0.303721	0.188396	0.293248	0.149913	0.205796	0.193635	0.082924
C17H22orf	0	0.085669	0.055671	0	0	0	0.042914	0
C17H22orf	13.61073	13.88658	12.73039	14.5543	13.9804	14.83093	14.49671	13.02586
C17H4orf3	3.542197	3.235699	3.125317	2.886099	5.081677	3.18637	3.65427	3.504279
C17H4orf4	0	0	0	0	0	0	0	0
C17H4orf4	5.503232	6.414079	6.422781	6.282737	6.941337	6.117806	6.518283	6.993112
C17H4orf5	0	0.187048	0	0.113519	0.110789	0.037177	0.037479	0.074901
C17H5orf5	0.302437	0.584957	0.352972	0.42263	0.494961	0.52596	0.25116	0.474056
C18H14orf	1.454861	1.285426	1.110963	0.953482	1.277398	1.584028	1.090342	1.372625
C18H14orf	3.109907	3.754051	3.308028	3.212368	2.787697	2.714776	3.190136	2.751518
C18H15orf	3.696443	4.565767	3.425361	5.299316	5.32382	4.12318	4.432884	3.64635
C18H15orf	9.933409	14.12265	13.8744	13.61011	11.80088	12.98507	11.58642	12.52394
C19H3orf1	0.817334	0.588843	0.735404	1.116773	0.835605	0.62176	1.161432	0.534225
C19H3orf1	0.588008	0.489818	0.593585	0.669525	0.44433	0.605181	0.636616	0.503608
C19H3orf2	0.026631	0.044967	0.026299	0.063678	0.079903	0.008938	0.045051	0.05402
C19H3orf2	0.224512	0.417007	0.369524	0.268419	0.598776	0.489766	0.22788	0.265659
C19H3orf3	0.067607	0.034247	0	0.034641	0	0	0.03431	0.034285
C19H3orf4	0.010828	0.01097	0.010693	0	0	0	0	0
C19H3orf6	1.941184	1.783715	1.382021	1.711696	2.302632	1.647643	1.42045	1.980265
C19H3orf6	0.877474	0.607856	0.940604	0.676328	0.555055	0.44551	0.746003	0.8139
C19H3orf8	0.03348	0	0	0	0.033484	0	0.101946	0.033956
C19H3orf8	0	0.031507	0.030712	0	0.031103	0.031312	0	0.126167
C1D	9.435244	10.6464	9.668427	10.94286	9.822571	9.390867	9.600283	8.896089
C1GALT1	4.877176	4.825951	3.872893	4.755545	5.082565	3.591257	5.379793	5.592617
C1GALT1C	14.52393	15.75361	15.60083	17.05352	15.07176	14.85632	16.55513	15.43534
C1H1orf10	5.075166	4.52783	5.011929	4.676911	4.034143	4.614107	4.613099	5.550757
C1H1orf12	8.501095	8.239551	7.970865	9.403572	7.182359	8.095677	7.289152	6.910141
C1H1orf14	0	0	0	0	0	0	0	0
C1H1orf14	0.057769	0	0.114099	0.0592	0.144442	0.232655	0	0.117183
C1H1orf16	0.406076	0.472354	0.78718	0.508609	0.496379	0.393708	0.366371	1.998268
C1H1orf18	0.029065	0.058892	0.028702	0.029784	0.116273	0.058526	0	0.029478
C1H1orf18	2.058447	1.795815	1.101099	1.816459	1.772781	1.381673	1.392883	1.217854
C1H1orf19	1.242385	0.827139	0.280435	0.400136	0.337262	0.92922	0.522423	0.612035
C1H1orf21	1.78121	1.722972	1.714819	2.192232	2.067903	1.856462	1.707992	1.516064
C1H1orf21	2.798916	4.149414	3.588182	3.868186	3.107456	3.645351	3.700989	3.346613
C1H1orf43	31.54952	30.38737	29.76774	30.00085	29.32435	30.75592	30.8536	29.81382
C1H1orf50	5.012502	5.864601	5.077789	5.476346	5.191036	5.404901	5.038454	5.469247

C1H1orf52	5.846027	5.771274	5.374525	5.393284	5.547715	4.636514	5.357039	6.657137
C1H1orf54	15.77207	17.39942	14.73496	13.39199	12.41898	15.3757	16.56768	12.08632
C1H1orf56	0.041816	0.042365	0.013765	0.057136	0.055763	0.014034	0.028296	0.070686
C1H1orf68	6.176145	6.285642	10.64586	6.530511	5.138093	3.957124	3.704283	4.98278
C1H1orf87	0.013134	0	0	0.01346	0.013136	0.013224	0	0
C1H1orf94	0	0	0	0	0	0	0.013128	0
C1H21orf5	0.08422	0.298639	0.013862	0.028769	0.014039	0.155458	0.014247	0.185075
C1H21orf6	0.362189	0.570799	0.695481	0.28868	0.583602	0.567254	1.000748	0.571426
C1H21orf9	23.70614	21.50102	20.1722	21.8261	21.43815	22.46469	23.33633	24.18756
C1H3orf33	2.948745	3.455746	3.510144	4.214166	3.252009	2.952841	3.122402	3.006888
C1H3orf38	13.22049	14.06369	13.31344	12.26447	14.30085	16.04302	12.41239	12.43832
C1H3orf52	13.73174	13.45732	19.41033	18.32892	15.5544	21.82914	20.28843	20.07809
C1H3orf70	1.839023	1.769171	1.859221	1.979644	2.286796	1.906529	1.689364	1.948223
C1H3orf80	0	0	0	0	0	0	0	0
C1H3orf85	0.036563	0.148172	0.198592	0.224814	0.27426	0.073626	0.148447	0.426463
C1QA	9.733213	8.759729	10.80733	9.316105	13.02047	4.899853	15.59612	15.65954
C1QBP	71.70624	77.62089	71.29527	67.04497	72.32051	75.87384	77.31594	71.35838
C1QC	6.914537	7.387221	8.63536	6.925628	10.13405	3.823421	11.80597	12.74831
C1QL1	0.015584	0.031577	0.01539	0.01597	0.046759	0.078453	0.031636	0.015806
C1QL2	0.077158	0	0	0	0.019292	0	0	0
C1QL3	0.376242	0.207163	0.565409	0.268217	0.245407	0.189406	0.381886	0.20739
C1QL4	0	0	0	0	0	0	0	0
C1QTNF1	8.462252	8.761636	8.619054	9.288692	6.515714	7.887296	11.63493	10.39622
C1QTNF12	14.79148	16.51532	14.96614	13.75383	11.38168	12.27437	15.43906	10.66308
C1QTNF2	3.076705	4.058937	4.185954	4.026206	4.30573	2.874866	3.673287	3.232753
C1QTNF3	4.467796	1.956882	1.996599	3.015312	4.531563	2.844391	4.727199	3.387106
C1QTNF4	0.111689	0.067893	0.044119	0.137346	0.111703	0.134942	0.068018	0.113279
C1QTNF5	3.075373	2.270508	3.66708	4.190892	3.615666	3.639896	4.48301	3.832576
C1QTNF6	0.724209	1.34342	2.135484	1.23343	1.581225	1.910186	1.573677	2.400118
C1QTNF7	4.913991	5.930651	6.567582	5.485166	4.073142	4.839418	4.851425	7.071937
C1QTNF9	0.559384	0.647686	0.591872	0.040946	0.879145	0.885037	0.202777	0.283673
C1R	49.98857	58.56097	64.46996	59.96669	70.75234	46.17313	71.36652	71.98777
C1RL	0.45321	0.570469	0.379751	0.914799	0.837861	0.539271	0.557586	1.002899
C1S	53.05117	48.47086	52.78652	60.26428	76.61543	47.3517	58.95439	63.62637
C2	2.311934	5.093205	4.779994	2.510346	4.457199	2.198961	6.420734	7.892653
C20H6orf1	24.49748	23.87052	24.16725	22.86404	23.37709	23.31976	22.74364	23.39373
C20H6orf1	30.45095	24.98526	27.08337	26.20003	26.75954	27.13258	23.85096	24.76432
C20H6orf1	4.253184	3.849968	3.01663	4.193781	3.625175	4.032074	4.806536	3.824545
C20H6orf1	5.602013	5.082568	4.417512	4.198479	5.435492	5.324596	5.686044	4.685333
C20H6orf1	1.287696	1.846126	2.183401	2.440001	1.263563	2.372827	2.811308	3.006327
C20H6orf2	0.014487	0	0	0	0	0	0	0
C20H6orf2	0.198653	0.031778	0.237478	0.150002	0.062741	0.294752	0.127347	0.031813
C20H6orf2	18.64765	18.46219	23.90145	25.41897	19.32245	18.91755	23.41678	19.88209
C20H6orf4	11.02263	11.47465	9.786771	13.47189	12.87825	11.65226	12.76181	11.49863
C20H6orf5	5.800785	4.327127	3.200784	5.540931	5.316811	4.010519	6.026152	6.636064
C20H6orf6	48.33231	42.12138	47.25463	46.7867	48.918	43.54402	45.30532	52.37224
C20H6orf8	14.77751	15.00106	13.57898	15.70839	14.13629	15.58896	13.9057	15.17422
C21H11orf	8.45636	8.2724	8.13195	9.688678	7.29282	9.67261	8.752055	9.856223
C21H11orf	6.51038	7.892816	8.039276	7.254713	7.583658	7.480248	7.30771	7.779403
C21H11orf	11.03102	13.4369	12.21237	13.98505	11.41666	11.67736	14.91008	12.00443
C21H11orf	3.921592	4.191092	4.439469	4.790621	5.285267	4.393779	3.871197	5.396192
C21H11orf	2.738626	2.391512	2.129291	3.204394	3.454385	2.880798	2.572265	3.554929
C21H11orf	21.53919	19.4503	23.00042	22.55595	21.64304	24.65359	20.08458	23.74174
C22H10orf	2.215247	2.01013	2.149599	2.32934	2.48525	2.307959	1.89654	2.442155
C22H10orf	0.224327	0.157342	0.017041	0.017683	0.069033	0.052122	0.157633	0.017502
C22H10orf	0.115658	0.146469	0.085662	0	0.144591	0.029112	0.117393	0.205282
C22H10orf	0.016315	0	0.008056	0.00836	0	0.016427	0.01656	0.008274
C23H18orf	6.155693	6.691223	6.466844	7.211685	6.404992	7.440522	6.874458	7.023738

C23H18orf	7.44798	7.54102	6.088783	7.399162	7.560468	7.353843	7.305037	7.417347
C23H18orf	26.11418	28.99732	38.57165	33.5902	31.38174	32.82024	31.29805	33.88642
C23H18orf	1.210472	1.141082	0.910396	0.977578	1.395029	1.041177	1.167605	1.430966
C23H18orf	0.065518	0	0.01294	0	0.039316	0.039579	0.0133	0
C24H16orf	1.825648	2.774412	2.777441	1.858229	2.479748	1.713923	2.040841	2.327058
C24H16orf	1.120088	1.688986	1.672059	1.481505	1.641271	0.708115	1.969726	3.276001
C24H16orf	5.815187	5.60176	6.345245	7.063155	5.978023	6.555584	4.896102	5.085791
C24H16orf	0.324584	0.472187	0.23835	0.358211	0.258036	0.301663	0.160503	0.464264
C24H16orf	27.88183	21.75671	24.96606	33.56441	32.03068	31.66343	25.73571	23.53073
C24H16orf	0.085594	0.390227	0.718482	0.109642	0.214012	0.086179	1.020813	1.171965
C24H16orf	0	0	0	0	0	0	0	0
C24H16orf	21.3569	21.8879	23.21697	22.64672	22.32492	24.69209	22.75734	21.76132
C24H16orf	0.021759	0	0.014325	0.014865	0.007254	0	0.007362	0.036781
C24H7orf2	10.92097	12.28016	9.283703	11.3828	11.94928	10.49883	9.650128	10.96825
C24H7orf5	5.021865	8.656447	6.581051	7.170616	8.212168	7.045093	5.821913	5.527859
C24H7orf6	0	0	0	0.021996	0	0	0.021786	0.02177
C25H10orf	0	0	0	0	0.071228	0	0	0
C25H10orf	0.096557	0.065216	0.105134	0.083726	0.07676	0.129622	0.082928	0.065288
C25H10orf	0.541422	0.143094	0.092987	0.096493	0.612121	0.213308	0	0.04775
C25H1orf1	24.36215	32.06062	30.3088	24.12969	22.4423	26.09049	22.9608	26.48919
C26H4orf4	6.514215	5.131458	6.18729	1.845159	8.462253	6.042863	3.996873	2.553697
C26H8orf4	0.53764	0.733954	0.643428	0.761068	0.542266	1.206485	0.966544	0.725518
C2CD2	12.57626	13.96012	14.64657	12.94237	15.94767	14.84777	13.94542	13.02993
C2CD2L	11.33334	9.917118	9.086351	13.02497	11.68745	11.63053	9.688373	11.06044
C2CD3	4.801048	4.40572	3.560249	3.66572	4.093168	3.947583	4.349806	4.734232
C2CD4B	0.277303	0.149836	0.657236	0.700959	0.462232	0.390877	0.412812	0.656253
C2CD4C	0.110804	0.089807	0.065654	0.136259	0.199474	0.022312	0.089973	0.269716
C2CD4D	0.048762	0.024701	0.361155	0.074954	0.12192	0.024547	0	0.074184
C2CD5	9.960336	10.52459	8.85761	7.870224	9.955993	9.484161	10.76125	10.73601
C2CD6	0	0	0	0	0	0	0.004702	0
C2H1orf23	0	0	0	0	0	0	0	0
C2H2orf69	2.573996	3.142495	2.582017	2.621113	3.061584	2.550704	2.777439	2.26475
C2H2orf72	0.500383	1.226492	0.781073	0.975932	0.831389	1.178245	1.26153	0.892229
C2H2orf76	7.96793	11.21344	11.35947	9.412362	7.96896	11.31882	7.763951	8.492773
C2H2orf80	0.02594	0	0	0	0	0	0	0
C2H2orf88	0.159142	0.342616	0.275029	0.584386	0.338221	0.280402	0.255755	0.181584
C2H8orf58	12.97941	10.11591	9.897081	11.06826	9.735814	11.82661	9.937034	10.37149
C2H8orf74	0	0	0	0	0	0.031951	0	0
C2H9orf13	0	0	0	0	0	0	0	0
C2H9orf13	0	0.026403	0	0.080119	0	0	0	0
C2H9orf15	4.683614	3.818864	5.194731	7.451676	5.514156	6.230836	6.695388	4.42219
C2H9orf24	0.027082	0.013719	0	0.013876	0	0.013633	0.013744	0.013734
C2H9orf40	7.864724	7.473199	9.111946	8.138551	7.146	5.874146	8.426178	7.298926
C2H9orf43	0.210992	0.302214	0.280212	0.417525	0.283784	0.300337	0.147694	0.140204
C2H9orf64	3.376184	5.008578	4.986289	3.768725	5.260895	3.20197	4.5895	5.27395
C2H9orf85	2.835504	2.347596	2.479006	1.968405	2.32765	2.271621	2.506677	3.143871
C3AR1	1.053728	1.020803	0.81273	1.087711	1.253867	0.449151	0.983659	2.176461
C3H12orf1	13.91288	15.11243	15.53765	12.53512	12.42048	17.22787	14.785	11.64863
C3H12orf2	19.59007	20.39386	19.73984	19.28193	20.2496	21.75534	22.7653	22.60539
C3H12orf4	4.990395	4.216695	3.948631	4.317565	5.226274	3.75807	4.141466	3.98277
C3H12orf4	0.012322	0.006242	0.030421	0.018941	0.012323	0.012406	0.025013	0.006249
C3H12orf4	3.174838	4.63919	3.876032	4.040036	2.294204	3.591702	3.187049	3.86581
C3H12orf5	0	0.075366	0.018366	0.038116	0.0186	0.074898	0.018876	0.018862
C3H12orf5	1.661242	0.893369	2.410275	0.580911	0.677181	1.609175	0.43952	0.726659
C3H12orf5	40.7305	49.06459	45.96913	43.39064	44.13787	42.75125	45.27874	44.1854
C3H12orf6	0.217815	0.088269	0.215101	0.066963	0.108921	0.263163	0.132649	0
C3H12orf6	2.077167	2.044491	1.979879	2.182508	1.834191	2.184012	2.248434	1.726724
C3H12orf7	11.55108	16.13941	16.79735	13.91848	16.37673	14.12214	14.62323	11.00744

C3H12orf7	7.88374	5.732445	6.537591	7.035322	8.978817	7.462862	8.499728	7.517743
C3H22orf2	3.970781	3.06623	3.63438	2.580429	3.390129	2.998431	3.661714	3.315162
C3H2orf40	28.9074	28.87332	10.34416	19.65603	25.57785	21.33217	24.09415	26.24905
C3H2orf42	1.536173	1.62181	1.423751	1.614981	1.481678	1.686818	1.695457	1.527788
C3H2orf49	6.707395	7.25613	6.316954	7.122074	7.13274	6.547187	6.323368	6.856687
C3H2orf68	20.58588	19.0975	19.9333	19.26987	18.97749	17.49606	17.77815	18.32491
C3H2orf73	0.263221	0.355567	0.086647	0.209799	0.117002	0.058893	0.148427	0.059326
C3H2orf74	0.048341	0	0	0	0	0.194683	0.098131	0
C3H2orf78	0	0	0	0	0	0	0	0
C3H2orf81	0.147635	0.074786	0.024299	0.050431	0.098436	0	0.012487	0.199649
C3H2orf92	0.054881	0.055601	0	0	0	0.055256	0	0
C3H9orf11	17.95472	19.11746	20.69136	22.63554	23.06959	20.42839	18.22404	18.01829
C3H9orf16	110.7171	116.5047	111.4698	114.1972	106.3326	109.5009	117.1263	113.956
C3H9orf50	0.097387	0.039466	0	0.07984	0.01948	0.039221	0	0.019755
C3H9orf78	46.44565	49.36844	47.0695	42.50699	43.1595	48.50626	47.10371	46.29631
C4BPA	0.011011	0.100397	0.021747	0.270802	0.187206	0.133031	0.022352	0.212181
C4BPB	0.04529	0	0.044726	0.023206	0	0	0.045969	0.068902
C4H7orf25	5.135506	6.706557	5.694107	5.585745	5.582793	6.373975	5.879104	5.901319
C4H7orf31	0.516356	0.576788	0.418398	0.556282	0.609114	0.319929	0.53754	0.322281
C4H7orf57	9.036502	12.01775	11.27992	10.21642	10.18404	9.111654	9.185577	8.367585
C5AR1	1.547614	1.682651	2.944832	1.982436	2.293407	1.263651	2.557385	4.574942
C5AR2	6.376202	8.345212	8.190444	6.805188	7.907512	7.475455	6.826598	12.8573
C5H19orf2	11.56453	12.37282	13.93097	13.02587	16.60124	15.65855	16.51921	11.14776
C5H19orf2	10.74821	10.82587	8.31221	9.940262	10.23325	9.151927	9.81373	9.647661
C5H19orf3	0.253384	0.256709	0.192482	0.35953	0.350884	0.156994	0.356102	0.751204
C5H19orf4	3.293774	2.574946	1.907853	1.858072	3.048719	2.694474	2.322546	4.248103
C5H19orf5	66.14297	61.72922	68.45389	56.97693	59.80144	64.19026	63.32163	61.14709
C5H19orf5	0.901346	0.36527	0.637135	1.147299	0.370074	0.897951	0.635591	0.211704
C5H19orf6	6.790112	9.858418	7.339098	8.930739	9.143774	8.101521	8.655656	7.917079
C5H19orf6	0.290746	0.333837	0.24884	0.417127	0.193856	0.507403	0.452498	0.235908
C5H19orf7	0.424417	0.475248	0.397069	0.366257	0.446812	0.483542	0.498801	0.521081
C5H1orf35	11.92682	12.7202	10.56935	12.15445	11.34932	12.09158	11.87066	12.26439
C5H5orf15	21.53703	25.31551	25.76563	24.18686	26.19673	24.11217	24.03438	27.88022
C5H5orf24	17.61713	18.67321	16.8208	17.15869	18.85854	17.92162	17.85144	18.11326
C5H5orf30	3.370718	3.595691	3.634701	3.357994	3.76555	3.573358	3.96449	4.066256
C5H5orf46	3.475578	5.328528	4.677609	5.957128	6.521395	6.007684	3.652586	4.648081
C5H5orf63	5.388627	7.899997	5.446696	10.39455	7.862073	5.680755	5.115551	6.333362
C6	0.202978	0.323151	0.229084	0.527442	1.870536	0.992626	0.110369	0.264686
C6H4orf17	0.01568	0.047658	0	0	0.047047	0.031575	0.047746	0
C6H4orf19	0.044273	0.089707	0.026233	0.018148	0.044278	0.07132	0.071899	0.215534
C6H4orf3	76.79203	92.38834	105.683	82.92685	101.0364	112.6563	96.39003	84.65356
C6H4orf36	0.121751	0.164465	0.360701	0.457478	0.581774	0.190684	0.466847	0.274409
C6H4orf48	0.124396	0.378087	0.491385	0.318694	0.311031	0.438361	0.252524	0.315418
C6H4orf50	0.005073	0	0	0	0	0.005108	0	0
C6H4orf54	0.047516	0.020269	0.022227	0.02819	0.017508	0.025179	0.017768	0.007609
C7	2.647624	5.221983	4.716594	3.670808	6.104414	5.704771	4.396094	4.18217
C7H14orf1	18.4196	19.24451	18.87222	17.5929	17.99022	18.27015	17.77571	15.23736
C7H14orf9	2.437512	2.418172	1.901251	2.595959	1.857928	2.340807	1.839843	2.318055
C7H15orf4	2.80517	3.113512	3.184867	2.416907	3.019968	2.536503	2.892586	2.736373
C7H15orf4	1.627277	2.596598	2.209626	2.959964	4.272154	1.802233	2.1059	4.497455
C7H15orf6	0.575726	0.897357	1.312045	0.589987	0.841555	0.936373	0.674264	0.718674
C7H15orf6	21.88738	17.39432	20.15091	10.82238	15.76293	23.96451	19.21933	21.00883
C7H15orf6	1.139679	0.509398	1.324092	1.064858	1.508593	1.754945	0.884592	1.053911
C8A	0	0	0	0	0	0	0	0
C8B	0.012352	0	0	0	0	0	0	0
C8G	0.477088	0.821694	0.596782	0.749655	0.874774	0.480347	0.484244	0.758078
C8H6orf11	0.112615	0.070211	0.025664	0	0.121293	0.008722	0.043963	0.052716
C8H6orf12	24.86494	27.47545	26.85584	26.58107	27.13378	25.932	25.98982	23.54201

C8H6orf16	0	0.020026	0	0.020256	0	0.019901	0	0
C8H6orf20	0.803459	1.214931	1.350046	0.921673	1.151374	1.593749	0.925057	0.814897
C8H6orf58	0	0.608136	0.643588	0.896327	0	0	0	0
C9	0	0.11175	0	0.014129	0.01379	0.013882	0.685735	0.055936
C9H8orf33	13.79155	12.10661	14.73678	14.87624	13.38722	15.72552	11.24584	12.66411
C9H8orf34	0.106504	0.01079	0.063106	0.032743	0	0.085785	0.054051	0.032406
C9H8orf37	4.806606	5.760367	5.067567	5.669904	5.304197	5.637999	5.80983	5.146405
C9H8orf59	71.61244	78.60325	109.2317	86.76746	89.36608	115.8461	59.31419	83.85628
C9H8orf76	3.495653	4.669482	3.865875	4.183371	3.96305	3.194097	3.487333	3.885387
C9H8orf82	2.599672	2.287744	2.754672	3.111318	2.846724	2.235325	2.099377	2.001567
C9H8orf88	0.122759	0.124371	0.24246	0.276761	0.122775	0.086519	0.137061	0.087155
C9H8orf89	0.6917	0.713292	0.500113	0.73415	0.80297	0.870532	0.626854	0.801768
C9orf72	16.79699	15.55147	14.01389	12.89862	14.40177	17.37687	12.2625	14.3662
CA1	0	0.013376	0.039114	0	0.026408	0	0	0.200856
CA11	0.759314	1.32487	1.228926	1.491402	1.729773	2.081143	1.348732	1.497462
CA12	12.55683	18.19676	11.16604	16.02931	13.06426	12.5917	13.57384	16.94903
CA13	19.42227	13.91111	14.62102	11.69527	18.7481	21.43832	15.43155	18.62905
CA14	0	0	0	0	0	0	0	0
CA2	43.42581	39.09809	51.78532	38.95979	51.65881	45.63637	33.5652	41.1022
CA3	0.05311	0.013452	0.039336	0.299343	0.039838	0.026737	0.026954	0.053866
CA4	0.513374	0.915397	1.541212	3.998289	3.840536	1.798747	2.251043	1.603702
CA5A	0.026036	0.026378	0.025712	0.320176	0.05208	23.17344	0.026427	18.14166
CA6	0.550295	0.422944	0.76831	0.408361	0.683214	0.496739	0.693372	0.365671
CA7	0	0	0	0	0	0	0	0
CA8	0.074563	0.245511	0.101247	0.028654	0.05593	0.04692	0.122983	0.094531
CA9	0.26762	0.171938	0.19338	0.274249	0.339465	0.111723	0.192132	0.086063
CAAP1	11.75744	12.07543	11.96688	11.3227	12.93983	13.06408	11.97164	11.86178
CAB39	50.7626	52.78297	48.20035	48.49146	49.56314	49.5369	52.10652	52.65669
CAB39L	2.74355	3.96003	4.627138	3.234928	2.851346	3.070138	4.311257	3.796748
CABCOCO	4.073442	4.126902	3.26584	3.991446	4.084468	4.524081	3.868142	4.02495
CABIN1	5.148481	4.976665	4.650402	5.501154	5.219626	4.753769	4.910146	5.764131
CABLES1	6.935988	5.864719	5.751213	6.83856	5.798255	4.452781	5.742245	8.00289
CABLES2	3.839877	3.690343	2.777033	3.471556	3.955502	2.905791	3.229813	3.669374
CABP1	0.065359	0.066217	0.087325	0.055159	0.038452	0.073548	0.081949	0.097485
CABP2	0	0.031437	0	0	0	0	0	0
CABP5	0.108726	0.015736	0.076694	0.095502	0.031069	0	0.141887	0.141781
CABP7	0.020122	0	0	0.02062	0.010062	0	0	0
CABYR	0.902579	0.657826	0.736714	1.019318	0.755317	0.769652	0.682414	1.060215
CACFD1	3.784488	3.590287	2.799698	3.891935	3.249998	3.460275	3.420482	2.970323
CACHD1	10.27496	10.20498	7.438697	9.998222	9.540048	9.645736	9.455636	9.411709
CACNA1A	0.163867	0.270186	0.212594	0.214024	0.179956	0.249098	0.202199	0.228118
CACNA1B	0.005889	0.023867	0.008724	0.018106	0.020615	0.008894	0.008967	0.011946
CACNA1C	0.652019	0.63863	0.410724	0.774722	0.686767	0.573596	0.674991	0.516298
CACNA1D	0.26976	0.295546	0.254008	0.192867	0.291755	0.176858	0.299277	0.302233
CACNA1E	0.087163	0.085854	0.035866	0.069473	0.193722	0.085321	0.073726	0.117872
CACNA1F	0.009388	0.014266	0.018541	0.01443	0.023472	0.051985	0.004764	0.014282
CACNA1G	0.436421	0.965359	0.420209	0.588855	0.654717	0.790925	0.627538	0.623377
CACNA1H	1.258322	1.139313	0.967247	1.308078	1.630361	1.282898	1.608578	1.379714
CACNA1I	0	0	0.003584	0.003719	0.00363	0.007308	0.003684	0.003681
CACNA1S	0.052024	0.047916	0.116764	0.465278	0.212855	0.247614	0.134412	0.187076
CACNA2D	1.608518	1.760192	1.769948	1.73885	2.00494	1.636326	2.151023	2.398714
CACNA2D	0.474746	0.327699	0.401855	0.486506	0.412196	0.267884	0.185334	0.306893
CACNA2D	1.118628	1.069907	0.950188	1.13832	1.24395	1.055386	1.016309	0.66645
CACNA2D	0	0.0085	0	0	0	0.008447	0	0
CACNB1	1.864499	1.508246	1.7699	1.762568	2.002066	1.979102	1.606396	1.634505
CACNB2	0.472958	0.453946	0.226158	0.448961	0.219083	0.681702	0.328457	0.24742
CACNB3	4.694481	3.842641	3.814095	4.797439	5.024568	4.338128	4.307327	4.057885
CACNB4	0.463205	0.539677	0.434562	0.427211	0.602244	0.582962	0.55243	0.751678

CACNG1	0.021815	0	0.021544	0	0	0.043929	0	0
CACNG3	0.013284	0.013458	0	0	0	0	0	0
CACNG4	0.383413	0.332953	0.477802	0.748401	0.538674	0.51471	0.379899	0.351836
CACNG5	0.47339	0.269776	0.175309	0.212238	0.532632	0.208523	0.510521	0.510137
CACNG6	0	0.01165	0.011356	0	0.023001	0.011578	0	0.034989
CACNG7	0.094736	0.309266	0.093555	0.107869	0.094748	0.084785	0.224366	0.10676
CACTIN	13.2733	12.11572	10.34303	10.54052	11.69567	10.44195	10.77792	13.47091
CACUL1	8.429137	8.769732	8.868115	8.433683	7.635654	9.044141	8.687631	9.467215
CACYBP	34.4715	36.54019	38.36952	32.40746	38.97985	41.31495	31.85252	36.23209
CAD	6.097525	5.550841	4.613347	5.186554	5.242621	5.289886	5.133027	5.805445
CADM1	15.40434	13.99564	15.14247	14.86897	14.37624	14.37536	13.55112	15.47347
CADM2	0.022785	0.003298	0.032144	0.03002	0.029298	0.013109	0.009911	0.009904
CADM3	2.18344	4.44022	3.859346	4.491262	4.93711	3.727647	4.079077	4.429045
CADM4	5.33753	4.888132	3.466412	4.553628	2.458737	5.043082	5.951346	4.640154
CADPS	0.037544	0.071319	0.041711	0.043283	0.126728	0.075601	0.104795	0.142795
CAGE1	0.675664	0.825189	0.658105	0.834675	0.879403	0.493902	0.648221	0.901194
CALB1	0.010959	0.044411	0.064934	0	0.043841	0.022068	0.011123	0.055575
CALB2	0	0.097714	0	0	0.019292	0.058264	0	0.019564
CALCA	0.035474	0.251579	0.175161	0.072706	0	0.178583	0.360063	1.079377
CALCOCO	51.12429	47.38236	50.47816	45.12784	43.39666	46.14037	46.79571	44.23338
CALCOCO	36.05572	36.94427	41.0667	43.79583	43.99499	35.17526	35.0895	39.41225
CALCR	0.007909	0.040063	0.062482	0.064837	0.087008	0.015926	0.016055	0.008021
CALCRL	3.764966	5.28789	4.126367	4.605943	6.270889	5.754595	4.952073	4.903942
CALD1	173.3315	160.9592	181.1727	167.4022	168.6496	162.4336	167.2456	170.924
CALHM1	0.027043	0	0.026706	0	0	0.027227	0	0
CALHM2	10.77528	7.551125	8.32672	8.219748	10.90957	10.03401	7.577369	8.588569
CALHM3	0.041312	0.125564	0.020399	0.063504	0.041318	0	0	0.041901
CALHM4	6.762307	9.086246	12.84042	8.842868	17.83495	13.84045	8.122537	11.92968
CALHM5	19.44338	17.63008	16.16399	15.60806	16.09434	13.36595	18.44093	15.46509
CALM1	149.2153	144.6527	147.8719	144.5311	157.8554	151.0641	149.6588	145.8983
CALM2	478.8307	544.8823	566.4952	529.4267	484.0681	525.641	553.4731	503.6747
CALM3	71.81666	75.54813	70.04936	65.05383	71.2625	64.1342	71.20445	65.60576
CALML4	2.825129	5.517861	4.156129	4.820206	4.689738	4.369277	4.064764	4.135552
CALML6	0.213658	0.432924	0.421991	0.372215	0.406002	0.193606	0.563843	0.325049
CALN1	0.067346	0.15693	0.013301	0.138029	0.212169	0.074587	0.143549	0.177593
CALR	179.3864	193.1041	166.8442	178.9039	209.1373	189.4627	192.2979	180.1957
CALR3	0.071401	0	0.052883	0.018292	0.017853	0.143777	0.018118	0.018104
CALU	17.9757	16.29209	15.51729	16.49543	20.67159	15.30017	18.69034	19.39092
CALY	0.176774	0.029849	0.145476	0.090576	0.029466	0.148318	0.119617	0.029882
CAMK1	2.215247	3.161564	3.04368	2.131938	3.352198	2.98677	2.600411	5.275055
CAMK1D	8.471277	8.64957	7.781022	7.463381	7.569646	8.283196	8.434448	9.146185
CAMK1G	0.183736	0.124098	0.050402	0.387035	0.132716	0.215823	0.217574	0.103529
CAMK2A	0.081415	0.029458	0.034457	0.017878	0.116322	0.03513	0.035415	0.023592
CAMK2B	0.589393	0.454955	0.512758	0.625564	0.673679	0.59342	0.534138	0.36294
CAMK2D	12.03693	11.87328	12.63804	12.49097	13.8641	12.47208	12.3853	13.44253
CAMK2G	8.518192	9.34383	7.987774	9.521686	8.885961	9.447289	8.884354	8.697554
CAMK2N1	18.29792	17.97093	15.65138	14.62806	13.89142	22.03353	14.32879	12.96959
CAMK2N2	0.05286	0.16066	0	0	0.026433	0	0.080479	0
CAMK4	0.471457	0.740837	0.437078	0.492995	0.75058	0.329368	0.59572	0.741649
CAMKK1	3.049759	2.186022	3.162347	4.539502	3.599181	3.1013	3.188371	3.301964
CAMKK2	8.476688	8.495534	7.581643	8.752633	9.486532	7.238202	7.645436	8.472207
CAMKMT	3.87489	3.820591	5.398252	4.78632	4.325214	5.834626	4.477315	3.701969
CAMKV	0.009023	0.018283	0	0.009246	0.009024	0	0	0.018303
CAMLG	14.60304	14.81458	15.19641	15.54807	15.21347	15.05852	14.24435	14.7512
CAMSAP1	9.725198	7.949291	6.86437	8.433892	8.667794	7.731174	7.538727	7.792501
CAMSAP2	21.07117	18.41484	17.77535	19.15319	20.53179	19.23429	19.24362	21.80279
CAMSAP3	26.32984	21.00687	20.4967	23.3774	22.50669	22.85772	19.93919	20.70306
CAMTA1	1.604401	2.186627	1.716444	1.961228	1.963736	1.688438	2.233327	1.944941

CAMTA2	10.75888	10.14791	9.341776	12.8323	11.62035	9.941617	9.702013	10.52927
CAND1	14.62139	14.3797	13.43844	12.97486	13.71273	13.84324	14.25265	14.31972
CAND2	0.908022	0.839133	0.799766	0.861354	0.809962	0.673314	0.778414	0.765383
CANT1	6.809735	7.030656	6.632275	6.246568	6.356093	5.832175	5.520718	7.250546
CANX	116.6299	120.0136	117.0078	121.376	114.8073	120.3917	129.6374	124.1788
CAP1	108.4408	97.28376	110.9183	106.6857	112.651	96.08064	110.0242	98.67253
CAP2	0.990493	1.411161	1.283824	1.141906	1.663934	1.760786	1.201708	1.687403
CAPG	51.36513	35.3394	49.33859	52.39455	46.4662	47.38342	56.83651	46.56677
CAPN1	42.29376	39.2202	36.23437	39.23002	37.04648	35.49537	37.57511	38.67137
CAPN10	5.23084	4.985624	5.224497	5.287147	4.945511	5.47052	4.934386	5.522834
CAPN11	0	0.011072	0.021586	0.067198	0.01093	0.044015	0.022186	0.066507
CAPN13	0.024233	0	0.023931	0	0	0.012199	0	0.012289
CAPN14	0.027082	0.013719	0.026744	0.124887	0.040628	0.204502	0.426066	0.068669
CAPN15	1.837559	2.040059	1.195022	1.417228	1.924244	1.485892	1.605175	1.915668
CAPN2	67.15802	61.43233	53.41448	68.15325	62.3881	60.09937	58.6552	57.62563
CAPN3	0.231463	0.135289	0	0.045615	0.071228	0.107559	0.072288	0.036117
CAPN5	3.046689	4.462661	3.54039	4.664427	4.491082	2.784159	3.464967	3.431333
CAPN6	0.30207	1.022799	0.408208	0.578374	0.842725	0.856376	0.476038	0.411181
CAPN7	11.24364	12.33982	10.42342	11.9583	12.80068	11.65547	12.65328	11.96879
CAPN8	4.447982	5.716842	3.469655	3.891408	6.033947	5.228734	5.559327	5.639128
CAPNS1	121.4993	136.7058	136.1911	132.3035	130.6396	128.4398	141.0521	132.543
CAPNS2	40.9054	45.27813	44.08741	43.49028	42.15694	44.36961	43.92693	36.5296
CAPRIN1	84.78424	83.28242	81.29454	77.64944	87.91848	80.41565	89.37898	90.85705
CAPRIN2	3.929206	2.745617	3.118703	3.431961	3.503689	2.891243	3.257605	3.292396
CAPS	0.308421	0.680734	0.739688	1.32068	1.090628	0.687597	0.838518	0.73734
CAPS2	0.01831	0.024733	0.012054	0.006254	0	0.024579	0.018584	0.00619
CAPSL	0.299527	1.11268	0.410827	0.630946	0.282923	0.619901	0.658711	0.421932
CAPZA1	41.23973	43.38082	41.03577	42.6347	47.59279	43.68213	43.31731	45.0095
CAPZA2	33.22784	38.08051	37.65701	33.50518	39.14035	39.92635	38.71669	36.90389
CAPZA3	0.031099	0.063014	0	0	0	0.031312	0	0
CAPZB	80.10525	80.74724	71.07991	79.20799	79.42063	83.58117	77.81311	75.37795
CARD10	2.52529	2.843422	2.790558	2.817145	2.979588	2.439553	3.056336	2.85951
CARD11	1.344094	1.35525	0.739522	1.108469	1.696338	1.024624	0.71461	1.603416
CARD14	1.771047	1.307987	0.719207	1.300407	1.147743	0.92768	0.616004	1.880196
CARD19	5.778408	5.752608	4.636457	6.229955	6.601882	5.232055	5.854902	5.565599
CARD6	5.798734	5.187566	4.592779	5.227147	6.164805	5.175049	5.422273	5.543886
CARD9	1.085	1.177062	0.886579	1.106956	1.195575	0.990905	0.881995	1.168615
CARF	1.904106	1.796193	1.397527	1.507245	1.661835	1.789039	1.666371	1.544163
CARHSP1	29.82179	27.88389	30.32768	33.06451	35.04652	35.73987	29.69417	26.84431
CARM1	18.52768	16.54697	15.66401	18.25405	17.84035	15.90227	15.97153	16.386
CARMIL1	14.3447	14.25649	12.07107	13.04852	13.52779	12.02176	13.19064	13.1546
CARMIL2	0.513501	0.405382	0.250259	0.328034	0.546916	0.174575	0.297831	0.547867
CARMIL3	0.433127	0.458757	0.142577	0.38333	0.137831	0.218043	0.452945	0.292862
CARNMT1	4.957506	4.956191	5.1416	4.47604	5.809988	5.422347	4.956502	4.842019
CARNS1	0.02122	0.028665	0.062867	0.043491	0.042445	0.035608	0.057435	0.064566
CARS	38.51795	46.12864	57.46415	48.04243	37.73452	45.72355	46.79346	39.25926
CARS2	9.52545	10.0074	10.97885	13.5055	11.11881	11.67942	10.87356	10.42864
CARTPT	0	0	0	0	0.146867	0	0.05962	0
CASC1	1.032094	0.921405	0.585303	0.869166	0.705185	0.730488	0.964599	0.601125
CASC3	28.26519	26.26844	26.36228	27.07328	27.2014	26.61789	23.33485	24.82905
CASD1	8.340055	10.95111	10.35042	10.35076	7.856183	10.4024	10.47166	8.057783
CASK	11.18004	9.716031	10.07119	10.16193	10.69563	11.14354	10.92362	10.28487
CASKIN1	0.368341	0.329558	0.330683	0.431389	0.416232	0.404572	0.276758	0.252291
CASKIN2	9.639893	9.065111	7.093607	9.913563	10.82145	9.111635	8.920646	7.952904
CASP10	0.049018	0	0.048407	0	0.049024	0.222087	0.049753	0.024858
CASP14	33.23612	37.06258	28.50668	24.68723	25.50706	20.91857	31.57472	18.94704
CASP2	12.00088	12.07226	10.34799	11.78341	10.8045	9.165235	11.09065	11.63876
CASP3	4.006169	4.095229	3.431714	5.563051	4.853043	3.562202	5.117093	5.743263

CASP6	3.644312	5.345017	5.649441	2.735799	3.072637	5.461147	5.978579	5.436839
CASP7	2.381188	2.2608	1.70653	1.910305	2.803361	1.959063	2.237364	2.580692
CASP8	5.878343	6.457006	5.163482	5.897132	7.219951	6.375368	5.83044	5.962025
CASP8AP2	4.888339	4.923937	4.282654	4.175896	4.574853	4.41902	5.24367	5.501095
CASP9	4.226683	3.220135	4.38438	3.705225	3.823569	4.227338	4.233186	4.656409
CASQ1	0	0.041947	0	0.099003	0.138031	0.013896	0.056034	0.013998
CASQ2	2.257536	2.677391	3.338827	2.499848	2.974768	1.518896	1.987327	2.398189
CASR	0.028433	0.023045	0.089851	0.040792	0.034124	0.057254	0.011544	0.011535
CASS4	0.509363	0.855725	0.311997	0.640912	0.625501	0.240192	0.746055	0.745494
CAST	177.3628	166.0489	163.2853	168.1145	170.6406	173.5262	152.9164	154.7948
CASTOR1	9.441359	9.371377	7.433775	11.57105	10.04869	8.927799	9.404918	12.39026
CASTOR2	7.438171	5.899757	5.693673	5.599366	5.592774	5.509664	5.181279	6.245525
CASZ1	28.19926	23.55427	19.8928	22.0291	21.79665	18.91934	19.7854	22.41072
CAT	44.77803	68.7519	64.62761	78.94496	64.15543	60.30996	72.62558	58.62939
CATHL3	0.727441	0	0.034208	0.212989	0.346445	0.31389	0.140639	0.1054
CATIP	0	0	0	0	0	0	0	0.016409
CATSPER1	0	0	0	0	0	0	0.017569	0
CATSPER2	0.434261	0.523762	0.377797	0.519188	0.496363	0.458048	0.325334	0.566284
CATSPER3	0.085009	0.107656	0.104937	0.130672	0.21255	0.106987	0.107855	0.150883
CATSPER4	2.275466	1.479964	1.183664	2.082334	0.646203	0.923947	1.4732	1.282144
CATSPERB	0.011961	0	0.005906	0	0.005981	0	0	0.012131
CATSPERD	0.478698	0.287029	0.318371	0.230261	0.224724	0.206558	0.16857	0.485512
CATSPERE	0.079289	0.08033	0.078301	0.812534	0.158599	0.079831	0.080479	0.402091
CATSPERG	0.278259	0.259358	0.113581	0.444837	0.18182	0.179302	0.158163	0.158043
CATSPERZ	0.322642	0.294188	0.047793	0.165317	0.419488	0.454784	0.180115	0.343596
CAV1	133.087	136.3874	140.516	146.9922	160.6283	149.0943	141.3532	169.5479
CAV2	12.70165	15.53427	15.07005	14.53718	16.4642	15.49279	13.92725	14.60937
CAV3	0.112406	0.256233	0.138757	0.259179	0.252947	0.113174	0.199662	0.285016
CAVIN1	90.50997	103.5881	103.4829	105.6611	96.82392	93.67071	102.1489	98.42823
CAVIN2	8.439856	12.38154	11.79255	10.5554	15.83056	11.97723	14.07785	15.74703
CAVIN3	33.22674	33.24167	17.92522	46.34632	40.74197	39.78734	35.07527	31.47933
CAVIN4	0.739551	0.724556	0.617977	0.416412	0.715263	1.120996	0.593916	0.717109
CBARP	3.407655	2.552814	2.306659	3.172368	3.024085	2.963812	2.638733	2.750328
CBFA2T2	3.402433	3.424933	3.044764	3.625643	3.582201	2.637507	3.81746	2.945214
CBFA2T3	2.913165	2.697478	3.018299	3.408941	3.366035	2.854414	2.517462	4.532642
CBFB	29.08351	29.57795	30.12237	27.2569	26.41599	28.74062	30.27281	28.34998
CBL	7.9087	6.730287	6.001196	6.988808	7.604813	6.265458	6.347494	7.966719
CBLC	2.881238	4.222199	3.014702	3.585308	3.121744	3.695221	3.707793	4.157254
CBLIF	0	0.019692	0.038389	0.039837	0.019439	0.01957	0	0.059141
CBLL1	7.16567	7.126055	6.219265	7.072779	8.034644	6.795168	6.920774	6.654996
CBLN1	0	0.009124	0	0	0	0.009067	0.036562	0
CBLN2	0	0	0	0	0	0.064456	0	0
CBLN3	0.312845	0.412035	0.257455	0.587755	0.479757	0.293983	0.497475	0.179802
CBLN4	1.236999	1.570719	0.749239	1.791598	1.699032	1.926295	1.188591	1.221152
CBR4	5.14321	6.590927	5.804714	5.654924	6.391571	5.132111	6.206944	7.203637
CBS	44.70983	32.74174	41.74833	34.17372	34.96496	47.7914	41.39899	30.28715
CBX1	30.56598	32.65783	25.26535	27.55076	31.45353	31.59019	30.22738	32.3452
CBX2	6.974724	6.072472	5.164378	5.975771	5.302438	5.162262	6.328043	6.732215
CBX3	30.89174	35.22807	36.69302	36.04278	32.06581	37.14143	35.41355	33.07382
CBX4	11.54567	11.4324	10.48159	13.31062	12.1268	10.6177	11.04987	11.0185
CBX5	23.84696	23.31676	19.63423	19.40809	21.32452	17.17904	23.41775	25.32579
CBX6	3.190618	3.265096	3.09638	3.966924	3.315177	2.916169	2.776504	3.124129
CBX7	4.484661	5.157276	3.988396	5.143271	5.053259	4.477176	4.53058	4.164483
CBX8	3.548027	3.141492	2.458562	3.215797	3.332297	2.791771	3.427238	3.371737
CBY1	21.71724	21.58232	20.32637	20.2208	19.34186	17.15423	19.21981	19.97974
CC2D1A	7.712232	6.719256	6.226604	7.217381	8.002285	7.045093	7.079091	6.510636
CC2D1B	14.43577	13.22554	12.41354	15.19122	13.81737	13.64091	12.51818	13.39858
CC2D2A	3.2088	3.021529	2.374686	3.424294	2.850032	2.994902	3.336176	3.468277

CC2D2B	3.614899	2.023028	3.303884	2.598284	2.9146	2.389675	3.722515	4.413289
CCAR1	23.16967	22.37608	19.59733	17.98158	23.61482	18.96455	21.48214	22.71009
CCAR2	40.21616	37.59245	34.444	40.08556	37.64619	36.33601	35.14042	37.89485
CCBE1	5.030732	5.065866	4.124986	3.171318	4.970396	2.931618	4.239688	5.411581
CCDC102A	4.361184	4.382692	2.937012	4.168053	4.702693	4.237116	4.677165	3.612535
CCDC102B	0.169158	0.145012	0.1028	0.066672	0.156166	0.157213	0.171696	0.079184
CCDC103	0.480688	0.597678	0.258927	0.537376	0.808535	0.571967	0.620962	0.376729
CCDC105	0	0	0	0	0	0	0	0
CCDC106	6.527508	6.379376	5.729933	6.27253	7.407591	7.501489	4.896565	7.512072
CCDC107	11.67646	15.16985	15.09201	14.11248	13.0175	15.90549	13.21106	11.28538
CCDC112	4.377759	4.697651	4.041819	4.061469	4.171067	3.416593	3.680944	3.520536
CCDC113	0	0	0	0	0	0	0	0
CCDC114	0.272199	0.215821	0.157778	0.333517	0.218971	0.196607	0.156159	0.426114
CCDC115	12.90553	10.99802	11.96256	11.67351	11.80054	13.39242	12.37795	12.45133
CCDC116	0.059174	0.02398	0.035062	0.157663	0.106526	0.023831	0.048049	0.096026
CCDC117	18.74384	18.84214	18.41937	16.79434	19.99704	19.09591	17.38182	17.64109
CCDC12	16.65443	16.88149	15.29696	17.25584	15.66791	16.10187	15.98591	15.3791
CCDC120	4.516173	3.577846	3.867684	4.848061	4.07989	4.051325	3.742278	4.006028
CCDC121	1.180122	1.485455	0.977067	0.855099	1.084899	1.09217	1.137328	1.196922
CCDC122	0	0	0.025499	0	0.025824	0.025997	0	0
CCDC124	38.63966	38.32112	38.57447	38.30088	36.08708	37.12113	35.79648	35.19949
CCDC125	1.295737	1.211762	1.14425	0.989491	1.707105	1.204236	1.087547	1.14991
CCDC126	2.559985	2.916339	3.078648	2.460163	2.173423	2.703483	2.34432	2.734904
CCDC127	5.170729	5.859607	5.897143	6.008019	6.100864	6.788772	6.442451	6.297216
CCDC13	0.081443	0.132019	0.048257	0.178048	0.141186	0.081999	0.104708	0.203752
CCDC130	5.175831	5.07932	4.577053	5.451886	5.735635	5.28382	6.095488	4.64591
CCDC134	4.350739	3.631558	3.966026	4.2419	3.514512	3.866156	3.790251	3.671273
CCDC136	0.199974	0.39796	0.218641	0.519636	0.235714	0.280438	0.260966	0.311474
CCDC137	11.66215	11.61945	8.845478	9.956832	10.24194	10.29667	9.651776	10.3724
CCDC138	3.744232	3.439046	2.478597	3.605073	3.569826	3.106987	2.944263	3.588878
CCDC14	2.131833	2.247479	1.569242	2.144333	1.982625	1.885028	1.357372	2.026547
CCDC141	0.111221	0.208026	0.105611	0.105208	0.179688	0.172278	0.199727	0.186561
CCDC142	1.687358	1.419756	0.875531	1.279769	1.220394	1.209376	1.141779	1.624359
CCDC146	0.52331	0.345419	0.414998	0.479395	0.507518	0.447054	0.338011	0.498593
CCDC148	0.13815	0.149294	0.100048	0.122696	0.092112	0.055637	0.186963	0.037364
CCDC149	5.32395	5.719433	4.912625	4.954635	5.149945	4.473968	4.942854	5.144634
CCDC15	2.336218	2.357669	1.683202	2.13791	2.06832	2.146248	1.932993	2.245009
CCDC150	0.900378	0.500236	0.535408	0.436537	0.464771	0.594605	0.569949	0.805183
CCDC151	0.03271	0.044186	0.010768	0.100562	0.043619	0.153691	0.088536	0.121645
CCDC152	0	0	0	0	0.062905	0	0.06384	0
CCDC153	0.039701	0.080444	0	0.020342	0	0	0.020148	0
CCDC154	0.1079	0.317016	0.202455	0.210087	0.280575	0.347638	0.197133	0.142266
CCDC155	11.73903	12.08823	11.47262	10.73125	8.830755	12.10501	9.908681	8.934745
CCDC157	0.253181	0.17314	0.181269	0.155671	0.215232	0.152946	0.199159	0.301723
CCDC158	1.876624	1.406618	1.009489	1.688583	1.617462	1.635982	1.695713	1.996185
CCDC159	0.347691	0.3229	0.371972	0.311766	0.753429	0.306308	0.470542	0.337948
CCDC160	0.134888	0.296092	0.133207	0.322534	0.112421	0.430062	0.205366	0.159609
CCDC162P	0.13565	0.139612	0.082928	0.134597	0.133514	0.108394	0.148612	0.109192
CCDC163	1.497439	1.55455	1.697859	1.458752	1.29425	1.265697	1.351023	1.256255
CCDC166	0.92918	0.576351	0.318352	0.75787	0.379306	0.133647	0.327204	0.250026
CCDC167	10.66937	15.85055	17.48197	10.49239	13.73321	12.86181	12.28629	14.41217
CCDC168	0.014012	0.020111	0.003459	0.009573	0.008175	0.004703	0.007111	0.014212
CCDC17	2.046873	2.268149	1.831866	3.961046	2.558922	1.840051	2.133226	1.844314
CCDC170	0.265149	0.515768	0.628429	0.760808	0.275791	0.619349	0.78585	0.193625
CCDC171	0.453109	0.544308	0.389932	0.477599	0.589117	0.400808	0.433625	0.430016
CCDC172	0	0.043161	0	0	0	0	0	0
CCDC173	0.008067	0.020433	0.0478	0.004134	0.004034	0.028428	0.012282	0.024546
CCDC174	10.76857	11.10406	9.264541	8.884363	9.674715	10.83295	10.75411	9.90373

CCDC175	0.098423	0.012464	0.036449	0.063038	0.036914	0.01858	0.031219	0.074868
CCDC177	0.026694	0.013522	0.013181	0	0	0.040315	0	0
CCDC178	0	0.010902	0	0	0	0	0	0
CCDC179	0	0	0	0	0	0	0	0
CCDC18	1.051182	1.620873	1.180678	1.319891	1.126412	1.244449	1.14316	1.716378
CCDC180	0.158327	0.090227	0.288277	0.481675	0.064328	0.104612	0.220964	0.105381
CCDC181	0.012916	0.052342	0.076531	0.013236	0.335862	0.039013	0.065549	0.0917
CCDC184	1.165709	1.119497	1.343048	0.435526	0.752951	0.819126	1.133895	0.73894
CCDC186	17.28779	17.39605	16.68006	17.26176	17.45732	17.56589	15.85777	15.22658
CCDC187	0.227277	0.362659	0.258112	0.390119	0.573949	0.400452	0.311426	0.518653
CCDC188	0.022056	0.20111	0.021781	0.180819	0.022059	0.155447	0.067161	0.11185
CCDC189	0.239053	0.444016	0.157383	0.142902	0.478169	0.280801	0.283079	0.34348
CCDC190	0	0.107547	0.139775	0.072522	0.070778	0.267197	0.071831	0.035888
CCDC191	1.436415	1.024338	0.702372	1.443413	1.004225	1.024996	1.224404	1.011317
CCDC192	0.013036	0	0	0.013359	0	0	0.039696	0
CCDC194	0	0	0.062677	0	0	0.063901	0.06442	0
CCDC196	0	0	0	0	0	0	0	0
CCDC197	0.036925	0.224458	0	0	0	0	0.131176	0.018725
CCDC22	6.795622	6.661919	6.759228	6.425373	7.273234	6.386731	7.31935	7.276649
CCDC24	6.136585	4.305283	3.527958	4.310489	4.970412	4.916699	3.962347	3.886312
CCDC25	13.97313	15.04039	14.23686	14.92749	14.17519	15.79656	13.44238	11.61905
CCDC28A	5.676243	6.873255	6.194685	5.013318	5.625833	6.384348	4.740601	5.791625
CCDC28B	13.11071	16.9542	14.63487	16.30372	18.4163	14.03086	16.47722	14.28345
CCDC3	45.3843	46.06826	41.28841	45.11234	32.20042	37.01417	41.54042	53.22677
CCDC30	0.494927	0.856597	0.631315	0.76078	0.57749	0.695555	0.727363	0.773875
CCDC32	21.84983	21.28047	23.07626	19.65182	17.81672	22.25954	20.39217	19.71216
CCDC33	0.011268	0.011416	0.033382	0.011547	0.011269	0	0.011437	0.011428
CCDC34	5.171552	5.390077	4.274964	4.063632	5.734059	5.223517	5.265895	4.07213
CCDC36	0.051383	0.141299	0.057992	0.067701	0.066073	0.059125	0.037253	0.089339
CCDC38	0.021689	0.054933	0.032128	0	0.043383	0.054592	0	0.010999
CCDC39	0.072118	0.058452	0.056976	0.088686	0.036064	0.014522	0.0732	0.051202
CCDC40	0.138206	0.11105	0.112952	0.151397	0.171589	0.095966	0.135442	0.130507
CCDC42	0	0	0	0.024898	0	0	0.049321	0
CCDC43	18.52187	21.81854	21.24286	19.54458	20.10027	19.35355	18.50774	18.8236
CCDC47	28.66137	32.42566	31.74789	32.45398	31.46352	34.8215	31.13818	30.35625
CCDC50	7.452449	8.751831	8.099316	8.475738	6.853393	7.90489	8.387889	8.223297
CCDC51	3.264008	3.270505	3.305987	2.413689	3.384006	2.9974	2.997447	3.0922
CCDC57	1.47072	1.351772	1.115499	1.173091	1.311688	1.099126	1.261938	1.214854
CCDC58	17.13016	17.95688	20.59993	18.70449	16.37314	20.53711	16.11407	17.70878
CCDC59	13.72732	15.23669	14.08414	14.51552	15.94033	20.59711	14.25382	15.99267
CCDC6	19.87162	17.68448	17.32949	17.44758	18.53167	18.25778	18.10863	17.97618
CCDC60	0	0	0.011389	0	0.011534	0.011611	0	0.023393
CCDC61	2.179809	2.326056	2.288166	2.471787	2.528483	2.311609	2.191077	2.408905
CCDC62	1.810958	3.297055	2.213358	2.296802	2.223642	2.617652	2.12931	2.464139
CCDC63	0	0	0	0	0	0	0	0
CCDC65	0.029155	0.088614	0.014396	0.074694	0.087477	0.044032	0.147964	0.014785
CCDC66	3.717925	3.713666	3.809499	4.14988	3.508918	4.033299	3.45479	3.779703
CCDC68	0.999613	1.012732	1.70696	1.045715	1.374646	1.132247	0.676405	1.140574
CCDC69	0.348365	0.549657	0.214311	0.380404	0.633993	0.321996	0.428946	0.486545
CCDC7	0.469649	0.504649	0.426318	0.388915	0.35584	0.353449	0.356316	0.447466
CCDC70	0	0	0	0.074115	0	0	0	0
CCDC71	9.019491	8.624811	7.690658	9.551471	9.362867	8.998427	9.238135	9.147889
CCDC71L	0.935199	0.507574	0.725643	0.718773	0.634681	0.302653	0.406811	0.745259
CCDC73	0	0.014742	0.007185	0	0	0.014651	0.007385	0.007379
CCDC74B	0.40248	0.186891	0.198733	0.326523	0.251583	0.236384	0.238302	0.27214
CCDC77	5.075154	4.310404	3.840415	4.040041	3.853691	4.804493	4.300279	6.052043
CCDC78	0.037352	0.100913	0.012296	0.038277	0.074714	0.037607	0.012637	0.012628
CCDC8	12.06803	10.90928	12.39213	12.09664	10.7882	7.977009	9.514291	10.79704

CCDC80	63.40998	114.9806	51.56121	75.9429	86.7307	83.70478	91.19972	112.7915
CCDC81	0.056872	0	0.056163	0.016652	0.040628	0	0.032986	0.049441
CCDC82	4.47421	5.030195	4.097992	4.24833	4.620838	4.382252	4.043136	5.105655
CCDC83	0.082128	0	0	0.100995	0	0	0.016672	0.033319
CCDC84	4.942945	6.867258	3.995708	6.411878	6.424573	6.005653	5.609825	6.747874
CCDC85A	0.331046	0.543014	0.490382	0.444251	0.417803	0.476154	0.496017	1.343035
CCDC85B	9.936885	11.0559	10.43944	11.26633	11.25567	12.93579	10.81226	8.87422
CCDC85C	13.2662	11.64302	12.46792	12.88488	12.34842	14.08693	12.42688	11.8134
CCDC86	11.84974	12.06263	12.05161	12.37539	12.94153	11.81666	12.1137	11.58766
CCDC87	0.07919	0.089143	0.052135	0.117219	0.0264	0.044295	0.098239	0.044621
CCDC88A	2.254714	2.32225	2.737041	2.490957	3.521105	2.232407	3.067853	3.528983
CCDC88B	0.214582	0.239695	0.287978	0.186067	0.225615	0.210508	0.223385	0.424112
CCDC88C	2.549778	1.967996	1.714551	2.477303	2.480028	2.28892	2.390465	2.613713
CCDC89	2.595359	2.216962	0.934749	3.421036	0.834694	3.145954	0.991732	1.166473
CCDC9	17.47592	15.02994	15.40245	15.04056	14.40194	17.69488	15.51955	15.58811
CCDC90B	11.46169	14.72301	15.1617	13.99322	13.86902	12.93619	13.27094	13.23224
CCDC91	6.513683	5.292622	6.367764	6.047845	5.301249	5.446812	7.199314	5.209752
CCDC92	2.549762	3.503277	3.230847	3.090166	3.429232	3.112267	3.184786	2.704144
CCDC93	5.356598	6.479181	4.644649	5.02147	5.085027	5.03477	4.841816	5.49232
CCDC96	1.585524	0.869309	0.91183	1.232935	1.333878	1.230133	1.041316	1.07837
CCDC97	15.64967	14.38017	14.24808	15.53116	15.2877	14.36937	14.97849	15.10783
CCDC9B	2.57034	2.204162	2.560976	2.892705	2.3641	2.712673	2.32937	2.737277
CCER1	0	0	0	0	0	0	0	0
CCER2	0.802749	3.198916	0.792746	1.453318	2.194463	3.152107	1.167865	0.651341
CCHCR1	5.565672	5.131865	4.808179	4.80635	4.860541	4.69523	4.279939	5.110313
CCIN	0.065664	0.05821	0.040528	0.084113	0.147762	0.033056	0.024993	0.033299
CCK	0.048875	0.297099	0.353952	0.050086	0	0.114821	0.08268	0.198284
CCKAR	0.0079	0.008004	0.007801	0.040478	0.015802	0.015908	0.016037	0.080123
CCKBR	0.035731	0.132733	0.011762	0.109848	0.047647	0.059958	0.120889	0.02416
CCL1	0.233521	0.33798	0.131778	0.376051	1.301214	0.134352	0.237024	0.507526
CCL14	72.79498	60.31986	73.31713	98.44582	98.1629	122.1344	75.17956	57.8614
CCL16	0.302297	0.018016	0.035121	0.236895	0.231198	0.071615	0.126342	0.108212
CCL19	3.384559	5.124203	4.13104	5.066196	12.43701	7.274825	4.245911	6.51835
CCL20	0.638189	1.53944	0.900339	2.211135	3.525692	1.77466	2.837816	0.523984
CCL21	32.8926	57.54609	50.34826	27.96462	66.52474	21.03562	46.61673	56.40454
CCL22	0.725873	1.6453	1.105617	2.408066	1.378106	0.545027	0.636861	1.297719
CCL24	1.731653	2.458518	2.408066	1.846977	4.029264	1.66046	4.148958	2.963016
CCL25	0.619981	0.445761	0.414754	0.625091	0.540054	0.382585	0.517635	0.557813
CCL26	31.333	47.26082	42.80595	42.98139	40.58541	42.72776	56.5247	66.57271
CCL28	0.445282	1.031145	0.314096	0.195562	0.954299	1.280926	0.193698	0.903242
CCL5	2.780623	3.706731	2.167876	4.724171	8.233171	3.683709	5.087625	2.783099
CCM2	13.23337	12.84018	11.44938	12.74776	13.18303	12.09228	12.46774	13.64611
CCM2L	0.784894	0.819789	0.823059	1.111145	1.165354	0.936902	0.870585	0.713999
CCN1	17.31615	25.59748	22.65563	22.02983	20.94988	17.42059	28.78593	16.23743
CCN2	57.5244	54.44447	57.28147	46.73876	64.27676	40.68709	83.33307	60.20877
CCN3	10.63588	13.17002	15.04867	10.65713	11.79429	10.35785	11.01007	14.86249
CCN4	1.815648	0.991133	0.884231	1.095983	1.409589	1.327215	1.127608	0.908134
CCN5	9.20371	8.499583	6.892155	9.327389	9.902901	8.754215	9.356523	8.700618
CCN6	0.192213	0.104858	0.350433	1.075782	0.369688	0.863424	0.180089	0.224941
CCNA1	0.510405	0.348744	0.246161	0.510884	0.498599	0.549744	0.265054	0.192622
CCNA2	11.18708	11.44267	10.18889	11.45318	9.180602	10.49603	11.62731	11.99967
CCNB1	11.58398	12.02333	10.11471	10.87609	9.392746	10.66373	12.87594	14.3155
CCNB1IP1	0.013431	0.136068	0.026526	0.178921	0.040297	0.067611	0.040896	0.190704
CCNB2	5.81518	6.93393	5.251793	5.79336	5.06437	6.448545	7.93246	6.155925
CCNB3	3.384437	4.100284	2.45269	3.164962	2.921546	2.902255	3.16091	3.45872
CCNC	10.4714	11.82109	12.32909	12.12566	11.52383	14.29735	10.76985	10.903
CCND1	37.32332	42.89846	47.48111	40.63236	47.24198	40.73101	36.95195	36.69486
CCND2	60.5145	57.62561	58.29904	54.26591	56.5969	44.05208	52.63829	63.23584

CCND3	22.28566	22.75657	22.47623	21.86587	23.0202	19.88721	25.71386	31.41049
CCNDBP1	22.62379	23.18239	26.23752	23.66544	25.35085	22.74289	21.85491	21.82655
CCNE1	1.439817	1.03878	1.604997	1.21839	1.538185	1.866977	1.051775	1.205865
CCNE2	3.188239	3.677816	3.262816	3.439739	2.462523	3.432497	3.545785	3.137578
CCNF	2.925437	2.389625	2.566731	2.446428	1.992532	2.835906	2.61486	3.007728
CCNG1	97.08071	117.2028	130.1922	113.5093	108.1713	108.5844	109.8643	112.7532
CCNG2	14.42534	14.22899	12.93975	12.43181	11.21114	12.37556	14.65189	14.49861
CCNH	9.990683	11.71347	10.46871	11.03532	11.19711	12.86929	10.41921	11.92744
CCNI	139.8216	134.4466	156.9183	129.4235	127.8687	135.8236	134.3368	134.8266
CCNI2	0.687034	1.119733	0.648975	1.010162	0.746873	0.631577	1.00053	1.272443
CCNJ	6.687618	6.228015	6.89709	5.45561	6.503973	8.577501	5.985418	6.154658
CCNJL	0.376325	0.41939	0.195109	0.269953	0.43283	0.530454	0.133689	0.248093
CCNK	10.09898	10.34143	10.19715	9.560822	11.205	10.51551	9.319514	10.37278
CCNL1	22.14817	22.70551	18.23302	24.84187	22.60232	22.79165	23.58723	25.04415
CCNL2	20.44084	17.64465	14.04457	18.31313	18.64603	16.85223	16.26617	19.92016
CCNO	0	0.093509	0.136721	0.047292	0.069232	0.139392	0	0
CCNQ	5.397639	5.750887	5.430484	6.180558	5.765829	5.804468	6.237377	5.397372
CCNT1	8.738459	7.539423	6.990641	7.626093	8.290986	7.472069	8.377013	8.924892
CCNT2	8.639933	9.147256	7.806611	8.806672	9.069477	8.071915	7.946761	9.752183
CCNY	47.38951	47.94083	48.14511	46.35702	49.58766	50.4424	47.25139	46.32034
CCNYL1	13.00407	12.5085	14.3032	12.55121	15.08568	15.26127	14.4256	13.33092
CCP110	7.2883	7.239546	7.436778	6.626521	6.994632	7.11326	6.828577	7.724552
CCPG1	30.12807	32.39858	33.58923	34.75714	33.869	37.31306	31.85601	32.70862
CCR10	0.025215	0.204371	0.149408	0.07752	0.075656	0	0.051187	0.127872
CCR3	0.900387	1.928659	0.406477	1.02814	1.15779	0.751131	2.010564	1.435035
CCR4	0.309804	0.551978	0.379792	0.624008	0.929531	0.150582	0.975882	0.931807
CCR5	0.883927	1.127912	0.872913	1.169543	1.437966	0.850537	1.06186	1.634146
CCR6	1.209331	1.288357	0.812591	0.638808	1.446398	0.276155	1.354015	1.175968
CCR7	0.155323	0.132183	0.061355	0.229205	0.099419	0.050043	0.157653	0.264657
CCR8	0.532302	1.51753	0.415645	1.078288	1.634254	0.211883	0.8921	0.828652
CCR9	0	0.025341	0.024701	0.051264	0.025016	0	0	0.025369
CCRL2	1.160173	1.616173	0.877189	1.727636	1.867394	1.879908	1.159176	1.323774
CCS	7.989385	8.584798	6.559271	6.860492	8.337828	6.740398	6.741662	7.729458
CCSAP	15.43103	15.1651	15.3727	12.45855	12.9852	12.78669	14.57995	15.73199
CCSER1	0.198187	0.3951	0.252539	0.26206	0.390032	0.296093	0.227116	0.194524
CCSER2	8.956312	9.632933	8.463242	9.517466	10.07232	8.450778	9.902823	8.876725
CCT2	94.70736	94.50721	99.06944	93.82742	95.54585	101.7935	95.15931	94.84206
CCT3	148.3215	152.457	153.1237	149.1632	153.841	149.4054	158.649	161.2758
CCT4	89.19003	77.9689	94.84369	75.95756	85.34633	80.56901	73.97126	77.92834
CCT5	90.7729	83.17732	87.27954	81.91807	88.31544	87.28446	89.90125	84.12489
CCT6A	46.29482	44.50794	46.29067	40.06351	48.18304	44.96962	40.24794	46.72081
CCT6B	0.092373	0.155976	0.091222	0.047331	0.076988	0.170508	0.062506	0.218606
CCT7	155.707	150.3852	155.533	154.334	157.8793	159.9181	159.0461	153.9419
CCT8	87.80371	93.95105	83.81022	88.81488	83.9922	91.4154	90.97628	93.19732
CCT8L2	0	0.017668	0.017221	0.017871	0.017441	0	0.0177	0
CCZ1	32.79883	33.19785	35.71497	34.77192	32.58583	35.02239	37.51122	32.82514
CD1	0	0.041886	0.061242	0.063551	0.041348	0	0.041963	0.251589
CD101	0.347647	0.208451	0.357329	0.348988	0.425746	0.142866	0.136824	0.424555
CD109	13.42313	14.07874	14.95181	12.80097	15.16623	15.72036	13.93262	13.34858
CD14	13.4448	11.59578	11.52507	12.49736	14.54625	6.894127	15.82787	22.12713
CD151	61.40193	59.30379	57.27893	67.11294	61.62066	54.12556	63.26422	58.67057
CD164	72.73482	76.20163	77.96881	83.80021	93.15813	84.50386	79.07564	80.7428
CD164L2	4.178594	3.281538	3.760268	5.34629	3.758748	4.530767	3.362903	3.485758
CD177	0	0	0	0	0.017216	0.017331	0	0.017459
CD180	0.025868	0.10483	0.08941	0.013254	0.025871	0.013022	0.013128	0.144299
CD19	0.087148	0.073577	0.071719	0.104192	0.029053	0.102368	0.044228	0.058926
CD1D	7.202279	9.744037	6.981229	10.40108	11.45864	7.853913	10.6843	11.44045
CD1E	3.416174	7.486471	6.283887	10.21691	8.504879	4.369508	4.003154	9.353486

CD2	3.71315	4.151559	2.688074	4.062844	4.201876	1.906495	4.879982	3.405913
CD200	2.586435	2.957384	2.058115	2.720069	2.92624	2.214514	1.715703	2.609484
CD200R1	1.982092	2.826696	1.670643	2.742753	3.81318	1.995633	2.639718	3.393196
CD207	3.194625	2.776981	2.497169	2.007773	4.102391	2.225278	2.703753	2.33953
CD22	0.336271	0.572639	0.226097	0.49857	0.243291	0.237718	0.450245	0.573267
CD226	0.30401	0.554399	0.50037	0.581542	0.425669	0.081623	0.287999	0.596119
CD24	73.80055	91.45388	98.17471	93.61488	77.11929	93.14212	100.4161	79.62564
CD244	0.419763	0.850543	0.510763	0.614515	1.004562	0.566023	0.63148	2.402379
CD247	11.12967	13.06891	6.932323	8.700041	11.0711	8.245704	10.38314	9.310405
CD248	10.8256	10.77374	15.73151	15.68164	17.39977	10.21431	15.01403	12.09062
CD27	0.970393	1.156621	0.281853	1.091923	1.274994	0.383146	0.907698	1.138592
CD274	0.234494	0.380114	0.277886	0.278751	0.300191	0.245539	0.257052	0.104646
CD276	4.679022	5.228246	4.693444	5.479913	4.385026	4.876385	5.35867	4.969699
CD28	0.339263	0.257786	0.083759	0.651874	0.6362	0.256185	0.473483	0.602161
CD2AP	33.1471	33.73884	33.9514	34.10192	32.39717	35.53939	32.49617	31.19695
CD2BP2	26.36052	25.60079	25.6507	24.01963	24.09697	23.26106	26.31284	24.77743
CD300A	0.639752	0.997151	0.801876	0.743853	0.972057	0.520253	0.524474	1.335153
CD300E	0.289949	0.192776	0.429505	0.417841	0.235614	0.145965	0.386267	1.194687
CD300H	0.42089	0.030458	0.148445	0	0.210472	0	0.122058	0.335407
CD300LB	0.045809	0.41769	0.158333	0.117359	0.274888	0.023061	0.092992	2.021049
CD300LF	0.662696	0.6319	0.769928	0.599216	0.721263	0.588726	0.455019	1.561714
CD300LG	4.123795	5.974801	4.660374	4.893873	5.4218	7.956886	4.357404	4.258777
CD302	28.57227	31.81288	34.84274	31.81292	34.96487	31.06325	29.44254	33.92213
CD320	4.04739	4.023383	4.034548	5.187808	4.720451	5.020348	4.430053	4.130744
CD34	60.53636	46.4259	64.33354	58.88716	67.0565	58.99337	60.06062	61.38782
CD37	3.312679	4.80312	2.781672	4.614401	5.654106	2.29677	4.510005	5.230885
CD38	0.123846	0.085849	0.032185	0.053437	0.110824	0.091878	0.172015	0.231385
CD3D	5.621353	6.743603	3.693131	5.881111	7.739181	3.007485	6.899329	4.4132
CD3E	7.978716	13.60854	5.343674	8.763483	10.99337	4.74302	10.53222	10.11537
CD3EAP	7.92247	7.755738	7.414756	6.160912	7.589451	8.554996	8.14979	7.506795
CD3G	5.931084	8.058791	3.134278	6.159311	7.737197	3.055703	6.22139	7.423832
CD4	0.428624	0.642689	0.338627	0.632508	0.865933	0.207145	0.513363	0.782507
CD40	1.820654	1.293937	1.449109	1.837906	2.147019	1.285901	1.737638	1.543404
CD40LG	1.102496	2.125836	0.667304	1.312031	1.956294	0.680339	1.191229	2.092098
CD44	103.1722	90.16944	83.69771	95.46621	84.03258	78.95418	89.33654	102.9268
CD47	33.36178	28.64514	31.11724	33.24274	37.47749	27.9345	30.21297	29.08939
CD5	0.780138	1.539154	0.689321	2.1319	1.546789	0.702787	1.833735	1.860117
CD53	7.312484	9.890103	6.581051	8.78562	10.41173	4.896204	8.884669	13.37177
CD55	12.39578	13.758	10.79368	13.23933	14.02459	9.398864	10.72593	13.06238
CD58	18.40785	19.87195	18.12626	20.09054	19.91998	19.12897	19.63061	22.58526
CD59	88.68801	96.32596	104.1612	97.94658	114.4	97.97417	95.31806	103.1899
CD6	1.27856	1.43633	1.168147	1.559798	2.018125	0.866954	1.659693	1.949553
CD63	96.16317	124.7536	128.6363	129.3074	137.1037	127.0067	132.8875	120.5304
CD68	12.3919	10.89733	10.65478	10.54851	14.17816	8.517327	9.274013	16.35555
CD69	0.29864	0.638735	0.278534	0.204025	0.398238	0.050113	0.202079	0.168273
CD7	1.350684	2.559433	1.580864	1.768624	2.276446	0.755506	1.878701	0.964012
CD70	0	0.032896	0	0	0.032474	0.032692	0.032957	0
CD72	0.119954	0.283565	0.059229	0.368775	0.199948	0.120773	0.101461	0.263599
CD74	156.8614	211.3751	194.189	214.5377	239.0636	179.2131	169.8345	229.3825
CD79A	1.100829	2.515304	0.855812	1.248107	1.147821	0.660293	1.117342	1.425319
CD79B	0.055205	0.11186	0.038941	0.024246	0.03155	0.00794	0.128077	0.06399
CD80	0.175542	0.296409	0.064205	0.273166	0.260096	0.183287	0.290359	0.336299
CD81	225.7635	244.9187	208.0612	252.0104	247.0137	213.7507	235.3432	244.5886
CD82	39.348	39.4702	43.08434	47.78267	40.05604	51.39443	52.89425	32.49936
CD83	1.615004	1.765034	1.180463	1.133746	1.335412	0.78101	1.264917	1.560609
CD84	2.247834	2.497722	1.521655	2.452128	1.903654	0.967331	2.35515	3.89778
CD86	1.258563	1.331215	1.109994	1.46008	1.931629	0.884621	1.526502	1.750141
CD8A	0.514941	0.489092	0.519119	0.230867	0.536466	0.205223	0.653331	0.641958

CD8B	1.111483	0.839434	1.317161	2.319448	0.990358	0.956301	0.882012	1.168299
CD9	403.363	377.7752	379.4835	425.0522	390.0793	407.825	420.6961	414.2619
CD93	8.409687	6.874301	6.984635	10.70989	11.27188	10.88425	6.697346	7.30467
CD96	0.716486	1.170788	1.129811	0.781603	0.982406	0.488677	1.360629	1.113469
CD99	63.9494	79.36859	71.0518	74.88519	77.41145	61.22088	77.41171	92.78177
CD99L2	11.00332	8.251605	11.11511	10.71175	11.01801	10.87148	10.81158	9.659859
CDA	0.404802	0.199199	0.239855	0.248897	0.300749	0.279475	0.258264	0.527869
CDADC1	5.669614	6.329219	5.643274	5.39628	5.889085	6.041475	5.92542	6.080215
CDAN1	3.51465	3.262424	2.18114	3.21464	3.399855	2.848976	3.37893	2.993295
CDC123	43.48241	48.03068	47.3389	49.46968	45.93687	49.64506	43.25624	42.36733
CDC14A	5.913511	5.637401	5.207719	5.523313	6.147064	5.726885	5.588777	6.779681
CDC14B	3.742219	4.054292	4.036074	4.279543	3.525735	4.251434	3.821945	4.243407
CDC16	11.90699	11.9327	12.17858	13.39043	12.35961	12.75382	12.98809	13.16129
CDC20	13.66796	15.32371	12.85254	13.93786	12.39656	13.30599	15.98113	15.35751
CDC20B	0.013227	0	0	0	0.026458	0.013318	0	0.026831
CDC23	9.240625	8.888001	7.805683	9.166259	7.790624	8.582904	9.001352	9.691677
CDC25A	2.71181	2.172943	1.52566	2.416872	1.97248	2.20909	2.58567	2.208666
CDC25B	13.22953	13.01362	12.28979	13.36424	12.98796	13.54902	14.3837	14.34899
CDC25C	0.860475	0.844311	0.428222	1.145632	0.623417	0.777672	0.715211	0.700929
CDC26	27.04534	22.81709	28.0261	24.41345	25.1935	27.19734	26.62518	26.93522
CDC27	9.282643	8.279484	8.421501	8.753966	9.400743	9.252891	8.334362	9.014683
CDC34	44.26858	38.36807	43.77339	46.43828	45.30261	46.00877	39.07687	39.20194
CDC37	44.27104	43.93188	43.75233	45.24811	44.39344	41.53648	45.45102	44.81676
CDC37L1	8.607146	10.56102	9.927043	10.1924	10.62748	9.981886	9.372598	9.376317
CDC40	10.91609	12.06981	10.69476	10.77604	11.31807	11.06973	11.00012	10.82457
CDC42	65.48951	53.2935	56.77843	62.6886	64.83685	70.89333	66.57447	67.73903
CDC42BPA	8.569702	8.042914	6.740051	7.70516	7.907069	7.539908	7.99657	9.193975
CDC42BPB	19.568	18.09778	16.40451	19.00185	19.37518	17.71274	19.39292	18.94158
CDC42BPG	26.11139	23.86072	22.69079	25.08758	25.29722	23.88653	23.17477	22.59354
CDC42EP1	35.53029	33.84169	35.79171	37.02961	38.53238	37.48866	30.59242	30.97387
CDC42EP2	5.107512	5.682589	5.777525	5.652749	7.318617	5.591196	5.95703	4.181851
CDC42EP3	41.46933	34.7527	38.36637	35.82313	40.2937	42.19993	40.92842	37.45884
CDC42EP4	7.730979	7.709262	6.860421	7.561585	7.937438	7.65716	6.629902	7.709217
CDC42EP5	1.457588	1.881057	1.936371	1.849334	2.776717	1.502487	1.866928	1.883121
CDC42SE1	75.20003	68.99454	69.92476	73.38722	77.37872	75.2472	70.36402	74.02398
CDC42SE2	12.95579	13.80779	13.11442	12.13554	12.40891	12.62595	12.91395	13.04751
CDC45	3.590853	4.0751	2.858879	3.48012	2.282852	2.186046	3.658842	3.529041
CDC5L	15.28288	14.95526	14.05423	13.76723	15.01768	13.96872	15.32844	13.62133
CDC6	3.32862	3.103417	2.47901	2.799119	3.185271	2.939399	3.647936	4.105043
CDC7	4.393601	5.994584	5.415653	5.923821	4.77906	4.778797	4.622261	5.610842
CDC73	18.72076	18.14857	18.93151	16.87543	18.50344	18.03071	17.79322	17.08024
CDCA2	3.114509	3.05468	2.985721	2.614442	2.758685	2.368519	2.665183	2.990822
CDCA3	14.60838	12.18832	12.60791	13.58643	11.83554	12.0879	14.0799	14.29341
CDCA5	4.847224	5.05495	4.484274	4.373011	4.333519	4.71509	4.975462	4.771959
CDCA7	12.95205	14.42279	10.12298	14.88331	13.85771	15.35701	16.00287	18.73061
CDCA7L	18.61892	17.057	18.8371	21.3224	19.47236	19.85783	18.17847	19.60317
CDCA8	10.5871	9.643246	7.748256	9.367544	8.224295	8.355367	10.51618	9.66658
CDCP1	9.783328	7.947106	7.708023	8.790511	7.699352	7.623723	8.48965	9.429674
CDCP2	0	0.015205	0.014821	0.046138	0.01501	0.03022	0	0.030442
CDH1	107.5938	103.2145	90.75932	102.6642	98.61806	107.9009	103.3715	111.1508
CDH11	30.2213	25.90706	22.69771	27.2535	27.53675	25.51661	33.41381	26.5055
CDH12	0.029831	0.045334	0.007365	0.038213	0.007459	0	0.060557	0.015128
CDH13	24.87714	20.3228	26.94281	27.44986	28.54016	23.07997	25.64432	25.83156
CDH15	0.010079	0.010211	0	0.041314	0.060481	0.060887	0.081841	0.061334
CDH17	0.072581	0.082726	0.053758	0.092974	0.018148	0.018269	0.064461	0.193238
CDH18	0	0	0	0	0	0	0	0
CDH19	0.13628	0.733193	0.90495	1.064276	0.864787	1.239634	0.600996	0.78166
CDH2	2.5648	1.904593	1.200857	1.833386	2.355448	1.146917	2.071268	1.772011

CDH20	0.011015	0.133915	0.076144	0.067727	0.088132	0.110903	0.067081	0.100546
CDH22	0.011214	0.124969	0.033222	0.034474	0.04486	0.02258	0.022764	0.011373
CDH23	1.727789	1.514331	0.910673	1.539528	1.043902	1.392694	1.283137	1.359255
CDH24	4.863691	3.972445	2.982364	3.650496	3.195599	3.074223	3.979801	4.382945
CDH26	0	0	0.006487	0	0.00657	0.026454	0	0.019987
CDH3	33.31504	28.24169	22.42566	29.73338	24.34919	22.69832	29.68568	33.88699
CDH4	0.632368	0.468894	0.375598	0.887522	0.728692	0.447527	1.093012	0.501942
CDH5	10.60615	9.130168	8.869232	12.51312	14.19634	11.11215	10.01599	9.930464
CDH6	0.138303	0.133113	0.163896	0.092124	0.107199	0.208872	0.122831	0.150793
CDH7	0.052089	0.017591	0.034293	0.142345	0.086826	0.069926	0.023498	0.129141
CDH8	0.078132	0.164403	0.065288	0.049272	0.114207	0.163382	0.146407	0.091435
CDH9	0	0	0	0	0	0	0	0
CDHR1	0.03142	0.267394	0.130321	0.103035	0.113128	0.069597	0.031892	0.044615
CDHR2	7.716733	1.044677	2.835256	1.919992	4.414946	2.212088	2.873051	4.203799
CDHR3	0.006902	0.013986	0	0.035367	0	0	0	0.021002
CDHR4	0.076874	0.120365	0.069015	0.107425	0.041937	0.049254	0.02128	0.085057
CDHR5	0.03211	0.032531	0.05285	0.021937	0.042819	0.021553	0.032592	0.097701
CDIP1	3.578133	3.12508	3.074281	2.830315	3.96778	2.924108	3.516204	3.195892
CDIPT	10.28835	10.58752	11.10683	11.94065	11.78857	10.67129	10.66194	10.76346
CDK1	12.19867	13.70907	11.97974	11.3665	11.16096	12.46396	12.38165	13.83867
CDK10	6.349183	6.294275	6.773114	7.112357	6.459173	6.77721	6.139742	6.873178
CDK12	7.069558	6.223276	5.96683	6.120944	6.721662	6.111864	6.544148	7.514367
CDK13	11.45454	10.70502	10.48817	9.895673	11.07687	9.80424	10.21911	10.92549
CDK14	3.661894	4.077489	3.839649	4.058757	4.33679	3.566592	4.630867	5.467147
CDK15	0.061201	0.020668	0	0.020906	0.020403	0	0	0.020691
CDK16	8.000015	7.275534	6.347791	7.50198	7.164793	6.993573	6.988429	7.367439
CDK17	8.010416	7.842815	7.946529	8.014986	8.346171	7.44249	7.40687	7.5046
CDK18	0.09328	0.084004	0.035824	0.132765	0.176218	0.062611	0.073639	0.084096
CDK19	17.94177	17.93331	16.8906	16.19883	16.13811	13.87808	16.06319	17.40355
CDK2	8.532143	9.199897	8.702466	7.882296	7.284194	8.449411	9.38279	8.949555
CDK20	3.198389	3.676821	3.674216	3.598686	2.872394	3.87743	3.312617	3.892704
CDK2AP1	28.50066	31.48649	31.84305	27.718	29.54794	31.78532	29.17737	26.49925
CDK2AP2	22.93532	23.68078	23.34272	24.02494	28.22094	24.52311	23.59995	22.99485
CDK3	1.086337	0.789838	0.492227	1.13944	0.61354	0.759197	0.596719	0.881442
CDK4	48.45856	51.80551	49.78361	50.37946	49.3048	51.66074	49.72071	47.97961
CDK5	20.57595	20.15195	18.26587	18.0519	18.69448	19.50949	19.17111	17.8167
CDK5R1	1.926555	0.84068	1.011843	0.857738	1.703093	1.365793	1.267017	1.375837
CDK5R2	0.024056	0.012186	0.011878	0.024652	0.01203	0	0.012208	0.024398
CDK5RAP1	0.126314	0.053322	0.08316	0.086295	0.052638	0.063589	0.064105	0.096084
CDK5RAP2	5.307637	5.066247	4.51467	5.339981	5.043328	4.611334	6.070263	6.19366
CDK5RAP3	16.52211	20.50067	19.64428	17.57317	16.10666	17.62587	18.67699	16.98417
CDK6	8.911557	7.799919	5.909836	6.896838	7.337418	5.606603	7.552626	9.82068
CDK7	8.848882	8.103413	8.961873	8.174503	8.085608	10.04739	8.380656	7.653736
CDK9	48.72765	44.88067	43.23916	48.56045	47.03411	46.21895	50.47148	48.72547
CDKAL1	6.671307	6.973916	6.767859	6.971217	6.844028	6.879716	6.97657	6.735521
CDKL1	1.19513	1.382672	1.347756	1.999081	1.449764	1.824349	1.26784	1.360728
CDKL2	1.607556	1.976521	1.592663	1.82864	1.441264	1.608062	1.85345	1.794012
CDKL3	0.018892	0.00957	0	0.00968	0.009447	0	0	0
CDKL4	0.438368	0.645995	0.432906	0.258646	0.464996	0.775729	0.485393	0.619758
CDKL5	1.712717	1.861063	1.297014	1.536885	1.783941	1.215133	1.369109	1.692096
CDKN1A	89.88907	75.32323	102.4476	77.30197	95.62835	96.1351	71.44885	74.50189
CDKN1B	31.47803	33.69245	36.06616	34.4923	33.29385	34.28256	33.50514	32.56126
CDKN1C	9.608764	15.1923	15.3478	9.977321	10.26523	10.33403	11.95099	12.12657
CDKN2AIP	16.3065	17.72028	17.83125	18.03989	17.82569	21.10011	17.8949	16.58587
CDKN2AIP	52.04597	53.4501	58.63704	55.39563	54.04582	54.76631	53.80192	49.12338
CDKN2B	31.50864	31.60568	34.34486	30.86993	30.08599	34.00935	31.3577	30.24632
CDKN2C	5.677164	7.346567	6.891742	7.215076	7.004394	7.587984	7.43566	7.555781
CDKN2D	2.540803	2.635438	3.186215	2.975703	2.64197	2.517555	2.722188	4.069981

CDKN3	2.620951	3.573246	3.099561	3.017462	3.592137	2.96464	3.037957	3.921756
CDNF	3.539565	3.948766	5.647292	5.336029	2.905684	4.099338	2.86582	5.592442
CDO1	14.44424	30.51345	50.65899	29.94261	12.75737	26.03682	44.69062	10.77798
CDON	8.050541	7.711749	6.34223	6.905338	7.624693	6.581522	6.83549	7.523798
CDPF1	22.76794	24.54094	22.82236	27.05099	21.94908	26.43957	25.64629	24.67204
CDR1	0	0.036636	0	0	0	0	0	0
CDR2	6.820539	8.2724	8.18671	7.998132	9.150687	9.658652	7.316831	7.803429
CDR2L	2.67816	2.592178	2.503105	2.499455	3.180726	2.720532	2.293593	2.558644
CDRT1	0.140086	0.106443	0.096838	0.150734	0.231172	0.09873	0.071094	0.191808
CDRT4	0.014864	0.022589	0.036697	0.030465	0.014866	0.037414	0.022631	0.007538
CDS1	36.88985	36.2356	42.55682	45.31271	43.42627	46.77531	35.64604	32.31076
CDS2	8.509707	10.34633	7.271911	8.744119	7.749973	7.927906	9.051839	7.391667
CDSN	361.3873	214.7351	241.4619	267.0135	294.2751	278.0217	278.4805	267.7062
CDT1	2.459185	2.777515	2.437537	2.688106	2.195334	2.03581	2.57003	2.087732
CDV3	11.5461	9.999966	6.014699	9.566049	19.93472	2.74254	10.15828	5.370209
CDX1	0.017659	0	0	0	0	0.017779	0	0
CDX2	0.01676	0	0	0	0.050286	0	0	0
CDX4	0	0	0	0	0	0	0	0
CDYL	18.25343	16.38124	16.26405	15.04005	16.67728	17.74768	15.73248	15.50313
CDYL2	1.333747	1.074409	0.877015	1.140098	1.197274	1.034984	1.195265	1.273549
CEACAM1(	1.061363	1.37013	0.676218	1.350795	1.506645	0.758371	1.303165	1.284821
CEACAM1ł	0	0	0	0	0	0	0	0
CEACAM1!	11.31959	10.28918	10.79051	12.56019	12.42444	11.88384	9.464554	10.46908
CEACAM2(	0.244714	0.650805	0.377601	0.799347	0.198856	0.369579	1.148779	0.729081
CEBPA	26.72862	29.16445	27.43718	29.68424	30.53992	27.42924	22.8212	25.935
CEBPB	35.59921	33.84356	33.30123	47.15592	50.52934	44.2607	34.94949	33.64012
CEBPD	25.82798	34.72773	33.13351	28.91846	36.79811	31.87236	40.58172	31.72944
CEBPE	0	0.054584	0.026603	0	0	0	0	0.437153
CEBPG	22.27012	20.86157	23.50605	23.18583	24.09832	24.28085	20.78	20.63012
CEBPZ	25.23634	27.21855	26.20422	25.34915	27.72006	27.23907	26.41227	23.94937
CEBPZOS	7.084034	9.374047	10.85058	6.222436	7.027114	9.898065	6.838117	6.686337
CEL	0.014967	0	0	0	0	0	0	0
CELA1	0.76937	0.846663	0.759784	1.046706	0.968471	0.974961	0.565487	0.753415
CELA3B	0.127079	0.09656	0	0	0	0.063974	0.128985	0.128888
CELF1	7.978126	7.503832	6.738674	7.402682	7.744813	7.49175	7.633741	8.737993
CELF2	1.712713	2.10717	2.171328	2.083546	2.237215	1.688087	2.011981	3.168529
CELF3	0.072723	0.036839	0.026931	0.288781	0.054549	0.118982	0.046133	0.119857
CELF4	0.081925	0.071143	0.115578	0.131929	0.111199	0.182645	0.041577	0.166183
CELF5	0.005571	0.028221	0.016505	0.011418	0.011144	0	0.016964	0.022602
CELF6	0.610044	0.562702	1.007068	1.651528	1.074544	0.852562	0.794787	0.683371
CELSR1	9.124931	8.206445	7.887315	9.928943	9.395562	9.052907	8.817116	9.201201
CELSR2	17.99822	13.62408	11.19897	11.99579	12.59984	11.6586	13.21825	16.35702
CELSR3	0.011883	0.02167	0.058675	0.043838	0.016638	0.0335	0.031359	0.009642
CEMIP	0.858728	0.806561	0.459349	0.852499	0.805162	0.822566	0.959375	0.719745
CEMIP2	9.658169	9.228281	8.905559	9.218035	10.1408	8.585779	9.563381	11.73913
CEND1	0	0	0	0	0.031901	0	0.016187	0.016175
CENPA	11.81523	11.87566	11.66801	10.8317	8.656278	12.6796	11.78705	10.1046
CENPB	26.8874	29.47272	26.02928	33.31813	26.71075	27.67906	28.91439	27.85037
CENPBD1	0.920034	0.801031	1.277677	1.49526	0.575096	0.730924	1.043268	0.626948
CENPE	1.632701	2.001833	1.240528	1.577536	1.712892	1.627081	1.816147	2.341978
CENPF	7.927476	7.71915	6.706258	7.433021	6.175032	6.597869	8.404418	9.322965
CENPH	3.350815	2.92884	4.130923	3.837795	2.080841	4.057268	4.090185	2.354528
CENPI	3.198942	3.157824	2.511066	2.554007	2.524146	2.985747	2.638528	3.45566
CENPJ	2.311341	2.763291	1.932663	2.472328	2.530993	2.53663	2.197602	2.675063
CENPK	2.868932	3.512122	2.748864	3.027496	2.032423	3.816988	4.142618	3.845057
CENPL	5.677235	5.175228	5.005333	4.990613	4.473551	5.449538	5.775825	5.395659
CENPM	2.726494	2.086094	2.159627	1.862687	2.187158	2.044543	1.902582	2.318825
CENPN	5.320541	4.88118	5.288476	5.772013	4.090586	4.815965	4.67913	5.870874

CENPO	8.674724	9.775869	7.87247	7.645383	8.659868	7.398957	7.004959	7.858442
CENPP	3.799716	3.576116	2.993695	2.89378	2.761899	3.21942	3.287689	3.832748
CENPQ	4.131968	4.252643	3.72425	4.015881	3.820925	4.308774	4.360374	4.25731
CENPS	6.488716	6.929218	9.282746	8.015276	6.279083	8.828438	5.909642	7.43485
CENPT	3.135464	2.354865	2.056294	2.605241	1.791926	2.437749	2.59269	2.701243
CENPU	7.228433	8.685773	8.582639	9.526369	7.767367	8.834928	7.388047	10.02517
CENPV	6.268407	5.346381	5.441707	4.601146	4.432191	5.166402	5.977728	6.475923
CENPW	4.6676	4.029519	5.437193	5.069537	3.566902	4.070682	5.304793	4.283978
CENPX	12.72235	11.90366	13.19202	13.49763	10.40373	12.31949	12.98914	10.47457
CEP104	9.114768	9.453995	8.15031	8.774113	9.685015	8.549593	8.679459	8.997195
CEP112	3.82303	3.433632	3.718804	4.949592	4.322957	5.687177	4.063177	5.612759
CEP120	9.799179	10.21075	8.890707	8.897045	9.265552	9.08832	9.819502	9.534861
CEP126	0.77604	0.707602	0.510913	0.596447	0.459216	0.312536	0.899268	0.669024
CEP128	0.771983	0.943708	0.497742	1.000323	0.567896	0.687326	0.71233	0.821798
CEP131	4.475522	3.700016	3.87011	4.195719	3.667809	3.607948	3.536615	4.34591
CEP135	0.815644	0.732067	0.562215	0.891944	1.051166	0.722008	0.922334	1.088201
CEP152	1.275736	1.409533	1.0982	1.164269	0.885908	1.294145	1.13851	1.450141
CEP162	5.542076	4.972004	4.699298	5.965047	5.018799	5.691245	6.474111	6.361983
CEP164	3.661504	2.862959	3.034678	2.951117	4.016942	3.419641	3.050662	3.298977
CEP170	3.061053	3.461834	3.159377	3.484465	4.020507	2.94705	3.872023	4.416983
CEP170B	14.25186	12.09521	13.14767	14.94165	13.98558	12.08539	12.62671	15.29238
CEP19	7.125114	9.374186	7.231788	8.400227	7.455944	8.718147	7.767694	8.748803
CEP192	5.483143	4.819923	4.072478	4.835428	5.190011	4.23757	4.613274	5.873546
CEP250	3.114218	2.897822	2.24648	2.793794	2.705452	2.450158	2.412761	3.165705
CEP290	2.94856	3.529453	2.797368	2.686252	2.962578	3.0648	2.719459	3.267116
CEP295	12.26007	12.44246	9.592953	12.7232	11.65688	10.21116	10.83239	13.88357
CEP295NL	0.014258	0.00963	0.004694	0.019482	0.019014	0.028712	0.028944	0.10605
CEP350	7.46324	6.84263	6.214169	6.182082	7.107519	6.607406	6.466724	7.318121
CEP41	5.139351	5.603079	4.874844	5.373206	5.328186	5.114641	5.342078	5.393294
CEP44	4.291598	5.297981	4.771975	4.69185	4.534897	4.032115	4.512743	5.214279
CEP55	2.23017	2.778353	1.801949	1.935492	1.643495	2.234662	2.566883	2.207805
CEP57	9.740354	10.75664	9.92828	10.29188	9.459702	9.607184	9.557971	9.328416
CEP57L1	6.617858	5.527231	5.495676	6.095199	5.880262	6.470335	5.995451	5.671974
CEP63	3.601232	3.99597	2.927344	3.527253	3.515942	4.316469	3.854175	3.441299
CEP68	5.574894	5.531861	4.348521	5.574464	5.616582	4.994356	4.85194	6.23173
CEP70	2.536718	3.477072	2.973502	2.337413	2.457097	2.918912	2.737044	3.724117
CEP72	2.663167	2.68762	2.097847	2.814089	1.917314	2.076229	2.555864	2.92179
CEP76	5.274064	5.632708	4.108808	4.323763	4.622729	4.852836	4.914512	5.066828
CEP78	4.838022	5.613884	4.487551	5.420308	5.237495	5.325425	4.963853	5.471028
CEP83	3.248664	3.229367	3.087447	2.917468	3.458703	3.24448	2.357814	3.117765
CEP85	2.802245	2.68392	2.64901	2.619279	2.463099	2.178031	2.601062	2.896144
CEP85L	1.18919	0.896302	0.950876	1.028892	1.267536	1.143455	0.835313	1.306281
CEP89	4.594591	4.512505	3.864747	3.035533	3.265284	3.341589	4.114861	3.881504
CEP95	10.28235	10.1237	8.752963	10.07624	8.40483	11.7108	9.209344	10.78344
CEP97	2.277231	2.168134	1.946944	2.068544	2.199125	2.170453	1.670884	2.754882
CEPT1	15.57462	16.10933	17.48578	16.79569	17.94699	18.23139	15.55367	16.0507
CER1	0	0	0	0	0	0	0	0
CERCAM	5.499422	5.652238	5.788194	6.295608	6.238309	5.041577	4.362706	6.003377
CERK	16.99825	14.0378	14.86278	16.82918	18.35138	14.73081	13.26283	15.62508
CERKL	0.027603	0.05593	0.095406	0.084859	0.096622	0.125061	0.056034	0.069989
CERS1	2.206489	2.357937	1.895428	2.013348	3.431081	2.328076	1.963473	2.115275
CERS2	73.97674	68.83126	70.93547	74.92169	78.31703	75.50571	70.6658	73.52772
CERS3	5.944601	6.303978	5.996539	5.584197	5.277266	6.710692	5.637614	5.290799
CERS4	19.64653	18.04643	20.1588	29.03676	19.73927	20.1137	14.21976	12.50152
CERS5	41.13781	51.62474	51.3867	63.20791	67.44382	65.49489	44.85134	35.27846
CERS6	0.45209	0.80061	0.972768	0.975543	1.010902	0.877052	1.01101	1.144451
CES2	1.571426	2.23788	1.727526	1.306511	1.245442	1.820978	1.294054	1.052507
CES3	1.964819	2.003448	2.516179	2.65001	3.156795	3.152424	1.505368	1.491378

CES4A	53.99148	43.75771	82.51057	45.91799	72.51783	65.28124	40.122	29.28973
CES5A	0.016169	0.016382	0	0	0	0.01628	0	0
CETN2	16.40283	19.43748	17.83679	17.19303	19.50931	19.15511	16.18716	15.49649
CETN3	30.7205	41.73597	39.81999	34.59786	33.73778	40.14427	35.41122	33.81381
CFAP100	0.062226	0.214344	0.110611	0.089274	0.074681	0.075181	0.164214	0.075734
CFAP126	0.734375	1.104019	0.842196	1.11671	1.172783	0.834797	1.045951	0.828923
CFAP161	0.057749	0.019502	0	0.019727	0	0	0	0.019524
CFAP20	18.45819	21.8463	19.35681	17.58956	18.61153	18.36718	20.09204	19.96755
CFAP206	0.169571	0.131374	0.137907	0.051109	0.089784	0.100429	0.212612	0.161868
CFAP221	0.020093	0.040713	0.009921	0.010295	0	0	0	0.010189
CFAP298	15.29024	16.3046	15.02489	13.9763	15.56502	15.27263	15.58111	16.62988
CFAP299	0.028914	0.146469	0.028554	0.148153	0.115673	0.087336	0	0
CFAP300	0.612008	0.806052	0.866281	1.003468	0.510073	0.657268	0.662601	0.579339
CFAP36	40.45493	51.73056	54.90075	48.99465	46.27684	57.48021	48.91817	44.30549
CFAP410	7.353086	6.855882	5.814683	6.348462	5.428311	6.4621	6.571175	5.306756
CFAP43	0.040048	0.031557	0.057126	0.01824	0.031153	0.035841	0.018066	0.013539
CFAP44	0.1243	0.088152	0.039894	0.076427	0.114992	0.065703	0.059928	0.081945
CFAP45	1.153752	0.952432	1.111243	0.715237	0.541336	1.061246	0.722875	1.170176
CFAP46	0.275013	0.354912	0.174591	0.288536	0.317616	0.267003	0.162831	0.225799
CFAP52	0	0	0	0	0.024459	0	0	0
CFAP54	0.126356	0.16784	0.11369	0.207177	0.219045	0.144181	0.213752	0.091132
CFAP57	0.132361	0.015473	0.110602	0.052169	0.040732	0.030753	0.031003	0.082612
CFAP58	0.047794	0.036316	0.064897	0.036733	0.017925	0.02406	0.133404	0.109067
CFAP61	0.226046	0.199652	0.14882	0.184128	0.139123	0.110877	0.141192	0.1646
CFAP65	0	0.009064	0	0	0	0	0	0.004537
CFAP69	2.179993	1.842325	2.200789	2.228462	2.03996	2.90116	2.201739	1.959276
CFAP70	0.336375	0.070998	0.186853	0.315981	0.259322	0.282227	0.184936	0.241657
CFAP73	0.025796	0	0	0	0	0	0.052367	0
CFAP74	0	0.010479	0	0.031799	0	0.020828	0	0.005245
CFAP77	1.193486	0.806099	1.140595	1.880602	0.808595	1.52466	1.224414	1.054285
CFAP97	6.823184	7.549127	6.450479	6.968874	7.402222	6.590239	8.496366	7.922123
CFAP97D2	0	0.011125	0	0	0	0	0	0.03341
CFAP99	0.090039	0.221535	0.228643	0.079088	0.244422	0.220159	0.169723	0.417465
CFB	5.247968	4.49359	5.528083	5.574576	9.255678	7.022367	7.927028	6.79929
CFD	5.564937	5.068619	4.426256	5.126885	5.99062	3.712302	6.385765	8.688666
CFHR5	0	0	0	0	0.042737	0.032268	0.010843	0.02167
CFI	1.674782	0.475608	1.678974	1.534239	2.25871	1.775645	1.545367	1.029469
CFL1	900.1401	816.9268	835.4896	806.1196	839.0356	775.6056	878.0767	816.1437
CFL2	6.505889	6.422984	9.058547	6.875092	8.140334	6.74545	7.783673	7.319192
CFLAR	4.586679	4.775954	2.905303	4.74777	4.494599	3.953299	4.490891	5.97943
CFP	2.092648	2.633576	2.211879	2.446051	3.00857	1.563747	2.655047	4.410691
CFTR	0.081754	0.124241	0.103803	0.101732	0.16937	0.094072	0.18967	0.136223
CGAS	0.049433	0.020033	0	0.030395	0.227423	0.019908	0.02007	0.030082
CGGBP1	24.9927	25.57369	26.0403	24.95215	26.18357	26.45961	25.05782	26.01822
CGN	5.573088	5.766129	5.928638	7.205071	5.326322	5.800727	5.716745	5.9034
CGNL1	13.50455	9.97031	12.25496	12.762	11.76224	10.8441	12.03318	14.30006
CGREF1	1.58103	1.25139	1.000227	1.898663	1.260046	1.094383	1.303855	1.503316
CGRRF1	3.207342	3.371137	4.057091	4.308525	4.012699	3.507428	4.169905	3.00933
CH25H	6.649069	4.619579	4.515899	4.430296	8.266413	7.356004	5.828514	5.237714
CHAC1	107.089	58.67111	155.0443	139.9646	164.1265	184.0373	121.9734	59.42521
CHAC2	1.297729	1.58217	2.291602	1.589087	2.221824	1.48376	1.339521	1.539289
CHAD	0.226371	0.294868	0.415165	0.414247	0.792401	0.439555	0.6893	0.426388
CHADL	0.070375	0.458348	0.277993	0.370894	0.110604	0.384646	0.438788	0.193737
CHAF1A	11.68896	12.04414	10.89277	9.560822	9.35391	10.61199	9.928379	9.462536
CHAF1B	6.240386	6.167813	5.452798	6.551209	5.816399	5.712831	5.737071	5.810071
CHAMP1	9.963801	10.53982	9.833173	10.7282	9.827323	10.11772	10.71246	10.57134
CHAT	0.005935	0.120257	0.11722	0.018246	0.284914	0.023902	0.018072	0.144466
CHCHD1	41.96062	45.22978	45.27546	39.20727	44.18695	47.3244	47.56747	40.68108

CHCHD10	16.65399	21.28945	19.58939	20.73001	25.42024	23.8348	21.06337	15.74142
CHCHD2	65.21688	71.67635	74.24798	71.09888	68.62943	69.05874	68.84787	62.35411
CHCHD3	51.08336	58.47505	56.89215	59.00025	55.071	58.79787	58.72892	52.42888
CHCHD4	7.656229	7.523584	8.387151	7.995934	8.9125	7.287301	8.62037	6.958995
CHCHD5	7.996904	9.26759	9.166126	8.479948	9.206259	8.350813	7.970871	8.53865
CHCHD6	5.280484	6.931002	5.368817	9.329798	6.607962	5.368937	6.732617	5.434802
CHCHD7	13.54105	16.19996	18.33737	16.54553	18.19531	18.65645	15.66441	14.96921
CHD1	8.788086	8.778806	7.777163	8.956015	9.944932	9.011071	8.611078	8.811419
CHD1L	4.040482	5.66335	5.391204	4.83736	4.256786	4.57495	4.658519	6.087322
CHD2	11.06933	11.19527	9.520494	10.24653	10.86088	9.57629	10.35754	11.3688
CHD3	28.79782	30.92914	24.61031	28.48498	28.3178	26.58246	29.59798	29.50671
CHD4	68.13254	64.68197	59.10724	61.9672	63.92131	63.48612	66.28913	64.28958
CHD5	0.33781	0.231088	0.225252	0.239662	0.421595	0.191861	0.313572	0.307479
CHD6	4.090265	4.53123	3.196992	3.542768	4.253278	3.543294	4.103118	4.468353
CHD7	8.383766	7.103217	7.219531	8.040214	8.06419	7.221838	7.269671	7.516817
CHD8	14.17268	14.00112	11.76936	12.82255	13.547	12.51584	13.43333	14.07614
CHD9	9.554945	10.06089	8.224302	9.176658	10.35642	8.828045	8.692121	10.91051
CHDH	0.113431	0.071825	0.046674	0.116241	0.089811	0.057103	0.081552	0.115046
CHEK1	4.278792	5.513478	5.444173	5.54576	4.117478	4.745954	4.969265	5.581084
CHEK2	4.276359	4.958795	4.290285	4.324165	3.993297	3.860169	3.972079	4.578832
CHERP	12.68129	12.38309	10.95872	12.12667	12.54394	11.91439	11.93347	12.7561
CHFR	17.67462	18.01223	18.19239	17.45347	16.29508	19.10771	19.27155	18.81637
CHGA	0.198103	0.157695	0.097817	0.304516	0.127369	0.142469	0.28725	0.215275
CHGB	0	0	0	0	0	0	0	0
CHI3L1	25.2979	27.59911	22.28065	44.49459	24.17966	29.7309	21.2764	21.98826
CHI3L2	0.01677	0	0.066244	0.360894	0.083861	0.151961	0.170216	0.085044
CHIA	0.018081	0.091591	0.017856	0.203816	0.018083	0.091022	0	0.018338
CHIC1	0.832554	1.034929	0.900612	1.080504	1.199691	1.017471	0.792228	0.997177
CHIC2	6.731761	7.540008	6.825159	6.668558	7.331087	6.468985	7.12123	7.419316
CHID1	35.62813	35.37338	35.82793	33.33763	39.36103	35.42031	35.50091	33.49077
CHKA	2.440152	2.086993	1.82267	2.387254	2.56491	1.844356	1.747059	2.467878
CHKB	15.27198	16.83367	16.48333	15.10726	16.61776	16.71007	14.88639	15.41261
CHL1	8.476202	15.111	11.63181	14.61714	9.019604	14.84275	11.56158	14.0735
CHM	4.029611	4.629406	4.943289	4.146189	4.673644	4.12851	4.582633	4.407739
CHML	8.778093	7.125571	6.581474	7.861871	8.662291	7.932522	8.133811	7.635099
CHMP1A	38.41792	36.72953	42.01286	42.33157	41.38632	42.9067	42.01643	38.68493
CHMP1B	44.68004	42.61036	46.45432	47.10617	43.37015	48.22601	46.33508	43.6696
CHMP2A	116.6752	129.8757	133.7159	117.0725	126.627	122.7838	123.3932	113.2235
CHMP2B	25.7392	27.34338	31.31277	26.7397	27.61774	30.11011	27.74292	25.61963
CHMP4A	18.66589	17.98981	17.09618	18.21644	19.38408	18.69603	17.8268	16.83246
CHMP4B	86.90184	88.92188	96.36423	89.42808	87.01727	91.63866	90.1792	85.74396
CHMP4C	26.11195	27.10572	32.36684	36.6373	28.14501	27.55036	27.70521	23.97938
CHMP5	40.54357	44.35608	39.99915	40.0211	41.40309	44.78059	44.21642	41.48338
CHMP6	20.34309	17.69855	15.78961	19.29418	17.61089	18.13224	18.20863	17.29401
CHMP7	8.177904	6.324782	6.779295	5.618458	6.312776	6.702986	6.757368	7.149472
CHN1	2.275743	3.107561	1.862644	2.484208	2.195634	2.253932	2.121574	2.433582
CHN2	3.029664	2.855279	2.304817	2.635393	3.259072	1.897609	3.209198	2.84948
CHODL	2.572533	4.547985	1.689419	6.814722	2.675781	2.059136	2.154175	1.865546
CHORDC1	21.0926	17.43058	19.23822	14.6013	22.45598	19.22946	16.99548	23.57408
CHP1	31.37314	32.93112	35.59232	30.38503	34.03784	29.31949	32.95072	31.11679
CHP2	21.21183	32.63225	21.3548	24.0758	28.69398	32.31885	40.81352	32.94691
CHPF	5.845606	6.015296	5.446521	6.385375	6.387861	6.181203	6.426955	7.138785
CHPF2	2.584289	2.98431	2.249298	2.984951	2.891273	2.06171	2.500792	2.598866
CHPT1	3.828071	7.40582	8.643566	8.870866	5.5216	5.093772	6.248028	5.423887
CHRAC1	19.91697	20.59991	17.8608	22.05906	17.83881	19.60617	20.21572	21.07266
CHRD	2.348506	1.334123	1.963074	1.8222	1.954546	1.300503	1.719694	1.276039
CHRDL1	16.30622	18.42354	15.30586	20.70342	17.02026	16.86094	19.38873	16.49369
CHRDL2	0.72873	1.928603	0.308421	1.310676	0.922185	1.662073	1.29818	1.191616

CHRM1	0.043802	0.026626	0	0.035909	0.017523	0.00882	0.044459	0
CHRM2	0.020234	0.066625	0.059947	0.09331	0.146718	0.117142	0.066748	0.118004
CHRM3	0.287538	0.211863	0.174245	0.267873	0.222217	0.414514	0.205622	0.45733
CHRM4	0.018861	0.009554	0.009313	0.009664	0.018863	0.018989	0	0.019129
CHRM5	0.067672	0	0.026732	0.041609	0.027072	0.013627	0.109899	0.123544
CHRNA1	0	0.063485	0	0	0	0	0	0
CHRNA2	0	0	0	0	0	0	0.009027	0.045103
CHRNA3	0.010807	0.021897	0.021344	0.022149	0	0	0.021937	0.01096
CHRNA4	0	0	0	0	0	0	0	0
CHRNA5	0	0.073462	0.014321	0.044584	0.116032	0.014601	0.147197	0.014709
CHRNA6	0	0	0	0	0	0	0.019131	0
CHRNA7	1.051898	0.054933	0.428368	0.088903	0.281989	0.731532	0.077049	0.076991
CHRNA9	0.016493	0	0	0.101411	0	0	0	0
CHRNB1	3.033205	2.914064	2.90245	2.658166	2.636092	2.83278	2.802682	2.397459
CHRNB2	0.005306	0	0	0.016312	0	0	0	0.005382
CHRNB3	0	0	0	0	0	0	0	0
CHRNB4	0.022961	0.023262	0.022675	0.011765	0.057409	0.011559	0.023305	0.011644
CHRND	0.299495	0.427554	0.645302	0.23716	0.503761	0.068532	0.262535	0.24853
CHRNE	6.421427	7.826656	6.395065	8.584698	8.486942	7.362341	5.999417	5.234516
CHRNG	0	0	0	0.015338	0	0	0	0
CHST1	7.825686	8.083148	8.377993	10.2231	8.402535	7.133823	9.243063	10.21334
CHST10	13.1494	12.49558	11.40361	13.01185	12.10924	10.58743	13.21697	13.19705
CHST11	0.62103	0.36883	0.401812	0.460851	1.15655	0.474344	0.717289	1.020824
CHST12	4.160691	5.125298	6.831087	4.898555	7.733897	6.236896	5.74258	7.036692
CHST13	0.128317	0.162501	0.142558	0.098622	0.304792	0.113044	0.113962	0.24402
CHST14	3.629421	4.9071	3.507667	4.169348	3.22944	4.655549	5.034175	3.995487
CHST15	2.402982	3.323067	2.307957	3.288535	3.812819	2.985966	2.536794	2.498891
CHST2	6.520067	5.983148	6.650901	5.53843	7.714124	5.988029	5.667269	7.127153
CHST3	4.986339	2.900096	2.573558	4.289753	3.386224	2.871754	2.978363	5.421534
CHST4	0	0	0.012496	0.012967	0	0.01274	0.012843	0.012833
CHST7	1.867453	1.914895	2.47009	1.844121	2.320469	1.789049	2.389435	2.651653
CHST8	0.331326	0.287721	0.37394	0.565889	0.142015	0.555983	0.512451	0.336043
CHST9	0	0.036779	0.017925	0.037202	0	0.018275	0	0.036819
CHSY1	2.85529	4.428152	3.441496	4.786663	2.687249	4.51859	3.002155	3.601358
CHSY3	0.256967	0.280893	0.153595	0.214824	0.243474	0.245106	0.411824	0.377221
CHTF18	2.826595	2.379328	1.722867	1.839391	1.979711	1.866306	1.787801	2.262842
CHTOP	35.8862	31.66169	31.79543	30.15506	37.34097	26.73356	33.65192	38.78677
CHUK	10.5419	11.41711	10.2088	10.81978	10.76394	10.12848	10.6005	9.91287
CHURC1	49.63385	53.03137	53.58456	52.01304	52.03341	48.23352	50.91248	45.32551
CIAO1	16.03731	15.64792	14.63463	14.99567	16.91918	16.45345	16.8445	16.70313
CIAO2A	34.20002	34.6761	34.78285	33.66952	33.7473	35.81425	36.37771	31.14183
CIAO2B	44.77414	45.05963	38.28429	42.21605	43.50172	40.49052	42.93781	38.58468
CIAO3	8.614089	9.035947	7.983264	9.316369	8.814015	9.153284	7.277117	8.454453
CIAPIN1	20.44599	21.57622	21.64746	21.01056	19.03055	20.10029	20.43606	20.2481
CIART	8.103157	8.169843	8.36944	9.948604	8.025903	10.36565	9.555755	9.310341
CIB1	23.54277	24.71103	25.35608	23.49381	24.23985	22.84965	27.54812	24.55567
CIB2	20.54696	19.4585	18.14544	21.81383	23.30035	22.33952	31.41177	25.64496
CIB3	0	0	0.041132	0.042682	0.041656	0.041935	0	0
CIB4	0.039255	0	0	0.040228	0	0.039524	0	0
CIC	7.612338	6.61864	5.454183	6.708297	7.788835	6.367504	6.809019	6.68971
CIDEA	10.98821	9.849963	12.24026	11.74684	12.58972	12.55018	10.68907	6.169669
CIDEB	0.779559	0.470836	0.666212	0.86032	0.704692	0.573569	0.441275	0.532173
CIDEC	0.703297	6.330128	0.372431	1.535442	1.743178	0.656789	0.620736	0.816686
CIITA	0.624364	0.990369	0.678866	1.05992	1.311965	1.015967	0.716946	0.959473
CILP	6.731882	6.540459	7.211597	6.640799	7.585854	6.005552	10.00949	18.24251
CILP2	0.163361	0.140986	0.053775	0.093004	0.187587	0.079193	0.079835	0.073638
CINP	24.51351	22.18961	22.50097	22.63314	23.15566	20.45947	21.48407	18.11062
CIP2A	2.995629	3.657108	2.529349	3.407514	3.085897	2.5486	3.283809	3.666184

CIPC	6.720183	6.813414	7.593628	6.545368	7.645693	7.186471	6.361881	6.830973
CIR1	18.59065	29.03203	26.87219	28.38962	26.38911	32.01661	24.09045	26.76132
CIRBP	61.6117	75.01023	55.09505	62.65587	46.96474	65.4315	78.50135	57.25223
CISD1	20.18166	19.64433	23.6762	20.43626	19.77911	22.91509	23.15707	20.17014
CISD2	19.07931	17.65947	21.09159	20.63391	21.52721	24.7301	20.28677	16.25101
CISD3	8.950252	11.73235	11.90127	9.896054	16.21461	14.14947	12.79003	13.69613
CISH	12.19134	13.75555	12.221	11.11645	14.1732	12.28886	9.790268	11.46119
CIT	2.291754	2.418323	1.357919	1.689716	1.937824	1.980777	1.761213	2.113072
CITED1	0.227051	0.376414	0.244605	0.359588	0.578022	0.31173	0.565666	0.544305
CITED2	63.47411	62.32443	57.79691	57.04329	60.10324	57.94078	66.22521	59.95852
CITED4	53.43758	42.86688	60.21806	56.08409	57.11265	64.61269	49.49738	46.01037
CIZ1	13.55576	11.94568	12.11506	11.8386	12.46032	12.79354	12.08399	12.51996
CKAP2	15.90192	14.19182	14.31867	14.65708	12.97335	13.98675	14.48106	14.75105
CKAP2L	4.849824	6.020111	4.090402	3.680454	4.29112	4.566224	4.727442	4.741608
CKAP4	19.03625	18.69971	15.70904	16.49589	18.10712	18.21026	20.53342	18.36252
CKAP5	24.64816	23.47611	19.56348	21.26291	20.10237	19.88738	21.95832	22.877
CKB	60.86082	52.82092	56.60772	54.84382	68.51513	48.8536	55.74234	59.42396
CKM	0.288362	0.365183	0.231375	0.535603	0.793099	1.506099	0.347567	0.347305
CKMT2	0.500765	0.253668	0.10597	0.568151	0.304075	0.090033	0.59904	0.362781
CKS1B	28.90629	30.83425	32.37015	32.44182	29.01194	33.1051	27.92632	34.55433
CKS2	46.95224	48.91076	44.44546	37.93214	40.58125	43.06261	44.71476	45.85691
CLASP1	16.60497	14.43904	13.91688	12.45012	13.80784	13.96608	14.32226	14.60197
CLASP2	15.38834	15.04779	15.31662	14.25561	14.54333	14.42285	16.27832	16.98249
CLASRP	6.349801	5.768584	4.487644	6.333842	5.417303	5.467224	5.388033	6.069832
CLBA1	1.628999	1.387818	1.413707	1.947575	1.987142	1.553151	1.5407	1.476957
CLCA1	0.009408	0	0	0	0	0	0	0.009542
CLCA2	18.40394	21.72163	17.83656	22.36027	24.73279	21.40689	18.54403	21.67754
CLCA4	0	0.008959	0	0	0.008844	0.017808	0.035904	0
CLCC1	15.30681	15.77963	14.87311	14.31067	14.79427	15.41888	15.77857	14.70802
CLCF1	0.81654	0.444267	0.22399	0.743792	0.877137	0.24359	0.184175	0.383409
CLCN1	0	0.026651	0	0	0.00877	0	0.0178	0
CLCN2	0.413633	0.232812	0.447382	0.477705	0.505617	0.43629	0.219915	0.49943
CLCN3	11.034	11.78171	11.87108	13.03517	10.62377	11.71353	12.57868	11.90529
CLCN4	2.227993	2.111292	2.351972	2.627627	2.132235	2.736334	2.261413	1.831144
CLCN5	2.582431	2.394295	2.146658	2.555352	2.772325	2.313826	2.783487	2.52608
CLCN6	3.856348	3.897406	3.314801	3.676496	3.583378	3.332091	3.928548	3.672172
CLCN7	11.33718	11.3149	9.004375	12.60677	10.67119	10.7508	10.21777	10.84617
CLCNKA	0.039942	0.020233	0	0.010233	0.009987	0	0.050676	0
CLDN1	340.4908	288.1573	272.7101	280.7815	266.9438	256.2819	316.3405	324.8953
CLDN10	10.10077	13.71736	13.58416	9.940094	10.44139	9.936881	9.469667	12.2622
CLDN11	1.027809	0.845111	0.779639	1.205915	1.608952	1.03483	0.710603	1.510783
CLDN12	13.94635	13.5919	20.33738	13.17153	13.12011	16.68809	14.00869	12.66925
CLDN14	1.666982	1.961256	1.274486	1.157218	1.667197	1.461806	1.582826	1.772521
CLDN15	0.210362	0.695452	0.196807	0.192881	0.531509	0.12262	0.179804	0.190898
CLDN16	0.462206	0.273159	0.088754	0.631541	0.937373	0.581705	0.847057	1.015703
CLDN17	0.115764	0.117283	0.26675	0.342713	0.463116	0.453269	0.261112	0.534877
CLDN18	0	0.00775	0	0	0	0	0	0
CLDN19	0.2763	0.64383	0.163714	0.226515	0.055267	0.083456	0.084133	0.25221
CLDN2	0	0.009771	0	0	0.019292	0.009711	0	0.009782
CLDN20	0.074143	0.122064	0.146439	0.465378	0.120499	0.2986	0.141104	0.291396
CLDN23	1.595536	1.168537	1.69905	1.812354	1.42271	1.490308	1.248747	1.364789
CLDN25	0	0	0.07977	0.082778	0.040394	0	0	0
CLDN3	2.385817	4.744733	3.75229	4.233324	1.966344	4.114735	4.932897	1.837241
CLDN4	85.44262	79.01388	79.32667	86.49894	80.52954	85.38981	83.61308	64.89706
CLDN5	7.974469	8.963056	7.90957	11.14038	13.6299	7.97624	12.41565	11.87536
CLDN6	0.119527	0.08073	0.059019	0.040829	0	0	0.14154	0.020205
CLDN7	2.068382	2.987241	4.215598	2.886286	1.804566	2.614217	4.154147	2.276365
CLDN8	24.20898	30.25137	30.31991	25.55102	22.05173	26.77467	25.14698	22.75016

CLDND1	24.26588	27.80561	25.22859	22.86475	23.90954	27.25247	28.89543	22.72317
CLDND2	1.570349	1.871716	1.09467	1.845901	1.755323	0.465023	0.750073	1.21795
CLEC11A	2.938729	3.671999	3.095586	3.011522	3.446773	3.299516	3.082237	3.657964
CLEC12A	0.689494	0.43659	0.446843	0.927378	1.292969	0.564041	0.546748	1.311206
CLEC14A	8.335061	10.33391	7.111988	9.12958	11.80833	10.91971	11.96935	11.55293
CLEC16A	12.33601	11.19074	12.0141	10.05392	11.64769	12.1459	11.18985	11.38441
CLEC17A	0.115521	0.100318	0.048892	0.067647	0	0.083079	0.033501	0.08369
CLEC19A	0.042152	0.042706	0.041627	0	0.042158	0.212202	0.042785	0.085505
CLEC1A	2.608983	2.833535	2.205462	3.443607	3.214682	3.257239	2.393898	3.598727
CLEC1B	0	0	0	0	0	0	0	0
CLEC2L	0	0.04769	0.018594	0.009648	0.009416	0.047394	0.009556	0.019097
CLEC3A	0	0.02453	0	0.024812	0.121076	0	0.049151	0
CLEC3B	65.89178	70.21465	80.01944	82.45829	93.00253	87.82833	90.67985	76.58332
CLEC4A	1.790752	2.435712	2.393744	2.149411	2.236256	1.882676	2.600895	3.391662
CLEC4D	0.218665	0.243689	0.172753	0.313714	0.021869	0.11008	0.133167	0.155245
CLEC4E	0.04634	0.11737	0	0.047488	0.023173	0.163297	0.164622	0.070499
CLEC4F	0.307867	0.067806	0.251156	0.178322	0.080324	0.053908	0.095104	0.162913
CLEC5A	0.552333	0.702731	0.64693	0.908256	0.411092	0.737164	0.795296	0.938003
CLEC6A	0.576321	0.355408	0.51965	0.462206	0.426032	0.302743	0.254333	0.584526
CLEC7A	0.971141	1.027941	0.687074	1.574491	1.261197	1.079931	0.750315	2.058138
CLEC9A	0.087878	0.111289	0.15187	0.090055	0.285641	0.066359	0.178392	0
CLGN	0.351069	0.530028	0.448664	0.592555	0.419961	0.353468	0.656775	0.425884
CLHC1	0.204599	0.456025	0.39063	1.607448	0.586593	0.865187	0.60916	0.428858
CLIC1	170.0862	176.653	189.305	189.657	186.1484	194.0379	173.1712	182.9558
CLIC2	11.47859	12.79625	16.04541	14.76577	15.94679	13.16441	12.36868	15.53632
CLIC3	34.24609	29.07838	34.08325	19.98874	30.39789	36.29596	33.72015	33.71734
CLIC4	109.1942	107.2549	120.0196	111.3896	117.0058	123.3715	110.3851	105.3111
CLIC5	3.21273	2.149291	3.017513	2.469233	2.291985	2.680915	2.051367	2.771466
CLIC6	0.021081	0.028478	0.159611	0.064811	1.012041	0.049526	0.199712	0.035636
CLINT1	41.18961	42.33771	41.7247	41.23001	42.7591	40.51643	41.69231	42.95948
CLIP1	20.90649	19.36419	17.80117	19.73416	19.81698	19.07198	19.41829	18.64247
CLIP2	9.450357	8.169727	6.866058	8.11216	8.054743	6.800036	8.852268	7.465253
CLIP3	4.576373	4.443492	4.792905	3.796731	4.184488	3.718647	4.604016	5.648256
CLIP4	7.560511	5.944676	6.567584	7.60431	7.529664	7.554493	5.942765	7.868234
CLK1	168.5165	156.3716	124.2534	149.6381	159.891	151.8412	157.3756	155.5033
CLK2	26.31113	24.47823	21.64626	24.1685	22.27622	23.62664	24.63001	23.3882
CLK3	36.15687	35.1136	30.073	33.61296	33.3954	34.48941	30.67529	33.77877
CLK4	21.85358	20.92836	17.46972	22.71656	20.6777	19.16456	20.28785	22.51307
CLMN	0.72714	1.469508	0.695522	1.174296	0.929031	0.866263	1.340849	0.980747
CLMP	3.840857	5.25467	5.58398	5.403857	4.372789	5.222034	5.481308	4.545766
CLN3	6.221643	6.136542	5.827162	7.320312	6.790369	6.902161	5.54648	6.218397
CLN5	15.13875	11.53386	13.11401	11.84233	13.19537	15.45097	14.65452	14.48392
CLN6	8.378731	10.61847	6.791475	9.848057	8.349778	9.191882	8.230076	8.43709
CLN8	17.05671	13.66339	17.42324	19.48218	20.12144	17.50122	13.26547	16.08367
CLNK	0.011495	0.058227	0.022703	0.011779	0.022992	0.069438	0.046668	0.058291
CLNS1A	31.69143	30.52517	31.86824	29.05497	26.28279	30.72867	30.97797	30.5177
CLOCK	3.234224	3.312819	2.874281	3.092337	3.945805	3.276847	3.349996	3.414681
CLP1	8.132701	7.03027	5.580235	7.154381	7.256997	6.869631	6.325023	7.241518
CLPB	4.602195	4.058183	4.203244	4.495282	4.893598	4.785061	4.437157	4.459239
CLPP	22.38099	21.83686	21.89795	24.52444	21.94448	23.36655	23.9496	20.99582
CLPS	0	0	0	0.068618	0	0	0	0
CLPSL2	0	0.157828	0	0	0	0	0.052707	0.052667
CLPTM1	31.96829	28.79046	29.2434	32.42516	32.15281	29.98492	31.55539	29.89674
CLPTM1L	38.16166	36.57127	34.7212	39.30935	37.48504	38.82011	36.48863	38.23412
CLPX	21.88122	22.89373	23.12574	23.00406	22.45837	25.33455	23.23134	21.30773
CLRN1	0	0	0	0	0	0	0	0
CLRN2	0	0	0	0	0	0	0.02035	0.04067
CLRN3	0.011875	0.024062	0.070362	0.048676	0.011876	0.011956	0	0

CLSPN	4.040211	3.849968	3.247568	3.284415	3.320293	2.985165	3.872992	3.377712
CLSTN1	72.44438	66.86789	68.20931	68.85969	70.39132	64.95516	70.91785	73.63412
CLSTN2	0.064451	0.146422	0.148511	0.15411	0.119151	0.175009	0.192288	0.229779
CLSTN3	2.66462	4.427328	3.122616	5.34474	4.782722	4.027812	2.488222	2.796241
CLTA	125.088	134.2684	136.7372	135.9115	135.1728	132.553	128.618	121.9113
CLTB	77.74507	80.54957	88.39368	79.21366	83.10944	84.35199	79.34024	79.82824
CLTC	69.19912	69.67052	66.16088	64.40716	68.44891	63.18325	68.62891	70.49066
CLTRN	3.108392	2.651947	2.504197	1.927998	2.29069	2.347221	2.795235	2.461273
CLUAP1	5.304096	5.620121	4.850006	5.445067	5.707085	5.575796	6.485076	5.550385
CLUH	13.11453	12.948	10.62498	13.73911	13.76917	12.67302	12.73342	12.88275
CLUL1	0.140146	0.263687	0.1384	0.123101	0.040047	0.060473	0.121927	0.182753
CLVS1	0	0	0.011672	0.012113	0	0.023801	0	0
CLVS2	0.012316	0.079022	0.028378	0.075724	0.075956	0.066132	0.045834	0.122827
CLYBL	0.958419	1.131889	0.905206	1.024986	0.989193	0.662012	1.012385	0.8336
CMAS	15.44123	23.08962	20.73413	22.08661	19.32724	18.28842	17.49454	20.41585
CMBL	16.66389	17.46097	18.86375	18.32579	17.0827	16.59131	15.00321	14.92932
CMC1	4.867681	5.496639	6.709811	7.014733	3.90479	8.576635	4.88923	5.554097
CMC2	18.50571	20.47158	27.04263	24.84238	43.47386	27.30971	23.72781	18.62297
CMC4	9.883498	9.128851	8.648057	8.396979	7.857129	8.845349	8.345502	7.625243
CMIP	25.1989	20.70857	19.86521	21.62268	23.60133	21.73417	22.16297	22.42051
CMKLR1	5.887062	2.561083	4.914101	4.002476	3.496251	2.476388	4.704013	5.023844
CMPK1	20.74438	24.31425	26.29246	22.08827	26.08759	21.94324	24.55719	22.74356
CMPK2	3.650391	3.294066	3.244416	4.053997	3.404642	3.880464	3.989496	2.875784
CMSS1	3.422119	4.246317	4.635157	4.005571	3.438261	4.58346	3.951448	3.66189
CMTM2	0	0	0	0	0.034817	0	0	0
CMTM3	17.29149	17.86101	22.96537	21.83081	25.05669	22.42359	21.88788	22.77556
CMTM4	2.528054	3.0003	1.640592	2.331607	1.914345	1.963536	2.675945	2.966963
CMTM5	0.264882	0.178905	0.348775	0.271443	0.470962	0.592647	0.3286	0.447754
CMTM6	68.90698	66.5666	70.86191	64.09006	68.6113	68.43805	69.16281	68.52284
CMTM7	8.752709	8.077155	6.910104	6.895784	12.4602	7.658777	8.042618	8.679486
CMTM8	14.14875	13.08494	10.55546	13.16515	12.30038	11.17557	10.57079	10.11113
CMTR1	11.14804	11.03849	9.220597	9.952719	9.814433	9.843879	11.05893	10.50173
CMTR2	4.355513	4.502577	4.038349	3.341867	3.816189	3.64074	4.120619	3.645014
CMYA5	1.950457	1.731526	1.159557	1.668984	1.487497	1.306998	1.584654	1.925294
CNBD1	0	0	0	0	0	0	0.015836	0
CNBP	218.6984	242.5851	234.8783	248.401	233.4327	246.761	234.0058	214.4804
CNDP1	1.336755	0.739977	0.653245	1.186276	1.9985	0.888008	0.629446	1.174081
CNDP2	63.34711	62.47709	67.05062	61.08844	55.00836	72.09052	75.42332	83.23247
CNEP1R1	12.60829	13.10331	12.95597	13.46028	14.252	12.91276	13.2219	12.22222
CNFN	4.063508	3.974878	3.943689	4.056469	5.850807	4.443964	2.737789	4.121355
CNGA1	0.045922	0.011631	0.011337	0.011765	0.011482	0	0.011653	0
CNGA2	0	0	0	0	0	0	0	0
CNGA3	0.072323	0.014655	0.014284	0.044469	0.079566	0.12379	0.014682	0.051347
CNGA4	0.032395	0.01641	0.031991	0.016599	0	0.016308	0	0.032856
CNGB1	0.084963	0	0.023973	0.012438	0.024278	0	0.01232	0.01231
CNGB3	0	0	0	0	0	0	0	0
CNIH1	26.88119	28.13199	32.85876	26.41162	29.40776	28.16321	28.4263	28.38761
CNIH2	0.26749	0.714456	0.360214	0.62299	0.680971	0.367251	0.543004	0.197307
CNIH3	0.219093	0.221969	0.270454	0.11226	0.109561	0.275738	0.333569	0.111106
CNIH4	29.28307	29.71912	29.33852	30.72374	29.96794	31.35152	27.75235	26.55726
CNKSR1	4.342633	3.535772	3.103794	4.125073	3.827564	3.71346	3.1901	3.44915
CNKSR2	0.328891	0.398154	0.338555	0.311331	0.270394	0.258175	0.265928	0.313785
CNKSR3	16.63413	17.55883	14.33439	15.74705	16.98947	17.17626	19.40194	19.04752
CNMD	0.039091	0.039604	0.09651	0.080119	0.175932	0	0.059516	0.019824
CNN1	39.59182	58.90121	66.17482	63.56605	50.98685	45.14611	59.31829	46.02124
CNN2	118.9216	90.19395	81.93181	99.49437	95.64739	90.9354	102.7284	100.3073
CNN3	126.5935	133.8461	138.4251	131.7687	143.733	150.2243	136.2858	126.2912
CNNM1	0.046927	0.02113	0.025746	0.005343	0.015644	0.020999	0.026461	0.042306

CNNM2	1.024384	0.999991	0.835114	1.008752	1.115229	0.966921	1.026212	1.082257
CNNM3	13.2745	13.13153	11.6436	12.07615	11.8985	12.23671	12.04364	13.10148
CNNM4	3.176702	3.337814	2.397947	3.098359	2.953124	2.551603	3.439709	3.281701
CNOT1	39.908	34.69664	33.63817	32.37673	36.01813	32.31651	35.54707	37.20895
CNOT10	13.56909	15.34832	12.03323	11.88315	11.23265	11.59039	12.70005	12.88966
CNOT11	12.48729	11.04563	11.81395	12.00292	12.46507	10.52115	11.10237	10.58644
CNOT2	25.44221	27.69059	23.818	27.06405	25.97504	26.43404	25.481	27.21174
CNOT3	7.327293	6.277782	5.488229	7.202926	6.844736	5.840931	6.122998	7.286636
CNOT4	1.777878	1.660334	1.409975	1.666698	1.934562	1.590021	1.297963	1.710006
CNOT6	11.92404	12.65263	12.12703	10.73915	11.44266	12.36393	11.38021	12.45901
CNOT6L	16.16245	18.55367	17.54191	13.00956	15.1106	15.99906	14.90869	16.63701
CNOT7	40.2542	47.89833	45.05367	42.05105	43.83497	42.8227	45.20392	46.77431
CNOT8	13.74401	15.63118	13.60435	14.45596	14.28965	14.11703	15.78999	15.11843
CNOT9	17.08034	19.25071	17.14221	18.66142	18.12576	18.48352	18.35998	16.34491
CNP	12.93578	12.77908	13.57017	14.25868	14.91721	11.8886	11.97337	11.89431
CNPPD1	31.5369	30.15364	35.07538	35.0656	31.60869	32.43401	33.26066	31.54636
CNPY1	0	0.011756	0	0.047566	0.011606	0	0.011778	0.011769
CNPY2	44.292	52.94459	48.79406	53.33695	50.65612	58.96365	57.62127	44.44092
CNPY3	7.089413	7.304191	6.708378	9.350123	7.76331	7.153976	7.390894	7.620943
CNPY4	23.73405	29.98508	37.16515	28.54681	28.37106	32.69304	32.76633	26.33526
CNR1	0.063203	0.226576	0.139234	0.099644	0.20422	0.044055	0.118433	0.10355
CNR2	0.09689	0.441728	0.31097	0.148935	0.230144	0.097552	0.368788	0.651036
CNRIP1	7.026223	8.223886	7.869273	8.165947	8.002662	6.341832	6.644988	6.925034
CNST	6.906899	6.133418	7.301501	6.949726	7.274044	6.277344	6.615279	7.320223
CNTD1	0	0	0	0	0	0	0.043373	0
CNTD2	0.086411	0.103462	0.023273	0.064401	0.054996	0.055364	0.015947	0.071706
CNTF	0	0.021646	0	0	0	0	0.021686	0
CNTFR	4.997016	3.018005	3.06195	3.486955	3.285617	3.70472	3.206712	4.612827
CNTLN	0.669913	0.766114	0.481139	1.040162	0.857802	0.689819	0.762381	0.741218
CNTN1	1.588968	3.271335	2.594888	1.904695	2.41292	2.396066	2.167666	2.173426
CNTN2	0.147803	0.248257	0.053775	0.13552	0.171162	0.144896	0.035531	0.019725
CNTN3	0.25057	0.037809	0.100032	0.076487	0.047988	0.059045	0.102814	0.091922
CNTN4	0.576362	0.728267	0.383717	0.809639	0.498715	0.378173	0.368094	0.341545
CNTN5	0.101807	0.142814	0.038669	0.184582	0.10182	0.134042	0.111283	0.095314
CNTNAP1	1.102345	1.399611	0.995167	1.396307	1.476293	1.205144	1.51265	1.713045
CNTNAP2	0.066101	0.035079	0.239351	0.351596	0.13222	0.15529	0.201278	0.197934
CNTNAP4	0.110298	0.138564	0.121994	0.343611	0.242686	0.253197	0.197036	0.246109
CNTNAP5	0	0.002538	0	0	0	0	0	0
CNTRL	6.707016	7.331676	5.203146	7.290142	5.978155	6.812876	6.280672	7.560304
CNTROB	6.356567	5.354434	4.804542	5.223087	4.764421	4.78906	5.437833	5.23548
COASY	6.710089	6.136853	6.36864	7.157238	6.332323	6.309039	6.81074	6.130346
COBLL1	10.59905	11.93445	11.83952	11.54478	10.80949	11.43939	12.20887	12.19395
COCH	15.15695	24.50584	45.79709	38.45757	28.86929	62.31448	28.94272	42.25394
COG1	21.15069	21.89205	20.40741	23.0149	21.13474	22.56499	23.00409	21.18648
COG2	11.1239	10.66816	9.280679	11.27264	10.06725	11.53003	10.28602	8.778573
COG3	11.0217	9.712335	9.362769	9.98952	10.54467	9.320479	9.232137	10.53587
COG4	12.85933	13.49861	11.98567	13.32592	12.73713	13.69533	14.0369	13.55529
COG5	11.01504	10.69782	10.84445	10.05962	10.99958	11.55769	10.8033	11.64269
COG6	7.673984	9.313591	7.564146	8.085386	8.89174	8.24102	9.652338	8.22861
COG7	7.986704	8.980245	7.212295	9.083478	7.824513	8.020726	8.634514	7.81073
COG8	10.31747	10.31641	8.366613	9.247956	9.860788	10.13029	9.610938	9.835937
COIL	9.383114	11.70987	12.05522	12.95031	11.39947	11.36552	12.63858	9.52524
COL10A1	0	0	0.009006	0	0	0	0	0.00925
COL11A1	4.368847	5.157187	1.977112	3.489835	4.524046	3.656264	5.919213	4.246079
COL11A2	0.043774	0.093132	0.03026	0.071773	0.100693	0.083739	0.053317	0.048837
COL12A1	59.50566	38.80568	45.66096	46.76096	42.37941	32.21344	49.79239	73.40837
COL13A1	0.406141	0.29766	0.085336	0.230239	0.406194	0.469818	0.298211	0.368101
COL14A1	12.13667	16.34749	10.49092	13.81922	11.8296	11.8578	14.48984	17.91322

COL15A1	20.44678	19.63908	17.78006	20.5457	26.24733	19.22031	21.04318	20.53854
COL16A1	22.52988	19.89249	15.76535	24.41241	20.83172	19.9251	23.98551	23.63711
COL17A1	96.43828	102.2737	95.5136	97.17934	79.08849	78.46549	89.55857	98.39426
COL18A1	35.51343	27.39241	25.75001	32.01619	34.38924	32.73692	32.87335	32.87104
COL1A1	133.3293	147.3551	306.123	172.6425	215.9247	178.2106	239.7772	350.1922
COL1A2	180.5227	232.883	333.6324	247.3957	275.0125	244.2155	342.7537	435.2538
COL20A1	0.087319	0.115685	0.039799	0.068832	0.047024	0.033814	0.136352	0.040875
COL21A1	1.550937	4.221183	2.25848	3.825227	2.589612	2.679749	3.255129	3.026056
COL22A1	0.032497	0.103474	0.032092	0.114178	0.120719	0.233707	0.056545	0.023543
COL23A1	0.557755	2.044544	0.701025	0.935298	1.196793	1.419229	0.833742	1.213673
COL24A1	0.134211	0.11125	0.208849	0.325085	0.174904	0.315299	0.181633	0.152622
COL26A1	0.515489	0.425541	0.999278	1.252168	1.069301	0.788128	1.298365	1.123111
COL27A1	1.779658	2.370983	1.456519	2.008814	2.336808	1.924402	1.699423	2.201862
COL28A1	2.475604	1.052292	0.254502	0.096035	1.679255	0.377416	0.760955	3.89696
COL2A1	0.047289	0.005989	0.005837	0.036345	0.035471	0.017855	0.023999	0.035972
COL3A1	137.8493	176.1286	277.8952	180.584	196.878	178.9225	256.7528	394.0999
COL4A1	35.72755	29.08715	30.24891	26.98219	29.91767	26.83506	28.656	36.03422
COL4A2	23.91625	21.29422	20.60816	20.55667	21.69706	19.75651	20.19927	25.09454
COL4A3	0.276836	0.473519	0.213028	0.386854	0.34519	0.340264	0.353972	0.444867
COL4A3BP	13.97403	13.33505	13.96227	14.57815	13.81656	14.53503	12.64013	13.14646
COL4A4	0.223247	0.429353	0.35125	0.414901	0.295179	0.415258	0.368699	0.422149
COL4A5	11.6763	10.60788	10.98992	10.54272	9.720188	9.58013	10.93781	12.19995
COL4A6	4.317157	2.932167	2.675519	3.390158	3.469449	2.638748	3.296636	4.366384
COL5A1	10.38533	13.02565	13.78407	13.09017	15.74636	11.11273	14.55565	20.88078
COL5A2	34.37762	30.71807	27.78643	30.87064	32.47702	25.53135	34.21793	45.51456
COL5A3	1.95034	3.430696	1.961378	2.700012	2.59035	1.477251	2.438171	3.620475
COL6A1	88.19841	94.6889	106.3485	81.70443	97.11146	80.9509	102.2896	122.0036
COL6A2	62.45665	64.63508	71.92842	63.08947	68.93806	55.45663	73.09991	81.70309
COL6A3	26.88191	20.75824	25.91592	25.55945	29.10794	21.32738	26.35297	34.64291
COL6A5	0.126648	0.083401	0.050028	0.308239	0.129831	0.054193	0.257095	0.089916
COL6A6	0.126277	0.055323	0.033704	0.045467	0.160427	0.079033	0.013856	0.048461
COL7A1	4.175313	4.439156	2.412243	3.355202	3.292733	2.997067	3.332452	4.247062
COL8A1	3.268661	2.53446	2.470458	2.219385	3.534822	2.342055	1.97942	3.005029
COL8A2	8.784832	7.084523	7.318163	7.091512	8.561928	6.607728	9.156265	8.111622
COL9A1	0.212899	0.050328	0.238279	0.145449	0.127756	0.185773	0.252108	0.079174
COL9A2	0	0	0	0	0	0	0	0
COL9A3	0.043784	0	0.021619	0.011217	0.054738	0.033063	0	0.022204
COLCA2	0.024296	0	0.023993	0.049796	0.048599	0.097849	0.049321	0.024642
COLEC10	0.234079	0.067757	0.033023	0.051402	0.016722	0.067337	0	0.067832
COLEC11	0.021781	0.132404	0.12906	0.133926	0.15249	0.043861	0.088433	0.110458
COLEC12	25.31384	17.45582	18.14934	19.76483	20.09664	15.93472	22.93381	22.11284
COLGALT1	11.80107	10.52479	9.886431	10.6278	13.1452	9.84988	10.7313	13.09923
COLGALT2	1.45719	1.10098	1.060982	0.942794	0.46315	1.174959	0.877401	1.553083
COLQ	0.197767	0.231998	0.431721	0.127999	0.270663	0.146718	0.147909	0.422278
COMMD1	29.92575	35.87133	37.92438	32.38947	29.05256	33.40441	33.82377	29.09174
COMMD1(	11.4082	13.52119	14.44524	13.21219	12.69131	12.76063	13.94278	11.84006
COMMD2	13.88834	15.372	15.24133	15.01425	16.06349	15.40294	16.24352	16.07456
COMMD3	34.39597	39.90249	40.03195	39.75608	40.39465	41.70576	36.23047	40.45534
COMMD4	14.61088	17.57433	19.23844	16.26675	17.31884	17.70732	16.35577	15.55068
COMMD5	4.27185	5.373538	4.704608	4.931003	4.764584	4.693288	4.169429	5.36557
COMMD6	14.59475	17.94585	19.94098	21.48598	16.08884	24.79195	19.89199	17.48768
COMMD7	11.40433	14.36049	10.30298	13.991	17.36056	11.935	12.85343	13.46405
COMMD8	18.25872	17.23875	17.83842	15.53107	17.85002	16.9765	16.58235	17.26809
COMMD9	18.208	22.86814	17.53532	21.34663	22.40283	23.61351	17.06286	14.39003
COMP	0.297635	0.208759	0.090439	0.293277	0.11449	0.334246	0.127811	0.139326
COMT	30.65367	32.8998	30.87081	40.84783	30.7528	35.30631	36.5707	36.08472
COMTD1	4.212231	3.902527	4.488147	6.588448	3.381306	3.599275	3.487838	3.710064
COP1	23.69807	22.29415	22.94495	22.48414	21.62	25.96589	28.28863	23.56521

COPA	56.37037	56.15953	50.90787	54.8227	54.79643	52.03825	58.4684	60.22345
COPB1	36.09306	36.54117	38.42732	37.37517	37.10769	37.77153	40.59771	40.29402
COPB2	47.05792	48.95167	46.64369	47.0058	47.68994	47.17202	53.31211	50.62042
COPE	61.4048	58.20426	57.40702	60.17915	61.94007	57.62156	63.48542	64.79683
COPG1	69.82331	61.29669	60.89487	65.52276	64.04698	54.35243	69.43502	71.47939
COPG2	28.86815	31.54835	28.5377	28.57114	26.04692	29.3935	31.31606	30.16058
COPS2	30.36472	32.22473	31.891	30.06437	35.95936	35.73461	30.87589	32.62587
COPS3	35.80945	35.76631	38.32	38.4977	38.16791	35.85911	38.35744	36.95375
COPS4	39.43928	44.64537	42.08881	42.86073	39.23761	42.63883	39.32064	44.51693
COPS5	23.17883	26.60755	25.49507	26.81403	27.10043	29.274	24.74713	26.24329
COPS6	77.68116	77.00734	73.73241	76.84447	73.8241	79.34207	76.49162	72.48707
COPS7A	19.68453	18.57889	18.29166	21.1161	20.52332	18.34935	18.9729	18.51303
COPS7B	7.746421	7.584916	6.59632	7.196761	7.932989	6.883969	6.29951	6.868729
COPS8	36.54964	39.39894	39.11234	40.13947	39.42382	40.22179	42.13079	38.01884
COPZ1	59.43724	66.5173	65.08872	62.4733	63.98532	61.87903	61.32266	60.02541
COPZ2	18.16876	27.02856	24.5936	20.68527	24.80628	29.01279	24.6839	25.42204
COQ10A	1.56802	1.438565	1.806563	1.446175	1.603073	1.66644	1.989426	2.208809
COQ10B	14.01093	16.44743	15.74147	17.08885	16.59619	14.78153	16.37832	13.01651
COQ2	14.18323	18.5047	18.12209	16.29206	18.67901	19.40854	19.70526	19.35993
COQ3	7.32465	10.36422	9.765741	7.981329	8.4715	9.036397	7.891389	6.930893
COQ4	9.769973	11.05298	11.22054	11.42107	9.662667	10.3832	9.347241	8.404347
COQ5	11.48477	13.48623	11.73384	11.47624	12.88431	12.92267	12.4632	10.72994
COQ6	7.114101	6.749365	5.388169	6.610714	5.833714	6.783321	5.247328	5.717267
COQ7	8.521291	8.965168	10.1305	8.900297	8.78462	8.183528	9.347693	8.243708
COQ8A	4.529837	5.745433	4.559421	5.72221	5.41037	5.472939	5.09293	4.638498
COQ8B	4.914035	3.923228	3.340085	4.269719	3.689066	5.157354	4.01756	4.06425
COQ9	16.70658	17.48944	17.33964	17.36982	19.21244	17.80089	15.49261	15.35752
CORIN	0.216858	0.200175	0.247469	0.385198	0.332559	0.470643	0.239676	0.102641
CORO1A	10.30054	12.86961	9.180973	13.47283	12.58935	7.455125	13.06046	20.17673
CORO1B	26.82183	25.28431	27.93192	27.67872	26.86556	24.97867	28.28643	26.07414
CORO1C	26.24743	23.15672	24.17919	22.66215	23.81635	22.87634	27.09759	27.98941
CORO2A	1.367939	1.162361	1.151684	1.091743	1.021358	0.932999	1.414052	1.349051
CORO2B	2.787572	2.856995	3.032923	3.329956	4.125167	2.937155	3.109034	3.706662
CORO6	0.335973	0.392749	0.348802	0.37078	0.379096	0.442351	0.542123	0.82131
CORT	0.297125	0.12041	0.117369	0.060897	0.178298	0.059831	0	0
COTL1	22.79912	20.03108	20.74557	20.76788	22.55489	25.59873	20.78937	31.01957
COX15	11.01889	9.835226	10.09603	7.631045	9.399682	9.48492	9.502064	9.860959
COX5A	77.07269	82.01106	89.50931	82.80105	83.7547	85.85445	81.40635	74.0497
CP	0.089741	0	0.019694	0.076636	0.154574	0.020079	0.020242	0.030339
CPA3	0.846806	2.438297	1.474449	3.128581	2.206437	1.32376	2.442812	5.6504
CPA6	0	0	0	0.018085	0	0.017768	0	0
CPAMD8	0.072645	0.021028	0.015373	0.04254	0.057086	0.020898	0.031601	0.084205
CPB1	0	0	0	0	0	0.016724	0	0
CPB2	0.01895	0.019199	0	0	0	0	0.076937	0.01922
CPD	34.67252	30.39973	25.46088	26.78944	27.45652	29.04762	32.54167	35.88185
CPE	2.790856	3.286822	3.134172	5.234705	3.718264	3.276552	4.018984	5.742921
CPEB1	2.738166	2.354865	2.643185	3.175913	2.756131	2.366832	2.868603	2.63427
CPEB2	17.764	16.28018	19.37277	18.68712	16.58756	18.44184	15.37394	15.88997
CPEB3	0.592262	0.6028	0.617226	0.657277	0.592339	0.590812	0.659322	0.642216
CPEB4	8.756534	7.791718	9.102751	7.571458	8.538441	7.454139	7.307474	8.325396
CPED1	2.162733	2.708121	2.193379	1.862692	2.42063	2.431958	2.229705	3.307533
CPLANE1	3.383389	3.085528	2.724157	2.712327	3.114543	2.703209	2.670998	3.130134
CPLANE2	6.890066	6.51092	5.294444	6.273846	6.579618	6.407796	6.389568	7.254767
CPLX1	0.199258	0.148041	0.209894	0.544518	0.172713	0.267493	0.202247	0.053892
CPLX2	0.00517	0	0	0.005298	0.010341	0	0.005247	0
CPLX3	0	0	0	0	0	0	0	0
CPM	13.22151	11.52736	10.9883	13.87403	13.00515	9.739393	14.26486	14.6026
CPN1	0	0	0	0	0	0.017449	0	0

CPN2	0.16578	0.615837	0.245571	0.693702	0.110534	0.20864	0.070111	0.126105
CPNE1	56.8525	49.48548	41.97705	45.5912	48.33556	40.29809	54.96421	53.4504
CPNE2	10.23143	10.00029	8.133026	10.36166	11.4352	8.885049	10.95846	11.42577
CPNE3	24.36835	28.47579	27.97182	26.64923	24.49516	24.04283	25.67208	26.65593
CPNE4	0.364849	0.712873	0.501851	0.373887	0.703729	0.629729	0.449677	0.224669
CPNE5	0.779281	0.542787	0.424867	0.840177	0.584536	0.899026	0.947516	0.625713
CPNE6	0.087349	0.139064	0.061615	0.05115	0.174721	0.087946	0.316641	0.101249
CPNE7	0	0	0	0	0	0	0	0
CPNE8	3.147253	3.606475	3.477683	3.326978	4.133214	3.453326	3.589893	3.866107
CPNE9	0.110132	0.039849	0.023306	0.088675	0.047205	0.095043	0.023954	0.071807
CPO	2.414065	2.321688	2.764041	2.348377	2.414377	2.007852	1.82883	2.608112
CPOX	11.55751	13.34128	12.91463	11.92845	11.03021	10.86292	13.22343	11.68014
CPPED1	3.297029	3.880995	3.818125	3.974223	3.854938	3.606133	3.900219	4.366399
CPQ	31.78684	31.78997	19.48019	25.16866	27.01583	26.34383	34.60071	26.34548
CPS1	0.05455	0.070339	0.009795	0.040656	0.019839	0.009986	0.105703	0.015089
CPSF1	12.31672	10.50777	9.432353	10.96608	10.57187	9.297521	11.02552	10.82813
CPSF2	8.693983	9.252187	8.532492	9.003906	9.437325	9.574126	9.273223	9.028344
CPSF3	21.32858	20.71537	20.55499	19.09655	18.20878	21.37566	24.53166	19.88126
CPSF4	4.759291	4.000465	3.348527	4.627069	5.221949	3.958066	3.512418	5.03038
CPSF6	12.07643	12.13945	10.23229	12.37771	12.01725	10.2361	10.6741	12.60303
CPSF7	19.83206	20.03008	16.31573	19.02457	19.1663	16.30193	16.62909	17.45012
CPT1A	10.8129	10.189	7.697599	7.251648	7.189872	7.480942	9.440647	10.00444
CPT1B	0.247044	0.203937	0.162644	0.26254	0.237925	0.340854	0.278611	0.232001
CPT1C	0.723679	0.558611	0.469635	0.854612	0.937458	0.485749	0.74153	0.566214
CPT2	16.8622	16.8515	15.08967	15.34923	16.53125	15.19581	16.93553	17.02834
CPTP	22.41351	20.55725	21.61803	23.39074	23.0184	22.61447	20.78825	21.12604
CPVL	2.67693	2.105567	2.63242	0.682916	0.790757	1.660345	0.89423	1.477805
CPXM2	24.23151	21.92547	21.29059	29.50822	18.84003	15.93632	28.76787	21.93285
CPZ	1.874809	1.683877	7.248223	2.711549	3.39105	4.417826	3.873341	4.409856
CR2	0.879109	0.848235	1.124468	0.471892	0.561028	0.26132	1.087751	0.755757
CRABP1	40.09768	72.64918	55.50434	61.19184	83.56336	52.98062	74.39177	74.29749
CRABP2	139.8608	123.8157	144.8044	145.938	154.527	138.9053	122.9236	139.1223
CRACR2A	0.797337	1.031449	0.969611	1.482237	0.912772	0.802784	0.933033	0.631578
CRACR2B	1.286586	2.103147	1.558963	2.531757	2.384044	1.923195	2.163122	1.5931
CRADD	1.054537	1.365148	1.251123	1.726051	1.464824	1.445147	1.5758	1.284944
CRAMP1	6.347831	5.832635	4.293903	5.283018	5.816539	5.190538	5.619977	6.724752
CRAT	71.99725	67.71049	82.68782	103.2209	119.9483	91.1438	68.75249	53.31436
CRB1	0.015468	0.047013	0.19094	0.095106	0.402217	0.054507	0.0314	0.015688
CRB2	0	0	0	0	0	0	0	0
CRB3	17.89944	12.87725	12.52618	14.90521	15.88355	15.64697	16.01331	14.91147
CRBN	14.99308	17.58724	15.73977	14.19048	15.10771	15.16484	16.044	15.98113
CRCP	4.546332	5.106422	4.303252	4.751701	4.880567	5.155423	4.554155	4.978257
CRCT1	167.6923	137.0291	149.3062	132.3455	123.9854	143.9968	166.1434	116.1349
CREB1	8.370659	7.764628	6.498575	6.654451	7.766511	6.840782	7.573037	7.901781
CREB3	27.04379	23.22218	26.53847	26.36373	22.93982	28.74211	27.74833	23.6249
CREB3L1	9.41015	10.74427	11.11971	10.22019	12.14629	11.02459	9.224762	10.13843
CREB3L2	7.564134	7.818566	6.289458	7.135208	8.55885	6.716955	6.835904	8.850054
CREB3L3	0	0.044272	0	0	0.010926	0	0.022177	0.01108
CREB3L4	0.553757	0.841537	0.313639	1.802564	1.278693	1.500439	2.149066	1.148059
CREB5	7.333999	7.120656	6.354054	5.636748	6.853874	6.096442	6.77198	8.655179
CREBBP	18.02345	16.30391	13.88922	14.87251	16.40687	13.90528	14.51957	17.30091
CREBL2	15.21331	16.4986	16.88681	16.1856	18.18889	16.18339	17.02702	16.79224
CREBRF	3.898459	3.629464	3.651556	3.743823	3.931454	3.94888	3.759089	4.163636
CREBZF	11.95028	12.18594	10.23071	14.24379	11.22564	12.55846	11.20392	11.02014
CREG1	40.16681	39.97732	55.93828	47.07449	43.79248	41.75387	42.07855	41.86399
CREG2	5.711185	5.189857	6.673309	5.897329	6.627575	5.113728	6.084483	8.069686
CRELD1	6.774055	5.59955	7.845134	5.774358	6.667148	5.378762	5.375521	5.886759
CRELD2	13.51682	10.83033	12.66033	13.23963	12.58282	14.6113	11.96169	11.79115

CREM	2.663683	2.335363	2.802487	2.771658	3.186587	2.279598	3.254765	2.597695
CRH	0.063086	0	0	0	0	0.084689	0.021344	0
CRHBP	0.211839	0	0.130749	0.135679	0.238349	0.479893	0.080631	0.134284
CRHR1	0	0.038126	0	0.009641	0	0.009472	0	0
CRHR2	0.068142	0.034518	0.033646	0.069829	0.076669	0.248701	0.077809	0.181418
CRIM1	24.24517	22.28339	23.04115	22.01212	22.22466	23.27281	22.95972	22.30272
CRIP1	46.28558	63.51707	83.49485	60.15505	79.76672	66.15626	65.73497	67.37915
CRIP2	138.7428	138.325	129.8857	157.9548	151.603	130.1736	136.4131	140.8175
CRIP3	0.024434	0.037132	0.036194	0.100157	0.134404	0.086103	0.024801	0.074346
CRIPT	29.67635	30.00477	39.30354	30.81281	30.76494	35.55156	28.43958	29.64045
CRISP2	0	0	0	0.02208	0	0	0	0
CRISP3	0.1676	0.0283	0.027585	0	0	0.168745	0.226818	0.028331
CRISPLD1	4.903839	6.285302	6.608023	5.40735	3.5469	5.235698	6.529801	6.825436
CRISPLD2	44.59926	51.0614	39.39222	43.13404	44.60503	32.81066	48.32844	66.51254
CRK	32.33706	34.98901	29.83266	33.68143	35.24022	32.017	35.82878	35.70859
CRKL	45.87834	42.48213	40.81106	44.38066	43.97135	41.94815	44.17714	44.23165
CRLF1	11.75333	5.631964	6.169424	7.794541	5.859775	8.51097	5.230409	4.009347
CRLF2	0.04779	0.048417	0.031463	0.032649	0.031864	0	0.032338	0.177725
CRLF3	10.36515	10.87661	9.084311	9.225746	10.84611	8.372907	9.591357	11.2644
CRLS1	11.85604	13.18316	11.66703	13.70591	13.1534	13.84471	11.143	12.61828
CRMP1	0	0	0	0	0.008613	0	0	0.008735
CRNKL1	21.1538	19.14168	18.15476	17.13746	19.37167	19.29336	18.93234	21.06358
CRNN	36.62502	35.61424	36.13162	19.65974	22.29107	30.08739	52.58283	29.29107
CROCC	1.660041	1.641995	1.125979	1.844416	1.931139	1.51304	2.323446	2.113452
CROCC2	0.003728	0.033992	0.044178	0.080225	0.063383	0.026274	0.056757	0.03781
CROT	13.80685	12.527	13.28931	10.3012	13.14295	12.98187	12.14312	12.89559
CRP	0	0.034793	0	0.035193	0.034347	0.034577	0	0
CRPPA	5.457457	4.629335	5.827969	4.653194	4.885127	5.624537	5.582936	6.450408
CRTAC1	2.301156	1.831065	0.86803	2.560573	1.37297	1.034142	2.085064	1.813041
CRTAM	0.12938	0.160206	0.070982	0.353559	0.488831	0.130264	0.102138	0.160382
CRTAP	18.93632	21.80706	18.92625	21.32989	20.66048	18.04756	19.96572	21.00073
CRTC1	1.466937	1.478263	1.236189	1.463187	1.428004	1.449389	1.465118	1.690164
CRTC2	10.07012	8.395944	9.000349	10.07459	10.27066	8.68477	8.330611	9.011298
CRTC3	11.7644	11.01696	10.80053	12.60313	11.2077	10.46893	11.30597	11.54146
CRX	0	0	0	0	0	0	0	0
CRY1	4.033071	3.98955	4.119567	3.529877	3.410376	3.128248	3.821248	4.055373
CRY2	9.391808	9.856186	8.036732	10.27782	9.658119	8.81403	9.990778	10.75343
CRYAB	337.2195	180.1144	342.3463	309.8218	266.5813	488.1746	211.4949	261.4701
CRYBA1	0	0	0	0	0	0	0	0
CRYBA4	0	0	0	0.180166	0.035167	0	0	0
CRYBB1	0.614108	0.191436	0.513155	0.242046	0.165358	0.332933	0.479477	0.45516
CRYBB3	0	0.038268	0.037302	0	0	0	0	0
CRYBG1	69.55192	71.86466	80.97327	70.81549	68.1993	80.40841	69.44078	73.68933
CRYBG2	24.10192	19.80397	24.84992	23.46088	22.94731	23.096	20.81548	21.27661
CRYBG3	14.44982	11.15594	13.33626	11.29516	14.64988	15.08238	11.69303	14.01455
CRYGN	0	0.05137	0	0	0.050712	0.051051	0	0
CRYGS	0.497127	0.285085	0.185257	0.341229	0.19231	0.146379	0.195169	0.366261
CRYL1	13.67947	11.53039	12.57547	14.37922	11.79034	13.36235	13.7906	9.587868
CRYM	7.235717	8.885035	14.20922	12.04664	15.38566	8.495861	8.585832	6.560692
CRYZ	26.61216	23.20077	31.51303	20.09306	20.50808	18.44534	26.61635	23.81827
CRYZL1	5.214077	5.979797	5.921475	6.401191	6.111688	6.131646	5.472226	5.277726
CS	72.99484	70.39284	78.43326	73.24735	73.754	70.55943	70.97971	68.67387
CSAD	12.54925	14.24465	12.24981	12.59851	10.14281	11.63474	13.29768	10.86664
CSDC2	60.17074	50.69037	76.59774	61.34156	55.21487	66.69632	59.11791	56.81243
CSDE1	139.0685	141.2755	142.9318	136.4798	139.6844	146.7316	150.2966	137.8582
CSE1L	28.64845	28.69823	26.61838	25.5479	27.55575	26.04234	28.36232	29.21179
CSF1	3.582822	4.749266	4.532486	5.286257	6.440759	4.285313	4.585522	5.410953
CSF1R	12.87589	13.04488	10.29656	13.47207	14.87391	6.610756	14.9689	16.78191

CSF2	0.025375	0.051417	0	0.026004	0.050758	0.025549	0.103024	0.102947
CSF2RB	9.074669	8.018622	8.417741	12.13623	15.66095	12.28199	9.754928	14.18145
CSF3	0.038029	0.141269	0.07511	0.051961	0.050712	0.063814	0.064332	0.102854
CSF3R	0.702944	0.31266	1.752394	1.352864	1.586115	0.914891	1.148542	2.156241
CSGALNA(	5.876539	7.395518	5.206002	6.210483	6.428851	6.883892	6.975855	6.008309
CSGALNA(	4.403999	5.106986	3.861952	4.503896	4.256554	4.235409	4.561099	4.903356
CSK	12.88374	10.52448	10.87984	12.26724	12.96657	10.33655	12.12969	14.09606
CSKMT	12.17257	15.47432	15.26221	16.4997	13.93177	14.41545	12.90603	17.27372
CSMD1	0.020559	0.002604	0.020303	0.034237	0.012851	0.0207	0.007825	0.013033
CSMD2	0.004238	0.051518	0.020924	0.05211	0.008476	0.038399	0.051614	0.058022
CSMD3	0	0	0	0	0	0	0	0
CSN1S1	0	0	0	0	0	0	0	0
CSN2	0	0	0	0	0	0	0	0
CSN3	0	0	0	0.033586	0.032778	0.032998	0	0
CSNK1A1	42.15262	44.99444	46.46154	46.6444	46.25543	45.93829	44.14281	42.69486
CSNK1D	24.08329	22.97838	19.73752	22.52843	21.98228	20.82023	21.56129	23.79589
CSNK1G1	4.877711	4.106975	3.189592	2.397945	3.584592	2.588247	3.161202	4.061342
CSNK1G2	47.19655	46.13938	45.35556	47.14461	48.97909	48.06325	46.53836	44.91441
CSNK1G3	7.566508	8.806675	8.750115	8.143654	7.997232	8.349188	7.910606	9.532666
CSNK2A1	28.23924	29.3366	28.53728	28.72967	29.63167	31.1422	28.71627	28.43433
CSNK2A2	25.84893	23.29083	24.10565	24.4094	24.43141	22.25451	28.35282	22.53044
CSNK2B	35.34677	36.37852	38.04291	36.94258	37.43345	35.04184	35.75055	33.80165
CSPG4	6.562639	4.536472	4.560098	5.233896	5.178018	4.758366	5.05617	6.049353
CSPG5	1.322007	0.987607	0.923105	1.067382	1.255402	0.927694	1.17919	1.449177
CSPP1	4.466422	4.781291	3.649241	5.01959	4.787833	4.291963	4.326784	5.643734
CSRNP1	2.348892	1.901274	1.859357	1.916804	1.994192	1.979587	1.942389	1.662309
CSRNP2	7.658301	6.506129	5.268664	6.431852	6.867688	5.746349	6.392243	6.521947
CSRNP3	0.031915	0.009949	0	0.01761	0.009821	0.002472	0.009967	0.00996
CSRP1	94.03044	98.91472	98.65156	105.2856	106.7873	91.4653	107.0663	104.9854
CSRP2	27.16368	31.4927	29.24534	35.05341	25.05161	28.18037	28.04243	22.2653
CSRP3	0.02499	0	0	0	0	0.025161	0	0
CST3	1055.006	806.1234	913.4699	905.4389	904.6231	955.8362	1026.726	942.9077
CST6	3508.115	2688.083	3021.79	3307.283	3462.703	3121.011	3287.906	2553.425
CST7	0.080152	0.154288	0.007915	0.016428	0.200407	0.01614	0.048813	0.040647
CSTA	70.40257	45.08434	87.34614	67.68056	68.2631	57.90572	43.73491	43.51522
CSTF1	8.573615	9.023616	8.38063	8.712837	9.502792	8.528376	9.008125	9.596604
CSTF2	10.74453	9.37103	7.882197	9.054741	9.891584	8.533399	9.429025	10.23416
CSTF2T	6.341378	5.617491	4.479111	6.857667	5.439205	6.480951	6.856974	6.657891
CSTF3	10.03735	12.14582	11.54059	12.23616	11.4747	11.14349	12.34962	11.07911
CSTL1	0.025725	0.013031	0	0.013181	0	0.038852	0.013056	0
CTBP1	17.45859	17.83235	15.1844	19.01838	17.64562	16.92683	17.89094	17.72416
CTBP2	11.98897	11.40227	10.96339	10.91064	11.08452	11.81939	11.62393	11.14255
CTBS	0.026256	0.008867	0.017286	0.035876	0.017506	0.008812	0.017767	0.02663
CTC1	3.391245	3.270889	2.551917	3.32183	3.020617	3.001537	3.171237	3.036814
CTCF	31.71087	32.18332	29.48494	29.92767	29.28444	29.03327	30.67129	30.40171
CTDNEP1	37.04541	36.2848	37.69006	38.9493	39.21199	35.93236	36.46409	37.95682
CTDP1	11.04734	10.01048	9.772943	9.729713	10.562	9.574949	8.946915	9.073379
CTDSP1	21.41299	22.12496	21.23019	23.09418	22.31765	21.15669	21.21608	21.14834
CTDSP2	72.93489	65.82242	70.33694	70.83266	70.05894	68.51924	65.60286	71.2784
CTDSPL	22.99657	19.62445	19.00279	19.37898	18.19786	19.54113	19.14886	20.27409
CTDSPL2	13.42007	13.78268	12.4817	12.80937	12.78028	12.1134	13.6327	14.26735
CTF1	1.356815	1.885196	1.933298	2.224679	1.395762	1.366085	1.888687	1.690675
CTH	43.73114	27.8865	67.11581	52.91202	42.36035	48.17131	42.22111	28.71697
CTHRC1	0.435261	0.632701	1.644595	0.795119	0.681366	0.438234	1.21012	1.055658
CTIF	11.21916	9.923285	9.131295	10.98541	10.36389	9.255464	9.88204	10.93206
CTLA4	0.123192	0.024962	0.024331	0.025249	0.135528	0.136437	0.012504	0.174924
CTNNA1	63.67207	64.13828	51.41348	54.0169	59.32603	55.93488	69.26822	65.72493
CTNNA2	0.006902	0.034965	0	0.007073	0.013807	0.00695	0.007006	0.021002

CTNNA3	0.027445	0.065722	0.059134	0.020455	0.017467	0.025121	0.055714	0.027836
CTNNAL1	19.40043	19.31643	20.33309	18.40744	19.62837	21.59113	20.2279	18.53354
CTNNB1	135.6422	132.1839	120.7875	124.5869	122.3905	132.6951	137.9702	129.5076
CTNNBIP1	29.04139	27.54631	27.74433	26.46119	28.16116	31.16792	29.26341	27.25637
CTNNBL1	21.6678	23.35392	20.33231	22.37362	18.34448	20.10328	22.08301	20.97207
CTNND1	56.96361	52.34524	50.18686	54.33587	53.42617	53.82011	55.51928	50.22771
CTNS	9.281787	8.436752	7.474918	9.96719	12.63009	11.54329	10.6285	12.99386
CTPS1	19.52808	20.55668	15.29112	18.40509	16.40527	20.28747	19.27225	20.16249
CTPS2	4.865238	5.669022	5.703402	4.271857	6.877392	8.043455	6.215538	5.321964
CTR9	15.35786	16.58056	15.64504	16.14878	17.20794	17.58997	16.5588	16.5922
CTRC	0.008869	0	0.008758	0.009088	0.00887	0	0.018003	0.008995
CTSA	71.82892	54.24243	46.70508	63.09865	62.31307	63.87642	77.40284	63.75533
CTSB	370.3536	283.5877	274.5733	289.8645	290.7833	305.7192	331.5334	324.9817
CTSC	28.58194	30.08943	20.14316	25.08161	34.38205	24.97332	36.76715	32.42591
CTSD	213.9713	206.2949	203.0657	220.0187	209.4831	211.6211	215.9995	186.9515
CTSF	69.62136	51.15081	68.23681	67.42142	55.82099	50.30596	57.34944	63.69384
CTSH	241.5347	138.1179	155.3365	165.9698	163.4879	160.9612	142.1628	172.9153
CTSK	57.7538	58.66683	68.12863	66.55629	70.0687	57.69631	79.37535	76.92493
CTSL	0.708843	1.764586	0.720011	0.601875	0.648169	1.733236	0.842814	0.924343
CTSO	9.291449	14.20062	10.61483	12.71659	12.98498	13.31209	11.99471	10.2568
CTSS	31.05426	35.8583	38.07527	36.56808	40.94852	27.56317	37.90596	53.69921
CTSV	217.0522	206.0924	192.2354	185.3731	191.6955	193.3652	208.2386	207.2942
CTSW	3.001169	2.688028	1.889943	4.145251	4.089078	3.043568	2.295677	3.286522
CTSZ	38.43062	43.0819	45.34375	55.69486	55.54966	58.32593	49.56669	60.71527
CTTN	60.8037	57.25166	54.8152	48.55458	55.01234	55.4618	58.58975	56.5412
CTTNBP2	12.34352	12.20535	8.206707	12.0959	11.54696	9.911485	10.19077	11.38994
CTTNBP2N	22.91555	20.5667	24.25423	21.58927	22.88945	21.53903	19.75535	21.5088
CTU2	7.198363	7.252092	4.236079	6.360144	5.952488	5.344554	5.197432	5.247901
CTXN1	3.159349	1.577865	1.40619	1.162803	3.182009	2.262494	2.032441	1.444203
CTXN2	0	0	0	0	0	0	0	0
CTXN3	3.514524	2.631162	3.428917	3.341225	2.738578	3.424846	3.810789	2.734258
CTXND1	0.041404	0	0.030666	0.010607	0.020705	0.020843	0.021013	0
CUBN	0.047367	0.083349	0.049239	0.028103	0.084774	0.045181	0.055669	0.058156
CUEDC1	8.134379	9.593196	8.606811	9.90815	9.969535	9.378224	9.08572	10.45084
CUEDC2	35.91906	41.14294	40.74369	42.41709	42.49184	41.38219	39.81341	38.60533
CUL1	22.32129	23.40788	21.67001	24.92362	24.51788	24.02337	24.43343	24.21873
CUL2	12.13474	13.53839	12.10397	11.09627	13.16436	12.8579	12.37865	11.07055
CUL3	32.21192	31.48369	30.41874	30.24207	31.24601	35.10272	31.75956	30.17576
CUL4A	34.78555	32.1172	31.54046	36.87858	33.92508	31.91002	34.70917	37.07389
CUL4B	16.49826	17.36071	16.84482	14.92674	16.00008	17.00599	16.28776	15.94223
CUL5	7.607474	9.175374	8.890009	8.018654	9.16638	9.109268	7.473392	7.706586
CUL7	22.64694	22.76495	18.46043	22.55532	20.58436	19.98318	22.45698	21.48021
CUL9	2.808614	2.7727	1.947207	3.099017	2.755096	2.462573	2.354962	2.783027
CUTA	80.75113	85.17348	91.7405	77.14111	81.58288	84.88562	77.82955	76.00105
CUTC	8.889442	10.23807	9.503334	9.388942	8.890592	9.604547	10.6188	9.164838
CUX1	35.53297	32.79453	29.7826	29.23496	27.264	27.45519	31.11523	31.65571
CWC15	47.166	57.62608	55.41269	52.95224	51.41191	57.57064	50.46537	49.7164
CWC22	7.282561	8.298564	7.923921	7.634258	8.119433	7.2445	7.583603	7.496805
CWC25	12.14024	12.60177	11.92024	12.21679	14.18036	12.42339	10.68744	12.12146
CWC27	4.868766	5.244644	5.53959	4.724974	4.719568	5.505354	5.110748	4.9634
CWF19L1	9.461524	11.07363	8.82764	8.972315	10.76211	9.256016	9.101774	10.9139
CWF19L2	9.38661	9.591167	8.645065	9.187031	8.905882	11.15894	11.26986	10.36535
CWH43	8.345653	8.89232	8.97052	9.564701	8.551142	8.837091	10.9948	4.618025
CX3CL1	16.71364	16.29386	11.48201	14.54172	15.54178	12.67916	12.39297	13.84959
CX3CR1	1.147993	0.803065	1.277649	0.989694	1.594641	1.109969	1.081983	0.933317
CXADR	42.1087	50.25682	46.49133	42.41301	41.05672	47.02213	46.72071	44.53111
CXCL1	0.082808	0.16779	0.490658	0.509156	0.331276	0.166748	0	0.083987
CXCL10	3.224008	3.290515	3.844187	2.936771	7.308696	7.045093	3.126929	6.128042

CXCL11	0.09696	0	0.031917	0.066241	0.711133	0.585735	0.360853	0.393362
CXCL12	61.0103	56.76434	52.75979	54.9944	68.48876	63.16227	56.5327	59.39245
CXCL13	1.683742	24.54926	9.253635	11.27794	6.003683	7.788299	7.380892	6.731848
CXCL14	258.5994	270.9604	316.4032	292.9065	280.558	266.2381	237.535	240.6556
CXCL16	18.26144	15.38793	15.01669	18.87573	18.17599	14.03715	15.77288	17.7378
CXCL8	0.056278	0.095028	0.148206	0.153793	0.243905	0.151101	0.057123	0.285398
CXCL9	2.420632	1.310594	1.442024	0.964116	4.263607	3.966565	1.35281	4.661691
CXCR1	0.253152	0.096178	0.406247	0.145925	0.379778	0.191161	0.465721	0.978883
CXCR2	1.956487	5.58085	5.214819	3.007425	2.754634	1.99854	2.544953	3.747126
CXCR3	0.218381	0.442494	0.179717	0.22379	0.618826	0.054968	0.277071	0.14766
CXCR4	16.19346	14.4048	13.70979	22.03699	18.59973	17.86133	13.82622	17.36898
CXCR5	0.006271	0.012707	0.012386	0.064267	0.012544	0.018943	0.012731	0.044525
CXCR6	0.565438	0.962402	0.429962	0.486733	0.678613	0.267571	0.591137	0.619366
CXHXorf21	0.91503	0.781621	0.726447	0.551586	0.663931	0.740638	1.311185	0.855268
CXHXorf38	7.379441	8.574845	6.915681	9.174994	8.156089	8.589821	8.904086	9.027214
CXHXorf58	0.243607	0.376701	0.189925	0.459865	0.230815	0.103272	0.169179	0.026008
CXHXorf65	0	0.027665	0.026966	0.111932	0	0	0.027716	0.166171
CXXC1	12.44962	11.73515	10.78493	12.70774	11.60098	11.84333	11.82326	12.73463
CXXC4	0.145956	0.313139	0.110223	0.136374	0.180322	0.142631	0.204789	0.217696
CXXC5	21.33326	21.02687	22.52314	20.50866	20.20545	21.49721	18.22031	20.44458
CYB561	6.547801	9.705914	13.12323	12.09449	8.051615	10.69395	8.726984	6.504927
CYB5A	111.8875	130.0198	147.8933	132.4351	120.4035	138.6057	144.5703	134.9288
CYBB	3.477983	3.188682	3.173457	3.923257	3.809082	2.889271	4.516716	5.110172
CYFIP1	51.28546	47.72113	46.31321	46.13304	49.61763	44.52336	51.65675	49.14332
CYFIP2	23.91479	24.62838	28.9127	20.47215	24.65475	23.58769	26.45568	19.74311
CYGB	4.200817	4.602363	4.051998	4.455043	6.741717	5.016402	5.453735	4.706497
CYHR1	6.816468	6.484022	7.121141	6.378873	7.825704	7.304388	5.138982	5.868699
CYLC1	0	0	0	0	0	0	0	0
CYLD	9.029194	8.635422	7.273376	6.971852	8.214284	7.598627	7.255673	9.909343
CYM	0	0	0	0	0	0	0	0
CYP11A1	0	0.015529	0	0	0.01533	0.030866	0	0
CYP11B1	0	0	0	0.00592	0	0	0	0
CYP17	2.171035	1.803929	4.102874	1.488543	1.437129	0.817734	2.552371	2.724703
CYP17A1	0.536066	0.34561	0.497303	0.482758	0.097479	0.99768	0.049464	0.362465
CYP19	0.037023	0	0	0	0	0	0.056367	0.018775
CYP1A1	0.194144	0.185765	0.127817	0.176848	0.118659	0.401801	0.175161	0.076575
CYP24	5.289861	3.156839	3.917962	3.267388	4.040382	5.709034	6.895389	5.971503
CYP2A6	8.932712	29.89067	27.92606	20.24337	11.06098	34.55308	98.17944	28.40487
CYP2C18	0.840815	0.115505	0.309618	0.540352	0.498853	1.549634	0.737708	0.534797
CYP2D6	2.555238	0.720056	0.451204	1.179207	1.184701	0.937074	1.219492	1.218573
CYP2E1	0	0.01672	0	0	0	0.016616	0	0
CYP2J	20.70619	23.15446	26.58716	30.38436	27.58135	26.69235	16.47011	19.7647
CYP3A24	0.203188	0.034309	0.016721	0.242925	0.084673	0	0.171863	0.15456
CYP4F21	0	0	0.013431	0	0	0	0.013804	0.027588
CYREN	3.354463	4.030486	3.696237	3.425492	3.637414	3.555135	3.966269	3.903595
CYS1	4.435128	4.596778	4.480697	3.49212	4.167644	3.91287	4.540518	5.009578
CYSLTR1	0.904553	1.48524	0.667395	1.310529	0.956663	1.203842	1.171396	1.360327
CYSLTR2	1.767372	1.813973	1.65409	2.923883	4.378566	2.674984	1.746984	2.026849
CYTH1	17.43623	18.1637	16.40413	18.40217	16.90281	18.87436	16.70599	16.91364
CYTH2	41.99305	40.09476	42.11261	41.10836	38.7807	42.1114	42.4153	39.28996
CYTH3	25.10905	23.35619	20.69899	21.98642	23.67839	17.78199	20.21856	21.65745
CYTH4	0.976745	0.912598	1.055673	1.217809	1.405609	0.846832	0.897764	1.629068
CYTIP	1.104973	1.288451	1.225031	1.602372	1.782781	1.09153	1.66116	2.114533
CYTL1	0	0	0	0	0.170861	0	0	0.028879
CYYR1	16.29857	15.77042	17.82301	15.63107	21.31463	21.42167	16.85341	21.69459
CZIB	38.20972	42.44327	46.93521	38.93413	38.14242	44.61589	44.06145	38.90018
D2HGDH	6.527533	5.973901	4.929854	7.357426	7.439966	5.703843	5.479692	4.899565
DAAM1	20.22615	19.88589	20.35563	15.96246	17.00909	19.35954	18.15357	19.25935

DAAM2	2.624628	3.239311	2.559373	3.534112	2.868096	2.899762	2.542717	3.614792
DAB1	1.631417	0.446856	0.404084	0.882202	0.143498	0.379876	0.501621	0.544361
DAB2	18.18463	19.098	17.70229	16.44866	20.37633	13.90704	18.89552	20.66373
DAB2IP	9.775186	9.241722	8.240966	11.71765	9.476098	10.28795	10.98486	9.723974
DACH1	13.99193	13.73408	15.95346	13.9083	13.91401	15.31275	12.83718	13.14012
DACH2	0.013463	0.054558	0.02659	0.013796	0.026929	0	0.013665	0
DACT1	4.887344	5.424463	4.423043	5.598949	5.405956	5.640481	6.411831	5.903912
DACT2	0.476627	0.369867	0.731069	0.571571	0.770816	0.265467	0.607294	0.668549
DACT3	1.832384	2.86625	1.998462	3.425388	2.688515	2.564617	2.8409	1.981006
DAD1	101.9142	127.1593	114.3702	105.456	115.1027	132.4055	107.2411	92.86969
DAG1	40.17684	36.19649	33.50982	40.99592	36.03778	38.18746	37.59117	37.07384
DAGLA	0.508894	0.670745	0.322024	0.612635	0.736262	0.616834	0.461364	0.591304
DAGLB	10.33269	9.462749	9.388868	10.57793	10.05191	8.64661	10.49829	12.07983
DALRD3	6.463773	7.536092	6.910104	7.643751	7.685702	5.960438	6.310797	5.889804
DAO	0	0.017207	0	0	0	0	0	0
DAP	49.07207	58.63395	59.64576	53.23435	53.38597	55.91826	53.18563	55.67996
DAP3	5.284406	5.750684	4.836267	5.042493	5.970798	5.405034	5.268992	6.059744
DAPK1	4.505215	3.278154	3.417146	4.161235	3.799904	2.874795	3.707545	5.65242
DAPK2	3.110702	2.187956	2.00559	1.927309	1.716471	1.475978	1.622231	2.527614
DAPK3	18.8505	15.60548	14.9802	18.26677	16.41457	15.96373	18.91202	14.86112
DAPL1	149.0135	141.6232	139.4961	160.3318	139.1776	117.7349	133.6926	142.8949
DAPP1	2.569778	3.026573	3.341381	4.169601	4.444149	3.126361	3.271274	4.126737
DARS	64.9935	68.13412	66.74636	70.02252	62.67619	68.83069	65.98551	65.81618
DARS2	3.360167	3.805642	3.245847	2.608864	2.531457	2.851278	3.313763	3.303826
DAW1	0.016562	0.201348	0.049066	0.033944	0.049691	0.016675	0.13448	0.06719
DAXX	19.09481	17.68317	19.85889	16.81348	19.24841	17.68202	19.55652	20.18772
DAZAP1	26.95777	27.35254	26.49206	27.2214	25.77804	27.55935	26.08948	25.97753
DAZAP2	115.8218	126.0157	128.2492	128.7647	124.1368	124.352	126.6932	115.5179
DBF4	5.293702	6.869036	5.939076	6.268431	5.534521	6.745792	5.825702	5.937278
DBF4B	0.278612	0.320412	0.141288	0.370395	0.15062	0.166793	0.114645	0.229117
DBH	0	0.013279	0	0.013431	0	0.013196	0	0.006647
DBI	251.3473	285.0179	402.1338	276.1102	269.7024	352.3245	286.4848	239.9574
DBN1	13.65707	11.47604	14.96647	14.63649	15.25607	11.19755	14.99828	15.59649
DBNDD1	2.311465	2.246217	2.12738	2.578199	2.437574	2.153107	2.617459	1.945668
DBNDD2	14.83261	14.43835	16.89615	15.2993	16.72397	18.26018	16.59896	13.53054
DBNL	33.32012	34.17235	32.59181	34.25378	33.50942	32.00471	34.23563	34.21655
DBP	21.9506	27.86359	20.97054	26.68818	22.29691	29.0448	24.34227	19.33144
DBR1	10.57688	12.45061	10.5872	10.07204	10.06013	10.9534	10.29735	8.903055
DBT	14.52502	18.86415	17.52616	19.92431	20.00051	18.68709	16.62475	15.15414
DBX2	0	0	0	0	0	0	0	0
DCAF1	6.328886	5.988807	4.928369	5.66089	6.22299	5.178117	5.610012	5.924263
DCAF10	4.059628	4.702944	3.645355	3.379054	3.837444	3.707944	4.042285	4.0653
DCAF11	19.56279	20.08438	17.28817	19.35977	19.29515	18.82786	19.21057	19.46124
DCAF12	21.629	22.61634	21.33761	20.55236	20.57532	21.15201	21.14368	21.50985
DCAF13	25.30627	25.73021	22.93224	21.01028	25.05572	25.40614	25.57546	23.16605
DCAF15	9.51656	8.666199	7.484692	8.653439	9.359363	7.765858	8.595671	8.836369
DCAF16	9.383896	11.58314	10.91381	11.41216	10.90454	9.198996	11.497	11.40234
DCAF17	3.339869	4.481604	3.035095	3.440804	3.473557	2.88869	3.281776	3.369396
DCAF4	3.231302	5.453946	3.171422	4.254532	3.675419	3.380045	4.395431	5.117425
DCAF5	12.92106	12.52105	11.05138	11.76602	12.12293	10.81834	12.04247	11.7188
DCAF6	8.388619	9.09774	8.408175	8.732739	8.899739	8.498016	9.609699	9.977266
DCAF7	22.42874	24.6846	24.37991	19.61047	21.20155	20.22301	22.96454	22.82392
DCAF8	21.12222	22.61047	19.71624	19.83404	20.36822	20.76078	21.57458	22.10648
DCAKD	3.500242	2.601394	2.447394	3.050212	2.964092	2.829613	2.658076	2.34512
DCBLD1	13.82493	10.53759	12.97643	13.51622	12.07447	12.80136	10.44439	11.12479
DCBLD2	4.21791	3.904416	3.218284	3.776403	3.916503	3.78182	3.50606	3.998762
DCC	0.022942	0.029054	0.005664	0.017633	0.034417	0.017324	0	0.023269
DCDC1	0.023505	0.014288	0.06035	0.019269	0.014105	0.037864	0.028628	0.047678

DCDC2	0.02619	0.291866	0.395338	0.513762	0.097288	0.052737	0.448103	0.390847
DCDC2B	0.090622	0.052463	0.063923	0.026533	0.025895	0.039103	0.131401	0.026261
DCDC2C	0	0	0	0	0	0	0	0
DCHS1	1.784138	1.365648	1.17028	1.4352	1.936789	1.153459	1.554867	1.812202
DCHS2	0	0	0.002443	0.002535	0.004948	0	0.002511	0.002509
DCK	5.102997	5.081844	5.373465	5.140262	4.75568	5.021089	5.356117	5.489317
DCLK1	0.781357	1.843675	2.540751	1.622166	2.026526	1.799615	1.356038	2.812687
DCLK2	3.626394	4.197251	2.311507	2.421174	4.286292	2.843485	4.807334	3.287925
DCLK3	1.442944	1.742242	0.897923	1.478686	1.957123	2.298611	2.17682	1.924584
DCLRE1A	5.825856	6.124444	5.078957	6.599278	5.106114	4.572688	5.995902	5.552992
DCLRE1B	2.11921	1.780808	1.742837	1.51075	1.956447	1.241678	1.553898	2.24283
DCLRE1C	2.207655	1.834458	2.166392	1.95546	2.096499	2.180666	1.625795	2.309716
DCN	740.7651	848.6843	962.5738	945.717	994.3214	860.8284	1012.443	1024.868
DCP1A	6.807506	6.917747	5.638905	6.718225	6.895038	6.309845	6.172593	6.749589
DCP1B	2.97159	4.095486	3.635157	3.621316	3.748436	3.97571	3.518858	3.108925
DCP2	7.065481	7.637642	7.152692	6.859949	7.710587	6.815964	6.79788	7.856005
DCPS	18.38342	13.87862	11.14495	14.18364	15.02571	13.55421	16.0416	13.38993
DCST1	0.114616	0.104508	0.079231	0.046982	0.14902	0.103859	0.081434	0.058124
DCSTAMP	0	0.007337	0.007151	0.014842	0.007243	0	0.00735	0
DCT	2.566391	1.832266	1.785996	4.042021	2.807891	1.543417	3.234256	3.406513
DCTD	7.808889	4.489037	4.823353	5.694533	4.124328	5.020641	5.684769	4.123174
DCTN1	41.50096	37.05845	34.50222	35.08509	36.52001	34.83946	40.36192	36.63186
DCTN2	103.1966	113.0672	103.0662	107.8827	103.7642	106.1492	103.9944	105.5869
DCTN3	37.39617	35.50434	33.33205	32.24656	33.75698	32.51582	35.13303	30.63846
DCTN4	38.18039	41.15857	40.1047	41.10501	39.40432	38.81085	39.93805	39.91542
DCTN5	28.6374	28.21055	27.32745	25.40531	27.69024	27.23764	28.90612	31.03205
DCTN6	23.80921	23.72266	23.81367	21.27424	21.95712	26.14647	24.54032	22.07352
DCTPP1	17.85391	16.4073	14.1764	15.74579	16.52151	14.81649	18.37799	13.64507
DCUN1D1	8.840554	9.683759	9.207623	9.041331	9.882941	9.670122	8.947916	10.20974
DCUN1D3	3.409902	3.178572	3.062895	2.994646	3.084011	3.17327	3.260885	3.283886
DCUN1D4	14.86924	14.85938	15.19965	16.368	14.05514	14.29392	13.66123	14.24677
DCUN1D5	15.88934	19.37581	17.02632	18.66745	18.21858	18.71998	19.79408	18.81269
DCX	0	0.101819	0	0	0	0	0	0
DCXR	11.04653	13.03626	11.35415	12.40419	13.11186	11.71563	10.47296	11.23896
DDA1	36.14349	32.98954	36.15562	37.71631	38.54518	35.14226	33.35821	33.72426
DDAH1	0.819797	0.896706	0.58032	0.892145	1.40762	0.964181	0.706911	0.985987
DDAH2	28.35668	29.08157	27.33478	29.37624	30.33358	26.81712	28.78203	27.74021
DDB1	57.88668	54.17499	49.53644	51.61231	50.96811	48.58275	54.38181	55.34519
DDB2	3.846537	3.854934	3.216101	3.975872	3.581148	3.22874	4.199372	4.474271
DDC	0.20833	0.211064	0.154301	0.298887	0.197939	0.188778	0.327756	0.316944
DDHD1	6.415494	6.929817	8.369766	7.170616	9.729327	6.760123	6.415966	6.932105
DDHD2	9.364453	10.13392	8.842618	8.915722	10.8376	8.871113	10.54927	9.867633
DDIAS	1.663717	1.787266	1.069358	1.410974	1.599383	1.59566	1.637721	2.138344
DDIT3	12.07622	12.68077	12.11209	12.40758	10.63097	10.82886	11.74664	11.00419
DDIT4	26.17565	31.60839	22.79066	29.14296	29.95099	34.17517	34.72935	35.40312
DDIT4L	1.772072	2.329075	1.125671	2.375481	1.916002	1.928841	1.876433	1.962456
DDN	0	0	0.015183	0	0	0	0.007803	0
DDO	0.068239	0.069134	0.101082	0.081584	0.022749	0.080155	0.080806	0.196095
DDOST	60.86016	62.04068	70.96178	69.40665	74.96721	65.75995	72.11783	70.76054
DDR1	63.07506	52.1884	50.2277	62.84943	58.57146	56.38805	50.17456	52.27318
DDR2	6.079228	6.635992	5.555979	6.422798	7.89872	5.2616	6.911105	8.165317
DDRGK1	23.94772	23.08593	23.47949	23.76988	22.14483	24.11132	22.67047	20.01523
DDX1	45.54358	54.05316	46.75581	48.11951	46.48404	47.56838	46.81388	50.76147
DDX10	7.963584	8.582903	7.847904	7.349882	8.022931	10.18184	8.201408	7.789695
DDX11	1.290082	1.356736	1.024745	1.422627	1.255187	1.411842	0.875327	1.415114
DDX17	180.4518	162.2381	154.396	177.228	183.1818	157.5854	156.5206	189.5457
DDX18	16.09293	16.82281	15.35357	14.28946	17.86014	15.28048	14.87114	14.38852
DDX19A	11.80239	10.29825	10.23195	10.4321	10.65673	10.62937	9.145416	10.9295

DDX20	12.90524	13.99509	12.08524	13.0729	11.59025	14.04818	13.23056	13.5403
DDX21	37.90361	39.45838	33.80222	32.68732	36.57605	34.35077	39.27226	37.66622
DDX23	30.74136	30.26701	26.6925	30.32768	30.88126	26.38436	30.71966	30.63622
DDX24	27.16232	32.2176	27.65919	30.42581	30.5877	29.24252	31.79493	29.66963
DDX25	0.030859	0.093792	0.060949	0.09487	0.231473	0.233024	0.297558	0.046948
DDX27	14.96567	14.2518	14.2056	14.05292	13.12502	13.44142	14.21371	13.8072
DDX28	6.674923	7.208236	7.176021	5.347842	5.128217	7.132918	5.974359	6.354516
DDX31	53.88169	54.44785	53.72337	59.62759	53.62886	54.35253	57.46773	56.19185
DDX39A	9.034429	8.804827	8.831358	8.960825	8.294724	9.073081	10.72023	9.116566
DDX39B	117.0202	108.9726	95.04465	111.2208	114.7765	104.4936	99.67184	117.4422
DDX3X	113.9933	119.8665	119.1424	131.9332	127.67	124.3759	123.3911	132.1475
DDX4	0.229419	0.297511	0.262811	0.075233	0.211093	0.304902	0.316691	0.186148
DDX41	20.10188	22.29012	21.35455	22.23697	23.602	20.01123	23.05917	22.18699
DDX42	20.33616	23.15841	19.47957	22.46732	21.02154	20.64149	20.15982	21.07753
DDX46	14.75661	16.56759	13.8257	14.74593	14.85309	15.81506	15.00619	14.70153
DDX47	19.40682	19.73723	20.57962	21.32995	20.87935	18.62388	20.14043	18.39446
DDX49	16.90138	15.16496	17.74136	16.82868	15.85458	16.23238	16.50095	14.40656
DDX5	183.9501	192.6808	183.8942	206.3204	195.8079	195.7269	180.5967	193.7614
DDX50	10.08421	11.74534	10.25499	9.785293	11.10786	10.28379	10.40017	10.16194
DDX51	6.210979	5.952357	5.278551	6.020783	6.308979	5.7019	5.909575	6.442768
DDX52	8.774333	10.5168	9.943447	9.900671	10.25003	9.196336	10.7796	9.896151
DDX54	32.87794	31.62694	33.7858	31.05355	30.56812	30.52874	31.3446	30.95196
DDX55	5.548667	6.012857	5.47953	5.410202	5.221573	5.409783	6.178453	5.259603
DDX56	12.1254	13.05924	10.81165	13.88102	13.76575	12.63595	12.5044	13.2336
DDX58	8.877835	7.275772	7.936493	6.490245	6.195124	7.342576	7.973714	8.686918
DDX59	6.608553	6.669874	5.411676	5.525744	5.417958	4.267458	5.294868	6.893407
DDX6	56.03501	58.78484	57.2616	53.87513	57.93926	55.61881	57.17207	59.11468
DDX60	0	0	0	0	0	0	0	0.016704
DEAF1	5.407897	6.04012	3.660206	6.596156	6.173715	4.727716	4.766072	5.979859
DECR1	13.53119	18.60842	22.38191	19.35764	15.70697	18.06869	16.67596	14.18526
DECR2	1.300263	1.127198	1.151684	0.879156	1.313837	0.836774	0.88438	1.046861
DEDD	21.52173	19.85968	19.87918	19.55665	21.02415	20.69797	21.11517	20.2505
DEDD2	19.02065	14.97997	15.72814	14.64077	18.26732	15.6765	14.9064	15.66163
DEF6	8.729739	7.588072	6.69532	7.665053	7.980793	7.007338	8.860686	8.458473
DEF8	6.777441	5.985688	5.844515	6.69154	6.04539	5.795855	5.909565	5.515541
DEFB112	0	0	0	0	0.014811	0	0	0.01502
DEFB114	0	0	0	0.1406	0	0	0	0
DEFB124	0	0	0	0	0	0	0	0
DEGS1	35.03768	37.03366	43.34244	41.28091	49.28451	33.25532	41.46966	40.52404
DEGS2	10.13119	9.247197	11.03465	12.84611	9.463226	11.24238	11.62581	10.24385
DEK	81.85716	97.70411	94.1948	85.69318	86.9623	94.05669	92.71472	89.19447
DELE1	7.265697	8.701725	7.06421	9.300693	9.376708	6.397774	8.679824	9.382345
DENND1A	6.739406	6.324072	4.816568	5.702883	5.893774	5.405987	4.053827	5.397455
DENND1B	5.040019	5.498124	4.727143	4.797231	4.632633	4.603479	4.805041	6.263966
DENND1C	0.817041	1.644341	0.730536	1.493525	1.57908	0.911554	0.997397	2.038085
DENND2A	1.937904	2.474227	2.74511	2.481315	3.080206	2.467251	2.63109	2.789729
DENND2C	9.034477	9.584899	9.638064	9.303648	8.216236	8.366048	8.900289	8.831825
DENND2D	7.778339	7.229147	9.725566	6.113305	7.046415	7.261915	7.125087	7.354438
DENND3	1.534483	1.989301	1.56521	1.825953	2.438325	1.865139	2.049342	1.807182
DENND4A	7.479153	9.266348	7.278588	7.5091	9.051933	7.798947	8.798597	9.961561
DENND4B	3.192981	2.896248	2.449591	3.23151	3.338548	2.9004	2.874827	3.300885
DENND4C	9.51124	9.670882	9.337907	9.519285	9.766277	9.342001	9.289003	9.91475
DENND5A	23.20403	22.13029	19.45694	22.57259	22.27828	20.47424	21.24071	25.82001
DENND5B	0.718431	0.707827	0.523973	0.486315	0.896507	0.656978	0.628858	0.818905
DENND6A	7.009694	7.891564	7.538295	8.512497	7.974647	7.271664	6.963767	9.136655
DENND6B	2.69166	2.595481	2.057683	3.108376	2.664678	2.359255	2.26745	3.097208
DENR	24.57045	27.63388	30.11785	26.33847	27.25484	29.67854	28.97069	25.90556
DEPDC1	3.781105	2.770612	3.157093	3.434002	2.234286	2.372064	2.599815	3.170818

DEPDC4	0.671252	0.535806	0.6428	0.312672	0.630651	0.778237	0.309691	0.474502
DEPDC5	5.049383	4.146855	4.351916	3.971578	4.260651	4.17204	4.611584	4.741531
DEPDC7	2.967802	4.418762	3.578519	3.59581	2.853394	4.3583	5.109293	2.86038
DEPP1	7.757586	6.123018	6.490153	9.243889	8.286998	8.991252	6.35671	6.103597
DEPTOR	2.557489	1.948473	1.838653	1.677337	2.005331	2.039369	1.88978	2.365634
DERA	7.918316	10.96091	9.103722	9.276104	8.252786	9.09694	9.170744	10.55025
DERL1	60.19763	60.45982	76.08916	70.97532	64.63881	74.41098	65.75596	56.99178
DERL2	38.64713	50.04945	50.82423	47.82051	47.19881	56.89245	46.85293	43.87176
DERL3	0.286481	0.319265	0.113164	0.851373	0.80225	0.374969	0.348933	0.232447
DES	13.89731	25.65881	24.9268	21.86695	48.79889	23.41836	31.74107	26.85848
DESI1	20.21342	19.48623	23.08838	25.85904	19.33252	20.6157	20.33019	16.92611
DESI2	9.58617	9.718876	10.21304	10.07479	10.41133	10.6319	9.184034	9.784783
DET1	9.350281	8.098285	7.869417	8.848711	9.154096	9.066405	10.02892	8.957923
DEUP1	0.178875	0.181222	0.055783	0.125419	0.084741	0.094788	0.238892	0.12413
DEXI	4.626744	5.257804	4.065278	5.480496	4.732909	4.552078	4.910418	4.978091
DFFA	3.439894	3.202468	3.320522	2.694109	4.199692	2.964166	3.161216	4.03367
DFFB	4.355845	4.425877	3.323371	4.580866	4.2421	4.411175	3.828255	4.662574
DGAT1	5.664474	5.696617	3.989762	6.302744	5.62355	5.311777	4.22753	5.590218
DGAT2	27.88892	28.99968	35.66966	32.92834	32.69352	27.92246	31.9248	32.17187
DGAT2L6	6.721536	3.527101	8.64614	3.479339	6.343192	8.520051	3.743551	4.474894
DGCR2	16.93881	15.5092	15.75996	17.30644	16.15521	17.2927	14.92051	17.52269
DGCR6L	17.82565	17.63278	16.92748	17.40375	17.30128	17.70883	17.18437	15.75606
DGCR8	21.58322	19.4779	16.81095	23.41328	21.29902	18.66979	20.79706	23.16473
DGKA	7.744494	9.401976	9.025941	9.154699	9.958494	8.354359	10.30769	8.750752
DGKB	0.206334	0.355371	0.193575	0.200872	0.381767	0.498584	0.314143	0.544105
DGKD	3.067226	2.677032	2.040413	2.862879	2.904637	2.654545	3.225476	2.969217
DGKE	2.866804	2.74284	2.225288	3.273087	2.773101	2.622866	2.249807	2.306182
DGKG	0.203165	0.486111	0.268934	0.407535	0.510139	0.235018	0.443137	0.464724
DGKH	4.007397	3.430496	2.130942	2.862817	4.330668	2.395647	2.478638	4.0791
DGKI	0.054586	0.023043	0.017969	0.083908	0.045495	0.105339	0.073874	0.073818
DGKK	0.006302	0.01277	0.006224	0.032293	0.012607	0.019037	0.006397	0.025569
DGKQ	3.920851	3.556027	2.771607	4.017972	3.949218	3.576012	3.244522	3.8354
DGKZ	10.00043	11.05018	8.354705	10.23866	10.15901	9.300315	11.57769	10.94502
DGUOK	7.023303	7.62498	7.312551	7.765946	5.272494	6.023686	6.371784	6.349397
DHCR24	87.15032	67.9625	90.42596	114.7568	83.09886	111.1622	75.895	68.30331
DHCR7	46.45864	43.15684	46.34744	52.3309	47.35573	51.81891	40.23047	37.84743
DHDDS	8.150068	8.301185	6.808959	7.807062	8.133686	7.06484	7.591069	7.938982
DHDH	5.775887	11.3734	11.30062	14.10759	6.732166	12.02421	8.220759	9.624038
DHFR	14.49695	13.53598	14.36357	11.66911	14.41509	15.57146	14.77511	13.79347
DHH	0.09424	0.254604	0.108576	0.160957	0.298464	0.268836	0.223191	0.350464
DHODH	9.661496	9.301486	8.185359	9.16221	7.174116	11.09246	9.161947	8.580681
DHPS	21.45021	22.74298	20.94154	20.60378	20.31208	21.24808	20.88289	19.0697
DHRS1	36.68133	38.71678	38.0196	42.18923	41.43992	44.19628	33.44497	32.80517
DHRS11	12.72699	10.92889	11.02256	12.44941	11.19711	12.52271	11.44995	11.00991
DHRS12	5.277957	7.273666	5.370138	6.810947	8.282309	6.208628	5.844138	4.668184
DHRS13	2.406712	2.528605	1.70772	3.708625	2.888427	3.320617	2.388528	3.073817
DHRS3	17.11439	20.31312	17.81662	21.38701	17.33056	20.51005	19.07203	16.79148
DHRS7	39.98452	40.86153	42.08296	40.03965	33.27403	41.50479	41.99592	36.16775
DHRS7B	7.658014	8.613406	7.971382	10.53975	9.171697	8.49579	8.419278	7.734735
DHRS7C	0	0	0	0	0	0	0	0
DHRS9	0.286701	0.199694	0.31852	0.532517	0.376343	0.360824	0.381939	1.163129
DHRSX	8.937267	6.470124	7.346114	9.304892	8.385346	9.914319	6.735595	6.911451
DHTKD1	11.48087	10.25046	8.368506	8.817753	8.562214	8.128018	8.458915	7.143479
DHX15	60.31651	59.60228	52.45532	57.04127	58.22462	55.94922	63.40874	63.2762
DHX16	8.538789	8.734033	7.799965	7.883682	8.52347	7.191823	8.07519	7.42791
DHX29	15.3883	15.7434	14.0083	15.28616	15.7894	16.41268	15.2202	14.16621
DHX30	12.11138	11.53493	9.799966	12.96277	11.83373	10.67624	10.93803	11.39829
DHX32	31.36212	27.05999	22.77491	27.68954	28.47731	30.03023	30.27386	31.49337

DHX33	5.504648	7.357992	5.364906	5.793592	6.014581	5.629304	6.464791	5.568395
DHX34	1.361573	1.231366	1.211666	1.470188	1.484845	1.4096	1.561578	1.852977
DHX35	2.599325	3.029798	1.74185	3.200531	2.473657	2.263243	2.867149	2.400942
DHX36	12.92469	15.06969	12.56261	14.99258	12.69561	12.80802	13.43998	13.56292
DHX37	5.273623	5.039745	3.936853	5.190364	4.982061	5.365689	4.448837	4.960599
DHX38	13.27301	12.70633	11.42044	11.35361	11.4107	11.22654	13.6823	11.48632
DHX40	17.28116	18.56323	16.17747	16.89249	16.06803	15.5004	18.55756	16.47074
DHX57	5.191539	5.030991	3.332167	4.566894	4.763867	4.108181	6.221078	5.406325
DHX58	1.68162	1.755949	1.507844	1.89243	1.785017	1.734656	1.497415	1.287017
DHX8	18.12343	17.40649	15.94514	17.21488	16.94595	16.93344	18.40866	18.90279
DHX9	42.66445	43.92713	37.33782	39.50934	43.12556	38.32781	41.11345	42.9043
DIABLO	22.15127	22.73679	19.75598	19.41984	22.80893	21.36874	22.81581	20.29004
DIAPH1	15.30718	14.68744	15.45859	15.68635	15.6947	13.45642	16.70564	16.84153
DIAPH2	4.443188	4.020255	3.485723	3.542247	4.977307	3.848129	4.057369	4.447145
DIAPH3	5.805877	5.998489	4.754056	4.772148	4.475942	5.656778	6.218306	5.943732
DICER1	12.35348	11.04006	9.017734	9.100006	10.77532	11.10336	9.93318	11.11859
DIDO1	6.580213	6.240769	4.831686	6.513063	6.892308	5.420297	5.649888	6.016585
DIMT1	11.80075	13.04688	11.66935	14.21949	12.00822	12.43955	12.5244	11.92055
DIO1	0.758996	0.313279	1.249232	1.517184	1.330758	2.877474	2.70109	2.024292
DIO2	16.85209	17.46753	11.03717	11.30092	9.122472	9.452158	9.577664	8.865307
DIO3	9.831319	6.64023	8.255799	8.498508	6.55506	8.316072	5.600341	10.03911
DIP2A	7.355424	6.961797	6.155171	6.317502	6.297904	6.271608	7.18953	7.874159
DIP2B	8.907774	7.97655	7.207188	7.363595	7.839855	6.790982	8.127735	8.394236
DIP2C	3.518984	3.633044	2.851314	3.82847	3.978913	3.600843	4.193509	3.588494
DIPK1A	2.459212	3.262158	5.299633	3.694763	3.334955	3.283863	4.717206	3.4137
DIPK1B	0.477088	1.790183	0.907387	1.412394	1.714204	1.38767	1.273383	1.129053
DIPK1C	1.536398	2.301004	1.56673	1.420432	1.436384	2.303527	1.000078	1.863149
DIPK2A	22.64805	25.87846	21.30953	29.03323	26.45942	24.05204	19.49183	19.86771
DIPK2B	6.001427	4.727067	4.711369	5.337246	8.743734	5.707715	5.647466	6.116438
DIRAS1	0	0.007284	0	0	0.035953	0	0.007297	0
DIRAS2	0.091124	0.404788	0.013844	0.237045	0.245366	0.098804	0.049803	0.028437
DIRAS3	6.407561	14.08502	10.98678	8.915052	3.547203	8.135756	7.845164	8.297394
DIS3	10.59716	9.937257	8.650233	8.326935	8.760509	9.499799	9.698369	9.798188
DIS3L2	5.029579	4.809519	4.036267	4.626087	3.890044	4.47048	4.288219	4.703823
DISC1	0.53971	0.230229	0.374025	0.164953	0.312504	0.238332	0.384425	0.345722
DISP1	1.742935	1.613208	1.827465	1.957001	1.931464	1.500281	1.905582	1.647714
DISP2	0	0	0.006912	0.003586	0	0.003523	0	0
DISP3	0.005536	0	0	0	0	0	0.005619	0
DIXDC1	3.073769	2.404037	3.343088	2.544337	2.799278	3.214684	3.361654	4.13502
DKC1	35.94653	40.47256	34.09782	39.0351	36.72395	37.38805	37.34022	37.3238
DKK1	0.26115	0.855986	0.348919	0.582465	0.583823	0.433068	0.826387	0.389512
DKK2	6.702486	6.105255	6.113772	7.472724	5.081713	6.861764	8.422371	6.410956
DKK3	17.18212	15.78187	23.03727	20.77353	17.38085	19.88233	17.96061	16.30848
DKKL1	3.898145	4.660669	3.63438	4.788682	4.625105	5.143649	4.915052	3.266048
DLAT	11.12325	11.52602	12.12173	11.56204	11.29127	11.29402	12.46615	10.86294
DLC1	7.632483	7.375232	8.147072	7.811114	9.183913	7.097178	7.419876	8.818015
DLD	28.59005	33.75019	32.17397	32.29087	31.5024	34.85945	30.26309	26.90058
DLEC1	0.475775	0.583014	0.362453	0.510777	0.598194	0.775565	0.597891	0.680163
DLEU7	0	0	0	0	0	0	0	0
DLG1	24.10313	27.4282	23.32909	24.40191	25.18434	24.66393	25.38374	25.23343
DLG2	0.470815	0.670259	0.493006	0.461681	0.61701	0.796864	0.832166	0.880938
DLG3	11.97865	12.85239	11.94488	13.44526	11.21716	13.54632	13.10228	11.64084
DLG4	1.824632	2.028786	1.354256	2.046228	2.415941	1.640678	1.980127	1.792412
DLG5	18.6418	15.74509	11.79255	15.27122	16.88946	14.98106	15.36742	18.96854
DLGAP1	0.031372	0.021189	0.010327	0.037507	0.088899	0.015793	0.063685	0.031819
DLGAP2	0.107428	0.008372	0.008161	0.008468	0.024794	0	0.016775	0
DLGAP3	0.151648	0.256064	0.214654	0.279728	0.247724	0.132326	0.128269	0.071777
DLGAP4	8.973155	7.442222	7.354892	7.393153	8.073397	8.327246	7.730841	7.447668

DLGAP5	8.146367	9.367608	7.735784	9.026271	7.154705	8.229029	10.59211	9.604625
DLK1	0.018659	0.037809	0	0	0	0.037574	0.037879	0.11355
DLK2	1.706813	1.989245	3.142972	2.761723	2.78516	2.196544	2.11016	2.043491
DLL1	4.598362	4.254485	3.457514	4.875815	4.838382	4.187889	3.948507	4.421021
DLL3	0.239798	0.038871	0.179975	0.422668	0.191863	0.183491	0.321281	0.145927
DLL4	3.528928	3.665236	2.296722	3.252224	3.989739	3.943301	3.639237	3.685639
DLST	42.71773	38.87616	36.28046	38.13966	44.4361	39.12736	37.77603	39.60369
DLX1	6.705737	5.242998	3.814953	4.892452	5.503789	5.137047	5.807569	6.024974
DLX2	8.510397	6.961934	6.055313	5.877332	6.581872	5.375293	5.016509	6.11178
DLX3	193.0791	187.1885	193.19	167.986	166.9679	181.248	169.6958	167.3621
DLX4	14.88876	15.63058	20.98261	17.23566	15.18878	20.27787	18.82889	16.32431
DLX5	1.523358	1.852021	1.123268	1.394576	1.076645	1.942769	1.649289	0.865225
DLX6	0.014172	0.114862	0.041985	0.043568	0.014174	0.185491	0.043153	0.04312
DMAC1	22.39133	24.18242	26.40212	20.78905	30.18742	24.1224	21.18175	17.57761
DMAC2	15.01223	14.07747	17.81741	18.70868	18.91694	17.65703	17.00812	16.21927
DMAC2L	8.622024	13.06993	11.58753	11.80847	9.855016	8.729878	11.63009	9.681703
DMAP1	9.605332	10.97901	9.885227	11.2855	12.72079	10.50343	13.0885	9.26032
DMBT1	1.551915	2.216433	0.695399	1.127962	2.146979	5.107435	2.053997	1.511602
DMC1	0.377594	0.494127	0.26413	0.564298	0.330438	0.538579	0.239536	0.638282
DMD	5.429247	5.066298	5.779567	5.018839	5.147158	4.870107	4.517932	4.940191
DMGDH	0.013058	0.006615	0.006447	0.006691	0.071827	0	0.01988	0
DMKN	130.5504	110.7364	125.1039	125.0806	120.8284	118.1508	125.1617	104.6524
DMP1	0	0	0.009247	0.009596	0	0.009428	0.009505	0.009497
DMPK	9.154806	9.348564	9.718392	12.28539	12.55517	10.07893	10.55402	10.76715
DMRT1	0.0269	0.013626	0.013282	0	0	0.054167	0.054606	0.081848
DMRT2	0.119938	0.03314	0.032303	0.167603	0.174478	0.087824	0.143871	0.055293
DMRT3	0	0	0	0	0.019727	0	0	0
DMRTA1	0.017499	0.044321	0.129605	0.107593	0.00875	0.0969	0.159851	0.115361
DMRTA2	0.02095	0.095512	0.124134	0.182486	0.188574	0.084372	0.14885	0.18061
DMTF1	10.7982	10.58793	8.701104	9.335331	10.52162	8.941042	9.267485	11.68487
DMTN	3.266815	4.959763	3.839721	4.756292	4.566599	2.009499	2.207359	2.797702
DMWD	9.652194	7.930017	8.989106	7.98738	11.30266	7.47123	8.692307	12.49902
DMXL1	8.304559	8.674667	7.782585	7.68235	8.474174	8.224058	8.610237	8.970726
DMXL2	2.123132	0.91916	0.543875	1.226912	1.559294	1.647427	1.663493	1.643351
DNA2	1.274463	1.610439	1.369227	1.550016	1.379679	1.395975	1.4997	1.62641
DNAAF1	0.012254	0	0	0.012558	0	0.012338	0.012438	0.012429
DNAAF2	2.343698	2.207027	2.586498	2.283718	2.569386	2.48072	2.561842	2.311033
DNAAF3	0.434229	0.439928	0.771873	0.673835	0.446693	0.687022	0.314816	0.541076
DNAAF4	0.564475	0.653581	1.382745	0.743731	0.718516	0.937378	0.848252	0.72865
DNAAF5	6.977401	8.132945	6.104776	7.680183	6.652065	7.241347	8.0188	6.971829
DNAH1	0.051207	0.02594	0.016856	0.019678	0.021339	0.066595	0.032485	0.049772
DNAH10	0.52753	0.640124	0.60217	0.598151	0.840546	0.622012	0.502868	0.35398
DNAH11	0.118718	0.083581	0.081471	0.076294	0.044273	0.028363	0.065355	0.048979
DNAH12	0.002312	0.002342	0	0.009476	0.004624	0.011637	0.004693	0.009378
DNAH14	0	0	0	0	0	0	0	0
DNAH17	0.01109	0.011235	0.004694	0.001624	0.01426	0.001595	0.00804	0.009641
DNAH2	0.012134	0.008196	0.007989	0.012435	0.026294	0.014253	0.026685	0.010256
DNAH3	0.083227	0.059203	0.054211	0.030849	0.067299	0.064184	0.041339	0.016164
DNAH5	0.028462	0.034243	0.012297	0.052866	0.01957	0.023284	0.041528	0.054127
DNAH6	0.002199	0	0	0	0	0	0	0
DNAH7	0.006521	0.013214	0.002147	0.017821	0.002174	0.017509	0.015444	0.006614
DNAH8	0.004103	0.006236	0.012157	0.031538	0.032831	0.004131	0.006247	0.010404
DNAH9	0	0	0.002016	0	0	0.008222	0.002072	0
DNAI1	0.062756	0.042386	0.10329	0.075029	0.073225	0.031592	0.021232	0
DNAI2	0	0	0	0.010811	0	0	0.010708	0
DNAJA1	70.76199	64.24885	59.92194	52.28723	75.87762	62.3076	58.59651	69.8274
DNAJA2	69.77593	70.28326	74.46174	70.05845	71.66336	71.59014	74.09589	60.64076
DNAJA3	23.11643	23.83745	21.32529	23.90567	24.31397	23.35949	21.62861	22.09474

DNAJA4	11.1611	10.46896	11.27355	8.109764	9.943488	9.423986	11.19926	10.07451
DNAJB1	79.28506	67.07689	68.14289	69.17727	78.97888	69.21924	79.98194	70.33795
DNAJB11	21.7056	20.99405	22.6762	20.35349	20.56083	22.30774	19.88214	19.978
DNAJB12	16.95654	18.26195	16.78999	17.47867	17.00403	17.63781	17.77172	16.49972
DNAJB13	0	0.019692	0	0	0	0.01957	0	0
DNAJB14	5.899629	6.40117	5.508855	5.387315	5.910128	4.920538	5.375477	5.104833
DNAJB2	41.25799	32.0106	33.40306	33.18993	34.53274	37.3032	26.59345	33.87479
DNAJB3	0.066325	0.013439	0.0131	0	0	0.026711	0.026928	0.026908
DNAJB4	8.58765	8.028399	8.286998	8.918574	9.886592	9.505723	9.167216	7.066192
DNAJB5	1.004356	0.798893	1.090206	0.884631	1.436166	0.919289	0.985722	0.724002
DNAJB6	27.75197	27.05882	30.31076	30.07839	29.21958	29.73609	27.41159	27.65214
DNAJB9	8.891706	11.24201	11.18882	10.21916	10.92259	9.863866	10.92198	10.94337
DNAJC1	6.378531	6.025218	7.325618	6.616243	6.379356	7.005934	6.431143	6.225479
DNAJC10	17.75855	18.69689	21.75646	17.97462	18.6209	21.41873	22.43071	22.04695
DNAJC11	19.86967	18.12408	18.65355	19.32781	19.74019	21.07831	19.82313	19.76038
DNAJC12	4.376563	8.255688	6.133167	9.140801	4.835685	8.246379	9.307491	5.516877
DNAJC13	8.395325	8.85554	8.016353	9.114293	9.878339	8.599712	7.989806	9.703374
DNAJC14	25.95918	29.06874	26.33867	28.04308	28.73105	28.16268	25.89367	26.72089
DNAJC15	16.64569	22.88961	18.56982	19.90703	18.60711	19.20423	18.40751	19.95223
DNAJC16	5.005242	4.824723	4.550584	5.316002	5.893955	4.822989	5.094551	5.981827
DNAJC17	11.03798	11.66544	11.41951	10.65493	11.22011	10.66687	13.30422	7.896553
DNAJC18	3.220271	3.625925	3.670597	3.267725	3.741044	3.512129	3.216566	3.597922
DNAJC19	4.204678	4.741875	4.818901	4.421342	4.363832	5.052242	3.665145	3.678882
DNAJC2	16.69391	21.9577	18.63399	19.71248	19.80201	22.73164	21.14715	19.61616
DNAJC21	35.95	37.5259	38.0351	34.86568	36.77874	41.72194	38.18893	31.7305
DNAJC22	0.011895	0.349485	0.058734	0.048759	0.03569	0.011976	0.060368	0.012064
DNAJC24	10.81707	12.26042	12.08434	10.60004	14.19924	14.09019	12.38605	12.10245
DNAJC25	3.996549	3.752073	3.821771	4.655269	3.830521	4.084209	4.333691	4.154778
DNAJC27	1.675534	1.921041	1.689987	2.101996	1.86062	1.158673	1.749084	1.741719
DNAJC28	1.758314	1.514182	1.483837	1.801868	2.222157	1.673763	1.776577	1.450994
DNAJC3	18.39288	20.78016	21.63743	21.58383	23.5777	23.25697	21.4987	19.55895
DNAJC30	2.409604	3.506987	3.258603	3.455164	3.156269	3.159316	3.568236	2.671882
DNAJC4	7.519477	8.301844	7.806592	9.549845	10.83073	9.285611	8.154134	7.072458
DNAJC5	13.8551	14.24419	12.54379	14.58581	13.97469	12.39562	14.21394	13.68138
DNAJC5B	4.984545	3.835414	3.775945	1.53887	3.950289	2.528356	4.418893	2.802924
DNAJC5G	0.053313	0	0	0	0	0	0	0.054072
DNAJC6	3.560492	4.094428	3.717049	3.151138	3.320472	2.569894	4.505641	3.986365
DNAJC7	62.23387	69.07731	70.68152	67.01063	69.79924	77.93382	67.23217	67.73002
DNAJC8	52.78827	63.1606	63.79775	58.97483	57.82931	59.21752	56.94817	56.14113
DNAJC9	8.449138	9.068206	7.342157	8.258631	7.424609	7.003008	8.326972	8.987713
DNAL1	4.762778	5.114224	4.534448	3.752627	4.586549	4.875716	4.145271	5.142976
DNAL4	15.3629	17.74432	17.65035	16.69114	14.21346	16.87402	15.13359	13.62911
DNALI1	0.380879	0.452028	0.784506	0.836386	0.663903	0.504004	0.717957	0.286966
DNASE1	1.077604	0.780852	0.775228	1.652789	1.705832	1.005929	1.803631	1.447609
DNASE1L1	22.83787	21.33891	21.82278	21.58421	23.15021	20.48866	21.59685	21.77156
DNASE1L2	65.88204	53.24269	74.86903	71.96847	67.53829	62.25548	54.95682	45.40884
DNASE1L3	1.161123	1.733585	1.227122	1.586512	1.303885	1.599759	1.757471	2.479267
DNASE2	16.96256	13.93084	15.95353	13.37873	17.9229	18.45909	16.62594	13.54603
DNASE2B	0	0	0	0	0	0	0	0
DND1	2.185476	1.450655	1.384253	1.529114	1.673236	1.866551	1.636922	2.430603
DNER	0.081259	0.009147	0.017833	0.03701	0.01806	0	0.036657	0.018315
DNHD1	0.007317	0.003707	0.003613	0	0.007318	0.001842	0.001857	0.009277
DNLZ	9.494122	10.99283	11.85169	10.78225	9.289822	9.683124	10.17885	10.00444
DNM1	4.939264	5.757841	5.306307	6.854707	7.323733	6.068872	6.069164	5.596474
DNM1L	19.22378	20.58958	20.26514	20.09172	20.78293	20.35173	20.87715	21.57458
DNM2	27.93447	24.64397	23.99463	25.72594	24.88171	22.63241	24.59245	26.88294
DNM3	0.312989	0.253204	0.193757	0.356646	0.341062	0.390382	0.312942	0.298493
DNMBP	7.56673	7.703531	6.528839	6.540526	7.611453	6.856242	7.219213	7.920139

DNMT1	23.04769	21.53265	18.55425	18.36206	18.18913	17.20782	20.14417	21.89403
DNMT3A	3.012971	2.747894	2.180033	2.765114	2.48205	2.338692	2.431985	2.730368
DNMT3B	1.126325	1.026996	1.077531	1.233577	0.844853	0.914303	1.093204	1.063821
DNMT3L	0.01793	0	0.141655	0.036749	0.107596	0.018053	0.072797	0.018186
DNPEP	6.025525	6.29628	6.312794	6.638916	6.249925	6.389937	6.16828	6.076413
DNTT	0.015438	0.031281	0.045737	0.031641	0	0.139892	0.01567	0.046973
DNTTIP1	19.34735	20.712	19.97667	19.82659	19.22085	19.26308	18.35021	19.49195
DNTTIP2	26.17384	27.1186	28.08859	30.15818	28.07134	31.168	30.67676	28.5735
DOC2A	4.105222	2.205749	1.604513	3.618608	3.206604	0.872457	2.418723	1.065634
DOC2B	1.84174	1.591709	1.245122	3.199718	2.640829	1.940724	1.842565	1.854568
DOCK1	18.0526	18.15568	17.00008	18.1021	15.80065	17.53584	17.17529	18.44357
DOCK10	1.371608	2.149653	1.252769	1.80812	1.996494	1.299934	1.470614	1.884234
DOCK2	1.096641	1.207048	0.891339	1.604775	1.457863	0.790614	1.319218	2.380128
DOCK3	0.350839	0.618735	0.311179	0.459399	0.42561	0.421921	0.385777	0.388781
DOCK4	2.943947	2.40836	2.090728	2.596626	2.547527	1.963731	2.412819	3.148167
DOCK5	2.364464	2.546132	2.453185	2.434182	2.680314	2.617949	3.132426	3.832585
DOCK6	7.112493	7.06781	4.954302	7.621282	7.818743	6.773779	7.231382	7.071502
DOCK7	7.311116	8.125416	6.209001	6.784143	6.87066	7.047825	7.182113	7.991238
DOCK8	9.25857	9.858545	9.008825	8.946523	8.171011	8.217709	9.145586	9.563566
DOCK9	17.03992	15.14146	16.70264	16.02191	17.4218	16.12031	17.74168	17.95461
DOHH	8.557302	9.474523	8.450677	8.531804	8.558408	8.449113	8.36646	7.302549
DOK1	4.994403	6.944447	5.771076	6.79914	6.606355	4.83682	6.496459	7.709664
DOK2	0.686301	0.95227	1.149228	0.932639	0.865449	0.660947	0.908603	2.58757
DOK3	0.249717	0.390034	0.297982	0.149277	0.468281	0.335231	0.264024	0.823135
DOK4	18.24146	20.18273	17.25482	19.57827	18.3148	14.80482	16.60594	18.32117
DOK5	2.759771	5.473002	2.97666	5.776608	3.073333	4.906932	7.41018	5.240789
DOK6	0.262335	0.327112	0.099641	0.454949	0.222005	0.16254	0.061447	0.102335
DOK7	0	0	0	0	0	0	0.012615	0.012606
DOLK	11.84515	12.31074	12.28901	12.75231	13.53717	13.29288	12.7388	10.51543
DOLPP1	10.7119	9.633688	8.189753	11.16085	10.72609	10.25678	9.885346	8.904389
DONSON	7.739467	7.703848	7.889914	7.28003	8.584314	8.190872	9.00799	8.874429
DOP1A	5.258419	5.12558	4.592096	4.462608	4.917507	4.757028	4.430894	4.83892
DOP1B	7.507399	6.252628	6.637676	6.645889	7.856876	7.897833	5.918378	5.187444
DOT1L	7.079315	6.270902	5.128057	6.375318	6.354538	5.711036	5.888656	6.728938
DPAGT1	6.126891	5.240287	6.490425	6.35318	6.254964	5.537228	5.203857	6.094254
DPCD	16.99916	21.55224	20.15116	15.83957	14.49454	20.86836	17.41723	14.82935
DPEP1	1.258207	1.358899	1.406637	1.325865	1.448312	1.517772	1.252984	1.938255
DPEP2	2.093296	1.867961	1.875551	1.889434	0.956663	1.298056	1.308587	1.940312
DPEP2NB	0.039055	0.013189	0.038568	0.013341	0.01302	0.013107	0	0.013204
DPEP3	1.276817	0.595725	0.879317	1.205146	1.008143	0.930324	1.023133	1.192757
DPF1	0.156857	0.059593	0.116177	0.060278	0.147072	0.029612	0.139309	0.079545
DPF2	35.22431	32.13362	28.83125	31.86815	32.50703	31.87835	31.84503	31.60786
DPF3	0.129676	0.141888	0.194651	0.098337	0.103754	0.12795	0.157945	0.157826
DPH1	4.709167	5.720034	4.000422	4.436636	4.988311	4.116807	3.841832	5.148544
DPH2	3.752334	4.066594	3.794656	3.697385	4.411367	4.132153	4.055813	3.220251
DPH3	13.55255	16.16968	13.84807	14.4858	14.10927	17.17715	16.24735	13.80271
DPH5	5.540871	5.534894	4.975786	4.813135	5.25156	4.760163	5.413741	5.82458
DPH6	4.570074	6.049934	5.85704	3.684177	4.712863	5.603354	6.122985	4.758735
DPH7	13.75082	12.12022	11.59194	14.60385	13.57756	13.42941	12.9674	13.05906
DPM1	57.48689	65.6648	61.74533	53.80378	61.99364	59.91381	58.78667	59.2342
DPM2	8.913539	8.183565	8.909692	8.0885	9.218725	8.810466	8.308917	8.357717
DPM3	20.99187	31.3678	31.6764	26.65185	33.13305	33.2304	31.803	30.14613
DPP10	0.015	0.022795	0.08147	0.046113	0.007501	0.015102	0.030449	0.030426
DPP3	10.64743	11.18273	10.15329	11.48632	10.91727	11.8749	11.83089	10.51026
DPP4	24.71143	22.58265	19.68274	26.99104	19.25623	22.97543	26.35439	21.5888
DPP6	0.142197	0.156069	0.087766	0.163934	0.088885	0.119307	0.084193	0.132203
DPP7	9.808388	8.729072	8.179436	9.801704	10.86115	9.746304	8.745236	10.19509
DPP8	11.77842	12.63494	10.61314	12.34251	11.50466	11.43842	11.73031	12.55254

DPP9	9.739496	7.527785	7.834022	8.630492	10.27738	7.775075	8.217309	9.389058
DPPA2	0	0	0	0	0	0	0	0
DPPA4	0	0	0	0	0	0	0	0
DPRX	0	0	0	0	0	0	0	0
DPT	34.1747	29.70524	61.58169	46.08494	66.97809	42.59748	39.53202	48.0317
DPY19L1	6.233024	6.653223	5.931731	5.151705	6.041323	6.452302	7.039043	8.31417
DPY19L2	0	0	0.008137	0.008443	0.016481	0	0	0
DPY19L3	3.055924	3.074629	3.080429	3.38417	3.204205	2.467112	3.380493	3.792156
DPY19L4	4.916892	5.194976	5.258376	5.095035	5.453735	4.553705	4.916227	5.670085
DPY30	37.56093	39.32457	40.54797	35.37955	41.72509	38.81448	34.37221	35.58494
DPYD	7.187988	5.272098	7.121531	8.857468	7.020427	5.069734	7.314807	7.200134
DPYS	0.849172	0.781077	0.739285	1.099208	0.435816	1.214962	1.281524	1.14457
DPYSL2	15.21043	16.89489	20.26819	17.65161	17.83106	14.81694	18.36919	19.28509
DPYSL3	35.17385	25.45833	34.81111	27.3552	28.85975	26.88148	26.82009	33.71966
DPYSL4	0.047279	0.011975	0.035017	0.012113	0.011821	0.047602	0.035991	0
DPYSL5	0.009786	0	0	0.020058	0.009788	0.009853	0.0298	0.019851
DQA	0.099252	32.80605	9.188968	6.611207	31.07	26.90626	0	22.97685
DQB	24.56827	0.382588	8.59924	32.55232	0.244381	0.067096	45.77006	26.08956
DQX1	0.373189	0.158269	0.068565	0.231238	0.164919	0.209714	0.361169	0.220059
DR1	23.41721	22.66717	22.83262	18.75906	20.86411	23.34433	22.72118	22.7582
DRAM1	9.477373	8.311547	9.204358	8.690797	10.4846	10.48053	9.5727	7.15072
DRAM2	7.697916	9.273045	8.239038	8.98301	8.817189	9.143286	8.788239	7.691188
DRAP1	56.10897	61.17988	60.63475	56.09313	57.58609	61.95079	59.5031	55.54961
DRAXIN	0.393844	0.159605	0.311149	0.188346	0.210077	0.185049	0.479702	0.23967
DRC1	0.848861	1.11918	0.849768	0.804348	1.959611	2.563981	0.418994	1.031955
DRC3	0.69631	0.365321	0.2947	0.509684	0.5223	0.287938	0.441721	0.315278
DRC7	0.575187	0.681504	0.423608	0.329684	0.234004	0.5693	0.494758	0.237305
DRD1	0.035664	0.245698	0.028176	0.014619	0.028535	0.078997	0.028959	0.043406
DRD2	0.020595	0.020866	0	0.063317	0.041196	0.062208	0.020904	0
DRD3	0	0.023455	0.015241	0.015816	0.007718	0.023309	0.007833	0.007827
DRD4	0.025284	0	0	0	0	0	0	0
DRD5	0.084178	0.127924	0.02771	0.057509	0.126283	0.042377	0.085441	0.099606
DRG1	38.20649	40.42649	38.54854	37.55604	35.31154	40.7116	40.46131	39.33054
DRG2	13.6866	13.5618	12.10659	14.04101	13.2977	12.05569	13.64791	13.19574
DRGX	0.07068	0	0.0349	0	0	0	0.03587	0.035843
DROSHA	5.030081	5.115017	4.469435	4.931394	5.105445	4.362442	4.707453	5.688887
DRP2	0.715632	0.667482	0.504797	0.690676	0.836905	0.758644	0.564952	0.691257
DSC1	116.1511	124.0244	115.226	110.6073	123.6556	106.93	126.1905	119.37
DSC2	28.07697	21.18397	23.83037	27.31021	24.64463	27.41785	26.77131	31.02599
DSCAML1	0.02027	0.032858	0.028025	0.045699	0.008109	0.036736	0.037034	0.037006
DSCC1	6.527171	8.421354	7.333995	6.842458	8.204311	7.587024	8.063509	10.19964
DSE	8.401845	8.80897	7.554879	7.996363	9.020888	7.279182	8.062364	8.708807
DSEL	2.459849	2.180302	1.845799	2.088356	2.244189	1.794387	2.111276	2.07444
DSG1	194.0519	167.7679	141.6856	160.1449	154.6455	154.5686	199.9027	198.1766
DSG2	22.34337	23.91199	13.37891	20.64065	16.99951	20.71828	24.74725	23.33024
DSG3	24.30682	18.84264	20.34289	26.92119	25.56386	23.50315	18.90703	18.80437
DSG4	91.77053	85.5405	105.077	98.42174	100.2022	114.6002	87.86703	93.83522
DSN1	5.658664	5.446281	4.749932	5.363918	5.33398	4.522248	4.7815	5.76074
DSP	517.9035	412.7188	408.6904	408.7434	416.0451	419.5509	442.5703	445.5385
DSPP	0	0	0	0	0	0	0	0
DST	18.46851	15.70863	13.47465	15.60872	17.58385	14.06112	17.34243	22.0052
DSTN	218.5548	230.4757	264.894	242.8393	231.9538	250.6246	248.9446	207.3482
DSTYK	6.235235	6.613911	5.528083	6.252725	6.344003	5.713707	6.057457	6.28229
DTD1	35.03585	34.73948	35.85666	32.93422	33.17419	35.78355	35.40542	32.23942
DTD2	11.02942	12.14201	14.53082	13.7564	12.81762	15.42676	11.44671	10.79635
DTHD1	0.004996	0	0	0.00512	0	0	0.005071	0
DTL	3.389971	3.857794	2.975022	3.252936	2.999467	3.345274	3.830735	4.196964
DTNA	0.35088	0.641848	0.574305	0.565992	0.601122	0.546271	0.738667	0.579944

DTNB	5.637457	5.766185	4.597843	5.924756	4.40427	4.787399	6.105925	5.270158
DTNBP1	16.15701	15.98325	16.35861	19.5955	15.83265	15.58273	17.08957	16.58013
DTWD1	8.450337	11.92309	10.5229	11.46876	9.007987	11.33025	9.685838	10.5147
DTWD2	1.642109	1.485411	1.795396	2.028357	1.96492	1.845231	1.413753	1.992635
DTX1	0.560433	0.839339	0.665744	0.615932	1.283476	0.588795	0.717232	1.062682
DTX2	16.46421	14.02461	12.82541	15.27364	14.47305	14.62457	12.8742	14.08394
DTX3	8.099397	8.109362	6.410732	8.787216	7.348322	7.449784	7.325978	7.031149
DTX3L	3.596768	3.174624	2.814874	1.800072	2.496828	3.368688	3.402549	3.674074
DTX4	0.966746	1.299633	0.908801	2.086171	2.584521	0.926555	0.811416	0.688242
DTYMK	15.55725	17.06315	15.26053	16.44082	17.1916	18.04103	19.45629	17.15231
DUOX1	2.554393	1.862416	1.884378	2.170202	2.061247	1.978341	1.529034	2.183322
DUOX2	0.376756	0.243403	0.167158	0.425257	0.365883	0.142936	0.221686	0.182753
DUOXA1	5.467953	6.842402	3.796549	8.132244	5.936288	4.406861	3.981215	4.439269
DUOXA2	0.078787	0.019955	0.038903	0.080739	0.118196	0.118988	0	0.039954
DUPD1	0	0	0	0	0.013622	0	0	0
DUS1L	16.44395	17.18967	17.57447	17.33498	18.15576	19.42057	17.99841	14.87956
DUS2	2.435709	2.569286	2.263868	2.65021	2.457518	2.308093	1.868726	2.223345
DUS3L	3.989091	4.107833	3.882762	4.10469	4.431986	3.917377	4.630908	4.120648
DUS4L	4.467248	4.562277	4.931987	5.130197	5.138598	5.667426	3.999385	5.247517
DUSP1	26.72629	48.73034	21.43183	28.52773	28.61057	25.49916	32.89537	29.37609
DUSP10	6.046514	3.528327	3.375929	3.711205	4.658341	6.593347	5.36735	4.58319
DUSP11	32.52686	34.96758	35.39321	36.15143	35.80425	43.68564	37.76319	33.15282
DUSP12	8.667697	9.900787	8.520729	9.691115	8.142637	8.700425	9.491918	9.271329
DUSP13	0	0.017255	0.050459	0.058179	0.022712	0.011432	0.040337	0.011516
DUSP14	133.2709	113.6719	135.6702	140.5674	152.7389	166.058	131.9902	97.27179
DUSP15	5.036774	4.090914	4.785129	5.466438	5.547545	5.092581	4.465196	5.130031
DUSP16	9.859977	9.768542	9.796661	8.982578	10.19058	8.998108	10.61042	11.64668
DUSP18	2.074979	1.681769	1.648407	1.819234	1.900004	1.810601	2.166947	2.160638
DUSP19	5.334568	6.129869	6.339963	5.30105	6.105137	6.804832	6.211539	5.184096
DUSP2	15.34629	17.87656	12.3095	14.13387	14.76542	13.96795	13.30902	14.53038
DUSP21	0	0	0	0	0	0	0	0
DUSP22	6.968188	5.425307	5.165128	7.12375	5.829751	5.785097	6.507231	5.296321
DUSP23	3.859417	4.080071	4.109606	5.605806	4.195561	4.020941	5.416105	5.582216
DUSP26	4.201723	2.018195	3.80221	2.916279	2.377377	3.236024	2.854493	1.765736
DUSP27	0.182186	0.08887	0.026654	0.069148	0.033743	0.067937	0.089035	0.109499
DUSP28	6.928852	6.656364	5.826394	7.613187	6.410489	6.596013	7.214833	6.108362
DUSP3	14.85043	13.48253	15.24666	14.58043	15.123	14.16852	14.24227	13.16109
DUSP4	6.121954	6.875629	5.906938	8.848375	8.133038	8.64996	8.577544	6.795489
DUSP5	20.70828	20.15914	18.79274	17.78123	20.01634	21.32758	28.54999	25.253
DUSP6	18.47438	16.45724	13.69732	16.32272	17.10776	14.36968	18.12968	16.04812
DUSP7	33.65071	31.50107	29.86064	36.56431	35.13891	32.5701	30.80183	28.93228
DUSP8	7.391929	5.947477	7.180873	8.237682	7.881094	8.380709	6.704911	6.40409
DUSP9	0.212218	0.621119	0.31436	0.31413	0.282994	0.510428	0.347035	0.286985
DUT	46.95434	58.03189	54.08288	49.4818	42.75853	43.67064	55.76693	49.96339
DUXA	0.031734	0	0	0	0	0	0	0
DUXB	0	0	0	0	0	0	0	0
DVL1	10.65591	10.12543	9.500049	11.08936	11.42349	10.15896	10.99247	11.14313
DVL2	9.841623	8.528768	7.000266	7.66721	7.098872	8.636073	8.525552	8.348179
DVL3	17.19216	14.19027	13.28123	16.60884	16.58919	14.12602	14.16235	16.02895
DXO	10.75483	9.971937	10.60206	9.755543	12.01048	10.67525	10.93544	9.366176
DYA	0.092373	0.374343	0.684169	0	0.970047	0.162758	0.257837	0.234221
DYDC1	0.076543	0.051698	0.214169	0.156878	0.051035	0.141287	0.090639	0.284652
DYDC2	0.078898	0.05995	0	0	0.019727	0	0.02002	0.040011
DYM	9.314636	9.070076	8.682576	8.622026	9.776694	9.162867	9.05764	8.984054
DYNC1H1	45.48337	38.2995	32.60904	36.20189	41.49224	34.57748	40.38971	45.98602
DYNC1I1	0.425178	0.672403	0.501808	0.552608	0.736379	0.334113	0.631545	0.410195
DYNC1I2	70.40163	76.4343	71.40945	65.91109	67.1643	73.15387	70.67024	67.98739
DYNC1LI1	25.49599	29.46872	31.32851	29.06097	27.40789	32.02315	30.91874	29.2409

DYNC1LI2	32.67435	30.02758	32.69821	28.95212	34.36032	30.79098	32.52606	36.56345
DYNC2H1	1.167369	1.193481	1.245386	0.943057	1.227174	1.010195	1.301638	1.134294
DYNC2LI1	6.689995	7.20651	7.820506	8.259889	7.819438	7.75011	9.101516	6.846543
DYNLL1	683.1658	598.2346	668.3217	563.0355	656.5356	638.0517	638.6811	649.8303
DYNLL2	31.34123	33.64859	35.44243	33.16145	36.43919	34.93031	35.28583	33.60141
DYNLRB1	157.2226	146.2297	159.3599	148.571	151.8457	158.5399	151.1195	146.0226
DYNLRB2	7.70987	7.242978	6.644774	7.278355	7.710867	11.99665	9.438049	7.061358
DYNLT1	65.71692	69.62969	65.5221	51.87984	68.92247	65.52966	63.68446	65.18217
DYNLT3	71.66067	85.93441	91.46889	83.44969	84.41184	88.81248	82.30745	78.34234
DYRK1A	21.02867	20.26073	18.50309	19.37844	20.70015	17.74241	17.73161	22.02652
DYRK1B	4.625641	4.250409	3.711504	3.954777	4.175719	4.122471	4.101324	3.675455
DYRK2	13.46545	14.46939	11.9688	12.71452	12.16244	10.78809	13.51928	13.92547
DYRK3	2.221362	2.032284	2.167094	3.228329	2.706373	2.819393	3.238544	2.990325
DYRK4	0	0	0	0	0	0	0	0
DYSF	3.060231	2.959821	2.816567	2.883255	3.939497	3.003526	2.225933	3.045159
DZANK1	0.482488	0.518445	0.640114	0.359592	0.589784	0.559388	0.371004	0.291637
DZIP1	23.77145	27.37979	24.54629	23.46984	21.73199	24.45109	24.87478	25.48002
DZIP1L	1.154996	1.45013	0.888692	1.474061	1.289794	1.041604	0.805521	0.812101
DZIP3	1.608732	2.152889	0.951391	1.955623	1.535177	1.893543	1.478466	1.519429
E2F1	3.89082	3.30389	2.498631	3.595391	3.194034	2.683306	3.595354	3.387352
E2F2	4.953892	5.012277	5.047268	4.694348	4.751639	4.368386	5.154404	5.269993
E2F3	9.63958	8.953324	8.513449	8.136268	7.806904	9.147724	8.394744	8.698387
E2F4	19.95749	21.64409	18.03971	21.28322	21.16363	19.5629	21.24499	18.33538
E2F5	13.44693	19.34251	17.09013	13.83182	13.46552	16.55873	15.37609	14.50998
E2F6	5.023696	4.8715	3.889148	4.798797	4.683408	4.732858	5.06263	4.986029
E2F7	2.478532	2.116883	1.987529	1.850799	2.036887	1.803892	2.033045	2.528431
E2F8	2.341299	2.304639	2.410654	2.119831	1.596547	2.102813	2.356165	2.293676
E4F1	9.333382	8.254421	6.636302	8.686883	9.213788	8.711704	8.637496	8.341435
EAF1	66.86655	66.39349	65.62394	59.20171	68.62315	71.39499	67.35808	67.32676
EAF2	0.661161	1.425554	0.786856	1.163977	1.542909	1.126532	0.929186	0.85971
EAPP	11.51322	9.441387	9.628446	10.05685	7.189708	8.924856	9.321198	9.184702
EARS2	4.543349	4.990311	4.855511	5.403018	5.148609	5.290534	6.154682	4.923645
EBAG9	7.433375	7.75039	8.072577	7.383836	6.522147	8.081052	7.787885	8.753328
EBF1	4.325984	5.706667	4.192883	5.221147	6.558228	5.139249	5.544855	5.698574
EBF2	0.785311	0.986118	0.786449	1.082464	1.001125	1.024538	1.212477	1.2957
EBF3	2.129244	2.407627	2.069425	2.844066	2.95548	2.607618	1.950196	2.29061
EBF4	1.409867	1.121606	0.878363	1.289644	1.154537	0.905049	1.306161	1.410743
EBI3	0.350412	0.251466	0.20186	0.149622	0.321253	0.367506	0.237112	0.133275
EBNA1BP2	50.38962	52.22956	51.72159	47.59372	43.30098	51.3999	51.88637	42.98629
EBP	30.82175	35.01917	40.31627	48.87996	41.80097	49.89901	44.85526	25.67542
EBPL	22.05926	25.01886	36.95799	26.65687	36.34844	25.39418	22.7379	21.81195
ECD	18.42098	18.99924	20.273	19.47947	20.36536	17.89087	20.36994	18.21679
ECE1	44.08239	35.23245	32.94686	36.64367	38.36867	33.5416	40.0234	41.45423
ECEL1	1.857214	2.026325	0.952307	1.537214	1.79792	1.198655	1.595061	1.654233
ECH1	55.0718	68.92809	73.61079	87.95182	89.9169	76.23533	60.79756	44.63865
ECHDC1	36.07262	44.47713	56.78448	59.7422	44.76772	49.73904	34.95497	39.36749
ECHDC3	7.770539	10.97105	8.394623	9.955546	5.176416	8.708397	7.026751	8.761911
ECHS1	113.6637	142.4188	150.7182	139.0555	131.7336	141.5829	139.5278	118.1891
ECI1	13.65128	14.05549	14.0994	13.84457	13.69344	15.39145	12.58523	11.94082
ECI2	52.27606	46.90449	70.45381	90.48635	84.62633	68.904	61.72194	57.84538
ECM1	15.83558	16.68029	16.56947	14.37191	18.54709	13.17093	5.98564	23.22622
ECM2	29.625	34.73514	35.57892	35.12292	37.21876	29.83305	41.54367	46.87177
ECPAS	25.17638	25.80332	28.00551	25.98944	24.31074	26.86208	26.3264	26.55968
ECSCR	25.66442	25.21616	27.36711	35.16769	42.36745	35.64808	28.29664	31.56882
ECSIT	9.250501	8.668086	7.456232	8.430509	9.416253	9.019192	8.906807	8.937183
ECT2	5.478672	6.292963	4.483874	5.488057	4.59583	4.936909	5.929258	5.924793
ECT2L	0.324109	0.172	0.20576	0.126529	0.177511	0.20201	0.328971	0.430471
EDA	2.997192	2.762072	2.692322	3.035994	2.51912	2.565018	3.124054	2.963863

EDA2R	0.360685	0.104405	0.132299	0.095045	0.113373	0.114133	0.062759	0.41808
EDAR	1.049701	1.220384	1.087603	1.463661	0.955179	1.394725	1.397307	1.23045
EDARADD	5.785995	6.147878	4.250585	5.278538	4.540006	5.091412	4.249414	4.747171
EDC3	7.785209	7.762883	6.717363	6.637265	7.489804	6.892256	7.997369	7.42682
EDC4	12.70735	11.59089	9.419034	10.99967	12.30834	10.558	11.41503	12.49987
EDDM13	0.595514	0.80444	0.261375	0.542458	1.125005	0.06662	1.074572	3.288399
EDEM1	16.28671	14.20274	16.079	17.23029	16.00831	15.71635	18.33726	17.36706
EDEM3	8.762439	10.19791	10.02647	9.35381	10.02144	11.08325	9.171941	10.34382
EDF1	88.49379	94.20969	105.3316	94.1883	96.9431	102.863	96.57186	81.79004
EDIL3	10.71002	10.2056	9.864423	13.4348	10.66633	12.69478	9.932858	11.09677
EDN1	10.19315	7.94379	8.83553	9.297768	9.046192	10.85487	9.02437	9.329994
EDN2	9.8732	7.953278	6.351034	6.311999	8.817576	5.715114	5.209849	6.951442
EDN3	0.564151	0.53468	0.341462	0.372984	0.291212	0.384778	0.609556	0.461437
EDNRA	23.77386	19.65553	22.99332	21.79604	22.63246	23.67763	22.12157	19.23299
EDNRB	27.12458	27.73778	29.87658	24.20325	19.35956	22.16985	34.72598	33.69767
EDRF1	10.24375	10.89709	8.654207	8.878058	9.651607	11.58408	11.34205	9.990462
EEA1	10.93183	11.51831	10.64684	10.53611	11.95491	10.87333	11.83802	12.21297
EED	11.94173	12.64514	12.17522	11.88892	13.25727	10.98397	11.93035	12.17122
EEF1A1	2294.977	2289.576	1934.557	2268.688	2269.495	1970.81	2403.016	2643.428
EEF1A2	0.075606	0.045959	0.179192	0.10847	0.257092	0.13702	0.092088	0
EEF1AKMT	4.593156	4.573628	3.907597	4.160479	4.163464	4.331688	4.163865	4.191459
EEF1AKMT	3.862734	4.780729	4.835268	5.156639	4.834263	5.728573	5.404174	4.75421
EEF1AKMT	2.798916	2.728238	2.366187	2.694413	2.820485	2.828712	2.313612	2.935537
EEF1AKNM	4.418779	4.357535	3.919952	4.637876	3.252984	4.459738	4.550219	3.808888
EEF1B2	367.1719	412.2651	469.62	532.8426	412.116	508.4379	376.5565	380.01
EEF1D	170.9348	182.226	180.1247	179.4276	171.8377	179.0022	173.1217	165.3153
EEF1E1	14.16158	13.9583	13.73775	13.58823	13.94628	16.74681	12.50945	13.85505
EEF1G	806.6245	842.4325	808.0185	800.1033	824.2578	772.6464	830.6139	748.9068
EEF2	504.7382	495.8506	467.9742	481.5117	447.2329	437.1991	512.0412	484.2681
EEF2K	19.71074	22.99832	22.15974	19.8624	19.16581	21.02399	22.4511	21.08037
EEF2KMT	3.157178	4.310187	3.60431	3.786085	2.776884	3.471822	3.863624	3.769874
EEFSEC	8.675558	10.11948	8.901604	9.944549	9.502386	9.061871	8.077608	7.700894
EEPD1	5.686687	7.821791	5.235592	7.790299	7.1389	5.745417	6.29307	6.328384
EFCAB1	0.275939	0.460897	0.493447	0.596118	0.201387	0.270315	0.393623	0.317687
EFCAB10	1.482477	1.922474	1.522565	1.033055	2.72811	2.447871	1.26396	1.022435
EFCAB11	7.608104	10.52794	7.055178	8.84244	5.722282	16.53456	13.78072	17.28351
EFCAB12	0	0	0.012257	0.063597	0.012414	0.012497	0	0.037766
EFCAB13	0.023208	0.011756	0	0.023783	0	0.03505	0.035335	0.058847
EFCAB14	31.78373	27.08326	29.2567	24.02019	24.04671	26.34124	27.53389	25.96681
EFCAB2	0.116379	0.218969	0.238067	0.161855	0.207847	0.225979	0.16875	0.143329
EFCAB3	0	0.018656	0	0	0	0	0	0
EFCAB5	0.067363	0.082871	0.090282	0.162716	0.101057	0.135646	0.131862	0.102482
EFCAB6	0.221433	0.235556	0.109337	0.107786	0.132877	0.072457	0.073045	0.247044
EFCAB7	4.355293	4.373519	4.199825	4.253144	4.266177	4.539813	4.238597	4.144461
EFCAB8	0	0	0	0	0	0.007631	0	0
EFCAB9	0.019992	0.010127	0.014807	0.005122	0.014996	0.025161	0.020292	0.020277
EFCC1	0.055169	0.149049	0.077182	0.047113	0.110353	0.069433	0.083995	0.121235
EFEMP1	64.45047	58.08292	68.83515	47.76323	61.90965	50.03552	78.72901	66.26607
EFEMP2	16.30673	20.81825	16.12071	20.30932	22.70719	18.97362	21.58025	24.15592
EFHB	1.146449	1.051353	0.624642	0.830495	1.215788	1.781175	0.782451	0.120286
EFHC1	1.290824	1.307765	0.933968	1.191828	0.626323	0.759197	0.972911	0.803667
EFHC2	0.741368	0.761386	0.802334	0.95747	0.843034	1.165661	1.350355	0.566516
EFHD1	194.7802	191.3612	241.4135	206.0601	180.4376	248.5228	206.4176	161.511
EFHD2	58.12086	43.82709	49.61821	51.08271	53.13674	44.82674	49.08724	49.69088
EFL1	13.04372	11.94857	10.77598	10.56929	11.86434	11.55009	11.24685	11.06728
EFNA1	49.2099	43.3938	48.03356	45.16912	44.17306	43.27538	43.10857	47.27716
EFNA2	3.819968	4.059242	3.588007	3.193481	3.676835	3.571345	3.90642	3.859783
EFNA3	51.41217	44.11361	53.81878	53.10089	48.48861	54.66616	46.36235	43.49816

EFNA4	22.44414	20.28649	19.31787	22.6393	19.98227	20.67004	17.66629	19.95167
EFNA5	14.47048	11.5079	10.22571	12.1386	12.47436	13.32671	12.79785	14.22032
EFNB1	34.0869	31.58451	27.80317	30.63404	31.25266	28.77956	31.38279	32.51064
EFNB2	41.8112	35.65476	40.83381	41.07238	41.04006	40.68844	34.13208	37.78764
EFNB3	1.110217	1.164254	0.865566	1.257478	1.081141	1.03936	1.393761	0.898842
EFR3A	33.44982	32.06609	33.40817	33.87829	36.73624	33.18432	33.29824	35.3601
EFR3B	0.768025	1.221329	0.68165	1.305107	1.098709	0.959249	1.095311	1.508616
EFS	17.41571	16.89889	14.26133	19.44118	18.10123	16.32832	14.19917	17.70628
EFTUD2	26.82775	27.3384	23.29669	24.03992	25.68614	24.45672	26.70346	24.92061
EGFL7	10.23792	10.56348	7.6876	12.59466	13.86073	10.59288	8.978825	11.07751
EGFL8	1.275017	2.11847	1.118107	2.090559	1.785254	1.468584	1.397673	1.500074
EGFLAM	3.643489	2.420529	2.589446	3.52563	2.735957	2.850512	3.091889	2.544344
EGFR	28.62097	25.50943	23.38408	24.37545	25.88282	23.49517	25.25276	31.02605
EGLN1	19.13381	17.71802	19.41366	19.27345	19.85974	19.81671	18.36508	18.9986
EGLN2	13.85693	13.98714	12.4256	15.09878	14.8377	12.1961	14.44771	15.14006
EGLN3	77.01975	80.78906	87.25841	74.25584	63.59151	75.62381	76.195	62.90135
EGR1	74.58632	80.79516	57.44871	67.78693	62.04038	47.917	52.34428	52.28747
EGR2	20.78026	16.55778	15.80896	18.18749	16.96871	16.60479	17.52313	16.54765
EGR3	31.50067	29.05134	26.77348	28.45053	25.32452	28.02155	31.17959	25.7304
EGR4	0	0	0	0	0	0	0.012803	0
EHBP1	6.283386	7.721827	5.786376	6.412853	6.442581	6.655485	6.538374	7.668126
EHBP1L1	6.018437	5.460378	4.920693	6.947254	7.3668	6.235114	6.097987	6.634672
EHD1	12.03736	12.45065	11.96203	14.49618	13.71075	11.93353	12.8318	12.31095
EHD2	30.26545	32.58137	31.29104	33.45044	36.83501	28.62957	30.66398	37.79781
EHD3	2.342868	2.746825	1.557002	2.416001	3.367387	2.29953	2.19106	2.047435
EHD4	10.78231	10.55267	10.80995	12.80317	14.42019	10.18435	13.13618	14.12448
EHF	14.21854	15.73485	11.47998	13.10832	12.6004	11.76717	13.71815	14.6745
EHHADH	4.618674	5.815688	5.382127	5.003542	5.424344	5.739708	5.337571	5.025718
EHMT1	15.27157	13.99797	12.14566	13.5153	13.6928	12.56208	13.09428	15.40405
EHMT2	15.23965	14.69963	12.38737	14.69849	14.37162	12.57587	14.65945	14.19717
EI24	13.83171	15.08701	15.97354	16.86471	16.17717	17.96456	14.13788	13.23966
EID1	60.74708	68.90186	66.8541	70.04526	59.2119	72.27105	66.59348	61.16929
EID3	1.79188	2.668633	1.911117	2.497321	1.93548	1.894327	1.418631	1.272172
EIF1	328.5271	340.7374	351.4556	318.5391	320.3618	328.4231	331.3288	299.187
EIF1AD	12.52722	11.11359	12.4743	9.888492	12.14889	11.99124	11.54869	10.87533
EIF1AX	132.8191	145.6144	171.8188	120.8499	130.6861	165.0336	160.5533	141.8169
EIF1B	52.85204	57.51976	57.46527	55.40038	58.40433	59.06297	54.36327	46.39534
EIF2A	42.39136	45.5576	58.72649	47.13949	46.16907	49.29103	43.46853	48.57313
EIF2AK1	21.82827	22.63245	20.49942	21.70698	20.93432	20.70573	24.24013	22.44215
EIF2AK2	4.447716	3.950792	3.318624	3.591063	3.900118	3.528201	4.067549	4.483693
EIF2AK3	6.233529	7.553395	6.204618	6.539729	7.258988	7.201996	6.997322	7.029611
EIF2AK4	6.440367	6.371877	5.976594	7.003408	6.743299	5.8381	6.24133	6.46093
EIF2B1	27.96164	24.42506	28.34705	28.00559	30.64893	33.22347	25.9927	24.60539
EIF2B2	33.35605	29.69921	30.1414	31.34497	31.65429	32.96298	31.36578	30.06451
EIF2B3	19.14655	20.65139	22.81598	19.7192	20.59987	20.63052	21.978	16.98496
EIF2B4	39.29936	35.20266	36.80205	35.66602	35.88231	39.45252	37.78179	34.8202
EIF2B5	36.34077	37.33224	39.70921	41.37974	39.94131	44.6081	39.84788	35.47633
EIF2D	11.6157	11.558	11.54778	13.17879	11.29306	10.78137	10.85569	11.67587
EIF2S1	21.10933	25.2333	23.36947	23.55141	24.78092	25.04418	23.84945	24.30146
EIF2S2	138.3185	151.3251	167.0147	163.855	157.8088	188.4731	156.2884	143.2196
EIF2S3	128.3026	141.969	145.4899	129.2453	124.4801	141.4438	136.7974	130.3589
EIF3A	98.77843	95.08018	91.94102	95.17364	90.5153	98.69535	97.20398	92.41564
EIF3B	66.38916	66.18493	68.31658	67.71103	62.86111	71.11066	70.34256	65.68579
EIF3C	131.895	125.2881	127.617	121.2025	113.7059	117.1635	130.3235	118.1972
EIF3D	100.9162	103.4932	102.7394	95.90285	96.68796	100.0135	106.9533	101.9009
EIF3E	280.11	337.457	331.685	333.3917	282.2757	331.6966	290.4257	305.2314
EIF3F	79.84389	72.00738	85.28942	67.24918	70.08373	69.29717	82.78399	81.31259
EIF3G	213.0718	246.5098	222.7999	229.584	212.3716	215.5286	229.8569	215.121

EIF3H	257.7297	269.991	288.8197	263.1499	236.2637	294.5773	275.218	250.858
EIF3I	124.4522	122.4453	128.6647	127.4177	124.6285	134.6344	130.0124	115.1056
EIF3J	16.44879	15.1842	19.574	11.97983	14.46481	12.71323	13.04456	11.85667
EIF3K	258.2017	285.6348	266.3164	261.3873	256.8389	263.6682	256.9943	238.7391
EIF3L	112.9087	125.5916	113.7331	116.551	97.25746	101.5771	116.8441	94.36788
EIF3M	229.1092	303.343	295.2816	269.12	241.5848	268.5639	270.3304	257.7439
EIF4A1	328.5749	340.3156	321.7793	356.5475	335.2247	342.2679	367.2392	329.3453
EIF4A2	234.334	256.517	264.7139	249.2239	253.3773	241.2376	215.5622	251.7874
EIF4A3	84.20531	84.64321	77.3209	85.22254	80.42894	79.12607	89.3491	80.13469
EIF4B	175.2095	172.7006	188.6216	171.4507	159.9184	172.7728	183.6442	178.6779
EIF4E	43.28413	46.91369	49.32469	46.61535	52.62891	51.65986	47.57879	46.97772
EIF4E2	23.33195	26.07093	26.16022	23.77667	25.88185	25.81661	22.68839	24.69567
EIF4E3	5.126965	7.135537	6.558009	5.441749	5.490187	6.228881	6.687801	7.058519
EIF4EBP1	20.99187	28.77162	31.90476	26.77676	28.02431	30.33833	32.8879	23.4965
EIF4EBP2	107.1019	94.26014	99.63387	89.25444	101.2692	87.5264	96.16545	106.9378
EIF4EBP3	11.52495	9.077181	10.73855	10.24094	9.650043	11.14108	10.80397	8.854138
EIF4ENIF1	13.90647	13.87163	12.41662	12.26444	14.59027	14.51062	13.24405	13.64595
EIF4G1	116.6996	101.4258	106.9494	110.4802	111.054	112.5163	110.3525	108.5384
EIF4G2	382.8603	363.7289	379.1023	345.3323	375.3952	372.2145	377.6118	390.0032
EIF4G3	20.19297	21.59764	16.4455	17.74304	20.55835	17.64047	18.50127	19.76795
EIF4H	218.2813	185.5569	183.6362	203.5371	213.2432	196.0683	203.1043	202.0083
EIF5	119.9936	118.649	133.1514	127.1993	115.8296	141.0394	117.9746	117.5672
EIF5A	302.9669	298.5625	315.3624	291.1499	288.8171	301.8417	334.8103	298.812
EIF5A2	3.344244	4.098549	2.993624	2.813524	4.019006	3.041243	3.646251	4.185107
EIF5B	36.91112	33.52874	39.3237	33.90303	37.12252	38.63032	33.35906	29.95918
EIF6	61.14706	63.25869	65.27279	73.40513	67.42164	64.04407	73.10121	60.60804
EIPR1	10.56195	10.29038	11.29954	13.65574	14.94709	13.25718	15.9555	15.72923
ELAC1	5.160358	6.195668	4.812099	5.961701	4.513971	6.188205	5.498348	5.275481
ELAC2	15.28129	19.02976	14.72409	16.08574	17.12292	18.66226	17.88914	14.18932
ELANE	0.030893	0.250388	0.122033	0.094975	0.123588	0	0.062713	0.031333
ELAVL1	16.67773	16.2765	15.26572	15.94821	15.99655	15.56871	15.52165	16.95846
ELAVL2	0.285326	0.068826	0.087215	0.090503	0.095121	0.061559	0.124117	0.041341
ELAVL3	0.023208	0.005878	0.017189	0.011892	0.023211	0	0.005889	0.035308
ELAVL4	0.193279	0.275365	0.2028	0.154739	0.199344	0.176355	0.32492	0.116393
ELF1	19.41044	21.21128	18.33662	18.96378	19.09518	18.47186	19.15433	20.8293
ELF2	15.81852	14.76957	13.40102	15.95887	16.1655	15.87984	15.71247	15.64009
ELF3	1.347234	1.901679	1.644373	2.156226	1.619918	1.447883	1.93593	1.427825
ELF4	3.657965	3.234655	3.121504	3.480818	4.059973	2.919415	3.02072	4.265897
ELF5	35.25245	28.70847	47.62557	30.01757	27.72132	33.68965	32.68468	31.21756
ELFN1	0.045089	0.079942	0.055659	0.034655	0.056369	0.034048	0.08009	0.068597
ELFN2	0.086867	0.036669	0.021446	0.051927	0.072398	0.058307	0.033064	0.066077
ELK1	11.1268	9.90733	9.13251	10.44465	9.898327	9.845797	9.39644	10.09781
ELK3	11.85716	11.28432	11.25111	12.53267	14.76126	11.81312	11.51089	13.14634
ELK4	12.06907	13.38658	12.68454	10.26187	12.93472	10.74132	11.95082	14.11306
ELL	7.32645	5.665561	5.131023	6.187694	6.293774	5.658881	5.597019	6.066649
ELL2	10.33461	10.20004	12.16235	11.2885	12.53174	12.17457	11.48542	11.08551
ELL3	0.223333	0.152728	0.104761	0.251751	0.245698	0.112429	0.170012	0.198198
ELMO1	4.70427	4.608403	4.473593	5.828318	6.055353	4.642431	4.736939	5.036308
ELMO2	7.289332	8.402801	6.975475	6.8474	8.318389	6.52367	7.60419	8.617385
ELMO3	13.76344	12.91157	14.17298	14.27663	12.9246	12.78907	13.30872	12.40359
ELMOD1	1.867335	1.860135	1.648329	2.191541	1.095506	1.943112	2.520068	2.772103
ELMOD2	6.580879	7.323146	8.274124	6.473361	7.216644	7.227035	6.896504	6.795686
ELMOD3	5.561414	5.196256	5.162441	5.605871	5.258606	5.477183	5.721846	6.01765
ELMSAN1	12.94082	11.27501	10.18173	11.64157	11.61185	9.991362	10.23016	11.57956
ELN	14.85814	13.83917	18.38614	15.03988	14.546	10.19219	12.68214	20.82764
ELOA	34.29124	33.50094	31.87773	33.37227	33.05959	34.13224	32.84107	33.02329
ELOB	73.63727	80.40675	83.93399	70.25794	74.37225	82.32497	75.09725	69.282
ELOC	21.56921	23.7123	22.61178	22.13898	23.01552	23.98354	20.6856	22.40326

ELOF1	32.25668	36.59292	35.57467	33.00682	37.26807	39.12607	34.67641	34.19087
ELOVL1	79.39113	63.70674	88.4417	83.46508	103.5994	92.27882	58.40554	46.46402
ELOVL2	0.133315	0.369651	0.152441	0.201331	0.238595	0.332032	0.313361	0.220611
ELOVL3	174.5921	197.3502	260.8991	233.2783	294.8769	251.6842	166.5656	138.4135
ELOVL4	65.42315	72.5169	106.4196	92.96824	105.6331	130.1757	58.14774	40.27415
ELOVL5	3.92377	4.969081	4.095306	4.749311	4.65861	3.926919	4.608638	5.80263
ELOVL6	23.22213	26.01716	27.58949	27.88981	29.76919	29.60526	26.44916	25.04618
ELOVL7	51.1032	52.94948	66.68615	57.7081	56.40616	67.04012	42.20247	40.59886
ELP1	10.48953	9.401979	8.354875	8.89566	9.00976	9.008226	10.02793	9.9788
ELP2	15.77913	13.15056	12.58659	13.35948	13.50793	12.52041	11.90699	12.60295
ELP3	6.4864	7.179217	6.749964	6.818608	6.284948	8.068589	6.307606	5.864273
ELP4	4.231285	6.017419	5.014275	4.400333	3.730281	4.559982	5.853591	3.560372
ELP5	24.66809	25.88648	24.43655	22.34793	24.55608	24.50803	25.77854	22.39077
ELP6	6.514804	6.113966	7.081828	6.856886	5.722011	6.756582	6.085513	6.776461
EM4B	0.209472	0.17477	0.139936	0.208347	0.234146	0.049624	0.293908	0.212454
EM4C	0.158897	0.047117	0.003827	0.059573	0.046513	0.042922	0.019668	0.027515
EMB	1.303445	2.924079	7.654269	5.295224	3.56166	2.741874	1.429309	2.785645
EMC1	19.80129	18.88262	19.29434	21.1809	20.5109	17.84649	19.77867	21.02834
EMC10	65.15148	62.94134	63.82125	67.58642	62.5209	67.15441	63.91324	63.13651
EMC2	51.94095	71.51859	71.914	72.11229	64.55681	68.13166	68.0559	61.27201
EMC3	25.9601	25.1218	26.83006	28.88122	30.2907	29.21686	25.82705	23.62103
EMC4	53.90776	56.73767	62.56982	55.26804	55.18159	66.02399	51.84828	47.48562
EMC6	19.13217	23.96476	23.99378	19.93514	22.96157	26.51003	21.42897	19.59451
EMC7	21.61398	23.24742	24.17177	21.40466	21.16527	22.74828	22.53051	21.40796
EMC8	11.60303	13.63338	13.85411	13.76665	13.18385	14.30909	13.33342	11.51288
EMC9	7.32359	9.256265	7.39942	7.331615	7.397057	7.787323	7.556108	6.864016
EMCN	12.28309	11.88676	12.68437	14.66268	18.43803	15.75796	14.50054	16.11943
EMD	31.60135	30.3896	33.73174	33.16342	34.52091	31.17919	32.48275	33.20815
EME1	3.611861	3.323551	2.737863	3.780562	2.518688	3.69214	3.217594	2.733456
EME2	5.509473	4.416715	3.586581	5.652603	5.04234	4.474321	4.02263	4.856469
EMG1	32.98723	31.8393	36.06737	24.2102	34.89344	33.23713	28.11204	26.33519
EMID1	4.610598	5.181843	3.920479	4.800142	2.867553	4.811283	4.647777	4.207544
EMILIN1	5.342359	4.676033	4.634613	4.722554	5.900647	4.732256	5.744836	5.568724
EMILIN2	8.68846	7.184664	7.518541	7.260813	8.425501	5.589234	8.301912	10.13843
EMILIN3	0.382431	0.589247	0.503554	0.416398	0.43029	0.609652	0.363907	0.387875
EML1	3.579629	3.845398	3.056796	3.742066	3.730626	3.51184	3.473909	3.803156
EML2	6.467157	6.328446	5.382158	5.762059	7.418039	4.772405	6.145382	6.257536
EML3	12.59681	12.36971	10.83498	12.87432	13.75217	10.81774	12.88832	12.41744
EML4	7.874482	9.444126	7.917411	9.450236	9.980077	8.771914	8.074748	8.266641
EML5	2.142667	1.231052	1.346526	1.135892	1.335861	1.017949	0.941475	1.514633
EML6	1.176276	1.317525	1.021953	1.36802	1.235077	1.010659	0.889313	1.14754
EMP1	410.4227	399.3161	436.9482	390.6523	375.8725	397.6781	406.0396	395.0367
EMP2	108.255	104.5766	107.8549	115.6994	110.2005	106.1073	97.35986	91.81578
EMP3	22.24206	23.79425	30.98183	24.83264	26.52472	22.11846	25.65649	30.20442
EMSY	5.618112	4.729973	4.337447	4.449017	5.558916	4.115922	4.415956	5.553183
EMX1	0.058863	0.089453	0	0	0.029435	0.029632	0	0
EMX2	2.045413	2.43323	3.036406	2.826325	2.454813	2.750277	2.61186	2.288676
EN1	1.534253	1.243511	0.757568	1.347649	1.052195	0.617894	1.06784	1.067036
EN2	0	0.016102	0	0.016288	0	0	0	0
ENAH	18.67882	16.65662	16.54694	16.81831	17.11155	17.47008	14.72568	19.06264
ENAM	0.005819	0	0	0	0	0	0	0
ENC1	19.58308	17.23966	16.15334	17.43186	17.14106	15.87569	17.48474	18.90338
ENDOD1	57.88257	52.83555	68.63553	52.32966	56.19162	65.32466	59.61264	54.22331
ENDOG	1.707672	2.004365	2.920341	1.472537	2.561839	2.138689	2.494243	1.689739
ENDOU	36.51693	26.92057	39.43758	39.09359	34.4609	39.46451	40.70345	32.83299
ENDOV	4.946725	5.236528	5.177359	5.166743	5.412502	4.618687	4.50596	4.770576
ENG	19.26024	16.33892	18.9923	23.71187	29.55003	23.10677	19.49168	17.09382
ENGASE	20.48816	19.08183	22.65519	17.84238	20.16225	22.18267	19.98411	20.09123

ENHO	23.06258	17.68771	18.79183	19.05854	14.62769	17.17484	19.31437	13.05393
ENKD1	1.951314	2.86242	2.208022	2.916155	2.297156	1.432553	2.537623	1.690475
ENKUR	0.768511	0.607305	0.576791	0.819049	0.399677	1.005889	0.499225	0.498849
ENO1	146.9247	159.2727	153.2919	159.5829	168.809	143.3871	170.454	160.9823
ENO2	0.746546	0.551234	0.537314	0.700205	0.771953	0.420412	0.565098	0.243836
ENO3	0.514636	0.604338	0.369617	0.527383	0.994311	0.894989	0.427382	0.98461
ENO4	0.304337	0.039785	0.03878	0.352117	0.088367	0.425028	0.139504	0.139399
ENOPH1	20.59549	21.83805	20.56137	19.31833	20.84018	22.71906	21.57356	21.77738
ENOSF1	3.982948	3.28755	3.50462	3.303099	2.594135	2.677081	2.996219	3.00497
ENOX1	1.734745	1.772281	1.360427	1.292205	0.874774	1.394341	1.035745	1.478521
ENOX2	3.835841	4.415398	3.31069	4.018741	3.150163	3.351816	3.45023	3.550428
ENPEP	0.556561	0.504511	0.03616	0.120073	0.153806	0.361287	0.14866	0.393653
ENPP1	10.83957	12.309	11.03248	13.35805	13.95026	11.93533	12.04152	14.66163
ENPP2	11.10298	16.47295	10.6101	14.45495	19.72159	13.54472	14.26413	15.31263
ENPP3	4.396573	7.073834	7.491327	12.47512	10.03192	9.977595	5.687927	5.112218
ENPP4	1.686028	2.329303	1.967751	1.980229	2.436297	2.76678	1.805775	1.754446
ENPP5	2.613293	2.817212	3.28522	3.737299	3.239737	3.356726	4.24842	2.931048
ENPP6	0	0	0	0	0.012858	0.025889	0.01305	0
ENPP7	0	0	0	0	0	0	0	0
ENSA	65.90277	76.34788	81.13728	67.9923	64.86296	81.41995	74.38405	71.90773
ENTHD1	0.086687	0.010978	0.074906	0.022209	0.032512	0.05455	0.021997	0
ENTPD2	16.50297	24.47246	15.04029	23.46468	19.89	26.83845	23.49937	24.52969
ENTPD3	4.757729	3.500051	4.402149	7.405611	3.918132	5.748278	3.541337	2.26927
ENTPD4	10.32343	8.503465	8.730084	9.849375	9.209768	9.602787	10.14928	10.88383
ENTPD5	1.476851	1.035854	1.058778	1.047761	1.306614	1.172395	1.109839	1.325043
ENTPD6	6.347634	6.045917	4.777481	4.652349	5.269834	4.384763	4.785223	6.823443
ENTPD7	3.213694	2.703837	2.624577	2.119562	2.680278	2.250399	3.013589	2.875979
ENTPD8	0.020371	0	0.030175	0.073063	0.132426	0.030765	0.082705	0.123963
ENTR1	13.50237	12.30054	11.34181	12.06831	13.47981	11.88152	12.28631	12.06751
ENY2	36.8524	42.79253	42.42385	39.59049	35.03339	45.55827	40.89175	39.65663
EOGT	2.864464	3.252395	2.777803	4.046116	3.902368	3.658304	3.813711	3.920767
EOMES	0.08066	0.081719	0.066379	0.19287	0.107561	0.094747	0.102338	0.149983
EP300	15.7533	14.09545	11.94781	13.14194	14.21217	12.04627	12.217	14.85028
EP400	8.578984	8.496987	6.812444	8.325202	8.127306	7.203365	7.811837	8.768364
EPAS1	59.03639	46.09279	54.40439	52.98383	58.28829	57.6679	48.41215	52.28484
EPB41	10.42872	11.19313	10.01795	10.02631	11.01295	9.221482	12.36267	12.27626
EPB41L1	11.72167	13.28358	12.00215	12.35384	11.00893	13.04176	13.33856	9.789279
EPB41L2	9.004051	9.605889	9.248191	10.05195	10.85956	9.803327	10.36179	11.00147
EPB41L3	2.960969	3.217722	3.729184	4.05942	4.632115	3.086949	3.751653	4.682231
EPB41L4A	8.779693	7.66477	7.973394	8.273993	8.510968	7.324598	6.952147	8.662589
EPB41L4B	16.88266	13.933	15.90543	17.2419	15.38908	17.5144	17.03372	14.20596
EPB41L5	2.310328	2.553839	2.801057	2.169885	2.459699	2.566667	2.525195	2.207604
EPB42	0.049255	0.049901	0.01216	0.10095	0.012315	0.012398	0.012498	0.012489
EPC1	12.22206	13.25767	11.28619	11.72479	11.78206	10.48227	12.53529	12.62969
EPC2	9.392633	10.39547	9.238323	8.767847	9.135878	9.254108	9.814483	9.494439
EPCAM	129.6385	122.103	130.8145	127.7317	133.8	115.9311	134.9482	102.4928
EPDR1	0.334399	0.191979	0.308216	0.673941	0.501663	0.628475	0.871164	0.76876
EPG5	5.676897	5.389177	5.046407	4.70346	5.410174	4.612345	5.124773	5.719385
EPGN	3.765952	4.102968	3.550831	3.839843	2.820098	2.896157	3.092528	3.627625
EPHA1	34.9821	29.49464	26.14401	35.46488	27.59214	30.83708	32.60759	31.86761
EPHA10	0.010444	0.026452	0	0.016054	0.010445	0.026288	0.079503	0.010592
EPHA2	19.82761	15.81772	14.08159	17.0227	16.63349	15.33493	15.84021	16.35838
EPHA4	15.73839	15.1667	13.67634	15.8206	14.73816	12.77007	15.63391	15.69826
EPHA7	0.249276	0.316574	0.211498	0.230266	0.221217	0.293398	0.302906	0.31336
EPHA8	0.411185	0.306651	0.118435	0.117047	0.142791	0.149498	0.110135	0.202727
EPHB1	3.982394	2.180017	2.299403	2.764575	2.344777	3.395226	3.072345	3.224754
EPHB2	0.549481	1.120342	0.685075	0.950217	1.325793	1.1203	0.745954	0.828988
EPHB3	22.83874	19.44897	14.70094	19.00994	18.60487	18.1833	19.49253	19.47031

EPHB4	16.71467	14.53172	12.69086	15.3675	15.22414	14.38292	16.51945	17.43754
EPHB6	14.18118	10.45753	11.19018	11.65551	12.63068	10.96473	11.71656	15.44974
EPHX1	19.4494	23.91329	22.75795	24.53712	23.57932	28.81118	19.78262	19.6434
EPHX2	16.37102	19.31321	17.85879	24.78862	21.66768	24.10829	15.11312	17.19194
EPHX3	15.04883	15.20444	15.60983	17.15864	15.28508	15.76214	15.24658	13.04069
EPHX4	0	0.018607	0	0	0	0	0	0
EPM2A	0.376295	0.435695	0.371606	0.55088	0.304659	0.369845	0.481971	0.463434
EPM2AIP1	20.55746	21.86505	18.79417	21.39392	21.27352	18.76047	20.06604	20.38322
EPN1	27.46806	27.84028	25.90217	29.37075	28.56028	26.78127	27.9506	28.43459
EPN2	20.6907	17.83159	16.70375	16.93489	17.73254	18.8685	17.40401	18.64016
EPN3	4.102394	3.528878	3.246118	4.378518	3.710695	3.335509	3.744918	3.485647
EPOP	1.088312	1.083748	0.881848	1.344044	1.004726	0.889709	1.085754	0.943423
EPOR	0.01584	0.048143	0.031285	0.032465	0.047526	0.031896	0.016078	0.144589
EPPK1	3.134547	5.454475	1.230549	2.564703	2.089381	2.64562	3.508475	3.684338
EPRS	68.06234	69.53192	74.21659	68.87678	70.25493	79.86078	72.32019	66.26883
EPS15	31.3445	33.06126	33.5092	32.3796	32.50045	31.02383	34.28084	38.88494
EPS15L1	10.76565	11.49819	9.304469	10.03296	10.70561	10.98609	10.35039	12.92045
EPS8	15.71746	17.39918	16.24885	16.68122	17.43984	15.86356	18.77571	17.83074
EPS8L1	8.200069	5.415835	7.291093	8.506548	7.172205	7.728023	7.084335	6.853945
EPS8L2	13.84305	12.76042	13.30965	15.3369	14.5402	14.34148	12.59405	13.50672
EPS8L3	0.022728	0.034539	0.056111	0.046581	0.022731	0	0.046137	0.115256
EPSTI1	3.217145	3.549089	2.047438	3.718097	4.200705	3.185138	4.045471	3.444218
EPYC	0.023639	0	0	0.024225	0.047285	0	0	0.023976
EQTN	0.016493	0.03342	0.065151	0.033804	0.082477	0.116242	0.117185	0.167281
ERAL1	11.88623	10.57177	11.07427	11.61702	11.26129	10.79837	10.82395	10.95526
ERAP1	5.18104	4.74381	5.458619	6.277651	7.176694	5.374989	5.637045	5.222851
ERAP2	4.106211	4.052465	3.152759	4.572646	4.260811	4.203791	4.345731	4.337071
ERAS	0.056339	0.034247	0.055637	0.057734	0.259192	0.068069	0.045747	0.148566
ERBB2	40.06148	36.63644	33.72832	39.47289	35.49916	35.26861	34.96445	36.19234
ERBB3	57.306	53.43888	51.24581	53.6524	50.08747	54.78567	52.99984	51.36805
ERBIN	28.85344	27.69978	28.48982	26.95794	26.30141	26.65338	25.63811	28.25696
ERC1	12.73369	12.06914	9.692304	9.653918	9.700924	11.61399	11.71821	12.44206
ERC2	0.071779	0.033937	0.014177	0.068654	0.047859	0.07227	0.058285	0.077655
ERCC1	26.06707	26.32165	25.91289	29.03657	27.02081	26.37561	26.65536	24.44488
ERCC2	8.811936	7.732706	6.467422	7.811771	7.029363	7.346785	8.360169	7.760641
ERCC3	14.99094	13.49675	10.94828	13.44458	15.62011	14.05454	14.01456	9.530932
ERCC4	6.422335	5.578485	4.221925	5.231885	5.430581	4.371657	5.675989	5.11035
ERCC5	11.19923	11.17183	10.03401	13.25865	10.57149	10.5049	11.41541	11.57535
ERCC6	8.006293	6.074446	5.699785	6.777989	6.319216	6.692425	6.646657	6.856778
ERCC6L	2.300294	2.159124	1.763855	1.878885	2.124664	2.172961	2.348532	2.44283
ERCC6L2	4.142852	5.509756	4.245883	5.310487	5.049902	5.308307	5.139438	4.115192
ERCC8	9.893645	8.309516	7.33835	7.483337	7.731748	5.972428	7.564141	9.448056
EREG	4.605164	5.693763	6.333209	7.008939	5.194273	5.100287	5.470594	4.013361
ERF	19.99226	17.4222	16.00289	20.35774	19.70996	16.1668	16.06238	16.33919
ERFE	0.138825	0.140646	0.137095	0.109433	0.117482	0.150525	0.075873	0.194955
ERG	6.451845	5.952337	6.317589	6.140539	7.044954	7.162773	6.556532	5.761294
ERGIC1	20.48795	22.60595	19.93556	23.55436	23.1986	17.63924	25.25358	22.21374
ERGIC2	17.88609	19.76072	21.04002	19.98788	21.0047	21.59364	19.71516	20.98631
ERGIC3	83.805	89.1645	86.93292	82.96684	88.22399	76.32014	91.30876	89.75676
ERH	99.21214	121.4263	115.2286	110.4494	105.1406	116.4666	118.3399	109.7159
ERI1	26.02408	27.95294	37.56631	26.95444	28.01934	34.94473	26.6705	31.76773
ERI2	2.585016	2.858703	2.52584	2.229304	1.747842	2.46521	2.337391	2.714132
ERI3	11.64315	12.01253	11.03365	11.85853	11.17431	11.96661	11.7744	10.27678
ERICH1	3.756778	3.841542	3.663882	3.180298	3.582236	3.711963	3.599974	3.774759
ERICH2	1.376516	3.09907	1.585925	2.664491	1.567902	3.503298	2.445037	2.016606
ERICH3	0.011893	0.020083	0.019575	0	0.04758	0.043907	0.008048	0.004021
ERICH4	4.509186	5.971506	6.774923	4.587874	9.857067	11.09055	4.2826	3.364699
ERICH5	0.040432	0.010241	0	0.031075	0	0.040708	0.020519	0

ERICH6B	0.049958	0.012654	0.012334	0	0.024982	0	0	0
ERLIN1	18.91755	19.95029	19.11648	19.97917	18.55317	18.35745	19.20958	18.03779
ERLIN2	22.07741	25.7887	19.29937	20.01378	17.85336	21.15309	21.82746	19.63841
ERMAP	0.031431	0.031843	0.01552	0	0	0.015823	0	0
ERMARD	3.85812	3.540956	2.896198	3.479536	3.922692	3.210785	2.890031	3.068345
ERMN	0	0	0	0	0	0	0	0
ERMP1	17.78954	21.46016	23.45784	21.35027	25.49692	24.07484	19.80573	18.82581
ERN1	2.770745	2.814334	2.644662	3.292509	3.38096	3.105621	3.004137	3.388863
ERN2	0	0.008582	0	0	0	0	0	0
ERO1A	10.25346	10.55648	9.620932	9.743834	12.09633	9.833679	10.58853	10.22454
ERO1B	4.083526	2.872998	3.276523	3.806893	3.516824	3.369082	3.238108	3.307573
ERP27	0	0.039576	0.019288	0.120094	0.019534	0	0.099124	0.05943
ERP29	95.05551	98.6297	108.3646	99.91059	95.85734	92.52556	102.3453	88.43832
ERP44	16.2228	18.86594	19.0912	17.64403	18.7206	16.89449	17.18733	17.36464
ERRFI1	6.775543	6.850798	5.332245	5.702623	6.65499	6.444915	7.561813	6.283653
ESAM	11.8137	9.392246	10.27937	12.63665	17.07959	12.78761	12.23103	10.72221
ESCO1	3.963694	4.060532	3.67403	4.039209	3.915539	4.035312	3.906406	4.307271
ESCO2	2.251544	2.506785	1.775649	1.622459	1.981297	1.962533	2.083434	1.629988
ESD	23.86641	30.11959	27.96776	27.00525	30.43775	30.49571	33.05622	28.78684
ESF1	8.749736	9.918869	10.66318	10.27177	11.85655	11.62587	9.100683	9.490682
ESM1	1.115318	1.02234	0.472037	0.680324	0.7702	0.828835	0.754698	0.700264
ESPL1	2.131242	1.755174	1.846364	2.01265	1.929945	1.968784	2.198031	2.270313
ESPN	3.97559	3.225626	2.7865	3.418073	3.066317	3.460004	3.137558	2.767856
ESPNL	0	0	0.008676	0.027008	0	0.01769	0	0
ESR1	3.574383	6.721474	3.813955	4.315101	4.298534	4.21762	4.760643	4.186741
ESR2	0.01767	0.017902	0.087249	0.036215	0.053017	0.035581	0.14348	0.017921
ESRP1	79.06109	68.46701	74.52029	70.67737	71.7032	82.81595	76.63842	71.65804
ESRP2	23.72599	21.12033	22.71228	26.23667	22.93731	22.95523	19.2905	19.12729
ESRRA	5.788214	5.576414	5.208164	6.183329	5.228493	5.333093	5.547781	5.333382
ESRRB	0.00588	0	0	0	0	0	0.005968	0
ESRRG	0.007104	0.007197	0.021046	0	0.01421	0.035762	0.050473	0.04323
ESS2	10.33709	9.812797	8.299506	10.34145	9.985979	9.612077	9.386568	9.563624
ESYT1	15.80074	16.41759	13.91999	17.10464	17.67234	13.24452	14.96729	17.09534
ESYT2	29.01899	27.32713	25.07977	28.17364	28.10314	26.05111	28.00769	30.74236
ESYT3	9.863993	9.783701	8.586929	9.122817	9.391041	8.855536	8.568117	7.843677
ETAA1	13.3177	14.37541	11.02107	13.43082	11.30543	14.22649	13.84381	15.26649
ETDB	0	0	0	0	0	0	0.07762	0
ETF1	59.54226	64.05112	56.86613	57.63255	63.85181	63.57597	65.27678	54.33687
ETFA	75.66467	95.04183	87.4847	86.60565	84.72983	94.62175	62.29007	68.53035
ETFB	57.54478	52.92513	60.4178	62.3624	67.17381	63.26449	66.50766	56.55039
ETFBKMT	1.612279	1.225079	1.019001	1.470472	1.644737	1.688225	1.260077	1.70064
ETFDH	15.5092	20.253	18.53151	15.04463	15.11406	18.0825	14.0951	14.78641
ETFRF1	5.810616	9.493621	9.011422	7.799618	7.530222	10.50584	9.490434	5.416057
ETHE1	28.83818	31.39571	32.81077	27.20339	29.91746	32.73938	28.8686	27.4691
ETNK1	8.39194	8.181319	7.254422	8.423216	9.771822	8.182961	7.936134	9.039811
ETNK2	6.051355	7.506524	8.332485	4.107145	6.078508	9.172227	6.53019	8.811792
ETNPPL	1.427929	2.165566	2.785669	2.55853	2.724806	2.434361	1.138138	1.323867
ETS1	17.29762	17.51962	17.00873	15.02125	20.88067	14.67354	16.19494	17.57902
ETS2	40.50522	37.69651	35.69049	34.84839	36.61994	37.18376	36.5144	35.91754
ETV1	0.410703	0.73175	0.470852	0.725648	0.519347	0.537086	0.666023	0.76128
ETV2	1.038559	1.192481	1.572615	1.365832	1.523417	1.254785	0.860879	0.790008
ETV3L	0.025916	0.026256	0	0.026558	0.051838	0	0.026305	0.026285
ETV4	4.013089	2.84603	2.799456	3.989995	2.553338	2.793966	3.336628	2.468111
ETV5	21.78734	18.77833	17.54638	16.74346	17.3406	19.25706	19.06318	20.82662
ETV6	11.82179	12.15747	10.91858	12.72056	12.39443	11.93517	13.08434	14.40945
EVA1A	1.13742	1.604534	1.293867	1.327892	1.324761	1.101702	1.315232	1.620897
EVA1B	18.49228	17.27428	16.0333	19.84968	20.75165	20.63826	18.38791	17.29324
EVA1C	4.407985	3.685095	3.460125	3.864341	4.120817	4.076016	3.577198	4.189361

EVC	6.298071	6.805558	6.82668	5.932577	6.604261	6.004981	6.557831	6.317533
EVC2	3.057317	3.564002	2.31179	3.015067	2.974553	2.749776	2.940877	3.827397
EVI2A	1.102441	2.331413	0.961865	2.127877	1.75557	0.883668	1.683899	2.073435
EVI2B	2.504472	3.043094	2.982959	3.294842	3.554102	2.027488	2.533451	4.582522
EVI5	5.163698	5.168029	5.276619	5.056327	4.951445	5.291438	5.046252	5.351519
EVI5L	4.939264	5.233173	4.221775	5.162989	5.194318	4.410965	4.403718	4.930743
EVL	27.35257	31.61294	24.45589	28.06219	26.88643	26.38364	30.1038	29.99645
EVPL	22.94456	18.51523	18.09028	19.21893	18.4288	16.84479	19.66642	21.39356
EWSR1	94.20199	102.0334	85.799	109.1547	92.62767	96.8094	94.99484	82.35028
EXD1	0.048996	0	0.012096	0.012553	0.036752	0.036998	0	0.024847
EXD2	2.260601	2.328715	2.103948	2.005495	2.044021	1.943098	1.958862	2.133332
EXD3	1.440041	1.903254	0.911436	1.918425	1.374762	1.324662	1.461642	1.560123
EXO1	1.658356	1.2869	0.679468	1.437287	1.314505	0.897012	0.743129	1.753536
EXO5	4.308941	3.712588	3.256952	3.522181	5.838675	4.262043	3.616857	4.126776
EXOC1	12.63029	14.47975	12.26682	15.74724	13.35088	14.49877	13.37679	14.61323
EXOC1L	0	0	0	0.253081	0.041166	0	0	0
EXOC3	18.69233	17.81467	15.45067	17.97189	17.64422	16.38937	16.95233	19.32924
EXOC3L1	0.296232	0.338845	0.20761	0.313363	0.401399	0.279014	0.310375	0.174454
EXOC3L2	2.735994	2.86297	2.407856	2.71165	2.888055	2.590649	2.263825	2.547021
EXOC3L4	0.101557	0.157765	0.12035	0.339971	0.203141	0.163602	0.178673	0.199139
EXOC4	12.73238	11.92643	11.52271	12.33619	12.50037	11.70861	13.33175	12.0909
EXOC5	18.08481	16.85691	19.60015	16.39565	19.85647	18.10435	16.44904	18.33571
EXOC6	5.503236	5.905714	5.226368	5.521653	5.900283	6.113577	6.117764	6.106674
EXOC6B	13.48904	13.30682	10.84133	11.70182	12.48916	11.20268	13.97834	12.99094
EXOC7	25.71587	25.00538	24.68276	26.94451	26.52518	23.84493	25.88186	23.8251
EXOC8	7.807408	8.173739	7.48283	7.206307	8.553518	7.946121	7.340478	8.041415
EXOG	6.038835	6.033297	4.393221	6.046574	6.184489	5.295771	4.518649	5.383688
EXOSC1	25.65245	28.43454	23.17738	22.05129	22.65104	23.9921	22.38838	22.2413
EXOSC10	14.12915	15.05681	13.84974	15.03072	14.56462	13.68374	14.50043	15.23256
EXOSC2	12.55214	13.63628	13.18816	12.01139	12.28625	11.6761	12.96343	13.76751
EXOSC3	8.212826	10.20243	9.189494	9.741215	12.16602	9.809043	9.500473	8.145046
EXOSC4	6.273994	4.940156	4.815403	5.513296	5.283191	5.907732	5.889671	5.341222
EXOSC5	15.68274	14.87229	14.54504	11.98446	11.8431	12.26733	12.46619	10.24832
EXOSC6	11.34933	13.85576	12.9639	13.36265	13.5024	13.7034	12.32171	14.13814
EXOSC7	20.9786	25.04734	20.45496	21.57988	22.54827	22.69937	22.82963	20.38844
EXOSC8	16.09082	18.00697	19.65152	19.00062	16.59488	21.22446	18.51979	19.13469
EXOSC9	17.87146	21.13325	21.79484	19.10872	18.40336	20.1642	19.27205	18.83556
EXT1	25.56073	20.79084	19.59466	22.36332	20.10971	19.01779	21.45187	25.58836
EXT2	20.30533	19.16228	15.93051	18.77686	19.67134	15.57336	21.44981	20.92641
EXTL1	0.026669	0.020264	0.019753	0.013665	0.053345	0.033564	0.013535	0.047335
EXTL2	3.594704	3.892746	3.786012	3.158746	2.818065	4.092086	3.631291	4.122179
EXTL3	7.903763	6.839822	4.880897	8.090921	7.18961	5.878323	6.771367	6.84306
EYA1	0.146893	0.279038	0.217593	0.332423	0.146912	0.382065	0.298192	0.335213
EYA2	0.401025	0.633331	0.524155	0.954873	0.424669	0.57002	0.478871	1.208238
EYA3	17.92609	16.40219	15.74929	15.18037	17.32158	15.68943	14.89044	17.10561
EYA4	0.049955	0.072301	0.066952	0.043879	0.071374	0.097	0.086922	0.068761
EZH1	16.43863	14.90963	14.21961	14.24764	15.88403	14.90898	15.58169	16.45211
EZH2	23.59934	21.05702	19.58795	19.72956	19.49247	22.96782	24.75251	22.23969
EZR	160.6609	142.2167	142.3052	147.3896	146.3817	130.7852	156.5456	151.4408
F10	0	0.015123	0	0.015297	0	0	0	0
F11R	58.64447	52.48514	50.38964	54.96753	50.23102	59.01327	55.63073	52.95956
F12	0.282149	0.185804	0.236838	0.216853	0.169311	0.48293	0.272062	0.157391
F13A1	34.04609	64.66063	55.80341	51.78819	45.47933	34.88347	73.09112	89.52903
F2	0	0.075738	0.02953	0.015322	0.029907	0.015054	0	0.015164
F2R	26.24825	21.42972	23.05322	20.78404	19.69071	22.97772	23.54164	29.04573
F2RL1	33.48891	21.80268	22.52054	27.5098	22.43393	26.8754	29.0253	32.91162
F2RL2	37.66902	35.23278	37.838	26.09906	34.66774	26.49638	36.78244	43.49926
F2RL3	0	0	0	0	0	0	0	0

F3	38.02576	57.12502	37.36548	48.06211	40.19514	41.02021	47.70707	62.5205
F5	0.087492	0.194165	0.152232	0.230553	0.216675	0.096479	0.190295	0.671871
F8	2.851492	2.35626	3.068353	2.263618	3.680762	3.025365	2.905382	3.677132
F8A1	0.505337	0.660606	0.788806	0.851954	0.717346	0.541615	0.529463	0.446398
FA2H	63.62209	47.98791	71.40159	68.54841	66.6153	55.36291	40.85023	24.37845
FAAH	2.150128	1.836396	1.839402	2.754234	1.837847	2.076714	2.030121	1.45805
FAAP100	2.720707	2.191643	1.684389	2.583468	2.496384	2.312064	2.237927	2.329067
FAAP20	3.912809	4.083465	3.425318	4.146863	4.384412	4.187445	3.775914	4.35939
FAAP24	8.969979	10.46788	8.56119	9.065255	8.581858	10.68788	9.571427	10.37171
FABP3	1.562038	1.130384	1.888867	1.43739	1.689769	1.187556	1.164834	1.681271
FABP4	84.45853	407.0116	149.1474	140.9682	145.6467	56.64326	119.2608	137.8827
FABP5	505.3995	558.4146	723.9137	644.3879	637.9165	711.0158	565.8801	471.9493
FABP6	0	0	0	0	0	0	0	0
FABP7	15.91065	17.69209	19.28921	26.19698	16.12294	17.45197	20.679	12.59485
FADD	7.642166	6.208066	6.415959	6.367178	7.573573	6.428143	5.899078	6.193167
FADS1	21.94397	21.1137	18.04676	20.2178	18.81051	18.80409	20.06956	15.68769
FADS2	9.557902	7.810823	6.185504	10.81183	6.82062	8.589371	8.606946	8.661215
FADS3	2.067569	2.384397	1.509634	1.386225	2.111833	1.550197	1.819516	2.007768
FADS6	35.4653	14.64773	50.27749	52.13075	40.94897	48.8128	32.81982	25.04543
FAF1	24.50346	24.09078	28.64924	23.49062	25.44547	26.16501	25.46652	24.15222
FAF2	6.685046	6.925235	6.345407	6.334045	7.12612	6.764805	7.148072	6.83363
FAH	3.998452	5.953922	3.618048	5.221924	4.705763	4.007043	4.624721	5.055069
FAHD1	8.429842	9.246299	10.14801	9.780953	9.058025	9.118727	8.573968	7.860912
FAIM	8.9358	12.83394	10.36004	12.06788	8.480776	11.79401	10.18515	9.041979
FAIM2	0.018544	0.031312	0.018313	0.044341	0.049457	0.08713	0.012548	0.025077
FAM102A	4.341917	4.2085	3.73104	4.210392	4.15452	4.110589	3.440127	4.239409
FAM102B	6.482904	6.634162	7.289071	6.370163	6.309536	6.072317	6.756945	8.910463
FAM104A	35.27082	33.2129	38.84301	33.36574	34.24951	42.93519	34.6763	32.22962
FAM107A	3.318999	3.105137	3.650103	2.945547	3.486193	3.577508	4.106209	5.947305
FAM107B	51.34661	49.19155	83.45311	64.24261	55.28119	69.28301	52.1925	63.00234
FAM110A	15.08628	15.03718	15.55203	14.54356	12.12633	16.54574	13.57974	12.25025
FAM110B	1.539202	1.459741	0.942871	1.488381	0.989615	0.967116	1.051315	0.962491
FAM110C	3.017681	3.606028	2.466033	5.002672	2.757771	2.379645	3.120064	2.818071
FAM110D	4.01401	2.900275	3.410879	5.962892	6.224076	4.636682	4.184761	3.503085
FAM111B	0.796573	0.994965	0.614231	0.64857	0.611148	0.593271	0.564857	0.65297
FAM114A1	15.17884	20.10843	17.01851	18.23429	20.13918	17.82107	22.37737	17.89186
FAM114A2	11.46931	12.44982	11.77679	10.72347	11.20894	11.1395	11.46133	9.645277
FAM117A	10.91371	11.64796	9.421505	11.74449	10.03996	9.299646	10.09056	8.591473
FAM117B	4.378161	4.855285	3.942949	4.26847	4.372973	4.796166	3.924121	4.29461
FAM118A	3.869259	3.688084	3.57234	3.437203	3.766718	3.319406	3.811105	4.295875
FAM118B	8.165527	6.994929	6.977967	7.555866	9.02367	7.3422	8.37007	7.658586
FAM120B	7.074831	8.419181	6.94443	7.721359	7.139924	7.29007	7.349215	8.526045
FAM120C	7.475283	7.312828	6.49655	6.907928	6.894124	6.551911	6.718415	7.619452
FAM122A	12.34142	12.43706	12.64024	12.17747	13.73447	12.96956	12.80897	12.23489
FAM122B	13.66863	15.32862	12.74133	13.82367	11.32751	12.02372	13.54645	12.94771
FAM124A	0.347729	0.265765	0.343397	0.400104	0.347774	0.405385	0.235297	0.346492
FAM124B	1.282791	0.997127	0.895501	1.167244	1.835955	1.113409	0.875505	0.740254
FAM126A	5.359148	4.859158	5.661504	6.059546	6.841679	6.166582	5.741224	7.96132
FAM126B	7.372979	7.351913	7.692166	8.041754	9.134004	8.578913	7.654432	8.629585
FAM129A	39.20262	40.44674	38.63466	38.91907	39.16109	38.65578	41.64819	39.59743
FAM129B	72.85302	60.34485	59.21433	69.24139	65.7455	62.86984	67.35281	63.00588
FAM129C	0.058883	0.198853	0.087224	0.090513	0.127597	0.148214	0.149416	0.218979
FAM131A	1.140163	1.099233	0.830847	1.201384	0.951791	0.814676	1.007941	1.109765
FAM131B	0.127481	0.16503	0.419642	0.341112	0.240829	0.128352	0.280352	0.344789
FAM131C	0.014086	0	0	0.014435	0.014088	0	0	0.057147
FAM133A	0.214165	0.284313	0.182325	0.348125	0.288052	0.200757	0.247361	0.134822
FAM133B	8.47291	8.933742	7.944269	8.707177	8.331185	8.578719	8.104779	8.641806
FAM135A	16.43814	14.01297	13.63458	11.6518	13.24088	14.44369	13.04179	13.4708

FAM135B	0.091991	0.012156	0.011849	0.049184	0.012	0.016108	0.016238	0.008113
FAM136A	4.834492	3.969909	4.296828	4.002508	4.631533	4.188628	4.403395	4.1033
FAM13A	4.652726	4.630506	5.418143	3.270913	3.882862	3.724435	3.633072	3.742295
FAM13B	6.160869	5.689045	5.093151	5.651869	5.838816	5.328698	5.552265	7.012513
FAM13C	6.257073	7.524627	6.129789	7.15051	8.177346	8.210596	6.524729	6.534288
FAM149A	3.342835	3.702417	2.573804	3.638531	3.418822	3.223059	3.182115	3.51493
FAM149B1	8.940116	8.433729	8.564387	8.786691	8.825267	7.66269	7.308277	6.805063
FAM151B	2.354683	3.139791	3.252284	2.951997	2.832458	3.177319	2.99777	2.306134
FAM155A	0	0.008086	0.007881	0	0	0	0	0.008095
FAM155B	0.993323	1.398911	0.932247	1.342799	1.458471	0.844064	1.301394	1.271833
FAM160A1	9.052617	7.526149	7.636792	7.538372	7.250764	7.83464	9.021612	8.759913
FAM160A2	4.681035	4.210387	3.946215	4.615636	4.413302	3.546255	3.528673	4.191848
FAM160B1	3.981844	4.323654	3.697826	4.383284	4.46683	3.696906	3.7859	3.65531
FAM160B2	7.151291	5.94325	5.172758	7.035795	6.446378	6.115811	5.98738	6.70694
FAM161A	1.275133	1.496413	1.61603	1.840295	1.764162	2.096945	1.337402	2.155475
FAM161B	3.550594	3.112075	2.435711	2.740481	2.981801	2.365041	2.760203	2.464902
FAM162A	47.1772	56.472	55.75064	47.93953	54.12202	49.85451	45.55098	51.46762
FAM162B	0.094986	0.048116	0.039084	0.121673	0.047499	0.023909	0.05624	0.08831
FAM163A	0.012937	0.006553	0.019164	0.006629	0.032347	0.006513	0.019697	0
FAM163B	0.055097	0.223279	0	0	0	0	0	0.055881
FAM166A	0.191871	0.097194	0.28422	0.172046	0.191896	0.217329	0.340811	0.316229
FAM166B	0.150641	0.228928	0.089259	0.061749	0.030132	0.106169	0.07645	0.10695
FAM167A	25.02732	17.52568	16.32944	16.82092	21.23841	12.60524	19.97422	15.76339
FAM167B	2.724953	1.853623	1.556936	2.233961	2.121845	3.037523	1.264378	1.954362
FAM168A	6.739386	6.476757	6.580677	7.420624	6.768143	6.488664	7.317532	7.602886
FAM168B	21.20483	21.61078	19.40752	19.9894	19.54403	19.2944	19.92672	20.22355
FAM169A	1.420548	2.48543	2.705151	3.025744	2.017148	2.308906	2.923081	3.156911
FAM169B	3.458737	0.88951	0.551757	0.654353	0.691837	0.803623	0.378067	0.296829
FAM170A	0.029339	0	0.014487	0.015033	0.014671	0	0	0.044635
FAM170B	0.032171	0	0	0	0	0.032391	0	0
FAM171A1	5.553083	5.035201	3.890281	5.780962	6.279389	5.556886	5.381744	6.450479
FAM171A2	1.713436	1.431213	0.999051	1.765219	1.540469	1.440677	2.025911	2.06136
FAM171B	3.883138	3.377616	4.065877	2.780143	1.905993	3.058484	1.997356	3.080976
FAM172A	6.160703	6.982149	6.789789	5.834709	5.901554	5.425908	7.052786	6.643821
FAM173A	7.043836	7.199154	6.496417	7.854289	8.255078	8.341641	5.984158	6.797919
FAM174A	12.80524	13.85693	13.6636	13.20368	11.6174	12.45365	14.16426	11.70084
FAM174B	2.055701	2.349691	2.08214	2.484733	4.111934	1.857463	3.638064	3.314561
FAM177A1	13.94927	14.34422	15.29	15.28779	17.7369	16.40989	13.75519	13.95695
FAM177B	0.34154	0.086505	0.016864	0.35	0.051238	0.206324	0.27733	0.242481
FAM178B	0.413007	0.354054	0.470609	0.358124	0.41306	0.575762	0.483695	0.064444
FAM180A	3.562571	3.265858	3.126641	2.891211	2.959615	3.072014	3.050279	4.440845
FAM180B	0.342343	0.514999	0.973254	0.754802	0.456517	0.626695	0.737076	0.694434
FAM181B	0.243667	0.69122	0.577513	0.73246	0.324931	0.703283	0.280298	0.23066
FAM184A	0.09866	0.192221	0.119915	0.202208	0.060722	0.183386	0.077031	0.146249
FAM184B	0.093923	0.044406	0.204057	0.154	0.068886	0.113478	0.063555	0.069858
FAM185A	3.352869	3.323027	3.034244	1.671995	3.633679	3.076583	3.289344	3.224314
FAM186B	0.540177	0.658746	0.484053	0.625315	0.59027	0.493509	0.609199	0.608741
FAM187A	0	0.044321	0.017281	0.017932	0.00875	0.017618	0	0.017748
FAM189A1	0.02489	0.018913	0	0.025507	0.03734	0	0.012632	0.012622
FAM189A2	2.386409	2.026286	2.626008	1.793382	1.94347	1.933611	1.787818	2.189868
FAM189B	6.044972	5.601605	5.111991	5.895106	6.845547	6.415256	5.690528	6.192074
FAM192A	13.57283	15.00508	14.44225	14.14478	13.04869	13.91923	13.40949	13.04386
FAM193A	8.132701	7.86773	6.598566	8.533532	8.16155	7.14164	7.521192	9.077269
FAM193B	5.124101	3.98662	3.349301	4.133996	3.887531	3.929675	3.471635	4.856628
FAM199X	5.157249	5.005835	4.423335	4.860544	5.128589	4.587245	4.278464	5.531792
FAM204A	9.363358	8.054966	11.42331	11.01581	8.185366	9.929972	8.754501	10.46086
FAM205A	0	0	0	0	0	0	0	0.006904
FAM205C	0	0.018283	0	0	0	0	0	0

FAM207A	6.809624	7.1525	6.42472	6.300618	6.792629	7.57595	7.41972	7.794809
FAM20A	5.337887	5.20504	6.651782	4.879537	4.34117	5.172712	5.978004	8.212011
FAM20B	11.88416	10.88164	12.61966	12.72127	11.33595	12.28448	12.97464	12.70036
FAM20C	14.36123	12.77814	12.81196	15.0291	15.15288	12.15357	12.68729	14.19953
FAM210A	2.832828	3.432208	2.939606	2.969694	3.93975	3.072906	3.212571	3.415129
FAM210B	124.2771	128.5284	129.906	115.901	120.012	127.9118	113.7276	103.3423
FAM214A	17.03366	15.62815	17.04073	18.47545	16.094	17.67588	15.54439	15.70369
FAM214B	3.438552	3.079177	2.647364	3.156324	3.650878	3.338985	3.308181	3.371803
FAM216A	0.388454	0.383196	0.353329	0.419028	0.541862	0.596955	0.425409	0.5184
FAM216B	0.034512	0.04662	0.090885	0.22399	0.241615	0.231651	0.140118	0.116677
FAM217A	0.334074	0.126922	0.917567	0.492127	0.135735	0.189201	0.211928	0.571776
FAM217B	2.783767	2.331065	2.454536	2.309346	1.652483	1.949556	2.594868	2.343226
FAM219A	4.741933	5.836467	4.644147	5.574246	4.915003	4.995289	4.367556	5.167165
FAM219B	12.40404	12.1304	10.67876	12.19061	12.06319	12.54438	11.42414	11.91966
FAM221A	0.353399	1.133782	0.721257	0.796735	0.518385	0.735346	1.052185	0.465958
FAM221B	0.612901	0.404555	0.366827	0.428239	0.724432	0.467491	0.273345	0.857091
FAM222A	0.764567	0.637907	0.577384	0.783506	0.419817	0.664133	0.426059	1.20119
FAM222B	2.798916	2.567137	2.288166	2.444388	2.815343	2.510762	2.893887	2.655484
FAM227A	0.361321	0.428562	0.496065	0.532829	0.414251	0.204077	0.286237	0.321774
FAM227B	0.08192	0.110659	0.148314	0.125923	0.08193	0.082479	0.110864	0.083086
FAM228A	0.735538	0.947831	0.656285	0.747146	0.851786	0.649617	0.785865	0.844168
FAM228B	0.756746	1.189672	1.01532	1.080339	1.08046	0.872262	0.709828	0.746346
FAM229A	0.060583	0.368266	0.239311	0.931249	0.363543	0.670961	0.430439	0.675895
FAM229B	2.032686	2.915312	2.469959	2.263052	1.815432	2.282391	2.71694	2.019202
FAM234A	13.50929	11.40548	12.26543	12.12644	12.34843	12.88002	11.35787	15.63608
FAM234B	16.89668	16.23212	14.06665	20.04497	16.35483	13.84458	15.17499	13.32073
FAM241A	5.072967	7.442328	8.241159	7.505376	5.545844	6.600111	6.865416	7.561862
FAM241B	12.84883	15.56014	12.33851	14.90035	12.14112	12.33235	13.31845	13.11474
FAM243A	0	0.012536	0	0	0.037126	0	0.012559	0.075299
FAM24A	0.241286	0.146672	0.047656	0.024726	0.506766	0.097174	0.024491	0.097888
FAM25A	341.595	218.1875	318.6326	245.4669	211.4303	295.3958	277.6334	274.772
FAM32A	48.25167	53.59526	52.07081	50.47076	52.77459	52.17594	47.98836	44.33115
FAM3A	2.816482	2.902537	2.566055	2.973145	2.744153	2.719854	2.655851	2.868862
FAM3B	0.041837	0.03179	0.030987	0.096466	0.083685	0.231677	0.063697	0.159123
FAM3C	23.38346	23.87915	24.23137	23.3261	23.1646	26.18791	26.50846	25.98447
FAM3D	0	0	0.014532	0.01508	0.014718	0	0	0
FAM43A	15.94342	16.82149	19.37062	17.18168	16.52433	18.06458	17.05458	16.2988
FAM43B	0.088608	0.022443	0.032814	0.056752	0.033232	0.011152	0.033727	0.067402
FAM45A	15.24749	16.90963	17.13441	16.98937	16.86056	17.43534	16.41863	16.12262
FAM49A	27.66916	32.99959	30.52409	29.10995	29.6216	36.42506	28.293	27.36176
FAM49B	12.41827	12.41246	13.18741	12.26622	14.04766	12.63214	12.76064	15.05167
FAM50A	29.79492	24.0306	26.433	27.91143	28.51953	24.5442	25.64065	28.8264
FAM53B	9.103715	7.817217	7.149936	7.366668	9.215927	7.489217	6.789345	8.300329
FAM53C	11.9107	10.68622	9.265488	11.79415	12.26125	10.93804	11.26068	11.69294
FAM57A	9.856367	11.26626	8.221313	9.714426	8.459825	9.152809	10.28793	8.591473
FAM57B	0.010057	0	0	0.020613	0	0.010126	0	0.0102
FAM71A	0	0.015011	0.014632	0	0.029638	0.014918	0	0.015028
FAM71D	0.021597	0	0	0.022132	0	0.021744	0	0
FAM71E1	0.44116	0.297967	0.193628	0.25116	0.612802	0.172734	0.273642	0.149147
FAM71E2	0.085791	0.021729	0.01059	0.087916	0.02145	0.053985	0.043539	0.021753
FAM71F1	0.009783	0.009911	0.009661	0	0.009784	0	0.00993	0.009922
FAM72A	0.948596	0.780143	0.991881	0.960657	0.591554	1.078675	0.917516	0.780999
FAM76A	8.861636	9.73788	9.216343	9.671059	9.063416	9.0539	9.959529	9.155866
FAM76B	6.568493	6.779376	6.410693	6.920364	7.015504	6.77599	6.27668	7.481098
FAM78A	0.749593	0.455659	0.611514	0.968551	0.743171	0.538144	0.913005	1.06437
FAM78B	0.743172	0.81936	1.41386	3.057522	1.224206	2.91597	1.553011	1.141712
FAM81A	2.829988	3.173409	3.574263	3.760647	3.250287	4.066835	3.000291	2.802137
FAM83A	0.041889	0.028293	0	0.035773	0.013965	0.056234	0.042518	0.056648

FAM83B	17.44277	16.84328	12.76187	14.46247	15.20742	13.01468	14.45172	14.91373
FAM83C	22.50822	22.45956	23.34305	23.89126	21.01038	25.63587	23.14243	23.78183
FAM83D	10.30982	7.783141	8.130261	8.706032	9.059801	9.208696	8.432533	9.352556
FAM83E	0.046276	0.085952	0.106632	0.126459	0.107991	0.132011	0.273991	0.015645
FAM83F	3.873869	4.064214	2.828406	3.903976	3.617303	3.262601	2.599577	3.668323
FAM83G	41.28402	33.98115	34.5778	38.24958	39.65951	39.2764	37.66062	39.06098
FAM83H	30.54592	27.83076	25.87886	26.98744	28.95507	27.32593	25.49019	29.23189
FAM84A	132.0512	149.5227	128.9991	137.1735	124.3478	134.6841	151.3504	146.2644
FAM84B	8.253444	7.819573	7.287392	8.707721	7.713329	8.585225	7.719378	7.013977
FAM89A	6.27962	7.654638	8.83942	6.966325	7.097885	8.309597	8.03303	6.530767
FAM89B	15.64557	14.30173	14.99104	14.44343	14.73888	13.76666	12.88864	15.23895
FAM8A1	17.59583	16.16977	16.8768	16.65291	16.83271	16.13148	15.45425	15.91208
FAM91A1	15.40518	17.05329	15.43331	16.53991	15.78654	15.59635	17.16668	17.56733
FAM92A	5.217432	7.478053	6.979032	7.313671	4.69455	7.343763	6.340663	6.212002
FAM92B	0	0	0	0.012231	0	0	0	0
FAM98A	15.39555	15.54672	13.91442	13.44072	15.63859	14.17613	14.9843	15.15635
FAM98B	14.57737	15.74486	13.98135	14.52438	13.83319	15.38107	16.62581	14.2805
FAM98C	4.321856	4.190923	5.162254	4.21801	3.602012	3.83336	3.404902	3.715437
FAN1	8.314611	7.528759	6.550442	6.980035	6.657198	7.146518	5.875284	7.183354
FANCA	3.16445	3.028363	2.16847	2.488269	2.489806	2.634463	2.651388	3.449543
FANCB	0.501925	0.610215	0.3695	0.411486	0.319448	0.441036	0.287147	0.546094
FANCC	2.794758	3.058962	3.088498	1.764419	2.903263	2.671483	2.203492	2.952649
FANCD2	4.223694	4.582279	3.756232	4.133719	4.218485	3.760087	4.707577	4.87912
FANCE	6.757278	6.147648	4.874145	6.81114	5.440989	5.650946	5.247045	5.854788
FANCF	1.01623	1.153611	0.906838	1.254701	1.077587	0.788952	1.068755	1.00586
FANCG	5.451898	5.118954	3.738866	5.608106	5.184104	4.303987	4.960746	6.486003
FANCI	3.753249	5.68637	3.446577	5.640338	4.280172	5.28814	5.011645	5.628778
FANCL	3.698797	4.266452	4.063837	3.216112	4.532012	5.432944	4.696912	4.271133
FANCM	3.284771	3.432148	3.040573	3.036553	3.139377	3.203591	2.98584	3.592487
FANK1	0.348459	0.43275	0.366319	0.506839	0.292294	0.271618	0.319459	0.35342
FAP	22.28352	23.40029	19.72594	22.79381	24.95142	23.71575	20.91448	20.15603
FAR1	8.627198	8.11495	6.017926	7.161072	9.570455	6.692772	8.048947	9.505719
FAR2	55.7894	61.27838	102.9605	88.08385	86.75663	90.40968	43.78537	43.18465
FARP1	44.63122	34.03008	31.10035	37.96532	34.87088	33.92114	33.7719	35.07615
FARP2	5.498276	4.745185	4.027635	4.95045	4.5146	4.801143	4.501346	5.24379
FARS2	7.102712	8.998247	7.157358	8.075383	7.340857	8.345318	6.861496	7.524587
FARSA	13.97601	14.5106	13.59405	15.17215	14.96209	13.58541	13.23073	12.57417
FARSB	49.17934	56.69864	53.6722	53.21352	57.24637	62.86391	56.54484	53.32733
FAS	3.975734	4.235061	3.701841	5.098588	4.998711	5.077957	5.326688	4.308833
FASLG	0.224765	0.19176	0.186917	0.303069	0.378601	0.083374	0.432259	0.347946
FASN	44.95096	42.33616	42.08138	55.18656	47.8027	47.49356	41.96176	34.95151
FASTK	11.8152	11.92064	12.06699	12.91087	12.56369	11.27955	10.25259	11.77229
FASTKD1	4.776912	3.32678	3.871755	3.858172	4.182992	4.866547	4.094344	4.636762
FASTKD2	10.52302	12.09306	11.73673	11.20657	10.78233	11.77904	12.24111	11.83427
FASTKD3	1.468935	1.166437	0.901744	1.22053	1.022431	0.959332	0.987263	1.127451
FASTKD5	5.679105	5.874132	5.344075	5.565861	6.462923	6.416424	6.569079	5.780055
FAT1	19.39662	16.02586	10.53135	13.03801	14.86732	12.30286	14.79691	17.2406
FAT2	5.691143	5.228097	4.325693	4.864289	5.106255	3.986102	4.844753	4.91965
FAT3	0.009905	0.020071	0.009782	0.010151	0.02972	0.059838	0.05027	0.02679
FAT4	3.147013	2.819983	1.9771	3.349715	4.045742	3.036007	3.257668	3.730733
FATE1	0.112632	0	0	0	0	0	0	0
FAU	776.5036	982.5624	993.267	839.9149	679.2165	896.1556	843.2308	795.1508
FAXC	2.088995	2.196168	1.91581	2.385644	2.021779	2.250467	2.174551	2.30979
FBF1	3.803324	4.56901	3.058237	3.191633	3.468184	3.106124	3.705002	4.162003
FBH1	10.81304	10.86535	9.294681	11.73439	11.0379	10.80724	10.44136	10.82062
FBL	95.32611	103.2778	99.67588	94.78966	92.46246	100.0307	111.8118	101.7871
FBLIM1	2.797885	2.253058	1.771865	2.863668	3.709224	2.56783	2.777056	3.318802
FBLL1	0	0.025593	0	0	0	0	0	0

FBLN1	156.7276	152.4925	140.781	195.365	190.0144	165.1131	184.5141	198.3567
FBLN2	24.04361	21.4813	23.2584	23.67282	27.26374	20.95762	28.86115	29.966
FBLN5	31.75588	27.41871	33.58663	24.77549	34.68392	23.133	30.93921	29.86555
FBLN7	7.487469	4.966111	5.445791	5.450171	5.821616	5.490484	6.094751	7.047186
FBN1	23.03665	21.96087	30.32992	25.51085	30.58785	19.8482	30.41913	38.41584
FBN2	0.154366	0.153786	0.152443	0.073822	0.162105	0.158011	0.219354	0.203533
FBN3	0.39268	0.290389	0.509501	0.431779	0.315331	0.317444	0.389842	0.482575
FBP1	33.80563	47.1842	34.91206	37.09486	53.81958	53.59878	32.5751	29.16163
FBRS	13.11582	13.07003	11.42919	12.01061	12.87091	12.62438	12.07183	12.29154
FBRSL1	5.335363	5.347324	4.697286	5.814013	5.318858	4.990996	4.944237	5.004449
FBXL12	4.924152	4.945396	4.129853	5.075378	4.610744	4.929051	5.099423	4.096733
FBXL13	0	0	0.017561	0	0	0.008952	0.009024	0
FBXL14	5.556407	4.151288	3.619578	4.217467	4.467317	4.016701	5.374676	4.694733
FBXL15	5.789252	5.98348	4.748895	5.023618	5.346411	5.852304	4.667701	5.137707
FBXL16	0.017519	0.011832	0.034601	0.005984	0.029202	0.029397	0.059272	0.017768
FBXL17	11.1773	12.19597	11.06637	11.83049	11.55176	11.88914	13.02225	11.8369
FBXL18	1.391515	1.254368	1.138835	1.013346	1.317726	1.158324	1.145479	1.208515
FBXL19	5.645811	5.290348	4.517668	5.78566	5.445676	5.032811	4.796178	5.556434
FBXL2	1.107229	0.889396	0.687301	1.483157	1.281388	1.019239	0.971317	0.922457
FBXL20	6.011418	5.475191	5.307245	5.199313	5.386927	5.265663	5.418212	6.170159
FBXL21P	0.12754	0.07048	0.10305	0.023763	0.139152	0.046695	0.047074	0.070557
FBXL22	0.202234	0.450754	0.738942	0.683903	0.930396	0.386869	0.718436	0.307669
FBXL3	30.80891	33.17726	34.65731	30.28323	30.9571	32.50341	29.62049	30.78439
FBXL4	2.46385	3.076792	2.47859	2.850671	1.932845	2.366966	2.178123	2.337703
FBXL5	37.28328	38.2547	39.32189	36.28601	37.04527	36.46239	34.9346	39.14377
FBXL7	2.418111	2.205505	2.14981	2.887758	3.529249	1.891472	2.11296	2.336667
FBXL8	1.640391	1.402244	0.82685	1.120682	1.093735	0.756982	1.595622	1.195815
FBXO10	0.582878	0.807798	0.543034	0.777639	0.571955	0.786172	0.597203	0.529828
FBXO11	23.61724	22.57297	22.69577	20.62262	21.41189	22.24688	20.26859	22.57526
FBXO15	0.456444	0.314456	0.450757	0.33678	0.127821	0.29412	0.500354	0.351836
FBXO16	0.121315	0.017558	0.119804	0.10656	0.017333	0.20939	0.070363	0.052732
FBXO17	1.040919	0.761641	0.713854	0.76052	0.684397	0.485199	0.763052	1.11439
FBXO2	0.539736	0.751126	0.720443	0.692901	0.605057	0.53148	0.571913	0.631638
FBXO21	10.56548	10.69856	9.574322	11.14896	10.39606	10.04976	10.52827	12.46462
FBXO22	10.56119	10.06242	9.914066	10.09579	12.77146	10.32337	9.809325	11.28173
FBXO24	0.089545	0	0.113695	0.078654	0.025588	0	0.038952	0.155691
FBXO25	15.38005	17.0139	15.83796	16.30598	15.59198	16.73914	16.98858	17.51515
FBXO28	13.39247	13.72668	14.39937	12.36193	13.02402	13.38423	13.45049	14.01668
FBXO3	30.02082	30.64479	32.07902	27.84502	28.91225	31.51722	30.95498	27.53575
FBXO30	6.690374	5.208847	5.427188	4.719899	5.677665	4.99399	6.213685	6.217363
FBXO31	10.15293	9.630882	8.966682	9.269578	9.41914	9.112246	9.409985	9.63151
FBXO32	13.89812	14.268	11.83534	12.24522	11.21444	11.19842	15.56121	12.70235
FBXO33	6.915329	6.573516	5.948223	6.602202	7.122108	7.323366	7.189312	6.944177
FBXO34	10.05608	10.55601	10.99878	11.28111	9.330928	10.0679	10.9278	10.02734
FBXO36	0.093654	0.054219	0.125518	0.075408	0.060214	0.047147	0.047529	0.040709
FBXO38	16.5098	13.40903	14.69959	13.72967	15.86195	14.92383	15.0576	15.71808
FBXO39	0	0.016075	0.015669	0	0	0.015975	0	0
FBXO4	2.559881	2.5076	2.712142	2.779645	2.492392	3.072361	1.789543	1.667836
FBXO40	0.097337	0.054238	0.057674	0.079798	0.05841	0.078401	0.049398	0.034553
FBXO41	0.143146	0.089574	0.112258	0.163949	0.122111	0.152601	0.188026	0.162264
FBXO42	8.590566	7.537151	7.530697	7.233498	7.089191	6.463506	6.696346	8.206397
FBXO43	0	0.027084	0.0264	0	0.008912	0	0	0.045189
FBXO45	3.71949	3.796013	3.927024	3.441672	4.442083	4.11939	4.1195	3.561627
FBXO46	4.671101	4.191829	4.233883	3.654856	4.4751	4.580489	4.741168	5.117365
FBXO47	0.094558	0.24634	0.04002	0.027686	0.04053	0.054402	0.178242	0.095904
FBXO48	1.290673	1.232891	0.965047	0.831376	0.95891	0.965336	0.96381	0.916333
FBXO5	3.97809	5.238075	3.429459	3.731376	3.615734	3.705197	4.99788	5.007259
FBXO7	11.97296	10.34109	10.03106	11.89747	9.861844	10.3184	10.12573	11.27303

FBXO8	6.514719	7.145536	6.231924	7.132576	8.817355	6.428414	7.328317	5.929772
FBXO9	25.58644	24.47204	26.3783	25.919	24.4138	25.61969	25.5681	24.7711
FBXW11	5.772746	6.014831	4.672076	5.695505	5.56751	5.505646	5.568492	5.724749
FBXW12	14.07948	12.67297	11.38875	12.97816	9.963053	10.88463	10.45589	13.04567
FBXW2	27.92977	29.48474	26.51134	28.25641	28.05218	26.93954	26.04284	28.8394
FBXW4	3.545375	4.703684	5.096983	4.115149	4.245351	4.43164	3.855595	3.644768
FBXW5	18.23816	18.32978	16.02357	20.30388	17.46267	18.06911	17.98875	16.12148
FBXW7	7.890404	6.984894	6.908631	7.786376	7.867565	7.920288	6.676993	7.549059
FBXW8	16.29307	17.27329	15.15913	16.74436	17.24961	15.8185	16.83278	16.01746
FBXW9	3.534468	3.016305	3.616209	3.654649	2.547694	2.644916	3.328927	2.890433
FCAMR	0	0	0	0	0	0	0.015063	0
FCER1A	1.579407	1.757526	1.252896	3.077859	1.631402	1.016683	2.154984	4.779412
FCER1G	10.40855	12.35514	12.5801	14.5933	16.62476	9.645456	12.29918	19.45904
FCF1	32.48853	34.73828	29.72417	34.50164	32.15136	37.24058	33.51128	33.2028
FCGBP	4.065107	1.04429	2.008734	2.528232	7.13392	7.464207	6.354727	4.348766
FCGR1A	3.297817	4.797473	3.423737	3.444496	4.90508	2.575397	5.342781	5.724691
FCGR2B	2.487318	2.173813	2.969185	4.46763	2.788344	1.912633	2.885289	2.744505
FCGR3A	7.806309	5.622408	7.681008	8.232391	9.794635	7.545252	8.859805	7.816669
FCGRT	32.38998	37.20229	30.59568	37.12485	37.32371	30.57305	33.39398	37.85014
FCHO1	3.755899	2.832174	3.277218	2.864732	2.864458	2.831851	2.880937	2.887465
FCHO2	12.82644	12.93442	13.68622	13.37475	13.29806	12.64752	13.41518	12.63984
FCHSD1	9.674793	8.830652	8.822215	9.678695	8.346706	8.421943	9.352918	9.831964
FCHSD2	3.396724	2.901332	2.356721	3.904761	4.940605	2.727133	2.418217	4.292229
FCMR	1.954356	2.772006	1.419932	1.845406	2.289684	1.41956	1.771135	4.304156
FCN3	0.060127	0.091374	0.118756	0.236196	0.050112	0.27242	0.091544	0.081311
FCRL1	0.035838	0.036308	0.023594	0.097934	0.143369	0.04811	0.206126	0.072696
FCRL3	0	0.061091	0.019849	0.041196	0	0.020237	0.040803	0.050965
FCRL4	0	0	0	0	0	0	0	0
FCRL5	0.006808	0.041386	0.053788	0.174425	0.102139	0.068549	0.062194	0.400506
FCRL6	0	0.018804	0.054988	0.03804	0	0.037375	0.018839	0
FCRLA	0.042312	0.021433	0	0.281838	0.021159	0.0213	0.021473	0.064371
FCRLB	0.330019	0.234949	0.105699	0.584983	0.374664	0.24247	0.181064	0.379949
FDFT1	38.08097	40.23019	46.87696	51.76806	44.17178	49.35053	41.96891	27.55612
FDPS	73.28524	77.66575	88.01182	96.31538	74.45418	96.40106	81.86499	54.90651
FDX1	10.97208	13.94754	13.18644	13.23806	12.40213	14.1944	13.51109	12.09413
FDX2	10.37177	12.16547	12.92579	12.84424	13.06135	11.82517	11.95076	12.65293
FDXACB1	3.85049	3.431913	3.273049	4.307989	3.838801	4.232577	3.648522	4.214268
FDXR	5.78395	6.672116	4.340464	4.636143	3.908967	4.583815	5.667269	4.966016
FECH	8.429605	8.46742	7.584384	9.153978	8.985211	8.383816	8.754058	8.070578
FEM1A	26.45604	23.15039	22.63425	23.37704	24.90302	21.67244	25.03871	24.7073
FEM1B	22.55528	23.13811	21.33536	21.32133	21.86514	21.90531	20.90789	22.74351
FEM1C	12.88013	11.33704	10.50949	12.22562	10.12272	14.32932	10.79245	11.48846
FEN1	7.176464	7.164153	5.91688	6.737295	5.915853	6.196026	6.993134	7.278625
FER	5.800064	5.41482	5.072567	5.75807	5.531464	5.371329	4.863228	6.846102
FER1L5	0	0	0.00337	0.003497	0	0	0	0
FER1L6	0	0	0	0	0	0.004408	0	0.00444
FERD3L	0	0	0	0	0	0	0	0
FERMT1	13.30394	15.29532	15.08457	12.76432	11.2779	11.49174	12.89679	13.23117
FERMT2	26.12858	30.67202	26.665	29.81235	30.73807	27.56751	28.29243	27.16451
FERMT3	2.352212	2.50333	2.067171	2.47682	2.946041	1.357959	2.157507	3.961571
FES	2.258149	2.488468	1.838782	2.740363	3.734352	2.35335	2.412654	3.043682
FETUB	0.01881	0	0	0	0	0	0	0
FEV	0	0	0	0	0	0	0	0
FEZ1	9.775707	13.65353	11.52927	12.56102	10.51078	7.083116	12.0908	13.99682
FEZ2	14.06806	17.81248	17.67931	16.34695	16.70214	16.45088	17.03184	16.26021
FEZF1	0	0	0	0.020044	0	0	0	0
FFAR2	0.096431	0.097697	0.023807	0.160582	0.024111	0.121363	0.061174	0.281186
FFAR3	0	0	0.030175	0.015656	0	0.015382	0.062028	0.092973

FFAR4	1.298048	2.087695	1.273863	1.579614	1.557859	2.164579	1.828058	1.226016
FGA	0	0.010113	0	0	0.009983	0.03015	0.020263	0
FGB	0.261445	0.809345	1.663876	1.696835	0.522958	0.131616	1.503746	0.235704
FGD1	3.033684	2.857272	2.476497	3.265052	2.813	3.03906	2.947666	2.458404
FGD2	1.728807	1.707154	1.037326	1.760417	1.061493	1.531303	1.377137	2.263906
FGD3	0.919236	0.826931	0.837351	1.153146	1.228449	0.686159	1.045631	2.065576
FGD4	1.987073	2.162756	1.933783	1.621798	1.968067	1.651585	1.570494	2.454898
FGD5	3.735672	4.287728	3.282854	5.248034	6.152832	5.03238	4.876423	5.357148
FGD6	2.075651	2.054064	2.081519	1.876823	2.323359	2.180412	2.149183	1.873823
FGF1	0.601119	0.622693	1.073867	0.629851	0.614706	0.884037	0.925486	0.527472
FGF10	0.832539	1.214991	1.751995	1.432092	2.002317	1.297255	1.388259	2.050663
FGF11	4.95165	4.531154	4.036719	5.743933	6.048765	5.533734	4.016318	4.572945
FGF12	2.205525	2.819689	0.881589	2.63685	0.997867	2.11485	3.731013	3.035823
FGF13	1.21692	1.186888	1.291441	1.554176	1.525888	1.133798	1.603883	1.639518
FGF14	0.00746	0.030231	0.007367	0.022934	0.014922	0.030043	0.030287	0.030264
FGF16	0.081187	0.26732	0.020044	0.187195	0.020299	0.122612	0.041202	0.123514
FGF17	0	0	0.042458	0	0	0.043288	0	0
FGF18	2.070058	1.519546	1.628067	2.273765	1.847177	1.497628	2.239505	2.099526
FGF19	0	0	0.033382	0	0	0	0	0.034285
FGF2	16.32701	14.84475	12.57993	14.46381	14.0562	10.65796	11.53357	13.40526
FGF21	19.4644	12.52756	34.07644	17.1737	18.56486	24.25504	15.56294	6.432951
FGF22	6.486099	6.459845	6.730973	6.962201	6.640864	8.168545	5.222079	5.240446
FGF23	0.01677	0.03398	0	0	0.058703	0.008442	0	0.017009
FGF3	0	0	0	0	0.080094	0.080631	0.203212	0.040612
FGF4	0	0	0	0.046188	0	0	0	0
FGF5	7.33587	7.350474	9.807889	5.766233	11.69053	8.165166	7.445908	7.832756
FGF6	0	0	0	0	0	0	0	0
FGF7	9.021082	12.2217	10.51538	12.47064	13.0439	12.49206	11.02259	12.23511
FGF9	0.133282	0.122171	0.144156	0.208127	0.253903	0.230044	0.115955	0.083682
FGFBP1	32.50058	53.4949	55.21264	69.79557	71.24808	55.29471	47.58975	36.51169
FGFBP3	0.019655	0.019913	0	0	0.058974	0	0.01995	0
FGFR1	53.43386	45.98372	41.51825	52.8676	46.27331	46.59907	51.04459	47.5889
FGFR1OP	8.343252	8.879423	7.800914	8.549918	8.071119	7.517822	7.567262	8.392758
FGFR1OP2	14.01309	12.73417	12.94273	13.75317	14.78323	13.23285	13.06777	14.4191
FGFR2	28.51883	31.86441	25.79514	28.02408	26.20737	28.63959	27.51298	29.0243
FGFR3	30.53482	23.76854	23.28464	26.1274	26.4089	24.16778	23.24131	23.27027
FGFR4	0.042305	0.128581	0.116979	0.112718	0.135394	0.204453	0.154583	0.068652
FGFRL1	1.528419	2.588861	1.084863	1.70088	2.113789	1.454704	1.502865	1.295851
FGG	0.172037	0.160888	0	0.013561	0.251472	1.958636	0.214914	0.268441
FGGY	1.741755	2.217563	1.9776	2.262145	1.816503	0.872138	1.834059	2.068847
FGL1	0.695244	0.522596	0.575842	0.988258	1.345807	0.745154	0.7512	1.160071
FGL2	30.41276	43.85622	41.07003	52.46666	50.12779	35.80585	43.93743	61.79383
FGR	1.550536	1.335924	1.662089	1.840187	2.650832	1.367655	2.158923	3.944964
FH	47.99201	56.36773	55.7637	49.78814	51.06571	57.34534	57.08871	49.81732
FHAD1	0.052963	0.070169	0.068397	0.02505	0.044821	0.061529	0.082705	0.05785
FHDC1	23.3243	18.52472	17.63603	18.23372	18.47917	18.73976	18.68738	21.11495
FHIT	0.743404	1.205057	0.789202	0.857046	0.855025	0.973028	0.6791	0.169647
FHL1	27.85036	36.37225	34.21028	28.97711	36.99648	29.46811	31.88088	38.46355
FHL2	14.43662	10.19538	10.1794	10.50534	10.77007	10.36879	11.1975	10.01579
FHL3	6.88305	6.293405	6.430627	7.419393	8.812014	7.57707	6.841352	5.561654
FHL5	1.675476	1.687653	0.841646	0.893226	1.840356	1.95995	1.218933	1.208192
FHOD1	1.961348	2.134281	1.506485	1.838715	1.874419	1.843097	2.138233	2.784977
FHOD3	24.59455	23.72471	26.57524	23.18387	21.53122	23.03712	23.71863	20.31891
FIBCD1	0	0.020255	0	0.020487	0	0	0.020292	0
FIBIN	7.084339	5.888382	5.594208	6.628532	5.143172	5.905743	7.12264	7.755658
FIBP	49.45679	57.23289	50.31865	57.97846	48.92856	55.7149	49.24372	49.05483
FICD	2.293133	2.988345	2.565892	2.823712	2.274934	2.029508	2.355685	2.203861
FIG4	4.171388	4.132393	4.401146	4.021866	3.477891	3.780673	4.460918	4.684346

FIGLA	0	0	0	0	0	0	0	0
FIGN	0.850436	0.844013	0.602742	0.767751	0.77822	0.830034	0.783919	1.02977
FIGNL1	2.911295	2.918938	2.338747	3.168906	2.934301	2.741341	3.054484	3.13633
FIGNL2	0.824255	0.847059	0.923035	1.103327	1.313456	1.080042	0.808598	1.087985
FILIP1	3.784046	3.471365	3.23549	3.429461	4.087936	3.240823	2.806868	2.71083
FILIP1L	14.58693	11.42692	11.75933	12.27425	15.40909	12.86987	14.36175	14.01203
FIP1L1	26.09451	26.79844	22.99926	25.10189	25.67953	27.22658	25.39952	23.82063
FIS1	51.06291	54.98456	55.33087	49.00426	51.74526	52.36413	52.94327	47.74669
FITM1	4.307511	4.510816	3.872329	4.325134	3.825101	3.57846	4.528971	3.50682
FITM2	19.33683	24.67435	25.34026	29.65163	28.86829	25.24508	22.75259	18.47057
FIZ1	5.488849	5.390243	4.667072	5.025777	5.866098	5.666001	4.766679	5.526791
FJX1	5.234242	7.132387	6.012453	5.588955	4.746814	5.933357	4.86693	5.407753
FKBP10	11.28375	12.03743	9.051226	10.80025	11.85157	12.48003	11.94264	13.46523
FKBP11	14.11088	18.14501	19.52439	17.7577	16.51652	20.17902	14.67667	17.10334
FKBP14	5.53756	5.743812	5.738269	5.201833	6.951101	5.916741	6.414011	5.024186
FKBP15	14.94477	12.52462	11.92062	14.64319	13.32422	13.20684	12.98467	12.28316
FKBP1A	197.822	199.4354	200.3965	192.0616	194.178	195.2952	208.8368	204.1598
FKBP1B	3.943794	5.695153	7.963695	6.876553	10.50833	8.089634	6.751246	4.686493
FKBP2	17.39421	18.75943	16.98191	18.75522	18.35376	18.01151	17.75563	17.49119
FKBP3	72.2193	83.82553	85.84237	68.66062	70.86109	92.43612	76.7298	73.24739
FKBP4	100.7442	95.53192	93.8167	94.63129	103.1628	101.9024	87.1339	94.30172
FKBP5	14.97505	14.03315	13.00427	11.73339	10.99585	11.26919	16.07185	44.85704
FKBP6	0.020007	0.020269	0	0.020502	0.020009	0.020143	0.06092	0
FKBP7	13.87747	17.53001	16.59581	16.59143	20.3504	19.39611	19.55348	23.3396
FKBP8	85.48667	88.74997	77.62146	88.80565	83.30124	85.47215	87.20474	79.20059
FKBP9	44.88331	48.67703	42.84107	48.28706	42.81202	42.47162	49.35091	48.66563
FKBPL	4.757052	4.614001	4.643153	6.065265	5.329324	6.887298	4.734834	4.488159
FKRP	2.623984	2.541246	2.162977	2.400082	2.689389	2.088783	2.318503	2.382741
FKTN	3.610075	4.425868	3.487274	3.396674	3.989821	3.421513	3.984159	4.325826
FLAD1	9.185927	10.99039	9.284473	9.725514	10.20227	9.465846	8.808594	8.814828
FLCN	5.178612	5.256986	4.454531	4.632997	5.276907	4.712247	4.95906	5.153331
FLG	31.01951	28.23127	36.13836	29.68266	18.88778	24.37836	22.22279	16.35303
FLI1	5.398185	4.628838	4.73944	6.161419	6.827321	5.821624	5.510333	6.66661
FLII	38.39893	35.48054	35.14281	35.27378	34.91514	33.71233	36.86173	36.43543
FLNA	122.4254	114.417	94.42402	108.0218	114.1958	91.5196	119.5592	115.5822
FLNB	32.18047	23.0236	22.85057	24.65527	25.23646	23.04177	26.09913	28.85844
FLNC	2.738309	3.165568	3.22518	3.475492	4.720425	2.69062	3.984209	3.697746
FLOT1	36.56145	33.47236	32.29464	39.33983	42.01322	36.62964	36.0848	37.16728
FLOT2	33.21502	31.18293	27.77018	35.64332	32.91603	28.63834	32.52313	29.71011
FLRT1	1.254651	1.334672	0.939588	0.867867	1.160702	0.842148	1.146123	1.410367
FLRT2	14.37249	12.81491	13.44701	14.64654	11.75865	10.10612	15.44453	15.84255
FLRT3	51.64719	46.22676	39.96087	41.94724	39.58301	38.10522	44.25501	51.02021
FLT1	4.448959	4.109415	3.893752	5.039073	6.035956	4.977481	4.795528	4.807239
FLT3	0.364537	0.568755	0.439194	0.694834	0.576043	0.484476	0.518007	1.035234
FLT3LG	7.06845	7.161216	7.448858	7.316459	6.618632	7.331673	8.330097	6.808215
FLT4	1.354859	1.314022	1.023719	0.785618	1.711876	0.655358	1.291986	1.95119
FLVCR1	4.612914	4.941861	5.155645	4.471654	4.987578	3.499011	4.919376	5.326628
FLYWCH1	2.324833	2.563168	2.172068	2.674383	2.906417	2.271871	2.486944	2.011174
FLYWCH2	1.213282	1.667198	1.749045	1.429121	1.338967	1.656843	1.71275	1.442719
FMC1	16.7935	22.88076	20.39672	16.51714	19.93279	19.38615	21.74758	19.13716
FMN1	7.175614	7.092369	5.301999	6.412302	7.091106	7.560064	6.130067	7.949223
FMNL1	1.758097	2.275563	2.125678	2.65109	2.620772	1.460507	2.401908	3.606928
FMNL2	16.22667	16.80164	16.00998	15.37958	15.23986	17.55975	17.16564	16.60164
FMNL3	13.42901	11.45986	9.826116	11.51073	12.77302	11.12383	11.06055	10.77208
FMO1	0.078695	0.026576	0.038857	0.094085	0.026235	0.066027	0.1065	0.079815
FMO2	45.75669	40.0462	59.54503	46.04863	73.37309	43.63183	53.78599	5.327164
FMO4	2.487926	2.810775	2.59432	3.463701	2.741983	2.01053	2.566778	2.714254
FMO5	8.623377	9.560263	8.813069	11.13315	11.80025	8.005495	8.401839	8.791588

FMOD	9.801611	17.13433	14.71665	20.64252	17.18476	16.04867	10.63966	17.22985
FMR1	15.66798	15.30849	14.02831	14.83663	15.97994	15.05741	13.68237	15.231
FMR1NB	0	0	0.030576	0.031728	0.030965	0	0	0
FN1	31.22049	24.89598	44.71127	26.31573	36.20288	15.63251	40.09242	47.01464
FN3K	0.362026	0.504319	0.312826	0.881111	0.475221	0.546749	0.75788	0.344231
FN3KRP	12.30984	10.58703	14.09972	9.686663	10.24455	10.77458	10.50631	10.10654
FNBP1	19.0746	18.96148	19.1277	19.20719	20.46824	20.95638	20.56647	17.00854
FNBP1L	16.82427	14.78501	14.72094	13.33335	14.7932	15.22426	14.4945	16.01052
FNBP4	20.95904	18.64987	15.43467	20.4723	19.02597	16.982	17.04259	21.37659
FNDC1	2.854214	2.391771	2.915266	2.907478	3.671394	2.449722	4.714685	4.029487
FNDC10	3.543887	2.704256	2.993385	3.98711	3.544345	3.44663	2.571505	3.074305
FNDC11	0.054033	0.082113	0.1334	0.387601	0.18914	0.081603	0.109687	0.274012
FNDC3A	13.31278	12.84503	11.62428	12.39382	13.14455	8.671526	11.0178	12.43876
FNDC3B	7.50802	7.65921	8.196186	7.53012	8.636539	6.887105	8.022368	9.451723
FNDC4	3.101702	3.797077	3.464168	3.840726	3.212892	2.751118	2.904614	3.61399
FNDC5	0.217774	0.346708	0.276507	0.43571	0.321518	0.428083	0.526288	0.378642
FNDC7	0.203373	0.064388	0.200839	0.586154	0.228824	0.243155	0.038704	0.180484
FNDC9	0.008939	0	0	0	0	0	0	0
FNIP1	6.699917	6.988698	6.402859	6.394916	6.723315	6.328336	6.750111	7.814361
FNTA	75.37284	87.10313	91.688	79.61557	84.93972	84.54112	80.83652	86.85461
FNTB	7.589773	7.861607	6.596569	7.900744	7.430739	6.705298	8.03856	7.687677
FOCAD	6.245516	5.419371	5.753661	5.10141	6.164389	6.045583	6.280503	6.896507
FOLR1	0	0	0	0	0	0	0.01988	0.019865
FOLR2	3.863239	4.833716	4.444594	5.661273	6.220618	3.520116	5.64651	7.954016
FOLR3	0.238815	0.314534	0.330176	0.244731	0.66877	0.264492	0.751433	0.823531
FOPNL	15.42491	16.0652	16.90943	15.55391	15.64303	16.65957	15.19673	14.88262
FOS	4.680598	33.50849	5.314952	7.160249	7.163454	3.191512	5.750256	5.321822
FOSB	0.179046	1.275109	0.358831	0.949786	0.474008	0.23329	1.924222	0.261711
FOSL1	0.676661	0.358351	0.66823	0.693422	0.999742	0.619349	1.342403	0.436732
FOSL2	73.31488	63.28394	66.16161	63.39892	66.53025	64.61025	65.29737	67.74229
FOXA1	1.215197	3.071173	3.267543	4.6631	2.080634	3.896578	3.666759	1.661191
FOXA2	0	0	0	0	0	0	0	0
FOXA3	0.030406	0	0.045041	0.062319	0.015205	0.015307	0.077156	0.046259
FOXB1	0	0	0	0	0	0	0	0
FOXC1	33.2061	30.25297	33.35496	33.61161	29.68586	32.0563	33.88593	32.32506
FOXC2	0.404496	0.339838	0.428685	0.353855	0.424283	0.288062	0.48066	0.540335
FOXD1	1.063044	1.208336	0.934576	1.408217	1.076147	1.187779	1.302682	0.710019
FOXD2	0.407877	0.681271	0.435454	0.80207	0.418955	0.566049	0.436373	0.491948
FOXD3	0.081324	0.205979	0.093696	0.263906	0.027112	0.2047	0.082544	0.096229
FOXE1	19.26942	19.44413	16.9718	16.47067	16.68101	16.21565	17.75702	19.23068
FOXE3	0	0	0.029157	0.030256	0	0.059452	0.029967	0
FOXF1	0.405164	0.377199	0.486627	0.359092	0.810433	0.31973	0.444585	0.533101
FOXF2	0.042237	0.028528	0.097325	0.144278	0.028162	0.056701	0.085741	0.214192
FOXG1	0.011619	0.023542	0.034421	0.059532	0.01162	0.011698	0.023586	0.082488
FOXH1	0	0.037509	0.036561	0.025293	0.024685	0	0	0.012517
FOXI1	0.06301	0.293651	0.062225	0.129142	0.214263	0.393333	0.076747	0.332318
FOXI2	0.059615	0	0.058872	0.274912	0.149056	0.090033	0.423563	0.060463
FOXI3	1.945534	1.859075	1.200808	1.925758	1.348783	1.558157	1.368838	1.749
FOXJ1	0.529622	1.209562	0.948533	0.82791	0.81698	0.39767	1.002242	0.482535
FOXJ2	19.98712	19.92733	18.16827	18.49669	19.53375	17.96152	20.06774	20.21689
FOXJ3	12.18258	12.74079	10.69716	10.7676	12.12147	10.41848	11.33584	13.40783
FOXK1	5.571459	4.134904	3.864449	4.631762	5.272139	3.986411	4.69468	5.022166
FOXK2	12.51319	10.59805	8.396191	11.94679	9.772367	8.599097	11.25608	12.02542
FOXL1	0.038106	0.125471	0.150526	0.117151	0.181029	0.028775	0.067687	0.125609
FOXL2	0	0.018224	0.008882	0.018433	0	0	0	0.009122
FOXM1	2.837062	2.600553	2.173877	2.711886	2.018787	2.392371	3.089338	3.078951
FOXN1	153.7343	125.2093	164.344	166.0635	147.6047	164.384	119.8146	112.8743
FOXN2	5.41872	5.27162	4.186924	4.821071	5.66885	4.211648	5.177825	6.047745

FOXN3	8.918397	8.193716	7.284695	7.802189	8.058652	7.040294	8.836513	8.514901
FOXN4	0	0.007905	0	0.007996	0	0	0	0
FOXO1	18.55136	21.16907	16.42317	18.78936	17.17303	16.67475	17.29872	18.71081
FOXO3	37.28641	32.82344	39.92504	34.57789	32.60952	36.71133	35.78117	39.67018
FOXO4	7.382502	6.08987	6.563135	7.539341	6.596	6.427103	6.702668	6.251113
FOXO6	0.978642	1.173258	0.789266	1.153316	0.848266	0.821106	0.844323	0.595544
FOXP1	3.871575	3.982984	4.201014	4.366823	4.597183	4.578718	4.11546	4.491059
FOXP2	1.406851	1.112108	0.991108	1.427925	1.097844	0.87965	1.200573	1.267831
FOXP3	0.082333	0.095926	0.109765	0.067498	0.10293	0.037303	0.054319	0.133608
FOXP4	4.952371	3.611921	3.506514	4.434218	4.931446	4.0237	3.859364	4.665657
FOXQ1	0.1959	0	0.072547	0.025094	0.024491	0.443785	0.124274	0.173852
FOXR1	0	0.027638	0.02694	0.027956	0	0	0	0
FOXR2	0	0	0	0	0	0	0	0
FOXRED1	4.136051	4.305136	3.888682	5.414251	5.029068	4.720498	4.01119	4.19493
FOXRED2	2.758939	2.390126	2.105565	1.866308	2.512489	2.022467	2.09899	2.477846
FOXS1	1.212425	0.971243	1.011687	1.627715	1.729574	0.965211	0.858566	1.229685
FPGS	2.321245	2.887643	2.128585	3.050293	2.779537	2.718671	2.07558	2.418352
FPGT	3.726441	4.959282	3.833342	4.488701	4.871153	3.282911	4.163889	5.404006
FRAT1	1.2827	1.599426	1.180104	1.381881	0.931996	1.192119	1.212919	1.634539
FRAT2	2.728186	3.160674	2.943654	2.847537	2.450632	3.204627	3.115247	3.445969
FREM1	0.579164	1.322368	0.691917	1.074109	0.842016	0.813524	0.93196	1.312357
FREM2	0.492513	0.624156	0.476208	0.30951	0.526903	0.304092	0.735046	1.032118
FREM3	0	0.004015	0	0	0	0	0	0
FRG1	28.69637	33.01368	35.67198	34.06217	31.91014	34.53397	31.72201	32.02917
FRK	0.036539	0.05923	0.02165	0.127311	0.087706	0.095651	0.051922	0.051883
FRMD1	0	0.015719	0	0.015899	0	0.015621	0	0.015736
FRMD3	3.653464	2.924378	3.065471	3.377532	3.26522	3.328842	3.534688	3.805338
FRMD4A	4.853545	5.076054	4.357336	4.997567	5.016753	4.67627	5.044204	5.546801
FRMD4B	9.877859	10.35338	9.024295	9.271218	10.10108	10.31772	10.7364	10.00693
FRMD5	0.028218	0.028589	0.03135	0.057835	0.042333	0.071028	0.042963	0.053663
FRMD6	33.43027	36.71817	34.0291	30.6531	31.92823	31.25246	32.80348	33.32556
FRMD7	0.050744	0.058755	0.071589	0.066859	0.145003	0.218962	0.088295	0.088229
FRMD8	15.25607	11.97163	10.95884	13.0788	14.51879	14.38787	12.02381	12.30463
FRMPD1	1.028383	0.544093	0.569847	0.591331	0.697105	0.707529	0.736465	0.504128
FRMPD2	0	0	0	0	0	0.0032	0	0
FRMPD3	0.004606	0.023331	0.013645	0.037759	0	0.013912	0.023374	0.018685
FRMPD4	0.002912	0.038347	0.005751	0.017902	0.020384	0.017589	0.005911	0.03839
FRRS1	18.62023	21.87687	17.46933	22.91748	18.64403	23.13009	22.41673	20.87037
FRRS1L	2.252633	2.405451	2.317578	1.886158	2.150876	1.7267	1.923941	2.125488
FRS2	10.6053	11.31283	10.16648	9.974873	10.48644	9.675867	9.40866	10.12634
FRS3	0.475712	0.394327	0.375828	0.416587	0.311415	0.330919	0.289709	0.342125
FRYL	12.58723	10.69604	10.40529	10.32635	11.34855	9.755104	10.94449	12.32595
FRZB	0.900351	1.249272	1.130744	1.263633	0.489384	1.448432	1.480049	1.012425
FSBP	0.520649	0.950627	0.966171	0.961554	0.778213	0.91592	0.836242	0.934261
FSCN1	32.55105	23.45305	21.09132	31.10961	33.08051	24.69671	28.28358	28.11606
FSCN2	0	0.018318	0	0	0	0	0	0
FSCN3	0	0	0	0	0	0	0	0
FSD1	0.015303	0.077519	0	0	0.045915	0	0	0.015521
FSD1L	1.219021	1.201132	0.987289	1.241599	1.066781	1.040253	0.87894	1.179833
FSD2	0.860323	1.319872	0.784871	1.05796	0.958769	0.981693	0.815012	0.930741
FSIP1	0.11646	0.268751	0.089451	0.059672	0.278245	0.071656	0.256114	0.275608
FST	7.174189	8.911187	8.142664	6.898697	7.224261	7.757519	7.760605	8.123561
FSTL1	112.0147	128.4642	152.1871	131.1453	141.3715	122.7358	143.4883	171.3558
FSTL3	13.32346	9.590535	10.75758	10.25098	14.46977	10.48407	10.71253	11.86519
FSTL4	0.014165	0.025113	0.045461	0.043546	0.084998	0.035653	0.046725	0.043099
FSTL5	0	0.007079	0	0	0	0	0	0
FTCD	0	0	0	0	0	0	0	0
FTCDNL1	2.443597	1.995691	1.366631	1.635348	1.720714	1.393328	1.391978	1.352995

FTH1	1617.811	1663.812	1474.605	1466.307	1824.533	1498.752	1610.358	1918.7
FTL	234.6041	291.5224	294.0103	187.8414	282.2658	315.5386	229.1702	316.721
FTO	13.16047	13.18564	12.38196	12.0754	12.93706	12.41056	11.66462	13.34782
FTSJ1	13.44689	12.03525	11.95513	12.95743	13.38251	12.79716	12.66137	11.27268
FTSJ3	10.75075	11.12743	9.369113	11.5413	11.23277	11.32366	11.59647	10.34564
FUBP1	26.49479	25.48765	21.12141	22.34078	24.02972	21.71023	23.58079	25.34267
FUBP3	8.817851	9.518196	8.948166	9.412579	9.973234	10.1409	9.472894	10.31705
FUCA1	24.03903	23.0525	26.99917	23.3175	20.71385	22.43261	26.76131	26.9058
FUCA2	9.991284	11.23948	13.85371	12.13355	12.6731	11.86991	12.01787	14.40026
FUK	2.405817	2.12706	1.317111	3.008193	2.999683	1.696219	2.137476	2.718376
FUNDC1	7.419769	8.921595	9.752373	9.227095	8.609101	8.613624	8.4959	9.092069
FUNDC2	34.83462	32.9202	34.86291	32.10569	35.20611	33.78587	30.5538	31.85264
FUOM	23.43537	19.76703	24.16708	21.92919	12.33015	21.54531	25.59065	21.77885
FURIN	19.91102	18.5355	15.36146	19.83468	19.23494	19.07104	17.14642	22.23292
FUS	113.2615	133.9234	110.4909	143.1331	111.9281	121.7444	129.456	104.8974
FUT1	9.688557	7.339675	10.4298	10.97209	10.28633	10.19415	7.308969	6.46246
FUT10	9.533305	7.859023	7.785349	11.79815	7.786304	7.442672	10.63111	8.736292
FUT11	2.222041	2.467664	1.730163	2.736877	2.842015	2.129662	2.721626	2.492042
FUT7	0.041921	0.099099	0.124196	0.042959	0.097828	0.028138	0.04255	0.127553
FUT8	4.336733	5.579381	3.852635	4.71378	4.654878	4.549052	4.991144	6.017989
FUZ	3.264209	2.262718	2.276269	2.171358	2.248014	2.52254	2.760977	2.497529
FXN	5.264351	5.038368	4.961469	5.155979	4.849946	4.303298	4.32343	5.132515
FXR1	16.39219	18.0176	16.93734	16.71235	16.7668	16.6073	15.97652	16.8758
FXR2	17.61932	18.63372	17.63252	17.71763	18.21558	18.00545	17.86447	17.84145
FXYD1	5.243088	5.628351	4.957428	5.155756	5.589631	6.065126	6.02375	5.600584
FXYD2	0.030893	0	0	0.031658	0	0	0	0.031333
FXYD3	75.57559	69.88377	78.13088	63.72777	71.84976	73.9918	78.43248	48.70664
FXYD4	0	0	0	0.100817	0	0.049526	0.049928	0
FXYD5	22.18009	19.94196	21.52444	22.63111	23.2633	18.27132	22.95136	24.02965
FXYD6	148.1156	127.8615	125.8273	146.0449	139.5606	133.9395	125.324	125.0024
FXYD7	0.851381	1.186013	1.786643	0.472588	0.851491	0.607182	1.656292	0.611647
FYB1	1.146202	1.581505	1.067239	1.431886	1.910584	0.796832	1.490254	2.67933
FYB2	0.016193	0.008203	0.007995	0.008297	0.008097	0.032607	0	0.016423
FYCO1	21.27412	19.42658	20.13511	17.91148	18.08357	17.81994	17.80636	17.04449
FYN	28.2961	28.97842	37.1284	35.40056	36.73293	38.33767	30.56628	31.33621
FYTTD1	26.10782	33.7235	31.47846	31.5913	32.82055	33.7882	27.32859	27.34148
FZD1	13.45838	12.43228	10.82482	12.40487	11.25173	12.11999	14.02576	12.78707
FZD10	53.83798	56.13676	58.03009	57.70237	51.92886	63.77505	59.21834	47.72762
FZD2	6.172228	6.465927	5.854825	7.280338	7.407631	7.778561	7.466632	6.830742
FZD3	3.877086	3.695465	3.283988	3.550258	3.998969	4.570091	3.906126	4.144895
FZD4	14.03383	15.21415	9.689444	12.97591	14.11415	11.28879	13.13314	15.87192
FZD5	1.256767	1.231892	1.353121	1.650555	1.647168	1.402395	1.280224	1.320675
FZD6	25.46808	24.72257	19.64879	23.84682	21.78115	21.82351	23.38073	22.08836
FZD7	32.1635	27.28139	27.9878	31.18182	27.17774	30.80125	28.20668	27.35295
FZD8	1.745249	1.533378	1.344476	1.410005	1.665806	1.093676	1.337759	1.336752
FZD9	0.073238	0.098932	0.08438	0.062544	0.195327	0.036869	0.099116	0.04952
FZR1	13.12926	13.45763	11.99929	14.72658	13.00409	12.55299	13.73086	13.647
G0S2	13.40516	20.20102	15.36687	18.39682	13.57532	12.27593	12.34134	13.01526
G2E3	6.270661	6.572241	6.302454	6.825235	6.098295	6.101804	6.195246	7.275657
G3BP1	26.17622	27.44631	26.57131	26.765	27.773	27.00253	26.19821	26.84712
G3BP2	17.31264	18.66354	15.90497	16.66697	17.45005	16.92887	18.069	17.98922
G6PC	0	0.00661	0.006443	0.020058	0.006525	0	0.013244	0.013234
G6PC3	5.210181	4.918248	3.670169	5.157013	4.019294	4.726568	4.34979	3.805454
G6PD	36.83824	31.69844	32.84859	33.29757	34.53168	33.32125	33.31091	32.07379
GAA	3.319309	6.437621	5.445086	3.410229	2.67447	5.769412	3.90333	3.043682
GAB1	5.974651	6.183704	4.480572	5.868147	5.118039	5.416806	5.262004	5.747274
GAB2	4.706833	3.809135	3.649894	4.150979	4.093736	3.91404	3.812588	4.683901
GAB3	1.009238	1.051532	1.121246	1.351561	0.917608	1.016133	0.89051	1.355114

GABARAP	179.4235	188.8804	196.7998	186.4802	203.6152	189.9444	172.7442	175.5315
GABARAPI	29.88355	28.75418	31.43254	31.02603	32.15756	32.09813	37.86712	33.77087
GABARAPI	105.6265	107.099	125.1668	112.9845	103.6812	115.1508	122.1933	101.9478
GABBR1	7.983441	7.43352	5.623807	7.056797	6.160028	5.943768	5.759689	7.585049
GABBR2	0.101896	0.423799	0.047665	0.065949	0.096545	0.032397	0.152415	0.081589
GABPA	15.01617	17.048	16.58883	16.78002	15.6683	16.39277	15.77165	16.63695
GABPB1	6.85803	6.948035	6.71847	6.971758	7.388633	6.776162	6.627222	8.3542
GABPB2	5.097962	4.726082	4.321599	4.188639	4.963259	4.251584	5.146957	5.573196
GABRA1	0.214428	0.530326	0.112106	0.168036	0.157688	0.342889	0.403282	0.211084
GABRA2	0.047557	0.128484	0.097061	0.055234	0.082444	0.054267	0.074015	0.061097
GABRA3	0.266129	0.223401	0.232777	0.155841	0.319396	0.237325	0.40904	0.20051
GABRA4	0.022633	0.603818	0.022351	0.146891	0.33199	0.121533	0.068917	0.153033
GABRA5	0.092679	0.070422	0.080084	0.047488	0	0.081648	0	0.164498
GABRB1	0.026203	0.026547	0.04744	0.013426	0.026206	0.114322	0.070923	0.031005
GABRB2	0.010889	0.029419	0.03943	0.052076	0.014521	0.0402	0.09579	0.033133
GABRB3	0.009943	0.035257	0.004909	0.005095	0	0	0	0.005042
GABRD	0.015008	0.015205	0.029641	0.030759	0	0	0.015233	0.076106
GABRE	1.931234	2.104946	1.102724	2.663774	2.700416	1.898351	1.709371	2.18152
GABRG1	0.01387	0.042155	0.013697	0.014213	0	0.041894	0	0.09847
GABRG2	0	0	0	0	0	0	0	0
GABRG3	0.032079	0.211252	0.03168	0.065748	0	0.145343	0	0
GABRP	73.69083	45.48363	63.65114	50.26988	55.56637	56.68897	49.94816	45.32885
GABRQ	0.043529	0.0294	0.028658	0.029738	0.116092	0.073044	0.132546	0.014716
GABRR1	0.01714	0.26047	0.050778	0.175643	0.239987	0.207082	0.121778	0.208605
GABRR2	0	0	0.033423	0.052024	0.11847	0.153339	0.051528	0.051489
GABRR3	0.318285	0.322462	0.196449	0.285398	0.298431	0.280402	0.34325	0.30264
GAD1	0.007893	0	0	0	0.015788	0	0.024035	0.016011
GADD45A	40.23442	19.71389	22.00996	21.57824	18.44586	25.26666	17.95291	17.74226
GADD45B	16.70288	15.92053	19.22016	17.0533	16.35487	18.10262	16.20087	16.37666
GADD45G	4.321435	4.523122	4.154541	4.120538	4.078703	5.878115	5.533655	4.513572
GADD45GI	11.8892	12.32406	10.32779	9.419807	10.43192	12.71858	11.22952	11.02567
GADL1	0.043327	0.043896	0.017115	0.11544	0	0.017449	0.017591	0.008789
GAK	19.45669	18.44374	16.23419	18.4667	18.82001	17.63927	18.81896	18.2635
GAL	0.078732	0.039883	0.038875	0.040341	0	0	0	0.159705
GAL3ST1	0	0	0	0.054084	0	0.053137	0	0
GAL3ST2	0.021926	0.011107	0.021653	0.011235	0.021929	0.022076	0.022255	0.044477
GAL3ST3	0.495496	0.501999	0.313668	0.46871	0.41932	0.358171	0.257912	0.270604
GAL3ST4	4.389948	4.658097	3.681113	3.966299	4.169691	4.093019	4.271237	4.215329
GALC	3.868742	4.166152	5.178517	4.726471	3.731055	3.616945	4.220613	3.810372
GALE	5.735336	4.674147	4.520378	5.562211	6.07988	5.464843	5.178629	5.963783
GALK1	7.761188	7.924959	5.813741	8.57969	6.743531	6.93229	7.215969	7.231196
GALK2	3.491956	4.310404	4.307254	3.976059	4.830495	5.347805	4.372705	4.165868
GALM	10.69144	12.07339	14.76538	11.88923	12.2804	11.64585	13.84909	9.742703
GALNS	3.664996	3.23157	2.73275	3.441896	3.79223	3.158329	3.870057	4.734839
GALNT1	11.2865	12.69827	11.52074	10.73943	13.41674	11.94373	12.25392	12.5747
GALNT10	6.067141	5.75953	5.384745	5.146482	5.864694	5.114847	6.172881	6.178046
GALNT11	16.91012	16.72063	15.48604	15.8031	13.09037	16.33371	17.18491	18.01684
GALNT12	1.43643	1.443	1.7178	1.490644	2.267064	1.672027	1.57486	1.721206
GALNT14	0.131921	0.14479	0.075995	0.168985	0.142933	0.254575	0.167374	0.211848
GALNT15	2.180815	1.998422	2.480317	2.285055	1.611316	1.233547	1.952381	2.354761
GALNT16	4.606095	5.290264	6.499726	5.689463	5.586242	4.423434	6.220327	6.981955
GALNT17	2.502316	3.191219	1.681636	1.384558	1.870983	1.094696	2.158467	2.610914
GALNT18	1.639322	1.436675	1.648692	1.679929	2.00164	2.004928	1.796617	2.662295
GALNT2	13.23768	13.26131	13.15544	14.89904	13.6195	13.41892	15.00545	14.50414
GALNT3	7.508836	7.635523	7.140974	7.780226	9.241415	8.566908	7.010624	8.31063
GALNT5	0.172468	0.279571	0.107058	0.146442	0.275985	0.14884	0.280089	0.27488
GALNT6	1.470457	1.438605	0.729183	1.739703	1.773613	1.366129	1.735928	1.504191
GALNT8	7.883912	4.497748	4.107008	5.503791	4.835581	3.44227	2.783927	4.062766

GALNT9	0.04244	0	0	0	0.010611	0	0	0
GALNTL5	0	0	0	0.015665	0.015288	0	0.093093	0.046512
GALR1	0.026656	0.027006	0	0	0	0.026838	0	0.027036
GALR2	0.096183	0	0.047492	0.123206	0.120244	0.12105	0.073219	0
GALR3	0	0.005317	0.005183	0.016135	0	0.010568	0.010654	0
GALT	8.985675	8.734538	7.15493	9.519343	8.561784	9.189695	9.058836	9.708849
GAMT	31.60721	38.03348	38.38788	39.77579	37.37196	37.56412	38.95588	31.02968
GAN	16.0521	12.08493	11.23964	10.79151	14.2916	9.789708	11.43762	10.62774
GANAB	36.19067	33.62641	32.31283	35.59751	35.13351	32.64992	34.07923	34.11861
GANC	1.528121	1.496036	1.086851	1.010175	1.429334	1.387096	1.30593	1.373205
GAP43	0.137202	0	0.067746	0	0.085762	0	0.156667	0.034789
GAPDH	568.6684	508.8593	451.9587	463.0666	476.6009	459.7407	538.7384	501.6902
GAPDHS	0.346401	0.865669	0.456112	0.047331	0.692891	0.488274	0.445356	0.32791
GAPT	0.661415	0.81719	1.226693	0.925774	2.210381	0.454784	0.736833	7.182801
GAPVD1	16.49055	14.07368	13.22158	13.83027	14.86144	15.18936	15.18865	15.66376
GAR1	19.55465	23.72252	26.21074	22.98467	20.1867	21.90633	20.84889	17.66245
GAREM1	4.368391	3.42452	3.481442	4.059112	4.598901	4.150503	3.635566	4.131933
GAREM2	0.08326	0.063264	0.123334	0.135093	0.124906	0.034929	0.133805	0.091482
GARNL3	0.940701	1.013451	1.046738	1.215186	1.159464	0.76704	1.10274	1.001125
GARS	61.41629	58.46958	80.34177	83.61296	74.97021	85.30061	72.21098	55.78176
GART	9.412636	9.811083	8.887809	8.730454	9.640011	8.634544	9.123473	9.970925
GAS1	19.65269	24.86522	25.93325	24.44012	24.26193	24.59628	19.79742	17.12946
GAS2	0.38062	1.019126	0.711482	0.752236	1.386724	0.834873	0.731266	0.703141
GAS2L1	4.440231	2.697427	2.882442	3.177526	3.655188	4.47889	3.021341	3.186792
GAS2L2	0	0.017057	0	0.025879	0	0	0	0.008538
GAS2L3	4.739506	5.422023	3.168822	4.76976	3.872609	5.171444	4.948702	5.284224
GAS6	38.99751	31.94265	35.86887	46.09637	45.51108	38.72505	41.37269	40.90974
GAS7	11.90455	9.944805	10.95781	10.54242	10.79868	7.435136	8.539053	13.4674
GAS8	1.083331	1.551707	1.328068	1.263292	1.242256	1.429235	1.867392	1.477633
GASK1A	1.571098	1.076479	1.381124	1.535564	0.826523	1.261808	1.05082	1.013185
GASK1B	4.647862	3.705091	4.230235	3.732752	3.759572	3.506043	4.199977	5.792786
GAST	0	0	0	0	0.057012	0	0.115719	0
GATA1	0.157749	0.159819	0.111274	0.392594	0.247923	0.136137	0.25161	0.914255
GATA2	1.528285	1.836175	2.041347	1.977755	2.586662	1.765589	1.511434	2.056785
GATA3	42.56505	46.80667	37.34798	40.82761	37.41627	35.46569	38.85323	43.88376
GATA4	0.035118	0.026684	0.01734	0.017994	0.017561	0.026519	0.124757	0
GATA5	0.010626	0.032297	0.020987	0	0	0	0.010785	0
GATA6	5.337969	3.714349	4.903681	5.249688	4.717885	4.632847	3.460825	4.876759
GATAD1	13.38029	13.8501	11.07381	11.97201	13.49373	12.18975	11.7445	12.57392
GATAD2A	18.25168	19.11335	16.84029	19.41797	18.80971	18.00345	18.3078	17.45371
GATAD2B	12.44552	11.21146	11.21822	11.37658	11.44966	10.67892	11.80657	11.49637
GATB	2.816566	3.79229	4.219412	3.835884	3.581841	3.628057	3.642563	4.579609
GATC	9.250501	10.98328	9.875445	9.27356	8.684893	9.166444	8.016126	8.992809
GATD1	5.148334	4.907006	4.275533	5.445473	5.750151	4.77128	4.80999	4.983072
GATD3A	48.61633	55.06315	48.68063	51.28077	47.29932	51.03209	53.37449	47.86323
GATM	78.31595	60.75685	58.36672	60.44207	63.17604	70.77661	54.60029	77.45001
GBA	8.076051	7.782654	6.702301	8.374363	7.437747	7.712862	8.132307	7.650714
GBA2	17.41052	14.01268	14.87906	16.90231	14.69402	15.89209	16.20713	15.69363
GBE1	26.48844	29.24453	25.77805	22.69419	24.56591	25.85374	29.03582	30.56972
GBF1	14.3274	12.46353	11.52352	11.88309	13.01811	11.79358	11.88515	13.07411
GBGT1	3.434623	1.688977	1.715749	4.075442	3.716301	3.538978	2.701251	2.291788
GBP6	2.610059	3.03666	3.515299	3.109623	3.963345	3.49317	3.004591	3.249832
GBX1	0	0.008554	0	0	0	0	0	0
GBX2	0.106625	0.108025	0	0.054633	0	0.026838	0	0.027036
GC	0	0	0	0	0	0.015492	0	0
GCA	1.165506	1.215278	1.285405	1.551817	1.403893	1.327647	0.984385	1.604892
GCAT	4.257913	4.654998	5.784651	5.423414	4.065991	4.977281	4.358407	3.464581
GCC1	3.657121	2.76237	2.556177	2.939869	2.670043	2.479284	2.84585	3.38772

GCC2	5.704798	6.863355	5.658281	6.640601	6.76703	5.990051	6.093354	5.823854
GCDH	12.15662	13.41582	12.14806	14.9048	15.84907	13.40535	11.4723	13.85621
GCFC2	2.911786	2.505979	2.798429	2.651682	2.996224	2.605264	2.534995	2.703582
GCG	0	0	0	0	0.023844	0	0	0
GCGR	0.182474	0.137161	0.081381	0.162866	0.211933	0.148162	0.227033	0.119401
GCH1	3.56926	3.407982	4.1841	3.341902	3.877899	2.533648	2.893027	2.812717
GCHFR	10.53897	6.814143	8.446409	8.466348	10.77868	9.491214	8.842537	4.109086
GCK	0.244493	0.150775	0.073484	0.054467	0.085052	0.085622	0.086317	0.118596
GCKR	0.024606	0.062322	0	0.012608	0.012305	0.006193	0.006244	0.037434
GCLC	17.94406	18.23996	17.42612	16.96053	13.10205	16.43105	15.24072	19.3332
GCLM	7.092302	9.014285	8.499648	8.568085	7.568336	7.883527	10.26763	9.211235
GCN1	20.41364	18.83851	16.98974	17.64358	17.83013	18.40424	18.08535	18.24306
GCNA	0.846456	1.02813	0.803581	1.188513	1.244124	1.181834	0.89238	1.237956
GCNT1	2.128784	2.376796	2.059356	1.814219	2.357173	2.766641	1.52132	3.949149
GCNT3	0.066711	0.030039	0.409921	0.07596	0.704268	0.029852	0.030094	0.255609
GCNT4	5.139307	4.858348	5.113005	4.202846	5.764157	4.687111	4.408405	5.567717
GCNT7	0.160114	0.149737	0.048652	0.100972	0.277156	0.105405	0.068757	0.049967
GCSAM	0.120643	0.081484	0.039713	0	0.04022	0.080978	0.122453	0.040787
GCSAML	0.831361	1.604328	0.938288	1.379355	1.069031	1.275491	1.687663	2.810654
GCSH	7.721149	10.78164	9.55626	8.120626	8.992237	8.004044	9.486854	9.73731
GDA	0.581361	0.617307	0.568597	1.042582	0.497575	0.388345	1.038316	0.419549
GDAP1	0.199231	0.888123	0.24172	0.717499	0.791336	0.498616	0.514216	0.577336
GDAP1L1	0	0	0	0	0	0	0	0
GDAP2	4.073351	3.088978	3.775665	3.050095	3.67859	3.419002	3.815157	3.247804
GDE1	20.68098	22.24682	22.1729	21.01868	21.67183	24.49274	22.25343	21.37278
GDF1	1.438529	2.033175	1.718757	2.293141	2.895193	1.931142	1.424055	1.567082
GDF10	0.172773	0.828523	0.295741	0.377711	0.299511	0.231937	0.280583	0.467286
GDF11	1.381833	1.699961	1.573485	1.300464	1.903791	1.391273	2.304206	1.258493
GDF15	0.044963	0.091105	0.133207	0.253419	0.112421	0.04527	0.022818	0.136808
GDF2	0.019014	0.009632	0	0	0	0.009572	0	0
GDF5	0.009828	0	0.048526	0.010071	0.049145	0	0.009975	0
GDF6	0.020064	0	0	0	0	0	0	0
GDF7	0.020415	0.020683	0.060482	0.062763	0.020418	0.020555	0.020721	0.207058
GDF9	0.313117	0.546334	0.687145	0.802182	0.452338	0.420341	0.353126	0.35286
GDI1	71.45151	75.34303	67.18842	76.50792	73.10615	66.61387	71.59322	78.80518
GDI2	247.405	263.1983	255.994	257.542	225.4277	218.8086	251.9653	248.1688
GDNF	0.199448	0.286259	0.131308	0.144775	0.29921	0.292848	0.269919	0.193858
GDPD1	1.185741	1.122175	1.346261	1.382463	1.398929	1.007974	1.434143	1.41866
GDPD2	0.02074	0.273164	0.040964	0.063762	0.062229	0.031323	0.07368	0.126214
GDPD3	15.7806	15.76462	18.15714	19.96992	20.16263	19.38632	13.981	10.8935
GDPD5	3.069308	3.794439	2.898755	3.588429	3.094067	3.237432	2.83101	3.230357
GDPGP1	1.197546	0.691278	0.715075	0.556525	1.03058	1.275828	1.074171	1.482935
GEM	2.423552	3.067131	3.44095	3.436879	4.529444	3.499953	3.031398	3.12843
GEMIN2	6.961408	7.125478	6.732922	6.118926	6.416807	7.601475	7.298928	8.064992
GEMIN4	3.442994	2.593582	2.051781	2.478919	2.174413	2.831485	2.523069	2.641583
GEMIN5	5.556672	6.036907	5.496888	5.203847	5.562177	6.389736	5.829481	6.140616
GEMIN6	3.435352	3.914506	3.854119	3.36079	3.373469	2.917645	3.281307	3.523761
GEMIN7	6.369051	6.320952	5.754854	7.037416	7.041528	7.437699	6.046808	6.811268
GEMIN8	5.811825	7.883647	6.315751	6.254821	7.466363	5.337377	8.539978	8.114269
GEN1	1.986803	2.3653	1.750648	2.008595	1.719442	1.508701	1.514151	2.150782
GET4	19.34556	19.213	17.87494	20.14189	18.05243	19.60815	18.22588	16.307
GFAP	0.197903	0.133667	0.148905	0.347666	0.113102	0.180278	0.057392	0.229395
GFER	9.401489	9.694528	8.977229	10.00209	11.7474	9.3212	9.178293	10.11763
GFI1	0.162217	0.260214	0.120147	0.083118	0.175758	0.061247	0.089185	0.486723
GFI1B	0	0	0	0	0	0	0	0
GFM1	15.79151	16.09658	14.72602	13.64314	14.32364	17.51957	16.06105	16.73444
GFM2	12.1916	14.08211	11.83442	13.70683	11.50624	14.14023	13.49359	13.75841
GFOD1	5.891697	5.064181	5.050102	5.048586	4.250062	4.119005	4.781134	5.97557

GFOD2	6.281573	5.044926	5.594252	6.522998	5.261973	6.201829	4.667856	4.007935
GFPT1	7.703863	9.2684	7.148664	7.850756	9.832271	8.161583	8.288051	8.27512
GFPT2	6.419181	5.84239	7.258333	7.256654	7.830686	3.893278	7.479641	14.37384
GFRA1	11.14935	14.49667	9.638206	11.49333	10.32358	11.24217	13.88549	13.53948
GFRA2	13.5391	10.60934	10.81187	10.14892	10.99985	11.11564	11.16341	9.468187
GFRA3	3.524507	1.744209	1.773131	4.603733	2.120889	2.060708	3.554875	2.697872
GFRA4	0.019587	0.158749	0.038685	0.12043	0.058767	0.039441	0.119282	0.178788
GFY	0	0	0	0	0	0.018311	0	0
GGA1	10.11217	9.005054	8.318274	8.690545	9.712658	8.01242	8.978145	9.298012
GGA2	19.09632	19.09121	17.44903	16.88316	17.65206	18.37638	19.95429	18.87584
GGA3	11.73692	9.904246	10.92611	10.17382	12.02792	11.31441	10.05479	11.8086
GGACT	55.14477	38.54682	50.74422	43.10912	46.87077	49.62804	42.6041	52.07839
GGCT	25.27981	25.02177	31.15628	37.42488	29.96347	28.11274	27.99991	24.5496
GGCX	13.1525	13.6153	11.34241	14.02423	14.95316	14.00367	12.12296	13.9614
GGH	27.24703	30.7625	37.92181	25.83595	29.43484	36.44235	22.40437	20.66704
GGN	0.011443	0	0.0113	0.035179	0.034333	0.011521	0.011614	0.023211
GGNBP2	21.30233	21.3206	21.29161	21.85937	19.25101	20.9862	21.21464	20.72392
GGPS1	11.67051	12.01001	13.40742	10.76021	12.74223	11.39672	11.93042	11.88752
GGT5	2.06062	3.516652	2.263467	4.099108	4.441375	3.550283	3.17644	3.60992
GGT6	9.949273	10.43984	9.672346	10.65322	10.68865	10.29244	11.66138	8.640109
GGT7	0.859882	0.969174	0.647491	0.726975	0.849243	0.530276	0.960058	0.752206
GGTA2P	16.28128	17.4084	18.68502	19.44302	18.48543	16.11623	16.21382	22.066
GH	0	0	0	0	0	0	0	0
GHDC	1.099701	1.377692	1.48303	1.345059	1.430979	1.333419	0.828146	1.127349
GHITM	53.13776	62.7716	68.00955	62.96792	65.86474	64.31705	60.11306	54.94481
GHR	8.352724	11.59677	9.725662	7.92306	10.93534	8.379426	9.047297	10.93666
GHRH	0.05643	0	0	0	0	0	0	0
GHRHR	0.124362	0.023624	0.184218	0.071686	0.069963	0.289551	0.19723	0.078833
GHSR	0.111511	0.081592	0.1713	0.374561	0.278813	0.286918	0.220079	0.295312
GID4	9.143592	7.982875	8.455256	8.495859	8.516507	8.784406	7.722148	7.582614
GID8	26.01075	26.63957	26.65596	26.04997	24.61064	24.92998	25.90278	25.08998
GIGYF1	7.081924	6.859488	5.483508	7.285259	6.557464	5.935391	6.591773	6.756511
GIGYF2	19.88206	19.40938	18.75566	18.93411	19.24563	19.32219	17.76884	19.44508
GIMAP1	0.352463	0.503586	0.080324	0.324148	0.325392	0.100092	0.155942	0.183323
GIMAP2	0.016631	0.067395	0.04927	0.017042	0	0	0.05064	0.067469
GIMAP6	2.09514	2.701537	1.062731	1.512685	2.133509	1.016373	1.672255	2.414734
GIMD1	0	0	0	0	0	0	0	0
GIN1	2.179706	2.812577	2.120419	3.011603	2.689737	2.675006	2.03629	2.111748
GINM1	19.71007	21.44508	22.9182	22.56546	19.82057	23.45285	19.93999	19.45422
GINS1	5.665746	5.689137	4.818909	5.361449	4.999834	3.931703	5.674141	5.038466
GINS2	1.605049	1.352049	1.068573	1.626325	1.310659	1.24076	1.519296	1.35963
GINS3	3.076954	2.566482	2.660314	2.342718	2.323463	3.185067	3.323791	2.581831
GINS4	6.410322	5.054717	5.186391	7.028154	5.8859	6.220833	7.008181	6.360579
GIPC1	32.48358	28.94271	35.49401	34.57065	29.04251	29.04746	35.37467	29.45214
GIPC2	1.450427	1.882564	1.835024	1.922113	1.37488	2.216895	2.885243	1.051612
GIPC3	0.277745	0.300576	0.19948	0.388125	0.252528	0.324131	0.205027	0.083229
GIPR	0.042666	0	0.016854	0.043723	0	0.051549	0	0.069238
GIT1	12.28811	11.74691	10.24057	12.67934	13.4917	12.14711	11.93288	12.05672
GIT2	7.855469	8.312095	6.783883	8.237165	8.964346	6.659033	7.116665	9.745123
GJA1	533.6103	491.4883	483.9337	499.6535	505.6056	549.1507	543.1841	532.7259
GJA10	0.01604	0	0	0	0	0	0.01628	0
GJA3	0.043027	0.261551	0.127473	0	0.021516	0	0	0.02182
GJA4	3.397348	3.726132	2.200768	3.35374	4.971992	2.996911	3.464127	4.109565
GJA5	8.312404	5.97038	6.356638	6.937472	11.73211	7.117723	6.634775	6.742338
GJA9	0.061799	0.068301	0.077673	0.069087	0.117994	0.07919	0.028512	0.051282
GJB1	0.861985	1.335632	2.503661	2.857854	1.977751	3.050321	3.229465	1.478521
GJB2	172.0737	139.6149	153.5117	158.6881	157.7137	174.8384	159.2399	168.6181
GJB3	8.434068	8.196917	13.10524	13.76758	9.987825	12.20398	11.98481	7.464048

GJB4	6.307061	5.029749	9.155106	8.202407	7.827846	7.446759	6.992985	5.138029
GJB5	14.99937	12.42564	12.43921	14.22446	14.54547	11.61841	14.59352	13.63698
GJB6	83.07693	64.38929	64.52222	71.14121	72.15917	93.19449	70.04684	78.43869
GJB7	0.042645	0.172818	0.014038	0.043701	0.170601	0.157432	0.100997	0.47577
GJC1	2.805637	2.65523	2.733348	2.832092	2.818601	2.998182	2.898878	2.7476
GJC2	0.035587	0.042063	0.035144	0.060781	0.047455	0.011943	0.042141	0.144374
GJC3	0.176402	0.702955	0.476158	0.903859	0.611607	0.745951	0.501335	0.703726
GJD2	0.011102	0.044992	0.043856	0.011377	0.022208	0.022357	0	0
GJD3	0	0.064939	0.042199	0.021895	0.106843	0	0	0.10835
GK	0.867144	0.923867	0.944735	1.387399	0.967969	0.828006	0.874473	0.873814
GK5	3.719904	3.831769	3.769147	4.045872	4.785327	4.761703	3.810793	4.64244
GKAP1	8.210379	9.798045	11.05948	10.01391	9.873879	9.686475	9.168592	9.144658
GKN1	0.097354	0.147947	0.192281	0.049883	0	0	0	0.197479
GLA	5.774683	6.856019	5.471056	5.806766	6.623695	5.21455	6.850398	6.698817
GLB1L	4.038419	4.559714	3.147235	4.549804	3.795632	4.286454	4.691621	4.256291
GLB1L3	0	0.014617	0	0	0.014429	0	0.007322	0.007316
GLCCI1	11.86955	12.06412	10.99063	11.18917	10.94565	9.290657	13.77053	12.54983
GLCE	10.63578	10.85056	9.367059	11.24153	9.937199	9.502001	9.64367	13.82545
GLDC	0.132336	0.067037	0.188771	0.067807	0.176471	0.022207	0.119397	0.186417
GLDN	10.06662	2.439698	2.71596	4.50397	2.500531	5.034575	4.23397	3.296539
GLE1	10.48738	10.60769	8.591168	9.949449	8.563807	9.749445	9.342325	9.543511
GLG1	21.47816	19.17718	19.08513	21.08831	20.1127	18.31297	18.85434	21.00924
GLI1	7.124402	6.457793	4.831256	5.890263	6.709834	6.030625	5.204751	5.647335
GLI2	7.571511	5.230343	4.902503	5.462696	4.658999	4.794351	5.651183	6.105849
GLI3	7.935264	6.359122	5.196518	6.110958	6.432642	5.3727	6.292202	7.484099
GLI4	3.396525	2.320136	2.070967	3.203787	2.714998	2.623087	2.317903	2.648901
GLIPR1	2.061314	2.390299	1.348911	1.501567	1.987065	1.275243	1.991403	3.476034
GLIPR1L2	0	0	0	0	0	0	0	0
GLIPR2	6.925621	6.741355	10.83266	6.757001	10.81985	6.547569	8.576303	10.85003
GLIS1	0.315285	0.350335	0.170744	0.198026	0.488246	0.532478	0.402599	0.381665
GLIS2	2.722888	2.494404	2.597291	3.628341	3.775401	3.084175	2.507996	2.721301
GLIS3	0.319752	0.397573	0.240414	0.264372	0.316159	0.299986	0.32086	0.751794
GLMN	5.301301	5.511361	4.76123	5.651233	5.344659	4.542796	5.521567	4.641114
GLMP	6.056263	5.77962	4.274067	5.620923	6.943265	5.161241	4.251687	4.441599
GLO1	36.41349	40.14609	42.14142	38.96859	39.61737	39.92451	37.29556	37.78489
GLP1R	0.162256	0.142756	0.345769	0.297545	0.230604	0.352523	0.377053	0.164566
GLP2R	0.057384	0.356091	0.056669	0.154365	0.078914	0.223883	0.014561	0.138228
GLRA3	0	0	0	0	0	0	0	0
GLRA4	0	0	0	0	0	0	0	0
GLRB	0.007478	0	0	0.038315	0.05983	0	0	0.015168
GLRX	57.58063	75.45038	82.14691	88.26583	72.52211	81.48624	52.83867	70.05273
GLRX2	44.98872	63.72962	58.13531	35.54514	51.43459	38.5247	39.6506	38.34359
GLRX3	73.53537	83.00065	83.38155	75.49334	79.60517	82.41809	74.41311	72.21844
GLRX5	27.9034	28.38546	20.49739	26.74882	14.89708	30.42559	20.05171	16.12207
GLS	5.293656	5.299356	4.539894	5.216696	6.094482	4.804402	4.977346	6.514075
GLS2	0.23068	0.112526	0.194057	0.166351	0.187986	0.24946	0.173437	0.346613
GLT1D1	0.049644	0.080472	0.117661	0.162795	0.0993	0.039986	0	0.090631
GLT8D1	12.47646	12.74766	11.94129	9.660461	12.45155	11.98767	11.84269	11.79343
GLT8D2	26.47492	31.1443	33.91136	29.93388	30.79369	28.54103	31.99525	37.94923
GLTP	122.0787	128.29	120.0446	119.3436	121.2286	118.8077	112.5016	111.3791
GLTPD2	0	0.013497	0.013156	0	0.013324	0.026826	0	0.054046
GLUD1	28.77795	32.53263	28.39424	37.65226	35.33453	28.59027	39.64347	37.34261
GLUL	151.9425	122.8228	147.1639	129.7194	119.7272	116.4056	136.6311	197.6054
GLYAT	0.320977	2.449758	0.063395	0.570141	0.70624	0.107723	0.086878	0.195328
GLYATL2	18.6104	50.5461	77.83628	135.7423	81.66094	64.47047	121.7182	50.471
GLYATL3	0	0	0	0	0	0	0	0
GLYCAM1	0	0	0	0	0	0	0	0
GLYCTK	0.597143	0.736834	0.695547	0.957128	0.80395	0.655178	0.567248	0.597879

GLYR1	36.39562	37.36492	33.78191	34.74182	35.87019	32.4499	34.86525	36.98945
GM2A	40.44447	37.55741	42.56269	38.84358	36.90891	32.19692	41.63703	40.10953
GMCL1	30.20818	32.68041	38.6922	34.31436	32.23905	37.4224	29.69281	30.18809
GMDS	16.44478	17.33	14.75364	16.47596	14.83159	14.52444	17.11992	16.21351
GMEB1	3.198518	2.967339	2.606529	2.832764	3.054171	2.63173	2.684769	2.987866
GMEB2	4.35787	3.476183	3.9254	4.501134	3.469895	4.175584	4.085082	4.357764
GMFB	14.65335	17.37301	18.12313	16.17458	16.15354	17.93174	17.01825	18.57913
GMFG	6.881824	10.15406	7.708301	10.83875	12.28726	8.974937	10.73541	12.41372
GMIP	3.17489	3.308899	2.685283	3.580443	4.026099	2.607736	3.723622	4.321696
GML	0	0	0	0.094818	0.046269	0	0.046957	0
GMNC	0	0	0	0	0.009269	0.009331	0.018814	0.0188
GMNN	21.30655	25.5278	23.09771	23.85124	24.90282	29.86244	28.13025	22.4919
GMPPA	29.41526	28.37237	25.87613	29.29273	27.61662	28.13304	26.38888	26.60742
GMPPB	9.295795	8.042933	8.828181	11.31654	10.11133	9.154352	9.52132	9.479741
GMPR	1.092444	1.144946	0.837025	1.003693	0.753507	0.455134	1.261773	0.878755
GMPR2	27.41122	27.25235	24.21594	24.97659	27.95975	24.53937	29.44815	26.67933
GMPS	17.06055	18.56829	16.05848	17.73589	16.82459	16.92021	19.97521	18.30401
GNA11	19.86671	18.18485	17.64673	19.16713	20.09124	18.04509	19.58807	20.17654
GNA12	15.31778	12.16949	15.40373	16.70257	20.53903	15.57184	15.49734	15.74488
GNA13	28.98992	31.2639	28.27671	29.77825	28.61434	28.14357	30.45445	28.98549
GNA14	1.119567	1.394194	0.87528	1.290711	1.43463	1.690822	1.811074	1.372068
GNA15	9.004061	8.172555	8.213897	9.727685	9.480574	8.015455	9.64834	8.569844
GNAI1	10.49807	12.28609	8.000729	9.989049	9.458865	8.998486	8.404979	9.808515
GNAI2	73.01303	75.63853	70.22998	78.74804	82.4204	72.32288	75.00079	80.91997
GNAI3	66.98281	66.2557	65.59562	60.14836	63.93687	64.11886	69.46658	70.80941
GNAL	3.122055	2.486116	2.867213	4.979595	3.644894	2.98438	2.231784	2.267066
GNAQ	7.542765	7.662889	7.06563	7.750978	7.789913	8.640189	7.715189	7.565516
GNAT1	0.021293	0.032358	0.021027	0.01091	0	0	0	0.064787
GNAT2	0.002578	0	0.007636	0	0	0.00519	0	0
GNAT3	0	0	0	0	0	0	0	0
GNAZ	2.073271	1.680385	1.569703	2.165096	2.902955	2.097375	1.072227	1.271685
GNB1	123.9001	122.3429	113.95	123.4053	117.9949	116.2699	125.2759	125.8341
GNB1L	6.401215	5.985445	4.616402	7.486569	5.427052	5.747235	6.616448	6.671028
GNB2	77.04251	72.26165	74.21745	76.50982	81.05144	71.02482	86.79857	81.67693
GNB3	2.105073	1.711773	1.039422	1.60372	1.897581	1.213109	1.124552	1.657465
GNB4	16.42605	13.06446	13.6197	12.017	17.68122	13.51182	15.3103	21.30577
GNB5	3.771411	5.201033	4.123018	3.593387	4.768487	4.063866	4.378494	4.656642
GNE	2.998537	5.043154	3.62107	4.719735	3.667232	4.41995	5.28859	3.937719
GNG10	18.29819	37.47449	31.13037	30.32422	31.6686	30.37851	23.41909	20.69332
GNG11	78.20088	103.6413	112.5062	93.39818	111.174	102.5256	85.75046	88.11095
GNG12	43.78377	40.78581	40.33183	40.9936	43.70227	43.19866	42.18822	48.07875
GNG13	1.205882	0.643004	0.626767	0.390238	0.825184	0.766813	1.095132	1.480533
GNG14	0.01746	0.035379	0.017243	0.017893	0	0.03516	0.035445	0
GNG2	4.573731	7.448445	5.58208	6.580006	5.468495	6.070535	6.607268	6.482387
GNG3	0.197803	0.0668	0	0.033784	0.065943	0.033192	0.100385	0.10031
GNG4	116.4934	99.55934	126.6852	86.45452	96.64175	138.018	95.0739	84.59741
GNG5	93.12696	111.9771	128.8541	109.8448	112.8225	116.4926	106.1363	112.618
GNG7	2.372757	2.356452	2.20445	2.687481	2.76337	2.239656	2.083538	2.113634
GNG8	0.325636	0.612689	0.413458	0.651513	1.132118	1.030418	0.755475	1.431176
GNGT1	0.306131	0.664605	0.302317	0.896327	1.443378	0.792573	1.376061	1.064535
GNGT2	1.0261	1.451276	1.093577	1.415904	2.336966	1.472949	1.60864	1.308612
GNL1	28.58848	28.80389	22.64603	25.47158	27.39339	29.30251	30.03115	26.24414
GNL2	23.36384	24.69178	19.93389	23.40781	21.99094	21.30245	22.57071	21.74779
GNL3	35.97358	40.09174	37.75557	38.25324	38.09117	42.42459	39.21951	34.86016
GNL3L	27.14238	28.27642	26.53658	26.93537	24.78586	24.82692	27.84747	27.55167
GNLY	1.507422	0.577862	3.339381	3.214774	7.538086	4.183986	1.778147	2.355304
GNMT	33.31485	25.29028	31.02306	39.30916	31.01966	34.4873	30.0521	26.07948
GNPAT	30.93361	28.67652	30.28079	27.85008	31.81767	29.47524	26.38224	27.91849

GNPDA1	13.00616	12.89598	12.35605	13.78537	13.97228	15.29168	13.95981	12.7512
GNPDA2	9.216264	10.07111	9.558223	7.825743	9.915747	10.80819	10.3998	9.613016
GNPNAT1	4.858936	5.609095	4.902933	4.805723	5.325405	4.785548	5.555013	5.615249
GNPTAB	25.51828	28.03818	24.46408	28.06581	25.43104	25.78912	30.37644	27.05896
GNPTG	26.94447	29.56925	22.31007	30.66749	24.09643	27.63254	27.63582	25.95943
GNRH1	0	0	0	0	0	0	0	0
GNRH2	0.02142	0.036169	0.049358	0.021951	0.035705	0.100644	0.036236	0.036209
GNRHR	0.008951	0.036273	0.061875	0.03669	0.035808	0.009012	0	0.009078
GNS	26.62037	27.23793	29.81467	27.20699	29.70399	26.14567	31.45633	27.9947
GOLGA1	7.091849	6.969375	6.974302	6.988929	7.571528	7.271203	6.766333	7.31868
GOLGA2	36.24146	36.11577	35.3238	38.01123	35.39506	35.61066	37.17703	35.8943
GOLGA3	8.787336	8.736751	6.881717	8.922878	8.593328	8.061558	8.250741	8.813481
GOLGA4	21.98464	24.567	22.04364	23.38383	22.39565	24.88605	22.73224	21.51656
GOLGA5	13.72845	16.18772	13.70615	16.0136	14.67437	15.14682	14.99449	13.14977
GOLGA7	62.81136	62.49671	68.51708	61.4496	63.57881	65.5778	55.48425	58.25605
GOLGA7B	30.17582	31.05519	34.34439	29.151	26.37246	30.91235	34.62347	24.92988
GOLGB1	10.92687	11.44232	10.08762	10.63176	11.15564	10.32995	11.32405	11.47261
GOLIM4	53.40631	57.11278	53.01226	50.57704	50.48849	53.13263	57.77641	53.53051
GOLM1	12.32359	13.39449	11.95612	15.36391	11.45863	11.53542	15.81419	12.85995
GOLPH3	24.01154	24.33721	22.23161	22.93103	24.58741	22.77075	24.56195	23.96261
GOLPH3L	13.48569	14.30714	13.26381	11.72442	14.76892	10.67743	12.81172	14.0832
GOLT1A	0.036444	0.036923	0.215941	0.112041	0.036449	0.073386	0.110973	0.036963
GOLT1B	9.465967	11.1619	12.54965	11.50916	10.93512	11.53216	11.59744	10.6748
GON4L	8.98427	9.206403	7.476382	7.933946	8.009914	7.242655	8.465465	9.355622
GON7	9.433385	11.3636	11.49978	10.70104	10.73402	9.469997	11.13212	11.36205
GOPC	13.89672	13.47181	12.66651	13.46371	14.14321	12.37818	11.83295	14.07594
GORAB	2.087237	2.641943	1.977312	1.752515	2.517757	2.181706	2.274876	2.127724
GORASP1	12.45778	13.34335	10.96085	11.68601	12.57076	12.27379	12.47886	12.51464
GORASP2	40.78805	43.52363	40.25572	46.56212	48.44435	42.93392	44.40919	39.01749
GOSR1	14.11044	16.28798	13.89941	15.07489	14.86689	15.95951	15.76938	17.4146
GOSR2	18.60077	20.73871	18.71668	20.28131	18.91336	20.75338	20.88772	21.22057
GOT1	12.52037	15.68597	15.20706	18.4152	16.46305	15.54634	13.11948	12.68443
GOT1L1	0	0	0	0.031869	0.015552	0	0	0.015771
GOT2	50.64187	60.28789	56.61445	58.34705	54.5478	54.7708	56.60798	52.97547
GP1BA	3.335784	3.941262	3.567977	3.219557	2.541438	1.097816	1.472504	1.93062
GP2	0	0	0	0	0	0	0	0.00541
GP5	0.05526	0.125967	0.109143	0.297301	0.165801	0.278187	0.280444	0.196163
GP6	0	0	0	0	0	0	0	0
GPA33	0.181056	0.258963	0.31553	1.517071	0.010652	0.310971	2.918733	1.263832
GPAA1	24.44952	20.94891	21.55359	22.15608	22.1564	22.00216	22.77714	22.44118
GPALPP1	5.471161	8.225704	5.921995	6.980735	7.264376	7.503007	6.230284	5.624218
GPAM	16.9088	13.6488	13.8147	14.79672	14.36281	14.31675	11.0276	16.04796
GPANK1	29.29991	31.10955	29.70832	24.98045	29.94316	29.35523	22.60065	27.15548
GPAT2	0.509461	0.632017	0.790606	0.586009	1.310212	1.716784	0.559315	0.432352
GPAT3	1.142824	1.279698	1.237483	1.088947	1.564067	1.069885	0.864887	1.830147
GPAT4	19.03064	20.36832	17.2814	20.25604	19.8883	19.1106	20.06291	20.29991
GPATCH1	5.119417	5.719034	4.635071	5.162658	4.494794	4.643513	5.288176	4.70523
GPATCH11	5.828572	4.717965	5.126947	5.135504	4.170672	5.91074	5.842813	4.94254
GPATCH2	3.435425	3.560218	3.577604	3.079017	3.188577	3.696529	3.490759	3.62112
GPATCH2L	7.203646	7.929961	6.932462	7.920359	7.416781	6.802159	7.637365	7.353432
GPATCH3	5.481597	5.941615	5.648089	4.85937	5.693671	5.798321	5.134803	5.907945
GPATCH4	7.617893	8.299462	7.369756	8.871849	8.08439	8.326019	7.826649	9.18978
GPATCH8	15.06675	13.52487	10.87864	12.24292	12.52903	12.4953	12.74396	13.91173
GPBAR1	0.012352	0.137653	0.024396	0.075947	0.024707	0.012436	0.100297	0.238025
GPBP1	19.71268	22.67255	17.91529	20.70672	20.32796	21.6179	17.59009	21.69057
GPBP1L1	38.95615	43.76657	39.05123	43.37026	37.62436	45.87012	42.22938	36.94636
GPC1	19.56794	20.00428	18.10751	21.90313	20.08526	18.44425	18.41404	18.21189
GPC2	0.030242	0.045959	0.029865	0.077478	0.090738	0.13702	0.046044	0.015336

GPC3	78.4074	100.2227	85.40578	87.87792	88.10492	85.12373	88.29135	84.49938
GPC4	11.21846	9.40299	11.37252	10.04308	8.927804	8.77091	10.44855	10.15173
GPC5	0.025176	0.012753	0.012431	0.025799	0	0.063369	0.025553	0.038301
GPC6	1.307104	1.749758	1.299281	1.128678	1.097252	1.044197	1.174465	1.547388
GPCPD1	3.85567	5.423154	3.429206	4.663684	4.851819	4.916153	4.526996	6.134288
GPD1	1.719906	3.373545	2.145901	2.916479	4.126549	2.085959	1.584975	1.570397
GPD1L	6.537665	6.86672	6.374909	6.355135	6.799227	6.630676	7.234547	6.616818
GPD2	2.551607	2.629284	2.446588	2.887467	2.879108	2.384594	2.811238	2.667559
GPER1	0.028396	0	0	0.0097	0.018933	0.00953	0.048037	0.0192
GPHA2	0.643864	1.036029	0.598439	2.018252	0.378793	2.478653	0.999505	0.845098
GPHN	7.582722	6.352619	7.022343	6.522338	7.703806	7.772705	8.166609	7.534069
GPI	78.6246	72.91081	74.73893	79.22419	82.14742	75.65283	74.80762	76.53926
GPIHBP1	0.077728	0.121702	0.125607	0.079653	0.070671	0.085374	0.086066	0.157669
GPKOW	10.56651	10.81026	9.865887	10.53304	10.78684	10.52268	10.88875	10.63513
GPLD1	1.161634	0.876606	1.251018	1.14142	0.626312	0.770087	1.586634	1.570894
GPM6A	1.367144	3.059782	1.620131	2.00599	2.187713	1.90205	1.810254	2.079909
GPM6B	7.307181	11.93324	11.0897	9.20507	11.49148	8.964151	9.077093	12.42855
GPN1	14.0028	15.05242	14.52623	13.54122	13.52793	14.94238	14.21284	14.45218
GPN2	7.344357	7.229015	6.147228	7.052062	7.703614	6.237256	6.727252	6.737326
GPN3	11.87419	13.20329	11.25912	12.10146	12.12042	13.17055	12.18475	13.58172
GPNMB	30.21053	27.91662	45.15997	34.66315	26.79107	40.9693	40.70727	32.20744
GPR1	1.44913	1.126503	1.548079	2.185509	1.203207	1.468206	2.155421	1.386565
GPR107	12.84398	11.95724	9.714173	11.48281	11.48131	10.31203	10.43993	11.77586
GPR108	18.71864	17.40494	18.98817	21.05079	18.85132	18.76307	19.98484	20.8464
GPR12	0	0	0.08852	0	0	0	0	0.022728
GPR132	0.239547	0.408742	0.336167	0.762282	0.39089	0.253877	0.524671	0.984615
GPR135	0.195232	0.152841	0.078872	0.109128	0.13313	0.196566	0.126102	0.135008
GPR137	1.442154	1.432243	1.2649	1.079239	1.698545	1.10811	1.097839	1.039275
GPR137B	8.638844	7.955611	7.444794	7.750203	8.470955	7.975643	8.001846	9.227022
GPR137C	0.050173	0.058093	0.028313	0.02938	0.050179	0.050515	0.050925	0.087235
GPR139	0.02643	0	0.0261	0.108338	0.026433	0	0	0.026806
GPR141	0.103091	0.278517	0.237548	0.228896	0.326497	0.41518	0.209274	0.888746
GPR143	0.414768	0.389085	0.075852	0.267619	0.153638	0.185601	0.42099	0.062322
GPR146	0.686078	0.375911	0.352592	0.832208	0.819198	0.683716	0.447666	0.397625
GPR15	0.149276	0.063014	0.245693	0.203964	0.236383	0.075148	0.10101	0.0757
GPR150	0.019303	0	0	0.019781	0	0	0	0
GPR151	0	0	0	0	0	0	0	0
GPR153	10.09312	7.508345	8.019256	9.553899	8.58094	8.1283	7.28225	9.024012
GPR155	10.73236	5.783811	5.029425	7.175062	7.618618	8.001619	6.895303	10.61235
GPR156	0.419243	0.602251	0.655015	0.666885	0.776013	0.548109	0.482693	0.558486
GPR157	3.705857	5.389152	4.911103	6.326903	4.317918	5.726572	4.292384	4.707605
GPR158	0.038606	0.03129	0.049562	0.047474	0.007722	0.027209	0.047022	0.058733
GPR160	4.547061	3.998075	5.188394	3.838815	5.2781	4.661148	4.842444	3.787404
GPR161	3.144129	2.626324	1.318021	2.367191	3.221544	2.248228	2.969854	2.941586
GPR162	0	0	0	0	0	0	0	0
GPR17	0	0	0	0	0	0	0	0
GPR171	0.689099	1.143766	0.854261	0.916517	1.099769	0.56095	1.116121	1.680356
GPR173	0.525374	0.511797	0.342084	0.520639	0.505233	0.340048	0.424846	0.532853
GPR174	0.267839	0.271354	0.252479	0.274473	0.487043	0.110319	0.284214	0.321043
GPR176	1.595193	1.581185	0.911129	1.678887	1.371181	1.814448	0.997728	0.787087
GPR179	0.038115	0.035397	0.03764	0.026039	0.02859	0.03198	0.029015	0.009664
GPR18	0.024233	0.061378	0.035897	0.211083	0.096945	0.097594	0.098386	0.061445
GPR180	4.912482	4.855564	5.647984	5.238807	5.288591	5.428582	4.297024	5.355082
GPR182	0.035295	0.071517	0.017428	0.018085	0.01765	0.088841	0.017912	0.035798
GPR183	0.586923	0.919351	0.497396	0.750761	0.807644	0.393955	0.625302	1.169448
GPR19	1.459398	1.394062	0.988261	1.260533	0.980008	1.374908	1.386063	1.607045
GPR20	0.104584	0.026489	0.11619	0.066984	0.196119	0.052649	0.106152	0.026518
GPR21	0.749362	0.591582	0.615086	0.553504	0.832192	0.921055	0.760603	0.503396

GPR22	0	0	0	0	0.0052	0	0	0.005274
GPR25	0	0.025161	0	0.02545	0.049677	0.05001	0.025208	0.100755
GPR27	2.998839	3.337412	2.602507	2.607497	2.510716	2.916394	2.202159	1.981603
GPR3	0.013918	0	0	0	0	0	0	0.014116
GPR31	0	0	0.029065	0	0	0	0	0
GPR32	0	0	0	0.029149	0	0	0	0
GPR33	0.10944	0.360346	0.108076	0.869166	0.766176	0.303015	0.194392	0.721484
GPR34	2.472097	2.943189	1.613737	2.075328	2.681943	1.518703	2.083894	3.343052
GPR35	0.152198	0.123356	0.150301	0.296339	0.197883	0.275827	0.123585	0.524839
GPR37	3.177604	3.39974	2.203087	2.622342	2.821777	2.519812	3.291867	1.90138
GPR37L1	0.088854	0.030007	0.014625	0	0.059244	0.029821	0.045094	0.075099
GPR4	0.263304	0.373463	0.442039	0.836459	0.750512	0.477184	0.507781	1.615663
GPR45	0	0	0	0	0	0	0	0
GPR52	0.009183	0.009303	0.027205	0.047051	0.036736	0.018491	0.009321	0.018627
GPR55	0.231489	0.127924	0.124694	0.15096	0.252566	0.063565	0.04272	0.149408
GPR61	0	0	0	0	0	0	0	0
GPR62	0.62227	0.525363	0.69133	0.86353	0.440831	0.587363	0.539495	0.657425
GPR63	3.590233	5.354577	2.561442	3.218643	3.199873	3.929515	3.549886	3.552167
GPR65	0.241009	0.293006	0.126936	0.279909	0.417803	0.113239	0.407707	0.374808
GPR68	1.150666	1.386317	0.598876	1.163233	1.065281	1.416847	1.025881	0.757003
GPR75	1.297511	1.48938	1.862051	2.344901	1.169829	1.505871	1.278048	1.192811
GPR82	0.10032	0.118576	0.123837	0.222743	0.15886	0.14309	0.093339	0.729192
GPR83	0.030876	0.046922	0.099097	0.047461	0.0386	0.038859	0.031339	0.093947
GPR84	0.073222	0.037092	0.054232	0	0.054924	0.018431	0.01858	0
GPR85	0.089518	0.191462	0.196449	0.254819	0.189006	0.140201	0.131243	0.110968
GPR87	35.43219	35.59614	32.14002	37.57142	33.34226	36.45782	31.94217	39.16571
GPR88	0.030297	0.07162	0.124664	0.119014	0.126253	0.137267	0.107629	0.128034
GPRASP1	0.245683	0.321374	0.089064	0.404741	0.152405	0.297459	0.107323	0.220793
GPRC5A	0.850169	0.699828	1.101943	1.592714	1.341847	0.829228	1.051686	0.741015
GPRC5B	13.28167	13.0899	11.27413	13.10026	12.79503	13.04793	10.84419	13.49055
GPRC5C	6.869429	6.045186	5.981659	5.939973	7.020762	7.683721	5.567798	5.87891
GPRC5D	450.543	439.8525	622.6578	560.9921	495.2096	601.9721	480.1655	435.3578
GPRIN1	0.538785	0.56685	0.484321	0.523816	0.573396	0.465964	0.616977	0.623519
GPRIN2	0.516172	0.352188	0.520143	0.766449	0.463561	0.54091	0.342148	0.438047
GPRIN3	0.086193	0.182587	0.108333	0.264984	0.109714	0.165674	0.230644	0.222523
GPS1	23.09655	21.92869	20.89297	22.09325	22.59923	22.62783	24.24796	20.36879
GPS2	26.99972	27.65783	26.9742	28.43666	29.83698	27.50115	27.4808	24.31269
GPSM1	6.765033	6.691243	5.087705	7.425937	7.880889	6.471112	6.455033	6.809942
GPSM2	18.77508	17.24706	15.62502	15.05223	14.82568	19.32928	17.63969	16.27053
GPSM3	5.203829	6.062942	4.863685	6.684957	7.97404	5.725893	5.564844	6.088445
GPT	1.093509	1.241431	1.110518	0.97754	1.4582	0.788644	1.117783	1.266391
GPT2	42.74298	45.46602	47.06031	50.00506	48.65901	49.72197	50.049	36.12968
GPX1	67.7839	76.8409	60.08138	70.23646	79.69035	68.58136	63.62145	90.76685
GPX2	111.364	99.72534	117.8243	96.86889	108.2801	100.2993	85.82854	132.4573
GPX3	120.2504	97.19656	111.421	89.63271	111.8646	78.37219	118.9717	135.1654
GPX4	122.0811	110.415	122.5596	122.1933	129.0542	128.1763	116.288	103.4806
GPX5	0	0	0.020414	0.021184	0.020674	0.062438	0	0.041931
GPX7	6.092568	7.95931	5.880939	6.149596	7.994665	6.595407	7.090627	7.131748
GPX8	12.83156	22.34854	17.66967	15.95217	18.48291	19.14721	18.36856	20.45463
GRAMD1A	6.171163	5.732295	4.425439	5.707046	6.507244	4.656547	5.979015	6.661477
GRAMD1B	0.543871	0.565446	0.384644	0.669298	0.570069	0.593019	0.532744	0.667235
GRAMD1C	3.33717	3.116098	2.498268	2.994323	3.206865	2.717393	2.996994	3.42367
GRAMD2A	1.565457	1.402118	1.770002	2.01498	1.452206	1.332495	1.696404	1.52638
GRAMD2B	19.53998	19.55586	22.38395	23.27852	20.9179	21.92471	20.83729	19.46694
GRAMD4	8.582009	8.227949	7.001579	9.20507	8.107391	8.691049	8.360073	9.283103
GRAP2	0.527942	1.228531	0.733168	0.896063	1.138522	0.398661	0.937757	1.581273
GRASP	3.617061	3.49003	4.039753	5.177553	5.426293	4.797889	4.137516	5.022423
GRB10	4.66824	2.906515	4.344781	4.290423	4.577596	3.516168	3.693891	5.382439

GRB14	0.40495	0.265465	0.482237	0.671292	0.39309	0.575599	0.519824	0.591912
GRB2	21.75829	24.09092	22.15558	26.25486	24.69776	24.00142	24.70128	24.04669
GRB7	7.304864	5.531074	5.505302	6.431885	5.604324	6.251553	5.590096	6.112304
GREB1	0.144969	0.153703	0.059929	0.065643	0.070808	0.013578	0.297709	0.612067
GREB1L	0.602832	0.591168	0.473203	0.637564	0.618369	0.350163	0.490284	0.603575
GREM1	11.30179	18.425	16.07336	17.42698	22.46056	8.721225	14.67574	11.07251
GRHL1	86.31137	79.49317	80.2873	87.59623	77.7218	83.35304	83.63551	83.44137
GRHL2	9.859864	9.088365	9.516504	8.907822	8.301848	9.02403	10.24235	10.2545
GRHL3	9.47479	9.847153	8.255414	10.14618	10.05636	8.416684	9.110759	11.03503
GRHPR	4.088305	4.317197	4.487685	4.785782	5.016684	4.401204	3.958108	4.034868
GRIA1	0.645968	0.658668	0.436254	0.367286	0.36679	0.835005	0.406085	0.515677
GRIA2	0	0.002053	0	0	0.004054	0	0.004114	0.002056
GRIA3	0.955654	0.94792	0.716457	1.184421	1.160945	0.906769	0.959832	1.334634
GRIA4	0.156386	0.071297	0.104245	0.192312	0.359734	0.125963	0.317463	0.32912
GRID1	0.307662	0.46755	0.327815	0.373362	0.28341	0.154882	0.312277	0.312042
GRID2	0	0.065133	0.011543	0.017968	0.052607	0.005884	0.011864	0
GRID2IP	0	0	0.022787	0.031528	0.076924	0.007744	0.02342	0.023403
GRIK1	0	0.085497	0	0	0	0	0	0.01902
GRIK2	0.005649	0.034337	0.022313	0.023154	0.028247	0.011375	0.022934	0.011458
GRIK3	0.619302	0.694059	0.238139	0.421223	0.498794	0.485583	0.378268	0.344631
GRIK4	0.306302	0.393074	0.342816	0.627778	0.319956	0.301541	0.324714	0.283048
GRIK5	0.514122	0.282506	0.172107	0.357191	0.549049	0.245657	0.327252	0.362357
GRIN1	3.222342	4.71154	3.153842	3.206553	3.287358	3.988607	2.644223	2.016248
GRIN2A	0.101417	0.014103	0.045167	0.038719	0.047732	0.100108	0.03835	0.038321
GRIN2B	0.00459	0.013952	0.009066	0.028224	0.018363	0.011554	0.025625	0.004656
GRIN2C	0.024667	0.020826	0.01218	0.025278	0.041117	0.020697	0.054248	0.016679
GRIN2D	0.370494	0.449291	0.321529	0.673054	0.516513	0.310855	0.495705	0.455477
GRIN3A	0.126505	0.054928	0.071388	0.120378	0.063261	0.072782	0.08713	0.064153
GRIN3B	0.361094	0.240914	0.147856	0.433215	0.317099	0.203949	0.107271	0.196516
GRINA	12.79035	13.10976	9.779773	15.20738	13.9141	12.69698	12.46595	11.88001
GRIP1	1.37606	1.523936	0.847258	1.3816	0.969495	1.239622	1.294917	1.955038
GRIP2	0.234607	0.237686	0.156252	0.206872	0.234637	0.340582	0.276891	0.381822
GRIPAP1	13.42091	12.27405	11.16649	14.0937	12.69036	13.67979	13.31576	11.65209
GRK1	0.083005	0.117732	0.131153	0.204146	0.033206	0.317572	0.11795	0.134698
GRK2	21.32177	18.46163	17.36099	20.3464	21.42872	17.90992	19.22718	22.18883
GRK3	3.49438	3.125368	2.476921	3.003931	3.331011	2.738327	3.816962	4.468228
GRK4	6.621691	7.024292	7.34147	5.998798	8.24201	6.448431	5.998084	5.982279
GRK5	3.648104	3.651772	2.805412	3.640088	3.954079	3.299573	3.277621	4.089515
GRK6	5.935882	5.372638	4.762315	5.417982	5.832497	5.5651	5.943612	5.346035
GRK7	0	0	0	0.017289	0	0	0.017124	0
GRM1	0	0	0	0.004466	0	0	0	0
GRM2	0.101594	0.025732	0.025082	0.078083	0.016935	0.04262	0.051559	0.034347
GRM3	0	0	0	0	0	0	0	0
GRM4	0.006255	0.012673	0.01853	0.025638	0.018766	0.018892	0.006348	0
GRM5	0.014431	0.010965	0.007126	0.011091	0.018041	0.007265	0	0.010977
GRM6	0.010699	0	0	0.010964	0	0.021545	0.032579	0
GRM7	0.286809	0.376035	0.316557	0.259335	0.523072	0.237809	0.428104	0.821341
GRM8	0.398283	0.11868	0.154243	0.104038	0.187452	0.149394	0.166459	0.182175
GRN	26.60438	25.08688	25.8209	29.82495	26.43176	24.32847	28.34944	28.10195
GRP	0.172347	0.034922	0.20424	0	0	0.034705	0.104959	0.174801
GRPEL1	41.1582	33.25388	33.08206	38.16175	40.44728	39.03593	33.63852	35.89307
GRPEL2	8.984956	9.454831	9.761862	9.191335	10.07582	9.698182	9.889057	9.673408
GRPR	0.382181	0.273316	0.111006	0.184305	0.179873	0.135809	0.228185	0.18241
GRSF1	12.66422	12.49649	14.36533	11.26869	12.26869	12.31492	11.64092	12.15173
GRTP1	17.754	13.64986	18.8633	24.00598	16.64788	18.09408	17.42478	19.06466
GRWD1	4.329318	3.743555	3.737922	3.832715	4.661371	3.97162	4.136749	4.170986
GRXCR2	0.066388	0	0.013112	0.027213	0	0	0	0
GSAP	1.512058	2.401067	2.112744	2.692415	2.145041	2.36456	2.427283	2.338443

GSC	0.024275	0.024594	0	0	0	0.048882	0	0
GSC2	0.308189	0.11354	0.249013	0.545512	0.308229	0.338503	0.341249	0.397824
GSDMA	211.2442	176.3849	239.327	207.3477	207.5874	223.7671	176.6333	160.9621
GSDMB	0.245923	0.093432	0.075894	0.126008	0.153722	0.092851	0.140407	0.109123
GSDMC	0.336618	0.227357	0.283176	0.229971	0.648385	0.025105	0.177161	0.177027
GSDMD	2.351506	2.108289	1.633764	2.089875	1.654592	2.325667	2.756412	1.910096
GSDME	3.658867	3.659664	2.474059	3.749498	3.379645	3.15592	3.607305	3.852773
GSG1	0	0.025896	0	0	0	0.051471	0.025944	0.05185
GSG1L	0	0.019225	0	0	0.151825	0	0.057781	0.019246
GSK3A	33.26364	31.37743	32.98004	31.92826	34.24875	31.83635	32.2157	33.4549
GSK3B	12.75526	12.4849	11.84095	11.80268	11.22398	12.0899	12.0339	12.38312
GSKIP	11.36665	14.35049	14.12242	14.99333	14.04011	12.129	12.72045	12.32659
GSN	572.056	612.3347	599.188	626.4859	581.4785	548.8893	696.3951	687.8604
GSPT1	28.16126	26.91806	28.45155	26.33769	29.30714	28.93253	26.09849	27.76876
GSPT2	5.56342	6.7521	6.649559	6.406534	6.493408	6.363683	5.623585	6.631531
GSR	9.434672	6.280666	7.258557	8.41115	8.252333	9.324228	8.066482	5.604846
GSS	15.82449	15.28005	14.39257	15.92284	13.83357	15.64411	15.348	12.80018
GSTA1	13.26822	13.3197	15.97214	51.55897	24.40893	20.06267	46.49639	41.47636
GSTA1-1	5.648264	10.75503	14.34313	6.563375	12.68502	40.26493	22.21533	7.774624
GSTCD	3.942461	3.743104	1.576688	1.577063	2.109835	1.816404	1.842841	1.812224
GSTK1	16.30355	15.60463	18.51963	24.15214	19.03735	20.951	20.40647	22.70437
GSTM3	128.9823	138.973	114.4466	139.8766	123.6133	82.88536	113.1451	129.4449
GSTO2	0.042026	0.191598	0.041502	0.172267	0.126094	0.042313	0.085312	0.191808
GSTP1	3.895399	6.029409	3.9181	6.505302	6.637464	4.757254	4.79585	5.340968
GSTZ1	3.59688	4.930233	5.64151	4.847523	4.519353	5.797366	6.151193	4.583097
GSX2	0	0	0	0	0.019226	0	0.019512	0
GTDC1	3.880178	4.205104	4.409603	3.557734	4.289172	4.223444	3.249304	3.857042
GTF2A1	7.419473	6.935789	7.547285	7.174115	6.742122	7.804712	7.794111	7.381978
GTF2A1L	0.039701	0	0	0.020342	0.019853	0.09993	0.020148	0
GTF2A2	25.11759	28.6369	31.15953	29.17882	25.53606	34.10208	29.81365	35.2652
GTF2B	16.20228	24.41388	16.85031	22.16372	19.23568	19.83552	18.78108	19.54494
GTF2E1	5.189528	5.249741	4.986355	4.647326	4.925233	5.805534	5.757726	5.105344
GTF2E2	19.76068	20.89473	24.26475	21.02659	20.83819	22.37044	20.72775	19.11271
GTF2F1	28.01701	30.30336	28.0117	28.95359	26.40513	30.35349	33.51132	30.15301
GTF2F2	10.03761	10.33577	11.95762	9.438581	9.434583	10.96264	10.03932	8.93695
GTF2H1	18.43707	19.7031	18.46687	20.65469	20.82526	20.85287	19.49336	20.1143
GTF2H3	8.698137	10.78804	11.69739	11.55928	8.765753	12.23827	8.806116	9.159106
GTF2H4	5.080225	4.56423	4.038782	5.779058	3.642896	3.409954	5.821251	5.103936
GTF2H5	58.24148	63.63834	69.05543	60.86741	60.88199	69.38138	58.31815	61.22542
GTF2I	96.30989	92.11211	92.8921	86.4111	88.73357	88.16378	83.0159	90.79194
GTF2IRD1	2.88944	3.575827	2.329705	3.457638	2.761784	3.139336	3.842315	3.22734
GTF3A	13.53261	13.47383	13.27182	15.18765	13.59036	12.9579	14.87234	14.06602
GTF3C1	11.15015	10.27496	8.39596	10.15988	10.38252	9.551464	9.557276	10.70803
GTF3C2	10.14898	10.5237	8.879014	9.911767	9.974309	9.275349	10.6008	10.47768
GTF3C3	8.167866	8.791838	7.426747	8.309873	8.201624	7.492819	8.735104	8.53618
GTF3C4	7.155888	5.270246	4.20161	5.148802	5.473355	4.394401	5.855225	7.111915
GTF3C5	9.415387	9.077392	8.029102	9.253567	8.902546	9.126866	9.402479	9.217679
GTF3C6	14.12123	15.39719	14.87079	12.78381	13.24907	12.59828	13.90884	12.81938
GTPBP1	10.58289	9.847251	9.742216	9.338327	8.715331	9.190775	8.873802	9.495674
GTPBP10	12.19911	16.88129	17.7641	14.66795	14.44951	16.6075	13.48917	15.65743
GTPBP2	12.01939	11.57743	14.38631	16.95918	13.90915	15.01866	11.80757	10.64219
GTPBP3	5.252465	6.378389	4.239618	6.009308	5.68491	5.348717	6.025045	5.947532
GTPBP4	31.23161	32.17984	28.91481	32.38307	31.8647	32.3512	33.26308	31.28026
GTPBP6	13.28826	17.33159	13.99651	18.53304	17.59058	16.19238	16.51283	13.98125
GTPBP8	2.420288	2.619237	2.272435	2.273553	3.03492	2.649121	2.484508	2.928954
GTSE1	3.233134	2.942296	2.635956	3.043539	3.139553	2.95241	2.80465	3.860638
GTSF1	0.030308	0.023029	0.037413	0.038823	0	0.045772	0.061525	0.01537
GUCA1A	0.643429	0.242363	0.236243	0.371951	0.321756	0.323912	0.477251	0.418326

GUCA1B	0.041312	0	0.020399	0	0.082636	0	0.083864	0
GUCA2A	0.246239	0.415784	0	0	0.246271	0.165281	0.083311	0.083248
GUCD1	11.1348	11.93708	9.632149	9.595472	10.34024	10.40953	9.274308	11.20626
GUCY1A1	2.929405	2.630671	2.48614	2.897287	4.590764	2.982594	2.354596	4.835986
GUCY1A2	0.016958	0.085903	0.05024	0.008689	0.084801	0.051222	0.025819	0.120396
GUCY1B1	11.23807	11.67199	9.876562	12.114	14.38518	13.79664	11.6667	9.722397
GUCY2D	0.05873	0.076501	0.024856	0.077381	0.142649	0.118263	0.085159	0.076585
GUCY2F	0	0	0	0	0	0	0	0
GUF1	6.253618	7.751232	6.565155	8.821821	9.161889	7.816537	6.347422	8.205436
GUK1	33.39915	36.61604	34.28153	36.86371	31.26174	34.93232	29.74365	31.41689
GULP1	4.151357	3.888271	3.661732	4.066157	4.297171	4.541497	3.546275	3.404032
GUSB	3.594568	4.427378	3.685389	4.279606	4.322118	3.488999	4.76353	5.292466
GVQW3	0.616904	0.231482	0.157945	0.280971	0.388471	0.345066	0.23191	0.254909
GXYLT1	6.393924	6.437949	5.859348	6.257772	6.426251	6.738871	6.369946	6.476959
GXYLT2	7.511155	6.025755	6.650975	6.027799	7.35904	5.28382	7.313099	8.660438
GYG1	22.68853	26.35431	25.23493	24.86756	24.85183	24.9104	25.65673	21.84618
GYG2	6.374926	6.555389	5.273328	4.008909	6.232415	5.216015	6.578303	8.683712
GYPC	13.77858	16.67758	16.0768	16.28679	17.2823	13.52933	13.06214	16.92649
GYS1	11.14592	10.34725	10.3853	12.4973	13.06744	11.31597	11.05277	11.14693
GZF1	7.041986	6.173424	5.017059	6.056092	6.287946	5.645075	5.705995	5.631183
GZMA	0.103376	0.130916	0.229699	0	0.129237	0.078062	0.026232	0.052424
GZMK	0.027043	0.027398	0.026706	0.166275	0.189323	0.081682	0.054897	0.109711
GZMM	0.18043	0.411295	0.300681	0.358242	0.372184	0.283847	0.28615	0.377434
H2AFZ	136.0819	152.2106	145.7375	138.0884	137.6258	169.1803	148.3728	145.824
H3F3A	753.3062	811.4823	911.1647	804.1674	770.0598	924.2851	744.688	676.8507
H6PD	9.835439	8.576504	9.619058	10.43084	9.787665	9.486035	9.214571	8.934076
HAAO	0.375101	0.59239	0.239688	0.553977	0.297913	0.388771	0.167968	0.458767
HABP4	6.585686	6.748933	6.599898	7.703418	7.095694	7.775776	6.167744	6.415775
HACD1	1.963168	3.040225	2.437231	2.557855	3.225621	2.654264	2.305741	2.104893
HACD2	28.08716	29.40277	33.00026	36.61591	36.88895	30.72291	27.46025	28.96102
HACD3	35.47419	42.03571	40.85974	36.74879	34.20374	39.2172	37.89828	37.83055
HACD4	2.741503	3.460946	2.919962	2.662321	3.4022	3.164873	2.811763	4.207189
HACE1	9.973573	8.1178	7.528405	9.114266	9.79123	8.540628	9.936814	9.266383
HACL1	12.62281	12.87772	12.76381	12.20037	12.51116	10.37757	12.07127	12.67486
HADH	98.41987	105.5769	132.2901	128.7463	142.2462	129.5843	101.1258	88.42018
HADHA	99.64761	124.9461	119.7344	118.3535	114.9405	98.88561	110.5178	92.90015
HADHB	37.73046	45.69091	47.2455	43.49309	44.45042	43.37337	41.17833	41.59745
HAGH	19.57482	17.24756	20.90403	22.2229	21.75696	25.15964	19.60062	18.71362
HAGHL	0.451532	0.621673	0.388738	0.49831	0.770019	0.303077	0.364293	0.457959
HAL	0.37456	1.004494	1.066161	1.166566	0.683109	0.842971	0.924355	0.856619
HAMP	0	0.227763	0.222011	0	0.449683	0	0.684554	0.114006
HAND1	0	0	0	0	0.016033	0	0	0
HAND2	1.119567	1.925604	2.082673	2.294597	2.473781	2.752502	1.876315	1.53161
HAPLN1	0.363113	1.053923	0.629953	1.106266	0.863732	1.383328	0.896497	1.154615
HAPLN2	0.013815	0.013996	0.040929	0.0991	0.013817	0.013909	0	0
HAPLN3	0.976053	0.913846	0.711285	1.165786	0.834802	1.111491	1.195665	1.037738
HAPLN4	0.007856	0.007959	0.015515	0	0	0.007909	0.007973	0
HARBI1	6.670939	7.74779	6.526914	7.078449	7.083007	7.347802	6.47888	5.775518
HARS	20.31222	21.00692	21.67276	21.86912	21.68138	20.56444	21.87507	19.45844
HAS1	0.171067	0.133317	0.12995	0.229244	0.315857	0.079493	0.240415	0.120117
HAS2	1.410203	1.741666	1.821473	1.812152	2.287958	1.866071	1.83577	1.943362
HAS3	7.150705	5.628106	4.988416	7.486699	6.770468	6.627233	7.723472	6.15185
HASPIN	2.503944	2.373451	2.013788	2.391015	1.565167	2.368259	2.166054	2.318337
HAT1	29.73958	36.46088	34.24369	31.21169	28.88561	34.66591	32.54865	30.10968
HAUS1	4.313115	4.444416	3.503971	5.279387	4.129327	4.648787	5.26647	5.066211
HAUS2	16.19952	14.89248	14.5164	14.75625	17.56675	15.79672	14.11315	15.01531
HAUS3	3.731142	4.029846	2.279394	3.306866	4.202279	3.373071	3.650636	4.147909
HAUS4	11.53322	10.59212	10.26291	12.18725	10.1046	11.79296	12.4676	11.72118

HAUS5	12.41918	8.696389	7.451716	12.37978	10.32979	12.08154	11.82089	12.9693
HAUS6	4.467853	4.355409	3.342142	3.821445	4.595095	4.399199	4.327767	4.981033
HAUS7	12.88848	14.32441	13.48776	13.77941	13.98677	13.86746	11.85173	13.11098
HAUS8	12.98743	12.96352	11.46158	12.28687	12.7205	11.74343	11.85818	12.86101
HAVCR1	0	0.034178	0.888402	0.011524	0	0	0.639174	0.02281
HAVCR2	0.474622	0.810578	0.428534	0.680931	0.562839	0.491518	0.523034	0.728945
HBB	1.405848	0.453186	2.902875	2.488433	1.789493	0.321694	2.789012	14.90673
HBEGF	3.821953	3.006443	2.634049	3.088335	3.348972	2.836638	3.269848	2.787232
HBP1	50.24777	52.98833	53.32509	52.37762	46.60898	53.71983	55.07519	50.48229
HCAR1	0.223832	0.670001	0.261233	0.344064	0.335791	0.338042	0.361438	0.454037
HCAR2	8.154565	9.638516	11.84733	13.404	9.320707	10.46347	10.47575	10.88243
HCFC1	9.977314	9.034652	7.458478	8.883895	9.307974	7.631305	9.2428	10.87837
HCFC2	5.92613	5.18036	4.345195	4.535882	5.250286	4.371997	4.684265	5.893474
HCK	0.783236	0.599026	0.87964	1.046576	1.02141	0.680349	0.86513	1.619924
HCLS1	3.676601	3.934901	3.780936	5.198254	4.520316	2.491006	3.647576	5.719577
HCN1	0.017464	0.011796	0	0	0.005822	0.029306	0.017726	0.023617
HCN2	1.082315	0.501509	0.621412	0.945765	0.872677	0.557525	0.936748	0.859457
HCN3	0.210827	0.206229	0.136407	0.20115	0.159959	0.219587	0.206611	0.117974
HCN4	0.028315	0.019124	0.041943	0.04836	0.202949	0.009503	0.02874	0.076581
HCRTR1	0.057909	0.224896	0.114374	0.098905	0.096527	0.048587	0.029389	0.127255
HCRTR2	0.038632	0	0.019076	0	0.019319	0	0	0
HCST	0.431179	0.759719	0.33324	0.88372	0.843721	0.41525	0.742097	0.513373
HDAC1	69.80414	71.78721	72.02775	72.903	71.56862	73.40763	78.07336	73.11213
HDAC10	9.595022	9.743345	8.798647	9.889334	9.242483	9.159734	7.59593	7.534152
HDAC11	7.57367	7.436104	9.272133	11.56201	9.368058	7.255353	8.908195	7.207041
HDAC2	44.13518	44.75593	41.5618	44.22055	42.37853	46.84351	44.06238	39.83132
HDAC3	34.286	34.98471	34.91421	33.37408	31.96493	33.70594	37.42424	33.99775
HDAC4	4.85923	3.977786	3.455052	3.76059	4.385536	3.420784	3.993043	4.045234
HDAC5	31.95827	25.83129	25.70526	26.27045	26.38273	26.17388	26.45384	26.38888
HDAC6	7.154875	7.304794	7.327822	8.403022	7.028611	6.524575	7.643829	8.069887
HDAC7	8.499431	7.839144	7.065479	9.850475	10.49481	8.370738	7.658664	9.587957
HDAC8	3.905465	3.95672	3.891864	4.402425	4.438602	4.504094	4.324415	4.213129
HDAC9	1.797945	2.119293	1.331657	1.807048	3.648226	2.349815	2.228501	2.82298
HDC	0.328426	0.739413	0.720741	0.772843	0.425792	0.269434	0.580279	1.554471
HDDC2	12.76646	15.36105	12.18814	13.45559	11.73695	14.22758	12.25004	12.64064
HDDC3	25.15949	25.20923	26.30396	23.95459	22.3327	24.92879	26.75441	21.27511
HDGF	86.13377	90.09219	89.8074	84.64475	85.67063	88.08944	87.53517	85.32679
HDGFL1	0	0	0.038712	0	0.039206	0	0	0
HDGFL2	14.34027	14.73378	13.40819	15.44065	13.22146	15.18236	15.8621	14.55651
HDGFL3	19.29099	20.72205	21.53117	19.69529	21.40371	20.50664	19.97371	18.96875
HDHD2	7.292429	8.413315	8.69384	8.475465	8.595518	8.612368	7.771564	6.684669
HDHD3	2.557035	2.077142	2.229433	2.124627	2.096579	2.644084	2.080989	1.448586
HDHD5	5.721836	5.402092	5.423119	5.228196	5.208784	5.796596	4.441154	5.156483
HDLBP	58.0449	49.20185	51.19493	50.75574	55.53775	53.94628	56.11444	57.54375
HDX	0.157741	0.129847	0.068152	0.282884	0.088741	0.12904	0.140094	0.23998
HEATR1	10.64318	10.40046	8.515771	9.508399	9.682151	9.48394	10.65969	10.34877
HEATR3	10.32912	9.608399	9.567836	9.53651	10.36605	10.56092	9.779704	9.682105
HEATR4	0.025669	0.249654	0.025349	0.105218	0.08215	0.108545	0.125058	0.109343
HEATR5A	4.542215	3.895884	5.148011	4.567973	5.614188	5.071549	4.150966	4.468175
HEATR5B	4.154673	4.180895	3.089988	3.946753	3.987564	3.371358	3.969888	4.30287
HEATR6	18.40538	15.73692	13.59719	14.27614	14.34224	14.27493	14.86351	15.92598
HEATR9	0	0	0	0	0	0	0.01252	0
HEBP1	27.79171	35.95823	27.4698	31.61647	34.50962	28.60283	33.49456	32.14258
HEBP2	80.92683	78.83182	94.39146	85.56355	84.62056	90.20762	99.43151	69.74311
HECA	18.50194	17.38933	18.10888	18.5522	17.10788	19.63325	18.45076	18.15148
HECTD1	42.43419	38.53382	36.76858	38.36659	39.89404	38.99341	37.56865	41.38921
HECTD2	2.272902	2.685523	1.766515	2.008559	2.651078	2.127942	1.893534	3.071682
HECTD3	8.45026	8.034089	7.288086	8.537718	8.399321	7.490322	8.607188	8.178586

HECTD4	5.185135	4.205131	3.339943	3.844734	4.581068	3.779265	3.854293	4.736194
HECW1	0.062184	0.029842	0.087265	0.238126	0.153842	0.098855	0.063116	0.102902
HECW2	1.639171	1.689165	1.561087	1.659829	1.764824	1.680851	1.573766	2.112128
HEG1	13.11395	11.55827	10.12814	11.44707	14.00374	9.941138	12.75256	14.12969
HELB	2.509281	2.224436	1.83874	1.716571	2.255975	2.136709	2.065986	2.193034
HELLS	5.999758	7.037486	5.256281	6.499071	5.046566	6.619895	7.146593	6.38795
HELQ	1.839554	1.635649	1.234084	1.216977	1.56033	1.219118	1.378695	1.952337
HELT	0	0	0.151662	0	0	0	0.03897	0.077881
HELZ	4.050683	3.651814	3.014947	3.501166	3.91899	3.233984	3.411177	3.892559
HEMGN	0	0.069467	0.013543	0.028106	0	0.055229	0	0.041726
HEMK1	3.535225	3.830278	2.908536	3.564752	3.488477	2.642207	3.147507	3.748313
HENMT1	0	0.013471	0.013131	0	0.053193	0.026775	0	0.107886
HEPACAM	0.303157	0.10714	0.055699	0.238418	0.218583	0.141967	0.100183	0.150161
HEPACAM	0	0	0	0.024909	0	0.024473	0.037007	0.073958
HEPH	0.319946	0.224408	0.249122	0.176546	0.344602	0.105312	0.231069	0.524194
HEPHL1	65.5417	60.69628	84.17835	57.38144	68.24953	57.1557	57.23506	63.80266
HERC1	6.619384	5.798781	4.622269	5.409033	6.05227	5.187656	5.441488	6.644766
HERC2	9.581451	8.235853	7.547422	7.731504	8.157451	7.45177	8.033678	9.091841
HERC3	10.24993	10.29608	10.01736	11.19941	8.472548	10.46885	10.39598	9.588173
HERC4	20.94271	22.55902	23.08978	22.684	24.50516	28.50375	22.09515	21.55331
HERC5	1.529139	2.148349	1.32654	1.636277	1.833515	2.152018	2.401002	1.739416
HERC6	1.034823	0.998817	0.711207	0.73802	1.013978	0.929255	1.014861	1.077921
HERPUD1	42.77645	49.11723	50.53996	52.56681	53.70346	53.40695	51.52421	44.58763
HERPUD2	15.37155	15.25092	15.13668	16.07837	15.593	16.15041	14.86712	15.03397
HES1	34.00998	36.0778	35.58068	31.72839	30.26041	35.39332	35.97055	35.5763
HES2	25.17385	23.73195	23.96942	23.08042	22.32042	24.91927	20.39123	23.63324
HES4	22.26852	20.69435	17.13961	23.01884	19.84708	21.02139	20.50303	21.1432
HES5	0.053211	0.05391	0.157645	0.545294	0.159655	0.214299	0.05401	0.539688
HES6	0.150121	0.050697	0.131778	0.068373	0.283598	0.419851	0.203163	0.507526
HES7	0.344129	0.377699	0.198241	0.470204	0.659666	0.77958	0.407506	0.494456
HESX1	0.36008	0.364806	0.504015	0.362582	0.20355	0.416133	0.219289	0.114325
HEXA	12.27551	13.13834	12.60878	13.9222	14.3052	12.19973	13.90804	13.92296
HEXB	28.89253	33.80204	41.31937	31.64483	32.51018	37.57888	38.37278	37.74834
HEXD	2.247717	2.231443	1.907389	2.60434	2.33838	2.80894	2.487794	2.48592
HEXIM1	22.59584	19.86696	21.17937	20.66102	23.4676	21.61133	21.04268	22.8808
HEXIM2	0.233784	0.298259	0.277901	0.403728	0.294433	0.335636	0.237291	0.276631
HEY1	9.500336	8.663782	8.654581	10.15784	10.47544	10.00516	10.78326	9.281051
HEY2	1.157447	1.353043	0.967175	1.128055	1.432829	1.173503	0.985853	1.288853
HEYL	24.25415	19.83055	22.60156	21.26762	21.27983	20.80092	20.54141	17.95029
HFE	6.917067	7.576783	7.649529	7.508595	6.712919	5.671973	7.201773	7.738787
HFM1	0.023203	0.158679	0.045829	0.017834	0.023206	0.128491	0.035327	0.088252
HGD	0	0	0	0	0.017027	0.017141	0	0
HGF	0.437185	0.292141	0.261799	0.424184	0.995423	0.440172	0.599525	0.334915
HGFAC	0.151442	0.334754	0.081575	0.380928	0.151461	0.332675	0.223583	0.076799
HGH1	7.450272	5.290476	6.412754	7.185033	8.284153	6.504035	6.750993	7.373701
HGS	20.94516	19.73408	16.24659	19.98965	19.47162	18.10655	19.3534	20.6843
HGSNAT	9.165749	8.685956	8.654979	9.084153	8.072524	8.500608	8.70204	9.703512
HHAT	1.406491	1.107076	1.2287	1.230674	1.320108	1.078414	1.778994	1.645977
HHATL	0.092577	0.281376	0.121898	0.585034	0.169747	0.590328	0.454168	0.250387
HHEX	1.980968	2.848598	2.066721	2.32472	3.994404	2.927413	3.615986	3.078565
HHIP	0.487192	0.490501	0.544268	0.380685	0.371532	0.159419	0.571765	0.799868
HHIPL1	0.858257	0.928614	0.592471	0.819743	1.575063	0.771835	0.701979	0.667645
HHIPL2	1.149088	0.804507	0.507417	0.74674	0.214898	0.432676	0.843926	0.54956
HHLA1	0.018116	0	0	0	0	0	0	0
HHLA2	0.355794	0.186907	0.208214	0.121536	0.342661	0.092873	0.187253	0.133652
HIBADH	25.12588	27.70436	24.90048	22.42737	29.6133	25.29753	26.96715	28.43263
HIBCH	16.14959	17.72938	13.9826	13.16165	14.10892	21.73217	18.60552	17.67862
HIC1	3.079993	2.094824	3.020345	2.891422	2.832667	2.168555	2.404764	2.861698

HIC2	0.354114	0.530967	0.139881	0.50804	0.35416	0.456362	0.287541	0.330423
HID1	5.34429	5.246539	4.925853	7.115425	6.239954	5.864654	6.374785	5.19347
HIF1A	35.43716	36.86838	27.22797	36.32922	35.1772	34.48084	32.77527	34.38847
HIF1AN	11.85039	12.7501	11.25408	11.91767	11.65234	11.16613	12.8599	13.77178
HIF3A	0.521222	1.729054	0.450956	0.505772	0.544355	0.371528	0.575858	1.534465
HIGD1A	12.84378	7.495108	4.82997	20.6379	5.960284	16.26268	7.613279	7.920184
HIGD1B	1.093972	0.480973	0.693049	1.290288	0.289011	0.789715	0.523765	0.628044
HIGD2A	64.93664	70.03331	74.03054	64.12449	69.40249	72.38048	68.85112	70.11016
HIKESHI	32.73482	28.1497	35.32531	35.79425	34.65287	39.71455	32.18996	33.87364
HILPDA	5.822174	5.790353	4.641902	6.130603	4.487393	4.89392	4.933624	7.205252
HINFP	16.25213	11.96879	17.64052	18.10342	15.82677	16.18661	13.30351	13.32684
HINT1	30.82986	37.11738	66.46076	88.87283	57.78212	66.07667	103.3748	49.2758
HINT2	10.62239	10.90415	14.15322	15.43554	13.18134	14.4863	12.09379	9.719051
HINT3	5.381947	6.030183	7.551944	7.501688	5.299014	6.214683	5.069178	5.265817
HIP1	8.267986	8.179098	7.127613	9.193321	9.548386	7.586696	7.94489	10.20287
HIP1R	22.37732	17.81154	18.40597	20.58166	20.01032	19.68265	20.09449	20.02767
HIPK1	14.0049	12.46288	12.03465	12.10782	13.63532	11.72971	12.71031	13.32547
HIPK2	4.32187	3.414598	3.427375	3.513625	3.306995	3.395226	3.561532	3.464564
HIPK3	32.39104	30.1427	32.86752	27.67038	33.65693	28.74748	28.29936	32.50782
HIPK4	0.410969	0.175311	0.331087	0.454398	0.129797	0.130666	0.19759	0.142596
HIRIP3	7.011642	8.732105	6.001039	6.412166	6.446624	6.787069	6.617391	6.063453
HIVEP1	2.950499	2.472421	2.31267	2.521633	2.904501	2.357848	2.39757	2.724999
HIVEP2	5.398368	4.965554	4.671492	4.553814	5.871865	4.299065	5.055236	5.960872
HIVEP3	0.474588	0.651203	0.4755	0.441487	0.645154	0.4268	0.530813	0.630888
HJURP	5.331269	5.484333	5.224342	5.505352	4.122014	4.252862	5.869113	5.438359
HJV	0	0	0	0.012885	0	0	0	0
HK1	31.68541	27.39872	26.10994	33.61375	32.96049	34.32825	32.34159	31.75923
HK2	2.161508	1.662387	2.04109	2.538414	2.69829	2.388078	2.306029	1.610871
HK3	0.836593	0.513004	0.587015	0.857315	0.469727	0.495041	0.491608	1.964952
HKDC1	1.309806	0.830983	1.174184	2.182786	2.117582	1.734866	1.677951	1.676687
HLCS	7.236506	5.698996	6.741365	8.03126	5.141193	6.551926	6.588265	5.331342
HLF	7.596158	9.867293	7.812775	8.91482	8.637413	7.870194	10.1415	11.47652
HLTF	9.326298	11.58714	9.707111	9.752038	9.173415	9.870887	9.502681	10.15703
HLX	1.682285	1.589977	1.471777	1.839658	2.292269	2.250792	1.776279	1.454307
HM13	26.53597	30.68758	26.23522	31.61431	30.20314	27.31164	33.04909	28.75608
HMBOX1	5.567108	5.232536	4.110663	3.920787	5.267852	3.844787	4.588804	6.596075
HMBS	11.06507	10.28057	10.02096	11.35724	11.5254	10.71423	11.14149	10.22025
HMCES	12.46703	11.50638	9.416412	11.09112	10.39967	8.993443	10.5543	11.22721
HMCN1	1.500775	1.406584	0.837632	1.410785	1.35204	1.081825	1.529214	1.602096
HMCN2	0.115855	0.167432	0.228823	0.21126	0.207884	0.102923	0.22308	0.205632
HMG20A	15.73848	16.16496	14.63484	14.12619	14.63325	14.11204	14.93896	15.63227
HMG20B	29.2775	33.96906	32.77727	30.18425	32.00326	30.87194	31.00798	29.13895
HMGA1	118.4539	114.2395	119.1407	120.8943	121.4919	117.1169	113.7101	100.682
HMGB1	213.0578	248.5501	248.6393	236.0978	205.4834	241.0764	238.5887	229.1027
HMGB2	96.44093	112.9755	103.8596	106.0244	87.46574	103.2024	105.1323	100.2586
HMGB3	21.30197	24.8051	23.75265	23.08229	18.73377	25.13972	27.58793	21.53228
HMGCL	13.64515	15.20489	15.25226	16.04213	16.28544	15.84926	14.86064	16.3025
HMGCLL1	0	0	0	0	0	0	0	0
HMGCR	64.30598	68.71138	83.7173	103.2643	90.98564	98.52757	58.48514	40.21475
HMGCS1	127.5682	155.5839	186.3692	236.5268	202.9032	207.7143	157.9399	111.9351
HMGCS2	0.041404	0.013982	0.204441	0.04243	0.124228	0.250122	0.042025	0.041994
HMGN1	95.47635	111.1772	108.3096	94.85	92.20436	97.24601	94.97958	98.80923
HMGN2	129.1168	159.8589	145.8858	133.4292	126.8949	146.2031	167.4249	166.8231
HMGN3	29.37678	38.40299	37.85099	31.95997	30.95453	36.95479	34.43776	37.84442
HMGN4	7.511162	5.79975	6.981244	6.575116	7.012605	4.699952	7.955301	6.994613
HMGN5	3.64816	6.907351	8.091318	3.983676	4.844905	3.974155	6.252409	5.034554
HMGXB3	9.466152	8.442091	7.467161	8.501496	9.126256	8.838726	8.479027	8.110745
HMGXB4	6.925691	7.313338	6.312299	7.057221	6.327671	6.219343	6.950295	6.3443

HMMR	3.746046	4.191081	4.018136	4.52652	3.848477	5.140032	4.578203	4.161218
HMOX1	10.50453	9.039939	10.62832	9.408131	10.31675	9.555016	11.63931	17.73354
HMOX2	33.88162	31.09521	33.29448	30.79902	31.57732	33.14978	32.72507	30.60563
HMX1	0.026887	0	0	0	0.02689	0.054141	0.02729	0
HMX2	0	0	0	0	0	0	0	0
HMX3	0	0	0	0	0	0	0	0
HNF1A	0	0	0	0.009164	0.017887	0	0	0
HNF1B	0	0	0	0	0	0.021316	0	0
HNF4A	1.635757	0.191846	0.071618	0.140378	0.149091	0.146033	0.06952	0.130762
HNF4G	0	0.013759	0.006706	0	0.006791	0	0.013784	0.006887
HNMT	3.53067	6.002233	4.237775	3.618126	4.540019	6.440537	5.420139	4.985696
HNRNPA0	34.76836	37.25012	32.48646	35.93689	33.94579	34.46606	37.1623	36.4707
HNRNPA1	196.9344	247.2505	208.9427	221.1214	208.9042	202.6953	247.1281	245.8936
HNRNPA2	222.7576	242.5744	237.1104	223.5121	228.0633	237.9142	222.0101	232.6561
HNRNPA3	122.0456	137.2324	128.3949	128.405	122.3542	125.2906	127.0382	131.1294
HNRNPAB	159.5008	176.7692	162.2178	163.1155	164.6763	173.6427	171.4528	161.9614
HNRNPC	83.2098	88.94468	91.29631	89.91102	94.97095	88.69557	83.8464	90.26445
HNRNPD	142.6545	167.4568	157.7574	165.7436	137.7141	156.633	157.8105	134.4415
HNRNPDL	67.06484	96.19421	76.16398	103.0682	76.90683	90.61844	84.44154	69.08533
HNRNPF	95.66753	96.11487	90.90079	99.22393	95.91153	94.79245	101.2381	92.5055
HNRNPH1	124.2005	126.1455	109.3189	122.513	118.2789	119.3623	117.7159	124.7523
HNRNPH2	40.77884	42.23105	40.3636	42.52377	42.47708	42.45401	43.43081	40.42939
HNRNPH3	34.04895	37.56272	33.90379	33.81189	36.12428	33.76173	30.21835	37.08579
HNRNPK	162.1728	173.8304	162.6411	164.6738	161.317	161.3499	173.522	168.1491
HNRNPL	144.7065	144.9194	135.6278	139.537	131.7142	133.8414	158.355	155.7036
HNRNPLL	10.49204	11.64134	10.88515	11.66324	11.14874	11.41981	11.81333	11.85191
HNRNPM	116.2341	119.4134	111.8269	106.3125	104.4072	109.5695	118.0825	119.775
HNRNPR	48.28707	49.83642	44.24288	46.59129	48.13652	52.10816	48.99266	47.9643
HNRNPU	137.0504	138.6384	134.5436	129.0711	131.529	128.06	138.1868	145.59
HNRNPUL1	64.08422	65.24271	61.77892	62.53741	62.03511	60.57169	59.29316	61.30729
HNRNPUL2	63.05406	60.47266	58.3419	57.53678	60.01622	59.26375	60.61758	60.89016
HOGA1	2.827188	2.414189	2.423023	1.96597	3.130506	2.948163	2.254683	1.618053
HOMER1	2.282649	1.937814	1.355771	1.460239	1.649859	0.951853	1.440754	1.762066
HOMER2	25.18886	14.97178	21.19235	20.17291	21.26841	20.76889	19.41774	19.96553
HOMER3	7.114701	5.738053	7.143729	7.642911	8.293379	8.490105	7.363716	9.583395
HOMEZ	12.7946	12.7951	10.53708	12.59545	12.04075	12.26405	11.39715	10.59049
HOOK1	4.68193	4.71373	3.727772	4.627978	4.57035	4.753198	4.380897	4.644705
HOOK2	5.984838	5.977682	5.513464	7.585087	5.700079	6.408986	5.945821	6.885094
HOOK3	17.9496	16.08084	16.14575	18.1474	18.00916	16.16344	15.52486	19.03955
HOPX	259.0058	276.3863	288.7062	267.8298	242.9222	275.4347	224.9763	288.697
HORMAD1	0.032006	0	0.015804	0.016399	0	0.016112	0	0
HORMAD2	0	0	0	0	0	0.091793	0	0
HOXA1	1.176407	1.486857	1.161749	1.647183	1.397892	1.630076	1.028541	1.582996
HOXA10	0.008695	0	0	0.026731	0	0	0.008825	0.008819
HOXA11	0	0	0	0	0	0	0	0
HOXA13	0.012049	0	0	0.061736	0.01205	0.006066	0.006115	0.00611
HOXA2	0.349301	0.159819	0.189165	0.392594	0.552193	0.374377	0.148678	0.217135
HOXA3	1.510561	1.79654	0.843157	1.020768	0.974328	1.432714	1.288793	1.210109
HOXA4	1.915608	2.063971	2.207029	2.461613	1.626957	2.00523	1.43511	1.958298
HOXA5	0.079177	0	0	0.040569	0.019797	0.079718	0	0.020076
HOXA6	0.437331	0.671752	0.473676	0.607189	0.952375	0.497134	0.508326	0.586639
HOXA7	1.276762	1.706344	1.225085	1.326947	2.019538	1.349303	1.36025	1.451065
HOXA9	0.061112	0.071439	0.134627	0.048173	0.065821	0.137257	0.062028	0.038143
HOXB2	4.205173	4.378432	3.251249	3.851532	3.214993	2.835638	3.528946	2.649641
HOXB3	2.068628	1.762358	1.501187	2.408942	2.256977	1.514735	1.845154	2.352389
HOXB5	0.345267	0.296568	0.392851	0.507654	0.585529	0.551667	0.23617	0.45676
HOXB6	3.52167	3.08358	2.464337	3.56396	3.82916	3.417676	2.62189	3.296023
HOXB7	9.077567	9.921666	8.701986	10.45475	9.140083	9.366012	7.906377	6.697734

HOXB8	1.744513	1.585468	1.165407	1.840305	1.642106	1.291493	1.015537	0.702535
HOXB9	0.808746	0.45397	0.140307	0.503987	0.284191	0.407135	0.343881	0.642906
HOXC10	0.39362	0.18515	0.333184	0.115248	0.323372	0.184	0.171225	0.114064
HOXC11	0.249458	0.063183	0.14781	0.178946	0.149694	0.20093	0.10128	0.075903
HOXC12	0.233243	0.033758	0.032905	0	0.299923	0.033548	0	0.202769
HOXC13	143.4078	139.4897	152.7834	129.6854	133.1248	147.5282	126.6237	128.6624
HOXC4	1.870471	1.788698	1.767914	1.878847	1.728712	1.783804	1.610303	2.135019
HOXC5	2.633973	2.111516	2.5759	2.607497	2.53201	2.355874	2.180316	3.20317
HOXC6	22.18009	21.95341	21.70185	21.24562	19.21842	19.3129	18.43213	20.30155
HOXC8	9.141415	10.99139	10.42221	11.92033	9.386571	11.85385	9.017903	8.946003
HOXC9	2.727989	3.979859	4.238556	4.52881	4.238024	3.863586	2.602774	3.209515
HOXD1	0	0	0	0	0.032139	0	0	0
HOXD10	0.640973	1.128694	0.979622	1.032193	1.236322	1.229242	0.898431	0.882276
HOXD11	0.2054	0.263588	0.135227	0.603398	0.451938	0.344672	0.416962	0.55553
HOXD13	0	0	0	0	0	0.023621	0	0.023795
HOXD3	0.7742	0.530377	0.356791	0.559142	0.538323	0.527083	0.710973	0.553394
HOXD8	1.993915	2.711705	2.476357	2.763657	3.481353	3.382189	2.558937	2.844931
HOXD9	3.968242	4.14635	3.992504	4.869645	5.984235	5.197713	4.090896	5.034069
HP1BP3	40.55142	44.88202	43.50821	45.0071	39.07952	42.08672	46.10182	43.85305
HPCA	0.549166	0.519282	0.632711	1.08802	0.915395	0.774085	1.003326	0.686947
HPCAL1	8.498961	7.872863	8.766395	6.413738	9.942409	8.120008	9.82022	13.03643
HPCAL4	0.617366	0.76576	1.618198	1.460435	0.836725	0.586728	0.66762	1.246457
HPD	0	0	0.014781	0	0	0	0	0
HPDL	0.079215	0.133757	0.143417	0.338237	0.343308	0.079756	0.187607	0.147294
HPGD	5.779772	7.019303	8.275791	5.593363	4.245786	5.532457	5.642628	6.216197
HPGDS	0.882466	1.062736	0.937242	1.092015	0.749361	0.854967	1.504105	2.178466
HPN	1.876212	3.53215	1.659255	2.955772	3.164767	2.481113	1.762239	4.033057
HPRT1	48.85208	58.78408	60.07309	58.14807	53.3487	53.61003	60.94846	58.18975
HPS1	3.880555	4.299134	3.809083	4.672447	4.191307	4.508153	3.950645	4.280106
HPS3	4.346936	4.985915	4.159718	4.098533	4.78012	3.905413	4.505709	5.271882
HPS4	3.562768	3.730556	2.58847	3.564605	4.06925	3.254575	3.537752	3.542215
HPS5	7.156695	7.116968	5.857032	6.01025	6.75631	6.535943	7.358891	8.529662
HPS6	1.411969	1.738329	1.641481	1.804101	1.653469	2.042447	1.478502	1.377687
HPSE	0.391799	0.583008	0.169276	0.690086	0.526548	0.394476	0.310685	0.335287
HPX	0	0	0.018042	0	0	0	0	0
HR	148.9942	115.4489	114.7848	122.6555	113.7558	114.1516	118.3255	121.9073
HRAS	34.4831	28.35649	29.85277	27.46194	34.26067	32.12049	30.53896	30.45844
HRASLS	0	0	0	0.018096	0	0	0	0
HRASLS5	0.124507	0.100913	0.147547	0.025518	0.074714	0.075215	0.176924	0.050512
HRC	0.2043	0.457539	0.201755	0.187323	0.290359	0.487175	0.196451	0.261737
HRCT1	0.532885	1.285426	0.526245	0.598093	0.913636	0.740916	1.493855	1.389783
HRH1	3.24779	2.917173	2.489265	4.421094	4.55719	3.572572	3.621238	3.746338
HRH2	0.124823	0.165372	0.123268	0.103316	0.153648	0.116008	0.063348	0.199638
HRH3	0	0.011471	0	0.011603	0	0	0	0.011484
HRH4	0.171888	0.580481	0.212183	0.601833	0.515732	0.30286	0.479784	1.307515
HRK	0.250584	0.171088	0.360434	0.195385	0.207031	0.285204	0.060821	0.099451
HS1BP3	7.828902	7.284862	8.436465	8.504885	10.47629	7.96696	7.110777	6.952067
HS2ST1	5.23427	5.316026	3.985007	3.923856	4.190536	3.387875	4.697758	6.747124
HS3ST1	0.905012	1.330608	0.828341	1.017911	1.258351	0.788962	1.400286	2.182801
HS3ST2	8.041831	7.661867	7.409228	7.504401	5.571597	5.020901	10.85288	8.794235
HS3ST3A1	0.009582	0.009708	0.037851	0.058916	0.009583	0.057885	0.048629	0.038874
HS3ST4	0.050522	0.051185	0.019957	0.051773	0	0.010173	0.061536	0.020496
HS3ST5	0.007238	0.007333	0.021443	0.007417	0.007239	0.080161	0.014693	0.022023
HS3ST6	21.45836	19.31635	23.2463	24.34332	25.1935	25.43459	23.28567	20.89037
HS6ST1	22.71395	16.18057	15.94419	19.65032	20.98005	13.96582	18.06169	18.8219
HS6ST2	6.518025	3.960753	4.401641	4.918397	4.156463	5.666406	6.268327	6.006673
HS6ST3	0.19683	0.139589	0.174939	0.040341	0.019686	0.059452	0.079913	0.179668
HSBP1	76.06112	70.59315	77.12495	78.31627	71.00056	71.93211	65.68529	67.8548

HSBP1L1	12.42436	20.00465	23.58933	22.20578	18.62914	26.63895	19.00574	14.69148
HSCB	7.745429	9.666176	11.26477	11.14828	9.190083	8.649071	12.47137	10.81943
HSD11B1	3.071981	5.365647	12.91771	14.66845	7.895683	9.057185	13.73512	4.589814
HSD11B1L	0.444914	0.655641	0.559199	0.721207	0.703865	0.602702	0.492642	0.557907
HSD11B2	0.947002	0.936587	0.475023	0.762504	0.774236	1.165354	0.854406	0.731797
HSD17B1	0.328277	0.462727	0.253711	0.775202	0.770836	0.617928	0.188331	0.318474
HSD17B10	66.8708	74.57445	76.32303	73.50421	73.25293	72.04792	70.69482	64.63377
HSD17B11	4.53085	6.930725	8.482444	6.761895	7.441766	7.029031	5.997888	6.653548
HSD17B12	5.07724	6.340951	8.08706	7.043239	8.611364	9.311007	4.423136	5.683454
HSD17B13	1.407035	13.77253	10.62435	15.88294	10.30515	11.94342	4.306392	7.772013
HSD17B14	14.98459	12.85329	15.90833	15.15536	17.35797	13.4872	13.17483	9.09891
HSD17B2	10.78501	10.57621	6.317809	8.19499	6.29027	9.705364	7.436797	6.159782
HSD17B3	0.065319	0.066176	0.048379	0.11714	0.048996	0.082206	0.049724	0.066249
HSD17B4	34.16568	36.37093	36.54923	31.48782	29.51275	39.45056	33.17515	34.63229
HSD17B7	6.184505	6.884808	6.554037	8.667365	7.872206	6.940494	6.128405	5.689918
HSD17B8	26.6331	26.26822	26.78335	31.04488	27.36891	27.25195	26.78484	21.84085
HSD3B1	0	0	0.03214	0	0.048825	0	0	0.016504
HSD3B7	4.679819	4.18268	4.862079	3.876009	5.975555	4.014703	4.645906	5.53968
HSDL1	6.346466	6.720325	6.666816	5.915853	5.788313	5.671615	7.128375	5.534316
HSDL2	21.10565	22.67943	21.01121	21.62844	21.43553	22.93951	20.58498	20.07904
HSF1	22.60463	22.16339	18.63673	21.51185	23.41404	20.97814	19.6698	21.71283
HSF2	8.007209	9.897627	8.650351	8.501246	10.06957	10.08517	9.581239	8.716961
HSF2BP	0.797112	1.279928	0.78718	1.017218	0.75209	0.741987	1.00752	0.991507
HSF4	1.463312	1.482516	1.050966	2.344764	2.035597	1.848332	1.458257	1.807956
HSF5	0.523291	0.348788	0.496372	0.762043	0.605994	0.422879	0.587049	0.663425
HSH2D	0.278301	0.362512	0.314096	0.611132	0.238575	0.120087	0.443891	0.907274
HSP90AA1	537.4466	498.5302	532.6016	467.7434	552.8188	516.4386	460.1519	573.2174
HSP90AB1	478.4243	468.6683	463.8981	406.3706	424.1738	402.6591	431.8436	454.4808
HSP90B1	218.0162	236.5767	213.1802	207.0626	233.4045	219.3767	227.7017	221.3495
HSPA12A	2.605044	3.108345	2.093013	2.762158	3.000706	2.12253	2.64412	2.649765
HSPA12B	7.039247	7.103292	6.260987	7.223166	9.949467	8.392157	7.949221	7.943235
HSPA13	11.02603	13.87921	12.22209	13.96228	14.65804	13.5744	13.69142	12.53872
HSPA14	23.67184	21.18852	25.36943	22.95199	23.19906	23.68571	20.247	22.15604
HSPA1A	36.82479	25.77997	23.59338	37.93055	29.92583	17.10429	24.91319	40.47371
HSPA1L	2.539163	1.764273	1.786972	1.664919	2.023809	1.812085	1.461429	1.79581
HSPA2	49.02663	40.4396	45.47666	41.65199	42.9923	38.70487	31.97031	41.89706
HSPA4	43.54417	43.71169	40.35714	38.49887	43.41687	42.16648	40.44011	43.09564
HSPA4L	26.08788	21.10983	18.6503	17.19499	23.74438	21.28472	18.61651	24.12947
HSPA5	196.3379	171	199.9761	198.7604	231.1893	215.9745	185.3679	190.2639
HSPA6	1.406289	0.675736	0.944361	0.790559	0.843882	0.67963	0.905369	0.929138
HSPA8	540.3691	409.1803	354.009	325.8126	501.1599	402.1766	373.6199	436.3556
HSPA9	73.09958	74.72582	76.08901	73.61037	73.04045	74.23409	75.42792	72.39466
HSPB1	763.3381	620.115	742.8957	708.7783	877.2178	745.5956	642.7231	646.3075
HSPB11	4.696487	6.368196	5.22357	5.25035	5.076657	5.588311	7.174477	5.436949
HSPB2	1.481431	1.520885	1.89211	2.105135	2.153291	2.167721	1.744237	1.00168
HSPB3	0.015885	0.032187	0	0.016278	0.047661	0.04798	0.112862	0.16111
HSPB6	19.93254	19.84762	21.7483	20.05631	32.47771	20.47047	16.60567	23.80088
HSPB7	0.111808	0.125861	0.07361	0.369193	0.273343	0.137587	0.290016	0.176399
HSPB8	138.5757	134.2047	151.3872	132.4522	137.3073	144.8713	139.1076	108.9846
HSPB9	0	0	0.046769	0	0	0	0	0
HSPBAP1	2.010936	2.00411	1.575752	1.926351	2.295386	2.112703	1.541918	1.485334
HSPBP1	9.684283	8.685483	8.842424	11.17689	10.7176	9.702488	10.03903	9.17807
HSPD1	53.75641	70.53766	72.99551	54.8734	67.85283	72.50063	53.67719	67.37043
HSPG2	8.063123	6.696955	5.343057	7.11124	7.532398	5.69451	8.176163	9.228788
HSPH1	35.05313	23.19164	27.55764	18.22621	32.13111	24.28284	23.25006	29.26095
HTATIP2	25.13245	26.96795	28.35792	30.1039	30.75077	33.48394	26.21338	26.89705
HTATSF1	28.24488	32.06984	29.10202	29.3495	28.12453	31.26992	28.45619	27.62526
HTD2	1.725907	1.151489	1.866528	1.7816	2.319751	2.398871	1.884249	2.258542

HTR1B	4.187773	3.343166	3.513945	2.607497	4.115416	3.796078	3.463692	4.906002
HTR1D	0.336237	0.196132	0.181117	0.407214	0.377041	0.328275	0.299913	0.206681
HTR1E	0.018139	0	0.017913	0.018589	0.036284	0.018263	0	0.018398
HTR1F	0.02055	0.02082	0.060882	0.273768	0.020553	0.02069	0.208583	0.125056
HTR2A	0.075476	0.025489	0.068325	0.045118	0.044034	0.082325	0.025536	0.025517
HTR2B	1.061038	1.100971	1.352023	1.245157	3.902387	1.412896	1.033528	1.362536
HTR2C	0.01945	0	0.038416	0	0.116718	0.05875	0	0.039455
HTR3A	0.053542	0.067806	0.052875	0.233191	0.093711	0.161724	0.040759	0.054304
HTR4	0.035133	0.053391	0.034695	0.024002	0.193256	0.047164	0.03566	0.011878
HTR6	0	0.008049	0.007846	0.008141	0	0	0	0.024173
HTR7	0.08326	0.210881	0.150741	0.227526	0.388596	0.125743	0.253526	0.50667
HTRA1	147.3782	121.168	156.3964	134.4281	146.3765	125.211	138.3262	137.2798
HTRA2	10.82248	10.15678	9.230223	10.77765	12.53737	12.17734	11.96622	11.9572
HTRA3	11.63818	15.16456	14.55219	15.29354	18.52171	13.57782	16.85451	16.15737
HTRA4	1.70834	0.996498	1.346235	1.220154	1.156299	1.598394	2.259409	1.52264
HTT	10.88567	9.228695	7.700461	9.374493	10.10006	8.787235	11.07013	11.88712
HUNK	4.969875	6.095432	4.919497	4.667567	5.327225	5.851534	4.84858	4.927951
HUS1	7.739825	9.275158	6.142314	6.821485	7.531142	9.305504	5.355505	8.558811
HUWE1	25.26184	21.20156	18.55063	20.03348	21.14275	19.00524	20.44393	22.92357
HVCN1	2.370884	2.837261	1.587085	2.18502	1.846028	2.050647	2.47105	3.792546
HYAL1	10.31112	11.51453	11.47769	12.9776	13.93853	12.65981	13.88479	11.74884
HYAL2	8.687726	8.324456	7.129038	8.363353	9.270877	9.863131	8.972744	9.078413
HYAL3	1.493162	1.497322	2.542858	2.139084	2.636228	3.236831	3.062048	2.317987
HYAL4	0.012451	0.012614	0.012296	0	0	0.012536	0	0
HYDIN	0.017803	0.023447	0.012307	0.005473	0.012463	0.00717	0.021684	0.009028
HYI	10.47232	10.60976	8.742581	11.34022	9.663854	9.185117	7.615914	7.528054
HYKK	0.867556	1.254766	1.400337	1.232402	1.262607	0.783123	1.603244	1.316826
HYLS1	1.836005	1.913628	1.969657	1.854411	2.047608	2.06133	1.970799	2.371216
HYOU1	14.72004	13.68305	11.84371	12.32186	14.68077	12.25121	13.22794	13.87549
HYPK	24.75309	26.11328	26.72467	23.60146	26.85717	25.09368	25.39331	24.10739
IAH1	12.14884	15.26673	16.56793	16.09154	16.06279	11.15011	17.78359	12.76884
IARS	28.10304	31.41392	29.15864	31.0489	30.35293	30.7984	28.51817	28.23361
IARS2	31.40498	31.961	32.4471	29.40195	30.63584	27.84678	31.52374	29.51575
IBA57	4.037781	4.064209	4.699518	3.922848	4.19564	5.068507	4.27133	4.720188
IBSP	0	0	0	0	0.012689	0	0	0
ICA1	14.12808	13.07374	13.82021	17.49904	13.05069	13.84378	15.1191	12.19674
ICA1L	0.058585	0.148386	0.101247	0.165101	0.058593	0.029493	0.059464	0.103984
ICAM1	9.766872	10.32162	8.547739	10.60296	12.55706	8.450637	12.46353	13.46224
ICAM3	1.152655	1.401338	1.004376	1.417447	1.613925	0.901117	1.431461	2.819506
ICAM4	0	0.023769	0	0.048085	0.023464	0.118107	0	0
ICAM5	0.060206	0.121993	0.184974	0.185092	0.16057	0.107764	0.332706	0.210329
ICE1	4.583566	4.374836	3.906635	4.234008	4.698942	4.152669	4.499427	5.059862
ICE2	2.734761	3.079114	3.162433	2.808074	2.887367	2.753444	2.538489	3.308579
ICK	4.801983	4.605042	4.090556	4.524417	4.4808	3.981831	4.849208	5.630429
ICMT	16.92285	14.68786	14.15729	13.21824	15.55953	14.3615	15.18915	15.30526
ICOS	1.455314	2.076638	0.728711	1.575383	1.752752	0.650078	1.570765	1.517609
ICOSLG	5.484191	4.708834	5.655441	5.83594	5.357195	3.728243	5.851581	4.901784
ID1	81.45637	85.26958	92.00469	91.45427	99.07526	94.11449	77.86701	82.64457
ID2	51.75036	64.29471	59.39349	52.55564	60.1718	61.00072	51.53959	50.79324
ID3	86.84438	89.45507	93.27644	92.08146	100.3007	98.83434	83.5896	91.78933
ID4	38.15017	47.61729	49.34725	43.00587	37.88828	43.89972	42.19555	44.81077
IDE	22.3468	21.47385	22.70942	21.12122	22.74225	21.77367	21.39103	23.76735
IDH1	49.46583	61.51667	57.32868	67.26653	74.23637	69.23772	66.5228	57.52555
IDH2	34.23602	30.71269	28.36147	30.33136	32.67177	26.94153	38.11198	33.59886
IDH3A	23.32608	21.94959	24.5494	22.64922	24.71329	26.19735	27.13389	25.73133
IDH3B	50.33045	53.00093	51.32716	49.19611	50.33696	53.51752	51.55708	51.50012
IDH3G	48.88802	50.17885	44.70643	49.9507	44.94558	46.51635	52.64268	46.64877
IDNK	4.152864	7.521387	8.398866	5.771472	5.291319	8.562939	5.254511	5.510196

IDO1	0.111867	0.158669	0.265136	0.114638	0.939806	0.405473	0.181672	0.81691
IDS	15.36269	14.51032	21.13375	19.85672	20.00638	20.19785	20.2701	20.94863
IDUA	3.415485	3.502767	2.653852	3.000842	3.82458	3.154008	3.349742	3.984785
IER2	44.1357	45.42316	46.50953	47.77134	44.47041	44.79603	45.46553	40.2876
IER3	109.2658	107.9737	102.3399	118.1708	108.6129	96.95253	94.21424	85.34943
IER3IP1	13.17865	16.01566	17.89486	16.74663	17.71303	16.14979	16.44566	14.39383
IER5	19.97763	18.81357	21.37172	22.00694	18.81545	19.36676	20.36823	18.73037
IER5L	4.024586	3.047757	2.870428	3.114037	4.004777	3.806499	2.878036	3.710798
IFFO1	1.143897	1.352061	1.022059	1.060591	1.334719	0.9689	1.437497	1.500869
IFFO2	31.82317	21.88263	19.28379	23.73273	26.16482	23.48759	25.63344	26.89603
IFI30	37.77399	36.34857	33.93254	42.36299	36.14786	38.37571	41.30469	45.77406
IFI35	6.910905	9.14327	7.667857	8.519338	8.843036	7.592533	8.706317	7.833908
IFI44	1.009097	1.431277	0.802464	1.132059	1.301372	1.443776	1.498615	1.44362
IFI44L	1.460973	2.337074	1.265587	2.377072	2.563442	2.141915	1.782067	2.300644
IFI6	19.59568	20.44844	11.4819	15.86405	18.12835	23.37952	21.87858	21.19961
IFIH1	6.54282	6.843028	6.577366	5.853728	7.194114	7.723568	7.189736	6.969746
IFIT1	5.0009	4.664426	3.527563	4.506547	5.708114	7.27287	6.534232	3.630973
IFIT2	2.410846	1.767497	3.377896	2.449447	3.221746	2.82841	3.102333	3.566738
IFIT3	2.606398	3.110712	3.090657	3.530928	3.396655	4.204691	3.427118	2.873807
IFIT5	13.93606	15.24543	12.43323	12.13094	16.43782	15.20966	14.80696	15.16889
IFITM10	0.15184	0.230749	0.099965	0.207468	0.430269	0.152877	0.154118	0.154001
IFITM5	0.826347	0.297068	0.710754	1.010715	0.879773	0.509931	0.730517	0.567752
IFNAR1	7.856518	8.392944	9.25705	8.442465	9.894484	7.730763	8.997648	10.74154
IFNAR2	5.546744	5.077304	5.585742	6.781078	6.958658	6.282717	6.370728	7.735346
IFNE	0.017976	0	0.017752	0.018422	0	0	0	0
IFNG	0	0.07188	0.035032	0	0.141915	0.035717	0	0.035979
IFNGR1	25.26569	27.82538	28.46117	34.094	30.51201	30.0728	34.66443	37.71182
IFNGR2	42.18044	41.08075	48.82037	49.61574	48.20413	44.27297	46.76172	45.92852
IFNK	0.380957	0.20433	0.02213	0.068893	0.112061	0.157936	0.068236	0.022728
IFNLR1	2.05468	1.79008	1.387969	1.721497	1.61986	1.489207	1.630359	1.764892
IFRD1	17.25841	17.13483	18.04227	19.5852	16.09192	17.62934	20.19558	17.22373
IFRD2	12.54984	15.43805	13.62856	14.77582	15.75762	15.57375	15.27695	14.53644
IFT122	4.632343	5.184477	4.459019	4.2958	4.967451	4.456327	5.465663	5.365433
IFT140	2.604161	2.79055	1.884824	2.834033	3.311273	2.313816	2.778773	3.013714
IFT172	4.227908	4.078674	3.714724	3.679554	3.798354	3.432559	3.218495	4.23029
IFT20	25.06036	27.42762	29.19975	26.28139	27.63619	31.28304	27.11651	26.16619
IFT22	6.9397	10.6191	8.815359	10.53461	9.705317	7.94385	9.323531	7.418161
IFT27	10.40081	9.894789	10.39647	10.63678	9.471878	11.15296	11.15761	9.433948
IFT43	1.898511	1.810946	1.480149	1.570083	1.865445	1.486708	1.633997	1.869236
IFT46	16.75574	16.22648	16.40713	15.10707	15.60924	15.25447	18.15685	16.57938
IFT52	3.374355	3.890177	3.729262	3.132741	3.258419	3.450658	3.983273	3.583319
IFT57	8.301761	9.320335	9.406222	9.320801	10.4371	8.777708	8.822507	8.208783
IFT74	3.865662	6.158606	4.633449	4.95933	5.356552	5.622705	5.151659	5.065475
IFT80	4.62621	5.620694	4.580311	3.954731	5.512919	4.891303	5.836309	5.240882
IFT81	4.380376	5.198343	4.031317	4.615327	4.494065	4.068658	4.560887	4.849463
IFT88	5.978763	7.047848	6.105434	7.379368	6.509239	7.055348	5.923711	6.177507
IGBP1	16.55273	15.09669	15.67957	15.45829	16.60626	15.83067	14.77447	14.65911
IGDCC3	0.697435	0.474158	0.344372	0.41378	0.201915	0.646763	0.428464	0.428141
IGDCC4	0.925997	1.019336	1.09911	1.510084	1.353556	0.94577	1.283308	1.228158
IGF1	4.825809	5.096105	3.439987	4.379853	6.166372	4.292499	3.866856	6.360977
IGF1R	25.49296	22.06213	18.20495	19.36834	21.69862	20.17698	19.35735	21.7418
IGF2	12.39924	11.85612	10.42997	12.67533	17.52074	10.9997	9.428673	12.47334
IGF2BP1	0	0	0	0	0	0	0	0
IGF2BP2	6.764048	6.687406	5.397559	4.644966	7.200366	6.559765	5.011032	7.286153
IGF2BP3	0.029629	0.070041	0.243829	0.222659	0.15804	0.159099	0.16039	0.160269
IGF2R	11.84435	10.12573	9.18344	9.916518	10.44892	9.213972	11.99647	10.75583
IGFALS	1.505045	1.176103	1.532378	1.703731	1.948643	1.691527	3.357212	1.260225
IGFBP1	0.867074	0.527072	0.82202	0.781926	1.214061	3.701511	0.10561	0.070353

IGFBP2	14.38835	14.21059	20.0104	22.0735	15.66744	20.63703	19.98352	19.38561
IGFBP3	92.72278	97.67757	79.07215	67.81862	93.25529	67.70277	76.68083	111.0636
IGFBP4	55.97833	56.89546	50.97647	76.19126	110.0617	75.83676	69.35892	65.10517
IGFBP5	45.50244	49.94876	63.5105	60.03508	50.88503	55.49973	60.74662	69.38792
IGFBP6	42.16089	63.61267	48.71089	56.67913	63.15109	38.04945	58.656	77.5369
IGFBP7	193.1351	172.0006	172.5825	200.1384	173.9294	193.9589	199.2294	190.0938
IGFBPL1	0	0	0.022843	0	0.046269	0.02329	0	0
IGFLR1	5.832924	5.70725	7.92855	8.386553	7.9409	7.123248	7.541217	5.893465
IGFN1	0	0	0	0.006495	0	0.025526	0	0
IGHMBP2	4.04165	3.788338	3.634405	3.67317	3.990539	4.158369	3.623177	3.747613
IGIP	4.255495	4.282409	5.076811	4.068227	4.42743	4.974699	4.203373	5.098183
IGLON5	1.771687	1.193678	1.008397	2.736774	1.029981	1.072032	2.799266	1.186136
IGSF1	0.031648	0.02565	0.031253	0.019459	0.050643	0.006373	0.012849	0.019259
IGSF10	0.528698	2.43618	1.170031	1.997459	2.009951	1.228161	1.299547	2.083524
IGSF11	0.108907	0.06896	0.067219	0.251111	0.081691	0.19189	0.110541	0.110458
IGSF21	0.269818	0.164016	0.071055	0.129034	0.82755	0.253551	0.036515	0.109464
IGSF22	0	0	0	0	0	0	0	0
IGSF23	0	0.027398	0	0	0.027046	0	0	0
IGSF3	3.286162	2.004255	1.848724	2.260058	2.769966	2.305332	2.398404	2.716144
IGSF5	1.06546	1.586312	1.42731	1.822917	1.454772	1.296614	1.78673	1.099421
IGSF6	0.714921	1.412392	0.941351	1.147787	1.370443	0.839773	1.620607	1.716067
IGSF8	15.71709	12.74365	11.38064	12.24941	13.30362	12.80028	10.87581	11.70072
IGSF9	4.599764	3.830297	2.572682	3.598423	3.284345	3.065823	3.144581	3.565277
IGSF9B	0.068778	0.022005	0.035748	0.111287	0.086889	0.036446	0.084507	0.040386
IHH	0	0	0.012802	0	0	0	0	0
IK	81.66486	84.3305	84.54549	82.22209	79.25805	84.19553	88.23194	79.95529
IKBIP	3.292843	4.181046	5.380468	3.685208	4.474079	4.035115	5.178266	4.361409
IKBKB	7.880717	7.467021	6.358957	8.081673	7.780759	7.652186	7.617484	7.230591
IKBKE	1.904879	2.720631	2.118308	2.198169	2.314536	2.286083	2.39327	2.568614
IKBKG	4.130052	3.705166	4.042202	3.961736	4.096804	3.839827	3.584241	3.836921
IKZF1	1.668744	2.321555	1.321244	1.550538	1.92198	0.871911	1.55057	2.733658
IKZF2	1.09237	1.162205	1.059666	1.079803	1.144075	0.775398	1.154545	1.310549
IKZF3	0.440801	0.737387	0.442058	0.73886	1.021834	0.378445	0.634702	1.514514
IKZF4	0.844144	0.849633	0.735552	0.949863	0.656641	0.527722	0.851206	0.973673
IKZF5	4.140978	3.769094	3.460245	4.982631	3.52239	3.866708	4.447104	4.26698
IL-15L	0	0.0501	0	0	0.098914	0	0	0
IL10	0.196033	0.639952	0.774362	0.602667	0.544607	0.175442	0.508488	0.795295
IL10RA	2.162282	1.899024	1.646129	2.682335	2.27638	1.597405	2.921758	3.252252
IL10RB	9.631406	8.964046	8.777699	9.468536	9.03821	8.40515	6.279596	5.713143
IL11	0.587185	0.854203	0.535264	0.570872	0.752899	0.727627	0.412611	0.534463
IL11RA	7.909981	11.59392	10.00694	12.32675	10.40367	10.60298	9.382572	11.38115
IL12A	0	0	0	0	0	0	0	0
IL12B	0.113777	0	0.02809	0.029149	0.056896	0.057277	0	0.057698
IL12RB1	0.152882	0.178717	0.12775	0.301286	0.164663	0.047362	0.202921	0.143131
IL12RB2	0.088898	0.117777	0.054025	0.035038	0.061553	0.04131	0.020823	0.027743
IL13	0.021663	0.021948	0.021394	0	0.021666	0	0.043977	0.087887
IL13RA1	48.96226	48.46327	59.34603	57.23407	47.29323	53.63801	50.59639	54.07055
IL13RA2	0	0.040567	0	0.020517	0.100117	0	0	0.101529
IL15	0.234165	0.639224	0.3276	0.786551	0.539949	0.785881	0.706429	0.343053
IL15RA	5.03414	3.977832	4.279599	4.340769	4.56227	4.166365	4.315919	4.296146
IL16	1.513705	1.640338	1.499574	1.63465	1.566599	1.085159	1.200928	1.773315
IL17A	0	0.061378	0	0	0	0	0	0
IL17B	1.052059	4.330083	3.354945	2.021474	2.148232	0.794435	1.53502	0.177839
IL17C	0	0	0	0.026314	0	0	0	0
IL17D	1.349307	1.451698	1.521166	1.2053	2.060049	1.256335	1.07261	1.489623
IL17F	0.088155	0	0.152349	0.022585	0.022042	0.022189	0.022369	0.022352
IL17RA	14.55983	15.75019	15.60721	16.40553	14.52377	15.04125	12.85297	14.00527
IL17RB	0.418304	0.461464	0.514069	0.733494	1.078433	0.608344	0.63215	0.952225

IL17RC	8.063075	7.621405	7.412002	9.370367	8.209958	7.254525	8.210141	8.63895
IL17RD	1.229705	2.027882	1.574078	1.359472	1.386939	1.443336	1.66194	1.528511
IL17RE	5.188394	6.375822	5.166533	7.459217	5.264896	6.587053	5.969848	4.449296
IL18	7.822743	12.35129	13.21824	16.50153	11.60862	13.27188	11.80701	9.68579
IL18R1	0.215117	0.10897	0.094416	0.036741	0.131478	0.012033	0.060651	0.339391
IL18RAP	0.105392	0.213551	0.118947	0.277722	0.240928	0.045477	0.213946	0.717707
IL19	0	0	0	0	0	0	0	0
IL1A	0.808538	1.176437	1.121246	1.084187	0.886065	0.718799	2.077856	1.832021
IL1B	0.88809	0.522433	0.76386	0.851373	0.658991	0.201906	0.378011	0.464894
IL1F10	87.98675	79.40408	122.4979	77.42177	92.8335	71.9318	44.93411	59.72918
IL1R1	12.57632	12.72077	15.79309	12.07518	17.22985	13.75751	13.36864	21.89651
IL1R2	63.12928	70.04829	69.22688	85.67869	76.94642	67.12272	76.99494	92.35476
IL1RAPL1	0.005739	0	0	0	0	0	0.005825	0
IL1RAPL2	0.013271	0	0.026212	0.0272	0.053092	0.026724	0.026941	0.01346
IL1RL1	1.177468	1.455582	0.960107	1.771206	1.03717	0.989739	1.118378	2.837665
IL1RL2	6.757642	4.961665	5.67055	7.391887	6.274408	4.921293	9.276999	9.799452
IL1RN	19.95022	10.88708	19.96898	19.8172	19.30537	22.3855	15.18412	11.43622
IL2	0	0.24615	0.095974	0.448164	0.097197	0.048924	0.049321	0.098568
IL20	0	0	0	0	0	0	0	0
IL20RA	14.51767	19.42069	16.06927	17.51626	19.94425	20.3696	15.86265	20.22556
IL20RB	51.838	46.90654	46.5232	53.88175	41.12883	54.22011	41.32864	45.1563
IL21R	0.032717	0.397753	0.096927	0.335271	0.409012	0.148231	0.381886	0.381598
IL22RA1	1.006374	1.14072	0.846236	0.796452	1.106158	0.652091	0.606814	0.74784
IL22RA2	0.036974	0	0.036513	0.03789	0	0.018613	0.093821	0.0375
IL23A	0.12156	0.030789	0.030011	0.093428	0.212757	0.030598	0.061692	0.12329
IL23R	0.138014	0.377527	0.163553	0.141432	0.179441	0.125061	0.126075	0.083987
IL24	0	0	0	0	0	0	0.024116	0
IL25	0.563162	0.475461	0.81568	0.78872	0.826078	0.963916	0.743093	0.723494
IL26	0	0.034288	0	0.034683	0	0	0	0
IL27	0.069973	0.085069	0.082921	0.086047	0.097975	0.042271	0.028409	0.028388
IL27RA	26.7287	23.44228	25.37317	30.71227	29.59108	27.81778	26.93544	27.17768
IL2RA	1.49036	2.388028	1.189957	1.819733	1.511695	1.202561	1.856026	3.473408
IL2RB	1.532707	2.762905	1.111344	2.518737	2.161258	1.160859	1.78695	2.506848
IL2RG	7.529795	11.01701	9.484094	13.05837	14.25801	8.860451	10.71501	16.01304
IL31RA	0.344482	0.290836	0.132296	0.205925	0.239255	0.35647	0.126262	0.368796
IL33	16.80006	16.74629	19.28834	17.62812	23.69377	20.21025	18.95876	16.49011
IL34	1.606855	1.254084	1.157834	1.924291	1.817288	2.144568	1.891721	1.160708
IL36B	0	0	0.027072	0.028093	0	0.027601	0	0.055607
IL36G	0.809813	1.777622	1.299549	2.178415	1.451103	1.970418	1.113071	1.694016
IL36RN	18.96739	14.01007	17.71895	18.23903	20.02372	16.24841	11.83831	13.58623
IL3RA	2.859263	2.270849	2.610799	2.871191	4.612774	3.081324	3.602168	3.103984
IL4	0	0	0	0	0.052519	0	0	0
IL4I1	3.488386	3.686621	3.188239	4.135546	3.324656	3.154108	3.068617	3.177303
IL4R	4.095437	3.657099	3.706465	4.558877	4.476858	4.151201	4.543424	4.48962
IL5	0	0	0	0	0	0	0	0
IL5RA	0.016282	0.313423	0.209032	0.200227	0.341971	0.016393	0.429688	1.238552
IL6	0.050843	0.077266	0.100419	0.182359	0.101699	0.204762	0	0.051567
IL6R	12.12044	13.43324	9.782367	12.63764	13.44409	9.714191	12.86566	15.35171
IL6ST	31.69514	31.68897	27.76702	32.29138	32.71301	27.73262	34.59018	41.03322
IL7	0.458839	2.239783	0.988629	1.581596	1.376694	0.923947	1.651194	1.819176
IL7R	4.874279	7.59494	2.547642	4.90793	6.653674	2.536295	5.224644	6.64286
IL9	0	0	0	0	0	0	0	0.066638
ILDR1	2.062269	1.788365	1.998307	2.029524	1.748203	1.612536	1.53822	1.637495
ILDR2	0.57431	0.472323	0.587171	0.723553	0.760214	0.676873	0.524632	0.760658
ILF2	90.87987	101.7771	87.88579	97.08179	88.34373	98.25511	95.81143	88.67682
ILF3	23.63617	25.57869	21.11971	23.41639	22.3897	22.00471	24.30631	23.92105
ILK	45.59964	47.8535	45.33028	48.52763	52.21789	42.49905	52.91753	45.34345
ILKAP	33.46397	39.40826	30.55633	35.04934	37.77488	35.17902	36.5883	35.41692

ILVBL	28.70528	20.38602	23.99487	20.54802	17.57241	17.43629	18.88815	27.31348
IMMP1L	17.08019	17.5505	17.99506	15.73552	17.2768	23.92396	15.43753	18.35831
IMMP2L	5.154074	7.680372	5.272449	5.234372	4.692432	5.491799	4.715284	5.110238
IMMT	25.85125	26.06495	27.05905	28.67188	27.6829	26.18383	29.17426	27.18297
IMP3	17.12354	18.9767	17.67209	19.30466	18.94764	18.66464	18.81607	17.1282
IMP4	21.32508	17.42841	14.27935	16.99349	18.75485	15.46332	19.4899	16.15862
IMPA1	6.790549	7.315608	7.057834	7.833765	7.954711	6.83017	7.213143	7.412282
IMPA2	21.66903	20.95983	20.10002	25.4521	21.14249	22.86717	21.06185	19.64081
IMPACT	3.3488	3.870712	5.712913	4.081644	3.805595	3.714296	4.050579	3.451381
IMPAD1	28.98313	29.39638	29.70373	29.36845	31.57151	29.31184	28.62313	31.42017
IMPDH1	16.08205	14.80408	18.80558	17.99748	16.85106	18.76547	18.10696	15.39282
IMPDH2	63.31265	65.76951	64.58411	61.44363	57.26964	62.90493	61.59085	59.31448
IMPG2	0.960677	0.738166	0.660897	0.707936	0.777277	0.575997	0.4985	0.908666
INA	0.017379	0.008804	0.017163	0	0	0.008749	0.00882	0
INAFM1	11.082	11.73124	8.348247	12.13302	9.994039	10.99082	7.859343	9.558602
INAFM2	2.353717	2.865226	3.04513	2.5803	3.613149	3.251582	2.481621	2.905381
INAVA	6.754909	6.392691	5.854557	6.466178	6.421967	5.616872	5.033281	6.093422
INCA1	0.25528	0.205578	0.148674	0.201233	0.288045	0.085675	0.252465	0.278831
INCENP	4.02807	3.555814	3.558642	3.389286	3.352222	3.379657	3.968242	3.885147
INF2	10.92408	8.635311	8.914139	9.753405	9.155101	7.953006	9.192777	8.921468
ING1	13.03282	12.06377	11.19056	13.23497	12.41943	13.14821	14.46349	12.30262
ING2	3.75076	3.63269	3.552603	3.626102	4.057951	3.717007	3.399982	3.170129
ING3	5.302504	6.460112	6.245423	5.128147	5.564246	5.594026	6.525063	5.36286
ING4	30.36141	31.36071	32.04922	30.79271	32.2436	32.22747	33.024	34.15201
ING5	8.039155	8.028892	6.936656	7.177505	7.616672	7.119537	7.395619	7.96271
INHA	0.176667	0.204555	0.348932	0.206907	0.151449	0.101642	0.051234	0.537547
INHBA	2.536345	2.069502	2.656035	2.376759	2.536673	2.566526	2.595988	2.045876
INHBB	17.26102	12.75097	16.45785	16.61379	17.36104	15.01641	14.75031	14.27944
INHBE	0.01263	0	0.049892	0.012943	0	0	0.03846	0
INIP	18.60613	20.56071	19.28687	20.31447	18.3686	23.51048	19.3542	18.97914
INKA1	7.673127	8.935754	7.415721	11.58492	9.940852	9.127692	7.621927	8.668606
INKA2	3.673342	3.137966	2.667058	2.254728	2.738835	2.5195	4.063906	2.585916
INO80	7.865137	7.669143	6.376141	7.142679	7.616218	6.962748	7.222159	7.907977
INO80B	8.166994	7.426028	6.83432	7.387475	6.707476	6.471465	7.250952	6.594529
INO80C	13.656	16.16778	15.82637	14.50315	12.39358	14.22654	20.0925	13.07204
INO80D	2.528817	1.956936	1.402743	1.709259	2.050221	1.522239	1.824031	2.646672
INO80E	5.127674	5.061001	4.461642	4.810524	4.775672	4.05324	4.928701	4.314022
INPP1	7.955407	7.176936	6.801374	6.846534	6.691278	7.43426	7.114139	7.983124
INPP4A	7.353729	7.29371	7.326343	7.921983	7.422452	6.92365	7.054104	7.331953
INPP4B	2.56683	2.335353	1.943175	2.591633	2.891999	2.263701	2.79097	3.294191
INPP5A	37.80355	33.95016	33.34507	42.20319	36.81606	40.46539	35.36916	34.60518
INPP5B	12.84749	14.05828	13.00897	14.08338	13.00458	12.61485	12.88997	13.3156
INPP5D	2.562107	2.919476	2.558358	2.923799	2.836374	2.188934	2.716376	3.408828
INPP5E	2.977899	2.3917	2.110362	2.466629	2.669653	2.322473	2.309993	2.159587
INPP5F	7.0996	6.87868	6.851933	6.76713	7.385778	7.466488	6.746666	6.527766
INPP5J	0	0.050524	0.043092	0.063881	0.087283	0.043934	0.094908	0.018967
INPP5K	12.61928	13.12449	12.75412	14.56855	12.81811	12.46726	12.76854	14.2688
INPPL1	13.38234	12.71133	10.26415	13.07154	13.23316	11.89033	13.41814	12.24263
INS	0	0.089171	0	0	0	0	0	0
INSC	0.327742	0.584396	0.569639	0.483639	0.616235	0.475173	0.572172	0.531852
INSIG1	24.45865	24.72996	28.35709	29.24526	28.64994	29.4245	23.87989	14.33306
INSIG2	10.33199	11.85061	13.04924	10.76164	10.63672	11.36378	11.46503	12.6328
INSL3	0	0	0.069799	0	0	0.142325	0	0
INSL5	0	0	0	0	0	0	0	0
INSL6	0	0	0	0	0.124412	0	0.042087	0
INSM1	0	0	0	0	0	0	0	0
INSM2	0.040801	0	0	0	0	0	0.020706	0.020691
INSR	6.23842	5.333229	6.289976	6.60823	6.808644	5.720067	5.964253	6.935556

INSRR	0.0164	0	0.005399	0	0	0	0	0.005544
INSYN1	0.579574	1.131395	0.558392	1.144401	1.088608	0.711626	1.305666	0.960591
INSYN2	0.122937	0.21378	0.29717	0.188032	0.223884	0.240163	0.255148	0.13027
INTS1	5.737402	5.463215	4.502972	5.274096	5.202795	4.555009	4.732821	6.088517
INTS10	19.44856	20.25464	18.40084	19.48922	19.51905	19.63845	20.55657	20.38026
INTS11	32.05851	37.15505	30.3481	34.09857	29.5438	33.10195	35.02014	31.99898
INTS12	10.43013	12.72943	10.96061	12.00085	10.92957	11.48991	11.91532	10.31883
INTS13	8.754117	9.926176	8.334985	9.131831	9.281672	8.944084	9.035394	8.944298
INTS14	6.074969	6.154697	4.863367	5.561866	5.666728	5.370141	6.027724	6.031827
INTS2	6.89838	6.721691	6.101128	5.114814	6.452849	5.663652	6.456123	6.86797
INTS3	20.54063	18.44926	19.17981	17.36277	19.6407	18.44669	19.06063	19.41617
INTS4	5.632179	4.861942	5.273066	4.846134	5.108102	5.11992	5.012974	5.202728
INTS5	5.227047	5.582603	4.89916	4.978312	6.12047	5.366456	5.235759	5.484267
INTS6	14.8347	15.65655	13.10518	13.95042	15.98087	13.2499	12.98152	15.30375
INTS7	8.125886	8.599756	8.441163	8.644586	7.995113	8.400366	8.548788	8.876558
INTS8	21.42467	21.09153	24.03024	17.95693	21.2253	22.05767	19.78373	23.64594
INTS9	7.467127	7.005123	6.312138	6.364727	6.674042	6.314023	6.732474	6.41142
INTU	0.933757	0.643924	0.565672	0.667408	1.012355	0.726828	0.653081	0.811757
INVS	4.284697	4.052533	3.004631	3.860445	4.144751	3.431819	3.726078	3.8995
IP6K1	18.03619	16.74246	13.72451	15.43946	17.60524	14.66986	15.6047	16.02657
IP6K2	33.74036	29.19812	29.54937	31.35098	29.51066	28.96852	30.30618	34.04245
IP6K3	0.01183	0	0.035047	0.024246	0	0	0	0.011998
IPCEF1	0.592363	0.760889	0.396952	0.574517	0.825184	0.298205	0.569039	1.555632
IPMK	3.08846	2.756493	2.850276	2.642178	2.973946	2.855056	3.237413	3.80366
IPO11	9.018081	9.327188	9.142944	8.69589	10.00623	9.628785	8.402106	8.382609
IPO13	14.13329	12.85435	10.80672	12.0221	11.85714	11.81165	11.74446	11.68379
IPO4	9.187229	9.4001	8.449912	10.16885	9.833218	8.473868	8.322116	8.941315
IPO5	61.73324	64.16909	65.73123	60.68832	59.05976	58.95111	67.82701	63.97855
IPO7	53.98758	53.42641	53.2283	51.37687	54.41236	53.20363	55.65065	54.02381
IPO8	14.44843	14.10251	13.25601	13.69558	14.00173	13.03651	12.71956	13.97177
IPO9	19.40859	18.93622	15.99613	18.50877	19.00814	17.84884	18.24285	19.73596
IPP	2.247305	2.849979	2.258102	2.842479	2.546227	2.579113	2.480404	2.550262
IPPK	3.381344	2.883186	2.93629	3.146291	3.228759	3.527682	2.598637	2.281147
IQANK1	1.891384	1.848971	0.90114	1.836222	1.427018	1.486694	1.734515	1.463973
IQCA1	0.020302	0.041136	0.006683	0.020804	0.006768	0.040881	0.013737	0.006864
IQCB1	5.194109	3.085607	4.290035	6.205865	4.262081	4.361956	3.366904	3.280559
IQCC	7.206406	8.360277	7.52963	7.681626	7.175162	9.069546	7.951255	8.695744
IQCD	1.593689	1.126859	1.032827	0.95268	1.029391	1.220147	1.196346	0.841863
IQCE	3.425482	3.39753	3.216977	3.426753	3.070857	3.337785	3.160343	3.464514
IQCF1	0	0.026955	0	0.027265	0.079827	0.080362	0	0
IQCF3	0	0.036331	0.017707	0	0	0	0	0
IQCG	0.165862	0.420096	0.397788	0.33994	0.319918	0.244528	0.336699	0.336446
IQCH	0.02648	0	0.00523	0.010854	0.010593	0.010664	0.010751	0.037599
IQCJ	0.545243	0.368266	0.119655	0.18625	0.484724	0.243986	0.307457	0.12289
IQCK	0.131366	0.26618	0.25135	0.311309	0.147806	0.148796	0.291674	0.174872
IQCN	0.113875	0.115369	0.053268	0.079844	0.07193	0.096549	0.158166	0.04863
IQGAP1	82.37427	75.81746	78.57357	78.6372	76.8618	71.71988	79.20772	90.30718
IQGAP2	6.27799	10.7101	10.11463	11.04783	6.772528	8.032888	10.41107	8.380236
IQGAP3	1.761891	1.605542	1.46098	1.383588	1.364684	1.258138	1.691128	1.903514
IQSEC1	6.135025	5.481731	6.058581	6.653696	6.256551	5.894785	6.419594	5.951253
IQSEC2	3.652062	3.169255	3.44101	3.484843	3.960904	3.261087	3.269314	3.367044
IQSEC3	0.004241	0	0	0.008692	0.021207	0.021349	0.008609	0.012903
IQUB	0.022418	0.130597	0.071951	0.109124	0.050448	0.203142	0.068264	0.045475
IRAK1	8.009183	6.626015	7.083724	8.655987	7.971144	8.063898	8.034148	7.132565
IRAK1BP1	5.140867	3.298613	4.540925	3.336532	3.684764	3.968257	4.058429	4.605745
IRAK2	2.1341	1.514472	1.612196	2.942825	1.632748	2.148679	1.557205	1.685701
IRAK3	2.064201	2.221259	1.497189	2.103381	2.309405	1.726048	1.964956	2.755964
IRAK4	4.450843	4.762833	4.277172	4.628003	4.19021	4.218291	5.73261	5.89385

IREB2	7.291839	7.830543	7.808727	7.231815	8.058099	7.671845	7.882029	8.397607
IRF1	7.067264	8.382888	6.426396	7.869752	11.35457	8.172308	6.729382	9.279199
IRF2	15.22116	14.45222	13.71529	15.15281	14.60493	13.13723	14.08687	15.86885
IRF2BP1	10.76157	10.61498	9.370583	11.84331	10.7175	10.24013	10.96913	10.83409
IRF2BP2	37.3305	34.95123	32.92411	34.45111	34.4245	33.74275	34.26416	36.77486
IRF2BPL	17.26223	16.23343	17.55757	20.22212	16.76168	16.42489	15.27173	16.71802
IRF3	8.479531	9.044157	9.18033	10.0423	8.849837	9.07809	8.708915	11.01693
IRF4	0.505977	0.675155	0.365614	0.822028	0.78992	0.397607	0.463463	0.69467
IRF5	3.037364	3.585304	3.569519	4.082258	3.965171	3.22007	2.948466	3.483672
IRF6	66.60614	57.87417	63.74402	63.46446	64.90835	63.92709	67.2483	61.08157
IRF7	1.454186	0.665348	1.003702	1.842728	0.969582	1.385404	1.396644	0.919822
IRF8	2.556088	2.887442	1.994149	2.737276	2.415815	1.852956	2.892789	2.34619
IRF9	8.114844	8.03774	7.148726	7.294426	8.498528	7.02482	8.359196	7.913812
IRGQ	14.39958	13.66007	12.01698	13.55148	13.2534	12.03736	13.94846	12.90047
IRS1	3.901586	4.081366	3.652445	3.6861	3.90209	3.475543	4.180934	3.938739
IRS2	11.704	10.54555	10.39234	9.429914	8.432198	9.686172	10.12244	9.489342
IRS4	0.044498	0.015027	0.058591	0.0304	0.014835	0.014934	0.045165	0.022566
IRX1	23.15229	23.97115	20.99272	25.47799	23.30935	22.86069	23.45267	24.65363
IRX2	35.06309	34.62741	30.49294	31.81168	30.59868	31.36165	30.82628	31.69989
IRX3	5.072296	7.07003	5.78545	6.919548	6.397735	6.256281	5.826086	6.444282
IRX4	10.08183	8.037061	6.879156	10.18475	7.486234	7.013541	7.706601	9.825781
IRX5	6.517294	7.705099	6.046341	6.164981	6.731496	6.830304	6.171163	6.599253
IRX6	3.435658	1.748109	3.242056	2.926144	2.24492	2.874921	2.681264	4.49122
ISCA1	31.98419	29.52333	34.01442	29.14965	29.81657	29.3158	31.01489	33.25475
ISCA2	5.219012	5.425561	5.100154	5.432074	6.82784	6.393405	5.435608	5.514438
ISCU	37.31889	43.48531	45.85855	40.08503	42.50462	41.64522	38.89807	41.06687
ISG15	2.707149	2.463761	1.314053	1.363593	3.395846	3.280011	3.213477	3.117983
ISG20	0.80614	0.914726	1.84694	1.586127	1.160991	1.071374	0.392751	1.079252
ISG20L2	16.03887	15.2789	14.43162	14.30532	16.91717	16.91293	16.29131	15.50892
ISL1	0	0	0	0	0	0	0.013502	0
ISL2	0	0.046909	0.022862	0	0	0.023309	0.046996	0
ISLR	12.62301	11.6355	13.70218	11.86257	12.43112	8.617991	12.32713	17.44354
ISLR2	0.013512	0.020533	0.020015	0.034616	0.06081	0.013604	0.006857	0.006852
ISM1	38.73183	39.863	40.14517	46.01353	33.01962	41.80903	45.09313	40.42089
ISM2	0.014487	0.102741	0.04292	0.07423	0.028978	0.058344	0.073522	0.117547
ISOC1	29.52142	33.07272	31.44493	30.49194	30.37173	33.8664	27.78691	26.58193
ISOC2	11.82488	11.12828	15.42721	13.59082	13.26402	12.45179	10.37809	11.05796
IST1	51.12412	51.35327	51.55238	50.19138	52.26697	50.90723	52.81116	51.08373
ISX	0	0	0	0	0	0	0	0
ISYNA1	9.645668	10.01233	7.777308	10.52738	10.20454	8.715145	9.012218	10.43329
ITCH	20.93267	21.74057	21.27818	19.85571	21.10644	22.74477	20.50327	21.23955
ITFG1	23.02885	26.78816	25.97196	25.81678	24.30536	24.30065	28.78069	25.8215
ITFG2	2.432904	2.297603	2.452201	2.426978	2.591127	2.167721	1.981346	2.380192
ITGA1	6.647128	5.424299	6.297577	5.99368	5.875855	5.970581	6.47997	6.829026
ITGA10	0.83886	1.019843	0.624856	0.697536	0.613646	0.48745	0.642231	0.952898
ITGA11	5.239454	2.563869	3.616745	2.88328	3.102158	2.326384	2.930243	3.677317
ITGA2	10.13207	10.04513	7.961787	8.543967	8.528476	8.788561	10.2093	11.25126
ITGA2B	0.741973	0.768171	0.449264	0.738152	0.574155	0.703419	0.522224	0.582254
ITGA3	13.54143	12.23442	10.43538	13.88674	10.39002	10.33345	11.99285	12.82967
ITGA4	2.282035	2.589423	1.695835	2.940742	2.573328	2.234441	2.594218	4.426523
ITGA5	6.248865	5.741495	6.997247	7.696439	10.68851	6.627192	7.244893	10.29565
ITGA6	50.49529	47.01087	48.86173	51.69964	47.04238	47.60602	49.49473	53.91041
ITGA7	13.59457	16.21068	14.49444	18.53604	14.78149	14.35364	14.4762	14.35548
ITGA8	3.912159	4.484639	3.047856	3.051532	3.105589	2.63701	3.635548	4.355543
ITGA9	2.123316	1.835274	1.517657	1.848764	2.234968	1.293158	1.861279	3.47127
ITGAD	1.006909	0.909241	0.864664	0.628083	0.401356	0.315891	0.488789	0.333014
ITGAE	1.47453	1.859174	1.748452	1.477966	1.539307	1.511695	1.573119	1.692013
ITGAL	1.374263	1.670759	0.791665	1.326153	1.695144	0.720652	1.261202	1.980396

ITGAM	1.152686	0.789508	1.05816	0.790265	1.071649	0.351437	1.153498	2.436987
ITGAV	20.51978	17.92322	20.19238	18.61892	18.07182	17.25384	16.73035	21.53302
ITGAX	0.982076	0.742146	0.453122	0.544447	0.853389	0.356611	0.555597	1.159344
ITGB1	135.9506	150.1527	130.4452	138.3139	134.1739	122.9675	140.7596	147.6798
ITGB1BP1	22.4209	23.36415	22.82281	24.84467	23.23697	22.54926	22.58216	23.03994
ITGB1BP2	0.021663	0.021948	0.064181	0.0444	0.064999	0.021811	0.087954	0.087887
ITGB2	2.882716	4.267624	3.078513	4.259432	4.883372	2.171182	3.935299	7.626691
ITGB3	1.213622	0.990471	1.015396	0.803213	1.559369	0.874007	1.069294	1.67539
ITGB3BP	3.838557	6.126403	5.066668	5.111422	3.200463	5.142956	4.605217	4.495086
ITGB4	29.62399	26.69534	20.90244	35.21621	30.28183	25.97765	29.24356	29.85064
ITGB5	40.56041	36.82251	42.42187	38.96788	35.18384	37.6478	38.32606	35.76392
ITGB6	5.403045	5.102759	4.227358	5.460837	4.313718	5.267185	5.2346	5.367069
ITGB7	1.575327	2.046021	1.407779	2.540613	1.461885	1.383277	1.693321	2.394009
ITGB8	3.599935	3.209788	3.364863	2.98804	3.142047	2.701678	2.82472	3.732021
ITIH2	0	0	0	0.009638	0	0.009469	0.019092	0.019078
ITIH3	0	0	0	0.008866	0.017306	0	0.017564	0
ITIH4	2.149255	3.018754	1.286349	4.83881	3.549986	2.483611	2.181494	2.221136
ITIH5	15.26492	15.66651	12.72574	11.21263	12.91934	11.94271	14.76695	19.44291
ITK	2.612406	3.919138	2.027915	2.355347	3.083791	2.516443	4.608416	3.044487
ITLN2	0.023151	0.117273	0.068587	0	0.023154	0	0.187983	0
ITM2B	1311.647	1427.1	1503.038	1417.15	1397.997	1406.908	1480.637	1566.422
ITM2C	68.49888	90.5159	68.12489	95.53714	94.87021	70.08427	92.09164	93.88665
ITPA	24.57236	23.92061	25.03397	24.23752	23.73657	24.78142	23.5288	21.83024
ITPK1	3.703317	3.462454	3.749416	4.369374	4.335749	3.269454	3.641751	3.778327
ITPKA	1.036636	1.158353	1.339868	0.703002	0.94529	0.690695	0.696299	0.618466
ITPKB	19.77076	20.61678	18.22322	19.96725	19.20095	15.993	19.59451	19.37728
ITPKC	16.72643	13.24187	11.71204	14.20712	13.61192	12.62435	15.90638	14.86575
ITPR1	3.016966	3.197323	2.791763	2.882485	3.839756	3.397289	3.447876	3.810514
ITPR3	23.30507	17.83243	15.55525	20.58041	20.04386	17.2052	18.56185	18.74623
ITPRID1	0.200127	0.214339	0.06776	0.140629	0.182997	0.236036	0.145092	0.347958
ITPRID2	40.65641	39.48416	33.58751	38.79351	36.20482	42.36945	38.07615	39.32256
ITPRIP	3.47029	3.422926	3.508078	3.857962	3.359713	3.333633	3.458658	3.705711
ITPRIPL1	6.325629	5.432053	5.210773	5.62938	5.404969	5.2541	5.732882	6.21936
ITPRIPL2	3.766911	4.063894	3.949205	3.989588	5.046276	3.800845	4.41036	4.741594
ITSN1	7.605022	6.985852	7.028606	6.714437	7.461473	6.196772	7.038078	9.338649
ITSN2	12.93095	13.63714	13.05233	13.09844	12.4852	11.75382	12.45462	12.04089
IVD	9.551789	12.92916	10.6375	13.68069	12.70275	11.19375	11.60071	13.3119
IVL	107.2716	52.10429	98.92045	97.70933	88.54305	107.0459	82.74199	97.56324
IVNS1ABP	43.33371	42.23081	35.39484	40.76415	39.80646	42.61036	40.38311	45.29695
IWS1	17.24153	16.66419	14.94356	14.20278	15.61354	15.31447	15.29715	15.04694
IYD	0	0	0	0	0	0	0	0
IZUMO1	2.06577	1.938993	2.980045	2.770701	2.491398	3.282952	1.644515	1.838416
IZUMO2	0	0	0.03585	0.037202	0	0	0	0.036819
IZUMO4	5.543012	6.510577	5.925094	6.959945	5.665569	6.071506	5.904374	4.66434
JADE1	8.923325	8.559816	7.8986	8.575571	10.75113	8.019466	8.421584	9.136961
JADE2	11.57872	12.55536	10.09756	12.2893	11.96332	9.71193	11.43429	14.41896
JADE3	2.884514	3.171841	3.126482	3.490681	3.271884	2.67991	3.368139	2.895845
JAG1	49.00038	42.30222	40.09821	42.97349	41.97112	39.85859	39.48574	43.01295
JAG2	23.70646	20.68981	17.57234	23.29034	20.32967	19.01587	20.84353	20.16795
JAGN1	19.31316	20.92722	17.3042	20.35953	21.44763	25.30439	22.06945	18.24763
JAK1	64.42675	70.01136	66.95147	64.72143	64.13272	61.3637	69.18697	68.35995
JAK2	7.929655	7.725366	7.806804	7.743476	8.498896	8.429188	7.163006	9.108566
JAK3	1.573712	1.569931	0.803847	1.699198	2.152827	0.892399	1.236238	2.219884
JAKMIP1	0.05196	0.201794	0.11973	0.159741	0.103934	0.034877	0.210958	0.0527
JAKMIP2	0	0.00809	0.055202	0.032733	0.095838	0.02412	0.040526	0.016198
JAKMIP3	0.048608	0.035815	0.034911	0.063397	0.039775	0.106778	0.035881	0.233053
JAM2	8.124349	7.520335	8.144171	9.826013	10.25721	10.59335	7.845308	7.072728
JAM3	3.631995	3.86942	3.433942	3.838794	4.259594	3.853586	4.967649	4.592235

JAML	3.198441	4.491105	4.000878	4.979754	3.872298	2.666627	5.410696	5.634269
JARID2	10.37626	9.682279	10.51528	10.49186	10.59326	10.84228	9.056737	11.48189
JAZF1	4.123484	4.232449	4.989888	4.475278	4.909115	3.988132	4.524193	5.86604
JCAD	3.516773	3.161709	3.244361	3.332379	3.260618	3.655923	3.204363	3.091636
JCHAIN	6.380837	50.5552	7.903364	26.99787	32.86015	26.22038	35.32464	14.28156
JDP2	2.233138	3.006472	2.789943	2.756896	2.308374	2.467197	3.065282	3.116177
JHY	0	0	0	0	0	0.009888	0	0.009961
JKAMP	8.552993	8.692534	8.792259	7.481182	8.810048	10.36083	7.943037	8.715733
JMJD1C	11.811	13.17804	11.78416	12.29938	12.43847	11.79131	12.27206	13.45933
JMJD4	6.481461	6.925539	6.299102	6.226151	5.659149	4.943218	6.207399	6.17374
JMJD6	4.066495	3.159946	3.024514	2.828884	4.220687	3.02172	3.06183	3.594552
JMJD7	5.19503	6.102272	4.702774	4.55216	4.405051	5.003106	5.521319	4.963536
JMJD8	25.00365	17.58103	21.35478	23.32841	18.30935	21.89719	23.21121	20.46011
JMY	23.5559	20.50373	20.01113	25.33476	23.65457	23.73608	21.83889	22.96675
JOSD1	25.02164	23.33432	24.4601	25.52912	26.97256	25.33687	26.80886	23.89005
JOSD2	8.674252	8.769474	10.65327	9.303439	11.3221	9.103574	9.028206	9.338274
JPH1	0.324042	0.357607	0.22286	0.30835	0.260425	0.337908	0.199691	0.61036
JPH2	1.355238	2.468865	1.897569	2.347281	2.189024	1.94745	2.350734	1.439063
JPH4	0.222894	0.14517	0.172949	0.220258	0.143308	0.088164	0.088879	0.121108
JPT1	35.09146	28.9948	31.33586	27.68464	33.54933	28.21882	30.34686	33.32543
JPT2	29.98005	23.49449	23.57476	25.10582	29.22803	24.06004	25.61488	26.33722
JRKL	6.759785	6.907731	6.726075	6.919738	6.351365	6.945763	6.608987	7.537911
JSRP1	0.022143	0.022434	0	0	0.022146	0	0	0
JTB	24.17246	29.64543	30.09474	27.83351	28.93968	29.13362	30.51115	23.85639
JUN	45.95647	54.4054	43.6367	49.41809	47.76361	39.23066	59.14062	45.2676
JUNB	94.83299	101.2471	84.72936	96.44383	95.46064	87.42715	90.01008	84.05167
JUND	42.36611	46.26912	41.65698	54.85717	59.10607	43.91827	54.22549	43.86894
JUP	222.0203	176.1503	191.2443	184.6388	191.8607	167.9211	184.7271	184.3379
K33	1532.541	1479.32	1980.141	1759.992	1565.101	2075.977	1621.012	1702.9
K38	7.689149	8.092262	3.92759	3.413366	3.679336	8.676018	4.003163	6.33637
KANK1	42.66262	28.85547	30.71206	30.65132	34.13895	29.71716	31.05264	33.10388
KANK2	20.5358	17.41972	17.35997	20.21551	20.61099	19.8504	20.8042	18.66102
KANK3	2.446472	2.63349	2.415988	2.702937	3.142593	3.248325	2.902203	3.45831
KANK4	0.998408	1.155037	1.105883	1.486317	1.841897	0.821843	1.150328	1.299987
KANSL1	14.50794	12.81896	10.2176	12.30047	12.21305	10.40222	12.30402	13.4367
KANSL1L	3.506393	3.229465	3.392749	3.243904	3.714955	3.396622	3.334306	3.920022
KANSL2	23.42711	25.11464	18.68076	20.68878	22.85178	20.76653	21.66545	23.26533
KANSL3	10.05628	8.314559	7.183246	8.473596	9.09559	7.818183	8.208442	8.909857
KARS	265.7895	298.6987	273.4288	262.1378	259.6074	237.5258	255.2827	245.5124
KAT14	11.84729	10.65605	10.99742	10.78331	10.16145	12.13881	10.10024	10.84216
KAT2A	13.52972	12.51418	11.28002	11.9412	12.84958	10.43949	11.1533	14.0097
KAT2B	18.59813	21.51437	20.34389	20.42803	21.33907	20.9894	16.88689	20.85512
KAT5	19.00453	18.3799	17.2707	19.28583	18.13183	18.37743	21.46625	17.26257
KAT6A	14.79925	12.91779	11.37642	12.26498	13.58264	12.46833	12.33261	14.37363
KAT6B	6.045279	5.83182	5.217833	5.320472	5.447575	4.878162	4.897033	6.245136
KAT7	25.2217	25.65625	23.69622	24.89574	23.61301	22.29099	23.19803	24.31287
KAT8	18.65045	17.71939	16.80548	19.4305	18.51789	17.7588	17.1495	16.7944
KATNA1	28.71709	28.41882	27.96441	27.57929	27.79796	27.86038	30.28212	24.78976
KATNAL1	2.955223	2.943506	2.524592	2.820449	3.041068	2.649192	2.605634	3.177852
KATNAL2	0	0	0	0	0	0	0	0
KATNB1	7.146003	7.707638	6.948751	7.651866	7.593121	6.777159	6.855976	7.00958
KATNBL1	4.250386	4.668793	3.81082	4.210481	4.075678	4.951367	3.757845	4.4319
KAZALD1	1.030069	1.514057	1.892722	1.687204	1.376417	1.445147	2.262436	1.755493
KBTBD11	1.555852	2.157648	1.716052	1.718629	2.247183	1.521724	2.165745	2.184608
KBTBD12	1.041498	0.576351	0.561797	1.246674	0.831555	0.449406	0.81727	0.21304
KBTBD13	0	0	0	0	0	0	0	0
KBTBD2	19.85595	20.03214	17.30517	18.3068	18.05595	17.3535	18.37054	18.08788
KBTBD3	1.289452	1.388023	1.854367	1.337909	1.434701	1.517342	1.701432	1.389546

KBTBD4	3.294909	3.487621	2.789018	2.915763	3.463966	2.942529	2.781002	3.142303
KBTBD6	0.784481	0.902639	0.78024	1.010633	1.070395	0.812407	0.699561	0.875214
KBTBD8	2.613195	2.997273	2.856921	2.867027	3.381519	2.852798	2.954488	3.18772
KCMF1	21.56791	21.05474	20.8141	20.69274	20.91867	21.31062	20.10567	19.71009
KCNA1	0.129767	0.201314	0.156183	0.083113	0.182509	0.114322	0.193455	0.057581
KCNA10	0	0	0	0	0	0	0	0
KCNA2	0.057502	0.123796	0.099375	0.058926	0.071887	0.108553	0.145912	0.087481
KCNA3	1.073761	1.612915	0.88263	1.40248	1.607746	0.887374	1.445807	1.384915
KCNA4	0.00812	0.024679	0.028065	0.008321	0.012181	0	0.004121	0.012353
KCNA5	0.093056	0.115228	0.071475	0.095361	0.196477	0.062461	0.073463	0.125841
KCNA6	0.834765	1.044713	0.795268	1.132203	1.213021	0.766308	0.583132	0.478107
KCNA7	0.025337	0	0	0.01731	0.008447	0.034014	0.017145	0.017132
KCNAB1	0.12795	0.318673	0.273772	0.40975	0.186619	0.257649	0.519479	0.297394
KCNAB2	1.542604	2.074699	1.603211	2.215902	2.155882	1.275069	1.85291	2.158961
KCNAB3	0.919865	0.540666	0.637962	0.870692	0.912961	0.685774	0.734101	0.754914
KCNB1	0.029049	0.098102	0.033469	0.03225	0.096844	0.070682	0.066342	0.19642
KCNB2	0.014597	0.014788	0.021622	0.007479	0	0.007348	0.029631	0.037011
KCNC1	0.023496	0.017853	0.005801	0.024078	0	0.041398	0.011924	0
KCNC2	0.009102	0.05533	0.035955	0.018655	0	0.009164	0.046193	0
KCNC3	0.08774	0.028284	0.051201	0.061305	0.055842	0.068263	0.048576	0.044495
KCNC4	4.412429	3.921422	3.186005	4.626883	3.345669	3.566456	3.699544	4.37533
KCND1	0.407386	0.418546	0.368312	0.470397	0.545165	0.502602	0.378554	0.290976
KCND2	0.116598	0.270008	0.126112	0.142246	0.161038	0.167707	0.163432	0.106996
KCND3	1.09093	1.842078	1.021225	1.385795	0.602362	1.006851	1.453323	1.175613
KCNE1	0.738568	0.335427	0.276656	0.39148	0.178298	0.230776	1.163244	0.64576
KCNE2	0	0	0	0	0	0	0	0
KCNE3	1.513926	1.745782	4.606736	0.971218	2.228097	1.251635	2.186269	2.284491
KCNE4	0.378508	0.445661	0.353587	0.817702	0.900353	0.69009	0.996807	0.632911
KCNE5	0.020371	0.061914	0	0.04175	0	0.02051	0.020676	0.041321
KCNF1	0.029447	0.029833	0.02908	0.030176	0	0	0.074721	0.059732
KCNG1	0.024802	0.050255	0	0.088958	0.074416	0.037457	0.037761	0.062888
KCNG2	0.427612	0.334764	0.3647	0.537796	0.272152	0.371824	0.414298	0.374559
KCNG3	0.051545	0.039166	0.012726	0.066028	0.051552	0.012974	0.026159	0.02614
KCNG4	0.987452	1.552363	1.176903	0.959572	1.396234	0.548523	1.192349	1.32959
KCNH1	0.073559	0.139733	0.127124	0.141339	0.082764	0.055546	0.102661	0.074606
KCNH2	0.041537	0.070137	0.129896	0.099321	0.11078	0.041821	0.175668	0.126386
KCNH3	0.045721	0.069482	0.037626	0.062472	0.038106	0.023017	0.038673	0.069559
KCNH4	0.006269	0.006351	0	0	0.012539	0.006311	0	0.006358
KCNH5	0.009433	0.057344	0.009316	0.009667	0.018869	0.009498	0.009575	0
KCNH6	0	0	0	0.008352	0	0	0	0
KCNH8	0.303802	0.222292	0.127785	0.305562	0.106907	0.056644	0.125628	0.1883
KCNIP1	0.647214	1.978628	0.706428	1.954829	0.692722	1.223245	1.463669	0.932818
KCNIP2	0.747819	0.70946	0.525061	1.009977	0.622542	0.483092	0.570374	0.727775
KCNIP3	5.043007	3.66046	3.530366	4.992078	4.233241	4.981476	4.170395	3.761165
KCNIP4	0.096886	0.032719	0.053155	0.077222	0.032299	0.043354	0.043706	0.06551
KCNJ10	0.326826	0.075994	0.148149	0.164716	0.187547	0.231959	0.05982	0.043473
KCNJ11	0.641997	0.232987	0.160865	0.304401	0.383331	0.279778	0.223693	0.310991
KCNJ12	0.056464	0.068646	0.022304	0.09258	0.045177	0.017055	0.017193	0.022907
KCNJ14	0.19895	0.286429	0.423964	0.2468	0.230393	0.084341	0.223191	0.116821
KCNJ15	3.539371	3.915897	4.23381	3.991264	4.384055	3.235524	3.404571	4.107942
KCNJ16	0.011469	0.145239	0.011326	0.023505	0.028675	0.063508	0.029102	0.058159
KCNJ2	0.315156	0.463814	0.47831	0.547336	0.537493	0.414172	0.656603	0.713307
KCNJ3	0	0.0599	0.052548	0.018176	0.035479	0.035717	0.072013	0.011993
KCNJ4	0.02857	0.048242	0	0.048796	0.019049	0.019177	0.067663	0
KCNJ5	0	0.005408	0	0	0	0	0	0
KCNJ8	3.780316	4.610934	2.400968	2.537061	2.994991	3.224026	3.89721	4.91671
KCNJ9	0.079824	0.764603	0.315317	0.423879	0.036288	0.621035	0.147311	0.375361
KCNK1	8.069603	6.525676	7.136253	7.643691	6.296558	7.261021	8.854754	7.048975

KCNK10	0.34199	0.145885	0.142201	0.184453	0.072007	0.244653	0.17356	0.33773
KCNK12	0.035273	0.008934	0.013063	0.013555	0.008819	0.013318	0.008951	0.004472
KCNK13	0.294004	0.23829	0.333892	0.331415	0.27934	0.118405	0.283493	0.55165
KCNK15	0.173846	0.264191	0.114453	0.207844	0.086934	0.087517	0.176453	0.029387
KCNK17	0.020934	0.021209	0	0	0.041874	0	0.021248	0
KCNK18	0.239663	0.069374	0.050716	0.245599	0.017121	0.103414	0.104253	0.052087
KCNK2	4.650454	4.384543	5.838803	4.682972	4.979568	4.937512	4.304926	4.149722
KCNK3	0.046301	0.023455	0.045724	0	0.069461	0.046618	0.023498	0.093921
KCNK4	0.399537	0.45948	0.287921	0.265578	0.766778	0.456626	0.372649	0.449032
KCNK5	2.567594	1.908688	1.546482	2.59861	2.178365	1.664731	2.614177	2.17684
KCNK6	6.459038	6.637826	5.847011	7.322778	7.146698	7.007719	6.141469	5.308558
KCNK7	19.31474	16.85456	14.9729	17.60232	15.53243	18.75393	16.76106	13.33395
KCNK9	0.011403	0.051988	0	0.011686	0.028512	0	0.005787	0.028914
KCNMA1	1.179088	2.416807	1.280629	2.1689	1.679525	2.334081	3.300197	1.68616
KCNMB1	3.200961	4.623641	4.457699	4.078274	3.663242	3.633089	4.356476	4.174108
KCNMB2	0	0.015886	0	0	0.015682	0	0	0
KCNMB3	0.361817	0.471299	0.382831	0.423748	0.387712	0.286227	0.209854	0.183484
KCNMB4	6.001061	6.278595	6.166812	7.03022	5.954472	5.810457	6.283354	7.266734
KCNN1	0.548807	0.444808	0.216788	0.604581	0.63121	0.221023	0.529187	0.403549
KCNN2	0.078539	0.248654	0.106645	0.120726	0.284739	0.256994	0.189327	0.308669
KCNN3	0.593394	0.495631	0.362336	0.751992	0.924183	0.401339	0.604594	0.638595
KCNN4	0.158697	0.194628	0.065987	0.094153	0.142011	0.168191	0.067822	0.135542
KCNQ1	0.807467	0.615846	0.671971	0.660115	0.789424	0.758175	0.791953	0.671733
KCNQ2	0.059362	0.060141	0.043967	0.298079	0	0.095628	0.045189	0.015052
KCNQ3	0.031311	0.039652	0.02319	0.066846	0.086116	0.002627	0.023835	0.010585
KCNQ4	0.351501	0.160601	0.12932	0.162447	0.365333	0.173482	0.139911	0.195728
KCNQ5	0.168144	0.230204	0.116683	0.218879	0.136351	0.059481	0.295206	0.170538
KCNRG	0.085073	0.215475	0.12602	0.108976	0.340338	0.321205	0.215874	0.215711
KCNS2	0	0	0	0.005936	0.040552	0.011664	0	0.005875
KCNS3	0.396276	0.279288	0.391339	0.573826	0.396327	0.251533	0.559611	0.436867
KCNT1	0.229838	0.332033	0.210161	0.353297	0.353315	0.274262	0.237606	0.358299
KCNT2	0.134844	0.109291	0.142042	0.092123	0.197797	0.149342	0.08212	0.141323
KCNU1	0	0	0	0.008188	0.007991	0.032179	0.03244	0.591577
KCNV1	0	0	0	0	0	0.005239	0.03697	0
KCNV2	0	0	0.014464	0	0	0.014746	0	0.014855
KCP	0.372018	0.303614	0.295947	0.333579	0.222206	0.275717	0.471997	0.44544
KCTD1	17.68643	17.38493	17.66537	18.88119	16.97979	17.98568	14.3444	18.24276
KCTD10	37.34247	38.85978	36.73746	36.81762	40.55455	35.04148	38.51689	38.24873
KCTD11	15.02074	14.40865	13.68327	15.60881	15.877	14.93348	14.76187	14.21068
KCTD12	16.59854	22.17611	18.64237	17.84447	23.28307	15.13152	18.29677	21.57848
KCTD13	5.320389	4.596563	3.400657	4.799923	3.590911	4.863785	5.38363	4.568501
KCTD14	2.075601	1.81609	4.596384	5.865403	2.767826	3.704611	3.894268	4.274089
KCTD15	10.88581	10.4349	9.451693	13.23927	11.74861	10.41114	10.37146	9.965643
KCTD16	0.024039	0.016236	0.007913	0.016423	0.020035	0.012102	0.040666	0.01219
KCTD17	4.177913	4.753401	4.107059	3.813276	4.321225	3.918998	4.105347	3.745118
KCTD18	1.896228	2.039337	2.041341	1.772368	1.825616	1.619658	2.085414	1.864046
KCTD19	0	0	0.055802	0.009651	0	0.037928	0.009559	0.009552
KCTD2	19.74986	20.54627	19.3346	21.77727	19.5974	20.30367	19.06906	19.02987
KCTD20	36.85943	37.67423	38.38327	37.1149	38.64085	40.64051	36.3751	38.18558
KCTD21	9.27146	8.556047	9.956198	8.434583	9.097853	9.150822	8.394485	8.355932
KCTD3	12.68997	15.74315	11.55316	15.18623	14.05447	12.84682	13.69762	12.74498
KCTD4	6.48908	5.265481	7.288367	5.326011	5.528449	6.795553	4.716199	5.647052
KCTD5	19.52614	20.36347	21.05757	21.90369	21.65744	19.0949	20.8669	20.38582
KCTD6	5.24391	5.53478	5.26675	5.082653	4.992912	5.402328	5.157783	4.388224
KCTD7	2.961816	2.525578	2.461801	3.920443	2.493184	2.98205	2.229631	2.002653
KCTD8	0.012789	0.032393	0.119984	0.085189	0.038373	0.012877	0.006491	0.045399
KCTD9	10.96049	12.63477	12.98532	13.07906	11.84607	13.73156	13.79069	13.41469
KDELC1	7.947711	7.758962	6.923706	7.763463	7.122179	7.031225	6.361268	7.921149

KDELR1	33.36422	36.09497	38.58832	37.52457	37.93084	37.40175	37.65135	36.67256
KDELR2	42.10973	44.55145	43.75487	44.73308	47.72986	43.61514	51.29375	48.38263
KDELR3	18.0417	20.04558	20.91198	20.54134	24.6947	20.56591	22.21269	22.61058
KDF1	7.610816	6.577699	6.564982	7.003488	7.28558	6.865252	6.984011	5.986288
KDM1A	44.51784	41.73447	40.84288	38.31191	39.93881	46.22868	39.07744	38.92761
KDM1B	7.907812	7.461903	6.916262	7.304826	8.426526	7.610208	7.24784	7.153829
KDM2A	22.00213	19.58055	18.04764	19.53314	21.43682	19.23583	18.23869	19.9126
KDM2B	8.856149	8.389278	7.650015	8.31037	8.520219	7.668946	7.910102	8.167091
KDM3A	16.52052	14.87564	14.39496	14.96792	15.52988	14.12291	16.15661	15.9347
KDM3B	17.64097	16.99351	15.74899	17.45516	16.26415	15.56715	17.91409	19.0696
KDM4A	18.0478	17.44356	15.26411	18.37022	17.47896	16.39285	17.551	17.19453
KDM4B	12.48422	12.09164	12.34953	13.34546	13.02457	12.31961	12.63397	12.73157
KDM4C	4.37459	4.71123	4.030532	4.458913	4.580424	4.433996	4.493777	4.204911
KDM5A	12.08018	10.73481	8.11318	10.1658	10.91964	8.993188	10.64558	11.83163
KDM5B	35.95257	31.84057	27.83337	32.72275	31.29755	31.65155	32.29177	33.51816
KDM5C	27.50635	24.4386	23.35842	23.23047	26.33522	23.79603	23.41422	25.1199
KDM6A	13.53629	12.5027	11.50919	11.54831	12.47519	11.21241	11.29439	13.36521
KDM6B	7.500119	6.817747	5.988447	7.104159	7.166453	6.684559	6.673921	6.379109
KDM7A	10.54877	8.82553	9.176961	9.157377	11.16773	9.238527	8.747551	9.811161
KDM8	1.767737	1.492447	1.66981	1.824651	1.562984	1.586355	1.391196	2.520456
KDR	17.31992	18.09126	15.17419	19.20133	26.68096	24.20082	19.29972	17.23613
KDSR	16.06359	15.86852	19.40249	15.88784	16.23033	15.7147	20.3375	19.8268
KEAP1	13.53444	12.9413	11.59661	13.48617	12.91031	12.19379	12.83449	12.55103
KEL	0.459404	0.244352	0.612467	0.517862	0.447976	0.242835	0.477952	0.198026
KERA	5.02588	5.782259	5.275714	13.16899	16.02891	4.692645	6.991085	9.701157
KHDC3L	0	0	0	0	0.103218	0	0	0
KHDC4	24.0731	21.68438	18.23404	22.75342	23.69479	22.23337	22.26269	24.42523
KHDRBS1	44.00837	50.44066	46.36534	49.87751	46.59266	48.65448	48.26167	42.22483
KHDRBS2	0.017119	0	0	0.017543	0	0	0	0
KHDRBS3	1.283696	2.032099	1.199789	1.151057	1.295325	1.350165	0.942313	1.697212
KHK	2.074261	2.435195	1.907751	2.244239	2.2526	1.837461	1.91562	1.88709
KHNYN	11.52282	10.16346	9.449348	11.17036	10.47467	10.20236	10.48349	11.17299
KHSRP	43.14326	39.26858	41.98351	42.94128	43.8996	39.55105	37.93309	41.18107
KIAA0040	35.33024	28.57797	24.56229	28.19828	28.51469	27.64786	30.01233	29.83238
KIAA0100	12.60589	11.0371	9.866512	11.43891	12.15401	10.48917	12.03557	12.26795
KIAA0232	7.85917	8.181352	8.064192	8.197557	8.859511	8.112888	7.59155	7.99772
KIAA0319	0.433311	0.452716	0.37888	0.444044	0.514623	0.508983	0.293207	0.297564
KIAA0319L	11.23366	11.48044	10.19191	12.66751	9.954079	11.61893	12.08642	10.89632
KIAA0355	14.85634	14.25692	12.10133	14.02875	15.81674	15.89222	14.24872	14.92952
KIAA0391	4.903125	4.704138	5.600422	5.230403	5.754531	5.376753	5.540295	5.308447
KIAA0513	4.864758	4.233222	4.39186	4.271008	3.917079	4.548899	4.517574	4.585939
KIAA0556	3.184271	3.424006	2.446708	2.909568	3.055799	2.996754	3.274229	3.039873
KIAA0753	4.455703	4.475651	3.17339	3.331998	3.676588	3.037558	3.525392	4.043434
KIAA0754	6.758662	5.052044	4.700435	4.456551	5.231256	4.223393	5.035332	5.769557
KIAA0825	1.153588	1.337103	1.013707	0.77642	1.109738	0.974452	1.180814	1.075814
KIAA0895	0.159221	0.259075	0.204885	0.266998	0.149591	0.199172	0.284041	0.132126
KIAA0895L	0.667402	0.697291	0.813559	0.491577	0.896937	0.51447	0.804428	0.719209
KIAA0930	32.10391	30.3209	32.66373	30.38127	32.04392	34.31518	29.44561	25.57036
KIAA1107	2.188092	2.643289	1.853376	2.102991	2.061195	2.061763	2.252069	2.486084
KIAA1143	5.027994	7.034545	6.549525	7.6552	6.10621	6.918535	6.512928	4.978152
KIAA1147	2.653637	2.943718	2.717511	2.581891	2.611438	2.39833	3.088657	2.824159
KIAA1191	21.84113	22.25189	24.78068	21.68591	23.32546	23.75091	27.62897	23.02186
KIAA1210	0	0	0	0.005483	0.005351	0	0	0
KIAA1211	0.392871	0.475313	0.760884	0.801295	0.629437	0.541487	0.894314	0.611235
KIAA1211L	5.474138	5.372669	4.606736	7.693158	6.238166	5.365798	5.582963	4.951482
KIAA1217	7.247244	6.930095	5.338613	6.056885	6.482113	6.012035	7.341001	6.904084
KIAA1257	0.142856	0.021442	0.005225	0.113862	0.037041	0.085234	0.042963	0.225384
KIAA1324	1.491675	1.781636	2.941471	4.229357	1.720653	1.751377	3.037775	1.875429

KIAA1324L	0.187077	0.177305	0.065555	0.197895	0.470771	0.267343	0.434894	0.612066
KIAA1328	1.831493	1.593754	1.12573	1.292775	1.459303	0.734541	1.295876	1.687995
KIAA1522	6.257563	5.34711	5.85969	6.27897	5.440517	5.86279	5.032223	5.869844
KIAA1549	4.435222	3.395545	2.949768	3.77984	3.570765	2.837783	4.157228	3.863387
KIAA1549L	0.47278	0.457212	0.533587	0.45303	0.300899	0.398735	0.807056	0.498192
KIAA1614	2.609016	2.603294	2.320526	3.158709	2.73334	2.422593	2.16199	3.257688
KIAA1671	3.178502	3.195098	2.685936	2.817686	3.20123	2.56866	2.75559	2.766088
KIAA1755	2.811524	4.176835	3.380348	2.105965	2.162505	4.036648	2.137074	2.391208
KIAA1841	1.766384	1.789566	1.55408	1.832079	1.284809	1.557492	1.830911	2.095547
KIAA1958	0.317733	0.345104	0.265719	0.30507	0.397934	0.377544	0.386418	0.513869
KIAA2012	0.266365	0.134931	0.345248	0.490481	0.129037	0.196948	0.312605	0.168848
KIAA2026	5.734385	5.505766	4.793811	5.31022	6.256142	4.990771	4.778897	5.459771
KIDINS220	29.96504	27.40827	23.60585	27.27422	27.30559	26.50137	27.51557	30.50086
KIF11	10.82343	12.13648	10.51296	10.32617	9.307215	11.14205	12.89295	12.54057
KIF12	0.279999	1.189249	0.14889	0.452474	0.290806	0.50961	0.185823	0.098303
KIF13A	8.505758	7.831868	6.477985	7.473489	8.625862	7.103062	7.815203	8.848743
KIF13B	5.950854	5.201503	5.120561	5.638481	6.279433	5.282806	6.141013	6.212684
KIF14	2.46561	2.147301	1.953819	2.053752	1.739399	1.746753	2.103567	2.219001
KIF15	2.714383	3.20965	2.38187	2.416035	2.253229	2.838339	2.845623	2.796286
KIF16B	2.2535	2.375296	2.225421	2.591683	2.342369	2.365497	2.524523	2.430301
KIF17	0	0.031223	0.018261	0.012633	0.012329	0.018617	0	0.025006
KIF18A	2.273653	2.33712	1.638922	2.125887	1.817498	1.712712	2.41725	1.986207
KIF18B	3.719756	2.846712	2.659806	2.983872	2.875721	2.953626	2.992366	2.805537
KIF19	0.079314	0.062499	0.06092	0.036124	0.035255	0.06211	0.008945	0.071505
KIF1A	0.744631	0.464517	0.398316	0.720683	0.444768	0.520638	0.888765	0.51048
KIF1B	13.16341	12.24831	11.34104	11.89386	12.64603	12.00941	12.06104	11.81736
KIF1BP	12.47723	15.21261	13.74779	12.94815	13.6297	12.25579	13.25982	12.6663
KIF1C	66.68664	53.87391	55.11372	61.25631	59.02517	62.67152	57.8623	56.50061
KIF20A	11.09057	11.45094	10.39702	10.7512	9.035878	10.03392	11.46279	11.53831
KIF20B	4.931492	5.598627	4.576444	4.642584	4.563989	5.392805	5.589834	5.322377
KIF21A	18.68038	18.41166	14.7123	15.31151	15.31535	14.30905	16.94047	15.87433
KIF21B	0.54104	0.40458	0.419806	0.673251	0.544331	0.460432	0.722401	0.862309
KIF22	10.37182	10.00498	7.55275	9.954988	8.655486	9.821252	10.89511	10.01595
KIF23	5.981371	5.921095	5.200426	6.57404	5.380886	5.524211	5.522685	5.989338
KIF24	1.865399	1.85258	1.426055	1.58462	1.603796	1.215026	1.77712	1.846315
KIF25	0.038184	0.019343	0.037709	0.058695	0.076379	0.038445	0	0.077456
KIF26A	2.404003	2.481349	1.69227	2.712011	3.185201	2.937611	2.531826	2.317356
KIF26B	0.607937	0.615915	0.544883	0.571593	0.533769	0.571688	0.710734	0.697989
KIF27	1.608939	1.662333	1.87206	1.67328	1.5733	1.527707	1.463298	2.411412
KIF2A	11.39473	11.65178	11.76226	9.82081	11.42914	11.05257	12.18218	11.07447
KIF2B	0.01366	0	0	0.013998	0	0.013753	0	0.013854
KIF2C	5.738075	4.45259	4.1841	4.999696	4.751069	4.942006	6.68624	5.469172
KIF3A	10.52713	10.24394	8.917422	10.07935	10.35171	9.944783	9.693042	9.928281
KIF3B	8.809453	9.366471	9.910077	8.350591	8.173739	8.802149	8.980471	9.449587
KIF3C	3.871389	3.578419	3.371278	3.061079	3.270281	3.483724	2.948399	2.909678
KIF4A	4.898267	4.691987	4.181119	4.398824	3.79795	4.157868	4.403164	4.637678
KIF5A	0.073514	0.049652	0.091957	0.045201	0.107834	0.049344	0.08954	0.059648
KIF5B	58.7421	58.43008	57.8057	56.83697	57.18945	60.68985	58.6849	58.89464
KIF5C	0.043431	0.100002	0.101375	0.178026	0.209284	0.18286	0.092172	0.212236
KIF6	0.008165	0.008272	0.008063	0.016734	0.008166	0.016441	0.033149	0.024843
KIF7	4.080996	4.240989	2.242517	3.739031	3.754193	2.953508	3.69518	4.319408
KIF9	0.959728	0.724823	0.422188	0.509632	0.628267	0.509496	0.65532	0.451299
KIFAP3	19.08437	21.63502	21.85137	20.53924	20.61304	21.54749	21.05175	19.18617
KIFC1	7.221425	8.199618	6.330977	8.042166	7.222359	7.382046	8.800683	8.955985
KIFC2	1.250236	0.939769	1.123141	1.215079	1.072922	1.226293	0.52397	1.194407
KIFC3	9.819738	8.454354	10.31066	10.06795	9.185359	10.81005	8.356748	7.8387
KIN	4.251299	4.711432	4.121215	3.858707	4.104335	3.817366	3.681018	4.320619
KIRREL1	10.76535	9.380779	8.562378	10.9121	9.498293	9.113491	9.880892	11.32824

KIRREL2	0	0	0.018925	0	0.02875	0.009648	0.009726	0.019437
KIRREL3	0.020007	0.293902	0.345752	0.276779	0.270123	0.694941	0.263986	0.182622
KISS1	0.120495	0.052318	0.016999	0.03528	0.137726	0.034662	0.017472	0.069834
KISS1R	0.566184	0.374096	0.486199	0.983831	0.738596	0.446127	0.424761	0.424441
KIT	3.486723	5.116194	2.977546	4.697147	3.821052	3.046383	4.415712	5.664623
KITLG	15.08867	16.79457	14.77166	16.01705	14.55401	14.01738	14.11216	15.24195
KIZ	5.938614	6.866993	5.876991	4.981556	4.573575	6.471142	5.480877	5.641942
KL	0.010832	0.043896	0.101619	0.0666	0.048749	0.032717	0.120936	0.076901
KLB	0.028855	0.248485	0.021371	0.088709	0.137078	0.007263	0.058575	0.021949
KLC1	28.43377	28.48318	26.45357	27.79259	27.38137	30.33464	27.10454	29.31061
KLC2	4.58021	6.055389	3.89617	5.409331	4.889213	4.657158	5.173642	4.369447
KLC3	72.62416	60.67406	56.70594	73.976	63.83966	77.36087	76.31939	58.30692
KLC4	6.196771	6.034369	5.775487	6.758317	6.015047	6.272513	5.481405	5.712859
KLF1	0.111857	0.07555	0.171832	0.152837	0.285894	0.350378	0.138765	0.226899
KLF10	32.37243	35.50675	30.49122	31.93756	32.90769	29.17189	36.48386	41.24478
KLF11	7.591745	8.458498	4.309386	4.948304	5.971882	5.008804	6.822475	8.238739
KLF12	2.206154	2.023092	1.645826	1.648447	1.999665	1.799827	1.893763	2.483053
KLF13	12.74162	12.75261	10.64118	12.42512	12.41553	11.52833	11.52404	15.5819
KLF14	0	0	0.025905	0.026881	0.026235	0	0.026625	0
KLF15	0.558977	0.566313	0.47234	0.608255	0.622446	0.713648	0.801325	0.759808
KLF16	8.640687	8.357077	8.117014	9.32657	9.523621	9.06467	7.875369	7.611098
KLF17	0	0	0	0	0	0	0	0
KLF2	8.329317	8.419386	7.559611	7.620757	8.653351	7.343918	8.300017	7.937356
KLF3	15.78048	14.64447	17.20303	14.53799	15.45943	15.08129	15.09847	16.09167
KLF4	50.36704	50.80417	49.33145	60.17115	54.56334	53.4005	60.28014	50.76246
KLF5	35.0398	30.78141	29.15207	31.15427	26.71237	30.44078	27.74986	30.1189
KLF6	43.98297	42.47357	43.24943	39.15308	42.08311	40.26443	48.27215	45.07179
KLF7	6.270587	5.792677	4.535318	5.300491	6.440375	4.751499	5.105747	5.374523
KLF8	0.096136	0.166967	0.176313	0.197034	0.247238	0.16593	0.139396	0.306441
KLF9	14.01843	16.03209	13.72262	11.62104	11.45066	13.38002	16.11711	22.47093
KLHDC1	2.386971	2.697832	2.192477	2.551306	2.464289	2.383897	2.416263	2.39492
KLHDC10	13.11036	12.00717	10.73227	11.0415	12.07036	10.91515	11.88994	11.67421
KLHDC2	42.44564	42.60863	39.38799	34.03033	39.71182	40.59801	41.74988	44.9402
KLHDC3	31.1278	31.60942	30.76963	30.49399	30.50041	31.68552	30.56333	28.412
KLHDC4	9.403218	8.725075	7.928367	9.290567	8.885568	7.482555	7.727565	8.05061
KLHDC7A	0.094982	0.312743	0.110548	0.29548	0.179811	0.3108	0.302993	0.123858
KLHDC8A	3.265075	2.849598	3.350648	6.228308	3.108123	3.277478	3.543639	2.703107
KLHDC8B	1.274791	1.737856	0.94418	1.239129	1.828062	1.330687	0.979949	1.235897
KLHDC9	1.070502	1.084551	0.462509	0.411382	0.75837	1.167633	0.565916	0.701208
KLHL1	0	0	0	0	0	0.008352	0	0
KLHL11	8.350434	7.664166	7.385426	6.928649	7.979124	7.726287	7.793579	7.331777
KLHL12	9.390555	8.127069	7.970991	9.053594	8.653371	8.385624	9.068315	8.539841
KLHL13	1.356492	0.976309	0.842548	1.358643	1.012897	0.951706	0.940737	0.927578
KLHL14	0.798468	1.430103	0.837802	1.307733	1.497322	1.342264	1.642599	1.438903
KLHL15	2.490148	2.677339	2.251725	2.436536	2.510474	2.464367	2.573178	2.786778
KLHL17	2.737424	1.989577	1.743442	2.347856	2.787375	2.286784	1.822122	2.766333
KLHL18	15.40196	11.71187	13.28829	11.07494	14.89808	15.54605	9.660393	12.88388
KLHL2	7.909066	7.87633	6.388155	6.80162	6.461169	6.487507	6.822264	7.047781
KLHL20	7.132815	7.361319	6.874884	6.714991	7.476156	6.549567	7.12397	6.76171
KLHL21	11.28706	8.778939	10.08029	10.33739	10.49444	9.656091	9.647465	10.515
KLHL22	3.58256	3.131268	2.960301	3.514641	3.882162	3.158665	2.927929	3.080773
KLHL24	10.03703	10.96212	9.778919	9.452961	8.358254	8.706752	9.969645	10.73076
KLHL25	5.646207	5.368053	5.336996	6.405725	6.727944	5.494526	6.05312	5.335613
KLHL26	1.087137	0.945839	0.727858	1.30289	1.179419	0.878124	1.0224	1.027859
KLHL28	4.364427	4.669105	4.139047	4.595382	5.630528	4.52388	4.294698	4.449073
KLHL29	4.992219	3.680094	3.319834	4.135281	3.771407	3.999509	4.568699	3.307418
KLHL3	0.558264	0.711798	0.671321	0.638253	1.063498	0.997975	0.593621	0.670209
KLHL30	0	0.030271	0	0.030619	0.014941	0.007521	0	0

KLHL31	0.009375	0.109228	0.009258	0.043233	0.014064	0.113269	0.023789	0.047542
KLHL32	0.319522	0.362368	0.466247	0.259017	0.319563	0.36972	0.455009	0.522382
KLHL33	0.813424	0.705479	0.797203	1.067225	0.850507	0.912046	0.856899	0.475002
KLHL34	0.007938	0.016084	0.015678	0.024404	0.015878	0.039961	0.048342	0
KLHL35	0	0	0	0	0	0.016168	0.016299	0
KLHL36	4.724748	4.271676	3.491858	4.062239	4.228355	3.887214	5.074976	5.040138
KLHL38	0.35337	0.323912	0.376663	0.390863	0.336587	0.378374	0.495307	0.164978
KLHL4	2.761295	2.445145	2.138049	2.662385	2.946235	2.172669	2.644206	2.28224
KLHL40	0.011721	0.118746	0.127322	0.096089	0.15239	0.129809	0.059483	0.166427
KLHL41	0.009099	0.009219	0.008986	0.009325	0.018201	0.155743	0.055414	0.018457
KLHL42	5.381207	5.424687	4.921075	5.208576	6.109187	5.394848	6.017441	4.611579
KLHL5	2.272644	2.29344	2.090304	2.370037	2.593823	2.687477	2.157474	2.133251
KLHL6	0.397221	0.487951	0.235363	0.49356	0.461829	0.284953	0.458616	0.599277
KLHL7	4.979667	4.036016	4.569995	5.12941	5.856159	5.316236	4.679642	5.329207
KLHL8	1.984751	2.163971	2.290396	1.964688	2.322812	1.580511	2.295314	2.368625
KLHL9	12.02872	12.68615	11.82118	12.28679	11.79668	11.43807	12.84793	11.86436
KLK1	0	0	0.035166	0	0.035614	0.035853	0.144575	0.036117
KLK10	376.4602	223.0603	314.5817	309.4483	288.031	289.2597	279.4225	240.9312
KLK11	12.41123	12.8043	10.72801	8.789553	10.18307	11.05197	14.08199	16.53423
KLK12	17.74448	10.66379	21.60008	14.57379	16.12557	12.09906	10.26673	8.329955
KLK13	8.760355	4.146388	3.824565	5.597847	5.683127	3.507648	7.003549	3.481985
KLK4	0.228794	0.309063	0.125524	0.260513	0.076275	0.204762	0.206423	0.051567
KLK5	53.56467	41.14748	32.63678	28.96786	32.72788	32.12936	43.95622	27.70499
KLK6	33.56142	22.8962	19.01632	16.2509	15.38507	17.0517	30.28192	13.5082
KLK7	211.0621	161.181	130.8855	165.4553	170.3657	127.3335	202.5611	157.8945
KLK8	0.746931	0.945917	0.819582	1.126887	1.431803	1.002712	1.010847	0.483999
KLK9	0.598753	1.06928	1.092388	0.821579	0.700327	1.093292	0.628335	0.216149
KLKB1	0.038447	0.025967	0.012656	0.013133	0.025634	0.064516	0.104062	0.051992
KLRF1	0.194949	0.084646	0.110012	0.228318	0.027854	0.224321	0.056535	0.056493
KLRG1	0.034943	0.088503	0.017254	0.017904	0.034947	0.017591	0.035467	0.05316
KLRG2	2.31519	1.986252	1.478825	2.301866	1.517384	2.023512	1.99993	2.488037
KMO	0.040801	0.165344	1.275918	0.013937	0.081612	0.451872	1.449439	0.993152
KMT2A	7.18677	5.718186	4.27721	5.447125	6.664469	4.500891	5.555175	6.746564
KMT2B	7.832073	6.876433	5.325008	6.664116	6.78162	6.12805	6.691182	6.914156
KMT2C	11.4556	9.768119	8.26697	9.418598	11.2105	8.796709	9.719916	11.01258
KMT2D	6.375028	5.088172	4.170472	5.040706	5.811408	4.587024	5.016697	5.93707
KMT2E	22.38972	21.95658	19.05728	21.51495	22.49744	19.4458	20.30325	22.47949
KMT5A	35.97584	33.02815	30.67326	34.47218	35.53275	36.8887	30.19024	35.06224
KMT5B	12.54638	12.9268	10.20274	11.36338	11.71494	11.41224	11.68257	12.51378
KMT5C	4.152511	4.181892	3.598885	4.738069	3.843119	4.530326	3.912843	3.909896
KNCN	0	0	0	0	0	0	0	0
KNDC1	0	0	0	0	0	0	0	0.005349
KNG1	0.032125	0.244102	0	0.024691	0.064259	0	0.008152	0.016291
KNL1	3.091612	3.152319	2.638161	2.490333	2.776848	2.498189	3.189853	3.570406
KNOP1	5.46019	6.044532	5.582055	4.999077	5.592483	4.804573	4.566191	4.716726
KNSTRN	15.02425	16.41883	14.32421	16.60757	14.95456	12.85515	15.50408	18.16226
KNTC1	3.165179	3.657854	2.931619	3.013365	2.660057	2.904069	3.029425	3.377055
KPNA1	15.60585	14.49915	15.22644	15.61984	17.20556	14.06523	15.62747	16.11853
KPNA2	43.08803	41.27523	40.19177	37.80676	41.62103	42.37545	44.58028	45.33564
KPNA3	8.858799	9.023464	9.132593	8.68657	10.00669	8.939933	9.303411	9.157963
KPNA4	34.94353	37.09434	38.09228	33.30557	36.68881	36.08597	37.90772	35.04516
KPNA5	1.031092	0.965997	0.919705	0.96574	1.064491	0.73116	0.703332	0.53413
KPNA6	21.29146	19.2425	19.66036	18.77398	21.15877	19.36678	18.98238	19.94625
KPNA7	0.01675	0	0.066165	0.03433	0	0	0	0
KPNB1	40.90506	40.87276	39.25321	39.0931	40.25408	40.46681	41.84446	41.95659
KPRP	39.75591	25.70734	25.66758	23.81631	25.99479	22.0304	42.73028	20.7709
KPTN	6.440737	6.61135	6.578654	9.438917	9.670852	8.401071	7.710276	8.066424
KRAS	18.69318	21.69644	19.11261	17.99342	18.93535	20.70351	17.69209	18.30012

KRBA1	2.493123	2.096673	1.843275	2.884978	2.511098	1.941482	2.024416	2.349597
KRBA2	1.409283	1.455235	0.836372	1.409475	1.355255	1.371158	1.354774	1.422473
KRCC1	41.26881	39.32944	43.64361	44.68267	36.78676	44.09906	44.00643	36.17211
KREMEN1	47.44552	41.30099	39.63323	40.25529	39.67371	39.75919	40.68415	44.2481
KREMEN2	1.24823	1.52402	1.311695	1.377546	1.200377	0.902288	1.13701	0.908923
KRI1	11.98634	12.93192	10.16528	12.35867	11.7881	10.12037	9.380734	10.77066
KRIT1	10.56631	11.09983	9.942852	10.13279	11.2462	10.31149	10.82736	10.77529
KRR1	14.46139	17.07498	16.98954	17.02212	15.45536	17.88935	16.50447	14.74617
KRT1	320.4854	333.7975	261.7464	276.7448	296.1745	192.8268	234.0873	260.6738
KRT10	298.4668	342.0722	395.1486	310.1595	328.1745	228.797	292.8	366.0344
KRT12	0.045241	0.015278	0.029785	0.030908	0.045247	0.075917	0.107146	0.09177
KRT17	1602.435	1148.288	1379.906	1325.032	1348.75	1262.484	1407.932	1288.977
KRT18	6.524501	10.15469	11.36565	14.41045	10.07848	9.955615	10.65338	5.740544
KRT19	71.29005	157.5453	111.0698	111.3765	114.297	117.9224	167.8847	116.4936
KRT2	0.929255	1.154846	1.774174	1.752183	1.610917	1.272421	1.408503	1.382309
KRT2.11	1371.165	1185.173	1349.669	1377.41	1345.617	1431.101	1333.693	1169.614
KRT20	0.071111	0.014409	0.014045	0.029149	0.014224	0.014319	0	0.028849
KRT222	0.444129	0.358129	0.4923	0.594455	0.462316	0.127761	0.358792	0.340136
KRT23	129.3806	97.10695	104.0792	99.62542	102.5651	115.5639	120.2988	106.6292
KRT24	0.13293	0.164602	0.262547	0.060543	0.07386	0.223064	0.164907	0.209724
KRT25	3414.61	2954.195	2919.803	3141.354	3309.47	3441.511	3631.72	3338.252
KRT26	145.3334	107.7902	96.48913	120.221	134.5318	107.5617	110.3609	106.8867
KRT27	1314.809	1026.112	928.2587	1110.465	1157.915	1188.416	1194.491	1166.073
KRT28	466.8699	410.924	493.3905	399.5021	406.9553	478.0245	414.9036	369.4282
KRT3	0	0.202037	0.011584	0.024042	0.164249	0.035432	0.083346	0.011898
KRT32	173.5398	129.2304	186.5257	140.3538	137.2393	153.1139	181.4618	177.9109
KRT35	518.3888	567.0446	578.9732	498.2231	573.3995	613.3151	514.5447	453.4974
KRT36	18.00998	18.17768	24.72914	23.64741	24.73938	23.23346	16.80121	14.60621
KRT39	74.66492	55.97024	98.10899	78.36758	77.41194	102.158	78.77793	64.8057
KRT4	0.214235	0.153209	0.087115	0.309941	0.289885	0.304516	0.102329	0.217285
KRT40	37.01003	37.79685	48.42684	38.80464	33.81514	38.00214	48.33612	48.31745
KRT5	2386.718	1730.776	1961.839	1911.933	1994.215	1722.494	2081.638	2128.608
KRT6A	470.6073	284.4923	376.3476	340.8999	413.1869	360.8542	386.6032	381.5525
KRT7	21.51548	28.50488	24.19079	26.67875	21.70136	27.14813	33.1364	23.99057
KRT71	2222.778	1623.864	1485.094	1874.518	1920.064	1959.752	1938.54	1809.782
KRT74	70.41267	40.58665	28.18539	59.92744	63.78146	57.78452	60.33631	65.94041
KRT77	72.16483	70.64354	92.7952	88.53551	89.23121	58.06685	77.8766	73.63677
KRT78	1.961024	1.277203	4.263012	2.035698	1.031581	0.923106	0.245574	0.852403
KRT79	243.5926	190.9157	297.5402	307.3063	289.1474	292.0401	196.6256	172.033
KRT8	57.34063	60.49281	52.52902	68.10499	44.09483	53.11876	59.56777	59.19319
KRT80	143.4806	100.9761	112.935	137.5338	112.4448	119.1241	118.8798	108.8533
KRT82	125.4585	103.864	130.4969	104.9747	96.14073	106.5535	112.2852	126.5438
KRT84	58.85661	53.13224	59.08465	46.55343	50.84209	48.07178	53.11248	41.65305
KRT9	0.477088	0.435014	0.204162	0.391125	0.15905	0.464336	0.355113	0.112905
KRTAP1-1	1524.522	1093.03	2104.741	1305.903	1329.571	1644.547	938.5428	911.7124
KRTAP1-3	1389.885	1241.075	1761.279	1305.896	1366.462	1546.789	1124.229	1135.937
KRTAP29-:	20.98698	17.47759	18.2531	19.60269	20.0703	26.2111	21.63928	28.44712
KRTAP4.3	125.1863	86.52834	120.0418	65.75755	100.69	100.3982	60.01516	91.24806
KRTAP5.4	94.32449	73.75759	85.1664	64.0437	75.41377	81.81973	105.7133	100.074
KRTAP6-1	1077.925	1448.957	1553.094	1418.682	1311.049	1096.138	1623.438	1492.179
KRTCAP2	89.92815	103.7638	101.0411	97.04234	96.62694	103.8499	95.74886	87.47589
KRTCAP3	5.439336	5.218763	3.521752	5.426412	3.458568	5.113813	4.67999	4.713001
KRTDAP	596.1018	603.863	857.8811	600.4489	635.0748	595.981	529.514	619.6201
KSR2	0.052972	0.033542	0.026156	0.013571	0.039734	0.040001	0.060488	0.026863
KTI12	6.832772	6.411773	5.434059	6.112421	4.313045	6.259173	6.210472	5.80817
KTN1	40.34239	46.06201	41.21148	46.39077	43.00736	42.68594	46.66548	43.5959
KXD1	43.67759	48.10509	48.87613	45.74343	49.15496	50.48756	44.36953	40.64297
KY	0.438766	0.444524	0.256956	0.758104	0.627618	1.551307	0.813017	0.81758

KYAT3	9.89981	12.67878	11.83893	15.53774	10.90436	14.88493	13.15293	13.37653
KYNU	0.381513	0.140553	0.251173	0.272489	0.485624	0.06984	0.199485	0.293139
L1CAM	2.675282	2.057793	1.790368	3.758319	4.239441	3.22181	3.153041	2.918845
L2HGDH	2.03152	2.894891	2.250286	2.959057	2.206625	2.215343	2.58208	2.286661
L3HYPDH	4.566051	3.32986	4.447529	3.996999	3.588818	3.633813	4.043347	4.251284
L3MBTL1	0.654263	0.713406	0.492797	0.931839	0.804072	0.574996	0.613427	0.612965
L3MBTL2	8.812568	7.759979	7.80557	8.099842	8.464235	8.105815	8.221232	8.654499
L3MBTL3	3.523154	3.836321	3.78785	4.112744	4.050619	3.689112	3.569783	3.989677
LACC1	6.911958	6.93543	6.170404	5.266225	7.557672	5.632256	5.485478	6.289125
LACTB	5.325769	6.305777	6.552084	4.45821	6.34041	6.53795	6.434685	6.924442
LACTB2	11.1091	13.64839	16.09983	13.28782	13.22082	15.5791	12.48385	10.16979
LAD1	29.12999	27.2401	29.43275	30.10397	25.71083	28.337	27.59318	23.62785
LAG3	0.143467	0.105709	0.11592	0.347504	0.104353	0.118184	0.105905	0.119053
LAGE3	9.575867	7.529554	6.680747	8.616945	7.504448	9.305519	8.196299	8.238446
LAIR1	0.337131	0.215328	0.246079	0.368013	0.300525	0.081169	0.632303	0.356796
LALBA	0	0	0	0	0	0	0.039293	0
LAMA1	0.231291	0.0721	0.193269	0.054697	0.086004	0.053739	0.231751	0.021052
LAMA2	25.6092	22.90781	20.39525	19.20035	20.05105	15.85694	20.1581	26.93987
LAMA3	13.78981	13.73647	12.03826	16.15086	13.9988	12.80706	16.036	14.80221
LAMA4	21.49597	25.02368	18.24348	22.34418	28.58191	17.20385	20.59963	27.41792
LAMA5	13.69771	9.725819	7.822079	11.40366	10.11702	8.92795	9.432619	10.8249
LAMB1	39.51003	35.79467	34.26413	31.87787	34.93396	32.78227	37.16417	44.04454
LAMB3	14.37655	14.72536	13.22838	18.35678	11.13783	12.2336	15.07348	14.23388
LAMC1	35.89325	34.95635	29.19124	35.29766	33.11807	29.90842	32.12587	39.56505
LAMC2	12.29185	8.669831	9.98086	10.64921	8.821746	8.483543	10.09521	11.56258
LAMC3	18.80259	10.94818	12.46492	13.39886	11.76014	10.0953	13.99603	15.66621
LAMP1	223.8182	206.4105	212.1201	219.6715	215.1639	211.6948	209.5095	191.2305
LAMP2	30.9087	32.16415	36.93843	32.44727	32.4832	37.75168	33.78127	33.5382
LAMP3	0.568824	0.686968	0.372011	0.571333	0.663086	0.288251	0.542945	0.515791
LAMP5	0.04098	0.138392	0	0	0.013662	0.013753	0.013865	0.096981
LAMTOR1	39.14717	40.04252	42.72757	37.51155	39.71726	39.84122	38.37246	34.85779
LAMTOR2	53.37366	47.72299	52.09826	49.62881	51.83522	53.82718	49.64854	46.6112
LAMTOR3	41.98375	46.71977	53.33647	49.35362	46.23635	52.31832	45.53277	44.872
LAMTOR4	26.59457	29.30938	26.2632	28.31687	31.33375	26.58033	27.65187	26.90737
LAMTOR5	48.32826	52.35045	57.8594	52.01337	49.61732	52.71713	53.37731	50.82822
LANCL1	34.99881	41.34592	35.00794	38.32134	31.22937	38.16662	36.71481	35.62231
LANCL2	11.07303	8.865058	10.26542	8.996536	9.27802	9.558085	9.167029	10.98191
LANCL3	1.216609	1.218075	1.470009	1.092735	1.274026	1.448288	1.118636	1.233928
LAP3	198.8205	201.861	259.3373	214.6895	216.9544	263.9751	225.6419	199.117
LAPTM4A	385.3068	458.8374	439.5461	411.2252	402.7046	402.839	410.3795	406.3724
LAPTM4B	41.58237	39.81024	38.4614	46.72555	38.37634	37.20564	43.63199	37.96775
LAPTM5	32.35161	42.68467	32.64746	39.65433	45.76659	26.67742	41.91471	54.36064
LARGE1	5.652927	5.843403	4.035263	5.475369	4.597543	4.651466	4.619303	4.74388
LARGE2	6.739406	6.192097	4.736902	8.077955	6.138466	4.933296	5.723681	6.050676
LARP1	38.42775	37.45333	33.23022	36.38814	37.15888	34.7396	36.51898	35.64899
LARP1B	1.704224	1.771166	1.610571	1.722391	1.856993	1.7011	1.440993	1.883185
LARP4	5.68402	5.033878	5.47276	4.402836	5.693374	5.202197	5.226906	4.803384
LARP4B	11.49098	10.57846	9.681718	11.03598	10.94206	10.72774	9.975354	10.51382
LARP6	9.925826	12.37349	11.86936	11.25137	11.82657	10.91484	11.41144	10.60141
LARP7	12.49888	12.41255	13.39635	13.7148	12.63057	15.53063	13.58404	12.25469
LARS	8.849597	8.545587	9.543787	8.932203	8.791489	8.16817	7.403854	7.691205
LARS2	5.233746	4.584464	3.57624	4.523901	5.403485	4.811283	5.47063	4.998329
LAS1L	33.59564	34.80264	32.51563	33.42044	33.39471	32.21546	30.20718	33.83286
LASP1	48.17675	48.86828	52.39259	48.30613	48.52667	41.85831	49.17399	49.53741
LAT	2.130339	2.266891	1.336372	2.402792	2.465616	1.16013	1.591122	2.038363
LAT2	2.475779	2.589708	2.952968	2.932498	3.521206	2.249898	3.443021	4.924215
LATS1	10.10605	10.6961	9.630858	9.465715	10.09607	9.031054	9.975039	10.10109
LATS2	8.42137	7.415093	7.238893	8.538223	8.332917	7.853547	8.320509	8.790965

LAYN	1.956232	2.237811	1.698336	2.384698	2.133858	1.801856	2.034669	2.583665
LBH	94.68776	90.3017	93.85674	94.16692	83.79695	103.5139	90.71115	103.0455
LBHD2	0.087194	0.088338	0	0.089353	0.087205	0.087789	0	0
LBP	0	0	0	0.012216	0.035766	0.072011	0.072595	0.169262
LBR	27.2663	34.3178	29.66062	32.05295	30.41468	33.12941	31.89409	28.63688
LBX1	0.013437	0.013613	0	0	0.013439	0	0	0
LBX2	0.094718	0.047981	0.046769	0.436789	0	0	0.096139	0.096066
LCA5	1.318918	1.851003	1.291808	1.65071	1.059595	1.708891	1.623999	1.436376
LCA5L	0.009243	0.009365	0	0	0	0.009307	0.009382	0.009375
LCAT	13.69683	11.02448	10.19363	11.83545	11.51476	10.93784	10.14738	15.63057
LCK	2.835504	3.08453	2.425956	3.588656	3.110309	1.894479	3.22287	2.849363
LCLAT1	5.882276	6.264941	6.932576	6.785852	6.264239	7.468945	5.849252	5.38903
LCMT1	14.3221	16.483	15.52479	17.10788	15.74041	16.13108	18.31042	14.74145
LCMT2	1.99269	2.678056	2.560227	2.375446	2.653874	2.272446	2.228966	2.608814
LCN10	0	0.147115	0.358501	0	0.290457	0.073101	0	0.036819
LCN15	0	0	0	0	0	0.041019	0	0
LCN2	2.110048	2.26349	3.799791	4.070239	3.196515	3.499115	5.480229	3.902509
LCN6	0	0.038476	0.037504	0	0	0	0	0
LCOR	3.126684	2.617001	2.451861	2.537665	2.916746	2.494328	2.518944	3.047585
LCORL	0.950354	0.896503	0.744568	0.825844	1.054329	0.834115	0.760689	0.913514
LCP1	11.39758	17.88599	12.34439	15.32817	16.59373	10.1781	18.90338	25.76037
LCP2	2.187079	3.085529	1.756122	2.848697	2.643914	1.762988	2.406605	3.724664
LCT	0.020418	0	0.004033	0.00837	0.004084	0.004112	0.004145	0
LCTL	0	0.06291	0	0	0	0	0	0.015745
LDAH	4.357258	4.703048	4.263344	4.975026	4.530369	4.803106	3.840265	3.983869
LDB1	24.6744	24.15369	21.1855	24.6625	23.78994	22.97678	21.7945	24.50346
LDB2	3.379445	3.500069	3.585158	3.651743	5.68891	4.472139	4.797097	4.403218
LDB3	0.386057	0.288687	0.340399	0.536913	0.648109	0.393322	0.438497	0.400876
LDHA	117.6253	113.6204	129.2219	129.8577	139.8753	117.983	130.2529	135.0807
LDHAL6B	0.113251	0.032782	0.031954	0.066318	0.097085	0.081446	0.016421	0.098454
LDHB	187.7494	207.6603	241.2236	221.8904	210.724	242.8024	223.7551	195.5778
LDHD	1.219411	1.475864	1.422431	2.73406	2.349102	2.051729	1.046647	1.286573
LDLR	9.493099	12.15278	10.30465	15.76214	14.84133	12.06432	14.19141	10.57667
LDLRAD1	0.122652	0.099409	0.072674	0.113121	0.331203	0.074094	0.049797	0.310995
LDLRAD3	5.899566	8.151827	5.70746	6.837949	6.343229	6.982747	6.247085	6.524049
LDLRAD4	6.240146	4.627084	4.443856	4.929021	4.851584	4.019322	4.154272	6.404729
LDLRAP1	17.13943	19.70418	16.35327	19.67891	17.48566	16.55399	17.25687	17.94161
LDOC1	11.58751	17.63774	16.52897	21.39712	20.04283	19.6699	17.95449	10.93869
LEAP2	0.314878	0.035446	0.138202	0.430237	0.734811	0.176127	0.355113	0.354845
LEF1	50.97629	52.00567	55.52233	45.96257	48.57048	56.99867	49.30395	46.58364
LEKR1	0.135599	0.250513	0.149663	0.130784	0.087752	0.128494	0.202401	0.194159
LELP1	0.179418	0.817976	0	0.551586	0.08972	0.090322	0.091054	0
LEMD2	17.2948	17.3419	13.46955	16.49242	15.17668	14.19534	16.16557	16.26991
LEMD3	8.071564	9.359996	7.661327	8.27745	7.927417	8.302102	8.157274	8.533951
LENEP	0.070113	0.099447	0.096935	0.07185	0.042073	0.197658	0.113864	0.014222
LENG1	7.242159	7.998756	7.767455	7.877793	9.855359	7.530704	8.676344	7.285049
LENG8	22.78175	19.10285	13.98177	21.46496	19.26826	17.17512	15.04602	21.41867
LENG9	1.415576	1.604549	1.134959	1.407552	1.037289	1.241799	1.2661	0.867123
LEO1	12.26277	14.69429	12.80161	12.48823	12.60821	14.92355	15.16095	13.5093
LEP	0.173726	1.877395	0.019062	0.326387	0.376455	0.106891	0.156739	0.16641
LEPR	5.891918	6.962393	6.413464	5.922757	5.136946	6.096666	6.975286	6.783613
LEPROT	25.39156	27.67664	27.53353	26.29781	26.44484	28.04961	24.72879	23.64536
LEPROTL1	28.21368	30.71773	32.99324	32.86761	33.34969	34.26208	32.93404	29.27675
LETM1	20.98246	17.66397	17.78832	21.37995	19.68537	18.79324	20.56434	20.89909
LETM2	0.421276	0.593363	0.253675	0.410652	0.462436	0.662094	0.49017	0.573171
LETMD1	3.751778	4.570378	3.205074	3.603837	3.426765	3.404218	3.707118	3.87854
LEXM	0	0.008017	0	0.008109	0	0.007967	0.008032	0
LFNG	9.318426	9.206134	8.973654	9.450861	7.839787	8.085652	8.414934	9.084493

LGALS1	61.26636	92.02332	81.57615	80.40943	119.2631	87.52587	101.9796	125.7127
LGALS12	1.384356	1.861532	1.423034	1.515373	2.133443	1.925997	1.373187	1.66573
LGALS15	0.059806	0	0.059061	0.429011	1.076645	0.120429	0.121406	0.242629
LGALS2	0.060648	0.107527	0.029946	0.015538	0.121312	0.030531	0.046168	0.046134
LGALS3	528.099	573.8907	687.9604	614.4265	601.6104	630.2409	532.5218	502.5865
LGALS3BP	10.13982	11.47454	8.899593	10.60548	11.50181	10.63057	10.43355	9.69448
LGALS4	2.603248	2.171987	1.781084	2.371316	2.484466	2.055711	1.243434	2.088085
LGALS7	290.3339	250.1845	262.7958	271.6772	305.87	254.7275	237.4838	213.705
LGALS8	13.27948	13.47476	12.31571	11.17278	14.62175	12.56326	11.42391	10.37118
LGALSL	55.79081	55.50273	54.51952	56.66744	50.51204	59.8291	59.102	50.04108
LGI1	1.085963	1.515391	1.15337	0.986878	1.065611	1.041807	1.102253	1.298847
LGI2	1.280906	2.857738	1.466799	1.508134	0.654164	0.809465	1.410768	1.893429
LGI3	0.652007	0.716274	0.65164	0.87746	0.534243	1.04401	0.52624	0.446171
LGI4	0.982218	1.24183	1.226503	1.563893	1.185309	2.043241	1.417154	1.416087
LGMN	11.39019	10.76602	12.2564	14.30005	11.90618	10.54538	12.09137	14.63405
LGR4	27.50659	28.71254	25.81706	27.83317	25.36977	26.40062	27.71155	27.10548
LGR5	43.32355	25.75562	29.09615	38.18781	31.92839	34.75852	33.32241	31.42212
LGR6	0.078401	0.059572	0.058068	0.020086	0.117617	0.088804	0.109418	0.039759
LHB	0.584214	0.322844	0.419589	0.544259	0.265586	0.481259	0.592977	0.700264
LHCGR	0.009585	0	0	0.009823	0	0.009651	0	0.009722
LHFPL1	0.21108	0.08554	0.08338	0.346093	0.042221	0.042504	0.214246	0.085634
LHFPL2	4.999998	5.685639	6.261543	5.405427	4.725838	4.874873	6.49904	5.835442
LHFPL3	0.038132	0.077266	0.037657	0.039077	0.038137	0.038393	0.193522	0.116026
LHFPL4	0	0.006795	0	0	0	0	0	0
LHFPL5	0	0	0	0	0.024194	0	0	0
LHFPL6	26.37483	25.24337	23.34153	25.88724	20.95562	21.18572	25.64042	19.90746
LHPP	5.313672	4.983204	4.435805	6.326726	5.333476	5.074135	6.712621	5.49917
LHX1	0	0	0	0	0	0	0	0.010986
LHX3	6.834117	6.288477	5.017487	5.980432	7.244588	5.321671	6.61188	7.203988
LHX4	0.126077	0.127732	0	0	0	0	0	0.051149
LHX5	0	0	0	0	0	0	0	0
LHX6	1.297773	1.579369	1.758296	2.124627	2.912452	2.326454	2.096339	1.990423
LHX8	0.489376	0.671727	0.187076	0.24266	0.236826	0.095365	0.448648	0.832575
LHX9	0.00689	0.006981	0.006805	0.003531	0.003446	0.003469	0.003497	0
LIAS	9.482869	12.69805	13.37557	12.84402	11.5978	12.54517	10.6697	10.79214
LIF	0.282862	0.250752	0.237437	0.297115	0.353623	0.142397	0.208151	0.236683
LIFR	6.978525	8.559201	6.786357	8.317642	7.972337	9.273998	8.341565	8.416328
LIG1	8.544061	8.379024	7.807205	9.488936	8.895952	8.171602	10.30271	9.875188
LIG3	7.419987	7.159984	6.13831	7.148802	6.441625	6.564398	7.494248	6.785391
LIG4	4.21321	4.639243	3.755385	4.013518	4.139569	4.217098	3.879887	4.704519
LIM2	0	0	0	0	0	0	0	0
LIMA1	25.92468	28.30548	21.60229	25.63067	30.86328	27.52131	27.21891	27.05249
LIMCH1	3.424455	3.494908	3.459936	3.019013	3.935754	3.049463	3.340732	4.560405
LIMD1	4.195322	3.552294	3.135279	3.405428	3.742257	3.380942	3.470342	4.042735
LIMD2	3.378371	5.394016	2.998425	4.207054	3.806505	2.540324	3.863103	4.445729
LIME1	0.95719	0.958069	0.341661	1.063627	1.107255	0.603782	0.667208	0.549739
LIMK1	4.225622	3.023003	2.955486	4.440152	4.333386	3.148143	4.189262	4.077378
LIMK2	78.7754	64.01984	67.93803	76.90354	68.15073	75.08131	72.03462	71.03877
LIMS1	13.19804	12.26244	13.80742	14.56687	14.07411	14.5466	13.12651	13.76031
LIMS2	5.237674	5.417659	5.974841	6.346375	5.930209	4.687516	4.747836	6.403639
LIN37	9.783183	9.031851	8.746605	10.70773	9.408122	10.69514	8.901683	11.41963
LIN52	6.112217	6.709349	6.879255	5.71942	6.029695	6.510421	6.848599	6.009133
LIN54	5.267565	5.273164	4.629099	5.258809	5.419812	4.819496	4.954072	5.353152
LIN7A	0.283109	0.473695	0.254165	0.3121	0.291726	0.215942	0.374433	0.574278
LIN7B	5.268127	3.304022	2.583547	4.22341	5.340492	4.510303	2.946388	2.835126
LIN7C	34.77259	40.43576	37.30928	35.57228	36.01364	42.07542	38.44848	40.03276
LIN9	4.91038	4.2286	3.978136	3.923105	3.974284	4.431222	4.485632	4.242463
LINC02054	0	0	0	0	0	0	0	0

LINGO1	0.344855	0.339938	0.598277	0.563525	0.438115	0.45982	0.368948	0.274139
LINGO2	0.042797	0.021679	0.021132	0.021928	0.085605	0.021545	0.076018	0.021703
LINGO3	0.029431	0.089453	0.014532	0.03016	0.014718	0	0.014936	0.014925
LINGO4	0.01937	0	0	0.069473	0.038744	0.068257	0.078641	0.039291
LINS1	2.354858	2.081294	1.873701	1.645565	2.404508	2.556104	1.67085	2.684085
LIPA	22.8739	24.61909	26.75084	38.48506	34.30993	27.28422	28.32192	30.87832
LIPC	2.155152	1.436842	1.936065	1.85232	1.821686	1.637905	1.510066	2.340955
LIPE	5.03232	8.922137	2.718528	3.140136	3.749812	3.11025	2.541259	3.208924
LIPG	1.644213	1.118134	2.09824	1.608003	1.772075	1.776392	1.257373	2.215878
LIPH	6.813741	5.914406	8.444209	7.156853	5.910184	6.355459	5.979886	4.531483
LIPI	0	0.013074	0	0.026448	0.012906	0	0	0.013088
LIPK	11.99163	11.63343	14.56115	12.87702	12.01928	8.119101	9.032606	9.833097
LIPM	58.39766	74.39882	89.49151	86.21838	66.47764	63.92031	60.75547	58.9963
LIPN	0.101779	0.030934	0.060306	0	0.040717	0.071732	0.010331	0.030968
LIPT1	4.240117	6.877674	5.141898	6.146243	4.504333	7.056153	7.804671	7.085761
LIPT2	2.322245	1.832647	2.269169	1.703413	1.271288	1.353642	1.513493	1.0165
LITAF	16.64535	15.61716	16.90346	16.91337	19.36596	16.36459	16.76454	15.71501
LIX1	0.901674	1.207944	0.765336	1.000374	0.924149	0.65274	0.877381	0.551729
LIX1L	7.353991	7.386825	6.219559	7.929063	9.14653	7.309304	6.870985	5.80757
LLGL1	10.61407	10.93014	9.634659	10.48959	11.88057	9.969265	10.47813	10.51448
LLGL2	7.326232	7.327728	7.342586	6.534336	7.18702	6.067207	7.293883	7.012016
LLPH	56.43243	66.66704	77.74376	65.36049	64.69718	75.35548	64.52782	59.74796
LMAN1	13.20446	14.85392	13.46842	14.00912	13.96693	13.57512	14.14197	16.51643
LMAN1L	0.096238	0.113751	0.01584	0.049311	0	0.032298	0.03256	0
LMAN2	44.14626	44.5222	46.12837	47.21843	48.33853	48.47584	48.63395	43.41173
LMAN2L	2.42234	2.425922	2.254677	1.975887	2.046724	2.172573	2.225525	2.689798
LMBR1	7.75306	9.59801	8.279262	10.3757	9.145817	8.792994	8.586017	8.593457
LMBR1L	14.19897	14.70201	17.22777	17.71709	16.24495	15.99067	13.86281	13.62069
LMBRD1	29.20785	28.76805	32.81547	32.10993	28.53449	32.48855	29.83839	27.0005
LMBRD2	7.079425	6.087895	5.431797	6.103577	6.232395	5.996732	6.58323	6.621267
LMCD1	34.78489	39.25899	33.36024	36.4559	41.76701	39.9429	38.81395	39.56909
LMF1	2.987885	2.696323	2.04201	3.649943	3.661127	2.94853	3.042746	2.839764
LMF2	9.423162	9.634217	10.24271	11.49066	8.734266	9.433261	10.27583	10.57427
LMLN	0.807427	0.798072	0.714713	0.797156	0.812455	0.70389	0.754575	0.813928
LMNA	121.5945	113.5623	124.3597	121.9396	130.3518	108.4931	129.5301	122.4335
LMNB1	35.84441	39.58484	36.61707	34.41099	31.22085	33.97502	39.21409	36.79839
LMNB2	27.1945	25.80601	22.54844	23.57259	22.91477	22.45146	26.29095	27.22479
LMNTD1	0.414972	0.195712	0.303818	0.307941	0.207513	0.237718	0.355839	0.478932
LMNTD2	0.0357	0.021701	0.014102	0.09512	0.057128	0.071889	0.072472	0.086901
LMO1	9.819728	10.09753	9.348964	10.27387	9.526955	10.53804	6.505423	8.915856
LMO2	23.41528	21.37702	25.14962	23.39733	26.67503	27.80135	21.3784	22.08774
LMO3	0.28037	0.28405	0.17558	0.287315	0.294085	0.385561	0.256812	0.138713
LMO4	72.10933	71.98404	53.7002	65.5803	62.06853	61.61567	75.18285	69.80444
LMO7	20.31203	17.40858	17.19472	17.49645	14.47985	17.71283	18.47528	18.93956
LMOD1	3.673843	4.534242	3.748653	4.134802	4.00352	4.153291	5.135391	3.855373
LMOD2	0.075476	0.101956	0.149072	0.180474	0.276783	0.253307	0.06384	0.063792
LMOD3	0.019374	0.006543	0.006378	0.006618	0.032294	0.039013	0.006555	0
LMTK2	8.971561	7.893998	6.994194	6.948418	7.848936	7.261634	8.478863	8.062921
LMTK3	0.3247	0.280714	0.444641	0.288377	0.255464	0.165639	0.303205	0.171248
LMX1A	0.217577	0.22925	0.24065	0.26756	0.235014	0.227826	0.220841	0.317772
LMX1B	0.989748	1.515767	1.022877	1.273728	0.667591	1.390479	1.471848	0.466901
LNP1	1.276263	1.472598	1.015291	0.862835	0.762311	1.168977	1.187457	0.898911
LNPEP	16.6269	13.64339	13.33494	14.40301	15.56941	12.78619	15.83428	19.53895
LNPK	6.120365	5.907063	5.669505	5.89635	5.660791	6.853062	6.37656	6.350144
LNX1	2.849155	2.572792	2.541804	2.560053	3.090425	2.986413	2.787114	3.524894
LNX2	8.778679	6.923887	7.280707	7.379942	7.633243	8.169056	7.740312	7.933627
LOC10003	2.131155	3.022773	2.665827	2.183944	2.984002	4.219904	3.388891	3.24224
LOC10003	0.178533	0.115103	0.144253	0.182956	0.154208	0.073535	0.403607	0.263382

LOC10010	48.35575	53.75667	57.16273	30.63531	77.24817	38.37327	37.89882	114.396
LOC10012	0.157242	0.11948	0.155283	0.020142	0.058974	0.039579	0.119701	0.059805
LOC10013	0	0	0.234638	0	0	0	0	0
LOC10014	1663.22	1532.532	1716.96	1488.135	1674.96	1896.182	1414.643	1185.559
LOC10014	13.9784	25.30776	17.35115	25.49921	21.19685	12.99881	17.2753	20.741
LOC10019	0.161679	0.126001	0.036846	0.121077	0.136823	0	0.18304	0.08199
LOC10019	1.522346	1.449414	1.123005	2.029952	1.045602	0.738673	1.172849	1.48821
LOC10052	2405.808	2266.873	2890.396	2559.681	2352.897	2770.759	2208.642	2226.556
LOC10052	1403.877	1389.329	1860.889	1633.562	1711.274	1800.757	1470.061	1202.097
LOC10052	1394.973	1364.329	1682.096	1150.718	1018.937	1645.174	1615.562	1697.033
LOC10052	1141.675	1092.627	1135.323	1272.299	1174.043	1325.815	1050.53	1060.378
LOC10052	4.262542	5.278146	4.737495	5.291852	5.546605	4.383955	5.163863	5.671323
LOC10110	0.057769	0	0	0	0	0	0	0
LOC10110	128.0002	62.58795	154.9619	93.4161	125.0396	132.5334	38.29722	80.96742
LOC10110	0	0	0	0	0	0	0	0
LOC10110	0.407389	0.611461	0.238408	0.402018	0.754522	0.258257	0.566649	0.505009
LOC10110	0	0	0	0	0.033524	0	0	0
LOC10110	0	0.029355	0.028613	0.029692	0.028978	0.029172	0.176453	0.029387
LOC10110	2.608487	3.074801	2.556396	1.778679	4.364573	3.065689	3.49324	3.510728
LOC10110	0.104307	0.140902	0.103008	0.053446	0.034774	0.05251	0.141163	0.017632
LOC10110	0.770728	2.317068	0.421928	1.013029	1.793013	2.412328	0.569711	1.283008
LOC10110	0.031842	0.06452	0	0	0	0.03206	0	0
LOC10110	2.627804	3.06473	4.345218	1.659575	1.955828	0.79988	5.489512	5.144479
LOC10110	0.177875	0.180209	0.103329	0.193004	0.303473	0.136951	0.233644	0.106122
LOC10110	55.37028	61.87221	62.78671	60.30523	56.44166	63.20974	63.3947	56.8523
LOC10110	7.27629	6.761237	4.364276	5.878304	6.183414	5.663042	4.712179	6.836569
LOC10110	0	0	0	0	0	0	0	0
LOC10110	0.833062	0.704779	0.398619	0.748085	0.609846	0.389112	0.400986	0.243895
LOC10110	0.064343	0.032594	0	0.032968	0.032176	0	0.065308	0.032629
LOC10110	972.7542	872.2359	1129.402	747.6372	956.5305	873.5916	964.5263	717.8212
LOC10110	0.010208	0	0	0.01046	0.003403	0.006852	0.010361	0.024157
LOC10110	0.432822	0.422262	0.189968	0.229985	0.480976	0.242099	0.341689	0.666605
LOC10110	4.217545	5.083564	2.140116	5.603245	5.051705	6.512201	7.867889	2.485395
LOC10110	3.730926	3.945718	2.957058	4.252543	3.105494	4.119938	4.725061	5.322066
LOC10110	11.27131	7.338201	13.09185	10.89158	11.38625	13.48088	12.13141	5.965234
LOC10110	0	0	0	0.017165	0	0	0.017001	0
LOC10110	216.0928	228.1156	245.0931	233.6394	193.4795	233.8318	240.7422	193.9314
LOC10110	0.02106	0.12802	0.041596	0.064746	0.252757	0	0.064129	0.106801
LOC10110	2.124537	3.163404	1.287449	1.434948	2.157006	2.041821	2.744515	3.428061
LOC10110	0.238332	0.452738	0.323624	0.335825	0.461829	0.194968	0.483815	0.287049
LOC10110	0.278192	0.140921	0.045788	0.110865	0.077285	0.093364	0.094121	0.579979
LOC10110	0	0	0	0	0.016065	0	0	0.008146
LOC10110	0	0	0	0	0	0	0	0
LOC10110	0	0	0	0	0.037549	0	0.057161	0
LOC10110	0	0	0	0	0	0	0	0
LOC10110	14.36726	15.46718	15.53346	16.93556	12.62117	16.01805	16.04365	16.60506
LOC10110	0.12315	0.221806	0.162153	0.224356	0.184748	0.144656	0.152774	0.194293
LOC10110	3.445998	3.68518	3.024943	3.452882	3.676207	4.009246	2.526108	3.029047
LOC10110	0.074737	0.037859	0.332128	0.038294	0.149494	0	0.037929	0.113702
LOC10110	4.103983	4.771126	3.809676	4.216154	4.750974	4.14235	3.436573	3.766977
LOC10110	0	0	0	0	0	0.058344	0.176453	0.058774
LOC10110	0	0	0.007857	0	0	0.00801	0	0
LOC10110	0.010211	0	0	0.031393	0	0.010281	0	0.010357
LOC10110	0.044866	0.022728	0.051692	0.053641	0.044872	0.082817	0.075899	0.060673
LOC10110	0.767445	0.949028	1.014225	0.90211	0.744969	1.306751	0.927875	0.515098
LOC10110	10.59447	12.24946	11.57071	12.63671	10.39541	9.684902	11.41784	10.93499
LOC10110	0	0	0	0	0.018072	0.036385	0.01834	0.036653
LOC10110	0.040042	0.202836	0	0.246201	1.501759	0.262049	0.162569	1.766611

LOC10110	1.69564	2.145491	1.689136	2.04116	1.880997	2.38564	2.119402	1.907527
LOC10110	0	0	0	0	0	0	0	0
LOC10110	0	0	0	0	0	0	0	0
LOC10110	0.035884	0	0	0	0	0.018064	0.018211	0
LOC10110	0	0	0	0	0	0	0	0
LOC10110	0.086721	0.362418	0.246216	0.177738	0.43366	0.294682	0.187046	0.109944
LOC10110	3.678385	3.45563	3.880226	4.711875	4.096913	5.67311	5.023336	4.968679
LOC10110	0	0	0	0	0	0	0.02729	0
LOC10110	0.080122	0	0.026374	0.054738	0.026711	0	0.054216	0
LOC10110	123.9453	84.73671	116.3307	94.84688	93.5752	99.70608	106.2063	112.7652
LOC10110	79.33353	86.46938	94.56982	87.10076	87.71971	86.52613	93.04333	82.01814
LOC10110	1.55288	1.830427	1.496667	1.86677	1.732282	1.601072	1.568595	1.983877
LOC10110	48.78923	62.38429	69.3115	59.72359	55.70137	65.42996	65.44165	55.65124
LOC10110	8.106125	8.560076	5.077008	8.367184	5.553462	7.041393	4.770484	5.104898
LOC10110	14.44408	17.86464	24.02975	28.88833	32.47826	29.63161	13.70748	10.68674
LOC10110	0.129579	0.30632	0.277257	0.442631	0.604782	0.413138	0.241126	0.569504
LOC10110	1.367152	1.361377	1.142051	1.218693	1.22685	1.263356	1.302119	1.519578
LOC10110	1.222542	1.083764	1.149265	0.939619	1.046349	0.840322	1.038044	1.287636
LOC10110	1.811134	2.124624	1.141388	1.855996	1.430027	1.907484	1.330349	2.187381
LOC10110	0.321715	0	0	0	0	0	0	0
LOC10110	406.976	482.864	541.6738	490.7372	556.4557	549.4602	572.4932	540.9161
LOC10110	0.062616	0.634373	0.80386	0.128333	0.500989	0.126087	0.699103	1.651181
LOC10110	0.088433	0.44797	0.30566	0.770303	0.17689	0.801338	0.2244	0.313923
LOC10110	0.204458	0.125375	0.170029	0.137843	0.457398	0.157099	0.098301	0.114598
LOC10110	0.028722	0.029099	0.127637	0.029433	0.057451	0.13013	0	0.043696
LOC10110	0	0	0	0	0	0	0.012198	0
LOC10110	15.13927	14.41429	13.32002	14.37757	13.9356	13.71282	13.16603	13.56708
LOC10110	0.008096	0.041013	0.007995	0	0.024292	0	0.008218	0.008212
LOC10110	0	0	0	0	0.029907	0	0	0
LOC10110	0.033681	0.20474	0.365878	0.31064	0.101057	0.305203	0.307679	0.170804
LOC10110	0	0	0	0	0.029716	0	0	0
LOC10110	0.04529	0	0	0	0	0	0	0
LOC10110	0	0	0	0.011774	0	0.011568	0	0
LOC10110	3.888771	4.161145	3.04513	3.786802	3.346149	3.607389	3.706348	3.988446
LOC10110	0.356298	0.447875	0.47566	0.554447	0.560911	0.544741	0.462098	0.542055
LOC10110	0.132381	0.421515	0.18676	0.17442	0.472851	0.133286	0.36471	0.210989
LOC10110	0.182425	0.224423	0.501851	0.440652	0.560377	0.288626	0.198387	0.488986
LOC10110	0.097693	0.214447	0.24119	0.15017	0.048853	0.081967	0.198318	0.08257
LOC10110	0.013953	0	0.013779	0	0.013955	0.07024	0	0
LOC10110	0	0	0	0.030448	0.029716	0	0	0.030135
LOC10110	5.237631	5.1388	3.593435	5.009544	4.723667	4.10771	4.495449	4.473425
LOC10110	0.044087	0.057428	0.00622	0.083905	0.06299	0.025365	0.063927	0.063879
LOC10110	2.682387	3.675429	1.932441	2.117951	3.672266	2.545753	1.584477	1.650183
LOC10110	0.243561	0.370136	0.340745	0.332791	0.690177	0.204354	0.412023	0.617569
LOC10110	0.965896	1.100203	0.873025	0.889162	0.649472	1.016449	0.95269	0.841278
LOC10110	0	0	0	0	0	0	0	0
LOC10110	1.779072	1.707556	0.978626	1.33537	1.240829	1.225575	1.085039	1.836054
LOC10110	0.4579	0.405921	0.452195	0.351932	0.57245	0.4034	0.406673	0.406367
LOC10110	4.541972	4.601581	4.164994	5.530971	4.763788	5.478692	4.056291	4.247672
LOC10110	0	0	0.021148	0.021945	0.042835	0	0	0
LOC10110	0.015702	0.047725	0.015507	0.040228	0.047113	0.007905	0.031875	0.047777
LOC10110	1.684403	1.336765	1.607968	1.956277	1.684621	1.187137	1.624186	2.078531
LOC10110	0.827817	0.838681	1.060543	0.756612	0.917429	0.855999	1.090034	0.794218
LOC10110	0	0	0	0	0	0	0	0.027831
LOC10110	0.131935	0.181405	0.223357	0.144861	0.141378	0.17079	0.220002	0.143372
LOC10110	0.028619	0	0	0	0	0	0	0
LOC10110	0	0.053302	0	0.053914	0	0	0.160201	0
LOC10110	0.021849	0	0	0	0	0.065996	0.022177	0

LOC10110	17.43004	17.54499	15.43796	17.36296	16.02797	20.52738	19.21171	15.95023
LOC10110	0.182221	0.110768	0.10797	0.186735	0.874774	0.256852	0.036991	0.332667
LOC10110	4.68564	3.957699	5.478835	5.121108	4.810935	5.104685	4.945739	4.805029
LOC10110	0.004799	0	0.004739	0.004918	0.0048	0.004832	0.004871	0
LOC10110	0.162007	0.05793	0.009411	0.996122	0.867328	0.105544	0.793169	1.043875
LOC10110	0	0	0	0	0	0	0	0
LOC10110	0.030893	0	0.030508	0	0	0.031104	0	0
LOC10110	1.42995	1.001319	1.100633	1.271424	1.282915	1.164478	1.643496	1.109058
LOC10110	3.599002	5.506751	4.429028	3.782719	3.276438	4.339016	4.168152	3.668956
LOC10110	0	0	0	0	0	0	0	0
LOC10110	3.677408	3.642878	4.196501	3.97786	4.597355	4.751581	3.359313	3.066691
LOC10110	0.022178	0	0.10951	0.022728	0.022181	0.04466	0.090044	0.022494
LOC10110	3.415564	0.635964	1.531527	2.327139	0.276973	3.383132	0.056218	2.434294
LOC10110	79.72038	102.3514	150.2346	88.33058	73.11548	47.73839	52.50719	114.8921
LOC10110	0.365606	0.473294	0.407853	1.019914	1.157902	0.961159	1.085782	0.638618
LOC10110	0.643629	0.704947	0.360751	1.372623	0.643712	0.66554	0.847503	0.882151
LOC10110	1.301822	1.426921	1.108276	1.431823	2.014717	1.175124	1.913678	1.809795
LOC10110	0	0.017407	0	0.017607	0	0.017299	0.01744	0.034853
LOC10110	0.017056	0.03456	0.033687	0.104872	0.085292	0.017173	0.051936	0.397876
LOC10110	0	0	0	0	0	0	0	0
LOC10110	0.153945	0.116974	0.180532	0.059159	0.182834	0.048437	0.126957	0.185412
LOC10110	3.056009	2.919195	2.289795	3.55424	3.779268	2.539652	2.653805	4.910023
LOC10110	0.018622	0	0.01839	0	0.018625	0.018749	0	0
LOC10110	0	0.032482	0	0.032855	0	0.03228	0	0.032517
LOC10110	11.87315	12.86095	12.19828	12.06206	11.2416	11.52364	13.2668	12.31981
LOC10110	0.062337	0	0.02052	0.063881	0.083126	0.041842	0.063272	0
LOC10110	0.10938	0.098503	0.084013	0.149453	0.07293	0.061182	0.061678	0.073958
LOC10110	8.321103	11.44478	21.36529	15.6074	7.716929	19.34545	10.18629	9.30909
LOC10110	2.90185	3.306039	2.184415	2.984951	3.72361	3.098077	3.890122	5.886319
LOC10110	0	0	0	0	0.022814	0	0	0
LOC10110	0	0.065375	0.031862	0.016532	0.032268	0.146181	0	0.016362
LOC10110	0.88098	0.658433	0.713117	1.361603	1.45886	0.596179	0.586357	1.025352
LOC10110	0	0	0	0	0	0	0	0
LOC10110	0.521075	0.326804	0.375727	0.313609	0.62868	0.416377	0.209877	0.260052
LOC10110	0	0	0	0	0	0.029632	0	0
LOC10110	0.009641	0	0.004761	0.00494	0.004821	0	0	0
LOC10110	0	0	0	0	0.017205	0	0	0
LOC10110	0.018772	0.228221	0.055615	0.019237	0.168971	0.056701	0.019054	0.076157
LOC10110	0	0	0	0.013347	0.013026	0.013113	0	0
LOC10110	9.857095	21.13865	24.31675	21.19496	16.33152	30.29463	5.820001	10.38642
LOC10110	0	0	0.077046	0.019988	0.039014	0	0.019797	0
LOC10110	1.476386	1.259589	3.775426	3.99739	2.766638	0.438118	2.08217	5.185743
LOC10110	0	0.018389	0	0	0	0	0	0
LOC10110	23.37591	20.3577	21.52528	25.98555	30.16037	23.28622	25.23499	17.20842
LOC10110	0.031496	0.055495	0.010819	0.01263	0.093131	0.041363	0.065327	0.029167
LOC10110	0	0.115741	0.451272	0.62438	0.038085	0.038341	0.077303	2.008375
LOC10110	0.043829	0.026642	0.008657	0.053898	0.008767	0	0.026692	0.008891
LOC10110	0.622307	0.615639	0.441032	0.405141	0.483265	0.272737	0.69109	0.891058
LOC10110	0.609157	0.541361	0.401045	0.689956	0.673366	0.537999	0.347113	1.235658
LOC10110	1.491528	1.305891	1.309283	2.245535	1.307558	1.557336	2.111985	1.438056
LOC10110	0	0.029507	0.143811	0.059693	0	0	0	0.02954
LOC10110	0.014143	0.014329	0.013967	0.014493	0	0	0.071776	0.043033
LOC10110	0.274308	0.317609	0.087072	0.331299	0.450706	0.197272	0.10938	0.218596
LOC10110	15.32611	12.38876	18.46272	7.685787	13.37131	15.81384	20.02421	14.38673
LOC10110	4316.439	3771.357	4696.463	4284.942	3906.801	4620.707	3783.744	3585.477
LOC10110	1.840095	2.203804	1.6679	1.710577	1.754889	1.865899	2.241236	2.27954
LOC10110	0.046753	0.015789	0.04617	0.047911	0.015586	0	0.205633	0.221284
LOC10110	0	0.119648	0	0	0	0	0	0

LOC10110	11.42038	11.6892	8.793925	13.903	13.63579	10.60352	14.87685	13.45392
LOC10110	0.117306	0.190153	0.208519	0	0.093857	0.047243	0.214318	0.142771
LOC10110	0.199389	0.126253	0.147678	0.051082	0.174488	0.276032	0.202379	0.126392
LOC10110	0	0.038011	0	0	0	0	0.076164	0.038053
LOC10110	0	0.520554	0.37388	0.193988	0.189323	0.136137	0.548966	0.246849
LOC10110	0	0.112526	0	0	0	0	0	0
LOC10110	3.421163	4.142367	3.2255	3.676926	3.123556	2.712421	4.041144	3.542406
LOC10110	0	0	0	0	0	0	0	0
LOC10110	0	0	0	0	0	0	0	0
LOC10110	4.727162	4.829418	5.481694	4.847645	5.061926	4.483014	4.616757	5.351405
LOC10110	0	0.021863	0.106555	0	0	0.108637	0.065711	0.109436
LOC10110	0	0	0	0.016132	0	0	0.07989	0
LOC10110	0	0.982651	1.535579	0.015777	0	0	0.468795	0.031229
LOC10110	3382.23	3069.695	5457.84	5035.761	3478.702	4594.191	3534.407	3324.449
LOC10110	0.199086	0.554672	0.294908	0.333029	0.638329	0.256453	0.350655	0.475106
LOC10110	3.013087	3.648266	2.954808	2.990895	2.509481	2.949109	2.845163	2.779132
LOC10110	2.153013	2.343795	1.951088	2.483218	2.600838	2.388744	1.825375	1.87538
LOC10110	8.785038	11.58846	9.65018	11.40667	10.113	10.22964	10.24695	9.468006
LOC10110	0.190033	0.171899	0.026809	0.09737	0.18327	0.129832	0.110219	0.220272
LOC10110	0	0	0	0	0	0	0	0.023755
LOC10110	1.552684	1.159098	1.466085	1.633016	1.798077	1.261603	1.023001	1.0913
LOC10110	0.03264	0	0.225636	0.267592	0.146901	0	0.26504	0.744864
LOC10110	0	0	0	0	0	0	0	0
LOC10110	13.91675	16.92768	15.75206	14.8783	15.18481	13.7297	16.91689	14.02013
LOC10110	5.851101	5.263225	7.586235	6.683976	5.31016	4.723611	5.311686	5.501115
LOC10110	0	0	0	0	0	0	0.031322	0
LOC10110	5.547294	7.534098	7.427025	6.609545	6.643171	8.78365	7.670186	7.420321
LOC10110	41.88496	50.04087	60.67884	49.53631	38.39951	53.77478	48.12819	36.60332
LOC10110	275.7167	218.6514	354.6976	234.8295	231.1428	253.0229	271.8097	269.9199
LOC10110	0.3674	0.372221	0.131935	0.479182	0.734894	0.235397	0.71192	0.575882
LOC10110	0.376958	0.413731	0.356751	0.539205	0.534093	0.474417	0.557977	0.971742
LOC10110	11.74282	13.98636	13.31728	10.86914	11.57596	14.65379	12.30345	9.656056
LOC10110	1.697783	2.206051	2.294053	3.010195	1.989089	2.691597	2.691552	2.864453
LOC10110	0.030357	0	0	0	0	0.061129	0	0
LOC10110	972.8738	926.6325	964.4995	964.7788	974.6865	952.348	1043.91	636.1283
LOC10110	0	0	0	0	0	0	0	0
LOC10110	0.730152	0.369867	0.300439	0	0.851954	0.122523	1.173415	0.308561
LOC10110	19.67688	18.18472	15.73494	25.20044	13.8236	17.14403	30.20161	17.75686
LOC10110	0.492625	0.875955	0.536129	0.587249	0.492689	0.435257	0.510219	0.530228
LOC10110	2.79531	3.233956	2.72486	2.66126	3.742592	3.023217	2.461991	3.146049
LOC10110	2.835196	2.736272	2.992277	2.685108	2.869159	3.131904	3.150494	3.31166
LOC10110	0	0	0	0.014552	0	0.028595	0.057654	0.028805
LOC10110	2.692936	3.194726	3.225573	2.724033	2.719349	3.655927	3.359351	3.066073
LOC10110	1.522423	1.858794	1.503454	3.560306	1.991118	1.297005	2.3377	3.088192
LOC10110	0.219141	0.468702	0.156296	0.174665	0.316578	0.318699	0.160642	0.246956
LOC10110	0.508073	0.77783	0.568641	0.312395	0.858189	0.329661	0.618833	0.732879
LOC10110	2.53797	3.549089	2.965255	2.19789	3.217561	3.814968	2.975145	3.045414
LOC10110	122.2312	39.60337	98.87571	96.47959	68.60003	69.01219	94.74462	99.22487
LOC10110	3.92531	4.466724	4.101118	4.139136	4.58012	4.181234	4.677092	4.587022
LOC10110	0.017786	0.018019	0.163934	0.133661	0.296471	0.376057	0.012035	0.210457
LOC10110	0.299434	0.127732	0.077816	0.0969	0.09457	0.190408	0.207949	0.671329
LOC10110	0.182786	0.092593	0.067691	0.374628	0.045703	0.161031	0.278292	0.185388
LOC10110	0	0	0	0	0	0	0	0
LOC10110	1.863851	1.568291	1.302485	1.65068	1.797583	1.629224	1.586194	1.71802
LOC10110	0.029247	0	0	0	0.058501	0	0	0
LOC10110	5.110406	4.424063	3.725879	4.613971	5.197426	4.149965	4.341034	5.047321
LOC10110	51.98232	56.83409	60.86288	69.50992	54.51676	58.6475	57.85799	52.80892
LOC10110	1.028541	2.162231	1.003028	2.503293	3.008871	0.996733	0.587232	0.912785

LOC10110	33.03324	34.07778	34.82801	33.02276	31.68037	32.23768	34.80868	34.15688
LOC10110	0.088976	0	0	0.065128	0.038137	0.038393	0	0
LOC10110	0.043171	0.014579	0.014211	0.073734	0	0	0.014606	0.058381
LOC10110	0	0	0	0	0	0	0	0
LOC10110	3.532613	7.18548	2.522523	4.483376	5.598557	5.170962	5.626637	3.445098
LOC10110	0.04086	0	0.040351	0.062808	0.006811	0.089135	0.048385	0.006907
LOC10110	66.60128	100.7451	63.03474	42.55039	51.68964	41.015	44.76996	47.59375
LOC10110	59.45349	72.07389	60.10399	62.09493	57.01886	54.33435	67.52245	53.97184
LOC10110	2.848426	1.700217	2.18431	3.083711	2.572275	1.896818	1.931786	3.006361
LOC10110	2.157498	2.422117	4.894657	15.47658	7.756334	8.336694	2.544973	4.672127
LOC10110	0.144678	0.299261	0.220266	0.352122	0.180871	0.139597	0.195798	0.250677
LOC10110	9.329721	11.84402	9.297741	9.823162	7.90853	7.503311	9.758377	8.510513
LOC10110	0.021952	0.088962	0.021679	0.067488	0.109776	0.110511	0.111408	0.133589
LOC10110	40.42523	59.75397	31.71301	57.29263	55.66484	60.62913	43.77226	26.62477
LOC10110	2.145812	2.319878	1.393754	2.184206	2.333332	2.044473	2.382643	2.117933
LOC10110	18.75316	17.65364	17.62272	17.43931	18.58752	16.50932	16.63014	17.35836
LOC10110	0	0	0	0	0	0.029539	0	0
LOC10110	5.492791	6.052637	5.264429	4.767703	5.581047	5.116093	5.584068	5.863961
LOC10110	2.266408	5.48708	4.444271	3.320777	4.254124	3.753024	5.035842	4.985944
LOC10110	0.044854	0	0	0.045965	0.074767	0.045161	0.015176	0.030329
LOC10110	1.299497	0.632957	0.913121	0.832304	0.974749	0.830315	0.317065	0.671671
LOC10110	0	0.029143	0	0	0.007192	0.007241	0	0
LOC10110	0	0	0	0.029631	0	0	0	0
LOC10110	0.07911	0.096178	0.046875	0.08107	0.031648	0.04779	0.096356	0.096284
LOC10110	2.751744	3.13634	2.940676	2.799763	2.575179	2.374751	2.583543	3.050073
LOC10110	0	0	0	0	0	0.026686	0	0
LOC10110	0	0	0	0	0	0	0	0
LOC10110	1.492755	0.538046	0.219706	0.397142	1.399639	0.332384	0.386071	0.38578
LOC10110	0	0	0	0	0.009121	0	0	0
LOC10110	0.120817	0.183603	0.03977	0	0.020139	0.040547	0.122629	1.061982
LOC10110	0.10056	0.10188	0.099307	0.343503	0.201146	0.101247	0.340228	0.305974
LOC10110	0.285339	1.208896	0.3074	0.744309	0.778298	0.443991	0.394935	1.157604
LOC10110	0	0.087791	0.085574	0	0.043332	0.218114	0.087954	0.175775
LOC10110	7.635568	7.326731	6.062832	8.141099	8.978593	8.309444	7.340298	4.50444
LOC10110	2.981384	3.944821	2.702904	2.771414	3.063238	4.051544	5.671879	3.602153
LOC10110	0.035162	0	0	0.018017	0.017583	0	0	0
LOC10110	23.77675	18.43646	24.4234	18.76351	22.48785	20.73472	22.95523	23.37468
LOC10110	0.048341	0	0.023869	0.049538	0.024173	0.024335	0.049066	0
LOC10110	9.083234	7.547568	7.073592	9.206438	8.278623	9.634221	9.118662	9.167765
LOC10110	0.027454	0	0	0	0	0	0	0
LOC10110	30.66562	28.76673	28.02079	27.88292	27.62732	26.29108	28.0782	28.57793
LOC10110	4.890653	1.496059	2.501819	4.248226	0.785857	2.346091	2.915154	1.951133
LOC10110	79.47078	69.12443	92.17267	80.52359	94.50087	90.11335	88.3283	81.0991
LOC10110	0	0	0	0	0	0	0	0.011159
LOC10110	0.200209	0	0.237257	0	0	0	0.040642	0.040612
LOC10110	0.859602	0.818103	0.488756	0.627304	0.937869	0.642549	1.269085	1.2285
LOC10110	0	0	0.025105	0	0	0.025595	0	0
LOC10110	7.429618	7.981347	14.10483	8.488785	7.985737	7.071963	8.862915	6.322793
LOC10110	384.241	402.335	427.4696	382.4696	410.7403	402.4374	377.1841	367.6923
LOC10110	11.31244	10.90634	10.63093	10.05943	9.489079	9.515928	10.51911	10.77027
LOC10110	73.15184	79.81279	87.75651	116.6021	148.2229	73.72515	109.2597	47.58445
LOC10110	0	0	0.015502	0	0	0	0	0
LOC10110	0.10602	0.080558	0.078524	0.19013	0.15905	0.026686	0.457342	0.026882
LOC10110	3.263212	3.71652	3.352319	3.38893	3.625043	3.076027	3.234358	3.431835
LOC10110	0	0	0.038766	0	0.039261	0.158095	0.079689	0.238886
LOC10110	0.048592	0.04923	0.047987	0.049796	0.048599	0.146773	0.098642	0
LOC10110	0	0	0	0	0	0	0	0.018938
LOC10110	0.053756	0.163386	0	0.073451	0.358422	0.108247	0.054563	0.181739

LOC10110	25.0725	26.85174	23.42253	25.02322	26.42266	24.5077	25.44078	25.80408
LOC10110	0.718823	1.274449	1.330999	0.920785	1.123306	1.6284	1.36801	1.959337
LOC10110	0	0.05137	0	0.02598	0	0.025526	0.025733	0
LOC10110	1.817924	0.472608	0.609715	0.646761	0.885066	0.366069	0.264594	0.918423
LOC10110	0.06138	0.248741	0.060615	0.0629	0.552489	1.112383	0.155751	0
LOC10110	51.82842	57.01988	53.45608	64.03888	47.89674	65.51022	60.3845	47.92713
LOC10110	10.52188	11.14525	6.275462	6.381153	8.09603	5.369221	13.98572	12.85782
LOC10110	0	0.073749	0	0	0.072803	0	0.036943	0.07383
LOC10110	4.801901	5.764599	5.261748	4.752324	5.52619	5.132735	4.773781	5.637492
LOC10110	0	0	0	0	0	0	0.047348	0
LOC10110	0.011259	0.011406	0.044474	0.011538	0.101342	0.022671	0.011428	0.034257
LOC10110	4.923302	5.302114	1.416476	2.900028	5.97076	4.410502	4.564327	6.094293
LOC10110	2.140166	2.041959	1.896209	2.121398	2.250473	1.859938	2.228905	2.061459
LOC10110	0.48314	0.444982	0.487963	0.708904	0.614985	0.254276	0.434661	0.311829
LOC10110	8.767382	8.519896	6.941657	7.334329	9.586569	8.595657	7.887065	7.907051
LOC10110	4.343635	4.601649	3.680724	4.966786	5.237131	4.25916	4.322484	5.116956
LOC10110	0	0	0	0.026558	0.025919	0	0	0
LOC10110	1.089779	1.122483	3.192728	9.362284	1.017259	2.578477	7.595398	3.665888
LOC10110	0	0	0.020369	0	0.020628	0	0	0.020919
LOC10110	2.092241	1.627929	1.85129	1.914223	1.727578	1.284143	1.617353	1.921706
LOC10110	12.9126	13.05322	16.26651	18.96603	14.60864	17.55898	14.63801	17.68811
LOC10110	0.346323	0.574147	0.621832	0.500088	0.503807	0.396237	1.581829	0.798302
LOC10110	0.017406	0.070539	0	0	0.017408	0	0	0.052962
LOC10110	0	0.002733	0.002664	0	0	0.002716	0	0.005473
LOC10110	1.042734	1.473426	0.51487	1.307583	1.19381	0.690695	0.919115	1.141071
LOC10110	2.025555	2.179038	1.911963	2.93756	2.244147	2.118663	1.73992	1.818462
LOC10110	1.443943	1.285573	0.619353	1.808535	1.998443	1.116054	1.865312	2.84024
LOC10110	79.05183	61.76092	64.89058	61.97971	78.18136	68.79558	71.61648	64.02191
LOC10110	0	0	0	0.029692	0	0	0	0
LOC10110	1.566506	1.615154	2.423157	0.014206	0.464467	0.704858	2.441289	4.499274
LOC10110	2.055931	1.505472	2.392152	1.001279	1.26222	0.717319	2.107431	2.250363
LOC10110	18.11799	15.52856	18.56405	20.5017	20.36975	19.98905	19.96803	18.03796
LOC10110	0	0	0	0	0	0	0	0
LOC10110	0	0	0	0	0	0	0	0
LOC10110	0	0	0	0	0	0	0	0
LOC10110	0.046035	0	0	0.03145	0	0.01545	0.155751	0.031127
LOC10110	0	0	0	0	0	0	0	0
LOC10110	0.048133	0.024382	0.023766	0.073987	0.096278	0.024231	0.195419	0
LOC10110	0	0	0	0	0	0	0	0
LOC10110	32.04989	35.53859	30.78961	28.753	24.94111	27.6491	36.67556	38.20693
LOC10110	0.058863	0.208723	0.087194	0	0.05887	0	0.029873	0
LOC10110	0	0.106603	0	0.107829	0	0.052971	0.106801	0
LOC10110	0.531203	1.182468	0.930952	0.161008	0.853028	0.482102	0.394886	0.698118
LOC10110	0.127281	0.39975	0.213682	0.299999	0.267325	0.230672	0.348814	0.206549
LOC10110	0.132128	0.178483	0.369699	0.090267	0.308339	0.110859	0.111758	0.111674
LOC10110	0.017515	0.656565	0.242156	0.287184	0.297796	0.423235	0.088889	1.190219
LOC10110	0	0.026452	0	0	0	0	0	0
LOC10110	6.084601	4.487723	7.258613	6.684262	6.134071	4.998953	5.138324	5.875001
LOC10110	0	0	0	0	0	0	0.058455	0.058411
LOC10110	71.45043	83.4596	89.17062	80.99083	81.99878	83.30612	82.62379	76.48255
LOC10110	14.37914	14.62807	12.63947	13.69278	14.07681	12.54847	12.23156	13.64775
LOC10110	2524.117	2294.897	4053.4	3385.089	2711.296	3538.185	2526.906	2030.851
LOC10110	0.009595	0	0.018951	0.009833	0.009596	0.028982	0.009739	0.019464
LOC10110	0.301431	0.769831	0.223257	0.115837	0.288909	0.177036	0.089236	0.184707
LOC10110	0.010267	0	0	0	0.041075	0.010338	0.010421	0.020827
LOC10110	49.29537	130.8996	191.4585	283.4074	163.2821	286.1932	296.5744	157.1714
LOC10110	0	0	0	0	0	0	0	0
LOC10110	0.053211	0	0	0	0.026609	0.053575	0.027005	0

LOC10110	0	0	0	0	0.029907	0	0	0
LOC10110	1.541776	1.121443	2.069127	1.093823	1.265211	2.388167	2.447668	1.924584
LOC10110	0	0.040336	0	0	0	0	0	0
LOC10110	0.757668	1.444917	1.276389	1.096145	0.802341	0.224366	1.99044	0.452032
LOC10110	2.478911	2.351625	2.114202	2.240096	9.285851	8.780841	3.042188	7.154043
LOC10110	0	0	0.005744	0	0	0	0.017711	0.005899
LOC10110	0.022651	0.13769	0.05965	0.077374	0.211437	0.098825	0.107291	0.091894
LOC10110	0.96319	0.63142	0.801982	1.006402	1.114423	0.741589	0.345049	0.689578
LOC10110	1.692583	1.138001	1.276413	1.513753	0.892568	2.029483	1.780443	1.154856
LOC10110	0	0	0	0	0	0	0	0
LOC10110	113.2848	126.2989	136.2445	123.4849	90.34215	111.3444	125.2048	116.6465
LOC10110	149.1343	175.7343	200.932	187.8551	109.0219	178.2351	179.3835	120.9861
LOC10110	0.031896	0.161575	0.125996	0.261493	0.191404	0.128458	0.080937	2.005734
LOC10110	8.190668	8.255553	8.836211	6.723463	10.1161	12.78809	7.737238	9.64027
LOC10110	0.081364	0.082432	0.455317	0.027793	0.135624	0.136533	0.44045	0.357596
LOC10110	6.718705	6.683401	5.710776	5.624975	5.598557	5.123306	5.80551	7.891579
LOC10110	6.620614	6.053296	5.284736	6.518396	5.552533	5.94097	6.539118	5.969603
LOC10110	4.925698	11.48279	9.321929	4.828243	9.358388	4.627619	9.363768	8.137001
LOC10110	5.427386	8.95229	6.732922	4.412687	5.248647	6.006394	5.099052	4.503803
LOC10110	5.997678	6.666332	5.338319	6.912037	5.920804	6.116822	6.678677	6.348824
LOC10110	0.059509	0.130629	0.107741	0.091475	0.069436	0.159775	0.110737	0.040238
LOC10110	19.20912	19.93534	21.25847	20.096	18.56528	18.59994	20.40201	17.85526
LOC10110	0.008497	0.034434	0.025173	0.026122	0.008498	0.008555	0	0.008618
LOC10110	0	0	0	0	0	0	0	0
LOC10110	8.487905	8.750696	9.577485	7.840589	9.774308	8.214887	9.783132	6.941551
LOC10110	103.2804	114.733	105.778	108.8975	115.2852	111.3462	137.8472	119.1176
LOC10110	24.43757	27.56423	27.35082	31.82118	33.04387	28.41002	28.93816	33.31165
LOC10110	15.61225	13.41751	12.57604	11.82622	14.53064	10.96467	12.50119	16.58585
LOC10110	0	0.04884	0.009521	0.019761	0.038571	0.009707	0.029358	0.068451
LOC10110	2.175217	2.047099	1.486372	3.23272	2.392017	1.972107	2.69964	2.843989
LOC10110	0	0.016894	0.016467	0	0.008339	0	0	0
LOC10110	23.12805	34.55087	10.33161	17.86853	23.70478	14.53098	19.53694	18.11356
LOC10110	6.13073	8.25105	6.709185	6.535871	9.049573	5.960182	5.427264	8.730886
LOC10110	1.116099	1.93215	1.690742	2.87602	2.080764	2.356547	3.101564	2.307936
LOC10110	0.077885	0.143468	0.104884	0.145117	0.184116	0.114062	0.114987	0.323158
LOC10110	0.067201	0.017021	0.033182	0.172164	0.084012	0.03383	0.085261	0.136315
LOC10110	0.819021	0.793693	0.797094	1.04204	0.763727	0.788764	0.795163	0.926991
LOC10110	539.3432	458.6622	633.9921	520.513	474.2457	497.6171	564.1541	557.6948
LOC10110	0.152322	0	0.037606	0.058536	0.095214	0	0.057978	0.405537
LOC10110	3.287798	4.033342	2.908031	3.178802	3.109515	3.02241	3.119477	3.240363
LOC10110	0.459082	0.210048	0.292491	0.273166	0.207354	0.805154	0.165343	0.120159
LOC10110	0.647436	1.866887	1.032827	15.9744	0.996184	6.067305	0.758247	0.8587
LOC10110	0	0	0	0	0	0	0	0
LOC10110	21.79748	19.61169	15.82505	21.81849	21.25242	17.64518	21.68523	22.1358
LOC10110	0.337042	0.593853	1.794456	1.892142	0.542269	1.578691	2.647541	1.887553
LOC10110	0.194687	0.208845	0.079166	0.070415	0.137444	0.184487	0.104616	0.081307
LOC10110	0.434877	0.575207	0.465247	0.56944	0.24163	0.109462	0.613056	1.384463
LOC10110	3.443276	3.195266	2.933768	2.887009	3.585486	3.246779	3.422988	3.354518
LOC10110	0.121375	0.307421	0.179795	0.099506	0.352034	0.073323	0.394227	0.36931
LOC10110	0	0.077583	0.113435	0.117712	0.114881	0.077101	0.038863	0.077668
LOC10110	0	0	0.007001	0	0	0	0	0
LOC10110	0.176923	0.071698	0.139775	0.543915	0.566226	0.249384	0.179576	0
LOC10110	17.50322	14.86651	14.94887	14.97189	14.38807	15.41793	16.04598	14.34782
LOC10110	0.383612	0.241987	0.335945	0.534041	0.571872	0.313358	0.257128	0.462482
LOC10110	0.197107	0.199694	0.183201	0.403978	0.301496	0.09339	0.164758	2.657663
LOC10110	4.153231	3.780866	3.38818	3.639281	2.769178	4.545222	4.154435	24.05316
LOC10110	5.650395	6.064769	5.608827	5.416303	5.651125	5.571394	5.868526	5.227348
LOC10110	0	0	0	0	0.023211	0	0	0

LOC10110	0	0.025662	0	0.025957	0	0	0	0
LOC10110	0.271739	0.440489	0.107341	0.167082	0.163065	0	0.110326	0.220486
LOC10110	0	0	0	0	0.028622	0.028814	0	0
LOC10110	0.0237	0	0	0	0.023703	0.023861	0	0.024037
LOC10110	89.48856	100.8389	113.9985	90.61692	118.4079	101.9324	90.8179	78.95324
LOC10110	0.182043	0.28587	0.125843	0.149242	0.10924	0.082479	0.13858	0.15694
LOC10110	16.04369	17.70652	18.09422	17.68407	16.32146	17.09696	17.27297	18.24785
LOC10110	1.311156	2.087428	4.01658	12.55886	1.793037	3.313753	5.078856	1.112707
LOC10110	0.252534	0.127924	0.872855	0.474447	0.252566	0.169506	0.341762	0.213441
LOC10110	0	0	0	0.061287	0	0	0	0
LOC10110	4.252574	4.730776	4.762276	6.038414	4.78129	4.211665	5.134497	4.707776
LOC10110	0.036876	0.03736	0.018208	0.056685	0.018441	0	0.018715	0.037401
LOC10110	0	0	0	0.005405	0.005275	0	0.005353	0
LOC10110	0.443832	0.702589	0.575271	0.653812	0.887779	1.228877	0.619423	1.012838
LOC10110	0	0.083565	0	0.084526	0	0.027682	0	0.027886
LOC10110	0.995933	0.92782	0.633073	1.055796	0.412164	0.426451	0.45315	0.383146
LOC10110	0	0	0.015079	0	0	0.015374	0.015499	0.015487
LOC10110	3.6018	2.95235	3.140956	3.26818	3.39593	2.726295	3.647102	2.973027
LOC10110	0.005641	0.011429	0.027852	0.00578	0.062056	0.022717	0.017176	0.022884
LOC10110	7.667747	8.572003	9.623788	8.941497	7.064305	9.317397	9.6997	9.156057
LOC10110	0.653483	1.629684	4.725876	8.345114	2.312622	2.054819	4.602177	2.25347
LOC10110	0.160923	0.221262	0.113513	0.170799	0.212676	0.127304	0.070002	0.215676
LOC10110	124.442	175.0735	167.9426	117.9637	146.8249	139.3133	142.5324	121.4823
LOC10110	0	0.026378	0	0	0	0	0	0
LOC10110	0	0.01016	0	0	0	0	0	0
LOC10110	52.80933	44.18494	41.88737	46.55507	44.77072	49.62245	54.59117	50.75156
LOC10110	0	0	0	0	0	0	0	0
LOC10110	1.064227	1.320787	0.906458	1.79947	1.503415	0.93756	0.783138	1.349221
LOC10110	0.089351	0.067893	0.066178	0.045782	0.022341	0.067471	0.113364	0.022656
LOC10110	0	0	0	0	0	0	0	0.027036
LOC10110	20.95399	23.70179	25.02525	21.89807	21.94589	23.80756	22.90774	20.68194
LOC10110	0.523743	1.319427	0.814469	0.347529	0.457582	0.355588	0.843227	1.196725
LOC10110	20.59886	25.94283	22.16675	21.55752	21.76856	21.57177	22.73378	20.72772
LOC10110	0.060583	0.092067	0.119655	0	0.06059	0.121993	0	0.12289
LOC10110	1.34633	1.052053	1.138766	0.971343	1.16536	1.136698	0.735349	0.924618
LOC10110	1.328691	1.999684	1.701973	1.243203	1.473304	1.192358	2.042477	1.347606
LOC10110	1.465787	1.949613	1.382829	1.401396	1.384078	1.137768	1.654006	1.561402
LOC10110	62.00459	94.48985	71.43734	63.49222	58.55177	50.19851	65.27619	102.1142
LOC10110	0.032283	0.032706	0.031881	0.033082	0.096861	0.032503	0	0.065485
LOC10110	3.356167	4.180765	4.460821	4.628995	4.274916	4.994259	4.027822	3.350423
LOC10110	0.870601	1.040001	0.551782	0.865534	0.389872	0.431733	1.173817	0.513982
LOC10110	0.103164	0.072359	0.039184	0.081323	0.039684	0.03196	0.008055	0.032195
LOC10110	0.014093	0	0	0	0.014095	0	0	0.014294
LOC10110	11.26608	12.83292	14.0205	9.772587	12.72956	11.58963	11.48945	11.73696
LOC10110	0.613399	0.322233	0.605756	0.628593	0.568035	0.182989	0.184474	1.059927
LOC10110	0	0	0	0	0	0.073386	0	0
LOC10110	0	0.048021	0	0	0	0	0	0
LOC10110	0.511116	5.009385	2.841948	5.499655	3.744965	0.559356	0.620284	0.597278
LOC10110	0.030927	0.125333	0.015271	0.015847	0.092794	0.062277	0.031391	0.047051
LOC10110	282.1548	212.427	284.2832	229.9294	220.2892	226.0776	256.2771	279.7647
LOC10110	9.595133	4.625672	4.508861	6.496677	9.475411	10.07674	12.0306	10.68241
LOC10110	0	0	0.032404	0.067251	0	0	0.033305	0
LOC10110	66.12612	75.93394	94.44035	67.31195	67.92731	94.1752	67.73378	57.16677
LOC10110	0	0	0	0	0	0	0	0
LOC10110	1.510207	1.203621	1.183169	1.919043	1.329154	1.409019	1.757679	1.235576
LOC10110	0.122521	0.045974	0.004481	0.018601	0.081691	0.027413	0.013818	0.110458
LOC10110	0.460651	0.35469	0.327536	1.057418	1.732272	0.259727	0.261834	0.280325
LOC10110	0	0.00915	0	0	0	0	0	0

LOC10110	1.068858	9.544504	1.742868	0.458512	1.789947	2.803021	4.036803	4.285873
LOC10110	0.28697	0.232589	2.021548	0.117631	0.038268	0.057786	1.048589	0.329863
LOC10110	0	0	0	0	0	0	0	0
LOC10110	44.99564	38.71845	33.29877	38.66677	41.85406	32.8371	41.69396	45.66842
LOC10110	184.2769	210.717	243.0713	175.4441	174.0188	195.5275	192.3488	188.7083
LOC10110	0	0	0	0	0	0	0	0
LOC10110	0	0.017504	0	0	0	0.017395	0	0
LOC10110	0	0	0	0.124706	0.121708	0.061262	0	0.185137
LOC10110	0	0.045226	0	0	0	0.022472	0.022655	0
LOC10110	0	0	0	0	0.03598	0	0	0.036488
LOC10110	17.8049	20.49353	21.84876	17.67013	16.54279	19.80051	20.38889	16.98982
LOC10110	0	0	0	0	0	0	0	0
LOC10110	0.079636	0.291695	0.13309	0.13183	0.251194	0.148026	0.304671	0.416277
LOC10110	0	0	0	0	0	0	0	0
LOC10110	2.620499	3.219761	2.863151	3.656729	4.237951	3.761126	4.470739	4.806667
LOC10110	0.258014	0.234171	0.238874	0.336015	0.193536	0.189421	0.207325	0.256235
LOC10110	2.568816	2.602529	2.445051	2.53723	2.541817	2.267197	2.540778	2.838206
LOC10110	0	0.014715	0	0	0	0	0	0
LOC10110	0	0	0	0	0.028799	0	0	0.029205
LOC10110	0.286401	0.474806	1.208465	0.453583	0.755154	0.419429	0.766382	0.422513
LOC10110	0.178275	0.511741	0.293423	0.426279	0.32688	0.388901	0.542847	0.512303
LOC10110	0.174569	0	0	0	0.029099	0.029294	0.029531	0.059018
LOC10110	16.2863	25.59863	11.41919	16.42745	16.19533	28.32092	8.855674	13.04933
LOC10110	0	0	0	0	0	0	0	0.018209
LOC10110	4.742708	6.006189	5.570661	4.749728	4.922993	4.051607	5.069129	5.02887
LOC10110	0	0	0	0.063038	0	0.24774	0.062437	0.06239
LOC10110	39.98113	26.43565	50.78721	37.01277	48.75627	28.27582	24.41587	62.06334
LOC10110	0.14627	0.061019	0.059478	0.123441	0.01721	0.017326	0.043666	0.034906
LOC10110	1.921308	2.550207	1.7908	2.091505	2.752119	2.017102	2.623981	2.829006
LOC10110	35.75027	56.0319	37.92798	72.05622	78.09117	64.36747	76.32874	95.29057
LOC10110	0.736988	18.79924	15.25156	25.51044	0.818981	20.44685	21.46052	27.15842
LOC10110	1.117519	1.011189	1.179414	1.022813	1.057941	1.030675	1.359407	1.496817
LOC10110	0.050659	0.12831	0.150084	0.155742	0.101331	0	0.077129	0.02569
LOC10110	60.02476	48.41669	10.34541	66.44498	73.64501	52.9469	48.76608	63.16648
LOC10110	19.21387	22.93796	19.20103	23.05471	22.7298	21.12084	27.71042	26.22073
LOC10110	11.70356	10.4589	10.70362	9.787114	11.53943	9.411985	10.34753	10.41439
LOC10110	2.487633	1.746861	1.598764	2.42786	1.770529	1.39146	1.92377	2.216009
LOC10110	0.271739	1.160217	1.073414	0.994538	0.660024	1.035756	0.591033	1.968627
LOC10110	0.066505	0.192508	0.140735	0.233666	0.304063	0.057394	0.077146	0.462527
LOC10110	1.356489	1.232309	1.172465	1.335896	0.936178	1.280563	1.451986	1.150523
LOC10110	0	0	0	0	0	0.036598	0	0
LOC10110	0	0	0	0	0.039904	0.020086	0	0.020234
LOC10110	0.09783	0.084247	0.169069	0.110279	0.640869	0.241321	0.263138	0.163717
LOC10110	0	0	0	0	0	0	0	0
LOC10110	75.14103	89.32884	82.99006	77.59738	80.42408	85.05562	79.07369	77.29439
LOC10110	2.97336	3.184918	1.995419	3.24983	2.987554	2.905623	3.031975	3.529195
LOC10110	2.098495	1.957748	1.569298	2.025646	2.635918	1.761622	2.051293	2.32492
LOC10110	0.008869	0	0	0	0	0.008929	0	0
LOC10110	1.403151	1.920361	1.312737	1.513587	1.871109	1.660585	1.149353	1.423125
LOC10110	212.28	211.1905	227.3727	211.3275	216.4009	212.6842	222.9579	190.8101
LOC10110	2.020088	2.317835	0.937371	1.421653	0.365123	0.906673	1.333988	1.629202
LOC10110	0	0	0	0	0	0	0	0
LOC10110	1.915648	2.24502	1.615681	1.549256	2.019457	1.720223	2.133565	2.121456
LOC10110	2.772896	2.112868	2.220591	2.236651	2.120724	2.893503	2.440013	2.603609
LOC10110	0	0.009481	0	0	0	0.018843	0.009498	0.047455
LOC10110	1.029771	1.105882	1.444054	1.435179	2.018611	1.721097	1.191548	1.483091
LOC10110	9.443971	9.456298	9.664777	10.09813	10.12466	10.55609	8.324527	7.976837
LOC10110	0.021204	0.214822	0.209397	0.239021	0.084827	0.192139	0.043044	0.387104

LOC10110:	0	0	0	0.044573	0	0.021896	0	0.022057
LOC10110:	31.94134	35.69177	40.96476	46.94098	41.69758	27.51174	32.83147	28.74902
LOC10110:	21.13834	20.87673	17.03764	20.12428	18.26526	19.15808	18.91911	20.59648
LOC10110:	0.022056	0	0	0	0.022059	0.044414	0.044774	0.02237
LOC10110:	70.535	56.59617	33.19127	27.97353	44.74233	40.65984	66.91183	61.92065
LOC10110:	0	0	0.025953	0	0	0	0.026675	0
LOC10110:	3.388583	4.271045	2.793026	3.486191	1.974705	2.619243	4.576855	2.990306
LOC10110:	1.287406	0	16.43357	0.684079	2.622833	0.672104	14.37389	3.143432
LOC10110:	126.2358	153.7855	149.6465	139.9132	145.9143	158.9769	131.4376	130.6565
LOC10110:	0	0	0	0	0	0	0	0.019194
LOC10110:	44.77035	26.42239	109.0717	65.15874	14.81246	19.46895	116.1675	84.25717
LOC10110:	10.82408	6.096459	9.803767	6.507548	12.21217	5.779396	7.824672	8.156281
LOC10110:	0	0	0	0	0	0	0	0
LOC10110:	0.412786	0.336886	0.520876	0.164504	0.045871	0.196258	0.06983	0.139554
LOC10110:	0	0	0	0	0	0	0	0
LOC10110:	1.62077	1.369594	1.248879	1.385339	1.969889	1.22937	1.17295	1.33424
LOC10110:	98.05236	109.5841	118.9875	100.5737	114.0932	122.721	101.9058	94.0454
LOC10110:	9.2182	10.91033	18.64202	16.72707	18.89542	19.75123	9.96772	9.265594
LOC10110:	0	0	0	0.046487	0	0	0	0.046009
LOC10110:	95.30743	62.26735	82.49382	70.64166	68.36588	64.03046	72.04757	80.89005
LOC10110:	0	0.012687	0	0.012833	0	0	0	0.050806
LOC10110:	0	0	0	0.04903	0	0	0	0
LOC10110:	0	0.007109	0.041575	0.086285	0	0	0	0
LOC10110:	63.79756	72.94343	100.7604	85.50479	78.01772	90.16976	80.47759	46.57166
LOC10110:	0	0	0.019943	0	0	0	0	0
LOC10110:	0.272944	0.075416	0.147023	0	0.074449	0.024983	0.805929	0.100665
LOC10110:	21.48315	22.56372	20.88655	22.22009	21.31937	27.45379	20.34044	19.46933
LOC10110:	17.53906	17.02628	13.23179	15.95384	12.78928	16.21294	13.94088	16.20821
LOC10110:	0.009831	0.00996	0.014563	0.005037	0.009832	0.004949	0	0.004986
LOC10110:	297.1026	354.7933	354.0283	340.0567	310.3206	319.293	322.8147	308.0199
LOC10110:	0.023208	0	0.022919	0	0.023211	0	0.023556	0
LOC10110:	0.547544	0.271926	0.106024	0.47309	1.030804	0.108095	0.795496	0.196002
LOC10110:	0.012111	0.01227	0	0.024823	0.024226	0	0	0.012284
LOC10110:	0	0.032954	0.096364	0.016666	0.097593	0.016374	0.016507	0
LOC10110:	0.149996	0	0	0	0	0	0	0
LOC10110:	0.772143	0.653967	0.564831	0.799643	0.690524	0.703378	0.754698	0.650541
LOC10110:	0.227554	0.098803	0.032103	0	0.065024	0.06546	0.098986	0.065941
LOC10110!	8.522271	7.407046	8.00289	9.703745	7.686452	7.715791	9.834745	9.134958
LOC10110!	7.164944	5.614085	6.517979	7.306252	9.072062	7.782474	7.66649	6.622582
LOC10110!	0.031915	0.210168	0.220619	0.114468	0.414944	0.562322	0.161967	0.178029
LOC10110!	0	0.124918	0.284116	0.168473	0.123316	0.082762	0	0.125056
LOC10110!	5.289112	10.99303	14.68327	15.9347	12.37313	11.359	25.32157	12.86997
LOC10110!	0	0	0	0	0	0	0	0
LOC10110!	64.88816	72.68102	75.45742	61.61535	65.66474	74.20935	74.53075	72.66729
LOC10110!	0.01767	0	0	0.036215	0.017672	0.017791	0.017935	0
LOC10110!	0.66723	1.678313	1.488243	0	1.380655	0.011583	0.151795	0.070006
LOC10110!	225.8764	287.3317	265.0954	282.5269	255.3858	296.2683	267.0929	280.8895
LOC10110!	0.652697	1.614259	1.232254	1.554125	1.305562	0.869765	0.759912	1.577089
LOC10110!	1.316004	0.91053	1.077723	3.683986	1.348276	1.16341	1.075111	1.237075
LOC10110!	0	0	0	0	0	0	0	0
LOC10110!	1.697185	1.486312	2.499853	1.650789	1.438478	2.259064	2.423378	2.1298
LOC10110!	1.368663	2.268123	1.64124	3.045571	2.356361	1.958748	1.538035	2.548241
LOC10110!	0	0	0	0.034391	0	0	0	0
LOC10110!	0.12988	0.021931	0.042754	0	0	0.108973	0.065914	0
LOC10110!	16.06701	17.58149	16.71019	17.2935	21.35332	22.27602	16.90902	16.7808
LOC10110!	0.627292	1.059207	1.061958	0.64283	0.477998	0.781953	0.636701	0.515037
LOC10110!	0	0	0.030374	0	0	0	0	0
LOC10110!	0	0.01823	0.017769	0	0	0	0	0

LOC10110	0.015724	0	0.015528	0	0.031453	0	0.01596	0.015948
LOC10110	2.481328	1.909565	1.950107	1.995715	2.405374	2.317285	1.885455	2.665827
LOC10110	0.734218	0.95135	0.477019	0.696965	1.19036	0.529136	0.537351	1.736257
LOC10110	5.532742	5.308599	7.520764	5.169514	6.998196	6.815718	4.558654	5.050354
LOC10110	0	0	0	0.017971	0	0	0	0
LOC10110	0.034433	0.089704	0.024289	0.075613	0.118071	0.024763	0.059914	0.034923
LOC10110	0.587699	0.427952	0.272051	0.941026	1.138814	0.563977	0.736323	0.884784
LOC10110	4.555425	4.136246	3.397774	4.091087	4.036494	3.951893	4.259425	4.688351
LOC10110	1.372089	1.286579	0.994622	0.987245	1.350369	1.102231	1.370447	1.894975
LOC10110	0.011743	0.029742	0.017395	0	0.023489	0.017735	0.029798	0.01191
LOC10110	74.92751	7.064661	0.267057	49.62522	13.21398	4.62866	79.99222	59.16534
LOC10110	1.728099	1.865458	1.408476	1.469309	1.743417	1.352415	1.432322	1.760353
LOC10110	7.090039	7.266613	7.612309	8.448444	8.080391	6.930961	8.409734	7.776281
LOC10110	0.669635	0.8238	1.06279	0.988487	0.82918	0.963157	0.712046	0.614485
LOC10110	0.882983	0.899305	0.659753	1.03412	0.90646	0.973683	0.815615	1.184594
LOC10110	0	0.01241	0	0.05021	0	0	0.012433	0.024847
LOC10110	15.29436	19.6238	16.11714	17.16541	14.65125	16.47135	15.742	18.59468
LOC10110	0	0.022171	0	0	0	0.007344	0.066636	0.014797
LOC10110	0	0.022961	0	0	0	0	0	0
LOC10110	1.152963	0.765303	0.549667	0.977811	0.79525	1.681215	1.291318	1.008083
LOC10110	1.048454	1.524046	1.170441	1.214567	0.957408	1.422788	1.202987	1.340783
LOC10110	0	0	0	0	0	0.01769	0	0
LOC10110	58.25745	63.43753	56.54439	120.3434	120.8488	59.62162	64.73194	124.8244
LOC10110	0	0.019396	0.018906	0.019619	0.019147	0	0	0
LOC10110	1.38581	1.813497	0.907178	1.732137	1.30199	1.537976	1.433237	1.639794
LOC10110	0.097736	0.074264	0.060324	0.300471	0.048874	0.123005	0.124003	0.223037
LOC10110	10.11164	14.88941	13.1621	13.56726	16.88454	13.43712	15.29577	17.46514
LOC10110	143.0825	186.8951	180.5725	188.1669	205.0976	174.1618	151.3959	247.0567
LOC10110	0	0	0	0	0	0	0	0
LOC10110	12.75925	13.21277	12.60026	16.56566	14.13759	12.86418	13.27306	12.42182
LOC10110	0.029247	0	0	0	0	0	0	0
LOC10110	0	0	0	0	0	0	0	0
LOC10110	0.31017	0.733229	0.685541	0.469212	0.38407	0.401515	0.524705	0.194743
LOC10110	0.016392	0.033214	0.024281	0.008399	0.008197	0	0.024957	0
LOC10110	12.33458	12.36281	10.12903	13.65403	13.00686	10.38224	12.36338	12.72202
LOC10110	0	0	0	0	0	0	0	0
LOC10110	0.335729	0.644466	0.401344	0.162969	0.406461	0.462557	2.761986	1.863833
LOC10110	1.83517	1.882068	1.612172	1.817172	1.689025	1.337604	1.457018	2.135351
LOC10110	0.250747	0.158313	0.071775	0.145237	0.119938	0.150012	0.173361	0.136373
LOC10110	3.192939	1.995967	3.891126	2.575853	1.358873	2.530762	4.275141	2.82498
LOC10110	0.03103	0.031437	0.030643	0.063597	0	0.031242	0.062991	0
LOC10110	0	0.019115	0.055896	0.096672	0.047174	0	0.009575	0
LOC10110	0.06138	0.994965	0.303075	0.503201	0.245551	0.092699	0.093451	2.21
LOC10110	0.070769	0	0	0	0.035389	0	0.035915	0.035888
LOC10110	116.0569	104.6075	100.5246	97.90495	99.44301	103.7129	109.3685	105.1275
LOC10110	3.950851	4.955153	5.264841	5.256013	4.736874	5.03221	4.650283	4.429529
LOC10110	0.110085	0	0.108714	0	0.137624	0	0.027934	0.027913
LOC10110	1.997632	2.006889	1.71375	1.440985	2.176472	1.477559	1.795386	2.507119
LOC10110	3.258542	2.814799	2.867919	2.776856	3.098874	1.968482	2.216552	2.643944
LOC10110	10.36489	8.490248	7.201484	7.959469	8.961647	7.919714	7.890274	9.295353
LOC10110	1.829612	1.727419	1.314746	1.723341	1.993366	1.685335	1.77013	2.029378
LOC10111	3.912573	3.0452	2.213648	3.664924	3.892699	3.354562	2.947421	3.833929
LOC10111	0.484242	2.256745	1.530265	0.396989	1.065469	1.560159	0.884709	1.178724
LOC10111	0.424025	0.177517	0.363371	0.337568	0.353984	0.314017	0.231199	0.312772
LOC10111	181.3261	203.0954	237.1287	185.4053	195.2597	220.3508	220.4469	199.0047
LOC10111	0	0.012536	0	0	0	0	0	0
LOC10111	58.44082	62.35266	59.04842	58.9821	59.22884	59.64322	63.03137	56.14204
LOC10111	0	0	0.062677	0.06504	2.856406	0.191703	0.837454	4.634712

LOC10111	1.251715	1.579981	1.398232	2.376187	2.113824	1.363566	1.603734	4.016722
LOC10111	4.324928	5.091223	3.154744	4.671288	3.477593	2.140125	3.417062	4.236956
LOC10111	0.10392	0.245663	0.478918	0.106494	0.138578	0.139507	0.03516	0.281065
LOC10111	0	0.018461	0	0	0	0	0	0
LOC10111	0.730505	0.397457	0.327303	0.42282	0.507361	0.633343	0.34327	0.29499
LOC10111	0.70663	0.618958	0.676018	0.433726	0.533722	0.822622	0.709758	1.011577
LOC10111	0.086707	0.035138	0.017125	0.17771	0.121406	0.05238	0.035203	0.123118
LOC10111	15.89328	15.05407	14.37417	14.29861	15.27922	14.6595	14.02993	15.13899
LOC10111	1.932334	2.692664	1.953846	2.149157	1.840242	2.051776	2.463363	2.214019
LOC10111	12.89553	11.36805	10.41942	11.62745	15.22121	12.22484	13.04647	11.78394
LOC10111	0.072292	0.122068	0.011899	0.037041	0	0.024262	0.232359	0.195524
LOC10111	14.56153	14.57125	16.61961	13.76024	10.44507	13.45928	14.11364	10.35028
LOC10111	147.4297	123.5083	124.1144	176.7827	120.6198	145.6895	152.7773	138.6821
LOC10111	0.906065	1.101547	2.993429	1.468731	1.235703	0.9786	5.885797	5.647446
LOC10111	0	0.063366	0	0.032047	0	0	0.031742	0.253744
LOC10111	0	0.006815	0.013285	0.041359	0.013455	0	0.013655	0.006822
LOC10111	304.7631	265.0666	305.8946	254.6608	273.3421	269.0367	255.0708	260.7775
LOC10111	0.030589	0.144623	0.030208	0.156735	0.081582	0.092395	0.020699	0.144782
LOC10111	0.723234	0.879271	0.3214	0.469394	0.048222	0.327679	0.244694	0.366765
LOC10111	1.188054	0.743429	0.759162	0.537125	0.943577	0.369406	0.727072	1.027766
LOC10111	12.90506	12.00275	10.69682	10.16786	11.06593	10.52238	11.87466	10.62121
LOC10111	0	0.025251	0.049226	0.051082	0.049854	0	0.505948	0
LOC10111	62.21504	60.75071	70.30279	99.54686	126.2262	119.108	57.32751	44.64877
LOC10111	3.577742	3.569252	2.489428	2.961834	3.543996	2.909986	2.919708	2.858535
LOC10111	1.646421	2.694508	3.960542	2.11982	1.604413	2.507756	2.528101	2.012395
LOC10111	7.838761	14.24645	16.22083	18.54932	20.28766	14.05253	11.70676	8.481386
LOC10111	7.570366	6.615865	5.345462	6.158933	5.625528	4.984416	6.804083	6.544975
LOC10111	0.022214	0	0	0	0	0.022365	0.112734	0.04506
LOC10111	2.928134	3.585097	2.493608	2.927774	2.513541	2.255862	2.677257	2.627146
LOC10111	0	0	0	0	0	0.009805	0	0
LOC10111	0	0	0	0	0	0	0.058575	0
LOC10111	0.047399	0.048021	0.023404	0.024287	0.023703	0.143169	0.024055	0
LOC10111	40.94685	46.93239	50.52109	54.0526	51.19375	63.69024	45.23375	36.10463
LOC10111	0.006044	0.012246	0.005969	0.012387	0.006045	0	0.042942	0.01226
LOC10111	8.053757	6.93325	5.964041	6.390699	6.475024	4.866092	6.737872	7.981154
LOC10111	0	0	0	0	0	0	0	0.060143
LOC10111	0	0	0	0.016832	0	0	0.016672	0
LOC10111	1.065771	1.516802	0.751779	1.976308	2.537877	1.839517	1.493855	2.650883
LOC10111	141.5617	185.165	136.4924	153.2886	150.0067	104.8775	152.5886	102.371
LOC10111	0.178275	0	0	0	0	0	0	0
LOC10111	3.679804	3.680606	2.711963	3.138458	3.660745	3.425695	3.243345	3.767846
LOC10111	0	0	0.014321	0.014861	0	0	0	0
LOC10111	1.384623	2.237792	1.448761	1.85811	2.439889	0.995773	1.455585	2.507739
LOC10111	14.18248	14.93263	13.81334	15.18987	12.27729	13.55068	13.95728	13.24096
LOC10111	54.78784	59.76676	38.57151	54.61349	56.31489	56.30974	57.51216	57.26333
LOC10111	2.192485	1.984955	3.31685	1.338515	1.492948	2.019593	2.27272	1.845195
LOC10111	118.273	133.5835	138.7869	124.2772	141.5432	130.6472	122.2909	111.7086
LOC10111	10.65024	7.125478	7.796014	8.825374	8.239927	10.49177	7.692284	6.434525
LOC10111	0.020611	0.020881	0.061061	0.042242	0.020613	0.041503	0	0.020904
LOC10111	2.519247	2.788426	2.011109	2.456547	2.57507	2.402371	1.954386	2.63953
LOC10111	0.047927	0	0	0	0.287597	0	0.291873	0
LOC10111	0	0.068329	0.466224	0.069114	0.26981	0.067905	0.068455	0
LOC10111	0.13925	0.126969	0.178769	0.456636	0.389949	0.252362	0.212007	0.112986
LOC10111	6.022329	6.083884	5.282693	7.055674	4.141965	5.924481	7.986748	7.823219
LOC10111	1.633171	2.053991	2.057737	1.327358	2.534558	1.81443	2.97237	2.056245
LOC10111	523.7294	828.3941	628.6555	463.2623	441.3212	758.6889	470.9474	143.1569
LOC10111	0	0	1.251436	0.112923	0.055104	0.332839	0.111846	0.279406
LOC10111	0.02436	0.024679	0	0.024963	0.146176	0.269786	0.04945	0

LOC10111	4.163409	4.929845	4.01838	4.406533	4.697452	3.828339	4.559306	5.004532
LOC10111	3.246327	14.39962	13.00846	12.4966	12.21692	18.50057	4.435606	20.85276
LOC10111	14.5539	13.03908	11.38872	11.98353	13.67921	11.2048	11.08501	13.32241
LOC10111	0.019263	0.243948	0.275833	0.217142	0.038531	0.465471	0.293279	0.253984
LOC10111	0	0	0	0	0	0	0	0
LOC10111	0.064827	0.032839	0.048015	0.033217	0.032418	0.040794	0.041125	0.082188
LOC10111	2.716393	10.5553	5.704656	8.52722	2.338463	8.377949	7.119701	7.53283
LOC10111	9.741448	9.944378	8.351488	11.40885	9.792121	9.857742	9.369371	9.429129
LOC10111	6.307417	2.738655	11.53074	6.983055	2.515783	13.13572	4.210858	3.84594
LOC10111	1.174077	0.977078	0.869586	1.03128	0.587115	1.160989	0.851206	0.871829
LOC10111	0	0.022084	0	0	0	0.021947	0	0
LOC10111	5.185757	4.541552	4.529976	4.900486	4.60861	5.249217	4.910285	5.817306
LOC10111	28.83597	27.72434	52.552	26.4445	33.96576	22.41417	27.32331	27.75476
LOC10111	0.040682	0.041216	0.120525	0	0	0	0	0.082522
LOC10111	1.085701	1.412779	0.954135	1.184045	1.534124	0.872488	1.314295	1.404227
LOC10111	0.010683	0	0	0	0.010684	0	0	0
LOC10111	0.109084	0.12433	0.134656	0.153706	0.240925	0.045762	0.064587	0.276592
LOC10111	0	0	0.01773	0	0	0	0	0
LOC10111	4.05363	3.78271	4.06862	3.946138	3.905879	3.794569	4.039195	3.446558
LOC10111	0.395917	0.445682	0.553894	0.484615	0.527958	0.376477	0.323718	0.568868
LOC10111	4.910925	4.786438	4.757651	5.542198	5.50219	5.44518	4.132792	5.028122
LOC10111	14.61979	14.81166	13.28548	14.21538	14.41511	12.92116	15.62665	16.28296
LOC10111	0.63299	0.511179	0.398616	0.235025	0.311948	0.517241	0.47488	0.353566
LOC10111	0.156146	0.158195	0.1542	0.306693	0.299318	0.183415	0.686782	0.461909
LOC10111	0.013116	0	0	0	0	0	0	0
LOC10111	0.028045	0.08524	0.193871	0	0.308538	0.141184	0.398523	0.426667
LOC10111	0	0.018656	0.054553	0.01887	0.073665	0	0.074761	0.037352
LOC10111	62.62922	62.95984	65.85124	61.34072	65.47817	77.91455	64.93988	59.69127
LOC10111	1.863964	1.602529	1.503392	1.53724	1.782507	1.772021	1.68841	2.086327
LOC10111	0	0	0	0	0	0	0	0
LOC10111	5.52282	6.19378	6.009591	6.332243	6.311271	6.211955	4.006764	4.812104
LOC10111	0	0	0.038658	0	0	0.118239	0	0.079406
LOC10111	1.04267	1.221409	0.032177	1.535964	0.097763	2.624482	0.066144	1.751506
LOC10111	27.92185	25.39672	33.95557	28.00645	26.82137	25.01533	27.13818	28.07356
LOC10111	0.102977	0.166926	0.040678	0.168845	0.061794	0.041472	0.041809	0.104443
LOC10111	0	0	0	0	0.020059	0.010097	0	0.010171
LOC10111	0.073079	0.888454	0.396925	1.497779	1.863749	0.331101	0.556312	5.929527
LOC10111	0	0	0	0	0	0	0	0.029056
LOC10111	5.113761	5.509074	4.542753	5.709935	5.197734	5.446901	4.626797	4.919017
LOC10111	4.179959	4.282745	3.981047	4.844473	5.491763	3.718598	3.988853	5.521241
LOC10111	0	0	0	0	0.037574	0	0	0
LOC10111	41.02568	40.57922	38.11725	38.39109	41.07594	37.87745	40.82546	42.70394
LOC10111	0.004852	0.009831	0	0.004972	0.024261	0	0.004924	0.004921
LOC10111	0.02283	0	0	0	0	0.022986	0	0
LOC10111	0.18914	0.29453	0.089933	0.183057	0.238207	0.183379	0.231083	0.234462
LOC10111	0.009079	0.009198	0.044827	0.083731	0.054478	0.073125	0.018429	0.08287
LOC10111	0.388738	0.447546	0.540942	0.669982	0.388789	0.747207	0.878814	0.358429
LOC10111	0.325455	0.439636	0.1607	0.389103	0.361664	0.018204	0.403746	0.42178
LOC10111	1.020916	0.813506	0.660802	1.434123	1.177838	1.231929	0.877109	0.934619
LOC10111	21.75689	20.70062	16.9819	19.82215	19.75678	18.41053	20.73895	20.90208
LOC10111	0	0	0	0	0	0	0	0.040323
LOC10111	0.806845	0.408717	0.522036	1.083433	0.4313	0.602264	0.423593	0.705457
LOC10111	0	0	0	0	0	0.017813	0	0
LOC10111	0	0	0	0	0	0	0	0
LOC10111	0	0	0	0	0	0	0	0
LOC10111	18.28333	17.47116	16.06359	16.24965	17.01737	15.73396	16.92581	14.77614
LOC10111	0.234359	0.152637	0.132251	0.068618	0.485521	0.067417	0.237874	0.254674
LOC10111	4.30163	5.366977	4.368943	5.202827	4.024626	4.667965	4.656127	5.53844

LOC10111	0	0.055783	0.018125	0	0.036712	0.110874	0	0.03723
LOC10111	0	0	0	0	0	0	0	0
LOC10111	7.200643	7.781487	6.650028	7.570313	8.455181	7.115578	6.94322	7.127332
LOC10111	2.255843	3.089588	5.610592	3.895678	2.548597	2.649796	2.289681	2.796391
LOC10111	1.277655	1.418688	1.258371	1.127746	1.611757	1.245219	1.576933	1.793447
LOC10111	0	0	0.051472	0	0	0	0	0.052863
LOC10111	5.334123	6.861421	4.959886	5.539954	5.958207	6.529157	7.056626	5.872041
LOC10111	0.040418	0.10237	0.878107	0.248512	0.626553	0.122081	0.082048	0.286951
LOC10111	0.903832	0.405307	0.512131	0.334047	2.148731	0.566889	0.496293	0.736365
LOC10111	10.66484	17.94282	15.58348	13.79726	13.12765	14.43029	12.93099	14.29169
LOC10111	0.982076	1.016594	0.632504	2.516006	0.555158	0.343925	2.622036	2.836595
LOC10111	0.936257	0.915262	0.665057	0.471308	0.394265	0.694587	0.300095	0.699695
LOC10111	0.029431	0	0	0	0	0.029632	0	0
LOC10111	0	0	0	0	0	0	0	0
LOC10111	4.239535	3.252657	4.064767	8.6891	0	3.439663	3.175124	3.548451
LOC10111	0.393787	0.358037	0.458679	0.858818	0.393838	0.528636	0.77889	0.624691
LOC10111	1.977434	2.198	2.421465	3.010675	1.77427	1.774783	2.557613	3.220395
LOC10111	0.0147	0	0	0	0	0	0	0
LOC10111	0.011971	0.060643	0.011822	0.012268	0	0.012053	0.048604	0.024284
LOC10111	0	0	0	0	0	0	0	0
LOC10111	0	0	0.018293	0.018982	2.815952	0.01865	0	0
LOC10111	6.055051	4.660008	4.976224	4.868351	4.823382	4.93865	4.184353	5.006301
LOC10111	0	0	0	0	0	0	0	0
LOC10111	0.011205	0.261089	0.055325	0.126304	0.089649	0.439966	0.17059	0.261375
LOC10111	1.254807	2.907453	1.514543	1.881208	2.498318	2.00035	1.721758	2.038629
LOC10111	1135.196	1357.193	1562.943	1486.136	1527.72	1565.095	1376.461	1186.497
LOC10111	3.096327	3.879928	4.0837	5.010038	3.483818	4.142966	3.742379	3.264368
LOC10111	1.331742	1.509297	1.448893	2.359364	1.986585	3.27256	1.947391	3.250839
LOC10111	49.27211	39.12976	46.81854	51.06529	56.09424	52.45332	39.70643	34.68547
LOC10111	0.017771	0	0	0	0.008887	0	0.009019	0.027036
LOC10111	3.809176	1.677899	5.978762	5.166993	1.049039	4.001947	4.146482	1.717068
LOC10111	0.481262	1.334423	0.091718	0.89119	1.1062	0.229523	0.359933	0.291155
LOC10111	0	0	0	0	0	0.009449	0	0
LOC10111	10.30134	9.329119	9.976718	10.79411	11.56152	11.77239	11.6998	11.69099
LOC10111	49.31796	2.394372	9.123459	48.518	8.888148	2.595819	58.64189	51.58502
LOC10111	38.4851	37.35968	42.32439	40.83666	47.16784	41.03767	40.02118	42.40399
LOC10111	1.205497	1.620595	1.541514	2.351931	1.901222	2.170713	2.227542	2.743143
LOC10111	0.482347	0.47547	0.257479	0.40078	0.404181	0.249384	0.304335	0.19833
LOC10111	0.82469	0.988351	0.754823	1.288289	1.207019	0.941708	1.000389	1.152641
LOC10111	0.908292	1.024881	0.488872	0.692579	1.003125	1.014181	0.90007	0.816438
LOC10111	0	0	0	0	0	0	0	0
LOC10111	2.683892	2.377284	1.605416	1.477343	1.994006	2.192665	2.194887	2.737654
LOC10111	2.015447	2.339075	5.223459	10.44321	6.472976	7.888218	3.9569	2.044138
LOC10111	1.349928	1.387323	1.102931	1.13456	1.194695	1.153811	1.429322	1.822244
LOC10111	1099.297	963.4011	1285.022	1070.036	924.4249	1095.649	1057.382	1037.949
LOC10111	0	0.051745	0	0	0	0	0.051841	0.103604
LOC10111	0.727937	1.216859	0.287547	1.118952	0.837235	1.062719	1.108284	0.44298
LOC10111	1.524613	1.406298	1.179774	1.504081	1.604464	1.191365	1.789998	1.453999
LOC10111	5.347451	5.363273	6.499471	1.539506	5.419689	6.446373	8.259486	5.822634
LOC10111	0	0.017301	0	0	0.034158	0	0.069333	0.01732
LOC10111	0	0.072616	0	0	0	0	0.018188	0
LOC10111	0.274608	0.042153	0.189008	0.221717	0.582576	0.171755	0.105578	0.151918
LOC10111	0	0.046793	0	0	0	0.093005	0.04688	0
LOC10111	14.03984	21.64561	13.51855	20.49162	20.80224	19.35834	16.19681	16.85586
LOC10111	7.587855	7.7237	7.245889	7.225634	8.14368	7.063112	7.247565	7.986284
LOC10111	10.71795	8.376645	7.741735	9.037801	9.433016	9.927878	12.05984	11.119
LOC10111	0	0	0	0.01071	0	0	0.010608	0.021201
LOC10111	0.213332	0.144088	0.175562	0.18218	0.149352	0.393781	0.303145	0.490436

LOC10111	0.007036	0.007128	0	0	0	0	0	0
LOC10111	14176.56	12744.57	17276	14385.08	13870.09	15918.12	13454.29	12780.16
LOC10111	0	0.018166	0	0	0.035865	0.018053	0	0
LOC10111	0.016973	0	0	0	0	0	0	0
LOC10111	0	0.008289	0	0	0	0	0	0
LOC10111	0.297316	0.434314	0.471143	0.333023	0.262778	0.250616	0.301775	0.28051
LOC10111	0.85995	0.833079	1.48771	2.457211	4.633028	1.295577	1.274232	1.426065
LOC10111	4.862764	5.09844	7.8299	4.93171	8.733999	7.755138	7.996608	8.971886
LOC10111	0	0	0	0	0	0	0	0
LOC10111	0	0	0.845121	1.392854	0.050347	0	0.204381	1.225364
LOC10111	0.585547	1.269517	0.971462	1.248107	0.726173	0.943276	1.176775	0.736415
LOC10111	92.78891	28.68895	60.59923	87.84747	82.68489	75.21245	48.88308	42.89465
LOC10111	0	0	0	0	0	0	0	0
LOC10111	2.811348	4.068919	2.455247	2.95938	3.156002	2.994225	3.387339	3.084134
LOC10111	2.75038	2.450029	1.757483	2.452015	2.886995	1.971855	2.19528	2.426808
LOC10111	181.0746	149.6978	205.7444	185.7082	123.3366	174.2734	186.5409	148.2212
LOC10111	0.035251	0	0	0	0	0	0	0
LOC10111	12.98955	14.41839	8.492499	7.063737	13.62657	13.29785	15.03213	8.414349
LOC10111	0	0	0	0	0.124412	0	0	0
LOC10111	0.654292	0.245511	0.191449	0.446999	0.169653	0.268385	1.033055	1.302635
LOC10111	0.122408	0.051316	0.091704	0.138416	0.029551	0.038248	0.072833	0.089902
LOC10111	5.071055	5.912678	4.004137	5.599857	4.361234	4.797596	4.559196	4.234309
LOC10111	1.45901	2.383152	1.293807	0.945911	1.280521	0.779457	1.329783	1.026786
LOC10111	0	0	0	0.007452	0	0	0	0.007375
LOC10111	1.67208	3.351222	2.261488	2.607497	4.253449	3.513397	1.438898	2.470091
LOC10111	0	0	0	0	0	0	0	0
LOC10111	2.759427	2.068677	2.449211	1.949342	1.560506	2.44857	2.483099	1.885344
LOC10111	1.706656	3.105382	0.822471	1.748931	1.986805	1.044736	2.300436	1.952514
LOC10111	750.6578	945.5575	1094.254	884.5732	842.8136	1033.377	930.2484	926.3652
LOC10111	0	0	0	0	0	0	0	0
LOC10111	0.058708	0.104088	0.014494	0.082723	0.102753	0.073887	0.104281	0.037215
LOC10111	0.031099	0.504115	0.122846	0.701127	0.279928	0.187869	0.189393	0.283876
LOC10111	0.25298	0.312018	0.213621	0.311848	0.337351	0.214103	0.208397	0.635876
LOC10111	9.138129	9.544998	10.01142	10.16691	9.747487	10.91989	9.714864	9.158383
LOC10111	0.110892	0.134817	0.065706	0.113639	0.066544	0.06699	0.045022	0.022494
LOC10111	0	0	0	0	0	0	0.028017	0
LOC10111	0	0	0	0	0.014377	0	0	0
LOC10111	4.427847	5.12447	4.029564	4.587207	4.4365	3.888631	4.617283	4.769512
LOC10111	175.372	140.3804	166.2895	161.1732	157.5435	192.9131	162.3278	147.6575
LOC10111	0	0	0	0.010964	0.021401	0.043089	0	0
LOC10111	44.83355	56.68721	54.82853	47.61289	64.35795	28.51341	68.64648	53.57516
LOC10111	0.054401	0	0.18803	0.139371	0.108815	0.027386	0.16565	0.220701
LOC10111	0.028885	0.097546	0	0	0.077036	0.126022	0.019545	0.068357
LOC10111	12.48451	10.19275	18.02801	12.47835	13.58275	17.19874	11.63695	13.30608
LOC10111	0.266103	0.310443	0.167228	0.338802	0.669375	0.511485	0.417418	0.335319
LOC10111	0.057238	0.057989	0.056524	0.058655	0.22898	0	0	0.116105
LOC10111	0	0	0	0	0	0	0	0
LOC10111	2.62801	0.417647	2.289939	1.566578	0.197556	1.798563	0.095888	0.209053
LOC10111	13.80459	13.71153	11.67405	23.78026	33.23004	25.40793	19.88155	24.92957
LOC10111	12.3659	13.96435	12.68093	14.14033	13.95869	12.90588	15.80404	14.01026
LOC10111	9.111163	9.756627	4.991193	8.874906	11.29821	8.925848	9.150777	5.583312
LOC10111	0.007827	0.00793	0	0	0.023484	0	0.023833	0
LOC10111	2.921356	2.937933	2.397058	2.744242	3.251144	3.157586	3.190471	3.26069
LOC10111	0.216133	0.364948	0.177866	0.313772	0.018013	0.562157	0.127968	0.182674
LOC10111	1.087248	1.010566	0.64028	0.879078	0.927772	1.245319	1.255423	0.667706
LOC10111	3.535099	9.555454	8.211346	6.633048	5.008112	4.869798	7.234008	6.6875
LOC10111	0	0	0	0	0	0	0	0
LOC10111	59.21845	67.00327	67.71675	65.30088	62.99525	72.90341	72.25869	56.49554

LOC10111	0	0	0	0	0	0	0	0.03004
LOC10111	1.186717	3.782821	2.158068	4.315717	0.07237	2.695641	1.292654	3.581477
LOC10111	1.458829	2.38882	1.758935	1.807865	2.188527	1.485874	1.687323	1.823689
LOC10111	8.75295	4.307229	4.665697	5.665843	5.144291	5.301258	5.371709	6.025769
LOC10111	5.34584	3.04536	3.963867	4.131751	7.578753	2.917711	4.877944	3.705906
LOC10111	0	0.189254	0.338203	0.159524	0.124551	0.062693	0.063201	0.221038
LOC10111	3.0177	2.5796	2.048818	3.846234	2.131526	2.430652	2.641807	2.965013
LOC10111	0	0	0	0	0	0	0	0
LOC10111	0	0.007214	0	0	0	0	0	0
LOC10111	0	0	0	0.027579	0	0	0	0
LOC10111	0.138218	0.070016	0.204744	0.070821	0.69118	0.208744	0.911894	0.210279
LOC10111	0.022284	0.011288	0.03301	0.102764	0.111436	0.13462	0.316661	0.327723
LOC10111	0.022693	0.036785	0.076194	0.069764	0.081704	0.022848	0.036853	0.092063
LOC10111	0	0.449212	0	0	0.203248	0.055803	0.112511	0.093688
LOC10111	0.019223	0.058427	0.018984	0.157596	0.038452	0.077419	0.019512	0.058491
LOC10111	697.749	719.7545	1065.145	1042.669	1085.288	1266.235	852.2926	872.3887
LOC10111	0.562031	15.82953	4.717741	20.56153	16.46965	2.263484	14.09041	11.17263
LOC10111	0.014002	0	0.027654	0.014348	0.028007	0	0.071058	0.014201
LOC10111	0.343727	0.273615	1.430513	1.081882	0.589322	0.271916	1.44537	1.120563
LOC10111	7.141863	7.001242	8.823232	7.3484	8.82004	7.947564	6.779421	6.334425
LOC10111	15.56019	74.09591	13.7571	60.88475	42.64426	36.57658	19.0042	21.69578
LOC10111	9.528715	18.24911	13.21933	30.52953	35.55933	15.28074	7.003204	36.58485
LOC10111	2.718784	2.62399	2.628383	2.404869	3.048294	2.233107	3.355051	3.120315
LOC10111	32.06821	40.44647	28.1118	31.55071	31.59495	41.30937	42.28317	41.30507
LOC10111	31.66127	42.32912	41.55851	49.82913	50.92515	29.63454	27.02927	26.6451
LOC10111	0	0.027846	0.004524	0.009389	0.009163	0.004612	0	0.055753
LOC10111	0	0	0.03855	0	0.019521	0.019652	0.019811	0
LOC10111	0	0.052904	0.206272	0.160536	0.208901	1.209232	0	0.26481
LOC10111	0	0	0	0	0	0	0	0
LOC10111	8.603718	8.79894	8.04189	8.951625	7.393809	7.225237	8.99193	8.110185
LOC10111	0.028415	0.086365	0.028061	0.116477	0	0	0.028842	0.05764
LOC10111	8.606105	9.338804	7.626521	8.733284	9.28749	7.808858	8.683642	11.17003
LOC10111	1.242648	1.238973	1.071334	1.09151	1.361171	0.57592	1.001022	0.26007
LOC10111	836.0841	542.8599	642.1903	942.9424	842.7845	1020.288	984.3794	779.1875
LOC10111	0.968179	0.936299	2.694506	1.037259	0.528166	0.841867	1.206043	0.535615
LOC10111	0.356594	0.269185	0.220958	0.243618	0.181817	0.260473	0.404525	0.666609
LOC10111	0	0	0.061151	0	0	0	1.131332	0
LOC10111	0.089518	0.120923	0	0	0	0	0.030287	0
LOC10111	0	0.007992	0.00779	0	0.015779	0.03177	0.008007	0.008001
LOC10111	0	0	0	0.079015	0	0.038816	0	0
LOC10111	0.310301	0.314374	0.143003	0.445182	0.889645	0.520702	0.419941	0.293737
LOC10111	0.020997	0.106363	0.103677	0.021517	0.063	0.084562	0.213121	0.10648
LOC10111	0.235203	0.166803	0.098716	0.241029	0.182306	0.088804	0.143239	0.262406
LOC10111	30.8511	16.97133	23.12875	30.40449	26.96866	21.65077	27.1831	20.87794
LOC10111	12.8974	13.45682	12.19135	13.12007	11.49652	12.46608	13.9685	12.03452
LOC10111	0.793586	0.865847	1.446827	0.938358	1.404218	0.921932	0.588627	3.900587
LOC10111	1.838158	1.786492	1.382548	1.59347	1.977344	1.608616	1.420992	2.249114
LOC10111	0.474758	0.644963	0.319666	0.326188	0.404676	0.651821	0.520211	0.470573
LOC10111	2.204991	1.621401	1.475092	2.624063	0.889224	1.324871	3.429295	1.08212
LOC10111	47.00302	54.04425	60.28486	69.60553	52.71878	56.49577	54.92213	39.26855
LOC10111	53.5	56.27532	56.24634	58.99594	55.05323	61.832	55.77949	54.53834
LOC10111	45.00693	48.04788	50.52572	47.99187	42.68185	50.87078	49.34195	42.77096
LOC10111	7.55588	4.544642	8.775537	8.057658	6.465122	7.332736	6.30157	4.566928
LOC10111	0.249903	0.506366	0.493579	0.695111	0.452264	0.275573	0.422753	0.929356
LOC10111	0	0	0.100877	0	0.102163	0	0	0
LOC10111	0	0	0	0	0.069289	0.069753	0	0
LOC10111	1.012088	1.279445	0.813734	1.890748	1.8632	1.118197	2.090903	1.962151
LOC10111	0.06165	0.156148	0.60882	1.547842	1.356478	0.868998	1.032486	0.281375

LOC10111	0.015391	0	0	0	0	0	0	0
LOC10111	0	0.676497	0.219804	0.684274	2.170415	0.72832	1.750853	0.451493
LOC10111	16.26447	15.32497	15.47256	16.49715	15.36835	14.61044	14.82887	16.20277
LOC10111	0.108485	0.329727	0.6428	0.528069	1.24774	1.474554	0.110112	0
LOC10111	0	0	0	0.067807	0	0.03331	0	0
LOC10111	0	0	0	0	0	0	0	0
LOC10111	185.554	218.9612	207.5984	257.8582	294.7338	239.5258	222.7272	236.6127
LOC10111	2.113254	2.273557	2.19676	1.716389	2.303286	1.956421	2.131671	3.211688
LOC10111	0.032736	0.033165	0.032328	0.033547	0.261921	0.098879	0	0.132808
LOC10111	2.889626	3.514955	3.186082	3.440363	3.413754	2.730474	3.417053	3.784382
LOC10111	0.510635	0.161668	0.063034	0.228936	0	0.385592	0.356327	0
LOC10111	0	0	0	0	0.033206	0	0	0
LOC10111	0.709442	0.906997	0.733964	0.969353	0.760214	0.952384	0.685794	0.822333
LOC10111	0	0	0	0	0	0	0	0
LOC10111	0.779664	1.583783	1.33769	1.218642	1.354744	1.458973	1.330923	1.509639
LOC10111	0.029618	0	0	0	0	0	0	0
LOC10111	0	0	0.025059	0.013002	0	0	0	0
LOC10111	0	0	0	0.005725	0	0	0	0
LOC10111	0.43962	0.28343	0.565701	0.327644	0.572913	0.563339	0.32452	0.378321
LOC10111	15.68258	14.22129	12.0922	13.48226	13.12717	13.85295	13.52623	13.76678
LOC10111	0	0	0	0	0	0	0	0
LOC10111	0.1816	0.045996	0.134503	0.093049	0.068109	0	0.046081	0
LOC10111	0.330841	0.720644	0.767789	0.457699	0.463238	0.399722	0.738768	0.603992
LOC10111	0.333204	0.900206	0.914035	1.441711	1.073797	1.453749	1.5407	0.075099
LOC10111	0	0	0.068038	0.017651	0.051679	0.017342	0.034965	0
LOC10111	0	0	0	0	0	0.027493	0	0
LOC10111	3.368852	11.75871	0.205081	5.036575	6.87704	5.970615	5.24618	0.187222
LOC10111	0.078073	0	0	0	0	0	0	0.019796
LOC10111	1.103515	4.065447	31.85087	2.672918	4.113635	10.70652	31.76921	10.1239
LOC10111	0.535583	0.884256	1.253711	0.508194	0.813398	0.778905	0.906028	0.543207
LOC10111	3.68989	4.558922	3.466161	2.674571	3.690367	5.482034	3.836585	3.879336
LOC10111	3.607773	3.814966	1.983961	3.492341	3.408366	3.70655	4.131636	1.802896
LOC10111	111.7839	133.9375	125.4521	133.5457	127.0357	120.7866	124.8434	127.6168
LOC10111	229.2696	165.7631	263.9169	188.5035	255.6722	199.6229	179.9573	200.3992
LOC10111	0.231315	0.745663	0.4361	1.594668	0.988475	0.211723	0.46957	1.130386
LOC10111	0	0.134817	0	0	0.044363	0.223299	0	0.224941
LOC10111	0	0	0.03189	0.016546	0	0	0	0
LOC10111	6.964779	6.836594	5.907657	7.041049	7.500389	6.997811	8.682565	6.529003
LOC10111	0.021697	0.021982	0	0.022234	0.0217	0	0	0
LOC10111	0.223162	0.118995	0.150787	0.180544	0.317165	0.141907	0.071529	0.131038
LOC10111	0	0	0.028882	0	0	0	0	0
LOC10111	7.096871	6.968552	6.850141	6.670341	7.03949	6.807893	6.172869	7.2718
LOC10111	0	0.024679	0	0	0	0	0	0.012353
LOC10111	2.320754	4.258798	2.750204	3.616719	5.63184	3.535773	3.315569	3.517363
LOC10111	14.31472	16.17061	17.7269	17.27041	13.47038	15.90902	18.10378	15.93324
LOC10111	0.401278	0	0	0.102805	0.050166	0.151507	0.254561	0.254369
LOC10111	54.41695	54.03748	81.30318	79.41013	81.62634	68.19961	69.80945	81.25269
LOC10111	0.280593	0.284276	0.450282	0.107829	0.491101	0.388451	0.498404	0.533602
LOC10111	0.753171	0.785498	0.962547	0.669674	1.00805	0.669103	0.640805	0.842529
LOC10111	0	0	0.028792	0	0	0	0	0
LOC10111	12.40633	11.14222	11.92622	10.74825	12.01831	10.71096	8.881615	9.23965
LOC10111	21.80725	23.82118	20.20929	29.94947	44.81404	71.81381	31.28668	47.99733
LOC10111	14.28636	15.02128	15.49571	16.0799	19.54091	16.82118	16.54084	16.17765
LOC10111	0.108256	0.229325	0.204096	0.110938	0.187012	0.148631	0.089902	0.129761
LOC10111	7.83183	12.16207	12.5523	10.65725	28.2496	12.28044	27.10584	39.32595
LOC10111	0	0	0	0	0	0	0	0
LOC10111	0	0	0	0	0	0	0	0
LOC10111	2.849257	3.192656	2.038232	2.930151	3.84649	2.149007	2.40148	3.267638

LOC10111	14.15544	9.824664	10.05312	18.27389	18.47799	8.676223	10.70586	8.087438
LOC10111	1.728203	2.249739	1.639928	1.592921	1.651178	1.380343	1.783525	1.899688
LOC10111	0	0	0	0	0	0	0	0
LOC10111	2.196952	2.314816	1.204115	1.632244	2.691614	2.543754	4.214522	1.18096
LOC10111	0.995847	7.418505	10.95667	3.721877	10.06522	3.857255	7.253868	12.88077
LOC10111	0	0.017	0	0	0.033564	0.016895	0.017032	0
LOC10111	0.233521	0.033798	0.032944	0.205119	0.533831	0.067176	0.237024	0.338351
LOC10111	195.1606	184.4106	234.7745	128.383	157.6865	93.18346	172.2179	179.3787
LOC10111	0.72777	0.372998	0.448132	0.42993	0.659359	0.456886	0.547497	0.42551
LOC10111	0.067223	0.173358	0.120701	0.125251	0.073344	0.123058	0.093043	0.123963
LOC10111	0.813638	0.777213	1.124901	0.619389	1.150866	2.047992	1.038203	0.990265
LOC10111	1.966636	2.359056	2.283947	2.160456	1.96689	2.043433	2.26761	2.728657
LOC10111	0	0.005039	0	0.005097	0.004975	0.010016	0.005049	0.01009
LOC10111	0.045734	0	0.609715	0.023433	0.02287	0	0	0.046385
LOC10111	6.308075	12.68705	11.48992	6.200967	2.920783	2.752173	6.023278	15.61555
LOC10111	0	0.013465	0.013125	0.027239	0	0.026762	0	0.026959
LOC10111	0	0	0	0	0	0.019022	0.009588	0.009581
LOC10111	0	0.028385	0.027668	0.086134	0.140104	0.084626	0.11375	0.14208
LOC10111	8.961854	7.761723	6.685078	7.992315	8.381574	6.706542	7.8111	7.805218
LOC10111	0.228069	0.35943	0.287791	0.402515	0.278787	0.153085	0.360096	0.385527
LOC10111	4.619898	10.93262	10.12372	9.08854	10.2865	10.25358	4.826108	10.46579
LOC10111	0.081128	0.082193	0.040059	0.041569	0.121708	0	0.082345	0
LOC10111	0	0	0	0	0	0	0	0
LOC10111	4.581347	3.553627	15.05731	13.25311	14.60493	9.561627	9.614982	38.60037
LOC10111	0.007504	0	0	0.084586	0.090057	0.037775	0.053314	0.053274
LOC10111	0.124488	0.126122	0.143427	0.10631	0.145255	0.438686	0.105297	0.084174
LOC10111	0.1211	0.061344	0.029898	0.139612	0.136255	0.076204	0.107552	0.291706
LOC10111	0.193195	0.23243	0.095394	0.086617	0.398517	0.145886	0.404442	0.465371
LOC10111	8.033284	8.338076	7.327339	7.152823	7.223944	8.0532	6.884895	7.548897
LOC10111	0	0	0	0.04175	0	0	0.041352	0
LOC10111	0.022249	0.022541	0.043943	0.0456	0.044504	0.022401	0.067748	0
LOC10111	0.015447	0.109545	0.137287	0.015829	0.139037	0.07776	0.09407	0.015666
LOC10111	22.39133	28.15249	27.95858	28.68247	30.28859	18.40847	23.74911	43.8272
LOC10111	0	0	0	0	0	0	0	0
LOC10111	1.117704	1.462648	1.9776	1.407875	1.583618	1.266007	1.441721	1.346168
LOC10111	1.440084	1.606221	1.259051	1.008659	1.433664	1.223788	0.938697	1.313187
LOC10111	0.029999	0	0	0	0	0	0	0
LOC10111	0.071129	0.036031	0.035121	0.072891	0.142276	0	0.072196	0.072141
LOC10111	21.43474	29.00708	26.78759	28.09248	28.1921	27.82366	28.20672	26.68076
LOC10111	15.80645	31.26741	18.41696	12.27473	11.82794	11.69348	14.88164	16.72347
LOC10111	0	0	0.007028	0	0	0	0	0
LOC10111	37.28117	57.0712	69.31752	33.16093	51.26116	61.4728	29.02628	27.91424
LOC10111	0	0.116406	0.045387	0.023549	0.045965	0.023137	0.023324	0.06992
LOC10111	0.041963	0.028342	0.2072	0.229345	0.083936	0.168998	0.085184	0.099307
LOC10111	0.0838	0.072771	0.082756	0.110412	0.083811	0.156692	0.085057	0.048567
LOC10111	0.229985	0.209703	0.136272	0.25925	0.529034	0.092622	0.186748	1.142969
LOC10111	4.917471	4.913761	5.155553	6.59248	9.802527	7.460508	6.461253	6.251423
LOC10111	34.19069	33.79612	32.37361	39.03065	31.69771	32.26929	34.22078	35.37319
LOC10111	0	0	0	0.030256	0	0	0	0
LOC10111	0	0.030007	0	0.030352	0.029622	0	0	0
LOC10111	4.40595	4.646937	3.74931	4.123962	4.419913	4.206831	4.893417	4.129107
LOC10111	9.781927	10.02605	10.50618	8.619374	7.797989	7.418915	8.682139	8.733535
LOC10111	6.486201	6.496009	6.533856	3.999547	6.449864	4.228927	7.922829	7.935713
LOC10111	0	0.01449	0.014124	0.058625	0	0.0144	0.029033	0.058023
LOC10111	0.100021	0.337778	0.197549	0.341661	0.183395	0.050352	0.219962	0.473409
LOC10111	13.23368	13.80036	11.71329	11.35985	11.72832	12.54297	12.9476	11.62137
LOC10111	10.50612	11.3866	11.98377	11.01674	13.73846	10.49589	10.3055	10.98609
LOC10111	1.430812	1.861202	1.456595	1.837343	2.137663	2.214236	1.891543	1.881161

LOC10111	2.473702	2.769052	2.477046	2.322252	2.110704	2.490602	2.282553	2.614186
LOC10111	9.107723	8.349119	6.857202	6.449054	8.058616	4.765727	9.791005	11.58099
LOC10111	0	0	0	0.009065	0	0	0.017958	0
LOC10111	0.197454	0.271491	0.320347	0.375784	0.24685	0.255604	0.372202	0.379074
LOC10111	1.727349	1.626123	1.864332	2.623874	2.430831	1.908447	1.954961	2.604652
LOC10111	11.12987	10.16501	6.361898	12.83828	12.09091	9.384258	12.27069	16.59883
LOC10111	0	0.030393	0.029625	0	0.030003	0.030204	0.030449	0
LOC10111	0	0	0	0	0.025448	0.025619	0	0
LOC10111	2.110167	3.342442	2.506995	2.788116	2.913906	2.04909	1.94612	3.552525
LOC10111	0.430306	0.540062	0.520081	0.625249	0.353282	0.368582	0.456317	0.227987
LOC10111	0.296182	0.060014	0.350989	1.123017	1.303368	0.029821	0.360749	0.03004
LOC10111	0	0	0	0	0	0	0.008273	0
LOC10111	6.707865	0.724436	2.858194	5.792323	3.064903	3.5654	2.626623	4.38592
LOC10111	0.085541	0.138663	0.06758	0.140257	0.153995	0.03445	0.312568	0.086759
LOC10111	3.203718	3.02313	2.581295	3.508269	3.47018	3.155736	3.521777	3.577777
LOC10111	0.084305	0.02847	0.138757	0.086393	0	0	0.028523	0.028502
LOC10111	0.302734	0.479579	0.250041	0.265108	0.291764	0.293719	0.212299	0.379618
LOC10111	39.91951	20.18877	15.02406	12.12592	24.2214	22.55084	13.5281	27.10176
LOC10111	65.19973	73.44736	73.93584	75.56714	77.80338	75.07153	71.21231	72.29668
LOC10111	0.622264	0.373112	0.36369	0.221235	0.736652	0.600943	0.48981	0.579602
LOC10111	1.347683	1.720212	1.112835	1.568329	1.507772	1.126908	1.375626	1.683487
LOC10111	0	0	0	0	0.034368	0	0	0
LOC10111	7.360727	5.38792	6.523938	5.223565	5.061154	5.447094	5.97691	5.599134
LOC10111	62.97048	68.00558	69.76108	67.26997	64.21269	72.94596	74.87369	69.6654
LOC10111	0.033147	0.053731	0.013094	0.040761	0.072932	0.006675	0.006729	0.006724
LOC10111	1.020013	1.043734	0.66482	1.013921	1.469009	1.273457	2.24663	1.365585
LOC10111	0.099252	0.033518	0.065344	0	0.132354	0.133241	0.369384	0.06711
LOC10111	0	0	0	0	0	0	0.008244	0
LOC10111	0	0	0	0	0	0	0	0
LOC10111	0.539159	0.779578	0.186096	0.654434	0.638698	0.811628	0.286906	0.737961
LOC10111	0	0.12041	0	0	0.118865	0	0.030158	0
LOC10111	6.771866	6.630049	3.61583	6.314249	3.939759	3.260046	5.066103	9.173087
LOC10111	0.122759	0.029264	0.007131	0.037	0.166108	0.036352	0.036647	0.087887
LOC10111	0	0	0	0	0	0	0	0
LOC10111	0.032928	0	0.097554	0.134976	0.197596	0.033153	0.066845	0.23378
LOC10111	0.026656	0.027006	0.131621	0.027317	0	0.053677	0.243506	0.054072
LOC10111	0.051808	0	0.025581	0.026546	0	0	0.026292	0.013136
LOC10111	0	0	0.062464	0	0	0	0	0
LOC10111	0	0	0	0	0	0	0	0
LOC10111	1.811831	2.657962	2.118478	1.841857	2.812325	2.043114	2.13325	1.264285
LOC10111	0	0	0	0	0	0	0	0
LOC10111	1209.21	1459.035	1589.296	1337.356	1352.001	1469.525	1305.995	1291.382
LOC10111	3.699411	6.19262	3.773589	3.84565	10.19373	3.502429	3.816711	3.528185
LOC10111	0.127555	0.203074	0.068381	0.037347	0.051029	0.084394	0.059185	0.0998
LOC10111	0.080122	0	0.105498	0.013684	0	0	0.027108	0.013544
LOC10111	24.62847	27.75777	28.35059	25.88791	28.4058	27.52802	25.17857	23.00039
LOC10111	4.732674	5.011744	3.912377	4.91574	4.133594	4.678761	5.477482	4.191896
LOC10111	10.94518	24.56827	0.88729	8.361069	17.39111	12.53679	8.511647	13.81641
LOC10111	1.992606	3.004662	1.20126	2.884867	1.610512	2.18118	2.292945	2.17372
LOC10111	4.353294	4.425428	3.768981	4.070399	4.109508	4.204135	3.731006	4.505374
LOC10111	5.580149	5.344836	3.696384	3.392766	3.841761	4.015876	7.059871	4.264618
LOC10111	0.037477	0.006328	0.030842	0.019203	0	0	0.00634	0
LOC10111	14.8473	111.749	81.18753	36.59842	47.20647	55.95673	59.66909	69.39193
LOC10111	12.39452	12.9685	13.61974	13.90072	13.62618	11.28898	13.98634	12.59518
LOC10111	9.084418	9.990631	9.278071	10.77583	9.559907	9.838021	8.672921	8.566871
LOC10111	3.81622	3.905903	4.428362	4.75917	7.391772	5.427146	4.51904	4.342652
LOC10111	3.718285	4.042724	3.41447	4.739751	6.145034	4.508403	3.877616	5.392381
LOC10111	18.23536	23.17062	16.6489	20.25003	16.03521	18.62961	20.88764	21.13602

LOC10111	0.02046	0.020728	0	0.020967	0	0	0.0623	0.145258
LOC10111	0	0	0	0	0	0	0	0.048278
LOC10111	0	0	0	0	0	0	0	0
LOC10111	19.35723	28.33031	45.14856	55.21971	51.65612	49.85558	21.7986	23.76119
LOC10111	0	0	0	0	0	0	0	0
LOC10111	7.904426	8.146236	6.830194	8.231152	7.726553	8.961805	8.074865	7.697309
LOC10111	0	0	0	0	0	0	0	0
LOC10111	0	0.035804	0	0.036215	0.035344	0	0	0
LOC10111	0.092137	0.010372	0.080879	0.020982	0.040955	0	0.041564	0.020766
LOC10111	0.007512	0.022831	0.007418	0.023094	0.007513	0.219332	0.015249	0.205707
LOC10111	12.55495	15.00927	19.42433	17.01735	14.04662	13.88794	13.47389	12.7167
LOC10111	2.178576	2.412921	2.935427	1.418987	2.160393	2.267814	1.948903	1.629104
LOC10111	0	0	0	0	0	0	0	0
LOC10111	8.670676	11.76112	11.84589	12.43963	12.47192	10.73167	9.159493	9.903175
LOC10111	0.544066	0.734942	0.41789	0.433644	0.181379	0.852106	0.85902	1.04231
LOC10111	24.45256	23.7601	59.93754	19.45722	52.77006	50.06647	9.663049	21.50357
LOC10111	0.148091	0	0.073123	0	0	0.074551	0	0
LOC10111	31.5931	50.8934	74.94411	61.8364	70.66555	54.9983	44.65273	32.23891
LOC10111	226.4779	178.2866	254.2981	323.716	176.1899	186.8065	254.5562	223.1831
LOC10111	0.341863	0.147383	0.265773	0.32797	0.290985	0.292935	0.273163	0.258203
LOC10111	0.470614	0.200754	0.146763	0.736099	0.569765	0.423953	0.553096	0.477314
LOC10111	0.1188	0.012036	0.05866	0.012174	0.035644	0.059806	0.012058	0.072294
LOC10111	28.84202	58.55891	176.1789	168.2811	13.37605	84.11178	70.78642	56.57863
LOC10111	0.481632	0.874632	0.341018	1.005749	0.663465	0.52152	0.581093	0.940109
LOC10111	110.5207	74.15429	72.03332	64.14896	77.74691	70.77766	85.30112	89.72953
LOC10111	0.15253	0.373451	0.238496	0.169333	0.114412	0.345536	0.309635	0.167593
LOC10111	0	0.03594	0	0	0	0	0	0
LOC10111	0	0	0	0	0	0.031381	0.031636	0.031612
LOC10111	0	0.031316	0	0.063352	0	0.015561	0	0.03135
LOC10111	2.39239	1.676605	2.167179	4.995468	1.67309	2.263847	2.756915	3.210938
LOC10111	9.252	8.352929	6.345967	7.531417	6.771996	6.545058	8.444129	6.167518
LOC10111	0	0.026878	0.104798	0.027187	0.0796	0	0	0.053815
LOC10111	0.045144	0.045736	0.267488	0.092524	0.090299	0.136357	0.045821	0.091573
LOC10111	0.042994	0	0.25475	0.044059	0.085999	0.432878	0.043639	0.043606
LOC10111	0	0.026207	0	0.053017	0.012936	0.013022	0.026256	0.013118
LOC10111	0.163905	0.068376	0.044433	0.108683	0.112498	0.132667	0.03262	0.029336
LOC10111	3.545646	3.853486	3.222209	3.203571	3.418681	2.856521	3.463205	3.633942
LOC10111	0.237953	0.331479	0.323108	0.304808	0.118992	0.449209	0.120761	0.301675
LOC10111	0.618166	0.591485	0.169573	2.146786	1.305185	1.417663	0.801726	1.010111
LOC10111	0	0	0	0.081716	0	0	0	0
LOC10111	0.048677	0	0.012018	0	0	0	0	0
LOC10111	0	0	0	0	0	0.069581	0	0
LOC10111	0	0.028906	1.746897	0.014619	0.042802	0.014363	0	0.014469
LOC10111	8.346662	8.811059	8.879107	8.56418	8.456336	8.86213	8.535855	7.92551
LOC10111	36.88477	43.85417	40.95926	41.96055	41.10612	41.55938	44.18182	38.48446
LOC10111	7.710843	7.03992	1.460972	2.89428	17.03779	6.003187	8.797235	9.866702
LOC10111	0	0	0	0	0	0	0	0
LOC10111	0.025561	0	0.025242	0	0.025564	0.025736	0	0.025925
LOC10111	13.1074	10.84389	12.15274	10.82191	10.61894	13.68679	11.27065	11.87171
LOC10111	7.800851	12.80616	86.63041	46.63214	24.27245	40.10703	46.11423	23.33278
LOC10111	28.89164	34.42654	31.24246	32.69005	31.79118	31.56252	35.05007	36.15511
LOC10111	3.613837	2.965624	2.391101	2.092394	4.102235	3.802258	1.925723	3.445365
LOC10111	22.25691	22.27552	19.78979	20.44836	21.77492	19.93787	20.37642	21.12098
LOC10111	0.189628	0.038423	0.037453	0.272056	0	0	0.038495	0
LOC10111	0	0	0	0	0	0	0	0
LOC10111	7.697572	10.10019	9.36594	8.453304	9.220633	8.993736	8.663738	8.858544
LOC10111	11.58385	10.40154	9.606451	11.16043	11.09235	11.80257	9.857939	11.45972
LOC10111	1.242216	1.385963	1.211209	1.305213	1.321008	1.282365	1.181048	1.08447

LOC10111	0.029524	0.119648	0	0	0.17717	0.029726	0	0
LOC10111	0	0.069416	0.101495	0.035107	0.102789	0	0.034772	0
LOC10111	156.3782	136.3246	164.4753	148.0077	142.703	136.6597	143.6029	176.1558
LOC10111	0.066641	0	0.065811	0	0.133299	0	0.06764	0
LOC10111	226.9455	274.5263	270.473	244.518	251.9934	260.962	245.3236	241.0581
LOC10111	0	0	0	0	0	0	0	0
LOC10111	0.054383	0.036731	0.053705	0	0.01813	0	0.0184	0
LOC10111	1.353386	1.444081	1.393395	1.741014	1.03677	1.174182	1.607505	0.97838
LOC10111	0.156851	0.132424	0.11187	0.142877	0.200446	0.140375	0.185738	0.114894
LOC10111	0	0	0	0.03016	0	0	0	0
LOC10111	5.268943	7.106124	6.263835	5.261891	7.85409	8.751459	5.552359	5.446064
LOC10111	0.196501	0.373275	0.327464	0.56635	0.368488	0.247305	0.373967	0.348773
LOC10111	0.095926	0.151177	0.210513	0.04369	0.085279	0.032194	0.086547	0.129723
LOC10111	0.034092	0.207234	0.084167	0.08734	0.068192	0.051487	0.086507	0.32848
LOC10111	1.364225	1.770496	1.063307	1.398017	1.138881	1.038672	1.5449	2.035446
LOC10111	2.510751	0.375774	3.014806	9.619298	3.395659	1.551213	3.156556	9.838723
LOC10111	16.34404	18.25579	18.50092	20.03585	16.46875	20.50791	17.97854	17.30194
LOC10111	23.9366	25.27899	20.60326	22.28091	22.91033	20.91939	22.98982	24.35709
LOC10111	2.440271	2.116436	1.277958	1.6482	2.34814	1.321537	1.951477	3.618764
LOC10111	0.676378	0.881042	0.604336	0.825157	0.934167	0.745856	0.784598	0.882009
LOC10111	0.169119	0.142782	0.055671	0.606578	0.02819	0.085137	0.228874	0.200114
LOC10111	5.370999	5.705638	5.664547	6.586146	5.554227	5.696452	6.126394	5.659011
LOC10111	0.668245	1.223834	1.142166	0.421414	1.773647	1.52676	0.834792	0.469217
LOC10111	0.03177	0.032187	0	0.032557	0.095322	0	0.032246	0
LOC10111	3.444445	3.677213	2.131367	2.875735	2.884548	3.232527	2.591178	3.567598
LOC10111	0.570431	1.309947	1.089092	1.870596	0.494438	0.957214	0.540389	1.581375
LOC10111	0.214271	0.70552	0.185151	0.617565	0.361629	0.43147	0.516527	0.325982
LOC10111	1.998857	2.354756	1.5761	1.556126	2.076601	1.934508	2.359116	2.446395
LOC10111	0	0	0	0	0	0	0	0.022692
LOC10111	0.046417	0.078376	0.030559	0.047566	0.247587	0.031156	0.047113	0.031385
LOC10111	5.292497	6.289986	3.685389	4.519661	3.121619	4.67965	3.994478	4.782882
LOC10111	0.702924	1.165335	0.504848	0.916791	0.319552	0.900743	0.389164	0.518495
LOC10111	3.169484	2.3057	3.047623	4.310307	3.813406	3.599026	4.88601	1.885256
LOC10111	0.027767	0.281314	0.027421	0.028455	1.027513	0.055913	0.056367	0.084487
LOC10111	0.224512	0.151639	0	0	0.074847	0	0.22788	0.075903
LOC10111	4.612739	2.534659	2.509256	6.850142	3.049493	3.778374	3.650319	3.726865
LOC10111	0	0	0.003919	0.004067	0.003969	0	0.004028	0.012075
LOC10111	27.24609	32.49704	58.84508	53.04987	70.19429	61.63343	21.17207	15.64728
LOC10111	52.42665	54.33412	52.08518	51.03598	55.15673	56.38061	54.25448	54.91406
LOC10111	10.09259	10.7435	9.166679	12.07606	10.98955	8.902078	10.43154	11.96634
LOC10111	0	0.048639	0	0.049198	0	0	0	0.048692
LOC10111	0	0	0.02469	0	0	0.012586	0	0
LOC10111	0.04464	0	0	0	0.044646	0	0.045309	0
LOC10111	1.353924	1.437012	0.668527	1.965571	2.135931	1.744538	1.488753	2.305012
LOC10111	0.265865	0.408675	0.076955	0.286543	0.206301	0.110765	0.404779	0.092983
LOC10111	3.599725	3.56391	2.609114	2.921327	2.921893	2.945226	3.203625	2.913972
LOC10111	0.015813	0.032041	0.015616	0.03241	0.03163	0.031842	0.032101	0
LOC10111	0.03069	0.031093	0.060615	0	0	0	0.0623	0
LOC10111	0.021374	0.048722	0.042215	0.049283	0.026721	0.1076	0.05966	0.130069
LOC10111	3.959916	4.345299	3.54296	4.726968	4.316543	4.486698	4.616189	4.656487
LOC10111	0	0	0	0.119885	0	0	0	0
LOC10111	0.055898	0.088993	0.023658	0.065466	0.031946	0.104521	0.048632	0.032397
LOC10111	420.4131	494.4488	479.9043	475.3966	430.7513	427.6422	455.0477	396.3595
LOC10111	5.046603	5.873501	5.91289	6.827303	6.00728	5.837021	6.058015	5.985978
LOC10111	0.627725	0.773132	0.182325	0.378397	0.418538	0.45852	0.512212	0.499342
LOC10111	0.0838	0.322619	0.347574	0.549604	0.402291	0.556858	0.357239	0.424964
LOC10111	0	0	0	0	0	0	0	0.032592
LOC10111	6.699738	7.546173	7.867471	7.652592	7.986967	5.933727	6.468991	6.580941

LOC10111	0.018772	0.038037	0	0.038474	0	0.0189	0	0.019039
LOC10111	0.662716	0.342849	0.431664	0.520186	0.930742	0.4259	0.429355	0.414731
LOC10111	0	0	0	0	0	0.048671	0	0
LOC10111	0.224587	0.056884	0.138618	0.431532	0.140385	0.282652	0.170967	0.056946
LOC10111	0	0	0	0	0	0	0.051936	0
LOC10111	0	0.014587	0	0	0.0144	0	0	0
LOC10111	5.937095	2.205505	2.484845	6.663603	2.09239	2.447992	2.496549	4.817291
LOC10111	18.56447	18.3862	21.16862	17.5419	16.76936	21.38354	18.13104	20.22923
LOC10111	1.927471	1.97759	1.33887	1.958476	1.723512	1.850185	2.006122	2.617591
LOC10111	3.701193	1.794147	1.171723	2.604197	1.540887	1.087632	2.786077	3.170144
LOC10111	0	0.366029	0.062962	0.217786	0.17004	0.042795	0.150997	0.150883
LOC10111	3.771306	4.185072	3.475764	3.045926	4.938493	3.612043	3.369666	4.732619
LOC10111	0.130486	0.132198	0.06443	0.200577	0.130502	0.065689	0.066221	0
LOC10111	0.044687	0.181095	0.080906	0.053427	0.044693	0.104983	0.037798	0.052877
LOC10111	31.87321	41.62198	38.4356	34.67337	28.67358	34.10672	32.51388	31.14921
LOC10111	0.022867	0.115835	0	0	0	0.046046	0	0.046385
LOC10111	6.479807	7.854371	6.487943	11.15942	7.830778	8.517541	6.629202	13.80913
LOC10111	478.6843	446.9974	386.4127	457.4932	379.6256	421.4581	467.1722	480.7129
LOC10111	1.492118	1.366448	1.103834	1.39304	1.951688	1.33123	1.150697	1.634536
LOC10111	22.22986	20.54482	23.64053	26.40717	25.5165	23.73655	19.84533	20.71749
LOC10111	27.01442	37.94974	31.76585	29.03921	39.20382	30.21329	36.67729	43.07572
LOC10111	0.157685	0	0	0	0	0.039691	0.080025	0
LOC10111	1.631038	1.966199	1.168958	2.249743	2.484729	1.475884	1.613592	2.212656
LOC10111	0.046463	0.018829	0	0	0.037175	0	0	0
LOC10111	0	0	0	0	0	0	0	0.01571
LOC10111	0.14707	0.121062	0.13616	0.39562	0.174668	0.157329	0.289221	0.773784
LOC10111	0	0	0	0.022478	0	0.022085	0	0.022247
LOC10111	15.6098	12.13572	13.87842	14.13584	15.91839	24.38184	15.67641	14.30142
LOC10111	4.170339	4.580118	4.822076	4.417291	4.25266	5.151504	5.430435	5.224931
LOC10111	14.49913	15.55034	18.9864	15.78352	17.36934	16.41627	12.45252	12.22768
LOC10111	0.015541	0	0.245556	0	0.015543	0.156471	0.173514	0.22067
LOC10111	1.888608	3.520644	3.916658	2.903084	2.379953	2.890295	3.143776	2.79662
LOC10111	0	0	0	0	0	0	0	0
LOC10111	0.501925	0.157177	0.108146	0.196391	0.146033	0.229706	0.185256	0.518326
LOC10111	6.292528	6.31136	6.201694	6.732126	7.098889	8.248841	6.156987	6.292757
LOC10111	0.078432	0.022703	0.17704	0.218161	0.224122	0.067687	0.079609	0.318196
LOC10111	1.442364	1.239325	1.226059	0.991631	1.35125	0.790448	0.481823	0.666637
LOC10111	37.89862	50.89417	45.09542	51.68647	34.78872	39.78645	68.39508	61.32872
LOC10111	0.038271	0.025849	0.037795	0.052293	0.127588	0.038533	0.129485	0.10351
LOC10111	213.1705	223.0451	253.7114	238.8889	238.6373	262.3054	233.5308	227.5895
LOC10111	1.107675	1.491424	1.543734	1.267788	1.932687	0.743495	1.601261	3.404373
LOC10111	0.240595	0.297919	0.184797	0.219159	0.106945	0.188407	0.054267	0.135566
LOC10111	200.7695	858.5492	530.9662	341.444	792.6917	758.6891	1431.038	877.7032
LOC10111	0.322828	0.065413	0.191283	0.033082	0.355156	0.227523	0.032767	0.360166
LOC10111	4.680173	6.411557	5.694598	5.338849	5.291549	4.875384	6.066877	5.738912
LOC10111	0.223466	0.424498	0.137926	0.143126	0.698423	0.421862	0.085057	0.226648
LOC10111	83.65143	95.70761	93.7069	88.1347	89.66822	95.82034	94.92242	87.83418
LOC10111	0.03648	0.073917	0	0.065421	0.072969	0.009182	0.009257	0.083248
LOC10111	0.044127	0.076639	0.118281	0.0323	0.138703	0.101551	0.076781	0.102298
LOC10111	1.811385	1.569833	1.438595	1.727657	2.23724	1.450218	1.617043	1.909108
LOC10111	0.20192	0.944167	1.073712	1.559868	0.155343	0.250214	0.331071	0.09452
LOC10111	0.291554	0.042197	0.959737	0.455277	0.985857	0.349459	0.549579	1.492602
LOC10111	0.235834	0.358393	0.296412	0.384483	0.235864	0.269824	0.315535	0.250063
LOC10111	0.036876	0	0.072834	0	0.110643	0	0	0.037401
LOC10111	0.125871	0.139116	0.033901	0.082084	0.137332	0.218899	0.127759	0.139269
LOC10111	0	0	0	0	0	0	0	0
LOC10111	0.039477	0	0	0	0	0	0	0
LOC10111	0.252838	0.333003	0.174781	0.285011	0.252871	0.407304	0.102652	0.076931

LOC10111[illegible]	1.04156	2.24517	1.138006	2.15743	1.108186	1.048676	1.37209	1.146293
LOC10111[illegible]	3.155861	4.15373	3.871256	3.455164	3.704795	3.630045	3.568236	3.757049
LOC10111[illegible]	0	0	0.024695	0	0	0.012589	0	0.006341
LOC10111[illegible]	5.528438	5.694734	7.104272	6.471333	8.397835	7.615688	9.579234	7.085172
LOC10111[illegible]	1.093465	1.031876	0.901318	1.129587	1.411104	0.972196	1.400759	1.144806
LOC10111[illegible]	0.005266	0	0	0	0.005267	0	0.016035	0.026705
LOC10111[illegible]	0.809555	1.182023	0.047028	1.11021	2.071775	1.294547	1.437972	0.784855
LOC10111[illegible]	12.86169	15.50155	11.88538	6.515017	16.92897	16.06281	14.16392	15.50504
LOC10111[illegible]	0	0	0	0	0	0	0	0
LOC10111[illegible]	0	0.060077	0.11712	0	0.17792	0.059704	0.120377	0
LOC10111[illegible]	19.55436	22.68091	22.71357	22.70334	21.56544	27.15873	20.81324	21.50511
LOC10111[illegible]	1682.436	1321.298	1252.631	1278.233	1469.838	1347.267	1403.181	1572.772
LOC10111[illegible]	0.652948	0.782526	0.511131	0.873122	0.868055	0.529134	0.557673	0.428035
LOC10111[illegible]	17.3607	23.0665	24.02832	21.10443	20.43791	19.64087	22.49046	20.56489
LOC10111[illegible]	0	0	0.060748	0	0	0	0.062437	0
LOC10111[illegible]	0	0	0	0	0	0	0	0
LOC10111[illegible]	0.058335	0.03546	0.115216	0.023912	0.046674	0.046987	0.047368	0.094665
LOC10111[illegible]	0.927845	0.814351	0.877085	1.20506	0.962702	0.969153	1.002197	1.071895
LOC10111[illegible]	1.815779	33.04667	9.96735	2.799516	13.97185	24.6393	0.365282	12.67571
LOC10111[illegible]	0.037481	0.066453	0.037014	0.028807	0.018743	0	0.028533	0.019007
LOC10111[illegible]	0	0	0	0.061092	0.178868	0.060022	0.030255	2.781321
LOC10111[illegible]	0	0.006624	0	0	0	0	0	0
LOC10111[illegible]	2.535931	1.938804	1.310137	1.937029	2.336646	1.465758	2.216474	2.226713
LOC10111[illegible]	9.633172	15.51139	8.40164	10.3128	15.59385	14.83178	17.52252	12.23806
LOC10111[illegible]	0.189305	0.2131	0	0.24429	0.266464	0.275309	0.113864	0.341334
LOC10111[illegible]	0.945935	0.862514	1.058702	1.260176	1.429598	1.153459	1.184153	0.948741
LOC10111[illegible]	22.37357	25.6805	25.84302	22.58659	23.13123	25.67744	24.71425	26.42907
LOC10111[illegible]	3.124134	4.59204	5.917527	4.303682	2.228154	5.46591	3.690831	5.194439
LOC10111[illegible]	0	0	0	0	0	0	0	0
LOC10111[illegible]	0.410564	0.458982	0.41943	0.420734	0.523891	0.741214	0.876555	0.646152
LOC10111[illegible]	0.6335	0.583467	0.739353	0.885261	0.633581	0.40589	0.643002	0.934571
LOC10111[illegible]	11.47168	13.36993	24.89341	3.734466	24.9951	12.15794	14.8611	13.9751
LOC10111[illegible]	3.862711	3.306514	3.977772	4.826275	3.098832	3.909897	6.541409	7.479244
LOC10111[illegible]	0.074193	0.131541	0.091585	0.247099	0.241157	0.056025	0.188264	0.131685
LOC10111[illegible]	20.18672	22.11243	19.3746	25.204	19.63318	21.631	18.20246	21.12621
LOC10111[illegible]	0	0.054848	0	0	0	0	0	0
LOC10111[illegible]	0	0	0	0	0	0	0.0356	0
LOC10111[illegible]	40.34495	42.0689	43.19543	42.60083	44.147	49.19101	44.68375	40.53664
LOC10111[illegible]	0.492066	0.587546	0.46858	0.702354	0.515564	0.530815	0.772953	0.433716
LOC10111[illegible]	1.318601	1.424126	1.351309	1.300272	0.70915	1.102166	1.527773	1.514006
LOC10111[illegible]	4.863535	4.653621	4.5518	4.772267	4.848267	5.424842	4.871958	4.93277
LOC10111[illegible]	0	0	0.049095	0	0.049721	0.016685	0	0.016807
LOC10111[illegible]	0.035266	0.083366	0.034826	0.024093	0.058784	0.023671	0.011932	0.071535
LOC10111[illegible]	0.715142	0.299804	0.730584	0.530689	0.345285	0.372428	0.325389	0.350156
LOC10111[illegible]	2.671693	2.620827	2.198669	3.346288	2.905312	2.391062	3.27134	3.204359
LOC10111[illegible]	17.56916	17.94175	21.89545	23.27158	26.05338	23.92274	15.55618	21.32625
LOC10111[illegible]	0.15482	0.011204	0.185653	0.079327	0.15484	0.033402	0.112244	0.437423
LOC10111[illegible]	39.83497	44.43262	57.65312	46.60336	53.48385	53.84226	39.25811	35.85333
LOC10111[illegible]	17.9183	18.43319	16.83396	17.17076	16.93229	15.31924	18.48208	16.38994
LOC10111[illegible]	0.495641	0	0	0	0	0	0.503076	0.266134
LOC10111[illegible]	1.050788	1.451698	1.320702	0.587354	0.764308	1.827396	1.163509	0.871974
LOC10111[illegible]	9.206266	10.828	8.882553	9.840959	10.05412	10.2014	9.129547	8.612875
LOC10111[illegible]	0.727163	1.285887	0.535313	0.975502	1.097497	0.519147	1.207752	1.930947
LOC10111[illegible]	25.31786	22.95473	22.87704	22.36523	24.81277	24.58705	24.53405	25.04208
LOC10111[illegible]	0.129579	0.19692	0.127965	0.730341	0.226793	0.195697	0.164404	1.248529
LOC10111[illegible]	0.076989	0.070909	0.027647	0.093241	0.132999	0.098656	0.085248	0.099381
LOC10111[illegible]	12.24788	11.58907	10.00049	14.20307	12.20152	13.04949	9.737275	10.11282
LOC10111[illegible]	0.045994	0.014338	0.010482	0.018128	0.014154	0.03206	0.03232	0.02153

LOC10111[illegible]	3.351252	3.646733	2.635339	0	8.627487	0.124968	3.90545	4.657834
LOC10111[illegible]	4.551786	4.236603	5.091976	3.337228	4.391992	4.309647	4.432255	5.730068
LOC10111[illegible]	0	0	0	0	0	0	0	0
LOC10111[illegible]	7.276033	9.234784	6.538349	8.940448	9.656648	10.9944	7.910186	8.265632
LOC10111[illegible]	61.52573	58.62897	63.21811	75.03307	50.87877	69.62583	84.17127	59.97202
LOC10111[illegible]	36.10673	43.42608	44.6	40.2949	38.90363	47.80979	37.12459	38.64332
LOC10111[illegible]	0	0	0	0	0	0	0	0
LOC10111[illegible]	0	0.177228	0	0.059755	0.058318	0	0.295927	0
LOC10111[illegible]	7.154584	1.784802	8.769663	7.239698	1.923717	2.081402	3.557968	9.90011
LOC10111[illegible]	0.690238	0.524473	0.54531	0.707336	0.655811	0.312729	0.385326	0.385035
LOC10111[illegible]	0	0	0	0	0.036616	0.018431	0	0.018566
LOC10111[illegible]	1.315369	1.151774	1.401042	1.309438	2.189432	2.204104	1.30649	1.810556
LOC10111[illegible]	0	0	0	0	0	0	0	0
LOC10111[illegible]	0.91269	0.385278	0	1.948537	0.126779	0.127629	1.055044	1.825652
LOC10111[illegible]	0.2653	0.638357	0.163747	0.679679	0.82917	0.233723	0.30294	0.302711
LOC10111[illegible]	0.022765	0.023063	0.011241	0.011664	0	0	0.023106	0
LOC10111[illegible]	31.47461	44.91562	42.29505	33.71543	48.88174	47.53444	37.30692	42.26004
LOC10111[illegible]	1.243126	0.992286	1.748452	1.29322	1.05491	0.929232	1.300008	1.184409
LOC10111[illegible]	0	0	0	0	0	0.021125	0	0
LOC10111[illegible]	0.857645	0.979237	0.806627	1.102099	1.198135	0.740146	0.469799	1.366913
LOC10111[illegible]	0	0	0	0	0	0	0	0
LOC10111[illegible]	2.846085	3.88373	3.686968	3.567899	3.901879	2.797109	3.440565	4.382606
LOC10111[illegible]	0	0	0.134503	0	0.136218	0	0	0
LOC10111[illegible]	0.998793	0.965377	1.054372	1.152946	1.010404	0.762881	0.897249	1.001368
LOC10111[illegible]	11.74428	7.07647	9.189552	9.926935	9.974388	10.22413	10.35778	10.31774
LOC10111[illegible]	109.5497	97.1924	94.87386	88.58442	95.53311	101.8509	100.304	106.6453
LOC10111[illegible]	0.027668	0.005606	0.005465	0.005671	0.005534	0.011143	0.005617	0.028062
LOC10111[illegible]	0.037469	0.01898	0.037002	0.153588	0	0	0.019015	0
LOC10111[illegible]	4.851254	5.37426	4.074424	5.03337	6.862162	6.056041	7.899911	5.778687
LOC10111[illegible]	1.441464	0.761549	1.702964	1.141861	2.069609	2.341687	1.13995	0.941769
LOC10111[illegible]	0	0	0	0.012503	0	0	0	0
LOC10111[illegible]	0	0	0	0	0	0	0	0
LOC10111[illegible]	10.8593	8.51957	7.279189	8.894118	11.00606	8.299413	9.483717	11.41401
LOC10111[illegible]	0.048932	0.049574	0	0.150433	0.048938	0	0.049666	0
LOC10111[illegible]	1.644288	1.022794	0.931297	1.598289	0.79202	1.667694	0.779253	0.950341
LOC10111[illegible]	2.704932	14.18986	3.305078	5.731795	17.88237	16.2943	2.303433	3.99891
LOC10111[illegible]	0.106423	0.215639	0.262742	0.054529	0.053218	0	0.05401	0.053969
LOC10111[illegible]	0	0	0	0	0	0	0	0
LOC10111[illegible]	0	0	0.033832	0	0	0	0.034772	0.034746
LOC10111[illegible]	0.06464	0	0	0.066241	0	0	0.131219	0
LOC10111[illegible]	0.023422	0.023729	0.02313	0	0	0	0	0.023755
LOC10111[illegible]	1.428329	1.231552	1.020384	1.297614	1.266413	1.274899	1.42919	1.66442
LOC10111[illegible]	0.012516	0.033813	0.01236	0.00855	0.012517	0.029403	0.004234	0.025388
LOC10112[illegible]	0	0.004745	0.004625	0	0	0	0.009508	0.00475
LOC10112[illegible]	0.154807	0.062736	2.90469	0.444197	1.579239	1.901552	1.979831	2.355167
LOC10112[illegible]	3.686565	3.356602	2.732836	2.639611	3.0454	2.882631	2.818546	2.758153
LOC10112[illegible]	2.232879	2.050992	1.876476	1.828086	2.537873	2.056417	1.959171	2.291094
LOC10112[illegible]	6.568185	5.866061	6.870553	7.434458	10.55165	8.5025	6.341503	5.988555
LOC10112[illegible]	0	0.062283	0	0	0.008783	0.017685	0.04457	0.062351
LOC10112[illegible]	29.72842	28.38912	24.0144	26.19599	27.49886	25.07805	24.86741	24.40224
LOC10112[illegible]	4.741466	6.543806	7.525264	6.024062	8.201861	6.746727	8.471137	8.513829
LOC10112[illegible]	0.308057	0.178343	0.06519	0.315688	0.418131	0.299084	0.335012	0.468663
LOC10112[illegible]	0.923788	0.607522	0.848258	0.714155	0.583521	0.685336	0.312548	0.723251
LOC10112[illegible]	0.046766	0.047379	0.011546	0.215658	0.093543	0.047085	0.023734	0.260872
LOC10112[illegible]	13.30283	19.22779	12.85682	15.37729	21.81947	7.821929	13.50238	21.44362
LOC10112[illegible]	0	0	0	0	0.010069	0.010137	0.020438	0.245073
LOC10112[illegible]	0	0	0	0	0	0	0.009438	0
LOC10112[illegible]	0.282719	1.37486	0.446714	1.796276	0.452409	0.28465	0.172176	2.121902

LOC10112	0	0	0	0	0	0	0.486218	0
LOC10112	0	0	0.09854	1.993968	16.11706	4.571148	9.520307	21.10095
LOC10112	0.043909	0.082616	0.08053	0.115707	0.050189	0.050525	0.070036	0.076345
LOC10112	114.6574	137.0947	156.2747	125.2584	113.1233	121.9248	121.2271	176.5702
LOC10112	0.161709	0.170658	0.073193	0.124286	0.134775	0.067839	0.150457	0.225515
LOC10112	13.74568	19.14836	16.9363	17.29505	13.52262	18.62524	15.45137	11.85667
LOC10112	32.06063	40.66214	22.96358	35.54321	34.91005	26.24222	36.10347	31.92958
LOC10112	0	0	0	0	0	0	0	0
LOC10112	0.112406	1.252697	0.055503	0.172786	7.194932	1.527852	5.476434	11.28664
LOC10112	2.928957	3.32447	3.072491	4.035223	4.54594	3.193694	3.466318	3.808845
LOC10112	40.58626	39.45714	20.1245	23.92895	24.13442	25.941	34.84864	38.72852
LOC10112	0.004012	0	0.003962	0.004111	0	0	0	0
LOC10112	18.35816	14.3156	10.9193	17.02217	16.13075	15.33894	20.03003	19.16953
LOC10112	0.091244	72.9134	11.17331	5.095988	38.39597	77.92666	0.740903	0.046272
LOC10112	1.158044	1.192165	1.079055	1.358996	0.906006	2.002809	0.99531	1.174529
LOC10112	4.204594	5.217595	5.872053	5.379556	6.121088	5.385583	4.74746	3.658847
LOC10112	0	0	0	0	0	0	0	0
LOC10112	4.129144	2.454891	3.906772	2.837841	6.058509	4.03288	4.090696	3.084519
LOC10112	0.018426	0.018668	0	0	0.018428	0.018552	0.130917	0.018688
LOC10112	1.173635	0.820026	0.783332	0.713329	1.060455	0.6112	1.068008	1.165714
LOC10112	0.045585	0	0.015006	0.015571	0.075985	0.015299	0.046269	0
LOC10112	0	0	0.013988	0	0	0	0	0
LOC10112	8.544719	7.748945	4.30145	8.051593	5.190024	7.32312	7.995981	8.920008
LOC10112	0.222844	0.49669	0.572174	0.091345	0.40117	0.403859	0.226186	0.406829
LOC10112	3.1978	2.89874	6.33253	3.94966	2.30608	7.066275	4.219497	4.028548
LOC10112	0.062588	0.164863	0.135977	0	0.075115	0.12603	0.114348	0.101566
LOC10112	3.979283	3.810603	3.370681	3.205561	3.564624	3.178999	3.59209	4.35064
LOC10112	0	0	0	0	0	0	0	0
LOC10112	2.59823	2.823539	4.26193	3.094534	3.705945	2.901014	3.448159	4.759981
LOC10112	0.034683	0	0	0	0.069375	0.03492	0	0
LOC10112	0.285022	0.399455	0.220485	0.238534	0.304063	0.157833	0.274833	0.284262
LOC10112	23.1128	26.43987	25.51691	23.28795	23.45659	22.05215	24.28242	23.01279
LOC10112	0.004463	0.018087	0	0	0.008928	0.004494	0.00453	0.004527
LOC10112	0	0	0	0	0.009325	0.018774	0.02839	0.028369
LOC10112	16.63882	15.01402	14.39484	17.8737	16.57467	14.86885	18.4808	16.55446
LOC10112	0	0.022856	0.007426	0.100181	0.323399	0.181711	0.25188	0.976253
LOC10112	0	0.015051	0	0	0	0	0	0.030135
LOC10112	58.37331	29.30262	28.09491	39.22776	60.64656	34.46967	19.73301	34.81379
LOC10112	1.139396	0.992336	0.552729	0.889029	1.099559	1.0546	0.900843	1.188054
LOC10112	0.009862	0	0.009739	0.040426	0	0.00993	0.02002	0.020005
LOC10112	0.624957	0.045226	0.088167	0.091491	0.089291	0.314613	0.271856	0.135826
LOC10112	0	0	0	0	0	0.018134	0	0
LOC10112	3.74437	3.698673	4.280104	4.777213	4.372117	4.250618	3.886047	3.152069
LOC10112	0	0	0	0	0	0	0	0
LOC10112	0.176588	0.447263	0.784744	0.814329	0.794748	1.955736	0.716946	1.07461
LOC10112	0.779387	1.021856	0	0.046982	0.962897	0.369276	0.442073	1.185723
LOC10112	0.708977	0.450599	0.67405	0.825817	0.497669	0.926636	0.424615	0.294774
LOC10112	0.024066	0.008127	0.015844	0.041104	0.008023	0.032308	0.016285	0.065091
LOC10112	0.180964	0.366679	0.536129	0.185447	0.120659	0.060734	0.244905	0.367081
LOC10112	0.1023	0.051821	0.111127	0.146767	0.08185	0.072099	0.0623	0.145258
LOC10112	16.39707	17.06918	14.22434	15.85039	18.97183	18.14599	18.491	20.03001
LOC10112	0	0	0	0	0	0	0	0
LOC10112	0.020007	0.243229	0.039515	0.020502	0.200091	0.241719	0	0
LOC10112	0	0	0	0	0	0	0.082226	0.041082
LOC10112	1.916798	2.576062	2.588271	4.028774	1.349757	2.304056	2.084518	2.360675
LOC10112	0	0	0	0	0	0	0	0
LOC10112	0	0	0.033382	0.103922	0	0	0.080058	0.182851
LOC10112	0.058677	0.029724	0.260759	0.060131	0.088028	0.088618	0.029779	0.089269

LOC10112	0	0	0	0	0	0	0	0
LOC10112	0.053364	0.054064	0.026349	0	0	0.053728	0.352066	0.027062
LOC10112	0.955163	1.920735	0.914678	0.963992	1.563196	1.078256	1.630506	1.526531
LOC10112	0.984734	1.438929	0.448829	0.931501	0.284095	0.877062	0.288319	0.633823
LOC10112	0.428953	1.086456	0.423608	0.494525	1.287024	0.755795	0.870774	0.761353
LOC10112	0	0	0	0	0	0	0.030547	0
LOC10112	0	0	0	0.022478	0	0	0	0.022247
LOC10112	1.121188	1.074317	1.347337	1.211253	1.215903	1.482462	1.419079	1.089196
LOC10112	0	0	0	0	0	0.029915	0	0
LOC10112	0	0	0.065499	0	0.066334	0	0.06732	0
LOC10112	0.13301	0.173258	0.206412	0.077889	0.038008	0.038263	0.520735	0.173448
LOC10112	0.038046	0.115636	0.06262	0.012996	0.012684	0.025537	0.038617	0.15435
LOC10112	1.033565	0.996733	0.311118	0.628703	0.657808	0.834727	0.729299	0.846471
LOC10112	40.86788	44.09421	43.37272	36.41127	50.16293	52.98585	36.0104	25.47101
LOC10112	0.260769	0.176127	0	0.089076	0	0.306308	0.13234	0.04408
LOC10112	0	0	0	0	0	0	0	0
LOC10112	16.6937	18.75113	17.38165	33.61009	86.88198	64.6276	97.56674	110.8359
LOC10112	4.918284	5.23958	3.707634	4.135969	5.613576	4.337623	4.487136	5.835548
LOC10112	0.007362	0.111875	0.08724	0.03772	0.080989	0.081532	0.007472	0.007466
LOC10112	0	0	0	0	0	0	0	0
LOC10112	0	0	0	0	0	0	0	0
LOC10112	1052.97	1258.861	1210.681	1105.094	172.3386	724.957	1101.568	692.1767
LOC10112	0	0	0.05618	0.023319	0.011379	0.011455	0.011548	0
LOC10112	776.9558	941.9255	60.70761	87.5491	841.6887	83.72964	78.23631	74.53605
LOC10112	0	0	0	0	0	0	0	0
LOC10112	0	0	0.095807	0	0.097029	0.048839	0.098471	0.049199
LOC10112	0.441644	0.05593	0.054518	0.113146	0.276063	0.22233	0.168101	0.111983
LOC10112	0.260607	0.316832	0.205888	0.413946	0.456121	0.380461	0.238064	0.118942
LOC10112	102.4122	110.8158	109.7521	109.0894	104.2409	114.9618	115.835	105.9487
LOC10112	4.053038	4.140795	3.126561	3.782841	4.469837	3.709752	4.245421	4.152259
LOC10112	0	0	0.334167	0.198152	0.096694	0	0	0
LOC10112	13.40156	13.20242	10.21688	12.07296	11.45022	11.41666	11.94252	13.2284
LOC10112	243.202	234.835	275.9178	255.9973	244.588	284.2003	251.8487	257.2367
LOC10112	27.41912	38.72307	46.56931	23.71707	25.52702	27.04685	29.2335	25.90152
LOC10112	0	0	0	0	0	0.121206	0	0
LOC10112	0	0	0	0.017447	0.017027	0.017141	0.01728	0
LOC10112	1.396514	2.565466	1.345881	1.396621	3.062631	1.617809	1.921258	1.220147
LOC10112	3.184043	3.260624	2.311039	2.765181	3.429791	2.4204	3.376214	3.975758
LOC10112	5.642812	5.882574	4.946605	5.887343	6.195627	5.249083	5.353924	5.723141
LOC10112	0.042653	0.021607	0.042122	0.008742	0.063988	0.060123	0.086586	0.043261
LOC10112	8.913818	9.759645	8.725047	9.284364	9.016149	8.872857	9.446849	9.439735
LOC10112	12.81277	11.15839	10.44147	11.48833	11.18214	10.63683	11.17906	11.85185
LOC10112	0	0.030199	0	0	0	0	0	0
LOC10112	11.5063	24.8882	26.70133	24.65458	20.51113	19.97838	24.323	22.21499
LOC10112	30.62158	33.55041	33.09961	32.80983	34.28507	34.08414	31.49505	30.58359
LOC10112	1.097956	0.821032	0.894957	1.2591	1.202679	1.798561	0.689882	0.830771
LOC10112	0.435065	0.23508	0.143215	0.683624	0.203056	0.408834	0.264954	0.058834
LOC10112	0.068901	0.085318	0.052922	0.054917	0.061253	0.07708	0.031082	0.031059
LOC10112	0.717243	1.537807	1.334251	1.281993	1.601494	1.343522	1.21898	1.116557
LOC10112	0.507003	0.96146	0.821638	0.812648	0.767104	0.287956	0.725729	0.369184
LOC10112	2.098289	2.053025	2.306325	1.848344	2.077	2.365849	2.268344	1.953242
LOC10112	0	0	0	0.02812	0	0	0	0
LOC10112	6.015262	5.974925	7.398964	7.831465	6.669026	7.834467	6.779051	6.187739
LOC10112	0	0	0	0	0	0	0	0
LOC10112	77.42587	83.29878	79.69119	85.97064	81.13162	86.25055	84.59344	79.3131
LOC10112	5.551001	5.414527	4.706488	4.890981	5.035119	5.838552	5.228822	5.294736
LOC10112	0.567348	0.779605	0.605359	0.79525	0.619598	0.643447	0.529525	1.157463
LOC10112	12.67966	17.46113	7.512999	18.7206	10.52078	22.22278	32.69895	16.67058

LOC10112	1.271006	1.342481	1.228463	1.330201	1.460493	0.054455	1.839037	1.645658
LOC10112	0.374415	0.259541	0.240013	0.48466	0.42045	0.462949	0.4067	0.473016
LOC10112	0	0	0	0	0	0.014731	0	0
LOC10112	13.40409	11.40242	14.0418	15.33362	12.51998	21.56928	13.78496	15.99049
LOC10112	0.018366	0.018607	0.036274	0	0	0	0	0
LOC10112	0	0	0	0	0	0	0	0
LOC10112	21.31841	16.9115	31.83254	19.08977	26.79531	31.94558	26.79127	25.54885
LOC10112	1.880992	1.676997	1.827834	3.17666	1.986585	2.954394	1.832839	4.777055
LOC10112	0	0	0	0	0	0	0	0
LOC10112	0.261198	0.16604	0.505772	0.257171	0.302209	0.319704	0.478249	0.254528
LOC10112	3.325737	2.832668	3.458685	3.528758	0.971358	3.526251	2.091094	2.626824
LOC10112	0.021547	0	0	0	0	0	0	0
LOC10112	0	0	0	0	0	0	0	0
LOC10112	0.529324	0.178757	0.13756	0.428239	0.204327	0.177647	0.358176	0.160116
LOC10112	2.236049	1.671705	1.690406	1.485483	1.742801	0.962635	1.549582	1.673539
LOC10112	0.480967	0.314374	0.44433	0.683673	0.356892	0.171832	0.740146	0.865476
LOC10112	0.007428	0.015051	0	0.060897	0.014858	0.014958	0.00754	0.022602
LOC10112	7.362508	7.020361	6.613272	6.763233	7.195373	5.792271	6.226361	6.975616
LOC10112	0.024487	0	0	0.025094	0.048981	0	0	0
LOC10112	6.670525	31.37023	3.249195	23.7404	5.950157	13.97674	17.61267	12.61671
LOC10112	0	0.034497	0.168129	2.71297	1.515424	0.308544	0.656656	1.010143
LOC10112	0.871743	1.640198	2.428913	0.914606	1.276645	1.274752	1.24296	4.820736
LOC10112	0	0.029818	0	0	0	0	0	0
LOC10112	0.163488	0.662535	0.387482	0.033508	0.457826	0.164605	0.132752	0.265305
LOC10112	0	0.032407	0.031589	0.1639	0.159959	0.128825	0	0
LOC10112	0	0	0.029249	0	0	0	0	0
LOC10112	8.7201	8.318796	9.095549	6.656187	8.664657	7.16611	8.114124	9.207567
LOC10112	0.056889	0.057635	0	0.116595	0.113792	0	0.115484	0.115397
LOC10112	7.809941	6.388498	6.579652	5.251412	6.723027	5.980896	6.361512	7.32639
LOC10112	4.410655	4.42818	4.114027	4.724034	4.024176	4.131364	4.690547	5.154562
LOC10112	0.023555	0.011932	0.058154	0.030173	0.047116	0.017787	0.005977	0.029863
LOC10112	0.192696	0.146419	0.190295	0.098735	0.096361	0.485032	0.097793	0.684039
LOC10112	3.291243	3.527338	4.270968	3.177649	3.781338	3.039865	3.561478	2.538056
LOC10112	2.409083	2.810503	2.263717	2.506162	3.088406	2.241788	2.874985	2.924649
LOC10112	27.07245	30.56968	30.18295	26.54377	28.46095	29.49611	19.77742	20.01969
LOC10112	21.51487	20.15284	34.39023	33.74625	39.75264	42.86976	18.08511	12.14521
LOC10112	4.964939	4.278348	9.676839	7.290826	9.909336	11.07439	8.262423	9.075181
LOC10112	7.970424	7.2404	6.406719	12.26233	11.12296	8.418861	7.211998	7.603451
LOC10112	0.147118	0.074524	0.018161	0.131917	0.165529	0.148123	0.093328	0.093258
LOC10112	0.820323	1.065334	0.828868	0.603705	0.92813	0.656915	1.854927	1.577268
LOC10112	23.50004	24.62723	23.84321	26.08791	25.5427	24.30263	25.5508	25.16014
LOC10112	11.12061	9.98407	10.34835	10.04105	9.819061	9.368007	8.480859	10.82926
LOC10112	1.315677	0.653897	1.532174	1.348267	2.668935	1.42464	2.658226	2.026787
LOC10112	4.316498	5.719979	4.705593	4.464452	5.029891	5.111976	4.511301	4.686595
LOC10112	3.252018	2.88286	0.900786	3.535777	3.113614	3.933074	1.559828	1.548598
LOC10112	8.68277	4.799388	9.02853	7.77253	9.80769	9.230615	12.16973	8.806375
LOC10112	0.033995	0.045921	0	0.011612	0.045332	0.022818	0.034505	0
LOC10112	4.070375	3.708822	3.665726	4.424795	4.617102	3.788886	4.763705	4.742808
LOC10112	0	0	0	0	0	0	0	0
LOC10112	0	0	0	0	0.029528	0	0	0
LOC10112	9.455799	11.12429	9.168197	12.77908	11.44013	10.81286	10.36545	12.10135
LOC10112	34.28315	35.1437	43.56779	27.96174	40.20088	34.73339	28.52999	32.4378
LOC10112	9.817738	12.50179	16.19546	7.514176	9.412958	16.27649	10.67679	5.69
LOC10112	0	0.035713	0.034812	0.036124	0.035255	0.035492	0	0
LOC10112	32.99041	31.72347	30.93037	29.29715	29.15206	31.12654	32.68722	31.42121
LOC10112	2.473883	2.761948	2.043944	2.572812	2.547694	2.229374	2.622867	2.812179
LOC10112	33.59312	27.28614	30.08995	40.168	34.14874	28.22662	27.63195	20.51424
LOC10112	1.301822	0.791344	8.806365	8.671443	1.106691	18.74323	1.453484	1.353363

LOC10112	1.00521	1.445475	0.629768	1.451015	1.237758	1.093697	1.091599	1.183959
LOC10112	0	0	0	0.028582	0	0.014041	0	0
LOC10112	0.114009	0.101067	0.098515	0.292082	0.185288	0.344363	0.332692	0.317987
LOC10112	0.362986	0.377689	0.455345	0.5831	1.069475	0.58277	0.706989	0.537305
LOC10112	0.069798	0.035357	0.034464	0.023842	0.093077	0.011713	0.04723	0.023597
LOC10112	0	0	0	0	0	0	0.012094	0
LOC10112	15.99381	18.66887	17.95854	17.67318	17.78901	16.44691	15.39603	14.46079
LOC10112	0	0.008549	0	0	0	0	0	0.008558
LOC10112	0.009866	0	0	0	0	0	0.010014	0
LOC10112	0	0	0	0	0.032778	0.230987	0.099797	0.132963
LOC10112	0.686009	0.834015	0.858117	0.796735	0.594618	1.289298	0.928399	0.603005
LOC10112	6.674681	6.581952	6.41574	4.879211	3.604794	3.404942	4.155212	3.565375
LOC10112	1.128162	0.888975	1.008	0.697334	1.00294	0.667098	1.4359	0.9626
LOC10112	3.059544	4.993957	3.301183	4.470749	4.07992	5.248167	4.313108	3.160564
LOC10112	0.451638	0.238185	0.482669	1.134872	1.528342	0.909446	0.200948	0.26982
LOC10112	3.283305	5.194926	2.041508	3.426942	5.290453	2.142606	3.353126	2.076139
LOC10112	0.097815	0.099099	0.0138	0.042959	0.139754	0.042207	0.04255	0.085035
LOC10112	0.870251	1.024646	0.766499	1.205146	1.481971	1.468221	1.193656	1.312032
LOC10112	1.78302	1.916627	0.896749	0.891783	1.329161	0.747604	1.099301	1.453438
LOC10112	1.82626	2.072723	2.003259	2.635494	2.705063	1.891105	1.894716	1.805365
LOC10112	0	0	0	0	0	0	0	0
LOC10112	0.012363	0	0	0.012669	0	0	0	0.012539
LOC10112	0	0	0	0.012128	0	0.011916	0	0
LOC10112	0	0	0	0	0	0	0	0
LOC10112	0.009665	0.039166	0.057266	0.049521	0.067662	0.077846	0.019619	0.137233
LOC10112	0	0	0.025905	0	0	0	0	0
LOC10112	0.023984	0	0.023685	0	0	0	0	0.024325
LOC10112	12.27111	14.52746	14.60313	11.02085	14.44455	14.36783	13.85464	11.81652
LOC10112	0	0	0	0	0	0	0	0
LOC10112	22.3994	23.18404	12.53484	19.56281	38.24537	51.46423	15.25934	14.20404
LOC10112	1.184727	2.060472	1.754947	2.094275	2.488247	3.657981	2.865952	0.941247
LOC10112	7.224243	4.191998	4.086138	6.443517	6.613522	4.339143	5.247276	5.359627
LOC10112	0.215855	0	0.23685	0	0.023987	0.024148	0.073031	0.194602
LOC10112	0.037544	0.133129	0.148305	0.096185	0.075098	0.056701	0.247698	0.114236
LOC10112	451.4549	333.6264	473.4701	320.199	221.6959	380.7829	447.9679	320.5872
LOC10112	0.046649	0	0	0.047804	0	0.046967	0	0.047313
LOC10112	1.989167	1.58725	1.790543	1.587457	1.584497	1.683733	1.304313	1.428307
LOC10112	2.070686	1.980005	3.607269	2.312718	3.769602	2.576759	1.487753	1.60462
LOC10112	3.546975	3.269938	4.200015	6.770096	7.885054	3.706608	6.796548	9.047662
LOC10112	2.507924	2.434391	2.201489	2.570046	2.037666	2.069719	2.578	2.594591
LOC10112	8.464642	9.258018	7.423766	8.881914	9.133795	8.472855	9.108442	9.756852
LOC10112	0.502756	0.494802	0.375915	0.287045	0.560287	0.339871	0.517588	0.502629
LOC10112	1.803388	2.738107	2.109108	2.25373	3.441054	1.613962	1.359187	1.873671
LOC10112	69.22051	73.12261	27.72147	62.8603	40.90832	97.84622	122.8557	110.4703
LOC10112	0	0.016749	0.016326	0	0	0	0	0
LOC10112	0.029339	0	0	0	0	0.029539	0	0
LOC10112	0	0	0	0	0	0	0	0
LOC10112	0	0	0	0	0	0	0	0
LOC10112	1.442412	1.885777	1.534415	1.749859	1.394866	1.324125	1.851591	1.549002
LOC10112	2.632378	2.77578	2.175157	2.532425	2.901363	2.758539	3.762421	3.160233
LOC10112	0	0	0	0	0	0	0.081635	0
LOC10112	42.77206	40.6132	36.72028	41.86392	30.84983	39.07217	42.27285	40.26196
LOC10112	0.090236	0.166708	0.104838	0.048955	0.143335	0.18705	0.199343	0.107672
LOC10112	0.248793	0.378087	0.214981	0.414302	0.435443	0.062623	0.094697	0.473127
LOC10112	0	0.105024	0.025593	0.026558	0	0	0	0
LOC10112	0.700403	0.65501	1.409954	1.214657	1.293218	1.274762	0.765594	1.366102
LOC10112	0	0	0	0	0.003401	0	0	0
LOC10112	2.061304	1.614243	1.573479	2.295054	2.440454	1.402288	1.538067	1.955038

LOC10112	1.255308	1.137911	0.693976	0.812465	1.165365	1.112701	0.731562	0.694461
LOC10112	6.624301	6.032725	5.788706	6.435038	6.578735	5.928494	6.29292	6.483216
LOC10112	0.208615	0.739735	0.034336	0.302858	0.295576	0.10502	0.229389	0.052896
LOC10112	0	0.079877	0	0	0.919951	0	0	1.999127
LOC10112	4.052841	3.396807	2.995692	4.908364	5.785257	1.879516	3.078992	5.206678
LOC10112	0.08488	0.343976	0.083822	0.217456	0.339564	0.192285	0.301536	0.430441
LOC10112	3.84165	6.522421	6.691232	7.159801	9.119821	9.435962	8.248466	6.315494
LOC10112	2.235096	1.986854	0.754736	2.024471	1.456605	1.81481	1.96126	1.813531
LOC10112	0.204799	0.169762	0.116445	0.101756	0.148964	0.15621	0.081888	0.088121
LOC10112	0.831876	1.062271	0.830068	1.411923	2.001961	0.697966	1.407257	2.56631
LOC10112	1.074152	1.872013	1.571915	1.625473	2.020557	1.305633	1.248437	2.139372
LOC10112	0	0	0	0	0	0	0	0
LOC10112	0.163892	0.202942	0.305717	0.317243	0.318721	0.201681	0.369668	0.591024
LOC10112	0.050797	0.205855	0	0	0	0	0.360913	0
LOC10112	1.294358	0.997056	0.665525	2.022517	2.492765	1.949416	1.465781	1.985451
LOC10112	1.059049	0.591216	2.945465	1.328917	0.907874	4.286899	1.29431	1.030284
LOC10112	0	0	0	0	0	0	0	0
LOC10112	0	0.019031	0	0	0	0	0	0
LOC10112	0.096514	0.032594	0.317706	0.164842	0	0	0	0.065259
LOC10112	0.005747	0.011645	0.022703	0.00589	0.011496	0.005787	0	0
LOC10112	7.439225	8.805437	0.290952	0.452881	8.4715	0.222477	1.270929	0.597634
LOC10112	0	0.010311	0	0	0	0	0.020661	0
LOC10112	1.80668	1.864827	1.598264	1.720144	2.063177	1.70846	1.528593	2.171831
LOC10112	8.558478	8.882474	8.253151	8.36605	9.991342	7.549566	8.097783	10.79153
LOC10112	0.026505	0	0.026175	0.081484	0	0	0	0
LOC10112	0.333886	1.111451	0.584085	0.420363	1.030409	0.42261	0.455086	2.302744
LOC10112	0	0	0	0	0	0	0	0
LOC10112	0	0	0	0	0	0	0	0
LOC10112	0	0.085104	0.029034	0.077474	0.100814	0.10149	0.059683	0.00852
LOC10112	8.639412	8.800843	7.2399	8.440308	9.419204	8.308475	7.894509	9.387712
LOC10112	0.251185	0.418077	0.088591	0.349338	0.233273	0.090322	1.038021	0.673296
LOC10112	0.398049	1.103696	0.910313	0.343503	1.152397	0.316396	1.467231	1.444878
LOC10112	0.41894	0.293064	0.157607	0.439539	0.618515	0.190815	0.435348	0.354086
LOC10112	0.024212	0	0	0.049624	0.024215	0	0	0
LOC10112	0	0	0	0	0.014017	0.014111	0	0
LOC10112	0	0	0	0	0	0.073006	0	0
LOC10112	7.357437	2.573678	3.347905	9.435083	13.71233	8.876429	5.456706	7.499048
LOC10560	4.591532	6.434787	3.297688	3.872884	5.630149	3.884598	6.503955	4.942945
LOC10560	392.3315	269.0666	397.721	299.6232	305.3927	217.5091	332.9475	252.0955
LOC10560	15.24397	16.10313	8.556409	10.576	11.34203	15.77982	12.83132	8.682739
LOC10560	5.328806	0.157245	3.780759	0.21207	0	3.177454	3.413281	0.20989
LOC10560	8.681229	8.943917	8.174313	8.764609	9.820419	10.36668	9.239912	9.046805
LOC10560	0.225901	0.366186	0.200778	0.393545	0.29371	0.386656	0.481509	0.366587
LOC10560	0	0	0	0	0.248604	0.500539	0.10092	0.100844
LOC10560	1.367708	0.989755	0.906876	1.35153	1.328802	1.9082	1.437803	1.456537
LOC10560	83.02138	96.65171	95.22318	95.2581	94.1898	106.0535	101.5522	87.40185
LOC10560	0.092679	0.328635	0.228811	0	0.185383	0	0	0.187997
LOC10560	0.001472	0	0	0.001509	0	0.001482	0.002989	0
LOC10560	0	0	0	0	0	0	0	0
LOC10560	0.018659	0.018904	0.036854	0.019122	0.223942	0.112721	0.094697	0.03785
LOC10560	0	0.015213	0	0	0	0	0	0
LOC10560	1.009901	0.878907	0.967889	1.028131	0.718613	0.983463	0.853648	0.792556
LOC10560	1.931819	1.540992	1.474667	2.269794	2.287391	1.766164	1.560748	1.751001
LOC10560	0	0	0	0	0	0	0	0
LOC10560	0.112708	0.228374	0.222607	0.423499	0.300594	0.245869	0.095332	0.838292
LOC10560	0.086754	0.439465	0.214184	0.111129	0.412137	0.163776	0.451286	0.439947
LOC10560	0	0	0	0	0	0	0	0
LOC10560	1.819072	1.503753	1.399654	1.452422	1.294722	1.235981	1.585829	1.969475

LOC10560	1.16043	1.487158	1.29289	1.250157	1.061385	1.188329	1.248305	1.599444
LOC10560	0.292188	0.222017	0.240456	0.299425	0.791444	0.392245	0.951498	1.173042
LOC10560	1.494643	1.296418	1.015087	1.348941	1.416161	1.077159	1.314788	1.755277
LOC10560	4.532624	4.985475	5.226339	4.316209	4.499443	4.895023	4.806227	4.383129
LOC10560	0.083967	0.070891	0.248764	0.057365	0.083978	0.112721	0.15625	0.099357
LOC10560	2.194034	1.862648	1.96106	2.092243	2.392415	1.784604	1.989816	2.276778
LOC10560	0	0	0	0	0	0	0	0
LOC10560	1.268478	1.50356	1.135567	1.524527	1.484275	1.179458	1.331273	1.538011
LOC10560	1.538122	1.400904	1.043324	0.947325	0.707006	0.727387	0.938293	1.630928
LOC10560	10.8402	11.13367	13.33117	10.96969	16.35061	12.14365	13.30252	15.4804
LOC10560	0.364572	0.414127	0.13092	0.288693	0.408815	0.339255	0.21866	0.196086
LOC10560	0.28288	0.121095	0.149514	0.036746	0.15939	0.100286	0.048528	0.161638
LOC10560	3.772616	3.165199	2.725409	2.8007	3.301465	2.76786	3.530048	3.570871
LOC10560	11.71907	12.14238	10.44414	12.01672	11.59835	11.08249	12.42027	12.20674
LOC10560	17.19378	20.55308	20.78272	22.24989	21.62389	21.46349	21.97619	21.55982
LOC10560	0.789188	0.624303	0.555157	0.697951	0.497361	0.533348	0.768107	0.701741
LOC10560	2.856037	3.13347	2.875484	2.598412	2.591666	2.328493	2.884737	2.868434
LOC10560	1.119235	0.965935	0.990667	0.934559	0.945254	0.843074	1.068704	1.479923
LOC10560	0.032621	0	0.12886	0	0.097877	0	0	0
LOC10560	0.572732	0.470926	0.311487	0.493352	1.18712	0.835717	0.05055	0.193628
LOC10560	1.556238	1.646909	1.68901	1.444782	1.425451	1.590139	1.634319	1.71904
LOC10560	0.992021	1.333217	1.029643	1.265555	1.295868	1.00899	1.068548	1.139611
LOC10560	0	0	0	0	0	0.390345	0.012694	0.050737
LOC10560	0.165944	0.308223	0.355065	0.170054	0.221287	0.083539	0.39301	0.112204
LOC10560	0	0	0	0	0	0	0	0
LOC10560	0.011341	0.01149	0.022399	0.052298	0.05104	0	0.017266	0.017253
LOC10560	4.988013	4.126741	1.86843	3.364982	3.354442	3.093544	3.555093	4.44052
LOC10560	0	0.062505	0	0	0.020568	0	0	0
LOC10560	5.345499	5.258678	3.770638	4.740716	4.859166	6.841736	6.043513	4.41136
LOC10560	0	0	0.049314	0	0	0	0	0
LOC10560	0.03405	0.241479	0.033626	0.209361	0.068109	0.102848	0.207365	0.06907
LOC10560	1.48208	3.046209	2.826287	4.407987	4.208288	4.845378	5.420707	2.228851
LOC10560	0	0	0	0	0	0	0	0
LOC10560	0.84221	0	0.670217	0.997141	0	0.008233	0	0
LOC10560	0.145625	0	0	0.059693	0.029129	0	0.029562	0.059079
LOC10560	0	0.045082	0.219717	0	0	0	0.090331	0
LOC10560	0.558666	0.441478	0.375159	0.194651	0.223495	0.303741	0.294864	0.47596
LOC10560	0	0.153278	0.149408	0.271321	0.037828	0.22849	0.153562	0
LOC10560	0.523706	0.884298	0.689574	0.894464	1.367631	0.849512	0.974529	0.56067
LOC10560	0	0	0	0	0	0	0	0.019052
LOC10560	3.135854	3.075615	1.762529	2.786199	2.352194	1.477874	1.608376	2.656052
LOC10560	0.123736	0.025072	0	0.101441	0	0	0	0
LOC10560	0.043583	0.070648	0.111905	0.062528	0.034871	0.026329	0.026542	0.053044
LOC10560	1.021612	0.563172	0.474768	0.554251	0.525898	0.665559	0.853947	0.670453
LOC10560	2.552909	2.680465	2.765569	3.313784	2.25923	3.177886	3.187964	2.008631
LOC10560	0.724288	0.539802	0.065771	0.383912	0.499574	0.771146	0.515452	0.692381
LOC10560	0.975657	0.741346	1.104011	1.29144	0.569207	0.716277	2.021846	1.752321
LOC10560	5.192958	1.09978	2.028123	1.623536	2.435431	2.008664	1.78673	1.606846
LOC10560	0	0.007844	0	0	0	0	0.007859	0
LOC10560	0.511218	0.615038	0.836154	0.311054	0.814858	0.386033	0.502671	0.291654
LOC10560	93.52132	81.69793	70.77001	79.41554	81.03384	71.53741	83.68456	104.3027
LOC10560	0.043655	0.063183	0.012317	0.083082	0.018712	0.018837	0.08862	0.126504
LOC10560	0.082321	0	0	0	0	0	0	0
LOC10560	0.139052	0.080501	0.117702	0.295171	0.27814	0.130002	0.181463	0.070516
LOC10560	0.199093	0.184897	0.016384	0.119014	0.033186	0.050113	0.03368	0.067309
LOC10560	0.727887	0.95479	0.506955	1.02858	0.796948	0.601716	0.715474	0.567286
LOC10560	1.391274	0.428388	0.646559	0.377401	1.03677	0.411994	0.623004	1.092895
LOC10560	0	0	0	0	0	0	0	0

LOC10560	0	0	0.011003	0.022836	0	0	0	0
LOC10560	0.141359	0.071607	0.093065	0.096574	0.047126	0.118604	0	0.143372
LOC10560	4.23403	7.766428	11.61954	17.03592	34.09949	29.61632	30.17326	43.30466
LOC10560	1.587445	1.735248	0.937063	1.492222	1.605556	1.400006	1.617314	1.682677
LOC10560	0	0	0	0.01887	0	0	0	0.018676
LOC10560	0.100862	0	0	0	0.252187	0	0.20475	0.102298
LOC10560	0	0	0	0	0.018625	0	0.056705	0
LOC10560	0	0.157536	0.230337	0.079674	0.025919	0.234836	0.105219	0.05257
LOC10560	9.65011	2.064415	54.17977	36.36527	1.807226	11.6515	7.960764	39.11095
LOC10560	0.193195	0.146798	0.023849	0.049495	0.19322	0.048629	0.073535	0
LOC10560	3.167627	3.327162	2.747826	2.661733	3.377049	2.678263	2.809082	3.461017
LOC10560	0.243838	0.299975	0.086	0.356969	0.174193	0.508544	0.212139	0.335634
LOC10560	0.018282	0.037043	0.090269	0.037469	0.127988	0.036813	0.037112	0.092709
LOC10560	0.392005	0.542771	0.154848	0.830211	0.470467	0.328903	0.251994	0.318068
LOC10560	111.6832	66.30786	87.41343	42.13215	66.84955	64.46949	70.49267	67.06131
LOC10560	0.83211	0.728072	0.522927	0.542641	0.870046	0.418897	0.652639	0.421978
LOC10560	63.16922	34.66105	70.68517	27.40706	50.89597	32.52438	55.38633	51.52968
LOC10560	68.0623	45.87819	72.6051	50.44859	61.09772	59.08385	55.23133	54.35925
LOC10560	0.040756	0.010323	0.040248	0.052207	0.081522	0.010259	0.031025	0.020668
LOC10560	0.114108	0.049545	0.112687	0.06682	0.065213	0.06565	0.066183	0.0496
LOC10560	0	0	0	0	0	0	0	0
LOC10560	0.156949	0.176676	0.068886	0.125095	0.069764	0.035116	0.070801	0.141496
LOC10560	0.237122	0.044488	0.069383	0.089998	0.175669	0.053054	0.106968	0.035629
LOC10560	0.058457	0	0	0	0.035079	0.023543	0.023734	0
LOC10560	0.049291	0	0.024338	0.067349	0.016433	0.008271	0.008338	0.033329
LOC10560	0	0	0	0	0	0	0	0.082045
LOC10560	0.009715	0.019685	0	0.019911	0	0	0	0
LOC10560	0	0	0	0	0	0	0	0
LOC10560	0	0	0	0.01626	0	0	0	0
LOC10560	0	0.01267	0	0	0	0	0	0
LOC10560	0	0	0	0.069281	0	0	0	0
LOC10560	0.02774	0	0	0	0	0	0	0
LOC10560	0.452644	0.605836	0.418297	0.553223	0.660364	0.380477	0.425714	1.25512
LOC10560	0	0	0	0	0	0	0	0
LOC10560	4.365998	4.660259	3.84964	4.314354	0	0	3.719284	0.079074
LOC10560	1.567393	2.041668	2.137525	0.305946	0.746474	1.728396	0	1.741107
LOC10560	3.360468	2.497432	2.805524	2.14099	1.65834	4.151374	4.286034	6.951152
LOC10560	0.02283	0.092517	0.135271	0.093581	0	0.183885	0.185377	0.069464
LOC10560	0	0.073084	0.071238	0	0	0	0.073219	0
LOC10560	0.101096	0.16095	0.014262	0.1036	0.231107	0.174491	0.058636	0.043944
LOC10560	52.35784	162.803	100.1564	104.2177	86.67085	63.04365	187.3675	273.472
LOC10560	0	0	0.047738	0	0	0	0	0
LOC10560	0.856643	2.186404	3.774324	4.017909	2.059504	4.146612	2.993061	5.530488
LOC10560	4.079066	4.981988	4.012318	3.943288	4.187092	3.490081	4.363902	4.763973
LOC10560	0	0	0	0	0	0	0	0
LOC10560	2.843022	3.146025	3.861182	3.316563	4.405167	2.730424	3.00061	3.113207
LOC10560	1.222457	0.709557	1.307827	1.161392	2.343345	1.448764	0.878895	1.007386
LOC10560	5.858197	5.955905	6.597161	5.989264	11.04636	8.767994	3.845821	6.553125
LOC10560	0.304794	0.321934	0.294592	0.43861	0.324291	0.195878	0.388353	0.41437
LOC10560	0.831007	0.660466	0.622564	0.712106	0.673489	0.238022	0.901642	1.256989
LOC10560	1.692734	0.039883	0	1.936369	0.39371	0	0	0.199632
LOC10560	0.932407	0.910293	0.318091	0.451692	0.525606	1.02412	0.774322	0.636185
LOC10560	0.399845	0.445602	0.282985	0.40292	0.346577	0.308642	0.696697	0.533957
LOC10560	0.308988	0.104348	0.025428	0.448576	0.206019	0.025925	0.156811	0.47008
LOC10560	3.817559	4.852041	3.720468	4.14338	4.95281	3.420775	3.194024	5.041741
LOC10560	0.19352	0.237336	1.66969	0.250502	0.896421	1.999699	0.26879	1.446239
LOC10560	0.869137	0.611903	0.305499	0.603841	0.883983	0.192813	0.627988	1.523966
LOC10560	0	0	0.014299	0	0	0	0	0

LOC10560	0	0	0.045914	0.031764	0	0.046811	0	0
LOC10560	0.015024	0.654498	0.445095	0.3695	0.841436	0.272274	0	0.015238
LOC10560	0.365076	0.431512	0.120176	0.397502	0.174955	0.260362	0.162117	0.069426
LOC10560	0.058117	0.129535	0.034436	0.202492	0.034875	0.093623	0.047191	0.011789
LOC10560	0.479878	1.164561	0.826567	0.932066	1.110559	0.039327	0.509731	0
LOC10560	0	0.025183	0	0	0	0	0.012615	0.012606
LOC10560	8.397886	7.978502	6.800687	7.988701	8.165982	10.46324	9.348557	7.076732
LOC10560	4.999505	4.909897	5.23185	5.371248	4.556589	3.848314	5.631055	4.367324
LOC10560	0	0.017259	0.016823	0.122202	0	0	0	0.017278
LOC10560	0	0.019409	0	0	0	0	0	0
LOC10560	0.071975	0.747423	0.133271	0	0	0.018117	0.15524	0.228123
LOC10560	0.267679	0.169495	0.11565	0.034289	0.117125	0.269507	0.050943	0.644787
LOC10560	1.833384	1.377859	1.164979	1.609298	1.378973	1.244476	1.71598	1.581323
LOC10560	0.436999	0.178323	0.308681	0.348307	0.160861	0.128329	0.172493	0.393973
LOC10560	0	0	0	0.05725	0	0.028124	0.141761	0.141655
LOC10560	0	0.007344	0	0	0	0	0	0
LOC10560	0.531287	0.489327	0.405425	0.346466	0.362288	0.401187	0.245116	0.477617
LOC10560	0.090171	1.059714	0.463048	0.683796	1.100232	0.526566	0.604057	0.237783
LOC10560	0	0	0	0	0	0	0	0
LOC10560	0	0	0	0	0	0	0	0.010278
LOC10560	0.73809	0.610029	0.345265	0.318473	0.388519	0.840913	0.354866	0.492498
LOC10560	0.23528	0.181467	0.208364	0.211552	0.306662	0.149774	0.171018	0.138559
LOC10560	3.206252	3.820363	3.923027	3.698964	4.033542	3.654515	4.134451	2.863303
LOC10560	0.942396	1.145717	2.140504	2.607497	1.60228	1.280926	1.147838	1.529299
LOC10560	2290.845	3175.581	4058.59	2716.789	2535.566	3342.381	2848.955	2606.986
LOC10560	0.026682	0.036043	0.035132	0.072914	0.01779	0.071637	0.036109	0.036082
LOC10560	0.026405	0.026751	0.234683	0.067647	0.026408	0.013293	0.026801	0.093733
LOC10560	3.381044	4.58708	3.977665	4.097495	4.263607	5.417026	4.207636	4.860483
LOC10560	0.104049	0.105414	0.051376	0	0.052031	0	0.052805	0.052765
LOC10560	1.415439	2.174152	1.758526	2.292726	1.461287	1.654965	1.76108	2.222847
LOC10560	0	0	0	0.020982	0.020478	0.020615	0	0.145364
LOC10560	0	0	0	0	0	0.027201	0	0
LOC10560	0	0.012286	0	0	0.024257	0	0.012309	0
LOC10560	0.864834	0.773773	1.630474	1.761002	0.921111	0.802892	0.661205	0.512617
LOC10560	117.3605	139.9575	123.0477	148.7725	156.8358	116.1045	135.6108	114.0774
LOC10560	0	0	0	0.031042	0.030295	0.030498	0	0
LOC10560	1.003859	1.298056	1.121791	0.812151	0.435942	0.718141	0.67034	0.669835
LOC10560	0.669232	0.600773	0.736185	0.68581	0.559178	0.605571	0.352533	0.472554
LOC10560	0.126853	0.128518	0.125272	0.157851	0.199366	0.155088	0.147149	0.238937
LOC10560	1.168582	1.508674	1.306288	1.47612	1.675996	1.564668	1.985088	1.576175
LOC10560	0.735822	0.349148	0.450709	0.591785	0.326039	0.77836	0.444334	0.434552
LOC10560	0.210841	19.22474	1.873926	110.6247	0	0.424563	0	0
LOC10560	0.819503	1.341186	0.435772	1.227403	0.693515	0.380816	1.919527	1.598401
LOC10560	1.600397	0.540467	0	0.583125	0.960362	0.859376	0.397076	0.577131
LOC10560	4.164416	3.603411	2.594598	5.12841	3.41419	4.17487	3.120274	4.676886
LOC10560	0	0	0	0	0	0	0.224577	0
LOC10560	0.516496	0.390379	0.380521	1.024974	0.951131	0.676857	1.090094	0.606999
LOC10560	4.887421	2.651657	5.195765	4.899009	5.101738	4.692247	5.340242	5.241414
LOC10560	0.100983	0.042629	0.141277	0.284582	0.252491	0.194873	0.17083	0.273122
LOC10560	0	0.118895	0	0	0	0	0.119115	0
LOC10560	0	0	0	0	0	0.037524	0	0
LOC10560	26.73074	26.95943	24.11216	26.4442	25.66849	25.04922	25.56076	24.32385
LOC10560	0.051316	0.022281	0.072395	0.03005	0.014664	0.036905	0.022322	0.342019
LOC10560	0.014026	0.01421	0	0.007187	0	0.007061	0	0
LOC10560	5.349809	12.54932	9.042588	11.82173	8.061806	9.291828	15.58763	16.13165
LOC10560	2.174543	1.777734	1.956091	1.643726	1.916429	1.950949	1.507862	2.172744
LOC10560	3.618852	0.871719	2.049289	1.374476	1.088327	1.936445	0.899019	3.465034
LOC10560	0.018116	0.899332	0.053671	0	0.126828	0	0	0

LOC10560	17.37994	18.39121	16.37366	19.42213	18.5019	16.02256	19.72396	19.5469
LOC10560	0	0	0.007875	0	0	0	0	0.008088
LOC10560	40.7696	81.05555	79.51352	40.46978	84.68136	87.5007	83.34605	90.86627
LOC10560	0	0	0	0	0	0	0	0
LOC10560	0	0	0	0	0.058808	0	0	0
LOC10560	28.22647	27.01522	21.31309	23.88926	23.35855	24.4582	24.55522	21.53455
LOC10560	0.107473	0.124439	0.227431	0.17307	0.092132	0.247332	0.109086	0.186863
LOC10560	0	0	0	0	0	0	0	0.033997
LOC10560	0.062756	0.174844	0.108455	0.032155	0.172601	0.078981	0.222941	0.254597
LOC10560	0.063272	0.040792	0.068164	0.058945	0.109302	0.09266	0.052544	0.081674
LOC10560	0.064136	0.064978	0.063337	0.13145	0.12829	0.193724	0.078118	0.195149
LOC10560	0	0.029173	0.028437	0.088526	0	0.057984	0.029227	0.146027
LOC10560	0	0.046259	0	0.04679	0	0.183885	0	0
LOC10560	1.749323	0.801168	0.804601	2.185565	1.126422	1.109843	1.167493	1.482572
LOC10560	0.04112	0.08332	0.121824	0.042139	0.027417	0.041401	0.069562	0.027804
LOC10560	0.976366	1.263952	0.982056	0.87085	1.030742	0.600744	0.954308	0.898574
LOC10560	3.676113	3.544562	13.35703	13.39294	10.04089	5.704994	8.427489	2.622768
LOC10560	0	0	0	0.039948	0	0	0	0
LOC10560	0	0.055384	0.053985	0.05602	0.218694	0.05504	0	0
LOC10561	0	0	0	0	0	0.011512	0	0.046385
LOC10561	8.23855	7.076795	6.886069	7.906384	7.010366	7.388159	8.016279	6.121849
LOC10561	0.848156	1.33667	1.395981	1.110601	0.659763	1.423251	1.482625	1.529299
LOC10561	325.5136	419.4841	530.6158	388.1671	435.3944	521.6947	418.2685	385.0692
LOC10561	0	0	0	0	0	0	0	0.090986
LOC10561	5.441272	4.105355	4.236631	5.528866	5.626189	4.07213	3.715682	4.288887
LOC10561	0.185851	0.301264	0.513899	0.076182	0.22305	0.823331	0.264094	0.339294
LOC10561	0.019383	0.039275	0	0.099316	0.019386	0.019515	0.019674	0
LOC10561	0.76437	0.592752	0.531188	0.406158	0.67009	0.513061	0.680054	0.47855
LOC10561	3.410792	4.062895	3.337672	2.986874	3.152806	3.038652	3.357046	3.857696
LOC10561	0.403967	0.662626	0.797868	0.965939	1.077385	0.671422	1.03483	0.591815
LOC10561	0	0	0	0.071527	0	0.070275	0	0
LOC10561	0.137385	0.683289	0.283681	0.179185	0.474666	0.188624	0.190154	0.608034
LOC10561	0.10032	0.101636	0	0.051402	0	0.050502	0.050912	0.203495
LOC10561	2.275958	1.515628	1.16168	1.899935	1.777523	1.441846	1.868843	1.958213
LOC10561	0	0	0	0	0	0	0.025052	0
LOC10561	0.011263	0.011411	0	0	0	0.01134	0	0
LOC10561	0	0	0	0.016269	0	0	0.016114	0.032204
LOC10561	0	0	0	0	0	0	0	0
LOC10561	0.258589	0.212081	0.072962	0.239757	0.135469	0.099183	0.174978	0.187336
LOC10561	5.223604	5.805577	7.891762	7.430277	7.251612	7.967432	7.0429	6.681763
LOC10561	6.137433	5.487681	1.220327	7.450265	4.078419	11.52928	8.591685	6.621686
LOC10561	0	0	0	0	0	0	0.008164	0
LOC10561	0.366324	0.411254	0.449756	1.227654	0.30696	0.358859	1.246097	0.331373
LOC10561	1.987471	0.782337	0.937599	1.28429	1.29139	0.739241	1.45193	1.91305
LOC10561	9.724908	11.8516	9.333552	8.725383	12.2883	10.5426	9.273318	8.744998
LOC10561	23.50605	73.71461	99.4337	69.347	74.34447	85.02909	79.81577	66.54498
LOC10561	1.874274	1.898872	1.727526	2.47557	2.915915	2.180624	1.944664	2.027686
LOC10561	0	0	0	0	0	0	0	0.008907
LOC10561	2.424385	3.011092	2.71234	2.19555	1.917204	2.173335	2.174617	1.976921
LOC10561	0	0	0	0	0	0.063469	0	0
LOC10561	0	0.044307	0.014396	0.029878	0	0	0	0.014785
LOC10561	3.504565	3.166715	3.019934	2.336474	3.592982	1.246557	4.896209	9.640945
LOC10561	0	0	0	0	0.043	0	0.130917	0.043606
LOC10561	0	0.060397	0	0	0	0	0	0
LOC10561	0	0.055061	0.617213	1.837906	0	0.02736	0.055163	0.771702
LOC10561	0.012585	0	0	0	0	0	0	0
LOC10561	0.003402	0	0.002519	0.013072	0.004253	0	0.000863	0.001725
LOC10561	0	0	0	0	0	0.02193	0.110541	0.022092

LOC10561	0.381021	0.257348	0.235171	0.959878	0.333436	0.527483	0.290052	0.144917
LOC10561	0.312598	0.274158	0.234983	0.296435	0.47129	0.300641	0.568275	0.421153
LOC10561	0.751387	0.888123	0.989366	0.385	0.313118	0.75652	1.58887	1.333646
LOC10561	0.026049	0	0	0.053388	0.026052	0.052453	0.026439	0
LOC10561	7.846019	10.61321	7.205453	10.41493	10.1106	11.11155	9.363922	6.941136
LOC10561	0.088761	0.05995	0	0	0.059181	0.089367	0	0.330088
LOC10561	0	0.157536	0.358302	0.106231	0.051838	0.156558	0.420874	0.262848
LOC10561	1.149115	0.784051	0.53266	0.660886	0.83263	0.625706	0.606979	0.998977
LOC10561	5.195178	5.087523	3.370782	4.327863	4.91812	3.378383	4.709388	4.647165
LOC10561	0.246963	0.166803	0.040648	0.08436	0.164663	0.041442	0.041778	0.083493
LOC10561	0.363496	0.136395	0.345671	0.386296	0.309684	0.447308	8.513135	4.123645
LOC10561	0	0.007139	0.006959	0.007221	0.007048	0	0	0.042882
LOC10561	0	0	0	0	0.055431	0	0	0
LOC10561	0.024704	0.125139	0.024396	0.050631	0	0	0	0.050111
LOC10561	1.794844	1.264974	2.979822	2.159182	2.341404	1.807106	1.768962	1.952308
LOC10561	0	0	0	0.12247	0	0.008022	0	0.016161
LOC10561	0.090055	0.675155	0.05336	0.018457	0.036027	0	0.109687	0.07307
LOC10561	0.757317	0.330346	1.402276	1.19645	0.757415	0.465966	0.192169	0.352045
LOC10561	5.548729	5.488889	5.641231	5.61908	6.155138	5.026324	5.366145	3.834817
LOC10561	2.342421	0.476501	0.519112	1.483739	1.189809	0.594248	3.219999	1.711675
LOC10561	0	0	0	0	0	0	0	0
LOC10561	0	0	0.037176	0	0	0	0	0
LOC10561	0.045008	0.034199	0.050003	0	0.005627	0	0.062814	0.085591
LOC10561	0	0.023474	0.022881	0.023744	0.046346	0	0.047035	0.0235
LOC10561	3.580214	4.83329	6.844791	2.521238	2.539988	3.08972	2.980529	0.795998
LOC10561	0.388738	0.157536	0	0.239021	0.388789	0.156558	0.236742	0.275991
LOC10561	0	0	0	0.123525	0	0	0	0.048902
LOC10561	0	0.909947	0	0.01427	0.320315	0.497713	0.494684	1.708906
LOC10561	0.117109	0.21904	0.053377	0.203094	0.21623	0.06349	0.15544	0.146187
LOC10561	0.098743	0.063661	0.070918	0.128786	0.062845	0.108456	0.054668	0.136566
LOC10561	10.141	12.32891	9.842963	6.591623	9.852532	12.42737	8.822671	9.109895
LOC10561	0	0	0	0	0	0	0	0.083739
LOC10561	3.68919	5.749166	6.325038	2.992953	5.675425	1.947477	2.62637	5.612561
LOC10561	38.78729	42.09932	38.30399	43.49165	38.73857	38.59251	43.04973	38.57663
LOC10561	0.196704	0.119571	0.105956	0.161261	0.082269	0.144035	0.061711	0.050783
LOC10561	0.062198	0	0.122846	0	0	0.062623	0	0.315418
LOC10561	1.64297	1.842875	0.84988	0.942052	1.780114	0.945254	0.952923	1.805222
LOC10561	0.013024	0.013195	0	0.026694	0	0	0	0
LOC10561	0.226148	0.4178	0.065685	0.340809	0.545487	0.040181	0.445578	0.256352
LOC10561	0.822217	1.939881	1.668436	2.516208	1.836146	0.918555	1.886312	1.05097
LOC10561	0.008552	0	0.01689	0.008763	0.008553	0.01722	0.01736	0.017347
LOC10561	0	0	0	0	0	0	0	0
LOC10561	0.006623	0.00671	0.006541	0.006787	0.013248	0	0.013445	0
LOC10561	6.15279	6.722445	6.473258	5.687041	6.073147	4.777707	5.755272	8.52444
LOC10561	2.030653	5.074681	0.334225	5.514525	0.778518	1.19264	5.084078	5.114575
LOC10561	0.025341	0	0	0	0	0.051028	0	0.012851
LOC10561	0	0	0	0	0	0	0	0.006231
LOC10561	0.20228	0.808953	0.105137	0.01091	0.010648	0	0.237732	0.226755
LOC10561	0	0	0	0	0	0	0.013086	0
LOC10561	0.077532	0.353474	0	0	0.077542	0.156124	0	0
LOC10561	0.053141	0.021535	0.036735	0.016337	0.026574	0.016051	0.021575	0.059287
LOC10561	248.7159	208.692	298.9046	183.3065	189.4692	243.2496	219.725	218.994
LOC10561	176.1961	118.3977	185.4221	101.4879	161.0017	161.013	118.0663	106.8975
LOC10561	1.556154	1.795546	1.430045	1.373215	1.85898	2.263134	1.228497	1.052205
LOC10561	0.212441	0.053807	0.157346	0.054426	0.265586	0.213893	0.107814	0.053866
LOC10561	0.486298	0.766392	0.640318	1.384289	0.864642	0.761632	0.987185	1.150849
LOC10561	0.063252	0.144186	0.031232	0.016205	0.110706	0.14329	0.032101	0.112267
LOC10561	0.515573	0.646395	0.400955	0.719868	0.348057	0.512606	0.379397	0.437939

LOC10561	0.034943	0.017701	0.017254	0	0.017474	0.017591	0	0.03544
LOC10561	0.012869	0	0	0.013187	0	0	0	0
LOC10561	0	0	0	0.023395	0	0.022986	0.023172	0
LOC10561	0.09308	0.075441	0.147072	0.019077	0.027928	0	0.188952	0.481466
LOC10561	85.27502	81.1361	65.94125	76.24725	74.396	63.29801	63.77984	87.11357
LOC10561	0	0.022649	0.022077	0	0	0	0	0
LOC10561	1.14464	0.717886	0.941981	1.312635	0.872219	0.54879	1.410768	1.243858
LOC10561	0.446042	0.150632	0.073414	0.114273	0.408925	0.187121	0.716827	0.075399
LOC10561	1.425374	1.203401	1.428941	1.040182	0.842375	1.935226	2.389337	1.423761
LOC10699	12.08798	11.01102	11.56385	12.75919	13.35563	10.46855	11.10975	8.494362
LOC10699	22.76705	29.46959	19.98522	30.26011	29.34257	19.87717	22.95712	19.6519
LOC10699	261.555	254.788	237.8654	236.5319	235.5022	213.3723	252.7481	240.3359
LOC10699	47.86939	57.61397	57.85964	50.7784	54.42502	59.60043	51.03281	53.56091
LOC10699	2.627097	4.011283	4.045239	3.483235	3.872667	2.507151	3.601675	4.268244
LOC10699	0.040697	0.113385	0	0.062557	0.050877	0	0.061961	0.010319
LOC10699	4.143912	7.748856	22.16845	4.003898	1.492001	5.555014	19.51617	9.918851
LOC10699	0	0.022258	0	0	0	0	0	0
LOC10699	0.009035	0.009153	0.04461	0.037034	0.054215	0.018193	0	0.009163
LOC10699	1.765398	1.670119	1.697218	1.928938	1.964406	2.530819	2.432684	1.577089
LOC10699	0.051088	0.073941	0.032433	0.078531	0.025548	0.069808	0.029631	0.018506
LOC10699	0.997069	1.746125	1.913026	2.028938	1.396078	1.520163	2.183084	1.314642
LOC10699	0	0	0	0	0	0	0.032635	0.016305
LOC10699	0	0	0	0	0	0	0	0
LOC10699	0	0	0	0.024996	0.024395	0	0.024757	0
LOC10699	2.21562	2.244698	0.491187	1.065746	1.311455	1.798263	1.032637	1.834417
LOC10699	5.217603	5.906711	3.177779	7.756893	5.830949	5.579359	6.460815	7.562887
LOC10699	1.95861	2.880456	1.996599	1.553903	1.958863	3.435079	2.052118	1.794251
LOC10699	1.563039	1.032194	1.049873	2.139999	0.949337	1.949626	1.406641	1.668727
LOC10699	0	0.47193	0.047588	0	0.71488	0.008086	1.720029	0.179204
LOC10699	0	0	0	0	0	0	0	0
LOC10699	0.252155	0	0	0.0646	0	0	0.191953	0.063936
LOC10699	356.6397	393.165	407.5316	369.6282	359.685	400.2284	405.1101	408.4661
LOC10699	0	0	0	0	0.104062	0	0	0
LOC10699	0.076237	0.010298	0.015057	0.072917	0.020333	0.051172	0.04127	0.118561
LOC10699	0.052976	0.035781	0.052316	0.036192	0.052983	0.035559	0.017924	0.05373
LOC10699	0.097125	0.034729	0.118483	0.204917	0.045712	0.115045	0.046392	0.034767
LOC10699	3.652746	4.995924	3.787594	4.257919	3.927209	4.551153	5.793027	4.167838
LOC10699	0	0.036947	0.018007	0	0	0.055076	0.055522	0
LOC10699	90.0807	57.26845	55.09133	62.33731	54.53246	46.53488	39.28845	57.97078
LOC10699	1.485288	0.814062	2.560856	1.472175	0.34093	1.83865	1.940067	0.864346
LOC10699	0.011232	0.056895	0	0.034529	0.033699	0	0.0228	0.022783
LOC10699	0.011763	0.063562	0.046467	0.052238	0.043138	0.035531	0.0597	0.015908
LOC10699	0	0	0.035873	0.018613	0.072661	0.182871	0	0
LOC10699	0	0	0	0.148871	0	0	0	0
LOC10699	0.006298	0.019143	0	0.025817	0	0	0.006393	0.012776
LOC10699	0.02478	0.025105	0.061178	0.012697	0	0.037424	0	0.012566
LOC10699	0.040214	0	0	0	0	0	0.040818	0
LOC10699	0	0	0	0	0	0	0	0
LOC10699	0	0	0	0	0.011069	0	0	0
LOC10699	0.141741	0.067014	0.102648	0.019367	0.122858	0.171251	0.057547	0.095839
LOC10699	12.82529	11.89626	10.97594	12.47448	11.95294	11.20275	13.69229	12.55846
LOC10699	0.02728	3.344187	4.983895	1.873027	2.591924	1.373313	6.839204	6.502035
LOC10699	0.868945	1.112019	1.21943	0.515535	0.640358	0.552556	0.417779	0.510235
LOC10699	0.806434	0.717381	0.213664	0.846566	0.727852	0.514891	0.47914	1.017405
LOC10699	5.591699	6.793959	4.593276	6.023527	5.469737	4.785929	6.75466	8.750599
LOC10699	0.046149	0.561052	0.053169	0	0.015385	0.054208	0.093682	0.171621
LOC10699	0	0.026339	0.005135	0.090582	0	0.01047	0.100273	0.063283
LOC10699	0.205299	0.077035	0.007509	0.023376	0.197721	0.022967	0.162073	0.154239

LOC10699	0.069395	0.890535	0.13706	0	0	0	0.023479	0.023461
LOC10699	784.1213	461.8002	986.7056	773.5562	810.5165	747.3046	444.2384	452.6355
LOC10699	0	0.033638	0	0.034024	0	0	0	0
LOC10699	54.14602	79.01615	48.41006	54.20823	67.67136	101.4921	98.75767	66.28264
LOC10699	0.872184	1.646765	1.409426	0.893788	0.317199	1.197466	1.488857	1.206272
LOC10699	0.821768	0.422489	0.41182	0.729002	0.870941	0.48161	0.473068	0.534911
LOC10699	1.136539	1.719506	1.646154	1.490805	1.288244	1.403679	1.045919	1.613806
LOC10699	0	0	0.016701	0	0	0	0	0
LOC10699	0.045095	0.106603	0.089067	0	0.030067	0.075672	0.030515	0.79278
LOC10699	0	0	0	0	0	0	0	0
LOC10699	0	0	0	0	0.016495	0	0	0
LOC10699	0.051672	0.069801	0.034019	0.141206	0.120584	0.121392	0.06993	0.034939
LOC10699	0	0.009192	0.013439	0.009297	0.013611	0.004567	0	0
LOC10699	0	0.016391	0.015977	0	0.016181	0	0	0.032818
LOC10699	3.745029	6.523325	6.942546	6.127006	6.96534	6.085902	6.001902	6.863671
LOC10699	0.06096	0.026468	0.0172	0.044621	0	0.035072	0.044196	0.03533
LOC10699	7.232813	2.986515	0.060023	2.460277	2.401118	4.497845	2.560203	1.417839
LOC10699	0	0.019927	0	0.080625	0.019672	0.019803	0.039928	0.059847
LOC10699	1.980503	0.118029	0.172573	4.447125	2.708979	0.263916	3.044877	0.147698
LOC10699	0.132232	0.133968	0.435282	0.135508	0.132249	0.266271	0.044739	0.223524
LOC10699	0	0	0	0	0	0	0	0
LOC10699	182.1556	152.8586	159.6731	156.132	171.4507	161.3026	156.8961	168.3664
LOC10699	0.0283	0	0	0.116006	0	0	0.028725	0.114813
LOC10699	0	0	0	0	0	0	0	0
LOC10699	0	0	0	0	0	0	0	0
LOC10699	0.03873	0.01308	0.025499	0	0.006456	0.006499	0.072071	0.019641
LOC10699	0	0	0.029625	0	0	0	0	0
LOC10699	0.010208	0.051708	0	0.00523	0.010209	0	0	0
LOC10699	0	0	0	0	0	0	0	0
LOC11410	0.162162	0.180719	0.304269	0.398829	0.486549	0.081635	0.246892	0.674329
LOC11410	0	0	0	0	0	0	0	0.014352
LOC11410	2.560864	33.19829	27.03489	13.19541	8.964184	1.07129	5.473126	5.706787
LOC11410	92.73518	114.5078	126.602	102.6924	83.64109	99.81964	101.7704	92.11722
LOC11410	6.476986	4.982952	7.649731	0.765121	4.246988	4.294242	5.844733	3.701085
LOC11410	0.141359	0	0.046533	0.096574	0.047126	0.332092	0.14348	0
LOC11410	206.8105	319.7547	308.5342	270.5977	401.9207	253.2808	266.354	258.1233
LOC11410	49.99715	39.17735	30.90162	20.15109	38.15518	42.47982	33.01492	21.33755
LOC11410	0.147311	0.137764	0.033571	0.290308	0.294661	0.205363	0.299042	0.298817
LOC11410	0	0	0	0	0	0	0	0
LOC11410	0.210301	0.910352	0.207681	0.156735	0.994279	0.115493	0.11643	1.861483
LOC11410	0.579556	1.416098	2.895342	2.934625	3.409596	5.217316	3.460293	3.388533
LOC11410	6.07664	6.58926	5.77321	6.477358	6.28091	6.500537	6.326114	7.126136
LOC11410	0	0	0	0	0	0	0	0
LOC11410	0.024563	1.007844	0	0.012586	0.012283	0.037096	0.024931	0.012456
LOC11410	0.016568	0.016786	0.005454	0	0.005523	0.022242	0.033633	0.016804
LOC11410	4.701305	4.763005	5.074607	5.732758	5.1393	5.779178	4.919788	4.176823
LOC11410	2.89643	2.78772	2.32641	2.057935	2.452627	2.673199	2.087312	3.211844
LOC11410	9.155153	11.69239	9.730652	9.501191	10.37847	11.26824	9.686229	9.049411
LOC11410	6.563623	7.59973	6.574437	5.332921	5.532912	6.325243	7.233113	6.60951
LOC11410	0.015939	0.121113	0.00787	0.106171	0	0.008024	0.016178	0.040415
LOC11410	8.846398	9.909722	10.56322	9.20762	7.444931	10.62598	7.696367	8.622761
LOC11410	1.608089	2.310687	2.013609	2.111064	1.534715	2.190513	1.984256	2.430482
LOC11410	0.330743	2.387474	3.388707	2.436103	0.413483	2.830525	1.720486	0.251589
LOC11410	3.058328	5.809623	6.84943	6.855809	6.854818	3.271673	4.379144	5.732912
LOC11410	0.066747	0.022541	0.076901	0	0.05563	0.078403	0.067748	0
LOC11410	2.341101	2.951605	3.647713	2.665657	1.196717	6.442725	2.323413	1.319127
LOC11410	0.938588	0.243822	0.023766	0.789199	1.010917	1.138846	0.512974	0.683451
LOC11410	6.722571	10.87108	8.170839	9.141294	7.887112	9.436846	7.020238	6.752711

LOC11410	0.387662	0.209466	0.535964	0.211874	0.827118	0.234186	0.393476	0.183484
LOC11410	1.839063	1.514472	0.86437	0.927191	1.367181	1.713002	1.617097	0.927634
LOC11410	0.185975	0.546404	0.6428	0.209639	0.111599	0.861327	0.830562	0.264071
LOC11410	0.646286	0.721924	0.654598	0.679277	0.812106	0.116793	0.790541	1.193322
LOC11410	14.00782	14.99648	16.97127	14.27339	12.52657	20.84868	11.93339	9.211873
LOC11410	38.01109	136.5228	7.645695	12.72206	18.75215	15.87797	9.179468	5.791988
LOC11410	0.036162	0.012212	0	0.04941	0.012055	0	0.036704	0.036676
LOC11410	0	0.164544	0.080195	0.527047	0.108289	0.109015	0.05495	0
LOC11410	0.198505	0.20111	0.065344	0.406843	0.595591	0.399722	0.402965	0.06711
LOC11410	3.28647	4.329701	3.471398	3.39255	2.793258	3.527092	4.239967	4.41992
LOC11410	0	0	0.019842	0	0	0	0	0
LOC11410	0.048592	0.04923	0	0.099592	0	0.048924	0	0
LOC11410	0	0.024031	0	0	0	0.023882	0.024075	0.024057
LOC11410	0	0	0	0.026706	0	0	0	0
LOC11410	0	0	0	0	0.011202	0	0	0
LOC11410	1.946028	2.060815	1.70034	2.650769	2.418833	1.74162	1.137986	1.754427
LOC11410	0.092145	0.046677	0.090997	0.13574	0.103677	0.098573	0.029227	0.058411
LOC11410	0.707253	0.70129	1.099672	0.724772	0.180599	0.727235	1.221893	0.412078
LOC11410	0	0	0	0	0	0	0	0
LOC11410	0	0	0	0	0	0	0	0
LOC11410	3.187832	2.002395	6.673995	2.156085	2.837537	4.814415	3.753353	2.974556
LOC11410	12.58316	21.44914	11.95389	9.560822	12.10628	10.04377	10.92654	7.691343
LOC11410	0.782802	0.712149	0.489071	0.515695	0.663071	0.957039	0.956696	0.745337
LOC11410	26.12322	29.25445	29.66738	26.57908	29.15915	34.05128	27.93552	29.04999
LOC11410	0.229419	0.193692	0.339841	0.235102	0.152966	0.038498	0.426911	0.349028
LOC11410	0	0	0.013162	0.013658	0.01333	0.026838	0	0.081107
LOC11410	0.954854	1.692925	2.593131	1.957013	3.22305	3.965682	0.666309	1.573727
LOC11410	0	4.982412	5.668627	9.058474	4.048674	11.0811	9.806723	10.64936
LOC11410	21.36577	19.46188	17.76199	18.01362	23.79678	20.24059	12.36117	14.45975
LOC11410	0	0	0	0	0	0	0	0
LOC11410	0.106558	0.053978	0.035077	0.418589	0.390762	0.125167	0.342494	0.198137
LOC11410	0.099429	0	0.049095	0	0	0	0.05046	0.050422
LOC11410	0	0	0	0.044957	0	0	0	0.222473
LOC11410	1.503707	2.137939	3.119686	2.317906	0.227481	2.162828	1.423657	3.03741
LOC11410	0.032851	0.066565	0.129767	0.06733	0.312126	0	0.100032	0.016659
LOC11410	0.020763	0	0	0	0	0	0	0.021059
LOC11410	7.67193	7.772617	10.43949	7.941958	0.144983	16.93068	7.866238	15.16645
LOC11410	0.597899	0.416451	0	0.229766	0	0	0.151717	0.189503
LOC11410	0.037721	0.038216	0	0	0	0	0.019144	0
LOC11410	0.184645	0.124712	0.364689	0.473048	0.184669	1.425286	0.718424	0.577428
LOC11410	3.297477	11.29927	0.088912	4.670848	0.022511	0.022662	0.034269	0.022829
LOC11410	0.149143	0.050367	0.147285	0.35662	0.348045	0.350378	0.45414	0.100844
LOC11410	0	0	0	0	0	0	0	0
LOC11410	0	0.015148	0	0	0	0.015054	0.030351	0.045493
LOC11410	1.941303	2.315324	0.849354	0.402914	1.277985	0.692757	2.743626	2.916023
LOC11410	0.373189	0.280064	0.218393	0.141642	0.317943	0.194827	0.196408	0.49065
LOC11410	0.426773	0.396342	0.316091	0	0.106707	0.322266	0.180489	0
LOC11410	4.199122	4.390488	3.408935	5.574168	1.614106	3.460484	7.189465	1.015467
LOC11410	2.721169	2.363041	1.023719	2.080364	2.030341	2.304876	2.49894	1.139009
LOC11410	12.04461	14.05818	15.42671	15.03677	15.08464	15.53283	14.23731	15.14443
LOC11410	29.5269	27.9338	21.33068	29.28104	31.66475	27.83232	29.81251	26.64663
LOC11410	25.4409	24.92832	24.54634	24.65836	28.24346	27.59153	27.00975	23.00667
LOC11410	0	0	0	0	0	0	0	0
LOC11410	0.049981	0.15191	0.098716	0.102437	0.299923	0	0.101461	0.101384
LOC11410	31.20776	29.85904	30.03986	30.62266	35.59849	35.96413	33.14917	32.10008
LOC11410	1.437448	1.809989	1.602049	1.725578	1.519784	1.571319	2.876333	2.228521
LOC11410	0.018585	0.031382	0.042825	1.542682	1.759617	0.024949	0.037728	0.025133
LOC11410	3.47331	3.780244	2.784058	4.078613	3.704114	4.257667	4.75977	3.606852

LOC11410	2.717395	2.648179	3.641941	3.712941	3.921319	3.543718	3.047105	3.281046
LOC11410	0	0	0	0	0	0	0	0
LOC11410	0.184625	0.448915	0.601671	0.681114	0.90478	0.873666	0.281092	0.374507
LOC11410	0.00741	0.007508	0.007318	0	0.007411	0.007461	0.007522	0.015032
LOC11410	1.251692	1.13179	1.017962	1.199439	1.396592	1.137542	1.20862	1.524443
LOC11410	0	0	0	0	0.012888	0	0	0
LOC11410	0.720695	0.371714	0.207044	0.684834	0.393157	0.514529	0.438903	0.770825
LOC11410	43.56835	236.0112	55.31377	324.4982	129.1538	99.55498	234.0441	131.6522
LOC11410	0	0	0	0	0	0.011263	0	0
LOC11410	0	0	0.005046	0	0.00511	0	0	0
LOC11410	0	0	0	0	0	0	0	0
LOC11410	0.076431	0.015487	0.060383	0.03133	0	0.092344	0.046547	0.031008
LOC11410	0.334532	0.621357	0.275303	0.914182	0.613388	0.561362	0.6791	0.56549
LOC11410	2.638978	3.662039	3.253672	2.261817	3.679051	2.286636	2.743498	3.601171
LOC11410	7.182514	19.26574	15.16158	18.45379	10.68254	4.392823	0.041778	0.041746
LOC11410	0.147622	0.717886	0.787227	0.544604	0.383867	0.029726	0	0
LOC11410	1.178809	1.929221	1.542212	2.281794	2.831523	1.917239	1.646454	1.532808
LOC11410	0.014743	0.014936	0.00728	0.015108	0	0	0.014964	0.014953
LOC11410	0	0.078117	0	0.237045	0.30846	0	0	0
LOC11410	2.729978	1.592434	1.307133	1.299895	2.620014	3.109558	3.274732	3.551947
LOC11410	0	0	0	0.203874	0	0.012519	0.025241	0
LOC11410	0	3.088991	0.043324	0.044957	2.983557	0.04417	0	0.044495
LOC11410	0	0	0	0	0.044222	0.044519	0	0
LOC11410	0.088814	0.077126	0.087708	0.182028	0.126894	0.127744	0.090146	0.102947
LOC11410	0	0.0296	0	0.05988	0.02922	0.029416	0.029654	0.014816
LOC11410	0	0	0	0	0	0	0	0.032295
LOC11410	1.980968	1.586151	2.587345	1.833582	1.374075	1.125928	1.945822	2.073981
LOC11410	0	0	0	0	0	0	0	0
LOC11410	0	0.010338	0	0	0	0	0	0
LOC11410	0.056773	0	0	0.058179	0.085171	0.057161	0	0.086372
LOC11410	6.563553	4.166647	3.901448	8.011383	5.283925	9.670561	1.108566	3.582902
LOC11410	0	0	0	0	0	0	0	0
LOC11410	0.390712	0.143942	0.21046	0.145596	0	0.071524	0.072104	0.07205
LOC11410	0.074489	0	0.01839	0.019083	0.018625	0	0	0
LOC11410	2.866484	2.468488	2.001756	3.441057	2.518736	3.360205	2.951045	3.98714
LOC11410	0	0	0	0	0	0	0	0
LOC11410	396.6269	236.4885	585.842	463.7232	319.755	485.8938	290.9729	214.0841
LOC11410	1531.965	1547.984	2566.699	1860.613	1304.817	2002.838	1045.247	844.5953
LOC11410	0	0	0	0	0	0	0	0
LOC11410	0.154636	0.26111	0	0.158467	0	0.20759	0.209274	0.470513
LOC11410	0.164836	0.0668	0.065113	0.270271	0.329715	0.165962	0.167309	0.234056
LOC11410	1.128909	3.391736	1.6146	0.957412	1.012256	1.215009	3.23997	2.803227
LOC11410	0	0	0	0.036538	0	0	0.03619	0
LOC11410	0	0	0	0	0	0.099577	0	0
LOC11410	0	0	0	0	0	0.049009	0	0
LOC11410	34.85288	22.69352	24.92102	24.84971	0.205527	1.200045	19.56508	0.250111
LOC11410	18.96647	19.29424	11.48535	16.57468	19.38411	16.93108	17.19683	14.95695
LOC11410	0.020997	0.202091	0.466549	0.677793	0.314998	0.031711	0.063936	0.063888
LOC11410	652.3577	292.8932	1104.101	613.5108	546.2763	628.3316	1320.866	268.8872
LOC11410	0.019964	0	0.019715	0	0	0	0.020263	0
LOC11410	9.858252	19.64933	16.01011	12.83411	14.83526	14.12489	13.0966	10.41811
LOC11410	0.046883	0.047498	0	0.048044	0	0.047203	0.095173	0
LOC11410	0.79108	1.297605	1.264837	1.389729	1.393988	1.441257	1.108831	0.534894
LOC11410	1.65463	1.878315	1.949004	2.28806	1.69472	2.227936	2.28648	2.2241
LOC11410	0.044199	0.167922	0.229155	0.056618	0.121564	0.18913	0.056078	0.201728
LOC11410	333.2323	361.8051	104.8454	91.82894	330.051	88.74605	94.89499	89.02711
LOC11410	0	0	0.047008	0	0.119017	0	0	0.024139
LOC11410	0	0	0	0	0	0	0	0.020571

LOC11410	0.033074	0.025131	0.008166	0.008473	0.041348	0.008325	0	0.008386
LOC11410	0	0	0	0	0	0.013548	0	0
LOC11410	0	0	0	0.175428	0.17121	0.043089	0	0.086812
LOC11410	1.074332	2.06229	0.837588	1.564498	1.017919	1.707901	2.066109	2.408646
LOC11410	0	0	0	0	0	0	0	0
LOC11410	0	0.022631	0	0	0	0	0.022673	0
LOC11410	0.10038	0.135596	0.066086	0.325742	0.150589	0.033688	0.118866	0.339362
LOC11410	7.326575	11.09239	8.576658	8.73128	10.49729	9.697364	11.15471	6.220226
LOC11410	0	0	0.095807	0	0.024257	0.02442	0	0.024599
LOC11410	0.254447	0.612242	0.408324	0.032594	0.12724	0.128093	0.032283	0.161293
LOC11410	0.291385	0.196806	0.543536	0.447904	0.145711	0.309674	0.115016	0.180604
LOC11410	10.30001	11.90973	8.446911	8.742416	15.94469	13.0306	9.74997	10.15141
LOC11410	0.049832	0.13463	0.13123	0.068089	0.033226	0.016724	0.10116	0.033694
LOC11410	10.04105	6.924624	9.335639	11.07154	12.10443	10.74279	14.62828	10.6545
LOC11410	3.22745	4.191711	6.139855	4.069933	3.913789	5.488042	5.277777	7.606097
LOC11410	1.429614	2.398873	3.375087	1.007206	0.960646	1.259458	2.788753	0.906229
LOC11410	0.027253	0	0	0	0	0.02744	0	0
LOC11410	0.716683	1.014963	1.385065	3.684023	1.206187	2.510008	1.693433	2.680878
LOC11410	4.288918	5.847253	5.333565	4.688167	3.992917	3.038746	8.115322	7.496992
LOC11410	0	0	0	0	0	0	0	0.030426
LOC11410	8.825181	8.992985	8.081845	9.464425	8.646717	9.04045	8.957559	8.144199
LOC11410	3.27649	2.957364	2.010034	2.4928	2.313699	4.278538	1.995382	2.366473
LOC11410	4.253209	5.690622	3.974193	4.172883	3.1474	6.586622	4.084701	5.126212
LOC11410	0.107651	0	0	0	0.215329	0	0.109265	0
LOC11410	0	0.042386	4.379498	0.042874	0.083685	3.159235	0.08493	0
LOC11410	0.07327	0.470134	0.24119	0.200227	0.170986	0.565575	0.173528	0.272481
LOC11410	0.642652	0.085669	0.350724	0.017331	0.490508	0.238384	0.46347	0.085763
LOC11410	0.056601	0.086016	0.027948	0.116006	0.198129	0.085481	0.05745	0.574067
LOC11410	0	0	0	0	0	0	0	0
LOC11410	0	0	0	0	0	0	0	0
LOC11410	0	0	0	0.061419	0	0	0	0
LOC11410	0	0	0	0	0	0.060343	0	0
LOC11410	0	0	0	0	0	0	0	0
LOC11410	4.593094	3.780866	2.834914	4.804926	3.612744	4.455871	3.375087	3.299756
LOC11410	0.115897	0.058709	0.057227	0	0	0.058344	0	0.117547
LOC11410	0	0	0	0	0.121973	0.061395	0.495146	0
LOC11410	0	0	0.070692	0.036678	0.035796	0	0.472272	0
LOC11410	0	0.064593	0	0	0	0	0	0
LOC11410	0.527232	0.046448	0.520663	0.046982	0.022926	0.02308	0.674743	0.534738
LOC11410	0	0	0	0	0	0	0	0
LOC11410	0.096765	0.049017	0.02389	0	0.072583	0.073069	0.024554	0.024536
LOC11410	0.140414	0	0.023111	0.23982	0	0.070687	0	0
LOC11410	0.377056	0.476036	0.435372	0.380451	0.516344	0.251141	0.376824	0.488326
LOC11410	39.322	19.98521	16.99708	44.41264	22.76659	22.06421	28.44215	24.04831
LOC11411	0	0.033879	0	0	0.033444	0	0.135766	0
LOC11411	0	0	0	0	0	0	0	0
LOC11411	0	0	0	0	0	0	0	0
LOC11411	30.8504	40.06646	37.1523	49.46854	34.48099	39.81761	36.34512	41.20226
LOC11411	13.7297	11.00268	11.05181	12.03397	14.6366	14.49022	10.7766	15.33558
LOC11411	0	0	0.005329	0	0	0	0	0
LOC11411	0	0	0	0	0	0	0	0
LOC11411	1.082753	1.014691	0.588094	0.582526	0.947531	0.490567	0.769296	1.098167
LOC11411	0	0.025478	0	0	0	0	0	0
LOC11411	0	0	0	0	0	0	0	0
LOC11411	0.242781	0.19326	0.051376	0.106626	0.13875	0.03492	0.123211	0.070353
LOC11411	0.316063	0.071158	0.173403	0.683773	0.421472	0.17679	0.21387	0.498653
LOC11411	0	0.064889	0	0.065635	0	0	0	0.06496
LOC11411	0.102996	0.104348	0.177997	0	0.077257	0.025925	0.104541	0.104462

LOC11411(	33.06939	41.75785	26.69383	26.325	33.2654	37.25214	30.25753	27.60987
LOC11411(	45.62976	71.00183	69.94189	49.93702	69.13038	30.55438	35.05697	61.29243
LOC11411(	0.746009	0.839778	0.191	1.35909	1.575112	0.890199	1.45831	0.616513
LOC11411(	0.041884	0	0	0.01073	0.020945	0	0.021256	0.02124
LOC11411(	0.015795	0.112018	0.109189	0.097119	0.094784	0.222644	0.14429	0.03204
LOC11411(	0.135332	0.11218	0.097197	0.050431	0.036914	0.099096	0.087412	0.212127
LOC11411(	0.01522	0	0	0	0	0	0.015448	0.061746
LOC11411(	0	0	0	0	0	0	0	0
LOC11411(	0.182936	0.139002	0.135492	0	0.274439	0.046046	0.04642	0.324695
LOC11411(	0	0	0	0.062218	0	0.030564	0.092437	0.123157
LOC11411(	4.551429	8.23114	4.704751	4.533399	6.636587	6.938025	4.403827	6.255628
LOC11411(	40.06597	45.10783	43.25213	43.97978	42.76675	45.71483	44.19178	41.00432
LOC11411(	0.456885	0.362255	0.19617	0	0.178804	0.100001	0.24195	0.161179
LOC11411(	1.277229	0.292192	0.935814	0.605175	0.673035	1.14768	0.738801	0.417875
LOC11411(	17.56737	35.82475	49.42363	52.70512	51.66377	33.55483	51.19927	55.57211
LOC11411(	34.22588	33.18532	34.80088	26.18834	32.81038	24.45362	31.23961	21.59927
LOC11411(	0.930586	0.609193	0.452426	1.496476	1.517767	0.691897	0.653916	0.522739
LOC11411(	0	0.068577	0	0	0.033849	0	0	0
LOC11411(	0.39507	0.41257	0.462174	0.435998	0.267466	0.373291	0.339304	0.246581
LOC11411(	0.209657	0.212408	0.207044	0	0.366947	0.316633	0	0.106321
LOC11411(	0.044408	0.073621	0.155485	0.016548	0.024226	0.036582	0.065562	0.024567
LOC11411(	0.024307	0	0	0.074726	0.012155	0.391564	0.148028	0.012326
LOC11411(	1.861535	1.637056	1.745023	1.897962	2.268153	2.197727	2.090872	1.562181
LOC11411(	0	0	0	0	0	0	0	0
LOC11411(	0.041343	0.195466	0.17692	0.141223	0.096479	0.138751	0.097914	0.265566
LOC11411(	11.95267	0.100913	3.983761	2.832521	1.195421	2.908295	9.149518	8.662767
LOC11411(	0.418443	0.282623	0.091829	0	0.232498	0.093623	0.094382	0.188622
LOC11411(	0.336206	0.042577	0	0.387601	0.252187	0.084626	0.426562	0.596737
LOC11411(	1.434673	0.78401	1.013224	0.801933	0.965268	0.656579	0.856065	0.758414
LOC11411(	0	0	0.03776	0	0.114725	0.076996	0.03881	0
LOC11411(	0	0	0.022862	0	0	0	0	0
LOC11411(	0.024552	0.024874	0	0	0	0	0	0
LOC11411(	0.304588	0.425347	0.065036	0.219337	0.18113	0.397841	0.342579	0.308924
LOC11411(	1.538234	2.845813	3.269296	3.084136	1.772542	1.885425	1.15401	2.06887
LOC11411(	2.326614	1.579289	3.745127	2.169663	2.489799	1.592906	6.30524	5.852142
LOC11411(	3.118908	2.571645	2.880043	3.984829	4.645208	2.909117	4.138697	3.519351
LOC11411(	2.523062	0.5794	3.488274	0.103422	2.99442	0.643542	3.687707	0.648275
LOC11411(	0.171713	0	2.543596	0.175966	0	0	0.174288	0
LOC11411(	0.225356	0.091325	0.178038	0.277125	0.270462	0.544548	0.411725	0.274276
LOC11411(	1.491126	1.807881	2.31745	1.803614	1.161273	2.510391	0.384576	0.458664
LOC11411(	7.826354	6.892337	4.185673	4.93253	3.480935	7.421913	6.090849	5.548108
LOC11411(	301.3717	217.8394	229.8226	195.3965	268.2099	218.2341	273.255	230.6658
LOC11411(	17.77153	52.36284	0.471143	25.05642	0.675962	1.200868	1.250965	0.524203
LOC11411(	51.73547	36.53758	56.33959	32.28238	49.05011	45.88785	43.06499	47.63658
LOC11411(	57.5413	28.21033	42.12551	24.27167	37.17942	28.253	40.00202	42.92284
LOC11411(	9.318217	14.1258	20.07423	13.79305	35.86252	14.2118	18.91598	21.24695
LOC11411(	95.73813	81.60735	45.82631	68.3432	67.75772	65.94535	73.55509	79.05619
LOC11411(	0	0	0.036985	0	0	0.012569	0	0.012662
LOC11411(	11.30457	11.5412	9.399892	0.491062	0.174274	8.399286	0.72957	0.154641
LOC11411(	0.051832	0.017504	0.068248	0.106231	0.051838	0.034791	0.070146	0.175232
LOC11411(	1.63096	1.358763	1.004985	1.153376	1.4357	0.726053	0.944002	1.298735
LOC11411(	0.481933	1.539889	0.915246	0.68382	0.704454	1.231725	1.053579	0.375995
LOC11411(	0.057296	0.116096	0.282911	0.117431	0.085955	0.028844	0.058156	0.029056
LOC11411(	168.1995	142.7872	129.6923	130.0422	143.0056	122.6845	142.224	139.8816
LOC11411(	0	0.014609	0.01424	0	0.014422	0.007259	0	0.021938
LOC11411(	0	0	0.02645	0.054895	1.31258	0	0	0.081496
LOC11411(	0.039201	0.039715	0.058068	0.040172	0.098014	0.059202	0	0
LOC11411(	2.342029	1.730952	2.862622	1.790195	2.995113	2.377357	2.279735	2.570072

LOC11411	0.439046	0.166803	0.379378	0.281201	0.384215	0.552556	0.167112	0.27831
LOC11411	0.05216	0.031707	0.030906	0.032071	0.125201	0.042013	0.063531	0.021161
LOC11411	0.031612	0.012811	0.006244	0.006479	0	0.019097	0	0.019237
LOC11411	5.73359	6.920857	4.278702	8.495219	7.770958	4.275041	4.177794	5.668733
LOC11411	0.007136	0	0.035238	0.007313	0.035687	0.021556	0.02173	0.028952
LOC11411	0.01785	0.072338	0.070511	0.018292	0.035705	0.053917	0.09059	0.018104
LOC11411	2.14995	2.783212	2.8702	3.141607	1.473304	2.084466	2.22261	2.382459
LOC11411	0	0	0	0	0	0	0	0.014762
LOC11411	0.161414	0.119924	0.053134	0.110275	0.150672	0.065007	0.14199	0.098227
LOC11411	0.124554	0.225338	0.19329	0.282631	0.142366	0.188108	0.325087	0.180468
LOC11411	0	0	0	0	0.021016	0.042313	0	0
LOC11411	0	0	0	0	0	0	0	0
LOC11411	0.291554	0	0.527855	0.298776	0.242993	0.048924	0.591854	1.034965
LOC11411	0	0	0	0	0	0	0	0
LOC11411	0.918682	5.429307	0.937476	0.564863	0	1.880747	1.087872	2.205164
LOC11411	0	0.019382	0	0	0	0	0	0.019404
LOC11411	0	0.033127	0.008073	0.008377	0	0.00823	0.008297	0.008291
LOC11411	0	0	0.030814	0	0	0	0.031671	0
LOC11411	4.988864	5.489374	4.872731	4.000344	4.9817	5.384534	4.722947	4.410571
LOC11411	0.157242	0.756704	0.038821	0.805687	1.218787	0.079158	0.837906	0.239221
LOC11411	8.181448	8.020357	8.079506	9.029037	8.248761	8.137291	9.279153	9.641708
LOC11411	0.302259	0.183735	0	0.371695	0	0	0.061359	0.183937
LOC11411	0	0	0	0	0.073088	0	0	0
LOC11411	0.503855	0.561515	0	0.671237	0.352744	0.304378	0	0.025551
LOC11411	0	0.010713	0	0	0	0.010646	0	0
LOC11411	0.157908	0.07999	0.038985	0.040455	0.19741	0.079493	0	0.080078
LOC11411	0.053929	0.464413	0.159771	0.027632	0.13484	0.081446	0.191583	0.054697
LOC11411	0.047845	0.032315	0.094497	0	0.01595	0.016057	0.032375	0
LOC11411	0.099606	0.151369	0.049182	0.204146	0.149428	0	0.05055	0.050512
LOC11411	17.54423	20.94357	16.9227	18.2018	18.19939	19.52634	15.81959	16.38702
LOC11411	0.460727	0.116693	0	0.118035	0.460787	0.115969	0.350728	0.233643
LOC11411	0	0	0	0	0	0	0	0
LOC11411	0.035295	0.178793	0	0	0	0.035536	0.035825	0.035798
LOC11411	0	0.085155	0	0	0.084062	0	0	0
LOC11411	0.065426	0.145826	0.193832	0.093865	0.143955	0.131746	0.265629	0.053086
LOC11411	0.714237	0.045226	0.044084	0.045746	0.044646	0.224724	0.135928	0.498028
LOC11411	7.416538	7.992463	7.277476	7.406611	8.102552	5.564732	8.151106	7.402338
LOC11411	0	0	0	0	0.046968	0.047283	0	0.04763
LOC11411	0.835997	11.6204	0.82558	0.651095	0.80266	0.538693	11.67586	23.6733
LOC11411	1.197618	1.255175	0.754478	1.206124	0.980935	1.039483	1.016478	1.267023
LOC11411	0.483498	0.163281	0.424421	0.578054	0.214916	0.081134	0.381695	0.790058
LOC11411	3.605747	2.025464	3.63133	3.182876	3.284867	3.486602	1.884271	3.946747
LOC11411	0	0	0	0	0	0	0	0
LOC11411	1.332044	1.398898	0.609597	1.348392	1.608407	2.044426	1.467441	0.807309
LOC11411	0.709664	0.373869	0.546641	0.319987	0.468439	0.342966	0.374561	0.690976
LOC11411	0.047845	0	0	0	0	0	0	0
LOC11411	1.052472	1.382837	1.429117	0.808906	2.203897	1.771622	1.352015	0.783912
LOC11411	0	0	0	0	0	0	0	0
LOC11411	0	0	0.026274	0.013632	0	0.026787	0.040507	0.013492
LOC11411	0	0	0	0.009599	0	0.009431	0	0
LOC11411	0	0	0	0	0.338281	0	0.085828	0.25729
LOC11411	8.283722	11.57162	10.79474	14.18883	10.32252	11.73933	5.419145	4.178197
LOC11411	0.13474	0.148918	0.157254	0.25105	0.196011	0.172659	0.149194	0.198775
LOC11411	0.041527	0.042072	0.082019	0	0.249194	0.250864	0.252899	0.042118
LOC11411	0.34602	0.038951	0.075935	0	0.192258	0.116128	0.273163	0.077988
LOC11411	0	0	0	0	0	0	0	0
LOC11411	1.017788	0.343715	0.558392	0.811221	1.187573	0.45544	1.20523	1.146974
LOC11411	0.041837	0.042386	0.041316	0	0	0	0	0

LOC11411	0	0	0	0	0	0	0	0
LOC11411	1.513763	0.179967	0.099152	0.221608	0.154486	0.163297	0.148943	0.156665
LOC11411	0	0.130375	0	0	0	0	0	0
LOC11411	0	0.215639	3.64335	0.618	0.035479	0.10715	0.720127	0.035979
LOC11411	0.028083	0.037935	0.009244	0.009593	0	0.009425	0.019003	0.009494
LOC11411	0	0	0	0	0	0	0	0.023539
LOC11411	0	0	0	0	0	0	0	0.016837
LOC11411	0.038874	0.078768	0.038389	0	0.194394	0	0	0.236563
LOC11411	0	0.030906	0.090377	0.015631	0.015255	0.030714	0.015482	0.06188
LOC11411	0	0	0	0	0	0	0	0
LOC11411	0.282529	0.324401	0.316209	0.115811	0.131864	0.208603	0.133824	0.210137
LOC11411	0.711007	0.422267	0.711833	0.62812	0.848415	0.70599	0.885915	1.118993
LOC11411	0.116379	0	0.172393	0.059631	0.349182	0.058587	0.295312	0.118036
LOC11411	4.301136	4.658107	3.891837	4.299113	4.767885	3.498548	5.118352	5.00705
LOC11411	0	0	0	0.016343	0	0	0.016187	0
LOC11411	0	0	0	0	0.051552	0	0	0
LOC11411	0	0	0	0	0	0.054297	0	0.054697
LOC11411	0.040447	0.020489	0	0	0	0	0	0
LOC11411	0	0	0	0	0	0	0.073314	0
LOC11411	1.794559	1.175243	1.458881	0.72138	1.715463	0.868471	1.851667	2.594404
LOC11411	0.545834	1.751159	1.497314	0.683656	1.152466	3.144722	0.153895	1.476279
LOC11411	7.981701	4.717097	8.801843	1.445029	7.690032	7.741566	5.670999	6.611183
LOC11411	0	0	0	0	0	0.02258	0.022764	0
LOC11411	0.522078	0.23508	0	0	0	0.087607	0.647666	0.588344
LOC11411	0	0	0	0	0	0	0	0.199913
LOC11411	0	0	0	0	0	0	0	0
LOC11411	0.876618	2.093432	1.669555	0.513333	1.377721	1.765214	0.699103	0.762083
LOC11411	29.79276	24.49893	20.97769	11.56882	20.37661	24.07774	12.47555	12.87266
LOC11411	93.48665	184.2452	37.78926	73.13786	134.659	147.0052	182.5675	189.3948
LOC11411	0	0.058588	0	0	0.057836	0.058224	0	0.351912
LOC11411	0.633239	0.17108	28.84942	12.41609	0.506657	10.03102	14.31162	9.762254
LOC11411	2.066816	2.068712	1.99188	0.71451	0.97128	2.131078	0.859347	2.172006
LOC11411	1.468284	4.648606	1.449989	3.056328	5.965675	1.201131	2.095746	6.096355
LOC11411	1.094548	0.871289	0.772079	2.002965	0.781921	1.259458	0.952257	1.189425
LOC11411	0.815635	0.043492	0.084787	0	0	0	0	0
LOC11411	0.613221	0.177505	0.043256	0.359092	0.131422	0.044101	0.444585	0.533101
LOC11411	5.099793	3.905282	1.66752	1.94013	4.861635	4.327516	2.250561	0
LOC11411	1.331664	1.52085	1.183564	2.022163	1.138115	1.608914	0.98301	0.613919
LOC11411	3.278183	1.913092	2.394872	1.355541	3.019769	2.653974	1.605303	3.373459
LOC11411	12.36369	11.1097	6.35024	6.525977	12.61384	7.131105	5.9908	6.931491
LOC11411	0	0	0	0.005938	0	0	0	0
LOC11411	0	0.135677	0	0.205855	0	0.067417	0.543713	0
LOC11411	0	0	0	0	0	0	0	0
LOC11411	0.406712	0.224754	0.365131	0.303117	0.25885	0.372264	0.487869	0.1125
LOC11411	0	0	0.03585	0	0	0.03655	0	0
LOC11411	0.012579	0.076467	0.062113	0.180474	0.805186	0.088657	0.051072	0.127585
LOC11411	0.293387	0	0.115893	0	0	0.177235	0	0
LOC11411	0	0	0	0.204666	0.05707	0	0	0
LOC11411	0	0	0	0	0	0.00843	0	0
LOC11411	13.23944	10.45236	17.25831	10.25092	13.73157	12.06602	13.3584	14.72097
LOC11411	0.147311	0.074622	0.072738	0.452881	0.294661	0	0.074761	0
LOC11411	0	0	0	0	0	0.015441	0	0
LOC11411	0	0	0	0	0.012701	0	0	0
LOC11411	0	0.040094	0.019541	0	0	0	0.070294	0
LOC11411	0.495676	0.418484	9.072061	0.524886	0.677511	0.582239	0.704355	0.703825
LOC11411	45.81815	16.05159	20.4516	9.893257	28.34318	42.99565	35.0865	11.64721
LOC11411	0.005637	0	0	0	0	0	0.011444	0
LOC11411	0	0	0	0	0	0	0	0

LOC11411	0	0	0	0	0	0	0.020292	0
LOC11411	0.032061	0.032482	0.063323	0	0	0.12912	0	0.032517
LOC11411	0	0	2.227735	0.299668	0	0	0.042401	0.04237
LOC11411	0.03332	0.067515	0.329053	0	0.533196	1.006442	1.35281	0.473127
LOC11411	0	0	0	0	0	0	0	0
LOC11411	0.552418	0.186556	0.207823	0.781759	1.446995	0.317824	0.480603	0.400201
LOC11411	0.189757	0.432557	0	0	0.379563	0.42987	0.096302	0.048115
LOC11411	0	0	0	0	0	0	0	0
LOC11411	0	0	0.353232	0.458186	0	0.180066	0.090764	0
LOC11411	0	0	0	0	0	0	0	0
LOC11411	0	0	0	0	0	0	0	0
LOC11411	0	0.015719	0.030643	0	0	0.031242	0.015748	0.031472
LOC11411	0.031698	0	0	0	0	0	0	0
LOC11411	0	0	0.046377	0	0	0	0	0
LOC11411	0	0	0	0	0	0.042504	0.085698	0
LOC11411	0	0	0	0	0	0	0.15524	0
LOC11411	0.848986	0.721398	0.676135	0.841951	0.766926	0.827213	0.277975	0.861072
LOC11411	0	0	0.04451	0	0	0.045379	0.274483	0.045713
LOC11411	0	0.05391	0	0	0	0	0	0
LOC11411	0.68418	0.126029	0	0.701127	0	0.751477	0	0
LOC11411	1.341691	0.717408	0.239231	0.019096	0.279555	0.150095	0.189141	0
LOC11411	0	0	0	0	0	0	0.053051	0.026506
LOC11411	0.841114	1.143678	0.448105	1.066094	1.095803	1.058575	1.213196	1.189833
LOC11411	0.263427	0.066721	0.260145	0	0.065865	0.265227	0.267379	0
LOC11411	0	0	0.053671	0	0	0	0	0
LOC11411	0.099961	1.04649	0.065811	0.307312	0.099974	0.16774	0.06764	0.033795
LOC11411	1.535456	2.000066	1.473	1.348705	2.018288	1.236756	2.181883	1.824282
LOC11411	0	0.058467	0	0	0	0	0	0
LOC11411	0	0	0	0	0	0	0.062911	0
LOC11411	0	0	0	0	0	0.025783	0	0
LOC11411	0.060583	0.122755	0	0.062083	1.030037	0.304982	0.491931	0
LOC11411	0	0	0	0	0	0	0.102931	0
LOC11411	0	0	0	0	0	0	0	0
LOC11411	0	0	0	0	0	0	0	0
LOC11411	0	0.134756	0	0.009736	0.009502	0.019131	0.144649	0.009636
LOC11411	0	0	0	0	0	0	0.061095	0
LOC11411	1.223655	0.894477	0.382408	0.555554	1.053409	0.093571	0.062887	0.628392
LOC11411	0.169247	0.450103	0	0.238479	0.063476	0.191703	0.128839	0.021457
LOC11411	0	0	0	0	0	0	0	0
LOC11411	1.813731	2.171631	1.709716	2.703502	1.896418	2.531668	2.34301	2.383054
LOC11411	0.036587	0	0.036131	0	0.109776	0	0.074272	0.18554
LOC11411	0.245212	0.42588	0.103781	0.466673	0.140139	0.246887	0.177778	0.603992
LOC11411	1.712923	1.145366	0.811958	2.352173	1.507567	1.621148	1.356121	2.050023
LOC11411	0	0	0.017707	0	0	0	0.018199	0
LOC11411	2.162896	2.364849	1.184287	1.755621	1.970418	1.5524	2.08666	2.258846
LOC11411	0.818696	1.244161	1.718051	0.891411	0.767627	23.23464	0.675168	21.95239
LOC11411	0	0	0.135492	0	0	0	0	0
LOC11411	1.178274	2.42936	2.000562	1.864148	0.84764	1.373637	2.077172	0.041931
LOC11411	0.068939	0.558749	4.28903	0.070646	0.068948	0.208229	0.209919	0.13984
LOC11411	0	0	0.007219	0	0	0	0	0
LOC11411	0	0	0	0	0	0	0	0
LOC11411	4.80622	7.518767	3.55975	5.866868	6.856818	8.468345	6.169632	6.881844
LOC11411	0.128444	0.34307	0.034594	0.071796	0.07007	0.2704	0.094815	0.071058
LOC11411	0.022382	0.022676	0.044207	0.091747	0.055963	0.067606	0.034077	0
LOC11411	0	0.071789	0	0	0	0.071343	0	0
LOC11411	0.331278	0.1483	0.28911	0.300009	0.508539	0.131865	0.453543	0.218787
LOC11411	0	0	0	0.036772	0.071776	0	0	0
LOC11411	0.108485	0	0	0	0.216998	0	0.330337	0

LOC11411	0.075851	0	0	0	0	0	0	0
LOC11411	2.554818	1.819531	1.673663	2.320001	1.530559	1.74456	2.182345	2.899051
LOC11411	0	0	0	0	0	0	0	0
LOC11411	0	0	0	0	0	0	0.049236	0
LOC11411	0.47359	0.527786	0.093538	0.048532	0.09473	0.143048	0.721041	0.096066
LOC11411	0.093878	0.448378	0.29137	0.590966	0.603582	0.243051	0.381146	0.476074
LOC11411	6.44418	11.34971	5.999739	8.169284	10.32607	6.576599	9.641951	9.812781
LOC11411	0.062569	0.08452	0.159622	0.096178	0.093866	0.125993	0.084677	0.111054
LOC11411	0.290344	0.411816	0.917524	0.178522	0.696916	1.052379	0.471519	0.824536
LOC11411	0	0	0.017136	0	0	0	0	0
LOC11411	4.355246	5.729852	3.709849	4.653497	4.252591	4.197961	4.546278	3.203027
LOC11411	0.023375	0.014209	0.050784	0.014372	0.023378	0.009414	0.00949	0.018966
LOC11411	2.91841	3.003274	2.042395	2.119394	2.711944	2.892074	3.382024	2.237447
LOC11411	1.48287	0.87636	0.732196	1.456284	1.668444	0.7465	2.320382	1.879974
LOC11411	0.361929	0.916697	1.548816	0.741788	0.482634	1.457606	0.673489	0.917703
LOC11411	0.046417	0	0.687573	0.047566	0	0.327135	0.565353	0.141232
LOC11411	8.073797	8.515338	6.501222	6.534171	0.662551	0.250122	0.336201	0.797877
LOC11411	0.920696	0.447734	0.727379	2.302145	0.515657	0.704509	0.299042	1.045859
LOC11411	0	0	0	0.008441	0	0	0	0
LOC11411	0.440081	0.089171	0	0.090196	0.264083	0.531705	0	0.178538
LOC11411	0	0	0.024723	0	0	0	0	0
LOC11411	0.321715	0.869165	0.035301	0.183158	0.393257	0.359903	0.362823	0.290039
LOC11411	5.951184	7.78783	10.2542	5.971543	9.857923	5.773388	7.204498	7.419131
LOC11411	0	0.041457	0	0	0	0	0.041534	0
LOC11411	0	0	0.021197	0	0	0.064832	0.021786	0.065309
LOC11411	6.146504	6.22717	6.584317	6.4411	7.154483	6.922722	5.956726	5.494376
LOC11411	3.153469	2.33138	1.683338	1.589589	1.670702	1.012571	3.062359	2.524111
LOC11411	0.051672	5.025643	2.704508	0.052952	0.103358	0.069367	0.06993	4.646833
LOC11411	1.969387	1.672771	0.687573	2.303567	0.835605	1.08155	1.090324	0.786863
LOC11411	2.799476	3.397787	2.106621	1.709817	1.713501	2.412723	2.932395	2.521324
LOC11411	0.152115	0.05137	0.183602	0.086602	0.101423	0.051051	0.223018	0.239992
LOC11411	0.539474	0.417953	0.156692	0.455277	0.253903	0.287555	0	0.289669
LOC11411	1.197318	1.460131	1.569234	1.643542	1.818385	1.406414	1.22278	1.116914
LOC11411	0.960413	0.667212	0.731658	0.506161	0.960537	0.386789	0.919115	0.974085
LOC11411	0	0.067757	0	0.068536	0	0.067337	0	0.067832
LOC11411	0	0	0	0	0	0	0	0
LOC11411	0	0	0	0	0	0	0	0
LOC11411	0.021285	0	0	0	0	0	0	0
LOC11411	2.692628	1.866503	0.944672	2.46887	0.850414	1.56954	2.733018	2.191955
LOC11411	10.7146	11.20968	13.8634	10.35258	10.40982	11.05199	11.3636	10.601
LOC11411	1.811596	1.223581	1.565396	1.933823	1.781633	1.535177	1.287139	1.913943
LOC11411	0	0	0	0	0	0.003675	0.003704	0
LOC11411	0.986068	3.68865	1.685391	0.660707	2.617211	2.78749	2.155697	3.538835
LOC11411	0.592363	0.450103	0.731228	0.086719	0.88866	2.683845	1.245444	1.609275
LOC11411	0	0	0	0	0	0	0	0
LOC11411	0	0.036184	0.011757	0	0.023814	0	0	0.036224
LOC11411	0.147133	2.373552	4.750172	0.104384	3.622196	2.769038	3.607128	1.434877
LOC11411	0	0	0	0	0	0	0	0.047313
LOC11411	0.710085	0.329727	0.584364	0.454796	0.739767	0.86388	0.93095	1.110298
LOC11411	0	0	0.012155	0	0	0	0.012493	0
LOC11411	0	0	0	0	0	0	0	0.053461
LOC11411	1.299273	1.237028	1.855061	1.491873	1.268129	1.812496	1.636536	1.428906
LOC11411	0	0.030491	0.089163	0	0	0	0.030547	0.030524
LOC11411	3.050101	12.63318	1.151684	26.1084	7.8954	8.038632	12.92974	19.47099
LOC11411	1.218813	0.970207	2.751145	2.408792	3.439238	1.051834	2.91601	2.604773
LOC11411	4.980664	5.9236	5.052262	4.687947	6.334924	5.014689	5.659821	6.094825
LOC11411	0.040001	0.052105	0.073362	0.035136	0.017145	0.086302	0.040601	0.115915
LOC11411	0.149675	0.212295	0.118248	0.092029	0.089816	0.120558	0.15192	0.060722

LOC11411:	0	0.03215	0	0	0	0.031951	0.257678	0
LOC11411:	0	0.007278	0	0	0.007185	0	0	0
LOC11411:	0.091468	0.602344	0.045164	0.468668	0.04574	1.657669	3.852855	0.46385
LOC11411:	1.815513	0.827703	0	1.395363	0.847349	1.370937	0.12285	1.258262
LOC11411:	0	0	0	0	0	0	0	0
LOC11411:	49.27087	140.9435	0.735987	32.92543	92.92279	1.417356	106.8279	48.79645
LOC11411:	0	0	0.039318	0	0	0.040086	0.040411	0
LOC11411:	0	0	0	0	0	0	0	0
LOC11411:	0.070124	0.122713	0.179422	0.166588	0.124327	0.089859	0.164999	0.064657
LOC11411:	0	0.069162	0.067416	0.069957	0	0	0.485032	0.553905
LOC11411:	0.06479	1.903561	0	0	0.129596	0.130465	0.131523	0.722833
LOC11411:	64.22947	73.06305	75.01257	68.55607	79.64998	80.22447	36.57864	34.82815
LOC11411:	5.795113	6.107125	6.318196	5.671912	7.042726	6.620939	5.506591	6.80858
LOC11411:	4.198375	2.238671	2.582197	2.226665	2.872943	3.262991	2.653999	2.652
LOC11411:	0	0.023281	0	0	0	0.023137	0	0
LOC11411:	0.12106	0	0	0	0	0.048755	0	0
LOC11411:	0.621981	0.360082	0.263242	0.182111	0.266598	0.089462	0.090187	0.450597
LOC11411:	0	0	0	0	0	0	0.018095	0
LOC11411:	135.5363	301.3877	303.7695	166.5295	180.8157	154.8752	305.0552	398.7918
LOC11411:	95.80906	213.9916	216.8267	217.141	216.9889	180.1918	176.5547	322.0902
LOC11411:	0	0.39384	2.495315	0.398368	0.388789	0.195697	0.394569	0
LOC11411:	703.6257	979.3823	1128.47	737.8065	554.4852	1063.965	620.7053	714.4616
LOC11411:	22.03299	71.43089	114.2917	56.96794	10.89684	23.25612	10.86227	6.728551
LOC11411:	140.4739	67.81482	309.5857	331.8778	104.3127	139.3068	149.2813	126.0704
LOC11411:	895.0211	804.7538	980.7523	554.2725	688.6684	1125.421	723.7587	443.826
LOC11411:	649.5284	642.2486	1098.58	732.587	682.4204	1190.815	943.3613	752.9878
LOC11411:	3.246327	2.782942	2.548261	2.900234	1.789873	2.68185	3.041559	2.152815
LOC11411:	0.028766	0	0	0	0	0.144812	0	0.058351
LOC11411:	0.145777	2.461501	0.527855	1.394287	0.048599	0.293546	0.098642	0.443556
LOC11411:	0	0	0	0	0	0	0.097625	0
LOC11411:	20.35979	141.0634	34.07962	358.9172	125.901	106.7817	210.0285	29.37735
LOC11411:	0	0.048225	0	0	0	0	0	0
LOC11411:	0	0.194756	0	0.039399	0	0	0.156093	0.272958
LOC11411:	0.103664	0.140032	0.102372	0.141642	0.034559	0.069581	0	0
LOC11411:	0.094345	0.031861	0	0.25782	0.188715	0.18998	0.03192	0.031896
LOC11411:	0	0.21294	0	0.107694	0	0.176348	0.142223	0.106587
LOC11411:	0	0.795825	0.408278	0.254202	0.330786	0.083251	0.083926	0.209657
LOC11411:	1.312654	2.144969	1.087218	4.643001	0.825808	2.53666	1.955536	3.199511
LOC11411:	0.719773	1.043061	0.953738	0.700256	1.011458	1.311782	1.146718	1.034965
LOC11411:	0	0.148076	0.433009	0	0.073088	0.147156	0.074175	0
LOC11411:	0	0	0	0	0	0.048295	0	0
LOC11411:	0	2.518144	0.16008	0.055372	3.080287	2.012884	0.054844	0
LOC11411:	3.947459	5.126718	4.250776	4.411032	3.82197	5.24286	4.219792	6.048073
LOC11411:	0	0.141253	0	0	0	0	0	0
LOC11411:	0	0.030071	0	0	1.66235	0.059767	0.030126	0.030104
LOC11411:	0.008706	0.194041	0.034389	0.080293	0.043535	0.043826	0.053018	0.070638
LOC11411:	0.021547	0	0	0	0.021549	0	0	0.043707
LOC11411:	0	0	0	0	0.007968	0	0	0
LOC11411:	0.864753	1.839814	0.711648	1.122486	1.758558	0.87066	0.585149	0.964769
LOC11411:	0	0	0	0.064167	0	0	0	0
LOC11411:	0	0	0	0	0	0	0	0
LOC11411:	0	0	0.214742	0	0.062137	0	0.063061	0
LOC11411:	0	0	0	0	0	0	0.001731	0
LOC11411:	0	0.024299	0	0	0	0	0.024344	0
LOC11411:	0.052316	0.021201	0.041331	0.021445	0.031394	0.052674	0.01062	0
LOC11411:	0.27712	0.018717	0.036489	0.113594	0.092385	0.223211	0.262525	0.168639
LOC11411:	1.901073	1.6434	1.142756	1.408183	1.095325	1.45635	1.65693	2.494002
LOC11411:	0.406622	0.228866	0.892346	0	0.610012	0.386656	1.215236	0.458234

LOC11411	0	0	0	0	3.737788	0.081801	0	0.082402
LOC11411	0.429188	0.248469	0	0.251325	0.06132	0.061731	0.248929	0.093278
LOC11411	0.071219	0	0.035166	0	0.106843	0	0	0.10835
LOC11411	0	0	0	0	0.338793	0	0.042979	0
LOC11411	0	0	0	0.190265	0	0	0	0.282464
LOC11411	0.025445	0	0	0	0	0	0.025826	0
LOC11411	0.102901	0.173753	0.372604	0.1406	0.102915	0.06907	0.034815	0.139155
LOC11411	0.060583	0.122755	0.53845	0.248333	0.454428	0.335481	0.245965	0.12289
LOC11411	0.186594	0.220551	0.107491	0.095608	0.279928	0.156558	0.126262	0.220793
LOC11411	0.406453	0.837773	0.761253	0.789953	0.350435	0.649122	0.739744	1.250931
LOC11411	0.237145	0.622204	0.294239	0.753982	0.930457	0.599973	0.623356	0.622887
LOC11411	0.814621	2.366934	1.684836	1.764106	2.628647	1.671324	1.123255	1.044464
LOC11411	3.619288	0.888918	0.693177	0.651874	0.680075	0.927567	0.957357	1.023378
LOC11411	109.3698	58.03692	85.81829	90.17417	60.49322	65.43647	83.07501	97.95686
LOC11411	335.0206	399.4086	321.1001	259.5929	217.1868	213.6333	266.7244	233.0902
LOC11411	3.176268	2.997922	3.270738	3.477506	3.746852	3.443964	6.530488	3.276552
LOC11411	3.217145	4.345823	2.118039	1.868207	3.003057	3.455065	3.265403	2.066531
LOC11411	2.906855	3.139894	2.60679	2.091772	2.661399	6.176225	3.026389	4.628297
LOC11411	3.345831	2.490156	2.351024	2.123162	2.895805	2.850429	3.474388	2.532044
LOC11411	0.178896	0.164768	0.032121	0.016666	0.097593	0.032749	0.066029	0.049485
LOC11411	0.047279	0	0	0	0	0	0	0.095904
LOC11411	2.395531	8.975604	2.821144	1.615417	18.12715	20.34864	9.879569	17.02838
LOC11411	197.3595	220.7582	220.4483	77.04692	235.7341	114.4763	144.8894	209.3889
LOC11411	0.643864	0.882543	0.29922	0.556313	0.555563	0.673685	0.679151	0.985948
LOC11411	0.748798	0.724142	0.436958	1.058007	0.975833	0.66253	0.702452	1.547683
LOC11411	0	0	0	0	0	0	0	0
LOC11411	0	0	0	0	0	0	0	0
LOC11411	9.077567	8.763523	9.419178	8.864264	10.9537	9.967441	10.28199	10.20753
LOC11411	0.407354	0.386074	0.947301	0.552104	0.341696	0.370446	0.386789	0.506446
LOC11411	0.144545	0.146442	0.155721	0.201989	0.302269	0.211684	0.346777	0.453136
LOC11411	80.08406	86.5089	70.34041	83.23254	85.49403	83.92125	84.79438	84.10609
LOC11411	204.2568	200.3895	140.734	184.0276	194.5605	171.4485	251.8859	210.6859
LOC11411	32.50301	42.82682	55.15068	56.99353	37.91136	66.01406	37.50523	37.79445
LOC11411	0.042152	0	0	0	0.042158	0	0.171139	0.042752
LOC11411	8.80856	8.641306	6.355228	7.467455	7.79051	7.983268	6.092704	8.282669
LOC11411	0.419591	0.225052	0.633731	0.505864	0.34559	0.745513	0.551145	0.175232
LOC11411	0	0	0	0	0	0	0	0
LOC11411	0.177427	0.029959	0.029203	0.045456	0.17745	0.208413	0.135067	0.119969
LOC11411	0.051333	0.130016	0.050693	0.11836	0.102679	0.15505	0.065128	0.026032
LOC11411	0.043529	0.0441	0	0	0	0.087653	0	0.088297
LOC11411	0	0	0	0.012742	0	0	0	0.025222
LOC11411	0	0	0	0	0	0	0	0
LOC11411	1.420024	2.897165	2.185701	2.930469	0.969659	0.828255	1.20276	2.503036
LOC11411	0.309915	0.686835	0.286925	0.337441	0.348699	0.253526	0.117961	0.392908
LOC11411	1.972805	3.216651	1.902562	1.642608	1.8035	3.289779	3.504194	2.751222
LOC11411	0.05643	0.228681	0.696583	1.214379	0.310404	0.59656	1.05961	2.575489
LOC11411	1.103021	1.257184	1.021198	0.777111	0.689477	0.971737	0.769702	1.887846
LOC11411	0	0.187378	0	0	0	0	0	0
LOC11411	0	0	0	0.078798	0.115355	0.116128	0.078047	0.233964
LOC11411	0.012582	0.031868	0.018638	0.025788	0.044043	0.006334	0.025542	0
LOC11411	0	0	0	0	0	0	0	0.025901
LOC11411	0.202575	0.342057	0.033342	0.069198	0	0	0.102807	0
LOC11411	0	0.103743	0	0.24485	0	0.103099	0.034645	0.103857
LOC11411	3.539565	6.819654	7.415228	4.067543	6.62987	8.136877	3.343457	7.076151
LOC11411	0	0	0	0	0	0.042039	0.014127	0.028232
LOC11411	0.090743	0.674182	2.449403	0.867913	0.988218	1.0659	0.358183	0.562435
LOC11411	7.743525	5.756032	6.522056	6.413287	7.730104	7.709314	8.254857	6.522861
LOC11411	0	0.013407	0	0	0.013235	0	0	0.013422

LOC11411	0	0.117176	0.032633	0.016932	0.115673	0.049906	0.117393	0.217851
LOC11411	0	0	0	0	0	0	0	0
LOC11411	2.767997	2.125618	4.35471	3.643775	3.556158	3.683757	3.728588	3.128461
LOC11411	0.040157	0	0	0.041151	0.160647	0.121293	0.163036	0
LOC11411	0	0	0	0	0	0	0	0
LOC11411	0.661161	0.64503	0.749653	0.677539	0.220416	0.51775	0.347967	0.596065
LOC11411	0	0	0	0	0	0	0	0
LOC11411	0	0	0.030643	0	0.031034	0	0.031496	0.031472
LOC11411	1.970893	0.100466	1.505656	1.003505	1.809985	0.599051	1.509779	1.722366
LOC11411	2.793273	1.515014	2.006159	2.631154	1.523801	3.636177	3.837506	1.888692
LOC11411	0	0	0	0	0.024794	0	0.050326	0
LOC11411	0	0	0	0.029692	0.028978	0	0.147045	0.029387
LOC11411	5.041819	6.92204	7.63136	5.553003	6.786129	7.732998	9.278361	7.598703
LOC11411	3.265617	3.021914	4.291438	7.220024	4.924775	2.588918	6.903244	4.681282
LOC11411	1.899026	0.464068	2.968541	1.672247	1.03076	1.114532	2.721763	1.277588
LOC11411	1.509143	3.247047	1.567161	2.184268	2.069505	3.618492	1.973944	3.487305
LOC11411	0.149239	0.206179	0.066991	0.152936	0.203535	0.122939	0.096395	0.041281
LOC11411	2.505634	2.122367	0.162257	2.390907	3.680882	1.546736	3.844012	2.60796
LOC11411	0.010366	0.010502	0.020474	0	0.010368	0	0.031566	0.010514
LOC11411	0.50928	1.741377	1.215424	0.761097	0.573014	0.833233	1.529218	0.968493
LOC11411	0	0	0	0	0	0	0	0
LOC11411	0.155954	0.213766	0.135892	0.319634	0.495447	0.064655	0.111736	0.139565
LOC11411	0.384055	0.483996	0.027751	0.710342	0.627683	0.23578	0.361293	0.636536
LOC11411	0.74257	0	0	0	0	0	0.057978	0
LOC11411	15.01187	22.9526	15.15064	17.52504	20.78835	19.10065	17.5254	26.54715
LOC11411	0.098727	0.150034	0.194994	0	0.04937	0.099402	0.150312	0.100133
LOC11411	0	0	0	0	0.035614	0	0	0
LOC11411	0	0	0	0	0	0	0	0
LOC11411	0.991283	0.679374	0.921347	0.776817	0.874774	0.528382	0.443891	0.561838
LOC11411	1.418254	1.004076	2.075562	1.453385	1.708961	1.032248	1.404841	1.733065
LOC11411	0	0	0.029157	0	0	0	0	0.029945
LOC11411	0	0.06564	0	0	0	0	0.065762	0
LOC11411	0.664547	0.910893	0.424643	0.681008	0.742825	0.511655	0.436451	1.030835
LOC11411	0.053211	0.010782	0.042039	0.065435	0.042575	0	0.021604	0
LOC11411	0	0	0	0	0.006289	0	0.006383	0
LOC11411	0.274943	0	0	0	0	0	0	0
LOC11411	0	0.05677	0.027668	0	0	0	0.056875	0.028416
LOC11411	0.761767	0.222175	0.159574	1.182782	0.496367	0.825075	0.09372	0.28095
LOC11411	0.014427	0.029233	0.014248	0.014785	0.028859	0	0	0.058531
LOC11411	0.420763	0.443337	0.265933	0.293206	0.134662	0.33891	0.034166	0.529174
LOC11411	0	0	0.016844	0	0	0	0	0.103794
LOC11411	19.88704	24.04875	33.64129	25.90342	24.1421	27.27024	23.78744	20.57762
LOC11411	0	0.01275	0.024856	0.03869	0.050347	0.025342	0.012774	0.038293
LOC11411	0	0.253749	0.123671	0.256666	0.125247	0.18913	0.12711	0.190521
LOC11411	0	0	0	0	0	0	0	0
LOC11411	0.106288	0	0.139951	0.036307	0.035434	0	0	0
LOC11411	0	0.028356	0	0	0.055986	0	0.056818	0.028388
LOC11411	0.167199	0.423484	0.280697	0.411218	0.300998	0.20201	0.186678	0.254369
LOC11411	156.4037	175.5689	257.0505	155.3957	719.9614	490.949	162.5413	429.3832
LOC11411	0.285895	1.274449	0.056467	0.410168	0.857797	1.727091	0	0.927889
LOC11411	0.011295	0.03433	0	0	0	0	0.011464	0.011456
LOC11411	0.271055	0.505287	0.230203	0.316657	0.645195	0.218326	0.390672	0.186941
LOC11411	0.123907	0.069741	0.115565	0.105813	0.103269	0.083169	0.090831	0.132652
LOC11411	0	0	0	0	0	0	0	0
LOC11411	0	0	0	0	0	0	0.014789	0
LOC11411	18.24805	8.529276	4.87516	17.0288	8.684375	15.66562	16.4638	14.84203
LOC11411	57.74528	43.3546	47.69988	53.98694	41.10558	42.39763	39.8781	54.75721
LOC11411	1.829845	1.967362	1.032597	1.664693	1.904779	0.939972	1.933101	1.874832

LOC11411	1263.265	1553.825	1578.36	1360.174	1403.314	1589.83	1332.684	1387.98
LOC11411	139.6895	184.7327	204.8226	173.9859	180.0049	212.7463	173.836	148.0731
LOC11411	65.46226	72.15087	58.93576	69.24682	67.46608	72.15398	76.15843	72.31802
LOC11411	0	0	0.033382	0	0	0.034034	0	0.034285
LOC11411	0	0	0	0	0.064351	0	0	0.065259
LOC11411	1.294297	3.879212	1.171655	1.879005	4.31488	1.846113	6.021176	8.149814
LOC11411	0	0.017123	0	0.01732	0.016904	0	0	0
LOC11411	0.009582	0	0.009463	0.009819	0	0	0.009726	0
LOC11411	0	0.00995	0.019397	0.090576	0.058932	0	0.069776	0.039842
LOC11411	0	0.06188	0.407142	0.046943	0.565048	0.415096	0.030997	0.015487
LOC11411	0.029095	0.014738	0.014366	0.044723	0.014549	0.014647	0.059062	0.014754
LOC11411	0	0	0	0	0	0	0	0
LOC11411	0.020209	0.020474	0	0	0.14148	0.162775	0.020512	0.020496
LOC11411	0.068769	0.023224	0.022638	0.070473	0	0.02308	0.046534	0
LOC11411	0	0	0.046377	0	0	0	0	0
LOC11411	0.04228	0.085669	0.020876	0.043327	0	0	0	0
LOC11411	0	0.015156	0.014773	0.03066	0	0.030123	0.030368	0
LOC11411	0.096514	0.019556	0	0.079124	0.038611	0.058304	0.039185	0.391553
LOC11411	0	0	0	0	0	0	0	0
LOC11411	0	0	0	0	0	0	0	0
LOC11411	0	0	0.01004	0	0	0	0.010319	0
LOC11411	0	0	0	0.024963	0	0	0.012362	0
LOC11411	0.169301	0.085762	0.083596	0.173495	0.114898	0.133931	0.184115	0.091988
LOC11411	3.890019	2.63379	1.761491	2.858524	3.150374	2.808485	1.964555	2.232517
LOC11411	0.113489	0.18684	0.028019	0.305287	0.156067	0.128547	0.316775	0.287761
LOC11411	0.020903	0	0.144498	0.042842	0	0.021046	0.06365	0.021201
LOC11411	0	0	0	0	0	0	0	0.024409
LOC11411	0.266057	0.700826	0.735676	0.545294	0.372528	0.375024	0.108019	0.323813
LOC11411	1.924472	1.7035	1.924984	2.028053	1.99417	1.992552	2.164783	2.857374
LOC11411	0.230363	0.216716	0.211244	0.033724	0.24685	0.066268	0.467638	0.417219
LOC11411	0	0	0.036561	0	0	0	0	0
LOC11411	0	0.061289	0	0	0	0	0	0
LOC11411	0	0.04906	0.047821	0	0	0.146265	0.049151	0
LOC11411	0	0.063049	0.061457	0	0.046681	0	0.047375	0.094678
LOC11411	0	0	0	0	0	0	0	0.014483
LOC11411	0	0	0	0.113369	0	0.111385	0	0.112204
LOC11411	0	0	0	0.015673	0.015297	0	0	0
LOC11411	1.328404	0.907658	1.00677	1.361309	2.039209	1.337479	1.066121	1.472646
LOC11411	12.43565	16.08441	11.92679	12.74368	10.32256	18.11338	8.94829	14.10293
LOC11411	2.239631	3.372021	3.551047	3.863431	4.286959	3.645472	3.795023	4.233849
LOC11411	5.474465	7.356023	6.116933	7.598615	6.786129	6.443446	5.856585	8.92815
LOC11411	0.089997	0.054707	0.177752	0.387352	0	0	0.073078	0.766739
LOC11411	0.018912	0	0	0.0969	0	0	0.076781	0.191808
LOC11411	6.300878	7.055526	6.92102	6.049146	7.208252	7.501411	6.417831	6.435421
LOC11411	3.59993	4.045902	3.920869	3.677239	4.758078	4.370405	3.571686	4.202963
LOC11411	0	0	0	0	0	0	0	0
LOC11411	5.475073	4.129103	3.661143	3.220487	5.058345	4.894486	3.912468	1.792902
LOC11411	1.099574	1.114005	1.233947	1.434123	1.149704	0.95612	0.862416	0.70969
LOC11411	3.98706	3.615251	1.319023	3.370803	0.019938	1.665934	0	1.900595
LOC11411	0	0	0	0.018982	0	0.05595	0.037603	0.037575
LOC11411	0.067607	0	0.066764	0.069281	0.135231	0.136137	0	0.068569
LOC11411	0.086187	1.309769	6.043016	0.463689	0.40944	0.021694	1.355934	0.043707
LOC11411	0.021236	0.021515	0.062914	0.021762	0.063717	0.064143	0.043109	0.021538
LOC11411	19.45645	19.49806	16.87593	4.10779	7.408475	12.74489	17.91623	6.276657
LOC11411	0	0	0	0	0	0	0	0
LOC11411	0	0	0	0	0.011029	0	0	0
LOC11411	3.437266	5.168289	3.448317	5.339523	3.164876	3.872741	4.734834	2.988169
LOC11411	0	0	0	0	0	0	0	0

LOC11411	27.41008	29.13874	36.82847	26.50655	25.09698	27.1309	29.85884	28.07437
LOC11411	0	0	0	0.02536	0	0	0.025118	0.0251
LOC11411	0.70112	0.710322	0.725355	1.037815	0.567647	0.571451	0.994033	1.467352
LOC11411	0	0	0	0	0	0	0	0
LOC11411	0.70404	1.004414	0.425674	0.647859	0.876571	0.419523	0.597931	0.714062
LOC11411	0.045944	0.102404	0.199635	0.160079	0.0919	0.05551	0.093267	0.25163
LOC11411	0	0	0.046769	0.048532	0.047365	0	0.144208	0
LOC11411	0.014718	0.049705	0.048449	0.085469	0.053974	0.024698	0.014939	0.029856
LOC11411	0	0	0	0	0	0.082762	0	0.041685
LOC11411	0.014157	0.186462	2.488616	0.188605	0.070796	0	2.256051	0.344614
LOC11411	0	0	0	0	0	0	0	0
LOC11411	0	0.019871	0	0	0	0	0	0
LOC11411	0	0	0.078822	0.054529	0.133046	0	0.027005	0.161907
LOC11411	1.807108	1.373118	1.110182	1.916471	1.439569	1.502107	1.738238	1.715617
LOC11411	0.011419	0.046277	0	0.175535	0.045684	0.114975	0.011591	0.046328
LOC11411	0.144274	0.526203	0.48442	1.123643	1.1832	0.522935	0.937204	1.638872
LOC11411	4.051607	3.015757	4.215469	3.44232	5.313253	5.171952	5.895812	4.539082
LOC11411	4.87805	5.227611	5.480981	4.654513	4.770265	5.118743	4.687155	4.870531
LOC11411	91.9644	125.9907	138.269	108.2017	107.1588	129.7798	106.3446	90.66164
LOC11411	0.318922	0.123088	0.194968	0.10894	0.227831	0.137614	0.138731	0.138626
LOC11411	0.375762	0.853987	0.260759	0.145702	0.507852	0.327203	0.340166	0.69012
LOC11411	1.860472	2.605582	2.080461	1.598143	2.189074	2.286384	2.638177	1.970205
LOC11411	0.827343	1.249394	0.755371	0.719861	0.983572	1.351652	0.712998	0.918283
LOC11411	15.90451	8.478803	14.30031	17.72192	7.050291	9.05548	8.318269	7.537157
LOC11411	1.166215	1.083061	0.335908	0	1.214965	0.782788	1.183708	1.182817
LOC11411	0	0	0	0	0	0	0	0
LOC11411	1.729308	2.159877	1.599331	1.547109	2.571419	2.13725	2.331043	2.607688
LOC11411	1.477434	1.510683	1.891328	3.658907	2.791069	3.402029	1.360744	1.900832
LOC11411	0	0	0.085442	0	0.302859	0.043555	0	0
LOC11411	0.968517	1.536266	0.908144	1.022584	1.242602	1.378977	2.005809	1.984454
LOC11411	0.203805	0.385428	0.107341	0.306318	0.665848	0.177837	0.330979	0.316949
LOC11411	0	0.060205	0	0.060897	0	0	0	0
LOC11411	14.33282	13.45006	9.352648	13.95129	14.88438	13.83478	13.00291	11.36362
LOC11411	0	0	0	0.017263	0.008424	0.00848	0	0
LOC11411	0	0	0	0.195119	0	0	0	0
LOC11411	0	0	0	0	0	0	0	0
LOC11411	0	0	0	0	0	0	0	0
LOC11411	0	0	0	0	0	0	0	0.00553
LOC11411	0.010059	0	0.004967	0.005154	0.01006	0.020255	0	0.005101
LOC11411	0	0	0	0.038243	0	0	0	0.03785
LOC11411	0	0	0	0	0	0	0	0
LOC11411	0	0	0.036274	0.075282	0.073472	0.036982	0.149129	0
LOC11411	0.98372	0.854254	0.693902	1.488128	0.679323	1.179095	1.283754	0.641394
LOC11411	0	0	0	0	0.574211	0	0	0
LOC11411	0.105859	0.150148	0.041816	0.043393	0.105873	0.063949	0.064468	0.107366
LOC11411	0	0	0.063541	0	0	0	0	0.032629
LOC11411	0.031413	0	0.031022	0.048287	0.047126	0.07907	0.015942	0.047791
LOC11411	1.928331	1.379039	1.092174	1.394892	1.106098	1.056407	1.669425	1.294268
LOC11411	3.921706	5.083487	4.329094	5.252029	6.114354	3.623964	4.907505	5.568548
LOC11411	0.398726	0.490522	0.309381	0.26997	0.327568	0.315425	0.455296	0.368295
LOC11411	0.85016	1.119713	0.055971	2.458774	0.018895	0.028532	0.028764	0.019161
LOC11411	0	0.027665	0	0	0	0.027493	0	0.027695
LOC11411	0	0	0	0	0	0	0	0
LOC11411	2.306457	1.894643	0.40014	1.820602	1.995031	0.5021	4.175933	1.264481
LOC11411	0	0.61496	0.039962	0.110583	0.512639	0.067905	0.50657	0.601954
LOC11411	0	1.211345	1.475944	0.250623	1.250163	1.121743	0.937773	1.708769
LOC11411	0	0.051464	0	0	0.025402	0	0.051559	0.1288
LOC11411	8.759967	25.0168	9.826363	18.76715	11.84427	1.106318	17.22509	29.81163

LOC11411	1.533464	1.861507	0.545714	1.585605	0.400686	0.083456	1.247977	3.264715
LOC11411	0.224993	2.32505	2.755154	2.674571	2.970295	2.219997	1.50723	4.153171
LOC11411	0.439365	0.189593	0.136595	0.275152	0.187161	0.221183	0.256011	0.56115
LOC11411	0.1336	0.236868	0.115443	0.239591	0.684788	0.319467	0.440712	0.237128
LOC11411	0	0.068994	0	0	0.068109	0	0	0
LOC11411	16.07335	0.338473	0.146634	16.24325	17.07782	13.52957	18.42441	30.68422
LOC11411	2.26269	3.57989	0.091829	0.031764	0.062	0.343283	0	0.062874
LOC11411	1.075325	0.311268	0.121363	0.314846	0.460913	0.154667	0.28066	0.405092
LOC11411	0	0.113653	0	0.11496	3.870745	0	0	0.113778
LOC11411	0	0.052241	0	0.010568	0.082514	0	0.020935	0.01046
LOC11411	0.686851	2.5747	0.118701	1.231762	2.644717	0.899006	1.516309	2.176964
LOC11411	0	0.036054	0.087859	0	0.035592	0.017915	0	0.036094
LOC11411	0	0.030231	0	0.030578	0.149215	0.030043	0.030287	0.121056
LOC11411	0	0.072461	0.094175	0.024431	0.047688	0	0.048397	0.072541
LOC11411	2.458882	2.782909	2.843889	2.8376	2.747214	2.375314	2.596945	2.662391
LOC11411	1.469598	1.015149	2.17693	1.848273	0.868511	0.672563	0.678019	1.490519
LOC11411	0.05419	0.082352	0	0	0.027099	0.02728	0	0.027481
LOC11411	0	0.06291	0.007665	0.015908	0	0.054704	0.094539	0.062979
LOC11411	0.974101	4.80284	1.41088	2.761769	3.507216	2.092278	2.93318	0
LOC11411	64.84536	64.79385	67.92463	66.36668	57.43597	40.98774	57.23857	82.74641
LOC11411	0	0.063722	0	0	0	0.063327	0.06384	0
LOC11411	0.19785	0.106905	0.169333	0.283851	0.250642	0.146081	0.214205	0.267555
LOC11411	0.042961	0	0.021213	0.022013	0.085933	0	0	0.021786
LOC11411	0.070383	0.023769	0	0.024042	0	0	0.023813	0
LOC11411	0	0.604759	0.130997	0	0	0	0	0
LOC11411	0.415039	0.262803	0.07685	0.451902	0.233489	0.679045	0.52658	0.736657
LOC11411	0.120513	1.343042	0.119011	1.790724	0.572511	1.304366	0	0.061114
LOC11411	1.092852	0.492087	1.019278	0.933269	1.032272	0.519595	0.400561	1.10841
LOC11411	1.706837	2.047387	1.802319	1.957344	2.933774	1.993754	1.931182	2.589208
LOC11411	5.985635	0.427056	1.248814	1.46868	1.011787	1.27321	0	0.598534
LOC11411	0.06676	0.625635	0.08241	0.393379	0.100153	0.033608	0.084702	0.220059
LOC11411	0.270909	0.375583	0.253453	0.263008	0.171122	0.301471	0.347334	0.549531
LOC11411	0	0	0	0	0	0	0	0.015666
LOC11411	2.265241	2.333868	0.189578	0.07869	0.076798	0.618499	0.03897	0.233643
LOC11411	13.22919	13.13828	11.11785	12.78565	11.44903	11.05669	12.8924	14.29506
LOC11411	0.244661	0.52053	0.265773	0.376081	0.367038	0.270965	0.223497	0.074443
LOC11411	0.074938	0.037961	0.037002	0	0	0	0.038031	0.019001
LOC11411	0	0.097781	0.047656	0	0.289581	1.603366	0	0.244721
LOC11411	0	0	0	0	0	0	0	0
LOC11411	0	0	0	0	0	0	0	0
LOC11411	0	0	0	0	0	0	0	0
LOC11411	0.696514	0.716512	0.444448	0.065886	0.085736	0.571807	0.511188	0.869452
LOC11411	7.894796	1.514102	1.074816	15.63136	3.005609	9.829602	1.64881	4.151873
LOC11411	7.483675	9.037792	9.399051	8.937783	8.435424	9.460236	9.088188	8.812267
LOC11411	0	0	0	0	0	0.035181	0.035467	0
LOC11411	3.274498	0.552912	1.92482	1.677804	2.33923	2.74739	4.668889	3.953706
LOC11411	2.170588	3.946761	2.222515	2.844832	3.347708	5.475044	3.838114	5.886084
LOC11411	48.85288	56.03783	32.60152	49.64273	55.47288	50.58016	53.33192	59.63477
LOC11411	0	0	0	0	0.192721	0	0	0
LOC11411	0	0.044868	0	0	0	0	0	0
LOC11411	0.483763	0.490112	0.204744	0.849851	0	0.278325	0.420874	0.49065
LOC11411	0.244447	0.454034	0.160934	0.292252	0.122239	0.328156	0.165409	0.165284
LOC11411	1.610398	1.585574	1.075154	0.023243	0.499061	0.159856	0.460438	0.805159
LOC11411	0.128982	0.490032	0.796095	0.198266	0.451496	0.292193	0.261834	0.621388
LOC11411	0	0.168789	0	0	0	0	0	0.168974
LOC11411	0.084779	0.294485	0.251168	0.273048	0.205918	0.158522	0.33191	0.135121
LOC11411	0	0	0	0	0	0	0	0
LOC11411	0	0.078441	0	0.039671	0.038718	0	0	0

LOC11411	0.024487	0.198471	0	0	0.024491	0.073964	0	0
LOC11411	0.159482	0.610396	0.113747	0	0.115197	0.035683	0.125903	0.332492
LOC11411	0.306227	0.558443	0.725788	0.910056	0.765667	0.431647	1.243282	1.180229
LOC11411	5.618588	5.061514	5.006897	4.800667	5.36726	5.477859	4.890321	5.412891
LOC11411	0	0.379874	0.130688	0	0	0	0.067161	0.246071
LOC11411	0.004896	0.00496	0.004835	0.005017	0.014689	0.009858	0.014908	0
LOC11411	8.394266	10.44183	5.371119	3.410338	5.514106	6.601259	6.226285	5.289616
LOC11411	0	0	0.017549	0	0	0.017892	0.018037	0
LOC11411	8.217569	4.694784	12.32531	5.002019	4.572772	8.460333	9.406954	9.775865
LOC11411	0.374323	0.51714	0.554489	0.523084	0.187186	0.359749	0.569907	0.345138
LOC11411	0.19315	0.231264	0.052021	0.053982	0.035123	0.053037	0.124757	0.053427
LOC11411	33.62913	40.40841	76.23121	57.19885	43.90993	89.81168	48.5766	50.38102
LOC11411	0.077252	0.036123	0.023474	0.030448	0.01783	0.005983	0.042221	0.054244
LOC11411	1.463405	1.258351	1.214429	2.217976	1.377501	1.981046	1.734996	1.359512
LOC11411	537.9767	433.1539	488.5346	156.0838	327.5369	298.485	566.078	608.6501
LOC11411	703.7276	62.40358	420.6702	334.1898	261.3136	325.3683	278.6305	271.9996
LOC11411	613.9583	745.9919	841.0261	257.7778	464.9291	327.9101	573.9804	680.1692
LOC11411	196.0815	149.4629	207.4197	3.010718	28.41254	28.78782	137.8915	163.4349
LOC11411	13.41147	11.51983	7.86984	4.332247	9.671118	8.2682	7.990032	16.26373
LOC11411	524.3021	254.4637	478.0954	71.27158	79.56736	128.6904	227.4155	311.8049
LOC11411	913.7444	307.8911	870.1352	404.515	385.4573	285.9044	344.0806	375.0431
LOC11411	1262.186	1271.547	1265.167	393.9175	613.5085	425.8558	998.357	1331.737
LOC11411	71.10953	74.84963	76.39698	76.47444	77.15777	153.8477	83.24414	71.72554
LOC11411	8.242764	8.305057	4.070029	1.670823	1.857126	8.344674	4.780803	7.349543
LOC11411	433.3481	540.4486	501.0721	398.2482	404.4433	451.4139	566.0514	544.8866
LOC11411	893.3135	1171.417	1350.452	940.4072	1103.74	1294.884	1193.416	1210.7
LOC11411	6.006008	4.667006	6.391846	8.186454	1611.218	848.4641	6.806323	791.068
LOC11411	0	0.04026	0.032703	0.013574	0.072863	0.04001	0.053779	0.040304
LOC11411	2.798916	3.502861	5.690674	3.036967	3.704927	5.470308	3.927127	3.924169
LOC11411	1.729202	1.043683	1.198993	1.489264	1.398259	2.43557	0.76554	1.091472
LOC11411	0.140649	0.035624	0.034724	0.036033	0	0	0	0
LOC11411	0.089208	0.022595	0	0.274255	0.08922	0.134727	0.226367	0.180957
LOC11411	0.347578	0.129736	0.957478	0.562401	0.109776	0.110511	0.445631	0.166986
LOC11411	3.701583	9.276718	7.503314	9.716102	8.638144	8.369113	14.96251	4.017736
LOC11411	0	0	0	0.138563	0	0.045379	0.091494	0.365702
LOC11411	0	0	0	0	0	0	0.056144	0
LOC11411	0.308846	0.391124	0.228748	0.263747	0.205924	0.414608	0.208986	0.36545
LOC11411	0.164116	0.113366	0.110503	0.091735	0.19398	0.150215	0.620879	0.090792
LOC11411	0.044008	0.044586	0.08692	0	0.044014	0	0	0.089269
LOC11411	0	0	0	0.034391	0	0	0	0
LOC11411	0.138802	0.247041	0.229688	0.157617	1.095549	0.17752	0.129461	1.221341
LOC11411	244.0711	184.2638	270.6472	194.3354	176.5266	188.3877	220.3925	223.0048
LOC11411	0	0.051093	0.049803	0	0.050437	0.101551	0	0
LOC11411	0	0	0	0	0	0	0.033344	0
LOC11411	0	0	0	0	0	0	0	0
LOC11411	0	0	0	0	0.055986	0	0	0.056775
LOC11411	0	0.061378	0	0	0	0	0	0
LOC11411	0.54444	0.488905	0.574315	0.92565	0.680638	0.436036	0.791232	0.752987
LOC11411	0.051262	0	0	0.210128	0	0	0	0.051992
LOC11411	0.26584	0.064126	0.137515	0.220535	0.265874	0.178437	0.231281	0.205428
LOC11411	0	0	0	0	0.052128	0	0	0
LOC11411	0.01995	0	0.078804	0.020444	0.019952	0.040172	0.080995	0.060701
LOC11411	0	0	0	0	0.070689	0	0.03587	0.071686
LOC11411	0.073656	0.012437	0.024246	0.08806	0.012278	0.02472	0.0623	0.099606
LOC11411	0	0	0	0	0	0	0	0
LOC11411	2.891869	321.1294	124.2969	134.3454	154.4251	285.5473	160.4602	309.0862
LOC11411	6.771743	9.323125	6.540622	7.68999	7.855388	7.117227	6.787134	7.880064
LOC11411	1.382918	1.401067	1.176554	1.338158	1.26618	1.187017	1.41376	1.465671

LOC11411	0	0.099964	0	0	0	0	0	0
LOC11411	0	0	0	0	0	0	0	0
LOC11411	0	0	0.095974	0	0.097197	0.017791	0.026902	0.035843
LOC11411	2.399071	2.430557	1.015362	1.34632	2.056613	3.278125	2.551012	3.533968
LOC11411	0	0.013426	0	0	0	0	0	0
LOC11411	0.150318	0.253818	0.405749	0.318352	0.240539	0.524661	0.549261	0.243932
LOC11411	0	0	0	0	0	0	0	0
LOC11411	12.77402	13.05979	8.182023	12.32876	10.23948	9.581767	12.00412	12.14289
LOC11411	1.979816	2.281105	1.533449	2.466453	2.018897	1.016213	0.157609	1.456784
LOC11411	0.027961	0.113313	0	0	0.027965	0.225218	0	0.028359
LOC11411	0	0.026789	0	0.013549	0	0	0.026839	0
LOC11411	0	0.076847	0	0	0	0	0	0
LOC11411	0	0	0	0	0	0	0.073982	0
LOC11411	0.059806	0	0.354364	0.061287	0.239255	0.060214	0.546327	0.485258
LOC11411	0.03717	0.150632	0.256949	0.076182	0.07435	0.261969	0.113183	0.150797
LOC11411	0.241598	0.257007	0.143153	0.198066	0.169141	0.04865	0.183917	0.171526
LOC11411	4.198869	4.053787	3.434319	3.543545	3.685601	3.630722	2.717557	3.60732
LOC11411	0.310252	0.044903	0	0.090839	0.022164	0.156186	0	0.022476
LOC11411	0.095461	0.038686	0.075417	0.156521	0.152757	0.096113	0.13565	0.058092
LOC11411	0.03561	0	0.035166	0.036492	0	0	0	0
LOC11411	2.203229	1.65344	1.934023	2.926782	2.856406	1.807488	2.898878	3.807084
LOC11411	2.68517	2.295346	2.108479	2.7899	2.844037	2.694126	1.911598	2.411339
LOC11411	4.007838	2.296477	4.736503	3.130833	1.182794	3.307556	3.701173	3.365198
LOC11411	1.197735	1.405051	0.684785	1.550404	1.323983	0.909727	0.298593	1.150849
LOC11411	0	0	0	0	0	0	0	0
LOC11411	13.9666	11.01538	10.69773	12.98535	12.01747	12.95122	9.623844	10.03013
LOC11411	0	0	0	0	0	0	0	0
LOC11411	0.114624	0.749557	0.123487	0.245606	1.26103	0.094424	0.211534	0.116256
LOC11411	12.60118	9.79588	26.37207	31.0168	16.20188	25.32574	11.83094	17.06943
LOC11411	1.090788	2.798029	1.26055	1.189157	0.719549	1.565576	1.813842	1.271087
LOC11411	1.344731	1.584162	1.111793	2.083084	2.032995	1.731755	1.777547	2.442286
LOC11411	0.032135	0	0	0	0	0	0	0
LOC11411	2.978891	2.074865	2.880487	3.370667	0.186205	5.373641	2.015716	0.944155
LOC11411	0	0	0.08692	0.018039	0.070422	0.088618	0.035735	0.035708
LOC11411	0	0	0.132568	0	0	0	0.034064	0
LOC11411	4.664861	7.213494	5.172475	6.541615	4.174362	3.872741	7.309919	4.648263
LOC11411	0	0	0	0	0	0	0	0.069748
LOC11411	0	0.11948	0	0	0	0	0	0
LOC11411	1.158002	0.024962	1.824851	0.454476	0.566755	0.347293	1.850586	1.849193
LOC11411	0	0	0	0	0	0	0	0
LOC11411	14.5338	13.68856	12.79659	11.9391	11.69672	12.75965	13.03332	10.79625
LOC11411	8.430471	7.459238	9.463234	8.034546	8.881244	10.75155	5.932804	8.550484
LOC11411	56.66208	60.65571	62.31083	31.13161	8.027154	47.93237	85.08431	88.09232
LOC11411	0	0	0	0	0	0	0.011708	0
LOC11411	0	0	0	0	0	0	0	0
LOC11411	1.114872	0.118895	7.185349	0.661441	0.23474	0.295392	0.178673	0.595128
LOC11411	1.474839	0.365248	0.744414	1.544957	0.917796	0.890949	2.494936	5.517966
LOC11411	1.291182	0.885819	0.271083	0.083349	0.111849	0.184252	0.134151	0.752741
LOC11411	0.730757	0.188025	0.469647	0.416032	0.023202	0.011679	0.423839	0.058822
LOC11411	0.776208	0.400907	3.471961	0.608274	0.532761	1.792879	4.464492	0.385911
LOC11411	66.78861	48.70567	50.99124	47.12358	50.07344	43.2406	43.42566	38.78867
LOC11411	0.770123	1.145963	0.689228	1.405761	0.553598	0.436154	1.367931	0.317316
LOC11411	2.009478	2.399396	2.48055	1.69153	2.655726	2.095464	2.476682	1.601352
LOC11411	1.716669	2.949075	6.191453	0.764866	1.791538	1.578101	1.212117	1.286905
LOC11411	0.085333	0.108066	0.042135	0.196755	0.277368	0.021479	0.043306	0.129821
LOC11411	2.387441	3.154437	3.020454	4.385802	3.642143	1.395726	3.338951	6.349272
LOC11411	0.2252	0.097781	0.031771	0.131873	0.064351	0.291521	0.16327	0.326294
LOC11411	0.398348	0.987698	0.517611	0.655292	0.691956	0.686039	0.627764	1.403432

LOC11411	0	0	0	0	0	0.020071	0	0
LOC11411	0.239503	0.474263	0.279522	0.234279	0.500843	0.142491	0.132597	0.088332
LOC11411	0.055061	0.185944	0.018125	0.056425	0.055068	0.036958	0	0
LOC11411	18.01646	4.983395	4.286074	11.9395	11.30515	12.04123	8.908405	14.41879
LOC11411	0.11534	0.116854	0.094919	0.177295	0.057677	0.038709	0.019512	0.116982
LOC11411	0	0	0	0.095291	0	0	0.062921	0.723051
LOC11411	0	0	0	0	0	0.035581	0.03587	0
LOC11411	0	0	0	0.060768	0	0.597042	0.060189	0
LOC11411	0.021382	0	0	0	0	0	0	0
LOC11411	0	0	0	0	0	0	0.157828	0
LOC11411	0	0	0.027019	0	0	0	0	0
LOC11411	0.275755	6.914514	1.429677	11.37408	6.825826	1.527015	9.37637	7.271703
LOC11411	1.960594	28.90445	11.61699	82.44477	40.7721	20.01215	70.74802	64.38639
LOC11411	0.660334	0.790637	1.259751	0.46138	0.795505	0.377753	0.33512	0.48708
LOC11411	0	0.045516	0	0	0.089864	0	0.091201	0
LOC11411	0	0.026929	0	0	0.026584	0	0	0
LOC11411	0.288209	0.166852	0.113847	0.210963	0.296481	0.157525	0.150445	0.116925
LOC11411	0.026505	0.013426	0	0	0	0.013343	0	0
LOC11411	0	0.063866	0.031127	0.0646	0	0	0.063984	0.031968
LOC11411	1.416171	1.648633	1.31166	1.379138	1.372368	1.602969	1.785607	1.891318
LOC11411	0.01904	0	0	0	0	0	0	0
LOC11411	0.213309	0.216108	0.962976	0.338298	0.284449	0.414192	0.371157	0.42754
LOC11411	25.46834	19.70158	20.05362	18.67743	25.11147	23.38629	23.45418	22.21904
LOC11411	2.527417	1.830419	2.846914	1.416418	0	1.491025	0.090187	0.040053
LOC11411	10.72178	5.671299	4.153375	6.085539	11.8488	10.12406	9.49973	12.61671
LOC11411	7.812237	2.875508	2.691888	3.830088	6.042619	6.648984	6.103942	7.353417
LOC11411	3.756122	2.209597	0.757818	4.014717	4.160543	4.879718	7.829898	5.038493
LOC11411	0	0.05137	0.050073	0	0.152135	0	0.051466	0.102854
LOC11411	0.179265	0.217941	0.271447	0.073482	0.155383	0.180489	0.206214	0.242422
LOC11411	1.883465	2.662582	1.730229	2.019892	3.373152	1.719929	2.178468	1.777002
LOC11411	0.237196	2.08268	0.07808	5.509626	24.43438	11.30399	49.83613	98.23396
LOC11411	0.06712	0.090668	0.243041	0.160493	0.044753	0.135158	0	0.068076
LOC11411	0	0	0	0	0	0	0	0
LOC11411	0	0	0	0	0	0	0	0.514488
LOC11411	0	0	0	0.019373	0	0	0	0.019174
LOC11411	0.079741	0	0	0	0	0.040143	0	0
LOC11411	0.174387	0	0.086107	0	0	0	0	0.088435
LOC11411	0.088016	0	0	0	0	0	0	0
LOC11411	0.02638	0	0.026051	0	0	0.02656	0.026776	0.026756
LOC11411	3.409242	4.32352	2.825255	3.83573	8.41691	8.017244	7.501525	3.868839
LOC11411	0	0	0.090328	0	0	0	0	0
LOC11411	0	0	0	0	0.049283	0.29768	1.875594	0
LOC11411	0	0	0	0	0	0.046967	0.142045	0.047313
LOC11411	0.070793	0.091283	0.088978	0.039571	0.077239	0.058318	0.052258	0.052219
LOC11411	0	0	0.020429	0.0106	0	0	0.010499	0
LOC11411	0.033202	0	0	0	0	0	0	0
LOC11411	1.546503	2.388726	2.453588	2.598049	2.231309	2.297313	2.264486	2.571341
LOC11411	1.307905	0.041844	0.054384	1.410844	0.357999	0.499013	0.489088	0.335122
LOC11411	0	0	0	0	0.016603	0	0	0
LOC11411	0	0	0	0	0	0	0	0
LOC11411	0.312922	0.093935	0.160234	0.201906	0.220233	0.256716	0.129399	0.340886
LOC11411	0	0	0	0	0	0	0	0.03564
LOC11411	0.66241	0.48206	0.469887	0.659697	0.886438	0.76087	0.662877	0.955716
LOC11411	2.623984	2.423855	2.642098	2.978969	2.727238	3.056327	3.081123	1.51331
LOC11411	1.506842	1.544165	0.724079	1.496837	0.76218	2.028661	2.607674	2.067002
LOC11411	0.328448	0.34305	0.25079	0.274125	0.189644	0.204551	0.230269	0.326255
LOC11411	0	2.534276	0.333821	8.036633	5.679695	1.089097	12.48898	7.131187
LOC11411	0	0.77689	0.054091	0.729691	0.438243	0	0	0.055553

LOC11411	0	2.29354	0.135492	18.20774	0	0.06907	0	0
LOC11411	13.88657	11.99395	10.7097	12.11411	11.25242	10.3795	12.17403	12.05969
LOC11411	0	0	0	0	0	0	0	0
LOC11411	6.46118	4.895375	9.722275	7.934055	7.478669	8.17371	6.134083	5.338565
LOC11411	0.217392	0.49555	0.161012	0.556941	0.869679	0.218877	1.434241	0.440973
LOC11411	1.681956	6.276109	1.660998	2.03921	2.238949	1.001757	1.803365	1.441605
LOC11411	0.039091	0.059406	0.09651	0.120178	0.078192	0.019679	0.039677	0.515417
LOC11411	0	0	0	0	0	0	0	0
LOC11411	0.249458	0.227459	0.394159	0	0.174643	0.075349	0	0.025301
LOC11411	4411.638	4220.061	6249.274	7686.69	4948.033	6069.555	4618.447	4337.407
LOC11411	4.830451	4.079309	4.03439	4.26017	4.60227	2.698261	4.471665	5.098102
LOC11411	0	0.016839	0	0	0.008311	0	0	0.008429
LOC11411	0	0	0	0	0.008737	0	0	0
LOC11411	5.345919	2.793309	2.504948	3.158521	3.175457	3.07401	3.364069	3.414519
LOC11411	3.922472	2.990566	2.048411	3.351965	5.691644	4.565087	4.696595	2.562302
LOC11411	0.04634	0.046948	0.297455	0	0.023173	0.163297	0.188139	0
LOC11411	0.682258	0.875909	0.788323	0.761431	0.663009	0.709168	0.703707	0.823641
LOC11411	0.433117	0.372567	0.330878	0.52759	0.506731	0.18924	0.174187	0.663068
LOC11411	11.03886	11.6762	10.29741	11.45692	11.5578	12.18781	13.14613	13.74056
LOC11411	0	0	0.026835	0.041771	0	0.01368	0	0
LOC11411	0.333741	0.237599	0.409752	0.517634	0.189445	0.535818	0.302126	0.548906
LOC11411	0.043619	0.044192	0.028717	0.0745	0.218126	0.102474	0.014758	0.029494
LOC11411	0.49809	0.150503	0.00863	0.394015	0.227228	0.211155	0.274954	0.008863
LOC11411	0	0.100003	0.075816	0.14611	0.010969	0.04417	0.05566	0
LOC11411	0	0.019913	0.038821	0.080569	0	0	0	0
LOC11411	3.317665	3.582338	2.636868	3.16126	3.805621	3.091271	3.190197	3.409169
LOC11411	0.382976	0.334113	0.451744	0.337954	0.383026	0.396303	0.410316	0.172635
LOC11411	0.776279	1.398163	1.022142	1.524723	0.927342	1.693428	1.510186	1.552789
LOC11411	0.770943	0.489818	0.580681	0.562401	1.489812	0.973552	0.716193	0.662643
LOC11411	1.705135	1.146057	0.624271	1.176279	2.129614	1.616289	1.359242	1.974057
LOC11411	142.578	154.9385	77.63577	86.94653	96.79009	103.0041	164.2201	157.6892
LOC11411	0.032736	0	0	0	0	0	0	0
LOC11411	1.547368	1.383244	1.123594	3.684414	2.548936	0	2.032514	4.615871
LOC11411	0	0	0	0	0	0	0	0
LOC11411	2.318629	2.516849	2.371515	1.75376	1.656378	2.390051	1.596956	2.295645
LOC11411	0.027603	0	0	0	0.027606	0.027791	0	0
LOC11411	0	0	0.047821	0	0.024215	0	0	0
LOC11411	0	0	0.026526	0	0	0	0	0
LOC11411	9.073314	8.838158	7.579102	7.829005	9.791354	9.839368	33.6259	22.60725
LOC11411	0	0.022979	0	0	0	0.022837	0	0
LOC11411	0	0	0	0	0	0	0	0
LOC11411	0	0.033879	0	0	0.033444	0	0	0.033916
LOC11411	0	0	0	0.173413	0.033849	0.034075	0	0.240282
LOC11411	0	0	0	0	0	0	0	0.030232
LOC11411	0.084687	0.042899	0.041816	0.043393	0.465841	0.042633	0	0.300625
LOC11411	1.161931	1.698056	1.472395	1.654352	1.110661	1.397631	1.513338	1.188901
LOC11411	0.026207	0.026551	0.051761	0.053712	0.104842	0.184703	0.106401	0
LOC11411	0.130182	0.131891	0.085707	0.177876	0.173599	0.043691	0.26427	0.088024
LOC11411	3.817926	2.052055	1.92058	2.498121	2.644211	2.282944	2.419723	2.245193
LOC11411	1.193109	2.635509	1.854284	1.182575	1.330195	2.560062	1.151448	0.733991
LOC11411	0.025057	0.025386	0.024745	0.487884	0	0.100914	0.3052	0.076242
LOC11411	0.098208	0.024874	0.024246	0.07548	0.171886	0.049439	0.099681	0.024901
LOC11411	0.010724	0.032594	0.02118	0.032968	0.02145	0.097174	0.010885	0.010876
LOC11411	69.32416	48.76315	46.92159	38.11665	40.47522	50.26433	40.70076	39.73012
LOC11411	9.318274	10.85549	9.756106	11.13279	9.30803	10.58061	11.3636	12.13295
LOC11411	0.061684	2.093537	1.812237	0.173833	0.200499	0.093158	0.156523	1.829945
LOC11411	0.163202	0.124008	0.050365	0.177697	0.387655	0.071889	0.269182	0.051727
LOC11411	5.364383	4.336086	6.278568	5.166407	4.835192	6.584589	4.991111	6.549049

LOC11411	1.000846	0.393355	0.230053	0.610077	0.940571	0.434346	0.814438	0.857579
LOC11411	0.050386	0.07657	0	0.07745	0	0.025365	0	0.127757
LOC11411	0	0	0	0.012851	0.087791	0.025251	0.038184	0.025437
LOC11411	0	0	0	0	0	0	0	0
LOC11411	44.3735	27.89193	32.11652	30.54402	113.5057	79.4271	49.46066	24.84587
LOC11411	0.107485	0.413804	0.191063	0.242325	0.558996	0.432878	0.567307	0.610486
LOC11411	25.15958	36.82955	23.77241	34.23898	22.84869	24.0684	34.70482	35.329
LOC11411	4.739142	4.45516	2.875542	3.821284	9.850963	6.371995	5.036415	3.119021
LOC11411	0	0	0	0	0	0	0	0
LOC11411	7.32561	5.86256	6.585876	5.341163	6.382519	5.826148	4.873685	6.493353
LOC11411	79.09409	104.0183	82.92124	88.74692	94.12161	98.92969	79.07729	84.42809
LOC11411	2.525547	5.862498	8.304458	0.170644	6.675522	0.2934	5.098691	3.532618
LOC11411	1.072166	2.115303	1.727526	1.503516	2.031734	1.874904	1.030972	2.575489
LOC11411	2.573059	3.244052	2.089265	2.255924	2.20168	2.734561	3.859446	2.812664
LOC11411	0	0	0.08082	0	0	0	0.083067	0
LOC11411	0.08774	0.088892	0	0	0.175503	0.029447	0.059371	0
LOC11411	0	0	0	0	0	0	0	0
LOC11411	0.425189	0.371353	0.535723	0.916517	0.483898	0.752854	0.654791	0.624557
LOC11411	0.308785	1.094932	0.3485	0.678072	0.286766	0.088827	0.671608	0.425031
LOC11411	1.084188	1.387474	1.098854	1.257233	0.485094	1.637392	1.100451	0.925997
LOC11411	0.370717	9.733852	10.49482	1.266334	6.828262	1.337479	2.1636	2.224636
LOC11411	3.539806	2.780048	2.303368	2.643286	5.13201	7.956811	2.005341	0.667944
LOC11411	49.43907	141.6397	102.6286	139.484	120.851	164.1443	99.50303	89.67968
LOC11411	0.044999	19.10188	33.50623	1.460254	19.62195	0.468163	15.52904	2.342814
LOC11411	0	0.030199	0	0	0	0	0.030255	0
LOC11411	0.16261	0.123558	0.682479	0.333275	0.569207	0.184185	0.041262	0.164924
LOC11411	130.5646	237.6619	195.9263	262.051	218.9933	338.424	168.8845	157.6741
LOC11411	0	0	0.052951	0.109895	0.241317	0.161956	0	0
LOC11411	26.66254	55.63249	43.2331	64.34895	50.10604	60.394	33.18784	33.6905
LOC11411	0.092557	0.018754	0	0.05691	0.037027	0.018638	0	0.112649
LOC11411	0	0	0	0	0.070689	0	0	0
LOC11411	0.87603	0.621269	0.216279	0.852843	0.963758	0.220504	1.378215	0.621951
LOC11411	0.054033	0	0	0.055372	0	0	0	0
LOC11411	0.09207	0.093278	0.151537	0.314501	0.214857	0.0309	0.186901	0.217887
LOC11411	0.060321	0	0	0.123631	0.120659	0.121467	0.244905	0.244721
LOC11411	9.35834	8.988255	13.02887	8.739647	11.47761	10.43077	8.801562	9.868903
LOC11411	0.028914	0.058588	0.114217	0.088892	0.057836	0.14556	0.088044	0.410565
LOC11411	2.040131	2.284475	2.792318	2.43911	2.434155	2.666685	2.27054	1.869516
LOC11411	0	0	0.011127	0	0	0	0	0.022856
LOC11411	9.254695	8.826193	7.20429	5.617096	8.276012	7.584973	9.08443	8.164459
LOC11411	2.467588	1.971809	1.61312	2.065707	2.572184	2.846031	2.469326	2.373467
LOC11411	0	0.021829	0	0.02208	0	0	0.04374	0
LOC11411	0.020165	0	0.019914	0.061994	0.080671	0	0.040935	0.020452
LOC11411	897.4079	727.3397	981.277	326.3837	672.4688	542.2352	455.8472	542.2728
LOC11411	0.024769	0.602262	0.269066	0.203062	0.024772	0.049877	0.35197	0.050244
LOC11411	0.432619	1.646935	0.906243	1.101622	1.324249	0.831552	1.131038	0.731297
LOC11411	0.010185	0.020638	0	0	0.010187	0.010255	0	0.020661
LOC11411	1.184309	1.119862	0.623761	0.890006	16.85884	1.152653	1.242143	0.76074
LOC11411	0.689693	1.387059	0.574359	0.996871	0.880244	1.243709	1.144092	1.14845
LOC11411	0.070275	0.071198	0.0347	0.040009	0.054666	0.027516	0.091143	0.067316
LOC11411	0	0	0	0	0.03652	0	0	0
LOC11411	0	0	0	0	0	0	0.006961	0
LOC11411	0	0	0	0	0.084827	0	0	0
LOC11411	0.378834	0.203191	0.462141	0.182691	0.334309	0.358986	0.113093	0.316422
LOC11411	0.706797	0	0.628191	0.144861	0.141378	0.28465	0.21522	1.003602
LOC11411	16.8987	7.150189	9.934965	6.58102	6.926953	5.561045	7.519376	7.647093
LOC11411	0.036492	0.184853	0.216222	1.084474	0	0.330669	0.074078	0
LOC11411	0.010442	0	0.020623	0	0.015665	0.005257	0.005299	0

LOC11411	0	0.079653	0	0	0.039316	0.039579	0	0
LOC44299	0.00921	0.004665	0	0.004719	0.023028	0.004636	0.009348	0
LOC44301	43.81683	26.5274	44.98814	38.90918	54.17994	38.78565	23.57486	26.3206
LOC44302	0	0	0.011638	0.012077	0	0	0	0
LOC44309	0	0	0	0	0	0	0	0
LOC44309	0.104828	0.053102	0	0	0	0	0.0532	0
LOC44316	8.57639	11.50698	7.897261	27.96986	16.05383	5.017623	6.999319	21.39356
LOC44324	47.1467	38.5569	45.3889	46.03346	42.40144	42.24981	42.73902	40.90909
LOC44329	0.782306	0.48435	0.987158	0.578994	0.521605	0.568858	0.749927	0.617122
LOC44331	0	0	0	0	0	0	0	0
LOC44332	1.197735	2.107577	0.684785	0.323001	2.269685	0.507754	0.703827	1.598401
LOC44332	0	0	0	0.1292	0	0.063469	0	0
LOC44334	0	0	0.020596	0.021373	0	0	0	0
LOC44342	0.045353	0.216612	0.223939	0.444844	0.414708	0.032616	0.08549	0.676834
LOC44344	0.222779	2.051845	2.040031	3.777286	2.531909	0.856422	1.171717	2.834653
LOC44344	0.569133	1.050241	1.947074	1.93716	1.016441	0.573021	0.123787	3.236641
LOC44345	7.626172	7.129448	6.281198	6.558789	7.300734	7.469883	6.399297	7.153419
LOC49444	0.584267	0.387576	0.439609	0.36352	0.4313	0.378166	0.62833	0.670185
LOC49444	0	0	0	0	0	0	0.037778	0
LOC55433	1.396677	1.195869	1.476922	1.75426	2.076744	1.791995	1.4239	1.679814
LOC65433	0.174279	0.706264	0.103264	1.750238	1.220109	0.070188	0	0.388871
LOC78044	0	0.296927	0.289428	0.30034	0	0.295082	0.446215	0
LOC78045	4.473316	4.693242	4.321543	4.556943	3.987601	3.943116	4.360952	4.151441
LOC78046	1.450658	0.864527	1.432582	1.574038	1.024126	1.116905	1.212579	1.038571
LOC78046	1.823238	1.248085	2.043833	1.363427	2.168455	1.587627	1.500475	0.949585
LOC78048	0.009617	0.009743	0.018994	0	0.009618	0.004841	0	0.004877
LOC78077	5.947697	3.603638	9.558977	10.45715	7.52306	0.410964	0.236742	0.473127
LONP1	23.81127	23.13705	20.96177	21.6401	21.62044	21.28878	22.69025	23.27322
LONP2	23.99918	26.44538	28.14758	29.7654	24.16032	26.80815	27.35645	25.25235
LONRF1	7.971791	8.223538	7.366752	8.137925	7.605904	8.280197	9.068849	9.333337
LONRF2	0.032877	0.033308	0.032467	0.056152	0.021921	0	0.10011	0.06669
LONRF3	1.432531	1.409209	1.64984	1.549357	1.848544	1.156899	1.135593	1.422256
LOR	51.71115	37.62959	64.88079	50.17167	44.31875	38.20938	46.9511	41.43541
LOX	11.95707	12.99511	12.54271	11.32837	12.24855	11.20716	13.33563	13.44764
LOXHD1	0.037926	0.008539	0.016646	0.021592	0.021073	0.004243	0.042772	0.034192
LOXL1	24.24968	18.78112	18.31811	19.7798	20.21638	20.16819	24.67128	25.24242
LOXL2	5.01258	2.360754	3.291163	3.304176	3.170528	3.109935	2.667641	4.396919
LOXL3	1.86523	2.121397	2.092527	2.350842	2.255004	1.762847	1.824298	2.667341
LOXL4	0.583108	0.46639	1.000147	1.21869	1.005223	0.471218	0.272564	1.501866
LPAR1	3.806145	3.825924	4.046947	4.864849	5.057645	3.796164	5.108663	3.648886
LPAR2	2.319193	1.786363	1.490261	2.051073	2.478362	1.887221	1.805794	1.9011
LPAR3	3.588835	3.427082	3.488596	3.255225	3.186323	2.65105	3.633157	3.345309
LPAR4	0.019511	0.128489	0.028902	0.059984	0.029271	0.108046	0.029706	0.059368
LPAR5	0.557149	0.564462	0.470177	0.923901	0.658535	0.448765	0.442123	0.842484
LPAR6	40.25944	43.33022	42.88977	45.34291	45.68459	44.03765	49.12976	45.14223
LPCAT1	7.539195	6.532092	6.390576	7.296275	7.650939	6.802159	7.555927	8.007584
LPCAT2	4.782566	5.836994	5.44488	5.459699	5.291243	5.538772	4.904609	8.947312
LPCAT3	49.46955	40.52591	62.1058	57.39438	57.983	63.70963	50.89703	48.68737
LPCAT4	13.95471	13.54541	11.96964	14.36847	13.75714	13.66199	11.99221	13.57375
LPGAT1	3.911833	4.058948	4.297689	4.38956	4.287271	3.951685	4.020142	4.172509
LPIN1	2.762279	3.492059	2.199427	2.7813	2.410677	2.533613	2.568847	3.295427
LPIN2	8.234298	7.66328	6.720487	7.975567	8.775386	7.629744	10.39411	8.866111
LPIN3	20.24045	18.8617	27.46826	23.00265	22.20905	21.23817	22.6552	18.35195
LPL	1.18411	4.655637	0.878531	2.866989	5.712688	1.27868	1.432282	2.520163
LPO	0.049856	0.090919	0.029541	0.194146	0.029917	0.190747	0.242898	0.070792
LPP	3.465101	2.962049	2.51995	2.508811	3.710397	2.810927	3.206466	3.920307
LPXN	1.412666	1.349755	0.884675	1.659511	1.60812	1.133228	1.468828	1.747288
LRAT	25.03528	30.3046	36.95383	21.09215	28.58111	32.3135	18.56055	15.64686

LRBA	9.08677	9.915935	7.219968	9.588699	8.755836	8.202512	9.671514	9.530085
LRCH1	5.299821	5.264832	4.777255	4.779706	5.308762	5.365118	4.69224	5.283168
LRCH2	0.73431	0.622839	0.668946	0.495833	0.728712	0.56739	0.450661	0.311761
LRCH3	8.466973	8.34433	7.237033	8.059876	9.566709	7.247103	7.911754	9.889876
LRCH4	8.265193	7.668616	6.976004	8.040078	8.428345	6.670763	6.976461	7.802725
LRCOL1	0	0	0	0	0	0	0	0
LRFN1	0.361261	0.244002	0.288806	0.46717	0.53336	0.311768	0.497638	0.226822
LRFN2	0.005292	0.010723	0.015678	0.005423	0.005293	0.005328	0.010743	0
LRFN3	1.0059	1.171966	0.974741	1.198324	1.314127	0.981123	1.212424	1.00109
LRFN4	1.4431	1.544574	1.31019	1.633887	1.850666	1.382655	1.33481	1.274787
LRFN5	0.044199	0.089559	0.065473	0.090588	0.022102	0.055626	0.078509	0.056036
LRG1	0.440081	0.356685	0.449084	0.405884	0.909619	0.531705	0.268009	0.654641
LRGUK	0.011823	0.047913	0.011676	0.004039	0.015766	0.051583	0.016001	0.015989
LRIF1	7.092303	6.983979	7.281087	6.291328	7.449928	6.375464	6.62303	7.555006
LRIG1	30.65875	21.35696	19.18564	29.85491	24.86958	24.73573	27.39359	24.44675
LRIG2	2.835441	2.625962	2.107202	2.534668	1.560495	2.145141	2.149536	2.079678
LRIG3	15.50645	18.07869	11.83876	16.40315	13.05206	13.67144	15.21992	17.06107
LRIT3	0.012798	0	0	0	0	0.012885	0	0
LRMDA	1.411508	2.057851	2.923832	2.010948	2.513497	1.767773	1.083246	2.025192
LRMP	0.705609	1.221236	1.698492	2.304841	2.499356	1.346856	2.58128	2.728431
LRP1	38.97785	31.02153	27.20374	32.62763	34.06369	24.76084	35.03045	37.11095
LRP10	44.02372	35.94917	38.68634	42.84654	39.17952	38.6501	39.40236	40.03898
LRP11	3.706257	4.829952	3.546174	3.987178	2.268646	3.050293	2.934556	2.752974
LRP12	9.786084	7.775325	6.798228	8.121594	7.926306	7.353301	8.91023	9.500021
LRP1B	0.007592	0.007692	0.011247	0	0	0.011466	0.015412	0.026951
LRP2	3.277552	6.066525	11.00267	4.919815	2.17259	4.608572	8.149806	3.285492
LRP2BP	0.206252	0.116088	0.301751	0.493176	0.084039	0.19997	0.248114	0.054234
LRP3	1.983232	1.436729	1.589982	1.322125	1.919505	1.642513	2.467525	1.103061
LRP4	8.296474	7.770856	6.441902	7.878213	7.14335	6.291432	6.863872	9.089031
LRP5	11.65133	9.600197	7.583478	10.41486	9.95335	9.578712	9.524596	11.13659
LRP6	10.53435	10.13061	8.332028	9.095223	11.62287	9.646535	9.872099	11.34523
LRP8	0.580511	0.721631	0.37984	0.810219	0.605519	0.473318	0.451855	0.538205
LRPAP1	31.70219	36.34575	32.59928	39.8438	37.24119	32.79106	32.75201	36.90568
LRPPRC	13.98561	15.53202	15.93243	15.24187	15.3911	13.81931	15.092	15.1443
LRR1	2.771059	3.297612	3.373607	2.674426	2.90339	3.528082	2.440585	2.988952
LRRC1	25.37927	23.66119	23.93544	22.77291	22.27331	23.48525	22.17597	25.7211
LRRC10B	0.169289	0.028585	0.069658	0.115655	0.19753	0.127834	0.042957	0.042925
LRRC14	4.241704	3.912303	3.17542	3.707999	3.877327	3.926271	3.757519	3.623622
LRRC14B	0	0	0	0	0	0	0	0
LRRC15	217.6999	203.873	243.2842	249.075	213.2192	234.2448	199.6355	201.1412
LRRC17	2.363818	3.34225	3.642635	3.766653	3.935876	3.282262	3.58573	2.76631
LRRC18	0	0	0	0	0.034151	0	0.011553	0.011544
LRRC19	0.005496	0.055677	0.103116	0.039422	0.280313	0.011066	0.178498	0.061312
LRRC2	0.635337	0.704563	0.712207	1.187771	0.918782	0.708831	0.67101	0.548595
LRRC20	2.910665	2.545151	2.9685	3.204695	3.344232	2.407237	3.587395	2.222861
LRRC23	0.032246	0.163344	0.143297	0.115655	0.209623	0.032466	0.163646	0.310694
LRRC24	1.765788	1.7024	1.546907	2.509989	2.321457	1.95707	1.503198	1.964241
LRRC25	0.264266	0.39578	0.408479	0.353232	0.517108	0.393322	0.32654	0.442828
LRRC26	0.351584	1.130543	0.830269	0.767581	1.498248	1.185084	0.791294	0.573644
LRRC27	0.446429	0.50145	0.383362	0.51716	0.456193	0.615591	0.394022	0.521686
LRRC28	2.689822	3.168123	3.314931	2.498457	2.433963	2.948333	2.559814	2.759471
LRRC3	0.441313	0.969776	0.730448	0.598746	0.777059	0.619555	0.643508	0.712369
LRRC30	0.014743	0.007468	0.029118	0	0.007372	0	0	0.014953
LRRC31	1.119567	0.226852	0.768113	1.183529	1.072566	0.949234	0.837318	1.004025
LRRC32	5.918542	4.253474	5.234404	5.210648	6.747427	6.12336	5.078109	5.446872
LRRC34	0.326446	0.460146	0.280329	0.116359	0.212927	0.328676	0.18728	0.187139
LRRC36	0	0.020759	0.020235	0.041995	0.020493	0.02063	0	0.031172
LRRC38	0.049847	0.126253	0.270743	0.140475	0.149561	0.025094	0.202379	0.189588

LRRC39	0.053774	0.081719	0	0.041329	0.080671	0.013535	0.040935	0.013635
LRRC3B	0.154508	0.297418	0.335683	0.451256	0.703103	0.443357	0.352858	0.274239
LRRC3C	0	0	0.090107	0.070128	0.11407	0.068901	0.023153	0.092543
LRRC4	0.078065	0.194681	0.047441	0.159997	0.234224	0.253932	0.457128	0.414151
LRRC40	2.28477	3.004701	2.151492	2.567948	2.211353	2.359743	2.746942	1.859816
LRRC41	13.74508	13.0829	11.12983	13.88928	14.02412	12.89507	12.09928	14.09923
LRRC42	18.40135	19.14355	18.51371	18.5364	17.92593	19.72129	18.56033	19.93315
LRRC45	3.655847	1.95562	1.444118	2.637468	1.716033	1.77661	2.731064	2.234622
LRRC46	0	0.018342	0.035757	0.074211	0	0.018228	0	0.036724
LRRC47	9.076438	10.71828	8.452096	10.07138	9.962825	9.483133	9.610921	8.426513
LRRC49	0.62036	0.817976	0.378389	0.467446	0.415147	0.665931	0.500028	0.481146
LRRC4B	0.009756	0.009884	0.009634	0	0.019514	0.019645	0.019804	0
LRRC4C	0.709414	0.754406	0.486924	0.531061	0.991296	0.648407	0.847725	0.515396
LRRC51	4.411987	3.397925	3.055823	3.559735	4.312725	3.919524	2.553163	2.956199
LRRC52	0	0	0	0.025473	0	0	0	0
LRRC53	0	0	0	0	0.007092	0	0	0
LRRC55	1.618255	0.498586	1.453606	0.813268	1.924421	1.04008	1.71003	1.537868
LRRC56	0.012156	0.018473	0.006002	0.006229	0.030394	0.012239	0.018507	0.012329
LRRC57	15.3398	14.58491	14.6987	14.66363	13.54069	15.24799	15.12945	14.92
LRRC58	8.586011	9.369023	7.035466	8.215264	9.741304	6.801091	8.215028	9.629002
LRRC59	34.79894	35.6056	34.41407	34.13375	35.90894	34.78825	36.78345	34.68373
LRRC6	0	0	0	0	0	0.017299	0	0
LRRC61	5.171331	6.197919	5.659704	6.678917	6.465	6.669355	4.775418	5.163841
LRRC63	0	0.01267	0	0	0.012508	0	0	0
LRRC66	0.734857	0.875402	0.358863	0.918567	0.953015	0.691094	0.655718	0.982837
LRRC69	0.140492	0.166059	0.077079	0.151971	0.093674	0.047151	0.087144	0.039581
LRRC7	0.021637	0.026305	0.004273	0.035476	0.021639	0.008714	0.008784	0.021945
LRRC70	0.74755	1.180592	0.814231	1.70112	1.649221	1.848438	1.461736	1.560984
LRRC71	0.113266	0.114752	0.093212	0.167658	0.125867	0.095033	0.102191	0.12126
LRRC72	0.162099	0.109485	0.124506	0.166115	0.396294	0.126939	0.255937	0.164407
LRRC73	0.183202	0.082492	0.100511	0.1043	0.183225	0.081979	0.103305	0.020646
LRRC74A	0.030193	0	0	0.030941	0	0.0152	0	0
LRRC75A	1.637716	1.619229	1.500396	1.951257	1.953673	2.433624	1.702337	1.961217
LRRC8A	9.589749	9.02036	8.966337	10.28409	9.684846	9.436764	10.12651	10.08319
LRRC8B	2.935875	2.609063	2.305766	2.276979	2.007291	2.1381	3.294172	2.985058
LRRC8C	1.098397	1.416307	1.045267	1.258631	2.087225	1.286867	1.165549	1.752071
LRRC8D	5.225228	4.618755	4.103855	5.071653	4.208782	4.197265	5.196823	5.352965
LRRC8E	9.002284	7.702605	7.202454	8.584055	7.38848	8.176373	7.156883	8.181909
LRRC9	0.124594	0.103688	0.096676	0.08664	0.146862	0.125445	0.081298	0.103802
LRRCC1	12.9978	12.97151	12.71502	12.4716	12.5676	12.34748	14.17163	12.68647
LRRD1	0	0	0.00797	0	0	0	0	0.008186
LRRFIP1	30.78993	26.97279	31.08066	31.1696	29.84789	30.50319	30.46966	27.84943
LRRFIP2	13.89854	12.2284	11.66922	12.1249	14.43054	13.66087	13.28821	13.94055
LRRIQ1	0.015074	0.022908	0	0.003862	0.003769	0.011383	0.003825	0.019111
LRRIQ3	0.302525	0.488122	0.24343	0.401876	0.280152	0.225623	0.398044	0.375016
LRRIQ4	0.365767	0.161116	0.141343	0.016297	0.270385	0.256185	0.11299	0.161293
LRRK1	4.738368	4.25061	3.521594	3.843644	4.365743	3.810518	4.243176	5.731047
LRRK2	2.623566	2.841074	2.60079	2.739992	2.239021	2.624643	2.527056	2.925648
LRRN1	0.76323	0.92171	0.367815	0.625708	0.384718	0.504099	0.768481	0.823637
LRRN2	3.39663	3.850687	2.287377	3.663964	3.000615	3.325926	2.571879	3.066587
LRRN3	0.1231	0.1641	0.140761	0.312055	0.149036	0.287022	0.17098	0.256277
LRRN4	0	0	0	0	0	0	0	0
LRRTM1	0.036468	0.073893	0.02701	0.037371	0.209718	0.036717	0.055522	0.073974
LRRTM2	0.005014	0.01016	0.004952	0	0.005015	0.030291	0	0.005086
LRRTM3	0	0.001861	0.001814	0.01694	0.005511	0.001849	0.001864	0
LRRTM4	0	0.034592	0	0.008747	0.017074	0.025783	0.008664	0
LRSAM1	7.649474	6.999604	7.246675	7.356026	6.219683	7.701732	5.910712	5.974542
LRTM1	0	0	0	0	0	0	0	0

LRTM2	0.020245	0.071789	0.009997	0.06224	0	0.010192	0.041098	0.051334
LRWD1	6.014827	5.30004	3.46909	5.451611	4.751822	5.063561	5.284203	5.369936
LSG1	7.439629	6.866816	7.078643	7.366925	6.932011	7.967373	7.643139	6.945002
LSM1	15.30017	19.4582	16.68654	15.85607	16.12229	18.90279	17.82955	18.05688
LSM10	7.139816	7.288526	6.35381	9.180615	8.362538	6.313934	8.569544	5.699549
LSM11	2.549678	2.82017	2.28215	2.686229	2.889054	2.557482	2.529768	2.552076
LSM12	27.1902	28.99892	29.3061	28.2276	30.17437	31.48745	29.74771	28.05318
LSM14A	46.01422	47.41486	44.74967	45.09893	47.68762	47.54795	46.95987	45.62437
LSM14B	18.08023	16.91362	16.01949	16.44311	17.22464	16.55389	18.37376	17.06603
LSM2	23.97278	25.05036	22.31065	25.72418	28.65181	26.03209	27.03951	27.56017
LSM3	30.94358	43.75565	40.96156	36.05227	32.65825	34.24698	41.90328	35.7605
LSM4	26.50659	31.72468	32.60948	32.66486	32.14901	32.79417	29.41221	26.99785
LSM5	22.02345	29.81713	26.8549	24.75964	23.41847	32.88544	20.99138	25.56397
LSM6	55.86547	63.68777	68.50662	58.75279	59.44704	69.23887	58.53629	58.95007
LSM7	92.29338	96.25654	85.89521	87.49967	94.07701	95.42089	98.77223	87.37874
LSM8	13.87673	15.56622	15.90051	13.6921	14.23212	16.78957	15.53524	14.59721
LSMEM1	1.38087	0.538074	0.402106	0.471691	0.548878	0.837747	0.628915	0.574575
LSMEM2	0	0.031719	0	0.016042	0.015656	0.078804	0	0.015877
LSP1	15.94731	20.91017	18.92244	20.0944	23.20526	17.46255	19.69636	24.71537
LSR	16.28045	15.77362	17.31915	19.33854	15.76182	18.36072	18.44523	15.90685
LSS	18.07565	19.71647	18.63776	22.66203	19.89494	23.04172	15.13643	15.29736
LST1	0.595514	0.974609	1.040474	1.314417	1.053738	0.830191	1.348381	0.929218
LTA	0	0.090741	0.176899	0.183568	0.179154	0.270532	0.227272	0.04542
LTA4H	31.94541	34.98038	38.4099	32.14437	33.37552	35.24163	31.61626	31.03225
LTB	0.858194	1.89145	1.219211	1.990338	2.017769	0.773103	1.46706	1.26744
LTB4R	1.75514	1.400986	1.050467	3.052196	1.688876	1.231636	1.484556	1.429495
LTB4R2	1.855417	0.759906	1.091582	1.800239	1.381872	1.192399	0.741279	1.12109
LTBP1	21.37599	18.76075	17.83802	18.95053	21.18509	17.24983	18.43231	21.90658
LTBP2	9.920642	6.637762	8.101448	8.864219	8.821555	7.932068	8.009004	7.42774
LTBP3	32.1319	26.66853	25.35346	30.46235	29.63336	26.36688	28.0956	31.91078
LTBP4	59.57612	56.31382	68.29471	63.80906	63.04209	48.81849	73.18778	69.18921
LTBR	20.07517	21.50122	20.51539	23.32985	23.94293	21.91217	21.86235	22.60842
LTC4S	3.204606	5.180845	2.861675	5.310274	4.80753	4.427854	4.117748	6.94995
LTF	0.118431	0.639921	0.15594	0.471973	2.250478	2.000581	2.617849	9.168917
LTK	0	0	0	0.025632	0.025016	0	0.025388	0
LTN1	8.942122	9.713554	7.971232	9.010103	9.22153	8.676409	8.565783	9.644624
LTO1	14.93158	15.21941	15.46192	17.43869	16.77753	15.64224	15.58507	15.52735
LTV1	19.5959	18.39467	16.03855	17.48786	17.40406	19.9986	15.20017	16.49566
LUC7L	40.35379	33.04366	29.52647	29.40796	34.80985	34.44636	34.05738	43.06567
LUC7L3	29.46533	30.41497	25.29815	31.13853	31.45974	28.79825	27.1548	28.39602
LUM	232.489	275.2781	237.4425	252.0652	293.6686	260.0572	286.5288	292.3535
LURAP1	0.914011	1.04422	0.941032	1.504621	1.171836	1.027945	0.932656	1.099607
LURAP1L	6.262416	5.260194	5.789973	6.515766	6.742554	5.742234	7.150896	6.173333
LUZP1	10.89546	8.827827	7.898079	8.463094	9.186988	7.964843	9.188293	9.24897
LUZP2	0.486918	0.580362	0.656219	1.027309	0.704689	1.430359	0.854712	0.906358
LVRN	1.923899	2.779341	2.15325	2.541115	2.651048	2.389018	2.148033	2.601716
LXN	5.630817	4.606737	7.166033	5.770294	5.395915	8.800437	6.910944	2.652378
LY6D	2.665635	4.664705	5.384496	3.021385	3.473852	5.164369	5.493227	6.308357
LY6E	24.98098	13.69239	18.03946	22.71812	21.49602	14.74861	12.92123	13.06626
LY6G5C	0	0	0	0	0.03274	0	0	0
LY6G6C	70.0946	55.36802	60.28451	58.33991	62.3365	53.48702	64.20659	56.9323
LY6G6D	308.9613	427.4324	438.7916	371.0794	485.9115	558.4273	409.4761	421.9526
LY6G6F	0	0	0	0	0	0.028124	0	0
LY6H	0.084816	0.057286	0.02792	0.144861	0.155515	0.028465	0	0.071686
LY6K	0	0	0	0.051867	0	0	0	0
LY6L	0	0	0	0	0	0	0	0
LY75	0.417749	0.977952	0.785034	1.654198	1.399436	0.820788	1.247345	1.369812
LY86	0.954924	1.934914	1.365762	0.944834	0.987981	1.094062	1.036093	2.938952

LY9	0.323341	0.051185	0.229505	0.258867	0.293065	0.396763	0.061536	0.143475
LY96	4.540937	5.703583	6.739646	6.041278	6.559978	6.416783	5.821959	5.19811
LYAR	13.90453	17.13433	15.27871	14.98723	14.66289	17.33619	15.44762	14.06593
LYG1	0	0	1.175188	0.04878	0	0.479258	0	0
LYG2	47.724	34.8935	59.15565	38.00501	36.27725	36.81299	39.6782	41.06328
LYL1	0.948055	1.237004	0.836944	1.015699	1.644944	0.744824	1.224714	1.551594
LYN	2.369937	2.557784	2.368186	2.248475	2.925879	1.777204	2.655314	3.930043
LYPD1	2.290928	1.441611	1.517623	1.735238	2.134681	1.948414	1.762023	1.905015
LYPD2	0.538253	0.793189	0.096645	0.351009	1.272399	0.098533	0.595993	0.496287
LYPD3	145.5935	131.6179	123.9636	144.3315	132.2223	130.8506	135.4022	130.8862
LYPD4	0	0	0	0	0	0	0	0
LYPD5	69.48705	46.28923	53.90728	37.23449	40.13125	54.71098	61.14994	51.3064
LYPD6	31.42943	30.60388	32.9748	32.01652	29.86104	34.52017	28.75661	29.68226
LYPD6B	13.31353	11.72988	12.3232	11.36942	13.46905	14.42198	13.89231	13.59218
LYPD8	0	0	0	0	0	0	0	0
LYPLA1	15.75977	16.45752	17.06733	18.1364	20.50419	18.30679	15.08277	14.36933
LYPLA2	43.39079	45.17236	47.06026	45.03544	44.99743	44.65427	43.76799	42.76086
LYPLAL1	7.156015	5.908966	7.30064	7.542794	4.735419	6.132292	4.751217	4.998664
LYRM1	5.238614	5.974577	6.769685	6.656786	6.885925	6.751234	4.922201	5.039939
LYRM2	3.627824	4.187946	4.367657	3.495514	3.751163	3.878167	3.660235	3.25435
LYRM4	6.928267	7.631079	7.702508	7.771852	7.878363	7.948533	7.443789	7.604449
LYRM7	1.964407	2.189207	2.148293	1.856494	2.321211	1.692141	0.989552	2.014509
LYRM9	2.555441	2.360383	1.896586	2.430535	2.172668	2.176662	2.519208	2.096872
LYSMD1	2.830239	2.912717	2.596122	2.785707	2.752288	2.69189	2.634248	2.189772
LYSMD2	0.83152	0.604823	0.510592	0.431521	0.709015	0.633267	0.719558	0.670361
LYSMD3	8.114372	8.479179	9.291107	8.067148	8.918675	9.010532	8.798962	7.447626
LYSMD4	4.931473	7.060736	4.376986	4.813475	4.687543	4.606111	5.419118	4.877667
LYST	11.05542	11.47246	12.25746	11.26743	12.6135	11.49548	11.74926	12.35877
LYVE1	4.412601	3.715597	7.036578	2.171468	4.110421	1.07845	5.955964	9.517642
LYZ	11.94204	17.20294	15.47863	23.71084	15.24051	18.97478	12.56309	22.58392
LYZL6	0	0	0	0	0	0	0	0
LZIC	11.30269	12.78999	11.20189	11.68237	12.23671	12.31871	11.42285	12.24483
LZTFL1	5.182134	6.145813	5.108276	5.676738	6.066984	5.672058	5.021215	4.931584
LZTR1	9.901868	9.699756	8.628579	10.33276	11.36964	10.34294	10.13794	10.00783
LZTS1	11.86264	10.75066	9.105639	9.498453	8.95855	9.634963	9.833047	9.362682
LZTS2	21.61231	21.90448	22.03258	23.94085	22.66683	21.48891	20.72397	20.59744
LZTS3	3.958215	3.892217	4.531945	4.818254	4.349342	4.083564	4.131941	3.896488
M1AP	0.010955	0.022197	0.032454	0.044904	0.010956	0.01103	0	0.011111
M6PR	42.49458	42.10346	40.59883	41.00611	42.23398	40.7276	41.22006	40.76806
MAATS1	1.04972	1.05327	1.2958	1.437744	1.070045	0.955267	1.444526	1.351303
MAB21L1	0.00576	0.017508	0.005688	0.023612	0	0.028998	0.011693	0
MAB21L2	0.010309	0.010444	0.010181	0	0.01031	0.041518	0	0
MAB21L3	0.33501	0.217733	0.137328	0.207281	0.208618	0.210016	0.37853	1.10268
MAB21L4	27.09494	22.75249	24.70102	21.78349	20.91474	21.00345	18.60653	19.52539
MACC1	2.372725	1.993727	2.132165	2.708067	2.209957	1.760566	1.894694	2.109967
MACF1	32.64044	26.04989	22.53417	23.76083	27.8437	24.16449	25.909	29.42701
MACO1	13.95126	15.4878	14.12685	17.02004	15.59969	16.91113	16.76248	15.2338
MACROD1	16.74292	17.13347	18.31594	17.74512	17.90864	15.29549	14.76926	12.56921
MACROD2	0.244742	0.33764	0.215981	0.325513	0.463508	0.267386	0.311838	0.264071
MAD1L1	1.741591	2.081059	1.443422	1.554551	1.797164	2.087799	1.876752	1.881942
MAD2L1	14.04467	15.64986	13.47396	14.47469	13.26501	15.9561	16.41093	18.14612
MAD2L1BF	3.052468	2.917951	3.006331	3.111261	2.806664	2.511531	2.856725	2.996054
MAD2L2	7.81703	9.195264	7.693724	9.032154	6.375114	7.738378	9.611668	9.178749
MADCAM1	0	0.037533	0.018293	0.018982	0.037052	0.130551	0.094007	0.056362
MADD	7.310323	6.984676	5.867942	6.757629	7.363759	6.00522	6.658954	7.235684
MAEA	35.06262	32.32882	32.72897	34.34294	33.54365	35.72893	32.85925	32.31055
MAEL	0	0.017269	0	0	0	0	0	0.017288
MAF	10.68614	9.525236	7.230622	7.946688	10.55488	7.175103	9.649142	11.28425

MAF1	30.42358	31.73933	32.40917	33.98545	32.40989	30.49749	33.14834	33.67655
MAFA	0.079515	0.187969	0.026175	0.19013	0.450641	0.26686	0.161415	0.026882
MAFB	44.65529	43.52353	41.96704	43.57463	43.4037	45.01032	43.96511	41.23082
MAFF	20.78691	15.98647	23.2309	22.73657	19.90654	19.57006	21.75164	17.32169
MAFG	6.016548	5.108585	4.761091	5.583538	5.502121	5.186572	5.004087	4.829511
MAFK	6.076813	5.00725	4.579438	4.772479	5.042549	5.376914	4.275828	4.44082
MAG	0.050088	0.091342	0.029678	0.061594	0.080151	0.040344	0.101679	0.050801
MAGEB1	0	0	0	0	0	0.027791	0	0
MAGED1	26.99085	30.10349	23.97474	30.45884	27.86571	28.6738	31.11725	26.55585
MAGEE2	1.357638	1.412298	1.041453	1.080717	0.703153	0.817707	1.070413	0.971252
MAGEF1	11.21618	14.89712	17.12119	14.59529	14.36404	14.37423	17.07664	14.21982
MAGEH1	7.493328	10.00817	8.525588	9.820392	8.402057	9.754745	9.85531	8.691832
MAGEL2	0.402182	0.511052	0.282732	0.530898	0.422687	0.452972	0.345945	0.470131
MAGI1	8.048988	7.160157	6.151931	7.792118	8.246371	7.902788	7.927763	7.982237
MAGI2	0.283137	0.538724	0.238691	0.233536	0.407494	0.305931	0.546731	0.448262
MAGI3	6.859327	7.193251	6.031367	6.695232	6.456454	7.991338	6.51486	7.242465
MAGIX	7.613053	7.429401	8.789652	8.71947	8.733748	8.228669	5.435589	6.358824
MAGOH	46.40522	54.08282	47.95011	44.06292	53.83539	54.5229	54.30648	49.69889
MAGOHB	10.15806	12.72067	14.35838	13.55939	10.33378	11.82961	11.3282	9.639406
MAGT1	21.49707	27.95815	27.17086	27.38937	25.84371	26.91912	26.67346	24.71512
MAIP1	2.952521	2.814217	2.388902	2.893696	2.695329	3.444988	2.772748	2.835962
MAJIN	0.027767	0.084394	0.054842	0.142274	0	0.08387	0.028184	0.028162
MAK	0	0.01269	0.006185	0.006418	0.006264	0	0.006357	0.019056
MAK16	18.85728	22.35722	18.88873	18.30149	21.8681	22.36775	22.37123	19.65927
MAL2	61.12479	70.9126	69.26767	62.42273	64.42348	68.85822	67.10476	67.78157
MALL	70.04298	64.22481	72.59226	64.97616	63.31185	85.41334	57.43868	51.5551
MALRD1	0.057949	0	0.057227	0.059384	0.405693	0.116689	0.058818	0.293868
MALSU1	16.2082	16.49694	14.78355	15.14865	14.93449	17.82994	17.44145	15.3354
MALT1	9.15794	10.01422	6.749501	7.558042	8.212793	7.697929	9.681184	9.999499
MAMDC2	0.611832	0.497636	0.868018	0.856588	0.663622	0.555278	0.804688	0.978883
MAMDC4	0.023515	0.095296	0.034834	0.096391	0.058796	0.023676	0.065637	0.0954
MAML1	8.28027	8.240813	6.646598	7.621355	8.330083	7.301479	7.612999	8.101565
MAML2	5.489702	5.421301	4.254214	5.259873	5.282442	4.522259	5.086604	5.813906
MAMLD1	0.464897	0.561575	0.306069	0.598569	0.882226	0.396061	0.405324	0.664957
MAMSTR	0.461619	0.620388	0.167461	0.617865	0.442834	0.436317	0.554602	0.793057
MAN1A1	5.178179	5.712323	6.143661	7.727039	9.099802	5.788034	6.445269	9.309045
MAN1A2	10.05927	10.12137	10.26171	9.81003	10.76064	10.5085	10.2235	10.89575
MAN1B1	19.03857	16.59306	14.13275	18.44597	17.73536	13.8512	16.62378	16.02947
MAN1C1	4.610056	6.290556	5.335791	6.025895	6.016417	5.128426	4.528687	5.689293
MAN2A1	18.01749	15.2969	17.20552	16.37181	15.76413	14.23669	15.64667	17.79651
MAN2A2	4.730962	4.023867	3.894277	5.138458	5.190565	4.192829	5.284995	4.804057
MAN2B1	35.43029	29.91416	42.76505	41.6875	36.56478	34.80853	40.11748	51.54569
MAN2C1	11.19408	10.10321	9.714885	9.227494	10.02123	9.625099	12.0545	12.10175
MANBA	3.466848	2.474106	2.713081	2.301449	3.118386	2.297751	2.500818	2.978082
MANBAL	29.32434	30.96168	27.42066	32.68585	33.4578	34.47864	29.93988	29.71671
MANEA	4.036738	4.072746	4.284182	4.63451	5.276916	4.114908	4.96435	4.722774
MANEAL	0.856161	0.682844	0.557665	1.00804	0.974692	0.816158	0.721088	0.785209
MANF	41.24067	42.59748	44.73222	41.81802	51.21688	48.5675	44.87617	39.22137
MANSC1	16.28692	14.53665	15.98617	19.31499	18.07219	18.56371	17.75204	16.49007
MAOA	7.832843	13.91721	10.59961	9.188649	10.64639	7.323043	10.31153	17.81505
MAOB	3.413052	4.975394	9.118378	4.429555	7.233609	3.878033	5.288679	6.207076
MAP10	1.051669	1.177626	0.883692	0.945368	1.273238	0.947396	0.87081	0.654955
MAP11	5.319999	4.878985	3.952986	6.242654	5.176606	4.910845	4.731352	5.510536
MAP1A	3.316361	2.913285	2.905521	2.896874	3.647443	2.348149	3.230838	3.04645
MAP1B	5.363482	4.834496	4.114586	4.74673	6.164558	4.755986	5.590564	5.467743
MAP1LC3A	97.39319	92.10097	114.1197	117.4893	104.5367	110.6213	99.93194	94.20224
MAP1LC3E	44.14284	42.5214	43.5148	41.9444	41.39666	46.48569	34.636	40.23136
MAP1LC3C	0.636117	0.840608	1.584134	0.538505	1.383043	0.362001	0.589515	1.570856

MAP1S	2.339546	1.89447	1.762308	2.598747	2.143438	1.67638	2.097309	2.25161
MAP2	4.50374	4.042624	3.626087	3.183674	4.317837	3.979969	3.526012	4.279817
MAP2K1	14.8469	13.84399	13.18667	14.97048	15.6096	14.24705	13.97195	15.04896
MAP2K2	22.48702	20.01769	20.42024	20.61552	20.43383	20.14927	21.44333	21.10115
MAP2K3	46.99624	41.29984	40.86198	43.83194	41.66974	39.94079	47.13221	46.17118
MAP2K4	19.8158	18.17244	16.99769	17.28983	17.88017	17.42655	17.14751	17.09708
MAP2K5	1.610844	1.726021	1.404493	1.601652	1.320584	1.534426	1.534718	1.688437
MAP2K6	1.92419	1.801918	1.540714	1.929218	1.643575	1.979224	2.523133	2.268055
MAP2K7	12.67131	11.59447	11.40464	11.76063	11.69441	11.23178	12.08807	11.26557
MAP3K1	23.20697	22.02772	18.47089	20.49814	19.98502	17.59338	21.28417	23.54965
MAP3K10	2.288373	2.574229	2.080629	2.789808	2.730618	2.439068	2.458856	2.737119
MAP3K11	11.20635	10.1017	8.989235	10.95696	11.87573	10.40206	10.31699	10.88496
MAP3K12	2.324153	2.032651	1.294725	2.592075	2.046314	2.100028	1.922161	2.135619
MAP3K13	0.50345	0.711396	0.484093	0.526782	0.742022	0.477543	0.661613	0.647677
MAP3K14	4.238865	3.762491	2.842764	3.870577	3.821799	2.891928	3.531861	3.503534
MAP3K15	0.052583	0.030442	0.074183	0.076979	0.037564	0.052942	0.030498	0.121901
MAP3K19	0	0	0	0	0.005433	0	0.005514	0
MAP3K2	5.590007	4.807126	4.528597	4.29836	5.262017	4.617064	4.993418	5.457934
MAP3K20	18.56398	17.13728	16.89052	15.39885	18.51993	15.48258	16.56668	18.08245
MAP3K21	4.100013	4.114356	3.957561	3.418144	3.189853	3.735812	3.607963	3.471901
MAP3K3	11.80266	10.8643	10.58994	11.05202	11.33202	9.376996	11.28892	12.00177
MAP3K4	15.38574	12.47706	11.73296	13.67594	13.52773	12.30127	13.17397	13.73338
MAP3K5	12.54439	11.9828	12.50244	10.2954	10.03681	10.84245	11.16548	13.05293
MAP3K6	6.531684	6.159038	4.446237	5.92825	6.198653	5.346841	5.095965	6.000959
MAP3K7	11.23885	11.98502	11.23348	11.50559	12.12111	12.39685	12.28404	11.48528
MAP3K7Cl	1.880586	1.613219	2.182495	1.997504	3.473357	2.584468	1.504744	2.575629
MAP3K8	1.545818	1.842196	1.832796	1.747815	1.461433	1.65099	1.864684	1.586885
MAP3K9	2.973321	2.623151	1.788066	2.194029	2.36667	2.044747	2.381064	2.457194
MAP4	29.43674	24.7528	25.62743	25.73225	26.86381	24.28222	28.26269	27.49805
MAP4K1	1.089274	1.229692	1.085947	1.498972	1.50443	0.783368	1.253029	1.094259
MAP4K2	8.064218	9.885218	8.100011	8.181194	8.139316	7.841436	8.540465	6.99108
MAP4K3	13.16711	15.62446	12.63841	13.51353	14.05244	14.89012	12.20014	13.29436
MAP4K4	26.97529	25.78151	23.07654	24.19916	25.99603	23.37352	27.52794	28.17631
MAP4K5	12.72064	12.51246	11.88662	11.64662	11.83731	12.20096	12.08338	13.35363
MAP6	0.048091	0.058467	0.066489	0.078852	0.057717	0.077472	0.126913	0.029266
MAP6D1	1.153476	1.436128	1.125379	1.56657	1.181423	1.609108	1.354153	1.733702
MAP7	17.45228	16.95114	17.09953	15.69936	15.94737	18.97482	16.32354	15.22383
MAP7D1	27.3935	24.1953	24.15202	26.43954	28.34607	25.58619	25.96953	25.58467
MAP7D2	0.479924	0.52512	0.385474	0.681979	0.543984	0.618499	0.428668	0.356954
MAP7D3	2.51416	3.344527	3.027977	3.108527	3.487482	2.701926	3.517147	3.592105
MAP9	2.67217	1.766658	2.447536	2.556355	3.156959	2.405939	2.040335	1.629401
MAPK1	40.69158	41.62273	39.01018	41.38635	43.5805	38.74885	39.778	42.44328
MAPK10	0.19363	0.176554	0.152974	0.287718	0.096827	0.506876	0.471682	0.324037
MAPK11	4.391114	4.629863	2.460604	5.015138	4.257585	4.691104	4.479663	5.473539
MAPK12	17.82861	15.1809	14.46452	18.31531	17.14173	16.84327	14.77744	18.87055
MAPK13	40.67112	35.10804	37.39234	34.80801	34.77891	35.7236	37.83768	33.75381
MAPK14	16.30764	14.23135	14.41659	15.56728	17.47213	16.18502	16.33946	17.83413
MAPK15	1.056195	1.31588	1.183985	1.053104	0.927859	0.68978	0.840246	1.447609
MAPK1IP1	20.55096	21.23093	22.37332	20.47578	21.1771	21.12053	19.12862	19.90321
MAPK3	53.25372	50.14143	49.50203	56.55586	56.48101	52.86937	52.73264	45.53322
MAPK4	0.141703	0.2297	0.083962	0.243958	0.181403	0.085603	0.143828	0.218455
MAPK6	5.890074	5.594005	5.714523	5.810701	6.05896	5.793609	5.7553	6.780501
MAPK7	4.958331	4.828148	3.616179	4.74899	5.099155	4.16311	4.597012	4.744596
MAPK8	4.743356	5.289577	4.744052	5.026326	5.093879	4.843502	5.251568	5.725289
MAPK8IP1	3.032623	1.803992	2.436166	2.280912	2.662004	2.334358	2.786304	2.09756
MAPK8IP2	0.425961	0.314673	0.490762	0.372854	0.514769	0.366327	0.558452	0.52203
MAPK8IP3	1.905733	2.085036	1.642166	2.454882	1.984194	2.034789	1.980275	1.962085
MAPK9	4.934345	5.011207	4.672284	4.619885	4.676085	4.627227	4.519246	5.053059

MAPKAP1	12.6132	11.76758	12.97028	12.14002	11.66728	11.89838	12.58218	13.64366
MAPKAPK2	28.64181	25.73054	25.43986	28.64425	30.54775	30.5084	28.9768	29.68311
MAPKAPK3	17.82037	18.06436	16.26661	17.67831	18.07243	17.88176	17.97615	19.00612
MAPKAPK5	13.22514	12.83017	12.66043	12.3848	12.54989	12.13305	11.47987	13.23151
MAPKBP1	7.001947	7.08252	5.987837	6.881502	6.786806	5.669824	6.339574	7.981772
MAPRE1	31.66451	33.2364	30.17386	31.20412	33.76309	33.21974	38.4207	33.9738
MAPRE2	14.97304	14.68754	14.15707	16.26382	17.72218	13.98173	15.40719	17.43498
MAPRE3	35.96582	38.59199	31.62359	34.21559	31.69164	37.56086	35.4197	32.8052
MAPT	8.070495	6.324915	6.758699	7.264884	9.911555	5.557041	6.224584	5.859143
1-Mar	0.247145	0.250388	0.254235	0.253267	0.288373	0.196993	0.209043	0.271552
2-Mar	24.92225	36.6471	33.24492	30.72387	35.51772	27.02603	35.98765	29.94289
1-Mar	0.579403	0.650938	0.617505	0.80539	0.734387	0.525602	0.459994	1.093845
10-Mar	0.208403	0.127963	0.049892	0.226509	0.044212	0.336995	0.141019	0.256206
11-Mar	0	0	0	0.072066	0	0	0	0
2-Mar	9.940863	11.4687	8.537965	10.77316	10.24495	9.399103	9.321704	9.263504
3-Mar	21.32256	18.8954	19.80287	19.0855	23.85207	24.66449	23.85765	18.84905
5-Mar	16.13225	16.51001	17.30341	13.71326	15.48582	17.65578	16.0994	15.48021
6-Mar	19.20581	20.41424	21.63605	20.02452	19.5516	18.80996	19.00611	20.04497
7-Mar	31.14718	33.2288	34.12291	37.50374	31.88452	34.24892	32.54612	34.01477
8-Mar	7.993942	8.256419	7.141763	8.861336	8.062378	7.928503	8.219089	8.412701
9-Mar	2.269392	2.53335	2.199613	2.325605	2.049021	3.258091	2.761986	2.312354
MARCKS	63.39793	69.37731	81.60595	73.25081	65.34898	67.91941	78.04562	78.15159
MARCKSL1	94.0994	86.53589	80.20026	87.97743	93.46624	84.59379	95.06838	89.30158
MARCO	0	0	0	0.075282	0	0	0.018641	0.037254
MARF1	18.4735	15.09019	15.07041	14.94443	17.59919	14.47495	14.44164	15.99949
MARK1	3.850968	3.857236	4.153614	4.881169	3.174045	4.416245	3.803391	4.066453
MARK2	9.746631	9.386009	8.137062	9.40434	9.787075	9.169925	9.329973	9.815076
MARK3	19.72478	18.31614	15.71221	18.4196	18.25541	16.74689	17.61641	18.61888
MARK4	12.41327	10.90203	9.744417	11.1813	11.99578	11.22439	10.80184	11.98053
MARS	26.1013	24.80717	27.27868	26.84409	26.09529	28.14255	26.62629	21.55744
MARS2	2.606509	2.621511	2.47107	2.476813	3.867611	2.633858	2.433958	2.403285
MARVELD1	25.46136	31.29869	22.79072	27.7333	25.42605	25.26617	24.09036	29.46401
MARVELD2	5.410035	5.224913	4.814782	4.256108	4.717235	5.141555	5.051304	5.303904
MARVELD3	3.375769	3.389192	3.815325	3.592141	3.429554	3.207023	2.83858	3.423825
MAS1	0	0.010368	0	0	0.010235	0	0	0
MASP1	5.491834	4.985299	4.354065	4.817868	5.698172	4.572526	3.94183	3.262304
MASP2	0.155866	0.056397	0.175914	0.079864	0.111348	0.168141	0.090403	0.056459
MAST1	1.634908	1.312261	1.347342	2.123753	1.312701	1.089657	1.092654	1.395442
MAST2	4.581125	4.007298	2.954385	4.287392	3.974171	3.140861	3.578941	4.308172
MAST3	5.737465	5.703945	4.203076	5.686962	5.514408	4.587084	4.765117	5.528639
MASTL	2.842532	2.750321	2.272425	2.835881	1.902787	2.733239	2.46537	2.715204
MAT1A	12.40931	7.839306	12.15956	11.77075	8.116061	12.47792	8.994419	9.003927
MAT2A	252.0711	193.1095	167.7818	257.7865	212.6737	205.7906	252.8623	238.4579
MAT2B	27.79123	31.74724	30.2081	30.16684	27.53388	31.61347	32.76487	29.7746
MATK	0.834163	1.162028	0.382464	1.160121	0.774681	1.004835	0.997868	0.740284
MATN1	0.143996	0.237064	0.266628	0.239789	0.180018	0.344326	0.200964	0.182557
MATN2	25.75652	23.73868	21.7736	25.27825	23.95187	32.77728	29.64793	33.68433
MATN3	0.087284	0	0.019155	0.019877	0.135793	0.009765	0	0.019673
MATN4	0.959832	2.232984	3.381914	1.772921	2.168789	2.63669	3.776641	3.99013
MATR3	79.19497	92.35325	83.21822	89.69976	84.23455	86.33533	89.94111	86.17949
MAU2	19.11127	23.86548	17.36571	21.92482	20.15798	18.11683	18.71003	21.53842
MAVS	9.977394	9.93117	8.413969	10.09511	10.25074	8.392489	8.588768	10.02089
MAX	27.45317	27.76049	30.94539	26.52559	34.12478	30.1942	29.51033	29.59418
MAZ	48.74659	42.31435	40.40561	44.48507	42.70954	40.71796	44.01187	44.82202
MB	0.17304	0.021914	0.02136	0.044331	0	0.087111	0.109772	0
MB21D2	4.074372	3.503967	3.098925	2.679796	3.948349	2.981106	3.322089	3.35381
MBD1	10.78158	10.61111	9.242814	11.03619	10.73435	9.680374	9.734237	11.083
MBD2	31.78345	34.32987	35.00713	33.15656	31.10932	34.06022	31.67427	33.86163

MBD3	30.96827	29.90211	27.54551	28.53474	31.28464	29.51078	29.10399	31.72589
MBD4	9.877072	6.888488	7.62719	7.365103	8.813765	6.970322	9.103946	8.511261
MBD5	1.561368	1.459321	1.270449	1.560606	1.608307	1.381065	1.802418	1.578019
MBD6	8.747026	7.952917	7.901915	8.01054	8.00265	8.262169	6.354029	8.075381
MBIP	11.89316	14.71276	14.0763	15.66997	14.50616	14.58537	13.23333	16.18003
MBLA	0	0	0	0	0	0	0	0
MBLAC1	3.127908	5.403642	6.423997	4.993252	3.888579	5.721394	6.880898	4.840808
MBLAC2	2.927141	3.910336	3.299966	2.900101	3.503955	3.716523	3.194534	3.3629
MBNL1	29.06865	28.2645	28.92231	28.2448	28.39638	26.46714	28.85273	29.47905
MBNL2	25.95977	27.30761	27.76758	27.44206	27.22007	27.64104	26.88765	26.43381
MBNL3	1.468845	1.832004	1.483173	1.794578	1.826531	1.699653	1.972593	1.721292
MBOAT1	9.833011	8.461058	8.48037	8.916127	9.579459	6.736306	10.21038	9.647576
MBOAT2	47.70145	57.94511	64.30133	74.04183	94.16391	85.92415	47.9907	37.9448
MBOAT4	0	0	0	0	0	0	0	0.021703
MBOAT7	14.35213	16.56085	16.58027	19.13541	19.31996	22.55241	13.88248	11.90053
MBP	3.017926	3.450341	2.752658	5.315545	2.944954	3.302305	2.680295	4.686984
MBTD1	8.794464	9.433697	8.81744	8.986833	8.502248	9.635385	9.325015	9.807087
MBTPS1	25.22237	25.65303	22.37002	24.13385	22.62242	23.38793	26.06654	26.1799
MBTPS2	4.191788	4.119396	3.57776	3.669686	4.114472	4.033519	3.944682	4.433666
MC1R	0.205371	0.089171	0.231785	0.481048	0.616193	0.561244	0.655134	0.178538
MC2R	0	0	0	0.01516	0	0	0.015015	0
MC4R	0	0	0	0	0.01447	0.021851	0.007343	0
MC5R	3.004972	3.769268	3.730608	6.070829	5.409648	5.6476	3.921488	5.108608
MCAM	13.73987	16.61297	10.66682	16.31973	18.67416	14.66628	16.97346	15.2384
MCAT	7.30031	7.438463	6.562707	7.381202	8.137275	8.135698	8.130999	7.460755
MCCC1	7.735025	10.33925	9.614537	9.713551	8.708617	10.08371	9.167122	8.219256
MCCC2	10.70288	11.28232	9.534921	10.88847	11.37045	8.822645	12.53724	12.39006
MCCD1	0	0	0	0	0	0	0	0
MCEE	11.22765	17.33797	21.67009	16.65222	18.52322	21.61032	13.11684	14.82644
MCEMP1	0.040243	0.020386	0.079484	0.18558	0.100621	0.081036	0.06127	0.387753
MCF2	0	0.005621	0	0	0	0	0	0
MCF2L	2.25802	1.657721	1.334237	1.959434	2.267663	1.539165	1.433025	2.256974
MCF2L2	0.248041	0.496881	0.239383	0.352393	0.315729	0.300818	0.274649	0.360205
MCFD2	22.98699	25.77257	30.97156	24.24773	28.04215	29.50573	25.23574	23.79819
MCHR1	0.048677	0.024658	0.04807	0.137177	0	0.134776	0.024703	0.061712
MCHR2	0	0.027477	0	0	0	0	0	0
MCIDAS	0	0	0	0.013856	0	0	0	0.013714
MCL1	78.52692	67.2744	69.25759	75.99489	80.82646	81.36173	81.46188	81.18187
MCM10	6.991478	6.957589	6.108294	5.059743	4.736525	5.416001	7.395309	6.957362
MCM2	17.39411	19.16926	15.02812	17.42732	14.5279	15.55101	16.38783	18.02673
MCM3	24.69941	24.93492	20.21837	24.04701	22.33122	22.54254	26.74833	25.13089
MCM3AP	18.22257	16.76333	15.11881	17.33977	17.95492	15.458	16.52036	18.65421
MCM4	19.70241	17.84516	16.32296	17.18206	15.83978	17.03094	19.74147	20.31455
MCM5	21.05101	19.99218	17.66317	17.83276	16.28876	17.29379	21.43536	20.28829
MCM6	16.84472	17.76146	16.03747	15.54192	14.67768	15.68686	18.15388	18.38341
MCM7	53.22152	48.90276	43.49009	52.62904	43.54321	44.26903	56.14644	52.8099
MCM8	0.988543	1.595636	0.703213	1.090294	0.586499	1.577291	0.816302	1.028106
MCM9	3.227707	3.685389	3.173529	2.979307	3.699383	3.562872	3.596561	3.397911
MCMBP	23.59138	25.64097	23.59548	23.13007	24.26999	25.28637	23.68997	24.57587
MCMDC2	0.006827	0.013832	0.013483	0.020987	0.013655	0.02062	0.034645	0.013848
MCOLN1	16.60093	12.73744	15.42136	16.7286	14.55536	16.39119	17.97327	14.03104
MCOLN2	0.374907	0.225523	0.185118	0.384194	0.292934	0.471835	0.463772	0.332714
MCOLN3	0.0083	0.008409	0.016394	0.008506	0.008302	0.008357	0.008425	0
MCP-3	0	0.030957	0.181051	0.031313	0.06112	0.030765	0.031014	0.030991
MCP1	2.021731	2.420676	1.529817	2.690663	0.420154	1.030989	1.412455	0.878791
MCPH1	4.714412	4.122234	3.657427	3.769198	4.018387	3.592035	4.017783	4.686755
MCRIP1	7.758894	8.174661	7.101858	7.889225	8.492419	7.832158	8.010072	7.791796
MCRIP2	2.47607	2.66795	2.985597	2.635534	3.269929	3.243635	2.603464	2.254637

MCRS1	10.94994	12.19646	10.94122	12.29249	10.77889	11.90368	11.48612	10.65765
MCTP1	3.120644	2.009833	2.239749	3.052318	3.808929	3.233532	2.071249	3.372614
MCTP2	6.093737	6.038883	4.572151	6.194436	5.681218	4.950606	5.829675	7.025862
MCTS1	12.95511	14.63275	15.45183	14.01606	14.90468	15.18083	13.57146	13.2949
MCU	7.344044	6.062236	7.972521	6.98839	7.131579	7.412119	7.228593	7.881439
MCUB	15.3489	18.96967	27.85271	24.3867	18.64036	22.20665	22.29954	16.37411
MCUR1	12.62591	12.64955	11.99297	13.47595	13.88969	11.55205	12.98238	13.83826
MDC1	11.06417	9.482791	7.336446	8.240352	8.850965	7.69282	7.383196	10.14856
MDFI	8.163506	8.254003	7.948241	11.56388	9.298072	8.235813	6.06859	8.512951
MDFIC	15.01229	17.39326	14.60472	15.8154	15.6069	19.69773	14.37448	14.4769
MDFIC2	0.781307	4.563114	1.361597	1.083246	1.792641	1.341923	1.306161	1.445018
MDGA1	0.145332	0.372428	0.430562	0.26282	0.401851	0.163539	0.477243	0.190754
MDH1	118.1506	128.5067	125.2122	108.9968	110.1394	120.4833	122.7789	111.1693
MDH1B	0.034321	0	0.05649	0	0.011442	0.011519	0.023224	0.005802
MDH2	109.5877	110.7592	107.1651	114.55	107.8819	111.7674	118.356	111.2545
MDK	21.06584	22.84107	17.53092	21.83021	15.77183	14.62639	22.73321	16.65446
MDM1	1.964152	1.905451	1.518805	1.689996	2.094131	1.576459	1.429384	1.68202
MDM2	13.19386	14.12709	11.89678	13.97901	15.23009	14.48239	12.43992	13.3012
MDM4	1.737093	1.318907	1.297431	1.317699	1.493694	1.27855	1.337562	1.887377
MDN1	3.963245	3.225627	2.505298	3.180627	3.875947	2.994678	3.423902	4.338363
ME1	28.74983	30.37946	44.87513	37.70885	27.16285	35.54313	38.51622	23.79369
ME2	3.163526	3.31369	2.829096	3.39888	4.198151	3.169714	3.405348	4.987642
ME3	0.622535	0.693775	0.725438	0.57416	0.709781	0.689466	0.834072	0.79556
MEA1	77.09448	70.59563	77.67063	76.81142	77.79616	73.66067	73.79759	69.99354
MEAF6	20.80008	21.75001	20.90749	20.88418	20.59239	20.05765	19.47938	19.55256
MEAK7	2.867505	3.399095	3.166366	2.997813	3.264585	2.329644	3.111822	2.774224
MECOM	2.0963	2.065317	1.951759	2.808169	3.069345	2.419166	2.154793	2.036052
MECP2	4.044804	4.572432	3.657121	4.969994	4.340553	4.23946	4.07081	4.149396
MECR	2.614344	2.871141	2.406208	2.727326	2.536241	2.332133	2.568645	2.237917
MED1	19.3918	18.86916	18.6943	18.86911	19.44985	18.24875	18.2735	19.13981
MED10	30.44386	30.2717	33.0459	30.41729	30.53245	34.25961	32.64744	32.33669
MED11	7.093283	8.660503	8.302103	7.393242	7.175045	7.874227	7.548385	5.718515
MED12	10.64237	9.831748	7.844218	9.076832	9.23461	8.344132	8.628752	9.798381
MED12L	0.400621	0.371482	0.28834	0.483608	0.451606	0.355502	0.28602	0.303022
MED13	12.84002	12.12619	10.48121	10.20767	12.08519	10.21937	11.6242	12.06115
MED13L	18.43901	16.29382	14.45562	16.9207	16.16229	14.63604	15.00573	19.25741
MED14	8.894458	8.814157	7.364767	8.553492	8.57011	7.828326	8.189946	8.252212
MED14OS	0	0	0	0	0	0	0	0
MED15	17.41261	15.45987	14.12885	17.62025	15.03334	14.4548	14.74328	17.43911
MED16	6.232675	5.349261	4.616261	4.142465	4.973427	4.746782	5.924002	6.497498
MED17	22.1003	21.9066	19.25399	22.54288	20.48862	20.8434	19.974	22.69164
MED18	6.443864	5.706828	5.887387	5.570283	6.137807	6.509953	6.051095	5.824239
MED19	18.67926	17.68366	16.05117	17.94786	16.58876	17.7534	16.34061	15.08619
MED20	8.7397	7.753968	7.379385	8.13168	8.087027	8.799405	8.707466	8.72131
MED21	62.78747	74.10449	31.39187	28.31521	66.2006	60.25765	65.43892	27.62431
MED22	14.35149	13.55022	10.57385	13.82148	10.52078	11.9341	11.32547	12.59343
MED23	7.260608	6.906309	6.230313	7.489767	7.432659	6.712689	7.304397	7.255515
MED24	8.209965	8.418446	6.536632	6.688451	7.57176	7.064757	6.754437	8.146761
MED26	3.391566	3.285528	3.133501	3.574111	3.689242	3.194714	3.415823	2.95224
MED27	10.19259	10.30586	9.666157	10.50723	10.15345	9.529201	9.216504	10.23513
MED28	17.27365	15.80469	13.08294	12.36606	13.24972	14.35548	12.87221	13.92656
MED29	34.48206	39.17136	35.24787	35.14824	37.89849	35.16083	33.84506	33.03826
MED30	31.00679	29.81499	42.6499	39.20788	29.87558	47.19028	28.5214	20.69403
MED31	11.64143	12.19544	15.18904	11.0898	11.65215	12.37933	13.53611	11.87254
MED4	13.35984	10.61582	9.631361	12.10074	9.210008	9.880376	13.13073	8.9516
MED6	17.7486	19.74088	17.27526	17.8742	17.77644	22.00949	19.3627	17.43144
MED7	10.14852	9.549758	10.3662	9.955681	8.101161	10.58583	9.00668	9.922301
MED8	13.04833	13.27003	14.63171	14.26468	12.73871	13.51354	13.98966	12.31224

MED9	2.798916	2.59898	2.981918	3.001241	3.539118	3.23617	2.810164	2.944063
MEDAG	5.796191	8.873638	17.01927	7.5897	11.47794	5.952504	12.96904	29.6548
MEF2A	8.07095	8.446052	7.656386	8.44941	8.464049	7.678779	8.510322	8.517166
MEF2B	0.278222	0.582539	0.457926	0.836334	0.445213	0.448197	0.263569	0.507929
MEF2C	5.249465	6.004823	4.62307	5.488209	5.198806	6.194927	6.22782	5.154126
MEF2D	6.373511	5.789009	5.635767	5.945829	6.34338	6.016735	6.034134	6.012687
MEFV	0.009174	0.046471	0.090595	0.075208	0.100924	0.018473	0.06518	0.102348
MEGF10	0.877567	1.065724	0.711672	1.066063	0.650995	0.971333	1.468821	0.618916
MEGF11	0.028763	0.055042	0.037872	0.05895	0.028766	0.0547	0.048657	0.012965
MEGF6	9.268809	8.742481	4.732055	7.115998	6.010782	5.36623	7.223326	8.597625
MEGF8	4.437973	4.034728	3.195112	4.414534	4.131363	3.236563	4.005212	4.295233
MEGF9	56.69912	51.35935	51.01443	52.46218	47.46113	50.60039	49.35567	50.25059
MEI1	0.760024	0.692999	0.470347	0.789235	0.349656	0.63768	0.869139	0.801678
MEI4	0.231055	0.234087	0.051858	0.053813	0.115542	0.169188	0.223861	0.15978
MEIKIN	0	0	0.018538	0	0	0	0	0
MEIOB	0.281141	0.52219	0.493579	0.512187	0.218694	0.534672	0.364624	0.36435
MEIOC	0.051013	0.120593	0.050377	0.043564	0.080781	0.089883	0.13376	0.064674
MEIS1	2.658275	3.08461	2.42674	2.446958	2.766818	2.878724	3.019731	2.923406
MEIS2	2.348538	2.105195	1.746615	2.1393	2.271514	2.004543	2.148333	1.715411
MEIS3	5.818643	6.046161	5.009457	6.299134	7.177254	6.669555	7.919994	6.85479
MELK	1.907605	2.654603	2.154502	2.673884	2.357402	1.909598	3.316053	3.035573
MELTF	0.126705	0.629003	0.450455	0.707648	0.253443	0.440119	0.295793	0.257018
MEMO1	7.463029	7.019009	7.636025	7.555886	7.86806	8.734334	7.20287	7.087417
MEN1	18.23536	17.75163	17.1297	17.28792	18.81703	18.79733	17.80549	19.70138
MEOX1	2.421544	1.951915	2.714294	4.229464	4.755326	3.176632	1.928619	1.066664
MEOX2	12.33302	14.1901	12.88422	15.7202	19.00513	16.43462	12.81493	14.5617
MEP1A	0	0	0	0	0	0	0	0.019058
MEP1B	0.022047	0.055842	0	0	0	0	0	0
MEPCE	18.16244	17.13286	14.6022	14.97853	15.77733	16.86311	16.90583	15.74913
MERTK	4.355224	4.767223	3.045258	4.548933	4.256791	4.208658	4.629214	3.675871
MESD	28.18681	30.0354	28.10584	30.95712	28.16004	27.95077	29.33488	28.51097
MESP1	1.061201	0.456929	0.392992	0.869989	0.902137	0.721204	0.807839	0.430523
MESP2	4.240782	4.027911	0.69799	4.490689	2.403421	1.334298	1.327188	3.871037
MEST	15.34325	12.6226	8.92519	11.55914	9.237501	9.052519	13.30968	9.154619
MET	9.460451	9.637588	8.400139	9.234834	9.126092	8.213243	9.027366	9.939862
METAP1	9.469597	9.048286	9.579524	9.388813	10.71588	8.914631	10.85391	10.3563
METAP1D	1.522666	2.260983	2.410501	2.406087	1.906485	2.363968	2.123597	1.67402
METAP2	100.541	104.3067	117.7575	112.0358	107.8558	123.2226	118.7406	102.5664
METRN	0.734899	0.276545	0.580594	0.77462	0.251998	0.147984	0.213121	0.468513
METRNL	40.18297	43.78576	45.33909	41.23104	49.75923	49.28112	42.49165	39.95486
METTL1	7.209556	7.168353	6.84023	6.945461	6.599682	6.82388	6.954838	7.433051
METTL11B	0	0	0	0	0	0	0.012272	0.012262
METTL14	5.76121	6.490196	5.604508	6.017212	6.09981	3.871168	5.997248	5.245198
METTL15	1.068708	1.502132	1.238938	0.943673	1.28853	1.23337	1.213364	1.640877
METTL16	10.11158	9.245814	8.097474	8.382552	8.851239	7.442	8.382656	10.11558
METTL17	3.731282	3.646559	3.752194	3.646511	4.173205	3.688051	3.685642	3.304427
METTL18	3.278171	3.429493	3.342889	3.761036	3.617145	4.071894	3.327343	3.397117
METTL21A	3.598446	4.129863	3.692422	4.033292	4.262461	4.149501	3.703785	2.571879
METTL21C	0.041373	0.020958	0	0.021199	0.020689	0.020828	0	0.020981
METTL22	12.83499	17.79925	15.26341	11.80181	16.84015	14.33009	15.05903	17.64156
METTL23	1.164542	1.373073	1.328485	1.460872	1.054248	1.546484	1.110682	1.252394
METTL24	3.150427	4.289907	5.151941	4.847887	3.525692	3.855295	4.791384	4.243244
METTL25	0.887121	0.98965	0.846537	0.868237	0.907174	1.154111	1.578278	0.940188
METTL26	5.372237	6.333725	5.90946	5.054697	5.143483	6.005653	6.190213	5.623228
METTL27	3.731889	3.213736	3.408985	3.919937	4.603258	2.473611	3.093425	2.334092
METTL2A	11.38592	11.48567	11.70892	11.19561	11.14219	11.65131	11.08887	10.91142
METTL3	15.1108	12.43954	10.59595	10.46638	14.62435	12.39037	13.25565	14.02399
METTL4	1.1885	0.903073	1.173691	0.487176	0.693381	1.156732	0.623269	0.462077

METTL5	4.442725	6.126403	4.679859	5.083929	4.986369	3.926366	4.083481	4.631135
METTL6	6.76079	7.156213	8.395511	7.936473	8.338544	7.556252	8.885015	9.185355
METTL7B	0	0.017913	0.017461	0.036238	0.017683	0	0	0
METTL8	1.734935	1.569988	1.729952	1.40967	1.504928	2.006827	1.29365	1.958944
METTL9	11.51004	12.65181	13.38386	12.20341	12.59096	13.63802	13.18989	11.51226
MEX3A	1.823855	1.855688	1.37009	1.094263	1.714957	1.19282	1.645523	1.612663
MEX3B	1.159839	0.807854	1.169249	1.064759	0.950546	1.04612	0.858402	1.078321
MEX3C	22.78666	21.88713	20.41283	22.69597	21.93711	21.08534	20.24953	21.5087
MEX3D	16.99997	14.52525	14.29184	14.85042	16.49075	15.02759	14.71862	15.93072
MFAP1	22.77167	24.42413	20.08986	19.69796	23.65244	22.64249	23.37061	23.23432
MFAP2	13.5844	11.89262	12.05694	14.83576	14.14118	12.09813	13.38407	14.75666
MFAP3	5.986855	5.633457	4.847425	5.373223	5.180585	5.307546	5.250461	5.921977
MFAP3L	4.797302	5.128619	5.281449	4.506814	5.285703	5.024801	4.369959	4.950881
MFAP4	74.88274	64.83974	73.73351	62.78095	75.09572	65.71222	80.3705	74.98135
MFAP5	45.44691	56.45416	88.49979	66.47001	47.04264	37.46904	35.78497	63.48344
MFF	44.43872	52.91276	53.45947	51.38835	48.47014	53.19892	52.88426	49.93742
MFGE8	44.36566	38.14907	38.8084	43.18643	33.7101	39.38579	45.24137	42.18228
MFHAS1	22.57815	19.7009	25.3716	23.45165	24.86937	24.33781	20.74293	22.01918
MFN1	34.62121	35.08532	32.6996	33.92253	37.6922	34.09322	35.16004	35.57225
MFN2	20.16534	19.5614	19.31077	21.89063	18.29389	18.6129	20.59288	17.89065
MFNG	10.07931	9.302991	5.935221	6.995723	9.210442	7.129325	8.939801	7.141024
MFRP	0	0	0	0	0	0	0.03271	0.065372
MFSD1	22.22247	22.14292	20.40388	24.06258	23.2928	23.68948	22.11925	23.21741
MFSD10	6.076523	5.215115	4.986283	6.327838	5.607301	5.446812	6.544831	5.775066
MFSD11	9.335615	8.120621	7.6129	8.425361	7.821946	7.850628	8.428782	8.470255
MFSD12	7.519283	5.621215	5.363641	6.345737	6.694068	6.319791	5.849495	6.992997
MFSD13A	2.612992	2.62183	2.208256	2.922316	2.500253	3.098829	3.085717	2.535518
MFSD14A	23.67688	23.14269	23.75348	24.30505	23.98509	25.61014	22.54552	23.33313
MFSD14B	23.2822	22.14605	24.0149	22.97876	22.88098	21.55069	23.74254	21.98246
MFSD2A	3.424277	4.165739	2.676564	4.958801	4.799896	4.139866	4.857845	4.009398
MFSD2B	0.451055	0.469001	0.328215	0.291933	0.510471	0.334627	0.325294	0.517671
MFSD3	1.335967	1.88954	1.528322	1.72149	2.447384	1.464953	1.866187	1.180581
MFSD4A	2.594793	2.046995	2.268632	2.382524	1.986138	2.006415	2.01567	2.161529
MFSD4B	0.369481	0.378189	0.57929	0.409861	0.308576	0.249282	0.282234	0.378604
MFSD5	24.57547	23.77718	22.84899	29.96192	26.04862	25.95268	25.50554	21.57522
MFSD6	34.00452	29.67435	26.05505	29.11163	29.52855	30.17364	30.89503	29.65543
MFSD6L	0.074866	0.010835	0.126742	0.131521	0.085572	0.043073	0.162833	0.054237
MFSD8	3.971221	4.313019	3.911281	4.883507	4.162377	4.776696	3.977045	4.231827
MFSD9	8.663127	6.560852	5.199453	5.915809	6.750143	6.984139	5.462965	6.377902
MGA	7.69122	7.00308	6.173681	6.531672	7.302735	6.066956	7.140096	7.676756
MGAM	0.008683	0	0.017149	0.004449	0	0	0	0.026419
MGARP	0.415338	0.187017	0.341802	0.023646	0.046155	0.325248	0.02342	0.140417
MGAT1	14.74672	14.8673	13.95454	15.46459	16.46917	13.29263	15.58516	16.06034
MGAT2	10.86277	11.79302	11.4087	12.25401	14.43446	12.65691	12.03715	11.8726
MGAT3	0.269521	0.053424	0.283521	0.16812	0.199237	0.123883	0.112994	0.148564
MGAT4A	1.833816	1.750088	1.820238	1.59062	2.115734	2.051143	1.689802	2.701012
MGAT4B	21.01395	21.37919	20.78948	22.59439	22.12679	18.3509	19.52399	22.27257
MGAT4C	0	0	0	0.027161	0	0	0	0.026882
MGAT4D	0.256419	0.159867	0.15583	0.262771	0.059181	0.29789	0.060061	0.060016
MGAT5	12.75373	10.42081	8.371545	9.589119	10.18014	9.490763	10.31048	12.35066
MGAT5B	0.074183	0.135281	0.102562	0.106428	0.059354	0.037345	0.045177	0.090287
MGLL	24.21763	21.17422	14.99089	22.38788	23.71209	19.81371	16.17018	15.51517
MGME1	10.51045	10.76605	9.003206	11.13776	8.721126	8.808803	10.67793	10.90547
MGMT	7.6945	11.77833	10.45226	6.30119	7.7291	10.0475	9.924393	9.337581
MGP	222.6791	310.5371	332.9908	290.4834	322.5562	276.4783	339.208	306.9885
MGRN1	20.34477	20.10479	17.91856	21.38807	21.08951	18.95486	18.71456	20.47688
MGST1	53.24026	89.2445	80.38117	64.19544	68.76488	55.16336	70.29263	58.96319
MGST2	18.76272	21.31097	21.18079	20.53284	23.61999	18.71759	16.52824	17.63314

MGST3	321.3811	371.9156	428.0729	390.2702	363.122	461.3549	349.5934	268.5585
MIA	2.053184	1.77596	1.568522	1.63758	1.850042	1.940448	1.405705	1.8172
MIA2	4.054581	5.203478	4.400066	5.724041	6.117091	6.822831	4.826652	4.128733
MIA3	20.99604	21.96743	19.07781	21.49052	19.78728	20.42281	21.77573	21.06695
MIB1	15.46309	16.30413	14.2393	13.99608	14.42083	13.65612	14.75832	15.84377
MIB2	3.679213	3.614544	3.149878	3.517005	3.781498	3.147963	3.355549	3.603761
MICAL1	32.37844	28.19659	27.99668	34.4282	36.78751	30.68513	26.98245	31.54348
MICAL2	17.37121	10.94238	13.68209	12.47216	13.43973	13.23667	15.29092	16.94688
MICAL3	7.560989	6.964851	5.58307	6.081677	6.839678	5.711563	5.948152	7.78884
MICALCL	7.531464	7.104476	7.639649	6.762974	6.990618	7.224368	7.133013	7.738582
MICALL1	23.99249	18.38505	18.17898	19.91058	21.68569	19.58711	19.44905	20.80453
MICALL2	2.006115	1.827245	2.228758	2.777317	2.729827	2.078084	1.928523	2.650945
MICOS10	117.9725	133.0534	136.4399	126.7984	139.3011	140.1319	122.226	116.3944
MICOS13	28.47394	31.48424	31.99939	31.87231	30.72316	32.65019	30.73973	28.77577
MICU1	18.53227	18.90433	20.10523	17.80659	21.12466	18.9848	19.15055	22.31376
MICU2	21.09328	25.89071	25.54504	24.89331	20.93997	24.37901	23.8642	24.1627
MICU3	1.067117	1.582819	1.246677	1.129287	1.171888	1.228897	1.514958	0.933756
MID1	5.616735	4.247319	4.120062	4.815001	5.050314	5.152128	4.714277	6.518332
MID1IP1	30.46608	31.79044	39.4201	38.6886	28.5795	37.9851	32.11395	31.12456
MID2	3.175291	3.337	3.404837	3.221567	3.582539	3.383872	3.471454	3.909455
MIDN	23.5377	19.65346	19.0763	21.93903	21.20304	19.5239	21.46542	19.86378
MIEF1	6.630941	6.415328	5.885972	6.408215	6.206213	6.054865	6.338537	6.430942
MIEF2	1.3804	1.565504	1.383547	1.847417	1.432093	1.618012	1.861171	1.368707
MIEN1	26.94721	26.50004	28.59843	26.25237	33.52666	28.723	28.44547	27.4037
MIER1	21.71326	21.92246	21.32577	23.18994	23.03125	23.73778	21.06479	24.48755
MIER2	6.965629	5.859487	4.981945	6.510876	6.459873	5.334294	5.516831	5.898029
MIER3	9.861218	9.502759	10.07652	9.288491	9.899952	9.907036	9.612681	9.719405
MIF	200.2673	191.2434	208.8916	222.6188	197.3169	224.4713	189.8015	190.3928
MIF4GD	20.99695	21.76675	21.0565	23.82915	20.34915	21.5497	21.14686	19.33738
MIGA1	3.199363	3.955728	2.930851	4.055126	3.599749	2.860952	3.706683	3.479737
MIGA2	5.78371	6.163102	5.855763	6.572409	5.892004	6.086156	5.940631	5.959528
MIIP	4.273984	3.192514	2.861512	3.572549	3.387027	3.819986	3.749878	3.765422
MILR1	1.184727	1.462835	2.211964	2.219477	2.573411	1.02508	2.761986	2.365634
MINAR1	0.039118	0	0.006438	0	0	0	0.013235	0.006613
MINAR2	1.300969	0.863226	1.239521	1.136032	1.062901	0.996229	1.469271	0.966387
MINDY1	35.8022	31.95615	35.22184	34.68275	34.93659	37.31527	32.59491	36.0177
MINDY2	4.502846	4.337416	4.645699	3.406591	5.520981	4.031563	4.534602	5.466032
MINDY3	22.52015	26.06662	27.70981	24.50611	22.81587	28.52231	25.31848	23.68405
MINDY4	2.769289	2.683116	2.603418	2.379362	2.455189	2.672539	2.749456	1.993081
MINK1	26.52332	23.63759	20.72244	25.0196	25.60782	21.88123	22.39298	25.03712
MINPP1	22.13181	24.37577	25.05362	24.37015	21.73443	24.45636	25.44255	25.71858
MIOS	4.946299	5.079064	4.468951	5.608057	4.363257	4.758539	5.204999	4.526686
MIOX	0.678982	1.299354	1.067869	1.082357	0.804824	0.607663	0.893365	1.224263
MIP	0.176699	0.286429	0.348995	0.108646	0.247411	0.142325	0.25109	0.071686
MIPEP	10.45464	13.36533	10.12627	13.86964	9.559087	11.01316	12.63554	11.62073
MIPOL1	1.294428	2.08216	1.289512	1.89665	1.555785	1.268974	1.509768	1.750473
MIS12	9.430677	9.88245	9.632891	9.942541	9.358781	10.76743	9.879549	10.5818
MIS18A	1.581266	1.942544	1.561563	1.76917	1.306432	1.615141	1.581724	1.719992
MIS18BP1	5.380147	5.625236	4.306272	4.609808	4.547191	5.11866	5.286046	4.918749
MISP	0.217392	0.3343	0.283688	0.182995	0.26401	0.234511	0.122147	0.192925
MISP3	0.430857	0.637979	0.900073	0.764186	0.828679	0.433801	0.55506	0.487413
MITD1	7.070434	8.669973	9.341882	8.419853	7.997938	9.278903	7.795153	7.838739
MITF	13.77661	12.73355	11.98668	12.02414	12.70155	13.24144	11.71225	15.57616
MIXL1	0	0.039715	0	0	0	0	0	0
MKI67	25.47414	19.04104	15.46684	16.77336	18.34638	17.31277	20.92538	23.28595
MKKS	14.32462	15.7403	15.39146	13.94244	13.95205	18.19177	13.49953	14.23877
MKLN1	5.464328	5.977406	5.366173	5.513274	6.472306	5.369599	5.28241	6.190637
MKNK1	14.82906	13.54533	17.3642	14.43002	14.99516	17.61159	14.80156	13.86544

MKNK2	37.83552	33.71375	34.99864	36.24166	36.13282	37.84365	32.63208	34.61657
MKRN1	22.87542	21.94507	19.74812	21.47577	20.69529	22.25186	21.41396	20.03201
MKRN2	15.53098	13.47892	12.68776	13.59964	14.23022	13.58576	13.04057	12.80491
MKRN2OS	0.019794	0.020054	0.039095	0.040569	0	0.01993	0.020091	0.100381
MKRN3	0	0.019774	0	0	0	0	0.019811	0
MKS1	6.394669	5.845917	4.020872	5.721134	5.533602	5.847333	5.856742	5.852332
MKX	1.087034	1.132992	1.12755	1.121974	1.032425	0.873993	1.095402	1.11044
MLANA	0.043394	0	0	0.044469	0.0434	0.087381	0.044045	0.088024
MLC1	0.011286	0	0	0	0	0	0	0
MLEC	18.52809	19.32703	16.3439	19.01475	20.99169	18.24626	18.37076	19.30514
MLF1	10.18889	11.32697	12.56382	11.0734	12.63586	14.16227	10.11812	11.30589
MLF2	108.3465	102.5395	116.7552	111.5236	128.8206	112.1086	106.1781	105.8684
MLH1	6.925946	5.493376	4.834363	5.24158	4.566666	5.415052	6.327966	6.378863
MLH3	1.74134	1.363044	1.572485	1.413617	2.129053	1.11024	1.214318	1.684084
MLIP	0.303342	0.502356	0.334128	0.346724	0.420067	0.428755	0.301971	0.414159
MLKL	1.52773	1.080526	1.100683	1.417877	1.575975	1.01577	0.858219	1.754126
MLLT1	18.94556	17.58019	17.07521	18.69708	19.27766	18.52189	17.91245	17.99646
MLLT10	7.152644	7.177045	6.961948	7.729466	7.530749	7.128155	7.455677	7.389211
MLLT11	13.20244	12.57316	16.23222	14.92751	12.45591	15.70746	13.86949	10.86853
MLLT3	2.434038	2.410671	2.223993	2.377773	2.620911	2.574345	2.267363	2.427159
MLLT6	13.95153	14.65006	11.81442	15.18342	15.02185	12.86872	13.72387	14.9797
MLNR	0	0.060938	0.0198	0.061639	0.040104	0	0.02035	0.04067
MLPH	3.605463	7.602055	4.739678	7.293576	5.865489	5.940154	6.629955	4.772822
MLST8	9.237633	10.07609	7.383282	9.554924	8.651687	9.544124	9.341146	8.283367
MLX	33.49258	33.6049	32.35143	35.27701	35.48486	34.57193	31.49831	32.55818
MLXIP	8.17297	8.272687	7.302629	8.348708	7.563302	7.010416	8.061248	9.448689
MLXIPL	0.400218	0.73161	0.481151	0.561702	0.504688	0.718306	0.432714	0.485334
MLYCD	4.843274	5.396198	5.401742	5.12378	4.569717	5.875292	5.816955	5.071107
MMAA	8.014289	7.045092	7.382223	7.428937	8.189193	7.841495	7.075783	7.246775
MMAB	20.72324	18.35137	20.51555	22.86208	14.1755	20.04052	18.15164	17.64495
MMACHC	4.369976	5.615147	5.220733	5.308331	5.351248	4.271054	4.002792	5.534827
MMADHC	73.29435	78.40099	94.68142	90.59259	85.43673	94.20646	84.28141	74.48153
MMD	25.05346	27.36508	30.05116	29.02752	35.03307	32.33327	22.16116	23.24369
MMD2	0.005593	0.034001	0.011047	0.074515	0.039159	0.095737	0.085159	0.045384
MME	3.436941	2.963179	2.047438	1.947575	3.647208	3.361019	4.139848	2.728615
MMEL1	0.059806	0.084827	0.070873	0.110317	0.13159	0.048172	0.036422	0.133446
MMGT1	9.963667	9.733156	8.796874	9.078362	8.790139	9.42631	9.013098	8.793563
MMP1	0.230729	0.41357	0.245381	0.18188	0.372764	0.214435	0.23419	0.234013
MMP11	0.860453	0.871745	0.666267	1.31764	1.007251	0.723583	0.575623	0.952039
MMP12	0.156729	0.198482	0.206368	0.401528	0.065312	0.09205	0.304903	0.490127
MMP13	0.355794	0.060077	0.507522	0.121536	0.158151	0.159211	0.160503	0.320764
MMP14	74.07767	62.80907	65.75859	68.2213	70.47011	62.87011	72.08642	70.83106
MMP15	2.902925	3.38139	3.387982	3.611159	3.299205	2.836793	3.332504	2.73957
MMP16	0.892332	1.440667	1.140394	0.963336	0.73257	1.076192	1.753313	1.592281
MMP17	0	0.060359	0	0.012211	0.119169	0.023994	0.072565	0.084595
MMP19	1.785242	1.821059	1.400737	1.679096	2.237955	1.440412	1.948542	1.872664
MMP2	80.90926	90.05257	93.59607	81.88801	100.9488	76.12674	114.5658	136.2205
MMP20	0.212039	0.136705	0.171325	0.177784	0.15423	0.19408	0.313047	0.29326
MMP23B	1.447331	1.421209	2.110962	2.236183	2.249221	2.084944	2.779878	1.55827
MMP24	0.324174	0.282448	0.307329	0.392	0.28531	0.359027	0.17768	0.236728
MMP24OS	7.438868	9.016264	7.866113	7.536109	8.47597	8.036878	8.153797	7.665343
MMP25	0.156261	0.446999	0.390324	0.414459	0.229826	0.222111	0.251902	1.006851
MMP27	1.901402	4.162545	4.267522	3.610857	2.965506	3.574423	4.426676	4.706544
MMP28	1.554319	1.389457	0.846481	1.967601	2.228907	1.875623	1.624034	1.170743
MMP7	0.186417	1.834673	0.368188	0.218325	1.651334	0.938452	0.892005	0.02701
MMP9	0.522274	1.274719	0.410269	0.583867	0.308657	0.74096	1.481895	0.866797
MMRN1	3.274947	7.101211	6.438826	2.555258	6.264369	2.114129	5.564338	9.365733
MMRN2	7.166085	5.985334	7.7082	7.479875	12.1343	8.631269	9.428134	9.426222

MMS19	10.02609	9.544779	8.618712	9.985135	10.63242	9.875312	9.112167	9.416178
MMS22L	3.37567	3.251777	3.382186	3.200943	3.277714	2.773464	3.008159	3.56716
MMUT	29.01146	30.06593	32.5804	29.53684	33.56156	34.70583	28.52989	26.86756
MN1	0.565402	0.670094	0.509193	0.335254	0.835765	0.343707	0.667725	1.013457
MNAT1	8.355534	9.926671	9.324477	10.09838	10.0804	10.12909	10.85779	9.405533
MND1	8.21646	7.545762	7.47196	7.965669	4.020083	9.700952	7.769727	6.714704
MNS1	2.502168	2.997534	2.687744	2.339222	1.95809	2.766768	2.227808	2.983014
MNT	3.595972	3.170904	2.579907	3.023627	3.186587	2.583889	2.854418	3.148406
MNX1	0.02298	0.116406	0	0	0	0.023137	0	0.023307
MOB1A	32.83344	39.48477	36.01536	35.46184	38.39618	37.75868	37.60127	37.21747
MOB1B	24.33142	22.16168	23.89461	20.45343	22.45259	21.93037	21.57178	24.43333
MOB2	20.04031	20.6441	20.76965	21.95179	18.90974	20.0881	20.32295	20.45129
MOB3A	12.65201	13.56731	11.03369	12.5923	14.4857	12.03966	11.82554	12.72827
MOB3B	3.979746	5.221196	4.359658	4.293983	4.003646	4.967468	4.091656	4.306758
MOB3C	2.868889	3.149596	2.951601	3.943839	3.849008	3.673513	2.780024	3.670112
MOBP	0.011657	0.005905	0.005756	0.005973	0.081612	0.035211	0.005916	0.029558
MOCOS	3.569088	4.524236	3.937789	5.197494	4.662617	4.8658	5.537935	4.840962
MOCS1	1.733908	2.324551	2.701306	3.469468	3.296346	2.535857	2.366785	2.152759
MOCS2	31.18956	34.58563	34.70911	32.90255	30.78059	36.08012	33.63672	31.88952
MOCS3	4.379514	6.123623	6.772322	6.891934	8.211468	6.990333	7.402278	7.051332
MOG	0.076023	0.051347	0.050051	0.019477	0.038016	0.012757	0.038582	0.038553
MOGAT1	1.606553	1.627637	0.966795	0.334414	1.506338	0.303287	2.216667	2.036779
MOGAT2	0.462206	0	1.3947	1.184138	0.924532	0.43951	0.23457	0
MOGS	8.828404	7.311741	6.230184	7.165613	8.614667	7.109006	7.196419	7.428718
MOK	0.898529	0.971009	0.798599	0.874746	0.733893	1.025289	0.577604	0.592358
MON1A	4.730749	4.018505	3.123211	4.523835	5.153097	3.953744	4.614457	3.541768
MON1B	5.572311	5.080419	4.826104	5.952508	5.313051	5.800722	5.003477	5.090787
MON2	6.518396	6.733824	6.390918	6.866814	6.960594	6.436335	6.531093	6.471164
MORC1	0.008087	0.081932	0	0.008287	0.008088	0	0.016417	0
MORC2	10.33921	9.741707	8.15283	9.130963	10.54339	8.896867	9.114087	10.21991
MORC3	7.600526	7.907609	7.256725	7.291326	7.668147	6.473675	7.622752	7.303256
MORC4	4.343015	4.910383	5.102962	4.904256	4.771063	4.58786	4.702554	4.520961
MORF4L1	118.5239	134.5385	129.5408	122.0134	124.951	127.0049	134.1095	132.4082
MORF4L2	137.3292	144.8141	152.7301	139.0907	145.6989	149.2957	150.3798	157.0847
MORN1	0.754545	0.818408	0.464618	0.600394	0.390638	0.589884	0.432487	0.585219
MORN2	2.382057	2.844268	2.982476	3.835953	2.850329	4.068595	2.115564	2.113971
MORN3	0.208299	0.148504	0.11428	0.245082	0.162031	0.155349	0.125288	0.062597
MORN4	15.23171	15.95324	16.17183	15.33054	14.51848	15.79656	15.18437	14.67974
MORN5	0	0	0	0	0.038505	0.038763	0	0.039048
MOS	0.02736	0	0	0	0	0	0	0
MOSMO	2.338365	2.260823	2.637444	2.262485	2.908495	2.163125	2.457776	2.528158
MOSPD1	14.69086	15.20675	11.46348	9.891257	10.06805	20.5172	10.70328	13.97278
MOSPD2	13.23793	14.84637	15.7671	13.10345	15.60014	15.92834	14.51452	13.75025
MOSPD3	13.74496	13.68825	13.25014	13.03022	14.09001	14.20014	14.03031	12.34244
MOV10	4.992553	4.310241	3.704386	4.765512	5.449566	4.445621	4.720076	4.491926
MOV10L1	0.308815	0.396808	0.200832	0.316464	0.323921	0.348842	0.504573	0.404882
MOXD1	7.859776	8.434733	7.010998	8.638828	13.3643	8.43019	7.427821	10.25617
MPC1	32.10244	32.61955	39.24285	35.12633	34.87277	37.48656	33.3038	30.06593
MPC1L	0	0	0	0	0	0	0	0
MPC2	43.07443	50.14524	59.49819	47.38432	48.64168	55.83488	45.37332	44.52897
MPDU1	29.64763	30.09896	32.6347	31.38915	26.27267	32.20025	30.81971	25.31419
MPDZ	4.164533	4.708603	3.729949	4.112649	4.480969	3.695918	4.189284	4.414953
MPEG1	7.989738	7.600934	7.5859	8.474031	7.589441	3.859831	5.476697	8.859318
MPG	19.67609	19.77025	16.01918	22.32088	19.3547	20.27248	19.97123	18.03996
MPHOSPH	11.81496	14.21718	12.51355	12.38893	13.39788	15.74298	13.64167	13.48662
MPHOSPH	9.008914	14.38605	10.25219	13.9532	11.58213	11.73917	11.37001	12.41758
MPHOSPH	12.92692	13.23497	12.25465	12.69455	11.96506	12.75464	13.24489	13.08178
MPHOSPH	3.902532	3.97254	3.388653	3.755901	4.236946	3.959072	3.96107	4.695525

MPI	8.537768	7.609355	6.584655	6.597281	9.044765	7.700989	7.296726	8.130733
MPIG6B	0.068854	0.034879	0.011333	0.04704	0.08034	0.046216	0.011648	0.128029
MPL	0.037926	0	0.012484	0.012955	0.012644	0.012728	0	0.025644
MPLKIP	7.696563	8.945909	9.135238	8.280633	7.222174	7.252167	8.238796	6.378404
MPND	8.094905	6.376736	6.943977	9.209321	9.536757	8.202008	6.875953	6.366339
MPO	0	0.038911	0	0.019679	0.019206	0.019335	0.009746	0.009738
MPP1	29.62588	36.2573	36.36039	34.78542	31.1841	33.23484	34.64921	34.62312
MPP2	2.555559	1.217309	1.37931	1.593818	1.561593	1.823833	1.615766	1.422783
MPP3	0.069329	0.020068	0.058685	0.040598	0.069338	0.029915	0.040211	0.040181
MPP4	0.234788	0.075117	0.085423	0.15196	0.086512	0.248833	0.163054	0.075199
MPP5	10.44715	9.820501	9.30851	10.1142	10.01537	10.06634	10.22398	10.79109
MPP6	1.318467	1.866609	1.49819	1.800331	1.486464	1.296442	1.567657	1.754037
MPP7	9.825796	10.92233	9.055317	7.811574	10.70605	8.730784	10.35974	9.490268
MPPE1	11.38604	9.570164	8.031545	11.58164	8.278537	9.741214	8.780742	9.177397
MPPED1	1.684678	0.452497	0.479761	0.682533	0.634775	0.93882	0.564681	0.866251
MPPED2	0.179226	0.494297	0.403151	0.459164	0.806622	1.022554	0.485106	0.32316
MPRIP	25.97616	23.50585	22.05996	24.21087	24.70405	23.95696	23.51267	24.71018
MPST	10.30605	10.58524	10.83599	12.21889	12.17648	12.43414	12.21332	10.53035
MPV17	11.36949	12.14765	10.96508	10.34241	9.86311	11.82218	10.33382	10.54191
MPV17L	2.08366	2.537267	3.06676	1.827301	2.083929	2.703057	3.09103	2.418128
MPV17L2	5.821543	6.335783	6.251102	5.939693	6.381643	6.501194	7.405435	4.82151
MPZ	8.828893	10.97437	7.969936	17.47929	8.558121	11.88589	9.426095	15.26777
MPZL1	21.06838	26.28163	22.30141	26.83302	23.28416	22.99778	24.33123	25.08487
MPZL2	38.95742	32.43274	37.37502	33.07447	32.60284	37.53274	34.3305	43.06932
MPZL3	13.13923	13.03083	15.37664	15.55866	14.63799	15.03113	15.21739	14.92478
MRAP	0	0	0	0	0	0	0	0
MRAP2	0.07614	0.123423	0.150383	0.109237	0.03046	0.045996	0.077282	0.03089
MRAS	2.49697	2.92536	2.661388	2.772995	3.074861	2.724453	2.266468	1.785033
MRC1	10.7971	10.44542	9.643771	7.761601	10.70878	5.070919	11.03711	19.19803
MRC2	13.17668	12.03295	11.44664	13.31282	13.45942	12.7716	14.8666	15.17602
MRCL3	370.6705	406.4983	394.6617	376.3774	367.7437	376.7665	397.7562	385.812
MRE11	6.995008	7.808444	6.258547	6.438348	7.297303	7.171514	5.894984	7.724393
MREG	28.44145	21.14216	32.87558	30.42864	26.57263	35.68879	28.409	26.16866
MRGBP	6.437342	6.252884	6.144135	5.882711	6.703666	6.731885	6.449702	5.165973
MRGPRD	0	0	0.086376	0.11951	0	0	0	0
MRGPRF	2.193925	2.977095	2.993831	4.196769	4.29533	3.212964	2.928624	2.683674
MRI1	4.707537	6.864626	4.162596	7.85185	7.347859	4.283575	5.997678	4.654689
MRLN	0	0	0	0	0	0	0	0
MRM1	4.821035	6.012512	3.781949	3.882779	5.364944	3.828484	4.948487	4.889666
MRM2	9.692746	9.53623	9.187864	9.900951	10.19192	8.913081	9.790762	9.041745
MRM3	5.903201	6.358402	5.899778	6.104007	7.35998	5.854153	5.532232	6.473419
MRNIP	5.180885	7.94651	5.442471	8.059011	5.759577	8.271231	7.416509	7.641207
MRO	0.01023	0.062185	0.020205	0.020967	0.040925	0.061799	0.020767	0.062254
MROH1	12.07163	10.99508	8.622466	11.32967	10.64614	9.371259	10.90994	11.28029
MROH2B	0	0	0	0	0.005843	0.011764	0.00593	0
MROH5	0.182899	0.247066	0.207987	0.295344	0.249441	0.223211	0.281277	0.202367
MROH6	0.557057	0.810529	0.58523	0.789481	0.800132	0.686162	0.649624	0.709239
MROH7	0.245519	0.210473	0.167857	0.290308	0.169997	0.139444	0.153355	0.204319
MROH8	0	0.008937	0.0784	0	0	0	0	0
MROH9	0	0	0	0.010964	0	0	0	0
MRPL1	12.64542	16.85537	16.20898	17.71997	16.67112	19.27454	12.54339	11.23845
MRPL10	27.82686	34.05703	28.1747	31.28828	28.76836	26.36463	29.36085	28.51565
MRPL11	26.12096	31.66016	31.53154	30.77099	30.60861	34.60988	32.33937	28.0431
MRPL12	21.77183	23.1864	22.49085	23.6813	22.3764	25.15143	23.97575	22.26257
MRPL13	33.62568	27.29278	39.67341	31.37233	35.50911	37.19364	27.98834	18.1591
MRPL14	41.55548	40.8132	47.23155	39.32867	43.8268	46.54074	40.38397	42.06298
MRPL15	35.77874	32.07735	34.93807	37.39344	31.36969	37.11161	27.80775	26.78048
MRPL16	20.85841	24.47389	22.59434	28.194	29.17504	28.25716	28.66058	29.02575

MRPL17	44.10909	35.14748	41.68665	32.30128	47.58119	29.23385	45.60043	41.05591
MRPL18	15.37802	13.27827	17.19976	10.92381	20.50668	19.47114	14.39665	10.95921
MRPL2	11.37815	15.22496	14.68149	13.72248	14.73446	14.29283	12.63833	13.85359
MRPL20	51.05013	57.62771	58.21577	53.93383	56.66281	62.30741	51.2813	48.68627
MRPL21	6.427619	6.326852	7.960949	7.644252	7.804439	7.131669	7.767746	7.587471
MRPL22	29.08452	35.36531	35.61087	35.68323	33.8162	40.28255	31.74435	31.25268
MRPL23	59.84525	65.59305	63.02742	64.30911	59.37417	61.8485	69.04135	54.42203
MRPL24	19.17597	19.85936	17.86585	18.2218	18.94598	21.6082	17.71385	16.91469
MRPL27	26.43199	32.40164	34.42237	29.45988	24.11932	29.22558	27.27426	19.6
MRPL28	14.63932	10.0362	11.985	12.03264	13.74605	9.393458	11.07104	11.81663
MRPL3	18.43027	20.55958	26.93955	19.23907	18.7321	23.94885	19.80976	16.11705
MRPL30	28.91719	37.49526	41.31399	33.13319	30.44308	43.01805	32.66658	34.7127
MRPL32	13.71771	16.54979	20.15861	14.03171	16.26199	18.11957	16.86146	16.28713
MRPL33	38.36353	45.16237	60.99034	51.10795	52.68912	63.81386	49.08505	48.50006
MRPL34	21.9515	21.49017	25.40944	22.70012	24.95357	23.08778	20.25156	20.60129
MRPL35	16.06513	17.10911	12.1525	14.83783	15.68536	14.01626	12.96738	16.29383
MRPL36	4.87962	3.359977	7.155217	4.719077	3.739413	6.593144	4.502559	4.520593
MRPL37	36.14334	39.68019	35.37973	31.85666	31.78114	31.93776	29.90521	40.89708
MRPL38	19.40246	19.85347	16.00235	17.93647	17.95105	17.34927	20.00828	20.93684
MRPL39	32.7289	40.16791	38.95391	36.348	35.85592	38.06386	34.83865	33.491
MRPL4	23.84262	25.92683	22.88852	26.70054	23.13389	27.1055	23.87047	23.38172
MRPL40	26.38103	31.22706	26.39263	33.3517	28.4906	33.92439	28.60332	25.90224
MRPL41	19.70234	27.54264	23.50688	23.00663	21.90372	25.8391	24.54675	18.3962
MRPL42	44.94828	42.4844	42.491	39.17108	46.84236	43.08762	44.9501	48.37652
MRPL43	15.47484	15.91369	15.26925	14.63932	19.57555	16.45265	14.53911	14.98708
MRPL44	9.802112	9.947848	11.57932	7.728187	12.19939	10.02195	9.281301	10.89988
MRPL45	31.49202	36.58022	35.50679	33.85981	35.42047	35.47132	33.12674	34.00706
MRPL46	10.36356	11.20848	10.36516	11.43423	10.17575	12.54788	11.07567	9.3986
MRPL47	16.63028	17.40718	18.44872	15.05321	16.58832	17.65437	16.7678	16.82229
MRPL48	19.54709	20.34894	21.14995	23.22467	20.93792	22.21914	23.72209	21.08958
MRPL49	41.89613	44.14059	47.79259	48.85977	46.37848	45.14955	41.1017	43.68816
MRPL50	17.80013	16.04381	23.18522	16.00809	21.57777	17.64363	19.46889	20.26352
MRPL51	9.859616	10.41729	11.44775	10.77499	10.35513	11.1019	8.684054	8.888866
MRPL52	20.55824	24.36262	23.14398	19.28997	19.65409	21.96878	18.926	18.7918
MRPL53	20.06417	22.73501	23.65215	21.91671	22.89999	22.22843	20.61465	22.05946
MRPL54	22.89341	25.88906	30.63757	25.18835	26.71868	27.9163	25.70882	24.01736
MRPL55	10.43907	14.74829	13.43007	14.20629	11.08696	11.28179	13.70631	11.58331
MRPL57	12.53681	11.63059	13.42432	15.27491	16.32912	16.54863	12.28097	12.75225
MRPL58	27.35377	31.89703	27.07568	31.77535	34.47465	28.66018	30.79523	28.64538
MRPL9	22.13165	25.06792	24.84286	25.23513	24.83493	22.53539	23.19744	26.74152
MRPS10	37.31428	40.14137	40.30098	36.0442	39.40544	42.4792	38.1264	37.77542
MRPS11	15.36775	11.66227	16.56117	15.05979	18.29173	14.91383	15.47964	16.65327
MRPS12	17.55708	18.57745	22.09109	16.28371	19.33411	18.24551	15.80097	14.28925
MRPS14	8.150344	7.937258	9.067886	7.920591	8.383091	7.993981	8.99939	6.835241
MRPS15	44.81986	56.02213	41.78202	44.37365	42.71767	52.08532	51.78429	37.66152
MRPS16	25.04294	30.09096	27.1686	27.13206	27.99278	32.71008	27.23708	25.4802
MRPS18A	8.028088	8.526606	7.820273	7.468874	8.963027	8.095142	8.776265	8.892652
MRPS18B	13.95574	15.08324	16.92688	14.85863	13.82807	14.78101	16.37263	15.38866
MRPS18C	27.3445	30.80571	34.53726	26.70057	28.15397	37.32142	29.00881	29.96772
MRPS2	21.23172	21.65828	19.50482	25.28417	20.69213	22.00673	19.8355	19.1225
MRPS21	54.65042	59.65182	44.99366	52.50305	50.38959	49.19559	43.6669	39.98586
MRPS22	23.60262	27.27072	28.45264	26.94568	24.77723	28.10509	27.27062	24.67169
MRPS23	36.26929	43.24365	43.41849	41.8286	35.02014	43.70893	38.9736	38.35284
MRPS24	22.09671	22.88419	23.27614	21.49388	23.99381	23.66021	21.11095	20.4903
MRPS25	18.35774	13.51989	16.9372	13.43747	15.83008	19.60475	13.30936	15.15889
MRPS26	0.14796	0.124918	0	0.176896	0.123316	0.099314	0.12515	0.100044
MRPS27	13.69355	19.41479	16.30595	14.64972	15.52735	15.77327	16.82437	14.51211
MRPS28	18.00242	19.83623	21.11961	20.33358	18.23474	20.53992	19.1394	15.62651

MRPS30	10.68867	11.59677	11.32973	12.07824	11.5003	11.7484	11.75088	12.44443
MRPS31	5.487875	5.620662	5.340523	5.439425	5.858489	5.887685	5.905014	5.1706
MRPS33	70.97674	76.12582	82.45474	84.89768	80.87788	83.82769	77.97492	83.8752
MRPS34	27.07764	23.57609	25.68123	24.45153	26.89345	27.88345	24.4361	23.33005
MRPS36	17.80751	21.45157	20.60206	19.95209	22.00769	19.01704	17.65281	15.80602
MRPS5	18.03486	20.19955	21.09172	19.29166	12.83979	17.49482	21.91594	20.04355
MRPS6	41.60889	56.4598	40.78195	47.37729	53.73615	50.76241	39.82333	38.0508
MRPS7	20.6131	20.45949	21.45873	20.32696	18.60204	21.83778	20.76042	19.61252
MRPS9	12.76206	15.26888	16.26994	13.36597	17.88168	13.36761	13.01683	15.00081
MRRF	6.140604	5.490736	5.832088	4.881415	6.627524	6.231492	5.591352	5.537843
MRS2	16.32857	12.32643	14.10716	15.69563	11.08087	13.20306	16.49739	13.94662
MRTFA	4.133582	3.545439	3.065835	4.12235	4.186096	3.572258	3.731363	4.441933
MRTFB	8.748307	8.303343	7.299273	8.557	8.343117	7.109177	8.192261	9.499163
MRTO4	20.2622	18.1779	24.29158	20.93699	22.20525	22.422	19.53012	18.69407
MRVI1	2.553622	2.383132	2.115061	2.635635	3.85839	2.50656	2.37361	2.784919
MS4A1	0.098653	0.414068	0.097423	0.144423	0.155046	0.184464	0.40053	0.214408
MS4A10	0	0	0	0	0	0	0	0
MS4A13	0.825702	0.836538	0.480885	0.759369	0.465841	1.428204	1.224896	1.030715
MS4A14	0.014904	0.030199	0	0.122183	0.119245	0.060022	0.045382	0
MS4A15	0	0	0.016661	0.017289	0	0	0.017124	0
MS4A2	0.865644	1.578609	1.125563	2.335995	1.067766	0.97324	1.830477	4.009384
MS4A3	0	0	0	0	0	0	0	0
MS4A5	0	0	0	0	0	0	0	0.046159
MS4A7	7.160019	6.090246	7.127521	10.08399	11.29668	8.67385	7.966957	3.980479
MS4A8	1.23827	0.562371	2.698683	1.42209	2.306042	1.139252	1.841926	3.421237
MSANTD1	0.512387	0.281186	0.274085	0.404749	0.373664	0.225701	0.346716	0.259841
MSANTD2	8.687912	8.040684	7.014451	7.20671	8.231099	8.569954	8.055573	8.632977
MSANTD3	0.924015	1.203611	0.991647	1.31407	1.093873	1.048992	0.93309	1.147554
MSC	0.385526	0.729093	0.609155	0.842827	1.105316	0.543423	0.782617	1.016636
MSH2	21.21191	24.69155	22.78161	22.27852	20.84494	22.93422	23.05775	24.12947
MSH3	4.863117	5.933596	5.265499	5.786687	5.248647	5.234504	4.971575	5.535584
MSH4	0.103894	0.12631	0.07182	0.127762	0.051954	0.083683	0.184543	0.105373
MSH5	0.40513	0.339064	0.30441	0.451266	0.352332	0.345826	0.277117	0.285841
MSH6	16.68014	16.35155	14.85176	14.54722	14.84336	16.06421	15.17109	17.33202
MSI1	2.248947	2.402742	1.473886	1.718015	1.513123	1.770279	1.867648	1.721089
MSI2	3.88088	4.650609	3.569782	4.009729	3.487567	3.710952	4.165934	4.34629
MSL1	22.65299	20.87617	19.15868	19.13613	20.88706	20.30133	18.03151	20.46291
MSL2	10.24965	10.05367	9.110384	9.754713	10.70773	9.334338	10.3173	11.21608
MSL3	17.38012	19.13615	18.44749	18.34356	18.04812	17.48838	18.34814	19.25209
MSLN	0.411894	0.576271	0.697305	1.065291	0.882744	0.533196	0.756512	0.875301
MSMB	2.713855	0.861775	5.640085	1.535819	3.645949	3.303343	4.399078	1.437867
MSMO1	64.51115	76.17989	90.1296	93.93149	83.12623	89.6881	75.74013	48.8174
MSMP	0.38254	0.129187	0.188887	0.196008	0.25506	0.192577	0.129426	0.129329
MSN	75.77742	69.75201	63.70787	71.30052	75.09353	64.10594	73.92029	78.84578
MSR1	1.792307	1.382986	1.276028	1.43092	1.271452	0.59802	1.713425	2.938107
MSRA	2.427533	3.929148	4.832775	4.232002	4.55584	4.576633	4.329084	3.178157
MSRB1	36.61333	36.93433	41.35697	38.16561	46.11811	46.11014	37.37559	36.26517
MSRB2	14.91194	18.84501	14.57193	12.7411	14.77722	17.13618	18.08746	14.41156
MSRB3	19.08676	26.60315	29.19475	29.8575	23.49827	26.53114	27.38975	24.37867
MST1	1.380185	1.171548	1.03145	1.669215	1.778306	2.290985	1.325164	0.731444
MST1R	1.508547	1.547292	1.083455	1.232899	1.165846	1.261527	1.202163	1.384607
MSTN	0.019343	0.039193	0	0.059466	0.009673	0.048688	0.02945	0.058855
MSTO1	6.047763	7.103923	4.818319	7.470016	7.728696	5.986123	5.856762	6.133857
MSX1	64.72958	64.88783	65.75401	55.55613	46.74364	55.12123	59.36232	49.14802
MSX2	91.41415	99.10235	100.5676	95.21093	87.48311	103.9009	92.00347	85.33134
MT3	15.40818	178.0158	122.5671	82.57073	125.402	25.4762	72.74426	167.0281
MT4	141.444	112.2531	112.9155	122.2669	127.0232	111.9673	157.0444	106.6435
MTA1	17.03734	17.26094	14.43778	16.58693	17.34438	16.28599	15.87405	17.8342

MTA2	37.50854	38.19007	34.15637	39.94312	37.58133	36.76444	41.38095	38.20614
MTA3	5.527743	5.860658	4.725173	5.731705	6.617326	5.269118	5.601174	5.848132
MTAP	12.08054	12.50489	10.57567	10.27778	10.13799	10.8423	9.974017	10.42613
MTBP	10.24342	9.0134	9.427314	8.573078	7.463686	8.27943	7.550539	9.079535
MTCH1	139.3149	127.9529	130.6123	139.1658	143.0471	138.7797	134.9282	118.7568
MTCH2	40.26966	45.6423	41.3604	42.75791	41.94406	45.06362	42.29301	37.86921
MTCL1	5.911036	4.865175	4.541181	5.454492	4.033242	4.903312	4.892559	4.943961
MTCP1	3.990423	3.245623	1.998102	2.534194	2.360837	1.7542	2.110709	3.591203
MTDH	37.20154	39.91198	40.53535	42.39655	40.95461	45.66769	40.37069	39.75803
MTERF1	3.836125	4.706128	4.057638	4.294452	4.49121	4.237581	4.087427	4.03825
MTERF2	2.43384	2.460327	2.302482	2.709329	2.70342	2.591423	2.738151	2.331956
MTERF3	10.54483	9.288376	9.81088	8.657648	9.99854	11.86128	10.3854	11.67875
MTERF4	20.39339	24.18052	21.97013	23.66534	21.29612	21.3301	22.28873	18.78511
MTF1	5.186184	4.192934	4.214544	4.490613	5.094627	4.400862	4.713618	4.440708
MTF2	4.915353	5.288733	4.59001	5.075476	5.065768	5.406668	4.701356	5.430154
MTFMT	8.688998	9.47866	9.646059	11.27601	11.25989	10.44668	9.536028	8.772905
MTFP1	0.277808	0.234545	0.228622	0.308414	0.331869	0.411789	0.281975	0.258283
MTFR1	30.87951	28.61546	32.17434	29.94179	34.65914	32.76327	30.75397	27.23097
MTFR1L	9.264661	10.83637	10.07442	8.907432	10.20286	8.437081	10.30173	10.51833
MTFR2	2.751117	2.152296	2.391656	2.340315	2.167183	2.930331	2.199407	2.305483
MTG1	5.250255	4.800217	3.847979	5.64276	6.147435	5.654493	5.087625	4.527174
MTG2	4.218955	4.378576	3.31648	4.150905	4.631159	3.597895	3.65557	3.804623
MTHFD1	14.27017	12.68278	12.0046	11.44358	12.97319	10.6047	13.80983	13.80708
MTHFD1L	2.825585	3.493098	3.229313	3.630857	3.619756	3.222075	3.499566	3.30373
MTHFD2	5.500166	4.543809	4.226014	5.715422	5.642255	6.637526	5.595713	4.887674
MTHFD2L	1.071831	1.097093	0.905706	1.03044	0.939355	0.956775	1.031831	1.131918
MTHFR	1.531889	2.172259	1.847538	2.345327	1.984092	1.862466	2.133609	2.081367
MTHFSD	3.566972	3.500188	2.99657	3.490155	3.174945	2.822271	3.357298	3.489814
MTIF2	4.303917	4.915866	4.223217	4.888099	4.752284	3.450095	3.793432	4.161292
MTIF3	17.07115	16.27012	15.94058	15.45646	15.63783	18.15911	14.05523	14.92766
MTLN	9.470018	12.6858	9.040286	11.75334	11.41811	9.746595	12.49569	10.35187
MTM1	4.444281	4.734359	4.090394	4.160883	4.38766	3.923537	3.963661	4.474404
MTMR1	4.854371	4.808789	4.834193	4.487611	4.808344	4.834695	4.873919	4.852507
MTMR10	10.16889	10.75002	10.16276	11.90436	9.402644	11.50864	10.80534	10.0306
MTMR11	1.672129	2.079091	1.95153	1.888798	2.527522	1.740946	1.446487	1.599573
MTMR12	7.748242	8.223444	7.508452	8.002104	8.426245	8.044581	8.864394	7.975012
MTMR14	10.62239	9.702704	9.235673	9.572341	10.06166	9.099205	10.56495	11.07003
MTMR2	9.46159	11.52296	9.742514	10.26184	9.042427	9.941133	11.87891	9.387304
MTMR3	10.63609	10.6134	9.591523	10.81224	10.79765	10.53376	10.9755	10.89465
MTMR4	8.948842	8.706134	8.722707	9.46789	9.947668	8.96615	9.248758	8.296275
MTMR6	15.67566	15.90322	14.11818	14.25289	16.12315	14.07572	15.40036	14.27395
MTMR7	0.132771	0.18625	0.126074	0.245954	0.209397	0.226224	0.217694	0.222709
MTMR8	1.40969	1.174802	1.392125	1.328108	1.580421	1.041598	1.038509	0.957015
MTMR9	3.967396	4.227002	4.074845	3.928577	3.942822	3.855597	3.891121	4.392765
MTNR1A	0	0.046524	0	0.02353	0.022964	0.023118	0.023305	0
MTNR1B	0.015489	0	0.045889	0.126984	0.030983	0	0.015722	0
MTO1	3.661285	3.816631	2.801393	2.798478	3.782808	2.962747	3.232484	3.613666
MTOR	13.8145	11.94009	11.46391	11.89611	11.3592	11.05005	13.2645	12.4261
MTPAP	9.992763	9.971094	9.942729	9.486757	9.588636	9.159333	9.434583	10.11589
MTPN	27.50595	26.90289	29.96764	26.17608	29.48722	26.62353	26.74898	30.35551
MTR	1.920798	2.199954	1.859197	2.186155	2.359754	1.772074	2.065757	2.627919
MTREX	21.23002	23.08633	22.51152	21.02589	22.56654	21.81706	22.29674	21.255
MTRF1	9.280553	8.219274	10.96148	10.56855	9.023585	10.81671	8.159634	7.829345
MTRF1L	10.84724	10.83974	10.32255	9.869598	10.02677	12.12931	12.77821	10.10152
MTRR	8.40446	7.561732	6.921525	7.71187	7.356781	7.010159	6.245285	7.648232
MTSS1	32.82295	33.34399	33.06291	32.36907	31.95276	28.43268	31.96437	31.4602
MTSS2	12.16581	10.74607	10.001	12.28283	9.990569	10.5284	8.800218	12.04102
MTTP	0	0.027318	0	0.013816	0.006742	0.027149	0.013684	0.006837

MTURN	22.2008	23.61408	23.41412	21.90829	21.45888	21.73414	22.92648	17.27877
MTUS1	10.4635	11.30568	12.17073	11.10179	10.64156	11.62461	11.54519	11.46369
MTUS2	0.191408	0.4865	0.490796	0.419827	0.26196	0.277239	0.405599	0.589209
MTX1	10.28099	9.730169	8.7257	9.879516	7.995324	9.946014	9.543939	8.070987
MTX2	23.28666	25.27743	24.86218	25.08401	24.00848	26.58838	23.74017	25.49261
MTX3	1.320184	1.964668	1.016788	1.061594	1.149783	1.297404	1.679792	1.377417
MUC1	0.160626	0.056957	0.111038	0.074072	0.064259	0.097034	0.171188	0.081457
MUC13	0.255479	0.129416	0.126148	0.072724	0.070976	0.185773	0.144062	0.028791
MUC15	17.69111	16.29465	19.4047	19.27495	18.9747	21.95276	18.69362	14.02
MUC20	0.294365	0.441129	0.272528	0.207389	0.680806	0.586577	0.342352	0.522471
MUC3A	0.003605	0	0	0	0	0	0	0
MUC4	0.011234	0.011381	0.016641	0.005756	0.011235	0	0	0
MUC6	0	0	0	0	0	0.008225	0	0
MUCL3	0	0	0	0	0	0	0	0
MUL1	8.624705	8.666468	6.440147	8.970796	6.816043	7.29945	8.08619	8.068183
MUS81	4.377984	4.151118	3.8061	4.313874	4.958615	4.238358	4.946888	4.64904
MUSTN1	38.12144	66.23291	46.46364	44.71997	58.72799	49.08839	52.94868	48.09275
MUTYH	3.203741	3.272948	2.462221	3.214414	2.895805	3.374087	2.898045	3.684407
MVB12A	17.60164	16.13524	19.07578	16.64391	19.75265	19.90487	18.6259	19.49149
MVB12B	1.586981	2.16562	1.10664	1.818791	1.483533	1.441301	1.663383	2.043172
MVD	29.05498	28.1687	30.64547	40.44354	36.45859	33.37477	30.54125	21.21237
MVK	13.74014	15.05682	13.79457	16.38998	12.14232	15.38157	15.73827	11.49198
MVP	20.08148	15.26364	14.22347	18.60963	20.87206	16.97788	19.2124	16.23541
MX1	6.51551	6.821829	4.315983	6.218453	5.98862	7.818899	8.441199	5.910207
MX2	0.362608	0.905108	0.285434	0.549307	0.683264	0.756627	0.661419	1.018031
MXD1	16.3219	12.8513	15.12575	11.71337	14.90987	14.71354	14.555	15.56365
MXD3	1.725094	1.944109	2.143855	2.224679	1.822245	1.756394	2.00673	1.59238
MXD4	13.96494	14.47425	12.21029	12.74005	12.93343	14.61457	13.83059	14.69628
MXI1	10.24955	12.03297	11.29531	10.07638	10.26214	10.34792	9.751661	9.635794
MXRA5	38.39169	25.22573	26.04857	26.90483	29.90225	23.43238	26.50436	33.42778
MXRA7	18.31959	20.01924	23.63699	20.06695	20.70932	21.94794	20.74621	19.57342
MXRA8	11.12797	8.493016	8.701612	11.07299	9.304479	9.81414	10.40375	8.448693
MYADM	20.33963	17.76246	28.80498	20.64025	23.92224	16.70649	20.21043	23.67751
MYB	4.606317	4.577802	3.484616	4.123207	2.754566	2.869479	4.221646	4.258951
MYBBP1A	19.60074	18.76178	16.56194	16.93045	20.22163	18.09458	18.22328	18.97532
MYBL1	1.366005	1.632475	1.32696	1.331278	1.477707	1.049747	1.482701	1.605992
MYBL2	12.94538	11.90952	8.433368	12.01433	9.553673	11.0367	12.7157	11.53081
MYBPC1	0.095935	0.115418	0.28422	0.221202	0.419772	0.07848	0.365154	0.352717
MYBPC2	0.530385	0.145851	0.202028	0.124234	0.591077	0.648438	0.446054	0.537935
MYBPC3	0	0	0.026274	0.006816	0	0	0	0
MYBPH	0.708403	0.278292	0.442589	0.933365	1.055513	0.902471	0.601637	1.129053
MYBPHL	0.048091	0.048722	0.047492	0.13142	0.048098	0.04842	0.048813	0.032517
MYC	41.02005	44.31763	39.77792	39.87492	48.68006	43.97207	49.00695	46.29277
MYCBP	44.75846	51.73878	52.27607	46.09745	46.2512	55.21195	46.13173	39.34638
MYCBP2	12.09369	11.59093	9.794994	10.75879	12.38817	9.584407	10.91372	12.86373
MYCBPAP	1.321297	1.291503	0.790251	1.973827	1.50759	1.330323	0.972782	1.330669
MYCL	2.771523	3.575171	2.657426	3.665807	2.586552	2.66878	3.573614	2.884521
MYCN	14.3195	16.22386	11.895	15.62552	11.97701	13.73968	13.98072	13.55826
MYCT1	8.49743	8.296144	9.544891	9.271934	12.48599	12.29935	9.755801	8.877084
MYD88	9.655301	8.43557	7.321231	8.220775	8.791784	7.835047	8.890002	9.66283
MYDGF	10.12062	10.37582	10.12232	10.07953	10.95032	9.477431	10.06225	10.64098
MYEF2	5.033376	5.10854	4.322698	5.240952	5.573386	4.714828	4.169208	5.569951
MYF5	0	0	0	0	0	0	0	0
MYH1	0.009029	0.013721	0	0.009252	0	0.318166	0	0.009157
MYH10	22.14148	22.4573	16.64259	16.67916	19.4426	16.34777	22.05455	20.28718
MYH11	50.01953	74.34034	76.64384	74.48687	61.7441	57.59606	74.19323	51.84858
MYH14	41.98988	34.71733	32.56019	33.67036	35.3888	35.12766	40.15371	38.27743
MYH15	0.025912	0.007501	0.014623	0.041728	0.059236	0.033544	0.026301	0.041299

MYH2	0.027671	0.018689	0.122968	0.018904	0.013837	0.018573	0.014043	0.028065
MYH7B	3.551698	3.068747	3.608916	4.335562	3.471731	3.46351	3.106172	2.881807
MYH9	141.0477	117.1698	105.8096	101.149	122.0603	101.6496	119.9023	121.065
MYL1	0	0	0	0	0	0.25476	0	0
MYL10	3.259291	0.99062	1.327706	1.252509	2.03732	1.025487	3.308181	2.355304
MYL2	0	0	0.035757	0	0	0.036456	0	0
MYL3	0.208208	0.723226	0.323108	0.73154	0.624706	0.419262	0.513234	0.754187
MYL4	0.028045	0	0.055392	0.05748	0.084147	0	0.028466	0.113778
MYL6	1145.723	1105.653	1186.867	1099.507	1096.337	1109.789	1208.313	1123.777
MYL6B	16.51645	6.909196	8.558707	8.699377	14.56477	7.152379	7.246459	7.709325
MYL9	99.06575	123.3329	133.7116	123.7101	110.3375	117.922	129.5657	95.88653
MYLIP	38.22394	39.92848	45.10087	43.3491	49.83654	47.00127	41.18885	41.75527
MYLK	31.96898	35.36006	36.93095	36.23265	37.4051	34.54781	39.24781	35.61595
MYLK2	0.240044	0.243195	0.237053	0.295188	0.230472	0.251352	0.389832	0.146077
MYLK3	0.150971	0.171492	0.144572	0.107829	0.169293	0.092123	0.116088	0.255201
MYLPF	0	0	0.037002	0	0.037474	1.131742	0.038031	0
MYMK	0.022187	0.078674	0.016433	0.039789	0.016643	0.005585	0.03941	0.016877
MYMX	0	0.008653	0	0	0	0	0	0
MYNN	8.659333	9.499507	8.242605	7.575354	9.345308	8.615344	8.400658	8.777136
MYO10	15.05954	11.71262	9.199679	9.70037	10.90587	11.29535	11.49686	11.21197
MYO15A	0.00508	0.00772	0.007525	0.007808	0.00254	0.005114	0	0.007728
MYO15B	0.046201	0.017553	0.028516	0.041427	0.063535	0.026166	0.020516	0.032215
MYO16	0.013995	0.047261	0.02764	0.009561	0.009331	0.051664	0.037879	0.018925
MYO18A	19.34952	16.46165	15.12553	17.33972	16.71375	16.28263	17.16007	17.04116
MYO18B	0.079761	0.105401	0.14726	0.135043	0.093645	0.087289	0.066878	0.024621
MYO19	5.171019	4.922887	4.301421	5.130881	5.25925	4.457072	5.404098	4.495398
MYO1A	0	0	0	0.007473	0	0	0	0
MYO1B	25.0357	21.63652	20.77512	21.93597	23.34474	22.34938	22.83215	24.22625
MYO1C	38.34856	33.24124	32.85417	36.84908	39.51646	34.1996	35.48443	38.25716
MYO1D	12.07394	9.579188	11.28739	10.66257	10.11464	9.538628	10.25549	10.72463
MYO1E	20.70743	16.65352	18.75811	19.46529	22.60264	19.60085	18.87248	21.42736
MYO1F	1.3286	1.465684	1.297467	1.686759	1.882426	1.010689	1.685661	3.278948
MYO1G	0.735191	1.034013	0.418535	0.850901	1.02075	0.496379	0.798893	1.184279
MYO1H	0.008954	0	0	0.009175	0	0	0.009088	0.009081
MYO3A	0	0.017298	0.039342	0.023329	0.017076	0.04011	0.011553	0.05195
MYO3B	0.01933	0.078333	0.019089	0.044569	0.038664	0.072981	0.068668	0.039209
MYO5A	8.378892	8.21986	7.304569	7.57273	7.693785	7.425973	8.194538	7.766556
MYO5B	31.36012	28.5721	25.35386	25.09716	25.37484	26.67622	27.82147	28.71569
MYO5C	4.333249	4.25894	3.703858	3.945221	3.410069	4.015214	3.657905	4.1498
MYO6	12.33768	8.672429	14.02266	11.43097	12.16785	12.3103	12.21232	13.37913
MYO7A	0.041233	0.078326	0.058533	0.036972	0.14691	0.036325	0.091549	0.138527
MYO7B	0.093896	0.169576	0.096758	0.230096	0.489955	0.534342	0.385361	0.120076
MYO9A	5.65644	5.476528	4.406506	5.211892	5.499534	5.172064	5.691715	5.583857
MYO9B	13.54409	11.81088	9.483534	11.39117	12.60811	10.42106	11.90338	12.83052
MYOC	36.35461	28.10274	34.65255	45.53261	17.55968	29.41602	29.12082	41.22871
MYOCD	0.399079	0.343971	0.182348	0.402861	0.768476	0.341835	0.205556	0.356431
MYOD1	0	0	0	0	0	0	0	0
MYOF	11.92671	8.850929	9.105856	9.816459	12.5546	13.68172	14.63627	13.67048
MYOM1	0.084238	0.193146	0.166377	0.236257	0.133024	0.290151	0.351006	0.215841
MYOM2	0	0	0	0	0.011393	0.011469	0.005781	0
MYOM3	0.149571	0.185752	0.071472	0.212609	0.284705	0.544079	0.142021	0.327869
MYORG	1.487858	1.009128	1.426285	1.575749	1.419563	1.579505	1.364845	1.597434
MYOT	0	0	0	0	0	0	0	0
MYOZ2	0	0	0	0	0	0	0	0
MYOZ3	0.703919	0.135839	0.331023	0.721355	0.335243	0.843724	0.272182	0.339971
MYPN	0.004544	0	0	0.002329	0	0	0	0
MYPOP	1.366424	1.46042	1.453198	1.323333	1.3666	1.602532	1.264993	1.385876
MYRF	0.364179	0.31117	0.155989	0.247301	0.043883	0.128113	0.080163	0.040051

MYRFL	0.447628	0.585774	0.74289	1.089449	0.553389	0.638471	0.523755	0.359417
MYRIP	1.483791	1.426926	1.654189	2.494644	2.336113	1.622312	1.841379	2.275006
MYSM1	7.6247	7.852866	6.356728	8.096269	7.349782	7.69222	6.615156	8.953463
MYT1	0.005389	0	0	0.005522	0	0	0	0.005465
MYT1L	0.010061	0.003398	0	0.006873	0.003354	0.006753	0.023827	0.023809
MYZAP	13.16403	10.43749	12.08206	10.57071	11.33656	9.280806	12.19525	9.514912
MZB1	0.019964	0.465192	0.059145	0.675122	0.319461	0.120601	0.526843	0.222727
MZF1	3.513284	3.013619	2.995342	3.504301	3.326339	3.407585	3.090519	3.90775
MZT1	6.976771	8.620596	7.65988	8.509412	8.811022	5.454266	7.400781	7.131589
MZT2B	11.61814	15.82155	15.64552	16.08074	15.05272	19.90096	13.09418	13.58167
N4BP1	17.341	14.30954	12.69993	14.93249	16.26372	13.94183	14.18828	15.53882
N4BP2	7.217996	6.73325	5.998375	6.893476	7.36194	6.203188	6.408117	7.427944
N4BP2L1	7.043316	7.165836	8.439329	8.361916	7.317443	8.412855	7.486523	7.258024
N4BP2L2	11.47613	12.03991	11.17063	11.17384	12.45793	10.71171	18.13325	16.90775
N4BP3	10.8656	10.38482	8.940271	9.057364	9.555137	9.09571	8.060836	9.051332
N6AMT1	1.431915	2.591931	1.979702	2.973898	1.126585	1.614701	1.879723	2.633503
NAA10	60.66673	62.57886	60.77544	65.38213	63.61226	68.04807	70.92217	66.68653
NAA11	0	0.041096	0	0	0	0	0	0
NAA15	19.2846	22.21224	21.08764	21.87743	22.76325	22.65924	20.87224	21.47162
NAA16	4.751784	5.475958	4.505289	4.750719	5.779801	5.553332	5.491445	5.813235
NAA20	74.92933	80.04802	89.43873	76.78535	74.19678	78.32329	71.50675	70.72708
NAA25	8.76448	8.324842	8.176817	8.763121	9.443991	8.775574	8.256658	9.031751
NAA30	9.408646	9.210179	9.482164	8.664761	10.72475	9.259624	9.056476	8.739996
NAA35	13.87021	17.65098	15.1418	15.21302	15.15571	16.47946	16.0375	15.79332
NAA38	18.66874	17.58498	20.91633	19.93188	19.4247	21.46541	20.25168	19.9534
NAA40	4.11958	4.824422	3.357786	4.572694	3.863141	4.27707	3.60763	4.117419
NAA50	27.66819	31.21816	31.21874	30.35634	31.34564	31.87887	28.12665	31.28135
NAA60	13.21211	14.22381	12.96755	13.88961	13.71754	13.90908	14.09495	15.32493
NAA80	1.722131	2.516088	1.521655	1.820519	1.577314	1.752148	1.379971	1.94889
NAAA	3.788702	8.769084	7.087546	7.154834	6.715803	5.88211	6.992825	7.612378
NAALAD2	1.472484	1.685865	1.666937	1.668441	1.987512	1.313798	1.555326	2.270523
NAALADL1	0.257253	0.651574	0.262516	0.465739	0.45454	0.32808	0.557039	0.521831
NAB1	9.806191	9.888913	9.728817	8.705184	9.258313	9.850338	9.382156	9.798511
NAB2	39.61509	34.59018	32.14258	35.81025	35.16099	33.93765	32.26701	32.12966
NABP1	7.312154	7.123191	9.3463	8.909232	8.022396	9.725861	7.545947	8.085942
NABP2	21.22743	18.51127	20.08719	18.72407	19.43789	19.28914	17.79547	17.96945
NACA	97.88452	80.37911	102.2765	94.08619	88.40172	72.27994	100.388	73.57896
NACAD	0.766389	0.566159	0.500618	0.609482	0.738543	0.534511	0.757627	0.639652
NACC1	14.12221	13.40242	12.66695	13.73305	14.54527	13.56333	13.75757	14.32325
NACC2	0.917391	0.947134	1.022441	1.078894	1.332574	0.932455	1.024267	1.289339
NADK	37.66474	37.14125	36.64246	39.75378	38.69905	37.93863	36.02318	34.60131
NADK2	2.835765	3.435675	3.343641	3.256262	3.247398	4.000419	2.975871	2.946548
NADSYN1	4.129549	2.73511	4.130783	4.538019	3.468416	3.266044	3.476664	3.712142
NAE1	11.4088	12.98746	12.87616	12.86375	11.26921	13.90084	13.60005	11.36458
NAF1	9.312827	10.8471	9.734832	9.984978	10.50521	9.580562	9.966943	9.740972
NAGA	14.38351	13.82498	10.86678	12.32095	12.36001	11.5044	13.86419	14.92832
NAGK	11.28289	12.4684	11.02995	11.71784	11.1516	13.09738	12.56848	9.808726
NAGLU	4.122955	4.471383	4.667423	5.862444	5.296838	4.432362	4.672459	4.940917
NAGPA	2.921625	3.125726	2.792894	2.910162	2.185658	2.741556	3.641571	3.899592
NAGS	0.026682	0.094612	0.013175	0.068357	0.026685	0.080592	0.040623	0.067654
NAIF1	2.922197	1.896601	2.227466	2.479887	2.511587	2.335339	2.391361	2.769297
NAIP	0.283027	0.291153	0.3139	0.160636	0.23516	0.214816	0.061874	0.260558
NALCN	0.016093	0.085595	0.007946	0.024737	0.056332	0.028355	0.049002	0.024483
NAMPT	8.366188	10.08217	11.32402	12.20766	9.742711	10.08365	9.822949	8.801708
NANOG	0	0	0	0	0	0	0	0
NANOS1	1.233365	1.066284	0.828238	0.75835	0.773009	0.943761	0.784502	1.150849
NANOS3	0.374124	0.157931	0.43104	0.423328	0.265039	0.306053	0.221513	0.126483
NANP	2.117854	2.122725	1.885894	2.518121	2.330846	2.264454	2.167995	2.170952

NANS	19.97296	27.21585	24.66068	24.0905	23.21847	23.46475	28.91182	24.16258
NAP1L1	46.49502	59.74251	50.73523	50.79779	49.45407	48.81032	52.25099	50.08007
NAP1L2	0	0	0.017752	0	0	0	0	0
NAP1L3	3.926278	3.334338	4.362025	3.239536	3.739108	3.299095	4.058429	4.772749
NAP1L4	49.32411	48.30572	46.90688	54.39321	50.5864	48.22728	52.71828	50.604
NAP1L5	1.197702	1.097857	1.661523	1.168924	0.926913	1.033615	1.157779	0.824296
NAPA	67.77198	68.84654	73.47265	69.97841	65.33864	74.67381	65.70319	57.53334
NAPB	4.726479	4.413948	2.247887	4.442402	4.922844	3.401205	3.759907	4.345267
NAPEPLD	2.159658	2.229187	2.398714	2.109005	2.541102	2.123249	2.878036	1.999708
NAPG	16.88626	17.49146	15.5822	20.50549	16.60444	19.63191	17.67757	15.99383
NAPRT	8.862961	6.12527	5.222233	6.989135	10.52819	10.25042	6.420869	6.549701
NAPSA	0.260607	0.281629	0.102944	0.231454	0.208512	0.13994	0.28215	0.211453
NARF	24.9351	23.69569	24.44306	25.58246	24.17471	22.85763	21.6599	26.34872
NARS	42.15236	42.96474	35.03569	45.74657	34.08677	44.74673	32.91762	30.94686
NARS2	5.648347	4.978088	5.872738	5.400021	5.476849	5.779404	5.127137	5.263002
NASP	20.87386	25.86954	21.95298	25.14663	19.25416	25.58788	24.49743	25.75864
NAT10	9.834396	9.018474	7.308924	9.288381	9.321914	8.28194	8.095296	9.131199
NAT14	2.893308	2.764966	3.222013	3.469653	3.406746	3.181655	2.145255	3.662917
NAT16	0.10215	0.165585	0.100877	0.062808	0.040865	0.226266	0.103682	0.041442
NAT8	0.024689	0.066702	0.032509	0.016867	0.032923	0.04143	0.016706	0.108509
NAT8L	0.061515	0	0	0.031519	0	0	0	0
NAT9	6.957118	7.594292	7.225154	7.613785	7.251086	6.205202	6.437839	6.193311
NATD1	18.5588	13.52057	14.67993	14.91364	13.5686	13.98379	14.95533	14.3214
NAV1	2.860934	2.682929	2.481066	2.876133	3.35026	2.666265	2.380052	2.768515
NAV2	11.58762	10.65502	9.180192	9.911483	9.929828	7.939677	9.001315	9.562446
NAV3	1.026254	0.776858	0.439245	0.579252	0.945295	0.499139	0.700702	0.831751
NAXD	16.94264	19.36113	17.70768	21.92058	18.35834	18.78692	18.48169	17.8258
NAXE	28.54086	30.83443	33.30652	34.2795	31.02748	33.04991	31.91813	30.86826
NBAS	5.044581	6.029404	4.625074	4.468716	4.818524	4.375018	5.287935	6.020433
NBDY	7.733381	8.58961	7.251668	6.398117	7.627945	6.178967	8.85756	7.951409
NBEA	1.267512	1.494663	1.000349	1.232367	1.340412	1.119265	1.138891	1.39093
NBEAL1	2.531326	2.253615	1.848539	2.048191	2.589998	1.652261	2.077577	2.742292
NBEAL2	5.743165	5.192125	3.946832	5.212618	5.377343	4.620785	4.693858	6.018871
NBN	12.96004	14.12108	15.3434	14.43761	13.74242	15.01817	13.3549	12.35281
NBR1	48.30957	47.05356	46.62145	44.88563	45.58703	44.96256	45.05596	47.94338
NCALD	2.719035	4.163206	2.980978	2.912325	3.694986	3.371746	3.968217	3.606878
NCAM1	2.226974	2.165819	2.319955	2.376944	2.171085	1.816372	2.595746	2.262028
NCAM2	0.049936	0.021682	0.024657	0.054828	0.046375	0.02873	0.086889	0.097676
NCAN	0.008601	0.017429	0	0.013222	0	0	0	0.008724
NCAPD2	13.88763	11.81611	11.58109	11.31626	9.878233	9.739835	10.04139	12.40941
NCAPD3	3.773486	4.139085	3.117616	3.897405	2.919629	3.141124	4.553885	4.05322
NCAPG	8.578722	9.736022	8.249337	7.592417	7.851846	8.166201	9.806823	9.975212
NCAPG2	7.312743	7.888102	7.264105	7.280821	6.195124	7.261643	7.444265	7.358673
NCAPH2	10.46255	10.0661	8.47825	11.29961	9.328454	10.31736	10.61195	9.339613
NCBP1	27.82633	29.01637	24.62087	26.27217	26.78947	25.17471	28.32636	27.13063
NCBP2	27.77723	33.58119	34.51912	32.08498	31.45479	34.51064	31.75039	34.23422
NCBP2-AS	11.41663	12.48228	12.39851	13.17472	13.32671	15.74191	13.93264	11.34146
NCBP2L	0.174776	0.252957	0.049314	0.102346	0	0.075416	0.050685	0.202588
NCBP3	13.87218	13.99223	12.7021	12.5329	13.11907	14.3572	12.46517	14.1938
NCCRP1	35.54216	41.52365	38.66074	45.60373	37.86058	60.08311	32.62201	26.66227
NCDN	4.812195	3.824861	3.511665	4.236641	3.676089	3.7208	4.351414	3.977366
NCEH1	0.717063	1.308854	0.561821	0.856284	0.811986	0.698095	0.902255	0.991733
NCF1	1.444868	1.589302	2.201449	2.834403	2.353376	1.620995	1.843652	3.684527
NCF2	0.241807	0.359815	0.38804	0.302002	0.589481	0.091297	0.444849	1.532808
NCF4	0.696656	1.037939	0.627271	0.902889	1.250044	0.660155	1.185442	1.766432
NCK1	6.907648	6.873036	6.654702	6.723971	7.197084	6.427831	6.10348	6.345515
NCK2	14.86489	13.86964	14.08664	14.35015	15.47611	13.91494	14.48718	15.08533
NCKAP1	45.37297	45.5134	45.56346	43.91841	43.90746	45.78445	46.28616	45.99564

NCKAP1L	1.414888	2.3596	1.60426	2.082417	2.236162	1.36593	2.228041	3.998005
NCKAP5	3.363383	3.260152	2.291397	2.890425	3.079951	2.997	2.992506	2.997449
NCKAP5L	13.27698	12.01897	10.02741	11.58977	12.7626	11.65749	10.7318	13.13888
NCKIPSD	5.089797	4.946123	3.991261	4.548164	4.240472	3.936125	4.763588	4.87493
NCL	182.4243	201.9019	184.3791	183.1456	172.9958	188.2351	195.522	176.8183
NCLN	11.75836	11.82604	9.424596	10.72635	10.82764	11.81285	11.63094	10.91097
NCMAP	2.71851	5.430792	10.13356	7.160807	2.527392	6.071695	6.004365	3.300885
NCOA1	11.86133	11.10596	10.47952	10.24431	11.23994	10.8175	11.54536	12.04993
NCOA2	11.00848	10.19304	9.068582	9.749949	9.675373	9.164103	10.0238	10.43213
NCOA3	16.0056	15.19786	13.03185	12.35026	14.09866	12.31469	14.60691	14.81425
NCOA4	44.92288	46.22272	48.31176	44.15663	42.95617	44.06625	41.9762	43.60112
NCOA5	4.172719	4.198056	3.659415	3.651037	4.061907	3.886864	4.254963	4.295946
NCOA6	9.181318	8.513257	7.581898	8.67493	8.179871	7.588953	7.931768	8.216302
NCOA7	1.830842	1.931575	1.82842	2.063106	2.419639	2.023526	2.011999	1.912752
NCOR1	15.45294	15.57698	12.66154	14.14567	14.84937	13.44261	14.6182	15.20219
NCOR2	22.35388	19.00454	19.42084	21.93921	19.78656	18.83139	19.05435	19.7413
NCR1	0.042248	0.085605	0.083443	0.021647	0.042253	0.148877	0.214408	0.214246
NCR3	0.668449	1.146068	0.914012	1.053857	2.399871	0.931868	1.113396	1.390697
NCR3LG1	2.671254	3.48886	2.351925	2.924314	2.49993	1.911815	3.11421	3.090104
NCS1	4.761102	4.141058	4.936587	4.745602	4.535238	4.485832	5.60825	5.064294
NCSTN	34.58898	37.39198	35.24562	35.56456	37.79026	35.38388	33.82184	35.87085
NDC1	5.178405	5.90513	5.513042	5.360704	4.792402	5.367128	6.623614	6.654274
NDC80	10.09601	12.40146	9.888294	10.57681	8.829222	11.00013	10.63233	10.82863
NDE1	61.0605	50.80971	66.85359	48.75161	62.12033	73.13589	57.98006	66.1874
NDEL1	28.09153	26.8181	25.57644	25.43919	26.88365	29.69203	27.4046	25.17767
NDFIP1	56.29314	69.31104	70.88155	66.7058	66.26103	66.90678	63.76049	63.30611
NDFIP2	25.88184	28.06536	26.64402	25.02436	27.87782	27.80746	25.50523	26.27619
NDN	9.138631	8.575277	8.192219	9.779684	9.173538	9.421751	9.241483	9.217422
NDNF	1.325964	1.417825	1.239887	1.280355	1.414952	1.066784	1.550995	1.661638
NDOR1	6.85997	5.602046	4.881975	7.25505	7.239302	5.874496	5.860208	5.645333
NDP	0.68475	0.371645	0.652067	0.576406	0.562546	0.923341	1.005299	0.930131
NDRG1	106.86	91.97965	103.6678	98.84879	96.70748	105.3231	89.68295	105.5625
NDRG2	47.77329	41.11044	45.68872	49.28407	52.24041	44.43234	44.00152	34.28549
NDRG3	20.47104	17.33407	17.14481	16.68761	16.7091	16.74001	17.80543	16.60792
NDRG4	8.709108	7.061843	7.802833	5.991939	9.071878	7.482749	8.246263	11.60318
NDST1	26.44023	22.03289	20.29362	23.50018	22.00957	21.71101	22.28353	22.97236
NDST2	9.462493	10.08334	7.977949	10.13123	9.412643	8.426862	8.925434	9.29162
NDST3	0.364996	0.229361	0.177943	0.231997	0.240281	0.246543	0.220407	0.281158
NDUFA1	47.06636	55.58437	56.31219	48.803	44.21746	52.85573	51.16447	48.86623
NDUFA10	40.42671	42.57275	38.73734	48.40647	39.45631	43.93267	45.18402	39.00442
NDUFA11	94.57591	86.02909	81.1908	77.79694	83.12578	90.93058	88.11117	75.12389
NDUFA12	70.44113	84.75324	85.69556	77.67724	75.54929	87.52677	79.62969	76.03447
NDUFA13	70.24308	80.63536	82.49437	78.46149	79.65514	82.74585	79.41358	74.47637
NDUFA2	62.21824	79.57226	83.81431	73.62914	76.10962	86.99951	77.88381	70.39577
NDUFA3	99.62982	107.1082	126.6017	113.318	108.7802	121.9203	105.1722	96.78262
NDUFA4	146.55	229.6133	260.6688	230.2654	203.6554	270.2466	192.8514	159.1088
NDUFA4L2	46.04937	39.45251	26.71178	39.22582	41.10403	31.46623	33.01283	42.94608
NDUFA5	17.17113	24.8907	23.84534	22.30858	14.90727	27.28576	14.31724	4.753798
NDUFA6	80.34869	96.98494	103.861	81.06418	87.42796	87.33069	91.19577	85.44957
NDUFA7	56.03289	68.21036	67.94262	55.07257	70.55491	65.25981	67.78288	66.84645
NDUFA8	56.3966	67.76602	63.30102	61.25832	67.83805	67.06438	69.20051	56.84592
NDUFA9	48.93837	51.89325	49.17129	51.92138	50.13951	52.58045	51.10156	46.54096
NDUFAB1	24.86989	29.76578	32.6773	34.34121	31.8713	31.1512	28.32343	26.93403
NDUFAF1	4.61586	3.286414	3.80346	3.334236	3.548105	4.114571	4.147953	3.946037
NDUFAF2	3.1569	3.611512	3.326396	2.879082	3.202628	5.079463	3.311573	3.799313
NDUFAF3	12.97532	15.99765	13.42078	15.45206	14.69217	16.06118	11.39644	14.04612
NDUFAF4	4.929141	5.815759	6.093759	4.648147	5.360058	4.925687	5.065461	4.899574
NDUFAF5	5.222041	6.353635	5.952208	5.376399	7.565617	5.503403	6.068183	6.236859

NDUFAF6	5.285879	4.903755	5.171117	4.706411	4.71705	3.988377	4.133818	5.072355
NDUFAF7	4.847558	4.154346	4.306035	3.053805	3.930521	3.482692	4.887304	4.430674
NDUFAF8	13.30097	15.37699	15.63335	16.38998	16.24071	19.14294	16.56502	15.55939
NDUFB1	26.36548	28.51316	37.49018	28.2863	29.92599	33.24039	27.46727	25.87821
NDUFB10	88.42594	103.0119	108.6048	98.1888	93.35056	103.3696	97.08504	90.27092
NDUFB11	44.05642	44.73229	48.61667	44.48911	49.81494	46.97807	41.78178	42.79326
NDUFB2	34.66558	40.72626	46.16322	44.05715	44.16279	47.84691	34.63818	42.46655
NDUFB4	70.85648	86.45554	97.21995	85.63848	72.51898	84.7707	87.19423	81.34698
NDUFB5	37.9465	46.84507	51.41904	45.417	40.4139	45.89792	43.62417	43.44443
NDUFB6	125.4088	158.4153	153.0579	142.1524	150.0905	174.0338	141.678	140.2509
NDUFB7	86.66645	84.30993	100.2459	85.2279	89.37696	94.25329	95.576	77.25485
NDUFB8	136.5532	159.0188	164.3151	154.7626	139.7144	156.926	171.5175	149.2753
NDUFB9	64.33742	71.69234	71.71634	63.74453	66.93162	73.74917	62.88199	56.36786
NDUFC1	19.84094	16.71671	17.86367	17.86913	20.9844	20.20209	19.82841	18.55318
NDUFC2	71.80579	83.05962	96.84793	82.6002	70.69364	86.62534	86.2338	74.6651
NDUFS1	20.08752	21.4273	21.46896	19.77476	21.35313	20.06433	21.6826	21.92962
NDUFS2	94.2757	99.38605	94.58415	107.2095	88.87708	95.6758	107.4519	87.18804
NDUFS3	51.11445	55.06058	54.70559	55.12645	52.28621	55.569	56.16764	48.59273
NDUFS4	59.72131	65.64549	73.72146	61.83991	59.8954	72.43989	73.53415	58.75771
NDUFS5	135.7454	152.1348	167.3182	147.0132	143.3166	156.9236	157.1309	152.015
NDUFS6	41.54948	42.46378	48.33614	44.38748	40.43402	52.60901	40.72241	41.65789
NDUFS7	13.69285	15.27	14.75669	15.22468	15.70642	14.26378	13.85449	12.8094
NDUFS8	21.17183	22.09318	25.96777	23.49706	23.90609	24.08761	22.84324	21.21555
NDUFV1	43.5151	42.13119	43.44493	41.79912	42.93643	45.84435	44.40142	40.36391
NDUFV2	33.3399	42.51121	44.72188	40.15355	42.56984	45.45523	38.08296	39.32621
NDUFV3	19.76044	20.51445	20.88986	22.69285	23.45419	21.52928	22.85547	20.66804
NEB	0.758771	0.806496	0.470253	0.529879	0.825015	0.707052	0.722552	1.140334
NEBL	19.13554	16.84912	15.63516	15.71854	14.96497	16.71768	16.01289	15.99783
NECAB2	0.181181	0.237947	0.258446	0.206299	0.221473	0.138503	0.163466	0.241611
NECAB3	9.30015	10.11127	5.037477	8.697166	7.157162	7.190238	7.173536	6.133403
NECAP1	19.01402	17.87155	16.28224	17.53196	19.57057	17.70477	18.14082	17.29554
NECAP2	19.51804	19.48935	20.77062	20.31752	21.25311	19.37743	19.73717	21.84724
NECTIN1	62.81677	54.91275	50.34618	56.92901	55.18316	52.83685	54.17276	54.73817
NECTIN2	23.24246	21.49308	19.90864	26.74421	23.57287	25.04007	22.30817	22.67873
NECTIN3	1.910397	2.182094	1.400573	1.306953	1.513696	2.019354	1.890711	2.83393
NECTIN4	48.36783	41.72626	41.3279	46.01935	39.72925	45.46749	43.78158	41.65277
NEDD1	11.40416	13.65016	11.62863	11.49241	11.07389	12.68276	12.83375	13.41683
NEDD4	5.499493	5.663635	4.758634	5.848897	5.768783	4.962031	5.662605	7.660818
NEDD4L	3.216692	4.156288	2.78706	3.832903	3.281665	3.014813	3.858237	3.200653
NEDD8	120.4071	133.2665	130.8725	120.8237	117.4266	128.7474	126.5244	113.9132
NEDD9	12.49667	11.52862	8.595131	12.00777	12.36539	9.66914	11.76454	10.35284
NEFH	0	0	0.075635	0.008721	0.025533	0.017136	0.008638	0.120835
NEFL	0.015358	0.062236	0.030332	0.023607	0.00768	0.007731	0.007794	0.031152
NEFM	0.129088	0.055219	0.093485	0.073492	0.031559	0.095311	0.078615	0.340407
NEGR1	0.823488	1.253826	0.552994	0.626886	0.583575	0.431139	0.711658	1.522469
NEIL1	1.392354	1.871241	1.360975	2.285861	2.358783	1.716571	1.701656	1.916524
NEIL2	14.1568	10.08463	15.17456	9.747203	15.17628	15.01073	16.38981	13.59554
NEIL3	2.486104	1.854451	1.713193	1.70779	1.448138	1.691648	2.454072	2.064301
NEK1	3.518802	4.171789	3.668327	3.69449	3.444379	3.055773	3.974922	4.573559
NEK10	0.233993	0.346478	0.242928	0.172156	0.20402	0.259755	0.286221	0.146046
NEK11	0.41357	0.444782	0.477534	0.423815	0.439078	0.358741	0.529561	0.406551
NEK2	8.801201	8.045457	6.528605	10.25849	6.894046	7.021418	8.114909	7.795349
NEK3	2.997126	2.995974	3.283385	3.489072	2.509918	4.321205	3.082644	2.504789
NEK4	6.946396	7.931318	6.195791	7.466165	7.693851	6.931738	6.330505	6.175572
NEK5	0.022261	0.142835	0	0.129269	0.037106	0.007471	0.022595	0.030104
NEK6	21.89527	22.865	18.70849	24.081	23.0208	21.30212	24.13354	26.00209
NEK7	23.86754	24.47905	24.35236	24.01339	22.65782	23.78359	23.82713	24.10778
NEK8	2.599474	2.482234	1.99908	2.350018	2.263628	2.519462	2.297285	2.431925

NEK9	19.25058	18.87829	16.8939	18.83192	20.64628	18.12963	19.09064	18.45064
NELFA	16.17522	17.95998	15.15029	18.46877	16.40824	16.84107	16.82146	21.98674
NELFB	14.28196	12.88129	12.80541	13.60052	13.49994	12.32782	13.73603	13.80517
NELFCD	35.38438	41.91452	34.08501	46.12281	35.57263	42.15961	35.8033	33.14371
NELFE	33.41794	31.42052	31.79575	37.38346	32.44533	32.96536	37.14143	30.19756
NELL1	0.018797	0.114264	0.018563	0.077052	0.178597	0.085166	0.219411	0.104857
NELL2	0.320581	0.67456	0.503291	0.539112	0.402833	0.628989	0.559002	0.383504
NEMF	8.822746	10.91868	9.535162	10.20495	11.76977	9.534348	9.520893	10.77012
NEMP1	4.343485	3.642987	3.230382	3.392496	3.409314	3.16472	3.195389	3.871491
NEMP2	3.029379	4.056965	3.125509	4.402823	3.817198	2.588101	3.884539	4.690724
NENF	56.6661	71.37596	79.05277	68.42729	74.19882	67.19935	57.7589	63.38686
NEO1	22.45447	24.30383	16.89049	19.4438	20.21124	18.36713	21.96384	21.88651
NEPRO	4.077282	5.114315	3.865016	4.827534	4.261771	5.103127	4.968204	5.099198
NES	1.954078	1.636992	1.802254	2.595471	2.733392	2.00328	1.739419	1.503351
NET1	104.7604	112.2082	108.9108	106.5069	116.4455	120.3677	109.7576	108.2136
NETO1	0.025117	0.101788	0.049609	0.042899	0.175845	0.042148	0.033992	0.195308
NETO2	0.221953	0.206127	0.082195	0.241666	0.175735	0.11639	0.173655	0.136005
NEU1	3.927535	3.313435	3.172087	3.979935	3.489971	2.851848	3.556683	3.154179
NEU3	1.280134	1.133744	1.176277	1.333563	1.314215	1.143774	1.084215	1.276862
NEU4	0	0	0	0	0	0	0	0
NEURL1	0.231954	0.07421	0.108504	0.104254	0.113957	0.069652	0.090869	0.156838
NEURL1B	8.890885	8.237036	8.244249	9.835811	11.22291	9.560638	8.857216	9.473062
NEURL2	0.431266	0.58985	0.404598	0.375656	0.345057	0.564476	0.393962	0.459276
NEURL3	0.893772	0.778414	0.820696	0.915911	0.909569	0.599918	0.668447	0.477103
NEURL4	6.038918	6.80324	4.869562	6.391057	6.663957	5.635027	5.395651	6.821256
NEUROD1	0	0	0	0	0.033835	0.011354	0	0
NEUROD2	0	0	0	0	0.009958	0	0	0
NEUROG2	0.142077	0.086365	0.028061	0	0	0.02861	0	0
NEXMIF	0.27782	0.295778	0.130204	0.120636	0.24489	0.260754	0.219854	0.25312
NEXN	4.652399	5.672982	4.192417	5.576437	5.176463	4.525254	4.224669	3.404154
NF1	6.398965	5.971629	4.399068	5.267208	6.245709	4.618725	5.082184	6.120969
NF2	10.64498	9.853114	9.210145	10.17865	10.00464	9.766483	9.804695	10.51469
NFAM1	0.443952	0.77689	0.817056	1.158052	0.766926	0.345398	0.532541	1.780621
NFASC	0.15347	0.200834	0.069464	0.111401	0.211049	0.070821	0.136301	0.097284
NFAT5	9.998163	8.840524	8.104835	8.762038	9.622781	7.34466	8.393067	10.8026
NFATC1	4.725814	3.763112	3.752731	5.200088	5.103625	4.171248	3.503274	4.72354
NFATC2	1.17722	1.269123	1.486725	1.492509	2.007571	0.84336	1.723382	1.905774
NFATC2IP	4.381346	3.539307	3.400763	4.438852	4.456604	4.561661	3.924873	5.39474
NFATC3	11.201	10.32659	10.43423	11.03697	11.50005	9.484382	10.65676	11.33342
NFATC4	2.901105	3.140973	2.787987	3.727301	3.81956	3.417917	2.513915	3.06537
NFE2	0.145608	0.098346	0.383451	0.348169	0.323616	0.114025	0.229899	0.918905
NFE2L1	77.42596	69.45647	69.97629	70.36481	74.34459	70.23817	68.50302	74.86856
NFE2L2	19.80005	19.97576	16.70728	18.96277	21.87092	18.681	17.26458	20.57049
NFE2L3	40.09079	52.57368	45.91752	52.91636	47.691	53.92389	43.21639	38.93866
NFIA	5.673156	6.017124	5.38994	6.159808	6.666372	5.997809	5.828032	6.696735
NFIB	57.21314	53.87734	38.3714	41.92702	48.95993	44.61892	48.62953	61.06119
NFIC	19.62328	20.31215	18.33118	20.60018	19.97355	18.21419	18.8157	19.76949
NFIL3	13.19417	10.5846	9.562929	10.94803	13.48799	12.45298	12.23474	14.68659
NFIX	31.64756	34.10966	31.4484	36.60806	29.79806	31.3243	33.02719	29.88427
NFKB1	12.8237	11.73279	11.13624	12.60563	13.18814	12.01439	13.14058	13.00084
NFKB2	9.64062	8.738583	7.662044	9.608753	10.75629	7.969619	8.629098	8.647714
NFKBIA	45.28975	49.81274	40.77329	49.54476	47.6822	50.57952	56.16202	51.45569
NFKBIB	9.350829	9.195545	10.40163	9.171467	9.056488	8.947162	9.276846	8.520564
NFKBID	1.154077	1.248685	1.139697	1.32045	1.792972	1.139399	0.955307	1.079593
NFKBIE	3.30513	3.435642	3.081943	3.815095	4.718713	3.129778	3.466946	3.900492
NFKBIL1	5.463815	6.12737	5.310896	5.423081	5.894122	5.431944	4.447081	4.565717
NFKBIZ	11.63853	11.94652	10.88819	13.38724	12.43698	11.50975	14.20061	13.10197
NFRKB	5.615284	5.756581	4.496066	5.144224	5.569809	5.477938	4.751331	6.106485

NFS1	5.916267	6.549231	6.212005	5.804254	5.429747	6.219479	5.748915	5.806356
NFU1	2.096354	3.444109	3.46904	2.09022	2.644392	3.251582	4.044736	2.739155
NFX1	15.23912	14.59603	13.58174	14.52778	14.10414	13.34584	14.06498	15.15463
NFXL1	2.649346	2.758863	2.914398	2.577504	3.079005	3.207687	2.491659	2.999984
NFYA	10.29236	9.266467	7.747441	7.463119	9.487656	7.957955	9.211197	9.455441
NFYB	15.11752	14.45255	13.4038	12.15343	13.53917	12.85232	15.092	15.17067
NFYC	14.60184	14.70026	13.84747	14.61467	14.77857	13.23792	14.95161	14.76305
NGB	0.042131	0.128052	0.055475	0.143916	0.056182	0.226235	0.114035	0.014244
NGDN	18.21067	21.10585	22.74211	22.31665	20.88238	19.35766	23.32655	20.4822
NGEF	1.657253	1.962892	2.443046	2.247996	1.441276	1.716939	1.673986	1.867606
NGF	0.213454	0.270319	0.526986	0.300769	0.266852	0.349232	0.487476	0.189431
NGFR	1.410863	2.268177	1.13465	3.956501	2.078546	3.045148	2.015127	1.83367
NGLY1	17.37671	13.2209	16.25658	15.93178	15.75396	14.97271	13.72277	16.45319
NGRN	7.708689	0.091522	3.538687	0.092574	5.496162	8.352548	12.37832	12.38427
NHEJ1	3.272446	3.534956	3.638305	3.142523	3.402917	3.185702	3.178553	3.26408
NHLH1	0.009725	0	0	0.009966	0	0	0	0
NHLH2	0	0	0	0	0	0	0	0
NHLRC1	0.268481	0.498675	0.320373	0.38977	0.42515	0.450526	0.374699	0.351725
NHLRC2	4.445014	4.40022	3.907942	3.433724	4.75101	3.019173	4.498774	5.024508
NHLRC3	6.559003	5.316066	4.745967	6.344566	4.880038	5.233674	7.379123	8.766213
NHP2	48.72725	50.76253	49.80287	52.38637	55.62296	56.68927	54.64685	52.03169
NHS	4.490733	4.481496	3.1652	3.810779	3.871455	3.205638	3.641442	3.42318
NHSL1	4.66382	2.61869	3.841173	3.288599	3.462413	3.611337	3.507558	3.688553
NHSL2	2.45308	1.91281	1.671781	2.30455	2.775675	2.012884	2.383924	3.134743
NICN1	7.461842	9.001128	6.092934	8.598789	8.18876	6.53352	7.500094	8.687079
NID1	22.42213	24.47618	22.64167	17.73116	21.02466	14.73208	23.35428	30.33965
NID2	9.531413	9.765753	7.631878	8.465993	9.286989	8.263509	10.3007	9.691403
NIF3L1	2.979961	3.239977	3.347387	2.89804	3.766734	3.046886	2.856996	3.015681
NIFK	49.63503	57.97751	52.83253	59.11707	47.57234	49.69868	53.62481	49.42253
NIM1K	1.345256	0.939483	1.199514	1.646264	1.476054	1.341296	1.272638	0.91402
NIN	4.605306	4.69406	3.264569	3.719044	4.917354	3.766495	3.699796	4.666817
NINJ1	12.64359	10.13478	11.26393	13.52747	13.63539	12.56384	13.31476	13.89047
NINJ2	5.67933	4.747584	7.293649	4.515074	5.756478	9.23106	4.989024	7.232512
NINL	2.287798	2.270931	1.841388	2.54097	2.360837	2.310085	2.375806	2.622149
NIP7	6.309981	5.716632	5.946752	5.261626	7.349954	6.066741	6.15445	5.692137
NIPA1	1.122594	0.690063	0.572987	1.318437	1.097509	1.155662	0.678539	1.151368
NIPA2	9.165812	9.744431	10.24618	8.757928	9.029295	10.25821	9.722547	9.665352
NIPAL1	13.71014	15.37418	13.06178	13.02953	10.88134	11.4268	13.64152	15.11698
NIPAL2	46.71336	33.70078	38.35421	42.08191	38.69371	44.88097	34.93109	43.72571
NIPAL3	32.31859	33.98568	38.54439	31.49305	33.26583	37.81203	38.98841	32.0691
NIPAL4	8.689949	6.746686	6.743863	7.112208	5.748092	5.055278	7.415724	6.711069
NIPBL	16.4717	15.65406	14.36787	14.85499	16.07744	15.42878	14.84245	16.60419
NIPSNAP1	10.793	11.05031	10.04361	15.50576	13.23352	15.45375	13.64096	11.07296
NIPSNAP2	54.20669	54.2224	52.51408	55.662	51.59023	55.62789	57.87742	46.03584
NISCH	11.65677	11.21031	10.32276	11.84097	12.46089	11.36664	10.84105	11.3916
NIT1	5.688242	4.331712	5.784771	5.134277	4.916633	6.10638	5.448564	5.769218
NIT2	13.84502	16.1264	16.47875	17.27516	13.05618	16.28438	14.74666	13.65206
NKAIN1	3.270495	2.234047	1.786147	2.996044	1.800657	3.218001	2.883652	3.325429
NKAIN2	0.034619	0.061378	0.034187	0.097559	0.017312	0.078424	0.122983	0.052667
NKAIN3	0.13925	0.047026	0	0.095133	0.046423	0.467336	0	0.047077
NKAPD1	9.317045	9.793778	11.12376	9.766068	9.964822	8.377535	9.618918	9.387967
NKAPL	0.14313	0.340771	0.402839	0.432694	0.178936	0.129697	0.283291	0.355662
NKD1	21.88164	21.10044	25.83243	20.30737	21.12276	22.37029	20.54487	18.30117
NKD2	1.745637	3.2981	2.166505	4.798541	3.338372	2.802599	2.729563	3.361533
NKG7	1.762281	1.848423	0.818975	1.954657	2.737072	1.44033	2.041239	2.292037
NKIRAS1	0.808935	0.309608	0.568075	0.552649	0.737125	0.705867	0.602118	0.492271
NKIRAS2	21.03847	19.77068	18.17793	20.87427	20.0763	17.77141	19.07287	20.44842
NKPD1	9.168694	6.921821	5.744221	8.688595	7.355624	7.047575	8.095415	8.139314

NKTR	11.98433	10.86097	8.233045	10.30024	11.23314	9.983922	9.078997	12.16472
NKX1-1	0	0	0	0	0	0	0	0
NKX1-2	0.061786	0.187791	0.152541	0.094975	0.123588	0.062208	0.125426	0.031333
NKX2-1	0.009569	0	0	0	0	0	0	0
NKX2-2	0.032851	0	0	0	0	0.033076	0	0
NKX2-5	0.01933	0	0	0	0	0	0	0
NKX2-8	0.078401	0.357435	0.348409	0.281201	0.039206	0.118405	0.079577	0.079517
NKX3-1	0.128391	0.225464	0.177507	0.1228	0.376661	0.206828	0.165068	0.121537
NKX3-2	0.169631	0	0.083759	0	0.056551	0.028465	0.028696	0.057349
NKX6-1	1.698618	1.361317	1.427087	1.299025	1.597414	0.689194	1.209441	0.899969
NKX6-2	0.023442	0	0.092598	0.048044	0.023445	0	0.023793	0.04755
NKX6-3	0	0.013158	0	0	0	0.013077	0	0
NLE1	6.913719	9.29822	6.647505	7.925828	6.869022	8.123658	8.066182	7.459068
NLGN1	0.137464	0.433278	0.286585	0.414781	0.25205	0.461343	0.37982	0.201385
NLGN2	6.1786	6.608641	4.474168	7.803238	6.762361	6.119441	5.351563	6.175192
NLGN3	0.384137	0.32134	0.271462	0.361149	0.331317	0.439986	0.300473	0.343139
NLGN4X	0.017678	0.035821	0.283693	0.244569	0.057462	0.053397	0.013458	0.013447
NLK	2.47467	2.818297	2.962931	2.451234	2.752614	3.276511	2.289985	2.27029
NLN	10.23572	9.080804	8.749042	8.192965	8.251888	7.673529	8.697493	9.13987
NLRC3	0.715558	0.676619	0.419703	0.551073	0.54216	0.484664	0.462186	0.580596
NLRC4	0.12447	0.235393	0.139308	0.093539	0.116187	0.133675	0.151605	0.336645
NLRC5	1.078353	1.346924	0.944566	1.27158	1.551256	1.33484	1.169496	1.610591
NLRP1	0.380532	0.339872	0.276899	0.374565	0.260398	0.252061	0.350666	0.71096
NLRP12	0.015606	0	0.007706	0.06397	0.03902	0.015713	0.00792	0.340303
NLRP13	0.130668	0.220639	0.215067	0.062489	0.087124	0.245581	0.150312	0.229716
NLRP14	0.016611	0.025243	0	0	0.024919	0.025086	0.00843	0.042118
NLRP2	0	0.017365	0.016926	0.008782	0	0	0.008698	0.026076
NLRP3	0.195397	0.089983	0.169573	0.048542	0.09475	0.065577	0.108179	0.186168
NLRP5	0	0	0.006428	0	0	0	0	0
NLRP6	0.258262	0.196239	0.26142	0.218344	0.109776	0.240525	0.157282	0.229196
NLRP8	0	0	0	0	0	0	0	0
NLRX1	5.81822	6.333695	5.346715	4.938503	5.848361	5.103532	6.009883	6.266136
NMB	1.086536	0.352255	0.257519	0.534456	0.608539	0.437583	0.970494	0.528962
NMBR	0	0	0.070692	0	0	0.024024	0	0
NMD3	25.24152	26.41575	31.0895	30.91321	28.21533	32.16439	30.78542	28.04417
NME3	17.75979	17.95911	18.06499	20.76064	18.76183	17.91467	16.60574	16.99877
NME4	17.42463	17.4587	14.93124	18.27804	19.29306	16.57669	16.73903	17.31087
NME5	0	0.057557	0.018701	0	0.075759	0	0	0.038414
NME6	2.970572	2.009843	3.085819	2.896687	3.320481	2.825282	2.243091	3.096261
NME7	15.13182	16.9301	13.79236	15.93042	16.26176	13.66436	14.84366	14.79435
NME8	0	0	0	0	0.014504	0	0.01472	0.014709
NME9	0.405907	0.616852	0.437291	0.151259	0.313696	0.352951	0.599267	0.224556
NMI	8.245943	11.33979	10.31563	10.93138	11.8447	11.26936	9.352642	10.51029
NMNAT1	15.83946	15.85114	17.23248	16.26315	17.65604	18.51325	15.66323	16.86096
NMNAT2	2.147637	2.208861	1.840241	1.885748	1.9895	2.059119	1.872497	2.192385
NMNAT3	0.968483	0.981194	0.970079	0.850691	0.761049	1.016889	0.786408	0.92614
NMRAL1	11.58098	7.960872	13.40336	10.69215	11.58247	9.763965	16.41263	9.967497
NMRK1	12.39111	12.75203	10.42277	12.23518	12.60353	12.00584	12.34775	12.43008
NMRK2	0.76358	0.72025	0.676059	0.809477	0.684678	0.874838	0.85521	0.453988
NMS	0	0	0	0	0	0	0.259443	0
NMT1	17.86687	18.13003	17.07958	18.64592	19.35262	18.49052	17.61772	18.38534
NMT2	7.41663	7.109791	8.076338	6.485378	6.882579	7.184306	7.96041	8.175116
NMU	0.260364	0.351708	1.371307	1.378537	0.390597	0.699048	0.924944	0.396106
NMUR1	0	0.019983	0	0	0	0	0	0
NMUR2	0.03394	0.080233	0.022345	0.173903	0.497851	0.011391	0.137796	0.103269
NNAT	2.235861	7.734845	7.472202	2.584635	18.96637	6.643595	1.923454	5.766017
NNMT	0.241026	0.244189	0.089259	0.216122	0.090396	0.045501	0.137611	0.183343
NNT	12.62561	11.75565	10.42567	11.24142	12.27449	11.74283	12.50554	12.53857

NOB1	35.65043	32.15116	28.51469	32.81735	33.39042	29.87737	30.86282	33.34335
NOBOX	0	0	0	0	0	0	0	0
NOC2L	19.6489	19.918	17.64305	18.53521	18.57693	19.27016	18.47091	19.20966
NOC3L	7.636295	7.463082	8.865941	6.816763	8.4153	8.551616	7.99255	7.857716
NOC4L	5.052485	5.443797	4.563836	5.249549	4.220975	4.451126	5.3216	5.785297
NOCT	3.548913	3.626046	2.968565	3.533795	3.680086	3.289735	3.826644	4.068483
NOD1	2.984693	2.842997	2.63347	3.172966	3.007397	3.033168	3.131389	3.519452
NOD2	1.332272	1.241467	0.999006	1.404392	1.592061	1.399026	1.146899	1.885538
NODAL	0	0	0	0	0	0	0	0
NOG	0.238206	0.522886	0.274444	0.162737	0.277943	0.679527	0.443261	0.402661
NOL10	15.35477	17.99211	18.01073	17.39402	15.86449	19.84778	17.65597	15.48592
NOL11	20.5638	20.08307	17.58418	19.99337	20.48413	19.68482	19.21785	21.02353
NOL12	7.993758	10.50819	8.93801	8.586686	8.479324	8.403117	8.482468	8.275066
NOL3	2.6019	2.646895	1.998484	3.697778	3.662407	3.1048	3.010441	2.866996
NOL4L	2.064034	1.816663	1.838731	2.119568	2.219124	1.848674	1.693454	2.141391
NOL6	7.444851	6.902761	6.265008	6.400269	7.468991	6.218232	7.074318	6.692918
NOL7	21.96106	26.69481	27.03039	24.88433	26.58675	27.73	26.91721	23.89398
NOL8	10.07201	11.72206	9.3547	9.853927	9.909854	9.64715	10.57561	10.38807
NOL9	7.498672	8.015733	6.850252	7.06334	8.078232	7.405669	6.839411	7.733947
NOLC1	42.19998	39.27939	38.9843	40.36849	40.56639	43.03828	43.14872	39.37636
NOM1	11.49646	11.36326	9.49877	10.77987	10.11275	10.3174	9.771524	10.60873
NONO	119.0796	118.1177	107.3614	113.6777	102.355	104.1392	113.1367	116.783
NOP10	36.36797	38.44486	37.82839	34.45573	39.13606	40.08477	37.66014	36.30338
NOP14	10.46559	8.608059	7.831303	9.766596	9.459816	9.921519	10.8416	9.419981
NOP16	38.31328	42.57919	47.97359	39.65194	42.03305	44.67046	48.89198	40.28252
NOP2	14.23596	12.32077	10.94459	13.82021	13.6009	13.0819	11.84317	12.64681
NOP53	133.4619	160.6345	154.8558	158.1139	146.1392	139.9672	135.7208	145.5756
NOP56	27.90348	25.91147	20.23673	23.42889	21.43733	23.39259	19.08911	26.2006
NOP58	37.1277	39.92636	37.96152	41.169	36.01075	45.77386	39.93561	38.03112
NOP9	8.517869	6.188811	6.318552	7.190853	7.096947	6.070842	8.017592	6.208955
NOS1	0.506074	0.283923	0.220328	0.284399	0.492534	0.290378	0.695934	1.388059
NOS2	0.049601	0.050252	0.034988	0.058091	0.17717	0.135551	0.050345	0.04312
NOS3	1.436223	1.629123	1.099373	2.225303	3.037763	1.653599	1.506591	1.819093
NOSIP	17.37415	18.07707	16.06758	17.29316	19.56924	16.95999	17.14363	18.28094
NOSTRIN	4.19913	7.330146	4.653972	4.953259	4.290313	5.444454	4.170129	3.278439
NOTCH1	32.99871	23.88497	21.11708	24.69467	24.47561	22.37617	22.53076	25.68559
NOTCH2	28.20554	27.43268	24.39459	24.64116	26.03746	21.8809	24.80624	29.8062
NOTCH3	22.91996	18.13042	17.53645	19.77614	20.91359	18.54173	18.4718	20.7577
NOTCH4	1.667813	1.389028	1.308544	2.013256	2.224038	1.645539	1.175223	1.32696
NOTO	0.024317	0	0	0	0.072961	0	0.024682	0
NOTUM	1.86688	2.858403	2.39809	3.04949	2.849817	2.388407	2.236817	2.107004
NOVA1	0.754046	1.022905	1.148529	0.977911	1.354902	0.789222	0.739412	0.972178
NOVA2	1.3934	0.690499	1.076899	0.900207	1.560204	0.853951	0.999234	1.367152
NOX1	0.276993	0.234944	0.273542	0.257449	0.431649	0.408599	0.359608	0.36587
NOX4	0.485489	0.648361	0.682838	0.783962	0.389913	1.007235	0.507704	0.499861
NOX5	6.228941	3.592239	3.589401	5.380154	6.031216	4.245319	5.676242	6.144053
NOXA1	4.557499	4.339947	3.51469	5.578063	4.622514	4.134635	4.462404	5.259384
NOXO1	0.06053	0.036795	0.011955	0.024812	0.169506	0.036566	0.073726	0.061392
NOXRED1	0	0	0	0	0	0	0	0
NPAS1	0.149196	0.136039	0.132603	0.168181	0.029843	0.030043	0.212007	0.166452
NPAS2	2.037496	1.651389	1.423381	1.500242	1.781153	1.375208	1.17956	2.342035
NPAS3	0.24019	0.357856	0.306961	0.395753	0.240221	0.369859	0.487585	0.305705
NPAS4	0	0.01183	0	0.011966	0	0.011757	0	0
NPB	0.425152	0	0	0	0.070868	0.071343	0.071922	0.359337
NPBWR1	0	0	0	0	0	0	0	0.028502
NPC1	21.41441	20.06207	19.27135	18.22289	18.1024	18.06078	21.48621	21.60984
NPC1L1	0.134031	0.13579	0.097832	0.131379	0.110736	0.070407	0.088723	0.076835
NPC2	91.52073	79.88374	86.04499	75.67456	90.70812	92.04945	98.01106	105.073

NPDC1	39.97303	38.74573	38.06514	47.43322	49.10803	47.08876	42.47064	39.03296
NPEPL1	5.762475	6.479651	5.565604	7.22902	6.295209	6.171629	6.607343	5.536226
NPEPPS	25.14988	23.52872	24.31363	22.2596	24.67654	23.88901	24.51947	24.56142
NPFF	0.779619	0.321148	0.34688	0.438973	0.556942	0.336405	0.356526	0.616933
NPFFR2	0.088854	0.022505	0	0.318694	0.066649	0.022365	0.090187	0.202769
NPHP1	3.370618	2.131925	2.197624	2.585811	2.924063	2.88741	2.769074	3.39027
NPHP3	3.070353	3.25153	2.477311	3.328808	3.326646	2.968124	2.721212	2.256567
NPHP4	0.721922	0.931853	0.491127	0.827484	0.775497	0.705317	0.629623	0.672538
NPHS1	0.059921	0.054637	0.02367	0.055265	0.017979	0.018099	0.012164	0.066852
NPHS2	0.460853	4.980284	2.745837	3.431821	4.071398	3.804814	4.038382	1.028311
NPL	0.43075	1.037669	0.586083	0.519894	0.899905	0.481881	0.612095	0.679594
NPLOC4	26.72036	26.75836	24.42326	27.08704	26.74439	26.38501	27.58061	26.50252
NPM1	260.4741	298.9039	289.4449	287.8509	264.3272	285.0336	273.7532	271.0509
NPM2	0.331887	0.934008	0.691921	0.718006	0.626979	0.44554	0.411725	0.336612
NPM3	13.37828	14.22393	12.49905	13.77128	13.59048	14.89231	15.04365	11.5258
NPNT	5.082413	4.552748	4.437779	5.52463	5.915889	5.81821	4.233298	4.317479
NPPB	1.004739	0.508963	0.566983	0.588358	0	0.505802	0.509905	0.072789
NPPC	2.703453	2.178143	2.234049	2.129194	2.342761	1.898076	2.182176	2.636167
NPR1	1.48208	2.830472	1.615021	2.519681	2.538603	2.92152	1.821312	1.935126
NPR2	5.197463	4.850126	4.183545	4.648667	4.959351	4.24678	4.281234	4.774731
NPR3	9.820637	9.610093	9.077912	8.168423	7.685799	7.12591	8.976111	8.438411
NPRL2	10.95143	11.0752	9.453372	10.98048	10.67705	11.1254	9.776215	9.688944
NPRL3	5.73664	4.790171	4.080201	5.189737	5.205942	5.339094	5.206299	4.641445
NPSR1	0	0	0	0	0	0	0.097042	0
NPTN	56.59404	63.04187	63.05866	60.34885	54.86945	59.77029	63.87469	58.96088
NPTX1	0.240595	0.260002	0.258716	0.136975	0.272709	0.123811	0.244203	0.401277
NPTX2	0.920253	1.421236	1.141525	1.897597	2.031553	1.412408	2.312361	1.183766
NPTXR	5.651609	7.267733	4.448919	5.048156	1.303996	5.046621	1.503612	3.658436
NPW	15.12928	14.08245	13.91359	16.53764	11.94737	9.266519	16.06004	12.40359
NPY	0.061923	0.125471	0	0	0.061931	0.062346	0	0
NPY1R	0.334857	0.347526	1.355001	0.719782	1.44579	0.682513	0.563704	1.101708
NPY5R	0.276209	0.221535	0.431881	0.43637	0.402857	0.231746	0.256989	0.443556
NQO1	4.273979	4.799032	6.018723	3.905496	3.74986	3.68178	5.027172	5.273773
NQO2	4.518985	4.038535	3.448006	5.966577	5.347364	4.195447	4.944055	4.863139
NR1D1	11.2501	14.84465	13.23037	15.3407	12.89997	11.72279	10.7282	10.88882
NR1D2	9.509523	11.46809	10.19598	11.50574	10.46582	10.51182	11.95571	11.10784
NR1H2	17.47173	18.19894	17.61803	16.19212	17.62144	17.4179	16.51164	15.78889
NR1H3	10.7347	9.237147	10.19853	10.56997	12.12426	11.51318	10.32702	11.06833
NR1H4	0.011956	0	0	0	0	0.024076	0	0
NR1I2	0	0	0	0.010922	0	0.021463	0.032455	0
NR1I3	0.102901	0.104252	0.087102	0.090386	0.014702	0.074003	0.193969	0.104366
NR2C1	11.39925	10.95315	10.63285	11.33822	11.00868	10.85329	10.9221	11.43969
NR2C2	9.669815	9.327713	8.695151	9.300734	9.250716	8.631964	9.054403	10.57045
NR2C2AP	9.591679	9.6359	7.561813	9.416274	9.572766	9.048558	8.712912	9.094662
NR2E1	0.228084	0.374125	0.235968	0.200343	0.347601	0.229642	0.242529	0.319457
NR2E3	0.269127	0.155805	0.170854	0.098497	0.288387	0.116128	0.156093	0.155976
NR2F1	4.590328	5.570026	3.442063	4.906211	6.695642	4.290621	4.939535	4.548954
NR2F2	5.456954	5.399599	5.472769	7.740265	9.760976	7.502234	5.728316	5.431347
NR2F6	8.655711	6.88846	6.305798	9.282116	8.503089	8.048134	7.21327	8.155291
NR3C1	15.42313	17.42387	14.49539	15.55453	17.09291	15.55834	15.31596	16.52217
NR3C2	1.024688	1.768676	1.166519	1.492461	1.366427	1.733809	1.444526	1.409757
NR4A1	1.142415	1.248782	1.147973	2.177115	2.054608	1.271295	4.343231	1.290808
NR4A2	1.645942	2.055536	1.215052	2.079166	2.298097	1.591555	2.886388	1.677637
NR4A3	0.120754	0.104862	0.106473	0.08839	0.49602	0.021711	0.468376	0.091855
NR5A1	0.017374	0.017602	0	0.035608	0	0.017492	0.017634	0.017621
NR5A2	0.933488	0.961414	0.763969	0.856193	0.969715	1.064488	0.842795	0.638159
NR6A1	0.759962	0.698019	0.494831	0.590509	0.57631	0.693684	0.487399	0.724195
NRAP	0.040557	0.041089	0.040051	0.088318	0.081124	0.045938	0.08233	0.071985

NRARP	18.84985	14.83586	13.38432	19.79569	17.29424	15.94622	17.18244	14.53614
NRAS	39.31897	33.52546	36.53455	34.28518	34.86221	40.6668	37.14322	35.06847
NRBF2	32.02188	29.82976	34.89473	33.81366	33.24049	34.5953	31.5588	30.24261
NRBP1	109.6307	99.52114	104.9016	96.22717	103.7969	100.4427	101.6359	101.3378
NRBP2	12.27337	11.50559	9.967974	11.75307	10.96009	11.08579	10.75433	15.19386
NRCAM	1.059811	0.391223	1.015987	1.424014	0.918998	0.842857	0.752424	0.577472
NRDC	37.51248	36.41346	38.9286	36.75089	36.19062	38.26046	35.24163	38.39456
NRDE2	5.103054	5.437442	4.088819	4.508154	4.896666	3.970681	5.142828	5.343673
NREP	15.68962	11.19783	10.25102	12.60421	12.90358	11.94633	12.78263	10.13031
NRF1	3.735887	3.366753	2.522266	3.517524	3.235364	3.200216	3.774022	3.155769
NRG1	0.721674	0.471026	0.500248	0.750217	0.829339	0.468101	0.433161	0.837517
NRG2	8.458947	5.345106	6.554211	8.795956	7.940436	8.995617	6.142286	6.352887
NRG3	0.107444	0.751093	0.053053	0.121116	0.085966	0.313716	0.425317	0.316023
NRGN	2.314734	2.02239	2.495607	2.285022	2.54866	2.095354	2.198572	2.563069
NRIP1	10.88141	10.68409	9.81274	10.61539	11.36742	9.53749	10.50012	11.39787
NRIP2	0.111019	0.224952	0.146181	0.176974	0.197393	0.322913	0.225369	0.23771
NRIP3	0.362343	0.404558	0.284802	0.416786	0.502909	0.342483	0.585443	0.390001
NRK	0.05992	0.050588	0.111771	0.057993	0.166465	0.090494	0.111501	0.236338
NRL	0.069526	0.030188	0.166745	0.091605	0.049668	0.090001	0.050406	0.030221
NRM	7.093561	7.515189	5.243873	6.9785	6.891778	5.815646	6.212541	6.372309
NRN1	0.79136	0.942403	1.604131	0.76828	0.666495	0.433329	0.521395	0.901194
NRN1L	0	0.059076	0	0	0	0	0	0.118282
NRP1	21.38175	18.53936	19.90848	20.11231	23.18138	18.93383	19.49937	22.292
NRP2	31.61678	31.94228	28.63205	26.2541	23.8874	23.71418	25.72658	26.09332
NRROS	1.001972	1.305157	0.924247	1.105776	1.123235	0.67624	1.419333	2.088309
NRSN1	0	0	0	0	0	0	0	0
NRSN2	0.07434	0.025105	0.036707	0.165061	0.037175	0.087323	0.088031	0.113098
NRTN	1.661303	1.008155	1.265846	1.279001	0.927751	0.993403	0.924427	0.906624
NRXN1	0.034046	0.122294	0.061131	0.091982	0.148585	0.068558	0.053406	0.072201
NRXN2	0.07632	0.097941	0.120591	0.229418	0.249345	0.102455	0.061972	0.149653
NSD1	18.54108	17.83773	14.84245	16.43619	16.70554	15.45854	16.81272	18.04263
NSD2	5.008543	4.475527	3.673912	4.68383	4.182121	4.081724	4.511866	4.898727
NSD3	11.63662	10.00396	9.071249	9.780858	10.37323	9.496277	9.788601	11.1941
NSDHL	25.21327	25.01281	27.74148	28.52516	27.7497	30.12518	27.33133	22.71787
NSF	9.80996	9.204471	9.079818	9.091058	8.706537	8.580267	9.416492	9.183575
NSFL1C	78.45124	77.6901	69.14899	71.11898	71.09895	75.13463	80.45603	71.78657
NSG1	1.695921	2.019843	2.863762	2.122662	3.923133	2.60688	3.153636	2.717962
NSG2	0	0	0	0	0	0	0	0
NSL1	5.67989	7.14433	5.875013	5.623498	5.001001	5.383058	6.88427	5.526676
NSMAF	21.98189	22.37448	20.18586	16.75101	18.52219	20.77119	18.76551	21.91802
NSMCE1	51.61187	62.39539	58.7449	53.90132	51.94744	51.33905	59.3585	53.79192
NSMCE2	2.467559	2.705295	2.597816	2.596413	2.225497	2.772785	2.468792	2.082592
NSMCE3	10.74742	10.33421	11.85087	10.79662	11.54305	11.35388	11.53556	9.414806
NSMCE4A	30.40202	31.545	31.05921	29.71452	27.84589	28.45499	31.62471	31.72858
NSMF	8.822567	11.81763	8.079506	12.23785	11.37895	10.26055	8.857781	10.72421
NSRP1	5.757637	6.095099	5.047683	6.297619	6.204949	5.122629	5.032997	5.756177
NSUN2	27.26668	27.2633	25.06447	30.03822	29.37225	32.0253	31.23752	29.72943
NSUN3	4.690296	5.524929	4.983514	5.692703	3.825986	5.121842	3.789932	2.837729
NSUN4	4.188634	5.000217	3.548576	4.591856	4.687113	4.304427	4.646948	4.544652
NSUN5	6.058114	6.703379	5.314179	4.751509	5.568093	7.07065	4.723383	5.37202
NSUN6	4.250206	5.177068	3.546885	5.030367	3.756766	4.80724	3.992115	4.50862
NSUN7	0.144239	0.160049	0.196705	0.330816	0.467119	0.221294	0.160345	0.202022
NT5C	8.070495	4.117201	5.652437	8.358387	6.440057	7.751043	8.511081	4.528085
NT5C1A	0.117001	0.105366	0.141219	0.093255	0.091012	0.104711	0.039585	0.065926
NT5C2	19.99331	19.60971	19.01319	18.73861	20.64825	18.28514	18.53764	21.11788
NT5C3A	20.4952	18.1742	29.32827	22.20047	23.0546	26.48896	23.79773	24.07613
NT5C3B	9.511288	11.31288	10.34447	10.90621	10.89539	10.77857	11.92923	10.64535
NT5DC1	3.858831	2.646151	1.547597	2.797447	4.305934	3.206557	2.095185	3.366863

NT5DC2	34.499	30.58678	32.19021	33.58104	34.2833	31.55252	31.45738	34.52762
NT5DC3	2.048413	2.35789	1.781864	1.652524	2.03996	2.19405	1.29172	2.236707
NT5DC4	0	0.028818	0.014045	0	0	0	0.014435	0
NT5E	6.501112	6.451854	7.616161	9.000305	6.744213	8.874221	7.597941	6.292507
NT5M	2.007662	2.751896	3.568762	3.001372	1.889808	2.901271	3.140573	3.234031
NTAN1	51.82648	54.3439	55.58716	49.907	54.81622	53.35772	51.19917	56.58161
NTF3	1.441715	1.544581	1.81651	1.664228	1.624211	1.718519	1.597901	1.07567
NTF4	4.580917	4.76253	5.542294	6.119909	4.749418	5.602268	4.5766	4.475853
NTHL1	3.68942	3.899186	4.482229	6.460015	5.720668	4.235741	6.304131	7.120457
NTM	0.356137	0.218674	0.17585	0.320722	0.512688	0.374869	0.61342	0.602012
NTMT1	8.517792	5.601657	4.737092	6.263283	6.650714	5.672184	8.160194	6.668739
NTN1	11.45542	9.284608	6.744377	9.01748	9.924448	5.583181	9.079624	9.620409
NTN3	0.27205	0.535028	0.158036	0.426383	0.336105	0.35447	0.276131	0.340846
NTN4	5.64569	5.068259	7.089179	6.023088	5.384666	3.726766	5.791095	6.234201
NTN5	0	0.022443	0	0.01135	0.011077	0.044607	0	0
NTNG1	0.587772	1.00382	0.768404	1.428387	0.862178	0.524155	0.960224	1.180925
NTNG2	0.110484	0.062185	0.133353	0.1258	0.184163	0.074159	0.0623	0.049803
NTPCR	27.9678	27.1876	32.78871	33.47747	40.38653	27.90072	36.45461	32.46156
NTRK1	0	0	0.010623	0	0	0.01083	0.010918	0.04364
NTRK2	1.273566	1.712958	0.936044	1.177601	1.387195	1.234365	1.274096	1.432743
NTRK3	0.754586	0.830967	0.571752	0.717902	0.636945	0.647043	0.967665	1.082421
NTS	0	0	0.031445	0	0	0	0	0
NTSR1	0.0334	0	0.065968	0.034227	0.066809	0.016814	0	0.016938
NTSR2	0	0	0	0	0	0	0.019105	0
NUAK1	9.692343	7.934432	7.539621	7.939888	9.171747	7.622479	7.039797	10.00007
NUAK2	2.375694	1.510607	2.120507	2.41326	2.002155	2.563373	1.783203	2.249441
NUB1	32.42706	39.10989	39.97339	36.71914	38.46404	35.66503	34.73476	30.5244
NUBP1	10.44144	12.63233	10.54813	10.85645	10.72621	12.42219	11.28395	8.887709
NUBP2	3.860574	3.137882	3.179947	3.758392	3.641694	3.577759	3.856144	3.693059
NUBPL	4.932642	4.821122	5.406809	4.949954	5.301741	5.038465	5.671413	5.117035
NUCB1	72.30256	73.64577	72.40098	70.37859	70.77714	68.70995	77.52937	74.36944
NUCB2	8.879896	10.58305	10.3158	10.44861	10.93052	9.796035	10.58574	9.665305
NUCKS1	66.28026	76.21801	61.78623	69.42318	64.01714	68.41861	67.01781	67.43605
NUDC	76.91822	73.81958	73.72146	71.66355	72.62318	76.06188	77.24885	69.66142
NUDCD1	7.96225	7.958323	7.271198	7.070085	7.399571	8.088478	7.183719	7.503938
NUDCD2	10.39646	10.18981	11.30364	9.196435	9.584946	12.12114	10.01963	8.603867
NUDCD3	24.70069	23.30258	23.27372	23.06592	24.05354	20.77285	23.67574	22.14102
NUDT1	3.847897	5.636536	3.049643	3.365543	4.14254	4.639151	4.428023	3.529808
NUDT11	0.558188	0.78768	0.433112	0.69459	0.518385	0.702502	0.627264	0.626792
NUDT12	3.79695	3.251998	3.651757	3.555032	3.797441	3.661683	3.157416	3.37156
NUDT13	0.77602	2.124878	0.890624	1.665711	1.741027	1.879395	0.755728	0.840248
NUDT14	2.506492	2.586413	1.856446	3.282073	2.599661	2.360048	1.766729	2.165556
NUDT15	12.11537	13.71684	11.34436	10.54862	13.20481	10.88929	12.81771	12.0098
NUDT16	4.542744	7.571629	7.090996	6.494852	5.312766	7.22952	7.548465	5.239074
NUDT16L1	5.056346	5.41078	5.359603	5.574331	5.242465	5.763036	7.24028	5.391639
NUDT17	12.60815	15.11725	13.01081	14.80436	12.68638	14.42181	14.29002	11.65337
NUDT18	5.095751	5.238549	4.995256	7.026628	4.546797	5.231163	6.059573	5.168293
NUDT19	7.927643	7.115674	7.453595	9.587678	8.164562	8.654647	7.301752	9.502406
NUDT2	12.62191	12.31269	11.83645	10.70446	9.643447	11.19125	12.36947	10.62837
NUDT21	25.00003	27.62661	24.7759	26.73965	25.81227	27.45503	25.56105	24.36886
NUDT22	10.97417	11.85771	8.799197	10.0595	10.72385	11.96145	9.350445	11.66649
NUDT3	12.19215	11.35672	11.66981	11.24236	11.53308	10.75814	12.74564	13.15351
NUDT4	36.36495	33.75501	36.0631	34.30826	33.69293	32.71748	32.852	34.1845
NUDT5	13.86098	13.67067	15.27149	14.94021	14.54756	13.85479	12.5264	11.14502
NUDT6	3.105451	4.162048	3.830616	4.221636	2.956863	3.52471	3.227635	4.178238
NUDT7	3.948415	3.580854	3.867771	3.71991	3.662309	3.302137	3.684444	4.198395
NUDT8	4.956415	7.975264	5.182578	8.033746	7.386984	6.588467	6.017184	6.242645
NUDT9	17.93709	18.59572	14.85157	15.38472	15.92873	14.6948	21.09474	22.29698

NUF2	8.221907	9.457044	7.662831	8.262682	8.22297	8.947305	9.107894	9.130346
NUFIP1	8.700246	8.37029	9.058064	9.347719	9.864926	9.116181	8.659611	9.131917
NUFIP2	4.695676	4.400504	4.283583	3.951846	5.201134	4.500232	4.13758	4.872655
NUGGC	0.052493	0.042545	0.134781	0	0.042	0.052851	0.031968	0.074536
NUMA1	29.54466	27.17456	23.2881	28.04818	26.89407	25.44619	26.37955	26.94801
NUMB	14.89779	15.09331	13.00934	13.82585	13.84242	14.32787	14.16283	14.40721
NUMBL	2.741994	2.370074	2.310223	1.913587	2.915915	2.103743	2.677436	3.639979
NUP107	13.00628	12.70884	12.12595	13.12677	12.09227	12.58683	11.82044	12.71411
NUP133	10.38926	10.75709	10.34608	9.33127	9.315245	9.722736	11.77968	10.78934
NUP153	15.7433	15.12806	13.88257	13.94798	15.97853	13.86521	13.76076	16.42067
NUP155	10.494	10.66711	9.359109	9.095507	9.132975	9.186364	9.737528	9.927003
NUP160	10.22609	11.27205	9.045954	9.58461	9.943465	10.04785	10.81989	11.20292
NUP188	18.15421	18.61112	17.58891	17.11096	17.60219	18.02635	17.20671	17.70619
NUP205	7.21753	6.767937	5.422749	6.416614	6.325539	5.270147	6.708294	6.030113
NUP210	5.382996	4.483562	4.970269	4.88142	5.677421	4.536728	4.900216	4.93733
NUP210L	0.129735	0.097361	0.094903	0.157569	0.379645	0.101594	0.043894	0.18519
NUP214	8.108457	8.151133	6.965838	7.622869	8.202037	7.239781	7.538922	7.540752
NUP35	15.18671	18.39671	16.61837	15.72224	17.59053	16.45595	17.74686	16.05126
NUP37	7.984301	10.07784	9.670878	9.425207	8.66916	8.061052	9.201024	9.149355
NUP43	5.713798	6.165199	5.581451	5.480359	5.996041	6.04189	6.290891	6.052065
NUP50	21.59907	21.26597	20.47216	20.37084	22.72512	22.39279	20.26086	20.00993
NUP54	13.09476	14.74202	12.81468	13.94084	13.92534	13.95906	13.08689	14.38594
NUP58	12.07997	10.41041	10.02509	9.450251	10.50418	8.806367	9.241314	10.51264
NUP62	19.23	16.94414	17.08285	21.5192	17.48234	15.7606	17.84905	19.68802
NUP62CL	1.390766	1.713238	1.092507	1.376629	1.232884	1.0502	1.138927	1.506739
NUP85	12.0155	12.39292	11.34103	12.76875	11.0084	12.54523	12.1517	13.71536
NUP88	30.10039	27.61929	28.94602	30.17383	26.36303	27.98432	26.64059	27.35854
NUP93	18.3926	18.6633	15.62036	17.46448	16.61047	18.37259	18.80555	17.05995
NUP98	16.70932	15.31677	15.03805	14.37574	15.81908	14.53938	15.90477	16.41786
NUPL2	0.88721	0.783404	0.766301	0.842457	0.936168	0.855027	0.696731	0.79252
NUPR1	181.6974	178.9752	233.7318	196.8691	182.8154	208.1475	192.3666	187.9165
NUPR2	1.319355	0.763811	0.372261	0.193148	0.754014	0.474417	0.573919	1.051393
NUS1	9.323357	9.419927	10.38872	9.841255	9.7892	10.69419	9.101474	10.15964
NUSAP1	22.92925	25.35785	22.24332	22.06247	18.47352	22.05958	22.33828	24.97462
NUTF2	36.91446	32.40916	33.32271	32.05543	36.3786	33.32057	35.432	36.14851
NUTM1	0.031047	0.007864	0.01533	0.015908	0	0.054704	0	0.007872
NVL	10.63076	13.35404	10.24555	11.89639	11.88459	12.57165	11.30051	13.76734
NWD1	0	0	0	0	0	0	0	0
NWD2	0	0	0	0	0	0	0	0
NXF1	25.31355	22.73516	18.84906	23.56511	24.28096	24.23264	21.20037	24.5359
NXN	42.23074	40.37708	39.38506	40.68841	40.78203	43.3638	41.01	37.23569
NXNL1	0	0.043161	0	0	0	0	0	0
NXNL2	0.284154	0.215912	0.070153	0.363991	0.035524	0.286095	0.288416	0.180124
NXPE2	0.099002	0.343889	2.248664	1.304407	0.537507	0.655026	1.019221	0.48771
NXPE3	2.044892	2.428924	2.920024	3.439584	2.416575	3.12379	2.714935	3.056173
NXPE4	1.484369	1.847951	1.441028	1.366446	2.32749	2.330422	1.685388	1.83722
NXPH1	0.035233	0.285564	0.187888	0.209414	0.063428	0.092232	0.100132	0.114351
NXPH2	0.075124	0.043492	0	0.021996	0.010733	0.032416	0.032679	0.032654
NXPH3	1.477925	1.111414	0.646999	1.74874	1.386687	1.334618	0.958823	1.128087
NXPH4	4.307315	3.854446	4.088133	3.675478	3.402715	3.324272	3.232162	3.722686
NXT1	13.76085	13.26066	13.0989	12.15562	12.76915	14.14902	14.59001	12.47514
NXT2	2.098122	1.413509	1.882661	1.855411	1.704279	2.026671	1.643139	2.484456
NYAP1	2.219777	1.455177	1.122607	1.912689	1.421148	1.446139	1.434483	1.651529
NYAP2	0	0	0	0	0.010391	0	0.010545	0.031612
NYNRIN	3.111151	2.505712	2.261924	3.455811	3.90623	2.846958	2.411906	2.954914
NYX	0	0.019692	0	0	0.019439	0	0	0
OAF	13.19433	16.01727	15.09879	17.33482	22.42286	13.5989	13.43187	15.01864
OARD1	24.21934	23.13198	24.53219	26.78731	23.67115	25.49482	24.2397	25.12321

OASL	0.04092	0	0	0.083867	0.08185	0.370794	0.041534	0.041502
OAT	122.1455	113.5304	105.0981	104.588	102.1505	113.2924	112.4763	115.4238
OAZ1	354.1347	390.3804	419.1839	411.9587	417.0446	396.3261	405.3205	393.1339
OAZ2	90.45963	88.21599	91.27249	85.74063	87.4026	99.98758	98.80484	95.40969
OAZ3	0	0	0.076993	0.053264	0	0.052331	0.052756	0.105432
OBSCN	0.004412	0.002235	0.001089	0.007911	0.005515	0	0.011194	0
OBSL1	6.590574	6.114369	4.297221	6.780714	6.692025	4.817083	5.548633	5.455743
OC90	0	0	0	0	0	0	0	0
OCA2	0.873874	0.689545	0.697026	0.421928	0.42859	0.693723	0.716408	0.767002
OCEL1	1.358538	1.127627	1.341611	1.694111	1.031313	1.038224	1.412143	1.278272
OCIAD1	49.18469	50.8268	54.26386	53.99423	51.88119	56.96428	53.93186	51.86467
OCIAD2	29.69952	31.8545	37.49057	39.34625	39.53521	36.61356	39.40918	34.8725
OCLN	24.32282	20.13628	25.1359	23.07969	20.74186	22.75894	22.56119	22.38576
OCRL	10.47912	10.90066	9.091659	11.61203	10.86739	11.0136	10.98345	10.64535
OCSTAMP	0	0	0	0	0	0	0	0
ODAM	0	0	0	0	0	0	0	0
ODC1	55.48938	51.97352	64.03754	63.47248	65.55439	72.04756	64.23546	72.72482
ODF2	11.68931	10.51123	9.169506	9.914859	9.387921	8.240567	9.73899	10.87496
ODF2L	3.559092	3.73318	3.610255	3.62743	3.182317	4.265032	3.033304	3.384148
ODF3	0	0.004999	0	0	0.004935	0.009937	0	0
ODF3B	0.028677	0	0	0	0	0	0	0.116343
ODF3L1	0	0.031161	0	0	0.030761	0.030967	0	0.031195
ODF4	0	0	0	0	0	0	0.050821	0
ODR4	6.687887	7.241837	7.184314	6.325102	7.886855	7.971659	7.23109	6.976206
OFCC1	0	0	0	0	0	0	0.010847	0
OFD1	4.940181	4.586615	4.532351	4.032474	4.270614	4.23647	5.172463	4.78132
OGA	33.32906	32.00474	29.98015	29.98925	31.28264	28.18767	27.5118	40.17475
OGDH	38.33256	37.26211	35.1998	36.84784	36.49048	36.15682	34.42994	38.30904
OGDHL	0.113439	2.911502	1.96418	0.240248	2.22369	1.515238	0.092112	2.400789
OGFOD1	8.398443	8.199881	7.407564	8.944834	9.127713	8.464341	8.679096	8.663974
OGFOD2	3.185468	3.142663	2.462424	3.936809	3.364861	2.702721	3.402783	3.896338
OGFOD3	8.055842	11.41739	9.670929	9.991022	9.989666	9.116537	9.741489	8.765142
OGFR	11.861	12.21576	10.47952	11.57943	11.60985	11.03755	11.41204	10.39265
OGFRL1	7.0888	9.149862	9.563018	8.775951	8.931013	6.940188	8.751135	10.84338
OGG1	3.718118	4.011066	3.246814	3.439779	3.580873	3.951491	4.070909	3.596462
OGN	18.73209	21.63799	28.40906	25.50262	25.60556	20.73039	32.01148	46.08118
OGT	29.62661	25.76505	23.14081	29.64133	28.1657	24.53495	24.41915	29.08389
OIP5	3.049667	4.188247	2.509723	2.430717	3.321178	3.411668	2.201182	4.330313
OIT3	0.012271	0.012432	0.012118	0.025149	0	0.086481	0.049819	0.02489
OLA1	31.0076	27.68601	35.2758	23.95168	26.41072	26.18142	26.73788	24.76958
OLFM1	1.615724	1.860609	1.684787	2.375636	2.378734	1.646971	2.088148	2.035684
OLFM2	0.682989	0.70552	0.357077	0.56267	0.495566	0.323602	0.43497	1.140937
OLFM3	0	0	0.005473	0	0.005543	0.011161	0	0.005621
OLFM4	0.060604	0	0	0.010351	0.020204	0	0.041009	0
OLFML1	11.49569	12.34552	11.90701	11.34474	12.03473	9.861919	12.62079	12.43229
OLFML2A	28.51959	23.81649	23.91499	25.00403	25.23055	24.30851	25.72637	24.05596
OLFML2B	2.014469	2.6895	1.854496	3.122794	3.022094	2.836085	2.599177	4.172866
OLFML3	25.88196	35.96773	24.12693	35.3015	30.20716	32.25446	44.14012	45.48522
OLIG1	0	0	0	0.054633	0	0	0.027056	0.229804
OLIG2	0	0.049316	0.04807	0	0.048683	0	0.024703	0.518382
OLR1	0.117987	0.013282	0.012946	0.026869	0.09178	0.026398	0.013306	0.132963
OMA1	4.371509	3.403245	4.373843	4.361904	4.164976	4.216053	3.619725	3.593664
OMD	0.097269	0.012318	0.156093	0.137058	0.145922	0.048967	0	0.110985
OMG	0.025538	0.068994	0.008406	0.087234	0.034054	0.077136	0	0.043169
OMP	0	0	0	0	0.056896	0	0	0
ONECUT2	0.010411	0.00211	0.012338	0.008535	0.012495	0.008386	0.004227	0.016895
OOEP	0	0	0	0	0	0.043826	0	0
OPA1	6.954457	7.717809	7.33202	8.004618	7.131108	7.271784	8.106665	7.111383

OPA3	2.804788	2.470955	2.332731	2.753924	2.651567	2.332827	2.585554	2.432438
OPALIN	0.011345	0	0.011204	0	0	0	0.023031	0
OPCML	0	0	0.004018	0.016678	0	0.004097	0.012389	0
OPHN1	5.80644	5.963412	5.937473	4.901822	6.132765	5.993262	5.718214	7.061527
OPLAH	2.366552	2.3273	1.864169	3.207486	3.074833	2.445606	2.373874	2.343931
OPN1LW	0.02533	0.051324	0	0.077871	0.126664	0.10201	0	0.07707
OPN1SW	0	0	0	0	0	0	0.024427	0
OPN3	0.026991	0.027345	0	0	0	0	0	0
OPN4	0	0.032187	0.015687	0	0.015887	0	0	0
OPRD1	0	0.023571	0.022976	0.143055	0	0.023425	0.023615	0.047195
OPRK1	0.022848	0	0	0	0	0	0	0.023174
OPRL1	0.154507	0.191977	0.095004	0.185222	0.227418	0.105665	0.168661	0.209928
OPRM1	0	0	0	0	0	0	0	0
OPTC	0.025013	0.025341	0	0	0.050032	0	0.025388	0.050737
OPTN	13.4221	14.83445	14.40207	14.1314	14.54388	14.47305	14.73468	13.50297
ORAI1	6.760926	6.976167	6.8	8.719908	7.689541	7.435739	6.844234	6.766708
ORAI2	1.425974	1.384991	1.111436	1.479411	1.479198	1.234004	1.300833	1.036895
ORAI3	2.566882	2.865034	2.506255	2.868247	4.177161	2.233988	2.737862	2.721092
ORC1	2.631621	2.599504	2.663801	2.667912	3.167753	2.573896	3.014521	2.81207
ORC2	10.00297	10.61968	9.97297	10.63601	9.340757	9.841237	10.22784	10.30238
ORC3	4.313691	6.086694	5.947048	5.274014	5.225509	6.170728	6.249691	5.530241
ORC4	7.526691	7.788708	8.427372	8.031562	8.718969	8.655732	7.300833	8.129922
ORC5	7.842125	6.625227	7.667986	8.366821	8.062437	8.752798	8.954727	7.077279
ORC6	4.466155	5.521751	4.634769	5.274936	5.034537	5.087329	5.743267	5.68136
ORMDL1	84.37395	86.61723	96.07017	80.07248	81.13281	95.64676	83.32074	81.31053
ORMDL2	66.75891	63.66339	69.11527	64.69063	66.45043	71.82948	63.63499	55.37192
ORMDL3	35.83684	51.99143	31.21119	38.93405	38.41306	33.49285	42.31446	42.06527
OS9	72.87804	76.12368	81.40966	82.10996	74.43914	78.69118	81.30597	75.13
OSBP	13.6301	13.16204	11.6131	13.66659	13.44535	12.68771	12.95698	13.1249
OSBP2	1.757318	1.391562	0.964123	2.256234	1.48819	2.41333	1.230123	1.720875
OSBPL10	3.657121	3.062897	3.019946	3.026721	3.580958	3.303374	3.994795	5.016227
OSBPL11	3.931367	4.48312	3.418636	3.899187	4.311214	3.255079	3.684799	4.591844
OSBPL1A	22.6374	23.49328	26.06626	23.31615	21.2219	25.23297	22.87854	23.29161
OSBPL2	18.79881	18.14018	18.68602	18.8864	19.15382	18.24213	18.40677	17.63624
OSBPL3	3.304034	4.381902	3.688456	4.328828	3.859734	4.018508	4.011703	3.488892
OSBPL5	3.775709	4.994522	4.121154	4.997109	5.847755	5.140494	4.088352	4.844963
OSBPL6	2.395342	2.255224	2.306271	2.396833	2.505026	1.726199	2.603144	2.400817
OSBPL7	0.986113	1.032356	0.687088	1.207033	1.123218	0.739121	0.889693	1.322421
OSBPL8	18.77588	21.76966	20.02187	19.38337	20.78798	18.98734	21.90909	23.88871
OSBPL9	26.42885	25.88762	26.67954	27.09249	26.94075	27.30663	28.01752	28.04977
OSCAR	0.478819	0.463053	0.472853	0.178429	0.391812	0.657396	0.44182	0.573933
OSCP1	3.543897	4.304628	4.158293	5.291319	3.753967	4.316259	4.428633	3.575022
OSER1	29.60802	26.16969	34.10527	27.85299	27.16905	29.45285	29.93683	28.48843
OSGEP	13.2876	10.13781	14.16034	14.71277	12.79981	14.29095	13.67091	14.35928
OSGEPL1	4.122074	4.138464	3.280461	3.756945	4.262199	4.684238	3.664459	4.058069
OSGIN1	0.838494	1.00504	0.921347	1.113412	1.098451	1.141483	1.018888	0.610873
OSGIN2	3.356665	2.447487	3.365066	2.779645	2.424572	3.763642	2.641297	2.037512
OSM	0.015379	0.01558	0.045561	0.01576	0.030761	0	0.015609	0.015598
OSMR	9.719814	8.623316	10.39592	10.69902	12.47375	7.876976	9.079222	10.35274
OSR1	3.441392	5.580999	4.119779	5.074297	4.853238	3.48983	7.023721	5.675011
OSR2	5.220148	8.459118	6.380607	7.478175	7.015059	5.771884	6.202062	5.403906
OST4	280.5647	303.3406	336.0609	290.7908	299.6742	322.6907	271.9066	276.0823
OSTC	50.39607	54.60861	57.66539	54.38494	47.15667	64.07244	64.48685	62.72661
OSTF1	28.34909	39.18859	40.51976	41.17952	35.06509	42.20661	43.49457	34.70074
OSTM1	5.789614	6.507146	6.432159	6.841519	7.201763	7.140728	7.419028	6.119759
OSTN	0.734574	0.591555	0.055802	0.366734	0	0.815448	0.764711	0.362964
OTC	0	0	0	0	0	0	0	0
OTOA	0.029408	0.014897	0.021781	0.022602	0.044118	0.037011	0.022387	0.029827

OTOF	0.14757	0.128742	0.190261	0.071412	0.094293	0.074289	0.108177	0.087308
OTOG	0.037058	0.021454	0.010456	0.059676	0.037063	0.029316	0.034927	0.010739
OTOGL	0.127897	0.170494	0.049856	0.103472	0.222165	0.084717	0.105902	0.139958
OTOP1	0.227925	0.13855	0.555209	0.109	0	0	0.215921	0.077056
OTOP2	13.8487	8.487945	16.01209	17.80107	11.63066	13.74396	10.03728	9.81835
OTOP3	0.561088	0.396594	0.99222	1.123229	1.735683	1.300633	0.83439	0.648481
OTOR	0.534825	2.34799	5.868453	1.705115	2.67447	2.453069	2.111072	2.350566
OTOS	0	0	0	0	0.032176	0	0.130616	0
OTP	0.028619	0	0	0	0	0	0	0
OTUB1	60.5145	56.45224	54.70896	57.92539	58.60788	56.76564	59.47931	52.94126
OTUB2	43.76729	36.8682	50.83117	50.71137	44.39884	47.2167	37.659	35.06798
OTUD1	7.422593	8.473248	10.67136	8.638828	8.431103	8.449328	8.160956	7.962025
OTUD3	12.68303	11.47359	9.686546	11.51493	10.7729	11.12605	11.33905	11.906
OTUD4	10.94821	9.897686	9.871301	10.31945	11.41415	10.75971	11.06885	11.44847
OTUD5	50.52875	47.37086	47.88806	46.85526	47.71645	47.90499	45.30062	47.86071
OTUD6B	13.88547	15.42698	17.29621	14.26255	14.87344	13.8013	12.99447	13.09945
OTUD7A	0.046294	0.046901	0.02743	0.056928	0.01852	0.055932	0.018795	0.028172
OTUD7B	6.964505	5.653816	5.94729	5.517219	6.223303	6.904365	6.326345	6.706616
OTULIN	5.674701	5.319706	4.163924	4.471499	5.556726	5.167161	5.318083	4.070113
OTULINL	10.05001	10.69823	10.20662	9.223094	12.25321	9.523447	10.3249	11.74374
OTX1	0	0.18246	0.125543	0.097707	0.042381	0.053332	0.032259	0.171916
OVAR	149.9474	2.636611	256.4738	355.2756	282.7858	209.028	154.2943	283.9796
OVAR-DRI	96.02734	142.5665	124.7321	132.8051	103.7152	100.839	98.25325	107.5328
OVCA2	0.395196	0.306175	0.229572	0.190581	0.116249	0.234056	0.21236	0.165044
OVGP1	0.330256	0.237001	0.149481	0.606365	0.137624	0.249384	0.23744	0.04187
OVOL1	72.37064	62.77804	52.64988	61.41195	61.87878	61.5992	64.70763	60.93958
OVOL2	3.578223	2.557645	2.991668	3.509384	2.327243	2.806986	3.208546	4.074457
OVOL3	0	0	0	0	0.037675	0	0	0
OXA1L	36.97367	37.36353	38.86394	37.83439	36.33278	36.80647	39.56237	37.47273
OXCT1	57.07796	58.90104	73.77288	62.9101	71.36656	62.61332	55.44493	52.60293
OXCT2	0.496659	0.292168	0.110752	0.344781	0.256374	0.306484	0.471586	0.731221
OXER1	0.028087	0	0	0.187091	0	0.01414	0.014254	0.014244
OXGR1	0	0	0.027259	0	0.027606	0	0	0
OXLD1	4.800592	4.55962	5.164059	5.62229	6.172987	4.186059	3.219397	3.477808
OXNAD1	13.222	13.01902	12.7482	14.23099	10.09383	15.5573	12.54681	10.81149
OXR1	31.80849	37.70058	36.70191	36.45119	35.62718	38.11613	32.86605	34.59723
OXSM	1.945675	2.513745	2.6618	2.981659	2.535061	2.821632	1.775563	2.679443
OXSR1	15.45554	16.23584	15.93249	15.56785	15.73956	15.85712	16.46078	15.88246
OXT	0	0	0.054518	0.339437	0.441701	0.444661	0.056034	0.167974
OXTR	0.064551	0.091557	0.012749	0.06615	0	0.064992	0.026208	0.06547
P2RX1	0.230901	0.183076	0.208195	0.370362	0.401618	0.141508	0.326072	0.570196
P2RX2	0.014731	0.014924	0.043643	0.015096	0.029466	0.014832	0.014952	0.014941
P2RX3	0	0	0	0	0.023445	0	0	0
P2RX4	8.977376	10.24152	9.174265	11.30516	7.891582	8.693945	8.824924	10.50945
P2RX5	1.460094	1.674861	0.875866	2.027991	1.822337	1.123813	1.365641	1.223868
P2RX6	0.028815	0	0.066398	0.068901	0	0.048353	0.019498	0.048709
P2RX7	0.567022	0.770025	0.595699	0.717062	0.567095	0.570895	0.379603	0.550622
P2RY1	7.898637	5.318845	5.941908	5.695981	7.686154	7.228186	4.943487	6.84831
P2RY12	0.179582	0.110463	0.107673	0.151168	0.096217	0.129149	0.123687	0.240683
P2RY13	1.805753	2.227158	2.626421	2.272874	2.591197	1.353686	2.460387	3.832128
P2RY14	0.908503	0.699524	0.818231	0.551156	0.995848	0.497602	1.09918	1.747043
P2RY2	1.191994	0.52331	0.38117	0.529326	0.56769	0.085724	0.120987	0.650537
P2RY4	0.025491	0	0.025173	0	0.025494	0	0	0.025854
P2RY6	0.251754	0.420096	0.175495	0.166935	0.222165	0.104372	0.285593	0.255338
P2RY8	0.055867	0.09905	0.068963	0.186064	0.069842	0.07031	0.127585	0.127489
P3H1	6.030274	5.570026	5.429367	6.43574	6.020187	5.316639	5.867077	7.129962
P3H2	9.204549	9.599624	7.847981	9.235664	7.921826	7.614416	9.085562	9.840447
P3H3	12.0395	11.87367	11.67905	12.87272	11.55089	11.62829	11.96055	11.89751

P3H4	9.897844	8.887147	8.616397	8.957262	11.49424	9.933975	10.15741	10.45108
P4HA1	9.024928	9.801101	8.397774	10.38228	11.74215	9.427214	8.269331	11.57391
P4HA3	5.025271	2.863035	2.366058	2.770036	3.969126	3.16689	4.177794	3.464335
P4HB	154.1929	158.1567	153.9758	164.687	154.8864	160.6811	180.7496	161.7987
P4HTM	4.776252	5.598632	3.799148	5.579466	4.890993	6.318837	5.443542	4.116782
PA2G4	211.5113	208.8459	204.693	202.4267	203.0324	206.3888	203.6953	191.8741
PAAF1	14.30479	12.31056	13.19003	12.20685	11.03579	12.31441	10.86703	9.932966
PABPC1	470.2824	548.8683	626.7831	533.4129	494.9865	545.4279	495.2309	489.0348
PABPC1L	0.151815	0.27965	0.068147	0.254578	0.138031	0.027791	0.252151	0.181972
PABPC1L2,	0.287605	0.596003	0.193651	0.4153	0.326866	0.329056	0.172497	0.13259
PABPC4	30.36686	32.28111	28.86282	28.94711	30.83392	29.45356	32.42224	32.13589
PABPC4L	0.614689	0.918301	0.658473	0.742018	0.729386	0.729029	0.766668	0.877041
PABPC5	0.786902	0.782601	0.84839	0.843384	0.714802	0.552414	0.75474	0.85668
PABPN1L	0	0	0	0.023112	0	0	0	0
PACRG	0.062198	0.105024	0.040949	0.127478	0.124412	0.041749	0.147306	0.084111
PACRGL	2.414253	2.533711	2.315726	1.349485	2.119965	2.08765	1.81147	2.13815
PACS1	8.954982	9.380161	7.656625	8.49483	8.64597	8.294085	9.227024	10.4717
PACS2	14.24565	14.05615	11.36064	14.58823	12.70071	12.24992	13.00168	13.79656
PACSIN1	0.43028	0.345552	0.191731	0.236601	0.346367	0.332839	0.287605	0.255457
PACSIN2	28.11838	28.93831	26.44945	26.71096	25.58766	25.02192	30.28885	32.03526
PACSIN3	18.0684	15.27688	17.69855	19.28183	14.83178	18.00003	19.41034	17.95405
PADI1	23.53584	16.8801	13.96149	15.54793	16.70028	19.05776	18.16985	22.07016
PADI2	1.204393	0.875741	0.591847	1.181077	0.691607	0.690439	0.707739	1.116334
PADI3	74.24291	59.71331	57.37563	61.03493	61.95056	58.95412	70.98056	72.40796
PADI4	13.17823	13.31896	13.67363	11.04927	9.224894	18.02903	16.59344	11.0002
PADI6	1.076506	0.657588	0.891122	0.876048	0.744152	0.828835	1.092654	0.915211
PAEP	0	0.469001	0	0.145967	0.071228	0.035853	0.108431	0.397282
PAF1	30.21839	28.32861	30.29031	31.19253	31.15814	29.09478	31.99854	29.47622
PAFAH1B1	40.69602	40.09336	39.75881	40.82594	42.57157	40.85487	40.528	41.74592
PAFAH1B2	27.46619	24.65432	25.32615	22.60406	24.69562	24.07462	26.70231	25.47365
PAFAH1B3	35.76698	39.59366	36.53836	39.17879	36.70287	36.67309	36.97062	32.27632
PAFAH2	9.758631	10.76347	11.08765	13.64109	8.154122	10.42146	10.51757	12.39215
PAG1	3.771078	3.460987	2.749255	2.790388	3.433234	2.172016	2.797558	3.537905
PAG3	0	0	0	0	0	0	0	0
PAGE4	0	0.11186	1.36294	0.339437	0.165638	1.667478	0.448268	0.111983
PAGR1	12.04212	10.75509	9.975488	13.13115	12.93234	12.76004	11.29715	11.09891
PAH	0	0.014115	0.027517	0	0.069668	0	0	0
PAIP1	25.77598	25.46672	28.02666	27.84174	28.59385	27.20069	26.14325	21.92444
PAIP2	117.8585	136.3831	138.1631	117.319	131.6727	136.2316	127.1193	125.0616
PAIP2B	7.262794	6.863521	9.011892	7.986856	7.957557	8.273889	7.884628	7.288006
PAK1	15.17339	17.90982	13.06545	14.7616	15.24445	14.04236	14.59457	16.76454
PAK1IP1	11.31452	13.88324	12.86365	12.89798	12.43331	13.01921	12.22009	12.53635
PAK2	15.0112	14.84502	15.01499	14.66841	15.33525	15.04326	14.07943	15.69925
PAK3	2.165458	1.995294	1.530116	1.865189	2.231083	2.167721	2.040042	1.991172
PAK4	20.01546	20.0357	18.49165	22.53435	20.99245	22.59593	20.1769	18.36743
PAK5	0.030578	0.123918	0.161051	0.229794	0.224268	0.112886	0.020691	0.010338
PAK6	6.366916	4.116017	5.000126	5.861807	5.114406	5.347097	4.600627	6.257676
PALB2	5.970278	5.483339	4.477017	5.038908	5.245595	4.536387	5.387304	5.878421
PALD1	1.18303	1.394582	1.020888	0.855432	1.271645	0.857155	1.290553	1.676456
PALLD	42.3759	34.10251	34.95224	40.3186	41.30428	40.07836	41.09265	45.51397
PALM	12.72977	12.85174	11.97776	13.56202	14.18558	12.74951	12.54423	13.19689
PALM3	0.644664	0.500729	0.838231	0.594569	0.795188	0.486801	0.436223	0.457689
PALMD	48.93719	57.76652	56.7408	64.04124	69.00459	66.59095	48.57761	58.91001
PAM	39.18942	41.64278	40.56095	35.01363	48.7347	34.30822	45.74502	49.80255
PAM16	20.0377	22.64837	22.88411	19.50966	19.67674	20.99804	18.81635	21.29071
PAMR1	8.280482	6.272713	6.067636	4.765881	4.963259	4.634867	5.842986	7.38212
PAN2	8.238251	7.643058	8.123467	7.97015	7.563458	7.341989	7.457675	8.897609
PAN3	16.5013	16.02846	14.45761	15.27441	14.91214	15.94906	15.23543	15.78217

PANK1	5.090908	5.272947	6.155982	7.320467	6.467373	6.679752	5.029845	5.597324
PANK2	14.72174	15.12174	12.77456	15.37401	14.39191	15.15626	15.87486	17.00151
PANK3	26.81238	27.78537	37.07657	31.7659	31.18531	33.75685	26.98569	26.84387
PANK4	8.733617	8.184619	7.628837	8.65162	7.538918	7.421948	8.854069	8.668133
PANX1	11.17211	9.661771	9.27909	9.75891	10.20844	10.08983	9.297993	10.02159
PANX2	4.150151	2.773468	2.732293	4.242968	2.864559	2.55026	3.470783	3.11246
PAOX	5.028132	4.089317	4.293546	4.939955	5.236392	4.516755	3.979835	4.65524
PAPLN	13.37825	13.53971	9.765959	12.74618	10.79592	10.60182	12.59638	14.72002
PAPOLA	41.36407	41.29079	42.39107	40.73618	46.39199	42.25896	38.17328	46.35007
PAPOLB	2.362554	3.498783	2.715594	2.831203	2.821228	3.412061	2.87628	3.024694
PAPOLG	5.471409	5.784962	4.774855	4.937708	6.016397	5.327549	6.016624	6.275334
PAPPA	1.26101	0.883645	0.583733	0.893803	1.195487	0.751194	0.770622	0.91126
PAPPA2	0.144147	0.06676	0.048806	0.105512	0.354235	0.041466	0.087785	0.154553
PAPSS1	37.99446	35.01893	37.65616	33.80903	35.74147	33.85433	40.93396	37.28638
PAPSS2	2.302072	2.827011	2.686731	3.510842	3.317893	3.355735	3.571777	2.193338
PAQR3	3.494802	4.553029	3.684039	3.738908	4.448505	4.189393	3.562828	4.698352
PAQR4	3.569972	2.682972	2.138412	2.099083	2.560762	2.150724	2.613688	2.760112
PAQR5	9.901355	6.689647	6.706138	7.395099	8.500492	8.677186	8.271136	8.252212
PAQR6	1.699199	1.584389	1.574078	1.756698	1.40365	1.730994	1.373644	1.53529
PAQR7	15.29124	13.58683	23.753	29.99536	32.46708	28.99177	9.868169	7.943081
PAQR8	3.34723	3.100658	2.427132	3.640029	3.031625	3.517391	3.777572	3.946845
PAQR9	0.01314	0	0	0	0	0	0.013338	0.013328
PARD3	19.23287	18.26401	18.04253	19.62349	18.86698	17.51423	18.2426	20.4491
PARD3B	4.263944	3.793182	2.352539	3.967993	3.372017	3.449303	4.524805	3.608461
PARD6A	0.314662	0.207214	0.201982	0.274088	0.534994	0.190087	0.271474	0.159571
PARD6B	7.009944	7.324146	6.839295	7.684964	7.727965	7.168245	6.926728	7.255181
PARD6G	10.89246	9.508392	9.71828	10.47081	10.62218	10.34927	11.08253	11.87409
PARG	7.642449	7.662147	5.956266	6.140055	7.364951	6.558232	7.11613	9.516329
PARK7	84.77387	92.02684	90.85887	83.56834	91.26271	81.79118	64.02586	69.09586
PARL	30.78201	37.02952	32.8767	35.71309	33.7816	34.61927	35.3726	32.53388
PARM1	8.089288	9.49431	8.089331	9.475561	10.83061	8.955579	8.596401	9.821169
PARN	6.814842	6.80535	6.202091	6.672913	5.669489	6.581975	6.317169	6.176885
PARP1	26.64736	26.21117	24.7487	26.99947	25.79809	22.45283	25.80573	26.04147
PARP10	3.2943	3.028039	2.755888	3.122074	3.236924	3.1838	3.18449	3.424936
PARP11	2.484953	2.667336	1.981267	2.712426	2.766892	2.728733	2.808033	2.427512
PARP12	3.38159	3.988181	2.491747	3.145475	3.538121	2.959463	3.969164	3.561642
PARP14	2.636901	3.321334	2.303759	2.69735	3.829941	1.808213	3.163522	3.291247
PARP16	10.87707	8.237641	6.987312	10.49554	8.44475	7.960168	8.689346	8.811656
PARP2	17.57596	25.03358	18.31559	19.12667	19.54935	13.4016	17.85453	19.70574
PARP3	0.312728	1.016504	0.437511	0.200297	0.104256	0.341103	0.224838	0.832597
PARP4	10.7196	9.554725	8.944633	10.70335	10.97957	10.43335	10.80542	10.44348
PARP6	7.16905	5.288471	4.498573	6.420648	7.110227	5.639082	6.329113	7.301402
PARP8	1.298722	1.373644	1.105767	1.455784	1.988329	1.560677	1.256351	1.618507
PARP9	4.17168	3.90555	3.815861	4.571761	5.059155	4.455287	5.033344	4.946951
PARPBP	2.608699	3.000833	3.206825	2.617623	2.255729	2.735959	2.496131	3.376196
PARS2	1.733794	1.685411	1.22147	1.4225	1.62598	1.490047	1.606298	1.402398
PARVA	30.83852	31.31496	29.07356	30.23311	30.16105	28.53675	29.01976	32.30066
PARVB	0.886626	0.973117	0.908744	1.073786	1.363699	0.473392	1.445334	1.185372
PARVG	1.360037	1.617518	1.10951	2.666257	2.089602	1.329637	2.120672	4.218159
PASD1	0	0	0	0	0	0	0.043011	0
PASK	1.007548	1.167343	0.739867	1.011326	1.038684	0.858365	0.886306	1.037612
PATL1	9.845991	8.343808	7.234821	8.01144	8.703948	7.147196	7.972487	9.225661
PATL2	0.320718	0.360055	0.350962	0.57738	0.485474	0.471273	0.325529	0.219786
PATZ1	8.000825	7.355031	7.087981	7.67866	7.583237	7.710051	6.644322	6.973372
PAWR	28.32963	26.08567	28.58371	24.08862	27.21718	26.15219	24.66529	24.60835
PAX1	0.054697	0.071247	0.084881	0.016015	0.148482	0.070805	0.063448	0.118876
PAX2	0.00368	0.018641	0.003634	0	0.007361	0	0	0.007465
PAX3	0	0.044238	0.034497	0.134239	0.017468	0.114304	0.035456	0.026572

PAX4	0	0	0	0.027956	0	0	0	0
PAX5	0	0.079153	0	0	0	0.019665	0.019825	0
PAX6	0.007945	0.004024	0.015691	0	0.003973	0.011998	0	0
PAX7	0	0	0	0	0	0	0	0
PAX8	0.411278	0.630645	0.625696	0.444248	0.478034	0.57077	0.913872	0.800445
PAX9	0.294223	0.586553	1.424667	1.206045	0.70243	1.05115	0.799575	0.442804
PAXBP1	13.45543	13.52514	11.06543	14.9598	14.50045	12.61508	10.26674	13.49838
PAXIP1	5.798883	5.84132	4.889785	5.738196	5.999047	4.960214	5.405215	5.443275
PAXX	12.26558	14.61243	10.06963	10.93386	17.73566	15.71197	13.22954	12.1704
PBDC1	15.90631	18.1271	18.56747	17.72683	16.44669	19.41605	17.82156	13.98673
PBK	5.107918	5.49927	5.676525	4.635406	3.827954	4.68038	4.294548	5.1524
PBLD	3.346333	3.436313	3.304637	4.006972	4.256213	3.909363	2.464329	2.582369
PBRM1	13.9979	13.17031	11.47208	12.62844	12.84219	12.2638	13.38656	13.24194
PBX1	3.614026	4.210675	3.192803	4.05138	4.010133	3.655925	4.10198	3.846271
PBX2	23.77646	19.89794	19.62186	23.17113	19.67138	20.69767	20.08022	22.81666
PBX3	4.228922	3.104514	3.733153	3.110854	3.360661	3.114066	3.28467	4.395626
PBX4	0.194511	0.150391	0.111209	0.110156	0.127985	0.123689	0.10391	0.192089
PBXIP1	14.71383	16.14224	12.92727	16.5384	15.98315	14.504	14.56604	17.38967
PC	2.468929	1.853286	2.314191	3.056931	2.439354	2.425607	2.396744	2.425255
PCBD1	20.22168	15.44001	15.72979	15.21447	16.58589	16.10307	15.53513	13.16334
PCBD2	20.3076	22.89988	22.10361	23.79649	23.18014	25.20232	21.19473	18.17882
PCBP1	217.9648	205.7936	217.9145	216.5984	221.2083	221.2367	211.4795	214.8077
PCBP2	172.1622	172.8194	167.6596	159.9654	147.1461	152.3683	173.6644	155.2945
PCBP3	1.826548	1.836913	1.432421	2.559951	1.611868	1.514492	1.417724	1.838929
PCBP4	9.614693	8.171792	8.234617	11.36379	10.27269	9.168913	9.821793	8.922179
PCCA	15.51852	15.02091	14.47621	14.88468	14.96224	14.27566	14.33482	12.6142
PCCB	16.61039	20.63134	22.009	19.26014	21.37393	20.71389	19.54115	17.17848
PCDH1	8.867337	7.185458	6.58789	7.355948	7.992077	7.561321	7.698363	7.836281
PCDH10	0.030456	0.037027	0.027069	0.037453	0.042644	0.027598	0.021639	0.055601
PCDH11X	0.037987	0.023092	0.037514	0.007786	0	0.015299	0	0
PCDH12	1.349615	1.740234	1.130859	1.62403	2.080925	1.425747	1.286837	1.441416
PCDH15	0	0	0	0	0	0	0	0
PCDH17	0.134055	0.216781	0.056009	0.134734	0.221735	0.212839	0.065416	0.107202
PCDH18	4.876237	6.363596	4.254033	5.848534	5.54935	5.041645	5.193521	4.751598
PCDH19	2.289462	2.194663	2.308972	2.612029	2.841117	2.853626	2.557501	2.233251
PCDH20	0.802055	1.193075	1.96758	2.048282	0.757594	1.442025	1.066064	1.239577
PCDH7	3.981554	4.009049	3.658744	3.721548	4.403447	3.519594	5.080123	4.379298
PCDH8	0.007018	0	0	0.014384	0	0.007066	0	0
PCDH9	0.270101	0.216636	0.155595	0.269102	0.273887	0.234175	0.217037	0.239702
PCDHAC1	0.011392	0.057706	0.022499	0.035021	0	0.011469	0.173437	0.115538
PCDHB1	0	0	0	0	0.011393	0	0.011562	0
PCED1A	0.028387	0.028759	0.056066	0.02909	0	0.028581	0	0
PCED1B	0.309809	0.262343	0.246586	0.180065	0.351471	0.256058	0.356697	0.206353
PCF11	20.24568	20.12151	17.8847	19.7228	21.24804	18.64044	19.71101	19.99603
PCGF1	11.22592	11.98637	12.81918	11.81408	10.83396	9.109115	12.5921	10.92539
PCGF2	7.009919	6.698205	6.772927	7.655176	7.516009	6.814825	5.821937	6.011092
PCGF3	9.599959	9.193486	7.404274	9.069985	9.019648	8.26947	8.671385	9.169549
PCGF5	12.69976	13.54216	13.61689	12.76326	12.75246	13.76299	12.48615	12.6148
PCGF6	5.590509	6.059608	6.34054	7.280068	6.079549	6.833095	6.442512	6.771924
PCID2	9.957874	8.912902	8.198096	9.503227	8.818421	8.577938	9.412621	8.681257
PCIF1	8.224657	9.449649	6.540412	10.03161	8.516303	8.160832	8.809286	8.575974
PCK1	0.142129	2.201048	0.010026	0.728245	0.852881	0.153321	0.103043	0.453049
PCK2	7.190692	7.182275	7.814961	7.355811	9.157585	8.797588	7.929291	6.14829
PCLAF	14.14394	15.32824	15.57539	14.00451	13.77233	16.54131	15.84173	15.55714
PCLO	0.002655	0.008071	0.010489	0.005442	0.014607	0.00401	0.012129	0.005386
PCM1	14.29869	14.48635	11.5567	12.7484	13.32896	13.48065	13.90155	14.5308
PCMT1	33.3948	32.17488	38.45043	34.12967	34.8408	32.73096	33.75855	34.61643
PCMTD1	29.95475	31.72577	35.49496	34.1934	31.0593	33.22226	28.56997	29.18698

PCMTD2	18.86446	18.95428	17.72216	17.0898	14.84902	17.77833	19.3845	20.94919
PCNA	39.6104	44.88396	44.06033	42.95381	37.51469	42.95722	48.62203	40.38179
PCNP	19.55113	21.24123	19.81293	20.19408	20.78382	21.24353	21.07103	22.15437
PCNT	4.94706	4.644787	3.843695	4.689866	4.287646	4.349058	5.00754	5.335707
PCNX1	8.293173	6.407751	6.021463	6.743165	7.295842	6.585015	6.355396	7.56274
PCNX2	0.070217	0.106707	0.077046	0.075954	0.050719	0.047131	0.166297	0.05539
PCNX3	6.921371	6.554982	5.802155	6.58279	6.825244	6.017419	6.468656	7.10118
PCNX4	6.646503	6.944586	5.03879	7.003778	5.761048	5.549553	5.492362	6.31214
PCOLCE	51.60683	54.30122	55.13835	60.46973	66.67765	54.79194	63.08556	72.02258
PCOLCE2	6.116363	9.940736	10.00091	7.985292	12.37757	6.114881	9.624749	8.309986
PCP2	0.07434	0.037658	0	0	0	0	0.037728	0
PCP4	11.77206	17.8156	23.92608	30.33434	11.48046	14.8033	25.68213	9.115742
PCP4L1	3.383142	3.095357	3.031909	3.176759	3.815843	3.121149	2.662398	3.189449
PCSK1	0.075632	0.109463	0.058685	0.02768	0.037821	0.032635	0.08225	0.082188
PCSK1N	7.33724	8.438066	6.546414	7.664141	7.111527	7.501456	8.971264	6.033803
PCSK4	0.404888	0.589007	0.512619	0.914945	0.508771	0.637612	0.421499	0.44224
PCSK6	14.02492	11.90003	13.63709	13.07329	15.53731	13.0481	12.20266	12.50805
PCSK7	10.97187	11.56491	9.478969	11.19152	11.58372	11.4053	12.13209	11.48917
PCTP	25.37607	31.72456	35.55406	27.52208	34.7006	33.82229	26.52293	17.59502
PCYOX1	30.06699	38.49505	33.92407	37.59101	36.7907	35.2385	31.78602	32.97663
PCYOX1L	2.883916	4.613312	2.668109	3.561976	4.447877	2.934182	3.096643	2.817208
PCYT1A	14.20151	15.79468	15.09468	13.84459	15.83716	14.81389	13.96131	14.45338
PCYT1B	0.223709	0.2914	0.170425	0.15065	0.2557	0.199496	0.188139	0.246341
PCYT2	14.83902	15.26776	18.26064	18.37216	17.52617	19.64923	15.85278	12.40036
PDAP1	168.9301	162.9748	168.2051	166.4538	146.6339	170.1907	166.8438	144.3001
PDC	0	0	0	0	0	0	0.038495	0
PDCD1	0.384329	0.338585	0.165017	0.102743	0.284106	0.555195	0.322251	0.440643
PDCD10	32.60109	36.10662	38.72658	36.50753	35.97494	39.24677	38.8504	36.51036
PDCD11	13.91635	12.08141	11.66863	10.79662	12.48602	12.10663	12.66686	13.28731
PDCD1LG2	0.393422	0.546631	0.333017	0.403167	0.775704	0.407427	0.581871	0.820847
PDCD2	6.88964	7.759595	7.096581	7.226679	6.841111	7.476861	6.649068	6.329041
PDCD2L	5.597833	4.653373	4.866603	5.613919	4.091253	4.407699	4.540584	5.022423
PDCD4	158.9074	211.6375	171.6972	181.8606	158.5604	212.9499	214.2921	171.8165
PDCD5	43.22669	55.12726	55.00806	48.02426	46.77741	56.26805	48.6878	42.86155
PDCD6	21.85904	27.51663	23.6764	25.21732	28.75642	26.73072	25.84046	24.16155
PDCD6IP	56.03837	49.58175	45.70247	50.31738	53.23248	47.95779	48.48751	52.02053
PDCD7	6.021478	7.073048	5.029803	6.60965	5.808026	6.014689	6.715733	6.50147
PDCL	6.247367	7.650337	6.588198	6.861167	6.976195	7.570606	7.234187	6.944782
PDCL3	35.14272	37.73829	36.32806	33.71198	35.05465	37.45729	37.80818	34.60987
PDE10A	0.196035	0.349787	0.184924	0.23987	0.371637	0.282805	0.380133	0.370944
PDE11A	0.033123	0.033558	0.073599	0.050916	0.091101	0.041687	0.058835	0.033595
PDE12	4.997896	4.891196	4.902966	4.937741	5.282014	5.09387	4.818742	4.479732
PDE1A	0.832191	1.279833	0.91642	0.908022	1.143662	0.861988	0.789983	0.977627
PDE1B	0.90402	1.629735	1.115945	0.958201	1.152331	0.593412	0.863605	1.186562
PDE1C	1.063411	1.072642	0.681683	1.419545	0.951596	1.056588	0.951543	1.116393
PDE2A	3.625715	4.662	3.406531	4.229448	5.347774	4.332818	4.26479	5.230743
PDE3A	1.482209	1.186041	0.961406	1.179722	1.32418	0.948331	1.245055	1.402101
PDE3B	0.680647	2.147548	0.542534	0.792168	1.22046	0.621662	0.552685	1.262329
PDE4A	3.822118	2.846619	3.464408	3.228858	4.012118	3.030617	3.313468	4.101652
PDE4B	2.190456	2.479481	1.328609	3.034522	3.299632	2.382399	3.218314	3.202177
PDE4C	0.327514	0.547042	0.375882	0.571472	0.681671	0.436698	0.566024	0.493776
PDE4D	1.416256	1.595011	1.342231	1.6651	1.405459	1.487832	1.609108	1.483184
PDE4DIP	1.776332	2.152199	2.246093	1.971832	2.281551	2.354089	1.664464	2.136106
PDE5A	3.840454	3.330603	3.118444	3.794887	4.157533	3.732296	3.952254	4.16202
PDE6A	0.738647	2.510564	0.33727	1.497609	1.255068	2.630915	1.362407	0.499441
PDE6B	0.251104	0.626914	0.726214	0.303275	0.735472	0.207673	0.68269	0.472975
PDE6C	0.01673	0	0.016521	0.025716	0.033464	0.033688	0.00849	0.016968
PDE6D	9.257398	11.47937	10.38004	9.783167	10.70525	8.54392	8.784523	10.31832

PDE6G	0	0	0	0	0	0	0.104701	0.034874
PDE6H	0.042026	0	0	0.043067	0.042031	0	0.127968	0
PDE7A	14.20144	15.05503	16.25907	17.44309	14.28772	18.02184	15.40856	14.77183
PDE7B	1.078028	0.871737	1.025528	1.094596	1.117733	1.155097	1.08416	1.94099
PDE8A	1.58839	2.30699	1.966875	2.918477	2.097442	2.14898	2.374239	2.630463
PDE8B	5.785553	4.77975	4.01659	4.20364	4.624074	3.310044	4.70795	5.092241
PDE9A	2.591432	3.328992	2.73477	2.915978	3.108427	3.052519	3.386731	3.092145
PDF	3.684537	3.575482	4.493485	4.230721	4.772758	4.022575	4.010153	3.534381
PDGFA	17.5733	16.50602	14.31068	20.68182	15.72795	14.96411	16.78157	14.69753
PDGFB	9.426583	8.478686	9.906012	9.45759	8.416435	6.612089	7.880903	8.016434
PDGFC	7.187392	5.635418	5.822283	7.237332	6.344475	5.747244	7.295203	8.515226
PDGFD	13.0054	15.40909	10.86342	13.76296	15.36416	13.44851	12.01453	15.0826
PDGFRA	20.27855	21.14108	16.19479	21.13028	19.06829	17.67386	21.07249	21.97014
PDGFRB	15.59911	14.79635	11.38634	15.54483	16.15485	14.30396	15.34206	13.76038
PDGFRL	12.64126	14.14709	11.73958	13.8842	12.98127	12.60375	14.5323	12.11933
PDHA1	36.57526	37.5087	38.377	33.96032	36.44915	37.3104	36.21536	35.55958
PDHB	48.5641	51.9012	58.23463	47.66788	48.42534	52.2359	54.84925	50.02764
PDHX	8.966176	11.16581	9.058528	10.03533	9.231418	8.842488	10.02122	9.268473
PDIA2	0.06438	0	0	0	0	0.048615	0	0
PDIA3	228.4152	260.8285	261.0687	279.1357	274.6328	264.0467	259.1722	250.0846
PDIA4	40.52246	36.76474	36.2341	38.92483	41.68939	36.01769	41.60569	44.092
PDIA5	6.934735	7.387899	5.965811	8.553456	8.13328	8.799619	9.016139	8.86433
PDIA6	110.5178	113.4106	110.3971	121.0468	117.0456	120.7425	118.1095	115.9314
PDIK1L	2.814837	3.155023	2.911834	3.099925	3.350217	3.180311	3.147937	3.248912
PDK1	7.940088	10.01579	7.308123	9.432245	8.928426	8.308956	10.82728	9.018342
PDK2	5.121422	6.433002	4.939989	6.674776	5.017855	4.459394	6.565804	5.888919
PDK3	24.9483	23.86928	21.90865	25.09208	25.25565	22.60287	19.82055	19.68494
PDK4	15.21963	20.56948	15.09029	9.487819	15.58802	13.05657	22.00206	35.02196
PDLIM1	39.60047	32.02661	45.09455	47.04862	49.13992	39.62145	44.58743	56.69787
PDLIM2	37.30125	38.04094	36.62747	44.94851	35.84205	39.04766	41.96011	30.55201
PDLIM3	6.014308	6.958086	9.873401	8.443036	9.31368	6.940915	7.338555	6.808303
PDLIM4	14.91342	17.9973	12.19156	20.57025	19.27449	13.71065	17.31323	14.60002
PDLIM5	15.7319	14.76006	15.61102	13.7156	15.66509	13.77829	14.56674	15.39729
PDLIM7	45.62526	40.85166	37.94323	44.39926	42.69365	40.61132	41.5883	41.39523
PDP1	5.190119	6.598681	8.091929	7.756893	6.701457	6.443352	7.613774	6.271882
PDP2	1.873223	2.305311	1.912301	2.361254	2.201034	2.267852	2.082121	1.864922
PDPK1	13.9631	13.38481	12.28895	11.87709	13.33514	12.25957	12.24719	12.90056
PDPN	19.82116	16.97673	23.41017	23.78411	22.80214	18.97884	20.85225	23.33168
PDPR	5.747673	6.401966	5.668583	6.037393	5.241315	5.577405	6.578967	5.729715
PDRG1	5.706793	5.836884	5.205275	4.996751	4.209134	4.443037	5.018232	5.843289
PDS5A	23.45038	23.87122	22.93367	22.0403	22.14287	23.90608	22.6735	23.98545
PDS5B	6.872	7.662254	7.210054	6.583116	7.605744	7.102433	7.333469	8.194361
PDSS1	2.888054	3.305247	2.658409	2.466327	2.157406	2.979581	2.750426	2.730273
PDSS2	3.907578	4.103537	3.064035	3.206157	4.141789	3.372234	3.808071	3.77887
PDXDC1	14.44014	14.73628	15.56289	15.58877	15.20691	14.65182	17.11377	16.24691
PDXK	6.102801	8.191777	6.414038	7.658291	7.619572	6.982983	7.513716	9.912424
PDXP	5.776013	5.370434	4.618955	5.751709	5.435203	4.574639	4.883033	5.19561
PDYN	0.216133	0.07299	0.142293	0.369144	0.432321	0.036268	0.255937	0.438419
PDZD11	40.6022	47.51138	49.82349	42.84017	50.22837	45.9541	43.834	41.9327
PDZD2	13.92412	13.28597	12.583	13.88094	12.79626	12.2081	12.25797	11.85348
PDZD3	0.477718	0.32842	0.370673	0.489551	0.528971	0.463804	0.467567	0.674865
PDZD4	0.086751	0.10387	0.023365	0.056573	0.078875	0.031761	0.032019	0.087986
PDZD7	0.042775	0.07945	0.028161	0.05114	0.071301	0.143558	0.079597	0.007231
PDZD8	10.98361	12.28586	11.24349	10.51719	11.57322	13.15612	11.16098	12.43796
PDZD9	0	0.025455	0.049624	0.051495	0.175897	0.126483	0.127509	0.178378
PDZK1	50.25703	31.11508	43.79574	30.01548	40.04324	43.84107	38.84839	26.09558
PDZK1IP1	25.78814	32.35037	23.39054	29.59142	27.4671	32.8771	27.8755	21.92376
PDZRN3	29.55511	19.96635	20.92362	21.76975	22.27461	23.65401	23.59703	22.54515

PDZRN4	0.171051	0.3918	0.238691	0.209584	0.353303	0.419317	0.279297	0.241372
PEA15	62.13509	62.58172	66.73246	61.57842	73.98704	58.63544	63.92569	63.84497
PEAK1	3.903143	4.077711	3.38562	3.787732	4.295717	3.591496	3.632991	5.153975
PEAK3	0.013097	0	0.012934	0	0.026198	0.013187	0.026588	0.013284
PEAR1	4.299996	3.352408	3.638206	5.031911	5.577278	4.532311	4.915741	5.565084
PEBP1	86.13793	99.10432	85.73561	85.91201	61.32741	57.38687	90.22684	75.51525
PEBP4	0.639123	0.446559	0.522339	0.158092	0.41879	0.6213	0.223693	0.201172
PECAM1	40.0895	38.38219	44.51655	56.08476	72.14593	56.221	52.15223	60.44374
PECR	6.089459	6.225977	6.215875	7.385306	7.226347	7.40602	7.371597	6.874976
PEF1	27.51584	34.41462	30.88123	29.00583	32.2059	29.86421	28.16879	31.09195
PEG10	6.330039	6.437044	4.256857	2.166313	4.275691	2.580228	4.435161	7.150803
PEG3	1.827812	1.607126	1.995834	1.906954	1.894153	1.382144	1.535273	1.750698
PELI1	24.64009	22.74689	24.78757	23.87908	24.08385	24.08841	24.55693	24.15065
PELI2	3.401775	2.844826	3.235151	2.934254	3.679023	2.979155	3.294464	4.083079
PELI3	1.224217	0.520119	0.867724	1.00161	0.878786	1.153059	0.86179	0.851128
PELO	9.108612	8.92492	9.298683	9.558059	9.012705	8.674017	9.483352	7.991194
PELP1	10.91327	11.0966	10.61439	11.23092	11.72635	10.51616	11.47879	10.03805
PEMT	1.103911	0.803214	0.981141	1.357506	1.094017	0.767913	0.672282	1.007663
PENK	11.62437	2.401323	2.747758	5.005703	8.636948	4.005016	7.886741	12.14521
PEPD	21.00997	25.98001	23.53654	24.49806	22.79392	25.6437	27.0128	25.58262
PER1	6.664337	7.269118	5.151712	6.980461	5.854424	5.951958	7.213119	9.49813
PER2	4.364744	3.675107	3.221494	3.523464	4.397934	3.619437	4.046131	5.161383
PER3	3.007318	3.153691	1.255672	2.05454	1.915435	1.790158	2.254513	2.547126
PERM1	0.139835	0.186409	0.145361	0.377103	0.272346	0.222301	0.186754	0.238865
PERP	901.2538	858.977	989.7676	877.6357	834.9628	931.7722	906.8643	877.6965
PES1	36.49496	30.07507	31.51725	30.22213	30.57394	29.4613	35.45591	33.4261
PET100	61.08746	64.96488	78.53388	62.82826	60.50292	69.25811	64.33361	56.47482
PET117	3.083144	3.099204	2.687923	3.505086	4.1676	2.594923	3.471668	3.786643
PEX1	4.886691	4.664197	4.908348	5.870911	6.08343	5.366765	5.123149	5.367104
PEX10	4.061594	4.732134	3.468324	4.0153	3.727592	3.596215	4.643896	3.283415
PEX11A	2.511716	2.11709	1.77899	2.278563	2.975327	2.839802	2.413564	2.171615
PEX11B	16.31687	20.40822	19.10264	18.87802	17.78038	17.18145	17.65631	17.02551
PEX11G	1.692178	0.981624	1.617187	1.762063	2.142792	1.264064	1.205062	1.647063
PEX12	4.922369	4.736116	4.186163	4.475926	4.328679	4.15825	5.146995	5.112984
PEX13	9.035932	9.907691	11.02741	9.094823	10.72341	10.80299	9.334831	9.343348
PEX14	4.865883	5.229996	3.831951	4.541449	5.35592	4.711764	4.86192	4.998064
PEX16	9.5985	10.96556	11.11742	11.52066	11.13508	9.991933	10.34056	9.058871
PEX19	39.9468	48.71304	46.21636	50.6904	55.29392	53.05658	43.91526	41.48468
PEX2	9.187516	10.86716	10.15639	9.446321	9.554423	10.41615	7.005595	8.978334
PEX26	9.485437	9.489599	10.42282	10.33705	8.726463	10.80978	9.105346	9.692742
PEX3	16.64991	17.53161	21.85376	23.41337	23.938	22.71651	16.24596	15.75186
PEX5	9.112751	8.66323	8.321183	9.603463	8.989081	8.411918	7.520829	7.108206
PEX5L	0.186715	0.079327	0.05948	0.13579	0.036143	0.036385	0.110041	0.042762
PEX6	5.343095	5.015545	5.490111	4.958274	5.888579	5.218289	5.626511	5.825391
PEX7	17.92967	22.71579	18.37724	20.94613	17.96974	19.62935	20.61233	20.71166
PFAS	4.46461	3.957803	3.066835	3.73417	3.769143	3.867117	3.372028	4.847802
PFDN1	53.20529	61.17061	57.36208	56.68292	50.95648	64.36442	54.0409	48.67519
PFDN2	55.43397	58.96496	54.78805	50.43837	57.02906	57.91364	56.21944	47.11331
PFDN4	23.67663	30.29833	34.62707	29.89562	26.94975	38.53872	29.38172	26.92963
PFDN5	247.4385	291.1176	290.4227	252.79	251.2654	276.1856	261.7525	257.2089
PFDN6	52.77722	48.9794	55.72664	46.38821	46.25787	59.91428	52.06929	49.57425
PFKFB1	0.047259	0.143638	0.011668	0.036322	0.070898	0.011895	0	0.083881
PFKFB2	2.165136	2.363696	1.962105	2.700648	1.917349	2.182529	2.281532	2.421319
PFKFB3	4.598663	4.181168	2.218588	3.701374	4.146159	3.04298	3.779468	4.903314
PFKFB4	1.821157	2.107466	1.087072	3.334399	2.120911	1.866196	2.234601	1.855292
PFKL	15.52655	14.30301	11.38306	14.51791	17.11491	15.3152	19.50931	15.5977
PFKM	32.39111	31.28497	31.27854	15.90459	25.7065	29.67356	30.69093	30.24626
PFKP	13.37237	10.34433	14.70663	13.4841	14.84311	9.766594	13.91461	13.08685

PFN1	448.9582	439.2053	454.2428	436.41	468.3882	432.8251	436.6068	447.5521
PFN2	13.09171	21.93996	14.76428	12.96698	16.73193	12.24536	17.72193	22.48923
PFN3	0	0	0	0	0	0.036598	0	0
PFN4	0.077063	0.218607	0.152205	0.071074	0.069365	0.108625	0.093862	0.101608
PGAM1	93.11031	80.74579	104.4245	94.42708	111.5505	96.88278	93.40033	101.4182
PGAM2	1.149855	0.795573	0.221566	1.350777	1.598786	0.931816	1.622558	1.052446
PGAM5	20.30254	20.52217	19.74533	23.17329	22.58526	20.27064	20.87288	19.96663
PGAP1	3.200006	3.214434	2.86723	2.570993	2.65613	2.084898	3.016005	2.27134
PGAP2	6.731512	7.253543	6.062309	7.187488	7.47572	7.364787	7.028244	6.636498
PGAP3	2.634232	2.730869	2.199276	2.898897	2.724674	2.430906	2.483547	2.101567
PGBD1	1.774975	1.636263	2.584545	2.67652	1.908478	1.61	2.385902	3.086902
PGBD2	1.771466	1.782337	1.375385	1.877944	1.942755	2.37399	1.016822	1.424956
PGBD5	1.237788	0.836022	0.947956	0.957808	0.816877	0.805396	0.940129	0.802778
PGC	0	0	0	0	0	0	0	0
PGD	60.41226	67.17489	63.01554	68.47023	63.38126	64.9808	64.1016	57.48528
PGF	4.03173	3.15879	3.255977	4.590664	3.745513	4.528344	3.31014	3.50756
PGFS	2.619363	5.521491	3.233407	4.199546	3.76054	3.339108	4.781289	7.627161
PGGHG	2.530294	2.161384	1.721839	2.752761	2.821253	1.826836	2.417175	2.544749
PGGT1B	7.496642	8.085863	8.494412	7.421033	7.922646	6.846128	6.75501	7.327503
PGK1	110.6924	107.1678	110.8777	101.348	100.9277	101.5879	104.3288	98.45582
PGLS	29.87231	32.87785	28.63394	33.38798	30.31476	31.99836	29.32542	29.51265
PGLYRP1	0.454914	1.018796	0.851202	1.766585	1.364918	1.374064	0.972079	0.898496
PGLYRP2	0.257795	0.385549	0.69101	0.440301	0.687542	0.469673	0.697765	0.249014
PGLYRP4	0.746217	0.536524	0.618061	0.431686	0.878724	0.563486	0.818492	0.695805
PGM1	8.558609	8.214173	8.736619	9.732498	8.811037	9.852341	8.349411	7.668477
PGM2	19.3552	21.15325	21.16874	18.52409	24.6196	20.35472	20.80906	19.30142
PGM2L1	1.588872	1.412235	1.263977	1.647075	1.445619	1.41087	1.42605	1.701018
PGM3	5.274881	6.761933	4.916805	6.453555	7.509602	6.408325	6.760796	5.923184
PGM5	12.9556	21.07058	22.30505	22.74784	17.24926	16.62806	24.27328	18.02402
PGP	12.02115	11.8653	11.59114	12.74189	13.95769	15.22	12.28002	11.87832
PGPEP1	5.230515	5.631038	5.764607	5.690267	5.913287	5.558216	5.870394	5.381227
PGPEP1L	0.004945	0.01002	0	0	0.009891	0	0	0.020062
PGR	0.080602	0.16332	0.086231	0.185848	0.20825	0.175831	0.265887	0.211187
PGRMC1	105.6068	130.3349	123.2357	130.5947	117.4392	141.8676	122.9434	107.4604
PGRMC2	34.94435	39.63886	44.56711	42.50081	39.88352	42.17579	38.19377	34.56757
PGS1	9.416683	7.576835	7.422304	7.10378	7.392679	7.542284	7.855663	9.122338
PHACTR1	1.195749	1.719372	1.163777	1.502921	1.901919	0.800678	1.412554	1.481626
PHACTR2	2.807192	3.056852	3.476268	3.238764	3.956678	3.085934	3.398484	4.435362
PHACTR3	0.045044	0.068453	0.061164	0.04039	0.016894	0.011338	0.04572	0.017132
PHACTR4	15.11064	14.35584	14.78964	14.71249	14.25937	13.11946	13.59362	13.63079
PHAX	34.47959	34.9321	35.39736	33.0469	37.82533	42.66213	35.47566	35.53947
PHB	44.78795	49.82488	49.8468	48.4186	52.66506	53.29766	47.56763	51.66384
PHB2	86.25109	90.49509	84.97681	84.18355	80.68389	89.81095	86.82694	80.61261
PHC1	3.454473	4.033183	3.12655	3.532651	3.967027	3.971828	3.62341	3.745028
PHC2	18.58982	17.97368	17.26694	17.97315	18.31593	18.20124	17.50771	18.06857
PHC3	4.428686	3.968843	3.319902	3.857797	4.4457	3.745576	3.922798	4.63512
PHETA1	1.236028	1.057455	1.012668	1.078994	1.172089	0.931049	1.087293	0.891466
PHETA2	4.321184	5.162208	5.282051	5.719184	5.680205	5.534039	4.486008	5.510494
PHEX	0.203576	0.332008	0.30401	0.122118	0.10925	0.324947	0.347742	0.261869
PHF1	11.28422	10.9595	9.607378	10.87702	11.80029	10.86734	9.704486	10.25546
PHF10	40.00118	37.79178	32.5762	36.04088	38.04019	31.78679	36.61041	37.93463
PHF11	11.52851	9.227356	9.427623	9.085365	12.2563	10.14493	8.875894	9.467651
PHF12	3.763492	4.03252	3.217055	4.034444	3.839142	3.364299	3.197956	3.73498
PHF13	6.006057	5.950189	4.479307	6.002561	5.967726	5.220337	5.342067	5.377703
PHF14	9.67225	9.915672	8.941444	8.468507	8.825452	10.22307	10.29872	9.146713
PHF19	5.455678	6.720963	4.869165	7.211629	6.814075	4.899813	5.453021	6.033422
PHF2	15.03559	14.54793	12.10858	15.26505	15.16097	13.16633	13.43654	14.17748
PHF20	8.767268	8.780875	7.347702	8.219912	7.51148	7.995362	7.87219	9.50147

PHF20L1	4.858286	5.089312	4.150378	4.921402	4.936618	4.380572	4.00703	5.146608
PHF21A	2.929937	2.961317	2.452176	2.82365	2.418267	2.464933	2.409344	2.667165
PHF21B	0.058259	0.059023	0.073971	0.119404	0.108209	0.125693	0.118265	0.109735
PHF23	21.61875	21.43559	19.703	22.02924	21.30976	22.3396	21.09007	20.79925
PHF24	0.265598	0.273976	0.267057	0.613635	0.502286	0.233378	0.16665	0.264481
PHF3	18.43151	18.74109	16.99098	17.37379	16.83419	17.22398	18.15352	20.38391
PHF5A	54.16692	51.62184	55.13657	58.08349	55.66414	63.92062	55.83941	52.21186
PHF6	10.50852	10.98432	10.39623	9.130929	10.91895	9.756694	10.35958	11.53247
PHF7	0.36667	0.3591	0.277611	0.450903	0.146687	0.221505	0.285331	0.322305
PHF8	8.461108	7.623743	7.18233	7.32858	7.904056	6.697313	7.528226	7.805565
PHGDH	41.02171	38.98637	37.00728	45.2573	35.47957	35.38438	42.43042	38.25162
PHIP	9.788633	10.02211	8.666665	9.216738	10.2657	8.169659	8.824029	11.63431
PHKA1	2.94057	3.184179	2.554209	2.579219	2.820782	2.534069	2.695838	2.116564
PHKA2	3.706415	3.393746	2.729452	3.015085	3.636833	2.571179	3.445278	3.914196
PHKB	13.99458	15.20914	14.24895	12.45734	13.88784	13.83065	14.10811	12.19855
PHKG1	1.661156	2.324084	1.653477	2.553456	2.096492	2.097268	2.274861	2.059205
PHKG2	17.0589	14.66471	14.79443	16.47945	13.8305	15.68116	14.56781	15.2652
PHLDA1	3.960365	4.768645	4.030694	5.469241	5.168047	4.656058	4.467505	4.070824
PHLDA2	0.46455	0.235324	0.573453	0.238029	0.406534	0.175397	0.412579	0.883432
PHLDA3	70.99477	62.12237	67.30415	69.05988	68.77361	65.20119	64.9146	62.0429
PHLDB1	15.35778	13.03896	12.87741	15.91693	14.8135	13.48018	14.65174	15.35761
PHLDB2	9.385104	8.057011	8.523163	8.043791	10.21267	9.055878	9.566901	8.530401
PHLDB3	17.22602	17.03416	15.45592	15.71835	16.04053	17.45698	16.72312	18.46019
PHLPP1	7.937226	7.236732	7.313734	6.833689	8.407635	8.136843	6.721422	7.610829
PHLPP2	3.932862	3.295757	3.107086	3.432121	3.613005	3.33979	3.435524	3.714503
PHOSPHO	0.012075	0	0.023849	0.049495	0.072458	0	0.024512	0.024493
PHOSPHO	10.43664	14.33497	16.80423	13.46406	12.56272	13.9552	10.06981	12.02865
PHOX2A	0.017965	0	0.017741	0	0.017967	0.018088	0	0.018221
PHPT1	48.43745	51.36978	45.73232	51.67585	46.59295	47.69703	51.84056	40.4934
PHRF1	11.22088	9.951985	7.935928	9.746196	9.626973	9.327229	9.130273	9.996263
PHTF1	3.408701	3.426831	3.578887	3.20249	3.325095	3.394971	3.075998	3.100317
PHTF2	4.734155	5.032168	5.945235	4.993442	4.618338	4.883705	5.474739	5.290699
PHYH	38.46428	38.75809	52.8952	33.63361	34.94932	41.38992	38.19573	35.42115
PHYHD1	2.136577	3.125167	2.070394	1.806338	2.363894	1.210035	1.436715	2.275343
PHYHIP	16.3792	11.22546	11.0752	11.84818	12.81597	10.43789	11.96992	10.68078
PHYHIPL	0	0	0	0	0	0	0	0
PHYKPL	1.467724	1.659892	1.146066	2.396035	1.604464	1.25437	1.576353	1.505928
PI15	7.797344	6.535889	6.521332	6.168552	8.662013	5.830422	7.501833	11.41172
PI16	60.93906	49.72878	79.06444	70.64497	73.72025	66.31027	69.50095	58.89578
PI3	0.844319	0.72709	3.960542	1.470896	1.308863	0.425043	4.841957	3.853521
PI4K2A	18.64206	18.8339	18.55688	18.43219	18.29438	20.35916	17.45513	18.35601
PI4K2B	7.494884	8.464845	6.256713	7.284554	7.459395	7.641513	6.800696	6.566344
PI4KA	19.97595	18.38778	16.60454	17.05117	18.59716	15.27778	15.61028	16.96464
PI4KB	20.25112	20.14658	16.77318	21.10341	20.66597	18.09526	19.02353	19.45091
PIANP	0.06256	0.101409	0.037068	0.115397	0.17519	0.062987	0.063498	0.16497
PIAS1	11.25932	10.73416	9.255986	10.7252	11.21003	9.775822	9.967496	10.97985
PIAS2	4.603059	5.033497	4.311673	4.851362	4.804419	4.390288	4.309882	4.937221
PIAS3	15.14621	12.9555	13.99307	14.96435	14.1346	13.90467	13.39095	14.23118
PIAS4	10.17217	9.751801	8.859448	9.873087	9.85977	9.014471	8.887478	8.708078
PIBF1	1.939036	2.001203	2.192263	1.810644	2.410515	2.216844	2.244026	1.948259
PICALM	80.82546	80.16764	77.53403	74.22148	73.8827	76.41565	78.69868	84.33518
PICK1	10.92231	9.337791	9.914237	9.717828	10.22208	10.29059	9.15865	9.740605
PID1	8.852216	12.21835	16.50445	11.45542	10.78812	8.25934	15.32297	17.32315
PIDD1	2.265155	2.231357	1.733815	2.899572	2.414387	2.414784	1.893403	2.225856
PIEZO1	10.34124	9.081445	8.451628	10.25704	10.30411	9.232715	9.130198	9.74029
PIEZO2	0.32127	0.367679	0.358394	0.445067	0.514694	0.377376	0.289857	0.527987
PIF1	0.194108	0.164937	0.142221	0.160416	0.181609	0.088261	0.241508	0.241326
PIFO	0.035474	0	0.035032	0	0	0	0.144025	0

PIGA	2.8431	3.347506	2.333405	2.861028	3.118848	3.210667	2.84025	3.383652
PIGB	7.353958	9.432297	5.519368	9.16392	9.987966	9.699498	6.598412	7.906165
PIGBOS1	3.40393	2.881709	5.29554	3.261272	2.82143	2.816864	5.833252	3.204099
PIGC	48.68663	51.23781	53.22572	47.82615	49.28989	49.79886	51.49643	45.55107
PIGF	9.684464	12.64182	11.87592	10.93315	10.08485	12.67045	11.23398	10.63186
PIGG	4.936284	3.622474	3.116426	4.40584	3.71355	4.306853	4.106706	3.861361
PIGH	3.944144	3.707046	4.551987	5.746233	4.229809	5.526033	4.148004	4.000291
PIGK	24.36194	28.5576	29.22649	26.44483	28.58837	30.17898	27.56644	27.76531
PIGL	15.43257	14.87335	14.25644	16.29642	15.84816	16.46536	13.241	13.97456
PIGM	5.304249	5.373862	4.056526	5.460919	5.452979	5.762756	5.734387	4.666628
PIGN	5.265013	4.708227	4.210093	5.720524	4.447451	5.149684	4.620911	4.854512
PIGO	10.64745	10.97179	8.535435	11.87728	10.46659	10.49685	10.87953	10.91151
PIGP	19.11271	37.00503	28.31184	32.59195	22.81733	34.72157	27.52721	30.26479
PIGQ	8.653224	7.064646	6.577905	8.303821	7.985192	7.260282	6.436356	7.223195
PIGR	0	0	0.015158	0.015729	0.030702	0.030908	0.023369	0.046703
PIGS	22.62077	22.61298	23.29582	23.31806	21.7437	23.40328	22.91486	22.94279
PIGT	12.13275	11.08027	10.59346	12.07949	10.74084	11.11076	11.96432	12.21793
PIGU	23.71935	24.76297	23.20799	19.98298	21.21735	20.85178	20.13371	18.78868
PIGV	14.12762	15.68657	14.62727	16.33433	15.04184	15.16816	14.72535	14.88133
PIGW	10.132	12.29699	10.37634	10.88687	8.088529	13.14563	11.49231	8.806327
PIGX	10.50309	16.16436	12.66146	12.80153	12.16455	17.1445	13.01353	13.42461
PIGZ	5.717573	6.641788	7.804352	11.59569	7.724212	8.499321	6.411015	7.954603
PIH1D1	12.48424	12.06701	11.29028	12.44082	13.57573	13.30098	10.12944	8.260329
PIH1D2	2.958097	2.459355	3.982316	2.732311	3.15748	3.005016	3.446779	3.350007
PIH1D3	0.397009	0.678746	0.392063	0.610265	0.248163	0.09993	0.402965	0.377495
PIK3AP1	1.3541	1.959176	1.74801	1.972628	1.960601	1.675229	1.652887	2.091482
PIK3C2A	7.904553	8.081064	6.769081	7.315203	7.532703	6.925422	7.63429	8.148905
PIK3C2B	10.24523	7.020886	5.912388	7.807152	8.450228	7.369092	6.859179	7.04377
PIK3C2G	1.034818	1.110557	0.872473	1.2826	1.268123	0.823626	0.929945	0.929245
PIK3C3	8.581327	8.148277	7.554051	9.035752	7.989291	8.103761	7.917666	7.936255
PIK3CA	6.793487	6.410352	6.352576	6.368813	6.471047	6.523846	6.353269	6.780903
PIK3CB	11.22119	12.93794	12.9149	11.38618	11.55921	13.24898	12.09032	11.02786
PIK3CD	1.583737	1.467332	1.244226	1.876367	1.672266	1.203327	1.476025	2.048159
PIK3CG	1.161649	1.487772	1.233754	1.609692	1.819421	1.243144	1.40154	1.911773
PIK3IP1	10.43451	10.68724	9.695025	10.96235	9.064221	9.424143	8.49138	11.06989
PIK3R1	15.96584	21.44344	13.59344	17.1277	16.07332	15.90057	18.23036	23.87227
PIK3R2	37.73187	36.18734	29.71901	38.46451	33.61105	32.85111	37.44758	37.55504
PIK3R3	1.052149	0.783049	0.748502	1.236622	1.311616	0.823371	0.951522	0.687817
PIK3R5	0.767075	0.881169	0.781376	0.786075	0.851744	0.729746	0.741797	1.347707
PIK3R6	1.103584	1.580715	0.892535	1.472146	1.103726	1.206909	1.342234	1.79473
PIKFYVE	5.325679	5.08247	4.163925	4.389647	4.84237	4.037828	4.666342	5.275143
PILRA	0.880888	2.08238	1.284156	1.859148	1.552241	1.657669	1.02183	2.467563
PILRB	0.046225	0.398068	0.593436	0.307904	0.184923	0.418866	0.304969	0.79701
PIM1	38.63381	34.15044	37.76081	42.75634	36.90622	40.24267	34.78567	34.94999
PIM2	1.158712	1.284966	0.48709	1.339852	1.174522	0.874971	0.993322	1.302257
PIM3	14.406	12.72933	13.32363	14.68253	15.12629	14.45181	13.50853	13.78978
PIMREG	3.251931	2.780422	1.63355	2.793121	1.767174	2.914558	1.831608	2.364045
PIN1	110.8995	118.701	124.0817	105.5482	111.5218	115.2761	103.0665	108.0284
PIN4	24.48345	37.00666	36.12798	32.10118	25.84384	38.37085	30.5325	25.92161
PINK1	13.9164	16.17825	15.37402	19.2485	16.69402	18.74428	15.10716	14.17398
PINX1	6.330367	7.713668	7.632799	7.019151	7.470511	7.970647	7.318136	7.063995
PIP4K2A	9.134048	11.05396	9.839555	9.993946	11.13919	9.882044	10.27205	13.47935
PIP4K2B	8.722683	8.483925	7.719395	9.0185	9.284226	8.189892	8.502794	9.317299
PIP4K2C	25.23306	24.62442	22.61088	26.53448	26.58333	24.72293	22.04459	24.24436
PIP4P1	15.04293	14.8526	12.80136	17.46186	15.877	15.39701	15.13345	13.09678
PIP4P2	3.245428	3.84677	4.388531	3.293212	3.288277	3.780164	4.047664	3.539039
PIP5K1A	20.76328	19.72527	18.47099	20.0663	21.48716	20.73297	20.78799	23.38113
PIP5K1B	1.239242	1.219634	2.13291	2.231463	1.469577	2.245874	1.976591	1.723726

PIP5K1C	2.514208	2.368662	1.70553	2.724975	2.820037	2.144973	2.257774	2.883644
PIP5KL1	0.083836	0.036401	0.17741	0.122732	0.035934	0.07235	0.048625	0.157911
PIPOX	0.009961	0.040365	0.049182	0.010207	0.039847	0.010029	0	0.040409
PIR	4.406457	7.258601	3.980864	5.017341	6.414673	3.877882	3.180483	4.634712
PIRT	0.303288	0.834015	1.15525	0.932402	0.606655	1.395932	1.187373	1.31831
PISD	8.473017	8.012641	6.850778	9.65718	8.557704	8.762318	9.140933	9.5685
PITHD1	62.762	66.96019	84.41951	65.44249	69.88603	86.01598	66.35847	64.66361
PITPNA	23.74589	24.72474	22.98441	24.29574	24.69951	23.74234	25.59089	23.59056
PITPNB	30.90747	30.81393	30.29779	31.3138	31.19575	31.83409	30.60171	28.4741
PITPNC1	3.903402	3.662527	4.805315	4.622832	4.303169	4.139969	4.277108	5.924957
PITPNM1	4.63686	5.514708	3.683177	5.839791	5.470858	4.709133	5.408965	5.404892
PITPNM2	3.401871	2.905584	2.402859	3.349349	3.287884	2.910577	2.872255	3.237557
PITPNM3	3.59764	3.161025	3.580324	3.450216	3.387155	2.90098	3.251706	3.406674
PITRM1	9.391335	9.536194	8.326526	9.507396	9.463651	8.832761	9.907432	9.157293
PITX1	0.012346	0.062542	0.158503	0.126522	0.012348	0.062153	0.025063	0.037566
PITX2	0.098669	0.055536	0.205706	0.213461	0.131576	0.242839	0.178043	0.15567
PITX3	0.00472	0	0.009322	0.019347	0.018882	0.009504	0	0
PIWIL1	0	0.008404	0	0	0	0	0.00842	0
PIWIL2	0.011847	0	0.0117	0.078915	0	0.005964	0.006012	0
PIWIL3	0.26373	0.143872	0.086815	0.069298	0.223185	0.129361	0.102956	0.123454
PIWIL4	0.018169	0.018407	0.017942	0	0.009086	0	0.027662	0.018428
PJA1	4.995134	5.945716	5.367127	5.329147	5.330004	5.294888	5.385452	5.2149
PJA2	30.46246	35.00698	29.1612	31.38174	31.07832	31.02556	33.57715	30.37022
PJVK	0.052024	0.026354	0	0.07997	0.026016	0	0	0.052765
PKD1	5.892665	5.230301	4.029461	5.643121	5.81185	4.320497	5.001713	6.127882
PKD1L2	0.04126	0.022801	0.007408	0.003844	0.011254	0.01133	0.015229	0.022826
PKD1L3	0.009007	0	0.013342	0.023075	0.009008	0.03174	0.009142	0.004568
PKD2	14.51353	12.94573	13.92942	11.57179	12.89313	12.40279	13.17694	16.76221
PKD2L1	0.010642	0	0.042039	0.021812	0.031931	0	0.021604	0
PKDCC	4.169814	4.606483	3.486077	4.870166	6.158412	4.370833	4.916497	5.793389
PKHD1	0.001864	0.007555	0.001841	0.001911	0.013052	0	0.007569	0.001891
PKHD1L1	0.556333	0.358279	0.362009	0.371237	1.11281	0.223619	0.77698	1.415006
PKIA	1.414955	1.768014	1.843138	1.608814	2.196834	2.177636	1.56612	1.435098
PKIB	16.40318	22.65016	23.23698	12.78433	14.52759	14.63738	15.59612	13.27929
PKIG	3.758649	3.863434	3.892001	4.749248	5.109504	5.566267	4.166901	3.682617
PKLR	0.053722	0.095247	0.039789	0.123869	0.201484	0.094656	0.163584	0.122595
PKM	107.9344	104.8443	103.2744	107.7536	112.9482	89.70002	119.0477	106.3086
PKMYT1	0.707266	0.693054	0.847304	0.867365	0.510225	0.828835	0.870864	0.682055
PKN1	18.04996	15.39895	14.00077	18.52589	18.76501	15.88863	17.15961	20.01873
PKN2	8.645398	9.571642	7.902885	7.863806	9.314316	8.067303	8.79531	9.309235
PKN3	2.667825	2.788272	1.847237	3.165991	3.151201	2.454494	2.700061	2.317038
PKNOX1	5.805235	5.114057	4.523068	4.901962	5.445018	4.898042	4.79331	5.289809
PKNOX2	2.831931	1.887955	1.948957	2.586309	2.546133	1.499506	1.757411	3.348473
PKP1	230.2135	179.8809	179.23	195.9037	187.1784	188.7598	197.8802	205.6501
PKP2	4.322452	4.647749	3.897728	3.773016	4.293552	4.114735	4.417185	3.345869
PKP3	111.0696	97.47609	106.5339	108.6903	106.9844	112.7112	101.8481	102.154
PKP4	21.20686	20.22018	18.66364	20.02445	20.2378	20.23156	20.90362	22.75049
PLA1A	0.02859	0.003621	0.024704	0.029298	0.021445	0.039579	0.007255	0.028997
PLA2G12A	19.49502	20.32458	21.44425	22.67052	21.29475	22.93504	19.16669	15.75217
PLA2G12B	0.026707	0.081173	0	0.027369	0	0.188228	0.081323	0.135437
PLA2G15	0.280785	0.265074	0.226871	0.379294	0.274439	0.212027	0.174884	0.207114
PLA2G16	14.3172	15.49293	15.19373	15.80327	15.91803	16.62382	15.94368	15.20453
PLA2G1B	0.043665	0.044238	0.043121	0	0.04367	0.087926	0	0.088573
PLA2G2C	0.39858	0.575804	0.262409	0.302558	0.118113	0.215514	0.202279	0.351851
PLA2G2D	0	0.023611	0	0	0.023308	0.023464	0.023654	0
PLA2G2E	9.454118	9.735729	12.77601	15.04236	14.15191	20.19593	10.10098	9.399453
PLA2G2F	124.5443	45.27156	88.16403	62.44011	57.6929	70.79091	60.87371	44.56017
PLA2G3	1.807541	1.673977	1.708362	2.340962	1.397421	1.674745	1.13681	1.270919

PLA2G4A	27.20551	32.59085	30.68822	27.92897	25.30284	24.79055	31.49245	28.23061
PLA2G4B	16.03151	15.76289	16.75473	13.74685	15.89062	11.54117	12.00868	12.79143
PLA2G4D	0.07224	0.024396	0.02378	0.082255	0.032111	0.032326	0.065177	0.048846
PLA2G4E	7.398186	7.126341	7.48266	8.4391	8.638722	8.394291	6.588347	5.825259
PLA2G4F	0.239516	0.159462	0.297354	0.532975	0.73233	0.275603	0.250055	0.291511
PLA2G5	0.210621	0.796637	0.342039	0.05276	0.332356	0.782265	0.3563	0.123424
PLA2G6	2.121144	1.871965	1.996525	2.088776	2.386596	1.693492	1.959532	2.445469
PLA2G7	14.59762	14.6347	19.26204	12.8993	18.42616	17.48886	16.4488	19.52966
PLA2R1	3.933437	5.697646	5.9711	4.763754	6.461749	7.012364	6.108265	5.629714
PLAA	17.18565	18.50183	19.24008	17.93639	16.50536	17.245	18.74097	17.28408
PLAC1	0.749102	0.551951	0.807019	0.453616	0.476763	0.411392	0.656656	0.552557
PLAC8	0.92233	1.509471	0.31529	0.836118	0.638619	0.607182	1.044184	0.971439
PLAC8L1	0.146221	0.07407	0.060166	0.099895	0.03656	0.07361	0.024736	0.037076
PLAC9	2.458687	5.008839	6.916666	3.799814	4.63888	4.054441	5.179989	7.561941
PLAG1	1.194087	1.483217	1.254539	1.211639	1.61384	1.107719	1.369826	1.633627
PLAGL1	21.80822	21.58244	22.9569	23.01768	23.29622	21.69889	23.74519	22.3789
PLAGL2	3.688055	3.426362	4.018999	3.148217	3.571376	4.093706	3.547769	3.571925
PLAT	3.901046	2.774497	3.311551	4.467317	5.142444	2.993602	3.188071	3.219681
PLAU	14.96593	16.87539	14.79129	18.12624	15.68747	13.25709	15.21476	15.79927
PLAUR	1.180813	1.19631	0.961071	1.409523	1.583273	0.927588	1.672667	2.592656
PLBD1	41.2106	45.89067	51.24614	48.7442	36.83186	39.04974	43.9022	45.94102
PLBD2	14.09839	13.88276	14.78191	14.83261	14.26831	13.478	13.97871	14.29909
PLCB1	2.714203	3.151196	2.884326	3.537259	3.228802	2.571496	3.529201	3.116182
PLCB2	0.612901	0.620945	0.530327	1.124568	0.776442	0.446655	0.853159	1.391259
PLCB3	10.25583	8.821422	7.509056	10.108	9.63893	9.188234	10.13981	10.08498
PLCB4	2.291759	3.117279	1.817897	2.723638	4.08007	3.327591	2.773055	3.056134
PLCD1	67.22076	65.91182	67.54729	68.96571	66.30382	77.80255	66.43929	61.66622
PLCD3	4.754997	3.138071	2.621851	3.116564	3.589539	2.524565	3.557363	4.03909
PLCD4	0.159077	0.1442	0.024804	0.077219	0.100483	0.042148	0.076483	0.067933
PLCE1	0.403201	0.707626	0.442071	0.741788	0.717601	0.485869	0.718603	0.869403
PLCG1	18.28743	17.32732	13.26914	17.87763	16.92883	15.56377	17.3445	18.24492
PLCG2	5.380542	7.072525	8.16099	8.751421	6.747244	9.282334	9.081078	8.332629
PLCH1	0.043173	0.030618	0.055426	0.079637	0.073404	0.039121	0.048203	0.039409
PLCH2	7.422443	6.401149	5.254011	6.958826	5.545406	5.65391	6.173264	6.917234
PLCL1	0.663775	0.772769	0.506003	0.477345	0.704624	0.674172	0.833299	0.572831
PLCL2	0.980165	1.45135	1.125141	1.468034	1.709227	1.197729	1.037383	2.098695
PLCXD1	2.802629	4.287321	2.676061	1.445535	4.288019	3.419767	3.541706	2.691928
PLCXD2	4.772413	3.811893	3.442	3.564292	4.094815	3.953401	4.359229	4.274596
PLCXD3	0.404398	1.11801	0.673496	0.899071	2.505537	1.280142	0.493949	0.907208
PLD1	1.758144	1.324256	0.930235	1.594169	2.052968	1.550816	1.348509	1.596453
PLD2	7.236223	6.460561	5.472564	7.308473	7.012252	6.339573	6.162756	6.997118
PLD3	46.02601	38.41119	43.56669	39.64551	38.83859	39.96898	39.36656	38.09283
PLD4	0.585241	0.859737	0.664641	1.094522	0.819444	0.854397	0.787076	1.023913
PLD5	0.115499	0.097512	0.066535	0.128223	0.067383	0.281028	0.107462	0.019524
PLEC	28.59883	22.62028	20.21056	24.70772	25.10425	18.55652	24.00265	29.37564
PLEK	0.586365	0.732675	0.801032	1.131676	1.602939	0.826519	1.06137	2.041846
PLEK2	15.55566	15.89006	13.7658	16.73144	16.80669	15.9208	14.37227	15.83298
PLEKHA1	16.31825	16.62296	16.52506	16.17833	16.38345	16.72085	16.41895	16.69985
PLEKHA2	12.08967	11.89196	11.84818	13.39846	11.7232	11.89438	11.61188	13.77117
PLEKHA3	6.531965	7.1464	6.570826	6.649198	7.411389	5.954833	6.011973	7.454173
PLEKHA4	11.84862	10.9821	10.19761	10.37531	10.58443	11.68027	10.5091	12.10101
PLEKHA5	16.17691	17.21394	15.09732	17.51831	15.89029	15.49107	15.9742	13.66681
PLEKHA6	6.29279	5.555919	5.02582	5.712621	4.403904	4.498566	5.15572	5.3881
PLEKHA7	15.18776	12.2373	12.09009	14.3972	13.36611	13.43876	13.4156	14.21511
PLEKHA8	9.017586	8.633125	10.2276	10.98743	10.80752	11.0685	9.105328	10.58006
PLEKHB1	0.640322	0.432483	0.295093	0.233309	0.35578	0.315185	0.288856	0.505118
PLEKHB2	25.00192	28.42229	22.30152	18.05132	22.1799	26.79097	23.47368	23.02788
PLEKHD1	0.683826	0.837805	0.745977	0.684468	0.652105	0.592428	0.637588	0.403233

PLEKHF1	3.902561	4.143559	5.642159	5.534932	4.496331	5.50091	4.309676	5.873844
PLEKHF2	21.71704	26.27168	28.41279	28.71359	25.09933	28.46325	27.96198	23.24303
PLEKHG1	14.67197	13.52885	11.27422	10.7295	12.57471	11.69805	13.02849	14.53629
PLEKHG2	4.447908	3.648831	3.302314	4.38368	3.781441	3.816044	3.426821	3.694821
PLEKHG3	10.97956	9.389713	7.881403	9.590549	9.885779	9.561394	9.940758	10.72764
PLEKHG4	0.128365	0.073506	0.170858	0.148703	0.212109	0.067431	0.124626	0.192458
PLEKHG5	3.026036	2.686761	2.289075	3.648629	3.393874	3.093788	2.440869	3.062339
PLEKHG6	3.743207	3.178024	2.985995	3.612234	2.789575	2.645471	2.970554	2.812522
PLEKHG7	0.202437	0.008917	0.060844	0.063138	0.140844	0.053171	0.026801	0.017854
PLEKHH1	0.445282	0.633725	0.434499	0.586687	0.593786	0.442987	0.54343	0.516138
PLEKHH2	4.94492	5.18768	3.327087	3.790917	4.126176	3.417333	4.119645	4.434504
PLEKHH3	7.234935	6.366835	6.13652	7.946306	8.441849	7.718587	5.806872	7.204025
PLEKHJ1	6.638578	7.268411	6.215787	7.665649	7.462191	6.895813	5.786659	5.801705
PLEKHM1	6.900092	6.211505	5.510362	6.208478	6.6446	5.39432	6.170968	6.369987
PLEKHM2	43.73351	34.63146	42.9352	46.40906	44.44487	45.09434	39.29659	38.5152
PLEKHM3	3.082311	2.696932	2.468456	3.050979	2.783355	2.683387	2.850596	3.084205
PLEKHN1	3.367895	2.934635	2.599448	3.203955	2.529122	2.152587	2.601728	3.101069
PLEKHO1	8.671515	8.402239	7.252393	10.3157	10.53826	9.043776	8.716301	8.786438
PLEKHO2	5.104549	4.68918	3.799979	4.863062	6.572762	4.094248	5.284106	6.340902
PLEKHS1	2.55548	3.938743	3.014013	3.835085	3.234129	1.877879	2.495468	2.825249
PLG	0.010156	0	0	0	0	0	0	0.0103
PLGRKT	18.66766	22.51031	18.14283	21.00011	21.77764	23.43813	19.72361	21.1594
PLIN1	1.031067	13.85181	0.275768	3.191832	3.974417	0.756956	0.817604	0.904133
PLIN2	88.86727	119.8181	102.9832	142.3806	150.4808	140.5527	85.62441	89.67852
PLIN3	12.47478	15.09095	13.58251	16.58932	14.56725	14.74796	13.86248	14.21473
PLIN4	8.708012	10.29061	10.00659	10.10811	13.07594	9.180017	7.504148	5.569603
PLIN5	0.011476	0.011626	0.011333	0	0.011477	0.023108	0	0.011639
PLK1	4.713545	4.277687	3.334857	3.80528	3.594266	3.395548	3.899275	4.134248
PLK2	36.97309	36.37672	36.95998	32.34647	33.23073	37.76312	36.9041	35.73223
PLK3	30.55344	27.93521	34.08365	31.72624	33.64679	32.71595	29.52446	26.6002
PLK4	7.694008	10.11395	8.668859	8.584164	7.678938	8.684568	8.673509	8.593666
PLK5	0.270688	0.095984	0.213852	0	0.230115	0.149896	0.206061	0.09609
PLLP	2.012671	3.68209	3.131532	3.26443	2.577711	3.411385	3.101034	2.907784
PLN	6.368919	8.761595	8.351023	10.65224	11.87669	11.07699	10.73445	8.933239
PLOD1	11.51058	12.36758	9.973069	15.53286	14.39008	15.37028	12.39048	13.92305
PLOD2	1.729469	1.778986	1.960622	1.835605	2.206241	1.643559	1.567331	2.935415
PLOD3	6.234475	5.856777	5.546	6.532571	6.919842	4.807436	5.022216	7.025808
PLP1	0.918883	1.210225	1.134291	2.16578	1.654203	1.591275	0.960646	1.491142
PLP2	212.6669	205.4918	236.8224	223.2495	210.7211	225.6133	197.0034	190.9088
PLPBP	18.53915	18.44391	19.85252	20.01198	19.19658	18.86766	15.60189	18.04389
PLPP1	51.55984	61.97943	56.58023	63.0082	60.63207	64.14592	57.41158	60.16921
PLPP2	10.97832	10.8977	11.21386	13.00029	12.84296	12.52709	11.9759	11.56199
PLPP3	88.77964	91.09239	78.79581	77.24672	74.11981	76.36364	84.55837	95.56781
PLPP4	12.2499	4.889051	10.4843	7.151596	10.5437	11.4366	8.590521	7.831064
PLPP5	12.65056	11.92419	10.96318	13.25961	11.10294	12.46175	14.33552	11.19793
PLPP6	3.835096	6.63018	6.523555	6.544145	5.920535	7.368344	5.937142	5.896986
PLPP7	1.318286	1.43452	0.843798	1.263379	1.574823	1.42561	1.33806	1.250392
PLPPR1	0.132764	0.036684	0.059596	0.111316	0.036213	0.06076	0.110255	0.073448
PLPPR2	0.174804	0.093758	0.233552	0.326656	0.349653	0.103528	0.114805	0.292011
PLPPR3	1.255119	1.174352	0.816599	1.316473	1.188826	0.981221	1.079107	1.115736
PLPPR4	0.539394	0.989116	0.426139	0.5417	0.938667	0.385586	0.443463	0.475953
PLPPR5	0.047574	0.064264	0.062641	0.065003	0.03172	0.015966	0.241436	0.160836
PLRG1	19.03263	19.61254	19.98361	17.95437	21.4667	21.52644	19.41989	19.93064
PLS1	1.546325	3.105874	2.727365	2.46344	1.870688	2.821437	3.036234	1.753252
PLS3	89.71737	81.06788	84.61263	93.31876	95.63691	86.028	85.28022	80.60734
PLSCR1	2.184984	2.430213	2.92001	3.748069	5.166254	3.622678	4.001607	6.130374
PLSCR3	21.82691	20.24872	19.21231	22.17198	20.23443	17.33649	20.30005	20.42304
PLSCR4	2.894138	3.828468	3.583702	4.156768	5.27911	3.442339	4.23129	7.916287

PLSCR5	0.058514	0.039521	0.154093	0.059963	0.039014	0.058914	0.019797	0.039565
PLTP	25.57283	29.49891	33.68551	28.00839	25.13309	27.84248	32.89177	26.3181
PLVAP	44.5098	46.10667	42.12762	60.49342	67.11783	61.13455	45.56135	40.21121
PLXDC1	4.305242	4.155515	5.6889	5.381874	5.791961	4.539602	5.030976	5.718813
PLXDC2	20.75631	15.73792	16.81491	14.98043	19.23923	15.46647	17.92906	19.36253
PLXNA1	13.24056	10.67204	9.832936	11.31646	11.74369	10.14506	10.24372	12.34727
PLXNA2	15.877	13.6686	10.49023	12.72186	12.62217	11.48262	12.37684	13.38793
PLXNA3	3.51136	2.930872	2.117531	3.323147	3.283933	2.449755	2.597654	3.239464
PLXNA4	0.86626	0.920862	0.5373	0.725917	0.88131	0.612243	0.714665	0.952169
PLXNB1	8.850729	6.937078	5.906008	7.31672	6.945662	7.029983	6.904225	7.637256
PLXNB2	27.46015	25.81071	22.14629	25.88605	25.31652	24.57183	25.07098	27.11779
PLXNB3	0.094604	0.063897	0.044488	0.133879	0.099121	0.058964	0.045725	0.137072
PLXNC1	1.682731	1.672949	1.30068	1.510876	1.92674	1.250878	1.408678	2.651743
PLXND1	5.930563	5.637507	5.021148	7.569737	8.315252	6.175847	5.788319	5.466296
PM20D1	1.141736	1.459938	0.952363	1.215455	1.751628	0.82588	0.776325	0.899409
PM20D2	0.984804	1.190273	1.535579	0.460336	1.244124	0.400092	0.999576	1.314241
PMAIP1	3.983806	5.148981	5.409479	5.418299	5.595627	6.031278	5.232846	4.827825
PMCH	0	0	0	0.02837	0	0	0	0
PMEL	0.356125	0.823071	0.428619	0.878151	0.556517	0.605066	0.361466	0.620803
PMEPA1	18.25919	12.3671	14.35196	13.1328	12.7847	10.8482	15.35223	17.67262
PMFBP1	0.008247	0	0.008144	0.008451	0	0.008303	0	0.008364
PML	3.030548	3.230234	2.669348	3.214798	3.058566	2.777116	2.803652	2.83756
PMM1	14.67763	14.5323	13.36529	18.47594	16.26822	15.68953	16.54236	14.06492
PMM2	6.442747	7.193583	5.382781	7.127781	7.391165	7.956943	6.686468	6.647516
PMP22	38.75637	34.67046	48.37073	40.30086	43.47786	33.33464	40.93325	47.54783
PMPCA	17.53722	19.38367	15.99808	20.09936	19.21717	20.45616	20.28874	17.04922
PMPCB	18.87923	20.08131	20.46454	19.40203	20.28131	21.93463	19.25803	18.88868
PMS1	9.232167	8.49976	8.048651	7.843146	9.029358	10.08993	8.434485	8.886868
PMS2	4.67419	4.826275	4.157118	4.606404	4.574021	4.931565	4.937485	5.189255
PMVK	3.750817	4.239091	3.187576	4.685977	4.9021	3.400301	4.140768	3.501088
PNCK	0.086431	0.145942	0.270287	0.265715	0.230512	0.174042	0.233939	0.277594
PNISR	21.07447	22.40234	17.61818	22.98412	22.1243	20.10809	18.47176	23.11124
PNKP	12.44641	11.79074	11.31224	14.4272	13.27178	13.56037	14.73862	13.64462
PNLDC1	0.006659	0.067467	0.039458	0.204729	0.059942	0.033524	0.027037	0.047279
PNLIPRP3	1.021164	2.809785	1.352226	0.83241	1.288225	2.628766	2.402751	1.56532
PNMA1	0.36814	0.266408	0.280455	0.409596	0.536502	0.550687	0.309606	0.256033
PNMA2	0.23996	0.405182	0.194437	0.390924	0.258452	0.241599	0.512099	0.237135
PNMA5	0	0	0	0	0	0	0	0
PNMA8A	0.012551	0.025432	0.012395	0.06431	0.012553	0.025274	0.012739	0
PNMA8B	0	0	0	0	0	0	0	0
PNMT	0	0	0	0	0	0	0	0
PNN	29.62574	30.87674	24.82466	28.79427	28.77117	27.97532	25.85455	29.93683
PNO1	21.87441	23.4601	25.15029	26.87366	25.47091	25.62044	23.82347	21.29071
PNOC	0.046455	0	0.114691	0.023803	0.046461	0.163703	0.094304	0.070675
PNP	8.372655	9.540312	6.920018	8.168536	10.7232	9.198042	10.67885	9.713694
PNPLA1	21.87008	17.92576	15.02718	21.11786	18.40821	18.05891	22.16527	20.39179
PNPLA2	23.12727	26.05084	17.93562	20.89852	19.21363	17.25936	18.25436	20.14411
PNPLA3	5.885794	3.44638	3.718722	7.312488	8.386758	5.384379	4.464501	2.856413
PNPLA4	0.483641	0.854338	0.624573	0.749785	0.768964	0.23723	0.440547	0.603724
PNPLA5	4.813166	5.61393	8.607673	8.351903	7.220682	8.45004	2.524788	2.153684
PNPLA6	4.0294	3.781284	3.74692	3.951609	4.289916	3.76403	3.989323	4.714528
PNPLA7	0.932351	0.864297	0.589269	0.941114	0.862536	0.924639	0.728679	1.030729
PNPLA8	18.34533	18.37051	20.86352	20.51306	19.14576	22.69427	17.88001	19.32331
PNPO	1.48421	2.024854	1.690571	1.486407	1.568743	1.519821	2.071401	1.436914
PNPT1	12.97081	13.60408	14.00075	14.78149	13.63985	14.36628	13.7406	12.53431
PNRC1	37.01793	35.98437	34.51652	35.59825	34.13007	34.22013	35.63163	34.92188
PNRC2	98.8729	116.7456	117.8366	112.3348	107.2045	114.7632	110.7753	114.085
POC1A	3.421789	3.534405	2.983158	3.095624	2.352784	2.583874	3.473116	2.670658

POC1B	9.940262	10.45188	10.1195	9.902401	10.44655	10.71592	9.31558	9.680104
POC5	4.266641	5.014258	4.311053	4.243459	4.18634	4.006388	4.458281	4.946878
PODN	14.5859	13.35512	16.683	14.87823	18.31888	13.17537	17.84956	21.87338
PODXL	17.50931	15.84929	16.36102	18.75754	19.90557	19.58564	15.39831	16.74272
POF1B	23.13991	23.99676	24.32016	28.44688	28.5101	27.2473	26.52042	22.74798
POFUT1	4.609828	4.791677	4.779369	4.465255	4.374075	4.494649	4.787406	4.632768
POFUT2	11.57866	11.26808	11.03362	12.20337	13.76174	13.76459	13.70892	13.71146
POGK	12.96765	11.91131	11.49156	12.60992	11.78865	10.86706	11.70106	11.88773
POGLUT1	9.403963	6.963763	8.816339	7.234017	9.293802	8.216161	7.265521	9.148792
POGZ	6.708113	6.259721	5.136323	6.456894	6.623438	5.462203	5.667745	6.795346
POLA1	5.838373	5.540078	4.757375	5.563132	4.979946	5.246187	5.282042	5.261309
POLA2	5.067488	5.429967	5.037635	5.803277	4.281514	4.830598	5.428617	5.492905
POLB	36.34649	41.13689	51.15423	50.59829	42.87627	58.64296	46.53475	34.28503
POLD1	5.625797	5.39965	4.889665	5.832579	4.441993	5.10112	5.301121	5.647491
POLD2	7.428975	7.146759	5.842689	6.77625	6.599924	6.361136	6.630126	5.715535
POLD3	7.216579	7.820252	7.087286	7.338135	6.84268	7.145451	7.033454	7.747957
POLD4	10.74206	11.43855	11.10047	11.18693	12.23905	12.37126	10.06837	10.5158
POLDIP2	41.22237	46.00933	40.81519	43.69346	44.58359	43.22709	43.19481	36.73851
POLDIP3	48.66036	53.82236	42.56494	49.21689	47.78674	43.65309	48.02991	47.67684
POLE	2.512261	2.202605	1.816984	2.322818	1.952891	1.961925	2.263893	2.556191
POLE2	3.334809	4.538384	3.358794	3.587433	4.164901	3.925541	3.70479	3.853445
POLE3	44.37494	49.34783	49.51977	47.43184	42.36745	51.84259	48.86661	44.03135
POLG	12.0555	11.72904	9.316357	13.05353	10.97894	11.05251	12.10684	11.87777
POLG2	3.923962	4.835019	4.232902	5.443057	3.853759	4.217713	3.731653	3.917078
POLH	8.908325	8.786158	8.23969	8.533055	8.496454	8.247914	9.854613	9.701878
POLI	2.907295	4.42689	3.601578	4.795102	4.094826	3.654615	3.928719	4.047895
POLK	3.385611	3.789172	3.650621	3.803077	4.261501	3.292202	3.487794	3.749307
POLL	4.027218	3.682801	3.652596	3.649113	3.349383	2.79563	3.323886	3.267638
POLM	0.645066	0.85723	0.512932	0.798404	0.837856	0.514517	0.603723	0.611765
POLN	0.350229	0.394251	0.249792	0.289118	0.282166	0.450573	0.246863	0.434152
POLQ	1.114852	1.365062	0.899643	0.975994	0.77094	0.862697	1.011951	1.347176
POLR1A	11.1657	9.89073	9.098378	9.162196	10.54498	9.102389	9.770779	10.39436
POLR1B	15.52016	14.942	14.56467	13.04148	15.38638	14.35998	15.74571	15.75559
POLR1C	7.677939	9.486788	7.537272	7.879798	9.798044	8.384149	7.908733	7.637041
POLR1D	54.13332	58.14473	70.82614	51.85371	63.52359	53.11981	52.27575	63.46402
POLR1E	11.06099	10.16207	10.28709	9.480815	8.661365	8.54844	10.95946	10.49985
POLR2A	23.31047	19.32633	18.02654	18.62531	20.79737	16.80707	19.56859	19.9707
POLR2B	32.74023	36.94279	33.93758	33.57393	33.31348	33.33586	36.86121	35.82924
POLR2C	26.78888	27.72843	25.97313	27.24969	28.32258	28.6983	25.62433	28.46787
POLR2D	11.65119	13.03476	14.07512	11.81531	14.15331	12.00596	13.05889	12.25852
POLR2E	108.273	103.6784	99.28291	105.4908	103.7617	105.6425	105.2704	96.62705
POLR2F	77.28988	73.17031	81.23756	68.67291	84.97302	67.9476	84.26562	76.75702
POLR2G	53.71372	60.83561	51.05265	52.14334	48.80158	58.17769	57.06941	54.04771
POLR2H	12.69149	14.1107	12.36576	12.86922	13.5054	13.73015	13.26328	12.93364
POLR2I	30.71185	36.55818	35.70668	32.14222	31.1515	35.34426	32.94118	29.93401
POLR2J	32.89789	37.34641	36.86942	37.58225	45.60038	38.06489	37.84673	33.84492
POLR2K	14.80508	21.62319	22.22439	16.03007	17.15774	17.10951	16.67246	15.28985
POLR2L	52.71579	51.89992	54.94168	47.27621	54.84067	58.26249	47.63882	48.12541
POLR2M	17.44852	19.94241	19.60438	16.58635	17.77497	16.97125	18.66326	16.84664
POLR3A	5.085077	5.305508	4.134826	4.576758	4.506155	4.386668	4.942704	4.311224
POLR3B	8.494557	8.382065	8.263015	8.40977	8.214254	7.76343	7.275642	8.826114
POLR3C	7.182436	7.907901	6.791114	9.191399	8.123903	7.184161	6.490759	7.570824
POLR3D	10.54625	10.51879	9.653598	11.35974	10.93649	11.12654	8.994222	9.457924
POLR3E	10.97093	11.87097	9.944789	11.77889	11.21255	10.91633	11.58823	12.10149
POLR3F	9.500661	10.33631	9.715459	11.24342	10.20373	11.1553	9.35552	10.33397
POLR3G	4.043696	3.500872	3.82576	3.477365	3.235375	4.543936	3.74845	3.62517
POLR3GL	8.187178	8.147536	8.17867	9.754885	10.14533	10.05443	6.291755	7.958006
POLR3H	5.958907	5.941474	5.232102	5.610735	5.546631	6.059018	5.856662	5.409443

POLR3K	33.20923	28.77401	27.74216	22.73481	32.25181	28.21495	29.00158	24.90447
POLRMT	4.587029	4.548028	4.001761	4.106316	5.024539	4.352943	4.518224	4.63705
POMC	0.212039	0.214822	0.348995	1.014027	0.176722	0.28465	0.14348	0.143372
POMGNT1	11.19158	10.71774	9.45885	10.24449	11.32579	9.982905	11.18326	10.0356
POMGNT2	2.713508	2.000999	1.827465	2.033118	2.58039	2.069188	1.589315	2.192686
POMK	3.179867	2.499448	2.1313	2.63369	2.427784	2.296533	2.684948	3.518328
POMP	148.3731	174.6955	186.4973	167.8439	171.4067	184.9529	167.4403	168.1113
POMT1	5.06649	4.632625	3.91019	4.790591	4.300686	3.832256	5.220273	4.413163
POMT2	3.099166	2.673184	2.437208	3.548887	3.440803	2.885597	2.53961	2.572303
PON1	0.016542	0.016759	0.098016	0.033904	0.264707	0.266481	0.100741	0.100665
PON2	61.76013	60.97312	51.25805	48.47741	56.80038	59.56246	53.96377	56.4287
PON3	29.78552	40.05078	23.97261	56.84253	40.38681	45.5212	46.90072	28.51071
POP1	1.619488	1.57202	1.775149	2.085366	1.80626	1.886659	2.332275	2.29612
POP4	6.036673	7.179532	6.179178	6.3691	6.48895	6.204756	5.850166	5.654096
POP5	6.732789	7.487024	6.379775	7.17883	7.535285	7.327543	7.858848	6.51972
POP7	21.05504	22.89566	20.46001	22.06565	22.01238	24.44937	23.87838	23.14857
POPDC2	0.013347	0.027045	0.026362	0.013678	0.013349	0	0.040642	0.054149
POPDC3	0.050674	0.119792	0.066724	0.138479	0.067575	0.034014	0.222883	0.359771
PORCN	2.564981	2.117413	1.852858	2.652854	2.897853	2.630328	2.278024	1.806594
POSTN	254.8757	219.9484	223.9015	216.6247	209.4615	211.3994	228.4347	308.1119
POT1	8.608242	8.269748	7.313499	7.284781	6.840794	6.829663	7.696348	7.762293
POU2AF1	0.042168	0.352454	0.093696	0.151245	0.295216	0.244124	0.160503	0.181766
POU2F1	2.871012	2.741845	2.378059	2.480838	2.419355	2.131583	2.124732	2.717015
POU2F2	0.172032	0.141864	0.169888	0.192692	0.272085	0.088618	0.121822	0.182596
POU2F3	14.86715	15.40227	13.32308	19.14457	19.52334	15.26155	14.85171	13.0819
POU3F2	0.035971	0.012148	0.023682	0.040957	0.023984	0.012072	0.016227	0.020268
POU3F3	0.010217	0	0.01009	0	0	0	0.005185	0
POU4F1	0.10602	0.035804	0.191947	0.018108	0.017672	0.053372	0.03587	0.071686
POU4F3	0	0	0	0	0	0	0	0
POU5F1	0.042344	0.085799	0	0	0.021175	0	0	0.042946
POU6F1	1.302921	1.111416	0.91668	1.345572	1.427997	1.192874	1.096344	1.379668
POU6F2	0.021037	0.138532	0.083098	0.032336	0.210393	0.063541	0.138789	0.010668
PP2D1	0.011476	0.046505	0	0	0.022954	0	0	0.011639
PPA1	39.87782	42.27199	42.76171	45.9047	43.97087	43.34639	45.53022	43.66515
PPA2	20.43209	18.49716	27.89556	23.96089	21.27451	27.03148	24.30062	22.62274
PPARA	3.416936	3.281165	3.358059	3.978601	3.942366	3.490138	3.263785	3.301508
PPARD	15.54696	12.77764	14.24732	14.61033	14.80744	13.10263	13.85174	12.61933
PPARG	8.902949	14.31637	10.32564	10.95789	14.38172	11.31512	8.278439	9.871337
PPARGC1A	0.190402	0.270062	0.210594	0.159997	0.175193	0.314393	0.220315	0.316705
PPARGC1B	1.568597	1.222655	1.314347	1.252947	1.663879	1.252284	1.15791	1.312381
PPAT	2.126855	2.743571	2.405638	2.914498	2.442489	2.247209	2.296818	2.771664
PPBP	0	0	0	0.077173	0	0.025274	0	0
PPCDC	2.868817	2.862206	2.473544	3.745733	3.626536	2.844429	3.340497	2.761958
PPCS	10.98425	11.88893	12.54182	10.86386	12.88557	12.01561	12.17529	8.996401
PPDPF	207.1097	193.3164	192.0084	182.8053	203.1122	182.1532	174.7179	181.0288
PPDPFL	0.202938	0.139809	0.180368	0.266195	0.142075	0.216583	0.135948	0.148195
PPEF1	2.133863	3.585097	3.090669	4.555665	4.063755	2.936202	3.284904	2.110134
PPEF2	0.021563	0.03641	0.056786	0.051561	0.050321	0.043421	0.014591	0.051031
PPFIA1	19.96286	16.38625	15.56076	16.01762	18.1077	17.20363	16.33089	16.76817
PPFIA2	0	0.010252	0.009993	0	0.01518	0.010188	0.015406	0.041052
PPFIA3	7.080164	6.42536	5.904857	6.967115	6.561884	6.190236	6.183322	5.740958
PPFIA4	0.327359	0.425949	0.072896	0.279556	0.189379	0.193879	0.221513	0.257152
PPFIBP1	38.73947	35.15404	33.61176	34.49165	31.56893	32.85174	32.94712	40.91849
PPFIBP2	10.7499	8.819298	7.856844	8.306576	9.202999	8.555465	8.686234	10.0179
PPHLN1	15.93098	17.64301	16.20568	16.00272	15.94617	16.96506	15.64361	16.20437
PPIA	1071.006	1131.965	1056.975	1148.871	1115.874	1150.981	1130.797	1017.951
PPIB	301.4877	330.5159	310.1164	311.8395	310.2457	318.5768	322.6828	321.8193
PPIC	14.32418	22.03901	17.06633	11.00925	19.93884	12.19774	14.66563	19.26237

PPID	31.77299	32.45776	35.85561	29.45227	34.4762	36.54205	29.77862	29.22005
PPIE	8.782314	10.84166	8.79628	10.08825	9.345805	10.30704	9.856184	7.857284
PPIF	20.16121	18.44789	18.05618	20.31434	17.68558	18.57901	22.73101	28.76839
PPIG	7.953742	10.74417	8.850453	9.671769	8.698877	9.605131	8.743885	9.373158
PPIH	31.94969	26.13729	31.83564	26.05229	24.18665	28.49225	34.11326	25.42542
PPIL1	22.16141	23.47135	22.32677	22.23586	21.20617	22.45749	22.38039	19.70777
PPIL2	13.17076	14.06203	12.33726	13.61455	14.18412	13.06125	12.45805	14.87071
PPIL3	8.361817	11.3249	17.87356	13.73716	16.15808	18.4865	9.772519	14.01666
PPIL4	9.792791	9.194518	9.631414	8.915136	7.971907	9.90555	8.934157	10.56162
PPIL6	0.051522	0.065247	0.05088	0.092396	0.068704	0.056196	0.065368	0.065319
PPIP5K1	6.179042	6.123396	5.91045	6.561179	6.76197	7.159751	5.62101	5.86489
PPIP5K2	13.97614	13.8887	10.46985	12.80602	12.4981	11.99244	10.97153	15.64311
PPL	95.36833	67.91398	76.45353	86.95635	75.29109	75.96703	85.69988	69.06076
PPM1A	34.2506	32.62623	32.96503	31.09552	32.14085	32.7873	32.59159	32.0514
PPM1B	23.80096	22.87063	23.24079	22.45621	22.74255	24.09153	24.62888	22.18024
PPM1D	19.01366	18.48911	24.07587	18.74665	18.79653	17.70226	17.74783	19.33778
PPM1E	0.045536	0.053822	0.082442	0.046663	0.030361	0.045847	0.092437	0.046184
PPM1F	6.449934	6.028336	5.934541	6.960063	6.431041	5.606948	5.372151	6.715133
PPM1G	33.49767	31.03377	29.69874	28.96217	29.50658	29.90417	33.97495	34.45269
PPM1H	5.319913	4.158457	4.347829	5.996858	5.632498	4.705194	5.153001	5.451463
PPM1J	0.341728	0.428645	0.49817	0.350193	0.227848	0.278527	0.247753	0.198053
PPM1K	20.55129	22.85598	25.45145	21.87921	20.21458	23.18792	20.28183	15.76473
PPM1L	2.771078	3.118663	3.096788	2.947929	2.777836	2.822226	3.312814	3.258392
PPM1M	8.556343	11.23662	8.145115	9.895245	10.9986	8.992874	11.52967	10.84088
PPM1N	0.286291	0.324172	0.349247	0.569507	0.151585	0.135646	0.188026	0.307447
PPME1	20.45485	18.68778	20.69601	18.21265	20.39337	18.9806	17.78952	18.74081
PPOX	8.034744	6.276595	6.786579	8.580611	5.725127	8.445222	7.527937	6.417808
PPP1CA	91.6611	93.92965	99.61706	89.12123	87.77695	93.56198	101.794	92.17577
PPP1CB	155.4317	153.5427	169.7866	150.8784	156.0445	165.2557	157.822	155.7496
PPP1CC	52.90538	57.43514	54.4987	51.40768	49.6963	57.82798	60.32291	57.54636
PPP1R10	12.13205	12.24687	12.03137	13.41309	12.87873	11.99431	12.53644	12.6233
PPP1R11	21.31524	18.51156	21.02818	19.29244	21.51368	19.65513	18.07144	18.91772
PPP1R12A	17.85145	20.65246	18.61338	20.20502	19.54548	17.59325	18.87419	19.11271
PPP1R12B	1.533993	2.112403	1.794323	1.943367	1.747687	1.954332	1.758557	1.706882
PPP1R12C	7.842238	8.2313	7.391231	7.930369	8.502349	7.9906	6.144295	7.953877
PPP1R13B	21.31219	18.26443	22.09213	20.63806	17.45262	20.15566	20.39855	17.89402
PPP1R13L	45.00127	42.4596	44.4868	42.87569	40.03319	40.22287	37.71226	37.29585
PPP1R14A	7.51726	7.194482	6.778064	6.972701	10.40072	10.38067	8.504606	7.473595
PPP1R14B	39.93538	43.53276	41.47606	42.78334	40.87885	44.36441	42.94679	34.92152
PPP1R14C	35.44401	26.36656	22.97857	24.3252	28.12672	23.80949	28.38635	29.90433
PPP1R14D	1.533186	1.587826	1.783253	1.658451	1.380046	1.337839	1.279529	1.382233
PPP1R15A	44.96821	36.8272	44.82737	39.9653	45.23115	44.6902	35.56798	33.9767
PPP1R15B	17.24907	17.34724	17.80028	18.67997	19.16628	17.56634	16.86558	16.27251
PPP1R16A	3.360864	2.891143	2.056837	2.993676	3.097534	2.280847	3.061226	3.305829
PPP1R16B	2.99809	2.735818	2.099429	2.681327	3.531075	2.511958	2.983477	2.802612
PPP1R17	0.016435	0.033302	0.01623	0.008421	0.008219	0.024821	0.016682	0.008335
PPP1R18	6.710604	6.712615	6.291445	6.981286	7.875359	6.191985	6.440523	7.245517
PPP1R1A	0.341576	1.099247	0.654798	0.597122	0.703336	0.971039	0.489459	0.65212
PPP1R1B	2.805793	12.69423	2.05096	6.018385	6.052494	4.902139	3.601731	3.515321
PPP1R1C	0	0	0	0.086917	0	0	0	0
PPP1R2	64.65996	78.70618	86.91996	89.48652	83.30199	112.4406	77.7365	73.74262
PPP1R21	6.188236	6.538805	6.518539	6.144229	6.757511	6.064365	6.811461	6.425102
PPP1R26	3.295319	3.652648	2.252658	3.616195	2.958898	2.77064	2.870813	3.210249
PPP1R27	0	0.032973	0	0.033352	0	0.065536	0.033034	0.033009
PPP1R2C	0	0	0	0	0.044014	0	0	0
PPP1R35	21.8941	23.64395	22.36049	26.40869	22.67228	25.5965	23.03653	21.47372
PPP1R36	3.241061	3.44575	2.96359	2.870297	2.941343	3.202773	2.700784	3.185743
PPP1R37	22.85093	21.17145	20.8493	22.56142	22.86249	22.78173	21.90953	20.77568

PPP1R3A	0	0.004043	0	0	0	0.004018	0	0
PPP1R3B	20.79073	21.79774	19.23727	24.11227	22.14696	21.22246	22.85483	22.07024
PPP1R3C	14.26047	15.32724	15.45463	18.82878	14.69648	17.84356	14.1329	12.44916
PPP1R3D	3.752564	3.754683	3.958475	3.543595	3.64449	3.520595	4.477762	3.790257
PPP1R3E	1.118211	1.201546	1.050737	0.986177	0.948908	1.153144	0.907987	1.2716
PPP1R3F	0.298987	0.432153	0.228336	0.498399	0.398701	0.357222	0.319657	0.363892
PPP1R3G	1.093849	1.340843	0.968899	1.326307	0.89774	0.891145	1.402652	1.024732
PPP1R42	0	0.018955	0.018476	0	0	0	0.01899	0.037951
PPP1R7	29.44256	31.01302	31.44319	26.60959	26.80893	29.76439	25.74756	31.06224
PPP1R8	24.20891	25.99668	26.65999	22.53902	26.22334	26.91168	26.94915	25.28937
PPP1R9A	0.243043	0.299186	0.144525	0.224961	0.274439	0.326271	0.363402	0.344574
PPP1R9B	22.29909	22.76223	21.41194	23.2091	24.64562	20.16738	22.53237	22.04132
PPP2CA	120.5511	124.4633	125.9027	118.3134	118.4144	127.1841	125.0215	117.5201
PPP2CB	66.68007	65.8306	70.25846	64.85726	65.08392	65.43578	66.13661	61.33195
PPP2R1A	61.88893	60.39322	55.15923	58.10944	63.0792	57.30299	63.71715	57.587
PPP2R1B	83.8279	83.81591	92.73419	72.79533	83.01374	91.14699	75.4761	68.14699
PPP2R2A	34.52058	35.25127	35.58794	31.26613	34.27837	36.29235	36.14174	33.9836
PPP2R2B	6.571486	5.14955	4.397038	3.875637	3.359939	3.16641	3.648113	4.067032
PPP2R2C	0.426302	0.00708	0.165635	0.200527	0.39141	0.105544	0.432696	0.25517
PPP2R3A	3.175257	3.594705	3.204026	3.529665	3.963818	3.29758	3.199476	3.789693
PPP2R3C	10.9069	10.85618	12.88397	11.92577	12.4741	13.85374	12.00629	10.77988
PPP2R5A	35.70135	42.43598	47.54708	35.20928	36.72428	47.81066	39.49115	37.5169
PPP2R5B	7.012728	7.033268	5.819035	7.168357	7.2783	7.282668	6.715018	6.173164
PPP2R5C	10.99666	11.15922	11.16433	10.44699	10.74094	10.17351	10.57965	12.39187
PPP2R5D	27.04486	24.30967	25.32772	24.41493	24.58341	25.11894	27.61347	25.95493
PPP2R5E	9.527775	9.863502	9.266559	9.354963	10.10202	9.635594	9.235618	9.938891
PPP3CA	15.01072	18.81533	16.24648	15.5686	16.8519	16.36926	19.51001	19.50126
PPP3CB	5.475933	5.858165	5.275345	5.52655	6.098986	5.542258	5.69087	5.919614
PPP3CC	13.28279	12.93154	11.79483	12.93184	12.02966	11.90378	14.03307	12.46852
PPP3R1	35.26251	36.52967	36.33719	33.07789	34.33611	32.64586	33.41091	34.82041
PPP3R2	0	0	0	0	0	0	0.055056	0
PPP4C	44.18617	42.06988	38.66637	42.08329	42.37159	42.36295	43.68466	42.98473
PPP4R1	17.02735	16.96947	14.49859	16.69275	16.08671	15.19386	18.43989	17.95164
PPP4R2	27.30946	28.3155	29.18265	29.22975	29.25525	31.05625	30.43763	29.95512
PPP4R3A	28.74251	28.97175	25.86402	28.54855	28.67703	26.60921	27.90184	30.18863
PPP4R3B	44.69366	47.56139	44.41226	42.54044	45.69586	46.59057	45.05395	48.54321
PPP4R4	0.671366	0.551029	0.595861	0.809919	0.764946	0.568998	0.707314	0.36632
PPP5C	6.703437	8.278162	5.367686	6.475273	7.060536	6.247163	6.652146	7.333526
PPP6C	13.96791	15.4901	15.23354	14.81796	14.78171	15.26263	15.54886	14.10064
PPP6R1	14.90544	14.77125	13.74293	15.29389	15.25172	14.83708	15.1036	15.3462
PPP6R2	6.603814	6.80199	5.679165	6.868904	6.334977	6.366349	6.373313	6.39084
PPP6R3	25.6312	23.06875	22.81037	22.96952	24.3268	22.68862	24.31091	26.80065
PPRC1	8.816123	7.789429	6.963493	8.30636	8.716847	7.461112	7.384459	7.719643
PPT1	105.1209	116.108	121.5466	122.7283	108.9506	126.0859	104.0234	102.3567
PPT2	26.17194	27.07066	32.83427	24.01208	30.39683	31.83966	27.60875	26.9931
PPTC7	5.662778	6.470106	5.839762	5.773857	6.432699	5.38599	5.741938	6.142078
PPWD1	14.46706	16.40982	14.74681	14.02072	14.48224	16.4285	15.46757	14.07907
PQBP1	20.21682	24.55203	21.62507	21.45592	19.8264	21.21222	25.70549	22.25394
PQLC1	17.64822	13.06228	12.245	12.2872	10.17372	16.35068	12.42665	11.81901
PQLC2	4.256204	4.054276	6.618851	4.272739	5.372995	8.780166	5.018532	5.272822
PQLC3	13.74744	15.77447	16.38289	22.61989	19.72781	14.12129	14.9257	14.6168
PR	23.82667	23.41228	16.4425	26.47612	27.84923	21.82173	27.09782	20.23527
PRADC1	13.08718	13.67749	15.25541	14.22783	14.09231	15.15234	14.31682	12.28583
PRAF2	10.03749	10.66702	11.59338	11.70676	13.63661	11.9082	10.20587	10.96349
PRAG1	5.344518	5.452525	4.82682	5.808838	6.01388	5.284865	5.563781	5.205799
PRAM1	0.181624	0.193692	0.1416	0.293878	0.162526	0.163616	0.106728	0.155124
PRAP1	0	0	0	0.044746	0	0	0	0
PRC1	10.37643	11.05468	10.14319	9.500898	8.026762	9.626013	10.26499	10.31064

PRCC	24.33036	25.06452	24.18373	25.60983	24.50524	24.05771	24.57466	25.49392
PRCD	0.450228	0.076023	0	0	0.375238	0	0	0
PRCP	12.27179	10.80127	12.93256	11.65465	10.73764	12.0791	10.77059	12.09196
PRDM1	29.02746	22.92074	28.39103	25.40389	24.61914	26.40472	24.63727	24.03424
PRDM10	1.829789	2.032483	1.689427	1.64016	2.037282	1.895561	1.991494	2.271724
PRDM11	1.256716	1.361524	1.044975	1.436729	1.339217	1.080016	1.317348	1.559489
PRDM12	0	0	0.025381	0	0	0	0.026087	0
PRDM13	0	0	0	0	0	0.013249	0	0
PRDM16	0.101277	0.164791	0.096984	0.21386	0.245551	0.166857	0.115256	0.164972
PRDM2	10.59049	9.158188	8.83307	8.164939	9.067335	8.236339	9.333339	10.51764
PRDM4	8.73725	8.058488	7.337151	8.632655	8.574918	8.412973	7.983552	8.405771
PRDM5	3.227132	3.133256	3.156278	2.236512	3.496512	2.280673	2.572138	3.482885
PRDM6	0.174639	0.263239	0.134606	0.266265	0.213002	0.227296	0.172936	0.146885
PRDM8	1.830219	2.188336	2.141216	2.264183	2.654986	1.975531	2.828348	2.567009
PRDX1	254.0024	254.8445	248.2726	267.7691	287.6652	276.2122	284.9594	272.8349
PRDX2	312.7985	308.7239	313.25	310.7434	334.3963	343.0419	325.1887	303.8764
PRDX3	26.53158	33.9645	30.36504	34.26603	29.06876	30.92329	30.46967	30.05954
PRDX4	43.61645	58.3146	49.59919	52.92977	45.28092	52.1076	53.05644	45.99844
PRDX5	93.93175	86.03378	91.79203	96.59377	114.8567	90.8001	85.67986	79.21983
PRDX6	108.8882	114.1168	144.6953	133.0141	135.9209	128.6588	139.7051	98.46746
PREB	19.21334	16.44045	13.96123	14.62061	16.16466	16.57361	17.31421	22.02805
PRELID1	125.1583	121.3468	138.533	129.2428	131.05	140.632	126.7409	123.6322
PRELID2	3.22993	3.044774	2.640468	3.019495	3.064552	3.300453	2.985279	3.194555
PRELID3A	1.801066	1.533084	1.058511	1.646222	1.727273	1.203393	1.56653	1.618177
PRELID3B	31.32052	34.26775	32.92699	34.21768	35.62893	38.28149	32.89463	35.06705
PRELP	56.4583	45.62603	42.72685	53.6268	50.6572	38.34243	42.85013	46.62884
PREP	40.77619	39.18943	56.54756	39.61939	37.04308	42.3765	38.39742	40.24881
PREPL	11.52821	10.00517	10.93675	10.76561	12.05882	11.06416	10.08507	10.35854
PREX1	2.727916	2.902534	1.980466	2.591251	2.728269	1.630373	2.515973	4.017472
PRF1	0.389753	0.35785	0.264617	0.474297	0.377622	0.527309	0.1978	0.370595
PRG4	0.20585	0.202034	0.076232	0.15162	0.360284	0.226687	0.195879	0.241402
PRICKLE1	10.35252	9.182001	7.723516	9.643522	7.50615	7.985676	7.542969	8.108457
PRICKLE3	5.20934	4.204275	3.923718	5.050483	4.768487	4.170081	3.601026	4.420554
PRICKLE4	23.59658	22.63959	23.80467	23.25005	22.97442	22.00385	20.39729	21.08359
PRIM1	10.88353	13.33007	11.76861	12.34998	11.68089	10.88503	12.65187	13.38662
PRIMA1	3.492706	2.399149	3.019015	2.54847	3.136712	3.221525	3.143158	2.835548
PRIMPOL	3.854038	3.66138	2.88259	3.366791	3.614418	3.015236	2.88579	3.204019
PRKAA1	8.00393	9.080555	8.177386	8.895155	8.693563	8.269049	8.342376	8.516906
PRKAA2	0.157822	0.190642	0.131878	0.186613	0.291402	0.256685	0.21564	0.190851
PRKAB1	15.07903	16.26516	14.89115	16.91078	15.10394	13.7609	14.82766	17.43528
PRKAB2	4.050257	5.422921	4.801766	5.88828	5.22566	5.538369	4.753842	5.371889
PRKACA	31.82261	28.64481	29.98787	30.34646	29.56654	31.0456	30.75376	28.60833
PRKACB	3.955631	5.054169	3.54161	4.541802	4.577133	4.068882	4.313778	3.941366
PRKAG1	16.1361	17.0038	16.44326	16.97811	18.01434	16.54719	15.4514	15.06934
PRKAG2	21.94425	21.76042	21.7996	19.57662	21.81667	20.2186	18.53912	19.97052
PRKAG3	0.070175	0.060158	0.06397	0.082977	0.129571	0.048915	0.049312	0.197098
PRKAR1A	118.5428	115.6951	110.9638	115.9016	112.3079	112.1617	126.7118	112.2399
PRKAR1B	1.672499	1.878184	1.522312	2.395368	1.8944	1.592627	1.881662	1.655433
PRKAR2A	51.47608	52.86954	54.6349	51.88538	54.16481	52.01433	51.39623	53.06023
PRKAR2B	4.856278	5.396438	5.331608	5.616018	6.132183	6.318959	4.5895	4.778657
PRKCA	9.98954	10.19712	7.819164	9.102407	8.514436	7.946578	8.019562	9.4597
PRKCB	1.084475	1.108547	0.802422	0.978641	1.401905	0.694242	0.883879	1.385562
PRKCD	16.17737	14.10767	11.8863	13.92083	14.38346	13.8408	14.14951	13.12613
PRKCE	4.735424	3.781177	3.872021	3.999134	3.815524	3.715991	3.713442	4.056039
PRKCG	0.067444	0.068329	0.123692	0.187596	0.125269	0.048503	0.058676	0.078176
PRKCH	22.85589	19.83394	21.99447	22.75134	20.68334	21.79747	21.19278	19.67999
PRKCI	17.12648	17.93683	16.73936	17.33181	16.43751	18.47696	17.96367	18.15405
PRKCQ	0.391178	0.630863	0.417839	0.531763	0.558898	0.305435	0.469972	0.421037

PRKCSH	37.98017	37.53421	36.37394	40.30487	34.78181	34.72611	38.27088	36.14511
PRKCZ	11.67786	10.53442	9.847082	11.29607	11.79845	10.96847	11.06753	10.60636
PRKD1	2.83981	2.554846	2.856774	2.86359	3.650574	2.699094	2.68256	2.972404
PRKD2	13.64672	11.77325	9.460104	11.06245	11.29346	9.644908	11.19754	12.26692
PRKD3	15.977	15.13661	15.87625	14.25775	14.05071	14.40006	17.04816	17.46683
PRKDC	2.344978	2.198793	1.50683	1.841389	2.172598	1.754577	2.135617	2.246003
PRKG1	0.386105	0.513847	0.446723	0.348844	0.715186	0.36804	0.586685	0.634904
PRKG2	0.005351	0.113838	0.073975	0.027416	0.032108	0.032323	0.016293	0.054268
PRKN	1.135913	1.45256	1.196999	1.369888	1.510128	1.094857	1.511491	1.243407
PRKRA	1.933678	1.361377	1.505011	1.141925	1.753646	1.56741	1.580126	1.180047
PRKRIP1	9.018622	9.306185	8.576387	8.859449	9.638792	8.694471	9.323418	7.791895
PRKX	13.95451	14.50712	10.4918	9.975617	12.44524	13.57371	11.57268	10.06411
PRL	0	0	0	0	0.033364	0	0	0
PRLH	0	0	0	0	0	0	0	0
PRMT1	65.17392	64.38343	53.98565	62.98535	62.46952	60.27719	68.28447	62.92738
PRMT2	8.295068	7.522875	6.368394	8.034381	8.483472	6.96427	7.645444	7.639686
PRMT3	7.693894	7.156984	7.977267	6.731319	7.592171	7.679017	7.360597	7.916646
PRMT5	28.61115	28.63656	26.02521	25.54274	26.76018	25.73344	27.48542	29.68433
PRMT6	7.319495	7.193828	7.000157	7.027329	7.065077	6.328954	6.688827	6.141194
PRMT7	16.42767	14.55703	12.87159	16.51629	17.25819	15.04575	14.81752	15.42475
PRMT8	0.521142	0.399206	0.326363	0.468923	0.94072	0.319941	0.399945	0.696154
PRMT9	10.17367	10.47923	7.478005	8.282003	8.592379	8.820939	9.323418	10.99961
PRND	0.02938	0.009922	0.029014	0.050179	0.029384	0.00986	0.00994	0.029798
PRNP	64.55479	67.80694	62.08167	63.87243	60.02286	62.04543	59.50645	59.35282
PROB1	0.744304	0.658406	1.031236	0.563517	1.916553	1.476408	0.64835	0.529556
PROC	2.325035	2.948931	4.39933	6.929626	2.857855	2.859137	7.962447	1.530087
PROCA1	6.851079	4.803675	5.6931	6.228804	6.84152	5.530924	6.116416	3.961554
PROCR	51.68223	45.18732	59.66906	44.62977	44.16516	57.12188	45.02771	52.64237
PROK1	0.03039	0	0	0	0.091182	0	0	0
PROK2	0.420161	0.327442	0.127669	0.198724	0.61416	0.423031	0.295244	0.163901
PROKR1	0.067705	0.013719	0.053489	0.090196	0.142198	0.115884	0.06872	0.013734
PROKR2	0.043171	0.080185	0.113688	0.088481	0.050373	0.072443	0.102243	0.131357
PROM1	1.11464	2.395796	1.398745	1.842746	2.629905	2.808737	3.206561	3.104212
PROM2	29.74778	37.94877	27.99509	41.85609	34.46457	36.75294	33.67598	28.5509
PROP1	0	0	0.162352	0	0	0	0	0
PROS1	13.60931	13.81088	18.82234	17.37205	16.93167	12.66405	18.81665	16.91764
PROSER1	6.819345	5.926019	5.538287	6.070612	6.137056	5.802522	5.966123	5.89759
PROSER2	8.300331	8.012868	7.313739	7.346033	6.736156	7.794272	7.885874	8.375974
PROSER3	0.905244	0.941912	0.507385	1.215996	0.880892	0.57888	0.484244	0.682394
PROX1	0.305964	0.718888	0.498228	0.320214	0.556665	0.327717	0.67727	0.722978
PROX2	0.060979	0.012356	0	0	0	0	0	0.012369
PROZ	0.13408	0.237719	0.049653	0.085876	0.452578	0.067498	0.068046	0.135989
PRPF18	10.86081	10.87798	10.41637	10.18992	10.41084	10.53924	10.12232	10.80132
PRPF19	41.1451	38.59859	35.9562	38.13026	38.05786	37.41683	41.18321	40.90917
PRPF3	29.04297	27.40629	22.95736	24.64359	27.6766	25.45358	24.48351	28.14178
PRPF31	21.74728	22.00395	19.85123	18.56437	19.16789	20.23902	20.00005	21.49574
PRPF38A	10.32112	10.94084	10.6121	9.639437	11.21954	11.16401	11.09287	10.31243
PRPF38B	20.3764	19.07	16.29179	18.95407	19.97927	18.97899	17.82363	19.50554
PRPF39	17.21063	17.622	15.13305	18.20798	17.49947	17.45644	15.67505	16.86624
PRPF4	15.09678	13.94138	11.04957	14.91169	14.0298	14.91968	13.89939	13.43725
PRPF40A	30.47598	31.71336	31.32768	31.73472	31.82682	34.53674	31.59127	31.17
PRPF40B	6.191642	7.30043	5.395616	7.476552	6.527832	6.192765	5.404518	6.329557
PRPF4B	27.36277	25.03519	24.13999	25.04623	25.65567	24.37364	22.89624	26.56916
PRPF6	23.56155	23.52428	21.62488	21.96653	22.79729	20.66064	22.72345	23.69072
PRPF8	55.92442	55.85866	45.78597	50.41627	50.17058	44.35433	52.22582	54.24849
PRPH	0.231188	0.187378	0.31963	0.142149	0.184974	0.310357	0.234656	0.468958
PRPS1	19.05862	21.99338	20.88443	21.2279	22.25917	19.45818	23.09444	20.54451
PRPS1L1	0	0	0	0	0	0	0	0

PRPS2	5.517804	6.73529	5.005362	5.100544	5.350013	4.622514	7.161035	5.624833
PRPSAP1	44.26142	41.08476	37.75947	33.76153	39.24336	37.82427	40.44863	41.34447
PRPSAP2	12.05726	13.9883	12.21971	15.37872	12.85885	13.43161	13.01621	11.43448
PRR11	2.155957	1.098588	1.222024	1.647215	1.441743	1.027543	1.217159	1.811424
PRR12	6.067526	5.820899	4.595747	5.553782	6.245057	5.452608	5.146067	5.600249
PRR13	31.18793	30.11984	32.28586	31.06865	36.69642	37.25019	31.63187	33.15863
PRR14	12.04125	10.33054	10.6018	11.06521	12.57136	11.67488	9.918434	10.98299
PRR14L	6.809192	5.79054	4.970286	5.042012	5.654456	5.33793	5.277594	5.587039
PRR15	2.872788	2.577863	1.459025	1.724312	2.585844	0.950365	1.541251	1.581714
PRR15L	1.273889	1.659353	2.318344	2.3125	1.41966	1.484142	1.200641	1.882663
PRR16	2.186419	2.950957	1.671143	2.486121	2.456296	2.435062	1.793613	1.807451
PRR18	0	0	0	0	0	0	0	0
PRR19	0.641463	1.148124	0.717933	0.679264	0.662931	1.033352	0.629382	0.845773
PRR22	0.242821	0.295209	0.079932	0.298603	0.226662	0.228181	0.394341	0.147767
PRR29	0.254601	0.386914	0.176	0.165242	0.297073	0.247796	0.163666	0.241011
PRR3	7.526498	7.511804	5.452871	7.10462	7.773906	6.788549	7.116461	6.804393
PRR30	0	0	0	0	0	0	0	0
PRR32	0	0.139687	0	0.023549	0	0.023137	0	0.06992
PRR35	0	0	0	0	0	0	0	0
PRR36	1.470684	0.868601	0.846667	0.973203	1.015761	1.162009	0.789883	1.083599
PRR5	6.201649	7.729574	6.318117	7.204124	6.300556	6.463489	6.880997	5.637726
PRR5L	3.540653	6.006907	3.318746	3.775951	5.065589	4.374483	2.472996	3.822345
PRR7	0.258041	0.134931	0.295927	0.226048	0.328837	0.205329	0.502702	0.287042
PRR9	3507.792	3012.593	3112.84	2970.55	3048.439	3000.367	2737.036	2701.426
PRRC1	11.24714	10.93993	10.68731	11.26198	11.41023	10.89006	11.41585	10.6666
PRRC2A	49.85621	45.13257	40.28942	45.62624	45.32262	41.48411	43.70935	47.86157
PRRC2B	26.13853	25.23915	21.74153	23.73335	23.66888	21.13135	24.14754	24.6427
PRRC2C	28.90775	24.84993	20.98114	24.99803	26.0922	22.04556	22.8736	26.43886
PRRG1	3.276749	4.003042	3.857971	4.029498	4.040786	3.798809	3.765048	4.775365
PRRG2	8.564793	6.938096	8.083349	8.573261	8.403256	8.350414	7.629532	8.393496
PRRG3	2.040253	1.278059	1.294503	1.624967	1.508361	1.355269	1.384148	1.715479
PRRG4	16.45066	15.50877	17.92941	15.20539	13.43875	15.2547	15.07913	17.61957
PRRT1	1.052224	0.942598	1.411007	1.293946	0.841888	1.092868	0.494656	0.640322
PRRT1B	0	0.03215	0	0.06504	0.031738	0.031951	0.161049	0
PRRT2	0.366516	0.285636	0.396752	0.397264	0.479353	0.326441	0.257548	0.235908
PRRT3	0.153078	0.1306	0.167084	0.107332	0.193387	0.137901	0.294394	0.065372
PRRT4	0.056633	0.184878	0.093212	0.090278	0.11328	0.114039	0.140512	0.108496
PRRX1	20.27977	23.56542	17.0404	23.51511	21.26132	22.4225	25.36717	20.96444
PRRX2	6.977525	6.828788	6.383062	7.312408	10.33914	9.473063	7.062125	6.415279
PRSS12	1.739178	2.02043	1.694607	2.35258	1.376061	1.042853	1.678963	1.771776
PRSS22	16.24199	13.27028	10.83831	14.15755	12.35544	10.93898	10.24403	10.20835
PRSS23	22.98026	20.32933	29.27426	19.02313	19.99268	22.69406	25.12749	33.00598
PRSS27	5.952447	5.880872	4.773495	5.948475	4.623242	3.697877	8.269891	4.303099
PRSS33	1.007034	0.502028	0.347281	0.80265	0.799337	0.724224	0.519182	0.340457
PRSS35	0.079264	0.151687	0.069579	0.108304	0.281866	0.310357	0.178785	0.116123
PRSS36	7.183313	5.595913	5.970386	5.982846	5.087645	5.481918	7.218443	5.101352
PRSS37	0.145967	0	0	0.224374	0	0	0.111117	0
PRSS42P	0.241564	0.212103	0.143132	0.132025	0.080532	0.081071	0.147112	0.179668
PRSS50	0.068059	0.017238	0.084013	0.069745	0.017017	0	0.08635	0.189826
PRSS53	24.52907	19.77338	23.28115	20.82047	20.59937	23.78447	21.3948	20.20564
PRSS54	0	0	0	0	0	0	0.024533	0
PRSS56	0.012033	0.32916	0.415913	0.209631	0.264764	0.109038	0.13435	0.158658
PRSS57	0.012174	0.024669	0.024046	0.06238	0.012176	0	0.024714	0.012348
PRTFDC1	2.7336	3.259649	3.201224	3.507838	2.189582	3.215047	3.904089	3.925685
PRTG	0.013221	0.020092	0.006528	0.013549	0.019834	0.073213	0	0.006705
PRTN3	0.031734	0	0	0.03252	0	0.063901	0.03221	0
PRUNE1	6.907964	6.877958	5.788525	6.730354	7.402347	6.347008	5.664531	7.964062
PRUNE2	2.682582	4.200628	3.705744	3.900159	3.895364	3.036842	4.063912	3.713423

PRX	3.470401	3.589375	3.170332	3.967049	3.381306	3.215095	3.868644	3.757629
PRXL2A	15.65789	18.78357	15.85873	17.25612	18.53181	18.13232	15.87077	14.55098
PRXL2B	8.043174	11.32072	12.66075	10.45513	12.28227	12.44611	12.30052	10.97727
PRXL2C	8.552245	8.076349	10.23719	7.946105	11.45631	9.289086	7.754605	10.1144
PSAP	216.648	216.4437	216.7444	241.0436	210.6381	221.251	234.7942	241.2894
PSAPL1	21.54572	21.20542	19.80034	29.24824	27.72019	25.28142	16.80423	15.5299
PSAT1	50.61685	46.41643	80.4029	68.0603	76.48872	62.57295	56.02255	43.57813
PSCA	0	0	0.006753	0.021023	0	0.01377	0.013882	0
PSD	0.411122	0.383196	0.200293	0.308957	0.296046	0.231801	0.239245	0.378057
PSD2	0.274864	0.237719	0.152271	0.370983	0.254785	0.269992	0.142896	0.190384
PSD3	5.237366	4.822752	2.826861	4.324099	4.81391	4.061603	5.262122	4.962455
PSD4	4.880496	4.29303	3.984272	4.94647	4.904097	3.869406	3.488961	5.070325
PSEN1	25.46946	23.06368	24.1049	23.58801	24.80139	24.3603	24.10371	24.10529
PSEN2	2.8964	2.146205	2.962134	3.48685	2.981147	2.727437	2.958875	3.165798
PSENEN	88.24477	92.26378	95.38814	99.66519	95.6074	100.6382	92.89828	90.13276
PSIP1	39.57626	43.13135	38.36476	36.68729	39.87586	37.89353	39.34366	40.94772
PSKH1	10.61263	10.56109	8.458711	9.843339	9.377336	9.407199	9.300665	9.833507
PSKH2	0	0	0	0	0	0	0	0.021408
PSMA1	83.39016	88.67253	86.86662	86.11839	82.82353	89.19135	87.03187	75.58318
PSMA2	57.293	59.89803	61.45241	67.32277	63.87461	65.01245	59.9896	54.08912
PSMA3	79.90734	100.7647	104.3056	104.1953	90.17017	106.5172	96.77182	83.20607
PSMA4	57.07036	75.97809	80.08453	70.98849	68.68797	72.97259	68.64906	65.03387
PSMA5	88.48903	96.73246	97.79612	93.32461	98.12733	97.89119	96.43294	90.31142
PSMA6	121.2209	139.9053	135.0693	130.3801	135.4997	154.8931	132.8698	125.3667
PSMA7	125.2994	129.0235	144.3931	135.8508	136.2186	150.5094	138.8071	129.5026
PSMA8	0.227412	0.460793	0.190028	0.268898	0.174955	0.317029	0.230823	0.070969
PSMB1	162.4342	179.8097	175.6205	168.6794	162.3905	169.2451	170.3226	152.5055
PSMB10	11.64126	11.08866	9.791033	11.21613	13.70393	10.43094	10.37423	11.55277
PSMB11	0.089997	0.030393	0.029625	0.030742	0.120012	0.030204	0.060898	0.060852
PSMB2	85.74324	101.6036	92.46874	97.89011	85.41604	93.82106	98.07257	84.79118
PSMB3	128.808	143.7975	149.7368	139.8548	138.3003	141.7393	151.2945	132.0354
PSMB4	145.1333	159.123	171.9494	163.5651	167.7515	195.9347	180.7467	157.8113
PSMB5	88.87396	84.32385	84.46294	91.71533	93.34645	96.75636	100.0993	86.99501
PSMB6	101.0351	109.8245	113.1417	100.3777	110.1292	110.436	99.50758	99.01997
PSMB7	130.3272	136.9384	150.5726	132.5239	129.4491	149.7417	135.7445	124.2135
PSMB8	30.58262	38.457	29.17045	37.41491	44.6335	35.60697	33.50279	32.65771
PSMB9	10.77346	15.26604	12.72394	16.00101	19.40202	14.07186	13.00386	12.36651
PSMC1	82.66895	85.14873	83.83281	81.51015	79.19031	85.15545	85.09997	76.67352
PSMC2	109.9201	126.7902	122.6474	108.89	119.9504	116.8601	129.0922	113.4178
PSMC3	127.4942	136.6928	140.0625	132.399	121.3021	135.3019	142.4821	131.4929
PSMC3IP	2.704383	3.370289	2.836126	2.881736	2.908186	2.987915	3.170051	2.973478
PSMC4	76.57449	75.56517	70.62603	73.76339	74.49941	81.27608	78.25211	69.73563
PSMC5	89.77696	89.88315	91.46539	87.23807	91.61463	95.29938	94.5984	84.17378
PSMC6	57.54995	65.75985	67.4714	65.7398	62.14426	67.29341	67.82486	61.7166
PSMD1	50.12213	50.82356	52.10006	52.85517	51.37752	52.81436	54.88619	47.61257
PSMD10	15.69063	16.39002	16.30252	16.13277	14.28345	16.30574	19.77507	21.22454
PSMD11	40.10887	40.91631	42.0652	40.47502	40.29583	40.26731	40.56487	37.87602
PSMD12	28.69385	34.08282	33.93979	33.0153	33.83654	36.37655	31.0018	32.46744
PSMD13	46.71666	46.5569	48.06567	46.44247	44.77771	47.12234	47.00299	44.53749
PSMD14	45.57422	48.76952	53.25258	45.53553	47.46928	52.7533	47.87077	47.81951
PSMD2	96.57119	93.59187	93.44869	92.90073	90.35247	91.05388	99.71192	89.32724
PSMD3	80.78333	73.8915	74.88534	80.93128	79.82399	78.18207	79.08236	76.29834
PSMD4	108.4238	120.6056	118.183	116.4538	103.7899	110.2443	123.4756	107.6639
PSMD5	38.66334	40.87683	38.26206	43.28116	40.12163	43.87716	37.25839	35.1505
PSMD6	56.20241	60.65483	65.52026	57.32318	61.8123	63.76476	58.43074	57.84957
PSMD7	61.4379	67.20076	69.30234	67.6463	67.40251	69.88875	68.33274	59.86747
PSMD8	158.6503	154.6896	160.3716	164.4791	168.227	165.8755	156.2495	137.905
PSMD9	20.28939	22.02094	21.77771	22.69754	24.12173	21.80096	25.01167	22.60391

PSME1	90.72724	115.4338	111.7949	110.6125	105.5092	105.1229	96.3867	89.07266
PSME2	25.63878	34.92472	31.43154	29.42512	29.61868	31.96668	30.03919	28.54653
PSME3	30.09508	26.89966	26.64181	29.52858	28.20618	25.80788	27.53329	28.36879
PSME4	10.37058	9.887056	9.352274	11.16078	10.36432	9.921266	9.542926	10.25237
PSMF1	34.56513	37.41249	33.51441	33.52496	37.0483	34.60164	36.03952	28.28706
PSMG1	32.35665	35.83009	39.39614	33.56749	38.24704	39.79037	33.1123	31.0346
PSMG2	33.3369	37.602	36.67873	36.50744	39.19522	39.40431	37.13154	34.54006
PSMG3	36.28713	40.63319	32.69157	34.80139	37.60913	41.1986	36.25211	36.84823
PSMG4	73.96737	79.7491	73.1009	79.1201	84.70471	73.9102	79.89677	70.48739
PSORS1C2	926.3601	1068.021	991.569	1131.997	890.3329	1159.924	840.0948	762.1566
PSPC1	16.96367	19.35501	16.08009	20.95426	18.11245	19.19872	19.6363	16.06057
PSPH	38.10506	38.10112	55.22624	51.32254	65.82635	59.64444	49.65497	33.95674
PSRC1	6.199032	6.15857	4.894794	4.805511	4.930471	5.098025	6.536105	5.352318
PSTK	0.437704	0.612765	0.353659	0.334371	0.533272	0.408643	0.654288	0.330933
PSTPIP1	1.772579	2.282502	1.63023	2.204744	1.908137	1.355546	2.389735	2.792786
PSTPIP2	10.50404	9.842659	10.98146	10.0887	12.18064	10.21675	11.80235	14.47878
PTAFR	1.15885	1.193958	1.370706	2.066479	2.468603	1.186542	1.435402	2.47022
PTAR1	4.143362	3.944897	3.370912	3.599746	3.990917	3.528626	3.949478	4.117972
PTBP1	130.3059	129.8573	117.1501	123.9983	120.9794	118.1084	130.6739	129.292
PTBP2	4.101933	4.864039	4.000915	5.18753	4.136159	4.64726	4.522528	4.66435
PTBP3	24.95183	27.4512	25.88742	25.4426	24.03332	26.82885	23.89854	25.25827
PTCD1	2.827253	2.903507	2.295948	2.600297	2.177073	2.515873	2.372864	2.815246
PTCD2	12.46025	12.04536	13.54535	13.61722	13.63239	13.37885	12.32843	13.81019
PTCD3	21.21262	23.54958	21.42106	21.82527	21.87501	21.79668	23.88473	24.34148
PTCH1	25.2926	24.85163	19.94242	21.2477	20.106	20.33961	21.85013	23.02878
PTCH2	15.37478	16.20092	12.05147	17.85418	15.81335	16.54627	16.79128	16.20568
PTCHD1	0.249683	0.399036	0.434106	0.378397	0.619014	0.5134	0.446177	0.431574
PTCHD4	0.017187	0.008706	0.008486	0.035226	0	0.017304	0	0.008716
PTCRA	0.124065	0.050277	0.294047	0.355988	0.124081	0.074948	0.201482	0.125832
PTDSS1	53.13967	50.99567	52.88122	51.44226	53.93011	59.24166	62.30388	54.96716
PTDSS2	14.81627	12.76894	14.04384	16.74801	15.06408	15.12594	13.09467	13.07169
PTEN	15.60122	16.17802	16.21676	14.66111	16.73874	15.89267	14.658	16.9469
PTER	7.008151	6.483999	5.719693	7.181745	5.763646	5.510699	5.849344	4.06796
PTGDR	0.660295	0.390227	0.326034	0.375917	0.513629	0.184668	0.16755	0.334847
PTGDR2	0.711589	1.11143	1.268804	0.840623	1.383824	0.796056	0.63198	2.435801
PTGDS	30.03353	64.80946	47.71918	77.66739	77.10097	69.04753	43.95284	74.07922
PTGER1	0.297155	0.07168	0.195635	0.130507	0.283041	0.18521	0.215437	0.186572
PTGER2	4.926003	4.48816	3.372954	4.412687	4.193844	3.926866	3.958725	4.527383
PTGER3	2.191369	2.871544	1.917853	1.990157	1.889808	1.611817	1.904589	2.934584
PTGER4	10.77967	10.88769	9.047732	10.21776	9.476766	10.06383	10.57274	10.00389
PTGES	10.19871	9.345743	12.75929	9.9229	13.15116	10.90295	9.246738	9.26883
PTGES2	11.44785	10.16934	8.077489	11.23836	10.52011	9.438003	10.37341	9.709336
PTGES3	100.8005	104.8847	109.3763	94.4698	102.3248	104.1195	99.84718	96.73557
PTGES3L	1.221134	0.765861	0.650813	0.794528	0.426483	0.565949	0.373803	0.884655
PTGFR	2.593571	4.083376	3.290885	2.495944	4.576282	3.668117	3.072082	4.041345
PTGFRN	14.81807	14.43896	12.31851	15.69816	14.21633	14.29275	16.50781	17.16924
PTGIR	0.580355	0.92259	0.647677	0.865503	1.434559	0.674581	0.632163	0.880533
PTGIS	3.056853	2.271745	4.054297	2.676838	3.600343	1.743893	2.926902	3.522931
PTGR1	35.68199	47.391	61.98404	49.18957	47.53744	51.87214	54.77664	50.63873
PTGR2	9.009441	8.647277	9.057381	9.309334	7.999721	8.455368	8.333983	7.720214
PTGS1	4.683986	4.73489	5.61462	6.371506	6.604335	3.896727	7.422553	5.60769
PTGS2	3.049121	3.790103	2.018012	2.547537	2.767898	2.211337	2.139453	3.043569
PTH1R	4.995211	5.457532	4.514654	6.60139	6.586537	5.222462	5.678556	3.817979
PTH2	0	0	0	0	0	0	0	0
PTH2R	0.22989	0.048522	0.009459	0.058896	0	0.028933	0.019445	0.01943
PTHLH	1.841392	1.27924	1.6799	2.480063	1.438226	1.69506	1.869013	1.19171
PTK2	12.32026	10.99986	11.2977	12.15696	12.08944	12.18196	12.29626	13.18727
PTK2B	8.524757	7.215592	6.880966	6.893839	8.371504	7.275893	7.362567	8.029191

PTK6	7.87884	6.903057	6.356933	7.161206	6.814516	6.712254	5.5364	4.870971
PTK7	30.77485	27.38595	23.83138	29.10227	25.77403	24.23982	27.58379	26.5978
PTMA	1018.04	1164.324	1236.892	1103.586	1007.695	1190.546	1015.789	936.2829
PTMS	68.38903	68.52453	86.66377	71.90627	73.82283	70.39996	64.81951	69.62666
PTN	28.59836	34.40839	23.66314	28.87322	20.35993	26.39141	33.61331	36.07663
PTP4A1	49.11937	48.91145	49.86203	48.63497	51.13179	48.79337	47.16503	56.9389
PTP4A2	29.62177	32.34282	39.4482	32.46788	32.36262	34.67262	32.95707	33.25389
PTP4A3	2.003201	1.761711	1.414991	2.337935	2.629541	1.834806	1.877931	1.678989
PTPA	44.58926	45.13091	44.28833	43.7559	47.04507	45.78094	44.61467	42.59776
PTPDC1	1.530146	1.369267	1.375847	1.299588	1.137337	1.079019	1.559141	1.636469
PTPMT1	24.94139	27.78993	27.03293	31.89169	29.61474	27.47657	28.60767	19.02906
PTPN1	19.89511	17.69386	15.94237	17.74303	16.49127	15.64204	17.76058	19.18892
PTPN11	41.90876	37.01215	39.12023	35.09516	37.56914	35.96326	39.29013	42.25647
PTPN12	16.3068	16.75863	17.40179	16.37394	19.40791	16.70969	15.23301	18.52297
PTPN13	28.36437	25.25542	27.4324	25.00609	27.52207	26.5268	27.09612	26.32129
PTPN14	28.14039	22.9431	19.93694	21.18577	23.32942	17.87125	21.91113	26.63016
PTPN18	5.051431	6.699363	8.743621	8.204785	6.423842	7.488569	5.987395	5.553114
PTPN2	26.61827	28.28284	26.24557	26.0324	28.87567	26.52824	26.45885	28.74575
PTPN21	8.99171	7.33009	6.150462	6.330022	7.229109	6.866367	7.701661	7.919441
PTPN22	0.13265	0.196787	0.15439	0.145646	0.194263	0.085858	0.1683	0.345956
PTPN23	6.773103	7.380384	6.32406	7.394952	7.723602	5.996163	6.499911	8.640405
PTPN3	12.26847	11.92784	10.87959	12.60055	11.93992	12.86927	11.87082	12.57332
PTPN4	4.899157	5.022129	4.315565	3.898247	4.712856	4.156022	4.221804	4.715248
PTPN5	0.541485	0.624259	0.497859	1.167198	0.494869	0.432387	0.521179	0.511318
PTPN6	14.46695	16.3981	14.44406	15.39685	17.10986	14.16468	15.35403	17.93995
PTPN7	0.935181	0.932534	0.458128	1.282825	0.972125	0.585701	0.85952	1.127737
PTPN9	12.46988	11.00213	10.63979	11.61488	12.53373	11.38023	10.20924	12.31602
PTPRA	36.24217	34.6643	35.26426	33.52356	33.64626	33.58717	35.82682	34.34196
PTPRB	5.363275	4.838415	4.011845	5.0674	7.865325	5.880981	5.350907	6.18624
PTPRC	7.402048	10.04464	6.026455	8.900498	10.34904	4.77578	8.997008	12.48857
PTPRCAP	2.794336	3.968053	2.2619	2.863552	3.688083	1.383652	3.649929	2.996728
PTPRD	1.285539	1.011694	0.920969	1.367372	1.090553	1.077638	1.057255	1.446447
PTPRE	8.651022	8.49339	8.64707	9.32572	8.327088	10.28853	9.877174	9.520707
PTPRF	59.3723	52.55749	41.66187	56.30937	49.28197	45.98616	49.30395	48.44758
PTPRG	3.880008	4.30758	3.275124	3.223225	3.954283	3.505465	4.063991	4.850971
PTPRH	0.369037	0.140205	0.125276	0.212725	0.219144	0.174168	0.175581	0.140359
PTPRJ	2.717016	2.059018	2.550501	2.540079	3.08575	1.814992	3.008478	4.162109
PTPRK	10.44261	9.58831	9.040824	10.66919	9.688454	10.29326	10.10663	10.70242
PTPRM	10.0308	11.41226	10.43506	12.6522	13.1469	12.05403	11.87985	11.59913
PTPRN	0.632345	0.816821	0.757165	0.729009	0.687764	0.604832	0.425212	0.296623
PTPRN2	0.0237	0.042019	0.070213	0.060716	0.088885	0.047723	0.09622	0.07812
PTPRO	0.057567	0.163304	0.210345	0.17108	0.178481	0.110125	0.140234	0.251063
PTPRQ	0.013796	0.02446	0.034061	0.045948	0.020697	0.055562	0.070016	0.122436
PTPRR	0.971905	1.243782	0.875603	0.86493	1.560365	1.708162	1.073017	0.657161
PTPRS	15.44491	12.83533	11.03226	13.73084	14.54293	11.53716	12.64797	17.16068
PTPRT	0.20879	0.194379	0.128171	0.046262	0.062081	0.170446	0.12028	0.131636
PTPRU	1.57116	1.275498	0.818751	1.201003	1.008334	1.169674	0.893463	1.557192
PTPRZ1	0.597955	0.560015	0.46691	0.584266	0.580648	0.682545	0.331691	0.550052
PTRH1	3.853581	3.639964	3.061619	4.572567	3.419408	3.588184	3.676113	3.35009
PTRH2	11.46979	13.10635	10.5195	12.14816	12.40917	11.79024	11.27574	11.65745
PTRHD1	10.92444	14.65531	11.68114	12.62337	15.55992	13.80573	15.37068	13.10491
PTS	12.43136	20.70175	20.54653	15.33291	17.68161	20.12348	17.22674	13.06132
PTTG1	16.1685	19.99408	15.83286	18.72714	16.884	15.08197	19.03127	17.12214
PTTG1IP	55.81424	58.49417	56.5309	60.46379	54.41478	54.41361	59.11404	59.64013
PTX3	1.180509	3.209783	1.016338	1.313148	3.794263	2.366989	1.536175	1.432684
PTX4	0.039786	0.141077	0.196449	0.061157	0.019895	0.080115	0.201912	0.221936
PUDP	3.673397	4.366294	3.713318	3.843431	3.760656	3.941175	4.384166	4.732898
PUF60	54.67586	52.29106	49.32856	53.51694	49.31996	50.68282	56.08668	51.84252

PUM1	22.64705	23.08062	19.36925	21.79698	22.24098	21.42584	22.30897	23.38421
PUM2	36.93995	37.04525	35.06023	35.97137	36.49604	34.947	36.17218	35.67226
PUM3	14.02356	16.62642	11.9489	12.33999	14.88312	16.30144	14.55153	13.6237
PURA	9.505281	10.41771	7.108243	9.0725	8.653683	8.964187	8.425967	7.809136
PURB	20.48004	19.87476	19.73005	19.0491	20.21056	19.79141	20.02607	21.09803
PURG	2.157859	0.670215	1.368799	1.129866	1.118451	1.046654	1.310939	1.150201
PUS1	9.496572	10.46641	7.208752	9.760303	9.275304	7.923543	9.554344	10.36507
PUS10	5.127626	4.912204	4.478133	4.639811	5.756243	5.050271	3.908716	4.910518
PUS3	13.24497	14.01265	12.23392	13.37668	14.5319	12.75664	12.32239	13.22773
PUS7	9.084513	10.29921	9.548984	11.34402	9.209785	9.913995	10.37225	10.31051
PUS7L	1.961433	2.155029	1.530594	1.823804	1.865473	1.808021	1.665374	1.821317
PUSL1	3.421135	4.527507	4.202019	4.316612	4.490821	5.166761	4.036726	3.860194
PVALB	0	0	0	0	0	0	0	0
PVR	1.658868	1.543444	1.49611	2.463205	3.199659	2.019579	2.955155	1.871333
PVRIG	0.717671	0.786043	0.153239	0.19877	0.329783	0.312464	1.870309	0.37378
PWP1	29.41536	32.85681	29.91444	29.74816	29.80548	30.85781	36.73266	28.65883
PWP2	9.329721	8.982709	6.97328	9.078883	8.985081	7.924207	8.311406	9.378362
PWWP2A	3.346216	3.58628	3.136157	3.73411	3.49404	3.403989	2.903666	3.470052
PWWP2B	4.23092	3.948477	3.189897	4.360735	4.361666	4.005873	3.253822	4.464447
PWWP3A	14.94298	14.53759	13.49279	15.13138	14.85237	13.21295	14.34537	15.50761
PWWP3B	0.197066	0.428665	0.480802	0.386076	0.411573	0.449345	0.635364	0.241021
PXDC1	7.900507	8.232071	8.648909	12.43243	11.62734	8.308327	8.361464	11.32082
PXDN	8.694011	6.440534	4.730525	6.88159	7.195202	5.837106	7.315818	9.966539
PXK	18.78986	18.74642	17.97145	19.33353	18.01922	20.82062	18.045	17.75072
PXMP2	2.522743	2.582755	2.963346	3.020639	2.788655	3.395548	2.884026	2.720255
PXMP4	5.094388	4.594521	3.728793	4.759938	5.025114	3.872036	4.734834	3.991691
PXN	16.57166	15.86765	15.76636	17.5126	15.07986	14.74165	14.72615	18.84757
PXT1	0.132776	0.026904	0.078673	0.027213	0.026559	0.026737	0.107814	0.026933
PXYLP1	3.387481	3.612136	2.682605	2.949439	2.547004	2.930368	2.502815	2.837121
PYCR1	14.89768	12.85192	17.50514	16.05587	18.63852	18.53786	13.61472	12.19858
PYCR2	6.623262	8.328123	7.42058	7.149083	6.674555	7.98867	7.968171	7.416582
PYCR3	9.342918	10.60862	6.841687	9.838045	10.49234	9.885053	10.34698	8.331582
PYGB	13.75298	14.45315	10.75517	13.87271	13.9555	13.67478	14.74401	14.63489
PYGL	51.09835	59.94631	63.62708	60.45423	66.58675	57.22414	67.30762	80.78722
PYGM	0.292774	0.840412	0.375863	0.990085	1.825192	0.756586	1.049984	1.207562
PYGO1	4.45629	4.312056	3.985471	4.163528	4.100724	3.851625	4.168581	5.045998
PYM1	3.195168	4.103726	3.627416	3.184076	3.648498	3.672948	3.792123	3.495822
PYROXD1	4.752581	5.960992	5.244154	5.377459	5.271725	5.655057	5.61323	4.724629
PYROXD2	0.414079	0.694819	0.523928	0.755849	1.345931	0.312681	0.748642	0.577464
PYURF	0	0.022942	0	0	0	0	0	0
PYY	0	0.044797	0	0.045312	0.044222	0	0.08976	0.044846
QARS	69.9558	75.45162	71.25886	68.01708	65.13771	65.18363	74.52586	73.0463
QDPR	24.24741	24.97256	23.29811	31.32541	22.79171	23.96609	29.63205	27.72294
QKI	17.90947	16.51342	15.93232	16.3627	17.01799	16.22774	16.58958	18.38305
QPCT	12.57708	9.713274	17.7652	10.48521	9.76912	9.030175	16.97739	11.65826
QPCTL	5.540813	5.889812	6.242964	6.008329	6.582891	6.764288	5.787485	4.312201
QPRT	0.924373	1.306751	0.297209	0.749004	0.644995	0.49781	1.134615	0.261637
QRFPR	0.027703	0.130977	0.164146	0.085167	0.203181	0.065082	0.074983	0.112389
QRICH1	29.8137	28.47448	25.46292	27.46515	26.78921	26.86711	28.26715	29.3798
QRICH2	0.202877	0.144145	0.085864	0.070201	0.063243	0.092847	0.128366	0.125597
QRSL1	6.043555	6.232627	6.579596	5.733321	5.680592	7.370371	6.055665	5.438931
QSER1	12.76944	11.71709	11.75029	10.19885	12.28658	10.44887	11.73879	12.80922
QSOX1	22.94992	20.62054	20.6059	20.56	23.22624	20.59567	19.36702	21.63938
QTRT1	4.521131	4.496952	3.857385	4.730592	3.931927	3.87528	4.144857	3.672253
QTRT2	4.13738	3.880544	3.588789	4.388478	3.992875	4.088344	3.991634	4.170322
R3HCC1L	4.845728	3.909134	3.631805	3.97724	4.183089	3.67999	3.434475	4.028075
R3HDM1	5.861605	5.386249	5.128672	5.502228	6.120307	5.346894	5.741296	5.778587
R3HDM2	24.07199	23.26517	22.06317	21.39495	21.7636	20.94882	21.42555	22.80986

R3HDM4	25.7899	23.57965	25.18731	26.75301	25.52423	22.79599	26.17675	27.20011
R3HDML	0	0	0	0	0	0	0	0
RAB10	114.4103	114.5078	131.8304	122.4825	121.5823	137.2192	119.7237	127.3064
RAB11A	108.3689	118.4342	118.7326	115.5682	119.4631	124.4525	115.9372	111.6184
RAB11B	52.15494	53.46846	48.98792	48.50736	49.35097	50.55358	51.69344	49.58303
RAB11FIP1	16.9151	18.6197	18.52218	18.23646	16.28823	15.81901	20.26296	18.23611
RAB11FIP2	7.160344	8.179395	8.56632	7.944012	7.669128	7.908141	6.619941	8.868765
RAB11FIP3	10.6506	10.40077	9.70882	10.46893	10.98641	10.01632	9.036914	11.31943
RAB11FIP4	6.736074	6.115002	4.862869	8.474883	6.970403	6.423961	4.197041	4.351714
RAB11FIP5	14.47082	13.78883	12.36633	13.63024	14.20674	12.58843	11.64529	13.81867
RAB12	38.32084	43.06269	42.12441	44.91987	44.54977	44.96998	43.1271	41.27157
RAB13	29.80455	30.63805	33.44989	30.51927	37.94424	32.66887	26.02992	29.43641
RAB14	63.50849	65.13009	67.23427	64.86638	66.06079	64.70991	63.98737	63.67093
RAB15	15.75602	11.63136	12.1539	12.46022	11.36815	14.38863	11.17845	11.52849
RAB17	0.182178	0.100674	0.065421	0.152747	0.082819	0.066699	0.201721	0.184771
RAB18	82.01097	81.11425	92.18839	84.33016	82.81878	90.39607	81.96319	82.52618
RAB19	0.469021	0.616446	0.425622	0.623532	0.456404	0.548803	0.617587	0.15428
RAB1A	188.0508	196.8356	194.7413	186.1795	194.584	192.7263	186.0674	183.6655
RAB1B	67.77541	67.84467	65.78876	67.32083	74.53657	64.20642	68.38117	63.71083
RAB20	2.655332	1.095999	2.330885	2.176884	2.380272	2.574454	2.675198	2.573437
RAB21	53.2589	49.76162	47.43079	49.56193	53.01473	47.80975	49.00447	48.33107
RAB22A	17.93067	17.22706	16.07469	17.31875	18.51274	15.89875	14.94178	16.17535
RAB23	10.49358	10.19867	11.0326	9.022396	9.879436	9.951965	10.2558	11.33084
RAB24	38.58184	34.64103	39.92631	42.07077	40.06561	42.26475	35.72523	37.63145
RAB25	99.97568	92.0821	101.2405	96.54539	92.63799	89.86295	95.1906	94.68493
RAB26	1.134903	1.2741	1.241925	1.71309	1.411143	1.636778	1.790156	0.808852
RAB27A	10.77401	12.80008	12.24721	13.0902	15.30262	15.56905	10.69173	10.9431
RAB27B	11.41353	11.83923	11.9256	11.54213	10.8906	11.33599	12.90493	13.41465
RAB28	16.50974	19.39436	18.53309	17.29319	14.35114	16.7091	16.74647	17.34435
RAB29	2.623073	4.576803	5.132809	4.01216	4.012849	4.430999	4.338758	5.636137
RAB2A	70.54559	74.31046	78.92398	74.56074	72.76789	81.46222	74.0756	69.67765
RAB2B	18.69417	18.64633	19.49004	20.24854	20.62992	18.82188	19.69127	19.44164
RAB30	2.570254	2.461581	2.444036	2.24552	2.329594	1.832192	2.392257	2.767225
RAB31	15.99669	15.41706	14.31781	13.87995	14.20469	12.50056	14.88643	17.41688
RAB32	8.129732	8.985192	8.00489	7.036244	7.892343	5.112793	8.009693	7.278254
RAB33A	0.42365	0.228912	0.334697	0.115772	0.451952	0.113745	0.258003	0.200518
RAB33B	7.694956	7.645379	8.006751	8.723192	8.174883	8.89469	7.642776	7.369055
RAB34	27.77551	27.79369	25.00297	29.07658	27.62952	28.89026	30.51257	24.11863
RAB35	24.16418	24.18401	23.72319	24.27536	22.62902	23.37158	24.06446	22.97898
RAB36	0.014872	0.015067	0.036717	0.00762	0.044622	0.029947	0.03019	0.113128
RAB37	0.38788	0.666341	0.260916	0.455094	0.54535	0.220734	0.490696	0.336387
RAB38	38.0716	35.70149	46.12604	44.37358	37.16522	41.38307	48.13245	44.84157
RAB39A	0.207174	0.125936	0.102296	0.063692	0.2072	0.020859	0.126169	0.126074
RAB39B	0.067261	0.025554	0.066423	0.180935	0.075679	0.160837	0.093872	0.051164
RAB3A	1.641929	1.063852	1.282093	2.465205	1.852183	1.403252	1.78283	1.587847
RAB3B	0.241575	0.822617	0.60304	0.660157	1.691245	0.770214	0.367798	1.177429
RAB3C	0.939714	0.898455	0.620719	0.577157	0.609959	0.382213	0.590604	0.833166
RAB3D	13.21909	15.68108	18.13632	19.62699	16.80044	18.38651	16.51742	14.36724
RAB3GAP1	19.63446	18.81235	21.97433	20.4606	18.60658	17.80723	22.53128	18.10033
RAB3GAP2	19.39168	19.90588	19.06826	19.48403	18.36472	18.44014	19.93474	19.31573
RAB3IL1	4.130305	5.142975	4.87638	5.320325	6.646267	4.530233	5.023686	5.66348
RAB3IP	26.43481	33.30883	44.20773	42.55683	35.65837	38.64953	29.70378	28.75727
RAB40B	2.08784	2.283847	2.300878	2.232581	2.133504	2.589548	1.95024	1.872048
RAB40C	13.00293	11.17553	10.94833	11.01854	13.50607	11.47632	11.36586	12.00145
RAB42	0.088387	0.089547	0.174571	0.15096	0.103131	0.103822	0.044856	0.089645
RAB43	5.33294	5.491794	4.850715	6.561642	5.438899	4.76885	4.860957	5.133077
RAB44	0.794547	0.987352	0.766257	0.814228	0.546322	0.581232	0.422134	1.202491
RAB4A	3.32853	3.062125	3.08735	3.12533	3.137641	3.131153	3.151008	3.381457

RAB4B	5.333285	6.443196	5.141431	6.15941	5.016476	5.966355	6.294017	6.310742
RAB5A	55.83821	55.54182	58.05986	58.96837	52.93754	59.20582	58.13357	50.88926
RAB5B	51.80877	54.14202	51.43675	53.00004	52.69142	53.81328	54.98688	51.35431
RAB5C	71.13213	73.8188	69.5324	79.78064	78.83682	76.87863	74.8422	71.83803
RAB5IF	52.08889	57.46118	63.13294	61.45162	59.55545	64.04405	60.59088	52.3061
RAB6A	20.93615	19.14628	19.59828	18.15088	20.42136	23.36342	20.0786	17.64941
RAB6B	0.041994	0.037227	0.072574	0.053793	0.063	0.052851	0.042624	0.05324
RAB7A	57.4304	57.16783	55.60642	57.68123	55.76106	59.03753	55.58624	55.83608
RAB7B	8.152357	7.211116	8.091883	7.385415	7.838217	7.86081	7.411569	7.761813
RAB8A	93.52149	93.11291	89.03403	92.20677	91.20084	88.24431	95.83485	98.58325
RAB8B	10.49737	13.78909	11.34038	11.24503	13.55488	11.85053	12.25509	13.54837
RAB9A	25.46637	22.25854	26.44378	24.91649	24.45643	25.34034	26.73551	21.03383
RAB9B	0.07062	0.166943	0.116234	0.120616	0.047086	0.047402	0.07168	0.119376
RABAC1	25.63921	28.40662	28.59311	26.04616	27.31245	28.82791	31.02017	29.86783
RABEP1	14.8375	14.20477	14.39411	13.04821	14.81848	13.34166	14.31078	15.13369
RABEP2	3.566958	3.976934	3.046655	4.600757	4.061099	3.319166	4.586715	3.969378
RABEPK	5.475308	7.079426	4.858814	7.043095	5.533457	6.476474	6.043228	4.09698
RABGAP1	31.26354	28.91885	28.33807	29.14787	29.86009	29.80052	30.58293	29.08138
RABGAP1L	2.947455	3.073866	2.802193	2.723537	2.877887	2.568559	2.981525	3.24951
RABGEF1	20.61535	17.19216	20.16705	18.83385	16.97165	19.8854	18.04131	18.89734
RABGGTA	6.738381	7.126596	5.804354	7.635793	7.344574	7.150042	7.344567	6.861589
RABGGTB	30.83846	39.50452	35.91078	35.36106	31.40922	32.91937	32.38754	34.34294
RABIF	18.76245	17.13018	14.09269	18.79742	19.29471	17.23492	18.94307	19.33178
RABL2B	15.10038	14.18567	15.91023	15.71457	17.59253	16.07358	15.32689	16.78597
RABL3	12.45215	16.79698	15.95549	16.16727	17.5536	16.53665	16.48494	14.32953
RABL6	20.94352	21.73158	20.64618	22.90633	23.10164	23.77574	22.00553	22.23182
RAC1	273.3471	260.9055	261.627	268.9185	260.1645	267.1858	294.7636	275.3695
RAC2	5.842311	8.058926	5.157059	6.033081	7.586997	3.275946	6.340481	10.03685
RAC3	10.0822	9.472769	7.154876	9.550401	8.588521	7.862862	10.31084	7.11624
RACGAP1	13.90944	14.8093	12.6475	12.75695	10.54099	11.57714	12.91732	13.14393
RACK1	2226.568	2465.622	2381.256	2195.121	2108.682	2037.539	2358.495	2200.997
RAD1	5.83769	5.673086	5.059572	4.708117	5.494488	5.975234	5.424025	4.471898
RAD17	7.440828	8.844078	7.443997	7.751773	8.06856	8.069309	8.788783	8.352456
RAD18	3.769508	3.784677	4.335533	3.284605	4.153768	4.079336	3.871872	4.590093
RAD21	67.16155	72.11642	73.09081	68.13181	62.68237	65.58455	69.29776	72.23499
RAD21L1	0	0.014045	0	0	0	0	0	0
RAD23A	35.01255	35.10948	32.05817	33.00699	32.57125	30.89782	33.26744	31.79049
RAD23B	89.66065	85.39141	91.48969	89.79213	91.27889	92.32605	89.08834	90.54313
RAD50	6.455534	8.076425	6.786393	7.71579	6.501927	6.113064	8.256511	7.101852
RAD51	7.554782	7.506131	8.458831	7.154599	7.08678	7.207713	8.323859	7.937116
RAD51AP1	2.354107	2.661715	2.363307	2.25248	1.782068	2.0821	2.138596	2.202942
RAD51AP2	0	0	0	0	0	0.007806	0	0
RAD51B	3.231149	2.790571	2.479477	2.659805	2.521681	3.274555	2.623693	1.719159
RAD51C	8.943283	9.270392	7.666535	6.905431	8.664926	8.066423	8.278955	9.847477
RAD51D	3.910496	4.939627	5.357179	4.621253	5.109265	4.289046	4.340733	4.759397
RAD52	4.60885	4.843047	5.398042	5.756172	6.207662	5.841852	5.186157	5.877856
RAD54B	2.308762	1.967783	2.108091	1.708743	2.079987	2.204618	1.813341	2.313753
RAD54L	2.603258	2.494632	1.817587	2.608269	2.247051	2.036733	2.044842	2.337605
RAD54L2	7.905135	7.032655	5.989316	6.610094	6.45871	5.87424	6.535566	7.30865
RAD9A	3.991257	4.030883	2.586238	3.419187	3.765111	4.018524	3.220454	3.307418
RAD9B	0.299632	0.535702	0.713638	0.487674	0.423065	0.479137	0.393576	0.178763
RADIL	1.616588	1.332723	1.252114	1.948978	1.878338	1.627632	1.640837	1.470819
RADX	0	0.01201	0	0	0	0	0	0
RAE1	22.82193	23.4398	23.70984	21.89647	22.16144	22.38026	24.03839	21.1048
RAF1	31.44877	30.64449	29.37239	29.5852	30.23541	29.30114	29.51447	31.72589
RAG1	0	0	0.044126	0	0	0.008998	0.009071	0.018127
RAG2	0.01767	0.089509	0.069799	0.036215	0.106033	0.142325	0.053805	0.053764
RAI1	3.616541	3.09068	2.579044	3.349509	3.636525	3.015817	2.815812	3.816461

RAI14	54.05499	42.5375	42.78157	45.61724	48.99963	47.29278	44.49103	43.67834
RAI2	1.192214	1.072399	1.265385	1.107563	1.236942	1.200358	1.029148	1.163982
RALA	27.7364	29.79451	29.79097	28.46329	28.32337	27.60287	28.50768	27.47061
RALB	9.922447	10.38179	11.55483	11.01509	12.67114	10.19107	10.18698	11.59551
RALBP1	35.55866	34.61745	32.20141	32.98819	34.5168	33.78517	35.1047	34.18286
RALGAPA1	14.25944	14.15329	13.12068	12.95887	14.57855	13.49793	14.31704	15.95866
RALGAPA2	1.39497	1.275637	1.066134	1.362398	1.273833	0.930634	1.438056	1.471421
RALGAPB	19.65001	17.62404	16.93524	17.48939	18.65856	18.10104	18.10093	19.30224
RALGDS	8.741692	7.037262	6.198484	8.223813	8.254484	7.92127	7.985535	8.139272
RALGPS1	2.069305	2.639025	2.233911	2.792293	2.489485	2.26461	2.204709	2.324716
RALGPS2	7.662236	7.244838	6.793471	6.664943	6.530571	6.962018	6.647378	6.931171
RALY	59.51694	59.43417	56.75442	56.40375	57.88302	58.50701	64.25021	58.14811
RALYL	0.017726	0.044896	0	0	0.017728	0.008923	0.017992	0
RAMAC	20.03285	20.72759	20.78877	20.45628	19.44338	25.34326	23.89048	22.33543
RAMP1	10.77026	13.2718	10.20261	13.56276	8.644333	10.80984	10.48631	10.44417
RAMP2	76.6038	67.07053	75.261	109.4549	104.1775	82.34571	85.40842	92.08752
RAMP3	4.289892	2.684981	3.784553	3.907693	5.739665	5.221432	3.657562	2.18515
RAN	217.8335	235.8694	218.3647	220.3667	217.3138	230.3787	235.1109	224.0732
RANBP1	85.66381	97.85139	90.77364	95.98848	90.95004	104.5022	100.2655	90.99626
RANBP10	25.30557	21.65226	26.37387	23.64619	24.48964	24.30652	23.645	21.72521
RANBP17	1.428455	1.979079	1.308172	1.645254	1.685062	1.235388	1.487058	1.207325
RANBP3	26.69442	26.27884	24.31945	26.42064	26.06344	24.40958	26.45098	27.68255
RANBP6	8.898994	9.68888	10.08827	8.869578	9.710911	9.739167	9.91098	9.2235
RANBP9	18.1477	19.78841	15.98482	16.71477	16.71224	16.49365	17.51918	18.00102
RANGAP1	26.1603	20.6585	20.76057	22.0939	22.49605	21.91596	22.46694	21.28354
RANGRF	23.69574	23.7743	22.33242	21.49506	23.89548	24.91356	23.55219	19.64529
RAP1A	19.16734	22.2801	23.14549	19.48299	24.00352	22.63029	21.59242	21.69427
RAP1B	22.33399	28.18621	29.69075	23.97129	26.47004	27.07546	27.9775	28.16701
RAP1GAP	1.773764	1.598944	1.696493	2.125422	2.116221	1.743696	2.863582	2.110656
RAP1GAP2	7.937831	7.101445	6.293559	6.397885	5.299329	6.832843	6.991149	7.150355
RAP1GDS1	13.45293	12.32959	11.29744	9.881003	12.93216	12.37678	11.89232	13.77691
RAP2A	4.575509	5.194836	4.853978	4.683407	5.706854	4.339553	5.290658	5.798114
RAP2B	27.4786	27.6005	26.61909	24.26875	28.52959	24.23934	26.86763	26.13041
RAP2C	8.225997	8.268177	7.674687	8.562824	9.233527	8.792067	8.362566	8.297005
RAPGEF1	7.04294	8.126395	6.212439	7.707466	7.530832	6.215615	8.181157	8.629163
RAPGEF2	10.26802	9.543986	7.853826	9.452921	10.00542	10.03221	9.241043	10.59669
RAPGEF3	10.57676	9.163608	7.708301	12.70043	11.67906	12.44711	10.32131	10.67268
RAPGEF4	2.268996	1.583188	1.608262	1.477627	1.650725	2.063416	1.630695	1.221172
RAPGEF5	4.662122	4.273865	4.011795	4.129366	5.472026	3.986774	3.823166	4.353544
RAPGEF6	5.182029	5.158584	4.379231	4.697542	5.213733	4.4591	5.039291	5.438908
RAPGEFL1	15.48243	13.76782	12.18724	14.05063	12.79933	13.27073	13.09805	12.28885
RAPH1	4.458402	3.804609	3.273207	3.207907	3.850682	2.797812	3.862627	4.850116
RAPSN	0.012478	0.063211	0.012323	0.038363	0.04992	0.087946	0.113991	0.025312
RARA	16.11705	13.67882	12.69106	14.08865	16.619	13.21472	12.5436	15.98539
RARB	1.438695	1.54276	2.121928	2.240219	1.756556	1.83417	5.632492	4.633358
RARG	27.18454	24.99903	23.36214	24.28483	24.38286	24.26707	22.0277	24.46397
RARRES1	2.47904	2.241513	3.211553	4.479928	3.439113	2.25443	3.219687	3.676871
RARRES2	134.4343	96.72601	97.01735	115.2764	93.27541	138.1145	116.3686	101.2629
RARS	41.26507	41.74044	42.9833	41.55074	45.30858	48.6118	43.0777	39.16218
RARS2	9.544692	10.95295	9.228911	11.04281	9.757015	11.90022	10.67569	9.954096
RASA1	22.14185	21.17522	20.05662	20.89932	21.83931	20.59234	20.67368	21.36978
RASA2	6.659865	5.669302	5.82774	5.401309	5.813355	5.316677	4.809831	6.195113
RASA3	8.28511	8.612015	6.329482	9.936363	10.06584	8.587055	7.454559	8.908846
RASAL1	1.881416	1.561832	1.047667	1.58828	1.276544	1.268409	1.800274	1.429047
RASAL2	6.608039	6.102283	5.392598	6.048528	6.083242	5.900413	5.798011	6.586986
RASAL3	1.032594	1.573471	0.70469	1.204423	1.024331	1.107267	1.20146	1.123925
RASD1	3.907031	4.606651	3.692041	3.278982	4.749678	3.730254	3.965637	4.714188
RASD2	1.839663	1.626077	1.501592	1.962181	2.130906	2.088485	1.571927	1.618341

RASGEF1A	0.734522	0.853828	0.526848	0.768563	0.734617	0.568278	0.667062	0.494038
RASGEF1B	24.80664	24.85391	22.63614	22.00445	21.34272	21.09475	22.01577	22.59481
RASGEF1C	0.119527	0.272465	0.275421	0.234768	0.338703	0.330944	0.181979	0.222252
RASGRF1	0.106161	0.037961	0.080171	0.076794	0.068702	0.125749	0.0824	0.076004
RASGRF2	1.456099	0.960537	0.952378	1.091287	1.312289	0.913544	1.036765	1.366617
RASGRP1	5.160014	6.670452	5.294253	6.523945	6.25537	4.441839	5.441469	5.341084
RASGRP2	1.011183	1.183593	0.659259	1.167017	1.423695	1.077398	0.876883	1.085321
RASGRP3	2.283292	2.133587	1.895818	1.635623	2.780211	2.642607	1.926034	1.996531
RASGRP4	0.88966	0.711581	0.705172	0.875709	1.112218	0.707161	0.879241	1.519705
RASIP1	3.366394	2.890868	2.738712	3.917966	4.160439	2.993963	3.180962	3.967111
RASL10A	0.023122	0.035138	0.034251	0.082932	0.0925	0.03492	0.023469	0.10553
RASL10B	0.585236	0.715265	0.486207	0.809163	0.984811	0.579882	0.584586	0.65952
RASL11A	2.395352	3.086242	1.851265	2.16119	2.682099	2.280644	2.114158	2.561487
RASL11B	2.447612	2.012961	2.630389	2.419714	2.966313	2.000459	1.724415	3.110371
RASL12	23.66754	12.92961	14.47881	11.6895	9.209978	10.78654	17.69741	14.56718
RASSF1	15.50787	14.19181	14.23018	17.15458	16.3135	13.37556	14.70744	12.30582
RASSF10	1.765983	2.014211	2.215621	2.59508	1.799536	2.767715	1.955937	1.78549
RASSF2	4.907751	6.145057	4.057514	5.959812	5.01736	4.72187	5.373057	6.920775
RASSF3	25.89247	28.23848	26.62978	29.96141	23.50761	28.15524	26.12885	26.47204
RASSF4	5.204235	5.593761	5.193375	6.946535	4.844064	5.492971	6.181177	5.710788
RASSF5	3.66233	4.164439	3.713511	4.097495	4.279107	2.749649	3.688835	4.225826
RASSF6	2.722382	1.63405	2.181204	2.158159	2.095991	3.206429	2.002029	1.958844
RASSF7	7.18306	5.169414	6.294513	6.684114	5.565527	7.149003	5.648279	5.727765
RASSF8	6.135168	5.90696	6.02863	6.656664	5.572143	5.415172	6.94889	7.422708
RASSF9	7.301193	5.362261	4.26499	4.534104	5.791871	6.119558	6.000607	6.103297
RAVER1	11.08168	12.44606	10.41987	12.65403	11.62935	10.38434	9.825851	11.67296
RAVER2	2.44884	2.88779	2.770185	2.764505	2.658438	2.847078	3.369587	2.833598
RAX	0	0	0.026249	0	0.026584	0.026762	0	0
RAX2	0.100862	0.051093	0.049803	0	0	0.050775	0	0
RB1	8.213284	8.499383	8.284751	7.564126	8.315691	7.394126	8.31483	9.504007
RB1CC1	36.96373	35.23175	40.15304	38.70389	36.50867	39.21038	35.06813	34.33775
RBAK	2.652	2.810446	2.68385	3.131358	2.995035	3.03401	3.144381	2.808769
RBBP4	75.76902	79.67177	71.41341	70.25397	73.95399	67.65249	82.86885	79.94424
RBBP5	18.94603	18.80638	18.67927	18.95888	19.798	17.92822	19.42999	20.36091
RBBP6	20.17212	21.85771	22.553	22.93781	24.11563	23.75575	19.73061	20.00266
RBBP7	72.34827	78.82119	75.43177	78.87678	74.31826	73.73576	79.38835	74.36508
RBBP8	13.81201	15.1433	13.56152	15.39873	13.09929	14.26601	13.49548	14.37898
RBBP8NL	3.281308	3.297883	2.6982	3.161636	2.771822	3.290562	2.614	2.691585
RBBP9	12.28143	21.15596	17.15809	20.66371	11.73891	24.12464	22.75775	21.70748
RBCK1	10.41022	10.68368	10.49597	11.61565	11.20127	9.874637	10.38711	10.03155
RBFA	11.9068	10.18775	10.57665	11.54886	11.25394	12.39555	11.43001	11.4738
RBFOX1	0.003509	0	0.005198	0	0.001755	0	0	0
RBFOX2	8.633474	8.358817	7.196592	7.736655	7.998601	8.204087	8.390785	8.094941
RBFOX3	0.743013	0.724358	0.761444	0.897898	1.156727	1.044502	0.782617	0.796246
RBKS	5.801551	6.311414	7.201669	4.538365	6.998196	5.707417	5.693787	5.644582
RBL1	5.917864	6.347238	5.522726	6.108921	5.045211	5.477697	6.259891	6.662286
RBL2	13.34644	15.43375	14.65997	14.78757	14.60831	13.8135	14.05188	14.36735
RBM10	29.74637	30.05693	27.15017	29.53034	31.33426	29.14035	28.15307	29.90609
RBM11	1.328559	2.213127	1.829238	2.290932	1.584256	1.839248	2.100529	2.021208
RBM12B	12.28519	12.47487	10.39202	11.23702	11.43723	10.0932	11.85668	12.30343
RBM15	2.960819	2.561522	2.157354	2.710288	2.778206	2.059936	2.566265	3.087321
RBM15B	10.86702	10.89007	8.965428	11.08038	11.39505	10.66704	10.37578	11.16905
RBM17	26.05851	28.08633	23.37427	24.56155	25.50714	25.80695	26.39525	25.79118
RBM18	13.03	14.30381	13.49559	13.39693	15.5216	15.21328	14.12246	14.03531
RBM19	7.901891	7.612535	6.810411	7.002254	7.166469	6.584721	7.795398	7.363914
RBM20	0.204057	0.23205	0.22619	0.179239	0.233238	0.335431	0.202891	0.173172
RBM22	21.01934	23.41041	22.33866	21.1861	22.0661	23.21759	21.52583	24.24808
RBM23	9.816802	9.145814	7.723627	8.939402	9.509111	7.968312	9.068185	9.300075

RBM24	0.118059	0.119609	0.089683	0.093064	0.217984	0.073148	0.138266	0.147372
RBM25	12.97726	12.78801	11.39221	13.25429	12.38012	12.16576	12.21771	13.31588
RBM26	7.610433	8.283717	6.724674	7.674922	7.80366	8.060239	7.132058	8.455269
RBM27	9.204665	8.437327	8.3123	8.073724	9.562498	8.811275	7.540545	8.544538
RBM28	7.423135	8.68921	7.200791	7.368141	8.727197	8.183922	8.838761	7.874333
RBM3	177.8828	202.0096	133.7856	160.3357	91.39654	156.1891	240.6019	154.3507
RBM33	8.659358	7.504124	6.255112	7.838801	7.142986	6.408945	8.360949	8.855477
RBM34	6.66645	7.776249	7.465526	7.01805	7.064372	8.144328	6.581755	7.214345
RBM38	4.253	4.242933	4.110099	4.265051	4.201519	3.928491	4.528015	3.917807
RBM39	71.77194	72.27802	64.75607	73.62991	73.66522	68.84306	70.57923	71.33924
RBM4	31.91217	30.61473	29.03564	27.4244	31.4773	29.23688	29.67596	32.38038
RBM41	1.874872	1.665812	1.822122	1.959435	2.567121	1.947607	1.910547	2.490141
RBM42	39.23824	43.35483	44.35322	42.72439	42.6819	41.38835	46.21333	38.8658
RBM43	3.485328	5.658963	5.185396	4.92858	4.870957	4.826981	4.72711	3.998037
RBM44	0	0	0	0.009097	0	0	0	0.009003
RBM45	9.816246	11.4639	10.2998	11.45372	10.51028	11.86096	11.66592	11.43634
RBM46	0	0.092366	0.030011	0.077857	0.060788	0.015299	0.030846	0.092468
RBM47	2.049426	2.721128	2.912072	3.326562	2.710802	2.706701	2.571526	2.322849
RBM48	7.683818	8.359815	8.552883	7.33297	8.173364	8.374356	7.490864	7.873544
RBM4B	10.63259	10.49769	8.379068	10.25153	11.13712	8.834981	8.926299	9.547578
RBM5	63.44747	59.78517	50.01599	52.90994	61.26385	52.95641	56.80893	64.79524
RBM6	16.64335	16.25189	15.18404	17.34592	17.50966	15.85146	15.5847	18.52521
RBM7	18.52724	21.75529	19.42942	20.66996	21.39125	19.52375	19.75403	17.59859
RBM8A	39.75803	46.27236	44.36116	39.07647	39.33626	42.87428	42.08741	40.15909
RBMS1	26.73786	25.40842	27.69287	28.07761	26.36941	27.56827	24.61075	26.29236
RBMS2	13.72428	12.5776	11.46671	10.95489	11.82231	10.76696	11.12866	14.15905
RBMS3	2.798916	2.545152	2.693251	2.894959	3.248988	2.430926	2.609395	2.994083
RBMX	57.13564	64.36732	52.39722	57.76026	55.62349	58.79874	60.589	64.42591
RBMX2	11.35431	12.90393	9.601407	14.56187	11.53711	11.72851	9.362319	12.4124
RBMXL2	0.01933	0.117499	0.095443	0.079233	0.019332	0.214077	0.117717	0.058814
RBP1	59.62275	31.1626	46.53523	61.62249	42.39011	50.52653	61.99673	53.30069
RBP2	0	0.036589	0	0.03701	0	0	0	0
RBP4	44.52502	69.21832	34.18622	51.10039	53.12772	48.39006	46.97589	39.93269
RBP5	0	0	0	0	0	0	0	0
RBP7	0.043461	0.132095	0.04292	0.089076	0.086934	0.350067	0.044113	0.13224
RBPJ	21.29673	19.54657	17.54765	18.52161	21.51727	16.53112	21.85195	23.27843
RBPJL	0.878923	1.059338	0.583636	1.133633	0.621388	0.808641	0.738296	0.660892
RBPMS	8.31678	11.76794	10.36516	10.83787	9.877453	10.66828	11.60711	10.3412
RBPMS2	2.694858	3.865566	3.39906	4.949024	6.15094	3.277412	3.628986	3.612722
RBSN	15.43513	15.1495	13.5948	14.66351	14.07249	12.98708	14.96647	15.70116
RBX1	65.79944	71.00224	73.49793	70.79772	72.26322	76.37784	72.20537	69.12029
RC3H1	11.26908	9.629934	10.67723	9.903885	10.42542	10.02108	9.06426	11.03611
RC3H2	11.27291	9.861677	9.172076	9.514797	10.03464	9.942113	10.31148	12.25635
RCAN1	21.63481	18.46646	18.38109	20.90789	24.10251	23.07727	25.3745	17.97592
RCAN2	2.138283	3.354602	2.923202	2.722708	3.95793	3.639028	2.883012	2.435767
RCAN3	6.372973	5.576989	5.453833	4.467017	5.406985	4.785346	4.551618	5.074728
RCBTB1	8.866498	11.60038	11.32677	11.51979	11.21669	13.53183	10.78085	8.469962
RCBTB2	10.11088	10.07932	9.024253	8.971767	9.787892	9.104086	8.310269	9.701716
RCC1	9.444677	9.068361	7.58544	9.54208	7.517524	8.854209	9.522704	8.680837
RCC1L	9.004061	10.12541	8.474656	10.45828	9.771066	9.903009	9.232926	8.944775
RCC2	55.84627	48.89358	52.49143	52.87648	50.87129	53.57595	50.77876	52.8017
RCCD1	1.894438	2.328907	1.540015	2.142603	1.698283	2.105096	1.700085	1.745668
RCE1	18.58808	19.89687	16.37666	17.37304	18.29848	17.14731	19.7164	16.85884
RCHY1	30.09013	30.73757	28.51956	37.40398	34.08282	31.69396	31.58965	25.64275
RCL1	6.849211	7.733589	7.011026	6.617058	7.584334	6.41723	7.299124	7.403624
RCN1	30.423	44.27915	46.93245	38.72531	45.21313	42.53125	46.06936	50.97164
RCN2	24.68557	23.54258	20.94896	20.57185	22.3408	23.01421	24.29961	24.89441
RCN3	12.46142	14.61172	11.1794	17.19121	16.77784	15.86717	15.90542	14.70008

RCOR1	10.24962	8.454847	7.255797	8.004166	8.611205	7.730763	7.995038	9.156127
RCOR2	0.581131	0.456588	0.327937	0.559065	0.69982	0.71645	0.68615	0.517232
RCOR3	7.270438	7.167702	7.22647	7.955065	6.536146	6.980584	7.12621	7.832244
RCSD1	3.117848	4.09101	2.02914	3.167614	3.725819	2.068779	2.575218	5.327773
RCVRN	0.059111	0	0	0	0	0	0	0.029976
RD3	0	0	0	0	0	0.047926	0	0
RD3L	0	0	0	0	0	0	0	0
RDH10	10.96962	12.05656	11.31441	13.67129	14.72779	15.49586	10.2007	11.09207
RDH11	14.69352	17.13316	18.4131	19.99048	18.86859	16.60346	17.16488	13.50623
RDH12	31.26221	30.63431	33.45547	35.59447	40.03121	36.51129	27.50861	24.36991
RDH13	5.169347	0.696964	3.37741	2.840048	2.516205	1.345692	2.85287	3.767737
RDH5	1.490439	1.741595	0.731419	1.789726	1.883866	1.555859	1.614886	1.632218
RDH8	0	0	0	0.02062	0	0	0.020423	0
RDM1	6.012836	5.542297	4.238042	4.373653	2.924267	4.392055	4.188353	4.998324
RDX	16.01105	16.33236	19.18194	18.14513	19.70086	16.53477	18.10023	19.21631
REC114	0	0	0.140164	0.087269	0.198732	0	0.028812	0.086372
REC8	0.306371	0.214318	0.144073	0.276584	0.167796	0.220331	0.133271	0.177561
RECK	6.78194	6.252236	7.744903	6.383397	7.747199	5.663258	8.142957	7.380517
RECQL	5.097683	7.113485	4.796704	5.482688	5.519196	4.793569	5.515837	7.035932
RECQL4	1.921321	1.397701	1.04142	1.524797	1.329206	1.439926	1.473602	1.260044
RECQL5	2.341157	2.48555	1.85402	2.322498	1.930021	2.289373	2.285171	2.640002
REELD1	0.08888	0.204651	0.111711	0.041401	0.169702	0.195245	0.057408	0.245851
REEP1	0.789473	0.929721	1.51929	1.023386	1.525161	1.49462	1.739606	0.547495
REEP2	0.394633	0.33192	0.198535	0.335735	0.327662	0.262387	0.264516	0.249213
REEP3	38.26987	43.10774	41.16072	38.74388	38.63706	39.1204	40.29746	40.97355
REEP4	30.31601	30.663	28.66536	27.20717	26.00047	28.02868	26.64194	28.40459
REEP5	78.71389	84.22956	89.12387	80.18347	75.8581	76.3846	79.18118	75.86785
REEP6	5.256306	6.271408	6.03399	6.179444	5.897431	5.883224	6.662168	4.898148
REG4	0	0	0.015858	0	0	0	0	0
RELA	23.49907	21.09712	21.75256	22.89638	24.32898	20.51943	22.37107	22.08152
RELB	4.324239	3.926478	2.978175	5.299743	5.321869	3.613512	4.161426	3.501054
RELCH	13.03795	12.51436	12.03734	12.60417	13.31779	12.23869	12.35259	12.9123
RELL1	17.48615	13.17439	14.79871	18.30611	17.31853	18.99277	15.93816	12.92086
RELL2	0.360155	0.273661	0.203238	0.10545	0.360201	0.090654	0.20889	0.313099
RELN	2.180663	4.36758	2.29548	1.556908	2.959853	1.727496	2.583079	3.256798
RELT	3.383205	2.5846	2.103958	2.2395	2.661189	2.227916	2.459453	3.403545
REM1	2.724786	3.389788	3.620756	3.798322	5.310012	3.267874	3.070694	3.027741
REM2	1.910688	1.24967	1.09869	1.734951	1.451343	1.223646	0.902165	1.661923
REN	0	0.039883	0.019438	0.020171	0	0.019817	0.019978	0
RENBP	2.252935	2.282502	1.543373	2.641532	2.415621	1.798312	2.575145	1.852708
REP15	0.012804	0	0	0	0	0	0	0
REPIN1	10.06974	8.665026	9.492476	9.342035	10.40337	10.15793	8.710399	9.690471
REPS1	39.66164	36.64805	37.93423	34.54164	34.64004	37.3764	36.91494	39.13764
REPS2	0.346848	0.672244	0.547298	0.575658	0.407223	0.497256	0.646703	0.466499
RER1	56.88679	56.18706	61.98801	63.15019	60.7664	63.43851	55.76419	51.22493
RERE	12.37236	11.16002	11.00833	10.9197	11.83611	10.34488	11.13008	11.80439
RERG	3.988684	2.94418	1.868204	2.966346	2.815235	1.744062	3.354483	4.241959
RERGL	1.736301	2.181269	1.600355	2.965515	2.407981	2.913604	2.326295	1.99582
RESF1	7.958666	8.076674	7.8595	8.681543	9.994235	8.08041	8.630468	9.587718
RESP18	0	0	0	0.034146	0	0.033548	0.03382	0
RET	14.68067	9.623924	8.015354	13.45356	13.82948	12.81799	11.65828	13.65915
RETN	0.199637	0	0.43373	0.163666	0.039933	0.0402	0	0.404959
RETREG1	3.842484	5.228857	3.374539	3.341929	2.821967	4.539695	3.259696	3.163766
RETREG2	56.35194	54.8961	56.93588	56.75234	53.32721	51.36308	51.80146	53.02918
RETREG3	20.44042	20.46658	17.29946	21.0472	17.98573	18.94526	20.31169	20.79355
RETSAT	5.817356	6.523847	5.916494	5.970827	7.867253	5.433471	6.953707	6.790761
REV1	6.155591	6.116693	5.224763	5.175296	5.932748	5.471257	5.609857	5.935124
REV3L	6.226269	6.409638	4.83968	6.418378	6.974428	6.343243	5.837246	7.905681

REX1BD	28.3397	29.58582	30.62281	28.26798	26.3747	27.5831	30.18799	26.77405
REXO1	11.36006	10.39055	9.51029	10.95629	10.73578	10.20249	10.59034	10.92453
REXO2	44.4658	38.95005	53.79451	42.96958	44.36592	44.76957	39.7726	39.23382
REXO4	12.83011	12.7583	12.18822	13.76244	14.6868	15.19241	13.94263	12.14995
REXO5	1.594549	1.848006	1.425563	1.689753	1.679325	1.477736	1.465203	1.513107
RFC1	13.67685	14.66255	14.19052	13.50522	12.8484	13.26881	14.13768	13.99348
RFC2	23.62487	23.07619	22.67155	24.21007	21.67998	24.36731	22.45995	22.62595
RFC3	16.66647	17.57304	14.72858	16.31624	13.97449	16.49368	20.07303	18.83622
RFC4	25.27802	29.40381	24.82822	23.46565	20.9507	25.47034	27.49833	26.74568
RFC5	9.576633	11.78394	9.7621	11.04284	9.366205	10.31682	11.09866	9.999164
RFESD	0.864061	1.117765	0.673468	1.057435	1.33554	1.096443	1.156075	0.956031
RFFL	7.409258	7.381908	7.614473	6.704337	7.004345	6.512687	8.219388	7.433662
RFK	12.5223	15.64862	13.90817	12.74657	14.80861	16.21611	13.35893	13.64646
RFLNB	6.871376	7.690514	7.73079	9.445306	8.455403	7.918033	7.478359	7.480025
RFNG	20.87379	21.11687	22.05816	23.54554	24.5794	22.94929	21.4498	21.09368
RFT1	4.072516	4.036118	3.6041	3.691041	3.847899	3.866817	3.849721	3.839903
RFTN1	5.265675	5.354577	5.605264	5.986776	5.81351	5.714763	5.215754	6.995341
RFTN2	3.522629	5.58266	4.979367	4.3413	4.605339	5.30845	5.055509	4.927161
RFWD3	7.455552	7.220073	6.144248	6.399971	6.193197	7.222552	6.708675	6.828757
RFX1	3.154064	3.227413	3.488536	3.025331	2.889496	2.750079	2.842821	3.512463
RFX2	2.579479	1.803567	3.013752	1.965776	2.739688	1.375366	1.652029	3.1247
RFX3	1.430557	1.298097	1.345167	1.271589	1.216131	1.130346	1.335223	1.510852
RFX4	0	0	0.007521	0	0.007617	0	0.00773	0
RFX5	5.867783	6.968344	5.74678	5.847479	6.749631	5.509129	5.832746	6.910411
RFX6	0	0	0.008231	0.008542	0.016672	0	0	0
RFX7	6.843996	6.833494	5.634146	6.498341	6.32304	5.679022	6.030486	7.18478
RFX8	0.044687	0.030183	0	0.061059	0.134079	0	0.060477	0.030216
RFXANK	13.48063	12.36009	11.59866	13.60733	13.63405	12.75836	11.5449	11.80966
RFXAP	12.72791	13.86207	13.94711	10.27151	12.71732	14.04706	11.50273	12.25123
RGCC	23.34415	29.29165	38.1967	29.50632	25.40196	27.49085	26.41433	34.63892
RGL1	17.91307	18.43613	22.87423	21.87001	18.80883	22.01865	19.65962	19.80955
RGL2	26.86766	23.49598	18.9921	26.82375	23.38155	24.04707	28.02253	26.78672
RGL3	0.145311	0.237814	0.275963	0.400913	0.536603	0.281354	0.351709	0.419465
RGMA	11.17826	10.07783	7.490295	11.50612	10.35273	9.426842	8.897533	11.53286
RGMB	27.79259	26.76971	24.05856	26.2197	22.41198	20.69157	23.2408	22.77311
RGN	4.766133	4.213075	3.769147	5.701467	4.272982	4.760457	3.180112	3.389565
RGP1	11.90389	11.32338	10.20221	12.70906	11.47854	11.21326	11.57701	10.19741
RGR	0	0	0.019302	0.02003	0.019548	0	0	0
RGS1	0.782914	3.393085	0.558392	1.248033	1.370276	0.678781	1.346503	1.301374
RGS10	4.154816	5.069317	4.941303	5.31072	5.428768	3.755884	3.945065	5.369404
RGS11	0.014585	0	0.043211	0.029893	0.131285	0.014685	0.044412	0.044379
RGS12	4.647826	4.414522	4.212733	4.729878	4.218017	4.419601	4.149691	3.731909
RGS13	0.098952	0.071607	0.0349	0.137618	0.077758	0.085395	0.100436	0.143372
RGS14	1.302934	1.895277	1.475572	2.070184	1.8889	1.125289	1.401341	2.182263
RGS16	3.336224	3.546044	3.479617	3.298903	4.390336	2.451492	2.340683	2.564502
RGS17	0.038695	0.039203	0	0.026435	0	0.012986	0.039275	0.013082
RGS18	0.669663	0.719989	0.998726	1.050383	0.779096	0.632958	0.665836	1.843531
RGS19	3.82252	4.180557	2.921987	4.507245	4.510837	3.6715	3.652586	5.158434
RGS2	52.0782	65.12274	61.29499	58.45495	57.4457	62.95437	68.12233	53.66569
RGS20	0	0.121818	0.105548	0.068455	0.093532	0.094159	0.108483	0.081301
RGS22	3.075422	3.251564	2.688811	2.573326	4.550238	4.410285	2.964044	1.724147
RGS3	2.376581	2.032036	1.998162	2.404425	2.732665	2.174364	1.894958	2.131502
RGS4	1.723806	4.044362	1.702327	2.184888	2.177721	2.009621	1.887794	1.564309
RGS5	29.85341	20.22729	21.19435	28.11719	25.86778	26.56052	20.17143	25.50741
RGS6	0.145049	0.173671	0.143242	0.105401	0.168806	0.14604	0.195407	0.184561
RGS7	0.51963	0.574309	0.256578	0.423581	0.578754	0.725318	0.731202	0.491094
RGS7BP	0.429025	0.542358	0.877354	1.381209	1.135356	1.024463	0.578045	0.539103
RGS8	0.454262	0.488988	0.528042	0.21821	0.520576	0.390666	0.489893	0.431933

RGS9	1.603169	1.441028	1.095141	1.704643	1.784208	1.395669	1.394757	1.564864
RGS9BP	0.087437	0.032637	0.045446	0.028296	0.032218	0.074134	0.037368	0.051342
RGSL1	0	0.022703	0.02213	0	0	0	0	0.007576
RHAG	0	0	0	0	0	0	0	0
RHBDD1	5.78657	3.929355	4.403623	4.559014	5.631745	4.90729	3.98926	4.354379
RHBDD2	9.043551	7.42268	6.601433	7.473841	8.119407	6.789137	8.172453	7.134944
RHBDD3	0.859107	1.492083	0.767602	1.481287	1.452488	1.510276	0.58825	0.767606
RHBDF1	10.80987	9.500538	8.713662	11.16571	10.70621	10.48952	10.18692	9.133236
RHBDF2	5.253652	4.805224	3.454188	4.45184	5.359278	3.86679	5.055538	5.222014
RHBDL1	1.762954	1.601958	0.879468	2.626122	1.545056	1.097937	1.272871	1.603716
RHBDL2	2.017401	1.694024	1.776884	2.793747	2.799278	1.646905	1.770951	1.695883
RHBDL3	2.945479	3.070632	1.784612	2.362257	2.888935	2.67907	2.527491	2.337973
RHBG	0.213964	0.433544	0.224504	0.397416	0.722222	0.094249	0.583654	0.515399
RHCG	5.124243	4.941995	4.078247	3.843742	3.457653	1.974057	6.220177	5.389323
RHEB	145.6445	165.3362	171.769	146.6682	162.8625	168.6253	153.511	144.8535
RHEBL1	1.10137	1.140081	1.489603	1.055044	1.029675	0.988362	1.652534	1.068481
RHEX	3.688789	5.779177	0.938873	3.994501	3.118761	3.503402	4.477506	5.669808
RHNO1	20.74491	17.29483	21.73581	20.45957	19.51695	19.01957	19.78955	18.08721
RHO	0.071933	0.021863	0.007104	0.0516	0	0	0.036506	0.138618
RHOA	156.8068	150.1385	169.6861	162.6492	179.8733	160.6508	158.4173	157.6424
RHOB	51.69052	56.20662	55.65751	49.45299	57.36679	59.71114	54.69534	51.55926
RHOBTB1	2.866392	3.407443	2.190158	3.066038	2.950463	3.033432	2.42095	2.620721
RHOBTB2	4.281054	3.114178	2.775062	3.378688	3.500366	2.604565	3.109648	3.097017
RHOBTB3	28.60871	26.92895	22.82162	25.12595	23.27157	24.42667	24.17409	29.01041
RHOC	28.03674	31.93921	31.50851	33.28141	33.14726	30.00052	28.90797	27.29392
RHOD	11.16508	9.787897	10.49732	13.11348	13.38453	12.0883	11.30668	11.40158
RHOF	3.299667	2.738886	2.307364	3.212746	2.655186	2.018317	2.245554	3.327485
RHOG	36.71973	35.81416	38.43877	38.74764	38.77899	38.41407	35.44609	34.74661
RHOH	0.881259	1.636846	0.725232	1.73092	1.312879	0.785612	0.875843	1.042772
RHOJ	8.848464	8.964591	7.594762	8.760018	10.89402	8.822558	10.0984	10.26477
RHOQ	6.293878	7.570736	8.619874	7.729669	9.164847	7.728586	9.628722	8.105662
RHOT1	10.2149	11.95631	11.24384	10.86786	11.0902	11.35245	11.45316	11.01429
RHOT2	20.11179	13.01525	13.91594	22.09481	21.24716	16.06785	16.76784	16.78626
RHOU	5.523383	5.932236	7.9881	8.433593	7.757885	8.012464	7.760906	7.224452
RHOV	18.20034	18.52226	19.67376	20.34825	18.35028	20.48731	19.17233	17.62792
RHPN1	0.785706	0.521979	0.725036	1.240751	1.133624	0.402021	0.483724	0.679317
RHPN2	3.17145	3.520803	3.387781	3.149304	3.448839	3.606872	3.336902	3.588092
RIBC1	1.021631	1.42764	1.652511	1.877266	2.131609	2.394179	1.430283	1.429206
RIC1	6.155599	5.474029	4.906787	5.242818	5.962432	5.006546	5.822743	6.152747
RIC3	1.336992	1.213904	0.955979	1.265293	1.19468	1.110738	1.216151	1.044806
RIC8A	17.53167	17.35043	16.88799	17.10862	15.7867	17.55235	16.49595	16.63327
RIC8B	2.978766	3.39272	2.622509	2.846168	2.908889	2.692603	2.961648	3.116177
RICTOR	4.637275	4.524566	3.951498	4.189741	4.170312	3.797729	3.722437	4.909445
RIDA	8.568613	13.55639	9.649899	12.83163	12.15476	13.3486	11.3636	10.20958
RIF1	5.090636	5.186856	4.625848	4.836368	5.410666	4.55337	4.392472	5.718322
RIIAD1	4.444631	4.259559	6.702504	4.062323	4.204924	4.414522	4.572264	2.741293
RILP	0.720248	0.678791	1.389464	1.287364	1.072135	0.978134	1.020072	0.747489
RILPL1	17.71419	14.83741	14.95977	15.63698	17.72876	16.84643	14.86489	14.35566
RILPL2	9.654068	12.08099	9.364203	11.71152	12.57481	11.46809	11.03826	11.18476
RIMBP2	0.216836	0.232862	0.132763	0.266648	0.203852	0.165921	0.184874	0.175938
RIMKLA	0.139052	0.060376	0.156936	0.210352	0.25165	0.173336	0.221788	0.134316
RIMKLB	2.990583	3.673854	2.805892	3.49401	3.121012	3.2389	3.216298	3.712075
RIMS1	0.003583	0	0.010615	0.003672	0.007167	0	0.003637	0.029071
RIMS2	0.020863	0.021137	0.009157	0.028507	0.006955	0.021006	0.01647	0.007053
RIMS3	4.653543	5.621117	3.684337	5.120846	3.223944	4.017724	4.743618	2.398382
RIMS4	1.309242	0.579614	0.57041	0.907602	0.682214	0.614784	0.770527	1.07681
RIN1	2.855367	2.347524	2.625321	2.650303	3.026424	2.346161	2.431821	2.995878
RIN2	14.97608	13.36263	14.91083	13.83914	13.63557	15.50666	12.74775	13.85346

RIN3	3.311619	3.233813	2.370679	3.236914	3.890658	3.233813	3.327545	4.255781
RING1	35.23957	36.5458	39.17069	36.51408	35.7994	35.98999	34.80683	32.19691
RINL	0.99257	1.238682	0.662124	0.990212	1.235942	0.655204	0.94074	1.613387
RINT1	7.386453	7.715316	6.533326	6.623241	6.799774	7.552156	7.946466	7.522561
RIOK1	6.379476	5.834469	5.366115	5.405644	6.576106	4.953974	5.729043	6.440319
RIOK2	20.83336	21.3209	21.85591	22.89647	23.02535	21.58367	21.85072	19.26193
RIOK3	58.47522	61.87662	59.35656	55.2986	58.86036	60.86166	62.37439	55.3393
RIOX1	3.854795	3.701626	3.351607	4.096268	4.073201	4.353615	4.388936	4.479123
RIOX2	26.11534	24.09789	23.98948	24.18461	26.81094	22.0446	26.16438	24.14145
RIPK1	11.00983	8.763626	8.621539	10.22074	10.65023	8.190952	8.406676	9.742774
RIPK2	9.514069	9.708969	9.634464	8.731371	11.03636	9.465961	9.884824	8.86072
RIPK3	4.98863	5.904323	4.496747	6.720711	5.76642	5.132239	6.451572	6.683146
RIPK4	16.13414	15.15463	13.6916	16.28212	15.45892	14.97808	16.07401	15.14106
RIPOR1	4.380774	3.590962	3.696925	4.272985	5.130151	3.672912	3.601654	4.354275
RIPOR2	0.658476	0.651234	0.661884	1.012181	0.772242	0.737954	0.735985	1.613971
RIPOR3	1.184694	0.984814	0.707959	1.13933	0.893193	0.795192	0.869475	0.807201
RIPPLY2	0.27712	0.44119	0.039095	0.5274	0.158375	0.318873	0.32146	0.200761
RIPPLY3	0.834667	1.212057	0.659413	1.739195	0.306084	0.672295	1.186062	2.088154
RIT1	30.20742	29.13316	32.2391	32.03377	31.23903	32.08884	30.95025	30.69534
RITA1	12.8227	12.16373	11.39364	13.29528	12.29505	13.36703	12.89225	10.78146
RLBP1	0.013366	0	0.0264	0.027395	0.106945	0.040373	0	0.013557
RLF	8.847227	9.013591	7.708323	9.168038	9.267789	7.527479	8.366627	8.849689
RLIM	8.769176	9.393241	7.854821	8.46848	9.0441	8.568602	8.787737	8.89096
RLN3	0.066168	0.156419	0.087125	0	0	0.088827	0.067161	0
RMC1	14.25433	12.97801	10.81092	15.07485	15.02917	12.3616	12.57764	14.18738
RMDN1	19.48214	19.89743	18.74674	24.99626	18.74939	20.98988	21.29343	14.30032
RMDN2	3.633286	4.951392	4.3977	5.667299	4.421606	5.001571	5.923301	5.494902
RMDN3	5.7117	7.758962	6.638052	7.923437	8.706417	7.276232	6.207479	3.67884
RMI1	2.531071	2.49937	2.303368	2.265442	2.069979	2.49675	1.944664	2.320141
RMI2	5.82221	5.859428	5.157507	5.807852	4.062532	4.537683	5.30092	4.924181
RMND1	2.574004	3.13103	2.78872	3.53409	2.624323	3.002552	2.697164	2.864107
RMND5A	32.72853	30.36988	32.93767	31.63515	33.23369	30.52829	36.18772	37.65168
RMND5B	18.32919	19.26282	17.8562	17.71955	16.53851	19.39256	20.29214	18.03983
RNASE10	0	0.355011	0.086511	0.089773	0.131422	0.352806	0.177834	0.222125
RNASE12	0.331015	0.074524	0.036321	0.301524	0.183921	0.037031	0.149325	0.484939
RNASE13	1.184845	0.348794	0.759449	0.66437	0.822791	0.661744	0.789643	0.312899
RNASE2	0	0	0.044013	0.091345	0	0	0	0.13561
RNASE6	8.185729	7.964251	8.885237	10.02817	8.233171	7.05093	9.767801	10.3249
RNASEH1	24.28964	22.03055	21.77179	19.642	21.22898	20.76449	22.10533	22.5471
RNASEH2A	9.269916	9.073472	8.932933	9.468891	10.39262	9.062278	10.18293	9.037935
RNASEH2E	8.012477	11.19626	10.8597	9.858725	9.567154	7.683062	10.20732	9.771197
RNASEH2C	16.27181	17.82768	17.37748	16.03842	19.42103	19.4678	17.31436	15.24364
RNASEK	121.426	128.7079	145.3604	141.1796	128.7752	140.1847	119.5475	110.4907
RNASEL	1.473114	2.423824	1.27975	1.657159	1.406839	1.248992	1.652601	2.550054
RNASET2	20.43702	24.255	24.95146	25.54466	25.22844	23.17329	23.40301	22.09315
RND1	0.369827	0.340619	0.348618	0.327307	0.487562	0.270802	0.375374	0.204595
RND2	3.685252	2.761854	3.468406	3.916956	3.678516	3.093233	2.137446	3.042836
RNF10	54.67599	50.25003	55.24268	53.40073	52.84521	53.03266	55.08725	54.24541
RNF11	51.28186	53.11696	70.65743	57.8277	57.64761	62.3351	54.15036	58.05765
RNF111	8.823795	8.327825	8.112153	8.133667	8.814054	8.769053	8.017468	8.441796
RNF112	1.296033	1.121738	1.144267	1.187407	1.776911	1.641911	1.219644	1.436356
RNF113A	0	0.108646	0	0.054947	0.026813	0.107971	0.027212	0.081574
RNF114	30.89278	30.47804	29.68706	27.79755	29.11552	29.07154	28.71577	29.78892
RNF115	15.27005	15.21802	14.50565	15.01604	16.49426	15.42215	15.67975	14.76541
RNF121	7.89438	7.553362	6.108323	6.88358	7.595902	7.069786	6.755064	7.88632
RNF122	1.04487	1.328859	1.350187	1.731428	1.912136	1.880184	1.28619	1.702355
RNF123	5.509096	4.807244	3.499172	5.178252	4.573017	4.609867	4.384569	4.237516
RNF125	4.306824	4.868022	7.122762	6.61494	7.587217	7.920179	3.865815	3.70502

RNF126	29.4089	27.0302	26.12911	31.47891	29.59711	28.55166	27.2674	29.92106
RNF128	5.540107	5.970216	6.753731	8.175037	6.33466	7.531449	7.462347	6.253243
RNF13	34.67736	38.79483	39.80359	38.3358	36.47188	38.18636	41.40041	38.56272
RNF130	29.11973	34.50143	37.17575	37.54397	32.65315	27.58046	33.23171	35.08163
RNF133	0	0.038633	0.037657	0.039077	0	0.019196	0.019352	0
RNF135	5.129064	6.221776	5.065155	6.363483	4.89379	5.187089	6.990252	5.989338
RNF138	3.612707	3.829919	3.613667	3.253733	3.594549	3.337396	3.610193	3.853009
RNF139	20.19066	24.47221	22.82268	24.96031	22.08082	25.96945	25.09582	23.17479
RNF14	5.421689	5.582889	4.723766	5.473184	5.874929	5.670243	5.708044	5.990572
RNF141	8.206362	9.999045	8.147331	9.792554	8.608674	10.80358	9.069853	8.559933
RNF144A	6.001235	6.500465	5.319878	5.346065	5.732211	5.536624	5.581543	5.871992
RNF144B	15.50483	16.943	17.81655	19.16347	16.46909	17.36028	17.49521	18.63528
RNF145	41.44593	42.02851	45.86395	43.87972	39.04036	51.99045	46.75987	41.50208
RNF146	15.46732	16.92452	14.36121	18.31478	14.87161	18.381	16.9703	17.33275
RNF148	0.056889	0.057635	0.022472	0.01166	0.011379	0.011455	0.06929	0.023079
RNF149	25.7569	24.55409	25.4178	28.62591	24.03675	28.29151	24.60578	28.12015
RNF150	0.863881	1.138073	1.055837	0.864827	1.114921	0.800889	0.882627	1.61934
RNF152	4.805136	4.049472	4.34127	4.553045	4.72527	5.80353	4.564092	3.914477
RNF157	2.597752	2.350041	1.85414	3.028583	2.538476	1.920366	2.470348	2.740525
RNF165	0	0.017072	0	0	0.016853	0	0.017104	0.017091
RNF166	6.827031	7.522464	5.200007	6.791872	6.744848	6.238148	5.73238	6.250328
RNF167	32.77144	33.40741	32.30958	33.19447	33.76477	35.65513	30.59537	31.57537
RNF168	6.194118	6.642689	5.394757	4.932001	6.638753	5.745574	6.204557	6.516852
RNF169	4.974137	4.093241	4.074084	4.760299	4.325013	4.366261	4.632485	4.719599
RNF17	0	0	0	0	0	0	0	0.011287
RNF170	28.34983	33.19042	37.33868	26.23387	31.58488	34.30819	30.08685	31.19208
RNF175	0.022608	0	0	0	0.022611	0.022763	0	0.02293
RNF180	18.72113	18.49812	24.79261	19.76957	17.36632	25.4322	16.73235	14.6552
RNF181	37.42327	39.65943	37.26624	44.04664	40.40357	38.27013	40.80566	37.91632
RNF182	0.141122	0.315734	0.162591	0.241029	0.241114	0.118405	0.238731	0.202769
RNF183	0.065818	0	0.048749	0.337242	0.04937	0.033134	0.100208	0.050066
RNF185	28.51464	27.04906	23.48901	31.90548	27.3745	29.12179	24.9971	27.54115
RNF186	0.092313	0.205753	0.109395	0.264878	0.092324	0.037177	0.037479	0.056176
RNF187	15.48337	18.56399	17.89163	18.73045	16.10387	15.33547	14.7857	15.28564
RNF19A	25.8651	24.71823	22.33028	23.83887	24.94343	24.71242	23.92235	26.01335
RNF19B	37.32923	32.5167	36.8641	33.48408	36.21683	40.26065	35.32753	33.5385
RNF2	10.73283	11.12186	10.72005	10.27865	11.56588	10.21555	10.76951	11.65056
RNF20	17.90669	17.56299	15.67646	14.81088	16.48744	14.96623	15.83596	16.85472
RNF207	0	0.010109	0	0.040902	0	0.050232	0	0.01012
RNF208	0.655394	0.507761	0.513975	0.987688	0.732593	0.543423	0.723921	0.56697
RNF212	0	0.032005	0.124787	0	0	0	0	0
RNF212B	1.558599	1.811757	1.960428	1.899835	1.509576	1.4371	1.631936	1.431028
RNF213	2.948158	3.11608	2.331973	2.803869	3.750657	2.560539	3.078154	3.633756
RNF214	9.849352	8.79923	7.487404	8.807021	8.888847	8.703812	8.57921	9.147689
RNF215	4.499542	4.587722	1.987498	4.198512	5.175861	4.400017	4.421124	4.826039
RNF216	4.554708	4.526242	3.207766	4.720032	4.662902	3.616043	4.368216	4.4013
RNF217	2.423325	2.151301	2.104139	2.287559	2.430895	2.157419	1.848438	2.308195
RNF219	8.600068	8.912278	8.719604	9.039934	9.535913	9.451238	9.261634	9.678728
RNF220	15.38736	14.45144	14.1891	16.00174	14.00876	14.04784	15.45245	15.13471
RNF222	22.3185	17.81505	17.85324	15.46081	18.1849	18.64724	16.89755	16.67376
RNF223	3.095238	2.526522	2.5931	3.487595	3.051624	3.249309	2.650316	2.871493
RNF224	3.025062	3.600352	4.394043	4.168438	3.745099	3.977188	3.085343	2.814931
RNF225	0.057063	0.028906	0.140879	0.14619	0.17121	0.086179	0.057918	0.028937
RNF227	0.65445	0.524264	0.871748	0.514694	0.806752	0.873454	0.556131	0.524839
RNF24	3.166767	2.885055	2.604109	3.146495	3.534473	2.642857	2.804847	3.822466
RNF25	18.13189	18.56156	17.43028	21.04909	19.13213	19.24302	18.12449	16.52309
RNF26	6.429423	7.293899	6.330301	7.821594	6.430254	6.512108	7.072943	7.467854
RNF31	7.045382	6.796788	5.651562	6.768142	6.525236	6.125116	6.345653	7.820412

RNF32	0.096657	0.111915	0.136361	0.155652	0.06905	0.097318	0.140153	0.196066
RNF34	20.0589	21.03823	19.29245	18.78846	16.2443	21.07835	19.32673	17.26196
RNF38	17.04896	17.52845	15.99893	16.60727	16.67247	16.82486	16.15173	17.46575
RNF39	84.85827	71.34247	82.39344	78.63896	78.93308	88.08137	74.64382	64.33574
RNF4	30.76471	28.77089	24.44744	27.13557	28.93756	27.91563	29.0614	30.45198
RNF40	21.86941	20.41827	19.97406	21.81834	21.97742	22.1247	20.92979	20.50735
RNF41	12.48666	11.50341	11.74587	12.32966	12.26073	11.63972	11.36897	12.8093
RNF43	3.6714	3.074287	3.115332	3.497561	3.11899	3.18829	2.915308	3.528644
RNF44	5.192319	3.853424	3.73461	4.309312	4.588029	3.975654	4.236301	5.047831
RNF5	30.35728	28.84144	32.79034	31.08343	28.39592	31.65536	29.4574	29.63963
RNF6	5.673275	5.954099	5.118319	5.087079	5.674009	5.917119	5.09218	5.960633
RNF7	27.41115	34.88817	30.56033	33.0566	29.81598	33.10466	32.3127	31.54429
RNF8	10.93186	10.10509	8.466996	9.313932	8.5659	10.21516	10.38974	9.896261
RNFT1	72.72805	69.66515	83.1501	75.42671	75.17105	81.43013	78.96762	71.67414
RNFT2	1.116361	0.99761	0.932843	0.821343	0.927558	0.887662	0.79608	0.923221
RNGTT	6.956186	8.111008	6.732121	7.251622	7.1405	7.875988	7.066948	7.812044
RNH1	15.96186	15.10012	14.97984	15.13826	18.21107	15.40554	16.41799	16.27157
RNMT	5.492014	5.707035	5.148397	5.783484	5.792583	5.955701	5.961057	5.312615
RNPC3	4.125788	3.847931	2.647215	3.717538	4.349189	3.784438	3.332844	4.101429
RNPEP	13.31835	13.168	10.88865	14.78756	12.99911	13.167	12.44784	9.392788
RNPEPL1	30.29542	26.27848	29.14261	31.11625	30.03433	27.83454	26.58612	27.32258
RNPS1	76.20053	73.24025	74.19136	76.45167	75.20106	71.94934	75.50234	74.56444
RO60	7.814511	7.894318	7.875538	7.607017	8.640065	7.561061	7.931728	8.208818
ROBO1	26.70607	25.94286	20.85829	22.06286	21.26025	19.20396	24.26315	25.10251
ROBO2	1.417367	1.26849	1.106463	1.537177	1.295083	0.81986	1.044015	1.155003
ROBO3	0.025543	0	0.025225	0.039264	0	0.032147	0.032408	0.01943
ROBO4	4.040293	5.150884	4.175356	4.971525	6.50436	4.400584	4.260969	2.536376
ROCK1	15.4891	16.51659	15.9641	15.56366	16.80298	15.93084	16.30131	17.68431
ROCK2	16.6808	17.18695	14.38715	14.6245	16.79283	15.79571	15.49942	17.34599
ROGDI	2.779066	3.784522	2.655333	2.75544	2.725281	4.160739	2.014823	2.580692
ROM1	1.881757	1.730227	1.686534	2.058007	2.024337	1.783165	1.605028	1.716087
ROMO1	31.66657	39.62221	40.17804	36.84755	39.05562	37.70117	41.11751	38.89606
ROPN1L	0.031806	0.161116	0.03141	0	0.06362	0	0.032283	0.064517
ROR1	2.275812	2.324934	1.745414	2.01084	2.428164	2.23874	2.020603	2.028719
ROR2	6.774799	6.58413	4.789552	6.53963	6.037388	6.334854	6.918436	6.136537
RORA	7.109423	7.853901	9.023205	8.441733	7.847968	9.688313	7.46012	6.110863
RORB	0	0.371212	0.060306	0.06258	0.061075	0.081979	0.144628	0.165164
RORC	8.102319	7.801552	5.54107	9.201449	7.423823	7.054288	8.016218	8.721539
ROS1	0.011982	0.039451	0.035497	0.046044	0.050929	0.027143	0.021282	0.012152
RP1	0	0	0	0	0.004759	0	0	0
RP1L1	0	0	0	0	0	0.005127	0	0
RP2	4.349805	3.747917	3.492689	4.374232	3.577872	3.213013	3.569177	3.648952
RP9	19.0225	15.91043	16.40915	18.81881	16.51701	20.19806	19.89916	18.70242
RPA1	49.35529	49.54344	43.58853	47.09131	43.84175	40.78618	47.63995	44.79752
RPA2	29.09691	27.7729	26.47354	27.91054	25.76222	29.32543	27.89929	25.15187
RPA3	19.46061	27.37462	27.48728	27.5304	28.80698	33.44956	32.85529	27.95511
RPA4	0.01439	0	0	0	0	0	0	0
RPAIN	3.116566	2.489542	2.337894	3.132346	3.116969	3.318888	2.646235	3.252114
RPAP1	6.625397	7.0634	6.259654	5.607927	6.004855	5.539834	5.718183	6.671239
RPAP2	1.741171	1.572424	1.658296	1.680718	1.571822	1.434625	1.270859	1.600606
RPAP3	12.04038	12.76255	13.77871	13.40453	13.61811	12.45056	13.57532	12.08391
RPE	7.763297	7.262743	8.155921	6.559136	7.995578	7.043015	6.362476	8.225622
RPE65	0.01508	0.381957	0.044677	0.139085	0.060329	0.546602	0.397971	0.244721
RPF1	23.40806	23.43111	23.08177	23.49687	25.56168	28.29804	26.81952	24.83175
RPF2	4.838904	4.291471	5.955108	4.099648	4.574753	6.293567	4.030704	3.953083
RPGR	0.718434	0.956127	0.768256	1.106523	0.799308	1.018671	0.832767	1.026162
RPGRIP1	0	0.005632	0.021959	0.022786	0.016679	0.027984	0	0.02819
RPGRIP1L	1.125402	1.44844	1.251331	1.324135	1.413187	1.086927	1.307279	1.428892

RPH3A	0.046805	0.04215	0.03595	0.053293	0.078018	0.047124	0.121406	0.026373
RPH3AL	1.356785	1.806923	1.880153	1.973461	1.575825	1.520286	1.676997	1.609149
RPIA	8.052101	6.310142	8.089133	9.265181	8.671425	8.402762	8.298378	6.755969
RPL10	1673.277	2014.305	1732.294	1777.772	1779.843	1768.641	1817.487	1850.589
RPL10A	1843.864	1611.791	1587.673	1453.165	1636.411	1935.405	1992.26	1885.095
RPL11	1989.549	2364.577	2289.62	2108.968	2065.824	2271.338	2236.188	2128.184
RPL12	1687.679	2004.302	1983.737	1824.758	1565.795	1812.421	1889.922	1747.55
RPL13	1266.149	1582.084	1525.597	1360.425	1254.954	1310.471	1417.463	1279.357
RPL13A	2025.33	2381.67	2322.297	2156.887	2139.225	2254.84	2201.129	2105.604
RPL14	1975.526	2280.635	2315.01	2122.54	2101.349	2147.198	2058.251	1981.971
RPL15	455.318	531.2436	540.9691	463.5038	457.105	511.0359	516.9117	467.4293
RPL18	1437.418	1678.591	1670.288	1461.329	1460.03	1477.272	1535.427	1488.867
RPL18A	1971.418	2191.895	2116.893	2099.515	1999.788	2007.841	2104.049	1962.599
RPL19	1560.141	1871.255	1927.371	1636.221	1613.032	1715.714	1717.193	1632.531
RPL21	2029.99	2485.861	2363.128	2012.45	2058.848	1715.231	2301.435	1708.192
RPL22	1166.634	1496.575	1712.463	1341.523	1321.899	1621.269	1259.838	1220.561
RPL22L1	62.08847	66.35562	107.4286	68.29847	70.83984	74.67444	66.884	63.83041
RPL23A	5111.439	5935.658	5505.196	5168.446	5199.228	5596.467	5358.505	5218.619
RPL24	1318.027	1552.266	1703.777	1496.918	1430.576	1591.006	1444.4	1359.16
RPL26	1566.901	2399.438	1721.041	1703.859	1636.077	1695.874	1722.115	2550.445
RPL26L1	10.52075	12.61629	12.42839	8.720012	11.44858	9.713235	10.15471	9.811509
RPL27	1195.919	1275.94	1394.123	1319.29	1437.215	1369.566	1532.653	1411.964
RPL27A	2544.609	3013.409	2907.6	2941.08	2674.155	3000.102	2694.752	2664.128
RPL28	857.0888	904.4793	916.0674	843.0983	812.4958	834.5863	872.4498	771.3278
RPL29	1438.846	1581.634	1470.862	1538.254	1442.283	1493.135	1546.427	1450.123
RPL3	1669.852	1765.979	1564.402	1476.07	1537.565	1624.56	1607.903	1478.585
RPL30	1463.474	1882.559	1773.659	1595.703	1489.775	1788.449	1684.231	1449.502
RPL31	3025.727	3421.333	3314.798	3166.443	3164.497	3237.551	3377.564	3316.083
RPL32	1848.679	2138.241	2159.543	1968.475	1898.528	1884.074	2031.193	2004.963
RPL35	3002.435	3436.14	3573.114	3147.397	3162.547	3387.901	3247.898	3205.699
RPL35A	1773.837	2199.056	2534.617	2043.963	2068.789	2215.185	2036.63	1968.176
RPL36	655.238	748.257	742.8937	712.9983	671.0686	714.7834	762.4266	689.5232
RPL36A	613.1343	1028.765	761.0116	665.1914	757.9502	1030.149	737.7633	795.2337
RPL36AL	543.0816	545.0025	698.5775	563.0697	545.2627	616.2724	551.2278	526.8926
RPL37	1900.382	2230.832	2184.812	2133.68	1961.038	2253.86	2094.851	1953.422
RPL37A	560.3874	696.0601	1290.052	1210.957	598.3709	1249.718	1202.835	1119.881
RPL38	645.5722	839.2538	825.9148	903.9021	866.0153	903.7892	777.889	742.7578
RPL3L	0	0	0.016221	0	0.032855	0.016538	0.016672	0.066638
RPL4	2655.748	2863.267	2642.814	2631.985	2445.886	2825.512	2831.982	2601.328
RPL5	2032.066	2533.295	2427.242	2237.313	2190.841	2304.048	2255.914	2181.84
RPL6	871.08	1072.702	994.9706	918.6265	836.4525	1000.264	944.9731	992.6352
RPL7	1551.393	1894.006	1665.83	1704.404	1617.754	1718.594	1752.147	1640.256
RPL7A	2387.376	2599.503	2638.073	2396.842	2324.95	2269.197	2530.24	2331.28
RPL7L1	73.21965	67.99887	78.09339	82.75847	69.53407	71.503	80.28384	78.5769
RPL8	1695.163	1859.254	1852.056	1761.029	1673.463	1784.626	1938.403	1735.608
RPL9	1238.653	1529.042	1444.323	1361.783	1336.542	1449.994	1357.89	1259.852
RPLP0	2521.549	2717.786	2658.099	2529.23	2389.064	2461.435	2703.854	2506.292
RPLP1	3933.107	4526.472	4677.907	4153.425	3674.918	4106.683	4286.408	4369.193
RPLP2	1366.481	1527.432	1498.275	1358.527	1307.568	1365.939	1417.3	1346.079
RPN1	115.0694	120.8775	113.9702	122.4728	108.1927	121.5547	130.554	122.194
RPN2	48.40638	58.37967	52.02051	55.72527	53.15699	50.42744	53.94737	57.81518
RPP14	0.008546	0	0.00844	0	0	0	0.008675	0
RPP21	7.207086	6.251432	7.653519	6.222122	7.109278	10.68568	7.114777	8.060679
RPP25	0.246684	0.288371	0.26235	0.661155	0.474454	0.324791	0.57781	0.365671
RPP25L	9.811973	9.207874	9.28674	9.864943	9.034119	9.692256	9.149619	8.72886
RPP30	10.38419	11.73276	11.51797	9.936677	10.35802	11.24445	9.520855	10.25302
RPP38	6.638456	6.988139	7.303842	8.192072	7.735897	9.112457	7.931858	6.69252
RPP40	2.694881	4.660472	3.812471	3.057065	3.597818	3.621929	3.320533	4.04266

RPRD1A	9.320584	8.956875	7.823781	8.708682	8.586098	8.708039	8.421604	8.4477
RPRD1B	13.35224	12.10503	11.85481	11.43126	13.19951	11.84608	12.34121	13.12936
RPRD2	6.934563	6.513986	6.086219	5.418942	6.261048	5.813544	6.462379	6.93785
RPRM	0.529883	0.843601	1.046561	0.795119	0.832781	0.171483	0.710705	0.940495
RPRML	1.280754	0.253183	0.246789	0.38414	0.281178	0.408867	0.190239	0.158413
RPS10	1967.943	2128.691	2188.775	2081.317	2003.951	2173.863	2034.749	1962.293
RPS11	3654.294	4224.106	4306.634	3707.514	3802.449	3978.419	3903.997	3735.56
RPS12	1720.827	2000.348	2036.125	1759.847	1675.408	1913.796	1944.312	1817.131
RPS13	941.5483	1451.312	1185.635	1317.494	1230.778	1346.967	1350.323	1289.366
RPS14	1153.896	1267.431	1281.137	1171.187	1175.558	1240.466	1221.924	1157.462
RPS15	1733.893	1843.276	1739.372	1753.203	1702.792	1787.028	1881.706	1824.034
RPS15A	2026.339	2349.205	2246.879	2017.988	2030.874	2192.168	2184.435	2067.24
RPS16	2254.743	2310.155	2688.896	1884.135	2145.294	1946.05	2381.229	1909.955
RPS17	3491.757	3961.17	4014.234	3668.43	3401.34	3997.239	3675.344	3531.645
RPS18	2599.325	2815.849	2711.858	2585.081	2367.079	2542.258	2851.284	2623.407
RPS19	1067.615	1308.508	1237.995	1070.242	1095.286	1157.278	1118.462	1107.175
RPS19BP1	43.17086	53.02021	45.57201	53.32129	42.47411	57.16879	43.00383	44.02284
RPS2	3921.637	4272.725	3906.045	3821.124	3808.333	4176.226	4288.292	4123.747
RPS20	2123.78	2750.919	2730.637	2269.347	2536.242	2396.44	2448.953	2132.953
RPS21	623.4308	764.9612	914.5288	650.0911	642.9162	759.0882	752.5392	679.3082
RPS23	727.1251	950.7125	910.1202	831.5256	800.1487	860.1919	855.8071	834.4453
RPS24	2578.322	3356.807	3275.218	3021.406	2844.513	3238.997	2762.763	2695.115
RPS25	2183.679	2697.984	2783.396	2479.253	2547.147	2946.796	2574.774	2542.187
RPS26	516.3518	584.9651	624.4065	551.7715	932.2562	599.0564	609.0106	559.1771
RPS27	1469.358	1919.482	2202.044	1637.679	1636.772	1906.38	1632.997	1561.021
RPS27A	2955.833	3493.081	3465.204	3088.683	3057.51	3514.573	3298.54	3127.826
RPS27L	114.0046	146.9962	151.2185	125.2773	141.0668	146.0304	125.6286	125.2676
RPS29	2416.343	2826.505	2747.196	2607.219	2579.311	2831.294	2724.154	2650.966
RPS3	1152.226	1634.585	826.342	1206.605	1194.968	1188.738	718.7113	1428.589
RPS3A	1978.417	1956.662	1828.042	2252.739	1705.98	1711.719	2288.752	2745.716
RPS4X	1770.489	2162.945	2074.292	1935.584	1874.635	1898.917	1993.35	1875.247
RPS5	1522.322	1766.847	1675.116	1606.81	1508.287	1537.121	1674.848	1526.667
RPS6	1695.631	2074.117	2166.918	1726.774	1801.506	1882.118	1844.154	1822.695
RPS6KA1	13.15696	10.94391	12.05659	14.14689	12.66867	10.48609	13.1538	13.80109
RPS6KA2	2.085753	2.473004	2.226164	2.338092	2.577923	2.77402	2.209486	2.678948
RPS6KA3	18.15999	17.53512	19.05235	16.72308	17.22859	17.30789	17.91218	18.39864
RPS6KA4	3.651531	3.313346	3.124645	3.560331	3.634275	2.766281	2.986634	3.307992
RPS6KA5	7.920516	9.914897	8.223367	8.065338	8.434044	9.78602	8.949542	9.055788
RPS6KA6	1.877723	1.935668	1.582467	1.915814	1.939606	1.638201	1.522209	1.908621
RPS6KB1	15.45612	15.51535	15.22492	15.55846	15.45812	16.4082	14.55676	16.84616
RPS6KB2	27.98916	19.486	27.32542	26.78664	26.2861	28.96718	27.98813	26.39805
RPS6KC1	3.298534	3.836414	4.252037	3.988684	4.438202	3.856868	3.972975	4.023511
RPS6KL1	0.630116	0.595827	0.438549	0.670323	0.894281	0.495453	0.536019	0.93124
RPS7	1787.163	2181.771	2207.55	2089.574	2001.39	2201.179	2048.66	1969.787
RPS8	5913.118	6637.151	6752.537	5729.114	5738.683	6672.318	6366.3	6141.048
RPS9	841.9938	940.2312	948.7403	918.7426	899.362	926.1379	896.4913	861.0356
RPSA	1819.711	1963.194	1658.502	1718.662	1765.543	1856.092	2013.169	1929.936
RPTN	1163.399	886.559	787.8122	926.066	934.04	1047.003	1007.773	899.0236
RPTOR	4.339589	4.766632	4.178629	4.298037	4.647986	3.878845	4.126601	4.596906
RPUSD1	2.142939	1.616236	1.798802	2.14722	1.047794	1.306534	1.643396	1.557636
RPUSD2	3.493924	4.525176	3.776777	3.919163	3.777703	3.698436	3.814704	3.936338
RPUSD3	6.539627	5.307066	6.087861	6.407785	7.058858	6.073548	6.324305	6.218877
RPUSD4	5.074671	4.823254	5.49364	5.754364	4.743948	5.26736	3.663965	5.023103
RRAD	0.403413	0.389245	0.512211	1.259902	0.902993	1.005751	0.584949	0.253287
RRAGA	159.972	153.0107	157.6267	171.7294	162.9049	162.5308	168.554	152.0636
RRAGB	5.243077	7.003235	5.486867	6.762967	5.79161	4.937475	5.454105	4.828274
RRAGC	37.6221	32.84627	33.26063	32.26777	33.2181	34.35657	34.70633	29.64139
RRAGD	0.58229	0.861698	1.266368	0.972173	0.759038	1.11325	0.883216	0.676843

RRAS	14.5439	17.96795	18.88331	19.64883	23.15291	18.77814	17.68261	18.01419
RRAS2	14.44563	18.95785	18.00961	15.81533	16.22752	14.88798	15.78828	14.96037
RRBP1	28.79699	30.91671	30.96442	38.47324	36.16015	33.1908	34.12234	33.57668
RREB1	6.519347	5.853536	5.43601	5.747109	5.783166	5.120256	5.405549	6.406793
RRH	0.054383	0.036731	0.053705	0	0.05439	0.018252	0.036799	0.036772
RRM1	34.06911	35.3122	30.4794	31.76572	29.18928	31.89281	35.39571	34.08324
RRM2	12.19215	13.03184	10.74169	10.83499	9.867702	9.879792	11.65777	12.54716
RRM2B	1.68597	2.043428	1.695606	1.870826	1.825841	1.39548	1.926479	1.762424
RRN3	12.62988	12.98097	9.654273	10.56565	13.31944	12.0973	11.9652	12.62414
RRNAD1	3.303675	3.31411	2.171441	3.396608	3.033274	2.835487	2.759543	2.93324
RRP1	12.13748	11.9104	10.64352	12.40415	12.96822	11.96998	11.57902	12.10846
RRP12	8.774431	7.755842	8.206729	8.099115	8.788284	6.683396	8.473653	8.241564
RRP15	9.52849	11.15129	8.992643	10.01913	10.36218	9.568569	8.397867	8.94594
RRP1B	4.929366	4.474894	3.795412	4.40093	4.3869	4.474053	4.956728	4.421625
RRP7A	12.45867	11.25828	11.33656	10.76063	12.38684	10.4244	10.44686	11.04721
RRP8	7.46039	6.547406	5.903069	6.419116	8.038858	6.469533	6.995582	7.782112
RRP9	4.360039	3.871307	3.741293	4.334165	4.327939	4.176088	3.994498	3.54431
RRS1	8.871699	9.684181	10.81133	11.38728	10.32178	10.18042	9.959826	8.573845
RSAD1	1.609821	1.490867	1.316675	1.700301	1.975496	1.54127	1.323214	1.171965
RSAD2	0.669344	0.55321	0.347897	0.523469	0.651814	0.301489	1.376648	1.05404
RSBN1	6.559006	7.126679	6.274865	6.573996	6.616555	6.046179	6.776906	6.603724
RSBN1L	5.233226	5.767697	5.0022	5.452987	5.505794	5.156272	5.445634	5.49019
RSF1	6.247168	6.707672	5.600159	5.921101	6.534739	6.047816	6.20565	6.91478
RSKR	6.574264	5.564505	3.652516	5.979067	6.325426	4.822409	4.598748	6.799146
RSL1D1	48.8969	48.40654	46.63905	43.7756	47.07204	50.03067	42.96195	44.86853
RSL24D1	61.64209	73.04681	70.52153	70.75418	76.63549	88.01087	60.65048	69.35816
RSPH1	0.302819	0.306794	0.106802	0.731469	0.367757	0.76222	0.219544	0.329068
RSPH10B	0.102049	0.075191	0.045808	0.038028	0.083505	0.037362	0.07533	0.103501
RSPH14	0.2534	0.140032	0.090997	0.165249	0.092157	0.046387	0.093528	0.116821
RSPH3	3.09206	3.064834	2.736282	2.400493	2.329385	2.358472	2.866714	2.878131
RSPH6A	0.01158	0	0	0	0	0	0.011754	0.058725
RSPH9	1.448521	3.36112	2.16878	3.30399	2.12633	1.646599	2.276514	2.014146
RSPO1	3.221488	3.424941	2.906417	3.556014	3.341234	2.452644	2.91659	3.559796
RSPO2	0.009135	0.037019	0	0	0.009136	0	0	0.092649
RSPO3	0.364367	0.272005	0.340889	0.294784	0.412364	0.502014	0.321171	0.41818
RSPO4	0.200209	0.162269	0.118628	0.164134	0.120141	0.564414	0.731562	0.24367
RSPRY1	9.591477	10.65905	8.870571	9.23784	9.143935	9.326227	8.80004	9.272906
RSRC1	10.34195	12.82863	10.496	9.864185	10.94498	14.53727	14.36443	11.94197
RSRC2	38.1703	40.0014	36.19775	40.13099	39.38089	39.54866	39.48185	38.66525
RSRP1	35.45958	42.78702	41.55899	40.40808	42.06389	39.52523	38.49369	36.2927
RSU1	44.72873	45.06204	42.17398	42.30318	41.09333	43.54091	46.51412	40.55931
RTBDN	0.024595	0	0	0	0	0	0	0
RTCA	14.56347	16.26592	18.25216	15.10558	16.60096	17.37408	16.20622	15.66832
RTCB	30.73109	31.52658	32.28078	31.27193	31.3588	30.8436	34.99011	32.40092
RTEL1	4.325821	3.985304	3.691036	4.527158	4.167052	3.960552	3.974026	4.045607
RTF1	20.03385	21.31882	19.05544	18.2058	18.30974	18.88235	20.69858	20.84779
RTF2	23.73453	27.73308	27.24109	25.79981	25.93552	25.7376	26.1784	26.01603
RTKN	12.1561	10.34981	11.01592	11.42214	11.56397	11.73067	10.24307	10.88237
RTKN2	1.065813	1.220644	1.098295	1.115957	1.073675	1.181958	0.877982	1.151484
RTL1	0.017173	0	0.00212	0.0022	0.002147	0	0.002179	0.002177
RTL10	0.123232	0.249699	0.258606	0.315712	0.261903	0.186111	0.093811	0.124987
RTL5	8.078403	7.899003	6.169363	6.842219	8.614789	5.545308	6.426697	7.279057
RTL6	13.01598	11.24183	11.07412	10.30793	11.31157	10.54207	10.97497	10.56535
RTL8C	46.40608	43.85927	37.16075	34.5308	43.4559	44.89172	38.7941	44.23025
RTL9	0.099943	0.040502	0.069089	0.035847	0.049978	0.035219	0.060866	0.055751
RTN1	4.78883	4.631532	3.581947	3.939664	4.338087	4.468133	4.156587	4.797667
RTN2	2.052995	1.566887	1.081288	2.005669	1.560478	1.653616	1.750384	1.693539
RTN3	28.84356	30.17114	35.2799	31.71528	31.77629	35.4198	30.82257	32.2036

RTN4	60.31696	60.60007	65.805	62.53109	63.89427	59.8082	57.78591	62.41993
RTN4IP1	7.758698	8.601757	7.729235	7.724214	7.096043	7.623262	8.255017	6.730193
RTN4R	0.726362	0.588716	0.526029	0.95939	0.855604	0.633815	0.688107	1.162353
RTN4RL1	0.641933	0.55485	0.638367	0.483025	0.453452	0.415813	0.788253	0.956118
RTN4RL2	0.31777	0.334818	0.200839	0.247487	0.330523	0.358334	0.348339	0.438319
RTP1	0.011156	0.011302	0	0.011432	0.011157	0	0.011323	0
RTP3	0	0	0	0	0	0	0	0
RTP4	1.627076	1.964086	0.905963	1.38357	2.042763	1.620764	1.792034	1.070899
RTP5	0	0	0.017221	0.017871	0.017441	0	0	0
RTRAF	123.7143	136.7247	143.7786	131.4473	127.7682	138.6771	134.2921	130.0684
RTTN	2.200585	1.806636	1.483747	1.893502	2.288145	1.772495	1.952469	2.255
RUBCN	6.826296	6.865736	5.950244	6.963107	7.367232	6.311135	6.257288	6.704405
RUBCNL	0.703789	0.493633	0.534631	0.515159	0.727085	0.552862	0.612297	0.90991
RUFY1	8.220662	10.83204	9.54973	10.85687	9.058492	10.706	8.729078	9.196125
RUFY2	5.323615	5.613819	6.209755	5.479708	5.584371	5.317141	5.36028	5.260339
RUFY3	4.600193	4.27746	4.640291	4.586094	4.949382	4.575338	4.292056	5.055876
RUFY4	0.177345	0.137396	0.05151	0.160357	0.125201	0.12604	0.084708	0.095225
RUNDC1	5.23501	5.645043	4.393461	6.010924	6.116941	6.331886	5.664264	5.335819
RUNDC3A	0.062516	0.081432	0.070556	0.082368	0.026796	0.035967	0.063453	0.036232
RUNDC3B	1.839266	1.481368	1.466758	1.332784	2.047314	1.224223	1.023255	1.116147
RUNX1	7.306782	7.440397	7.091646	7.745315	6.879503	6.981835	7.463622	7.264471
RUNX1T1	4.774044	5.591655	3.420683	3.709084	4.266893	3.536975	3.77346	4.94167
RUNX2	3.0157	3.138249	2.259759	2.789259	2.919729	3.628041	2.581748	3.40065
RUNX3	0.975401	1.387688	0.922258	0.949938	1.376808	0.947239	1.081311	1.50147
RUSC1	5.297261	4.662117	4.090561	5.409384	4.987398	4.758213	4.61402	4.717621
RUSC2	2.188841	1.774633	1.721353	1.853599	2.131967	1.438508	1.603898	2.422873
RUVBL1	36.87568	39.9003	34.98682	37.35792	37.89041	40.61837	45.85073	46.56729
RUVBL2	16.26377	19.41689	14.82678	16.54464	17.47131	18.69703	19.83042	18.11012
RWDD1	56.1142	62.2191	53.64835	52.65414	65.24855	46.4657	62.49521	56.98192
RWDD2A	0.584166	0.585399	0.49537	0.650691	0.635045	0.53062	0.554259	0.605362
RWDD2B	10.49832	11.13798	9.784218	10.47525	10.5092	10.88656	10.32703	9.372355
RWDD3	3.194416	5.99108	3.379942	6.430172	3.670249	5.341253	5.519684	4.647699
RWDD4	12.66825	17.19783	17.13208	17.69527	17.57241	14.16635	13.75917	16.21418
RXFP2	0.03252	0.010982	0	0.033326	0.010842	0	0	0.010994
RXFP3	0.020371	0.010319	0.010058	0.031313	0	0	0.010338	0
RXFP4	0	0	0	0	0	0	0	0
RXRA	41.68146	41.62578	35.1583	45.43497	38.53784	37.83679	39.03722	39.97909
RXRB	12.99257	12.0945	10.60543	13.01962	12.22706	11.69114	12.45753	11.72849
RXRG	1.234816	2.77108	1.311215	2.068185	3.240151	2.165664	2.264095	2.127724
RXYLT1	5.484363	6.009208	5.823503	5.532122	6.465164	5.314112	6.107586	5.440377
RYBP	24.06129	23.92032	22.21845	21.42325	22.66229	19.59319	20.29125	24.11569
RYK	31.19656	34.56904	34.04267	30.04116	34.43766	34.68807	31.01143	30.31571
RYR1	0.052927	0.066565	0.048663	0.099124	0.034681	0.056963	0.111146	0.059233
RYR2	0.221469	0.18611	0.183105	0.153062	0.250687	0.171125	0.268355	0.322131
RYR3	0.515182	0.22756	0.265824	0.454871	0.372619	0.400244	0.26055	0.493587
S100A1	45.60505	62.86715	39.4072	49.17678	24.02052	25.49877	32.10834	21.98973
S100A11	547.7449	579.936	642.3177	548.1538	595.1225	522.549	527.2976	638.2124
S100A13	20.82756	28.97643	26.16328	26.40645	26.47415	34.66072	24.91251	22.89397
S100A14	329.0836	348.5712	338.4626	361.733	345.3657	351.5019	321.7611	324.5715
S100A16	151.3255	139.5584	149.8225	122.8185	146.0984	157.7386	147.8284	138.8766
S100A2	701.6594	524.3635	709.3203	606.7565	662.0725	709.01	531.5216	575.7378
S100A3	362.3946	323.297	407.889	345.6571	312.1846	308.018	355.0134	311.9666
S100A4	134.3935	236.3502	263.4603	265.511	195.6764	253.0735	260.9009	246.626
S100A5	1.046923	1.342063	1.687964	1.029066	1.474429	2.086638	0.780705	1.603575
S100A6	0.056544	0.057286	0	0.115889	0	0	0	0.172046
S100A8	3.72388	11.56166	6.050048	3.015967	16.03878	17.59762	30.60369	9.320395
S100A9	5.797481	7.818457	6.293977	5.665672	9.753315	8.697646	8.066754	8.255381
S100B	3.489463	6.716991	5.513574	6.154309	5.106295	4.585783	2.963931	7.823357

S100G	0.437331	0.332303	0.10797	0.784286	0.601407	0.330239	0.110973	0.499001
S100PBP	8.185853	9.398442	7.500346	7.056501	7.145933	8.022117	7.985707	8.993865
S100Z	0.055978	0	0.02764	0.401555	0	0	0	0
S1PR1	8.000481	7.972291	6.349073	8.423189	11.41374	11.71714	11.08783	11.40965
S1PR2	7.729655	7.497861	5.968624	8.69006	9.539957	7.399547	7.605642	8.069045
S1PR3	13.69176	12.23522	9.904126	11.32669	7.953642	14.10471	8.715899	11.064
S1PR4	0.501041	0.355915	0.244555	0.442631	0.33407	0.237736	0.362419	0.794386
S1PR5	11.67608	9.992967	11.62618	12.19974	11.04399	12.34054	10.72594	11.55673
SAAL1	4.645676	4.977197	3.910307	4.27005	4.904148	4.760856	4.771441	5.174119
SAC3D1	0.621981	0.822688	0.597169	0.956082	0.881254	1.200275	1.017112	0.753498
SACM1L	17.17623	17.76418	16.44751	17.47414	15.40459	16.1579	16.75484	17.07359
SACS	0.612251	0.646602	0.624777	0.549465	0.656864	0.468863	0.668514	0.758333
SAE1	80.78848	74.6931	68.88947	72.60559	69.86446	76.33054	78.04409	74.01806
SAFB	32.96557	31.413	26.89311	29.01115	30.57905	30.01197	29.97061	32.60636
SAFB2	13.77385	14.58078	11.79731	12.55876	15.09138	12.39225	13.77433	16.02064
SAG	0.012798	0.012966	0.012639	0	0	0	0	0
SALL1	0	0	0	0	0	0.005317	0	0
SALL2	0.682236	0.614391	0.524016	0.663282	0.530697	0.575349	0.828596	0.555924
SALL3	0.008665	0	0	0.00888	0.004333	0.004362	0.026386	0.017577
SALL4	0.184785	0.128707	0.279427	0.094681	0.20791	0.261629	0.064473	0.117135
SAMD1	26.12901	24.6753	26.90562	24.89541	25.57427	25.14633	24.69582	23.39431
SAMD10	0.167349	0.169546	0.13772	0.185786	0.195266	0.042123	0.212324	0.155587
SAMD11	14.17444	13.24113	11.24901	12.48056	13.12269	12.26208	10.79681	10.45859
SAMD12	0.564216	0.489961	0.915377	1.011836	0.665055	0.872395	0.449963	0.592686
SAMD13	0.170517	0.135736	0.168392	0.099852	0.133995	0.12263	0.123625	0.172945
SAMD14	1.512324	1.947292	1.118271	2.023118	2.138389	1.207623	1.535009	1.934317
SAMD15	0.296324	0.51855	0.629602	0.37728	0.484957	0.669024	0.437482	0.264113
SAMD3	0.042365	0.114456	0.041837	0.043414	0.084741	0.042654	0.043001	0.085936
SAMD4A	4.779005	4.052562	5.529569	3.667253	4.019399	3.099159	4.014232	5.266859
SAMD4B	22.29822	18.69952	19.08634	21.06143	22.54004	20.42753	18.47922	18.68276
SAMD5	4.067159	3.665053	2.963317	4.885769	4.381388	5.273951	3.565717	5.196088
SAMD8	3.554948	4.141353	4.692031	3.826645	4.097922	3.506089	4.483303	4.499576
SAMHD1	7.630379	9.134842	9.58201	10.33252	11.41706	7.856957	9.144993	14.1127
SAMM50	23.56421	29.42324	26.85069	28.79173	25.7267	27.92541	30.62761	27.4008
SAMSN1	1.897317	3.019547	1.239282	2.028517	2.241216	1.195804	2.494412	3.613795
SAP130	8.464409	7.582249	6.368068	7.287356	7.264037	6.88869	7.687389	7.632583
SAP18	71.36637	79.71971	79.5238	73.32545	75.5365	78.7681	74.25049	67.05372
SAP25	1.963773	1.136883	1.108174	1.87973	2.050358	1.47746	1.423736	2.407584
SAP30	1.375739	2.03233	1.740075	2.111252	1.59281	2.708864	2.26309	1.876469
SAP30BP	18.04993	17.54807	16.68395	17.64633	18.78153	16.63715	17.75136	18.97817
SAP30L	8.697121	9.078689	8.889877	9.257674	9.144309	8.467859	8.818339	9.582549
SAPCD1	0.267697	0.180807	0.293734	0.274328	0.505714	0.838523	0.573614	0.331842
SAPCD2	7.235564	7.451224	6.063008	7.789194	7.951412	7.620856	6.771726	7.080791
SAR1A	33.745	38.20738	36.53953	38.50425	40.94726	40.94196	38.72753	36.10087
SAR1B	14.33267	16.84056	15.42886	15.82155	16.54771	16.27179	15.7227	15.35757
SARAF	15.55573	34.41473	21.95541	35.61025	27.32363	32.22218	23.72604	19.86284
SARDH	1.799448	3.215966	1.970037	2.928333	2.089517	2.11709	2.510499	1.913925
SARM1	1.784317	1.676829	1.269916	1.670884	2.301451	1.486764	1.904758	2.396316
SARNP	28.49367	31.64549	28.8968	31.42483	31.37122	36.44014	32.66145	31.32426
SARS	144.5866	130.8558	148.4958	155.1905	157.7486	158.631	146.1978	131.6886
SARS2	4.070467	4.962386	4.472992	5.258452	4.545065	4.469468	4.780655	6.043813
SART1	22.75909	23.78287	21.19243	23.75161	21.01111	21.4734	22.02759	21.79882
SART3	12.60666	12.68996	10.16418	11.41673	12.17783	11.89517	12.54251	12.15979
SASH1	16.17572	17.8861	15.22345	15.9946	17.08246	15.59189	17.5611	18.74506
SASH3	1.532957	2.26276	1.403576	2.257561	2.386037	1.124353	1.689908	2.491767
SASS6	5.118355	5.833719	5.308761	5.214994	4.714496	5.57536	5.269773	4.945083
SAT1	153.0217	162.9592	200.9526	154.6604	148.2209	161.9711	190.4832	158.201
SAT2	7.792604	7.451341	9.251901	9.720335	7.85199	10.51989	8.057611	7.844334

SATB1	6.919559	8.568232	6.911499	8.070021	8.153018	8.105201	9.221019	11.16927
SATB2	0.956202	1.078214	0.938953	1.029712	1.102206	0.897464	1.069244	0.767085
SAV1	19.04992	20.16411	18.02636	20.09288	19.09788	17.5655	20.66319	19.57488
SAXO1	0.015804	0	0	0.064783	0	0.159121	0.016041	0
SAXO2	0.333734	0.3306	0.190422	0.334401	0.155763	0.283745	0.210772	0.165482
SAYSD1	2.585666	2.322532	2.211233	3.605796	3.012557	3.059583	3.219687	2.595438
SBDS	101.4953	116.2077	117.3929	110.2148	110.1404	124.0151	107.7058	102.6595
SBF1	13.68908	12.26202	12.13428	13.08436	12.19461	12.39394	12.88665	12.97719
SBF2	9.351011	9.001759	7.85433	9.028239	9.897046	8.620546	8.663792	9.259314
SBK1	0.997263	1.343715	0.944843	1.219096	1.042958	0.698266	1.048189	0.888236
SBK2	0	0.015966	0	0	0	0	0	0.015984
SBNO1	10.86013	9.857105	8.927823	9.108111	10.18828	8.636263	9.258815	10.73736
SBNO2	7.64339	6.793922	5.905074	7.620673	8.552412	6.028933	6.472178	7.139732
SBSN	744.4238	555.7637	600.4219	607.994	552.8801	557.7577	602.0266	531.8444
SBSPON	2.002502	2.965739	2.143604	2.921507	3.92908	3.232047	2.20321	3.589149
SC5	0	0	0	0	0.048853	0.04918	0	0
SC5D	30.60691	32.43953	49.9061	35.298	32.82218	42.46041	35.20164	30.90012
SCAF1	9.059163	8.471627	7.327023	9.512441	9.196539	7.738908	8.338026	9.011325
SCAF11	17.44182	18.48892	16.49711	16.63629	18.05359	17.59109	17.72622	18.57367
SCAF4	8.955967	7.951846	6.708884	7.314246	8.424692	7.243129	6.479414	7.77422
SCAF8	12.14758	12.3295	11.59722	11.6386	11.77171	10.35754	11.02291	12.1674
SCAI	2.00682	1.844653	0.99747	1.498134	1.874161	1.445147	1.740153	2.251059
SCAMP1	6.831779	8.077391	7.643889	7.008222	7.156685	7.644299	7.005743	7.0719
SCAMP2	36.53993	36.21237	35.92729	38.30497	35.63104	35.63454	36.646	35.28251
SCAMP3	18.24111	18.02219	18.87849	19.02195	19.19213	17.96903	18.57398	19.28522
SCAMP4	20.19797	17.85795	17.16575	19.53886	20.6892	17.90237	18.75229	17.56459
SCAMP5	0.357179	0.569748	0.487816	0.420541	0.646046	0.244847	0.34711	0.832436
SCAND1	49.78297	57.85999	52.85841	58.33176	56.39445	59.62207	56.818	55.75454
SCAP	7.938789	7.092184	6.606811	8.919673	7.315426	7.672653	8.045606	6.805836
SCAPER	4.69283	4.815139	3.403263	4.66781	4.723407	4.356794	4.598973	4.133528
SCARA3	9.966375	8.727525	7.44374	10.25352	9.60606	8.633751	8.624019	8.968284
SCARA5	17.5117	21.56681	34.07622	26.35788	23.71405	22.07264	30.65892	39.33647
SCARB1	36.25849	35.28313	30.66914	41.36219	38.46041	39.29326	32.94909	30.99909
SCARB2	55.53475	55.08629	53.93583	58.25486	50.80565	53.08981	55.53072	55.8881
SCARF1	1.937403	2.166081	1.647217	2.390695	2.981005	2.169941	2.199181	2.720746
SCARF2	3.480518	4.148466	3.369755	4.537092	4.120852	3.435583	3.714557	4.213583
SCCPDH	18.29958	29.77432	26.77172	22.63183	28.60596	32.64898	25.71691	21.8179
SCD	80.1709	91.83686	136.708	174.8592	105.3048	115.9117	119.793	72.97396
SCD5	3.482271	3.57221	3.686826	3.188184	3.908509	2.813641	4.664661	4.140782
SCEL	38.07793	30.60799	36.35884	31.60874	35.67146	38.88364	33.58776	30.03022
SCFD1	20.56403	21.82667	23.06094	20.40868	24.23833	22.06629	22.90371	22.45253
SCFD2	7.23429	8.447118	7.92686	7.302005	6.51248	8.073888	8.021087	6.588573
SCG2	0.022231	0.022523	0	0.011391	0	0	0.022565	0.011274
SCG3	0.229717	0.073494	0.167156	0.185848	0.25393	0.194767	0.122717	0.171675
SCG5	0.300152	0.405455	0.296412	1.255979	0.85054	0.604405	0.35543	0.532743
SCGB1A1	0	0	0	0	0	0	0	0
SCGB1C1	0.474393	0.411959	0.267704	0.173623	0.305006	0.341167	0.378328	0.171838
SCGB2A2	1.170861	9.885232	1.321454	2.514001	2.899651	7.746796	2.490032	1.300627
SCGB3A2	0.343425	0	0.169573	0.234621	0.171735	0.4034	0.058096	0.058052
SCGN	0.019014	0	0	0	0	0	0	0
SCIMP	0.313663	0.307187	0.258129	0.450005	0.564666	0.410547	0.541225	0.64686
SCIN	14.84659	11.00686	12.85795	12.20428	16.00664	12.46458	9.670336	11.8592
SCLT1	1.890659	2.215938	2.599297	2.564326	2.641703	2.668737	2.060123	2.678963
SCMH1	4.867017	4.459443	4.94951	4.065108	5.317789	4.93867	4.529645	4.309593
SCML1	0.498189	0.587319	0.456201	0.547659	0.652259	0.392154	0.496468	0.385851
SCML2	2.160592	2.060605	1.591565	1.875142	1.632971	1.651	1.342945	2.062866
SCML4	0.115139	0.339345	0.124041	0.15017	0.20937	0.137003	0.308101	0.467111
SCN10A	0	0	0	0	0	0	0.004405	0

SCN11A	0	0.004866	0	0.039379	0.004804	0.029017	0.014626	0.019487
SCN1B	1.845742	2.553759	1.795539	2.187885	1.956189	2.094112	3.103739	2.640383
SCN2A	0.028366	0.060351	0.008404	0.043604	0.068088	0.01428	0.057584	0.034524
SCN2B	1.598485	1.492447	1.392854	1.284769	2.351018	1.451621	2.417787	2.861013
SCN3A	0.186871	0.178806	0.109359	0.06738	0.186895	0.031358	0.151037	0.084236
SCN3B	0.497625	0.307077	0.478022	0.245704	0.637948	0.227739	0.743859	0.724946
SCN4A	0.025589	0.015555	0.010108	0.05769	0.015355	0.015458	0.005195	0.005191
SCN4B	2.22598	1.390275	1.161572	1.646034	1.170055	1.598119	1.899924	1.821528
SCN5A	0.115494	0.451324	0.065174	0.209657	0.075906	0.169441	0.482303	0.137219
SCN7A	0.123555	0.015647	0.186837	0.502507	0.370714	0.186599	0.105814	0.031329
SCN8A	0.76356	0.790663	0.604188	0.54551	0.713069	0.703297	0.422957	0.549674
SCN9A	0.174199	0.18729	0.165007	0.342455	0.241777	0.103802	0.22733	0.685081
SCNM1	14.43353	12.63486	13.43773	14.84397	14.95186	13.62225	15.48867	12.28209
SCNN1A	10.02178	8.27921	7.769531	8.638333	7.962253	10.15311	7.359722	6.80796
SCNN1B	4.856369	3.97562	3.575484	3.576977	4.889522	4.136468	3.751925	3.122417
SCNN1D	0.60096	0.394623	0.450599	0.684274	0.411822	0.302533	0.304987	0.225746
SCNN1G	2.607892	2.305543	2.214514	1.889468	2.359036	1.647339	2.571141	1.844773
SCOC	15.87621	18.75871	23.53694	16.02844	22.72617	22.64165	17.65284	17.59979
SCP2	22.00764	32.76996	33.51431	33.181	40.12494	33.75572	38.6893	33.33271
SCP2D1	0.022214	0.011253	0	0.034146	0.033325	0.033548	0.06764	0
SCPEP1	24.63682	33.26069	30.35166	36.0863	33.9324	31.09007	34.29027	33.67823
SCRG1	0.378719	0.420232	0.356191	0.462024	0.234476	0.472094	0.842019	0.310947
SCRIB	9.101903	8.375057	7.236861	9.149484	8.723332	8.475957	7.597756	8.587802
SCRN1	3.34515	2.518807	2.849466	2.244014	3.787223	2.765051	3.450586	3.454122
SCRN2	1.708551	1.463459	0.874308	1.257444	1.289346	2.079905	1.765709	1.181504
SCRN3	6.234367	9.885628	8.277897	6.147201	14.78889	7.041797	9.093008	7.518665
SCRT1	0	0	0	0.007683	0	0	0	0
SCT	0.033006	0.033439	0.097784	0.135295	0.03301	0.099695	0.134005	0.301284
SCTR	0.051672	0.05235	0.051028	0.264761	0.155037	0.069367	0.052447	0.139754
SCUBE1	0.10141	0.102741	0.135492	0.018339	0.310235	0.090091	0.199808	0.266209
SCUBE2	4.243597	4.616289	2.665067	4.318652	4.98736	3.672063	3.870571	4.294091
SCUBE3	1.088979	1.411538	1.083318	1.538539	1.141173	1.451142	1.385706	1.64048
SCX	0.055556	0.101313	0.153619	0.034159	0.011113	0.100684	0.056389	0.045078
SCYL1	20.70985	21.77951	19.85275	22.0626	22.40487	22.27642	21.64701	21.14499
SCYL2	11.47485	12.59551	10.46096	11.99148	11.22937	10.72113	10.49097	11.46953
SCYL3	11.67538	10.90569	10.46523	10.28595	10.98313	10.88845	10.29816	10.65489
SDAD1	16.71689	20.95553	15.25672	19.73847	17.02005	16.91373	17.78224	18.89732
SDC1	122.1916	107.1494	105.3068	117.7564	120.6372	127.6149	114.3079	113.6021
SDC2	80.82059	70.10683	75.10267	66.04133	62.10534	69.47862	80.16544	65.47219
SDC3	6.994576	6.789273	6.135164	6.416547	9.320065	5.347382	8.742079	9.638788
SDC4	65.65304	75.90605	76.02225	83.71868	69.8807	71.43634	83.55992	71.10594
SDCBP	64.14296	75.45781	64.03674	70.65512	73.3832	59.75761	67.89943	77.88114
SDCBP2	2.489243	1.816377	2.194845	2.399063	3.230506	2.49133	2.616817	2.179038
SDCCAG8	0.89395	1.175231	1.040457	0.927	1.426248	0.857198	0.939765	1.57589
SDE2	18.61984	20.49784	17.48269	19.09845	17.2642	19.2939	21.46611	17.88643
SDF2	25.41347	26.35588	25.66913	29.93622	28.59385	32.19997	29.69438	24.42553
SDF2L1	5.018747	6.225391	5.051524	4.648538	6.467298	7.061289	6.726729	5.090192
SDF4	49.28776	47.24977	44.24728	46.92123	47.05274	45.52341	50.5916	43.86723
SDHA	33.09736	36.51286	34.95153	36.94466	40.25875	37.10376	37.51231	34.52353
SDHAF1	8.096039	5.910858	7.208336	5.320347	6.991769	7.323274	6.469635	6.28229
SDHAF2	22.42736	25.25393	20.14659	22.12911	23.60132	22.62592	21.50673	21.71892
SDHAF3	2.405515	2.817879	3.043663	3.671972	3.132585	3.027435	3.30633	2.948042
SDHAF4	5.408534	13.80153	7.243925	7.135976	6.761542	7.113162	5.626904	8.194008
SDHB	93.20862	91.88415	106.3823	93.84132	95.9402	93.98947	101.3095	92.9181
SDHC	90.23724	89.18854	104.687	96.85814	99.43002	101.152	87.46411	87.24632
SDHD	12.92435	14.94091	13.81125	15.92128	17.10771	15.60605	14.58194	13.82585
SDK1	3.656656	3.536253	3.040921	4.047549	3.849609	2.748016	4.164345	4.547163
SDK2	2.007729	1.503449	1.257807	1.817559	1.535754	1.374263	1.377357	1.714364

SDR16C5	113.9125	112.2416	160.5808	165.0221	200.4013	185.0624	108.7985	111.1791
SDR39U1	8.143493	7.011707	5.950667	7.360789	5.917351	8.155163	6.093975	6.554393
SDR42E1	4.379348	5.26746	5.792704	6.140876	4.606576	5.59715	5.114838	3.028733
SDR42E2	1.195997	1.168675	1.586441	1.000804	1.125374	1.717186	1.14929	0.70341
SDR9C7	16.53969	14.17118	14.10253	23.89491	21.71637	16.6948	12.86686	12.27725
SDS	4.608515	2.23535	12.03667	7.749554	9.413975	4.550423	6.266957	17.63172
SDSL	0.032793	0.099671	0.080962	0.11762	0.213185	0.13207	0.31621	0.149671
SEC11A	51.23674	53.23838	55.23537	56.70448	55.13381	58.07567	52.05202	51.12571
SEC11C	35.14918	35.19398	30.71909	36.05495	34.29715	37.2519	38.70161	34.7809
SEC13	37.11293	36.82797	33.53866	40.43827	34.29579	34.75372	39.4465	35.59418
SEC14L1	15.39219	14.48769	13.2078	13.38325	16.25507	13.94108	13.76296	17.52634
SEC14L2	1.933855	2.193053	1.453931	3.718853	1.76696	1.89899	2.318278	1.872597
SEC14L3	0	0	0	0.013924	0	0.02736	0.013791	0
SEC14L4	5.697259	3.373679	6.615932	6.816836	6.479299	6.149312	4.341043	1.352553
SEC14L5	0.048466	0.018413	0.149569	0.0745	0.151476	0.067096	0.122983	0.073734
SEC16A	10.01434	8.783998	7.437909	9.164021	10.58349	9.059019	9.114873	9.293108
SEC22A	5.044872	5.529425	5.112029	5.948683	4.429048	5.272146	5.0841	4.861765
SEC22B	14.95802	17.1605	17.22355	17.38118	19.01986	17.79006	16.34967	16.72358
SEC22C	2.770931	2.985815	2.695461	2.740431	2.380821	2.824647	2.893152	3.255411
SEC23A	8.655629	7.526858	6.359491	7.223895	8.219249	5.90611	8.093982	7.636943
SEC23B	16.66257	20.4065	18.89042	19.3177	19.47207	20.1082	20.4989	18.53699
SEC23IP	12.44157	11.54413	9.810854	10.90196	12.26866	11.07421	11.68359	10.91377
SEC24A	6.74157	6.321894	5.627927	6.463369	6.805639	6.696169	6.21735	7.025803
SEC24B	17.38674	17.50937	16.17149	18.23192	18.21645	15.45077	15.73785	17.16185
SEC24C	29.9945	29.55195	26.60797	29.00826	29.8822	26.01976	29.3026	28.40001
SEC24D	10.73944	10.72631	9.289754	11.68078	11.87068	10.12744	12.56905	13.19124
SEC31A	27.07579	26.82965	26.15777	27.41302	27.37047	26.3469	27.27913	26.70855
SEC31B	3.214007	2.640152	3.222979	3.56067	3.444024	3.467104	3.180347	3.184245
SEC61A1	50.72646	49.64871	49.84785	57.11822	53.10514	52.98521	55.4333	53.28442
SEC61A2	9.987172	9.633122	8.444119	10.00089	10.07968	9.676601	9.164941	9.921209
SEC61B	101.4961	109.6069	127.5926	110.5772	116.5895	111.2034	118.8491	110.4918
SEC62	41.33603	45.17949	44.16264	44.65293	44.94549	45.56288	45.15159	41.34319
SEC63	19.71564	20.11049	18.79292	21.99844	20.15832	21.8279	20.00667	20.64468
SECISBP2	2.340207	0.821822	1.623405	1.405066	1.665639	1.821353	2.010999	1.652728
SECISBP2L	22.20651	23.52259	21.70656	24.92696	26.60842	25.98009	23.76743	21.23137
SEH1L	30.86649	33.72025	32.91845	32.73679	29.43882	31.15702	35.12611	31.69256
SEL1L	22.98712	22.12067	22.29011	23.53895	21.45542	20.23549	24.76325	23.80125
SEL1L3	4.396664	4.316281	5.088685	2.752904	3.913537	4.581635	2.896237	3.594158
SELE	7.178616	8.930882	9.054302	16.86643	22.05943	15.972	11.43911	10.90235
SELENBP1	129.6925	114.7899	142.0668	141.359	146.3515	178.1605	132.3847	131.5864
SELENOF	90.37607	109.9325	119.7973	105.4046	109.2963	121.1383	105.7826	98.62305
SELENOH	28.05307	35.44562	31.2059	36.04954	32.01914	31.33297	30.67913	30.20234
SELENOI	16.21686	15.65131	14.36682	16.10214	14.83915	13.8157	15.45389	17.10127
SELENOK	45.90983	47.05767	56.18759	43.28327	51.17233	60.75995	40.97452	39.72338
SELENOM	16.18935	20.60986	20.53221	22.24947	27.75676	20.5769	20.38454	19.12454
SELENON	24.49319	22.8814	22.69031	25.9244	24.19727	23.74291	29.29069	26.95054
SELENOO	7.542063	7.665575	6.527537	9.937135	7.821513	8.495554	9.117422	8.668534
SELENOP	274.2711	263.8265	289.011	203.1276	228.6308	266.7556	234.9211	289.2443
SELENOS	36.81256	43.60971	42.00108	40.26533	42.24609	57.37281	35.21084	36.4757
SELENOT	33.92928	37.57299	39.08547	34.00349	36.55098	35.46595	37.84494	33.87301
SELENOV	0.712005	0.323364	0.48492	0.20128	0.540212	0.395514	0.423643	0.199211
SELENOW	112.1574	129.9576	114.4698	132.3038	132.1053	105.0412	105.9516	127.8315
SELL	0.512414	0.588357	1.214469	1.213579	1.469109	0.710815	1.028642	2.367559
SELP	2.568417	2.101717	2.43886	3.171943	7.530607	4.774087	5.336436	6.267539
SELPLG	1.178701	1.275131	1.058793	1.044115	1.451922	0.670482	0.892217	2.09378
SEM1	115.1335	146.8877	162.9757	138.9781	144.7846	153.9718	128.5354	132.2163
SEMA3A	0.397859	0.946997	0.525447	0.658238	0.867733	0.670846	0.68602	1.050132
SEMA3B	5.3841	4.768784	6.380416	7.749532	7.359222	6.349003	5.241299	5.61795

SEMA3C	4.01618	4.194612	7.219262	5.422038	5.2014	3.19741	5.633708	5.217553
SEMA3D	7.980214	9.487066	7.743221	6.197881	6.803833	7.021342	7.324588	7.095763
SEMA3E	5.625916	4.073282	4.183039	5.007441	4.587446	4.919784	4.18534	4.571449
SEMA3F	14.67504	13.26575	14.12684	15.48086	14.83992	15.42715	11.94922	11.66326
SEMA3G	14.85164	13.12585	11.66388	18.91888	16.5679	15.21533	12.99646	11.08843
SEMA4A	11.77171	12.95145	11.61649	12.56852	11.56562	12.9581	12.14142	11.99192
SEMA4B	11.41873	11.3303	10.05515	12.19918	9.932923	8.784882	9.726367	11.3889
SEMA4C	8.983321	8.617691	7.928755	9.62393	9.407915	7.486865	7.258516	9.314196
SEMA4D	6.976838	7.124764	6.166013	5.91557	5.855149	5.706583	6.951952	7.291895
SEMA4F	1.659517	1.510433	1.225798	1.590014	0.870347	1.093526	1.588549	1.525774
SEMA4G	1.497389	1.209792	0.929666	1.1978	1.150042	1.125943	1.276161	1.018878
SEMA5A	11.20548	10.37749	9.770803	10.23136	9.606329	10.54362	10.02589	12.12543
SEMA5B	0.483534	0.307745	0.189779	0.616212	0.440197	0.405698	0.453034	0.377244
SEMA6A	8.016629	6.457504	5.593198	6.225651	6.707221	5.563446	6.889891	7.073324
SEMA6B	0.656406	0.95098	0.674156	0.881192	0.997867	0.766633	0.393089	0.559231
SEMA6C	0.587469	0.643066	0.49346	0.567422	0.587545	0.509898	0.651111	0.808139
SEMA6D	1.979406	1.981956	1.543698	2.132702	2.382069	2.132619	1.624176	2.50948
SEMA7A	4.671776	4.094163	3.998969	4.268781	5.460792	3.868222	4.59867	4.965517
SENP1	8.372776	8.409795	7.836383	8.782854	8.529707	8.375666	8.145536	9.215335
SENP2	11.33931	11.88312	11.16559	10.68233	10.76203	11.82997	11.00953	11.52986
SENP3	15.81827	14.39561	14.33064	16.31003	16.36651	16.32891	16.19412	15.05433
SENP5	9.106382	9.706513	8.471307	8.820748	8.838539	9.429568	9.362114	9.533633
SENP7	4.633901	4.160648	3.693652	4.290794	4.94581	3.836575	3.92885	5.331269
SENP8	6.01387	7.173558	6.281314	6.668434	6.761477	6.095231	5.251402	8.195755
SEPHS1	14.85689	15.5803	13.5502	14.76798	14.03425	13.1627	14.89188	14.06155
SEPHS2	32.78906	34.47553	38.5511	32.31809	33.18618	40.55255	35.05023	30.25521
SEPSECS	3.403844	4.024793	3.637835	4.080312	3.630032	3.908891	3.665678	4.038366
1-Sep	1.60974	1.733713	1.346217	1.501	2.523702	1.109693	1.957719	2.735801
10-Sep	33.94127	36.53829	31.16718	30.78071	29.65959	32.47599	34.79837	33.73866
11-Sep	21.7974	19.31852	19.10635	18.46938	18.41435	18.87562	21.66921	20.71919
14-Sep	0	0	0	0	0.022593	0	0	0
2-Sep	127.3327	126.5977	115.6356	124.5137	120.0529	125.4684	126.4287	123.1281
4-Sep	43.03102	38.94793	31.97868	36.12851	36.82627	39.84444	36.88467	35.41871
5-Sep	11.83891	9.23805	9.312133	9.663203	11.28704	8.762843	10.0508	9.084502
7-Sep	108.2594	125.5177	121.857	122.0639	118.2855	128.0797	114.8979	122.0042
8-Sep	23.50681	23.44551	19.49392	22.88463	23.11558	22.75598	23.08139	22.29397
9-Sep	36.12045	40.10251	33.39076	37.89042	34.75145	34.10116	37.92162	36.56441
SERAC1	4.019633	3.493796	2.922932	3.80407	3.097494	3.461039	3.171419	3.648005
SERBP1	201.8712	214.2146	218.0319	202.9049	202.4366	234.8175	212.3405	198.0276
SERF2	367.2198	445.1434	447.9833	415.084	453.9824	451.8527	384.1306	401.8088
SERGEF	4.051866	3.293311	2.215453	2.869811	2.983963	3.111519	4.337252	4.264333
SERHL2	7.876098	10.53289	10.18503	7.765336	6.732861	6.26045	7.051761	5.785152
SERINC1	75.48506	85.40706	85.26222	83.23769	82.45493	83.85099	86.25128	83.80138
SERINC2	45.90537	32.69283	47.52425	35.81289	36.30769	43.46636	32.34396	38.4676
SERINC3	49.55175	47.13934	51.9607	54.47258	48.40966	51.56186	54.65538	50.0607
SERINC4	0.011178	0.022649	0	0.022909	0	0	0	0.022674
SERINC5	34.60887	34.1043	31.95451	34.44737	30.99474	30.97146	33.23601	34.03023
SERP1	31.24285	37.11745	32.50007	32.004	33.40592	35.79703	38.70166	31.20807
SERP2	0.152668	0.257786	0.42717	0.4172	0.483512	0.486752	0.180785	0.387104
SERPINA1	64.31837	57.16926	63.63225	99.00408	69.63163	50.01261	59.0528	50.90484
SERPINA12	4.220588	3.225739	5.118595	5.250864	4.813574	4.681819	3.607492	3.920194
SERPINA14	0	0	0	0	0	0	0	0
SERPINA5	0.012851	0.026039	0.025381	0	0.141378	0	0.026087	0.013034
SERPINB1	28.58903	13.09399	11.04001	16.2343	16.7984	24.51542	12.46786	16.85557
SERPINB10	9.384797	11.76778	9.153644	14.44282	10.64188	7.835275	9.743583	8.747539
SERPINB11	0.726991	0.122755	0.051281	0.319285	0.917512	0.139421	0.17569	0.12289
SERPINB12	48.96113	26.07749	16.44467	33.11948	34.79188	44.70578	40.83541	29.09427
SERPINB13	9.766037	12.88504	9.057622	17.32364	12.2834	17.22601	11.39187	8.219696

SERPINB5	259.9849	245.9614	270.5568	213.8464	252.1494	298.5375	274.1188	256.4758
SERPINB7	1.987658	1.879494	3.213325	2.640416	3.519345	1.497221	2.495688	2.15035
SERPINC1	0.943096	0.777334	0.836632	0.999218	0.671443	0.466722	0.665202	0.632277
SERPIND1	0	0	0	0	0	0.012798	0	0
SERPINE1	3.14854	1.761273	3.204686	2.553511	3.380771	1.730886	3.313403	4.17292
SERPINE2	4.349134	5.221881	4.934118	5.354707	4.910184	4.951036	4.574602	4.923401
SERPINE3	0.04634	0.082159	0.228811	0.154334	0.139037	0.198289	0.141104	0.117498
SERPINF1	55.74444	49.01171	76.58859	68.21074	81.50266	52.39666	69.16286	93.20862
SERPINF2	0.48314	0.578477	0.574712	1.136496	0.241601	0.464329	0.412371	0.868667
SERPING1	41.20885	42.56829	39.66197	49.64116	52.83257	51.14611	49.32921	45.87964
SERPINH1	91.43216	92.42918	91.07095	96.76227	101.9813	90.22829	84.8475	106.1691
SERPINI1	0.416773	0.811368	0.790879	0.535964	0.841827	0.625316	0.987057	0.80397
SERPINI2	0	0	0	0	0	0	0	0.0107
SERTAD1	28.44989	25.18243	24.9105	28.28115	27.67023	28.36592	25.90714	22.75682
SERTAD2	17.60929	16.13685	14.65275	16.61112	16.9509	15.33433	15.04613	15.96247
SERTAD3	20.8009	21.51045	19.67131	23.56418	21.3325	23.01364	19.34357	18.73304
SERTAD4	17.51455	15.00801	15.54161	17.93619	15.99758	16.32441	16.31998	17.39782
SERTM1	0.941737	0.811602	1.129289	1.936395	3.455522	1.760889	1.756554	1.600175
SERTM2	0	0	0.04038	0	0	0	0.020752	0
SESN1	17.50075	17.2502	14.50379	15.57489	14.86928	16.04463	17.80145	20.87863
SESN2	4.442492	4.523076	5.147285	5.581737	6.048729	5.284743	4.590978	4.505734
SESN3	28.61214	28.36857	27.81703	22.93375	23.75064	24.75011	25.37447	34.02885
SET	100.114	118.3278	114.8675	104.1889	105.7067	111.6736	112.8651	102.7945
SETBP1	6.603676	6.191149	5.274163	5.841542	5.358392	4.846887	5.337466	6.1405
SETD1A	4.50425	4.147526	3.77139	4.169601	4.60835	3.927716	3.937846	4.651629
SETD1B	3.811083	3.531373	2.956882	3.095038	3.538158	2.781993	3.055619	3.541849
SETD2	16.25156	15.71525	13.28983	14.16332	15.06255	13.83044	14.22591	16.42038
SETD3	9.824514	9.98143	9.974822	10.61973	9.867214	9.9959	8.969789	9.656268
SETD4	5.782885	4.374555	4.309773	5.183388	6.601053	4.952914	6.68355	3.628609
SETD5	20.47037	17.57047	15.71633	18.68995	18.52988	16.84506	17.17646	19.29777
SETD6	5.781799	5.783719	4.339992	5.775395	4.64356	5.262688	4.934897	6.382399
SETD7	12.31269	10.56671	10.55406	9.607795	11.09414	9.137854	9.802501	12.88135
SETD9	1.64147	3.134139	2.514654	2.480063	2.672995	2.140013	3.075862	2.433224
SETDB1	10.88604	10.22601	7.680589	10.715	9.560309	8.449164	9.103851	10.42416
SETDB2	2.044101	2.232359	1.721906	1.730838	2.012493	1.980143	2.093246	2.16093
SETMAR	3.353821	2.98083	2.724899	2.171494	2.439458	1.995342	1.763958	2.643944
SETX	7.887196	7.372334	6.830436	7.03924	7.567983	6.671714	7.380907	8.872238
SEZ6	0.436897	0.189699	0.130121	0.113707	0.194202	0.111716	0.147817	0.211008
SEZ6L	0.088826	0.026998	0.074562	0.072821	0.182117	0.040245	0.009016	0.004505
SEZ6L2	0.011946	0.006051	0	0.012242	0.005974	0.006014	0	0.036348
SF1	68.92129	63.33184	60.54567	61.88086	66.84982	61.03808	64.59861	64.78438
SF3A1	27.34679	32.10637	29.28172	36.5819	32.46959	28.68341	32.67294	34.2306
SF3A2	26.32083	24.5986	23.57753	26.50804	26.12984	23.10856	24.36467	24.54345
SF3A3	38.07814	41.22415	37.53033	36.31928	38.52877	39.38522	37.85773	39.67485
SF3B1	76.63757	79.73293	67.1605	78.30106	74.87206	69.19912	75.84297	77.2186
SF3B2	61.33796	63.69993	59.92867	62.22737	58.72336	60.96399	64.84155	60.6622
SF3B3	51.44625	50.4211	43.18251	45.64032	50.0291	43.86828	50.1234	49.38358
SF3B4	37.87084	36.85262	35.32417	36.03554	33.65544	34.13195	34.71609	31.62004
SF3B5	91.95158	86.29644	88.90114	74.92375	74.69228	86.12175	76.5313	73.56212
SF3B6	101.5787	128.5778	129.7823	109.7167	114.6271	135.4839	121.0131	112.6999
SFI1	3.047093	2.640344	1.920481	2.676489	2.685634	2.453189	2.920659	3.531967
SFMBT1	4.5751	4.409173	4.222617	4.448709	4.167633	4.261289	4.317947	4.116065
SFMBT2	1.181932	1.136935	0.816423	0.927735	1.48075	1.0824	1.103944	1.141371
SFN	921.95	858.3037	980.7229	935.9633	928.184	982.4577	885.7253	793.1365
SFPQ	46.18572	46.02385	38.46619	43.36189	44.31265	40.49078	43.01811	51.22098
SFR1	4.580429	5.282092	4.690116	7.39774	4.897681	6.099372	5.806063	6.379719
SFRP1	56.62848	46.72729	47.74312	50.85558	60.03655	65.23053	57.04897	72.5615
SFRP2	1.89757	4.101278	4.15387	2.333489	2.656942	3.470803	2.182839	5.003917

SFRP4	0.627873	0.664384	2.576649	0.972287	1.730361	1.784101	3.767096	3.452929
SFRP5	20.57636	21.30105	13.11442	19.46797	14.69455	18.37414	14.42381	15.25581
SFSWAP	9.704289	9.183827	7.596123	8.263133	8.98139	7.834456	8.212984	8.82672
SFT2D1	39.54341	45.48644	50.43638	49.22462	49.41075	49.79201	42.48713	45.03123
SFT2D2	27.63539	26.82172	28.25853	25.03383	25.81541	25.34909	28.83254	26.58434
SFT2D3	1.63999	2.124131	1.841848	1.772882	1.78171	1.573491	1.586256	1.572016
SFTA2	0.2095	0.254699	0.041378	0.085876	0.041905	0	0.212642	0.59495
SFTPA1	0	0.015221	0	0.015396	0	0	0.015249	0.030475
SFTPB	0	0	0.0071	0	0	0.028955	0.021892	0.007292
SFTPC	0.597009	0.74716	0.762973	0.935689	0.878067	0.848593	0.605964	0.498653
SFTPD	0.207467	0.057324	0.037251	0.077311	0.018863	0.170905	0.248866	0.095646
SFXN1	23.73985	28.15509	22.54424	24.92208	26.72528	28.55567	26.48368	27.68191
SFXN2	4.122253	3.96279	4.649141	4.008345	4.122786	3.537285	4.219747	3.919628
SFXN3	5.421278	5.702864	5.569108	5.566208	6.003647	4.757725	6.113997	6.246327
SFXN4	2.820797	3.397028	2.277575	2.805828	2.756103	2.42924	2.358914	2.483091
SFXN5	10.89673	8.719439	8.094523	12.89105	8.87282	9.7624	7.027619	8.32312
SGCA	1.900254	2.586979	1.075122	3.265825	2.039078	1.375138	2.61186	1.606088
SGCB	11.97321	13.36694	15.52302	11.5749	13.88659	11.85344	16.05299	15.3466
SGCD	2.004743	2.802925	1.702389	1.655034	2.12439	1.640202	2.323466	2.108061
SGCE	15.70446	16.13361	18.75548	16.63493	19.11201	14.92511	17.19139	17.20822
SGCG	0.017988	0.291583	0.071055	0.0553	0.161912	0.199219	0.127804	0.054732
SGCZ	0	0.004092	0.007977	0	0.008079	0.004066	0	0.004096
SGF29	11.27638	11.76109	11.0421	12.18822	11.20661	10.32563	10.55398	10.20894
SGIP1	0.508445	0.615141	0.50211	0.596919	0.646747	0.59144	0.636322	0.796055
SGK1	18.10669	16.18917	13.80367	15.91182	16.2663	15.9284	17.70675	18.35596
SGK2	0.025988	0.052658	0.051329	0.053264	0.116961	0.104662	0	0
SGMS1	25.43072	24.58296	23.72112	25.20833	30.6691	24.41207	27.40881	29.09363
SGMS2	2.223198	2.756881	2.725335	2.330944	2.1046	2.810921	2.579376	2.443837
SGO1	3.766455	3.934823	2.879009	3.168004	3.130965	3.821735	3.91232	3.780384
SGO2	3.285765	3.914959	3.162561	4.05007	3.735148	3.522547	3.37263	3.665794
SGPL1	17.38901	14.82683	13.1243	14.1073	13.28665	12.76111	14.91473	17.02753
SGPP1	8.361837	6.737579	8.57719	6.413633	7.298392	7.038663	7.562482	7.358151
SGPP2	6.393873	5.932862	5.431146	8.157588	6.18625	5.882475	6.721803	6.512171
SGSH	8.701549	8.865555	6.845372	10.74753	10.09576	8.216526	7.800833	11.28524
SGSM1	1.601972	1.342289	1.164121	1.884287	1.889362	1.668708	1.569756	1.124059
SGSM2	5.696228	6.131296	4.928681	6.456896	5.9993	5.436747	5.023616	5.386552
SGSM3	2.976821	3.114039	2.626622	3.808685	3.523321	2.615862	3.986915	3.930317
SGTA	28.82315	28.48451	28.40162	26.68829	29.96428	27.54425	26.80579	28.79772
SGTB	0.672876	0.662944	0.804706	0.670565	0.598875	0.671257	0.795753	0.720021
SH2B1	6.346483	6.554797	5.315688	6.26281	6.60002	5.934267	5.600683	6.305708
SH2B2	0.862967	1.199251	0.822048	1.252168	1.833088	0.676637	1.387507	0.906235
SH2B3	5.544728	4.863223	4.041177	5.281927	6.977368	4.764873	4.63443	5.327957
SH2D1A	0.537761	0.389156	0.17702	0.524839	0.179277	0.541434	0.129959	0.623333
SH2D1B	0.134166	0.115015	0.081535	0.169218	0.11354	0.10391	0.157129	0.167479
SH2D2A	0.482156	0.439636	0.22617	0.333517	0.687161	0.29127	0.562797	0.464569
SH2D3A	0.995705	1.141798	1.372296	1.244626	1.181869	1.652485	1.399349	1.709027
SH2D3C	2.195881	2.140431	1.478536	2.437796	2.828394	1.775405	1.908004	2.589895
SH2D4A	3.972708	5.344332	2.248767	2.979887	3.924069	3.702952	4.938527	3.123717
SH2D4B	0.042237	0.071319	0.041711	0.12985	0.014081	0.014175	0.085741	0.057118
SH2D5	0.347164	0.281376	0.159991	0.118588	0.084873	0.302931	0.148779	0.08607
SH2D6	0.026183	0.026526	0.038784	0.013416	0.052372	0	0.013288	0
SH2D7	0	0	0	0	0	0	0	0
SH3BGRL	30.20957	39.97148	40.25984	37.30423	36.90576	40.57487	38.05242	39.17275
SH3BGRL2	24.57275	30.33435	26.25735	26.68912	21.13077	29.0777	28.60285	27.22542
SH3BGRL3	234.1137	218.9136	259.1507	241.0702	286.5995	233.0122	230.6754	248.0783
SH3BP1	10.78882	8.761595	9.150366	9.58265	9.874166	7.420403	9.426427	9.981031
SH3BP2	8.040747	6.566767	6.400938	7.686398	8.164562	6.946215	7.762635	9.387832
SH3BP4	9.677552	11.40739	6.947501	9.882774	8.47246	8.63091	9.281755	10.76556

SH3BP5	49.77995	46.08388	52.73234	48.03479	56.10617	49.22156	43.0725	49.74253
SH3BP5L	18.07142	13.35053	14.94814	15.41172	14.88666	13.60319	14.84397	15.2368
SH3D19	25.76493	23.00592	21.92435	21.3674	24.9664	19.70178	22.14548	23.08887
SH3D21	13.20777	12.65563	10.75584	14.46807	12.94423	12.31	8.784717	14.83042
SH3GL1	81.4985	76.94549	79.74939	85.19655	81.69478	79.43742	74.20493	68.68649
SH3GL2	0.722442	0.998078	1.167448	0.874944	0.251793	0.319605	0.822161	0.666115
SH3GL3	19.99226	23.91469	32.88794	27.29866	23.30978	26.92674	23.78097	21.59308
SH3GLB1	25.73972	26.6225	24.35658	23.06989	26.70341	24.67125	23.71177	27.06201
SH3GLB2	33.02122	30.89233	28.38711	38.1943	29.98761	32.34115	29.93068	30.86012
SH3KBP1	13.65858	12.74283	14.14649	12.7975	14.67409	12.95688	13.03358	12.21724
SH3PXD2A	23.92114	19.96168	17.62137	19.12752	21.32666	19.66188	18.97262	22.32344
SH3PXD2B	5.752585	5.615619	4.87751	5.720931	5.86543	4.990995	5.409491	5.845478
SH3RF1	7.106575	6.991059	6.623339	7.148218	7.532195	7.909618	6.889912	7.074734
SH3RF2	11.44616	10.42547	11.78586	9.55917	8.265319	10.32664	9.075371	10.84889
SH3RF3	1.600412	1.914002	1.087316	1.541405	1.594602	1.774903	1.648846	1.897796
SH3TC1	0.814959	0.629392	0.679466	0.951519	0.928639	0.457343	1.023809	0.921412
SH3TC2	3.596835	3.039692	2.589648	2.796223	2.906193	2.473736	2.457837	2.815399
SH3YL1	44.13464	46.65551	58.78013	47.5592	43.21644	52.80697	50.42248	45.58798
SHANK1	0.140377	0.122825	0.113422	0.16347	0.185066	0.080304	0.161912	0.093838
SHANK2	0.414584	0.811193	0.350037	0.54485	0.411472	0.659581	0.343709	0.542459
SHANK3	2.200039	2.059278	1.830389	2.853083	3.539989	2.301319	2.221182	2.725021
SHARPIN	18.50739	15.00352	14.5442	14.24106	15.93536	15.87812	16.46995	14.77213
SHB	8.277268	7.386596	7.993377	9.378463	8.77158	7.81679	7.518968	7.652536
SHBG	1.35705	1.28112	1.233539	2.496876	1.619417	1.770007	1.252187	2.033272
SHC1	26.25597	24.70386	23.47247	28.38418	32.8027	26.93487	24.0043	28.5218
SHC2	0.9432	0.863474	0.606002	0.722011	0.977417	0.789462	0.611318	0.887473
SHC3	0.957475	0.707191	0.62833	0.727981	0.908175	0.783652	0.940488	0.701702
SHC4	0.775418	0.745159	0.681298	0.765411	1.020719	1.274403	0.787049	0.676584
SHCBP1	2.603428	2.54373	1.976276	2.174209	1.788351	2.024211	2.360364	2.734457
SHCBP1L	0.263787	0.801746	0.507289	0.156502	0.88866	0.68494	0.324111	0.295704
SHD	0.040311	0.08168	0.01327	0.151468	0.107509	0.094701	0.122747	0.068141
SHE	3.874047	4.726082	3.449241	4.814178	4.237051	3.668283	3.898713	3.704807
SHF	15.54651	11.75084	11.96497	14.99226	13.62424	12.1987	11.96605	11.57964
SHH	4.764113	3.86131	2.724835	4.699042	3.196339	3.597494	3.465495	3.36222
SHISA2	42.91998	35.49923	34.30531	38.91071	36.33851	35.27055	33.49089	41.60708
SHISA3	0.429188	0.854111	0.650897	0.612605	0.413913	0.709911	0.420067	0.886141
SHISA4	7.798508	5.438602	5.459509	7.772729	8.547415	7.771114	6.831172	7.828262
SHISA5	15.36931	13.40319	12.74663	15.89148	16.10764	14.16547	12.84419	14.25798
SHISA6	0.232795	0.489843	0.742737	1.431371	0.608928	0.576949	0.436223	0.56303
SHISA7	0.20759	0.143629	0.290004	0.342446	0.151915	0.214106	0.097643	0.159192
SHISA8	0.018306	0.018546	0.036155	0.093795	0.036616	0	0.03716	0.129963
SHISA9	0.019273	0.004881	0.009516	0.014813	0.009638	0.024256	0.024453	0.01466
SHISAL1	6.857894	7.628453	7.019581	6.15492	7.152979	6.290301	5.516917	5.276755
SHISAL2A	0	0	0	0	0.012337	0	0.01252	0
SHISAL2B	0.01415	0	0	0	0	0	0	0.028703
SHKBP1	28.64374	26.72314	27.13764	31.85008	26.29552	26.02016	29.03649	26.92458
SHLD1	0.639634	1.076564	0.336209	1.08894	0.815123	0.976393	0.900543	0.617349
SHLD2	2.470185	3.24482	2.86607	3.339871	3.447433	3.139558	3.956287	2.543453
SHMT1	7.401097	6.618958	6.96129	7.638776	7.170589	7.490494	6.8025	6.347479
SHMT2	31.69673	32.61355	32.83607	36.94466	32.9133	36.20313	31.37176	32.17183
SHOC1	0.010009	0.06084	0.093897	0.235901	0.110109	0.010077	0.350478	0.076133
SHOC2	26.68321	27.74633	27.10827	26.40866	27.20023	27.92506	27.36015	26.76087
SHOX	0.094879	0.160206	0.374785	0.064819	0.189782	0.159211	0.224704	0.096229
SHOX2	4.95716	5.44829	4.815865	5.290851	5.655831	6.468513	5.303982	5.499645
SHPK	3.458066	4.003943	3.791323	3.268904	3.839656	4.462248	4.713344	5.826403
SHPRH	7.200904	7.302679	6.136803	6.930707	6.706642	6.545678	6.602425	7.150577
SHQ1	5.73743	5.826871	5.252368	6.022603	5.137827	4.989542	7.792993	6.753561
SHROOM1	0.846247	1.386358	0.764579	1.254685	1.332561	0.942669	1.535128	1.570495

SHROOM2	3.316319	2.628186	2.430442	2.629185	2.699016	2.72667	2.599296	2.963568
SHROOM3	4.934374	5.630868	4.084689	4.98844	4.211598	4.411423	4.991512	4.95824
SHROOM4	3.391301	2.603748	2.682937	2.828885	4.309945	3.593681	3.153738	3.262215
SHTN1	13.90443	15.47515	14.02318	14.67011	14.0577	12.88121	15.59897	15.73351
SIAE	3.765894	4.200148	4.394172	4.804508	2.409615	2.862837	3.095359	2.685761
SIAH1	5.255748	5.488203	6.219207	5.664681	6.012615	5.565519	5.563878	5.400706
SIAH2	19.37798	17.88063	17.97237	21.6758	19.57137	20.50509	19.32678	19.74493
SIDT1	0.417146	0.354456	0.35215	0.324057	0.6258	0.23032	0.184385	0.320725
SIDT2	36.04005	26.2476	27.45699	28.20333	32.51519	32.86206	28.11646	30.59626
SIGIRR	3.849868	4.230669	4.047182	5.933755	5.822126	4.954619	3.813077	4.865098
SIGLEC1	0.429049	0.579574	0.39878	0.431056	0.513243	0.19905	0.824006	0.452222
SIGLEC11	0.609832	0.27696	0.3115	0.49564	0.778161	0.698687	0.234785	1.471634
SIGLEC15	0.068496	0.112767	0.084553	0.149159	0.068505	0.025861	0.121666	0.147626
SIGMAR1	13.0279	13.68103	12.82151	13.79258	13.4758	13.86558	13.96298	12.1575
SIK1	7.54241	7.02776	6.384369	7.480962	9.373437	7.099657	10.36702	8.762007
SIK2	9.001964	10.15902	8.377049	9.284805	9.713901	7.981989	8.507879	9.373671
SIK3	9.918145	9.764364	8.914664	9.044986	9.944529	8.301228	8.440756	9.444151
SIKE1	5.548429	4.889714	4.766236	4.887508	5.682174	5.337627	4.995201	6.95718
SIL1	11.08979	12.48676	11.4878	12.56075	11.67497	12.17687	12.67521	10.49047
SIM1	0.0179	0	0	0.007338	0	0.003605	0	0.003631
SIM2	0.224813	0.156587	0	0.043196	0	0.014147	0.014262	0.042752
SIN3A	10.33223	11.08155	9.00283	10.54882	9.889845	9.324736	10.48721	9.994723
SIN3B	9.147624	10.13176	7.712452	8.968626	8.044928	8.227928	8.526661	9.515305
SINHCAF	20.83593	22.41407	13.73079	14.78456	16.48173	17.43293	21.31186	18.69378
SIPA1	2.72482	3.365971	2.44696	3.184219	3.808867	2.591022	3.137687	3.854509
SIPA1L1	8.347094	7.858054	6.06438	7.516829	8.049575	6.910615	7.821567	7.978238
SIPA1L2	4.813618	3.34265	3.205505	3.666415	4.417504	3.364133	3.917949	4.642291
SIRPA	12.44103	12.43191	11.52669	13.18192	14.59205	11.26327	12.62764	16.74747
SIRPB2	0.226426	0.258997	0.187539	0.20958	0.13149	0.110309	0.24465	0.548195
SIRT1	12.55461	12.91941	11.58464	10.58412	11.494	10.62505	12.34904	12.38807
SIRT2	20.95657	22.79107	23.87507	25.03565	21.43948	24.78224	20.85525	19.09217
SIRT3	5.444367	5.867499	5.55695	6.19706	6.906835	6.180552	4.450496	5.577461
SIRT4	1.163461	1.513596	1.331754	1.300669	1.031383	1.397704	1.073557	1.086158
SIRT5	4.792737	5.302867	4.17253	5.600782	3.899858	5.111198	5.195338	5.543206
SIRT6	2.901993	2.97221	2.364704	3.006377	3.901544	2.95375	2.800661	3.023723
SIRT7	12.29643	12.68823	12.45765	13.8751	13.81442	15.40303	13.60431	11.62569
SIT1	1.345402	2.14195	1.186284	1.575689	1.730026	0.58054	1.316812	1.608225
SIVA1	17.13541	18.1256	19.67024	17.35615	17.25691	20.17459	17.4328	15.80674
SIX1	0.032736	1.094461	0.032328	0.10064	0.392881	0.03296	0.265815	0.166009
SIX2	0.1471	0.270965	0.132061	0.315192	0.414609	0.201962	0.24432	0.122068
SIX3	0.012608	0.012773	0	0.02584	0	0.025388	0	0
SIX4	0.040434	0.068274	0.09317	0.059851	0.062905	0.04071	0.04104	0.068349
SIX5	4.443327	3.982221	3.562876	3.813951	4.016603	3.613359	3.825771	4.52584
SKA1	6.060914	5.053803	4.287291	4.379136	3.780047	5.056697	4.821236	4.403187
SKA2	16.12364	12.83971	13.34651	14.42747	9.257986	11.53142	14.74262	10.38717
SKA3	2.876169	3.660456	3.298071	3.032668	2.508058	3.398397	2.943438	3.134088
SKAP1	4.197446	5.061644	4.420265	6.547291	6.259833	3.066743	5.014462	5.199058
SKAP2	8.452802	8.912041	7.867739	11.34279	11.81751	13.23612	9.049917	12.84833
SKI	32.17724	28.96578	27.54946	29.38759	29.95434	28.29493	29.99828	31.02525
SKIDA1	0.33689	0.319787	0.380648	0.323463	0.349075	0.326968	0.27109	0.35092
SKIL	7.366124	6.765273	6.580556	6.331885	8.157535	7.030947	6.439089	7.574304
SKOR1	0	0	0	0	0	0	0	0
SKP1	76.03596	87.07839	83.56142	78.27109	79.91672	82.14157	80.58248	79.84498
SKP2	12.84238	11.5071	10.43096	11.69821	10.58034	11.55949	11.39523	11.43655
SLA	0.840102	1.000195	0.78745	1.011693	1.172497	0.664248	1.09358	1.73782
SLA2	0.81447	0.936668	0.380423	0.823366	0.880622	0.43218	1.206517	1.205608
SLAIN1	3.145702	2.67822	2.105313	3.563461	4.728639	4.006688	2.106151	2.854137
SLAIN2	23.32707	22.77036	27.30727	24.13158	23.99381	24.85619	23.03144	23.00849

SLAMF1	0.857905	0.597551	0.873691	0.824209	1.179772	0.99873	1.006832	1.413942
SLAMF6	1.500412	2.008038	0.987811	2.050103	1.778496	1.585263	1.842543	2.029028
SLAMF7	0.425893	0.471934	0.35487	0.69558	1.011627	0.522603	0.972633	0.742424
SLAMF8	0.197803	0.300599	0.325564	0.253379	0.527544	0.016596	0.418272	0.367802
SLAMF9	0.193029	0.097781	0.158853	0.19781	0.643512	0.25913	0.032654	1.370436
SLBP	43.41074	44.92737	40.99088	43.49404	40.9626	37.47301	45.94229	42.75915
SLC10A1	0.004192	0.093432	0.045536	0.064436	0.071271	0.08019	0.127643	0.034012
SLC10A3	5.649694	5.922861	5.804332	6.933072	6.72707	6.439872	6.149169	5.690273
SLC10A4	0.068702	0.041762	0.013569	0.056323	0.041226	0.09684	0.083679	0.013936
SLC10A5	0.717863	1.250019	1.078146	1.172433	0.904923	1.837032	1.077766	1.023865
SLC10A6	0.612683	0.878098	0.885438	0.903505	0.762217	0.481459	1.243747	0.833592
SLC10A7	3.503106	3.627414	3.230393	3.205902	3.568991	3.071936	3.054602	3.776167
SLC11A1	0.081263	0.035284	0.1605	0.047586	0.116104	0.07013	0.106048	0.200161
SLC12A1	0.005881	0	0	0	0	0	0.035817	0
SLC12A2	13.13768	11.33905	9.032138	12.08767	10.87879	12.48353	11.05386	14.79242
SLC12A3	0.019879	0.006713	0.032718	0.061113	0.006627	0.120087	0.087433	0.100808
SLC12A4	23.98588	20.72502	18.92286	19.34137	19.50268	16.05438	19.79266	24.49043
SLC12A5	0.018877	0.014343	0.013981	0.014508	0.023599	0.03326	0.01437	0.023932
SLC12A6	5.144639	4.476976	3.985331	4.672257	5.128187	4.173466	4.467895	5.342855
SLC12A7	4.630292	4.611551	3.443288	5.543477	5.516703	4.18783	4.352671	5.315925
SLC12A8	0.116138	0.176493	0.066903	0.228111	0.067756	0.13642	0.14735	0.137423
SLC12A9	3.029233	2.484133	1.845437	2.872516	2.827253	2.312912	2.337717	3.337943
SLC13A1	0	0	0.012587	0.013061	0.17846	0.012833	0.051747	0
SLC13A2	0.255761	0.146458	0.406315	0.467215	0.455981	0.682957	0.406327	0.248124
SLC13A3	0.014612	0.051813	0	0.067382	0.029228	0.014712	0.014831	0.01482
SLC13A4	0.126291	0.103958	0.085743	0.161774	0.205249	0.174836	0.168243	0.056039
SLC13A5	0	0.007367	0.007181	0.014904	0.007273	0.021964	0	0.007375
SLC14A1	0.015	0.121571	0.170346	0.122969	0.225022	0.113265	0.076123	0.144524
SLC14A2	0.022961	0	0	0	0	0	0.005826	0.005822
SLC15A1	14.80608	8.503922	9.636288	11.7342	12.44788	10.79696	13.84572	12.1892
SLC15A3	2.714009	2.165908	2.665219	2.408333	2.593048	1.251783	2.139139	2.983313
SLC15A4	14.5457	11.46623	12.60535	13.19151	15.23658	14.82343	12.14675	15.40158
SLC15A5	0.016141	0.032706	0	0.016541	0.113004	0	0.016384	0.016371
SLC16A1	4.37803	5.472739	4.593095	6.025266	5.33428	5.36021	5.571945	4.919991
SLC16A10	0.279289	0.517677	0.307151	0.338244	0.587217	0.287587	0.373676	0.431335
SLC16A11	6.868927	8.011027	9.71302	9.225628	4.884187	7.697045	6.18443	5.520134
SLC16A12	0.090385	0.110648	0.137607	0.158232	0.064031	0.326092	0.175836	0.171884
SLC16A13	5.930307	6.378419	7.591218	9.508467	6.9923	8.036273	8.580141	6.241926
SLC16A14	2.336139	2.913416	1.455626	2.656206	2.775914	1.500863	2.862354	2.200152
SLC16A2	5.557243	6.126067	4.179367	6.435051	6.363896	5.402891	5.349786	4.946188
SLC16A3	0.59899	0.839346	0.633778	0.888854	0.770229	0.704901	0.939597	1.009898
SLC16A4	0.007838	0	0	0	0.007839	0	0	0
SLC16A5	1.428019	1.101404	1.28285	2.228131	1.658559	1.678949	1.318522	1.205399
SLC16A6	5.752281	4.78666	3.987233	5.171941	3.72183	3.752894	3.987012	4.02718
SLC16A7	4.972215	6.064094	4.574622	5.256625	4.52593	4.968161	5.110682	5.31749
SLC16A9	0.224223	0.885161	0.412316	0.78441	0.564495	0.544924	0.53365	0.807714
SLC17A2	0	0	0	0	0	0	0.015915	0
SLC17A3	0	0	0	0	0	0	0	0
SLC17A5	7.381064	7.286191	7.524451	8.461794	7.311914	7.247995	7.840401	8.111761
SLC17A7	0.455184	0.169405	0.293559	0.209431	0.334464	0.205764	0.207434	0.32976
SLC17A8	0	0	0	0	0	0	0.012466	0
SLC17A9	0.410325	0.302335	0.233304	0.318552	0.497428	0.087633	0.454342	0.353111
SLC18A1	0.126305	0	0.024946	0.03883	0	0.025434	0.02564	0.025621
SLC18A2	0.573421	0.518142	0.244876	0.889379	0.185999	0.078019	0.220225	0.487273
SLC18A3	0	0.072016	0.070198	0	0.035546	0.053677	0	0.072096
SLC18B1	5.348599	4.569663	3.46322	5.040339	5.15076	4.474661	3.548327	5.122744
SLC19A1	1.507729	1.584091	1.397033	1.818485	1.427253	1.755417	1.139887	1.195665
SLC19A2	7.015414	7.066683	5.965557	6.487602	5.421336	6.114533	5.518295	5.05667

SLC1A1	5.229836	9.05768	9.658312	8.559361	8.513899	9.431893	8.020545	9.710328
SLC1A2	1.138821	2.259955	3.670703	4.311995	2.129988	3.231759	2.714585	1.790896
SLC1A3	3.867157	4.90634	3.874825	4.701925	3.563618	3.502082	3.221935	3.707096
SLC1A4	5.725198	5.085572	6.671186	6.647576	7.274502	5.820885	7.509406	6.205634
SLC1A5	36.07191	37.96903	56.18048	48.7443	40.77757	47.78369	41.26585	33.47929
SLC1A6	0.101096	0.058527	0.071312	0.0296	0.028888	0.159951	0.073295	0.014648
SLC1A7	0.168609	0.138793	0.135288	0.226782	0.158092	0.02122	0.149746	0.064129
SLC20A1	11.98936	11.14369	10.39001	11.52973	12.04965	9.486379	14.19599	12.56849
SLC20A2	12.66878	15.74714	14.31695	12.31616	17.92041	20.73193	17.40279	14.83152
SLC22A1	0	0	0	0	0	0	0	0
SLC22A13	0.017662	0.005965	0.017442	0.012067	0.017665	0	0	0.011943
SLC22A14	0.135672	0.123707	0.120583	0.347582	0.230672	0.05464	0.137707	0.041281
SLC22A15	0.584087	0.496528	0.490619	0.474716	0.389441	0.574558	0.660991	0.667303
SLC22A16	0.282821	0.644698	0.508719	0.66246	0.828368	0.650864	0.563874	1.034698
SLC22A17	1.639685	2.030361	1.264561	2.101709	2.035555	1.656128	1.579746	1.784454
SLC22A18	1.295974	1.332579	2.13941	1.66505	1.412214	1.772228	1.040551	1.961825
SLC22A20I	0	0	0	0	0.016782	0	0	0
SLC22A23	18.53569	15.56182	13.9446	18.26792	15.67384	16.35957	14.72262	16.2311
SLC22A3	0.842836	1.165214	1.19648	1.682441	2.414685	1.944693	1.372329	1.656241
SLC22A31	1.102313	0.77689	1.688085	1.849954	1.949269	2.219687	1.102633	0.696728
SLC22A4	0.418165	0.48014	0.880969	0.385671	0.697031	0.575396	0.60836	0.537216
SLC22A5	2.567729	2.444046	2.869622	2.640697	2.86051	2.346064	3.032891	3.012071
SLC22A7	0	0	0	0.01151	0	0.011308	0.0114	0
SLC23A1	0.788352	0.483565	0.439577	0.736434	0.697272	0.550756	0.729413	0.734303
SLC23A2	7.951897	7.379583	7.89015	6.965065	6.848002	6.7797	6.924241	7.575141
SLC23A3	0.186206	0.341929	0.218365	0.333933	0.290985	0.36324	0.153562	0.188857
SLC24A1	0.099517	0.157536	0.073708	0.121104	0.068427	0.056361	0.151515	0.138784
SLC24A2	0.034986	0.139251	0.019743	0.030731	0.057485	0.020129	0.032975	0.088711
SLC24A3	4.513624	3.827889	3.252268	3.374879	3.614496	3.89075	3.930256	3.348119
SLC24A4	0	0	0	0.014597	0.014246	0	0	0
SLC24A5	0	0	0	0	0	0	0	0
SLC25A1	46.55142	43.10581	41.28787	44.13913	49.12345	42.38798	43.10672	36.60819
SLC25A10	6.241486	7.476102	4.430171	5.829769	6.209781	5.793178	6.731053	5.308252
SLC25A11	71.02436	66.3752	66.77573	63.19248	65.34611	75.02838	60.99668	63.29385
SLC25A12	8.656719	9.222875	7.798955	7.919034	8.211097	9.030672	8.641487	9.432333
SLC25A13	4.912274	5.642131	4.442368	5.024733	5.137859	5.896954	5.122935	5.484073
SLC25A14	2.444318	2.022972	1.886892	2.557784	2.117535	2.218381	1.956832	2.007734
SLC25A15	8.971199	9.889688	8.819183	9.711123	8.776776	7.769087	10.95835	11.6195
SLC25A16	9.481601	12.27682	12.20249	11.97326	10.86077	11.56706	11.07731	9.990678
SLC25A17	29.10994	27.52789	24.55929	30.01726	27.53835	28.02788	29.11615	25.00813
SLC25A19	6.10595	5.904899	5.966619	6.421284	6.619193	6.470093	6.490081	6.247006
SLC25A20	35.27994	41.62708	43.80975	46.88602	50.85664	44.23012	36.71848	32.58622
SLC25A21	0.315303	0.147434	0.251494	0.124274	0.121286	0.183148	0.086162	0.135296
SLC25A22	7.530296	7.024323	5.785791	7.087593	7.556857	6.817555	6.17173	6.443866
SLC25A23	5.779333	5.768079	5.471481	6.353215	6.200449	4.847409	5.807847	5.357798
SLC25A24	12.52267	11.1108	12.80346	13.12203	13.04296	11.18001	12.36991	14.87449
SLC25A25	1.223347	1.484733	1.214315	1.456683	1.610371	1.380522	1.471522	1.157832
SLC25A26	5.565825	5.476731	6.040853	5.430352	5.513191	4.905605	5.811753	5.933623
SLC25A27	0.335037	0.348608	0.371102	0.412931	0.484508	0.392027	0.252749	0.344398
SLC25A28	23.1621	21.41527	18.68323	23.59621	21.54938	20.55717	18.35324	23.61028
SLC25A29	4.565988	5.184279	4.66406	5.494891	5.477598	4.909312	6.231101	4.460189
SLC25A3	137.9717	157.037	159.3496	140.8578	131.5477	146.0899	152.8958	140.0376
SLC25A30	12.14112	9.697579	8.858271	10.75686	10.18253	12.0319	11.47387	11.6588
SLC25A31	0.01673	0.016949	0	0.017144	0	0	0	0
SLC25A32	6.949268	8.368428	8.101309	7.573005	8.385404	8.043409	7.282889	7.071116
SLC25A33	9.039979	12.18214	11.38808	10.45158	9.012169	13.68178	12.17529	8.316453
SLC25A34	0.353131	0.45794	0.146467	0.412541	0.331986	0.191993	0.129034	0.308016
SLC25A35	3.704448	5.480667	4.238971	13.57295	12.86433	12.95054	8.116858	3.921187

SLC25A36	15.69997	18.24085	14.66228	18.545	17.6842	18.11015	14.62555	16.93439
SLC25A37	25.13609	24.91732	23.49892	24.67585	25.82628	27.08986	22.6843	27.93882
SLC25A38	7.840102	8.829963	6.80042	8.516362	7.880321	8.998775	7.108882	7.8987
SLC25A39	57.88549	57.45921	53.29353	58.55105	59.27419	57.73798	57.20516	48.59777
SLC25A4	26.77224	33.00354	34.14223	34.40617	36.4187	34.77779	35.31331	31.55234
SLC25A40	1.565306	1.969725	1.637814	1.731387	1.577933	1.54473	1.576176	1.587589
SLC25A41	0	0	0	0.022952	0.0224	0	0.007578	0.007572
SLC25A42	2.044703	2.487085	2.430325	1.982428	1.928637	2.126472	1.857894	1.850286
SLC25A44	10.62379	10.77107	9.664962	10.2358	10.91967	10.25166	10.92472	10.24846
SLC25A45	0.060192	0.030491	0.01486	0.046262	0.0301	0	0.015274	0.015262
SLC25A46	19.13606	17.39536	16.97962	17.65028	18.7274	18.61896	19.58032	18.32082
SLC25A47	0.177734	0.219211	0.083944	0.237569	0.139114	0.217849	0.070591	0.180264
SLC25A48	0.091418	0.030873	0.015046	0.015614	0.045715	0.030681	0	0.092719
SLC25A51	9.549771	9.748785	9.734641	10.26217	9.54541	9.051741	9.840656	10.02617
SLC25A53	0.492324	0.73226	0.506879	0.440113	0.900965	0.390222	0.691087	0.520581
SLC25A6	793.4632	824.4247	776.859	724.9917	752.9341	681.9525	790.4797	806.0878
SLC26A1	0.008905	0	0.008794	0	0	0	0	0
SLC26A10	1.152495	1.308086	0.710265	1.536244	2.019294	1.587872	1.495211	1.248
SLC26A11	4.156999	4.329913	3.701412	3.970651	4.323059	4.959746	4.130422	3.544748
SLC26A2	9.441261	11.22537	8.931406	11.05957	11.95089	13.76273	12.28094	8.113464
SLC26A3	0	0.013976	0	0	0.006898	0.027778	0.014001	0.013991
SLC26A4	0	0	0	0.022338	0.010901	0.010974	0.011063	0.033163
SLC26A5	0	0	0	0	0	0	0.00685	0.013691
SLC26A6	0.570181	0.959941	0.877736	0.893642	0.821837	0.894883	0.97874	1.046038
SLC26A7	1.277972	1.204165	1.53731	1.643772	1.635805	1.350243	1.740198	1.408179
SLC26A8	0.068939	0.078574	0.0851	0.026492	0.017237	0.06941	0.069973	0.01748
SLC26A9	0.775306	0.913351	0.741905	0.954645	1.418573	0.786654	1.09805	1.048458
SLC27A1	4.939986	5.601666	3.933047	5.521422	4.553967	4.269379	4.584307	4.686663
SLC27A2	0.206562	0.28775	0.114743	0.171989	0.335707	0.142982	0.288283	0.039282
SLC27A3	10.65115	11.02175	7.188533	9.444084	8.168834	9.416397	8.614044	10.2713
SLC27A4	17.19999	13.63529	16.1968	14.90079	15.04814	14.31416	12.59315	13.89639
SLC27A5	1.152336	0.766145	0.506756	0.867209	0.810341	0.72513	0.584811	0.995255
SLC27A6	6.197813	5.38643	4.927757	6.422356	4.762833	7.207078	6.0496	5.974753
SLC28A1	0.060172	0.060962	0.045709	0.061662	0.083326	0.037282	0.032886	0.032861
SLC28A2	0.016841	0.008531	0	0.034516	0.050528	0	0.05128	0
SLC28A3	0.245406	0.294426	0.293369	0.311047	0.316485	0.338112	0.183538	0.530548
SLC29A1	17.87721	16.58915	18.87829	20.08989	20.13423	15.39766	17.06146	15.33706
SLC29A2	0.718149	0.566941	0.534203	0.783726	0.764881	0.788788	0.880386	1.371611
SLC29A3	8.831787	7.71971	5.829591	9.748816	6.696775	13.04794	8.299259	5.940665
SLC29A4	0.119561	0.060565	0.023614	0.11027	0.203279	0.096302	0.109219	0.060631
SLC2A1	20.6282	24.52244	19.84191	29.29893	21.76852	25.14099	21.91456	16.35144
SLC2A10	1.334221	2.053736	1.397672	3.15001	1.585054	2.627298	2.296962	2.242898
SLC2A11	1.363879	0.889144	1.733382	2.090417	0.865878	1.4329	1.938072	1.347209
SLC2A12	2.775648	3.696111	2.864164	4.053698	2.418616	2.41809	3.365556	2.992162
SLC2A13	0.426592	0.564249	0.725532	1.056467	0.592565	0.50109	0.962202	0.432665
SLC2A2	0	0	0.013556	0	0	0.013821	0	0.013922
SLC2A3	7.60496	7.358237	8.72477	8.263175	10.29896	6.191476	8.280419	9.706112
SLC2A4	1.16142	1.727891	1.446601	1.801366	1.674335	1.169354	1.370004	1.294687
SLC2A4RG	16.1931	16.54874	14.42184	17.40854	14.9236	16.37484	15.55774	13.3968
SLC2A5	0.03487	0.011776	0.011479	0.047645	0.011625	0.011703	0.011798	0
SLC2A6	0.60599	0.486038	0.411427	0.388125	0.6692	0.495731	0.486938	0.550594
SLC2A8	1.385322	1.360539	1.368061	1.535526	1.484466	1.238229	1.85089	1.333357
SLC2A9	0.027961	0	0.041419	0	0	0.042228	0.028381	0
SLC30A1	47.35318	48.3627	52.23613	44.57679	46.46547	54.17069	43.74382	49.91212
SLC30A10	0	0	0	0.01302	0	0.012792	0	0
SLC30A2	0.108036	0.140727	0.022862	0.150253	0.10805	0.116544	0.11749	0.117401
SLC30A3	0.218324	0.342844	0.118582	0.03356	0.152847	0.032972	0.121879	0.022143
SLC30A4	3.445054	2.998247	3.068659	3.017161	3.335456	3.033105	3.454368	4.113644

SLC30A5	13.17417	15.01632	13.71599	13.05398	13.76828	13.20934	13.63439	14.07296
SLC30A6	6.597176	6.211362	6.11051	6.896189	6.427879	6.635901	6.408334	5.726091
SLC30A7	9.601941	10.83113	9.726689	11.28532	10.76645	8.944964	10.75071	11.345
SLC30A9	18.02042	18.28046	19.38501	19.27643	18.48174	17.23109	20.30731	18.77777
SLC31A1	27.51477	26.22698	29.90354	34.12727	26.32124	29.86311	34.35748	25.10101
SLC31A2	8.256319	6.414553	8.105623	7.269864	8.257387	8.166458	7.040812	6.43387
SLC32A1	0.021875	0.033243	0.043205	0.011208	0.393802	0.033037	0.022203	0
SLC33A1	7.93624	9.331065	9.151344	9.167558	8.531853	9.672158	9.74105	7.972649
SLC34A2	0.048162	0.167295	0.101919	0.19037	0.082575	0.214747	0.069835	0.209348
SLC34A3	0.480984	0.476928	0.495203	0.503385	0.419636	0.391537	0.685555	0.903006
SLC35A1	16.72541	18.23841	20.78985	18.15732	17.89098	18.38223	19.10732	17.86995
SLC35A2	3.956066	4.141584	4.216059	4.062505	4.385206	4.066073	4.232908	4.246438
SLC35A3	5.13757	5.015855	6.03792	5.242754	6.061033	5.805244	5.473361	5.130598
SLC35A4	23.33853	22.83433	22.65798	23.1952	24.17365	23.06839	22.86578	21.48544
SLC35A5	14.14817	17.88528	14.08787	17.47057	14.54757	14.57229	13.82853	14.14799
SLC35B1	42.75473	43.17383	42.68345	41.56324	39.11512	40.7651	41.09583	38.31616
SLC35B2	17.87767	18.32572	14.85343	19.68771	19.07386	16.21821	20.16996	17.30604
SLC35B3	5.291413	5.50458	5.715808	5.277109	5.377225	6.155976	5.788352	4.863158
SLC35B4	4.060879	3.255563	3.208224	4.473578	3.637605	3.737533	3.082383	3.160645
SLC35C1	2.499194	2.660428	1.922578	3.164258	2.535742	2.060422	2.077138	1.781687
SLC35C2	6.008381	7.423086	7.102462	9.412394	7.178021	7.195952	8.242869	7.947922
SLC35D1	9.539214	10.46402	10.86372	9.32617	10.75858	12.5475	9.118573	11.19692
SLC35D2	2.676756	2.22047	3.619156	2.963978	3.593425	2.948268	3.683324	2.67842
SLC35D3	0.104221	0.105589	0.343075	0.522147	0.5675	0.501347	0.364369	0.16443
SLC35E1	12.80496	12.1578	11.58363	12.67431	13.04942	12.23594	12.6802	11.98822
SLC35E3	6.401578	7.567786	5.753809	6.062587	6.13346	6.648372	6.452111	6.636655
SLC35E4	2.314116	1.558625	1.621401	1.815011	2.08168	2.824546	1.732096	2.569964
SLC35F1	0.401758	0.736532	0.560491	0.562017	0.516613	0.802585	0.517822	0.724405
SLC35F2	4.063523	3.643233	3.657768	5.171442	4.183932	4.791267	3.868978	4.364519
SLC35F3	0	0	0	0.056284	0	0	0	0
SLC35F4	0	0	0	0	0	0	0	0.017205
SLC35F5	19.57034	21.54156	23.93496	20.24898	21.63318	22.97394	24.51517	20.47788
SLC35F6	15.38237	16.23249	17.24858	17.17226	16.79394	18.67374	15.35859	13.4703
SLC35G1	14.22143	11.8173	13.3355	11.83479	11.39347	14.75047	13.73144	16.03026
SLC35G2	0.188076	0.640923	0.270157	0.578205	0.205201	0.154932	0.312378	0.450872
SLC35G6	0.024097	0.012207	0.011899	0.049389	0.01205	0	0	0.01222
SLC36A1	7.858906	5.751568	7.177179	6.923138	7.463726	6.234352	7.348916	7.059042
SLC36A2	7.552433	4.194372	3.976949	5.631071	6.210859	3.347288	5.819325	5.496839
SLC36A3	0.111314	0.025061	0.024428	0.114071	0.098958	0.062263	0.050215	0.025088
SLC36A4	4.622657	4.656033	5.331222	5.935255	5.598557	5.137019	5.085731	4.366063
SLC37A1	0.548996	0.780633	0.713362	0.898178	0.992174	0.426681	0.752751	0.840101
SLC37A2	16.93928	12.89827	12.79329	15.21764	17.07754	15.87103	16.05898	14.99221
SLC37A3	12.02487	11.37435	10.38362	12.54173	11.68444	13.39088	14.13581	12.87521
SLC37A4	3.945254	3.303669	3.766889	5.115607	4.197408	4.276202	3.779695	3.582902
SLC38A1	9.391236	7.478636	6.135565	6.860678	7.239159	6.234779	7.794265	8.797039
SLC38A10	18.01615	16.72244	13.76694	16.83483	18.39012	15.90049	18.43239	18.23615
SLC38A11	0	0.390553	0.046144	0.442926	0.280395	0.070569	0.035571	0.438373
SLC38A2	74.09929	68.13979	60.10721	69.60086	69.02194	63.49129	67.02433	70.81468
SLC38A3	11.00109	9.828103	10.38363	11.6187	10.14177	12.22531	7.74791	6.603535
SLC38A4	1.15049	1.295099	0.811539	1.559508	1.278487	1.244153	0.864998	1.247129
SLC38A5	0.142237	0.112081	0.187287	0.3725	0.189674	0.143209	0.176453	0.176321
SLC38A6	2.417337	2.894346	2.702881	3.357548	2.397669	2.494194	2.25082	3.059621
SLC38A7	2.750944	2.680729	2.724069	2.696183	3.433501	2.777284	3.073711	3.010577
SLC38A8	0.140121	0.05915	0.034594	0.035898	0.105104	0.023513	0.071111	0.035529
SLC38A9	6.274873	6.656189	6.90598	6.704177	6.503992	6.614848	5.979058	7.346783
SLC39A1	28.90577	28.75448	26.7029	29.94606	30.75386	30.66332	30.03712	26.83819
SLC39A10	53.92323	55.45292	48.21839	40.98491	44.64463	49.17991	44.53423	57.50942
SLC39A11	7.08547	8.670907	8.1446	7.771269	7.885291	8.585718	8.339485	6.555033

SLC39A12	0.052513	0.031921	0.010372	0.150677	0.010504	0.095168	0.0533	0.021304
SLC39A13	9.374072	8.00207	7.083304	8.837769	9.955946	8.56128	9.465575	8.820523
SLC39A14	17.1241	14.54775	12.62931	16.9575	13.23744	14.33302	15.25536	13.16164
SLC39A2	2.951349	2.34935	3.074716	3.439902	2.432745	2.791914	2.287863	2.532846
SLC39A3	0	0	0	0	0	0	0	0
SLC39A4	0.012676	0.012843	0	0.02598	0	0.012763	0	0.012857
SLC39A5	0.290043	0.205695	0.257786	0.341812	0.232065	0.189816	0.235515	0.455967
SLC39A6	87.89099	82.13386	76.00727	80.59019	72.17501	81.22161	89.34448	97.31533
SLC39A7	20.2325	20.0886	18.64151	22.45702	20.3655	19.36006	23.47348	19.34377
SLC39A8	49.41353	46.54745	48.41119	47.03588	50.18485	42.3989	45.959	49.74758
SLC39A9	12.18389	11.82793	10.3132	10.76591	11.00589	10.88086	12.30336	11.27705
SLC3A1	0.044944	0.113836	0.055481	0.034543	0.123613	0.135755	0.079833	0.068376
SLC3A2	100.3198	83.82495	91.54338	106.3782	96.46215	100.0077	95.2687	86.95798
SLC40A1	140.5514	138.5216	180.785	120.7996	153.5123	167.5287	121.8286	152.8337
SLC41A1	5.911625	5.347711	3.724124	4.628242	5.587348	4.366689	5.374676	5.967365
SLC41A2	5.502054	6.982332	7.397165	6.198442	5.757861	6.577342	7.766642	8.26762
SLC41A3	1.417467	1.706804	1.147381	2.857531	1.731393	1.567526	1.945822	1.555485
SLC43A1	5.871853	4.633891	4.333757	6.027857	4.759906	5.351885	5.039801	5.34945
SLC43A2	4.762263	4.599208	3.260099	3.650366	3.687839	2.878979	4.544169	4.025635
SLC43A3	10.63503	5.723329	8.156881	11.79753	9.876658	10.97699	5.147503	9.766379
SLC44A1	26.08138	26.73204	28.70327	26.87213	24.51565	27.0778	26.89301	27.83704
SLC44A2	31.27417	32.65157	32.25811	32.15499	32.32896	32.06886	31.84091	32.30469
SLC44A3	6.129904	4.649342	7.79405	6.031837	6.862389	6.707487	5.243008	4.823083
SLC44A4	0.01219	0.01235	0	0	0.012192	0	0.012373	0
SLC44A5	0.083572	0.007697	0.05252	0.007786	0.045591	0.053546	0.038557	0.038528
SLC45A1	0	0	0	0	0	0.010999	0	0
SLC45A2	0.019144	0.058187	0.047265	0.019619	0.019147	0.048188	0.009716	0.029125
SLC45A3	4.929266	7.265429	4.668017	9.323046	6.006548	5.704529	4.2556	5.278552
SLC45A4	2.74072	2.700998	1.891105	2.361327	2.52871	2.533779	2.785822	2.045918
SLC46A1	2.910873	2.800448	1.746112	2.539882	3.204691	2.938533	2.445133	2.57642
SLC46A2	3.231367	1.752504	0.984616	2.585115	1.489745	2.273781	1.255849	1.047783
SLC46A3	8.068948	8.203765	9.965208	9.297546	9.221488	8.78032	8.141693	9.090983
SLC47A1	1.380442	2.112109	1.126761	1.905428	1.183389	2.084809	1.830072	1.62868
SLC47A2	1.112805	1.27012	1.043297	1.645597	2.718977	1.120407	1.415446	0.757193
SLC48A1	19.70333	19.67548	23.59207	22.84346	21.17403	22.48737	21.4116	19.04639
SLC49A3	3.065036	2.475817	2.229231	2.36633	3.862859	1.991288	2.469891	2.363008
SLC49A4	13.76039	12.6439	17.20716	15.29589	14.59497	14.67182	13.96679	13.88237
SLC4A1	2.492127	1.384305	1.748212	1.338574	1.23763	1.80832	1.238587	1.333529
SLC4A10	0.297046	0.376181	0.880034	0.631638	0.490189	0.613104	0.633154	0.406721
SLC4A11	1.110008	0.999623	0.654661	1.445577	1.333724	1.513405	1.126658	1.571443
SLC4A1AP	17.41481	18.65829	17.7874	18.16764	17.84211	18.00243	18.19984	17.68324
SLC4A2	13.0325	10.7633	9.487495	10.76816	12.44048	10.77202	11.96752	12.38757
SLC4A3	0.928517	0.990589	0.778016	1.052435	0.738689	0.651569	0.785371	0.941735
SLC4A5	0.296056	0.269948	0.274825	0.285186	0.373079	0.155001	0.078129	0.198179
SLC4A7	3.493462	3.784367	3.705863	3.714556	3.55612	3.517328	4.149117	3.704408
SLC4A8	0.075284	0.102801	0.048486	0.090566	0.101482	0.026365	0.119603	0.109553
SLC4A9	0.480999	0.604939	0.540524	0.654385	0.622062	0.551083	0.387204	0.277568
SLC50A1	19.49099	22.80184	19.78956	23.6539	22.59163	20.86617	21.14676	20.57476
SLC51A	0.070017	0	0	0	0.017506	0	0	0
SLC51B	0	0	0.006537	0.006784	0	0.006665	0	0
SLC52A2	5.209094	4.450394	4.120469	5.231894	5.326404	4.775008	4.31396	5.138683
SLC52A3	0.624758	1.151983	1.048855	0.76828	0.662329	0.893217	0.621447	0.760382
SLC5A1	4.019589	3.647619	2.021281	3.411571	2.873268	2.731138	3.078685	3.48905
SLC5A10	0.375041	0.379963	0.137173	0.213517	0.430658	0.223765	0.22558	0.098617
SLC5A11	0.30246	0.212143	0.103393	0.09537	0.372306	0.117125	0.212536	0.235973
SLC5A12	0.025375	0.025709	0.025059	0	0.012689	0.012774	0.025756	0
SLC5A2	0.272162	0.374998	0.354778	0.345841	0.283085	0.613808	0.243095	0.264995
SLC5A3	6.89313	7.455662	4.331216	5.244851	6.977854	5.485654	5.32747	5.921065

SLC5A5	0	0.035713	0.027849	0.007225	0.014102	0.028393	0.021468	0
SLC5A6	6.596365	6.575641	4.848278	6.821776	6.347553	6.550033	5.758582	5.715883
SLC5A7	0	0	0	0	0	0	0	0
SLC5A8	2.812854	3.427426	6.093654	3.59667	4.599991	3.189268	4.912738	3.264125
SLC5A9	0.636244	1.198719	1.747161	3.088441	2.260633	0.837263	1.971352	1.850997
SLC6A1	0.039975	0.080999	0.059215	0.116068	0.146594	0.087204	0.033812	0.033787
SLC6A11	0	0	0.019957	0.020709	0.020211	0.006782	0.061536	0.040993
SLC6A12	0.007877	0.02793	0	0	0.059081	0.003965	0.027981	0.011983
SLC6A13	0	0	0	0	0	0	0.012427	0.024836
SLC6A14	20.57785	15.99977	13.44522	15.89303	17.10314	15.73458	19.50275	17.35365
SLC6A15	0.968011	1.209348	0.973543	0.456436	0.588009	0.562053	1.048836	1.379326
SLC6A16	0.184409	0.435934	0.364222	1.196853	0.614776	0.350707	0.363951	0.789699
SLC6A17	0.13359	0.144075	0.170226	0.163395	0.267214	0.221278	0.113723	0.174827
SLC6A18	0	0	0	0	0.029053	0.014624	0	0
SLC6A2	2.504561	3.824873	2.715213	2.779694	3.447683	4.219845	3.456733	3.107311
SLC6A20	0.053127	0.182405	0.136992	0.154256	0.088557	0.172357	0.185738	0.179612
SLC6A3	0.030193	0	0.014909	0.015471	0	0.0304	0.015323	0.030623
SLC6A4	10.57941	13.50761	9.255894	9.292687	11.23695	10.34499	9.239763	11.03897
SLC6A5	0	0.009144	0.017827	0	0.009027	0.018175	0.009161	0
SLC6A6	3.407012	3.040113	2.036616	3.792349	3.186619	3.081256	3.762915	3.133028
SLC6A7	0	0.023756	0.007719	0	0.007817	0	0.007933	0.007927
SLC6A8	9.295515	10.76039	8.200074	11.00678	8.621059	10.19418	10.97127	9.462536
SLC6A9	3.492922	3.850227	3.16518	4.987089	4.422323	4.069986	3.385976	3.54928
SLC7A1	12.16126	12.21951	10.05536	10.83114	10.29665	10.38973	10.71022	10.70877
SLC7A10	0.029556	0.104802	0.014594	0.075719	0.162577	0.029758	0.029999	0.074941
SLC7A11	0.406819	0.233164	0.427003	0.593184	0.590545	0.45875	0.372809	0.337162
SLC7A14	0	0.003019	0	0.003053	0.00596	0	0	0
SLC7A2	6.598662	6.222588	6.841308	6.369475	7.122058	5.813761	6.980475	7.717096
SLC7A3	0	0	0	0.012697	0.012392	0	0	0
SLC7A4	0.127127	0.203926	0.397553	0.379972	0.392026	0.426652	0.258068	0.064468
SLC7A5	61.02716	53.60562	51.26305	57.95336	65.72736	62.31832	60.81246	47.4784
SLC7A6	6.349307	6.007572	5.100863	7.442918	6.462025	5.529061	4.703289	3.196206
SLC7A6OS	27.93704	28.30369	27.17717	25.31748	27.81902	27.21829	27.05115	25.58585
SLC7A7	2.748936	2.543338	2.748337	3.608589	3.703591	2.939268	3.597244	3.882558
SLC7A8	42.47316	31.65314	33.76676	31.46803	30.42694	31.78949	32.3092	35.65221
SLC7A9	0	0	0	0	0	0	0.008361	0.008354
SLC8A1	3.07224	4.499259	3.444067	3.599964	4.110976	3.357752	4.01296	3.872405
SLC8A2	0.103843	0.007014	0.02051	0.127698	0.138475	0.041821	0.06324	0.140428
SLC8A3	0.00288	0.037937	0.019912	0.026566	0.031689	0.043502	0.014618	0.011686
SLC8B1	3.663944	3.430427	2.853043	3.435336	3.260068	2.917258	3.376935	4.860833
SLC9A1	4.193387	4.371145	4.169284	4.282654	4.881633	3.70549	3.69939	4.466278
SLC9A2	5.50626	4.19293	3.664249	4.405656	4.66249	5.915065	5.437967	6.410883
SLC9A3	0.1116	0.033919	0.077146	0.045746	0.022323	0.033709	0.056637	0.135826
SLC9A3R1	40.64862	30.93563	28.70614	34.19778	39.11794	36.12047	31.96188	26.71775
SLC9A3R2	14.30701	12.03896	12.19242	14.42955	16.45303	15.99925	14.05141	14.1501
SLC9A4	0.134604	0.042616	0.008308	0	0.025241	0.042351	0.008539	0.025597
SLC9A5	0.136799	0.127507	0.037827	0.196263	0.202489	0.099168	0.099973	0.149847
SLC9A6	3.542357	3.915524	3.547553	3.760414	3.420179	3.68087	3.406499	3.270356
SLC9A7	0.812553	0.700847	0.487963	0.911448	1.109169	0.862326	0.858177	1.258454
SLC9A8	3.633229	4.289327	4.295384	4.400176	3.517867	3.606224	3.853175	4.7813
SLC9A9	4.182472	4.857661	3.777	5.239439	5.113454	4.050929	4.794019	4.814603
SLC9B1	0.397226	0.572702	0.573327	0.438378	0.458397	0.276881	0.325649	0.154954
SLC9B2	1.361082	1.171585	0.798389	1.656976	1.146322	0.865503	1.236077	1.639942
SLC9C1	0.008162	0	0	0	0	0	0	0
SLC9C2	0	0	0	0	0.008128	0.008182	0	0
SLCO1A2	0.073628	0.031969	0.18697	0.226355	0.126236	0.116492	0.256225	0.149352
SLCO1C1	0.00886	0.008976	0.00875	0	0.017723	0.017841	0.008993	0.008986
SLCO2A1	3.397723	3.280424	2.126186	3.998998	4.6178	3.175373	3.790143	3.69346

SLCO2B1	4.504365	4.846209	5.58553	5.759855	6.56087	4.293738	5.509455	4.568487
SLCO3A1	10.58991	10.96103	9.892285	12.40779	8.1207	9.061839	9.600484	9.949098
SLCO4A1	1.861452	1.536012	1.031421	1.018517	1.64267	1.534953	1.470463	1.3583
SLCO5A1	3.735952	2.475983	4.80886	4.462411	5.522365	4.350547	3.720852	2.997929
SLF1	3.696268	4.056224	3.592387	3.697818	3.30877	3.891014	3.930011	3.659804
SLF2	13.38612	13.37002	11.42623	12.63691	13.64672	11.94117	13.30851	14.83835
SLFNL1	0.022004	0	0	0	0.022007	0	0	0.022317
SLIRP	120.2279	163.2556	173.5731	119.7247	147.4976	161.7955	148.7912	156.0195
SLIT1	0.036649	0.055696	0.068766	0.018779	0.08797	0.04059	0.148797	0.059474
SLIT2	4.894772	4.51748	4.015703	4.628466	4.484753	3.158552	4.42714	3.974484
SLIT3	31.3174	26.12533	25.14945	25.44148	24.60892	20.93939	27.21572	27.86139
SLITRK1	0.006418	0	0.006338	0.006577	0	0.038771	0	0
SLITRK2	0.339647	0.310072	0.836695	0.596675	1.369963	1.266407	0.617505	0.514831
SLITRK3	0.00364	0	0	0.00373	0.003641	0.018325	0	0.003692
SLITRK4	0.012294	0.07058	0.012141	0.046194	0.036887	0.049512	0.020797	0.03325
SLITRK5	1.191424	0.933586	1.397188	0.79647	1.354489	1.054304	1.369905	3.360821
SLITRK6	1.986116	3.127847	2.420964	2.156223	1.835592	2.455058	1.337286	2.180594
SLK	21.16148	21.84869	20.93344	21.10136	20.46043	19.48937	21.62542	21.8507
SLMAP	21.63657	21.63088	26.03676	23.32684	23.0004	22.94153	24.18392	22.73328
SLN	0	0.035894	0	0	0	0.2497	0.035961	0
SLPI	0.142077	0	0	0.072798	0	0.357619	0.432624	0.07205
SLTM	24.66325	27.12122	21.94108	24.43424	24.8358	24.55894	25.44572	26.76624
SLU7	20.49373	24.83448	22.65827	26.6753	22.79449	25.37173	21.13678	23.90716
SLURP1	8.881639	3.939753	7.111599	11.51234	7.010199	1.595115	3.84959	1.752923
SLX1A	10.29279	9.29971	11.35337	11.65803	12.672	9.817679	10.56937	11.78238
SLX4	3.940098	2.892552	3.206086	2.694704	3.366113	2.75035	3.943055	3.465377
SLX4IP	2.471137	1.993286	2.036212	2.040395	1.951978	2.836658	2.348446	2.23094
SMAD1	15.76884	15.05397	15.21294	16.56109	17.19887	15.3551	15.92012	15.66678
SMAD2	33.11909	32.14641	31.90019	34.12676	33.11118	33.57844	31.90911	32.2064
SMAD3	9.06218	9.842984	8.193507	8.990169	8.47064	7.634729	8.866564	10.33653
SMAD4	15.84363	17.80596	16.58022	15.56321	16.09747	15.58193	16.59926	17.25271
SMAD5	18.8719	19.37615	17.74472	18.21473	18.91233	18.62684	17.57408	19.06349
SMAD6	5.518339	5.562668	5.595632	5.996037	6.203156	5.900382	5.441619	6.346899
SMAD7	15.79654	14.80332	14.80667	15.52546	14.49591	15.76642	13.55839	13.97529
SMAD9	2.996407	2.788504	2.230512	2.907793	2.713007	2.256944	2.044849	2.935458
SMAGP	28.72434	29.6156	28.86773	29.39998	29.34078	32.70968	28.26687	25.86663
SMAP1	35.38488	33.86885	37.82114	34.70476	35.96226	32.48014	35.54917	32.18862
SMAP2	11.48795	12.23683	11.40215	14.56934	12.57352	11.58591	12.19072	14.01712
SMARCA1	8.602701	11.47258	8.602204	10.78101	11.06205	11.0002	9.581613	11.42353
SMARCA2	20.24884	25.32226	22.67504	23.1702	23.12824	21.70898	23.24541	23.13082
SMARCA4	26.3729	25.70487	24.28422	26.82601	24.89658	23.96217	27.02126	23.84619
SMARCA5	40.39841	44.30002	43.56051	41.00561	42.58557	44.27675	41.8429	41.62257
SMARCAD	8.016028	9.352706	7.40321	8.43957	7.947444	7.774268	8.168879	8.586342
SMARCAL1	8.9215	8.647565	7.710877	9.203337	9.375467	8.130535	9.205993	9.394785
SMARCB1	40.38833	44.57275	37.93279	41.85081	39.12746	38.08017	41.11737	40.80499
SMARCC1	37.6467	34.05569	32.94803	32.67342	33.12217	29.82406	35.14192	34.41224
SMARCC2	34.10523	33.57328	28.92805	31.39445	33.41341	28.90908	31.54936	31.92345
SMARCD1	10.76886	11.54525	8.923667	11.35318	10.8266	8.743432	10.78741	10.20068
SMARCD2	57.53496	64.86281	59.66442	62.39748	50.64213	55.66306	61.08904	69.1305
SMARCD3	9.399975	10.84721	9.352229	10.20877	11.95876	9.747127	9.555237	10.36034
SMARCE1	49.89074	52.67862	49.2465	47.01063	48.75276	46.80984	49.36151	48.26822
SMC1A	20.01764	19.76502	17.48256	18.13614	18.38103	16.1441	19.39188	22.68006
SMC1B	0.005061	0.010256	0.014995	0.005187	0.015186	0	0.015412	0
SMC2	9.500621	10.90797	9.619898	9.778844	8.853045	10.54525	9.887395	10.72892
SMC3	28.43667	32.06592	27.85468	30.29446	28.37931	30.69258	32.53134	29.73547
SMC4	14.87885	16.9716	15.11719	14.86711	14.45743	15.73944	17.09131	17.04903
SMC5	9.918608	11.43924	9.775796	10.3189	10.8155	10.05497	10.31438	10.7015
SMC6	7.706315	8.770604	6.928461	6.977404	7.999456	7.844518	8.910831	9.16268

SMCHD1	13.66109	14.83145	14.70688	15.43308	14.17256	14.36052	14.19235	15.04894
SMCO1	0.045217	0	0	0	0	0.022763	0	0
SMCO2	0	0.026751	0	0	0	0	0	0
SMCO3	0.586599	0.1132	0.027585	0.200377	0.083811	0.168745	0.510341	0.793266
SMCP	0.420258	0.170309	0.207511	0.086134	0.084062	0.423129	0.085312	0.21312
SMCR8	5.998578	5.621931	4.866007	5.467017	5.411182	5.210599	5.257136	5.674965
SMG1	15.06106	13.78121	12.13736	12.93788	14.85867	12.45249	13.41521	16.61884
SMG5	13.78403	11.72357	11.05265	12.99494	13.70276	12.79732	13.35867	12.74104
SMG6	7.143092	5.922298	5.678987	6.569494	6.081456	5.758064	6.002096	6.075526
SMG7	19.88436	17.76506	17.73288	17.75084	18.64023	17.24415	16.7726	19.24153
SMG8	6.18894	5.894807	6.028671	5.746848	6.501332	6.629679	6.44416	6.533249
SMG9	6.888459	6.576735	6.284211	6.15374	5.864911	6.342518	6.316	6.168396
SMIM1	1.520245	2.004365	1.336776	2.155453	1.707893	2.075786	1.902389	1.309547
SMIM10L1	40.38673	48.3954	56.29719	52.61281	53.99791	58.77859	55.89345	54.21961
SMIM11A	14.91947	13.33339	16.82981	17.18462	15.77058	15.54042	16.18975	15.34715
SMIM12	12.10986	12.35658	12.57938	13.83063	12.17643	12.34527	12.6873	11.64507
SMIM13	6.638747	9.020429	7.344288	9.110823	7.937671	8.637712	7.370664	6.608198
SMIM14	15.08797	20.81353	22.4619	19.57633	19.38356	19.51345	19.29318	19.84981
SMIM15	18.79379	21.80803	20.47602	20.95732	22.09564	22.09341	20.09085	21.04468
SMIM17	0.16583	0.036523	0.028481	0.022166	0.07932	0.072592	0.021954	0.058501
SMIM18	0	0	0	0	0	0	0	0
SMIM19	23.47069	22.24668	26.93398	27.38336	21.25495	28.16214	23.96062	21.16902
SMIM2	0	0	0	0	0	0	0	0
SMIM20	66.32397	73.48326	70.24697	71.60588	66.64016	70.04273	66.75122	72.62804
SMIM26	35.68756	41.28126	52.67427	37.41681	37.94699	52.98353	40.24144	33.18394
SMIM27	30.05937	35.40367	38.51667	33.77403	36.27467	38.01849	35.55328	32.41898
SMIM28	0	0	0.050623	0	0.051269	0	0.156093	0.051992
SMIM3	9.120636	8.845302	10.73138	8.391054	8.104512	8.056412	9.102582	11.14184
SMIM30	24.99513	27.2829	31.85956	27.78614	27.47128	27.75699	25.2164	23.86675
SMIM32	0.054827	0.111093	0.243647	0.33711	0.219336	0.110403	0.361721	0.083411
SMIM33	0	0	0	0	0.0353	0	0.071649	0
SMIM4	2.115861	2.330032	2.4756	2.403954	2.001127	2.315561	2.28766	1.889397
SMIM5	4.094746	3.835753	4.510309	4.577058	5.601077	4.465222	3.970738	3.309651
SMIM6	0.343325	0.496901	0.419773	0.26806	0.196211	0.34567	0.514415	0.414539
SMIM7	36.9084	35.02232	46.00366	39.39107	40.44966	42.93477	40.05116	36.05993
SMIM8	2.494084	1.123029	1.657209	0.694185	1.570552	1.596578	1.375133	1.405327
SMIM9	0	0	0	0	0	0	0	0
SMKR1	2.042743	2.360015	2.265028	2.497321	1.720427	1.262885	2.400761	1.381215
SMNDC1	11.66352	11.59128	12.64887	11.5135	12.0687	14.01555	12.32339	11.94652
SMO	12.70804	11.95271	9.582912	11.92181	12.53858	10.34888	12.09293	12.54191
SMOC1	1.984393	3.109162	2.498955	1.836498	2.246192	1.293246	1.389613	2.051646
SMOC2	20.83596	17.23217	13.2883	16.22951	14.69574	16.42111	15.89254	17.26066
SMOX	8.110415	6.077428	4.990827	6.990893	6.307325	5.70094	6.938772	7.180395
SMPD1	31.65548	28.61318	38.16379	36.70073	33.96994	39.77964	33.26485	27.25392
SMPD2	12.19836	14.70397	13.67042	14.31669	13.06223	15.15921	13.46713	12.06433
SMPD3	10.3236	9.119153	11.70875	9.396393	10.91854	11.03406	7.622853	8.484525
SMPD4	11.20055	10.10963	9.846294	12.45467	12.14704	9.997635	10.07875	11.93833
SMPD5	0	0.035313	0.034421	0.071438	0.01743	0.017547	0.017689	0.017676
SMPDL3A	4.004571	5.239551	4.694852	4.400026	2.955627	4.118172	3.901619	4.702308
SMPDL3B	9.268663	8.128367	8.562258	8.53466	7.584432	8.606571	8.007075	5.214303
SMPX	0.01673	0	0.016521	0.017144	0.117125	0	0.016981	0
SMS	27.74422	28.30686	34.47795	33.26764	26.37593	32.24137	29.9173	25.4561
SMTN	26.5589	20.97013	19.42652	23.46611	22.32967	21.27559	23.92729	26.88491
SMTNL1	0	0	0	0	0	0	0	0
SMTNL2	5.016463	5.939704	3.621597	5.593207	3.998969	4.532076	4.668438	5.262033
SMU1	11.88097	23.13136	16.17493	16.833	14.12004	17.94913	13.91169	13.23242
SMUG1	10.67774	10.91971	9.038311	10.29394	10.02868	9.310119	9.133608	11.12358
SMURF1	5.499625	4.516034	4.051772	5.127061	5.522163	4.636303	4.330573	4.532057

SMURF2	11.1987	10.2732	9.973758	9.851488	12.06638	9.714732	10.28194	11.70745
SMYD1	0.018201	0.00461	0.013481	0.046631	0.004551	0.12828	0.046186	0.055381
SMYD2	11.52313	13.03585	16.33713	11.77691	14.02729	10.74649	14.59646	13.01882
SMYD3	3.65567	3.188854	2.299883	3.644973	3.162069	3.368002	3.911073	3.10646
SMYD4	0.988081	0.857107	0.676023	1.111826	0.781524	0.643714	1.232324	0.799097
SMYD5	8.52479	9.046313	7.764161	10.24374	7.885607	8.028919	9.370867	8.748669
SNAI1	2.141694	2.882027	1.017141	2.429298	3.18843	2.255088	1.460276	1.840552
SNAI2	43.07626	41.9901	32.19577	38.98967	46.93214	41.31026	39.56842	42.07135
SNAI3	0.008027	0	0.007927	0.024677	0	0.032326	0	0.008141
SNAP23	27.27118	26.3373	27.71376	24.16634	29.18133	28.21776	24.42596	28.06117
SNAP25	0.283109	0.130375	0.292289	0.487932	0.694993	0.531216	0.640019	0.574278
SNAP29	14.4867	14.30289	14.26063	17.30404	16.28811	15.74695	12.79966	13.95984
SNAP47	7.996904	9.221736	7.27734	8.866252	7.588487	6.732512	7.424293	8.650539
SNAPC1	4.950464	5.06366	5.640901	4.243834	4.736874	4.70871	4.928092	4.900242
SNAPC2	4.531149	4.739438	4.47469	4.990772	4.633444	5.677031	5.333139	4.76756
SNAPC3	3.119955	3.481573	3.425549	3.104564	3.023453	3.082736	2.983175	3.007134
SNAPC4	3.095999	2.637813	1.730616	2.873378	2.478788	2.058495	2.126017	2.716882
SNAPC5	8.964501	8.534339	8.164251	9.259464	7.400227	9.3266	8.636799	9.279732
SNAPIN	38.40509	42.59369	47.63805	44.1865	44.02607	43.29583	42.84975	37.24029
SNCA	0.774251	0.902074	1.095932	2.07614	1.974595	1.818927	0.864451	1.138645
SNCAIP	2.270878	1.52848	1.986509	2.222448	2.381194	2.563291	3.054646	2.621989
SNCB	0.017592	0	0	0	0	0.017712	0	0.017843
SNCG	2.45827	14.33835	1.872751	4.606469	13.83834	5.268351	4.847709	5.200239
SND1	11.49229	13.54748	13.38551	14.02832	11.81107	11.59476	15.3436	15.46075
SNED1	3.166746	3.268242	2.429662	4.5183	5.683478	3.202395	2.610251	3.607129
SNF8	20.92394	21.8974	21.77792	24.41972	23.6862	24.07644	23.805	19.16535
SNIP1	8.546913	8.395993	7.704529	9.765228	8.581894	8.491625	8.915774	9.573231
SNN	16.57892	14.76919	14.75781	16.23042	14.75859	17.02654	15.34968	15.56266
SNORC	0	0.020989	0.020459	0	0.02072	0.208589	0.021028	0.147086
SNPH	0.464246	0.49922	0.550958	0.550864	0.517254	0.414116	0.653081	0.631937
SNRK	18.86626	17.68618	17.96653	18.89174	21.55071	17.40693	18.70095	18.50554
SNRNP200	37.3134	35.76127	30.52834	32.10497	32.78508	29.33575	34.24944	34.41971
SNRNP25	60.5486	64.00639	67.69984	60.42603	66.62103	68.02989	54.75631	54.65448
SNRNP27	28.05235	29.06061	27.95238	26.83721	28.68786	30.56585	27.19056	25.15155
SNRNP35	9.682178	9.368145	10.7081	10.38943	9.268721	10.64592	10.85855	11.22888
SNRNP40	21.4396	24.17473	21.07139	21.07925	19.37831	20.14356	24.9466	24.63373
SNRNP48	9.446586	9.886293	7.636218	9.081783	9.428328	8.589425	7.492701	10.09469
SNRNP70	62.51453	58.97495	55.56842	67.66078	55.80758	58.15256	57.86897	60.07343
SNRPA	29.16589	31.06015	27.41215	33.20448	30.72481	29.9152	31.73618	27.76956
SNRPA1	44.60773	44.45471	44.15988	43.84533	42.57236	45.64633	43.64925	47.42357
SNRPB	94.57098	102.991	98.71577	96.16147	90.76141	92.64362	106.4952	88.65353
SNRPB2	41.69363	55.25737	50.47855	47.53984	48.66268	55.41491	54.51836	48.14404
SNRPC	50.14843	51.63806	51.06071	54.52279	51.79655	56.87364	51.18791	48.73828
SNRPD1	107.1654	120.4241	106.6187	110.3109	106.101	117.3445	114.406	96.25869
SNRPD2	196.8037	217.889	201.9641	173.1021	189.8438	203.5133	207.3174	185.91
SNRPD3	109.3482	109.7765	108.3128	115.6633	100.9976	115.7646	129.3114	118.1697
SNRPE	40.04678	42.53474	47.43822	39.94703	41.98917	46.95104	44.23547	50.5378
SNRPF	76.31234	100.8837	96.03272	86.47641	89.95971	100.7388	83.1024	73.81991
SNRPG	120.8074	137.299	151.2561	129.1594	122.7774	167.8092	147.4701	115.7655
SNTA1	0.446084	0.572805	0.338078	0.443845	0.443548	0.331626	0.397494	0.36826
SNTB1	11.32833	11.08829	11.07158	12.6752	10.73794	12.06701	11.41905	11.62151
SNTB2	12.071	11.67309	9.896708	9.92146	12.20332	10.35332	11.33308	13.13461
SNTG2	0.01428	0	0.042307	0	0	0.028755	0	0.014483
SNTN	0.01576	0.015966	0	0	0	0	0	0.015984
SNU13	61.96511	67.72334	67.48178	66.56755	68.07509	66.24691	49.71575	54.99616
SNUPN	8.152184	9.690763	8.980899	9.152398	11.93996	11.43631	10.90391	11.77764
SNURF	15.26156	18.46392	16.66087	15.79185	15.50293	17.111	15.90938	17.02754
SNW1	47.92754	53.85362	51.50468	50.66081	52.82467	52.91677	52.22398	49.62555

SNX1	16.81366	17.99729	16.40861	16.79109	16.70421	16.56965	17.19742	16.96903
SNX10	0.423452	0.77431	0.550768	0.433941	0.764379	0.644717	0.5556	0.932287
SNX11	6.851627	6.558942	5.785947	6.955	7.262584	6.040203	6.877738	5.624993
SNX12	39.0588	39.89665	43.10449	40.95232	42.06052	40.82204	38.98092	37.20296
SNX13	21.31058	22.47515	21.83789	20.29245	20.3648	22.78857	20.30046	21.6229
SNX14	17.44569	20.34332	18.51504	18.89549	18.65951	20.53607	19.8091	18.95126
SNX15	7.594119	7.506131	7.885648	7.961493	8.047925	7.314465	8.167588	6.91948
SNX16	6.50833	5.679202	5.640339	6.378323	6.681932	6.362043	5.59357	7.646513
SNX17	23.87688	26.20849	21.61208	25.94108	25.82964	24.34085	24.11949	24.80849
SNX18	13.48744	13.94411	16.45112	15.88061	17.0268	16.05506	15.18187	13.88779
SNX19	9.601707	9.778985	8.926136	9.871954	10.26718	9.196036	9.200023	9.719147
SNX2	30.58709	35.25607	32.92989	33.33871	33.93777	36.13503	30.65524	31.03336
SNX20	0.504742	0.827927	0.735812	0.701975	0.829327	0.592889	0.756272	1.145743
SNX21	4.669113	5.505864	4.610935	6.013644	6.149736	5.445979	5.25709	5.822436
SNX22	0.426741	0.432341	0.520582	0.077173	0.351479	0.45493	0.305747	0.280057
SNX24	0.813921	0.88614	0.671816	1.655715	0.935523	2.091512	0.974093	0.862471
SNX25	4.795465	4.193931	4.55907	4.373072	4.957943	4.67386	4.677197	5.001955
SNX27	10.29327	10.57403	9.430547	8.815603	9.878055	8.776102	10.16584	10.27385
SNX29	4.161329	4.215942	3.637084	4.257035	3.356112	4.620683	4.004713	3.954275
SNX3	91.38246	101.2542	113.9541	107.5737	103.5414	109.4517	90.27623	97.65261
SNX30	6.817685	7.531033	6.345423	6.408105	6.162371	7.192712	6.752533	7.200995
SNX31	0.01233	0.062459	0.012176	0.290615	0.024663	0.037243	0.07509	0.025011
SNX32	0	0	0.00644	0.013366	0.006522	0.013132	0.006619	0.013228
SNX33	12.53752	10.27227	10.54084	10.45594	10.26854	9.598158	9.607241	11.32602
SNX4	24.77711	28.59797	27.32657	26.43765	26.44898	29.9745	28.39748	26.45366
SNX5	30.44975	32.89997	32.77461	31.30568	28.27063	34.6236	30.26198	32.14109
SNX6	20.19777	25.54921	24.22708	22.88463	25.57878	24.44762	21.84328	24.48052
SNX7	4.871227	5.806508	5.491473	5.174241	5.516988	5.546495	4.771207	4.850333
SNX8	8.809642	7.344544	7.328186	9.885799	10.31413	9.003926	7.705773	9.263122
SNX9	35.59231	35.51818	35.70698	36.82305	36.379	33.91779	38.62524	38.26634
SOAT1	24.49529	27.5441	47.63541	42.25577	51.76564	40.56455	18.42903	19.56247
SOAT2	0.013341	0	0	0.054685	0.013343	0	0	0
SOBP	0.331468	0.651487	0.48446	0.47555	0.590089	0.493924	0.61905	0.66565
SOCS1	1.70052	1.670097	1.25093	1.778206	1.318941	1.240426	1.479452	1.795125
SOCS2	19.53936	34.5116	19.51859	21.95364	20.94815	23.82644	27.23764	23.40969
SOCS3	2.283766	1.637577	2.749624	3.889872	3.327011	2.288868	1.979316	2.379736
SOCS4	8.605083	9.238038	8.472305	8.301074	9.30073	9.384772	8.681623	8.797577
SOCS5	5.335018	5.515467	4.20407	5.330644	5.680924	5.225136	5.691635	6.295391
SOCS6	2.385253	2.963613	3.510115	2.449149	3.465639	3.096485	3.159733	2.347776
SOCS7	6.978445	5.629839	5.118989	6.161746	6.243356	5.197304	5.709492	5.992818
SOD1	105.831	164.428	155.5779	176.9698	143.8744	130.3055	168.0067	131.7925
SOD2	53.52928	61.58676	62.98991	52.45754	72.21263	46.16738	59.12624	58.81558
SOD3	3.163642	3.684915	2.278494	2.818687	2.640068	2.160698	2.065737	3.402834
SOGA1	2.660709	2.244959	1.836837	2.531554	2.793484	1.997707	2.280616	2.751046
SOGA3	0.114228	0.125647	0.061237	0.130436	0.039169	0.115009	0.096066	0.129095
SOLD1	0.508028	1.415412	1.144498	1.708258	0.857408	1.246778	1.466375	1.851716
SON	37.40165	36.80835	32.43881	34.10957	35.89175	32.39756	34.89824	36.61707
SORBS1	13.45499	13.44518	15.48085	14.2941	12.66463	14.52729	12.60927	12.50193
SORBS2	4.108468	4.144243	4.909764	5.711533	3.682695	4.551271	4.239173	3.807296
SORBS3	19.1909	18.37717	18.47366	21.77917	21.68126	18.9438	19.64822	20.15833
SORCS1	0.069098	0.060671	0.086433	0.108574	0.101356	0.074208	0.154295	0.242949
SORCS2	2.155645	2.41669	0.772348	2.038729	2.239032	1.63393	2.813116	2.21112
SORCS3	0.010124	0.010257	0.009998	0.005188	0	0.025484	0.010276	0.005134
SORD	3.636505	5.14383	3.591193	5.101141	3.716472	4.891801	5.203779	4.433989
SORL1	4.747205	5.039919	3.294029	5.028205	4.292906	4.534864	4.754734	6.082112
SORT1	47.04258	41.96215	42.62193	45.35463	41.86819	43.26288	43.69036	44.82
SOS1	4.809732	5.182709	4.524075	4.754018	5.618519	4.336938	4.774046	4.819428
SOS2	8.868862	9.365298	8.54138	9.126988	9.014716	9.35569	8.545001	8.93533

SOST	0.411056	0.340732	0.036903	0.076589	0.149494	0.150496	0.037929	0.644312
SOSTDC1	57.44398	46.23897	43.96631	45.7046	41.31142	45.11081	59.93724	62.25773
SOWAHA	0.043829	0.088808	0.043283	0.179658	0.105203	0.114734	0.204637	0.097796
SOWAHB	0.434904	0.700257	0.61355	0.573013	0.660207	0.758462	0.685789	0.322944
SOWAHC	3.244358	2.43411	2.961473	2.749635	2.402889	2.630874	2.349617	1.814246
SOX10	1.302799	3.129433	2.137359	2.627073	2.679489	2.665711	2.05816	2.312354
SOX11	0.180025	0.14399	0.233924	0.048549	0.189525	0.124017	0.125023	0.105709
SOX12	2.4902	2.707201	1.532771	2.796584	2.968156	1.951962	2.406369	2.813966
SOX13	9.855247	8.541032	5.915036	8.335018	8.160988	7.026829	7.030274	6.978146
SOX14	0	0.078441	0	0	0	0	0.039293	0
SOX15	1.529654	1.514239	1.176188	1.208564	1.343	1.739965	1.481487	1.148768
SOX17	1.573465	1.725395	1.352772	2.257416	1.869888	1.677403	1.14613	1.652189
SOX18	4.670285	3.907261	4.917423	6.670341	6.542499	5.128173	4.112699	5.71053
SOX2	0	0.082113	0.1334	0.1938	0.16212	0.24481	0.082265	0.054802
SOX21	16.57871	14.65426	15.41102	12.89679	12.41886	10.67746	15.43079	13.68324
SOX30	0	0	0	0	0.01215	0	0	0
SOX4	15.04371	14.34907	14.37986	10.94791	11.96813	12.05305	15.03167	15.11061
SOX5	1.585699	2.14395	1.506475	1.151883	1.431042	1.137493	1.484335	1.137133
SOX6	0.150479	0.250812	0.139017	0.218874	0.208756	0.222374	0.130565	0.125544
SOX7	2.337997	1.714474	2.044995	2.601274	2.605533	1.322706	2.282664	1.897024
SOX8	0.558999	0.228521	0.397077	0.442196	0.519838	0.473952	0.43798	0.447597
SOX9	44.9694	29.42242	35.8768	35.92984	38.16691	34.83787	36.01868	41.88533
SP1	23.86872	24.03778	22.48805	23.07617	22.78862	21.3511	22.58133	24.24019
SP110	5.108702	4.102056	3.622773	4.288446	4.420867	4.113058	3.475276	5.190614
SP140	2.783256	4.327034	2.474422	4.267356	4.705806	2.809437	3.294635	3.941921
SP2	2.308669	2.217826	1.562324	2.035956	2.253773	1.879926	2.053888	2.304219
SP3	30.37796	30.26637	29.46653	28.95528	29.23051	28.43392	29.60671	30.76662
SP4	3.845915	3.530789	3.090142	3.520719	3.480558	3.493928	3.145963	3.193731
SP5	0.437819	0.269307	0.432364	0.833234	0.50043	0.645472	0.190451	0.126872
SP6	44.71059	40.21024	34.33106	39.51533	39.64643	39.59766	44.55214	40.37619
SP7	0.263386	0.213474	0.277445	0.51283	0.351227	0.300544	0.204958	0.204804
SP9	0.019356	0.039221	0.019115	0.019836	0	0.019489	0.039293	0.019632
SPA17	12.22473	10.38875	16.07252	15.78097	6.605859	11.94084	13.63039	12.87991
SPAAR	0.265161	0.358187	0.290952	0.211344	0.324127	0.474617	0.119617	0.448225
SPACA4	0.14687	0.042513	0	0.021501	0.062952	0.105624	0.063888	0.170241
SPACA6	0.293323	0.25025	0.091474	0.205666	0.370561	0.170979	0.109687	0.125262
SPACA9	0.338501	0.323346	0.238773	0.58475	0.299854	0.262913	0.539908	0.421792
SPAG1	6.452331	5.476955	7.484152	7.140832	6.809252	7.20604	6.836746	5.033421
SPAG16	0.05296	0.053655	0.091525	0.217086	0.158899	0.01333	0.174701	0.147712
SPAG17	0.045878	0.085213	0.079285	0.074439	0.053531	0.115478	0.042685	0.089184
SPAG4	0.66998	0.547071	0.46413	0.543112	0.520052	0.503401	0.66988	0.537529
SPAG5	6.629404	6.588405	6.289919	6.808849	4.727406	5.971306	6.789193	6.738853
SPAG6	0.102901	0.029786	0.029034	0.030129	0.044106	0.029601	0.119366	0.059638
SPAG7	29.56387	37.69715	28.79419	31.85784	31.72685	29.15211	29.33706	31.65914
SPAG8	0.873903	0.577951	0.77911	0.932864	0.643373	0.708786	0.788455	0.824792
SPAG9	19.07926	17.20869	15.56675	16.59986	17.36651	15.82026	18.2265	19.19799
SPARC	501.8146	447.479	614.9748	441.0689	485.3855	423.5396	464.6704	608.4653
SPARCL1	65.04712	71.58757	54.77176	72.4692	82.24073	82.45547	70.04389	63.86244
SPART	36.49153	34.99084	33.95077	36.45998	33.87806	35.03177	36.00516	34.85325
SPAST	10.26497	10.5788	10.78472	11.1504	10.35756	10.20304	10.49999	10.49786
SPATA1	0.255687	0.431737	0.437668	0.384296	0.443247	0.360407	0.449838	0.138307
SPATA13	2.295438	2.44088	2.165672	1.990704	1.984577	1.822155	2.299061	2.108772
SPATA16	0	0	0	0.015064	0	0	0	0
SPATA17	0.045198	0.022896	0.066953	0.023159	0.033903	0.045507	0.022938	0.022921
SPATA18	0.050034	0.11152	0.029646	0.030764	0	0.050376	0.071099	0.040597
SPATA19	0	0.060982	0	0	0	0	0	0
SPATA2	3.894258	3.509924	3.189773	3.403457	3.777528	2.439064	3.688279	3.711921
SPATA20	3.722918	3.771777	2.365382	3.668046	3.50807	3.206421	3.405438	3.320986

SPATA21	0.012676	0	0	0.01299	0.050712	0.012763	0	0.025713
SPATA22	0	0	0.050485	0	0	0.025736	0.025944	0
SPATA24	8.122549	9.434693	9.451898	8.906939	8.53754	10.15744	9.635955	8.474305
SPATA25	0	0	0	0.03804	0	0	0	0
SPATA2L	2.220333	1.740468	1.824555	1.777084	2.253038	2.317089	2.187839	1.956066
SPATA3	0	0.040451	0.03943	0.040916	0.039933	0.0402	0	0.121488
SPATA32	0.01603	0	0	0	0	0	0	0
SPATA46	0.01402	0	0.00923	0	0.009348	0.018821	0	0.00474
SPATA48	0.021156	0	0	0	0.084634	0	0	0
SPATA5	4.261493	3.942701	4.168692	4.012744	4.64804	4.549661	4.113225	4.199833
SPATA5L1	3.498646	3.647137	2.755154	3.308628	3.757874	3.228057	4.179138	3.959204
SPATA6	1.886502	1.768835	1.598773	2.016881	2.059093	1.630005	2.098915	1.784574
SPATA6L	0.088201	0.282969	0.275823	0.301286	0.132319	0.444018	0.238731	0.253461
SPATA7	3.793844	5.482994	4.243236	5.400371	4.078636	5.085677	4.916089	4.657342
SPATA9	2.948815	3.336058	1.965648	2.644125	2.162744	2.746288	2.6688	2.567097
SPATC1L	0	0	0.023365	0.072737	0	0	0.024014	0
SPATS1	0.329715	0.222695	0.289428	0.131399	0.183199	0.479509	0.130146	0.130048
SPATS2	4.582628	5.029036	4.636266	5.400945	4.65051	4.41071	4.757597	4.450728
SPATS2L	38.97647	40.47819	36.31649	35.59443	38.58407	38.6588	36.71586	36.84071
SPC24	5.899157	6.428045	6.077119	5.784334	4.414328	5.234414	4.91073	5.660304
SPC25	5.793106	5.770217	5.078123	3.435226	3.971069	5.70161	4.657755	5.182389
SPCS1	65.20381	80.00016	85.7522	75.57856	73.60486	78.66365	71.60444	67.32145
SPCS2	84.50422	98.54284	106.1054	95.64217	94.03825	105.8165	101.3281	89.71936
SPCS3	15.53851	18.88856	20.08325	18.07683	18.66964	19.42359	17.28239	17.9502
SPDEF	1.082248	1.984955	2.65348	6.424872	4.92673	2.79925	4.602258	1.249055
SPDL1	3.436883	3.658665	2.98505	3.395432	3.822483	3.189679	3.65069	4.421746
SPDYA	0.924837	0.468487	0.253698	0.368568	0.590944	0.375049	0.586693	0.846808
SPDYC	0.323574	0.070247	0.228245	0.118425	0.069346	0.605029	0.070377	0.070324
SPECC1	7.489113	6.803747	8.177324	7.651712	7.530327	7.79687	8.105188	7.178528
SPECC1L	9.952464	9.969119	8.664495	9.998518	10.26679	9.345859	9.739378	10.66457
SPEF1	1.281118	1.393259	0.99354	1.63921	1.129267	0.969224	0.830157	1.255309
SPEG	1.5729	1.503931	1.211499	1.602009	1.475025	1.277758	1.46573	1.599192
SPEGNB	0	0.039059	0.038072	0.15803	0.115673	0.116448	0	0.039101
SPEM1	0.371455	0.206982	0.128389	0.304525	0.53868	0.261795	0.113108	0.339069
SPEM2	0.016349	0	0.016145	0	0.016351	0	0.016594	0.033163
SPEM3	0	0	0	0	0	0	0	0
SPEN	11.9173	9.574404	7.579484	9.559247	10.31629	8.247518	8.321451	10.88036
SPERT	0.019683	0	0	0.020171	0.039371	0	0.019978	0.019963
SPESP1	0.150885	0	0	0.103082	0	0	0	0
SPG11	12.67541	11.15358	9.978819	10.62868	11.49481	11.1094	11.5826	11.82305
SPG21	17.79538	17.48113	18.3903	20.41996	18.33845	18.92568	17.77177	17.67774
SPG7	13.18105	11.65932	11.74402	15.48404	13.11876	13.07783	14.08389	13.98057
SPHAR	0.144933	0.051824	0.058935	0.05242	0.085266	0.06867	0.008653	0.129703
SPHK1	5.548173	3.938161	3.542776	3.413117	4.598386	3.784394	3.823787	3.386714
SPHK2	7.896557	8.234801	7.295057	8.614232	8.090578	7.826154	7.051324	6.427531
SPHKAP	0	0.004061	0	0	0	0	0	0
SPI1	3.122297	3.340805	4.105947	4.423989	4.954898	3.320055	4.252457	7.997647
SPIB	0.038132	0.111606	0.058578	0.043419	0.055087	0.046925	0.025803	0.103134
SPIC	0.025284	0.051231	0	0	0.126435	0.127283	0	0.051287
SPICE1	9.108794	7.277411	6.465646	7.452852	6.978041	7.241229	6.954524	8.357312
SPIDR	5.461519	5.869367	5.402324	4.659407	4.852321	5.399508	3.968704	5.093579
SPIN1	19.65504	19.31105	18.60931	18.06847	18.967	19.06177	20.56108	21.82409
SPIN4	0.507679	0.63616	0.567321	0.841991	0.654724	0.531324	0.535635	0.623308
SPINDOC	5.480102	6.2691	4.975828	7.131119	6.097577	6.053771	5.498287	8.458
SPINK4	11.8469	4.21136	10.9467	8.59054	9.700469	7.324107	14.13418	10.46969
SPINK5	108.4699	94.18169	112.7083	124.7317	127.9118	130.1864	99.83502	86.10242
SPINK7	16.65049	14.6957	5.901329	12.40464	33.50961	11.05617	17.10761	23.31099
SPINK9	1.399458	1.181521	0.057584	4.959676	0.524865	2.230946	8.996184	5.677522

SPINT1	31.66996	30.69675	31.74437	28.17364	26.85925	32.53839	30.47969	31.83616
SPINT2	183.9311	195.6199	181.85	192.0683	169.0068	190.5066	185.3912	174.5855
SPIRE1	13.8787	12.35716	11.32123	12.53788	12.84013	12.0525	12.28704	11.25555
SPIRE2	5.817861	5.271657	5.686643	6.540902	5.313078	6.476297	6.277343	5.337755
SPN	0.643077	1.039324	0.604823	1.012045	0.880517	0.470187	0.621641	1.343287
SPNS1	10.16222	8.594199	8.770516	9.387992	9.097654	9.570488	9.527942	9.149545
SPNS2	8.791109	7.998817	7.101667	9.230196	9.680271	8.488744	9.288339	8.194194
SPNS3	0.337544	0.465085	0.22667	0.29056	0.405105	0.299068	0.561876	0.314961
SPO11	0	0	0	0	0	0	0	0
SPOCD1	0	0	0	0	0	0	0	0
SPOCK1	0.461928	0.461832	0.840317	0.305199	0.382963	0.50792	0.339304	0.51782
SPOCK2	7.680391	7.301227	6.511966	10.47947	9.241591	5.979115	6.722185	6.814191
SPON1	1.372018	1.837012	1.275221	1.218536	1.840356	1.668503	1.245147	1.72443
SPON2	7.642648	7.74295	8.089388	9.6858	10.63197	8.922762	9.799765	9.957309
SPOP	22.0297	20.89882	23.41405	20.72242	24.37598	23.55905	20.23164	21.43178
SPOPL	5.043302	4.789516	4.612755	5.420335	5.033979	5.08445	5.274174	5.742261
SPP1	93.26983	79.32745	87.38265	102.9875	126.8404	134.0377	77.82774	69.06332
SPP2	0.034217	0	0	0	0	0	0	0.034704
SPPL2A	19.04902	21.46215	18.9166	19.95122	20.29629	18.96011	19.8483	19.1252
SPPL2B	5.948027	5.696259	4.674801	5.572387	5.429597	5.758328	4.99234	5.167062
SPPL3	17.69545	17.333	18.45758	18.49322	17.84808	19.34428	17.66299	15.52243
SPR	17.48721	14.15384	13.84399	14.66212	12.54295	14.66414	14.52233	9.999995
SPRED1	9.242933	9.045881	8.4495	8.459832	9.082502	8.031519	8.105792	9.583948
SPRED2	9.727199	9.777747	8.36328	9.276563	9.875626	8.812753	8.559783	9.484837
SPRED3	0.198324	0.407006	0.170744	0.505487	0.528933	0.276479	0.299368	0.381665
SPRN	0.431599	0.185837	0.394254	0.420175	0.205036	0.336774	0.48188	0.470573
SPRTN	7.314455	6.958377	6.195116	7.23562	7.017884	6.932777	6.296337	7.640428
SPRY1	38.5168	28.64277	22.4243	23.64095	27.57974	26.18156	26.54105	31.15068
SPRY2	7.5562	8.85745	6.524667	8.344921	8.606438	7.494646	8.747082	10.0199
SPRY3	0.198908	0.166972	0.252552	0.22713	0.198933	0.33187	0.196123	0.161391
SPRY4	4.564328	5.20647	4.92959	5.675166	5.14919	5.140659	3.818077	4.301737
SPRYD3	23.33151	23.71435	21.11984	24.59604	22.56715	23.17537	22.50783	22.78681
SPRYD4	7.792879	10.30144	8.416299	9.306291	8.228605	7.705449	9.201807	5.904245
SPRYD7	14.60799	15.21126	13.49526	14.64513	14.16297	15.58305	12.68303	11.49513
SPSB1	11.65624	7.15611	7.829009	9.545398	9.515869	8.878214	9.60498	10.10151
SPSB2	2.225499	2.153143	1.920573	2.58197	2.720407	2.395466	1.933277	1.61324
SPSB3	18.27278	18.29777	16.14821	19.99081	15.99231	16.55157	17.04034	17.22991
SPSB4	0.167012	0.338407	0.058211	0.281892	0.324241	0.247283	0.219375	0.36867
SPTAN1	95.87637	84.39273	85.8691	89.06717	92.27354	84.71977	89.35858	92.75638
SPTB	2.438067	1.973688	1.946887	1.585944	2.049485	2.012325	1.858939	2.397842
SPTBN1	66.49414	61.20411	64.53438	62.62576	68.64743	62.89047	68.13469	74.59056
SPTBN2	50.44268	43.11404	43.1405	45.35287	48.72288	48.14627	45.44642	47.21361
SPTBN4	0.847066	0.990721	0.781606	0.868048	0.683628	0.783703	0.849814	0.693271
SPTBN5	0.13969	0.162751	0.13565	0.245741	0.300372	0.159397	0.087434	0.23613
SPTLC1	39.28687	42.52419	43.49664	37.35763	40.53122	40.67703	44.92808	39.65605
SPTLC2	13.82452	13.25376	13.31639	13.35086	14.00884	13.20485	15.15596	16.24673
SPTSSA	22.64482	24.05487	19.65077	22.46974	20.87311	19.77943	21.52652	17.5896
SPTSSB	52.55489	63.04019	86.16814	69.07933	69.16412	86.49489	43.23707	64.04616
SPTY2D1	12.05435	10.04035	12.40081	13.51933	12.72832	14.7911	11.65484	11.35612
SPTY2D1O	0.550645	0.759655	0.752041	0.900454	0.808498	1.179589	0.868086	0.522836
SPX	0.851786	1.227028	1.419479	0.572831	1.291155	1.433809	0.351229	0.458953
SQLE	81.7075	85.55722	107.9733	123.0208	93.05962	125.3643	92.82808	58.48417
SQOR	17.84659	22.3925	18.23777	22.71358	22.68006	16.04499	19.07597	22.16502
SQSTM1	163.0057	144.8046	161.1417	166.935	166.8564	142.8615	155.3154	149.8128
SRA1	10.47929	12.70119	10.58258	9.419255	12.31623	12.07092	11.4928	10.53839
SRARP	0.091269	0.030822	0	0	0.060854	0	0	0
SRBD1	10.86576	10.87804	9.744012	10.34399	9.285522	9.743903	10.30681	9.477885
SRC	4.741247	4.413857	3.480414	4.63735	4.57853	3.410499	3.924752	4.883545

SRCIN1	1.773906	1.721473	1.208006	2.031473	1.557777	1.354369	1.049967	1.392253
SRD5A1	7.804833	5.988275	7.473768	5.311889	6.241291	5.644588	6.531496	5.79759
SRD5A2	0	0	0	0	0	0	0	0
SRD5A3	3.034489	3.552989	2.352655	2.495906	3.121402	2.854218	3.694656	2.895455
SREBF1	12.01139	10.86908	11.48111	15.40864	13.27181	12.4422	12.45896	9.841895
SREBF2	47.60559	45.56502	44.59355	53.36196	44.39351	48.41437	49.71711	43.71529
SREK1	11.67989	11.223	10.26157	11.61946	12.35602	10.37309	10.60396	12.64839
SREK1IP1	4.871364	6.490987	5.542724	6.131515	6.937296	6.791865	5.202405	4.983674
SRF	15.27799	14.76794	11.6888	14.95252	14.59298	13.11104	15.36038	12.12945
SRFBP1	5.093883	4.168992	4.690278	4.216917	5.148932	3.267025	3.495926	4.98254
SRGAP1	1.897003	2.113163	1.710987	1.816057	1.970336	1.477691	1.724567	2.316221
SRGAP2	8.708023	7.893643	7.590585	6.650392	7.547292	7.094763	7.459221	7.453603
SRGAP3	0.859713	0.677805	0.57132	1.046612	0.863057	0.937176	0.961182	1.012907
SRGN	25.52758	50.15497	41.37059	48.58268	44.56506	32.99949	38.1717	69.05031
SRI	65.33106	71.88773	81.59205	74.94223	72.76052	78.35603	71.18014	66.54111
SRL	0.427707	0.514207	0.49559	0.62531	0.855525	0.505272	0.636714	0.775049
SRM	8.897921	6.820189	7.927221	9.140088	9.812341	8.595228	6.681935	10.74766
SRMS	5.847778	4.825621	4.346709	5.670445	5.211799	4.637377	4.738823	5.213562
SRP14	78.16254	91.42001	99.58085	85.90776	93.65453	104.5171	89.68061	87.34649
SRP19	32.54421	38.1376	38.87199	35.61088	40.88608	40.15054	38.44084	35.73875
SRP54	25.10735	27.65658	26.30518	27.17552	26.64052	29.5417	29.23042	27.75699
SRP68	38.21641	36.33391	35.9303	35.89366	37.50852	34.51355	38.74372	38.85224
SRP72	26.68501	28.47265	27.56965	28.33734	27.13996	29.79645	29.07086	25.63315
SRP9	28.25219	33.57635	37.12892	28.99957	34.83237	35.73564	32.24084	32.29502
SRPK1	21.32369	20.27654	17.29251	18.95858	20.56957	20.23853	20.22546	20.41097
SRPK2	32.81464	34.49442	30.66337	31.81938	28.23373	31.52635	30.17108	29.72434
SRPK3	0.627437	0.760042	0.5388	0.810713	0.532027	0.755322	0.346114	0.581033
SRPRA	53.19605	56.31967	55.23519	59.35414	57.39075	54.61111	59.97039	54.55333
SRPRB	12.06498	13.4243	15.89486	14.64493	15.04365	18.49959	15.30744	14.7482
SRPX	23.30691	28.71907	19.76209	24.48019	24.84602	23.30098	29.54415	25.33258
SRPX2	3.447756	3.286775	3.693765	3.806945	3.740854	3.945252	3.460733	3.148444
SRRD	5.507545	5.925389	5.08227	5.65425	4.665464	5.141151	4.867205	6.003114
SRRM1	44.34351	45.10604	40.39753	43.49127	41.6398	43.03859	42.04909	40.5279
SRRM2	45.71358	38.37732	35.22103	40.94666	41.82034	33.95713	33.31015	43.91777
SRRM3	0.457747	0.379436	0.246569	0.622605	0.424511	0.335181	0.329453	0.405175
SRRM4	0.112433	0.040203	0.071844	0.088108	0.185207	0.099883	0.114119	0.234775
SRRM5	0.263676	0.186995	0.27341	0.121593	0.309859	0.139375	0.113743	0.247372
SRRT	25.05285	22.49158	20.42209	22.58574	22.93553	21.07384	21.62709	26.81013
SRSF1	65.48276	66.81294	61.59864	62.293	62.94593	63.12839	65.39555	65.65319
SRSF10	20.45362	23.117	17.87732	21.24844	21.83173	20.01382	20.2966	22.6165
SRSF11	28.87619	29.03155	24.56423	30.50899	26.73913	25.69399	25.66574	29.64205
SRSF12	1.16901	0.844579	0.681311	1.531826	1.197911	0.984046	0.982304	1.360584
SRSF2	69.14495	73.44864	63.92594	81.60844	72.61764	74.21238	80.95336	78.97736
SRSF3	144.4021	163.3947	162.0258	153.8423	156.4294	162.6153	146.8613	145.286
SRSF4	10.73611	11.06271	9.955851	11.19324	11.35294	10.43046	10.5915	11.90148
SRSF5	32.65403	38.33732	31.58284	45.00802	32.5262	37.77006	31.68893	35.28047
SRSF6	68.54104	72.6671	67.33272	77.81739	77.23666	68.59738	63.80047	78.58139
SRSF7	78.44604	82.63524	79.8885	81.5143	82.86437	80.37574	80.0911	76.94833
SRSF9	95.66932	98.82885	100.7626	92.8521	95.13035	102.8571	106.006	104.6555
SRXN1	10.17416	11.13562	11.86319	12.47792	15.14062	14.84098	12.34858	11.09603
SS18	39.38584	41.6665	42.93614	41.56613	40.61152	39.59166	40.0495	40.14676
SS18L1	1.209633	1.203361	1.172973	0.963301	1.275381	1.437999	0.998494	1.175118
SS18L2	38.37767	43.42443	48.60137	37.52846	40.55029	45.48741	37.43616	41.27384
SSB	57.86127	65.21795	68.23909	64.46017	63.45946	69.50303	59.68479	56.9642
SSBP1	11.85794	13.19575	11.33646	11.81233	11.46521	11.52617	11.92378	13.89793
SSBP2	13.32724	17.43447	17.03297	16.22691	15.53246	14.80655	14.1796	16.16959
SSBP3	28.50413	28.21157	26.37847	28.8486	26.47631	26.94178	25.52454	28.37794
SSBP4	9.099995	9.57566	8.653256	11.54505	10.83138	8.737332	9.436357	10.44207

SSC4D	0	0	0	0	0.009891	0	0	0.010031
SSC5D	0.450561	0.291199	0.583034	0.246782	0.512774	0.148606	0.362702	0.480612
SSH1	3.838257	3.622141	2.700127	3.862397	3.149924	3.167548	3.959063	4.173719
SSH2	2.917019	2.592859	2.307709	2.448761	2.921983	2.195783	2.946808	3.556301
SSH3	19.04916	18.56396	18.31414	21.24589	20.45277	21.79742	20.05101	17.71542
SSNA1	18.85449	20.18877	21.21988	20.5541	22.49826	23.72912	20.32514	22.15618
SSPN	3.911753	5.399787	5.210022	4.464317	5.745191	5.233152	4.805988	4.710953
SSPO	0.010942	0.007918	0.007718	0.01762	0.012507	0.017312	0.00476	0.003171
SSR1	54.94434	56.60823	52.06043	53.39552	50.14678	55.00738	57.71411	55.79105
SSR2	99.46059	109.0258	112.6184	102.0023	107.0686	104.2316	103.3758	104.0707
SSR3	54.54425	58.79213	60.40847	57.49162	60.18943	64.42651	62.06313	57.62785
SSR4	293.547	302.2974	329.0885	277.6984	281.2426	313.1864	326.4022	309.9411
SSRP1	83.69707	85.68066	80.49719	76.51855	83.11495	82.97031	89.09088	84.41384
SSSCA1	20.48289	21.71011	20.04489	21.68571	23.509	22.69338	20.01781	18.04209
SSTR1	0.023344	0.110367	0.122949	0.03987	0.108952	0.062675	0.102674	0.071028
SSTR2	3.629168	4.850792	3.15639	4.345433	3.005594	5.692487	3.179533	1.96311
SSTR5	0	0	0	0	0	0	0	0
SSU72	52.01739	52.30099	50.05098	49.87475	55.67915	54.09133	48.37749	47.47544
SSUH2	0.135113	0.231653	0.236067	0.234316	0.29105	0.313929	0.200435	0.115954
SSX2IP	3.268327	3.462337	2.694724	3.358276	3.536392	3.409914	3.197126	3.621867
ST13	104.7892	113.55	117.6927	105.7857	103.0202	112.6753	101.6326	104.6439
ST14	15.41461	11.70252	13.0125	14.29495	14.69897	12.10978	12.41175	13.81211
ST18	0.019632	0.02652	0.045238	0.013412	0.01309	0.006589	0.013285	0.006637
ST3GAL1	4.710339	4.019851	5.035971	4.758255	5.708613	3.017556	3.936488	4.3237
ST3GAL2	10.50307	10.12043	8.793823	12.44012	10.60592	9.61696	11.02756	9.970721
ST3GAL3	5.375402	4.794939	5.235204	5.98976	7.143412	6.295483	5.34315	5.765254
ST3GAL4	11.84536	10.63784	9.656323	10.323	13.0447	14.22233	11.64003	12.62966
ST3GAL5	10.7197	9.430828	9.681424	8.319857	8.003956	8.789138	8.20847	7.849847
ST3GAL6	3.62221	4.170493	4.525386	5.209731	4.286483	3.931319	4.601054	4.28249
ST5	4.024496	3.565035	3.655043	3.993761	3.478027	3.674496	3.302804	3.764315
ST6GAL1	4.48898	3.817951	3.29569	3.444641	4.886013	3.675584	3.35199	5.060872
ST6GAL2	0.262721	0.34697	0.273346	0.240383	0.394132	0.259792	0.642845	0.437757
ST6GALNA	0.373451	0.816444	0.795827	3.021327	0.55042	0.613477	0.538654	0.498378
ST6GALNA	8.327762	7.001757	9.951523	12.37184	10.73139	9.86065	8.390159	9.845702
ST6GALNA	0.098459	0.217639	0.229821	0.284348	0.143231	0.198263	0.317977	0.15433
ST6GALNA	2.731932	2.993999	2.749774	4.037888	4.072155	3.42502	3.092862	3.436885
ST6GALNA	0	0.139917	0.136384	0.047175	0.230204	0.092699	0.560704	0.280141
ST6GALNA	9.124771	7.49312	8.060688	8.748106	8.038679	8.011803	7.986357	7.889966
ST7	4.726323	6.130309	5.39579	7.133982	5.968883	5.811871	6.768049	5.549285
ST7L	2.018945	2.432964	2.013497	2.321184	2.707799	2.782875	2.467853	2.462621
ST8SIA1	3.251194	3.674486	3.496077	2.769035	2.543485	2.880596	2.390639	3.458686
ST8SIA2	1.667353	1.045472	1.024091	0.896001	1.637064	1.300003	1.651086	1.830294
ST8SIA4	5.211705	5.535004	4.370948	4.662019	4.734791	4.539051	4.330927	5.530662
ST8SIA5	0.01389	0.014073	0.027435	0.056938	0.138922	0.041956	0.014099	0.098617
ST8SIA6	0.994151	1.045936	1.170564	1.48898	1.376694	0.846951	2.561467	0.89196
STAB1	4.08707	3.792358	3.679088	3.548983	6.111899	2.680276	4.31388	4.921813
STAB2	0.006679	0.027067	0.01649	0.017112	0.00334	0.006725	0.00339	0.010161
STAC	0.280379	0.333461	0.529694	0.437233	0.713231	0.527768	0.303145	0.290553
STAC2	0.735589	1.946947	1.688935	1.140137	1.167899	1.101664	1.969218	1.464144
STAC3	0.570405	0.259054	0.135968	0.524065	0.11803	0.356463	0.219606	0.438881
STAG1	13.20037	13.8083	12.84657	13.04089	13.03773	12.6012	13.52346	13.07348
STAG2	24.48884	25.94994	22.44428	22.53261	23.1159	22.39539	24.15561	24.03742
STAG3	1.463	1.35659	0.80197	1.435711	1.029192	1.167161	1.724046	1.5467
STAM	13.54157	13.75877	12.97839	12.26967	12.81257	13.52619	13.36894	13.72446
STAM2	8.635807	8.650643	9.126872	8.591846	8.802798	9.07484	9.293587	8.793546
STAMBP	11.96749	14.28411	12.17609	12.57803	12.23378	12.89087	12.92475	12.7172
STAMBPL1	3.679661	3.006414	2.55539	4.111397	3.181538	2.461899	4.011958	3.623694
STAP1	0.084178	0.237573	0.095005	0.166364	0.174391	0.133183	0.140367	0.32321

STAP2	13.40429	14.53707	13.50632	16.77022	15.60402	15.28799	16.35223	13.18983
STAR	5.78917	18.14219	9.181172	10.20603	10.8929	10.8424	16.83128	27.01923
STARD10	9.886988	10.01674	8.964939	9.781881	8.539866	11.25767	9.907571	9.061427
STARD13	3.342796	2.760059	2.215305	2.882331	2.739385	2.920835	2.795063	3.813558
STARD3	4.611769	4.518262	4.733046	5.304698	4.829588	4.38086	5.048366	5.345623
STARD3NL	2.826854	2.892737	2.656024	2.484423	2.429419	2.412328	2.893287	2.953541
STARD4	10.85685	11.80526	11.82719	13.75249	13.14187	12.19172	11.7374	9.726491
STARD5	52.96762	39.80006	41.68617	44.83231	41.01316	46.72339	42.12878	41.48634
STARD6	0	0	0	0	0.043739	0	0	0.044356
STARD7	23.81181	24.02003	22.62567	27.51337	26.5258	24.98493	24.85679	24.32478
STARD8	0.839339	0.963735	0.740467	0.825725	1.091282	0.647889	0.914404	0.913715
STARD9	2.954443	2.286353	1.747607	2.661458	3.050749	2.315702	2.586454	2.912577
STAT1	20.56744	24.2695	20.8649	21.42007	22.78211	22.825	23.27894	23.17452
STAT2	9.515959	9.990109	8.023879	8.606567	9.243741	9.186	9.085572	9.838305
STAT3	36.10417	29.86064	31.39745	30.29899	39.0701	26.9064	34.00861	40.75051
STAT4	0.319998	0.9834	0.421348	0.699572	0.821435	0.236268	0.47637	1.103482
STAT5A	9.329721	10.69398	8.603242	8.491588	9.432241	9.301665	11.93733	9.976246
STAT6	27.9088	25.80285	24.50913	30.08252	28.47504	24.69369	27.02971	29.72881
STAU1	55.20875	50.34208	53.24734	53.49276	51.76966	55.36951	54.50982	53.53353
STAU2	10.02246	9.836538	8.118291	9.756005	10.46619	9.009925	8.844488	10.07167
STBD1	6.887501	6.028721	6.22343	7.800671	6.544338	5.615441	6.173609	7.586856
STC1	2.777828	3.042669	2.276438	2.958415	6.741831	3.433774	4.863999	4.115429
STC2	5.022841	3.770954	10.69123	10.49275	10.62738	9.644189	4.922151	1.846954
STEAP1	11.07581	9.737004	14.66411	15.58088	11.12164	14.6377	12.39092	11.88633
STEAP2	9.46835	8.258355	10.39082	9.319315	7.764219	9.770313	8.822409	7.620646
STEAP3	4.612163	4.359323	4.50718	5.543492	5.795166	5.045062	4.883669	6.058161
STEAP4	4.207812	5.084147	7.641955	5.791105	4.252771	4.135953	6.040153	2.905198
STIL	2.11608	1.969701	1.738501	1.883008	1.985934	1.700848	2.286197	2.392687
STIM1	16.06935	14.10576	14.53595	13.52201	13.93728	13.61728	15.88732	15.91321
STIM2	8.57967	9.421148	8.563465	9.545136	9.307964	9.393458	9.236616	9.183085
STIP1	98.51924	80.6201	91.12922	80.49899	97.24706	86.15143	83.09799	93.68576
STK10	4.408071	5.889303	4.255149	5.031073	4.951886	3.649408	5.062623	6.536725
STK11	19.24034	16.4188	14.8161	18.89199	17.51761	17.08279	17.63441	17.31608
STK11IP	1.863983	1.903345	1.415968	2.535571	2.000272	1.780475	1.746409	1.767467
STK16	14.31264	11.53255	12.34892	13.84375	13.00861	11.86542	12.47142	13.22604
STK17A	58.14711	58.44018	61.2053	60.12924	57.24815	69.33198	52.20729	61.36057
STK17B	15.66558	17.33737	16.95848	15.99246	17.96552	17.33484	16.59716	18.21891
STK19	11.65611	11.36831	9.398457	12.1306	11.49445	10.95092	9.03421	9.652522
STK24	28.02026	29.396	26.85437	26.19083	25.74142	26.76541	28.14391	28.63989
STK25	23.19994	20.38424	23.56737	22.38428	22.14456	23.98678	21.02789	22.26425
STK26	20.02075	20.09024	18.70951	18.67737	19.72849	19.29325	19.08895	18.03686
STK3	13.29005	14.93258	14.13646	13.98024	14.65297	15.83937	12.87181	12.87836
STK31	0.018104	0.018342	0.017879	0	0.009053	0.082026	0.018376	0.100991
STK32A	0.137523	0.172501	0.439763	0.469764	0.406072	0.270331	0.345641	0.332096
STK32B	0.111289	0.075166	0.045793	0.057023	0.120579	0.102712	0.02824	0.065843
STK32C	1.439361	1.111736	0.971074	0.890851	0.954947	1.162225	1.6056	1.19968
STK33	0.072997	0.137345	0.092684	0.106865	0.052148	0.073496	0.232861	0.03173
STK35	6.224394	5.791017	4.676843	5.604134	5.524349	4.980331	5.248529	5.876342
STK36	1.535741	1.443063	1.232965	1.381564	1.412829	1.103608	1.231553	0.921483
STK38	16.94101	15.83835	14.30453	14.90616	18.60102	15.89309	15.1808	18.79397
STK38L	12.18665	11.60985	10.43016	10.6692	12.39888	11.33577	12.16583	12.16176
STK39	4.235203	4.734966	3.325163	3.612265	3.70957	4.246479	4.25423	3.833039
STK4	8.970586	8.627967	7.443251	8.237508	8.606317	8.126324	8.030595	8.993764
STK40	10.78967	10.02529	10.1331	11.56529	11.87474	10.17333	9.103071	11.03595
STKLD1	0	0.384874	0	0	0.024512	0	0	0
STMN1	139.6493	167.5968	145.9513	149.6537	124.1538	137.4798	151.26	144.0434
STMN2	14.85759	9.974435	12.84522	7.909965	4.999204	10.5659	7.315984	5.93801
STMN3	0.136866	0.196439	0.146424	0.046752	0.011407	0.149285	0.115766	0.034704

STMN4	0.033301	0.050606	0.065771	0.051188	0.016652	0.117348	0.152101	0.168873
STMND1	0.023422	0.023729	0	0	0	0.023582	0.047546	0.023755
STMP1	88.05686	91.81907	101.9633	94.98378	103.4799	114.2484	82.09336	94.03481
STN1	14.64853	15.1058	14.6304	13.76856	13.40181	14.2937	14.47001	13.42201
STOM	46.9535	50.70499	48.19007	53.6937	54.14072	47.05938	45.78553	52.98711
STOML1	0.625144	0.741481	0.516972	0.708338	0.426983	0.588477	0.784124	0.747449
STOML2	34.22088	37.90267	37.07242	36.71443	41.42161	38.12005	38.47279	37.33611
STOML3	0	0	0	0	0	0	0	0
STON1	4.361189	4.262354	3.902917	4.327683	4.069552	3.840104	4.372689	4.412498
STON2	8.039191	9.487073	8.30558	8.618703	11.36722	8.325912	11.03549	9.735754
STOX1	0.989591	0.548674	0.918916	1.024194	0.763216	0.8179	0.83453	0.773981
STOX2	3.595192	3.451406	3.216694	3.434546	3.225515	3.071235	2.911829	3.280335
STPG1	0.875106	1.030752	0.07026	0.3208	0.341549	0.050143	0.426063	0.671085
STPG2	0	0	0	0.020921	0	0	0	0
STPG3	0	0	0	0	0	0	0	0
STPG4	0	0	0	0	0	0.080286	0	0
STRA6	3.545512	5.474769	2.897656	7.124678	7.328338	3.569733	1.398113	2.926387
STRA8	0	0	0	0.021996	0	0.021611	0	0.02177
STRADA	7.813871	7.927562	6.576138	7.184704	6.764464	6.234008	7.149692	8.137037
STRADB	3.976672	3.874401	3.575814	4.192353	4.002599	4.477136	4.023427	4.35543
STRAP	73.88846	75.7364	75.05715	78.17289	78.48265	82.05592	76.95037	72.70506
STRBP	4.841105	4.131839	4.055768	3.937756	4.631221	4.735157	4.409531	4.705081
STRC	0.004914	0	0.009705	0.010071	0.019658	0.004947	0.024938	0.009968
STRIP1	15.96277	14.64924	13.83308	13.4463	14.13979	13.98957	15.11736	12.56775
STRIP2	1.569376	1.12374	0.570987	1.305947	0.908703	0.671244	1.676754	1.154892
STRN	14.47391	13.20525	12.99915	12.96766	12.35253	12.30895	12.91125	14.34407
STRN3	28.58482	29.12455	29.65245	30.42353	29.8003	31.24035	26.97996	29.17024
STRN4	19.46759	17.66404	15.41637	18.80186	17.02134	15.38723	17.10388	18.63292
STS	2.149532	1.432725	0.983168	1.310072	1.34646	0.831515	0.953091	1.732631
STT3A	29.54607	31.4188	28.69312	32.39803	31.27532	29.8397	32.54472	32.04364
STT3B	23.43572	24.47169	27.29787	23.35701	25.39408	27.13266	26.66785	26.01451
STUB1	65.6893	55.61983	57.43324	70.21344	63.02153	60.64104	58.46078	58.06656
STUM	6.454122	3.78734	6.573539	4.529385	4.441777	5.83338	4.680783	4.364001
STX10	1.876007	2.468632	2.108167	2.806374	1.833118	2.713826	2.407543	2.711913
STX11	4.598478	3.252621	3.194321	4.675279	4.321439	4.022296	5.782255	4.957732
STX12	22.71908	23.43815	27.59483	22.00461	23.94545	24.74884	23.70801	21.74719
STX16	19.9287	18.29942	15.60622	19.14928	18.5848	17.38876	16.51523	21.02699
STX17	8.000333	7.031373	9.160801	7.751685	8.589468	9.778755	7.158078	7.039088
STX18	6.971201	7.327013	8.543588	7.935268	7.045163	8.300707	7.096954	7.112779
STX19	7.428073	7.726083	7.565471	7.20641	4.529614	8.486934	5.116929	4.345524
STX1A	0.035564	0.036031	0.011707	0.048594	0.035569	0.059679	0.012033	0.036071
STX1B	0.856889	1.259947	1.018449	1.119012	1.653326	1.198677	1.062163	0.930615
STX2	3.92821	4.450838	4.069269	4.184343	5.072589	3.664471	4.301764	4.564133
STX3	5.304944	4.395349	4.045732	5.297533	5.389846	5.237974	5.02778	4.752929
STX4	10.47447	12.45232	10.80805	10.39126	11.41585	10.9282	10.28301	10.68775
STX5	17.49131	17.44057	16.83318	17.5938	16.34065	18.35361	18.50251	15.93198
STX6	18.54928	16.31593	18.47297	19.67306	17.75927	17.29813	16.70308	18.05273
STX7	27.51136	32.44564	28.59653	30.87295	27.38916	28.69942	27.98784	26.35992
STX8	17.38274	13.96504	10.93147	13.86679	14.20686	15.61574	15.33659	15.41042
STXBP1	3.543203	3.820832	3.125915	4.865645	4.663087	3.395139	3.777254	3.123649
STXBP2	21.1763	19.91549	20.56961	23.12385	19.09585	19.68985	19.32106	18.57298
STXBP3	25.49445	27.32488	32.47969	30.02594	28.18373	31.28162	28.27981	23.19806
STXBP4	2.692291	3.078705	2.619258	2.376547	2.472696	2.140366	2.529754	2.581921
STXBP5	3.747004	3.185647	3.349728	3.35067	3.55355	2.997656	3.349004	3.894143
STXBP5L	0.127863	0.113348	0.063135	0.055688	0.111894	0.096552	0.181693	0.184798
STXBP6	3.746172	4.913547	4.821524	4.471032	4.753124	3.63449	4.540432	5.379883
STYK1	0.48981	0.578945	0.921347	0.979984	0.769802	0.457931	0.887781	0.343017
STYX	4.709107	5.404896	5.031205	4.825754	5.47465	4.83674	4.818239	5.942934

STYXL1	0.423204	0.292335	0.626896	0.729382	0.538693	0.910294	0.370977	0.585312
SUB1	26.30059	35.54159	35.77473	31.44267	29.52505	33.84465	39.41979	35.9064
SUCLA2	19.20442	18.10909	20.91707	18.4423	18.45034	22.95951	22.92287	20.83903
SUCLG1	87.63043	95.87507	95.92224	94.48212	93.50322	96.21244	97.71472	90.60433
SUCLG2	34.97431	46.31314	41.26868	43.0486	43.14339	46.74713	43.98217	43.71495
SUCNR1	0.24786	0.27043	0.320087	0.195385	0.533922	0.134375	0.387044	0.560791
SUCO	12.63627	12.40032	11.12764	10.71909	12.66765	10.50151	12.77047	12.42398
SUDS3	11.76374	12.68848	10.81042	11.73315	11.5013	12.05923	11.78712	11.60616
SUFU	6.653883	5.627879	4.948578	5.259015	5.863427	5.183551	5.220029	5.595045
SUGCT	0.315775	0.222974	0.349639	0.225537	0.344527	0.327567	0.553611	0.339681
SUGP1	19.87732	20.27182	16.29508	21.14014	18.89051	19.68111	19.76046	20.88268
SUGP2	10.05555	10.69317	8.301789	11.49806	10.36516	9.212814	8.045906	10.6652
SULF1	3.278866	2.561232	1.909957	2.722156	3.169981	2.286964	2.387514	2.650795
SULF2	69.88118	51.85955	52.80467	49.7678	52.8526	62.94233	63.18399	64.48722
SULT1C2	0	0	0.020673	0	0	0	0	0
SULT2B1	48.80098	40.76089	45.5932	48.86454	46.547	41.98301	46.81057	42.34212
SULT4A1	0.989342	1.013981	1.215588	1.54435	0.943448	1.204586	0.583827	0.898416
SULT6B1	0	0	0	0	0	0.021014	0	0
SUMF1	18.99404	22.08293	16.81341	19.95883	20.16993	18.97942	19.04078	20.56019
SUMF2	12.06944	14.839	14.97642	14.60696	14.53864	14.66772	12.34619	13.45263
SUMO1	18.35986	19.54851	19.07297	33.08069	31.56984	27.6079	21.18574	32.98246
SUMO2	222.7057	253.937	255.2412	238.972	235.7835	250.5955	237.679	235.7228
SUMO3	34.54055	41.35322	37.07406	42.72492	35.93436	42.82312	44.54573	36.54557
SUN1	13.39521	12.87057	11.55446	14.32695	12.40437	11.47143	12.18135	12.98366
SUN2	66.91904	68.63956	59.21069	62.01092	56.41239	50.4624	54.174	53.48113
SUN3	0	0	0	0	0	0	0	0
SUN5	0	0	0	0	0	0	0	0
SUOX	7.739093	9.007225	9.195249	10.86938	11.37207	8.940134	8.810387	6.748795
SUPT16H	43.30804	41.67051	39.95843	40.14445	38.49903	41.38167	43.50356	41.37742
SUPT20H	20.78455	23.00902	19.04117	20.4938	23.02493	16.77776	17.65041	23.74396
SUPT3H	2.262282	1.89754	2.29644	1.649781	2.094197	2.118825	2.157375	2.059702
SUPT4H1	38.4348	36.55852	38.92546	37.1767	36.79787	44.27187	40.75932	36.51701
SUPT5H	28.82748	28.57074	24.64964	26.54637	25.87784	26.75082	27.48146	26.84797
SUPT6H	29.83906	26.26239	24.37352	26.72871	28.29013	26.38436	25.80329	26.39172
SUPT7L	3.758585	3.359504	3.226103	3.635704	3.829334	2.999116	3.772179	3.733712
SUPV3L1	11.04662	11.14719	9.296443	8.950636	12.06737	10.89038	11.29019	9.269877
SURF2	3.85534	4.603061	3.647416	4.375994	3.359944	3.188885	3.861817	4.413113
SURF4	22.28698	25.42078	21.61363	24.29989	25.90544	23.47114	26.52705	26.37477
SURF6	10.19275	9.604933	7.860599	8.837514	8.162957	9.642184	7.805637	8.297616
SUSD1	3.446228	3.282896	2.898818	3.711297	3.534365	2.993829	3.610133	3.974729
SUSD2	9.928682	10.56983	9.406614	13.37826	11.11276	9.830363	10.42191	12.45038
SUSD3	3.519105	2.238671	3.131772	3.606275	3.765111	2.471963	3.883394	5.540564
SUSD4	1.653089	1.818337	1.464578	1.277788	2.14457	1.417103	1.65871	1.896978
SUSD5	0.429108	0.123039	0.095946	0.348471	0.226728	0.105972	0.501287	0.312042
SUSD6	15.07369	12.44418	12.78792	14.126	13.47246	12.74607	12.60934	11.79185
SUV39H1	6.880628	6.940752	5.637899	7.061238	5.05439	6.267859	8.19316	5.719816
SUV39H2	4.519746	4.073582	4.173596	3.81965	3.747374	4.205879	3.92225	4.008596
SUZ12	10.42222	12.22555	11.56128	12.23887	11.29893	12.31212	11.44803	11.73531
SV2A	0.031392	0.07633	0.080602	0.115811	0.08163	0.025285	0.133824	0.044575
SV2B	0.009826	0.014932	0.019407	0.020139	0.004914	0.004947	0	0.004983
SV2C	0.003966	0.008035	0	0.016255	0.003966	0.011978	0	0.004022
SVBP	23.96434	33.4442	28.04153	28.41759	26.81103	26.58175	29.1211	27.41389
SVEP1	7.666608	7.909337	5.533815	6.852762	7.849009	6.267716	7.754604	9.377502
SVIL	6.262502	7.436162	6.131407	6.219398	7.751889	6.500042	7.229052	7.749506
SVIP	0.578742	0.808741	1.241602	1.145254	1.25743	1.165392	0.972287	0.414935
SVOP	0.01702	0	0	0.008721	0.017022	0.008568	0.025913	0.051786
SVOPL	0.00831	0.151549	0	0.076646	0.008311	0.008367	0	0
SWAP70	30.7024	33.76738	28.21391	31.93119	33.38187	34.16151	33.30811	29.55594

SWI5	49.16326	50.69462	51.7538	52.50983	51.61169	52.47127	46.31259	46.3886
SWSAP1	6.276513	6.436433	6.903805	7.713152	5.05248	6.730408	7.354746	7.970267
SWT1	0.737209	0.778266	0.688257	1.025281	1.062586	0.726652	0.820579	0.898501
SYAP1	27.53905	31.07192	29.43866	28.2841	30.24366	31.70292	29.07354	25.83521
SYBU	2.012907	2.094243	2.18411	1.796126	1.532465	1.684637	2.560295	2.382433
SYCE1	0.025631	0	0.050623	0.13133	0.076903	0.258062	0.052031	0.155976
SYCE2	2.798916	2.430557	2.581797	2.080267	1.66111	2.074819	2.903338	2.77637
SYCN	0	0	0	0	0.060986	0.061395	0.061893	0
SYCP1	0.04879	0.024715	0.040152	0.066665	0.008133	0.032749	0.024761	0.041237
SYCP2	0.149582	0.238976	0.136356	0.224036	0.120832	0.098472	0.186863	0.245073
SYCP2L	0.021613	0.076639	0.010672	0	0	0.087044	0	0.032881
SYCP3	0.014904	0	0.058872	0	0	0	0.060509	0
SYDE1	2.589652	2.314455	2.565995	2.367867	3.287626	2.686353	2.708148	2.573456
SYDE2	2.302989	2.280186	2.303368	2.155543	2.587926	2.387888	2.03206	2.382223
SYK	1.440059	1.394687	1.79174	1.202687	1.776514	1.32854	1.384392	2.168319
SYMPK	9.939592	8.354451	7.816714	8.344111	8.396537	7.172473	7.948462	8.929528
SYN1	1.492521	0.818318	1.005737	0.998668	1.053681	1.025384	1.568377	1.567195
SYN2	1.52056	1.837615	1.480162	3.372444	1.292643	1.968361	2.061499	4.72576
SYNC	0.71621	0.750346	0.667099	0.975821	1.139572	1.147209	0.603041	0.932771
SYNCRIP	39.43438	41.28001	39.00537	39.79469	37.991	41.68916	41.75599	39.24247
SYNDIG1	1.698488	0.799799	0.66148	0.956082	1.196273	1.469233	2.306714	1.33446
SYNDIG1L	0.032659	0.011029	0	0.022312	0.010888	0.054804	0.198896	0.033124
SYNE1	2.634982	3.314733	2.30426	2.885117	3.334329	2.409817	3.333878	4.924065
SYNE2	16.34616	12.54637	11.47754	14.77505	17.64426	15.16355	13.71849	19.99411
SYNE3	9.632911	8.191157	8.362512	8.225359	8.149932	8.290235	8.200926	8.54002
SYNE4	0.049892	0	0.04927	0.051127	0.033265	0.016744	0.06752	0.033735
SYNGAP1	0.303706	0.263736	0.233273	0.35569	0.467671	0.296074	0.17615	0.244468
SYNGR1	4.480041	3.898456	3.170325	4.358053	4.308715	3.706769	4.47408	3.537204
SYNGR2	24.34225	30.95718	32.83283	36.12068	28.80725	27.60999	33.26063	33.25354
SYNGR3	0.36972	0.291334	0.446249	0.547268	0.424548	0.482541	0.486456	0.55553
SYNGR4	0.070591	0.25031	0.139422	0.03617	0.1059	0.035536	0.071649	0.071595
SYNJ1	3.625804	3.809081	3.089353	3.421504	4.193974	2.963482	3.17202	4.369563
SYNJ2	2.776218	2.399895	2.276067	2.63027	3.032697	1.863456	2.487043	2.79803
SYNJ2BP	6.127603	5.666692	5.254149	5.411456	6.207985	5.522758	5.152161	5.903516
SYNM	11.27464	15.49684	14.78749	16.06784	13.18164	13.06155	14.15975	11.13964
SYNPO	18.06974	15.0463	12.80646	14.92492	19.90018	15.75472	14.53083	15.8772
SYNPO2	7.545735	8.360567	8.152928	8.550799	8.482983	7.671976	7.913501	6.68576
SYNPO2L	0	0	0	0	0	0.005819	0	0
SYNPR	0	0	0.010822	0.01123	0	0	0	0
SYNRG	12.79236	11.64255	12.05835	12.53488	10.68878	11.6607	11.74227	13.40405
SYP	0.363774	0.433586	0.549427	0.635926	0.331719	0.678656	0.206334	0.607686
SYPL1	81.04732	88.5921	90.44843	85.59091	80.21628	87.7927	80.59265	75.31125
SYPL2	0.007713	0	0.02285	0.047422	0.023141	0.023296	0.007828	0.007822
SYS1	20.52024	20.70013	20.46066	22.7583	21.36146	23.14856	18.67805	17.20936
SYT1	0.344592	0.030096	0.052805	0.578398	0.308984	0.023927	0.012061	0.006026
SYT10	0.015939	0.161483	0.236108	0.16334	0.095647	0.08024	0.048535	0.145495
SYT11	1.000054	0.725486	0.615722	0.961565	1.123663	1.205777	0.858411	1.083162
SYT12	0.507983	0.333435	0.367408	0.527898	0.450804	0.432214	0.363101	0.471675
SYT13	0	0.11972	0.005557	0	0	0.118976	0	0
SYT14	0.148606	0.22843	0.364356	0.399097	0.2665	0.196055	0.712566	0.322232
SYT15	1.909385	1.888386	1.316923	2.733142	2.576488	2.563239	2.230268	2.781893
SYT16	0.007984	0.004045	0	0.004091	0.003993	0	0	0.004049
SYT17	0.251545	0.294053	0.267519	0.327176	0.309633	0.301967	0.166938	0.19625
SYT2	0.399193	0.358212	0.326639	0.151945	0.787083	0.218185	0.243109	0.613098
SYT3	0.11534	0.129837	0.126559	0.105064	0.147398	0.135483	0.162597	0.077988
SYT5	0.916944	0.883662	1.225764	1.065709	1.397962	1.249709	1.191748	0.839266
SYT6	0.008218	0	0.008115	0	0	0.008274	0	0.008335
SYT7	0.922154	0.631536	0.554538	1.029465	1.236567	0.881771	0.684995	0.559079

SYT8	0.149276	0.113426	0.147416	0.165721	0.19906	0.175345	0.088384	0.03785
SYT9	3.146128	2.273435	3.907989	2.322876	3.047968	2.610425	3.316436	5.197731
SYTL1	17.14242	16.46444	13.81084	18.4998	16.1774	14.74887	14.90703	12.22244
SYTL2	8.532784	7.900539	7.338312	9.043864	7.608731	7.9268	7.021131	7.288337
SYTL3	1.876682	1.801989	1.472957	1.392144	1.666821	1.410076	1.819541	1.931806
SYTL4	2.792749	3.579148	3.027656	2.852899	4.299804	3.273075	4.03288	3.654558
SYTL5	0.135705	0.051557	0.072591	0.133272	0.101792	0.051237	0.109045	0.057349
SYVN1	11.36629	10.06621	9.377998	10.87956	11.09197	10.36809	11.37934	9.55285
SZRD1	26.88229	24.64599	25.47837	26.69746	27.61021	26.91025	25.54249	25.54802
SZT2	3.08133	2.736018	2.024474	3.214503	2.77406	2.777417	2.654066	2.920599
TAAR1	0	0	0	0	0	0	0	0
TAB1	8.145783	8.784192	7.456191	8.434737	8.012119	7.287782	8.332732	7.729655
TAB2	24.18345	23.57883	22.47433	23.1651	25.7778	21.93302	23.17678	25.07637
TAB3	10.58357	10.23335	9.643259	9.968093	11.10978	10.44453	10.09037	10.65334
TAC1	1.496497	3.506066	1.016022	2.779587	1.356375	2.26008	3.275223	1.802388
TAC3	0.072417	0	0	0	0	0.036456	0	0
TAC4	0	0.009219	0	0	0	0	0	0
TACC1	11.40248	11.18072	11.75563	12.39375	12.1995	10.33826	12.08953	14.92515
TACC2	19.81341	16.67821	16.34042	17.99479	16.44682	16.89415	17.00757	18.48605
TACC3	9.847873	9.622091	7.746415	9.069736	8.024897	9.091902	8.782622	9.176989
TACR1	0.005785	0	0	0	0	0	0	0.005868
TACR2	0.021547	0	0.021278	0.01104	0.032324	0.032541	0	0.010927
TACR3	0.016058	0.016269	0	0	0	0.016168	0	0
TACSTD2	281.9971	289.6941	317.5737	310.2568	278.5521	297.4626	284.7596	284.1039
TADA1	7.48249	8.653199	7.333818	7.388406	8.028875	6.718072	7.06562	8.093315
TADA2A	13.87378	14.12176	13.02183	13.29373	14.73989	15.5123	13.7329	13.0534
TADA2B	7.376103	6.133287	5.385505	6.819044	6.497814	6.368649	6.405809	6.125518
TADA3	13.03064	14.24884	12.6389	12.15137	12.98586	12.7104	12.02373	12.88633
TAF1	9.850978	9.592032	7.859037	8.02046	9.170917	8.421766	8.796537	9.747812
TAF10	73.11675	75.67798	73.5895	77.76299	75.67754	77.92108	78.51679	71.7525
TAF11	38.00703	40.00291	39.40047	38.2804	39.03341	43.53955	38.66536	35.83715
TAF12	9.284588	10.28829	9.589142	8.444181	10.0596	9.562241	10.28771	9.41676
TAF13	15.2639	16.89669	19.62755	15.77371	18.97789	19.94627	16.71597	17.38782
TAF15	40.27809	41.20922	41.94091	37.99058	38.47466	39.90495	37.61446	39.23965
TAF1A	0.986603	1.368616	1.109214	1.322131	1.320701	1.1156	1.109245	1.400907
TAF1B	9.128361	10.20018	10.738	7.75871	9.330928	9.839478	8.161627	7.65172
TAF1C	3.437414	3.260056	2.518831	3.583144	3.437858	2.755112	2.65745	2.878164
TAF1D	41.80141	44.81311	43.33633	41.20185	40.04396	42.13287	40.36176	41.41015
TAF2	15.1709	14.58308	14.05077	14.58508	14.58463	14.57839	13.1108	14.67649
TAF3	4.123453	4.205271	3.86685	3.317706	4.025535	3.733156	3.663529	3.827169
TAF4	7.789204	7.546688	6.879555	7.46226	7.647896	8.122725	6.838506	7.749491
TAF4B	1.907313	1.879404	1.857746	2.014801	2.33872	1.808542	2.121559	2.345207
TAF5	2.943176	2.886325	2.663101	2.399491	2.950806	2.941386	2.884312	2.845378
TAF5L	4.768607	4.898759	3.90237	4.898118	4.908186	4.829163	4.896548	3.895123
TAF6	16.68409	16.25649	15.56587	16.19423	16.26074	16.34931	15.76222	15.86336
TAF6L	5.168202	4.67274	4.604656	5.78829	5.295249	4.580106	4.54031	4.588155
TAF7	34.4397	37.69248	35.1754	34.77655	33.31362	36.36975	36.19092	35.88951
TAF7L	0.010736	0.054385	0.010602	0.011002	0.04295	0.043238	0.032692	0.021778
TAF8	5.968478	6.695667	5.820097	6.535245	6.091905	5.693698	6.244725	7.228198
TAF9	32.78793	35.96809	33.47299	39.87508	32.72702	37.97464	38.98799	38.29794
TAF9B	11.78751	11.41755	11.23475	10.0157	9.939288	9.79899	10.47087	9.979432
TAFA1	0	0.013996	0	0.028314	0.013817	0.013909	0.014022	0.014012
TAFA2	0.05549	0.033731	0.043839	0.011373	0.077696	0.033521	0.078851	0.067536
TAFA3	0.401419	0.422636	0.139912	0.169385	0.338495	0.182269	0.07989	0.335287
TAFA5	0.100882	0.068137	0.21032	0.057434	0.156948	0.090286	0.102395	0.034106
TAGAP	0.887687	1.149588	0.663187	0.862214	1.096242	0.48962	0.948011	1.017758
TAGLN	267.9134	243.8999	298.1419	284.2172	256.3044	270.9279	296.0286	233.4552
TAGLN2	235.2747	226.2397	223.6128	185.7725	231.6787	195.1069	218.2989	209.9037

TAL1	0.771486	0.659747	0.704527	0.935113	0.991446	0.826869	0.762008	0.849777
TAL2	0.02763	0.11197	0.054571	0.014157	0.04145	0.013909	0.014022	0.014012
TALDO1	119.1183	125.0671	128.1978	126.8764	129.4241	131.4323	128.4731	126.9972
TAMM41	3.68702	4.091162	2.831951	2.923722	3.365572	3.918442	3.861129	4.125329
TANC1	8.037796	6.73967	6.481844	7.227817	7.045432	6.458603	7.522081	7.513513
TANC2	4.071947	3.108827	1.934937	2.568746	3.20762	2.321731	3.196059	3.785755
TANGO2	5.636803	5.89409	4.76938	6.674985	7.071275	4.274	4.845494	6.013486
TANGO6	3.252339	3.591872	2.19908	2.702352	2.835176	2.839425	2.936811	3.231774
TANK	9.161786	10.37848	9.517516	9.962376	9.946769	10.22008	10.57762	9.670712
TAOK1	7.553624	7.39704	6.917536	6.741696	8.036316	7.029356	7.319073	8.161408
TAOK2	8.935508	7.763527	6.662522	7.604716	9.247924	7.057279	7.86916	9.083754
TAOK3	2.463046	2.368113	2.178874	2.456898	2.501599	2.067477	2.876931	2.780601
TAP1	8.144502	10.30717	9.339096	11.6752	12.11128	11.43334	10.44907	9.789807
TAP2	11.88489	10.56115	11.10519	13.88145	11.40906	11.1299	11.93721	12.53818
TAPBP	36.35865	32.12775	36.19829	35.04825	35.6164	36.11767	36.95216	32.13896
TAPBPL	6.813914	6.639331	6.433545	7.04204	6.718268	6.559225	6.054056	6.294218
TAPT1	9.891666	11.53237	9.889363	10.46153	10.89449	9.748886	10.01079	9.374852
TARBP1	6.152003	5.740802	4.711584	5.788535	4.914219	5.108618	5.593178	6.149218
TARBP2	7.799294	7.290392	7.455065	7.283718	7.564821	7.363641	6.825927	7.358092
TARDBP	18.64988	17.3534	15.88766	18.06764	17.69419	16.62068	17.29449	20.88769
TARM1	0.10059	0.229298	0.099337	0.051541	0.075452	0.025319	0	0.051011
TARS	56.32109	50.61108	66.02558	61.55992	65.4275	65.24476	57.57346	53.63649
TARS2	13.29787	14.99526	12.33038	12.8627	11.97183	13.47709	11.91597	14.57772
TARSL2	3.382193	2.657139	2.450037	3.144279	3.352247	2.395943	2.497608	2.906546
TAS1R1	0	0	0.010709	0	0	0	0	0
TAS1R3	0.017143	0.028947	0.033859	0.023424	0.017145	0.023014	0.034801	0.023183
TAS2R3	0.118222	0	0.038916	0.030288	0.019706	0.019838	0.01	0.039968
TAS2R4	0	0.016583	0	0.016773	0	0	0	0
TAS2R41	0	0	0	0	0	0	0	0
TAS2R60	0	0	0	0.030065	0	0	0	0
TASOR	11.56068	11.82482	10.10742	11.53882	11.36066	11.28097	11.71334	12.31462
TASOR2	8.136181	7.069901	6.924036	6.536962	8.197567	6.720342	6.867698	8.218452
TASP1	4.857356	6.036554	4.509023	6.161255	4.836393	6.009929	5.740963	4.543331
TAT	0	0	0	0	0.017232	0	0.008744	0
TATDN1	16.41623	17.89713	21.19602	20.74124	18.76383	21.12245	20.07776	15.77089
TATDN2	4.962016	4.606616	3.428269	3.84753	4.462618	4.32156	4.165122	3.66446
TATDN3	5.671007	4.178497	4.401445	5.129781	4.341127	3.76743	4.878313	4.486693
TAX1BP1	51.38226	61.10398	56.32342	59.01625	58.68481	60.55663	53.88525	53.2328
TAX1BP3	94.52291	95.67577	96.89098	82.61171	99.66209	97.73842	101.5611	106.2012
TAZ	4.648994	4.629632	4.215007	5.414547	4.348085	4.840506	4.316107	4.473784
TBATA	0	0	0	0	0	0	0	0
TBC1D1	3.238019	3.426531	3.4254	4.174859	4.434833	3.806721	3.866862	4.273246
TBC1D10A	21.48855	20.09818	17.80181	21.46694	21.66665	20.85583	21.114	20.10542
TBC1D10B	12.42728	11.57736	11.33986	11.68605	12.40508	10.73043	10.32615	11.21983
TBC1D10C	0.447019	1.197956	0.455689	0.546755	1.052794	0.66785	1.09772	1.184645
TBC1D12	5.150422	4.723886	5.214688	4.744869	5.196616	4.655262	5.108868	5.368846
TBC1D13	8.359469	8.490969	6.952584	7.802454	9.450432	7.629778	7.71351	8.536642
TBC1D14	26.82858	27.36904	24.22987	26.93889	22.68497	27.32952	25.65845	24.8163
TBC1D15	11.12433	12.13926	11.86626	11.53042	12.13643	11.76462	12.39446	12.51432
TBC1D16	3.037839	3.396988	3.275016	4.160798	3.43771	3.191414	3.555775	2.60164
TBC1D17	16.48574	15.37203	13.50672	15.85751	16.29415	15.05987	15.26943	14.614
TBC1D19	1.306066	1.363084	1.243854	1.21741	0.873209	1.574383	1.322025	1.139569
TBC1D2	2.377798	2.863227	1.739385	2.896141	2.193942	2.281192	2.901034	2.273607
TBC1D20	19.88726	22.47434	17.57601	21.24314	23.24334	20.20605	19.47303	18.65424
TBC1D21	0.055369	0.112192	0.136698	0.226963	0	0	0.0281	0.028079
TBC1D22A	12.81784	14.14457	12.32986	13.59862	13.51101	14.47173	12.3758	11.85402
TBC1D22B	6.631939	6.268362	5.357095	5.957879	6.8711	6.293404	6.134861	6.565239
TBC1D23	29.53251	31.83387	42.85773	32.61168	38.21875	34.22596	22.80389	35.97775

TBC1D24	1.702958	1.867941	1.395656	1.883865	1.79795	1.611008	2.16269	1.894704
TBC1D25	3.409986	3.344187	2.864617	3.485134	3.837867	3.433281	2.584315	3.052729
TBC1D2B	6.020673	6.406377	6.050048	6.376617	6.232899	5.757021	6.41824	6.569356
TBC1D30	5.453825	5.08672	6.53415	7.049714	5.026342	6.67649	5.165273	3.651457
TBC1D31	4.312036	3.792861	3.725142	3.778223	4.028403	3.468904	3.821516	4.841744
TBC1D32	1.0708	1.124065	1.267675	1.269194	1.309642	1.675625	1.139241	1.347741
TBC1D4	6.423743	5.657265	4.809073	6.059426	6.004638	5.844399	5.817123	7.928862
TBC1D5	6.981598	7.716245	6.722413	7.690589	8.398532	7.387429	7.76593	7.944003
TBC1D7	10.28461	10.18169	11.16129	10.50728	8.923874	10.71737	7.118139	8.065383
TBC1D8	6.066818	5.953068	5.196883	5.93069	6.360756	6.410246	6.005605	5.925032
TBC1D8B	1.159027	1.452111	1.306225	1.31467	1.49985	1.331742	1.297645	1.875458
TBC1D9	6.403269	6.959109	7.875611	7.017416	6.742826	7.725759	7.734725	8.228059
TBC1D9B	20.84354	17.54263	18.54875	19.1892	19.07874	17.69062	18.55943	19.53549
TBCA	67.10986	76.25159	97.3974	71.58881	70.14231	83.52644	65.55924	61.14254
TBCB	33.41868	38.75296	33.61889	42.81505	32.52702	37.80161	37.8603	41.38368
TBCC	20.75075	21.06178	20.79397	19.36629	18.48039	20.1618	20.27691	18.03402
TBCCD1	3.528132	4.41609	4.091875	3.39481	3.600391	3.417993	4.976	4.285707
TBCD	7.91244	8.021492	6.930413	8.145314	7.671791	7.366031	8.401634	7.237693
TBCE	7.757529	7.63299	7.252289	7.814011	7.009575	6.573338	7.991475	7.565833
TBCEL	3.565563	3.643057	2.608433	3.333004	2.843727	3.396696	3.321731	2.986282
TBCK	3.618815	3.304547	3.451661	3.004763	4.148096	3.159576	3.526731	4.025524
TBK1	8.085594	9.442181	8.138121	8.566113	9.594569	8.870083	8.439432	8.867848
TBKBP1	1.70327	1.390236	1.15476	1.630556	1.388425	1.166566	1.241065	1.50007
TBL1X	34.18327	30.58641	33.44379	34.10903	34.74743	34.12962	33.50525	32.19737
TBL1XR1	18.44639	19.08444	18.33715	17.25954	18.82336	17.54356	18.99581	20.34824
TBL2	9.84876	9.04463	9.296764	10.1858	10.18745	10.13814	10.54307	10.71938
TBL3	7.97975	7.044886	6.252512	7.327229	7.107372	7.517697	7.25737	7.894058
TBP	8.878128	9.432696	8.127022	8.256178	9.326123	9.852973	8.777241	8.551365
TBPL1	3.842514	4.011963	3.451426	3.762128	3.696144	3.97226	3.726259	3.996507
TBPL2	0	0	0	0	0	0	0	0
TBR1	0	0	0	0	0	0	0.007106	0
TBRG1	23.53839	24.08704	22.24364	22.22776	21.62566	22.11093	23.53693	23.18778
TBRG4	17.20503	15.34616	14.00382	16.1577	16.98411	16.51135	18.4193	14.35724
TBX1	20.84404	19.23299	13.00511	18.3179	15.14222	14.5243	16.53024	19.50399
TBX15	11.05152	9.556785	10.50173	10.91061	9.699781	10.33768	9.696409	7.89364
TBX18	2.555639	3.024859	1.789711	2.965198	2.248762	2.449392	2.656329	2.766483
TBX19	0	0	0.144931	0.04297	0.020968	0	0.04256	0
TBX2	0.913518	0.805534	0.893782	1.733603	1.615782	1.439251	1.133269	1.878781
TBX20	0	0	0.020249	0.010506	0.020508	0	0	0.010398
TBX21	0.164521	0.093758	0.111699	0.358267	0.246814	0.175998	0.052184	0.281582
TBX22	0	0	0	0	0	0	0	0
TBX3	0.699063	0.84314	2.130232	0.94835	2.15073	1.669104	0.804156	0.911591
TBX4	0	0	0.025381	0.008779	0.017137	0	0	0.017378
TBX5	0.020773	0	0.274886	0.008515	0.120497	0.087841	0.029518	0.016855
TBX6	0.139539	0.14137	0.126317	0.119163	0.186076	0.163907	0.165237	0.1887
TBXA2R	3.136161	2.655021	2.375845	3.973281	2.8358	3.190027	2.801655	2.037023
TBXAS1	0.904759	0.340041	0.576442	1.181395	1.080014	0.411392	0.977578	0.873237
TC2N	3.342268	4.908946	5.448794	4.582689	5.695663	3.947884	5.221268	5.207867
TCAF1	18.66905	22.1021	19.86922	19.31857	19.56035	17.41321	19.11488	19.72905
TCAF2	1.947449	1.748303	1.036382	2.27841	1.271416	1.677533	1.608782	1.371647
TCAIM	1.770871	1.722665	2.530352	2.425001	2.162936	1.759301	2.083751	1.891448
TCAP	1.247262	1.429107	1.964889	2.054182	2.851254	2.063072	1.838673	1.551153
TCEA1	40.98989	43.54164	40.80808	40.57385	40.49014	42.38415	43.37144	41.27918
TCEA2	6.978379	9.982252	7.862577	8.003958	7.508875	6.778522	9.232926	8.535463
TCEA3	4.43881	4.621984	4.444384	6.022897	3.905014	3.869111	5.423158	4.043463
TCEAL1	5.070743	4.889439	5.402894	5.549607	5.138127	5.418872	5.541843	4.590289
TCEAL2	0.069886	0.070803	0.207044	0	0	0.175907	0.106401	0.248081
TCEAL3	0	0	0	0	0	0	0	0

TCEAL4	35.68861	42.8291	39.38042	38.59479	44.07168	40.95418	35.31465	34.12161
TCEAL7	0.010578	0.03215	0.031338	0.04336	0.074055	0.0213	0.118102	0.042914
TCEAL8	71.00333	89.3181	82.2113	84.59854	79.85612	89.36653	88.92407	74.78482
TCEAL9	1.947619	4.582868	5.087574	3.830767	6.220618	4.17487	3.18844	3.950688
TCEANC2	4.591076	4.119748	4.112868	4.65439	5.296818	3.450321	4.077449	4.406981
TCERG1	13.06079	14.47912	12.6078	15.34208	14.1893	14.69744	14.44378	14.55711
TCERG1L	0	0	0	0	0	0	0.015507	0
TCF12	29.88578	28.64643	26.41023	27.54966	31.09623	26.42334	28.29509	28.9738
TCF15	0.469384	0.369867	0.377695	0.641347	0.973662	0.822657	0.388198	0.634754
TCF19	9.97566	10.85317	8.395894	6.572774	7.834158	7.350804	10.88712	9.162651
TCF20	11.00872	11.42541	8.464784	10.22269	9.976876	9.152144	9.990781	10.80848
TCF21	0	0	0	0	0	0	0	0
TCF23	1.62206	0.594402	0.511228	0.282934	0.621295	0.625458	0.525444	0.472543
TCF24	0	0	0	0	0	0	0	0.056662
TCF25	48.28497	48.48565	48.09336	47.22846	46.00734	48.23368	48.07963	41.15708
TCF3	11.67406	10.05853	9.178704	10.63893	11.18469	9.727077	10.13391	10.61778
TCF4	13.16459	12.41549	11.33805	11.98717	13.89292	11.72999	11.22136	12.52689
TCF7	10.68163	9.870756	10.29653	12.05766	12.48783	12.09434	11.29515	9.659736
TCF7L1	14.37448	12.45148	11.57937	11.70026	12.95924	13.0771	11.77629	12.71507
TCF7L2	8.551315	7.619579	6.085781	6.959766	7.722608	6.656039	7.212613	7.614367
TCFL5	6.647587	7.098875	7.033668	7.68022	6.853804	7.494093	6.226274	6.065395
TCHH	0.00464	0.002351	0.002291	0	0.009282	0.002336	0	0
TCHHL1	35.32067	24.99198	24.57042	27.70391	26.25252	30.70144	22.99212	26.89182
TCHP	4.725319	5.309589	4.619305	5.096714	4.200827	4.613431	4.398938	4.347216
TCIM	9.422095	9.41617	9.578362	9.633639	9.913666	11.24639	11.33764	11.76151
TCIRG1	5.953857	5.48178	5.18444	6.997986	6.859891	6.045161	5.451099	6.18142
TCL1B	0.079515	0.120837	0.19631	0.122226	0.039762	0.120087	0.201768	0.12097
TCN1	0.796721	1.000198	2.548528	4.738997	2.113317	3.853875	4.430117	0.966162
TCN2	6.507328	7.608786	7.393962	7.052937	7.603073	6.297419	9.254683	8.727481
TCOF1	10.07448	9.61594	8.141607	8.430799	8.874775	8.178586	8.033981	9.544514
TCP1	185.1269	191.6659	199.0281	187.8635	174.2511	178.4418	192.768	191.9933
TCP11	0.080583	0.027214	0.026526	0.068816	0.12089	0.067611	0.040896	0.108974
TCP11L1	3.793764	3.308747	3.041878	2.984165	3.782652	3.095461	3.102911	4.400815
TCP11L2	19.32327	18.26834	23.83532	19.06249	25.48866	20.66125	21.02662	18.57379
TCTA	7.243152	7.354027	7.461216	7.598534	8.165212	9.17866	7.541933	6.174661
TCTE1	0	0	0.005566	0	0	0	0	0
TCTE3	0.235486	0.238576	0.166108	0.137896	0.134581	0.203224	0.136582	0.341197
TCTEX1D1	0	0.072523	0	0	0	0	0	0
TCTEX1D2	6.194633	7.577039	8.206331	8.051218	8.613164	7.682099	6.709279	6.972395
TCTEX1D4	0.320138	0.046334	0.067746	0.374934	0.06861	0.184185	0.2321	0.18554
TCTN1	5.575203	4.929299	3.423308	4.669729	5.77139	5.727213	3.445414	4.517396
TCTN2	5.839119	6.306875	5.373201	6.713181	5.333108	3.874801	6.489988	6.448392
TCTN3	7.568166	9.270392	7.191984	8.357362	6.704565	9.007538	6.920142	9.445993
TDG	21.81207	23.84681	21.62466	21.64318	20.77242	22.36538	25.55596	22.99778
TDGF1	0.545598	0.608034	0.619619	0.531157	0.682085	0.714123	0.802983	0.055336
TDO2	0	0	0	0	0.00739	0	0	0
TDP1	2.903904	3.564364	3.612225	3.004448	2.664118	2.563897	2.811426	3.687217
TDP2	5.452613	7.717186	7.752304	9.546783	9.440542	6.551971	9.010785	9.406956
TDRD1	0.007025	0	0	0	0	0	0	0
TDRD10	0.123804	0.472769	0.206903	0.117111	0.161918	0.210945	0.376982	0.164202
TDRD12	1.739508	1.168772	1.515034	1.807354	1.431655	1.605442	1.593945	1.623374
TDRD3	10.57858	10.75801	11.01066	10.75849	9.958778	10.8324	10.95079	11.59279
TDRD5	0.017499	0.070913	0.043202	0.017932	0.070004	0.008809	0.062164	0.017748
TDRD6	0.032688	0.028977	0.024211	0.066996	0.028606	0.049367	0.033178	0.024865
TDRD7	3.187867	3.399689	2.86195	3.141785	3.592534	3.086788	3.614987	3.349274
TDRD9	0.041239	0.101465	0.069814	0.175077	0.070704	0.077109	0.029898	0.143402
TDRKH	0.821044	1.269057	1.455306	1.58568	1.189614	0.975026	1.378248	0.939492
TEAD1	7.420365	6.653479	5.53942	6.015996	6.0396	5.884778	6.501063	7.248968

TEAD2	13.40353	14.1203	12.01266	13.75508	14.30149	14.92523	12.93693	12.66613
TEAD3	22.65626	22.68241	19.90001	23.02053	21.97276	21.05581	20.30707	20.93917
TEAD4	5.108289	2.975815	3.930614	4.536846	5.25796	4.221699	5.076894	4.770845
TECPR1	1.992701	1.799035	1.37018	2.673988	1.838775	1.19574	2.254407	1.627278
TECPR2	3.385389	3.637221	2.875232	3.201185	3.54507	3.084077	3.201448	3.660437
TECR	95.43126	94.17821	90.9098	123.8486	113.6817	105.9498	92.47588	79.6072
TECTA	0.007821	0	0	0	0.003911	0	0	0
TECTB	0	0	0	0	0	0	0	0
TEDC1	1.352555	1.182441	1.44342	1.654328	1.178184	1.273937	1.671769	1.106298
TEDC2	2.865891	2.635245	2.476409	3.862636	2.258739	3.05797	2.371369	2.653933
TEDDM1	0.028974	0	0	0.059384	0	0.029172	0.029409	0
TEF	21.696	23.23551	19.53827	22.18832	23.96238	20.30607	23.4253	22.31093
TEFM	4.07801	4.76855	5.144878	4.712908	4.976894	4.757021	4.595038	3.917417
TEK	6.478325	5.541331	5.994515	7.570976	9.890783	8.142954	7.415933	6.984534
TEKT1	0	0.059572	0.038712	0	0.039206	0	0	0
TEKT2	1.865944	0.166803	1.192332	0.581148	1.207532	0.976183	0.631311	1.039024
TEKT3	0.388269	0.555842	0.291741	0.60548	0.354553	0.212458	0.351257	0.350993
TEKT4	0	0	0	0	0	0	0	0
TEKT5	1.256656	1.215278	1.69227	1.502415	1.218733	1.514455	2.415731	1.969752
TELO2	2.190943	2.250597	1.86746	2.135817	2.155637	1.970477	2.399235	2.299468
TEN1	8.868146	9.432265	9.921454	8.7557	7.749581	12.57735	8.37318	7.679595
TENM1	0.748216	0.622918	0.448694	0.949751	0.82744	0.509805	0.825976	1.398517
TENM2	17.25455	13.814	11.82158	13.6913	12.26048	11.71629	12.83366	13.85374
TENM4	7.006135	5.807906	4.222813	5.554816	7.454475	5.373776	4.585231	5.966231
TENT2	4.960311	5.387899	4.727191	5.01096	4.841672	4.710374	5.194286	4.986938
TENT4A	9.001315	7.479633	7.113853	8.149098	8.733748	7.413854	7.545431	7.461886
TENT4B	4.91376	4.227851	4.424369	4.06664	5.269726	4.225843	4.058429	4.226382
TENT5A	13.58963	12.33329	12.34569	9.796745	11.56883	10.90595	12.32082	12.84069
TENT5B	36.34956	29.52267	28.02331	30.1973	29.32577	36.3783	33.88173	33.57357
TENT5C	2.418384	2.546504	2.007515	2.755364	2.619003	2.45507	2.754504	2.097331
TEP1	2.872066	3.217128	2.699792	2.570126	3.233178	2.742351	2.083715	3.470242
TEPP	0.058801	0.089359	0	0.2109	0.088213	0.059202	0.059683	0.238551
TEPSIN	2.922084	3.125963	2.525741	2.801274	3.060729	2.030961	2.41738	2.657753
TERB1	0.124095	0.068577	0.122549	0.057804	0.180526	0.283962	0.034352	0.091536
TERB2	0	0	0	0	0.042031	0	0	0
TERF1	3.712969	7.834874	6.679475	4.37447	5.369127	4.369327	10.70589	10.09817
TERF2	25.98388	24.72325	21.12323	24.32247	23.17522	21.40768	23.15366	24.40603
TERF2IP	26.53403	24.19019	25.3787	25.82978	26.01861	23.93803	26.43116	25.24341
TERT	1.029258	1.168906	0.860689	1.122801	0.946375	0.936003	1.204771	1.16177
TES	21.19036	21.62707	16.77419	19.77864	19.96289	18.79067	21.96221	22.39933
TESC	1.786542	1.942427	1.879032	2.188024	1.278342	2.632313	1.371062	2.032948
TESK1	4.258974	4.624713	4.440811	6.244908	6.094746	4.732843	4.47232	5.698201
TESK2	3.761427	4.975996	4.170733	5.612837	4.073049	4.337632	4.669449	4.264356
TESMIN	0	0.018461	0.017995	0	0	0	0	0
TESPA1	0.427487	1.002963	0.388832	0.772398	0.427542	0.600305	0.525247	1.118161
TET1	1.219225	1.559311	1.092114	1.492942	1.601468	1.326936	1.521381	1.308885
TET2	11.70316	11.35821	9.997036	9.933453	10.0889	9.365602	9.315216	11.68336
TET3	12.38517	10.69447	9.769148	10.93935	11.51944	10.11228	9.567926	11.21554
TEX10	9.402397	10.35412	8.720053	10.64072	9.439955	9.027598	9.193046	10.1075
TEX11	0.131915	0.110061	0.068967	0.389643	0.310427	0.273444	0.086637	0.196754
TEX12	0.09112	0.07693	0.089985	0.093378	0.212642	0.045871	0.061658	0.077015
TEX13B	0	0	0.023544	0.17102	0	0.024004	0.072595	0.120901
TEX15	0.572132	0.398862	0.581786	0.696599	0.441902	0.538969	0.528967	0.528569
TEX2	31.1814	23.89757	33.54921	33.96337	32.73439	28.90956	33.63391	32.99268
TEX22	0	0.090021	0.087747	0.091055	0.044433	0.089462	0	0.04506
TEX26	0	0.039687	0	0	0	0	0	0
TEX261	21.60325	22.83227	21.99969	25.09605	21.77024	20.81989	20.58534	18.18595
TEX264	27.8555	26.88044	31.08557	31.80548	26.18809	30.02408	26.48251	21.24326

TEX28	0	0	0	0.043958	0.02145	0	0	0
TEX29	0.095526	0	0.047168	0.048946	0.047769	0	0	0
TEX30	3.942136	5.717544	4.507703	4.954051	3.672885	5.097117	5.096352	5.68173
TEX35	0.212441	0.188326	0.078673	0.054426	0.106234	0.08021	0.161721	0.026933
TEX36	0	0.164385	0	0.083138	0.081139	0.081682	0.082345	0
TEX37	0	0	0.050903	0	0	0.051898	0	0
TEX38	0	0	0	0.037493	0	0	0	0
TEX45	0	0	0	0	0	0	0	0
TEX49	0.071804	0.014549	0	0.029433	0.014363	0	0	0
TEX50	0	0	0	0	0	0	0	0
TEX52	0.039985	0	0	0.010244	0	0	0	0
TEX9	3.651633	4.573628	3.150413	5.983224	4.71563	4.565423	3.095461	4.660045
TF	2.279071	1.946142	2.036324	2.068607	3.777235	2.097957	3.734258	3.225114
TFAM	12.31858	13.13292	12.54267	10.60028	15.47879	13.94254	11.84949	13.27482
TFAP2A	31.30008	31.38777	28.2932	29.71639	29.73255	27.47397	27.60858	30.59887
TFAP2B	4.098102	4.499348	2.893202	4.545113	3.709116	4.787445	4.908887	4.881607
TFAP2C	16.41946	20.14606	17.52959	22.04511	18.58788	18.5316	19.77584	17.69269
TFAP2D	0	0.019057	0	0	0	0	0	0.019078
TFAP2E	5.569983	5.309628	5.013029	5.266884	4.22867	4.728593	3.327875	4.647813
TFAP4	6.369123	6.117021	6.380655	6.159043	6.30551	5.75469	5.614538	6.534471
TFB1M	3.775606	4.207673	3.826682	4.551318	3.587289	4.48165	3.993598	3.718505
TFB2M	9.745696	11.0777	9.550908	10.77876	11.33678	9.946894	10.2387	9.100911
TFCP2	15.83475	14.80491	14.36233	13.38251	13.94999	13.30394	14.62043	17.97359
TFCP2L1	6.359997	5.171815	4.121144	4.518516	3.633367	5.061523	6.340474	9.455974
TFDP1	19.42411	19.79582	16.05148	20.95663	18.30829	18.18725	19.58677	20.25014
TFDP2	10.62479	11.09979	9.558931	10.5333	10.33213	9.749413	10.312	13.05581
TFE3	10.62693	10.01375	10.2613	10.991	11.57304	10.34288	10.33191	11.07261
TFEB	5.840999	4.520784	5.075043	4.547064	5.766538	4.883925	4.274709	4.474894
TFEC	0.26497	0.226061	0.220352	0.371572	0.251056	0.098287	0.16986	0.579916
TFF2	0	0.325003	0.118798	0.493108	0.360938	0.040373	0.122102	5.897139
TFF3	0.446933	0.905597	0.05517	0.687005	1.061603	1.349958	1.134092	1.586533
TFG	39.96077	40.42906	38.47765	40.62403	43.90155	38.83869	40.99616	39.53185
TFIP11	10.14054	10.40165	8.651928	10.43489	10.858	9.881154	10.70949	9.996539
TFPI	1.476318	0.801815	0.961929	0.889016	0.928526	0.635936	0.772404	0.424502
TFPI2	50.60788	44.23977	65.10492	57.30361	60.59368	64.31331	53.85715	55.81935
TFPT	6.376091	5.937289	6.06515	6.149674	7.642921	6.27804	6.305181	7.370317
TFR2	0.299434	0.191598	0.194541	0.1938	0.260068	0.142806	0.183955	0.23976
TFRC	19.00677	17.81078	18.79633	18.8369	17.46476	17.27514	19.46543	19.68923
TG	0.010012	0.003381	0.009887	0.00684	0.020026	0.00336	0.010162	0
TGDS	4.950198	6.095506	5.101062	5.423703	4.941054	4.314227	5.342203	5.625926
TGFA	10.31589	8.23507	5.630783	10.01406	10.11398	9.218948	9.979246	9.456474
TGFB1	13.22352	11.84405	11.35345	12.67101	13.10517	10.92443	11.49107	11.22018
TGFB1I1	6.53307	6.664694	6.529936	7.391072	7.881463	6.258505	7.29762	5.96002
TGFB2	5.229809	5.102206	5.051071	5.781154	4.927795	6.002956	5.73218	7.354748
TGFB3	3.915915	2.430789	3.565994	2.952123	3.073749	3.894192	3.502257	4.232098
TGFBI	17.57604	15.14304	19.67744	18.46752	17.85782	12.06837	17.41943	19.86293
TGFBR1	13.47297	15.21763	14.13514	13.8067	15.63066	12.97761	14.19196	15.05381
TGFBR2	14.18013	13.96796	17.47247	14.55393	17.43416	14.51448	15.70475	17.45678
TGFBR3	34.11954	34.90674	39.80426	32.06658	35.91239	29.89757	33.18726	44.25166
TGFBR3L	0.41114	0.224289	0.312321	0.388915	0.253042	0.254738	0.160503	0.737757
TGFBRAP1	7.990473	8.648331	7.57972	8.517089	8.233502	8.011067	8.464443	7.904471
TGIF1	19.76001	17.03088	16.51804	15.9557	16.82919	17.04322	18.15114	19.82033
TGIF2	4.607205	4.564121	3.967487	4.382939	3.852939	4.100403	4.365091	4.473441
TGM1	28.63819	25.04824	22.14238	23.86049	24.46326	21.46201	26.67976	24.35575
TGM2	0.734303	0.936124	0.592208	1.341943	3.188509	1.238359	0.875748	1.266085
TGM3	117.6517	94.69292	135.1448	113.7554	119.7592	136.2211	111.7914	105.6096
TGM5	24.26929	20.19635	19.46655	23.02417	23.72126	23.69883	19.38395	14.75812
TGM6	7.288172	4.829772	5.433927	4.18148	4.097096	3.758468	4.994539	4.52366

TGM7	45.66893	29.7068	23.53429	32.41807	37.63132	37.92938	44.64272	40.90034
TGOLN2	36.63548	39.06033	36.79373	40.19993	39.89014	35.62652	38.59938	37.19176
TGS1	8.449031	8.872045	8.150142	7.988612	8.235368	8.027365	8.149347	8.597715
TH	1.47542	3.285565	1.904246	3.742493	2.366822	2.750381	2.268569	3.259537
THADA	4.978392	4.738367	4.615289	4.615324	4.895878	4.548485	4.58187	4.610044
THAP1	4.889462	5.242931	4.333811	4.502331	4.875063	5.089314	4.744156	4.293454
THAP10	0.09386	0	0.018538	0.057711	0	0	0.095268	0.019039
THAP11	13.68857	16.42408	16.23045	15.78683	17.24355	16.71284	13.9848	15.33688
THAP12	18.61296	22.41426	21.40269	22.85687	23.31638	22.16768	23.63809	20.78319
THAP2	6.977875	7.353925	8.047284	7.009909	6.883177	8.240579	6.548927	7.549831
THAP3	5.791697	6.837475	6.55712	7.114742	8.2403	6.965798	6.616469	6.722087
THAP4	16.20236	15.63911	13.96792	14.42094	16.13265	15.25282	16.51827	15.48636
THAP5	4.904814	6.440163	5.638934	5.376399	5.94267	5.171725	6.068183	5.791369
THAP6	0.954441	1.391489	1.164775	0.954227	0.954564	1.187529	1.149907	1.204132
THAP7	11.85619	13.93753	14.07831	14.7795	16.04001	15.19061	13.67394	12.60333
THAP8	1.3268	1.877631	1.351864	1.748141	2.000989	1.611519	1.987989	3.33218
THAP9	1.863307	2.308599	1.761954	2.071624	2.164248	1.70078	1.762764	1.929956
THBD	16.50048	13.50589	11.87557	18.49235	15.40727	11.73513	11.63893	12.04947
THBS1	110.0658	80.90418	77.63064	82.60431	90.08798	67.07056	94.63106	85.47262
THBS2	16.37361	11.53442	13.2922	14.48198	14.81567	9.561375	13.6419	12.29831
THBS3	1.259424	1.329489	3.018006	1.615532	1.946635	1.197089	1.734219	2.751222
THBS4	12.54346	5.434615	13.08816	6.730941	9.193135	7.255428	12.19353	15.55623
THEG	6.032368	6.65536	6.361082	5.736493	7.157972	6.279462	6.771461	3.966488
THEM4	3.017645	3.754876	4.460067	5.437631	4.010543	4.03742	5.735247	3.820619
THEM5	10.03533	10.29942	16.93008	17.19341	16.25725	10.94588	13.81988	9.117962
THEM6	2.579646	2.286814	2.725829	2.656763	3.753871	2.597269	2.238682	2.106177
THEMIS2	1.619084	1.906127	1.895004	2.020217	1.986633	1.373548	1.749885	3.345085
THG1L	9.046877	10.36662	8.712338	8.158454	8.898285	8.05333	9.283905	7.580997
THNSL1	1.055829	1.339561	1.162246	1.220953	1.661451	1.077671	1.012676	1.434363
THNSL2	0.992932	0.71438	0.866872	1.179742	0.698021	1.173579	0.540428	0.6057
THOC1	15.66226	17.80793	14.55286	16.11521	16.88192	16.31831	15.38231	17.18434
THOC3	10.65642	10.79628	10.72289	8.815253	9.70392	9.602749	11.65402	9.784982
THOC5	26.32153	24.52083	22.94466	25.53017	24.91628	24.53871	25.97296	24.53631
THOC6	3.280992	2.711726	2.046385	2.315243	2.97918	2.723861	2.293169	2.218466
THOC7	35.91504	44.68296	45.69189	44.95684	43.93434	51.35483	44.14231	38.35442
THOP1	10.69783	11.27535	9.177055	10.69074	10.47793	9.790731	11.57695	10.99599
THPO	0.214242	0.077519	0.181348	0.062728	0.06122	0.369781	0.046598	0.18625
THRA	29.70917	27.60062	24.7374	29.46534	25.4817	27.03787	26.15079	26.84249
THRAP3	58.69083	64.02967	59.66057	69.92047	57.37633	63.43609	57.93322	54.30269
THRB	11.78383	15.68879	11.21051	11.02199	17.21525	11.0154	10.22808	13.41163
THRSP	5.712429	5.22449	11.98551	14.00316	13.8922	7.901693	5.128424	6.445325
THSD1	7.015051	5.085936	4.659351	5.866316	5.476591	5.197395	5.660169	6.094209
THSD4	3.741818	3.846358	2.278353	3.438906	3.471737	2.515668	3.544955	3.344978
THSD7A	0.348224	0.616033	0.423243	0.661544	0.562588	0.496238	0.573672	0.345031
THSD7B	0.014155	0.014341	0.013979	0.00967	0.028314	0.028503	0.023946	0.066997
THTPA	7.158399	7.208231	7.310004	7.755153	7.594806	7.452806	7.150866	8.028729
THUMPD1	14.14486	17.23264	17.45168	15.59527	16.74782	16.6439	16.66686	17.14579
THUMPD2	1.388906	1.098858	1.172888	1.42331	1.443079	1.116739	1.404759	1.61774
THUMPD3	19.84135	22.05152	22.32098	19.5611	21.65361	22.1673	20.66978	21.5347
THY1	32.45526	31.54968	43.22721	37.83591	48.28146	32.2604	45.20737	44.54386
THYN1	23.39593	25.71678	27.24252	23.61762	24.5004	24.27244	24.42847	21.27708
TIA1	14.52069	13.88836	10.99273	12.61786	13.46652	13.08096	12.10034	14.82851
TIAL1	15.87919	16.02832	15.45024	15.35612	16.06931	15.72682	14.98916	16.11372
TIAM1	25.64968	19.49558	17.6657	19.84587	21.30438	20.04084	22.34368	22.42051
TIAM2	1.632342	1.113111	1.054002	1.318919	1.106682	1.556577	1.250586	1.703336
TICAM1	1.336925	1.325342	1.362856	1.266919	1.667777	1.085531	1.429934	1.538209
TICRR	1.975758	1.763285	1.332913	1.688007	1.638537	1.470708	1.532211	1.747207
TIE1	3.624803	3.024309	3.474354	4.52306	5.892843	4.021679	4.039878	4.822577

TIFA	1.620921	2.730625	2.214956	2.028053	2.940656	2.277202	2.180893	1.854275
TIFAB	0.046726	0.04734	0	0	0.233663	0	0	0.047392
TIGAR	8.649243	6.433277	8.10644	7.663338	7.984122	7.43183	8.408192	7.584559
TIGD2	1.645789	2.03247	1.81118	1.664509	1.624485	1.97111	2.270973	1.740132
TIGD3	0.498416	0.757436	0.630638	0.462878	0.607523	0.595912	0.474274	0.679281
TIGD4	0.143167	0.172242	0.159056	0.055018	0.125287	0.108109	0.072657	0.127055
TIGD5	9.744801	9.555679	7.983747	10.56206	9.905728	10.04283	8.938173	8.691802
TIGD6	0.282719	0.25915	0.239311	0.096574	0.323149	0.298205	0.163977	0.163853
TIGIT	0.094879	0.064082	0.124928	0.081024	0.110706	0.015921	0.096302	0.112267
TIMD4	0.869057	1.04817	1.185173	1.243991	1.986674	0.763884	0.994105	1.594967
TIMELESS	3.597657	3.503978	3.325926	2.980399	2.957112	3.646585	3.712993	4.403176
TIMM10	27.40169	28.95168	27.01882	26.83337	28.4964	26.5749	29.68677	25.15304
TIMM10B	10.64805	10.88587	12.67308	12.3431	13.47084	12.43334	13.09565	10.63133
TIMM13	68.04811	72.28413	72.88837	68.1913	72.42807	65.24908	63.69257	63.64461
TIMM17A	18.98051	20.69335	17.56901	20.29255	20.25793	20.13699	20.32911	16.80845
TIMM17B	14.38102	17.89759	19.76262	18.72563	16.31532	22.59432	18.32297	13.52191
TIMM21	15.07562	14.65617	12.53912	13.03199	11.57848	13.95166	12.80799	13.91471
TIMM22	10.66458	10.86963	11.39876	9.78759	11.03006	11.94486	10.75934	11.01187
TIMM29	9.4565	10.92925	11.1825	12.24187	11.48067	12.56716	9.563253	10.67823
TIMM44	15.99381	16.23524	15.14922	12.94617	15.88695	16.88629	16.02843	14.58041
TIMM50	25.1539	24.83955	26.00712	27.36009	28.79258	24.92316	23.4282	24.92211
TIMM8A	14.85269	17.83819	17.40865	15.63314	17.24915	17.10875	16.53787	14.29051
TIMM8B	52.85045	62.6684	60.81956	56.09384	55.66195	79.98014	51.23473	56.82991
TIMM9	13.67783	16.68715	18.99568	17.9413	17.39469	19.62769	16.13351	17.49401
TIMMDC1	3.906709	6.217102	6.007254	7.220888	6.101677	6.483821	6.771808	7.092379
TIMP1	78.64462	65.92706	67.43008	78.85872	76.78625	72.36748	74.38579	77.29724
TIMP2	157.0433	159.3609	164.0915	143.4123	162.2568	134.5687	176.0244	197.0006
TIMP3	222.6811	169.5148	184.1391	195.0498	189.2594	161.6384	198.9524	210.2736
TIMP4	0.759981	1.371153	0.966412	1.1202	1.363978	1.708537	1.706548	1.73166
TINAG	0	0.009681	0	0	0	0	0.009699	0
TINAGL1	9.681029	7.840365	9.738823	10.15227	12.84446	11.03567	9.856808	11.45277
TINF2	4.149162	4.251384	3.259346	4.861947	4.862928	4.254649	4.408809	4.614705
TIPARP	8.349377	8.079001	6.529999	7.666188	9.396731	7.899646	9.075654	7.647437
TIPIN	6.397968	8.027506	7.425095	8.781812	7.044902	7.828752	7.181252	7.428459
TIPRL	11.50956	12.01649	12.71681	10.42372	11.53347	11.05393	11.56842	11.60519
TIRAP	2.68357	1.800602	2.034093	2.183148	2.33077	1.82497	2.867183	2.662084
TJAP1	2.462084	2.827524	2.558124	2.736751	2.221777	1.849085	2.067589	2.545937
TJP1	43.7284	38.26738	40.25048	38.57106	40.75114	41.93598	40.68668	44.02228
TJP2	40.09914	39.16347	37.67081	36.18452	40.11677	37.61141	39.86099	36.38661
TJP3	4.837438	4.50853	4.144891	4.795261	4.664146	4.297487	4.428627	5.41938
TK1	7.041747	7.71066	6.567672	7.525958	5.281993	6.373706	6.551758	7.484662
TK2	6.430988	7.444559	5.827075	6.873374	7.183304	6.997811	7.032153	6.791508
TKDP1	0	0	0	0	0	0	0	0.060701
TKDP5	0	0	0.009278	0.019256	0.009397	0.037839	0.038146	0.009529
TKFC	3.806173	4.157909	3.628009	3.708257	3.563921	3.132387	3.784881	3.79322
TKT	84.23974	95.31407	95.65965	104.5269	112.4638	91.27622	92.60887	85.70191
TKTL1	0.06464	0.043659	0.010639	0.055201	0.043099	0.032541	0.054675	0.054634
TKTL2	0	0	0	0	0	0	0	0
TLCD1	14.42339	11.90078	15.0136	13.3958	15.179	14.41726	12.55588	11.96655
TLCD2	4.124297	4.516485	6.597057	7.693794	7.061603	6.215271	4.118425	3.587371
TLDC2	0.118383	0.154204	0.100207	0.485262	0.490508	0.391631	0.30898	0.120068
TLE1	7.282293	8.522879	7.4633	9.335901	8.6986	7.183621	9.216966	9.618695
TLE2	2.821655	2.73303	1.837251	3.071608	3.462918	2.414271	2.066681	2.893272
TLE3	38.17233	31.2321	28.97487	30.91531	30.9479	31.48078	33.04212	35.11565
TLE4	12.49234	11.97393	12.863	10.77318	11.09988	12.51871	10.65858	10.50205
TLE6	0.011905	0.036184	0.011757	0	0.023814	0.059933	0.060419	0
TLE7	0.080122	0.027058	0.065936	0.054738	0.040066	0.040335	0.013554	0.094806
TLK2	7.861866	8.642424	7.667141	8.753586	8.168405	8.333405	8.038297	7.552881

TLL1	0.357407	0.587821	0.458382	0.594578	0.775256	0.59819	0.829185	0.894469
TLL2	0.443852	0.214771	0.189721	0.21724	0.669177	0.113388	0.282409	0.309072
TLN1	32.40922	30.67061	26.78206	29.62152	31.16337	24.70362	29.92041	31.89676
TLN2	5.607358	5.706684	4.283245	5.066205	5.061883	4.168713	5.09525	5.345823
TLNRD1	5.511141	5.14432	3.82196	6.758147	7.245919	5.954039	5.845215	5.840813
TLR1	0.925664	0.999511	0.841962	0.511741	0.913603	0.821621	1.508224	0.840016
TLR10	0.203393	0.160271	0.078112	0.023159	0.067806	0	0.103222	0.068763
TLR2	2.641834	4.243829	4.207172	4.756021	4.986809	2.731771	6.945228	5.455442
TLR3	1.365691	2.090626	2.060063	2.414556	2.574135	2.58383	1.888856	2.275575
TLR4	6.055426	6.831114	7.608699	7.067055	7.24481	7.057303	7.664358	7.633987
TLR5	0.652428	0.627941	0.526177	0.646303	0.739514	0.416027	0.485624	0.849202
TLR6	1.228833	1.564612	1.85308	1.412426	1.37016	1.462938	1.550655	1.296853
TLR7	0.827613	0.517357	0.469513	0.685709	0.82772	0.230478	0.991947	1.33053
TLR8	0.363234	0.620149	0.43842	0.585919	0.612195	0.277673	0.628125	0.68223
TLR9	0.096243	0.132963	0.060482	0.125525	0.043752	0.026427	0.035522	0.088739
TLX1	0	0	0	0	0	0	0	0
TLX2	0	0	0.017617	0	0	0	0	0.018093
TM2D1	13.79668	14.57925	16.24921	13.76179	13.14813	12.1261	14.9219	14.73862
TM2D2	12.82108	14.60252	14.51554	15.02505	14.75581	15.05857	13.15353	13.84374
TM2D3	12.55396	11.90556	10.85293	12.73839	13.83173	12.68117	14.14183	14.33992
TM4SF1	233.9598	201.1383	241.5055	223.3367	227.1329	260.6152	287.3281	225.5635
TM4SF18	1.700999	2.573794	2.028854	2.807124	3.535	4.337153	2.600982	1.657998
TM4SF19	0	0.02082	0	0	0.020553	0	0	0
TM4SF20	0	0	0	0	0	0	0	0.036915
TM4SF4	0	0	0.054161	0	0.018284	0	0	0.018542
TM4SF5	0.076788	0.505671	0.530817	0.590174	0.460787	0.154625	0.272789	4.594976
TM6SF1	1.097951	1.607708	1.355338	1.177889	1.509877	0.984543	1.175365	1.670371
TM6SF2	0	0	0	0	0.017506	0.017624	0	0.017753
TM7SF2	27.29488	25.92909	24.34669	32.11432	22.91433	24.39741	23.26885	19.09536
TM7SF3	26.54375	31.11813	29.53328	31.8059	24.33164	28.02219	29.34188	30.92919
TM9SF1	18.31009	19.08754	15.20832	20.45679	18.11515	17.25582	19.62349	17.49527
TM9SF2	67.85855	76.77322	75.61073	72.32305	74.29581	71.67813	75.95596	70.80251
TM9SF3	39.22028	42.5946	41.55937	40.76469	39.70266	42.18361	40.89272	40.66831
TM9SF4	19.4038	18.15021	16.79978	18.0404	18.4378	17.28076	18.96868	19.12297
TMA16	8.56267	10.57615	11.64526	11.16329	9.33422	11.37556	9.863958	9.666211
TMBIM1	56.92802	60.77711	58.60458	56.09586	56.7986	56.51374	58.73816	63.34043
TMBIM4	48.59979	59.74354	61.66384	62.41381	60.61974	61.49841	56.0798	50.63607
TMBIM6	208.7476	217.469	254.2282	258.4922	238.2705	255.7949	221.1999	203.395
TMBIM7P	0.736033	0.423231	0.333964	0.285398	0.238744	0.520746	0.242294	0.282464
TMC1	0.033878	0.045764	0.039032	0.023145	0.022588	0.017055	0.028655	0.028634
TMC2	0	0	0	0	0	0	0.033921	0
TMC3	0	0	0	0	0	0	0	0
TMC4	2.832911	3.684152	3.560598	2.934754	2.462376	3.484949	2.781301	2.716518
TMC5	0.060615	0.08444	0.157133	0.116469	0.053045	0.144945	0.084596	0.01537
TMC6	2.810214	4.120419	3.14476	3.907299	3.58737	3.419465	3.167704	4.160747
TMC7	2.075751	1.987668	1.467984	1.537051	1.660816	1.719138	1.984552	1.711406
TMC8	0.34455	0.401061	0.246143	0.345572	0.469235	0.258332	0.46133	0.91453
TMCC1	6.327321	5.154883	5.67578	5.892886	5.363503	6.219965	5.396781	5.429865
TMCC2	0.819093	1.851187	0.822714	0.781988	0.8262	1.240557	0.625311	0.795251
TMCC3	6.961276	6.505313	5.421383	6.690818	5.938175	6.651397	8.292922	6.627254
TMCO1	54.25627	74.83416	90.01841	67.88262	82.00347	83.31589	63.73964	63.27246
TMCO2	0.093453	0.165689	0.161505	0.287303	0.023366	0.070569	0.118568	0.28435
TMCO3	15.58259	15.58295	15.60636	19.08559	16.4003	15.6987	14.80346	15.51246
TMCO4	5.694825	5.86081	4.063962	6.091474	4.801732	4.71533	4.809841	4.995938
TMCO5A	0	0	0	0	0	0	0	0
TMCO6	12.69838	10.22377	8.663845	9.695904	10.44419	9.791611	9.543998	11.91359
TMED1	4.837201	4.51005	5.417314	5.064781	5.574017	4.182059	4.233777	3.910629
TMED10	54.50238	60.64589	64.11272	60.59024	61.39135	58.3082	63.04979	57.94408

TMED2	93.13022	102.5402	109.3639	104.8673	107.2639	109.8543	104.6971	102.8533
TMED3	30.4921	27.37036	27.8562	28.56033	31.92647	30.1404	32.17939	31.81266
TMED4	36.8478	35.70753	36.0653	36.98343	34.12913	34.94783	33.02721	33.01982
TMED5	4.77575	4.54138	4.777493	4.2469	6.1636	6.250321	4.996184	5.712978
TMED6	0.03069	0.062185	0.030307	0.03145	0.061388	0.0309	0.03115	0.062254
TMED7	62.98526	69.5952	75.30331	68.06321	66.04085	71.05286	64.86961	68.55014
TMED8	2.993767	2.756801	1.997156	2.887457	3.096421	2.533774	3.027141	3.531886
TMED9	65.14925	67.85447	67.08191	64.83051	66.0312	68.11256	67.79879	62.99633
TMEFF1	9.210164	9.538683	10.98027	7.810557	8.455486	9.995327	8.65922	10.84835
TMEFF2	0.305893	0.20144	0.105728	0.172408	0.198856	0.153991	0.388101	0.031025
TMEM100	5.804406	5.173144	4.439131	5.992906	9.573415	5.52183	6.496861	5.031276
TMEM101	4.890485	4.294045	3.877637	4.067405	4.281499	4.652723	3.913522	4.241248
TMEM102	4.122332	3.14922	3.425409	4.361157	3.122169	3.022204	3.425871	3.328576
TMEM104	2.455045	2.185778	2.032624	2.456561	2.736447	2.222138	2.567382	2.095955
TMEM106/	1.425374	1.717581	1.300976	1.792654	1.70635	1.630809	1.753642	1.774226
TMEM106I	9.791177	11.32221	10.94091	12.52048	9.180667	11.64086	10.82034	9.318319
TMEM106(	22.30161	18.86104	14.6192	17.40321	18.49094	19.42139	16.87953	16.96432
TMEM107	5.195205	5.723933	5.857288	7.202042	4.539175	5.223431	5.185247	5.071568
TMEM108	1.294083	1.098799	1.3023	1.288248	1.516122	1.191244	1.513646	1.362505
TMEM109	49.08888	50.01592	54.36617	64.26901	57.00072	51.42982	49.82522	49.62041
TMEM11	24.84534	21.46064	25.37975	23.36511	24.36013	20.03665	27.32469	23.74405
TMEM114	0	0.253183	0	0.042682	0	0	0	0
TMEM115	23.82364	22.56538	22.64438	24.18484	21.88168	22.87505	21.8736	23.4431
TMEM116	1.141461	1.082051	1.041542	1.169736	1.288482	0.793056	1.09083	1.245724
TMEM117	5.358765	3.920442	2.661003	4.536459	3.110309	4.212267	3.999675	3.667889
TMEM119	8.259461	7.403198	5.674595	9.371723	8.349113	7.167714	7.888892	5.72693
TMEM120/	3.742652	3.747161	3.869948	3.760155	3.214693	4.37409	3.173108	3.959677
TMEM120I	5.017496	5.322202	4.82364	5.494891	5.163248	4.783968	5.037536	5.217679
TMEM121	0.391296	0.349792	0.159114	0.306638	0.207183	0.162223	0.163539	0.350176
TMEM121I	0.132713	0.053782	0.006553	0.0612	0.073002	0.060129	0.006735	0.02019
TMEM123	43.22266	54.01697	49.9236	57.36493	51.73313	60.0955	48.84146	52.77508
TMEM125	3.222186	2.472501	2.05232	2.198077	2.107086	2.284376	2.12874	2.040118
TMEM126/	25.63916	25.24308	28.41399	23.00155	28.95701	29.24207	26.39071	25.42356
TMEM126I	10.73557	10.81172	11.80082	8.414833	10.96064	13.57548	10.70202	11.08283
TMEM127	17.16767	17.60667	15.05189	17.03844	18.98593	16.65708	17.08077	16.95629
TMEM128	15.9973	18.40945	20.98356	16.31847	15.53526	18.90987	15.91499	13.89655
TMEM129	7.963444	7.528654	6.970557	8.728023	8.347791	7.53549	8.309822	7.871649
TMEM130	0.644624	0.11587	0.800874	0.372915	0.447136	0.314046	0.306041	0.253084
TMEM131	11.39201	11.24525	11.04379	10.68957	12.02018	11.03661	11.47808	12.87611
TMEM131I	6.201568	6.30253	4.989107	6.107707	5.381184	5.242182	5.475903	6.613162
TMEM132/	10.39226	10.90761	10.18247	12.23296	10.97102	15.39195	11.31573	9.954632
TMEM132I	0.144684	0.131925	0.053581	0.177922	0.148321	0.098329	0.121155	0.135738
TMEM132(	0.768811	1.106264	0.830753	0.844945	0.863631	1.329369	0.916055	0.745853
TMEM132I	0.097614	0.059337	0.012853	0.126707	0.117152	0.131041	0.118894	0.019801
TMEM134	15.5892	17.79515	13.89072	19.78017	16.04348	21.90209	19.10844	13.39721
TMEM135	6.694889	7.005568	7.275811	7.901433	7.290933	7.222564	8.101851	7.091982
TMEM136	0.22684	0.40774	0.238466	0.704876	0.534241	0.331534	0.438204	0.497247
TMEM138	2.157838	1.804851	1.573435	1.74846	1.944815	2.37468	1.719057	1.8959
TMEM139	0.46136	0.253183	0.341708	0.37429	0.23071	0.367738	0.312187	0.077988
TMEM140	2.408764	1.993547	2.557436	2.387308	2.205492	2.744028	2.617072	2.087493
TMEM143	3.008455	3.154675	2.762885	2.627127	3.043966	2.404348	2.875357	3.751771
TMEM144	1.908714	1.928392	0.968645	1.825594	2.030922	1.606799	2.141842	2.91996
TMEM145	0.228042	0.413431	0.308169	0.565778	0.432136	0.483368	0.645659	0.340846
TMEM147	144.7534	142.7166	150.5915	144.5259	146.0565	149.0576	155.5811	135.292
TMEM14A	7.482601	6.382893	6.483124	7.4871	9.63686	5.383766	9.171128	7.535424
TMEM150/	5.943224	4.728709	5.65407	6.058553	5.975113	7.503279	4.974339	4.986373
TMEM150I	0.011905	0.060307	0.011757	0.0366	0.083347	0	0.048335	0
TMEM150(	7.400436	10.70323	8.229286	10.16526	11.46388	9.451966	12.22711	13.01357

TMEM151/	0.855904	0.585599	1.009896	0.330338	0.444683	0.481238	0.868742	0.338216
TMEM151I	0.098858	0.012519	0.018305	0.012663	0.012359	0.031104	0.006271	0.0188
TMEM154	8.386472	8.510419	8.823281	10.57425	10.92986	8.195418	8.018502	9.534345
TMEM155	0.08733	0.029492	0.043121	0.029831	0.087341	0.146544	0.04432	0.059049
TMEM156	0.617408	0.868765	0.44035	0.527251	0.463116	0.310813	0.539632	1.426338
TMEM158	6.034148	4.796621	2.536379	3.741881	2.25923	5.467834	5.041067	4.331111
TMEM159	30.04216	29.74312	36.6962	38.3777	37.69444	35.77667	33.18435	33.71462
TMEM160	11.22894	11.29204	11.78722	13.42493	13.47647	13.81801	11.98835	11.64187
TMEM161/	3.229963	2.780622	2.738935	3.45208	2.964554	2.716811	2.205158	2.660606
TMEM161I	4.68394	4.668094	4.079175	5.337637	5.495516	4.687124	4.124824	4.760294
TMEM163	0.404963	0.583857	0.476824	0.686336	0.87234	0.627276	0.600747	0.537106
TMEM164	8.460447	9.312661	10.8058	11.08392	10.28757	10.87084	7.873608	8.081961
TMEM165	18.95362	18.63467	19.93485	17.22671	17.41493	19.43571	18.99621	19.52181
TMEM167/	25.53132	30.23437	30.42339	27.07477	27.84169	30.04073	28.13181	25.91835
TMEM167I	24.76512	26.18236	33.33522	30.75652	27.51471	31.45716	26.41666	25.57154
TMEM168	6.947933	6.915699	5.840868	6.939237	7.293332	6.767012	6.728111	6.693221
TMEM169	0.29967	0.161921	0.019729	0.081891	0.159845	0.18103	0.223054	0.202624
TMEM17	3.252647	4.389164	3.617007	4.145513	2.952364	2.985909	3.037877	3.354395
TMEM170/	11.84157	12.26765	12.12081	12.42477	12.95118	12.95886	12.81675	14.02644
TMEM170I	0.755141	0.765052	0.649063	0.566054	0.748246	0.598384	0.681305	0.85099
TMEM171	0.196926	0.112767	0.033821	0.201804	0.068505	0.137928	0.139047	0.078155
TMEM173	2.341613	2.772387	2.094795	3.114795	2.498044	2.477802	3.187624	2.393575
TMEM175	4.374231	3.85273	3.414919	5.885913	4.719656	3.670094	4.159854	4.216674
TMEM176/	0.722967	0.418546	1.300425	1.508211	1.420298	1.039866	0.733812	0.36663
TMEM176I	1.710088	0.899162	1.838419	2.284837	1.796907	1.896127	1.296312	0.856239
TMEM177	2.183701	2.020905	2.052814	1.893516	1.889985	2.050635	1.832839	2.044419
TMEM178/	0.03832	0.186352	0.090823	0.054977	0.053655	0.054015	0.070011	0.085505
TMEM178I	0.369182	0.278346	0.18653	0.334335	0.223255	0.172886	0.322433	0.235112
TMEM179	0.029145	0.009843	0	0	0	0	0	0
TMEM179I	17.91744	21.2951	22.27089	23.55926	15.98545	19.3809	20.05668	15.91483
TMEM18	4.075853	4.079443	4.462839	5.060127	5.628113	3.570588	5.699298	3.496934
TMEM181	13.8068	14.44097	16.3782	14.52145	13.62971	15.4797	14.49797	13.73126
TMEM182	1.018366	0.499745	0.384986	0.619633	0.787745	0.801034	0.646026	0.653609
TMEM183/	40.06352	38.61277	37.51633	38.44269	37.01131	41.59002	33.52325	37.95401
TMEM184/	18.55214	11.56917	14.53966	19.41101	15.4367	15.57426	14.37647	13.31743
TMEM184I	13.29368	12.60815	14.33549	12.98452	16.41869	13.67462	13.38243	12.96952
TMEM184(	34.90969	35.66697	33.4484	34.93936	35.92998	34.58933	37.74681	34.54425
TMEM185/	13.71784	13.19418	11.81068	11.9223	11.65733	12.29272	12.13909	12.5042
TMEM185I	9.175352	9.999549	8.846648	11.24163	10.49367	9.077752	9.459875	9.628889
TMEM186	1.814692	1.743413	1.377165	1.442826	2.069731	1.894592	1.819229	1.690926
TMEM187	1.592304	1.541503	1.694766	2.030617	1.521732	1.656621	1.741892	1.991798
TMEM19	16.64757	17.13257	17.48419	18.42819	18.3276	18.70029	17.34063	17.03055
TMEM190	0.010495	0.074426	0	0.010755	0.010496	0.031699	0	0.010644
TMEM191(	0	0.020298	0.019786	0	0.040076	0	0.020336	0.02032
TMEM192	7.016039	8.087383	7.24954	5.31979	7.658639	7.332434	7.518786	6.85393
TMEM196	0	0	0	0	0.040277	0	0	0.081691
TMEM198	0.158683	0.024733	0.144651	0.062544	0.146495	0.036869	0.037168	0.061901
TMEM199	11.92631	14.12784	13.13937	14.04261	13.09363	14.98469	10.89324	11.59149
TMEM200/	0.149942	0.843943	0.370184	0.239021	0.266598	0.041935	0.456573	0.405537
TMEM200I	2.223923	2.194739	1.911729	1.830293	2.22421	2.877206	3.00581	3.295721
TMEM200(	0.097372	0.043845	0.053422	0.033261	0.064923	0.032679	0.142759	0.076812
TMEM201	2.538684	2.263934	1.696973	1.89139	2.425853	1.552128	2.009732	1.821741
TMEM202	0	0	0	0	0	0	0	0
TMEM203	30.78615	33.35943	33.69807	33.48594	34.62925	34.3952	32.30521	29.01366
TMEM204	5.30667	4.995691	3.988382	4.234995	6.403271	5.27988	5.481603	5.461598
TMEM205	12.05898	15.68805	15.25805	15.44711	12.98564	15.79757	15.68233	11.81369
TMEM206	3.353845	3.464924	4.129175	4.126567	4.468694	4.654149	3.762485	4.900975
TMEM208	39.51838	43.41629	43.42982	45.1786	44.56364	43.00062	40.40555	40.15449

TMEM209	9.489399	9.467571	9.746607	9.623158	8.783391	9.35518	9.431079	9.570503
TMEM210	0.235203	0.71487	0.232272	0	0.058808	0.532822	0.179048	0.059638
TMEM211	0	0	0.056873	0	0	0	0	0
TMEM213	0	0.144308	0	0	0	0.107559	0.325294	0
TMEM214	19.78217	19.83993	19.96669	20.4472	20.89495	19.5161	21.11896	19.32282
TMEM215	0	0	0.017254	0	0	0	0.008867	0.00886
TMEM216	0.767457	0.870833	0.485053	0.849385	0.698476	0.811335	0.903604	0.770599
TMEM217	0.04326	0	0	0	0	0.043555	0	0.043876
TMEM218	3.531497	3.211739	3.795691	4.123946	4.041212	3.009876	3.384406	4.081552
TMEM219	11.69289	12.88428	13.07153	12.59845	12.11791	13.09308	11.14731	11.51298
TMEM220	1.152986	0.761816	2.194731	1.113051	1.253408	2.002068	2.578011	1.135504
TMEM221	1.10183	1.973928	0.703285	1.597295	0.886953	1.109357	1.063803	0.327078
TMEM222	12.62323	11.92595	11.64161	14.59436	12.71005	14.71623	15.16415	12.56104
TMEM223	12.50775	14.28695	13.13904	13.57155	15.66737	16.26622	13.37992	11.81521
TMEM225I	5.338331	6.178335	5.381644	5.812473	8.397835	5.057537	5.907567	6.711507
TMEM229/	1.110896	2.19605	4.160779	6.358456	3.590555	4.787663	4.867757	1.85495
TMEM229I	21.29502	13.621	14.38499	18.2452	16.52812	17.11401	13.86826	13.29045
TMEM230	30.03864	31.3471	31.79403	29.82663	34.15731	30.36705	30.00798	27.45687
TMEM231	3.00823	3.175586	2.077446	2.640813	3.050698	2.689897	3.245523	2.891034
TMEM232	0.02379	0.012051	0.023494	0.036569	0.118966	0.119764	0.096588	0.132709
TMEM233	0	0	0	0	0	0	0	0
TMEM234	7.25645	7.009214	5.6082	6.651007	6.986926	7.396781	7.113687	6.674059
TMEM235	0.023984	0.048597	0	0.024578	0	0.048295	0	0.024325
TMEM236	0.011518	0.023339	0.102372	0	0.01152	0	0.011691	0.011682
TMEM237	4.619461	4.496103	4.830908	3.989491	5.437365	4.742439	4.815476	4.558594
TMEM238	1.564781	1.681814	1.693093	1.296777	2.190976	1.520672	1.450144	1.69746
TMEM240	0.398422	0.807302	0.270502	0.663473	0.796948	0.576645	0.556048	0.8587
TMEM241	4.206643	3.612626	3.654087	3.791847	3.121795	2.98662	3.283611	3.123895
TMEM242	12.21286	12.86545	11.90073	11.81824	11.98765	11.0895	12.42894	12.78099
TMEM243	3.433699	2.586185	2.431643	3.125208	3.660073	2.729334	3.347631	1.787113
TMEM244	0.969826	0.746741	1.130135	1.172741	0.814759	0.781161	1.200935	1.180358
TMEM245	6.633231	6.112177	5.782697	4.864871	6.132694	5.476751	5.493492	6.371387
TMEM246	0.417656	0.606286	0.886463	1.188182	0.723199	0.762565	0.771915	0.812429
TMEM247	0.038713	0.078441	0.229381	0.039671	0.038718	0.038977	0.039293	0.039264
TMEM248	15.13211	13.77993	13.74946	15.34578	13.77445	14.08843	17.91303	16.10959
TMEM249	4.930872	4.878148	3.530999	5.464197	4.307268	4.81194	4.280809	4.648369
TMEM25	3.573898	4.346898	3.661482	3.936617	4.128673	4.002402	4.257956	4.099678
TMEM250	6.880577	6.283925	5.334885	6.173906	6.836998	6.267279	6.487361	4.89286
TMEM251	6.186895	7.009669	7.383398	7.911789	7.285793	7.211789	6.934202	7.035036
TMEM252	0	0	0	0	0	0	0	0
TMEM253	0.249236	0.328259	0.270743	0.383114	0.423755	0.301126	0.354164	0.15167
TMEM254	23.40522	21.63055	17.61672	17.78336	19.52381	20.14345	19.91724	22.00927
TMEM255/	1.902258	1.163126	1.919933	2.258529	2.204222	2.092435	1.820218	2.056827
TMEM255I	0.173846	0.117418	0.057227	0.178152	0.173868	0.087517	0.235271	0.08816
TMEM256	66.50946	82.21671	84.64529	61.05845	65.39159	87.77307	72.88024	65.62849
TMEM258	31.5293	34.77313	31.60075	33.69906	30.64822	30.88146	35.14631	33.57704
TMEM259	48.12172	44.21242	44.48879	48.05036	48.79668	46.85158	42.27947	46.42259
TMEM26	1.138254	0.98968	0.847248	0.870485	1.155393	0.957876	0.750101	0.83569
TMEM260	10.88066	12.88508	11.59407	12.69023	10.96739	11.09375	11.55677	10.53636
TMEM262	0.355892	0.453611	0.204072	0.388237	0.160746	0.208059	0.31462	0.221232
TMEM263	5.404316	6.686649	5.772015	6.191156	7.206686	6.059018	5.820731	7.204612
TMEM265	19.25681	17.42984	19.30646	22.23809	19.68293	24.93258	17.39597	16.75497
TMEM266	0.220753	0.317819	0.126212	0.107157	0.395083	0.292449	0.117929	0.17676
TMEM267	3.133167	3.541911	3.004543	3.875816	4.487499	3.913355	3.952187	3.375938
TMEM268	2.740436	3.29999	2.780158	3.818754	3.788139	3.560203	3.243982	2.779448
TMEM269	0.023933	0.030308	0.011817	0.061314	0.017952	0.03012	0.018219	0.030342
TMEM273	0.28254	0.66195	0.209265	0.199058	0.282577	0.088897	0.322626	0.447754
TMEM30A	29.1634	30.33274	31.75289	31.31925	30.84853	30.27355	30.25214	31.36173

TMEM30B	7.623065	7.677002	7.393249	8.278273	7.874392	6.804529	7.737411	7.039204
TMEM31	0	0	0	0	0	0	0	0
TMEM33	14.17361	14.51888	14.18673	14.88266	15.49429	14.00666	13.93858	15.36297
TMEM35A	1.126001	1.249424	1.337012	1.181366	1.260211	1.079708	1.551066	1.305177
TMEM35B	3.262985	2.742597	3.986139	4.706104	4.617117	4.088344	4.980162	5.858928
TMEM37	0.413124	0.444705	0.637463	0.343978	0.490648	0.727906	0.183453	0.60232
TMEM38A	1.247925	1.011442	1.115006	1.583321	1.152994	1.088923	1.21839	1.108985
TMEM38B	2.236527	1.891899	2.605361	2.447689	2.356258	2.306462	1.796225	2.00409
TMEM39A	9.253725	9.429519	8.158359	9.084384	9.590244	8.560784	9.079446	9.263038
TMEM39B	5.062155	4.495431	4.240842	4.830731	4.053819	4.395599	4.494694	4.590915
TMEM40	6.365299	6.186155	6.887699	6.509684	7.857168	5.717	6.802897	6.889813
TMEM41A	5.550842	5.147611	6.680492	6.129761	5.042421	5.362058	5.534737	6.592995
TMEM41B	26.0297	25.85246	31.05973	28.55697	30.06385	30.66886	29.59554	22.96754
TMEM42	4.727765	4.97094	3.786089	6.31055	4.112495	4.120055	4.959982	4.210795
TMEM43	57.82316	52.50965	54.366	64.46455	56.43693	55.49182	61.58364	60.83862
TMEM44	1.44608	1.000723	1.27199	1.870596	2.39464	1.336619	1.531937	1.466666
TMEM45B	0.752124	0.522266	0.275403	0.441668	0.760673	0.77428	0.317371	0.162852
TMEM47	7.204644	10.91675	10.46754	9.925775	9.630867	11.20987	9.980963	10.54377
TMEM50A	65.34116	69.1677	81.32708	72.3297	73.91883	81.2905	83.79017	81.25858
TMEM50B	14.82416	12.52168	13.16719	16.84232	14.77798	18.36324	11.45879	17.43741
TMEM51	6.290494	3.308258	4.788213	4.606578	4.495811	4.782124	4.304394	4.430187
TMEM52	0.425199	0.580615	0.292105	0.58729	0.443743	0.577009	0.600454	0.562502
TMEM53	2.99615	3.660871	2.869607	3.718383	3.613915	3.077254	2.93412	3.664888
TMEM54	45.93577	41.14347	53.08616	48.25529	42.35612	53.19441	42.6667	38.99841
TMEM59	91.3952	84.28872	94.56358	103.0687	85.61823	90.66585	95.96152	87.77375
TMEM59L	1.051317	1.134958	0.544639	1.801485	1.51685	1.527015	1.591884	1.852886
TMEM60	14.77803	19.2986	21.05647	16.27754	16.03977	20.13768	16.18471	16.57762
TMEM61	0.339606	0.028672	0.251531	0.20301	0.679299	0.256444	0.3447	0.746287
TMEM62	18.07881	21.03445	28.50585	23.89282	22.18214	27.22178	22.51197	20.47595
TMEM63A	12.56574	11.61336	12.44892	12.81101	13.75889	13.1784	14.21676	14.56528
TMEM63B	19.24568	17.95292	18.52023	21.78945	20.26517	19.76319	17.80004	16.74683
TMEM63C	0.091289	0.062891	0.01803	0.026194	0.025564	0.051471	0.055595	0.025925
TMEM64	6.669988	7.488953	6.762458	6.520499	6.352934	7.051874	7.141897	6.896085
TMEM65	10.71721	12.10347	13.15096	11.50638	12.49638	12.78892	11.49441	13.04072
TMEM67	2.140388	2.507953	2.255533	2.24246	1.860258	1.90715	2.477892	2.080694
TMEM68	10.66148	10.23922	10.8209	11.72926	9.922898	10.22032	10.14554	10.63316
TMEM69	12.75164	10.93684	12.29726	12.97315	12.52309	13.69622	10.77019	12.04606
TMEM70	19.18577	23.46971	27.42681	26.35506	24.92093	23.9555	21.93245	18.66913
TMEM71	0.309615	0.399939	0.275181	0.356944	0.456741	0.116899	0.212125	0.753653
TMEM72	0.007266	0.007361	0	0.007446	0.007267	0.036579	0.022125	0
TMEM74	1.276076	0.796242	1.00981	0.883337	0.853645	0.918925	0.969268	1.517091
TMEM74B	0.012226	0.020644	0.008049	0.012529	0.004076	0.012309	0.020682	0.008267
TMEM79	31.80348	27.90343	28.64056	28.81725	31.75497	31.68968	27.15409	29.78831
TMEM80	3.515332	3.290976	3.032096	3.602408	3.782808	3.539347	4.064881	2.888405
TMEM81	0.681344	0.777941	1.003941	0.476563	0.757146	0.424666	0.878176	1.382086
TMEM82	0.028795	0.145867	0.056873	0.132789	0.0144	0.028992	0.058455	0.204438
TMEM86A	16.31056	15.43221	19.51176	17.00249	21.02876	19.79354	14.15055	11.51369
TMEM86B	2.248208	1.35827	1.018438	1.52184	1.44399	1.6198	0.921147	1.819987
TMEM87A	22.12301	25.30993	23.5743	25.61036	23.22707	25.77689	24.70879	22.879
TMEM87B	17.48692	16.7729	16.08341	17.44289	17.04592	17.00199	16.49647	15.68175
TMEM88	1.861737	1.854201	2.39945	1.422806	3.313689	2.382782	2.209945	1.824232
TMEM88B	0.714519	0.381408	0.394538	0.559005	0.484091	0.456394	0.483491	0.148055
TMEM8A	0.727476	0.434655	0.405258	0.630804	0.792865	0.478907	0.691055	0.699994
TMEM8B	0.623253	0.567001	0.401951	0.560484	0.604252	0.582689	0.387307	0.548272
TMEM9	11.77359	13.15829	9.47151	13.46706	12.48223	11.94687	12.26221	11.58264
TMEM91	0.099252	0.502775	0.392063	0.55941	0.19853	0.249826	0.151112	0.100665
TMEM92	1.621492	1.941458	1.219162	2.114836	2.266697	2.114919	1.066039	1.775394
TMEM94	7.133718	6.706523	5.291433	7.160486	6.798473	6.792274	6.835339	6.356931

TMEM95	0.367795	0.260835	0.544818	0.866881	0.993174	0.444369	0.597298	0.298424
TMEM97	27.43807	25.97089	30.59246	32.58438	29.52938	34.13255	23.08655	20.20669
TMEM98	6.716625	8.099054	6.824086	7.993799	5.401097	7.444608	10.78599	8.225732
TMEM9B	46.2104	50.40982	51.20496	48.45159	50.63468	55.4527	50.20837	46.40312
TMF1	10.03291	8.777882	7.976285	8.276993	8.367113	9.15089	8.198079	9.341887
TMIE	0.3354	0.118931	0.132489	0.120298	0.117405	0.151961	0.459582	0.085044
TMIGD1	0	0	0	0	0.029281	0	0	0
TMIGD2	0	0.020371	0	0.082421	0.100549	0.080978	0.061226	0.06118
TMIGD3	0.082808	0.755055	0.163553	0.424297	0.082819	0.083374	0.336201	0.223965
TMLHE	2.188383	1.751253	1.564076	1.998261	1.86505	2.349079	2.281709	2.556353
TMOD1	1.315686	1.947361	2.121501	1.927613	2.199947	1.645494	2.159627	2.56458
TMOD2	1.865526	2.039304	1.98471	1.561519	1.912804	1.638357	2.049445	2.645737
TMOD3	28.70765	21.7776	33.7972	27.68988	20.7797	22.40237	24.7996	18.69754
TMOD4	1.102603	1.718575	0.963227	1.455852	1.336019	0.939346	0.968489	1.182817
TMPO	17.94642	18.19776	17.45759	16.85171	17.50525	18.1319	16.88756	19.30725
TMPRSS11	0	0	0	0	0	0	0	0.008095
TMPRSS11	0.343798	0.379043	0.169757	0.14507	0.151695	0.39705	0.328428	0.256391
TMPRSS11	5.573859	3.886115	3.511773	4.38119	3.936173	3.741292	6.346908	4.093003
TMPRSS12	0	0	0	0	0.026137	0	0	0
TMPRSS13	6.650705	4.754553	4.88425	6.863441	6.604723	5.215444	6.617358	6.621875
TMPRSS2	0.506828	0.841045	0.250257	0.268646	0.515634	0.712648	0.620865	0.859693
TMPRSS3	0.008788	0	0	0	0.035156	0	0.00892	0.017826
TMPRSS4	8.178064	7.538134	7.194431	9.149746	10.05566	7.413145	8.773561	10.11875
TMPRSS5	0	0	0	0.021294	0	0	0.021091	0
TMPRSS6	0	0	0	0	0	0	0	0.008461
TMPRSS7	0.025318	0.012825	0	0	0	0	0	0
TMPRSS9	0.008728	0.008842	0	0	0	0.043936	0.008858	0
TMSB10	1159.481	1412.308	1321.954	1243.558	1330.91	1321.572	1295.871	1391.821
TMSB15B	1.218883	1.097671	0.847045	1.757958	1.489938	1.590828	2.107765	1.785672
TMSB4X	437.3471	621.2771	485.0224	496.8197	641.3448	483.7264	453.7906	584.9353
TMTC1	6.186356	6.337125	7.564855	5.543761	5.981093	5.106296	5.474819	8.181932
TMTC2	1.370629	1.901023	1.428471	1.637813	2.473522	1.751725	1.278251	1.774867
TMTC3	9.669378	10.10818	9.424718	9.157949	10.55096	10.22439	10.05208	10.78333
TMTC4	9.274008	9.308765	10.01497	8.378835	8.001165	8.850711	10.93536	11.98089
TMUB1	4.086392	4.644255	3.324093	4.617112	4.322704	3.718702	3.921692	4.038293
TMUB2	8.870941	6.819503	7.305649	8.815203	7.613865	7.578279	7.377828	7.241403
TMX1	22.43541	28.98168	26.3128	26.50305	27.70624	27.55907	25.75824	28.99112
TMX2	26.12664	28.09682	27.94416	30.90632	29.4454	29.84917	27.52447	24.12427
TMX3	24.05854	25.61035	26.79761	24.54978	25.14962	30.32249	25.29176	27.93653
TMX4	15.00002	16.66701	18.92429	17.06467	16.33909	15.491	16.4717	17.15934
TNC	1.463868	0.763786	1.001098	1.27511	1.669026	0.700088	1.474683	1.67772
TNF	1.068289	2.56424	0.616756	1.178962	1.643734	0.281307	1.134358	0.950143
TNFAIP1	18.87891	15.39596	16.37558	17.1577	18.55977	16.59528	18.39281	16.2825
TNFAIP3	2.81729	2.3951	1.808421	2.290832	3.050417	2.244564	3.425243	2.397728
TNFAIP6	2.808469	3.561498	6.01863	2.427731	5.063541	2.212111	4.906128	5.231846
TNFAIP8	17.97725	18.33099	18.4078	16.94494	18.80529	16.29708	18.55378	20.21452
TNFAIP8L1	6.567173	5.930631	5.573667	6.235319	6.25326	5.724799	5.398562	5.064656
TNFAIP8L2	1.061089	1.696134	1.979305	2.150581	2.641274	1.70934	1.651408	2.080642
TNFAIP8L3	31.28293	22.90028	35.87242	35.19321	32.28615	40.8639	29.32798	30.68332
TNFRSF11/	1.174165	0.981399	0.579767	0.752031	1.233033	0.738867	1.370544	1.354626
TNFRSF11I	0.739526	0.380965	0.272319	0.680775	0.376079	0.13882	0.559783	0.559362
TNFRSF13I	0.957094	1.229806	0.576322	1.794149	0.933871	1.222168	0.710817	0.947043
TNFRSF13(	0.537645	0.865113	0.312321	0.680601	0.253042	0.382107	0.256805	0.609451
TNFRSF17	0.162886	0.247535	0.026809	0.02782	0.054302	0.136665	0.027555	0.027534
TNFRSF18	1.814823	1.994018	1.539786	2.724179	3.144395	1.801485	1.842045	3.266519
TNFRSF19	13.73295	15.7451	13.22882	13.948	11.88559	14.25409	14.48186	13.32107
TNFRSF1A	57.63695	55.43169	51.63639	64.14249	65.17134	53.10435	49.17914	51.98086
TNFRSF1B	6.239623	6.104774	6.887217	6.913022	8.558303	5.463762	7.129393	8.968314

TNFRSF21	12.47662	19.05691	13.0335	14.16659	14.19855	11.8356	17.05667	10.4514
TNFRSF25	0.354144	0.322912	0.338073	0.568568	0.460446	0.368449	0.389411	0.646533
TNFRSF4	0.532224	0.31023	0.424794	0.50058	0.335418	0.168833	0.281204	0.695086
TNFRSF6B	6.213758	5.108034	4.898301	8.434376	6.48713	8.423929	5.86437	8.928138
TNFRSF8	0.842267	0.80956	0.223939	0.663946	0.853175	1.065462	1.468675	0.700929
TNFRSF9	0.185975	0.459262	0.286964	0.27396	0.244123	0.269165	0.283146	0.165044
TNFSF10	8.216854	7.897784	8.946724	7.473717	9.903642	15.48534	6.300317	7.281824
TNFSF11	0.012523	0.038062	0.012367	0.025667	0.012525	0.012609	0.063555	0.025403
TNFSF12	7.815842	6.998169	7.113495	9.741215	9.126703	8.273332	8.404776	8.591269
TNFSF13	0.528097	0.550315	0.640721	1.37614	0.769613	0.987452	1.072038	0.703951
TNFSF13B	0.434151	0.673518	0.468936	0.597841	0.52919	0.341497	0.358039	0.481612
TNFSF15	1.044106	0.949624	1.218569	0.753842	1.756224	0.836213	2.023193	1.359813
TNFSF18	0.436499	0.345153	0.262842	0.294571	0.27684	0.182224	0.410628	0.367128
TNFSF4	0.18523	0.276552	0.346588	0.149856	0.253505	0.245388	0.227589	0.197754
TNFSF8	0.679408	1.14311	0.53915	1.106519	1.189117	0.659619	0.874313	1.033619
TNFSF9	0.238318	0.214619	0.509923	0.271357	0.291316	0.199955	0.389717	0.792275
TNIK	1.618593	1.117457	1.212598	1.266484	1.477921	1.150656	1.812824	2.09989
TNIP1	24.82875	23.6962	20.9708	24.81155	25.73041	22.39392	24.3443	27.09227
TNIP2	4.432966	4.720619	4.090142	4.526193	4.821878	4.284068	4.515882	4.053028
TNIP3	0.044747	0.045334	0.088379	0.022928	0.201387	0.135158	0.090836	0.11346
TNK1	10.86819	7.921156	7.933355	10.1516	9.027289	9.293856	9.680874	9.486755
TNK2	10.28753	8.54656	8.090322	9.7977	9.289118	9.132253	8.858157	9.652086
TNKS	12.48522	11.57717	11.56556	10.30767	11.29091	10.10952	10.83112	12.7878
TNKS1BP1	29.04078	29.27728	24.9431	27.92176	30.20574	28.29536	26.60748	25.13959
TNKS2	21.08268	20.61844	18.06982	18.51282	19.08004	18.19363	20.01756	21.28948
TNMD	1.886179	2.372193	0.363971	3.799149	2.775427	5.107829	1.276315	2.968495
TNN	0.494155	0.483765	0.126112	0.091037	0.149932	0.844125	1.003135	0.309724
TNNC1	0.077425	2.431677	0	1.309158	0.46461	0.272839	0	0.078527
TNNC2	0.065243	0	0.06443	0	0	0.952483	0	0.033086
TNNI1	1.142415	1.109183	1.222195	1.365832	0.856922	1.246071	1.425282	1.062121
TNNI2	0.313524	0.664154	0.365912	1.168329	0.598624	0.774817	0.318227	1.561029
TNNI3	2.057547	4.202188	3.45102	2.744413	2.221131	8.056233	5.70169	9.208582
TNNT1	10.06725	5.57603	5.380595	5.668471	7.80036	3.814932	6.428519	5.526046
TNNT2	0.023402	0.094838	0.023111	0.071946	0.140432	0.117811	0	0.284826
TNNT3	0.40564	0.428087	0.166911	0.433008	0.388789	0.612617	0.308794	0.291419
TNP1	0	0	0	0	0	0	0	0
TNPO1	15.32825	16.57133	14.34735	15.10572	15.9441	14.29563	15.6112	16.57296
TNPO2	17.11158	16.18155	14.93305	16.33707	16.57367	15.38318	16.88916	16.68667
TNPO3	17.36819	17.62195	16.68618	16.62969	16.1661	17.3842	16.95616	16.85938
TNR	0.016635	0.008427	0.004107	0.004262	0.008319	0.004187	0.008442	0.016872
TNRC18	11.4761	9.941323	7.751684	10.34578	10.42377	9.39163	10.18081	10.41891
TNRC6A	4.803507	4.454782	3.354123	4.503556	4.747078	3.651771	3.990191	5.202145
TNRC6B	5.326844	4.518383	3.948221	4.207198	4.488025	4.444992	4.315963	4.869477
TNRC6C	7.245535	6.44946	4.714945	5.868452	7.133103	5.017047	5.784888	7.109548
TNS1	22.77292	23.31139	21.03537	23.56575	26.2733	21.71919	21.47195	24.86779
TNS2	8.632234	7.937934	6.812524	8.321897	9.075149	7.958857	8.188616	8.509208
TNS3	10.46931	9.51435	8.325426	9.887448	9.274717	9.109033	9.036567	10.07607
TNS4	34.55012	33.60315	29.11868	30.00105	28.02464	26.39104	28.88744	30.71986
TNXB	5.02617	4.405652	9.95885	5.719793	6.470101	3.313941	6.744771	8.264224
TOB1	33.9502	33.58722	36.4223	37.68694	34.43511	39.73293	34.11229	32.07957
TOB2	35.22629	31.82512	28.94901	32.19711	32.79017	34.82099	34.44622	33.56889
TOE1	14.40295	16.54895	10.85645	13.04594	11.64313	12.08663	14.04002	14.18723
TOGARAM	5.247165	4.981605	5.35959	4.968582	5.547965	4.739171	5.42595	5.269686
TOGARAM	0.007424	0.112824	0.051322	0.121729	0.059401	0.112124	0.052749	0.037649
TOLLIP	14.24692	14.00641	14.68218	14.87961	16.26775	13.90276	15.30038	14.68469
TOM1	27.16733	24.58594	23.69841	28.68247	25.91446	29.27969	27.71784	24.63673
TOM1L1	25.70689	26.55245	25.83239	36.76547	21.62167	32.3847	31.9474	30.57518
TOM1L2	12.39619	12.01132	10.4191	13.64597	11.08671	11.83252	10.86638	10.56663

TOMM20	59.11819	78.85194	67.27661	64.56399	67.27784	72.75805	77.662	67.14916
TOMM20L	0.124396	0	0	0	0	0	0	0
TOMM22	86.50274	91.28531	92.00953	86.82681	91.4583	96.84451	99.01978	90.94834
TOMM34	4.366196	2.73767	3.132019	2.958608	4.238745	3.622782	3.724351	3.76482
TOMM40	21.81677	23.05109	21.13965	24.76281	24.6435	25.18879	27.37595	22.59354
TOMM40L	9.488932	8.667172	10.05201	10.16993	9.150466	10.60104	9.555853	8.848925
TOMM5	10.09445	10.41894	12.44114	9.966288	12.40022	10.03286	9.820646	11.21442
TOMM6	0	0.019864	0.009681	0.010046	0.029414	0	0	0.009943
TOMM7	145.5559	155.8063	179.6928	154.9103	150.6975	168.3455	151.5147	155.1732
TOMM70	21.90183	25.8345	25.21654	24.33079	22.56718	23.00625	23.00889	22.99863
TOMT	0.144088	0.182474	0.142293	0.184572	0.180134	0.036268	0.036562	0.14614
TONSL	2.418817	1.466374	1.738391	1.70371	2.210471	1.516344	1.515411	1.739096
TOP1	29.47068	31.42768	31.40683	32.79331	33.72963	33.84093	30.14408	31.6548
TOP1MT	0.059977	0	0.118459	0.040975	0.079979	0	0.081169	0.040554
TOP2A	35.76255	38.23104	32.76794	31.77692	27.52433	31.04005	37.30547	37.39866
TOP2B	72.07169	72.32794	67.1031	61.5945	67.71001	68.93068	69.96149	75.36058
TOP3A	7.495295	7.39992	6.163881	7.169256	7.065933	6.546361	6.933759	8.264679
TOP3B	3.811533	3.643822	3.395751	3.860603	3.862599	3.519365	3.470928	4.481242
TOPAZ1	0	0	0	0.011061	0	0	0	0
TOPBP1	15.52563	14.60405	13.19269	13.285	13.74118	13.66703	15.68742	16.53829
TOPORS	13.98396	14.2177	11.46032	13.74262	13.47591	14.05811	13.69065	14.52773
TOR1A	11.11752	10.93956	10.25993	11.99349	11.38539	12.60607	11.55468	10.78945
TOR1AIP1	11.99006	11.0513	12.48466	12.18075	11.53952	11.90783	12.5836	11.76241
TOR1B	13.23812	11.43019	13.00914	9.867211	14.08286	12.17523	12.02172	12.11132
TOR2A	2.811781	3.965813	3.085273	3.446416	3.951706	2.923506	2.891264	2.646776
TOR3A	4.09295	3.832525	4.736024	4.310843	3.876401	3.038652	5.18244	3.952041
TOR4A	0.051325	0.173328	0.03379	0.035064	0.102663	0.03445	0.191014	0.312333
TOX	0.646657	0.853673	0.51604	0.749693	0.535685	0.460356	0.576799	1.020231
TOX2	0.494389	0.535028	0.699055	0.495121	0.449503	0.712711	0.364949	0.307694
TOX3	1.362732	2.105195	2.223832	2.159108	1.063262	1.634773	1.785372	1.313515
TOX4	34.83585	34.80008	33.89841	31.92349	33.73965	34.78223	33.18303	33.76903
TP53	65.41699	71.28195	65.81543	68.6718	65.37315	60.41472	72.71431	70.75025
TP53BP1	11.89177	10.84948	8.915108	10.02214	10.77831	8.984319	9.747195	10.78784
TP53BP2	23.13352	22.22639	21.99432	23.35848	20.7524	21.87937	23.31453	24.19007
TP53I11	9.860638	10.63064	8.386183	10.99071	9.749845	9.815183	8.521888	8.986297
TP53I13	2.282646	2.511343	1.805124	2.595037	2.13134	2.432903	2.371188	2.215662
TP53I3	9.818984	6.084938	6.064564	6.79458	6.918048	8.408249	8.527838	8.98342
TP53INP1	25.89638	25.75547	23.76007	24.72392	23.6643	22.8004	28.2067	29.19621
TP53INP2	22.73656	20.63036	21.92056	22.00072	23.31271	24.04598	25.24712	19.11428
TP53RK	3.975551	4.211789	3.936569	3.854993	4.307404	5.100228	4.599243	3.869559
TP53TG5	0.036852	0	0	0	0	0	0	0.009344
TP63	56.49865	49.16518	45.16091	44.04164	45.3548	44.56676	47.95991	52.71103
TP73	4.969199	4.973759	4.690495	5.13319	4.670454	3.847801	4.213242	4.068384
TPBG	5.217703	5.81766	4.467259	4.28831	3.821967	4.566716	4.165507	3.470688
TPBGL	0.077532	0.209466	0.204177	0.335467	0.224011	0.078062	0.314781	0.174747
TPCN1	2.840897	2.54317	2.450553	2.852335	2.809631	2.556325	2.930207	2.598455
TPCN2	3.579216	2.796468	2.396351	3.364808	3.162555	2.756195	2.809343	3.107178
TPD52	33.82444	35.99003	40.8744	32.28467	34.24789	36.83683	39.64028	38.90999
TPD52L1	5.240038	6.735631	6.640716	6.48801	4.974239	6.744894	5.357273	5.958052
TPD52L2	48.41499	46.74429	49.35788	47.51613	47.18061	50.96476	43.45697	40.63923
TPGS1	6.535251	7.470363	6.717242	7.478138	6.212149	6.215413	6.575263	6.821529
TPGS2	9.180047	8.319927	7.686105	7.750912	9.171254	7.765928	9.175956	11.8307
TPH1	0.109333	0.055384	0.017995	0.018673	0.036449	0.036693	0	0.036963
TPH2	0.094959	0.184393	0.125034	0.024328	0.023743	0.11951	0.048192	0.112363
TPI1	186.4684	182.7418	187.7971	185.0579	191.8935	179.5124	174.549	167.7043
TPK1	2.989134	3.012169	2.320469	2.833847	2.749719	3.267056	2.969073	3.096535
TPM1	249.6053	173.2492	239.9719	243.8877	235.1118	203.8707	249.5673	272.3772
TPM2	23.29393	38.47838	37.8137	36.66874	37.30942	34.77486	42.85831	30.83388

TPM3	29.36127	26.66581	25.97412	33.5848	25.69328	24.32529	27.59033	29.74164
TPM4	271.7583	246.6119	233.5039	258.7191	254.8107	223.3679	253.9055	264.5533
TPMT	11.10165	12.16325	13.23097	11.63604	13.29115	13.65328	13.61723	11.46141
TPO	0.0621	0.062915	0.008761	0.027273	0.008873	0.035728	0.045022	0.098974
TPP1	15.36922	13.25624	12.37471	14.49123	14.20007	12.01414	13.24203	12.98678
TPP2	14.42661	11.85696	10.2276	11.97494	12.01479	10.09742	11.18941	12.47435
TPPP	1.343689	1.49349	2.595919	1.664399	2.315879	1.149281	1.966319	2.910871
TPPP2	0.564855	0.537232	0.421209	0.543408	0.645633	0.313373	0.362718	0.479362
TPPP3	12.03065	13.11457	24.39578	10.27809	19.44424	10.14792	15.87193	30.49225
TPR	29.97667	31.39041	27.5274	28.96393	30.70164	29.32495	30.95679	31.31652
TPRA1	11.23325	11.11703	9.722687	12.80044	13.9241	9.417718	11.31371	12.15564
TPRG1	7.240253	4.775312	5.374525	6.075106	5.029953	5.161509	4.956779	4.016649
TPRG1L	60.66965	65.0927	58.35991	62.51269	59.88348	65.31336	63.52833	64.78348
TPRKB	7.880314	9.623858	10.08109	8.144065	9.687751	9.860437	8.215797	10.63857
TPRN	6.275305	5.873688	4.696083	6.007964	5.939508	6.187002	6.413514	6.133397
TPSB2	4.43115	14.60445	9.278095	12.50188	5.160994	5.788554	6.120176	7.879126
TPST1	11.04437	11.4262	11.95261	12.57236	13.52182	11.21676	11.1542	9.513685
TPST2	6.83833	7.547957	7.831253	8.101905	7.607126	7.70642	7.768943	7.166867
TPT1	2768.846	2254.566	2324.266	3043.385	2113.42	2265.801	2063.96	1990.975
TPX2	10.67512	11.16183	9.921981	9.65254	8.623492	9.213234	9.59128	10.85783
TRA2A	36.10939	33.72031	31.70322	30.17534	33.74648	28.89337	32.92706	35.19379
TRA2B	41.98183	45.37271	36.53414	37.12771	38.66745	38.22197	41.27243	44.15807
TRABD	31.48068	26.76122	27.58407	36.35295	33.08238	30.23675	28.83751	27.51966
TRABD2B	3.289192	3.533559	2.77017	3.128996	3.624786	3.136707	2.759012	3.19754
TRADD	3.64973	3.065351	3.120566	3.359645	4.36128	3.205388	3.696458	3.837896
TRAF1	2.918413	3.442026	2.766355	3.783065	3.75459	3.169014	3.56731	3.530298
TRAF2	10.75759	11.25952	8.439171	11.86441	11.27697	10.57036	9.571884	11.3047
TRAF3	5.095115	4.164	3.141973	4.293088	5.005182	3.659345	4.21768	4.547529
TRAF3IP1	5.214866	5.056289	4.754259	4.968287	5.419272	4.840297	4.851998	5.075611
TRAF3IP2	5.398532	5.792841	6.630642	7.366427	5.30247	5.123704	6.294563	5.936572
TRAF3IP3	1.075398	1.935829	0.777535	1.879366	1.622909	0.725052	1.520345	1.7724
TRAF4	20.0698	16.59652	15.49018	18.64146	18.36026	17.48837	18.87341	18.28043
TRAF5	3.624496	4.355476	3.519679	3.848403	2.869764	4.186509	2.830681	5.626465
TRAF6	10.09151	8.834732	9.78876	8.697501	9.949404	9.185917	9.487861	9.907939
TRAF7	27.51292	28.44696	25.97474	28.84515	28.5187	29.31918	29.50669	29.44422
TRAFD1	17.07567	17.00549	15.52084	16.75447	17.39952	15.64648	18.57431	16.5191
TRAIP	1.558785	1.97056	1.375891	1.85185	1.903896	1.27777	1.694179	2.364468
TRAK1	10.19449	9.881007	8.213366	9.941082	8.997002	9.6643	9.670432	9.992958
TRAK2	8.319796	10.97728	13.7225	6.601699	11.61931	8.80609	8.846291	14.11575
TRAM1	70.3636	74.69713	82.11004	77.00328	76.62448	82.50374	75.65905	75.44967
TRAM1L1	0.062014	0.062828	0.040828	0.042367	0.206741	0.083251	0.041963	0.083863
TRAM2	2.620561	3.01901	2.280648	3.031261	3.330909	2.373734	2.864474	3.462337
TRANK1	1.673469	1.814599	1.227583	2.142282	2.721743	1.598772	2.040456	2.014517
TRAP1	18.53175	19.11456	18.51364	18.78223	17.48052	20.3458	19.12565	18.2856
TRAPPC1	45.9108	39.53271	42.80034	45.58463	46.84506	49.85482	45.15002	41.89345
TRAPPC10	9.703018	8.437726	8.464203	8.87031	9.190755	8.149228	9.134543	8.598702
TRAPPC11	12.07692	14.48633	10.90908	12.039	10.29092	12.04071	11.66596	10.48013
TRAPPC12	6.9469	6.658148	5.925261	6.297557	7.154076	5.611533	5.723669	6.732014
TRAPPC13	10.36103	12.80234	11.24446	11.21676	10.87509	11.06569	11.4841	10.75482
TRAPPC2	21.27391	23.67577	26.49094	24.09474	21.99305	23.88931	25.42815	21.59492
TRAPPC2L	22.9679	22.94024	21.38523	20.70741	23.60707	21.2443	19.86432	20.9338
TRAPPC3	23.61518	26.85026	27.21485	24.04558	25.0405	27.33431	23.13836	22.92425
TRAPPC3L	1.032453	0.85805	0.597415	0.760457	0.838977	0.722782	0.916948	0.646288
TRAPPC4	25.91827	23.66286	24.18273	22.75344	26.41272	23.06449	24.27009	22.34631
TRAPPC5	3.200794	2.522179	3.512124	2.952071	1.920725	1.682945	2.490751	2.344593
TRAPPC6A	5.81593	5.818605	7.107535	7.449991	6.834601	7.319578	8.965438	5.714389
TRAPPC6B	9.116215	11.2279	11.44451	10.96009	10.89022	12.46295	11.0975	10.39419
TRAPPC8	17.73274	18.16113	17.94845	17.38552	19.05156	17.12212	16.72969	19.04707

TRAPPC9	5.601815	5.574468	5.427143	5.828995	5.63573	5.767053	6.177627	3.675512
TRARG1	0.456606	5.372638	0.110752	1.034342	1.72251	0.153242	0.471586	0.723096
TRDMT1	5.154671	4.147137	4.848589	4.433831	5.516911	5.319046	5.078109	4.435564
TRDN	0.183141	0.201448	0.206696	0.284197	0.507628	0.368784	0.424887	0.41926
TREH	0.501085	0.232074	0.212075	0.689553	0.658654	0.547752	0.174378	0.188767
TREM1	1.455641	0.905545	1.235748	0.863614	1.123798	1.208465	0.725777	1.424561
TREM2	0.054243	0.054954	0.026783	0.055586	0.027125	0.027307	0.027528	0.055015
TREML1	0.024487	0.124044	0.120912	0.025094	0.244906	0.024655	0.024855	0.298033
TRERF1	1.054575	1.256344	0.814151	1.415114	1.36391	0.868099	1.122693	1.393599
TREX1	1.600354	1.431623	1.630852	1.98893	1.583533	1.508438	1.313312	1.951216
TREX2	3.91441	5.043192	2.390885	3.570816	3.349177	3.758902	3.169315	2.822697
TRH	0.011677	0	0.011531	0.011966	0	0	0	0.023686
TRHDE	0.004985	0.050501	0.009845	0.035757	0.219356	0.04015	0.075892	0.060668
TRHR	0	0	0	0	0	0	0	0
TRIAP1	35.09411	38.19644	43.13795	40.52318	40.55365	42.39099	41.90935	36.68552
TRIB1	15.55206	15.74081	12.7836	14.09658	16.09983	12.56785	16.31614	13.25216
TRIB2	4.228488	3.526096	4.783075	3.30027	4.190992	3.829722	3.481148	4.153657
TRIB3	0.421318	0.311483	0.427313	0.735149	0.649141	0.573238	0.554773	0.265629
TRIL	1.317711	1.270727	1.301292	1.165303	1.181212	1.444643	1.500946	1.593864
TRIM10	4.062647	1.811768	2.517705	2.076941	4.457566	3.767232	2.345697	2.62297
TRIM11	8.99091	8.573088	7.638449	6.97796	9.372251	8.028952	7.34759	9.018325
TRIM13	11.81353	11.69314	13.87512	14.62616	11.28362	12.91445	12.35447	12.99689
TRIM14	1.068893	1.163138	1.172861	1.514586	0.82487	1.1692	1.091624	1.499016
TRIM15	0.014065	0	0	0.028827	0.014067	0.014161	0	0.014265
TRIM16	5.231353	8.24339	6.056889	6.645225	7.434989	6.624491	6.562595	4.69917
TRIM17	0	0	0.009668	0	0	0	0.009937	0
TRIM2	23.12345	27.5856	26.36502	24.594	21.6291	24.88404	24.81874	24.54141
TRIM21	6.830781	6.245647	4.757149	6.619031	6.568312	6.612329	6.225769	7.320767
TRIM23	3.497284	4.315838	3.399911	3.90211	2.865746	3.921558	4.329359	3.718346
TRIM24	6.902333	7.506009	7.255962	7.625777	8.255277	7.336026	7.192415	6.901175
TRIM25	10.73539	12.19356	10.43899	10.23739	12.08484	9.88364	9.959427	11.05086
TRIM26	8.268134	8.146176	6.437046	7.710822	7.114139	7.540606	7.717231	7.684798
TRIM27	14.75361	14.90879	13.73588	15.12879	15.30588	14.73977	14.96528	16.26273
TRIM28	49.88468	48.90968	45.95523	46.58234	45.69883	44.26167	48.94262	49.70231
TRIM29	356.4182	271.2736	295.3516	302.8674	282.8712	316.3879	285.3651	260.1217
TRIM3	3.206032	2.983876	2.85827	3.357152	3.670872	3.247148	4.384026	2.825857
TRIM31	0.364942	0.250665	0.207685	0.221853	0.179401	0.211742	0.150678	0.144291
TRIM32	2.918302	2.125057	2.267889	2.837661	3.101096	2.020039	2.532912	2.161023
TRIM33	8.410469	8.058665	7.465622	7.725988	8.342947	7.020919	7.446919	8.286677
TRIM35	5.185377	5.185789	4.153754	5.040147	5.334432	4.548595	4.803855	4.867952
TRIM36	3.478964	2.863755	3.095016	2.97351	3.314441	3.555574	3.135416	2.319373
TRIM37	9.360951	9.206962	7.540398	8.416724	8.67503	8.961123	9.113069	8.836979
TRIM38	7.340237	7.838876	6.797213	7.411076	7.822575	7.947689	8.28087	7.896295
TRIM4	14.84952	11.46633	10.55361	13.66328	11.33962	10.22691	12.38632	13.44024
TRIM40	0.091001	0.052683	0.025676	0.013322	0	0.091622	0.05278	0
TRIM41	10.8546	10.24289	9.596624	10.21501	9.827253	9.899923	9.252158	9.657003
TRIM44	3.331939	3.805379	3.294741	3.562343	3.677852	2.936464	3.368604	3.689813
TRIM45	2.323806	2.258988	2.434215	3.220149	2.738118	3.144835	2.711985	2.519102
TRIM46	0	0	0	0	0.012463	0.006273	0.012649	0.012639
TRIM47	6.444158	5.965275	7.020546	7.111765	9.34705	6.31776	6.577635	6.830193
TRIM50	0.015786	0	0	0	0	0	0	0
TRIM52	3.232406	3.232051	2.756629	3.519203	3.561268	3.523728	2.866614	3.616256
TRIM54	0	0	0	0	0	0	0	0
TRIM55	0.1676	0.526378	0.157236	1.004745	0.662105	0.750914	0.170114	0.212482
TRIM56	3.879861	3.698154	2.982653	3.535533	3.72138	2.999426	3.902204	3.755954
TRIM58	0	0.016158	0	0	0	0	0	0.016175
TRIM59	10.60287	11.13264	7.186746	12.17431	13.183	15.69755	11.15325	9.922832
TRIM6	0.256657	0.547423	0.346839	0.276858	0.18914	0.353615	0.15082	0.082203

TRIM62	3.635694	3.16543	2.608645	3.213461	3.596394	2.539494	3.061737	3.687451
TRIM63	0.009079	0.055186	0.008965	0.018607	0.036319	0.054843	0.018429	0.009208
TRIM65	7.906652	6.381181	5.633956	7.464896	9.027768	6.861979	6.509587	7.329906
TRIM66	0.462508	0.42701	0.486212	0.443386	0.399151	0.627149	0.620879	0.692288
TRIM67	0	0	0	0	0.083505	0	0	0
TRIM68	5.66308	5.500109	4.634272	5.529047	5.496491	5.482792	5.331994	4.937713
TRIM69	0.016963	0.085929	0.016752	0.017383	0.067861	0	0	0.034409
TRIM7	3.24287	3.07257	4.212818	4.577587	4.540605	3.31101	3.254426	3.400213
TRIM71	0.010093	0.040904	0.019935	0.010343	0.030284	0.010162	0.010245	0.020474
TRIM72	0	0.005306	0	0.005367	0.010476	0.010547	0	0.005312
TRIM8	7.68199	7.579779	8.081028	8.171765	7.774845	7.019872	8.763397	8.782205
TRIM9	0.251588	0.223029	0.134579	0.193365	0.204442	0.406346	0.313882	0.260486
TRIML2	0	0	0	0	0	0	0	0
TRIO	7.353046	6.209127	5.819635	5.852018	6.979712	5.826332	6.438686	8.282453
TRIP10	25.5373	27.00047	25.09043	28.82812	27.53078	25.35399	30.51293	34.44235
TRIP11	10.61699	10.47601	8.833655	9.296389	10.73611	9.87454	10.57906	10.09642
TRIP12	30.42381	28.43467	27.16744	26.26931	29.32336	26.95875	28.19835	29.12165
TRIP13	13.41807	12.13078	10.61072	11.24446	9.237998	11.71099	12.48761	10.79474
TRIP4	13.63054	16.17953	14.48617	14.77545	14.69472	14.85328	15.14339	15.24094
TRIP6	10.90012	11.01353	12.07914	12.5945	10.68203	12.37403	13.15754	10.65463
TRIQK	0.63308	0.819552	0.646031	0.951517	0.833663	0.885284	0.410535	0.806182
TRIR	102.868	102.8799	108.543	101.3555	110.8388	110.4101	108.3373	103.3755
TRIT1	9.864376	9.13619	12.20543	13.60614	11.7461	11.13398	10.98011	11.24298
TRMO	4.94343	4.391238	3.622909	4.615903	4.504911	4.221352	4.773172	4.30986
TRMT1	7.642992	7.890154	7.235438	8.764087	7.591263	7.655403	7.035374	9.155137
TRMT10A	1.484232	1.295331	1.421821	1.5153	1.801323	1.365643	1.032542	1.398238
TRMT10B	9.214064	9.745104	7.842873	9.165747	6.978917	12.49873	10.68272	9.403884
TRMT10C	14.992	18.06483	18.83609	15.92946	17.81777	17.75178	17.17935	14.31831
TRMT11	1.674403	1.940553	1.7287	1.917364	1.433576	1.596441	1.635143	1.801163
TRMT112	60.04948	67.30566	65.74307	61.86879	67.92298	66.74789	59.21281	57.24443
TRMT12	4.748041	5.309156	4.768804	5.176667	5.052191	5.56817	4.723424	5.150812
TRMT13	8.139543	9.225572	6.941657	8.963271	8.540036	9.079806	8.18056	9.527348
TRMT1L	9.506786	9.156702	8.802041	7.668937	9.734582	8.997585	9.340163	10.09372
TRMT2A	8.140209	8.821835	7.551582	9.588909	8.379743	8.626307	9.21373	9.031663
TRMT2B	0.560133	0.266008	0.259291	0.215253	0.227584	0.475841	0.088834	0.355067
TRMT44	3.51467	2.954703	2.257652	3.459412	3.472387	2.947108	2.949332	4.171462
TRMT5	2.225629	2.444853	2.302853	2.396078	2.388484	2.574441	2.227286	2.111475
TRMT6	7.424917	8.180689	7.889173	8.333485	8.018417	9.678886	7.885462	8.761488
TRMT61A	8.259351	7.882886	7.028258	9.23914	8.723619	8.020448	8.398911	8.517848
TRMT61B	1.406847	1.587005	1.465205	1.902068	1.507531	1.231961	1.355952	1.354931
TRMT9B	0.573235	0.480094	0.460422	0.493445	0.810277	0.892661	0.729232	1.35659
TRMU	7.398305	7.329573	6.28779	8.336366	7.104601	6.476542	7.592347	8.682292
TRNAU1AI	18.80363	20.70024	18.01652	22.94597	23.43759	23.54342	23.47617	22.52944
TRNP1	2.674914	3.481747	2.291705	3.739613	2.196902	2.942887	2.121685	1.976352
TRNT1	9.672546	10.61818	11.54707	9.786668	11.29018	11.60006	10.77454	11.42458
TRO	0	0.029334	0.057187	0.059343	0	0	0	0.029366
TROAP	2.570873	2.234082	1.497809	2.105438	1.968747	2.209376	2.107201	2.531101
TRPA1	0.041931	0.134525	0.04831	0.057293	0.020968	0	0.007093	0
TRPC1	2.027711	1.965302	1.768826	2.174908	2.433567	2.000732	1.769989	2.070287
TRPC3	0.0451	0.036554	0.080169	0.02773	0.099233	0.009082	0.064087	0.11893
TRPC4	0.02197	0.066774	0.043392	0.007505	0.029296	0.029493	0.022299	0.037137
TRPC4AP	34.30277	30.76185	34.30954	32.54825	33.07598	34.20069	30.93484	32.03528
TRPC5	0	0	0.00945	0	0.066991	0.009634	0.009712	0.009705
TRPC6	1.327481	1.502099	1.021512	1.254361	2.025964	1.622953	1.408639	1.538719
TRPC7	0	0	0.020892	0.01084	0.010579	0.0213	0	0.021457
TRPM1	0.021068	0.026681	0	0.070168	0	0.005303	0	0
TRPM2	1.045708	1.980469	0.632632	1.226076	1.479255	0.701901	1.056612	1.294689
TRPM3	0.033014	0.069124	0.089114	0.054131	0.052829	0.033239	0.055848	0.042413

TRPM4	1.771035	2.153134	1.520256	2.136003	1.86664	1.954589	1.825256	2.321304
TRPM5	0.006704	0	0	0	0	0.0135	0	0.006799
TRPM6	0.508008	0.215036	0.281765	0.452844	0.400195	0.343321	0.236624	0.395253
TRPM7	8.175113	9.910253	8.591532	9.759402	9.51262	9.088607	9.985971	10.68272
TRPM8	0.021263	0.014361	0.006999	0	0.028354	0.007136	0.028776	0.043131
TRPS1	9.147312	9.267361	7.908626	8.852964	9.615226	9.855859	8.001662	9.362051
TRPT1	1.894405	1.567852	0.922225	1.175735	0.933982	1.343202	0.974951	1.948435
TRPV1	0.008601	0	0.016989	0.008815	0	0.01732	0.034922	0.008724
TRPV2	1.721052	2.112486	2.364198	2.509857	2.306067	2.532568	2.329157	3.905114
TRPV3	14.51011	9.736822	10.197	12.25503	13.45069	12.05565	11.79443	12.6252
TRPV4	9.95131	6.786935	6.545725	5.84155	6.832466	7.367651	8.216813	10.7025
TRPV6	13.50743	30.07269	46.85094	67.57625	47.10494	49.63893	55.73918	23.34566
TRRAP	12.05653	9.573423	8.272303	10.27084	11.62314	8.755002	9.883164	11.54505
TRUB1	3.887837	4.277421	3.235715	4.009197	3.244359	3.90619	3.482874	3.85225
TRUB2	12.99246	12.50393	11.02491	11.69279	11.93913	12.16075	10.42139	12.96344
TSACC	9.2034	10.31145	10.15799	8.710149	9.746036	11.33756	8.956803	7.357717
TSBP1	0	0	0	0	0	0	0	0
TSC1	7.815261	7.716141	6.497099	6.610425	7.321733	6.229618	6.808123	8.180529
TSC2	14.57505	12.414	11.31532	13.10656	12.77677	12.35163	12.79346	14.30264
TSC22D1	24.37079	28.9495	26.85594	23.87219	24.85671	27.52982	28.24697	24.45559
TSC22D2	9.057982	8.987586	8.536997	8.829837	8.928928	7.689184	9.446683	9.88169
TSC22D3	20.03884	22.39384	27.58262	20.73225	19.73207	25.01555	35.49428	48.66084
TSC22D4	25.1819	21.94932	19.86977	25.30303	22.99333	21.44524	22.23359	23.56108
TSEN2	1.869293	1.762966	1.360584	1.441293	1.85877	1.780916	1.799006	1.757623
TSEN34	18.14741	18.02366	16.08683	19.85991	18.9179	16.77874	18.60092	19.47459
TSEN54	13.44393	13.02167	14.54968	17.9059	17.48877	13.64395	12.35055	13.60807
TSFM	13.69588	16.45349	14.66144	14.39792	16.6629	16.3513	13.67674	13.35227
TSG101	50.05824	50.80691	50.22117	47.51345	49.91986	53.42605	50.07408	48.7694
TSGA10	1.025533	0.743279	0.755671	0.808412	0.702186	0.722777	0.424373	0.592075
TSGA10IP	0.015849	0	0.015651	0	0	0.031914	0.016087	0.016075
TSHR	0.102294	0.127552	0.054395	0.112891	0.062958	0.102993	0.047921	0.151635
TSHZ1	6.077029	6.521811	5.746312	5.679879	6.336062	5.894843	6.216945	5.828565
TSHZ2	1.701746	1.826059	1.42582	2.259655	1.89387	1.764045	1.807091	1.544123
TSHZ3	1.865565	1.884286	1.701899	1.760233	1.678088	2.136005	1.691493	1.753675
TSKS	2.074942	1.987925	3.574768	4.668701	5.62788	4.518851	2.483785	2.012981
TSKU	11.26477	11.01252	7.263531	10.00598	10.61455	9.224475	9.209126	13.16744
TSN	31.02339	37.61	31.3095	34.15033	31.25448	33.98894	31.50969	30.44969
TSNARE1	1.473114	1.414354	1.353264	1.667585	1.670316	1.491801	1.364814	1.563577
TSNAX	13.05037	14.36046	12.51034	15.34339	12.17517	15.17666	15.79039	12.58631
TSNAXIP1	0.119306	0.096697	0.223857	0.207844	0.202846	0.108109	0.169534	0.181506
TSPAN1	0.145129	0.504115	0.184269	0.297448	0.165883	0.14612	0.315656	0.252334
TSPAN11	5.203433	5.976857	4.254889	4.703992	5.867049	5.422511	6.374785	6.067452
TSPAN12	3.26962	6.122541	4.75835	5.165956	5.254341	5.585116	6.00031	4.466043
TSPAN13	10.84618	11.84319	16.52772	15.88496	12.20353	16.48658	12.87426	10.67973
TSPAN14	15.98772	14.39421	14.04125	15.57547	15.45679	16.91252	13.64195	14.55054
TSPAN15	9.831258	7.60446	8.037164	11.16111	10.37973	9.123762	9.978727	10.33538
TSPAN16	0	0	0	0	0	0	0	0
TSPAN17	22.44498	18.25506	19.53815	21.50039	20.18968	21.10152	20.54661	19.74889
TSPAN18	18.09447	18.23912	15.42616	15.85516	20.08975	17.29565	17.94742	15.50632
TSPAN19	0.01664	0.13487	0.032866	0.102316	0.149783	0	0.05067	0.067509
TSPAN2	11.46124	14.14008	15.47566	13.35684	11.72645	18.09157	11.9964	13.05715
TSPAN3	60.16581	65.18208	62.95189	64.049	62.69574	66.04677	68.57345	69.2165
TSPAN31	11.30695	12.96507	14.89763	14.04623	15.27627	17.57559	10.58278	11.25199
TSPAN32	0.207987	0.105359	0.029342	0.304485	0.148582	0.074789	0.16587	0.316422
TSPAN33	7.766052	4.657204	4.19874	5.514622	4.926981	6.981909	6.481202	5.85574
TSPAN4	10.4097	8.998694	9.112091	12.27084	10.81763	10.4312	10.64087	12.28214
TSPAN5	3.115597	2.669645	2.629457	3.604481	3.442963	3.382755	3.222299	3.279795
TSPAN6	34.85071	44.23513	43.82493	49.92874	43.74181	51.46521	42.26277	39.37717

TSPAN7	43.34113	38.71474	43.09898	43.53618	42.06062	42.19863	40.04332	37.93602
TSPAN8	0.926794	0.970255	0.274574	0.949751	0.834222	0.777604	1.128835	0.595325
TSPAN9	8.739988	8.230365	8.225379	8.739189	8.833897	8.959811	8.353215	7.80256
TSPEAR	0.401239	0.395519	0.107092	0.588985	0.216914	0.174694	0.319202	0.285966
TSPO	75.08356	84.26298	74.02223	77.4655	77.83204	72.80731	81.9304	75.99095
TSPOAP1	0.14706	0.234659	0.192426	0.350383	0.20591	0.222097	0.253752	0.152882
TSPYL1	17.74904	19.84955	17.7285	20.45553	18.72958	18.68844	18.0611	18.03224
TSPYL2	10.90747	13.22019	10.42568	11.41351	11.39928	11.23386	11.325	10.89021
TSPYL4	26.76769	26.10777	27.06141	25.34929	23.03645	24.65958	25.9365	23.4534
TSPYL5	2.897937	4.482185	2.718288	4.328902	2.871055	3.905556	4.711782	4.781944
TSR1	14.09182	14.67079	13.86136	14.14907	14.7813	13.46085	13.50661	12.91178
TSR2	24.66258	24.73446	23.1092	22.62858	22.33304	26.35173	25.16836	24.27683
TSR3	19.26707	20.64203	18.82191	22.43995	15.80796	20.62998	21.26577	17.31808
TSSC4	4.180271	4.201785	5.103346	5.509372	5.080619	5.20304	5.267526	4.97423
TSSK1B	0.061313	0.077646	0.015137	0.078539	0	0.061731	0.031116	0.031093
TSSK2	0	0	0	0	0	0	0	0
TSSK3	0.070104	0.071024	0.069231	0.107761	0.10517	0.158812	0.035578	0.15998
TSSK4	0.178315	0.206464	0.125781	0.293677	0.203815	0.173122	0.142207	0.245445
TSSK6	0.14262	0.270922	0.176054	0.036538	0.160468	0.143594	0.108569	0.108488
TST	3.409076	5.125728	4.867629	3.916303	4.538783	5.552998	6.61187	4.382571
TSTA3	20.16446	19.10441	18.7632	22.21321	21.08653	21.94778	18.93242	18.19293
TSTD2	11.53956	11.12799	9.258667	11.10181	11.46913	11.17736	10.80352	12.93059
TSTD3	4.80411	6.524815	6.738211	8.811653	7.520449	7.010043	7.314258	8.226757
TTBK1	0	0	0	0	0	0	0	0
TTBK2	2.43061	2.408743	1.758316	2.033986	2.311501	1.824725	1.979581	2.467903
TTC1	28.839	30.35923	29.75678	34.77867	30.06222	31.37973	29.15907	31.49807
TTC12	0.441056	0.688382	0.423788	0.916178	1.490246	0.300047	0.677557	0.640777
TTC13	5.486556	5.652509	5.380037	6.089679	6.383776	5.920835	6.180644	5.164946
TTC14	12.50675	13.86245	12.63018	16.86042	15.1116	13.82812	13.61414	13.19323
TTC16	0.356903	0.498187	0.46211	0.536425	0.515594	0.678757	0.474959	0.17697
TTC17	8.294661	8.803688	7.459191	9.122842	8.490212	8.700063	9.5848	9.306402
TTC19	7.91753	9.808673	7.407357	8.554919	7.932446	7.747855	8.149083	8.086594
TTC21A	0.080275	0.169434	0.105699	0.041132	0.046833	0.080823	0.074689	0.094987
TTC21B	4.804679	4.153029	5.083728	5.817578	4.28568	4.847101	4.862236	3.050508
TTC22	1.092136	0.866305	1.35443	1.926046	1.007605	0.877973	0.953841	0.798562
TTC23	2.445065	3.692652	3.044495	4.217552	3.675667	3.883785	3.499101	3.296228
TTC23L	0.129918	0.164529	0.128299	0.066568	0.560343	0.040877	0.057692	0.057648
TTC24	0.007967	0.008072	0	0	0.007968	0	0	0.008081
TTC25	0.03766	0.038155	0.044629	0.030875	0.030132	0.053085	0.01529	0.030557
TTC26	1.972157	1.755141	1.397678	1.82287	1.307206	1.245886	1.515042	1.482525
TTC27	10.78094	11.68291	12.00871	10.05761	11.6269	12.04491	11.25694	13.71322
TTC28	1.876241	1.88541	1.655148	1.981335	2.242626	1.695161	2.095984	2.036389
TTC29	0.292715	0.3248	0.302833	0.371387	0.236991	0.126306	0.183923	0.325157
TTC3	13.27178	11.0138	8.789205	11.84542	11.13963	10.5976	9.899839	11.25259
TTC31	3.22872	2.640015	2.276028	3.117197	3.40565	2.759502	2.918877	3.411567
TTC32	2.184057	3.848209	2.977381	4.476314	3.893822	3.011643	2.337297	2.793013
TTC33	4.658364	5.385957	5.206638	4.618956	4.20574	5.313256	4.911236	4.744447
TTC34	0.064003	0.084795	0.029172	0.09586	0.044316	0.069398	0.069961	0.029961
TTC36	0	0.034372	0.033504	0	0.101792	0.034158	0	0.034409
TTC37	12.88281	14.77975	13.38783	12.68982	13.77859	13.82477	13.22496	13.72184
TTC38	10.16486	12.46187	12.86587	16.9454	12.49294	14.60671	13.55059	11.3381
TTC39A	3.131655	4.544971	5.033262	5.062555	3.852433	5.98291	6.913519	3.63679
TTC39C	3.719358	4.085551	3.301775	4.155864	4.152775	4.088849	3.665305	3.523899
TTC4	13.32403	14.20341	13.857	16.23728	14.10816	13.42756	17.36806	12.79583
TTC5	8.493158	7.922454	6.981887	8.750583	7.989192	6.995019	8.387568	7.015418
TTC6	0.085652	0.115701	0.070487	0.112155	0.118976	0.028746	0.135235	0.077219
TTC7A	9.910196	8.717049	10.2675	8.663745	9.251336	9.798795	8.205778	7.710566
TTC7B	4.186034	4.220967	4.026628	5.169926	4.413677	3.667922	4.609574	4.596089

TTC8	7.715567	7.584508	6.407248	6.565866	6.340534	6.76329	6.43481	5.759606
TTC9	11.2644	11.54118	10.6464	10.12821	9.757375	10.50196	9.799103	8.888071
TTC9B	0.033681	0.068247	0.074839	0.07766	0.17685	0.076301	0.042733	0.111023
TTC9C	4.951152	5.067316	4.465374	4.607833	5.137063	4.238925	4.461341	5.183898
TTF1	7.612243	7.230137	6.377788	7.396238	7.886574	8.255372	8.178505	7.761675
TTF2	6.272552	6.828344	5.30021	5.529961	4.637469	5.117021	5.217829	6.347034
TTI1	3.178359	3.078219	2.433573	2.38901	3.122757	2.791253	2.25254	2.953788
TTI2	2.589047	2.921096	2.275928	2.532579	2.814971	2.221648	2.667699	3.282379
TTK	2.688153	2.529209	2.557895	2.868247	2.279473	2.432005	2.953325	3.046159
TTL	11.75841	12.02675	12.60637	14.7874	14.19486	14.66572	13.64835	11.87173
TTLL1	5.157132	4.97149	4.537288	4.884507	4.485722	6.844479	5.853111	5.278098
TTLL10	0.257462	0.216631	0.219779	0.514263	0.401518	0.202104	0.217032	0.314238
TTLL11	0.183912	0.110631	0.306485	0.088344	0.31614	0.098371	0.215838	0.361403
TTLL12	33.37038	29.75617	28.17941	31.93349	30.14214	30.06937	28.66003	35.35044
TTLL2	0	0.016353	0.01594	0	0.016143	0.016252	0	0
TTLL3	3.32351	2.092901	1.52521	2.030144	1.79232	1.909311	1.673452	2.974255
TTLL4	8.628037	7.403252	6.805832	7.302865	7.891618	6.830788	7.763993	8.009725
TTLL5	5.09598	5.942257	6.419347	5.195754	4.378875	5.920945	5.596904	6.069219
TTLL6	0	0	0	0.028668	0.013989	0	0.009465	0.009458
TTLL7	2.027907	2.413464	2.665742	2.136922	2.011296	3.193436	2.339162	1.540015
TTLL8	0.010283	0.010418	0	0	0	0.010353	0	0
TTLL9	0.018017	0.018253	0	0.027695	0.027029	0.01814	0.009144	0
TTN	0.00026	0.003957	0.001029	0.002402	0.004688	0.004457	0.003965	0.000792
TTPA	0.463482	0.469565	0.635704	0.369415	0.721065	0.337024	0.156811	0.208924
TTPAL	7.712945	5.061716	5.646315	6.504085	6.117425	6.498408	4.845188	7.488039
TTR	0	0.044586	0	0.045098	0	0.044309	0.044668	0.089269
TTYH1	2.003843	1.195164	1.164983	1.374506	2.133399	1.366716	1.427011	1.147305
TTYH2	1.339638	1.869378	1.464393	1.631844	1.70215	1.459068	1.197249	1.538161
TTYH3	16.98321	14.77889	11.18107	13.35981	12.53381	12.02395	13.80025	13.9687
TUB	4.673107	3.854989	3.353449	2.972873	5.40693	2.98423	4.857332	4.288021
TUBA1A	139.1957	180.1971	174.4847	134.2032	169.8543	191.0431	157.3318	148.5613
TUBA1C	47.65589	49.48585	54.38802	54.34439	40.14363	49.56504	45.3287	39.74265
TUBA4A	329.234	295.8168	301.6786	316.0917	309.3088	315.8614	280.7705	262.5661
TUBA8	3.809176	3.411728	2.689534	3.356659	3.404776	3.279373	2.85771	2.799567
TUBAL3	0.034555	0	0.017062	0	0.017279	0	0	0
TUBB	98.43111	108.7073	76.65206	96.49872	86.41108	81.00485	106.2625	114.3248
TUBB1	0	0.038299	0.022399	0.147209	0.068054	0.038061	0.023022	0.161032
TUBB2A	128.1252	93.89647	127.6021	104.7571	106.3148	135.2419	104.5686	105.799
TUBB4A	0.223714	0.07555	0.09819	0.17831	0.174023	0.012513	0.02523	0.163872
TUBB4B	204.711	175.1974	168.0693	192.1028	192.9426	176.1093	186.7292	176.3335
TUBB6	38.51443	34.62209	33.86365	43.55613	38.45236	33.54646	37.05078	34.67708
TUBD1	3.861005	4.253474	4.010328	3.802988	4.086446	4.3277	3.995016	4.397545
TUBE1	7.063932	7.062117	5.830811	5.955026	6.851567	5.810525	5.96589	6.042505
TUBG2	11.04287	11.71552	10.18512	9.465925	8.630529	11.20572	8.371139	8.699428
TUBGCP2	26.01391	24.70386	22.69752	28.55473	25.05829	21.89554	25.34328	24.99354
TUBGCP3	20.46798	19.10763	17.2254	19.47565	17.96324	19.6411	20.40374	19.2368
TUBGCP4	9.480622	9.587405	7.737597	9.135566	8.219343	8.765279	8.995448	8.573676
TUBGCP5	8.492045	9.895965	7.961229	8.544886	8.393223	8.410781	9.867487	9.540481
TUBGCP6	5.896638	4.562374	5.080228	5.081732	5.007227	4.240658	4.04921	4.502899
TUFM	34.69344	34.45211	33.89076	33.96734	35.32311	35.1822	35.53108	33.77666
TUFT1	54.02248	50.247	58.53384	49.04185	45.0726	47.9968	50.6117	45.38468
TULP1	0.20316	0.237492	0.092598	0.144133	0.140667	0.346158	0.222069	0.364553
TULP2	2.844278	1.788583	1.416526	2.211176	1.863733	1.456541	1.866557	2.076536
TULP3	5.824754	5.814318	5.237543	5.2998	5.931066	5.01442	5.83178	5.874221
TULP4	3.415811	2.912516	2.974909	3.303585	2.993624	2.655911	3.35982	3.172217
TUSC1	3.09724	4.658704	3.602824	3.329736	3.515726	3.960175	4.39732	3.546042
TUSC2	14.34595	15.17671	14.42109	15.77272	16.07914	14.3059	14.57853	13.22901
TUSC3	14.89822	14.39319	17.14916	16.07249	8.628881	9.778479	17.3708	15.07843

TUT1	3.42934	3.984056	3.315633	4.040377	4.271826	3.65952	3.40783	4.030082
TUT4	4.633682	5.054114	3.884343	4.620654	4.591104	4.138917	4.313689	4.908843
TUT7	13.97105	13.49112	13.60681	12.19476	13.45502	13.42887	12.80381	13.97464
TVP23A	0	0.039808	0.012934	0.026844	0	0.065934	0	0
TVP23B	1.532138	1.296205	1.825016	2.185177	4.597009	1.749346	3.687399	8.795032
TWF1	8.526013	11.01961	10.52968	11.1806	10.67731	11.55132	10.12228	10.72602
TWF2	10.87041	11.37728	10.71806	11.33264	11.83059	10.23801	11.83274	12.72668
TWIST1	8.65824	10.08667	10.50144	11.37374	10.7903	10.96012	9.181318	9.056531
TWIST2	9.044794	10.55313	10.34552	8.148428	10.41777	8.326019	9.805948	9.375169
TWISTNB	8.910338	9.617046	8.576781	9.208024	9.324667	9.339889	9.692035	7.942055
TWNK	8.052659	7.149645	7.39385	7.860334	7.456245	7.802932	8.343224	7.416
TWSG1	27.04076	27.94141	26.71133	29.3278	29.55595	28.86789	28.64003	30.75773
TXK	0.110629	0.199255	0.157806	0.327512	0.122937	0.247522	0.187148	0.47375
TXLNA	24.10224	23.39761	21.93189	25.2383	23.98353	21.73038	24.2446	23.22112
TXLNB	0.051503	0.081168	0.339077	0.111422	0.211763	0.155565	0.261379	0.104473
TXLNG	4.966423	5.208594	5.286553	5.102231	5.316507	4.786769	4.559626	4.992827
TXN	238.175	262.4058	325.8826	274.8371	287.4068	292.6672	260.5604	251.9671
TXN2	48.96027	52.88444	51.50795	51.74759	51.87387	54.31203	53.65677	47.25652
TXNDC11	11.61181	11.12669	11.87312	13.14613	11.73079	10.85485	12.31397	12.28768
TXNDC12	21.61682	23.43766	24.88636	22.55209	22.68904	24.17955	19.57977	22.20301
TXNDC15	20.504	26.10856	23.28448	23.55962	22.48513	21.82835	24.61028	23.53433
TXNDC16	2.205513	2.042385	1.878475	1.839201	2.085712	1.966073	2.161624	2.063854
TXNDC17	48.59005	55.07388	53.31032	47.0304	46.00741	58.64124	48.05993	40.3115
TXNDC2	0.029493	0	0	0	0	0	0.074839	0
TXNDC5	62.49432	63.21448	48.26104	62.49102	64.29618	62.37455	67.69059	52.76991
TXNDC8	0	0	0	0	0	0	0	0.073926
TXNDC9	24.25728	25.14913	27.68182	20.79747	28.60087	24.71851	24.79138	22.3486
TXNIP	470.9817	529.6053	428.6794	406.3102	423.7885	453.2416	491.4806	652.6208
TXNL4A	42.31756	45.34437	52.41536	47.18134	47.40796	54.2149	44.20336	44.79512
TXNL4B	5.80445	5.017416	5.321937	4.704012	4.558946	5.393732	4.756454	3.769892
TXNRD1	56.82577	54.00215	63.84339	50.31292	54.17274	56.63726	60.37809	56.01121
TXNRD2	4.857729	5.391728	3.660691	4.847741	5.576936	4.173922	4.091619	4.5593
TXNRD3	6.974096	7.183123	6.138921	7.79656	7.152852	6.827124	6.717709	6.893013
TYK2	12.9067	12.92633	11.71034	13.86075	13.01001	10.52055	12.33135	14.41635
TYMS	14.428	17.03732	12.66932	14.60773	15.35461	13.88654	15.75888	18.81436
TYR	0.153599	0.017291	0.219101	0.069957	0.460857	0.051549	0.13858	0.311571
TYRO3	18.62538	17.08784	18.37889	19.31859	17.70808	17.06988	14.78602	16.86972
TYROBP	8.698022	14.12902	13.00443	15.38695	17.1067	8.463897	16.67056	23.36064
TYRP1	0.016861	0.034164	0	0	0.033726	0	0.034228	0.017101
TYSND1	3.220975	3.138877	2.262958	2.56992	3.27401	2.677959	2.604752	2.887378
TYW3	5.526197	6.946563	6.442693	6.423404	6.448063	6.748865	5.790866	6.772546
TYW5	5.258116	4.529953	4.791745	4.447638	4.827849	4.514393	4.715818	4.433585
U2AF1	82.8204	85.30191	90.96415	79.02372	79.44673	81.59604	85.9838	84.8138
U2AF1L4	17.32256	19.16369	19.26964	18.50952	18.46272	18.64372	16.71627	17.59801
U2AF2	63.13985	62.71238	58.14081	64.54266	62.84264	61.36335	61.65458	61.79583
U2SURP	15.1299	15.88011	14.81554	16.82641	15.96608	16.57852	14.424	16.68085
UACA	34.35102	34.04593	33.91809	30.92018	28.18182	37.72073	34.13422	30.78898
UAP1	5.031079	7.3079	8.784474	5.124642	7.168699	7.231996	4.152913	5.625265
UAP1L1	4.75905	5.333793	4.190612	7.020752	5.860338	6.239013	5.494621	5.440204
UBA1	82.55736	73.99562	73.7153	72.87783	70.98061	70.18233	73.74581	72.39922
UBA2	30.34924	31.37107	28.8401	29.00055	29.20241	28.51983	31.3857	29.53288
UBA3	28.5816	31.13079	30.53967	33.01317	28.04545	35.19233	30.77419	27.37281
UBA5	14.23702	15.49105	15.92544	13.96428	14.09672	14.08177	16.0628	13.96487
UBA52	1474.775	1620.304	1588.616	1426.396	1500.644	1442.695	1425.897	1448.19
UBA6	12.3035	14.53363	13.6134	13.10723	14.94415	14.2167	13.92286	14.69826
UBA7	0.986438	1.462513	0.958307	1.454669	1.836776	1.267713	1.269858	1.691869
UBAC1	46.28117	42.80989	44.94723	47.0674	48.01273	49.29957	43.95936	45.44934
UBAC2	14.92838	16.7282	16.11034	19.04982	14.69528	15.34168	15.67956	14.38824

UBALD1	15.49111	15.91979	14.61906	17.49133	14.32001	16.43176	17.13982	13.96818
UBALD2	21.67624	19.24393	19.51196	18.60639	20.46844	20.54936	19.7899	19.94499
UBAP1	18.75687	16.92158	17.02059	16.38495	18.80228	16.45298	16.25491	16.72218
UBAP1L	0	0.014662	0.028584	0	0.014474	0	0.029378	0
UBAP2	22.46556	20.60467	18.31292	19.45337	19.01653	20.78809	18.19432	22.76655
UBAP2L	27.26181	25.17937	22.61239	24.98006	25.55604	22.84203	24.7661	25.50531
UBASH3A	0.471449	0.972031	0.383895	0.737404	0.769305	0.283136	0.470125	1.258316
UBASH3B	1.097441	1.282207	1.166797	1.274272	1.190523	0.966821	1.068987	1.656131
UBB	411.5755	442.5261	474.7574	505.1865	464.6904	463.8116	417.0266	460.8797
UBC	67.52457	71.87087	68.50834	63.42261	68.83385	64.64796	62.22326	62.56772
UBE2B	47.08545	49.205	51.41364	49.29099	48.09016	52.77929	48.40932	45.60373
UBE2C	29.15229	29.88589	28.39802	28.80927	23.14169	26.85981	27.83128	30.06928
UBE2D1	8.197192	7.983364	8.18601	7.131289	7.553446	8.541693	8.06047	9.216891
UBE2D2	55.35244	62.38651	62.70872	60.43685	58.67612	64.7629	60.33987	58.36777
UBE2D3	103.6526	102.8148	101.7865	109.2117	114.9795	113.8634	115.7482	111.2098
UBE2D4	7.412084	7.213716	7.229158	5.852693	6.687582	6.497352	8.047948	7.898131
UBE2E1	63.42754	67.48	64.54007	61.02912	62.70065	64.42085	64.74188	59.51526
UBE2E2	9.511543	11.01793	11.2952	11.70356	10.43336	8.838546	10.72691	9.646946
UBE2E3	38.01074	43.29583	42.46253	42.73005	35.8004	43.24526	43.20306	36.46245
UBE2F	25.21745	22.94705	25.86041	20.98037	24.38739	28.21461	25.25053	25.47296
UBE2G1	25.28297	28.24516	28.42723	27.02838	28.61103	26.65206	26.29663	26.0748
UBE2G2	21.90405	20.96334	21.41985	19.34087	22.69139	21.13229	19.96471	20.38363
UBE2H	40.07183	41.11109	39.8769	43.31277	39.07914	40.45603	41.26145	38.93627
UBE2I	37.30737	35.88905	34.15241	36.22489	34.36892	35.01672	36.44653	36.52422
UBE2J1	9.985567	10.95695	12.36929	12.65112	11.40161	10.85653	11.40145	10.96246
UBE2J2	9.313226	8.658357	8.903967	8.879596	9.848946	9.735582	8.634207	8.627705
UBE2K	17.78568	19.10552	18.31284	15.95629	16.98092	17.61819	16.99703	16.13831
UBE2L3	41.69641	40.78807	44.70811	44.15791	44.23498	46.3101	41.01482	42.87284
UBE2L6	13.78044	17.1262	15.84783	17.03909	16.22624	18.8461	17.56704	14.94757
UBE2M	50.36231	49.38775	50.1165	49.83899	48.48141	53.68235	50.65618	46.74385
UBE2N	152.0196	157.1036	154.3407	158.2847	162.8948	162.8199	155.2283	156.9668
UBE2O	8.071526	7.266294	6.208104	6.870386	8.054398	7.147992	6.583586	7.324943
UBE2Q1	57.1191	56.64787	51.50447	55.24091	55.56776	55.27689	54.48592	53.36936
UBE2Q2	20.13926	22.68718	20.59482	19.45306	20.68094	18.11595	19.36706	18.51755
UBE2QL1	1.47049	0.479763	1.02144	0.5619	0.648096	0.602252	1.619035	0.480289
UBE2R2	39.10971	35.81766	40.62273	35.88573	37.96768	38.41121	37.34766	37.61885
UBE2S	30.2493	31.54833	30.21057	32.14495	31.509	34.11066	31.30552	32.81015
UBE2T	8.004631	11.86315	8.278772	12.27665	9.249789	9.447913	10.26567	8.612279
UBE2U	0.176774	0.131335	0.558627	0.362305	0.023573	0.261039	0.119617	0.011953
UBE2V1	31.29802	34.94437	37.25717	35.8504	35.45962	36.35089	35.21101	34.20744
UBE2V2	14.47866	15.65509	14.70338	16.27787	15.88646	16.30684	14.5737	14.00241
UBE2W	9.096707	9.808588	9.745886	8.132785	9.95695	9.669465	9.363006	10.26421
UBE2Z	10.58218	9.767027	8.323429	10.1946	10.08001	8.523577	9.737796	9.522426
UBE3A	20.38356	20.29891	19.86183	18.79393	22.26351	21.69875	19.24982	19.82761
UBE3B	9.82388	10.50852	8.910911	9.150638	10.30455	9.721831	10.07155	10.34965
UBE3C	16.62438	15.71744	15.53208	15.54572	17.10576	15.26224	15.55772	17.08974
UBE3D	2.282312	2.843461	2.756427	3.208011	2.822413	2.344483	2.582637	3.800655
UBE4A	24.97901	23.01513	22.48609	22.58068	22.74194	21.62711	20.63226	22.74579
UBE4B	21.2651	19.32971	20.22726	18.46517	19.58661	18.4077	19.23389	19.83939
UBFD1	18.66542	18.42022	16.92872	18.00152	17.41932	17.44585	17.12058	17.70136
UBIAD1	3.793761	4.239453	3.392744	3.904419	4.022232	3.524596	3.949826	3.905567
UBL3	23.21635	25.65555	28.19255	28.13746	24.07175	23.38182	27.84151	23.93411
UBL4A	17.72432	18.1534	18.75463	19.06423	18.03692	18.49622	18.5413	17.54394
UBL5	246.3356	272.1806	284.0905	256.9273	302.2499	247.6032	256.3612	235.9361
UBLCP1	25.58599	28.39619	29.05919	27.43773	25.91586	30.62627	29.09835	24.91698
UBN1	7.60359	7.69331	5.904016	6.676618	7.137364	6.679829	7.667202	8.261238
UBN2	5.743436	4.931395	4.556467	4.285343	5.000701	4.299291	4.40631	5.462827
UBOX5	3.266669	3.551434	2.550763	3.269734	3.039154	3.365473	3.712227	3.026984

UBP1	50.98241	50.26417	49.84934	50.24058	49.04004	51.58826	47.29488	52.24236
UBQLN1	65.2379	67.55872	67.07251	68.38222	77.45371	65.44406	65.26919	64.9602
UBQLN2	16.7675	18.29564	17.1576	18.4438	18.13898	15.95725	18.48784	17.77961
UBQLN4	16.6235	14.02964	14.95301	14.93092	17.0018	14.5408	15.02524	17.33315
UBR1	8.581158	8.477886	8.478282	8.172193	8.528987	7.835217	7.578507	8.66591
UBR2	10.96376	10.14255	8.663834	9.71438	10.02316	8.782793	9.361029	10.61686
UBR3	12.537	12.60957	11.40508	12.11053	12.49672	10.71747	12.22895	12.5278
UBR4	13.01258	10.86519	10.27072	10.89902	12.85217	9.762505	11.25146	12.25929
UBR5	16.3791	16.59675	14.92472	15.80629	16.40516	15.78133	16.06055	17.93952
UBR7	13.93848	13.75599	12.58541	13.31451	13.54748	14.30808	14.28586	12.86872
UBTD1	10.62433	9.349188	8.875363	10.41055	9.132966	10.01825	8.405416	8.464195
UBTD2	19.70904	20.80461	21.03743	20.2072	20.61552	18.58143	19.52132	21.57655
UBTF	34.47967	35.86626	32.63909	33.89462	35.74209	31.86276	33.61484	33.09411
UBXN1	85.70563	94.1332	90.11533	83.8209	75.46547	84.747	95.55284	72.92634
UBXN10	0.399286	0.745597	0.208753	0.224646	0.117452	0.275892	0.222504	0.254099
UBXN11	1.014308	1.260289	1.152865	1.46109	1.148422	1.40661	1.476297	0.980222
UBXN2A	25.02672	22.99106	36.00086	32.80114	30.81093	33.49348	23.7295	23.77542
UBXN2B	12.00418	11.63839	11.22805	10.86309	11.69127	11.32303	11.53457	11.16712
UBXN4	28.99853	34.08144	35.02644	34.74453	31.64186	34.16131	31.77643	32.49932
UBXN6	16.44092	17.18199	16.58983	18.00745	16.33934	17.95799	18.04627	16.12997
UBXN7	10.97494	11.23993	10.19981	10.14271	10.16966	9.56057	9.839114	12.14123
UBXN8	6.720009	8.130183	11.70476	10.94257	10.76645	9.196392	9.160634	8.139104
UCHL1	2.496803	1.315377	1.183532	2.277198	0.923937	1.885395	2.002062	1.418114
UCHL3	54.23972	45.17688	53.32206	61.65601	49.618	63.58459	65.34319	52.50342
UCHL5	30.35523	32.86872	33.16575	33.40345	33.03176	36.33601	35.2605	33.13064
UCK1	11.98248	9.798144	9.008428	12.70763	11.85287	11.2639	12.91091	9.892022
UCK2	8.599422	9.205536	12.62939	9.502807	10.80304	11.72472	9.567352	9.674984
UCKL1	4.208897	4.627375	3.394437	5.000062	4.794086	3.98653	3.670782	3.794501
UCN	0	0.051651	0	0.10449	0	0	0	0
UCN2	0.090113	0.310406	0.195779	0.295505	0.18025	0.072583	0.274395	0.201071
UCN3	0	0	0	0	0	0	0	0
UCP1	0.017603	0	0	0	0	0.070894	0.017867	0
UCP2	5.532542	6.997735	5.395735	6.954926	4.742792	3.425239	5.720167	11.60598
UCP3	0	0	0	0	0	0.020767	0	0
UEVLD	14.2426	13.54833	11.72319	13.31412	13.1299	14.20991	10.38008	12.86023
UFC1	63.89834	68.21658	81.63035	69.47355	71.00733	71.25093	65.97548	60.15469
UFD1	37.04561	35.70193	38.79778	38.10764	46.55642	40.54878	37.19824	35.4163
UFL1	8.024175	9.383252	8.756265	9.835145	10.35575	9.914826	9.033154	8.03826
UFM1	29.75739	34.72079	33.94739	31.60317	31.06431	32.54915	33.67518	28.87135
UFSP1	6.024695	4.926804	3.841911	5.454098	6.782035	5.385824	6.992563	5.480233
UFSP2	20.74197	18.10258	18.73837	18.69482	20.20908	20.45234	23.19102	18.44832
UGCG	12.52753	12.37289	13.50921	14.57737	13.3972	14.77072	14.09536	12.52669
UGDH	29.3389	25.1358	25.95673	28.04548	31.89152	31.79232	28.03046	28.27953
UGGT1	12.92291	14.86048	11.69064	12.77044	13.35435	11.89791	14.11674	15.44018
UGGT2	0.942534	1.109112	0.687975	1.091865	0.895816	0.719098	1.206239	1.615024
UGP2	35.29031	32.4698	42.60557	30.94385	36.74322	32.95377	39.41563	43.92342
UGT1A1	0	0	0	0	0	0	0	0.024793
UGT1A3	0.036909	0	0	0	0	0	0.012487	0
UGT1A4	0	0	0	0.012647	0.012342	0	0	0
UGT1A6	0.024487	0	0	0.012547	0	0	0.012427	0
UGT1A9	0	0	0	0	0	0	0	0
UGT3A2	0.120643	0.033334	0.010831	0.011239	0.054845	0	0.05566	0.033371
UGT8	0.560017	0.567366	0.414779	0.454328	0.560089	0.704803	0.17763	0.414159
UHMK1	8.969147	9.389181	8.412009	9.761212	9.506151	9.35135	9.203498	9.949764
UHRF1	16.87242	14.72966	15.00269	14.75258	14.52032	13.186	16.44877	15.58765
UHRF1BP1	4.433712	4.208995	4.237523	4.375022	3.860217	3.942316	3.766452	4.223054
UHRF1BP1	11.01468	12.31894	12.98823	9.688853	11.3707	11.78687	11.15933	11.52765
UHRF2	3.875269	3.808074	3.25162	2.824918	3.629372	3.117372	3.554927	4.315376

UIMC1	10.14768	8.153035	8.370432	8.762867	8.284235	8.242651	8.385659	8.455421
ULK1	16.15039	13.69384	14.10462	15.1636	15.1793	15.32044	13.94056	13.39691
ULK2	12.36511	11.59315	9.613607	11.94655	11.0986	10.36586	11.31148	12.70587
ULK3	7.743055	6.548111	5.828127	6.698865	6.565412	7.165602	6.849934	6.545958
UMAD1	2.25214	3.306631	2.842538	2.900335	2.565604	2.170519	2.298146	2.540715
UMODL1	0.177536	0.193701	0.195553	0.307887	0.177559	0.158124	0.318813	0.145435
UMPS	16.87428	16.98965	16.30947	16.40312	16.89142	16.1461	17.2185	15.30898
UNC119	14.09703	15.90123	15.07475	16.29399	17.13175	15.69931	17.40727	16.93697
UNC119B	18.85665	17.54713	17.05029	17.05199	18.17899	17.67093	17.47609	18.2009
UNC13A	0.016747	0.008483	0.005513	0.011441	0.005583	0.00281	0.002833	0.005662
UNC13B	3.173984	2.698282	2.93011	2.539828	3.535421	2.787898	2.993714	3.589751
UNC13C	0.143237	0.10711	0.01684	0.080382	0.08186	0.054939	0.006923	0.038048
UNC13D	2.362068	1.964763	1.948281	2.454961	2.442909	1.743116	1.586981	2.041784
UNC45A	23.15036	23.21824	22.36787	22.38912	22.18719	22.71798	22.78848	21.40662
UNC45B	0.00805	0.008155	0.015899	0.008249	0.024153	0	0.008171	0
UNC50	26.12702	27.49021	33.73894	29.01349	27.19462	28.10385	30.35694	26.17133
UNC5A	0.477734	0.253526	0.673974	1.220034	0.386787	0.587886	0.908226	0.423007
UNC5B	37.29604	33.17313	33.06018	34.10821	31.21109	33.7588	33.26731	29.47142
UNC5C	0.105091	0.14703	0.079071	0.128207	0.085084	0.095732	0.157461	0.111662
UNC5CL	1.098621	1.123441	1.034234	1.262618	0.790699	0.651271	0.802455	0.728955
UNC5D	0.292357	0.579368	0.123735	0.539936	0.118886	0.362282	0.570658	0.606072
UNC79	0.003116	0	0.003078	0.003194	0.018701	0.009413	0	0.003161
UNC80	0.008218	0.012488	0.004058	0.008421	0.004109	0.008274	0.00417	0.006251
UNC93A	3.942434	4.176704	2.323428	4.76774	4.907481	4.033389	3.528267	4.804718
UNC93B1	12.71903	13.63672	11.75677	16.95552	14.99266	12.72031	15.90608	15.09324
UNG	14.40012	22.40264	19.701	19.72922	16.67897	20.67325	20.39324	20.65209
UNK	5.193099	4.635631	3.526688	4.910023	4.524086	4.2323	4.62156	4.761452
UNKL	5.21123	4.260346	3.538777	4.963827	4.296146	4.040401	4.543605	4.373939
UPB1	0	0.036684	0.017879	0	0.018107	0.018228	0.018376	0
UPF1	30.51596	30.45525	26.30689	29.65742	30.73988	28.03618	29.5356	29.56524
UPF2	18.60948	18.2408	16.8708	16.65646	18.67078	19.20556	17.98657	18.57575
UPF3A	5.30123	6.02113	4.43733	4.706692	4.449624	4.334585	4.77415	4.770555
UPF3B	0.107789	0.200206	0.017741	0.202508	0.107803	0.2894	0.164108	0.163985
UPK1A	0	0.019624	0	0	0	0	0	0.019645
UPK1B	0.079696	0.064593	0.078703	0.147006	0.079706	0.144432	0.080891	0.113162
UPK2	0.086475	0.204424	0.085398	0.265852	0.230631	0.116088	0.029257	0.029235
UPK3A	0	0.041548	0	0	0	0	0	0
UPK3B	9.321055	10.52338	11.72118	8.939124	8.555156	11.12555	10.95191	7.92427
UPP1	0.851552	0.885431	0.442601	0.872645	0.582716	0.631746	0.318436	0.886402
UPP2	0	0	0	0	0	0	0	0
UPRT	5.53087	5.760015	5.468313	6.017577	5.177409	5.601068	5.914458	5.85123
UQCRH	273.6541	340.656	332.7379	293.326	287.3392	316.8548	289.9342	304.5314
URAD	0	0	0	0	0	0	0	0
URB1	4.02086	3.453477	2.965791	2.779643	3.86833	3.262545	2.982246	3.814245
URI1	9.393987	9.23405	8.315448	8.470885	8.455199	9.705656	9.251149	9.297956
URM1	38.76592	32.75457	34.42958	34.32549	33.93935	35.76543	33.89535	29.7239
UROC1	1.132614	1.193378	0.680049	1.234952	1.051202	0.821053	1.085226	0.891421
UROD	15.96697	18.48406	12.73036	16.24217	14.44258	14.70486	15.15782	14.9797
UROS	4.082214	2.970147	4.096899	4.330712	4.237643	2.840314	3.548318	3.411001
USB1	1.8595	1.67074	1.516236	2.19693	1.598125	1.442798	1.789271	1.851366
USE1	11.31155	13.70606	11.79258	11.46266	12.44684	12.96145	10.4584	11.03821
USF1	13.93234	12.84345	11.45366	11.87484	13.29087	11.92809	13.29895	14.16224
USF2	44.69287	46.04793	47.57576	48.30648	45.34893	49.44035	47.8302	45.14069
USF3	5.413086	4.31992	3.594166	4.48696	4.844691	3.556493	4.582944	5.651289
USH1C	0.528507	0.579452	0.264536	0.734497	0.839928	0.568564	0.529086	0.734289
USH1G	0	0	0.0199	0	0.040306	0	0.020453	0.020437
USH2A	0.003878	0.007857	0.010211	0.013245	0.003878	0.001301	0.001312	0.013109
USHBP1	3.20576	2.675955	2.755627	2.990485	3.802673	3.46357	3.080885	2.862525

USO1	28.90684	31.88521	28.64964	28.2835	28.08317	28.81734	29.57331	29.28309
USP1	22.80398	27.9807	24.51923	24.20034	22.84557	24.22796	25.16181	22.58782
USP10	31.86485	30.22587	28.87184	29.10554	31.20327	30.12297	29.77729	33.06191
USP11	19.94514	20.11824	17.64918	19.91493	18.7763	18.14984	19.60891	19.47808
USP12	22.66975	20.81935	19.95937	21.86359	20.64172	22.24597	20.02825	20.99116
USP13	0.569558	0.889747	0.457226	0.677805	0.602707	0.525353	0.533345	0.648476
USP14	23.34765	25.72616	26.19245	23.51445	25.85386	26.10234	24.66474	23.74632
USP15	11.53311	11.97011	10.93538	12.04992	11.72548	11.93943	11.66209	11.93925
USP16	30.84364	35.45047	38.14983	37.69359	34.94787	38.85651	37.05226	32.97315
USP19	13.5217	11.02803	9.674586	12.0097	11.19444	9.769256	12.48837	12.23011
USP2	13.90647	11.27894	14.15692	13.1831	11.25688	13.44604	12.64523	11.45451
USP20	7.047471	5.767327	5.924519	5.999321	7.377381	6.438822	5.319614	6.175153
USP21	4.275025	4.555292	3.589664	3.846463	4.939438	4.216713	4.010305	4.424043
USP22	63.22534	56.2384	54.13113	57.15315	54.88322	50.86755	59.91552	57.70447
USP24	9.392867	9.721389	8.192351	8.719599	9.575647	8.490139	9.89161	10.77811
USP25	6.305859	6.180467	5.993179	6.348688	6.061678	5.937875	4.967431	5.671644
USP26	0	0	0	0	0	0	0	0
USP27X	2.100233	1.585251	1.280797	1.517727	1.66534	1.684925	1.859962	1.66337
USP28	15.09303	12.49973	10.96747	12.13345	13.88107	12.56874	12.65839	14.98782
USP3	10.05275	11.42505	10.7277	12.08942	10.11421	11.14746	11.43902	11.42682
USP30	10.7741	11.93488	9.755014	11.4775	11.64958	11.41708	10.71208	12.25338
USP31	5.664411	4.137424	3.748204	4.21722	4.904898	4.325495	4.147746	5.021932
USP32	12.44359	12.37841	11.3117	11.96522	11.83579	13.81531	11.54994	11.42086
USP33	5.484227	6.910877	5.43575	6.155809	6.601028	5.698833	5.872622	5.557201
USP34	19.49293	19.3055	17.0028	18.84612	19.19233	17.15038	19.15517	19.92909
USP35	2.164145	1.466141	2.059227	2.366051	2.434155	1.914014	1.889482	1.741285
USP36	7.552249	7.309131	6.476435	7.54151	6.896842	6.515497	6.632033	7.11648
USP37	3.069876	3.272152	3.067414	3.113148	3.232315	2.968502	2.968783	3.574127
USP38	36.57361	29.92952	45.48129	41.01536	41.99608	38.40062	31.9111	33.56681
USP39	28.77379	23.4306	25.31588	24.54354	26.60343	28.10267	28.44817	26.07851
USP4	30.51997	28.68652	26.32892	28.02339	28.00949	27.44874	30.19781	30.63253
USP40	13.72742	16.50865	11.70739	11.43203	13.73354	13.20887	14.74023	14.62339
USP42	3.95964	4.087739	3.282371	3.607522	3.601715	4.36499	3.661135	3.775634
USP43	2.249255	2.884073	1.86722	2.060067	1.490324	2.002774	2.090366	1.910567
USP44	0.019756	0.025019	0.043897	0.025307	0.029638	0.019891	0.015039	0.010019
USP45	2.483511	2.440621	1.945702	2.460194	3.071672	2.017051	1.966195	2.275375
USP46	9.913744	9.737807	10.8967	12.1968	11.83761	10.28562	10.6701	11.70933
USP47	18.15837	19.00742	18.41677	19.1982	20.03901	19.59359	17.88163	19.14194
USP48	18.41604	20.21871	18.92211	19.86745	21.49302	20.77687	19.12957	20.87041
USP49	3.336343	3.062153	2.670633	3.238343	3.143276	2.467406	2.8409	3.030628
USP5	30.2752	29.92046	27.95697	28.61331	28.5496	29.82805	29.82175	28.06249
USP50	0.211683	0.142974	0.162591	0.265132	0.35285	0.094724	0.262604	0.095421
USP53	7.308518	6.766618	5.843145	6.165299	7.219515	7.163048	7.173117	7.979064
USP54	6.477735	5.027747	4.928787	5.616931	5.048344	4.772187	5.008274	5.937197
USP6NL	13.41028	14.43361	15.0591	13.99488	12.43805	14.93802	13.9196	15.19312
USP7	64.25282	60.08776	60.76925	63.18132	60.64611	64.41823	61.85357	61.14208
USP8	28.2008	30.29964	29.03158	27.29985	28.52195	27.71425	27.97266	26.33501
USP9X	19.59379	19.34556	17.65941	17.82102	20.23023	17.80856	18.41987	20.65946
USPL1	5.948029	5.00472	4.778779	4.812129	5.285462	4.67447	4.987059	5.236236
UST	9.919955	12.29257	10.74353	8.949094	11.00124	9.366446	10.1981	13.80945
UTP14A	38.23342	45.56093	44.68352	41.44786	39.35586	40.46613	38.90798	39.24895
UTP15	10.83149	10.69787	9.982806	9.580059	10.83289	10.07387	9.660204	10.65249
UTP18	17.3922	21.94216	20.14646	21.84162	20.76256	21.92644	22.1651	20.12939
UTP20	9.353382	8.521658	6.993742	8.259228	8.218745	7.403236	7.577917	8.949868
UTP23	7.428733	8.098984	8.080561	7.287501	8.312527	7.110002	7.379092	7.403714
UTP25	15.94882	16.61792	14.83546	16.34388	18.39632	17.17679	16.28206	16.03496
UTP3	15.30945	16.12129	15.78034	15.31105	17.35519	17.45463	16.10013	16.12199
UTP4	15.88269	17.67991	14.65648	16.41753	16.02276	16.30698	15.61158	17.68659

UTP6	14.56529	16.14017	15.116	15.83584	14.76231	15.34254	14.70455	15.59389
UTRN	27.19698	26.50066	23.27873	25.35961	27.46531	24.4109	25.54739	26.68602
UTS2	0	0	0	0	0	0	0	0
UTS2B	0	0.040742	0.039713	0	0	0.040489	0	0
UTS2R	0.611334	1.272192	1.403232	1.388407	0.793184	1.031395	1.559644	0.854645
UVRAG	3.225324	3.34132	2.759425	3.459566	2.929658	2.876082	2.678006	3.086869
UVSSA	3.00935	3.660105	3.291564	3.521218	4.694899	2.896563	2.994745	3.425319
UXS1	13.66421	15.40997	12.78924	14.00268	15.2784	13.51806	14.87516	14.79694
UXT	26.69461	31.65797	28.38984	29.04843	26.92129	26.51742	33.84613	28.88564
V15	3589.31	3241.819	4320.668	3562.171	3380.16	4040.544	3358.281	2947.937
VAC14	18.89995	20.31555	19.38689	19.58377	18.14069	19.09969	20.33283	18.15262
VAMP1	1.050788	0.720472	0.482161	0.750508	0.594462	0.80149	0.775672	0.699732
VAMP2	30.14615	35.8022	32.87003	40.91255	31.98437	33.29107	29.30047	29.7107
VAMP3	44.76794	46.88473	46.05524	42.67943	45.89675	45.42591	45.33669	45.51726
VAMP4	9.091661	7.901688	9.090327	9.003204	7.811676	9.290735	8.00837	7.450454
VAMP5	5.242683	3.812278	4.133536	4.02941	4.820509	4.937951	4.978012	5.188672
VAMP7	9.478336	10.13807	11.02355	10.6269	11.11454	11.43841	10.38311	8.80937
VAMP8	83.19431	74.84704	82.80671	83.31385	81.69717	87.81067	84.0106	80.02639
VANGL1	14.52452	11.8477	10.54277	10.35166	12.82682	10.86199	11.14243	13.59026
VANGL2	9.876812	7.331155	7.13882	8.753497	7.864535	6.925747	7.426174	7.864485
VAPA	38.97789	40.53771	40.63283	41.45334	39.61824	39.13208	38.31958	37.95863
VAPB	36.97022	37.12654	37.05573	39.43839	42.59039	41.48374	37.17089	37.27703
VARS	21.01043	20.35623	20.68865	22.03577	21.43841	19.26486	21.35984	22.84289
VARS2	4.739312	3.472079	2.991506	4.835249	3.713756	2.986698	4.633289	3.369701
VASH1	1.770482	2.011559	1.874922	2.025307	2.649203	1.791789	2.279964	2.088006
VASH2	1.564649	1.682733	1.854184	1.221055	1.625038	1.551102	1.856877	2.178967
VASN	8.702602	9.849702	8.998963	9.704432	11.71009	9.22767	8.67312	9.061009
VASP	29.54886	30.98598	27.82091	29.91867	33.15993	28.58523	32.77486	34.18377
VAT1	94.27714	87.44803	93.81814	105.054	92.92134	90.29641	94.48946	83.41319
VAT1L	3.920235	2.640393	1.629299	4.713081	1.34342	2.234438	4.579229	4.064892
VAV1	1.258382	1.882465	1.466	1.803358	1.740331	1.237284	1.616529	2.692186
VAV2	9.329721	8.481527	6.870682	9.800427	9.422184	9.324557	7.767906	8.114877
VAV3	5.8846	6.369671	6.7694	7.00375	5.837861	6.749081	6.707426	6.005071
VAX2	1.650769	0.973506	0.36497	0.706962	1.28136	0.769007	1.375436	0.849629
VBP1	14.22159	18.02044	17.65769	15.54546	16.58733	19.19922	15.57345	16.61813
VCAN	16.38374	13.44727	12.24729	13.18101	20.64967	10.86918	15.02204	17.92635
VCL	42.85117	41.78632	37.8842	43.49057	43.38701	38.14224	41.93162	40.53732
VCP	154.7083	151.6371	149.7141	161.284	156.3147	153.4584	163.4348	156.3234
VCPIP1	5.290592	5.349341	4.339427	4.80923	5.403781	4.707347	5.826664	5.661834
VCPKMT	3.320951	3.473459	2.972849	3.868399	3.835119	3.878861	3.35864	3.234952
VDAC1	40.38943	46.22886	43.76399	39.46865	51.57109	42.27056	48.03758	46.33948
VDAC2	116.0298	127.2664	136.796	121.4341	122.1192	123.119	132.6058	124.6459
VDAC3	49.02943	58.12493	53.88543	50.60095	50.7587	50.94294	56.38578	50.25745
VDR	76.45587	64.11718	58.39924	63.08304	66.46491	62.75552	52.87838	63.71441
VEGFA	8.276191	8.062949	7.465164	9.290915	11.43826	7.479773	6.523487	9.137571
VEGFB	17.91952	18.58403	16.69983	17.43282	20.90881	19.54544	18.65941	15.43205
VEGFC	3.021009	3.955095	2.969744	3.604746	4.138903	2.902759	5.516583	3.483743
VEGFD	0.860795	0.997962	1.086687	0.472888	1.242546	0.643306	0.882715	1.026058
VEPH1	0.056992	0.051324	0.175098	0.162231	0.303994	0.312407	0.14783	0.160563
VEZF1	5.768742	6.332426	5.457941	5.286126	5.771708	5.569032	5.699824	7.086772
VEZT	4.038327	4.002384	3.972448	4.498215	4.196441	4.426272	4.044067	3.869792
VGF	0	0.021779	0	0	0	0	0	0
VGLL1	0.022004	0	0	0	0	0	0	0.022317
VGLL2	0	0.008013	0	0	0.00791	0	0	0
VGLL3	7.788494	6.114704	8.202662	5.85947	8.436665	5.010634	5.69613	4.85656
VGLL4	25.00895	24.3496	23.18901	24.22867	25.25436	24.77973	24.04808	23.84735
VHL	11.94337	15.0162	15.23599	14.23952	12.04418	13.70157	16.33126	14.75306
VIL1	0.201593	0.506512	0.278713	0.776765	0.137101	0.34099	0.474711	0.269891

VILL	0.104686	0.009642	0.046992	0.009753	0.066627	0.038328	0.019319	0.019305
VIM	671.9043	966.5885	1102.68	842.6029	858.7492	792.5862	910.256	1128.381
VIP	0	0.017082	0	0	0.033726	0	0.017114	0.017101
VIPAS39	10.07392	9.764785	9.399892	9.017678	9.595969	9.824753	9.351757	10.2836
VIPR1	3.194082	2.178077	2.183734	2.622757	4.105745	3.308669	1.85999	3.073421
VIPR2	0.968916	0.902468	0.895111	0.976901	1.156597	0.550705	1.237243	1.299712
VIRMA	22.05461	23.83466	21.48727	21.98365	21.90933	22.38423	21.2178	23.27994
VIT	16.27564	17.48165	25.32014	18.27226	13.31815	14.0626	15.35171	19.69625
VKORC1	30.64426	32.20461	30.59018	34.8422	33.37743	40.0243	29.90618	29.36004
VKORC1L1	12.09449	13.10358	13.72557	12.65821	13.08753	13.4281	14.11394	12.32029
VLDLR	3.283089	3.334576	2.423449	3.678764	2.106092	3.414032	3.567955	3.540042
VMA21	17.37024	18.33172	18.67546	17.86392	19.00638	19.10882	18.22121	18.86649
VMAC	7.141384	7.149653	7.468882	7.779272	6.678338	7.614787	6.877518	6.187957
VMO1	1.811196	1.333485	1.788628	1.279644	1.057605	1.359182	1.43872	1.643013
VNN1	0.250276	0.2113	0.020596	0.235102	0.448469	0.157491	0.264614	0.222109
VNN2	5.806413	6.310962	5.761899	6.340183	7.287144	3.675084	6.565827	9.605475
VOPP1	1.529099	1.395213	1.069225	1.956286	1.291828	1.195299	1.330316	1.541234
VPS11	16.22258	13.96663	14.03507	14.26988	14.14436	14.40564	14.125	14.14967
VPS13A	2.901233	2.963415	2.57975	2.717066	2.893109	2.465868	2.770516	3.268333
VPS13B	7.370911	6.911202	6.050824	6.405377	6.926049	6.48359	7.645082	7.691801
VPS13C	4.8714	5.021991	4.225588	4.179653	4.642512	4.247221	4.787772	4.629702
VPS13D	11.74996	11.75391	9.619389	10.98767	11.13053	9.972265	11.26802	11.97321
VPS16	9.442766	10.80145	8.780882	9.948178	9.507583	9.219176	11.84336	10.44784
VPS18	6.029982	5.396388	5.104155	5.612906	6.016402	5.912169	5.566678	6.246876
VPS25	52.00972	57.7499	53.75442	57.27931	55.79755	59.36806	52.78986	48.85211
VPS26A	50.86256	54.62986	56.53412	54.05813	52.06953	56.68762	52.72391	50.33936
VPS26B	18.97418	19.38263	14.34193	19.3981	18.42577	16.37631	17.05681	18.21823
VPS26C	11.05975	10.766	10.02437	11.07256	12.10614	10.53664	11.84642	9.933509
VPS28	41.5053	50.19342	43.82305	50.45172	46.43931	54.55431	46.52278	52.40789
VPS29	53.59912	58.63592	59.47837	51.83116	58.95328	60.88253	59.31431	59.21541
VPS33A	5.906435	7.002632	7.356622	6.700784	6.628205	7.691345	7.054111	6.407415
VPS33B	5.135706	5.309113	4.158978	4.565963	5.258457	4.942539	4.805635	5.023103
VPS35	51.64463	54.94288	56.15199	54.54759	56.10549	56.88676	55.1837	55.15083
VPS35L	10.81795	12.43691	9.652468	10.37288	9.576696	9.907749	11.27448	10.45108
VPS36	28.52493	27.88233	26.77684	27.59701	26.34832	29.58164	31.91559	28.30017
VPS37A	4.847899	4.91947	5.08187	4.791129	5.860597	5.339107	5.382423	5.553405
VPS37B	5.199715	5.136022	4.390867	4.975822	5.42366	6.115581	5.702571	4.792591
VPS37C	3.816478	3.412862	2.840206	3.288923	3.418852	2.740385	2.924233	3.073432
VPS37D	0.312215	0.632625	0.272051	0.658718	0.312255	0.961535	0.298257	0.670573
VPS39	14.01229	13.32911	12.45626	13.76662	13.4474	12.72336	12.44916	13.14618
VPS41	22.94691	24.37379	21.24779	22.85971	21.847	20.28987	23.17149	20.97695
VPS45	8.760616	10.84794	9.742426	9.191373	9.982521	10.04164	10.90724	11.22027
VPS4A	30.05564	31.36682	30.0434	30.44928	29.68042	28.48815	26.60939	30.80595
VPS50	11.19567	11.73072	11.47309	12.06586	11.22839	12.04357	12.11747	10.67311
VPS51	14.12765	14.81863	13.11934	13.57985	11.93353	13.30864	13.1915	12.96787
VPS52	17.41058	19.89048	18.98918	18.70759	19.84659	20.07204	18.34282	17.51873
VPS53	9.544692	9.690864	9.278008	9.796979	9.530446	9.355365	9.132774	9.047405
VPS54	9.357137	10.576	9.353007	9.802868	9.086265	9.644007	9.375484	9.881762
VPS72	18.77867	21.30512	19.83987	20.40457	20.18223	20.45253	21.26893	18.69833
VPS8	10.87165	10.91406	9.527275	10.59111	10.00821	10.92494	10.90783	9.737272
VPS9D1	3.275785	3.547282	2.99102	4.171395	2.653192	3.017009	3.292217	3.202593
VRK1	10.44178	12.48095	13.54922	11.51443	10.42868	13.79937	10.39318	11.98197
VRK2	7.441272	14.37899	12.56646	14.70973	9.071599	11.79231	13.57137	12.93594
VRK3	5.967633	6.827221	6.602892	7.148166	6.906172	7.067151	6.163882	6.799161
VSIG1	0	0.038191	0	0	0.037701	0	0	0
VSIG10	1.655468	1.603874	1.491903	1.696474	2.026627	2.021992	2.681134	1.76161
VSIG10L	3.147152	2.471524	2.402981	4.22381	4.861022	2.218681	4.618274	4.551839
VSIG2	0.029323	0.059416	0.130311	0.150249	0.087982	0.221428	0.044645	0.059482

VSIG4	1.003831	1.076829	0.860118	0.423581	1.254951	0.371577	0.958954	1.676905
VSIG8	369.5832	280.9212	378.7915	333.1767	261.6308	320.1545	341.4349	328.2006
VSIR	30.5903	27.95574	26.55137	33.75564	32.34455	30.87169	29.26066	33.82198
VSNL1	5.79246	7.355161	5.521981	9.149263	6.349356	7.931562	7.243355	5.248261
VSTM2A	0	0.009668	0	0	0	0	0	0
VSTM2B	0.246897	0.250137	0.066497	0.253013	0.179585	0	0.227819	0.159353
VSTM2L	0.658749	0.605311	0.92286	0.989051	1.057199	0.555278	0.746378	0.605975
VSTM4	3.107238	2.961408	2.957801	3.061104	2.987499	3.692879	3.844766	3.744403
VSTM5	1.680452	1.311767	1.197026	1.044539	0.826559	1.442303	0.363501	1.48085
VSX2	0	0	0	0	0.025776	0	0	0
VTA1	21.72919	25.55893	24.9706	25.43791	25.5492	26.46277	24.24157	25.2204
VTCN1	7.455461	6.53259	7.248858	6.096376	5.949786	6.434052	6.077209	6.422976
VTI1A	2.657467	2.279395	1.835255	1.97475	2.024134	1.779685	1.929297	2.095661
VTI1B	56.13092	57.02217	55.77595	47.9158	50.77507	61.27695	64.27426	61.50648
VTN	0	0	0	0	0	0	0	0
VWA1	9.778078	8.982124	8.96321	11.95003	9.627271	9.31495	10.47085	9.573255
VWA2	0.617878	0.748306	0.427827	0.662295	0.873665	0.479088	0.475766	0.590658
VWA3A	0.081959	0.041518	0.055645	0.020997	0.020493	0.04126	0.07279	0.025977
VWA3B	0.103345	0.145418	0.062368	0.070603	0.057421	0.127173	0.09324	0.069877
VWA5A	12.91635	16.76508	12.07512	15.86053	12.43125	12.60503	11.73448	11.66489
VWA5B1	0.069482	0.020704	0.008073	0.020942	0.03679	0.004115	0.012446	0.012436
VWA5B2	0.051747	0.110677	0.039746	0.05892	0.092006	0.063678	0.058359	0.093304
VWA7	0.110484	0	0.101833	0.03774	0.066299	0.081575	0.029904	0.052293
VWA8	9.142518	9.897647	9.798181	10.92155	8.956471	9.222508	9.650842	9.034227
VWC2	0.457475	1.200832	0.492845	0.255713	0.41594	0.062809	0.358806	0.674891
VWC2L	0	0	0.034988	0	0	0	0	0
VWCE	0.10032	0.033879	0.181628	0.025701	0.091972	0.067337	0.076368	0.08479
VWDE	0	0	0	0	0	0	0	0
VWF	23.13178	22.27153	20.23972	25.43614	29.3433	21.24401	27.32097	24.6424
VXN	0.024806	0.167542	0.195973	0.245729	0.173663	0.149851	0.092319	0.22643
WAC	35.9567	35.184	34.23113	33.76947	35.15396	35.34411	34.77075	35.54299
WAPL	32.00783	30.78859	29.67928	27.79449	28.94341	30.12108	29.49493	29.59826
WARS	33.99951	34.90903	38.95833	39.968	40.87376	39.71939	37.7871	31.08533
WARS2	4.767563	5.428149	6.066158	4.480181	4.560585	4.682579	4.411131	5.335423
WAS	1.322115	2.009199	0.861723	1.707128	1.785085	1.064917	1.48956	2.50755
WASF1	0.570572	0.514999	0.840072	0.531551	0.726277	0.501356	0.884491	0.61026
WASF2	33.79003	31.52517	28.17031	30.00513	31.91854	29.53342	28.31578	32.61388
WASF3	6.515249	8.11252	6.925264	7.834504	7.928627	6.473201	8.420483	9.205109
WASHC1	17.00046	14.41122	16.73997	17.18591	15.35989	16.22356	14.93807	17.69228
WASHC2C	20.83922	21.85106	23.04989	24.11399	21.83345	20.99481	25.31687	23.63969
WASHC3	36.24031	46.8128	46.3656	39.02805	43.72052	45.41438	43.02404	38.59402
WASHC4	7.073726	7.865853	7.566575	7.628719	8.93712	8.348704	8.054389	8.297338
WASHC5	14.44895	16.76926	14.58942	15.55186	14.50925	17.00495	17.07703	16.45191
WASL	13.72743	13.91949	14.07243	13.07468	14.82731	14.93258	13.97506	14.39925
WBP11	18.51513	17.38201	15.45918	14.32818	17.16755	17.51337	16.77656	16.13539
WBP1L	6.876648	7.093066	6.060753	7.158652	7.438016	7.71904	7.343206	6.737715
WBP2	49.87703	50.54603	55.15443	55.20792	52.75672	51.2774	44.89431	46.17314
WBP4	12.84952	15.87376	14.951	14.90936	14.27103	15.51433	14.73566	14.60895
WDCP	2.092998	1.444046	1.600196	2.052591	2.183302	1.827834	1.796978	1.757582
WDFY1	17.2063	16.53152	15.78336	15.4924	16.81272	17.01734	15.83908	17.00663
WDFY2	8.041753	6.866887	6.36755	5.733193	6.376268	6.232588	7.345324	9.00533
WDFY3	10.59862	8.155329	7.55735	7.51802	9.93958	8.231313	9.161813	10.0718
WDFY4	0.3932	0.47691	0.36369	0.360375	0.454177	0.195155	0.379424	0.766701
WDHD1	7.076066	7.835164	5.817611	6.802083	5.494423	5.786425	7.168302	7.392172
WDPCP	1.253342	1.146699	0.745161	0.799466	0.850592	0.772594	0.889201	1.271184
WDR1	67.5421	59.77372	57.19941	56.42187	62.20515	57.43705	64.99821	57.4446
WDR11	10.7919	9.878999	9.392319	9.957399	10.25563	9.362748	9.961146	10.97453
WDR12	10.65879	11.42453	9.328085	10.63507	11.27799	11.86244	10.61348	10.94723

WDR13	19.80193	19.58285	18.63208	20.65578	19.97642	18.87706	18.37588	17.17423
WDR17	2.478595	3.79413	2.046447	1.950102	2.993663	2.250066	2.19271	2.69933
WDR18	8.248767	7.919446	7.474729	8.329742	7.782461	8.04848	7.589149	8.215387
WDR19	3.948081	4.342025	3.575206	4.08659	3.839322	2.99504	3.357061	3.969027
WDR20	4.443458	4.817756	4.342462	4.411473	4.062785	4.485444	4.424129	4.213816
WDR24	5.983191	6.229836	4.934488	5.942448	5.403086	4.826678	5.324349	5.516696
WDR25	0.954824	1.602633	1.407353	1.343578	2.380242	1.377452	1.591136	1.618845
WDR26	46.68862	44.87788	53.47313	51.06085	46.86186	51.95392	50.17372	47.01847
WDR27	0.589628	0.183805	0.125414	0.227749	0.684964	0.351627	0.179542	0.57502
WDR3	16.93396	18.94697	20.12833	20.04619	17.46491	19.25831	18.78983	16.66281
WDR31	0.71456	0.860529	0.665713	0.980951	0.687684	0.665144	0.506326	0.546967
WDR33	13.81692	14.54337	11.82512	14.44527	13.78825	14.07485	13.41107	13.43186
WDR34	5.194033	4.420908	4.325347	4.805439	3.2243	3.770498	4.676991	4.739525
WDR35	5.308088	5.146002	5.566312	4.381709	4.657642	4.623896	4.93526	5.425293
WDR36	9.007893	9.060565	7.947111	7.808118	8.536206	8.367927	8.294378	9.974025
WDR37	6.485711	6.763798	5.970831	7.647323	7.160604	5.939957	7.276987	7.117954
WDR38	0.044463	0	0	0.022782	0.044468	0	0.157953	0.022548
WDR4	2.445766	2.392953	2.208374	2.342382	2.007921	2.025213	2.347116	2.523085
WDR41	12.6016	12.99673	14.68397	14.40763	13.04062	15.98192	14.63195	13.94738
WDR43	11.76105	14.07805	11.28894	14.02873	13.43588	14.16487	13.14209	14.10185
WDR44	6.263921	6.577889	5.421774	5.667545	5.336124	5.331239	6.521778	6.318972
WDR45	23.18259	24.15488	31.82161	27.57998	24.64337	26.78271	21.98043	23.44222
WDR45B	5.920165	5.52616	5.107199	6.235638	6.1251	5.632469	5.885371	6.810847
WDR46	19.79408	21.84973	20.58761	22.7064	22.77706	18.79123	20.62554	20.75331
WDR47	16.29732	16.23045	16.82403	15.17966	16.17405	16.18943	15.38988	13.6116
WDR48	16.45505	17.80441	16.47377	16.52888	17.15824	17.30887	17.02528	16.53131
WDR49	0	0.00596	0.005809	0	0	0	0.011942	0
WDR5	21.81234	17.85893	20.25265	22.30858	19.92318	19.71039	20.19032	20.61118
WDR53	4.002581	4.735842	4.32372	5.271532	5.123098	5.288366	5.177273	4.162148
WDR54	1.231059	1.529603	1.490977	1.594793	0.952452	1.099151	1.461708	1.2957
WDR55	18.24279	19.09828	16.44746	18.85046	17.58629	18.50347	19.59655	17.56023
WDR59	7.140777	7.732903	6.021267	8.417597	5.113994	7.309636	7.278152	8.535182
WDR5B	4.206292	5.805661	5.561314	4.51333	4.949219	5.45073	4.952489	4.587389
WDR6	11.62679	12.16071	8.72464	12.32154	11.19711	11.54775	11.90539	13.34703
WDR60	2.25582	2.308906	1.731817	2.240447	2.232932	2.209005	2.266133	2.319275
WDR61	29.89134	36.5239	34.01381	37.54711	33.83594	34.88347	35.76408	30.82214
WDR62	1.347821	1.241372	1.05445	1.479865	0.905958	1.255692	1.301413	1.344816
WDR63	0.158131	0.028272	0.036744	0.095322	0.009303	0.018731	0.028324	0.028303
WDR64	0.020945	0.007073	0	0.007155	0.006982	0	0	0
WDR66	0.465395	0.803029	0.502684	0.350241	0.589092	0.585718	0.774992	1.320182
WDR7	4.891417	4.105548	3.83181	4.115196	4.280085	4.07636	4.057366	4.128634
WDR70	12.25219	14.47142	13.72901	12.49258	13.01732	13.50129	14.91066	14.06267
WDR72	0.056521	0.171788	0.173032	0.081089	0.18089	0.273154	0.103264	0.108919
WDR73	2.949201	2.781844	3.961384	2.755925	2.983486	2.559784	2.649366	3.529828
WDR74	23.24562	24.07455	24.35465	26.47081	27.45316	27.27498	25.4426	22.9381
WDR75	18.70548	18.81104	16.69921	18.37012	19.46766	20.52191	19.27646	17.40079
WDR76	5.440147	6.541075	4.931154	6.4457	5.405805	6.147643	6.295328	6.370552
WDR77	14.61182	14.31053	12.70662	12.58963	11.31345	13.45679	13.98764	14.04934
WDR78	0.124535	0.147197	0.07174	0.202064	0.300998	0.08359	0.284406	0.199987
WDR81	2.446263	1.899421	1.658193	2.510869	1.929862	1.635415	1.950611	2.151599
WDR82	44.11734	42.72042	41.66893	41.12784	43.56277	40.38699	41.71146	45.74144
WDR83	17.48168	19.02131	17.24105	16.58945	15.35908	18.71716	16.07968	16.58286
WDR83OS	74.96141	70.31461	75.18457	72.94129	63.41906	72.60828	71.49825	65.97742
WDR86	0.382223	1.161719	0.25736	0.391691	0.469153	0.577252	0.229247	0.176211
WDR87	0.097123	0.041745	0.061036	0.063337	0.088305	0.050375	0.06572	0.04179
WDR88	0	0	0	0	0	0	0	0
WDR89	5.696759	6.860163	6.93958	5.645604	5.970429	6.096303	5.799522	6.296825
WDR90	1.547212	1.635671	1.134087	1.994232	1.633913	1.393296	1.360706	1.695946

WDR91	5.893015	6.6911	5.746186	6.768017	6.276075	6.211228	6.326284	6.504596
WDR92	5.176963	5.768087	5.367442	5.583026	6.4559	6.317187	6.643618	5.997013
WDR93	0.273974	0.155439	0.205626	0.370604	0.219207	0.165507	0.177973	0.222299
WDR97	0.261706	0.346723	0.16567	0.323202	0.234895	0.290519	0.095355	0.258626
WDSUB1	10.06753	11.31366	9.434407	9.54131	8.92627	9.345532	9.290903	7.777625
WDTC1	18.62778	17.25082	17.98431	18.49008	18.35976	17.76909	17.40886	16.71385
WDYHV1	10.53577	10.807	13.4592	10.56621	10.16215	11.476	12.06384	11.12307
WEE1	15.55435	14.92447	14.28231	14.74971	14.19572	13.67141	14.90813	14.0444
WEE2	0	0	0	0	0	0	0	0
WFDC1	20.51114	18.10408	22.05862	19.00885	20.57207	22.17664	22.94801	22.24123
WFDC2	1.139382	1.65622	0.831659	1.522963	1.486342	4.488909	2.162101	1.859012
WFDC3	3.540798	3.871971	2.525379	2.591789	2.641889	3.253758	2.937879	2.764657
WFDC5	0.195501	0.198066	0.225242	0.166953	0.391052	0.295254	0.330722	0.330473
WFIKKN1	0.765292	0.690238	0.847922	0.468636	0.933404	0.911469	0.663098	0.634201
WFIKKN2	0.071547	0.036243	0	0.007332	0.050089	0.007204	0.065358	0.145131
WFS1	4.429461	3.753396	3.972625	5.941097	5.354498	3.737529	5.869442	5.707522
WHAMM	7.585496	7.437144	7.44451	7.754103	8.414777	7.334099	8.033615	7.960748
WHRN	0.094691	0.103929	0.101304	0.072778	0.173623	0.063559	0.104121	0.088036
WIF1	12.39137	11.68686	6.711411	8.901906	8.483436	8.97759	10.56747	10.3263
WIPF1	6.55588	7.068392	7.101597	6.993872	7.043999	5.929612	6.428719	8.981569
WIPF2	8.653874	6.311644	6.588508	4.863599	6.117403	4.938123	7.082131	7.203169
WIPF3	2.009649	2.103219	2.312101	2.426455	2.421177	1.996666	2.935148	2.966572
WIPI1	31.07054	34.493	37.96269	39.81182	33.56717	32.45381	28.77695	30.10343
WIPI2	17.92283	18.31885	18.65803	19.68049	18.99358	20.3501	18.27222	16.59297
WIZ	4.504322	4.500251	3.900727	4.84669	4.484112	3.7118	4.068979	4.15377
WLS	143.9566	150.8513	133.3338	123.9916	119.637	146.2136	144.47	126.7081
WNK1	17.22885	16.386	14.53128	14.83803	16.7391	13.14988	15.63484	16.77274
WNK2	6.322541	4.784187	4.520074	4.936902	4.867949	5.201118	5.988151	5.916363
WNK3	0.451929	0.361005	0.240314	0.280546	0.321606	0.28001	0.370495	0.458362
WNK4	7.565756	7.162423	4.858507	5.359427	6.278047	6.361744	6.483295	6.862785
WNT1	0	0	0.024834	0	0	0	0	0.051011
WNT10A	7.94503	7.548978	7.114502	7.204107	7.379519	8.291786	6.678401	6.747029
WNT10B	4.132715	4.695648	3.648942	4.868367	3.669707	4.86092	4.090165	3.838987
WNT11	13.75661	16.28593	11.52316	12.53973	12.99062	13.71146	16.81476	12.31739
WNT16	0.58749	0.778903	0.816535	0.423659	0.776167	0.766763	0.795069	0.765046
WNT2	1.632387	1.984573	2.504874	1.158108	1.05491	2.66759	2.243151	1.719303
WNT2B	3.836206	4.078323	3.713636	3.013081	4.227945	3.544781	3.560732	3.35327
WNT3	7.2508	7.064122	8.587227	8.193676	8.60695	9.48682	6.576242	7.044568
WNT3A	2.456088	3.162242	1.718051	2.674234	2.123767	2.936529	2.311153	2.361309
WNT4	2.188026	2.807333	2.105561	3.216037	2.004619	1.929612	2.780111	2.769918
WNT5A	10.53619	14.0869	11.14266	9.926007	9.532747	11.19175	12.04735	13.21402
WNT5B	0.357404	0.52051	0.683841	0.43493	0.558515	0.157432	0.634838	0.770294
WNT6	3.941331	3.202162	2.858057	4.273102	4.113225	4.255812	4.019777	3.456722
WNT7A	0.159786	0	0.026299	0	0.026634	0	0.081091	0
WNT7B	1.592556	0.766392	0.609426	1.509603	0.935747	1.102362	1.0911	1.938272
WNT8A	0	0	0.010566	0	0	0	0	0
WNT8B	0	0.028131	0	0	0	0.027957	0	0
WNT9A	0.84978	0.969519	0.869433	0.698233	0.89583	0.624346	0.536166	0.559056
WRAP53	9.239724	9.877664	8.739467	9.576193	9.435938	9.151825	8.891135	8.032507
WRAP73	6.66906	7.482124	7.219511	7.078881	7.162332	5.152382	6.133073	6.219247
WRB	10.38916	12.29653	12.59414	12.31575	13.64872	13.76018	11.91605	10.55721
WRN	4.806499	5.72497	4.31064	4.461843	4.056525	4.805998	4.80018	5.04283
WRNIP1	18.04866	17.63579	15.33568	20.53777	19.12764	16.76525	19.37717	17.20215
WSB1	56.96394	56.6676	51.03356	65.68618	59.25643	58.25523	57.96619	52.12915
WSB2	38.06077	35.22355	39.0518	33.83149	37.62725	36.95137	39.2706	36.93809
WSCD1	0.233974	0.879041	0.308078	0.439578	0.614262	0.5693	0.30675	0.425171
WSCD2	0.978414	0.680662	0.547526	0.568168	1.089442	0.512251	0.609095	0.86003
WT1	0	0	0	0	0	0	0	0.01053

WTAP	21.27326	22.08106	19.35197	22.06755	21.17164	20.63059	21.25947	20.91082
WTIP	8.439946	6.575744	6.212804	6.651336	8.219487	7.661225	6.835249	7.178347
WWC1	10.32836	10.04812	8.666337	10.78747	10.2476	10.18543	11.3997	9.816341
WWC2	7.459327	8.546256	7.4735	8.764206	7.702292	7.707704	8.718756	7.552822
WWC3	13.33536	12.86724	12.18283	13.63839	12.36033	12.99739	13.2335	12.86785
WWOX	1.896948	1.822652	1.873312	2.571012	3.096709	2.094737	2.534078	3.20245
WWP1	3.131526	3.897902	2.700959	3.284338	4.765823	4.247016	3.413254	4.721339
WWP2	10.97583	9.667353	8.599939	9.479199	10.19657	9.944127	10.54221	9.941856
WWTR1	39.35884	35.97179	34.91728	35.69387	39.93811	34.64595	32.53008	33.20192
XAB2	12.47034	12.25514	11.81722	13.27074	12.49634	11.72068	12.16232	15.46766
XAF1	1.262523	1.475876	1.054978	1.234082	1.787187	1.740495	1.656042	1.319896
XBP1	69.4289	76.68235	90.44255	93.01198	94.90736	91.41554	85.46708	71.6967
XCL1	1.387074	1.706408	0.929501	1.77679	1.089984	0.448891	1.609005	1.356576
XCR1	0.249347	0.543131	0.320112	0.076657	0.224441	0.112973	0.265741	0.379344
XDH	9.499281	5.950549	7.044451	6.291716	5.584359	6.658	8.837289	6.425006
XG	58.77662	68.43993	58.0628	48.05003	57.36924	59.12143	54.25096	61.25146
XIAP	9.343411	8.650499	9.1986	9.713739	10.37561	8.613312	8.620663	9.526406
XIRP1	0	0	0	0	0	0.004518	0	0
XIRP2	0	0	0	0	0	0.002321	0	0
XK	4.18616	4.401645	4.857775	5.101812	5.362147	5.424672	4.356181	3.810462
XKR4	0.023371	0.023678	0.073855	0.0479	0.014024	0.084711	0.042699	0.023704
XKR5	0.028663	0.159714	0.056611	0.16155	0.043	0.086576	0.029093	0
XKR6	0.415536	0.446376	0.414482	0.40443	0.352938	0.27331	0.387858	0.44263
XKR7	0	0	0	0.012619	0	0	0	0.012489
XKR8	1.769277	1.576858	1.458216	1.785838	2.128729	1.526883	1.741807	1.888909
XKR9	0	0	0	0.044434	0.021683	0.021828	0	0
XKRX	13.35079	12.04573	13.58123	11.79116	13.21552	12.66048	10.10304	11.26242
XPA	7.127971	7.919598	6.745857	6.330595	7.556626	6.710182	6.969611	7.602963
XPC	16.8467	16.84322	13.98803	18.6118	17.14447	15.8682	15.89194	17.31135
XPNPEP1	14.88174	13.11319	13.11628	12.62281	12.53469	11.92683	14.48037	14.23021
XPNPEP2	1.426583	3.046678	1.025189	2.264947	1.366482	1.874646	2.297737	2.35035
XPNPEP3	3.281732	3.288923	3.252499	2.612996	3.329382	2.377088	3.462759	4.046821
XPO1	27.52261	27.76262	24.54125	22.31994	25.16918	24.77978	24.75416	29.97301
XPO4	7.096988	6.957727	6.081519	6.733924	6.102575	6.285598	6.70196	7.004735
XPO5	18.01083	16.79873	16.93107	18.62719	18.83787	18.44447	16.50468	17.65351
XPO6	21.73883	21.84767	17.30668	20.48551	22.36475	19.16238	23.38405	23.98474
XPO7	26.25859	26.5623	26.09091	25.5553	26.62545	25.20668	26.45339	27.0946
XPOT	56.75325	59.84671	69.57447	57.95043	59.30897	66.80111	60.25081	56.06253
XPR1	5.919499	5.733339	4.976873	4.857951	5.561247	4.694957	5.708234	5.700366
XRCC1	11.54496	12.05266	11.03153	12.38878	10.61639	11.31554	11.53396	10.20252
XRCC2	4.099169	3.397881	2.784928	3.932357	3.434883	3.478182	4.048206	3.953222
XRCC3	4.108918	2.872516	3.024574	4.241773	3.396741	3.434769	3.385688	3.81372
XRCC4	5.144691	7.369582	5.534812	5.970422	4.634228	4.974016	5.256443	5.891768
XRCC5	10.29625	9.478031	9.692515	9.586985	9.827021	9.121716	10.30632	10.0877
XRCC6	22.15369	24.23678	20.01322	19.82067	14.11523	19.55346	25.30009	19.22859
XRN1	8.048958	7.612311	6.613549	7.550457	6.908975	7.096961	7.815154	8.053945
XRN2	23.3988	23.59088	21.54481	23.46945	22.50776	23.05862	25.09642	23.73909
XRRA1	0.790139	0.658881	0.654247	0.741197	0.729454	0.783298	0.684777	0.869197
XXYLT1	3.431858	5.114718	4.944969	4.752332	4.891544	4.772594	4.686164	4.446419
XYLB	15.48782	7.60046	6.305507	6.873754	7.150525	9.309266	8.645164	7.330134
XYLT1	2.23102	2.011231	1.717663	1.974518	2.194428	1.432533	1.762306	2.605626
XYLT2	4.624437	5.160194	3.880197	6.048007	4.722064	4.330433	5.473369	4.460676
YAE1	10.05184	11.47136	10.8679	10.36002	9.966471	11.02204	11.90305	9.462536
YAF2	4.968149	4.926051	4.478375	4.992545	4.198436	4.992395	4.915627	4.384601
YAP1	48.76107	47.00317	53.27902	42.78401	49.70863	48.81383	56.36016	58.83114
YARS	32.84942	36.10581	39.81389	38.06055	38.5228	37.87561	33.83895	28.26062
YARS2	5.534165	5.962389	5.715093	4.901699	5.543044	5.81852	5.90715	5.199014
YBEY	5.751952	6.022618	5.734639	6.289272	6.275668	7.730899	6.871794	8.653057

YBX1	278.0257	296.1372	276.1716	274.9061	275.9962	281.8037	278.6289	269.6547
YBX2	0.241928	0.829578	0.679984	1.029823	0.614203	0.487161	0.35889	0.792739
YBX3	271.04	225.12	290.253	252.9016	248.7909	265.2631	241.2265	258.0451
YDJC	0.823211	0.548694	0.556231	0.910202	0.69332	0.327172	0.461756	0.966761
YEATS2	11.86597	10.86018	9.702827	10.85187	9.604842	10.44644	10.95399	12.12016
YEATS4	12.07135	14.30262	17.33587	14.9283	10.76331	18.21012	14.4537	12.3677
YES1	13.55854	13.98098	14.94955	14.32156	14.42152	14.7004	14.391	14.30228
YIF1A	18.10039	18.27173	19.29452	16.8077	18.66912	18.50913	17.576	17.60694
YIF1B	10.66352	9.695957	10.79545	13.18928	10.64427	11.73317	9.651105	10.18774
YIPF1	8.213821	8.236829	6.883538	7.559625	7.808324	6.163328	10.90974	7.700184
YIPF2	9.730305	10.43404	10.13227	10.22293	10.61038	9.991933	11.48951	9.90814
YIPF3	43.13084	41.48416	39.62095	42.52528	40.2729	40.74605	41.33743	37.13658
YIPF4	15.40381	17.01955	17.18225	16.55748	17.97343	19.94822	15.90394	16.38726
YIPF5	28.96732	33.05662	34.70576	32.14785	30.81582	32.06344	31.50095	29.25213
YIPF6	12.4338	13.38363	13.03529	12.97835	13.12276	13.38502	12.50316	12.79704
YIPF7	0.343425	0.049705	0.012112	0.226242	0.073601	0.592751	0.186738	0.236356
YJEFN3	0.10517	0.134963	0.090011	0.150885	0.098171	0.014118	0.064048	0.113778
YJU2	6.780301	6.641689	6.151278	7.105215	5.647577	5.531208	5.949204	6.04829
YKT6	33.61626	33.86361	35.64247	35.01406	33.65437	38.33301	34.80588	32.20002
YLPM1	17.12063	16.33331	14.48243	14.55534	15.61832	13.11852	15.43449	16.14134
YME1L1	59.70049	59.78155	62.32247	56.63198	59.81039	62.34954	64.85549	56.7086
YOD1	33.68726	30.71357	35.62085	35.18016	34.87799	34.75271	36.06668	36.26868
YPEL1	3.236816	3.467581	2.252069	3.019416	2.551839	2.28047	2.314691	3.357505
YPEL2	11.62513	9.857257	9.003853	8.999313	8.827354	10.37256	10.27133	11.08469
YPEL3	85.96032	77.78753	88.69256	83.82164	83.95595	84.92437	79.31793	81.18856
YPEL4	1.6546	1.281888	1.7301	2.16579	2.183242	2.183874	1.340713	1.57944
YPEL5	17.98071	22.27261	24.25495	19.5932	23.33307	25.67854	21.4965	20.71024
YRDC	17.09647	20.26247	20.65907	20.01489	19.55166	20.4453	20.04372	20.35787
YTHDC1	35.97511	37.39707	33.18701	35.05829	35.80076	33.36337	34.44721	39.44356
YTHDC2	7.838042	7.872766	8.276183	7.554305	8.731488	7.93222	7.696191	8.036031
YTHDF1	13.31048	12.27869	10.51737	12.16884	12.60266	11.6412	14.1102	12.04375
YTHDF2	30.2734	30.05554	27.38343	29.83806	30.97135	29.54864	30.65903	28.16278
YTHDF3	22.50308	22.51022	20.60741	19.96841	21.7033	20.39625	21.63931	21.82909
YWHAB	176.6946	187.2069	188.3278	173.0168	182.7194	177.8718	174.3175	174.5372
YWHAE	174.6154	182.8756	192.0816	171.6046	168.8417	181.3785	177.3722	165.0378
YWHAG	84.0127	84.19169	83.91823	87.04337	95.69538	84.61698	90.16704	84.05483
YWHAH	14.55372	13.2095	14.24434	13.41947	15.07428	13.04586	15.77958	16.98407
YWHAQ	225.575	230.3215	239.9165	211.5869	236.5441	227.4652	230.8066	222.8361
YWHAZ	148.0528	152.0469	157.5247	151.3776	152.2142	150.8594	149.3593	157.8743
YY1	19.14115	20.5664	19.66109	20.38835	19.92537	20.15041	19.34063	19.53348
YY1AP1	2.698729	2.706715	2.15704	2.330855	2.81643	2.462716	2.152891	2.764611
YY2	1.286373	0.984249	0.815296	1.168705	1.063795	0.931742	0.888634	1.156691
ZACN	0.071859	0.054602	0.097575	0.101254	0.035934	0.054263	0.054703	0.027331
ZADH2	3.693052	4.440009	3.947877	3.948837	3.688184	3.669852	3.856941	4.282263
ZAN	0.009421	0.009544	0.006202	0.006436	0.006281	0.012647	0.003187	0.01274
ZAR1	0.019276	0	0	0	0	0	0	0
ZAR1L	1.433322	1.691496	0.99549	1.404263	1.496519	1.205238	1.231003	1.597502
ZBBX	0	0.009781	0	0	0	0	0	0
ZBED1	6.637252	8.094605	5.75069	6.814492	5.974299	6.644768	6.317349	6.985765
ZBED2	0.014379	0	0	0	0.014381	0.007239	0.051082	0
ZBED3	2.533998	2.888161	2.341283	3.108251	2.630323	2.213068	2.630463	2.823185
ZBED4	7.126373	6.064715	4.907714	5.280131	6.245123	5.388061	5.661056	6.72051
ZBED5	11.34381	13.49748	9.958909	13.09959	11.3982	13.07271	11.37434	11.83801
ZBED8	4.549203	4.042558	3.483606	5.807607	4.231691	4.395905	5.184839	6.099817
ZBED9	0.063969	0.0707	0.034457	0.101309	0.046529	0.040985	0.041318	0.058981
ZBP1	0.077793	0.15348	0.056607	0.104895	0.110562	0.131915	0.162076	0.207633
ZBTB1	10.15322	10.38639	9.710181	9.40417	9.829037	10.48572	8.974162	10.53782
ZBTB10	1.239515	1.357181	1.026394	1.289942	1.320523	1.193722	1.457373	1.382095

ZBTB11	9.850377	9.851777	9.088825	9.205121	10.40919	8.953967	8.797075	10.07595
ZBTB12	1.279992	1.926661	1.85393	2.186164	1.682493	1.681494	2.004468	1.730952
ZBTB14	4.469474	3.80606	3.716524	4.484622	4.303507	3.608054	3.907759	3.675121
ZBTB16	9.65973	9.29559	10.56526	5.337103	3.214516	6.250996	10.96873	23.31751
ZBTB17	9.583079	10.01062	7.353872	9.799404	8.762511	9.079768	8.621741	8.250637
ZBTB18	7.120432	5.900264	5.901486	6.168512	6.476591	6.009479	6.433198	8.088705
ZBTB2	2.334513	2.603186	1.994415	2.238656	2.14983	2.043442	1.963616	2.251134
ZBTB20	0.467948	0.334826	0.2455	0.314698	0.403658	0.229683	0.313182	0.602161
ZBTB21	2.850855	2.198359	2.625364	2.613948	2.851224	2.998159	2.905333	2.844614
ZBTB22	4.262397	4.292217	3.674639	4.896362	4.529382	4.421299	3.907655	3.033125
ZBTB24	5.445035	7.038288	6.138052	6.50879	5.840733	6.238403	6.052438	6.500979
ZBTB25	0.226203	0.312323	0.171987	0.336427	0.28229	0.151161	0.296647	0.263939
ZBTB26	2.554903	2.426657	2.316098	2.352279	2.395531	2.336223	2.294398	2.682373
ZBTB3	2.161595	1.695456	1.129304	1.600616	1.603971	1.867459	1.5712	2.178222
ZBTB32	0.024906	0.040372	0.044272	0.122509	0.024909	0.085258	0.045503	0.040417
ZBTB33	13.16361	13.04773	11.60845	11.33241	12.3316	12.47798	11.27792	12.52694
ZBTB34	3.117427	3.274046	2.878079	3.355029	3.794699	3.125947	2.915175	4.264362
ZBTB37	0.874401	0.514652	0.427641	0.537636	0.691283	0.385688	0.447985	0.836172
ZBTB38	9.83068	10.39473	9.194559	10.14856	10.60012	9.489869	9.339736	10.44293
ZBTB39	4.511622	4.553503	3.82616	4.443701	4.99978	4.223827	4.462101	4.758015
ZBTB4	7.126754	7.089832	5.904583	7.96018	8.779415	6.693508	6.608595	7.378677
ZBTB40	10.0664	8.002801	7.659901	7.383701	9.131285	7.622913	8.27812	9.317923
ZBTB41	3.381264	3.509683	3.14252	3.373208	4.068661	3.755156	3.617222	3.708731
ZBTB42	4.055248	4.624042	4.043981	5.02758	4.771497	3.994888	3.704469	4.51621
ZBTB43	5.89872	5.406979	4.894314	5.288619	5.456664	5.943811	5.673102	5.408083
ZBTB44	11.436	11.46969	10.14054	10.02645	10.51826	9.457026	10.03486	11.40039
ZBTB45	2.793595	2.61462	1.957425	2.95822	2.567779	2.772499	2.403423	2.846856
ZBTB46	0.725995	0.609946	0.419677	0.834707	1.192438	0.796324	0.467293	0.933882
ZBTB47	8.501285	9.35631	8.115056	8.974355	8.869343	8.670884	8.790418	8.980398
ZBTB48	4.162426	4.089265	4.135474	4.963527	5.008172	4.305158	4.186455	4.618263
ZBTB49	2.814887	3.074313	2.109504	3.109654	3.534039	2.844168	3.049611	3.320662
ZBTB5	5.467961	4.998662	4.554795	5.590966	5.074147	5.047049	4.977119	4.591751
ZBTB6	4.752535	5.108947	4.352962	3.754931	4.753149	3.871834	4.437181	4.452237
ZBTB7A	14.29802	12.8256	13.11701	13.7442	14.63976	13.15736	10.99319	11.96974
ZBTB7B	22.19763	20.56625	20.27955	23.51868	22.04342	20.54393	19.74084	20.73483
ZBTB7C	2.487184	1.667366	1.859533	2.214525	2.424484	2.310111	1.930051	2.360935
ZBTB8A	1.868744	1.962406	1.752149	2.232412	2.063234	2.378314	2.514825	2.156223
ZBTB8B	0.927922	1.151143	0.785452	0.630704	0.861753	1.020061	1.057169	0.777874
ZBTB8OS	14.1285	14.39872	14.54759	14.46131	13.4439	12.84297	14.85016	13.24302
ZBTB9	3.979416	3.651182	3.200091	3.140733	3.531658	3.415064	4.075994	3.692048
ZC2HC1A	3.57926	5.022824	4.688999	4.295761	5.168022	4.261145	4.320263	4.594999
ZC2HC1B	0.246818	0.425097	0.316865	0.429984	0.29622	0.944316	0.325676	0.125166
ZC2HC1C	0.693791	0.987855	1.012289	0.710977	0.993938	0.71741	0.926243	0.919206
ZC3H10	2.929099	2.906196	2.929974	2.94736	3.058162	3.162481	3.395559	2.763531
ZC3H11A	34.34759	32.44087	29.70755	31.74261	33.47209	29.43113	30.91482	37.36445
ZC3H12A	5.114507	3.957456	5.091926	6.020436	4.844304	5.117984	5.751581	5.166186
ZC3H12B	0	0	0	0	0	0	0.006124	0
ZC3H12C	1.2413	1.177474	1.249498	1.434123	1.449969	1.495876	1.427747	1.764504
ZC3H12D	0.102364	0.259268	0.151632	0.23977	0.336381	0.228211	0.170691	0.407868
ZC3H13	9.524112	8.736849	8.119353	8.546217	8.981383	8.347937	9.05052	9.77002
ZC3H14	35.78294	33.13312	30.93446	29.07688	32.50499	30.61306	31.14356	34.47067
ZC3H15	35.62257	38.91089	37.71779	38.40256	40.99657	41.13718	38.69898	35.31899
ZC3H18	16.32381	14.65643	13.36926	15.14736	15.33578	14.89766	14.37198	15.26408
ZC3H3	3.600796	3.242713	2.765723	3.33233	3.286257	3.162578	3.119115	3.462114
ZC3H4	9.580941	10.22791	8.610332	10.16974	9.189504	9.505537	9.90788	9.822607
ZC3H6	0.99626	1.49713	0.852269	1.33746	0.969132	1.036604	1.183324	1.335996
ZC3H7A	15.73202	14.74358	14.16462	16.84299	14.7257	15.53943	13.47722	16.56051
ZC3H7B	8.716004	8.73391	6.251785	8.002464	7.896214	7.647784	7.78808	8.416935

ZC3H8	12.86681	13.48578	12.16651	11.69712	11.09116	14.02137	13.56278	12.53006
ZC3HAV1	10.07424	10.40445	10.08657	9.107815	10.55488	8.48925	9.719988	11.23234
ZC3HAV1L	4.40855	4.721361	2.218228	3.572175	4.148115	3.54718	3.358374	3.970295
ZC3HC1	10.27234	11.76635	10.17032	10.60766	10.12897	11.16356	11.68133	9.55147
ZC4H2	7.069159	6.433826	7.819837	6.440834	7.174621	7.314794	7.042568	7.328827
ZCCHC10	5.08067	5.594944	5.671803	4.926886	4.95137	5.926513	5.974595	5.732874
ZCCHC12	0.025375	0.025709	0.01253	0	0	0.012774	0.012878	0.025737
ZCCHC13	0	0	0	0.050676	0	0	0	0
ZCCHC14	5.311388	5.161567	4.138755	4.71181	5.549929	4.389881	4.74735	5.328037
ZCCHC17	14.0434	18.38226	14.7362	17.6097	15.46113	17.05568	17.95383	18.08885
ZCCHC18	0.21981	0.267234	0.188128	0.30034	0.337086	0.162295	0.148738	0.267527
ZCCHC2	3.970943	3.606516	3.633651	3.386643	3.596693	3.254	3.232858	4.592268
ZCCHC24	4.591445	4.058839	4.19766	4.243885	5.646792	3.823207	4.069618	4.930737
ZCCHC3	5.464788	5.15229	4.690643	5.415392	4.936977	4.869909	5.95062	5.958749
ZCCHC4	4.01265	4.442895	3.557799	3.908352	3.093743	4.352761	3.84587	4.120172
ZCCHC7	6.549294	7.759132	6.952239	6.121258	6.048745	6.400725	5.922151	6.891352
ZCCHC8	5.084588	4.815606	4.421303	4.969062	4.934271	4.470604	4.402237	5.119628
ZCCHC9	10.24549	10.67213	10.53753	8.243876	8.44034	8.573313	8.488807	8.26689
ZCRB1	12.3947	14.24949	13.71604	14.03495	13.11722	14.2672	10.81398	11.92921
ZCWPW1	0.328568	0.431512	0.168246	0.324237	0.31644	0.15928	0.160573	0.320903
ZCWPW2	0.484752	0.459085	0.353831	0.345572	0.611288	0.350133	0.374366	0.577158
ZDBF2	0.115872	0.04048	0.051296	0.110553	0.035965	0.056321	0.11761	0.141837
ZDHHC1	2.834439	2.90163	2.557944	3.124584	2.827404	2.339671	2.381188	3.295124
ZDHHC12	11.37284	9.751335	11.25478	9.887967	10.17702	9.594344	9.404862	7.770774
ZDHHC13	19.94403	20.80734	19.35059	21.67889	18.72396	22.97568	19.34507	18.5039
ZDHHC14	2.373926	2.850901	3.270649	2.302192	3.312344	2.565032	2.515319	2.748325
ZDHHC15	3.570181	3.251319	3.312676	3.604481	3.403338	3.395119	3.400323	3.732627
ZDHHC16	7.314852	6.789583	6.675802	6.598314	7.987508	6.497509	6.639141	7.685534
ZDHHC17	5.187647	5.781302	3.877998	5.44596	5.924335	4.48821	5.412405	5.463391
ZDHHC18	7.47397	6.870436	5.99527	6.589962	7.78077	5.623397	6.07069	6.804999
ZDHHC19	0	0	0.023345	0.0969	0	0.023801	0	0.047952
ZDHHC2	6.105879	8.34246	9.351559	10.64348	11.9958	7.297332	6.788829	7.004325
ZDHHC20	28.91037	31.48051	30.53846	27.39362	27.68384	29.33003	27.46838	30.12757
ZDHHC21	8.707158	10.11237	9.357118	9.373004	9.176721	10.15794	10.72461	11.06766
ZDHHC23	3.807551	4.650354	4.352145	4.159802	3.981969	3.672295	3.957565	4.205228
ZDHHC24	2.915955	3.618037	3.126587	3.182954	2.816286	2.704228	2.924157	2.845863
ZDHHC3	13.60808	15.6956	15.46888	16.55265	17.14786	16.03049	15.5612	14.97781
ZDHHC4	47.10654	44.48834	43.16874	45.13545	45.01028	44.32868	45.00752	45.09114
ZDHHC5	28.13216	25.45282	23.048	27.40826	27.38965	25.84557	24.23789	25.17808
ZDHHC6	17.06395	19.47599	19.95452	18.32408	21.26946	20.94183	17.76427	18.49092
ZDHHC7	27.12414	24.77289	25.01847	24.5135	22.86191	25.98148	25.48813	26.12042
ZDHHC8	3.627288	3.196302	2.449151	3.829515	3.889293	3.15097	4.178298	3.071474
ZDHHC9	31.1106	27.24822	31.11409	30.66357	29.25661	30.38315	29.23217	26.23558
ZEB1	5.389956	5.204959	4.599404	5.046694	6.128217	4.783936	5.109099	5.19561
ZEB2	6.529578	8.855578	6.629944	8.529938	9.579384	7.534529	7.938084	8.653772
ZER1	8.267907	7.72047	7.276149	8.187408	7.705624	6.916349	8.457642	8.116374
ZFAND1	19.02756	18.78205	15.60807	18.2744	14.10503	14.92867	15.35299	16.86547
ZFAND2A	2.264534	2.136448	1.34889	1.559364	2.228877	2.147306	1.374237	2.588425
ZFAND2B	19.13981	19.32257	19.9687	18.99088	20.9214	18.70379	18.7184	20.23445
ZFAND3	25.412	23.44825	26.1706	23.45165	24.84584	25.05257	25.67152	23.99067
ZFAND4	2.008659	1.366069	1.118795	1.673797	1.327693	1.427563	1.552019	1.783478
ZFAND5	37.7556	38.1132	35.90734	35.49128	35.6674	35.5895	34.52255	36.86075
ZFAND6	58.18173	56.20016	60.32374	57.07506	57.1023	60.06317	58.23845	55.43134
ZFAT	1.225058	0.941194	0.991358	0.934578	1.129921	0.78117	0.991293	1.314976
ZFC3H1	13.59588	11.47251	10.42303	11.9411	12.66789	11.48194	11.0789	13.08228
ZFHX2	0.267944	0.24376	0.186303	0.215742	0.374623	0.176179	0.260862	0.257893
ZFHX3	1.147704	0.928338	0.548421	0.8821	1.194137	0.696588	0.955892	1.26261
ZFHX4	0.248936	0.35424	0.108843	0.173314	0.252769	0.233417	0.15816	0.188878

ZFP1	3.282951	2.985284	2.158956	3.746404	3.137122	3.459973	3.272824	3.240696
ZFP3	3.393523	3.564062	3.327845	3.538273	3.583503	3.08875	3.342236	3.237605
ZFP36	55.778	58.39466	45.41733	58.3914	58.99093	54.48782	49.99171	49.08328
ZFP36L1	117.1433	123.1043	120.5916	115.387	117.1679	101.0999	92.49198	108.3951
ZFP36L2	52.42343	65.03302	62.42053	56.39655	62.70623	53.43095	56.26231	64.26766
ZFP37	0.730218	1.182156	0.959014	1.103172	0.986298	1.197552	0.962758	0.946763
ZFP42	0.058159	1.252105	0	1.057899	0	0.541649	0.413222	0.339177
ZFP57	4.113299	5.242709	2.358608	3.935241	3.083421	2.860473	3.35109	3.752294
ZFP62	4.433066	5.535429	4.764109	5.382734	4.415011	4.844674	5.098244	5.333697
ZFP64	6.641497	5.113781	4.303784	4.971627	5.763034	5.343126	5.412156	5.363173
ZFP69	2.863014	3.629343	2.700743	2.977722	4.045776	3.520754	3.310769	3.741675
ZFP69B	0.046513	0.082466	0.03445	0.202577	0.081408	0.105369	0.059013	0.271257
ZFP90	3.145987	2.833134	2.214553	2.603404	2.836913	2.798229	2.42348	2.758749
ZFP91	21.79574	22.86844	19.68838	24.24062	22.28277	21.77449	21.94187	20.37812
ZFP92	0	0	0	0	0	0.02258	0	0
ZFPL1	18.71875	19.66747	17.78264	21.46171	20.18043	18.84663	17.39216	17.05422
ZFPM1	2.268403	2.381575	2.21304	2.774513	2.003405	1.961575	1.986773	1.911061
ZFPM2	0.465658	0.629026	0.619273	0.814409	0.801036	0.662626	0.781437	0.963466
ZFR	24.50625	21.96125	20.18715	18.13464	23.06452	20.83501	20.42601	23.55399
ZFR2	0.116168	0.136082	0.053775	0.167407	0.032676	0.069446	0.081063	0.106776
ZFX	9.633355	10.03902	8.982222	8.855845	10.95238	9.665323	9.276339	10.30231
ZFYVE1	8.099497	7.161881	7.258538	7.12902	7.582159	7.38776	7.770959	8.322471
ZFYVE16	8.997483	8.95118	7.324996	8.388978	9.226607	9.066729	8.446237	9.584001
ZFYVE19	8.209936	6.209209	5.340361	6.028692	6.260681	7.903044	7.285212	6.415467
ZFYVE21	10.09878	10.67702	10.04053	10.46915	12.02624	10.96505	10.1808	10.87712
ZFYVE26	7.814405	7.06841	6.472174	6.5659	6.766215	6.328872	6.5033	7.370767
ZFYVE27	11.16132	10.35557	8.933825	11.30813	9.951583	12.22939	11.13611	10.8894
ZFYVE28	0.008191	0.016597	0.024267	0	0.008192	0.016494	0.016628	0.033231
ZFYVE9	9.690493	10.05227	8.77434	9.636483	10.58792	9.989841	9.84096	10.47176
ZG16	0	0	0	0	0	0	0	0
ZG16B	0.356802	1.04429	1.60518	0.568774	0.594748	16.1658	1.770533	2.774426
ZGLP1	0.025584	0	0	0	0	0	0	0
ZGPAT	19.11322	18.90433	16.68821	20.14469	18.18986	20.24893	19.6947	18.13346
ZGRF1	0.902232	0.938341	0.851566	0.80594	0.806524	0.759676	0.692902	0.805753
ZHX1	10.57586	11.76511	11.61023	11.88855	11.64302	11.38466	11.85121	11.84229
ZHX2	5.617007	5.005095	4.767317	5.548089	5.392122	4.894516	5.386434	5.731288
ZHX3	5.467355	5.871197	4.333841	5.557126	5.082942	5.200312	5.891759	6.800761
ZIC1	1.584909	1.7878	0.839055	0.803712	1.241945	1.11866	1.044814	1.24289
ZIC2	0.063015	0.042561	0.020743	0.010763	0.021008	0.021148	0.01066	0.010652
ZIC3	0.00723	0	0	0	0.007231	0	0	0
ZIC4	0.070025	0.06385	0.041492	0.014352	0.035017	0.049353	0.035538	0.035511
ZIC5	0.004236	0	0	0	0	0	0	0
ZIM2	0	0.015966	0.031127	0	0.015762	0	0	0.015984
ZIM3	0	0	0	0	0	0	0	0
ZKSCAN2	1.945408	1.728611	1.515676	1.376726	1.231985	1.344598	1.335276	1.382789
ZKSCAN4	0.746143	0.749983	0.545382	0.848914	0.804998	0.763071	0.852747	0.619713
ZKSCAN5	5.430066	4.985757	4.224759	5.100379	5.453139	5.3264	5.148245	5.15004
ZKSCAN8	7.691018	7.879684	5.799395	7.929929	6.495302	6.33672	7.155185	8.415283
ZMAT1	0.106965	0.12643	0.114435	0.164422	0.115894	0.071797	0.054285	0.027122
ZMAT2	45.04743	52.39245	54.09491	51.4734	48.05112	51.66719	48.74639	41.21957
ZMAT3	3.916822	4.301436	4.077986	4.00363	4.608426	3.806441	3.42594	4.639731
ZMAT4	0.026669	0.01351	0	0.006832	0.026672	0.107405	0.047371	0.033811
ZMAT5	3.49153	2.768363	3.785331	4.297508	4.232129	3.324329	3.563163	4.195592
ZMIZ1	17.50938	14.94048	14.16543	16.9813	15.5165	15.25704	14.91415	16.06679
ZMIZ2	12.35642	12.29973	9.471838	12.67725	12.13477	11.39685	11.77435	9.757099
ZMPSTE24	6.836192	8.165383	8.014521	8.268819	8.537005	9.195998	8.654461	7.54919
ZMYM1	6.889275	6.229289	6.04385	6.32036	6.9424	6.152356	6.819124	7.064397
ZMYM2	18.62924	18.21401	16.4959	17.43114	16.9044	17.30844	18.04658	17.91813

ZMYM3	4.068351	4.020765	3.503147	4.730889	4.182153	4.109828	3.789093	4.264115
ZMYM4	4.457745	4.481597	3.54653	4.026839	4.671165	3.749731	4.5979	4.540476
ZMYM5	14.02738	12.74001	13.24186	14.38444	13.60747	14.50058	12.20246	12.59243
ZMYM6	6.685773	7.523282	7.533184	7.168996	7.42046	6.776027	6.490735	7.493046
ZMYND10	0.968797	1.134873	0.881982	1.101382	0.802389	1.508846	0.752862	0.690883
ZMYND11	31.47309	34.48627	30.76871	32.79267	30.73936	32.23735	32.05844	31.63819
ZMYND12	0.332462	0.105258	0.18468	0.340697	0.207816	0	0.253087	0.126448
ZMYND15	0.152654	0.131459	0.082914	0.156436	0.14504	0.046109	0.069725	0.139345
ZMYND19	11.31001	12.03766	9.794428	9.577804	12.20644	11.26214	12.66547	11.09285
ZMYND8	3.551487	3.140734	2.888766	3.90687	4.133933	3.441757	3.326655	3.18388
ZNF10	2.421572	2.628288	2.075319	2.47724	2.737783	2.773091	2.838335	2.584186
ZNF106	11.29534	11.37353	9.900025	10.1906	11.47831	10.28195	10.87998	12.12521
ZNF112	4.503422	4.225645	3.355112	3.829761	3.246064	3.719052	4.138088	3.885701
ZNF114	0.813439	1.311618	0.780675	1.056661	1.19167	0.888207	1.314047	1.870817
ZNF12	6.284111	7.045411	5.75466	6.247305	6.38138	5.7342	5.986808	6.275752
ZNF131	10.41385	10.17824	9.697308	10.55162	11.40041	11.02026	11.05412	9.792932
ZNF132	3.787393	3.572472	3.441953	3.354234	3.347052	3.246208	2.758879	3.377703
ZNF133	3.524378	3.312012	2.878702	4.075796	3.598954	3.415803	3.217848	3.131907
ZNF142	3.982973	3.038509	2.503816	3.304391	3.766759	2.849456	2.973751	3.789117
ZNF143	16.87643	14.95542	15.23294	14.91488	15.53081	15.73926	15.53025	16.70663
ZNF146	24.01378	26.96204	24.31445	27.97328	25.39345	26.09781	25.95834	25.8374
ZNF148	8.24232	9.044823	7.658341	8.156196	7.975308	8.037954	8.62256	8.718014
ZNF157	0.039004	0.079031	0.061629	0.015988	0.054612	0.054978	0.031671	0.063295
ZNF16	2.592129	3.322519	2.237694	2.727968	2.732282	2.65089	2.601449	2.510871
ZNF165	1.998027	1.530944	1.77416	1.187217	1.37697	1.386197	1.209981	1.600741
ZNF169	0.439355	0.399468	0.428318	0.415604	0.456312	0.317589	0.320166	0.348489
ZNF174	6.021198	6.15741	4.645448	5.63044	5.946702	6.422283	5.462168	5.286297
ZNF175	6.550504	7.740147	5.322766	6.253084	5.782279	4.057515	6.251281	9.958411
ZNF18	2.63758	3.388454	2.738979	2.767932	2.683246	2.619096	2.493148	2.546427
ZNF180	3.384918	4.004203	3.584269	3.679296	3.688668	3.595189	4.021547	3.482716
ZNF181	2.166618	2.660941	2.458381	3.126525	2.494144	2.488599	2.176677	2.121223
ZNF182	0.925967	1.031266	0.71987	1.07677	1.004903	1.057921	0.633237	1.065701
ZNF185	31.89329	26.30125	28.1507	27.63323	28.9498	28.14184	26.14599	28.1935
ZNF189	4.173958	4.19749	3.720923	3.903344	4.354433	4.813177	4.768747	4.879853
ZNF2	2.972519	2.415238	2.412858	2.87889	3.012477	2.694042	2.193803	2.738935
ZNF200	2.805398	3.414411	2.404707	2.827448	3.666934	3.085578	2.509164	3.399376
ZNF202	3.989441	4.00186	3.620505	4.702308	3.911104	4.326283	4.033278	4.55801
ZNF205	16.78414	17.14661	15.47771	17.73926	13.96715	18.15885	14.21044	13.52356
ZNF207	23.73568	22.24793	18.75926	20.74069	21.8491	19.81811	21.29265	21.65941
ZNF212	4.826811	4.965062	4.5372	5.552492	4.964758	5.859391	5.821165	5.002646
ZNF213	1.833313	1.920487	1.520443	1.687204	1.71784	2.249054	1.689184	1.516413
ZNF214	1.223041	0.775547	0.755962	0.676261	0.976799	0.885897	1.134217	0.812094
ZNF215	4.080358	2.442186	3.107894	3.696813	3.289822	2.886297	3.304755	2.729251
ZNF217	6.784163	5.967871	5.573728	6.536444	6.086504	5.563685	5.786054	7.766225
ZNF219	2.509148	2.461144	2.251969	2.887596	2.647475	2.515321	2.362503	2.537502
ZNF22	10.15838	11.24121	11.49854	11.48438	10.96993	11.65119	11.72153	11.34215
ZNF23	1.677094	1.775297	1.554696	1.392378	1.564487	1.569923	1.450348	1.736564
ZNF232	0	0	0	0	0	0	0	0
ZNF235	2.026713	1.780403	1.836783	1.866595	2.039804	1.950154	2.044094	2.127119
ZNF236	5.257614	3.94371	3.295445	3.718423	4.239564	3.242418	4.055445	4.761994
ZNF239	2.745214	2.306198	2.054034	1.737211	2.917919	2.195035	2.082676	2.690807
ZNF24	19.19735	20.33451	17.86409	19.16306	18.99077	19.00018	18.33113	19.15687
ZNF248	4.769491	5.096404	3.937129	4.277723	4.49287	4.317762	3.732147	4.605854
ZNF25	3.83179	3.805659	3.520851	3.993696	4.19942	3.837715	3.537089	3.66703
ZNF250	1.871036	1.908486	1.395219	1.845643	1.750345	1.396844	1.899101	2.130039
ZNF260	7.677751	8.406895	8.212438	8.812083	8.509854	8.809398	8.544704	8.361152
ZNF263	3.900802	4.376743	3.465177	3.978746	4.676098	4.166034	3.53378	4.390789
ZNF268	1.710388	1.993037	1.451634	1.528761	1.442814	1.870622	1.580743	1.961971

ZNF274	1.619917	1.793339	1.303094	1.605075	2.263885	1.911815	1.644216	1.523291
ZNF275	3.308937	3.244083	2.233356	2.742512	3.14689	2.466375	2.902951	3.186939
ZNF276	9.665323	9.904724	7.912934	9.235882	11.14572	8.242287	8.520586	9.556434
ZNF277	12.86844	14.2808	16.65672	18.27016	18.91917	14.06471	19.57293	14.2708
ZNF280B	1.149298	1.082116	0.857402	1.12656	1.224411	0.767241	0.938302	1.152989
ZNF280C	1.789168	2.104339	1.997042	2.486797	2.255603	2.325933	2.059531	2.336085
ZNF280D	4.94336	4.394136	4.39642	4.423664	4.583542	4.52354	4.098811	4.893269
ZNF281	5.370462	5.644904	5.087193	5.212236	4.583544	4.72753	5.643337	5.651098
ZNF282	7.499678	6.227517	5.149341	6.931708	6.791304	6.03015	6.265711	6.514097
ZNF286A	6.992978	6.333164	5.139629	6.240956	6.148404	5.384785	5.901274	6.264288
ZNF287	4.204721	4.484956	3.259187	4.384753	3.887886	3.759513	3.881275	4.071465
ZNF292	4.315184	4.701511	3.464523	4.259396	4.384539	3.689367	3.896537	4.830106
ZNF296	3.663594	3.113585	3.069252	3.416274	3.785624	2.884468	2.872622	3.469205
ZNF3	13.05963	13.59187	11.0503	14.78227	12.69521	14.06528	13.65721	14.45972
ZNF300	0.476956	0.55393	0.631847	0.417243	0.53519	0.667615	0.649416	0.471947
ZNF304	5.13548	5.81069	4.455328	5.533199	5.152144	4.357125	5.650948	5.80084
ZNF311	0.931989	1.404379	0.914551	1.190821	1.126791	1.395656	1.38303	1.196527
ZNF316	3.129682	2.895186	2.802178	3.303559	3.234199	2.877204	3.060742	3.395614
ZNF317	5.267308	5.094194	4.687759	4.749182	5.260955	4.545678	5.046524	4.743156
ZNF318	4.134923	3.246354	3.101714	3.793021	3.955655	3.272331	3.273833	3.63962
ZNF319	0.782888	0.74103	0.882025	0.862546	0.838129	0.658714	0.858053	0.835039
ZNF32	16.05871	18.71088	14.70774	15.42416	16.45588	16.42695	15.95876	14.00348
ZNF322	10.38599	10.26853	9.52602	8.536093	10.67279	10.13438	10.09244	10.12029
ZNF326	24.33311	23.84991	21.98396	21.23085	22.68065	22.28049	22.98696	24.82371
ZNF329	9.047073	8.328034	8.692128	7.066486	8.578659	8.02936	8.862698	8.358495
ZNF330	11.30984	12.95895	11.59557	13.15535	10.50887	11.48032	11.79271	9.561648
ZNF331	3.889923	4.357641	4.309648	4.038959	4.530261	3.657697	3.56562	4.124893
ZNF333	15.37363	11.5907	10.2476	10.07625	13.18617	10.3108	13.0935	15.881
ZNF335	11.26836	9.553571	9.593248	10.55955	10.71134	9.101736	10.37332	11.03873
ZNF34	6.821178	7.067364	6.617411	9.155852	8.196782	6.908142	8.574438	8.289158
ZNF341	2.395469	1.928144	1.760876	2.048748	2.233658	1.988705	1.949996	1.765853
ZNF346	3.471984	4.237867	2.539361	4.359443	3.211704	3.173573	3.439871	3.653613
ZNF354A	4.977641	5.532576	5.703073	5.56316	5.461613	5.765826	5.019605	5.734705
ZNF354C	2.021196	1.887465	1.831123	1.67502	1.863256	1.822655	1.65905	1.720191
ZNF358	6.691175	7.863237	7.293337	8.33896	7.926306	8.396839	8.875978	7.373151
ZNF362	4.581403	4.966392	3.93004	5.65817	3.94939	4.391574	4.641207	4.86492
ZNF365	2.616961	2.981802	3.157064	2.800645	3.081309	2.094734	2.509057	2.66892
ZNF366	0.979459	1.176318	0.730245	1.223076	1.336388	1.14942	1.099491	1.276291
ZNF367	2.984609	3.396991	2.954844	2.919864	2.496267	2.891459	3.098054	3.354968
ZNF382	1.121465	1.232368	1.078193	0.608066	0.890167	0.758725	1.397263	1.654991
ZNF384	9.45303	9.190779	8.284256	9.150628	9.152581	8.40598	8.676356	9.95702
ZNF385A	31.48021	30.29209	34.35541	28.53118	30.87631	29.48316	27.58044	27.0312
ZNF385B	0.045071	0.117418	0.146246	0.105572	0.122352	0.045379	0.176453	0.124077
ZNF385C	0.189266	0.214309	0.14293	0.3765	0.289504	0.235397	0.169505	0.191961
ZNF385D	0.121776	0.344148	0.348116	0.190472	0.525626	0.245215	0.208172	0.383528
ZNF391	0.442711	0.498357	0.456625	0.76621	0.403411	0.406114	0.619107	0.668531
ZNF394	1.772574	1.806923	1.491156	2.264995	1.674314	1.344021	1.810269	1.78671
ZNF395	13.38744	13.54263	13.15068	12.6769	11.76486	11.52787	13.3777	14.28617
ZNF397	4.186767	5.278223	4.367753	5.282445	5.064911	4.354032	4.365393	5.044557
ZNF398	4.721799	4.767286	3.759289	4.663714	4.563775	4.1685	4.128015	4.409525
ZNF404	0.214347	0.354313	0.490197	0.289021	0.417466	0.283962	0.320618	0.34326
ZNF407	3.203038	2.718129	2.169107	2.538475	2.922776	2.524178	2.772536	3.153757
ZNF408	2.719111	2.309382	2.052856	2.840333	3.211738	2.699196	2.750197	2.563948
ZNF41	1.246858	1.376346	0.918897	1.125177	1.209794	1.264744	0.972782	1.123047
ZNF410	18.03184	16.67763	15.52155	16.38126	17.93741	17.36828	17.90953	16.87708
ZNF414	7.360007	9.676057	6.841311	9.324509	7.918315	8.513123	7.626447	6.919053
ZNF423	2.147936	2.224754	1.87232	2.60079	2.442243	2.283426	2.758688	2.215025
ZNF428	5.86545	6.217635	6.140107	6.08291	6.168072	6.037204	6.014702	4.959158

ZNF436	15.40616	15.3076	13.53902	15.2104	15.60204	15.548	13.8786	14.69765
ZNF438	2.149661	1.361171	1.240735	1.570317	1.547084	1.455084	1.164666	2.408601
ZNF444	2.150649	1.898733	2.336237	2.597479	2.258474	2.397342	2.635083	2.804484
ZNF445	8.963211	9.551183	6.547155	8.772085	7.734366	7.983004	8.696718	9.739267
ZNF446	0.88612	0.758443	0.761922	0.821959	0.687596	0.684512	0.837383	0.736032
ZNF449	4.171209	4.693529	3.871158	4.190717	4.323661	4.046775	3.427344	4.011357
ZNF451	2.505922	2.288802	1.777938	1.894831	2.207304	2.012134	1.975542	2.506344
ZNF461	0.68137	1.15052	0.314011	0.449982	0.696602	0.861342	0.791487	1.259283
ZNF462	1.443975	1.334067	1.086111	1.344291	1.464115	1.398598	1.336537	1.560648
ZNF467	0.409797	0.155691	0.515981	0.65092	0.655761	0.639525	0.384749	0.249379
ZNF469	0.623521	0.708474	0.545197	0.703306	0.53699	0.499173	0.551567	0.584089
ZNF470	5.001663	2.785817	2.405295	3.734854	5.381632	4.391437	3.891727	6.196829
ZNF473	3.665125	3.828284	3.155555	3.665981	4.05281	3.445885	4.134029	4.20945
ZNF48	5.059266	4.59863	3.656513	4.756021	4.815085	4.066847	4.32761	4.241589
ZNF483	0.037397	0.102839	0.036931	0.060223	0.042745	0.043032	0.092184	0.124626
ZNF484	2.214908	2.594597	2.212942	2.021515	2.682461	2.508794	2.213004	2.755397
ZNF488	0.036635	0.111347	0.066327	0.050057	0.079386	0.04918	0.086764	0.092891
ZNF496	5.367248	5.732537	5.15667	5.127399	5.144045	5.296851	5.351186	5.9375
ZNF500	1.277474	0.720103	0.701919	0.984299	0.816536	0.928386	0.68244	0.993664
ZNF502	1.106015	1.544515	0.993835	1.419317	1.026435	1.163732	0.960789	1.071231
ZNF503	10.04566	10.35315	10.47216	10.10697	9.594223	10.89008	9.756429	10.82363
ZNF507	4.798142	4.619209	3.735292	4.260618	4.654724	4.201299	4.085357	4.501271
ZNF511	5.801976	5.71484	4.748209	7.40459	6.648957	6.693515	5.98443	5.39419
ZNF512	15.98573	16.28555	13.3376	14.62682	14.54171	13.2159	16.09434	16.23788
ZNF512B	7.404716	7.482697	5.010644	8.296942	6.552813	6.43455	6.217475	6.755751
ZNF513	7.35362	6.836739	5.418468	6.695591	6.799509	6.311692	7.028635	6.550002
ZNF514	0.695743	0.440546	0.114512	0.371342	0.507378	0.59834	0.544346	0.367525
ZNF516	3.165746	3.388899	2.528858	3.365532	3.399855	3.100357	2.979306	3.528972
ZNF518A	4.914586	4.939616	4.459754	4.891988	4.546579	4.208953	4.577682	5.252011
ZNF518B	5.404057	4.511676	3.874769	4.939724	4.899188	4.253413	5.344631	5.706819
ZNF521	3.25827	4.035236	3.020441	3.707377	4.902304	3.780364	3.909496	3.183116
ZNF524	7.293934	7.584125	6.410371	7.20638	6.579351	7.115369	6.287529	5.645665
ZNF526	4.171087	3.574182	3.195201	4.144576	4.132639	3.463674	4.461163	3.904285
ZNF527	2.524137	3.243517	2.606277	2.881344	2.492508	3.017487	2.386875	2.449889
ZNF529	0.095244	0.06433	0.068976	0.461991	0.095257	0.06393	0.032224	0.02576
ZNF532	11.26041	10.34581	8.579857	10.05004	9.910445	9.700511	9.854216	10.62483
ZNF536	0.071978	0.048615	0.050772	0.084298	0.099411	0.093175	0.0661	0.06605
ZNF541	0.018386	0.024836	0.012104	0.043963	0.006129	0.043193	0.012441	0.018647
ZNF544	0.085856	0.125642	0.075366	0.019552	0.104949	0.038419	0.154923	0.106429
ZNF550	1.282043	0.938786	0.902544	1.014618	1.485331	1.099098	0.76015	1.44191
ZNF554	0.724452	0.500957	0.601866	0.907375	0.483031	0.555735	0.245106	0.51317
ZNF557	3.511974	4.395257	3.323431	3.08678	3.132526	3.260872	3.537597	4.122962
ZNF565	1.422935	1.539653	1.143278	1.256166	1.616692	1.288308	1.404261	1.577695
ZNF567	3.550811	3.855394	3.05253	3.551784	3.735201	3.247473	3.568176	3.206788
ZNF568	3.429412	2.544321	2.457592	2.783498	4.65914	2.299347	2.633743	3.162729
ZNF569	1.636525	2.091872	1.132804	1.75543	1.422584	1.971086	1.30402	1.566748
ZNF570	2.020193	2.746434	2.233741	2.707228	2.482395	2.212185	2.168793	2.372931
ZNF572	1.117362	1.314775	1.202403	1.170713	1.493349	1.089681	1.062922	1.067203
ZNF574	6.325336	5.65496	4.565446	5.986225	6.335114	4.681695	5.410805	5.134122
ZNF575	6.803471	6.267603	6.499951	6.307224	6.519517	6.069374	5.460181	5.504211
ZNF576	15.53514	15.42968	11.92421	14.25116	10.29024	15.79033	14.30311	13.39144
ZNF577	1.305926	0.917802	0.77844	0.976578	0.823663	0.876565	0.955326	1.014269
ZNF579	2.010585	2.854063	2.501547	2.782106	2.72657	2.321679	2.605696	2.327231
ZNF580	2.731434	2.799837	2.554596	3.227189	3.647739	3.235405	3.229037	2.998461
ZNF581	2.885643	3.402779	2.647251	3.441896	2.901787	2.619584	3.585136	3.182611
ZNF582	3.340554	3.662946	3.95057	3.888193	3.175998	3.474103	3.516242	3.067423
ZNF583	11.42355	12.08259	12.06022	12.5628	13.47043	13.91957	10.21894	10.5372
ZNF584	0.89901	1.14357	0.917401	1.300026	0.839184	0.65372	1.226798	1.063775

ZNF592	3.987368	3.615906	2.968255	3.844149	4.078977	3.525519	3.83489	4.259681
ZNF593	15.7333	15.682	13.31765	14.99311	13.14813	16.48125	18.85325	14.925
ZNF596	3.491868	3.246549	4.144456	3.806178	3.839382	3.543019	3.544246	3.503081
ZNF597	0.044908	0	0	0	0.011229	0.011304	0.011396	0
ZNF598	7.660991	7.787149	6.616721	8.403505	7.543975	7.704842	7.399514	7.778598
ZNF599	0.454609	0.373439	0.606682	0.579189	0.552974	0.865947	0.72332	0.797545
ZNF605	4.631192	3.848883	3.41225	3.556341	3.657788	3.330159	3.48265	3.981543
ZNF606	3.450621	3.1319	2.901752	2.653894	3.362122	3.030071	2.801905	3.348208
ZNF608	7.222532	6.596106	6.048744	6.736523	6.066524	6.249032	6.657243	7.223817
ZNF609	10.44809	9.803442	8.708499	10.12173	10.87236	8.794713	9.934379	10.28691
ZNF613	3.542235	3.371789	2.881853	2.495218	2.679965	2.192833	2.912311	2.990782
ZNF614	3.556916	4.185866	3.449534	3.200029	3.748976	3.330466	3.791756	4.300564
ZNF618	6.593003	6.940809	6.325086	7.029925	7.049023	7.38952	5.885052	7.000738
ZNF619	4.550514	3.716559	3.705668	3.831025	4.551103	3.848545	4.149789	3.578628
ZNF622	17.57221	19.37723	17.48817	17.35998	16.6522	18.82703	17.3158	16.71388
ZNF623	6.621234	6.421971	5.429483	6.966992	6.411865	5.886067	7.06058	6.622221
ZNF624	3.075318	3.440626	2.692309	3.180496	3.330452	3.314779	2.738448	3.099961
ZNF628	1.344849	1.610226	1.086621	1.236169	1.304265	1.091436	1.505661	1.074662
ZNF629	2.737226	3.000001	1.931445	2.683274	2.682737	1.877158	2.356208	2.451762
ZNF638	30.53792	31.05153	28.24111	26.97925	30.79035	28.92219	29.45249	34.32233
ZNF639	5.635571	6.155589	4.844837	6.574408	6.189865	5.281445	6.230828	6.851301
ZNF641	0.870029	0.848801	0.668258	0.647221	0.476967	0.558027	0.745712	0.568667
ZNF644	7.094372	7.665635	6.37765	7.052764	7.767474	6.733816	7.031001	7.937624
ZNF646	4.201272	4.264797	2.983955	3.758158	3.920314	3.429821	3.99541	3.996599
ZNF648	0.046571	0.018873	0.009198	0.01909	0	0	0	0.009447
ZNF652	2.760074	2.879543	2.613188	3.425515	2.85755	2.859778	2.742716	3.017178
ZNF653	4.024909	3.852581	2.840854	3.580564	4.1983	3.393578	3.320858	4.157338
ZNF654	5.154209	5.558937	4.623068	4.390601	5.154875	5.524411	4.402062	5.132858
ZNF655	6.856911	7.404358	6.316992	6.30599	6.742005	6.676446	5.949145	7.286251
ZNF662	0.144336	0.219345	0.083147	0.345126	0.024059	0.169542	0.061042	0.073195
ZNF664	120.2847	118.9728	118.0164	107.0347	115.0425	122.9949	110.2709	112.539
ZNF667	0.776655	1.393845	1.037246	1.190049	1.065264	1.511791	0.593105	0.810217
ZNF668	2.244414	2.140113	1.727526	2.085793	1.793563	2.137898	2.054739	2.142461
ZNF672	1.01566	1.086368	0.794201	1.071772	0.864744	0.855333	1.007901	0.876941
ZNF674	1.191313	0.800111	1.678782	0.288059	1.070982	0.080862	0.135863	0.638076
ZNF683	0.28785	0.566101	0.568526	0.138814	0.541905	0.306864	0.429658	0.257601
ZNF684	0.317431	0.67116	0.272588	0.325294	0.621142	0.500243	0.378226	0.51792
ZNF687	5.166029	4.742097	4.357897	5.208237	4.949093	4.560985	4.994367	4.888757
ZNF689	3.976197	3.878068	3.494428	3.633771	3.375753	3.29381	3.275355	3.716798
ZNF691	2.197446	2.574143	2.497171	2.479549	2.702724	2.411742	2.66091	2.581064
ZNF692	10.71406	11.01967	9.581091	10.87216	10.98304	10.07279	8.737598	11.19694
ZNF696	2.818747	2.726586	2.252078	3.265969	2.578283	2.538515	2.889782	3.390423
ZNF697	1.697151	1.669017	1.359365	1.201004	1.879823	1.469409	1.413997	1.429753
ZNF7	7.318454	6.389781	6.2112	6.248786	6.674084	6.666136	5.876504	6.886132
ZNF70	2.929529	2.678679	2.360376	4.10214	2.813558	3.508571	3.182796	2.627986
ZNF703	4.100378	4.668022	3.56541	5.391176	4.960637	4.812128	4.816268	4.071564
ZNF704	8.626678	8.675023	7.432733	8.233906	7.477032	7.433367	8.177066	8.310632
ZNF706	38.81552	34.82267	44.46831	35.43498	38.9125	37.42983	34.9727	35.18727
ZNF71	1.151489	0.531323	0.607977	0.724364	1.299869	0.83795	0.358729	0.404711
ZNF710	11.09924	9.486443	10.87073	10.34961	9.989084	11.55319	9.040399	10.2741
ZNF711	1.863114	1.782435	1.555763	1.9576	1.934115	1.875842	1.953298	2.181454
ZNF74	10.05718	8.549668	7.168951	7.993953	8.240546	7.670431	7.848334	9.131193
ZNF740	11.24859	10.10945	10.45226	10.94272	10.64431	10.9347	11.11293	11.7904
ZNF746	5.627666	5.990035	5.182578	6.010255	5.764018	5.857258	5.753373	5.570243
ZNF750	20.11333	17.46491	19.48257	19.51718	18.26858	20.30075	18.80174	19.82032
ZNF75D	63.35491	64.4779	75.8174	62.03812	54.8083	63.64726	63.13702	50.70858
ZNF76	6.525724	6.03227	4.97678	7.4879	7.098238	5.659095	6.62361	6.647609
ZNF768	23.0844	20.13761	22.27905	22.15379	21.90546	23.41208	20.54467	21.48446

ZNF770	1.951559	2.522597	2.076769	2.080355	1.99668	2.269788	1.792995	1.871273
ZNF771	7.774768	10.0403	9.704857	10.30444	12.46197	9.268212	9.448624	8.579366
ZNF774	1.931813	1.479079	0.975715	1.344963	1.85832	1.410503	1.242333	1.615312
ZNF775	1.975359	2.668378	1.407254	2.386848	2.083732	2.275803	1.985039	1.694485
ZNF777	6.458344	6.223259	5.228786	5.999008	5.990077	5.948484	6.143229	6.211791
ZNF782	2.579363	2.561365	2.769601	2.695701	2.876567	2.808247	2.37391	3.010571
ZNF783	4.571992	4.70437	4.261054	3.60178	4.872659	3.222258	3.523934	4.492169
ZNF784	3.35445	3.306207	3.237706	4.355255	3.081635	3.652445	3.004206	2.617079
ZNF786	3.290987	4.111798	3.779531	3.631101	2.63944	3.81102	3.585809	3.433813
ZNF787	1.419045	1.251379	1.010561	1.35998	1.56315	1.299952	1.245582	1.447354
ZNF789	3.260874	3.588807	2.779376	3.341646	3.115702	2.931384	3.102925	3.474628
ZNF79	12.35546	12.70286	11.25995	12.27336	10.94631	12.34782	13.68086	13.19368
ZNF790	0.61423	0.653021	0.569133	0.683841	0.72807	0.648966	0.492597	0.44608
ZNF792	4.446366	5.55619	3.743647	4.657262	3.955264	4.190758	4.790277	5.107997
ZNF793	1.493735	1.190824	1.321968	1.212878	1.306167	0.912226	1.118465	1.208688
ZNF8	4.460613	4.087998	3.494452	3.828098	4.350842	3.915814	4.211556	4.679979
ZNF800	6.604736	6.14457	7.196006	6.723081	6.531976	6.778309	6.594235	6.89783
ZNF804A	0.12255	0.172443	0.154641	0.083724	0.245133	0.25363	0.14512	0.145011
ZNF804B	0	0	0	0	0	0	0	0
ZNF81	0.789108	0.73964	0.763372	0.533602	0.821422	0.670189	0.670178	0.931009
ZNF821	1.205004	1.115044	0.713135	1.185815	1.03113	1.081839	1.196587	1.844268
ZNF827	2.166997	2.486442	1.841976	2.414424	2.025459	2.247694	2.420928	2.12778
ZNF830	9.74059	10.7974	8.446507	9.750806	10.55367	10.77574	11.12254	9.924991
ZNF831	0.010418	0.015833	0.005144	0.026691	0.041679	0.005245	0.037012	0.116234
ZNF839	3.86934	4.082267	3.635184	5.286913	3.672111	3.876816	4.108937	4.039004
ZNF852	3.904772	4.424096	3.878195	4.665864	4.821973	4.066497	4.137307	5.026028
ZNF853	1.226901	1.243002	1.047459	1.411411	1.282475	1.171526	1.534536	1.05169
ZNF862	1.75235	1.565696	1.313104	1.601742	1.664507	1.387519	1.608816	1.929125
ZNF865	3.019765	3.067007	2.603807	3.887452	2.884924	3.221911	3.026939	2.971328
ZNF875	0.089365	0.07243	0.026475	0.009158	0.196629	0	0	0.018127
ZNF879	1.080362	1.15096	1.044902	1.689218	1.592904	1.356874	1.560065	1.547593
ZNF891	0.120194	0.105535	0.087044	0.041057	0.096168	0.056474	0.154529	0.097524
ZNFX1	6.742685	7.380674	7.379255	7.301573	7.094783	6.98911	7.913172	7.574778
ZNHIT1	46.38113	50.59943	58.29211	52.16788	45.80929	52.48271	44.27504	44.07892
ZNHIT2	3.196686	3.753877	2.672564	4.541546	4.014539	3.602553	4.332327	3.44483
ZNHIT3	12.07053	13.46184	11.69277	10.6843	12.07209	13.90806	11.91776	11.37506
ZNHIT6	7.001333	9.550454	9.97993	8.765468	8.215097	8.042233	9.551727	10.43011
ZNRD1	16.27739	18.663	25.36645	15.78553	13.6986	16.06881	20.22882	16.46884
ZNRF1	11.99536	14.47991	12.32597	14.53428	13.30964	14.63471	12.92776	12.35101
ZNRF2	5.510482	5.442197	5.103218	4.994438	5.392805	5.042631	4.760383	4.843737
ZNRF3	3.666994	3.643069	3.673976	3.926359	3.756376	3.602541	3.514469	3.579444
ZNRF4	0	0	0	0	0	0.035649	0	0
ZP2	0.012751	0.038756	0	0.052269	0.076518	0.038515	0.064713	0.012933
ZP3	0.021714	0.021999	0.042887	0.066755	0.021717	0.065587	0	0.022023
ZPBP	0.220002	0.123827	0.531082	0.250502	0.611196	0.393787	0.148868	0.049585
ZPBP2	4.367373	4.655544	4.988026	5.117699	4.557848	4.703102	5.049633	4.35251
ZPR1	32.33613	36.25077	35.52516	34.31896	35.99283	35.32595	35.05728	35.12086
ZRANB1	24.93788	22.8461	26.03978	23.67167	23.16775	24.52229	23.53983	23.30145
ZRANB2	33.88519	37.67461	32.53557	35.23052	33.27956	35.06219	30.20729	30.89153
ZRANB3	2.543268	3.07859	2.518102	2.606266	2.873934	2.820033	2.842912	3.182467
ZRSR2	3.69895	5.425073	4.655089	4.584268	4.834631	5.230041	4.350806	5.173696
ZSCAN10	0.035564	0	0.011707	0	0.011856	0.023872	0	0.012024
ZSCAN12	0.544434	0.578161	0.855059	0.746134	0.400178	0.250962	0.512654	0.51892
ZSCAN16	0.994404	0.891038	0.868537	1.077914	1.091776	0.76981	0.977919	0.93684
ZSCAN2	1.069001	1.194772	1.06406	1.147646	1.170962	0.939631	1.136705	1.290737
ZSCAN20	1.609227	1.447773	1.086509	1.389899	1.729589	1.266891	1.488962	1.632135
ZSCAN22	0	0	0	0	0	0	0.026675	0.013328
ZSCAN23	0.220236	0.155219	0.189124	0.264942	0.162804	0.053025	0.199242	0.21366

ZSCAN25	4.544902	4.06941	3.525271	3.937742	4.725451	3.594137	3.434767	4.95694
ZSCAN26	2.616163	3.090652	1.884044	2.497073	2.994335	1.73542	2.156918	2.734828
ZSCAN29	7.495351	7.645875	7.122351	6.615378	6.497497	6.623969	6.824013	7.596831
ZSCAN31	0.169631	0.081406	0.299768	0.347666	0.20537	0.188768	0.14499	0.153936
ZSCAN9	0	0	0.023444	0	0	0	0	0
ZSWIM1	0.664368	0.423393	0.444448	0.505128	0.375095	0.442341	0.413301	0.315176
ZSWIM2	0	0	0	0	0	0.012786	0	0.01288
ZSWIM3	1.201122	1.043044	0.887125	0.868852	1.322414	1.097541	1.260118	0.962292
ZSWIM4	4.36904	3.25691	2.800814	2.842808	3.215136	3.192943	3.634589	3.323427
ZSWIM5	0.043363	0.107835	0.070074	0.113114	0.130107	0.119072	0.076024	0.095958
ZSWIM6	2.899458	2.870632	2.612601	2.48734	3.229936	2.280197	2.659478	3.14674
ZSWIM7	10.69249	12.08853	10.24869	9.497631	9.8058	10.02052	9.782611	9.606382
ZSWIM8	9.476089	8.538519	7.329746	8.264111	9.198708	7.91028	8.707685	9.266198
ZSWIM9	0.662214	0.541648	0.599966	0.616359	0.565081	0.538284	0.573483	0.646993
ZUP1	7.788007	8.109694	6.792439	8.058618	7.854012	7.274113	7.662955	7.580282
ZW10	17.56202	16.81675	16.32415	18.66173	16.31602	21.23423	18.31423	15.86835
ZWILCH	6.340447	7.809585	7.451547	6.408495	7.285941	7.203594	6.270257	7.201511
ZWINT	2.43856	2.947081	2.95841	2.691762	2.388216	2.236653	3.393216	3.126453
ZXDC	7.177221	6.744795	5.054247	6.692575	6.351696	5.790396	6.067351	6.731919
ZYG11A	0.020028	0.010145	0.009889	0	0	0.020165	0.020328	0.010157
ZYG11B	7.14187	7.793271	7.145773	6.926062	8.167175	6.717129	7.072194	7.526214
ZYX	67.76516	61.43291	63.19697	69.18196	69.61091	59.85115	66.05309	66.2871
ZZEF1	6.198614	5.432392	4.609152	5.687862	6.143146	5.076014	5.747845	5.770807
ZZZ3	12.31338	13.86696	11.43287	12.96295	12.5295	11.55115	12.68049	12.97669

SF3	gene_Dbxref
0.360214	GeneID:101119665
0.003101	GeneID:101118147
0.092733	GeneID:101123200
0.410491	GeneID:101103704
1.058871	GeneID:101106675
0.027452	GeneID:101111851
9.862535	GeneID:101104648
20.47147	GeneID:101114977
1.026267	GeneID:101112496
4.786058	GeneID:101103322
1.959868	GeneID:101107425
36.34654	GeneID:101110977
45.6028	GeneID:101110742
0	GeneID:443531
4.402832	GeneID:101121030
0.243861	GeneID:101120843
50.69544	GeneID:101114460
2.012328	GeneID:101114732
6.268331	GeneID:101108833
19.48106	GeneID:101115306
14.15757	GeneID:101109984
2.137835	GeneID:101101878
1.530956	GeneID:101105462
16.53664	GeneID:101119015
12.94149	GeneID:101108240
7.682187	GeneID:101104887
3.555476	GeneID:101111169
0.708827	GeneID:105605850
0.575223	GeneID:101107561
3.122145	GeneID:101115292
6.104568	GeneID:101115555
0.397006	GeneID:101116568
3.105142	GeneID:443367
11.90706	GeneID:101102120
0.016208	GeneID:101121238
0.073856	GeneID:101122517
5.243666	GeneID:101115097
7.087227	GeneID:101113989
6.808233	GeneID:101103060
2.700625	GeneID:101119252
6.865486	GeneID:101110991
1.76678	GeneID:101112932
9.821865	GeneID:101115563
0	GeneID:101115303
0.009089	GeneID:101119776
3.361659	GeneID:101116746
3.623931	GeneID:101123431
0.032549	GeneID:101114921
0.295569	GeneID:101120522
4.125084	GeneID:101122673
0.136805	GeneID:101119199
29.48087	GeneID:101118659
3.923035	GeneID:101106333
33.25688	GeneID:101110874
33.37011	GeneID:101103598
12.39646	GeneID:101103804

17.76564	GeneID:101121904
10.29382	GeneID:101103525
7.656048	GeneID:780508
0.035491	GeneID:101107988
0.087394	GeneID:101113924
0.390023	GeneID:101113666
0.726343	GeneID:101114505
6.674511	GeneID:101112190
38.70214	GeneID:101106539
12.53141	GeneID:101116488
2.611144	GeneID:101114562
42.11257	GeneID:101114818
0.551051	GeneID:101121787
23.49538	GeneID:101118906
0.107197	GeneID:101116851
23.52623	GeneID:101117506
6.908007	GeneID:101102206
12.52218	GeneID:101109488
2.557886	GeneID:101121294
17.84648	GeneID:101103014
6.630567	GeneID:101122746
13.74676	GeneID:101105755
84.98091	GeneID:100125355
5.293569	GeneID:101122635
2.876348	GeneID:101115366
39.32131	GeneID:101119744
2.147589	GeneID:101113308
1.293844	GeneID:101112745
18.13444	GeneID:101108803
17.29153	GeneID:101115428
11.48214	GeneID:101122941
1.960283	GeneID:101121110
27.00901	GeneID:101102193
0.966243	GeneID:101118876
6.230116	GeneID:101118949
0.300869	GeneID:101114008
23.46261	GeneID:101112992
0	GeneID:101123385
70.67288	GeneID:101115375
6.696404	GeneID:101109880
2.638188	GeneID:101114481
4.657862	GeneID:101107052
6.555033	GeneID:101103837
26.81484	GeneID:101110625
81.82395	GeneID:101111925
9.718466	GeneID:443186
0.321405	GeneID:101114816
7.229566	GeneID:101118915
4.796998	GeneID:101113789
41.38075	GeneID:101111495
6.563696	GeneID:101123271
3.54746	GeneID:101119850
1.647865	GeneID:101102606
71.57158	GeneID:101117173
0.051298	GeneID:443001
12.29751	GeneID:101119319
10.61036	GeneID:101102615
5.999937	GeneID:101107717

44.80279	GeneID:101104601
44.42484	GeneID:101111975
20.42106	GeneID:101105589
3.473896	GeneID:101118970
8.31567	GeneID:101119491
13.6964	GeneID:101123137
0.104706	GeneID:101116927
2.96966	GeneID:101110616
0.013162	GeneID:101116173
8.999443	GeneID:101109037
0.468457	GeneID:101103622
19.27638	GeneID:101106585
4.581666	GeneID:101123535
16.14837	GeneID:101112511
0.801561	GeneID:101123006
43.76517	GeneID:101122696
2.165813	GeneID:101113185
13.1367	GeneID:101102850
0.922941	GeneID:101119176
40.32871	GeneID:654404
0	GeneID:101107168
21.88187	GeneID:101112791
47.90331	GeneID:101103148
0.936864	GeneID:101102743
0.225606	GeneID:101118240
0.008378	GeneID:101118188
7.197489	GeneID:101118059
1.408299	GeneID:101103981
15.97728	GeneID:101121789
10.3807	GeneID:101115474
4.785081	GeneID:114108762
18.73084	GeneID:101118626
0.167124	GeneID:101121460
12.7343	GeneID:101119642
0.049705	GeneID:101118010
43.72246	GeneID:101119876
7.373926	GeneID:101117016
0.069077	GeneID:101119583
26.94605	GeneID:100820744
12.1813	GeneID:101114832
4.303669	GeneID:101114897
45.00006	GeneID:101120130
0.018703	GeneID:443195
0.254835	GeneID:101112553
0	GeneID:101111841
19.68053	GeneID:101121296
0.203273	GeneID:101110508
25.54729	GeneID:101105582
3.438576	GeneID:101109383
41.91756	GeneID:101110905
38.72014	GeneID:101120728
11.29764	GeneID:101111781
32.89556	GeneID:101121475
1.200737	GeneID:101102389
10.43331	GeneID:101120375
26.33822	GeneID:101121307
0	GeneID:101120794
2.594635	GeneID:101112785

9.059972	GeneID:780456
27.24793	GeneID:101114126
30.97201	GeneID:101103801
0.764753	GeneID:101107662
311.5478	GeneID:101121051
739.9275	GeneID:443052
151.7875	GeneID:101110871
1.704128	GeneID:101111554
1192.474	GeneID:443340
70.34198	GeneID:101114256
0	GeneID:114117534
21.7831	GeneID:101108709
0	GeneID:101103114
0	GeneID:101106921
58.62312	GeneID:101122194
0.751888	GeneID:101116372
1.170072	GeneID:101115917
150.268	GeneID:101111737
28.16598	GeneID:101115545
59.80329	GeneID:101108440
13.2156	GeneID:101112894
61.59335	GeneID:101112965
75.2402	GeneID:101108990
4.135654	GeneID:101105653
5.915414	GeneID:101105517
10.98324	GeneID:101104475
10.97073	GeneID:101110005
0	GeneID:101102916
0.020817	GeneID:101120792
34.80123	GeneID:443161
18.1706	GeneID:101113836
0.177145	GeneID:101111867
21.49893	GeneID:443304
0.612629	GeneID:443156
6.65822	GeneID:101114095
7.994304	GeneID:101113801
4.59734	GeneID:101109618
5.240668	GeneID:105608759
3.070312	GeneID:101106352
0.532575	GeneID:101121925
0	GeneID:101123319
3.500174	GeneID:101106885
25.73262	GeneID:101116733
0.443459	GeneID:101117948
2.366418	GeneID:101106128
17.49963	GeneID:101105128
19.38549	GeneID:101116815
0.019161	GeneID:101120301
1.603956	GeneID:101113590
0	GeneID:101101897
0.472183	GeneID:101116564
0.239307	GeneID:101105313
16.79609	GeneID:101111218
0	GeneID:101119797
0.501637	GeneID:105605819
10.0073	GeneID:101115001
0.216661	GeneID:101112701
15.16803	GeneID:100415773

3.440973	GeneID:101105076
1.405036	GeneID:101118642
0.081946	GeneID:101112214
0.144335	GeneID:101106305
0.938132	GeneID:101120284
0.245379	GeneID:101107994
2.907018	GeneID:101110624
3.263223	GeneID:101118979
0.730119	GeneID:101102235
0.066714	GeneID:101115444
0.148271	GeneID:101102903
0.124843	GeneID:101106565
2.638601	GeneID:101110635
0.215246	GeneID:101103840
0.824795	GeneID:101114142
0.626483	GeneID:101120031
2.045295	GeneID:101119080
1.158099	GeneID:101122835
4.333877	GeneID:101111183
6.279455	GeneID:101107392
10.47267	GeneID:101106381
2.816598	GeneID:101119210
2.238599	GeneID:114110560
2.623936	GeneID:101121946
12.60767	GeneID:101104366
6.907076	GeneID:101105469
0	GeneID:114117586
0.811389	GeneID:101118042
3.12468	GeneID:101117852
0.863663	GeneID:101117762
1.14622	GeneID:101116318
7.75037	GeneID:101106933
4.115686	GeneID:101112143
0.209524	GeneID:101117327
0.008057	GeneID:101111248
2.456624	GeneID:101102089
4.021634	GeneID:101108203
2.842317	GeneID:101119139
0.421366	GeneID:105602258
4.601745	GeneID:101105735
15.86984	GeneID:101112667
0.022005	GeneID:101121944
1.881456	GeneID:101122401
0	GeneID:443337
1.039549	GeneID:443231
44.6065	GeneID:101123212
0.162717	GeneID:101106757
33.31756	GeneID:101117808
0.058026	GeneID:114110287
10.91853	GeneID:101117990
37.72052	GeneID:101102902
0.391121	GeneID:101117595
0.577038	GeneID:101106749
0.004644	GeneID:101109454
3.969568	GeneID:101113363
0.068281	GeneID:101116830
0.488569	GeneID:105610502
8.008857	GeneID:101122596

0.058057	GeneID:101121464
4.30937	GeneID:101121719
0.110414	GeneID:101110223
27.2706	GeneID:101121979
9.96235	GeneID:101121209
79.48745	GeneID:101120942
1.61328	GeneID:101109070
0.164883	GeneID:101121194
0.168064	GeneID:101102583
7.443633	GeneID:101116915
0.056835	GeneID:101110721
4.171436	GeneID:101118356
22.61382	GeneID:101104103
3.510505	GeneID:101111884
3.671386	GeneID:101103604
53.03052	GeneID:101112223
0.02445	GeneID:101111713
1.90783	GeneID:101113943
76.68163	GeneID:101113992
0.578971	GeneID:100127222
23.43581	GeneID:101111848
10.4093	GeneID:101103911
20.01015	GeneID:101116044
41.97162	GeneID:105605046
30.6995	GeneID:101122324
8.606687	GeneID:101103753
4.070608	GeneID:106991013
0.480542	GeneID:101108073
18.46486	GeneID:101122715
8.08073	GeneID:101121903
16.31179	GeneID:101121567
0.33573	GeneID:101109816
0.190108	GeneID:101112926
5.141036	GeneID:101107715
1.863398	GeneID:443330
10.44223	GeneID:101116836
1.475141	GeneID:101106222
0.039283	GeneID:101119915
5.530463	GeneID:101116791
4.516301	GeneID:101113772
1.060475	GeneID:100169940
0.348289	GeneID:101114869
0.338999	GeneID:100170316
1.99046	GeneID:100170317
0.519296	GeneID:100170318
0.434884	GeneID:100170319
1.947378	GeneID:443528
3.137022	GeneID:100170320
0.05719	GeneID:100294559
42.57965	GeneID:101112587
17.83791	GeneID:101108120
6.821791	GeneID:101107881
6.130155	GeneID:101103928
18.47904	GeneID:101105035
41.74942	GeneID:101119132
13.36294	GeneID:101121336
6.858329	GeneID:101116260
134.5826	GeneID:101119474

3.592244	GeneID:101109449
1.903675	GeneID:101119212
35.33496	GeneID:101119095
45.71565	GeneID:101115800
11.06691	GeneID:101111967
0.293158	GeneID:101116804
12.84297	GeneID:101121933
0.967958	GeneID:101116838
37.59041	GeneID:101108875
0	GeneID:101103897
1.406959	GeneID:101123646
0	GeneID:101103646
24.07213	GeneID:101114001
8.161414	GeneID:101106396
11.67903	GeneID:101111683
1.063393	GeneID:101116895
17.1422	GeneID:101113584
0	GeneID:101115736
0.323139	GeneID:101120523
2.144392	GeneID:101112799
0.304732	GeneID:101111586
17.40965	GeneID:101103789
10.29682	GeneID:101104547
12.78742	GeneID:100037685
5.088637	GeneID:101108220
3.810106	GeneID:101111501
0.017185	GeneID:101110431
1.336124	GeneID:101103313
14.85691	GeneID:101111060
7.277954	GeneID:101119144
2.755941	GeneID:101111326
10.64544	GeneID:101116530
4.940561	GeneID:443121
28.73898	GeneID:101114511
16.08372	GeneID:101107246
0.314384	GeneID:101112654
11.81613	GeneID:101103870
4.599754	GeneID:101104553
0.158682	GeneID:101105323
15.67483	GeneID:101107299
0.022733	GeneID:443269
0.906834	GeneID:101119440
3.357324	GeneID:101115757
8.886134	GeneID:443107
0.07526	GeneID:443335
17.24031	GeneID:101111567
0.022834	GeneID:101103446
0.029821	GeneID:101111660
11.47871	GeneID:101114886
203.1649	GeneID:101112245
30.69895	GeneID:101109660
19.36544	GeneID:101114101
2.868692	GeneID:101112709
2.59044	GeneID:101120502
200.1983	GeneID:101110887
8.315013	GeneID:105603281
15.03645	GeneID:443467
0.334626	GeneID:101109301

79.53366	GeneID:101115128
4.341488	GeneID:101119030
0.909179	GeneID:443392
0.097348	GeneID:100127218
0	GeneID:101103056
12.1569	GeneID:101106862
7.959054	GeneID:101121889
6.235752	GeneID:101122182
8.763551	GeneID:101121574
3.497328	GeneID:101108883
0.279662	GeneID:101118054
6.594519	GeneID:101117593
21.12947	GeneID:101121350
23.55865	GeneID:100302342
13.03651	GeneID:101119947
0.027135	GeneID:101104920
0	GeneID:101102698
0.022632	GeneID:101123222
0.004782	GeneID:106990977
47.64617	GeneID:101102150
13.74188	GeneID:100302085
71.84469	GeneID:101110653
18.54136	GeneID:101113201
20.68813	GeneID:101103031
0.119637	GeneID:101121984
6.089941	GeneID:102238525
0.093847	GeneID:101121552
0.978314	GeneID:101113475
0.004317	GeneID:101107607
13.14507	GeneID:101123647
6.664736	GeneID:101118286
13.78646	GeneID:101108581
11.22664	GeneID:101105509
12.47615	GeneID:101114495
9.69077	GeneID:101114239
0.088156	GeneID:101116040
1.177086	GeneID:101102535
0.11293	GeneID:101109277
0.236755	GeneID:101117111
1.235038	GeneID:101121177
12.89235	GeneID:101119815
23.72867	GeneID:101119556
8.448388	GeneID:101109958
7.549885	GeneID:101107130
25.61688	GeneID:100158234
65.16834	GeneID:100820738
2.333002	GeneID:101118854
0.078036	GeneID:101102953
27.27513	GeneID:101121637
5.847678	GeneID:101120824
0	GeneID:101103641
2.677831	GeneID:101110956
8.07598	GeneID:101109421
49.78594	GeneID:100294652
6.031833	GeneID:101109114
15.45211	GeneID:101119242
10.77978	GeneID:101118462
46.58923	GeneID:101114547

5.783081	GeneID:101106393
45.90011	GeneID:101112853
0	GeneID:101115684
2.671384	GeneID:443393
35.15178	GeneID:101114151
5.444007	GeneID:101111900
11.4346	GeneID:101108974
40.88741	GeneID:443343
0.585588	GeneID:101117509
20.0445	GeneID:101109831
0.096832	GeneID:100913154
7.760507	GeneID:101114182
228.7094	GeneID:101116511
0.65672	GeneID:101108502
85.70363	GeneID:101118798
1.269264	GeneID:101112687
11.66221	GeneID:101105895
15.95674	GeneID:101107828
24.42646	GeneID:101106176
32.02676	GeneID:101108127
0	GeneID:101108757
35.6368	GeneID:101109411
393.7552	GeneID:443085
0.247453	GeneID:443440
3.179308	GeneID:101105422
4.508879	GeneID:101104800
6.499841	GeneID:101120844
7.365997	GeneID:101112896
20.23362	GeneID:101108526
5.660149	GeneID:101101953
5.707214	GeneID:101121650
14.78215	GeneID:101108665
4.411118	GeneID:101108436
3.67934	GeneID:101109656
4.672183	GeneID:101109477
0.191015	GeneID:101114094
0	GeneID:101106950
0.946983	GeneID:101112199
4.62333	GeneID:101111374
1.098998	GeneID:101115575
13.91553	GeneID:101110359
6.132077	GeneID:101117557
18.43042	GeneID:101111111
6.311994	GeneID:101105845
13.0053	GeneID:101109968
2.997007	GeneID:101104088
3.404428	GeneID:101104651
6.889575	GeneID:101113685
2.251864	GeneID:101105929
0.594179	GeneID:101116587
2.186353	GeneID:101117390
3.520747	GeneID:443326
4.711101	GeneID:101121693
2.695929	GeneID:101119049
0.082501	GeneID:101116008
0.542786	GeneID:105603294
1.278888	GeneID:101123195
9.478197	GeneID:101109592

4.308583	GeneID:101116432
0	GeneID:101107577
0.016366	GeneID:101107946
0.84587	GeneID:101104710
0.330254	GeneID:101116426
41.63888	GeneID:105606265
5.371938	GeneID:101113530
14.13041	GeneID:101121095
0	GeneID:101104205
9.147119	GeneID:101117963
17.68118	GeneID:101110851
1.063952	GeneID:101121504
12.69398	GeneID:101116953
0.311657	GeneID:101118759
3.298749	GeneID:101110818
0.153825	GeneID:114116651
0	GeneID:101117058
43.74914	GeneID:101119154
0	GeneID:101121773
0.435402	GeneID:101109693
3.317104	GeneID:101106213
4.269528	GeneID:101111179
0.571407	GeneID:105603614
10.39684	GeneID:101113829
18.6523	GeneID:101107573
0	GeneID:101122221
4.91682	GeneID:101106760
7.012532	GeneID:101108810
26.09069	GeneID:101103590
23.0591	GeneID:101114081
0	GeneID:101102023
5.326064	GeneID:101107149
1.969298	GeneID:101103503
5.040646	GeneID:101104276
0	GeneID:101102238
0.118616	GeneID:101111338
10.2391	GeneID:101116329
8.628654	GeneID:101117916
2.894965	GeneID:101111128
23.30597	GeneID:101114121
16.92824	GeneID:101115335
41.19306	GeneID:101120520
32.25459	GeneID:101113822
13.97883	GeneID:101108299
8.651725	GeneID:101105914
9.943412	GeneID:101102840
13.80251	GeneID:101104605
2.447304	GeneID:101109279
11.75426	GeneID:101113611
0.767932	GeneID:497271
1.294657	GeneID:497273
3.656819	GeneID:101107460
2.766881	GeneID:101120347
14.29655	GeneID:105610840
10.40187	GeneID:101104317
2.096864	GeneID:101120690
0.023278	GeneID:101113349
0.013827	GeneID:105609434

0	GeneID:101114904
0.195725	GeneID:101121569
1.921622	GeneID:101115623
3.170376	GeneID:101120296
0	GeneID:101113304
0	GeneID:105613904
0	GeneID:101105255
0.029382	GeneID:101108328
10.25984	GeneID:101102652
25.10199	GeneID:101103426
7.772861	GeneID:101111273
0.350432	GeneID:101115620
14.42378	GeneID:101115403
0.135617	GeneID:101104451
14.97819	GeneID:101104569
10.10835	GeneID:101113362
0.064861	GeneID:100820746
16.0367	GeneID:101116585
25.07133	GeneID:101110169
11.93803	GeneID:101106986
37.70266	GeneID:101121549
3.030914	GeneID:101110425
13.49231	GeneID:101118233
5.472765	GeneID:101108527
2.996381	GeneID:105609034
12.73436	GeneID:101103397
0.148689	GeneID:101109057
20.20025	GeneID:101120533
0.53287	GeneID:101113927
0.655639	GeneID:101115875
13.85362	GeneID:101108593
4.904685	GeneID:101119271
5.607296	GeneID:101120373
0.141883	GeneID:101104753
0.181482	GeneID:101113483
2.959726	GeneID:101105441
0.018272	GeneID:101118396
0.006641	GeneID:101121170
0	GeneID:101113278
15.82867	GeneID:101117123
0.878703	GeneID:101112948
4.638188	GeneID:101113669
13.99923	GeneID:101104837
0	GeneID:101104325
1.778659	GeneID:101106210
2.559509	GeneID:101121070
2.087373	GeneID:101116848
10.45474	GeneID:101103486
5.851414	GeneID:101115060
7.079821	GeneID:101123497
5.861365	GeneID:101109431
0.115969	GeneID:101102636
11.40288	GeneID:101119635
0.811218	GeneID:101112514
2.105002	GeneID:101109885
0	GeneID:101103663
0.129671	GeneID:105608913
0.02877	GeneID:101105587

0.070298	GeneID:101116515
0.156732	GeneID:101121676
2.090286	GeneID:105603272
8.556247	GeneID:101116686
0.049746	GeneID:114108648
2.381042	GeneID:101103287
0.620443	GeneID:101102029
0.497931	GeneID:101103301
0	GeneID:101102780
7.481588	GeneID:101116023
9.66344	GeneID:101116045
1.809814	GeneID:101116524
28.33432	GeneID:101118480
0.31177	GeneID:105614855
3.015619	GeneID:101114464
0.035269	GeneID:101106248
0.009469	GeneID:101120120
15.33201	GeneID:101108825
3.372647	GeneID:105608534
5.40936	GeneID:101105118
2.172945	GeneID:101118853
72.9841	GeneID:101105917
109.3689	GeneID:106990102
31.34515	GeneID:101102602
38.50806	GeneID:101104860
26.75927	GeneID:101108204
6.803621	GeneID:101113070
382.6657	GeneID:101123269
0	GeneID:101122664
23.95649	GeneID:101105975
1.18238	GeneID:101107203
659.2847	GeneID:100048993
25.47802	GeneID:101111885
59.77517	GeneID:101107176
81.83718	GeneID:101114445
20.52533	GeneID:101105245
23.98205	GeneID:101118663
26.4018	GeneID:101109752
3.437141	GeneID:101112561
0.097404	GeneID:101106783
68.38721	GeneID:105607129
44.99911	GeneID:101104557
5.281601	GeneID:101119564
15.40573	GeneID:101121713
24.37645	GeneID:101104002
6.435911	GeneID:101119310
21.15227	GeneID:101116140
38.17766	GeneID:101114351
33.83352	GeneID:101122493
16.98331	GeneID:101104633
7.254229	GeneID:101117145
21.21315	GeneID:101108595
22.50871	GeneID:101121898
30.12851	GeneID:101115974
90.2254	GeneID:101122320
61.98228	GeneID:101122460
19.34905	GeneID:101112650
0.112084	GeneID:101105605

37.98755	GeneID:101121852
32.69908	GeneID:101122075
0.237759	GeneID:101118077
30.72468	GeneID:101107603
5.904857	GeneID:101120710
12.93466	GeneID:101106174
14.05892	GeneID:101107757
13.93895	GeneID:101115405
5.32539	GeneID:101102439
8.628884	GeneID:101121268
5.932866	GeneID:101120100
0.46188	GeneID:114108638
3.178415	GeneID:100135699
1.970993	GeneID:101105978
0.883639	GeneID:101119256
7.955384	GeneID:101115028
6.307439	GeneID:101118129
4.768878	GeneID:101110258
16.07087	GeneID:101116399
4.361594	GeneID:101118948
7.361538	GeneID:101104664
0.770573	GeneID:101115458
54.97103	GeneID:101104951
6.766038	GeneID:101102919
14.419	GeneID:101103273
0.18967	GeneID:106990124
85.23637	GeneID:101115462
11.39727	GeneID:101116123
33.27516	GeneID:101103438
3.808853	GeneID:101111553
33.49852	GeneID:101108774
18.36815	GeneID:101105523
4.046591	GeneID:101116898
2.659258	GeneID:101119419
3.985642	GeneID:101105352
121.4972	GeneID:101119774
26.72157	GeneID:101104594
0	GeneID:101116760
0.002011	GeneID:101107590
0.022714	GeneID:101103930
6.35952	GeneID:100037691
0.016716	GeneID:100301555
2.178	GeneID:101122247
535.3164	GeneID:101104361
34.29821	GeneID:101112253
0.14781	GeneID:101102138
0.023491	GeneID:101117521
0.564435	GeneID:101111443
2.26939	GeneID:101121210
15.14747	GeneID:101113910
13.31483	GeneID:101123352
19.57009	GeneID:101102428
249.9309	GeneID:101111593
15.90383	GeneID:101108927
14.7304	GeneID:101109218
21.49179	GeneID:101114693
8.840897	GeneID:101108335
4.340539	GeneID:101105385

119.6796	GeneID:442999
0.324102	GeneID:101102866
0.058925	GeneID:101111499
61.75445	GeneID:443047
0.043133	GeneID:443268
0.186942	GeneID:443251
0	GeneID:101121248
0.20422	GeneID:101103697
6.041305	GeneID:443183
13.01153	GeneID:443184
6.821532	GeneID:101103571
3.841772	GeneID:443042
17.15468	GeneID:100305131
9.475033	GeneID:101123146
15.77235	GeneID:101109800
3.279472	GeneID:101105821
1.537186	GeneID:101120169
61.72806	GeneID:101103008
13.49282	GeneID:443427
7.557837	GeneID:101106945
86.67424	GeneID:100302302
37.7861	GeneID:100302303
138.0289	GeneID:100302304
78.79212	GeneID:100302305
49.26455	GeneID:100302306
7.445097	GeneID:101110163
34.34239	GeneID:101119250
10.9164	GeneID:101115447
15.48554	GeneID:101102247
8.982188	GeneID:101116164
1.990229	GeneID:101113941
12.76036	GeneID:101103593
17.51114	GeneID:100302307
26.20465	GeneID:100302308
0.500859	GeneID:101122363
1.217776	GeneID:780468
57.31412	GeneID:101115718
19.35385	GeneID:101118219
31.85663	GeneID:101107633
4.899221	GeneID:101103069
14.0005	GeneID:101114797
0.747236	GeneID:101123443
18.97155	GeneID:101119180
12.06309	GeneID:101118792
5.072764	GeneID:101113027
0.970842	GeneID:101106623
15.94031	GeneID:101114376
0.470741	GeneID:101116628
15.49673	GeneID:101123648
4.366405	GeneID:101119904
3.467313	GeneID:101109429
2.19508	GeneID:101109966
8.698778	GeneID:101119739
4.053248	GeneID:101102444
19.82648	GeneID:101107830
4.684638	GeneID:101123168
6.054199	GeneID:101105202
10.31266	GeneID:101116614

0.604392	GeneID:101105098
14.58256	GeneID:101117354
0.064432	GeneID:100913153
5.584894	GeneID:101106270
1.538345	GeneID:101107503
6.73662	GeneID:101121370
2.232754	GeneID:101104674
1.526921	GeneID:101112901
20.28969	GeneID:101116866
1.497232	GeneID:101118504
3.76476	GeneID:101108216
0.665416	GeneID:101119898
92.98811	GeneID:101114713
22.0231	GeneID:101106069
0.11339	GeneID:101122156
17.88931	GeneID:101104265
11.07956	GeneID:101104295
10.50683	GeneID:101107175
6.510267	GeneID:101110561
30.00659	GeneID:101117190
2.358381	GeneID:101107803
4.154635	GeneID:101119743
4.925043	GeneID:101104090
6.217723	GeneID:101122356
7.025149	GeneID:101110311
6.218658	GeneID:101121729
6.275043	GeneID:101118430
9.262083	GeneID:101113898
6.098453	GeneID:101112674
4.856935	GeneID:101103763
0	GeneID:101119128
27.73159	GeneID:101121007
0.052959	GeneID:101104491
5.263741	GeneID:101115939
7.499408	GeneID:101114961
15.62209	GeneID:101105817
7.859016	GeneID:101104043
1.016199	GeneID:101110030
19.54332	GeneID:101115263
12.30511	GeneID:101108839
9.348961	GeneID:101112969
7.203806	GeneID:101111883
0.792549	GeneID:101110365
0.028741	GeneID:101116967
7.711077	GeneID:101122697
8.284822	GeneID:101116701
1.679854	GeneID:101114695
15.48898	GeneID:101121742
20.10042	GeneID:101116578
19.35947	GeneID:101109306
2.326836	GeneID:106991757
43.9323	GeneID:100145883
3.489467	GeneID:101105498
0	GeneID:101119452
2.781456	GeneID:101123516
0	GeneID:101105298
13.63649	GeneID:101118726
0.621188	GeneID:101107869

32.27799	GeneID:101118640
3.434876	GeneID:101116745
52.67799	GeneID:100302310
64.56864	GeneID:100302309
56.72021	GeneID:100302311
16.97305	GeneID:100145881
19.65295	GeneID:100302312
6.698406	GeneID:101109813
54.19343	GeneID:100302313
8.44134	GeneID:101111823
0.051149	GeneID:100302314
2.736124	GeneID:101102858
112.4338	GeneID:100302315
11.31361	GeneID:101122815
29.91706	GeneID:101116352
6.232318	GeneID:101116126
24.14944	GeneID:100302316
112.8647	GeneID:100302317
0	GeneID:101117670
19.98522	GeneID:101119911
18.55228	GeneID:101103718
0.137061	GeneID:101118823
0.63953	GeneID:101116405
0	GeneID:101104515
0.128243	GeneID:101118121
0.852199	GeneID:101106993
3.827113	GeneID:101106680
4.312665	GeneID:101122203
8.959173	GeneID:101111147
1.565991	GeneID:101107335
10.34288	GeneID:101106745
4.851016	GeneID:101108027
13.00458	GeneID:101107578
0.204586	GeneID:105605646
3.854177	GeneID:101122765
0.508105	GeneID:101109503
0	GeneID:105603822
7.564242	GeneID:101105456
6.831861	GeneID:101122031
6.838197	GeneID:101102988
28.86084	GeneID:443285
0.438858	GeneID:100135448
6.368509	GeneID:443262
0.517151	GeneID:101112046
142.6973	GeneID:101113818
86.40054	GeneID:101113558
193.4962	GeneID:101110485
159.6598	GeneID:101105028
40.33424	GeneID:105601868
62.8558	GeneID:101106701
39.49416	GeneID:100037671
20.90597	GeneID:101102340
26.00115	GeneID:101102906
0.011857	GeneID:101106887
8.424931	GeneID:101108418
7.629234	GeneID:101112493
13.95283	GeneID:101101973
14.04443	GeneID:101110324

51.14824	GeneID:101107094
38.87057	GeneID:101112169
1.712796	GeneID:101110504
5.623823	GeneID:101122266
9.663417	GeneID:101108314
0.434772	GeneID:101115759
2.426283	GeneID:101109286
16.39283	GeneID:101116287
0.368124	GeneID:101110236
5.237041	GeneID:101113591
2.340734	GeneID:101109354
0.335002	GeneID:101108229
0.437058	GeneID:101117410
0.041914	GeneID:105607299
20.01315	GeneID:101110644
0.591454	GeneID:105602970
0.029753	GeneID:101108992
1.768268	GeneID:101115498
45.54395	GeneID:101117904
3.605245	GeneID:101121732
3.398583	GeneID:101122198
6.475989	GeneID:101102555
14.12805	GeneID:101120897
11.81509	GeneID:101108274
0	GeneID:106991148
1.421157	GeneID:101101864
0.468689	GeneID:101111073
8.861442	GeneID:101105590
0	GeneID:101103272
0	GeneID:101106002
0.039476	GeneID:101115385
0	GeneID:101103518
1.33516	GeneID:101114059
8.1696	GeneID:101103551
0.582639	GeneID:101117496
0.015561	GeneID:101108021
10.31698	GeneID:101104650
6.196515	GeneID:101115908
16.21791	GeneID:101108118
1.640901	GeneID:101123093
15.63212	GeneID:101113387
18.28011	GeneID:101109043
14.97555	GeneID:101123305
0.045066	GeneID:101110666
1.63909	GeneID:114110104
0	GeneID:101106621
0.053345	GeneID:105608623
2.394108	GeneID:101113564
12.652	GeneID:100307033
5.648503	GeneID:101123293
6.799327	GeneID:101116157
16.04057	GeneID:101119775
18.21749	GeneID:101111689
0.019664	GeneID:101120562
0	GeneID:101120341
0	GeneID:101113062
0.644595	GeneID:101113830
100.3822	GeneID:100127221

39.94856	GeneID:101106412
0.419048	GeneID:101114754
13.39961	GeneID:105611964
45.43164	GeneID:101106257
25.21762	GeneID:101120073
3.713092	GeneID:101121435
0.733967	GeneID:106991749
14.06971	GeneID:101118195
0.01505	GeneID:101118658
0.791334	GeneID:101118136
3.625543	GeneID:443284
13.91118	GeneID:101108885
245.8801	GeneID:101115358
18.85156	GeneID:101107539
11.36726	GeneID:100135685
10.60088	GeneID:101115012
2.394576	GeneID:101117135
0.023228	GeneID:101118463
1.217124	GeneID:101123440
7.479155	GeneID:101113692
6.925193	GeneID:101109427
0.01964	GeneID:101117128
0	GeneID:100126570
29.05039	GeneID:101119092
5.346344	GeneID:101103903
10.31573	GeneID:101105229
2.295275	GeneID:101122447
3.416488	GeneID:101106803
0.012538	GeneID:101116652
6.903045	GeneID:101110286
10.9083	GeneID:101117917
13.39544	GeneID:101108548
2.653741	GeneID:101116073
462.7676	GeneID:100216465
16.10854	GeneID:101114972
14.62404	GeneID:541606
22.96512	GeneID:101113631
10.29493	GeneID:101123619
10.38677	GeneID:101109004
0	GeneID:101107247
4.433398	GeneID:101122947
13.70469	GeneID:101112968
15.53456	GeneID:101107357
20.89184	GeneID:101115063
6.805337	GeneID:101119220
1.24535	GeneID:101103839
9.729822	GeneID:101119171
9.93029	GeneID:101121043
38.33909	GeneID:101121560
8.145106	GeneID:101113390
33.10716	GeneID:101122243
4.209421	GeneID:101108002
11.09133	GeneID:101118272
6.684021	GeneID:100913169
7.5715	GeneID:101121977
9.474137	GeneID:100913171
22.50924	GeneID:101112811
11.32617	GeneID:101105137

2.508902	GeneID:101104070
53.95676	GeneID:101120404
17.46009	GeneID:101120873
2.053101	GeneID:101105847
12.78066	GeneID:101120266
18.14137	GeneID:101108302
0.092957	GeneID:101103067
0	GeneID:101104713
2.913277	GeneID:101102971
33.57124	GeneID:443451
3.180648	GeneID:101107997
0.804285	GeneID:101117246
5.784326	GeneID:101122863
7.121127	GeneID:101117857
15.22295	GeneID:101110296
5.551462	GeneID:101122839
7.142038	GeneID:101120253
10.10278	GeneID:101103810
10.58235	GeneID:101108996
7.852131	GeneID:101106047
5.710291	GeneID:101107059
0.797784	GeneID:101107321
103.9269	GeneID:443381
0.563101	GeneID:101118148
0	GeneID:101103751
36.8743	GeneID:443384
9.14069	GeneID:101103490
32.13074	GeneID:101107568
0.00363	GeneID:101113044
0.107167	GeneID:101111766
40.41165	GeneID:101104344
3.082291	GeneID:101111207
12.65255	GeneID:101111268
4.764781	GeneID:101121046
0.139349	GeneID:101101873
28.05679	GeneID:101104306
37.81988	GeneID:101121651
7.776104	GeneID:101123576
0.432634	GeneID:101116166
0	GeneID:114116709
259.8524	GeneID:101111413
255.6157	GeneID:101106318
162.6053	GeneID:101115299
93.43091	GeneID:101117502
310.576	GeneID:105606223
112.5063	GeneID:101110916
0.345831	GeneID:443410;GeneID:114108664
312.6999	GeneID:443542
157.1465	GeneID:101108285
135.3649	GeneID:101111410
223.0001	GeneID:101111455
180.0021	GeneID:105610140
143.5859	GeneID:101123491
155.9138	GeneID:101114570
206.4709	GeneID:100217408
105.0222	GeneID:101113030
273.5474	GeneID:100305130
56.66536	GeneID:101119886

0.095284	GeneID:101123629
65.44792	GeneID:101103453
20.03763	GeneID:101123476
9.751475	GeneID:101121550
3.723311	GeneID:101104057
68.15947	GeneID:101114221
150.9408	GeneID:443003
74.99991	GeneID:101108509
1.877732	GeneID:101117677
239.3628	GeneID:101108422
2.169242	GeneID:101122020
33.45436	GeneID:101102025
69.80415	GeneID:101107683
53.34617	GeneID:494439
13.5151	GeneID:101122445
32.42721	GeneID:101103310
89.37432	GeneID:101121176
107.2332	GeneID:494438
0.070021	GeneID:101110043
92.58332	GeneID:101112724
0	GeneID:105608858
163.1933	GeneID:101103451
0.07597	GeneID:101123332
0	GeneID:101102659
35.00238	GeneID:101113504
7.65747	GeneID:101116379
0.329328	GeneID:443046
6.252642	GeneID:101119308
10.22626	GeneID:101115500
14.22201	GeneID:101103116
0.211473	GeneID:101116987
1.848055	GeneID:101108837
13.59766	GeneID:101119494
14.8604	GeneID:101108356
22.07957	GeneID:101112620
8.244754	GeneID:101111895
6.683792	GeneID:101122627
2.724763	GeneID:101106566
40.22875	GeneID:101115524
3.454939	GeneID:101110882
8.963647	GeneID:100188914
2.376975	GeneID:101119865
8.441993	GeneID:101117394
2.519705	GeneID:101113284
49.89402	GeneID:101109264
7.057358	GeneID:101103748
12.03787	GeneID:101106796
18.98875	GeneID:100037683
14.44021	GeneID:100171397
5.350731	GeneID:101106632
3.254463	GeneID:101123462
0.624969	GeneID:101103448
18.13538	GeneID:101113945
47.16254	GeneID:101121247
5.911378	GeneID:101109520
1.666751	GeneID:101114173
62.09059	GeneID:101119707
4.076098	GeneID:100216431

26.56348	GeneID:101106608
10.50808	GeneID:101106860
0.256721	GeneID:101104774
3.955951	GeneID:114110670
12.90486	GeneID:101111025
0.288364	GeneID:101114855
5.828188	GeneID:101121004
0.05622	GeneID:443477
9.701615	GeneID:101115666
1.451631	GeneID:100528010
0	GeneID:443050
0.641393	GeneID:100528011
9.237924	GeneID:101122414
1.879838	GeneID:101121241
0.155099	GeneID:101118888
9.526828	GeneID:101122656
9.991579	GeneID:101109815
18.72063	GeneID:101102750
3.504114	GeneID:101123437
28.16598	GeneID:101103677
21.19782	GeneID:101122702
0.727476	GeneID:101110393
0	GeneID:101110329
264.3597	GeneID:443295
1.921805	GeneID:101104544
13.75013	GeneID:101116957
0.009376	GeneID:101122666
0.436006	GeneID:101121025
7.621383	GeneID:101111585
0	GeneID:101118412
3.39861	GeneID:101105768
0.642676	GeneID:101116508
0	GeneID:101102248
7.986386	GeneID:101111917
16.77151	GeneID:101117403
0.246967	GeneID:101113066
0.016656	GeneID:101102267
1.595131	GeneID:101103203
0.070662	GeneID:101110268
0.314829	GeneID:101107579
1.585416	GeneID:101123400
2.995234	GeneID:101111309
0.874117	GeneID:101103996
0.34109	GeneID:101119388
0	GeneID:101119323
13.11715	GeneID:101115274
6.207979	GeneID:101105615
38.19591	GeneID:100620048
12.30342	GeneID:101114825
11.87782	GeneID:101103686
5.349147	GeneID:101104949
2.682162	GeneID:101116670
0.876403	GeneID:101114836
12.0215	GeneID:100316848
5.88441	GeneID:101105702
3.278828	GeneID:101110346
3.138495	GeneID:101102082
0.660463	GeneID:101103328

24.26303	GeneID:101113145
17.94998	GeneID:101121412
0	GeneID:443356
5.866673	GeneID:101119667
5.008565	GeneID:101102027
12.61067	GeneID:101117134
3.858722	GeneID:101108838
10.5774	GeneID:780444
79.20924	GeneID:101117995
15.11077	GeneID:101112009
26.01908	GeneID:101108530
5.838144	GeneID:101118503
21.98164	GeneID:101105952
47.5305	GeneID:100135439
2.039596	GeneID:101109374
14.90883	GeneID:101114113
24.90261	GeneID:101109895
27.99582	GeneID:101114328
0.012614	GeneID:101120915
0.072355	GeneID:101107923
4.277807	GeneID:443062
192.0538	GeneID:443242
188.1177	GeneID:101112433
0	GeneID:101120182
1.152031	GeneID:101122360
5.60291	GeneID:101105771
17.32552	GeneID:101111666
1.818917	GeneID:101108373
0	GeneID:101115337
0.11124	GeneID:101110572
56.33196	GeneID:443110
9.428897	GeneID:101119849
0.660973	GeneID:101110146
0.249256	GeneID:105607156
1.460693	GeneID:101115558
15.4524	GeneID:443059
24.24655	GeneID:101104014
20.05127	GeneID:101113982
20.44101	GeneID:101106579
5.124446	GeneID:101111257
1.419506	GeneID:101123490
18.26331	GeneID:101120288
0.059401	GeneID:101105921
4.396197	GeneID:101115823
5.319494	GeneID:101115491
2.430217	GeneID:101122679
3.491746	GeneID:101103758
9.000525	GeneID:101118468
11.81035	GeneID:101110416
6.342796	GeneID:101120552
2.631024	GeneID:101107867
3.590145	GeneID:101118868
8.248221	GeneID:101115417
63.88742	GeneID:101111740
0.075738	GeneID:101105963
10.41919	GeneID:101104394
108.3262	GeneID:101111605
1.88506	GeneID:101117693

2.312667	GeneID:101106808
6.245268	GeneID:101102166
30.41216	GeneID:101109325
6.494637	GeneID:101108670
8.169234	GeneID:101118977
0.15933	GeneID:443485
9.916113	GeneID:443450
31.24917	GeneID:101116189
2.211905	GeneID:101122013
4.414331	GeneID:101102112
36.12957	GeneID:100145857
27.04768	GeneID:101113939
10.03227	GeneID:101104290
10.13639	GeneID:101113372
2.525847	GeneID:101119124
3.686114	GeneID:101116868
4.543825	GeneID:101119602
5.326158	GeneID:101114560
25.40045	GeneID:443061
0	GeneID:101106530
2.636984	GeneID:100505402
7.983351	GeneID:101112670
16.54088	GeneID:101117580
0.162262	GeneID:101113402
0.58056	GeneID:101120453
6.379352	GeneID:101111483
9.811743	GeneID:101110557
2.258515	GeneID:101114452
6.389186	GeneID:101103013
11.00514	GeneID:101113721
34.75662	GeneID:101103285
3.740933	GeneID:101114572
11.51576	GeneID:101114641
30.44905	GeneID:101111718
4.550189	GeneID:101110907
0.278002	GeneID:101121875
13.35808	GeneID:100286796
8.08413	GeneID:101122580
1.558831	GeneID:101121071
13.91432	GeneID:101113436
17.64736	GeneID:101104112
11.30836	GeneID:101106011
0.257878	GeneID:106991729
0.733723	GeneID:101120781
2.150191	GeneID:101117275
10.12296	GeneID:101117279
0.22354	GeneID:101103417
56.57201	GeneID:100913170
0	GeneID:101108148
0.040791	GeneID:443287
0.007479	GeneID:101103875
1.835573	GeneID:101119224
0.015553	GeneID:101111023
2.73207	GeneID:101107646
0.386331	GeneID:101106552
3.732023	GeneID:101115641
0.028898	GeneID:101109837
0	GeneID:101123465

0.00399	GeneID:101123543
0	GeneID:101118062
18.8318	GeneID:101114348
21.40391	GeneID:101105700
33.69071	GeneID:101118422
1.355932	GeneID:105605636
13.0134	GeneID:101108890
11.17371	GeneID:101112619
0.085553	GeneID:101121536
0.054833	GeneID:101115341
1.963494	GeneID:677670
135.0265	GeneID:443011
0.466889	GeneID:114110552
6.701814	GeneID:101112909
0	GeneID:101119410
41.73748	GeneID:100169939
12.51549	GeneID:101112806
0.034759	GeneID:105608451
0.016866	GeneID:101108575
7.152394	GeneID:101119363
0.079221	GeneID:101105646
12.52565	GeneID:101112119
2.378008	GeneID:101116936
28.84026	GeneID:101118239
1.934997	GeneID:101102925
4.414963	GeneID:101110097
28.69107	GeneID:101117837
1928.726	GeneID:443550
8.655344	GeneID:101119119
3.18305	GeneID:101114507
12.59168	GeneID:101114242
15.83449	GeneID:101119930
15.51624	GeneID:101120189
10.27472	GeneID:101123390
9.897361	GeneID:101103635
9.929829	GeneID:106990123
4.232498	GeneID:101103339
0.974601	GeneID:101111167
1.791713	GeneID:101107310
110.5045	GeneID:101111816
1.966431	GeneID:101111241
0	GeneID:101105150
20.89029	GeneID:101121559
4.626296	GeneID:101112341
8.613449	GeneID:100145878
3.568806	GeneID:101107314
16.43957	GeneID:101113763
27.83912	GeneID:101118353
20.60047	GeneID:101119750
35.01634	GeneID:101122875
7.104292	GeneID:101108051
24.18591	GeneID:101103337
7.431778	GeneID:101122334
14.56833	GeneID:101117688
16.59894	GeneID:101107523
3.634489	GeneID:101108049
45.58136	GeneID:443502
0.035626	GeneID:101107231

1.763694	GeneID:443174
23.52967	GeneID:443175
6.794061	GeneID:101120626
5.601637	GeneID:105604271
0.390157	GeneID:101118437
32.32524	GeneID:101112282
1.232213	GeneID:443454
28.41007	GeneID:101103793
17.29446	GeneID:101117987
5.161075	GeneID:101111794
1.104548	GeneID:101103124
19.02045	GeneID:101107140
9.233063	GeneID:101122574
5.191579	GeneID:101108952
8.575699	GeneID:101103240
31.2617	GeneID:101108441
64.88168	GeneID:101110026
25.29638	GeneID:101110283
16.43585	GeneID:101123082
16.26566	GeneID:101107045
5.007163	GeneID:101115795
14.82323	GeneID:101122750
8.710537	GeneID:101123165
35.47063	GeneID:101114744
14.41877	GeneID:101115107
0	GeneID:101123609
7.371149	GeneID:101109624
3.872822	GeneID:101122704
7.670902	GeneID:101112472
2.157296	GeneID:101106602
14.30953	GeneID:101109576
6.186031	GeneID:101106256
14.89756	GeneID:101121089
14.12244	GeneID:101109920
0.089369	GeneID:101104332
0	GeneID:101108410
0.380799	GeneID:101106611
0	GeneID:101107120
0	GeneID:101107375
1.693037	GeneID:101106867
19.00815	GeneID:101118431
19.73836	GeneID:101104841
10.13518	GeneID:101102998
19.25464	GeneID:101107438
15.36581	GeneID:101107047
2.411096	GeneID:101111600
2.403411	GeneID:101108584
1.604791	GeneID:101109980
13.13177	GeneID:101114756
13.65234	GeneID:101121337
55.02485	GeneID:101110276
13.80364	GeneID:101109872
2.324523	GeneID:105607210
13.55023	GeneID:101123297
45.47912	GeneID:101116595
20.95012	GeneID:101108568
21.15583	GeneID:101110363
5.402891	GeneID:101106207

2.987127	GeneID:101110733
58.46185	GeneID:101104367
10.74	GeneID:101120525
0.028595	GeneID:101114923
0.184846	GeneID:101123181
0.052527	GeneID:101118716
0.042854	GeneID:101123479
1.607215	GeneID:101107879
30.77435	GeneID:101112163
56.52875	GeneID:101122468
18.14945	GeneID:100307034
7.078808	GeneID:101120698
22.27486	GeneID:101107116
8.509947	GeneID:101104433
5.437919	GeneID:101120116
0.117277	GeneID:101121543
0.384108	GeneID:105605935
8.46744	GeneID:101117901
4.564642	GeneID:101120385
5.065736	GeneID:101113634
16.36443	GeneID:101113659
218.2681	GeneID:101114353
0.046499	GeneID:101117454
0.147742	GeneID:101123422
0.025352	GeneID:100233168
0	GeneID:101103917
2.160343	GeneID:101119885
1.049321	GeneID:100422799
1.177216	GeneID:100422801
30.32967	GeneID:101116575
12.65383	GeneID:101101938
21.23491	GeneID:101106630
5.246397	GeneID:101120519
2.804949	GeneID:101103558
0	GeneID:101110555
0.011839	GeneID:101105933
0	GeneID:101122724
1.74158	GeneID:101110018
32.55701	GeneID:101118022
21.02867	GeneID:101109555
17.35711	GeneID:101106971
7.723434	GeneID:101115910
15.65309	GeneID:101101928
3.970831	GeneID:101103979
10.42488	GeneID:101113196
352.31	GeneID:101113885
33.06867	GeneID:101112859
189.5654	GeneID:100217400
20.27366	GeneID:100620047
35.04806	GeneID:100620049
0	GeneID:101107134
1.558964	GeneID:101105725
0.11177	GeneID:101120975
0.021534	GeneID:101107826
0.008336	GeneID:100820745
1.360519	GeneID:101105155
7.689406	GeneID:101101939
8.063913	GeneID:101117404

7.631925	GeneID:101108313
32.0231	GeneID:101113551
8.451022	GeneID:101115900
19.14792	GeneID:101112944
71.55973	GeneID:101105991
0.291775	GeneID:101121685
13.76217	GeneID:101107825
103.9025	GeneID:101105056
65.79113	GeneID:101104821
0	GeneID:101121181
0.839383	GeneID:105616325
0.049632	GeneID:101110426
0.185628	GeneID:106991400
0	GeneID:106990625
37.672	GeneID:101114201
0	GeneID:101112334
0.451075	GeneID:101115140
2.763399	GeneID:101107206
0	GeneID:101109192
0.038088	GeneID:101119485
7.370164	GeneID:101115384
4.427973	GeneID:101113345
0.031826	GeneID:105611241
0.030565	GeneID:101117949
0.325789	GeneID:101107976
2.826344	GeneID:101109467
15.69191	GeneID:101105767
21.931	GeneID:101118803
0.27668	GeneID:101116074
0.989336	GeneID:101106538
0	GeneID:105606459
17.92186	GeneID:101123480
31.54789	GeneID:101107718
5.029639	GeneID:105611350
2.883409	GeneID:101121281
0	GeneID:101102579
0.043089	GeneID:101113873
3.938237	GeneID:101112247
0.535967	GeneID:101106705
0.107641	GeneID:105616788
11.59776	GeneID:101121189
0.109922	GeneID:101108769
0.122919	GeneID:101120516
14.43852	GeneID:101110530
3.310554	GeneID:106991551
1.998807	GeneID:114117867
0.058436	GeneID:101104264
2.519186	GeneID:101106870
0.138456	GeneID:101111738
7.834675	GeneID:101110439
1.824362	GeneID:101118295
3.836876	GeneID:105616911
1.549898	GeneID:101120771
0	GeneID:105602077
0	GeneID:101118554
0	GeneID:114117758
0.160817	GeneID:101109995
0	GeneID:101106876

8.086216	GeneID:101118987
3.852781	GeneID:101108160
0	GeneID:101106880
46.08539	GeneID:101115649
0.102199	GeneID:101105102
6.493092	GeneID:101114723
25.57108	GeneID:101107893
0.035874	GeneID:101106620
0	GeneID:101107987
0.040468	GeneID:101102420
0.74121	GeneID:101117191
18.70515	GeneID:101115904
0	GeneID:101103419
7.284604	GeneID:101115572
2.651885	GeneID:101122307
0.247613	GeneID:101105949
17.53684	GeneID:101116604
0	GeneID:105602442
7.066087	GeneID:101120605
15.21239	GeneID:101110621
3.580086	GeneID:101118220
0.246829	GeneID:101109829
0	GeneID:101120196
14.27613	GeneID:105612811
2.980218	GeneID:101119077
0	GeneID:105602819
6.270294	GeneID:101106969
0.185791	GeneID:105602732
0.249012	GeneID:105602946
1.523636	GeneID:105603239
3.720506	GeneID:101104937
5.73893	GeneID:101106884
11.10071	GeneID:101111052
0.895609	GeneID:101120445
0.368183	GeneID:101119764
0.053598	GeneID:101109126
0.112965	GeneID:114109347
0	GeneID:105603619
0	GeneID:101119763
1.4878	GeneID:101105446
0.550942	GeneID:101107905
0.033691	GeneID:114109327
0.062591	GeneID:101119258
8.795555	GeneID:101111532
5.31088	GeneID:101120567
14.31604	GeneID:101114753
5.050057	GeneID:101115074
6.547355	GeneID:101116264
0	GeneID:101114223
0.029067	GeneID:101103684
1.574026	GeneID:101114830
0.087744	GeneID:101109658
1.985141	GeneID:101109753
0.73228	GeneID:101102854
2.323896	GeneID:101109222
3.656409	GeneID:101116275
29.74665	GeneID:101111141
5.833335	GeneID:101106120

5.461672	GeneID:101112861
11.13539	GeneID:101103855
0.084161	GeneID:101110552
4.322362	GeneID:101120885
0.013217	GeneID:101105197
0	GeneID:101106635
0.254257	GeneID:105606174
0.728954	GeneID:101122997
23.59577	GeneID:101116190
2.341818	GeneID:101121061
14.16176	GeneID:101122486
17.586	GeneID:101114237
1.817691	GeneID:101118154
0.038011	GeneID:101106815
0.25756	GeneID:114113704
15.48756	GeneID:101102126
68.7175	GeneID:101106021
14.70294	GeneID:101123189
0.015683	GeneID:101118714
0	GeneID:101118254
0.337465	GeneID:101118208
0	GeneID:101118941
9.753873	GeneID:101122202
12.07113	GeneID:101105516
3.34116	GeneID:101115622
1.989148	GeneID:101113100
0	GeneID:106991628
4.098965	GeneID:105602415
0.595345	GeneID:101102801
6.188049	GeneID:101105159
0.201042	GeneID:101116326
57.0389	GeneID:101107430
1.119453	GeneID:101107516
63.42517	GeneID:101107688
4.841182	GeneID:101115321
25.34989	GeneID:101120449
28.19238	GeneID:101110098
3.426983	GeneID:101107907
4.711859	GeneID:101117798
2.396064	GeneID:101106118
0	GeneID:101123243
0.136779	GeneID:105603925
21.43606	GeneID:114109734
11.1488	GeneID:101120363
6.29467	GeneID:101117119
51.0765	GeneID:101107053
15.90455	GeneID:101122225
7.840159	GeneID:101122064
9.293232	GeneID:101118909
13.51231	GeneID:101111405
5.197652	GeneID:101106982
2.889609	GeneID:101114571
21.57358	GeneID:101112432
2.655705	GeneID:105606169
0.13892	GeneID:105604361
0.116388	GeneID:101120621
0.024628	GeneID:101116691
6.049394	GeneID:101109579

7.957918	GeneID:101112104
33.55366	GeneID:105604515
1.129382	GeneID:101119778
0	GeneID:101111765
2.333717	GeneID:101110475
1.651425	GeneID:101102448
4.508859	GeneID:101107249
0.368511	GeneID:101103526
37.12259	GeneID:101106302
0.71061	GeneID:101104548
0	GeneID:101121056
23.75804	GeneID:105604846
0.007299	GeneID:101102778
11.60723	GeneID:101106388
5.245195	GeneID:105604881
0	GeneID:101105209
0	GeneID:114110781
0.149488	GeneID:101117301
0.165822	GeneID:101110297
21.24725	GeneID:101113388
7.557085	GeneID:101113197
0.664833	GeneID:101122920
15.42093	GeneID:101120722
10.47565	GeneID:101107467
4.185547	GeneID:101105438
0.558111	GeneID:101102490
0.066903	GeneID:101107443
0.073604	GeneID:101119360
9.853277	GeneID:101116387
0	GeneID:101108809
0	GeneID:114112906
2.255239	GeneID:101102877
0.96648	GeneID:101121755
9.38866	GeneID:105609916
0	GeneID:101120472
0.407042	GeneID:101111429
11.35409	GeneID:101121833
0	GeneID:101117647
0	GeneID:101109944
0.026225	GeneID:101105471
6.581498	GeneID:101113984
0.013627	GeneID:101122498
5.328	GeneID:101114086
0.263576	GeneID:101105879
5.520957	GeneID:101118163
1.963641	GeneID:101101954
1.106825	GeneID:101104226
12.14496	GeneID:101105232
19.92631	GeneID:100528013
5.196865	GeneID:101116469
0.0186	GeneID:101111707
3.730303	GeneID:101102805
0	GeneID:101109258
0.617988	GeneID:101111356
38.88587	GeneID:101108904
0.175352	GeneID:101117662
2.143207	GeneID:101111620
11.43245	GeneID:101111954

6.775778	GeneID:101103059
3.557295	GeneID:101103144
32.17013	GeneID:101114506
1.831039	GeneID:101106066
6.833663	GeneID:101123107
19.18092	GeneID:101103387
0.088295	GeneID:101102634
0.048646	GeneID:101120740
0	GeneID:101105404
0.037142	GeneID:101116894
0	GeneID:114113614
18.5127	GeneID:101109255
101.7999	GeneID:101121160
0.019601	GeneID:101104898
42.46917	GeneID:101103145
0.055401	GeneID:101119740
0.068364	GeneID:101114204
5.64377	GeneID:101120235
0.386383	GeneID:101107694
8.516856	GeneID:101108217
2.99133	GeneID:101118296
5.86514	GeneID:101118552
14.59715	GeneID:101114192
8.989812	GeneID:101118693
0.137299	GeneID:101115789
2.629356	GeneID:101117416
55.68716	GeneID:101102725
0.448746	GeneID:101120162
10.84135	GeneID:101111277
0.273077	GeneID:101123552
0.550731	GeneID:101116824
12.06876	GeneID:101122859
23.235	GeneID:101123295
18.05496	GeneID:101104063
4.488038	GeneID:101114866
3.868953	GeneID:101115873
9.569415	GeneID:101106424
0.401225	GeneID:101102674
0	GeneID:101111454
0.026731	GeneID:101110592
83.07986	GeneID:101115708
0.44924	GeneID:101111714
0.375546	GeneID:101103156
0.005105	GeneID:105607318
0.035233	GeneID:105612764
9.157302	GeneID:101102931
17.60917	GeneID:101122441
2.294292	GeneID:101101893
2.319445	GeneID:101104494
1.473801	GeneID:101114276
0.668496	GeneID:101103816
25.10824	GeneID:101110513
1.045683	GeneID:105610829
0	GeneID:101102273
0	GeneID:101102522
0.160034	GeneID:114113848
0.008717	GeneID:101114026
25.69187	GeneID:105613054

0	GeneID:101114025
1.677408	GeneID:101119482
0	GeneID:101121610
0.235873	GeneID:101105026
11.29558	GeneID:101105761
0.096459	GeneID:101114711
4.86582	GeneID:101108579
76.42526	GeneID:101118453
4.059852	GeneID:101108578
2.272374	GeneID:101118030
0.172949	GeneID:101114531
0.73336	GeneID:101106597
13.72774	GeneID:101111774
0	GeneID:114116405;GeneID:443309
1.018815	GeneID:443426
13.22507	GeneID:101111288
14.97913	GeneID:101113860
0.009124	GeneID:101103199
30.55712	GeneID:101113341
0.040084	GeneID:101118196
2.252452	GeneID:101109718
0.052402	GeneID:101106195
0.64925	GeneID:101117861
0	GeneID:101104929
0.103172	GeneID:101110343
0.151077	GeneID:101115682
11.60658	GeneID:101109512
53.59066	GeneID:101106309
4.440695	GeneID:101115803
4.426686	GeneID:101121486
5.185177	GeneID:101112167
5.693123	GeneID:101120977
3.690372	GeneID:101121191
0.135413	GeneID:101112516
0	GeneID:101120205
0.328238	GeneID:101120597
0.010124	GeneID:101122220
1.028767	GeneID:101119268
3.404679	GeneID:101111957
10.2564	GeneID:101110191
0.223103	GeneID:101120920
0.002963	GeneID:101122774
0.725894	GeneID:101116562
0.407196	GeneID:101106371
0.114516	GeneID:101102503
0.047235	GeneID:101104552
0.329384	GeneID:101117004
1.348408	GeneID:101119120
0.007304	GeneID:101123461
0.561606	GeneID:101108767
2.13022	GeneID:101123550
0.335997	GeneID:101115165
1.535038	GeneID:101105365
0	GeneID:101116306
1.759919	GeneID:101122960
0.526046	GeneID:101112498
4.719775	GeneID:101104978
0.559357	GeneID:101117907

0	GeneID:101110606
0	GeneID:101105970
0.477701	GeneID:101110347
0.446606	GeneID:105616470
0	GeneID:101118891
0.264817	GeneID:101116675
12.60984	GeneID:101115790
9.189948	GeneID:101105965
33.99833	GeneID:101116245
5.032526	GeneID:101115776
15.44335	GeneID:101115479
0.016377	GeneID:101120371
3.614289	GeneID:101121565
4.88175	GeneID:101107547
0.051949	GeneID:101107141
0.782388	GeneID:101119428
0.033085	GeneID:101114786
0	GeneID:101105843
1.49933	GeneID:100294596
49.08848	GeneID:101122936
35.16845	GeneID:101114034
0.031835	GeneID:101113579
4.097137	GeneID:101115511
171.9917	GeneID:101121342
0.027214	GeneID:101112364
9.676357	GeneID:101112099
0.041573	GeneID:101112615
12.20438	GeneID:101105416
18.75131	GeneID:105612589
133.5441	GeneID:101115216
495.3952	GeneID:443230
64.57817	GeneID:101116338
4.484282	GeneID:100037684
0.408514	GeneID:101123572
0.091492	GeneID:101111598
183.0197	GeneID:101104396
0.125741	GeneID:101115872
16.34021	GeneID:101111450
0.029648	GeneID:101118913
2.96586	GeneID:101103762
8.012304	GeneID:101108939
0.287618	GeneID:101105428
0.052669	GeneID:443453
0.437777	GeneID:101118870
12.02645	GeneID:101113850
9.540462	GeneID:101108884
13.11478	GeneID:101102884
0.07979	GeneID:106990878
0.639036	GeneID:101108308
3.460326	GeneID:101102157
8.37367	GeneID:101103267
2.837487	GeneID:101116304
0.00908	GeneID:101123415
13.6682	GeneID:101119046
8.304215	GeneID:101118095
21.13964	GeneID:101107980
25.06786	GeneID:101123375
1.64529	GeneID:101115890

10.5529	GeneID:101108500
14.20792	GeneID:101107775
0.777929	GeneID:101107312
5.909048	GeneID:101122609
117.8479	GeneID:101122273
102.5092	GeneID:101120787
1.425041	GeneID:101102097
50.974	GeneID:101101968
35.74885	GeneID:443130
5.3718	GeneID:101104198
0.010998	GeneID:101113966
0.024386	GeneID:101104136
0.013627	GeneID:101104645
1.684869	GeneID:101123435
56.55964	GeneID:100127211
0.035835	GeneID:443038
4.278766	GeneID:101113269
0.943932	GeneID:101110122
10.29456	GeneID:101112192
4.857024	GeneID:101103336
127.209	GeneID:678676
43.09298	GeneID:101115561
85.18171	GeneID:101113623
3.155831	GeneID:101109694
0.975445	GeneID:101107783
0.006142	GeneID:101123367
0.418638	GeneID:101110872
44.31176	GeneID:101117461
35.10777	GeneID:101109168
0	GeneID:101122590
71.66713	GeneID:101108893
2.682781	GeneID:101108123
1.043423	GeneID:101110813
1.204813	GeneID:101120931
5.613005	GeneID:101107161
5.008306	GeneID:101103184
0.811644	GeneID:101116560
1.752123	GeneID:101112293
32.83796	GeneID:101106225
16.12265	GeneID:101119559
14.18662	GeneID:101116184
0.402659	GeneID:101108771
0.264159	GeneID:101120924
5.019592	GeneID:101122760
0.014236	GeneID:101109312
51.26226	GeneID:101119091
10.72802	GeneID:101116327
0.029555	GeneID:101109912
0.832948	GeneID:101114856
25.64752	GeneID:101107033
12.67919	GeneID:101114863
10.6319	GeneID:101102544
0.332157	GeneID:101114411
9.609435	GeneID:101120933
0.049327	GeneID:101103049
29.16315	GeneID:101120827
10.10149	GeneID:101115025
4.004298	GeneID:443031

6.567087	GeneID:114108735
2.861784	GeneID:101122741
6.019259	GeneID:101107334
4.757137	GeneID:101109100
4.603132	GeneID:101110953
0.055554	GeneID:101118652
4.199057	GeneID:101105292
0.028612	GeneID:101112527
0.454185	GeneID:101105178
164.5187	GeneID:443364
8.891141	GeneID:101122727
6.055457	GeneID:101112277
25.4948	GeneID:101112837
60.24422	GeneID:100307035
0.244012	GeneID:443311
0	GeneID:101111003
0.087093	GeneID:101103937
0.520243	GeneID:101109215
0.042773	GeneID:101104315
1.545407	GeneID:101116549
0.042129	GeneID:101112425
0.285101	GeneID:101111283
0.319161	GeneID:101107374
0.287484	GeneID:101111312
0.178574	GeneID:105604107
140.1011	GeneID:445468
12.25046	GeneID:100126571
0.282791	GeneID:101107228
90.16925	GeneID:101110525
11.89501	GeneID:101118507
34.58001	GeneID:101110967
0.907789	GeneID:101122919
3.292337	GeneID:101113676
3.947638	GeneID:101110436
2.564712	GeneID:101109116
32.90697	GeneID:101106455
7.731787	GeneID:101115401
3.676075	GeneID:101111043
0	GeneID:101111743
6.113925	GeneID:101104902
0	GeneID:101111385
0	GeneID:101112017
0.251857	GeneID:101112982
0.829876	GeneID:114117528
6.885531	GeneID:101122167
36.95083	GeneID:101106301
28.36355	GeneID:101116158
5.922668	GeneID:101111469
33.3819	GeneID:101106931
12.3848	GeneID:101110952
19.87063	GeneID:100307036
2.993329	GeneID:101122432
5.194597	GeneID:101122687
3.367916	GeneID:101111208
20.44942	GeneID:101105234
7.255801	GeneID:101119902
13.75571	GeneID:101114826
2.529831	GeneID:101105915

3.64197	GeneID:101107489
21.16678	GeneID:101106390
38.00516	GeneID:101114492
4.817057	GeneID:101117553
3.998362	GeneID:101120432
0.170227	GeneID:101103523
0.395775	GeneID:101117001
0	GeneID:101103234
6.767797	GeneID:101117695
12.13038	GeneID:101116205
3.988334	GeneID:101123303
0	GeneID:101104419
0.23819	GeneID:101105683
11.07651	GeneID:101103950
0.023819	GeneID:101116259
18.64606	GeneID:101106715
14.50873	GeneID:101115164
4.526077	GeneID:101113309
1.955304	GeneID:101112804
0	GeneID:101102572
35.85789	GeneID:101110586
1.617365	GeneID:101119672
2.839497	GeneID:101110932
6.068968	GeneID:101102759
0.131132	GeneID:101117705
5.517047	GeneID:101102978
3.429904	GeneID:101112803
0.251546	GeneID:101112476
10.56919	GeneID:101117002
4.016318	GeneID:101109087
2.406537	GeneID:101120380
0.271199	GeneID:101122576
1.448591	GeneID:101106927
0.813861	GeneID:101118014
0.129754	GeneID:101112454
6.095832	GeneID:101104578
1.90731	GeneID:101117460
0.565073	GeneID:101123103
0.010972	GeneID:101119302
0	GeneID:101116092
0.059928	GeneID:101115156
0.249737	GeneID:101107406
10.4462	GeneID:101105594
0.26115	GeneID:101123238
1.873126	GeneID:101109802
0.379045	GeneID:101120327
0.113116	GeneID:101108991
0.12784	GeneID:101108397
1.265051	GeneID:101111334
0.248074	GeneID:101112490
13.28856	GeneID:101123505
0.005875	GeneID:114116677
2.473604	GeneID:101111597
0.981914	GeneID:101105760
0.550422	GeneID:101122165
0.042871	GeneID:101103108
0.024355	GeneID:101119534
10.85497	GeneID:101104788

0.055713	GeneID:101117082
0.040295	GeneID:101119221
0	GeneID:101117383
0	GeneID:106990164
0.993888	GeneID:101117639
0.243969	GeneID:101104813
0.090984	GeneID:101123136
0.610976	GeneID:101107853
19.52639	GeneID:101101940
0.451708	GeneID:105607222
0.066586	GeneID:105612806
0.160376	GeneID:101102198
0.106824	GeneID:101110285
1.094642	GeneID:101116278
0.026238	GeneID:105608687
0.127737	GeneID:114114766
0	GeneID:101111970
0	GeneID:101114311
6.838552	GeneID:101104125
4.247366	GeneID:101107922
11.69385	GeneID:101114166
5.231805	GeneID:101122529
14.73525	GeneID:101105986
34.75778	GeneID:101109471
0.523994	GeneID:101102114
19.00293	GeneID:101108835
0.011339	GeneID:101120277
4.954818	GeneID:101116505
0.073868	GeneID:101105694
0.010913	GeneID:101118778
0.087089	GeneID:101105124
0.1151	GeneID:101110430
0	GeneID:101109283
19.8219	GeneID:101117433
32.4953	GeneID:101103333
8.448747	GeneID:101104368
2.839458	GeneID:101111750
1.289288	GeneID:101106275
17.40445	GeneID:101113031
13.3984	GeneID:101106509
18.82317	GeneID:101120801
0.011605	GeneID:101106285
2.655665	GeneID:101120433
2.417836	GeneID:101123584
0	GeneID:101106546
0.02934	GeneID:101104220
4.23324	GeneID:101104693
1.215496	GeneID:101120290
0.454012	GeneID:101111284
0.315077	GeneID:101111568
0	GeneID:101120419
7.891983	GeneID:101105954
0.840274	GeneID:114114512
0	GeneID:101122132
0.286891	GeneID:105602964
5.322634	GeneID:101120068
0.012529	GeneID:101104626
14.04981	GeneID:101116079

46.22437	GeneID:101122067
0.008176	GeneID:101113447
4.951498	GeneID:101123145
0.016529	GeneID:101103433
6.128514	GeneID:101104854
0.499706	GeneID:101120398
9.852365	GeneID:101103437
13.09591	GeneID:105603308
11.63966	GeneID:101121127
0.035418	GeneID:101106215
3.275289	GeneID:101122010
0.215937	GeneID:101116874
2.099717	GeneID:101112005
1.597779	GeneID:101104101
15.87447	GeneID:114118013
14.88041	GeneID:101105956
6.631818	GeneID:101109260
2.870493	GeneID:101116427
5.525303	GeneID:101110305
1.313974	GeneID:114115352
13.3069	GeneID:101102506
3.154701	GeneID:101113337
0	GeneID:106990133
2.127258	GeneID:101113431
5.771643	GeneID:101104535
0.132157	GeneID:101110996
0.065579	GeneID:101116683
0.023849	GeneID:101108047
0.095884	GeneID:101118381
0.134284	GeneID:101114628
67.51679	GeneID:101115044
0.035789	GeneID:101115290
2.755368	GeneID:101120217
0.611639	GeneID:101104302
71.47232	GeneID:101119957
0.730458	GeneID:101123141
4.433534	GeneID:101117556
0.452829	GeneID:678679
30.36774	GeneID:101117297
0.512109	GeneID:780500
3.755464	GeneID:101115553
13.60505	GeneID:101114100
1.001566	GeneID:101112919
17.1764	GeneID:100037673
49.6526	GeneID:100303607
13.16916	GeneID:101113859;GeneID:114116377
0.942759	GeneID:101120418
7.24268	GeneID:101106613
0.223185	GeneID:101109099
0.322511	GeneID:101105579
9.842427	GeneID:100144758
13.17193	GeneID:101120694
0.175699	GeneID:101115966
5.537797	GeneID:101115712
3.166245	GeneID:101102288
10.69683	GeneID:101102491
35.61223	GeneID:100144763
54.77797	GeneID:100147799

15.84166	GeneID:100147798
25.16507	GeneID:101120086
1.251333	GeneID:101107124
2.88013	GeneID:101109811
2.517597	GeneID:101116197
134.3337	GeneID:101119213
14.26445	GeneID:101110594
12.1413	GeneID:101104905
147.1264	GeneID:101110333
0.480956	GeneID:105611485
7.054756	GeneID:101110720
0.284027	GeneID:101115368
10.11315	GeneID:101117113
23.38546	GeneID:101109758
18.39968	GeneID:101106347
0.04644	GeneID:101115826
5.941759	GeneID:101111859
7.661114	GeneID:101106415
8.906737	GeneID:101106913
45.117	GeneID:101122455
11.65517	GeneID:101118334
7.492122	GeneID:101117720
36.77567	GeneID:105601860
0.076124	GeneID:678671
1.553271	GeneID:443111
0.354762	GeneID:101105364
1.171002	GeneID:101117453
0.652397	GeneID:101115284
0.093782	GeneID:101109462
0.585494	GeneID:101113281
0	GeneID:678667
1.933675	GeneID:101117199
7.573778	GeneID:100846985
13.1648	GeneID:101101863
0.34741	GeneID:101117333
8.996232	GeneID:101110815
95.52065	GeneID:101102222
155.0611	GeneID:101103858
87.60348	GeneID:101119712
89.2223	GeneID:101122217
40.27801	GeneID:101108445
0.092957	GeneID:101118966
162.6018	GeneID:101112301
85.30626	GeneID:101108792
0	GeneID:101115871
33.63055	GeneID:101115090
0.020802	GeneID:100144425
0.392682	GeneID:101106981
14.14888	GeneID:101117513
14.73684	GeneID:443216
56.86926	GeneID:105604195
81.44391	GeneID:101114873
3.682478	GeneID:101113212
0	GeneID:101107126
0.208251	GeneID:101122054
0.029233	GeneID:101111510
7.537689	GeneID:100144424
1.932815	GeneID:101110634

2.456335	GeneID:101106727
2.329517	GeneID:101120715
2.09625	GeneID:101122319
1.544273	GeneID:101107427
0.619191	GeneID:101102835
0.26514	GeneID:101112529
100.662	GeneID:101119731
0.550647	GeneID:101121900
7.969795	GeneID:443436
11.7929	GeneID:101105455
0.402098	GeneID:101114260
0.594657	GeneID:101115003
5.056309	GeneID:101111370
0.29873	GeneID:443480
32.40304	GeneID:101117542
25.07397	GeneID:101120718
0.940929	GeneID:101112832
0.255308	GeneID:101103409
0.423549	GeneID:101104164
0.092196	GeneID:101105174
0.490355	GeneID:105606306
8.186171	GeneID:101114629
33.63978	GeneID:114108631
4.417692	GeneID:101102807
60.74473	GeneID:101105675
2.994257	GeneID:101110864
0.163985	GeneID:105614340
3.384651	GeneID:443397
7.23902	GeneID:443437
6.883523	GeneID:101113091
4.012305	GeneID:443398
0.258799	GeneID:443509
1.367281	GeneID:443232
0.787359	GeneID:767628
75.49428	GeneID:101113884
28.95102	GeneID:101111153
1.363536	GeneID:443328
8.264921	GeneID:101112271
10.02628	GeneID:101120257
19.27401	GeneID:101106474
97.20623	GeneID:101102670
1.557969	GeneID:101122317
113.3695	GeneID:101114944
9.909636	GeneID:101102500
0.050088	GeneID:443215
1.006827	GeneID:101111110
0	GeneID:101105074
0.201186	GeneID:101108894
178.2968	GeneID:101102561
1.131353	GeneID:101105681
0.134917	GeneID:101104586
0.346759	GeneID:554317
217.2465	GeneID:100147790
49.01891	GeneID:101111121
1.049346	GeneID:101103355
1.878948	GeneID:101102779
1.338203	GeneID:554318
0.237505	GeneID:101103464

0.711776	GeneID:101103712
383.4385	GeneID:100141299
6.177131	GeneID:100302099
0.709383	GeneID:101117717
63.41545	GeneID:101115513
12.22751	GeneID:101107422
0.360804	GeneID:101102378
6.314913	GeneID:101115550
3.105215	GeneID:101118193
44.28813	GeneID:101117525
6.31685	GeneID:101121483
4.537744	GeneID:101110906
12.70841	GeneID:101117773
13.67002	GeneID:101110548
0.013311	GeneID:101116347
8.107806	GeneID:101109612
1.89372	GeneID:101113178
13.28941	GeneID:101120511
0.53182	GeneID:101110327
25.08737	GeneID:101108105
9.439303	GeneID:101121276
50.7754	GeneID:101107962
43.80443	GeneID:101113585
10.07303	GeneID:101112951
10.77957	GeneID:101112230
51.40937	GeneID:101102792
8.165958	GeneID:101114291
20.70568	GeneID:101122467
24.57472	GeneID:101118650
34.72102	GeneID:101122773
5.476199	GeneID:101122234
41.41364	GeneID:101120399
7.868417	GeneID:101107208
1.676339	GeneID:101106030
69.64085	GeneID:101110804
12.81184	GeneID:101103563
3.039293	GeneID:101121376
15.0629	GeneID:101118824
3.171594	GeneID:101107536
5.115219	GeneID:101102190
17.73356	GeneID:101118545
2.65072	GeneID:101111159
12.89705	GeneID:101110222
4.063036	GeneID:101120206
12.39303	GeneID:101111162
19.00159	GeneID:101108042
9.097637	GeneID:101115324
9.19955	GeneID:101105034
0.105717	GeneID:101117722
115.1856	GeneID:101119747
28.25668	GeneID:101106961
0.01501	GeneID:101118385
23.73422	GeneID:101123062
0.030428	GeneID:101109639
0.03652	GeneID:101111295
0	GeneID:101119421
0.926886	GeneID:101113295
1.371377	GeneID:100170321

0.110846	GeneID:101118069
0.011284	GeneID:101114377
1.019768	GeneID:101107570
4.743302	GeneID:101123215
0	GeneID:101122970
30.0019	GeneID:101111652
0.45191	GeneID:101122716
8.867641	GeneID:101107462
0.135698	GeneID:101116091
0.168903	GeneID:101104029
0.235876	GeneID:101106707
0	GeneID:101117108
0.037942	GeneID:101113303
4.048902	GeneID:101118269
0.03473	GeneID:101104990
0.098458	GeneID:101102094
0.150793	GeneID:101103359
3.552982	GeneID:101102693
11.07346	GeneID:101119178
10.77536	GeneID:100216432
6.361832	GeneID:101107722
7.126865	GeneID:101102654
11.08	GeneID:101119901
5.059397	GeneID:101109265
0	GeneID:101109860
7.914697	GeneID:101116802
7.277615	GeneID:101122426
0.05215	GeneID:105606089
17.4261	GeneID:101111460
8.05749	GeneID:100216433
3.428788	GeneID:101102036
25.36585	GeneID:101122554
22.03833	GeneID:101111140
1.453313	GeneID:101107113
46.48468	GeneID:100144756
20.09042	GeneID:100190973
1.038344	GeneID:101120847
0.012104	GeneID:101112037
0.074149	GeneID:101109200
5.69668	GeneID:101116809
16.08627	GeneID:101116665
8.537696	GeneID:100144761
9.576433	GeneID:101119934
50.14957	GeneID:101109788
7.089811	GeneID:101121212
1.69927	GeneID:101103157
1.774771	GeneID:101106773
0.019012	GeneID:101102479
0.54808	GeneID:101117576
1.723536	GeneID:101108546
98.0139	GeneID:100302083
32.7459	GeneID:100127219
14.00974	GeneID:100216434
19.14778	GeneID:101108190
48.47127	GeneID:100135688
33.71954	GeneID:101107164
7.908428	GeneID:101109224
3.327969	GeneID:101118528

3.353868	GeneID:101116234
7.875356	GeneID:101108409
32.35855	GeneID:101102489
9.887126	GeneID:101113286
22.58446	GeneID:101120820
0.145561	GeneID:114111662
7.003131	GeneID:101115091
2.490542	GeneID:101111563
0.126874	GeneID:105611616
0.059832	GeneID:101116067
40.61795	GeneID:101110414
8.318227	GeneID:101114631
265.5618	GeneID:101109128
2.337236	GeneID:101108074
3.888414	GeneID:101115093
0.01777	GeneID:101123037
0.016866	GeneID:101112831
0	GeneID:101111777
16.03812	GeneID:101120964
1.351993	GeneID:101119749
1.274791	GeneID:101110784
0	GeneID:105602081
11.52798	GeneID:101111222
0.677215	GeneID:101110170
30.65121	GeneID:101118553
40.91626	GeneID:443277
26.15158	GeneID:114116361
0.027109	GeneID:101122954
23.29406	GeneID:101110612
24.26547	GeneID:101121334
7.18698	GeneID:106990321
0	GeneID:101112299
0.480557	GeneID:101114258
0	GeneID:101103454
8.428227	GeneID:101118301
2.402694	GeneID:101107886
0.036591	GeneID:101118582
0.058888	GeneID:101114837
0.028031	GeneID:101118445
0.44897	GeneID:101115798
10.27212	GeneID:101111090
15.37728	GeneID:101103606
0.01435	GeneID:101118998
0.690122	GeneID:101116178
8.978934	GeneID:101102701
0	GeneID:105613317
10.29202	GeneID:101110750
28.9762	GeneID:443351
1.113909	GeneID:101113004
1.847554	GeneID:101105752
7.809275	GeneID:101112149
2.512458	GeneID:101120440
3.384235	GeneID:101104558
2.461764	GeneID:101119996
2.37151	GeneID:101103592
4.834161	GeneID:101113689
1.957757	GeneID:101113226
5.144869	GeneID:101121445

8.472301	GeneID:101107680
2.235727	GeneID:101109147
3.943567	GeneID:101123157
6.42382	GeneID:101114968
1.778647	GeneID:101115894
8.221421	GeneID:101108985
5.105084	GeneID:105611619
4.581313	GeneID:101117252
10.76935	GeneID:101107297
8.937859	GeneID:101102160
4.629798	GeneID:101117261
10.10626	GeneID:101110325
0.494597	GeneID:101122133
0.77686	GeneID:101116835
3.575414	GeneID:101119062
0.864866	GeneID:101108311
1.361307	GeneID:101109362
6.147855	GeneID:101117339
3.363642	GeneID:101112846
3.725124	GeneID:101119149
16.87435	GeneID:105603316
6.755188	GeneID:101123256
4.719859	GeneID:101107490
2.952885	GeneID:101120102
3.389111	GeneID:101109526
11.86423	GeneID:101106980
0.047828	GeneID:101122872
7.303568	GeneID:101122613
5.121993	GeneID:101117581
4.518547	GeneID:101120139
1.846949	GeneID:101104542
10.09605	GeneID:101102008
5.462576	GeneID:101115715
4.375896	GeneID:101114919
5.24325	GeneID:101117660
3.330364	GeneID:101111770
2.617423	GeneID:101109828
4.503903	GeneID:106990433
4.80522	GeneID:101112035
3.198882	GeneID:101115779
2.625699	GeneID:101112118
0.666675	GeneID:101103576
3.677134	GeneID:101109823
9.58307	GeneID:101106443
2.386279	GeneID:101112438
17.22516	GeneID:101112775
0	GeneID:101122006
4.252568	GeneID:101107949
16.21136	GeneID:101111625
0.083331	GeneID:101123449
2.29647	GeneID:101107095
76.6923	GeneID:101109498
6.276731	GeneID:101110093
17.01758	GeneID:101102559
44.80281	GeneID:101117661
1.209491	GeneID:101122252
1.626108	GeneID:101109730
2.627804	GeneID:101105682

38.89532	GeneID:101109991
0	GeneID:780451
17.79896	GeneID:100302545
30.03615	GeneID:101106324
0.075143	GeneID:101117883
1.048923	GeneID:101106219
0	GeneID:101116681
17.88041	GeneID:101104170
0.36136	GeneID:101113597
0.01011	GeneID:101112377
16.4848	GeneID:101102107
0.145485	GeneID:101113333
0.451641	GeneID:101121882
47.14413	GeneID:101121587
5.195121	GeneID:101108011
0.031345	GeneID:101114833
0.093814	GeneID:101115253
0.516018	GeneID:101116271
0.250394	GeneID:101110287
0.012305	GeneID:101110523
0.129979	GeneID:101119811
0.005123	GeneID:101106130
0.168335	GeneID:101116527
0.17498	GeneID:101123318
0	GeneID:101112547
2.258926	GeneID:101112217
0.23977	GeneID:101118160
0	GeneID:101112598
0	GeneID:101122804
1.330168	GeneID:101120156
6.953319	GeneID:101111772
0	GeneID:105616252
0.038832	GeneID:101102149
5.337907	GeneID:101115072
5.158705	GeneID:101115705
0	GeneID:101122966
1.391701	GeneID:101119307
772.6294	GeneID:494432
8.738604	GeneID:101118388
4.994464	GeneID:101106914
3.422035	GeneID:101114678
0.111653	GeneID:443347
0.039797	GeneID:101117253
25.48563	GeneID:101121901
6.71263	GeneID:101117550
13.68169	GeneID:101119219
0.969526	GeneID:114108615
3.421018	GeneID:101117166
4.35052	GeneID:101121645
163.8093	GeneID:101114872
1.92569	GeneID:101104387
0.585774	GeneID:105608408
0.252927	GeneID:105614872
10.4524	GeneID:101112053
5.381127	GeneID:101116195
9.789113	GeneID:101118032
0.029862	GeneID:101117817
42.97058	GeneID:101120800

16.18886	GeneID:101110091
62.0202	GeneID:101108017
53.17434	GeneID:100145872
8.736085	GeneID:101105782
7.478121	GeneID:101113220
4.737957	GeneID:101116871
16.59967	GeneID:101116061
8.749141	GeneID:101111801
5.276598	GeneID:101113972
11.33551	GeneID:101119938
29.69463	GeneID:101119918
68.5456	GeneID:101109874
0.255685	GeneID:101118206
4.532042	GeneID:101104333
9.10068	GeneID:101111107
14.43979	GeneID:101109181
9.367039	GeneID:101114129
0.095123	GeneID:101106116
3.695067	GeneID:101111403
4.046541	GeneID:101105532
12.46072	GeneID:101117160
19.03674	GeneID:101111490
0.071198	GeneID:101114907
0.010265	GeneID:101115639
13.81216	GeneID:443279
0.135008	GeneID:101106229
0.03639	GeneID:100049063
1.030732	GeneID:101112030
7.557093	GeneID:101105753
37.08316	GeneID:100101234
1.68342	GeneID:101113448
14.03538	GeneID:101118779
14.25919	GeneID:101113017
4.181273	GeneID:101102290
7.865114	GeneID:101120764
44.08334	GeneID:101111221
47.30007	GeneID:101108355
132.1884	GeneID:101112505
26.40495	GeneID:101121224
18.29718	GeneID:114108645
92.0112	GeneID:101111216
30.004	GeneID:101119649
41.49934	GeneID:101104370
19.19213	GeneID:101110159
5.517286	GeneID:101113461
2.122094	GeneID:101109591
2.738605	GeneID:101103716
3.585467	GeneID:101115923
19.83045	GeneID:101108786
30.94565	GeneID:101112321
24.81816	GeneID:101118159
6.612079	GeneID:101114088
2.484909	GeneID:101114606
8.440115	GeneID:101108821
19.317	GeneID:101121430
1.890674	GeneID:443243
22.59777	GeneID:101113311
1.272109	GeneID:101106877

0.008816	GeneID:101117029
0.076358	GeneID:101102897
0.085491	GeneID:443189
0	GeneID:101114554
0.095339	GeneID:101110766
0	GeneID:101110120
0	GeneID:101107330
0.02175	GeneID:101123239
0	GeneID:101118889
0.218907	GeneID:101115407
0	GeneID:101119532
0.109128	GeneID:101113104
0.033195	GeneID:101115624
3.210248	GeneID:101121614
0	GeneID:101104117
0	GeneID:101122833
0.069317	GeneID:101123499
0.164393	GeneID:101102707
6.188643	GeneID:101110155
0	GeneID:101102965
9.920763	GeneID:101112159
12.22372	GeneID:101110832
0.732703	GeneID:101113667
5.516833	GeneID:101118845
0.112987	GeneID:114109348
3.314409	GeneID:101108574
2.943628	GeneID:101113976
7.389668	GeneID:101105798
3.913089	GeneID:101112877
0.012733	GeneID:101104927
1.583126	GeneID:101119189
0.444559	GeneID:101118812
0.036532	GeneID:101116693
2.446012	GeneID:101109305
0.210956	GeneID:101104485
1.899115	GeneID:101105623
35.78721	GeneID:101106216
11.0937	GeneID:101111143
52.6451	GeneID:105607024
15.50874	GeneID:101113574
32.35976	GeneID:101110334
42.44189	GeneID:101109473
7.26821	GeneID:101104879
19.09123	GeneID:101123398
6.637717	GeneID:101116794
24.20878	GeneID:100141300
26.23553	GeneID:100302744
0	GeneID:100302745
0.158014	GeneID:100303602
7.113967	GeneID:101102258
7.4838	GeneID:101108619
1.086208	GeneID:101113497
1.292394	GeneID:101102430
1.643756	GeneID:101106564
8.009488	GeneID:101119218
0.115684	GeneID:101107342
21.98501	GeneID:101103845
2.863826	GeneID:101115924

7.442901	GeneID:100171393
25.68967	GeneID:101105893
49.48504	GeneID:101117758
19.97561	GeneID:101120458
19.83362	GeneID:101106939
8.335234	GeneID:101120095
12.45338	GeneID:101119259
1.54248	GeneID:101107309
0.373885	GeneID:101109515
69.59747	GeneID:100135454
55.67214	GeneID:101122913
13.2382	GeneID:101109005
14.35719	GeneID:101121357
4.871668	GeneID:101112209
15.57092	GeneID:101114945
20.51802	GeneID:101115651
69.81506	GeneID:100190977
0.634777	GeneID:101112841
0.413941	GeneID:101122189
31.82345	GeneID:101113974
41.08233	GeneID:101103869
13.75828	GeneID:101114679
16.26136	GeneID:101106631
6.076883	GeneID:101113265
1.738641	GeneID:101105191
0	GeneID:101115328
22.96115	GeneID:101115078
0	GeneID:101115586
17.34676	GeneID:101122902
0.410849	GeneID:101108788
0	GeneID:101115453
0.409639	GeneID:100125625
12.59085	GeneID:101114498
2.618967	GeneID:100125626
2.557022	GeneID:101103039
3.615037	GeneID:101111209
11.46544	GeneID:101107658
0	GeneID:101111082
311.1986	GeneID:780473
12.86479	GeneID:101107876
1.349089	GeneID:101113649
15.16692	GeneID:101109008
1.569286	GeneID:101109068
0.133699	GeneID:101121229
0.878572	GeneID:101105382
0.168271	GeneID:780474
0	GeneID:101112106
0.083414	GeneID:101102362
0.009706	GeneID:780475
0.027979	GeneID:106990125
1.199375	GeneID:114110977
0	GeneID:101114210
3.223415	GeneID:780476
83.88102	GeneID:780477
7.498051	GeneID:101115864
0.040094	GeneID:101117294
2.878599	GeneID:100462658
29.18658	GeneID:101106811

23.44537	GeneID:101106127
0.650699	GeneID:101122378
2.204528	GeneID:101119074
0.54207	GeneID:101119973
8.84969	GeneID:101106287
12.58535	GeneID:101107063
0.049822	GeneID:101117076
0	GeneID:101112020
2.562453	GeneID:101118775
0.033611	GeneID:101119293
0.037896	GeneID:101110836
0	GeneID:101120184
72.07265	GeneID:101120024
2.498996	GeneID:101104478
0.022005	GeneID:101104735
0.023316	GeneID:100135686
0.05388	GeneID:101102383
0.439488	GeneID:101111012
0.35302	GeneID:678675
0.335483	GeneID:114108611;GeneID:678685
0.442166	GeneID:101119033
0.644229	GeneID:101115069
0.562771	GeneID:101123282
172.5076	GeneID:101118140
13.76777	GeneID:101106057
35.98613	GeneID:114113844
112.7652	GeneID:101115514
2.292988	GeneID:101119340
0.007072	GeneID:101112021
42.85241	GeneID:101113932
20.64058	GeneID:101121120
10.09482	GeneID:101110113
4.053578	GeneID:101106102
7.089527	GeneID:101102710
162.2508	GeneID:101105634
23.46945	GeneID:101118834
38.35676	GeneID:101109742
21.69364	GeneID:101104233
1.344703	GeneID:101115830
5.54485	GeneID:780518
7.354128	GeneID:101112942
12.82365	GeneID:100034668
5.560665	GeneID:678673
22.30462	GeneID:101104038
0.057836	GeneID:101115279
26.21509	GeneID:101111236
3.616284	GeneID:100171391
7.057438	GeneID:101106031
4.626223	GeneID:101121966
23.92678	GeneID:101106323
0	GeneID:101119081
0	GeneID:101122733
32.89623	GeneID:101112757
37.05108	GeneID:101112164
24.26342	GeneID:101111198
0	GeneID:101118841
0.020176	GeneID:101108133
0.0717	GeneID:101117462

3.340836	GeneID:101110630
76.30408	GeneID:101113351
0.186712	GeneID:101108443
3.661077	GeneID:101106761
129.7691	GeneID:101106572
80.05565	GeneID:101117328
69.373	GeneID:101107114
2.524374	GeneID:101123445
7.286253	GeneID:101121306
13.63362	GeneID:101105264
0.261915	GeneID:101103443
0	GeneID:101117938
0.035115	GeneID:101120248
0.919614	GeneID:101111978
19.35341	GeneID:101115615
16.08597	GeneID:101121802
5.051507	GeneID:105603337
22.1905	GeneID:101120939
7.083999	GeneID:101104636
22.40871	GeneID:101121541
5.156506	GeneID:101103843
22.05278	GeneID:101106892
3.43425	GeneID:101123366
3.838661	GeneID:101108270
0	GeneID:101107723
19.76723	GeneID:101104422
2.071562	GeneID:101104681
0.681196	GeneID:101120333
64.36949	GeneID:101112171
6.722542	GeneID:101111914
9.687442	GeneID:114109374
10.22369	GeneID:101123242
3.884449	GeneID:101106100
1.792102	GeneID:101109097
0	GeneID:101112328
217.3065	GeneID:101106550
0.679534	GeneID:101110987
89.8546	GeneID:101111244
12.00211	GeneID:101112154
2.74962	GeneID:101104084
0	GeneID:101123557
0	GeneID:101113826
0.101892	GeneID:100233164
0.0326	GeneID:101118637
0.036643	GeneID:101121447
0	GeneID:100233165
26.26195	GeneID:101116658
0.391534	GeneID:101113182
0.165358	GeneID:101110075
27.58347	GeneID:101109287
3.871201	GeneID:101112380
0.269262	GeneID:101106407
14.63428	GeneID:101105165
0.078676	GeneID:101106181
64.19975	GeneID:443517
92.22244	GeneID:101115961
126.683	GeneID:101108091
0.026235	GeneID:101118492

1.226825	GeneID:101109844
13.64821	GeneID:101114184
3.333182	GeneID:101110923
37.10421	GeneID:101105519
10.98219	GeneID:101105110
10.02416	GeneID:101103552
27.57804	GeneID:101122772
6.387899	GeneID:101107815
1.916546	GeneID:101123114
11.73171	GeneID:101120323
17.84125	GeneID:101111369
43.10859	GeneID:101120985
13.72354	GeneID:101109796
16.13858	GeneID:101111075
12.73955	GeneID:101120423
33.07152	GeneID:101112796
0.023355	GeneID:101106678
52.13759	GeneID:101108903
7.78716	GeneID:101110884
29.32687	GeneID:101107493
0.141882	GeneID:101111639
0.146253	GeneID:101119188
7.636258	GeneID:101110489
8.102734	GeneID:101115637
0	GeneID:101105930
0.094862	GeneID:101118980
0.021501	GeneID:101107894
3.31439	GeneID:100885760
0.822253	GeneID:101122503
2.728579	GeneID:101118005
0.027399	GeneID:101113348
0.059014	GeneID:101113628
0.645172	GeneID:114108678
0.055166	GeneID:101121624
0.976002	GeneID:101111565
0.164713	GeneID:101106322
0.20867	GeneID:101118808
0	GeneID:101116541
6.837637	GeneID:101115515
5.296603	GeneID:101120679
7.015221	GeneID:101102655
12.78101	GeneID:101110310
37.50513	GeneID:101102269
22.28083	GeneID:101107716
10.83929	GeneID:101122408
10.74121	GeneID:101103330
14.74767	GeneID:101113782
9.266913	GeneID:101106255
9.736388	GeneID:101122530
9.320229	GeneID:101104954
10.5453	GeneID:101113170
12.35231	GeneID:101123134
0.009178	GeneID:101106693
4.492194	GeneID:101117548
0.088101	GeneID:101116356
59.7357	GeneID:101109808
0.495666	GeneID:101105550
15.39111	GeneID:101120758

20.39703	GeneID:101102453
19.81607	GeneID:101107002
93.96186	GeneID:101116007
29.93088	GeneID:101106232
111.0552	GeneID:443483
194.4664	GeneID:443512
0.067593	GeneID:101112800
2.651927	GeneID:101106802
0.065404	GeneID:101119401
1.234813	GeneID:101108307
0.25784	GeneID:101114569
0.653236	GeneID:101118329
1.847694	GeneID:101119374
1.744656	GeneID:101115616
0.059485	GeneID:100196905
116.8218	GeneID:100101231
31.16245	GeneID:101115552
21.96911	GeneID:101115805
0.506517	GeneID:101102790
13.19376	GeneID:105613553
0.460739	GeneID:101118172
13.34808	GeneID:101112706
3.660809	GeneID:101113128
10.48776	GeneID:101107777
28.66926	GeneID:101114493
2.520848	GeneID:101115457
79.73478	GeneID:101111927
57.57009	GeneID:101106490
24.50302	GeneID:443051
0.114703	GeneID:101118494
0.034345	GeneID:101111424
3.572658	GeneID:101119261
3.410389	GeneID:101107564
8.285903	GeneID:105602841
0.314225	GeneID:101102992
0	GeneID:101121906
0.02203	GeneID:101120183
0	GeneID:101107466
0.033651	GeneID:101114116
0.065757	GeneID:101120299
21.74999	GeneID:101102949
11.00151	GeneID:101114107
0.49087	GeneID:101107209
0.398032	GeneID:101112947
29.122	GeneID:101117656
10.67822	GeneID:101116310
15.75468	GeneID:101120624
38.74308	GeneID:105601866
15.7318	GeneID:101106628
4.615231	GeneID:101116237
14.40473	GeneID:101102498
13.015	GeneID:101104771
16.39913	GeneID:101112319
18.86385	GeneID:101106458
0.126718	GeneID:101107864
34.84537	GeneID:114108863
4.378276	GeneID:101103946
24.20582	GeneID:106990105

56.86229	GeneID:101120034
40.19961	GeneID:101118300
46.29489	GeneID:101109234
57.2384	GeneID:101107594
69.57097	GeneID:780443
27.15224	GeneID:101117839
32.07135	GeneID:101109182
35.66092	GeneID:101121690
39.41637	GeneID:101117334
25.58947	GeneID:101101995
68.63261	GeneID:106990099
19.79462	GeneID:101111008
6.824438	GeneID:100302337
35.69783	GeneID:101102596
56.74146	GeneID:101119708
17.12686	GeneID:101122121
1.832147	GeneID:101102384
14.54365	GeneID:101122002
11.77034	GeneID:101109616
7.782705	GeneID:101103985
8.102043	GeneID:101120646
12.30863	GeneID:101119164
5.460287	GeneID:101105664
9.234747	GeneID:101116112
5.961054	GeneID:101114037
4.155838	GeneID:101120265
17.7918	GeneID:101123654
0.130937	GeneID:101112571
11.27633	GeneID:101114326
26.05961	GeneID:101109841
21.97392	GeneID:101102337
1.395612	GeneID:101102958
3.465704	GeneID:101105413
0.485471	GeneID:101110686
0	GeneID:100233238
19.05711	GeneID:101103415
9.843269	GeneID:100170322
69.90864	GeneID:100170667
0.080274	GeneID:443053
4.776555	GeneID:101123173
0	GeneID:101102739
0.062661	GeneID:101116142
0	GeneID:101123433
0.01907	GeneID:101112329
29.63502	GeneID:101122802
3.700714	GeneID:443028
2.409923	GeneID:101110092
19.72886	GeneID:101116316
0.791009	GeneID:101102192
9.397534	GeneID:101110448
2.577439	GeneID:101102975
3.190048	GeneID:101108255
6.355793	GeneID:101110040
0.173782	GeneID:101111194
0.005202	GeneID:101108392
0.014204	GeneID:101108426
10.14667	GeneID:101104474
0	GeneID:101120287

0.250241	GeneID:101106297
54.13272	GeneID:100302101
10.23557	GeneID:101121960
24.15496	GeneID:101116917
0.275366	GeneID:101120625
0.890398	GeneID:101102679
0.037672	GeneID:101118614
0	GeneID:101110962
3.274756	GeneID:101120909
0.12666	GeneID:101104863
2.411712	GeneID:101117140
12.92742	GeneID:101108542
3.783312	GeneID:101111418
23.66223	GeneID:101107799
0.034933	GeneID:101107584
10.50596	GeneID:101108319
9.093289	GeneID:101113701
19.44266	GeneID:101116553
4.324456	GeneID:105604798
11.60482	GeneID:101114513
17.52258	GeneID:101107398
10.36331	GeneID:443434
0.248605	GeneID:443193
0.534051	GeneID:101113171
17.56577	GeneID:101118317
22.73044	GeneID:101107298
0.841115	GeneID:101112477
37.64014	GeneID:101103861
1.766228	GeneID:101120411
0.918364	GeneID:443017
43.46367	GeneID:101122057
114.2034	GeneID:101106810
1.356078	GeneID:101117491
2.605313	GeneID:114110135
1.355976	GeneID:101115270
5.85622	GeneID:101109762
65.93816	GeneID:101105648
0.023348	GeneID:101106957
0	GeneID:101113926
15.79721	GeneID:101108914
16.23434	GeneID:101111322
4.710378	GeneID:101109066
116.1643	GeneID:106990821
7.358745	GeneID:443118
23.48725	GeneID:101114243
9.215224	GeneID:101113882
8.386267	GeneID:101108743
0.054969	GeneID:101113586
1.425918	GeneID:101107485
6.719744	GeneID:101112976
17.48312	GeneID:101123600
16.7067	GeneID:101112213
4.55613	GeneID:101108688
11.74742	GeneID:101114225
43.18411	GeneID:101114834
6.931814	GeneID:101110318
6.460513	GeneID:101102184
13.8426	GeneID:114114097

2.845465	GeneID:100125358
0	GeneID:101121525
0	GeneID:443354
0	GeneID:443025
0.017143	GeneID:101102228
23.94134	GeneID:101122183
52.53266	GeneID:105603284
145.7713	GeneID:101112710
0.024588	GeneID:101114314
28.43885	GeneID:101109344
0	GeneID:100462690
0.112439	GeneID:100462691
6.233454	GeneID:101109455
47.13904	GeneID:101102507
36.39736	GeneID:101115380
43.3028	GeneID:101117537
4.244432	GeneID:101108913
0.064123	GeneID:101113732
7.754419	GeneID:101116414
12.67399	GeneID:101116926
0	GeneID:101102645
20.33054	GeneID:101102164
44.87685	GeneID:101121219
1.784827	GeneID:101109254
0.033763	GeneID:101102952
14.04005	GeneID:101111626
0	GeneID:114113241
5.578421	GeneID:101117925
2.285877	GeneID:101110105
0.130197	GeneID:101111901
18.89846	GeneID:101113179
1.507697	GeneID:101108130
9.181508	GeneID:101102117
13.21869	GeneID:101104349
0	GeneID:101119581
3.509861	GeneID:443263
9.487184	GeneID:100169934
231.7155	GeneID:497274
0.010416	GeneID:101109193
0	GeneID:101105105
0.3803	GeneID:101118903
0.038011	GeneID:780513
75.12636	GeneID:101109711
24.45257	GeneID:101116029
13.58715	GeneID:101103442
0	GeneID:101115362
0.259573	GeneID:101114405
12.38557	GeneID:101118708
6.947608	GeneID:101102280
28.37685	GeneID:101120615
5.268004	GeneID:101121053
67.96799	GeneID:101109163
15.41249	GeneID:101106670
75.21083	GeneID:101111621
144.0866	GeneID:101102685
26.92393	GeneID:101120005
5.302915	GeneID:101104200
12.91309	GeneID:101109969

0	GeneID:443400
8.450284	GeneID:101123199
0.025513	GeneID:443523
4.856798	GeneID:101118070
6.868441	GeneID:101120469
4.309968	GeneID:101118243
12.83242	GeneID:101108956
16.98281	GeneID:101112860
0.015517	GeneID:101103122
0.036247	GeneID:101114234
0	GeneID:101116738
0	GeneID:443382
0	GeneID:443391
0	GeneID:443394
43.91085	GeneID:101120241
23.07413	GeneID:100171396
3.896971	GeneID:101109276
46.90629	GeneID:101120239
9.150091	GeneID:101109703
29.30507	GeneID:101121957
27.33047	GeneID:101122459
35.66855	GeneID:100216435
5.312144	GeneID:101105108
1.330359	GeneID:101122060
4.525678	GeneID:101102495
1.621376	GeneID:101112518
6.86282	GeneID:101116301
0.017293	GeneID:101109781
92.3091	GeneID:101111647
28.86688	GeneID:101123618
0	GeneID:101115999
857.6619	GeneID:101104079
2793.196	GeneID:101112685
0.064527	GeneID:101105518
54.3554	GeneID:101112780
8.508057	GeneID:101119326
10.39603	GeneID:101103214
6.189033	GeneID:101117378
12.49286	GeneID:101123147
0.129439	GeneID:101104001
17.48437	GeneID:101104910
12.77373	GeneID:101115416
0.008807	GeneID:101111767
2.855899	GeneID:101122709
29.808	GeneID:101114297
37.40645	GeneID:101118539
9.640026	GeneID:101107836
21.98487	GeneID:101110306
75.84561	GeneID:101114599
22.12032	GeneID:101114730
13.07826	GeneID:101120412
2.165113	GeneID:101104201
54.68246	GeneID:101118487
0.209483	GeneID:101121943
12.63479	GeneID:101114917
0.086779	GeneID:443040
62.4818	GeneID:101112313
0.013892	GeneID:101121501

0.012554	GeneID:101123346
19.23359	GeneID:101101872
135.6175	GeneID:100294600
28.29304	GeneID:101116924
19.4671	GeneID:780469
53.81057	GeneID:101122978
9.248334	GeneID:101122533
18.10631	GeneID:101103276
6.727723	GeneID:101105631
17.54197	GeneID:101123659
0.008925	GeneID:106991457
65.82059	GeneID:101113615
314.4165	GeneID:780470
31.89964	GeneID:101112686
212.6701	GeneID:443060
71.29561	GeneID:780471
186.4755	GeneID:101108427
57.96462	GeneID:101108972
1.834253	GeneID:101113725
12.10542	GeneID:101112852
35.89336	GeneID:780472
222.797	GeneID:780510
2.407349	GeneID:101110464
54.90173	GeneID:101101911
53.32355	GeneID:101117549
13.60931	GeneID:100126575
21.61575	GeneID:101116359
4.734798	GeneID:101106960
1.612043	GeneID:105609446
0	GeneID:101111808
3.735579	GeneID:101105654
0.010416	GeneID:114109012
0.042649	GeneID:101110433
8.715648	GeneID:101120504
34.79724	GeneID:101107917
23.41755	GeneID:101106584
10.5721	GeneID:101122714
32.07567	GeneID:101122669
38.25552	GeneID:101119145
17.17004	GeneID:101113913
8.37096	GeneID:101105597
22.80781	GeneID:101112931
2.913099	GeneID:101114062
80.91693	GeneID:101114061
9.620739	GeneID:101119521
29.71733	GeneID:101120041
49.94969	GeneID:101102755
7.935624	GeneID:101121571
12.36702	GeneID:101121107
4.698518	GeneID:101104091
9.308131	GeneID:101121304
10.33493	GeneID:101103667
7.987026	GeneID:101104067
13.28341	GeneID:101119927
0.953536	GeneID:101113016
46.50482	GeneID:101116618
0.083331	GeneID:443380
7.522143	GeneID:442997

0.097573	GeneID:101108750
47.17825	GeneID:101121145
1.252367	GeneID:101111100
280.1851	GeneID:101105243
15.44358	GeneID:101111722
0.075512	GeneID:443418
2.751508	GeneID:101108489
1.146382	GeneID:101108811
8.066538	GeneID:101110214
0.128194	GeneID:101107671
16.89584	GeneID:443532
0.018933	GeneID:101104174
0.375546	GeneID:101118482
0.631929	GeneID:101118078
7.395748	GeneID:101103699
0.154829	GeneID:101114165
0	GeneID:101103295
13.36732	GeneID:101112691
0.172797	GeneID:101105249
22.9423	GeneID:101116569
10.1646	GeneID:100048995
167.2572	GeneID:100307037
5.562664	GeneID:100036757
50.66369	GeneID:100188913
29.69339	GeneID:101113147
4.030734	GeneID:100307038
6.528684	GeneID:114116438
0	GeneID:101121662
8.397026	GeneID:101113429
0.0218	GeneID:443399
0	GeneID:100048994
0	GeneID:767576
1.886115	GeneID:105601846
0.261553	GeneID:493968
0.018628	GeneID:100144423
0.075978	GeneID:100170113
4.94044	GeneID:100170323
65.40671	GeneID:100568289
1.175973	GeneID:100568290
1.192031	GeneID:100568286
0	GeneID:100568285
28.68729	GeneID:768095
0.170393	GeneID:100170111
0.013686	GeneID:443099
3.55332	GeneID:101122102
3.474189	GeneID:105607467
1.119524	GeneID:101122003
2.859608	GeneID:101114623
16.08964	GeneID:101112995
46.46919	GeneID:100216436
21.24578	GeneID:101105381
1.441609	GeneID:101103309
2.255376	GeneID:101113467
0	GeneID:101113759
14.83437	GeneID:101111331
43.79355	GeneID:101118570
5.580984	GeneID:101121480
18.9285	GeneID:101123636

3.122182	GeneID:101103274
0.235296	GeneID:101102768
19.16826	GeneID:101104604
9.716054	GeneID:101122097
15.08567	GeneID:101121866
0	GeneID:101102538
6.056016	GeneID:101116320
0.74497	GeneID:101116322
2.168173	GeneID:101121703
97.9469	GeneID:101106014
40.44506	GeneID:101103515
0.402726	GeneID:101109927
11.38625	GeneID:101105875
7.712605	GeneID:101108782
0	GeneID:101115829
47.2592	GeneID:101105188
5.115967	GeneID:101120032
4.224666	GeneID:101113985
2.137275	GeneID:101123304
18.54714	GeneID:101114352
95.94366	GeneID:101111778
3.210965	GeneID:101110675
66.1502	GeneID:100302097
3.647181	GeneID:101114203
0	GeneID:101104559
19.37837	GeneID:100505403
24.88945	GeneID:101115209
120.7805	GeneID:101114764
5.235098	GeneID:101111186
0.219751	GeneID:101117684
0.006595	GeneID:114113851
280.8684	GeneID:101111606
14.55518	GeneID:101123207
2.484303	GeneID:101108508
15.98899	GeneID:101109472
31.84211	GeneID:101123075
23.64445	GeneID:100192428
10.78851	GeneID:101111415
18.26848	GeneID:101120981
0.022074	GeneID:101113223
5.942436	GeneID:101117795
3.90428	GeneID:101105805
19.41453	GeneID:101117933
22.29342	GeneID:101102205
23.25882	GeneID:101102821
7.933561	GeneID:105610491
9.656501	GeneID:101121517
3.977741	GeneID:101116207
4.291197	GeneID:101123126
12.62714	GeneID:101117336
8.739114	GeneID:101116363
20.60799	GeneID:101102573
20.42385	GeneID:101119172
2.943865	GeneID:101117519
13.53978	GeneID:101104072
4.771774	GeneID:101107057
0.011543	GeneID:101118654
0.056767	GeneID:101111830

0.207075	GeneID:101108088
0.065139	GeneID:101116209
0	GeneID:101114755
1.538922	GeneID:101109908
0.012447	GeneID:101122136
4.571136	GeneID:101123378
2.336471	GeneID:101106340
3.826238	GeneID:101103094
1.491842	GeneID:101114472
5.723973	GeneID:101123252
1.37656	GeneID:101121133
1.899224	GeneID:101108937
898.9716	GeneID:443048
6.107334	GeneID:101106973
3.488756	GeneID:101121671
6.802562	GeneID:101103649
12.85683	GeneID:101114475
0.05767	GeneID:101115170
0.014575	GeneID:101102317
2.703934	GeneID:100170232
3.958542	GeneID:101108545
36.35221	GeneID:101116037
104.1429	GeneID:101119807
33.46585	GeneID:101121236
40.53478	GeneID:101103473
26.90482	GeneID:101105464
23.29479	GeneID:101104551
14.37336	GeneID:101121995
9.886877	GeneID:101103689
2.994845	GeneID:101122244
13.96476	GeneID:101113593
19.02263	GeneID:101118636
0	GeneID:101109861
13.55946	GeneID:114116835
31.79762	GeneID:101112562
1.109702	GeneID:101113635
27.4847	GeneID:101118391
53.74502	GeneID:101106122
4.43934	GeneID:101116344
0.513633	GeneID:101106765
8.21244	GeneID:101112738
8.755176	GeneID:101112451
1.630928	GeneID:101114477
12.50063	GeneID:541600
31.49292	GeneID:101114584
1.763988	GeneID:101109448
0	GeneID:101121002
0.125894	GeneID:101111973
69.86335	GeneID:100037660
57.75117	GeneID:101104279
6.573641	GeneID:101109754
23.49328	GeneID:101122540
46.55895	GeneID:101122091
9.363512	GeneID:101106358
1.150064	GeneID:101114265
171.355	GeneID:101121675
13.7271	GeneID:101110033
11.1859	GeneID:101115151

13.4719	GeneID:101115665
32.91431	GeneID:101104037
33.27774	GeneID:101104730
28.67287	GeneID:101116607
0.341594	GeneID:101113022
13.92773	GeneID:101120854
5.709526	GeneID:101111117
52.83105	GeneID:101119632
8.768726	GeneID:101123034
106.3831	GeneID:101101929
125.1249	GeneID:101104719
0.332451	GeneID:100568293
20.54534	GeneID:101102307
20.53694	GeneID:101103580
15.37039	GeneID:101103809
19.379	GeneID:101111007
17.67159	GeneID:101107191
188.5855	GeneID:101106186
10.15608	GeneID:101106650
6.241325	GeneID:101112597
8.106012	GeneID:101112403
30.82297	GeneID:101109648
5.7133	GeneID:101116935
11.53022	GeneID:101116224
5.981598	GeneID:101107257
5.855293	GeneID:101103411
60.01341	GeneID:101103918
0	GeneID:101115261
5.335874	GeneID:101103106
14.278	GeneID:101115040
1.24103	GeneID:101110109
21.07185	GeneID:101106303
17.74901	GeneID:101104519
7.023885	GeneID:101117371
6.576131	GeneID:101108245
0.014903	GeneID:105603737
0	GeneID:114109725
0	GeneID:114117517
36.4137	GeneID:100192315
10.9704	GeneID:101118389
83.84802	GeneID:101122732
9.610604	GeneID:101108385
5.248848	GeneID:101113668
4.038374	GeneID:101107457
0.899978	GeneID:101105327
3.015387	GeneID:101112131
9.046984	GeneID:101122903
7.736911	GeneID:101113024
2.36706	GeneID:101118882
8.6106	GeneID:101122440
2.929899	GeneID:101107240
10.03686	GeneID:101123605
24.61845	GeneID:101123580
0.646693	GeneID:101109436
8.095312	GeneID:101102518
2.323676	GeneID:101116722
26.08694	GeneID:101119506
2.403152	GeneID:101116779

0.573145	GeneID:101121339
4.918354	GeneID:101104430
5.577095	GeneID:101113098
5.044459	GeneID:101120888
2.326576	GeneID:106992034
9.293599	GeneID:101103966
73.7971	GeneID:100037686
47.00772	GeneID:101105423
0.28829	GeneID:101110877
44.3657	GeneID:101117308
24.75085	GeneID:101111872
9.90708	GeneID:101117263
8.565238	GeneID:101115993
0.04737	GeneID:101119682
5.611952	GeneID:114110584
3.711111	GeneID:101114715
4.741188	GeneID:101101905
4.596531	GeneID:100126245
32.97426	GeneID:101110535
6.122285	GeneID:101122168
18.03201	GeneID:114108858
17.64679	GeneID:105605852
22.58585	GeneID:101122137
8.283623	GeneID:101113224
0.311456	GeneID:101102720
2.694188	GeneID:101112442
2.358139	GeneID:101119735
0.439346	GeneID:101114659
3.232956	GeneID:101108320
0.036621	GeneID:101108481
0.012685	GeneID:101110739
3.448037	GeneID:101106172
10.07291	GeneID:101117698
6.456885	GeneID:101114690
83.28941	GeneID:101123162
45.98478	GeneID:101118320
7.227894	GeneID:101110654
10.72886	GeneID:101103175
10.37567	GeneID:101121421
0.110641	GeneID:101123616
7.943808	GeneID:101102664
20.47876	GeneID:101107277
37.63722	GeneID:101113760
11.00956	GeneID:101109465
4.81057	GeneID:101106859
2.368095	GeneID:101121023
21.25306	GeneID:101109288
40.23711	GeneID:101103158
10.79856	GeneID:101114453
0	GeneID:101110775
0.54096	GeneID:101120807
6.390751	GeneID:101115005
8.260075	GeneID:101117008
59.25793	GeneID:101103896
8.171356	GeneID:101104019
15.83842	GeneID:101114811
11.29908	GeneID:101121300
32.67327	GeneID:101115922

6.303147	GeneID:101105763
1.308245	GeneID:101101915
2.502308	GeneID:101106193
13.46158	GeneID:101113451
5.117905	GeneID:101120780
12.64701	GeneID:101123399
18.04941	GeneID:101104508
5.096202	GeneID:101118258
1.256205	GeneID:101121528
18.19813	GeneID:101110072
43.16839	GeneID:101105091
19.67409	GeneID:101121971
15.13381	GeneID:101106682
3.743207	GeneID:101111608
4.862709	GeneID:101105168
11.91222	GeneID:101120020
6.173012	GeneID:101119661
10.74356	GeneID:101107044
3.847245	GeneID:100310792
9.258119	GeneID:100310793
5.75434	GeneID:100142657
7.523607	GeneID:101107497
8.045625	GeneID:101116899
3.839794	GeneID:101109113
4.204973	GeneID:101104442
0.960204	GeneID:114113857
1.209994	GeneID:101111509
20.32256	GeneID:101105549
5.710668	GeneID:101107931
0.007235	GeneID:101120078
0.338584	GeneID:101105051
13.48533	GeneID:100147791
8.658824	GeneID:101122118
4.915727	GeneID:101108891
0.485949	GeneID:101114672
1.791839	GeneID:101107807
0.003522	GeneID:101113596
0.011142	GeneID:101112836
3.544949	GeneID:101110264
35.11753	GeneID:100216437
0.340094	GeneID:101116876
5.750118	GeneID:101123633
23.02492	GeneID:101122131
2.923804	GeneID:101105351
9.808902	GeneID:101110965
6.898975	GeneID:101123183
29.42371	GeneID:101105152
0.469661	GeneID:101115235
0	GeneID:105613439
26.82087	GeneID:101103028
0.80871	GeneID:101104867
15.55125	GeneID:101102123
1.916996	GeneID:101116743
16.6591	GeneID:101110731
0.057879	GeneID:101105792
0	GeneID:101102620
0.142432	GeneID:101114484
8.153026	GeneID:101121283

8.674762	GeneID:101118701
0	GeneID:101119678
1.523882	GeneID:101113181
3.834428	GeneID:101117853
0.183397	GeneID:101116337
2.665448	GeneID:780478
36.05442	GeneID:101108658
5.794563	GeneID:101113912
6.542805	GeneID:101106667
176.4464	GeneID:101109372
17.68229	GeneID:101109628
1.021988	GeneID:101119298
0.014261	GeneID:101115361
15.63775	GeneID:101118249
15.80005	GeneID:101122972
10.92716	GeneID:101103572
10.81673	GeneID:101115583
0.784299	GeneID:105601930
0.269152	GeneID:101104983
6.136422	GeneID:101114682
0.00657	GeneID:101113594
105.0145	GeneID:101117186
0	GeneID:101123558
11.04867	GeneID:101118379
0.013535	GeneID:101110028
0.296253	GeneID:101110302
0	GeneID:101116889
0.237725	GeneID:101107414
0.137035	GeneID:101108701
10.28604	GeneID:101111877
2.984286	GeneID:101117729
10.18887	GeneID:101118634
9.227388	GeneID:101115363
1.480588	GeneID:101105079
1.289561	GeneID:101105877
0.049327	GeneID:101102412
2.368583	GeneID:101122790
0.561821	GeneID:105602098
0.811487	GeneID:101122030
7.661979	GeneID:101115933
0.021471	GeneID:101112094
0.597472	GeneID:101121295
0.070871	GeneID:101120490
0.004652	GeneID:101108960
0	GeneID:101104330
0.006377	GeneID:101113255
0.028492	GeneID:101104254
0.040986	GeneID:101113559
0.059074	GeneID:101104179
0	GeneID:101122589
0.006562	GeneID:101119280
0.006194	GeneID:101105194
0	GeneID:101114286
0.063152	GeneID:101122250
0.042467	GeneID:101113084
67.75249	GeneID:106990091
71.35996	GeneID:101114549
23.13482	GeneID:101123174

8.952333	GeneID:101121551
74.02325	GeneID:101122104
17.67762	GeneID:101113897
16.41648	GeneID:101114845
0	GeneID:101104684
5.515634	GeneID:101109970
35.70543	GeneID:101116542
0.026698	GeneID:105610609
7.086474	GeneID:101101935
0.902113	GeneID:101119367
27.90001	GeneID:101107859
9.418045	GeneID:101113840
6.667894	GeneID:101102829
19.40918	GeneID:101118669
18.83742	GeneID:101120259
10.73789	GeneID:101102697
9.222584	GeneID:101119603
27.44967	GeneID:101114181
20.58737	GeneID:101102071
5.83166	GeneID:101110690
10.94242	GeneID:101109709
3.264414	GeneID:101115207
3.49466	GeneID:101104447
17.68285	GeneID:101119299
43.0113	GeneID:101111907
0.047881	GeneID:101101970
15.61375	GeneID:101119247
3.921251	GeneID:101121664
1.068075	GeneID:101108467
1.769422	GeneID:101121989
23.59852	GeneID:101110685
2.877219	GeneID:101105485
7.372954	GeneID:101123423
14.66003	GeneID:101108591
4.355695	GeneID:101120672
0	GeneID:101116034
4.099492	GeneID:101111237
69.90752	GeneID:101120677
52.87977	GeneID:101104209
7.291074	GeneID:101117902
4.012231	GeneID:101104495
15.21305	GeneID:101106580
0.394236	GeneID:101114567
1.601482	GeneID:101122154
21.56098	GeneID:101120146
68.69614	GeneID:101115336
1.127459	GeneID:101122895
16.44591	GeneID:101105493
0	GeneID:101110100
1.941435	GeneID:101120240
0	GeneID:101119703
0.003682	GeneID:101110446
10.71217	GeneID:105607221
4.873415	GeneID:101108737
19.0086	GeneID:101108646
27.07897	GeneID:101115027
0.253853	GeneID:101107117
7.455402	GeneID:101120033

20.18805	GeneID:443549
2.721593	GeneID:100500776
1.296372	GeneID:106990108
0	GeneID:101102451
5.850124	GeneID:101117054
0.046607	GeneID:101111504
19.66427	GeneID:101114798
30.0806	GeneID:101107316
2.21273	GeneID:101116792
3.035814	GeneID:101102501
17.25686	GeneID:101117205
1.347872	GeneID:101123187
1.226187	GeneID:101104011
0.601502	GeneID:101114650
2.486122	GeneID:101113058
3.240657	GeneID:101119284
7.798569	GeneID:101119818
7.572087	GeneID:101123342
9.875545	GeneID:101116636
20.13675	GeneID:101110423
7.545351	GeneID:100135444
5.99872	GeneID:101120650
0.78072	GeneID:101119612
0.356001	GeneID:101107279
20.82092	GeneID:101102081
3.505975	GeneID:101121032
0.162457	GeneID:101112783
0	GeneID:106991220
13.20573	GeneID:101116296
8.770476	GeneID:101105901
7.673054	GeneID:101112786
5.080402	GeneID:101114709
6.502838	GeneID:101115672
6.55894	GeneID:101121261
6.083266	GeneID:101107219
17.00954	GeneID:101103361
1.803674	GeneID:101107461
1.883312	GeneID:101110863
0.0131	GeneID:105606371
0.828411	GeneID:101110613
0.078924	GeneID:101110262
32.34078	GeneID:101107647
0.185302	GeneID:101103652
4.662482	GeneID:101104511
3.521882	GeneID:101113967
14.86853	GeneID:101120039
5.153775	GeneID:101115329
4.680703	GeneID:101103817
13.0829	GeneID:101102470
63.02782	GeneID:101122969
8.467275	GeneID:101122767
27.3559	GeneID:101116108
0.120754	GeneID:101109248
9.077614	GeneID:101115241
24.89105	GeneID:100170324
0.202719	GeneID:101105907
9.160717	GeneID:105605898
12.14572	GeneID:101119728

8.837324	GeneID:101113589
0	GeneID:101113457
0	GeneID:101116692
0	GeneID:105602093
52.55798	GeneID:101122371
6.380643	GeneID:101109090
0.008291	GeneID:101117923
3.99638	GeneID:101107888
5.759546	GeneID:101110069
32.05209	GeneID:101105231
7.732576	GeneID:101109311
1.978927	GeneID:101102070
16.43338	GeneID:101108984
27.8427	GeneID:101114775
0.023789	GeneID:101108975
0.009848	GeneID:101111869
3.995174	GeneID:101108962
41.19833	GeneID:105603928
0.157205	GeneID:101119457
21.54817	GeneID:101105865
7.772807	GeneID:101109084
9.008786	GeneID:101112523
58.53397	GeneID:101111673
0.052844	GeneID:101111822
0.596786	GeneID:101110744
0.475481	GeneID:101121436
0.461094	GeneID:101102834
0.035889	GeneID:100568292
0	GeneID:101113525
0.031063	GeneID:101103110
0	GeneID:114110137
0.112946	GeneID:101114778
39.7976	GeneID:101115484
12.85082	GeneID:101121183
0	GeneID:101116883
4.986681	GeneID:101115827
0.662998	GeneID:101104809
153.3417	GeneID:101115748
27.96164	GeneID:101116192
0.040797	GeneID:101113097
7.199191	GeneID:101116411
8.531843	GeneID:101105922
1.850921	GeneID:101103774
201.5035	GeneID:101115499
26.81691	GeneID:101118655
15.0731	GeneID:101115247
112.7777	GeneID:101118914
5.431044	GeneID:101123060
484.5302	GeneID:101119169
0	GeneID:101123300
20.42952	GeneID:101113020
238.3844	GeneID:101119923
6.848807	GeneID:101109553
30.64017	GeneID:101122541
12.57231	GeneID:101116351
0.005028	GeneID:101108656
2.909512	GeneID:101117523
0.850047	GeneID:101111507

5.573384	GeneID:101109165
19.76272	GeneID:100145862
11.15854	GeneID:101108491
1.445922	GeneID:101115791
1.765482	GeneID:101109123
14.5626	GeneID:101116532
7.411184	GeneID:101118862
3.64539	GeneID:101119106
1.889581	GeneID:101110361
14.7819	GeneID:101121994
1.102911	GeneID:101111972
0.186821	GeneID:100462743
4.44382	GeneID:101115034
0.099106	GeneID:100379136
0	GeneID:101109415
17.87012	GeneID:101111894
2.761088	GeneID:101117185
4.690208	GeneID:101110760
4.362895	GeneID:101104143
20.33365	GeneID:101107632
5.031175	GeneID:101106025
36.24804	GeneID:101113742
9.185709	GeneID:101107242
0.022853	GeneID:101102034
141.9901	GeneID:101112145
3.015492	GeneID:101112411
10.98637	GeneID:101118942
1.90248	GeneID:101102676
6.003475	GeneID:101117733
14.14001	GeneID:101115699
0	GeneID:101107669
5.129455	GeneID:101102433
4.39038	GeneID:101115745
3.537593	GeneID:101108886
0.081483	GeneID:101110992
6.811132	GeneID:114113948
14.67872	GeneID:101114374
8.324732	GeneID:101103535
26.01101	GeneID:101118748
15.07516	GeneID:101110315
33.60682	GeneID:101112604
7.259996	GeneID:101122905
0.201694	GeneID:101123448
41.77259	GeneID:101110765
0	GeneID:101113006
0	GeneID:114117803
11.85337	GeneID:101104259
8.019153	GeneID:101117599
15.42622	GeneID:101107926
9.675482	GeneID:101114397
0	GeneID:100144749
0.051351	GeneID:101108020
0.039698	GeneID:101108281
8.756585	GeneID:101115171
45.13486	GeneID:101102427
0.459171	GeneID:101118013
71.28807	GeneID:101115433
29.6734	GeneID:101112428

34.55354	GeneID:101108246
1.547746	GeneID:101118215
6.975592	GeneID:101114180
621.5541	GeneID:101118902
32.55239	GeneID:101121274
156.7974	GeneID:101112751
7.664532	GeneID:101120685
62.5786	GeneID:101110149
79.80581	GeneID:443256
22.5476	GeneID:101117130
3.897094	GeneID:101117958
13.66986	GeneID:101107511
3.40051	GeneID:101117860
0	GeneID:101111959
2.998114	GeneID:101109257
0.377639	GeneID:101121697
23.69823	GeneID:101108499
1.219337	GeneID:101112535
1.498289	GeneID:101116191
2.331134	GeneID:101117526
5.123482	GeneID:105606975
8.290896	GeneID:101121809
18.31469	GeneID:101106963
13.90494	GeneID:101114117
4.631137	GeneID:101120153
2.10266	GeneID:101102051
2.195447	GeneID:101115742
9.977983	GeneID:101115422
72.21865	GeneID:105610361
0.801818	GeneID:101111144
9.739263	GeneID:101117880
4.598892	GeneID:101117387
7.985141	GeneID:101117425
6.014882	GeneID:101114190
1.241062	GeneID:101110734
2.45364	GeneID:101107152
1.11407	GeneID:105606246
0.146927	GeneID:101113064
55.29898	GeneID:101105871
43.69414	GeneID:101112029
22.43731	GeneID:101117596
19.87431	GeneID:101117214
41.96123	GeneID:443125
0.611002	GeneID:101102212
59.59328	GeneID:101112922
37.80868	GeneID:101116321
6.075356	GeneID:101108411
141.8023	GeneID:101112867
10.89247	GeneID:101115592
70.34044	GeneID:101121214
15.21264	GeneID:101105541
30.60978	GeneID:101108189
28.73953	GeneID:101112375
24.43403	GeneID:101115456
8.425878	GeneID:101120161
4.500216	GeneID:101113294
0.333923	GeneID:101114710
2.441902	GeneID:101120990

0.072593	GeneID:101118670
1.324745	GeneID:101109349
5.254494	GeneID:101115598
7.128786	GeneID:101109486
11.64344	GeneID:101110083
1.331725	GeneID:105602015
16.48693	GeneID:101103353
9.147782	GeneID:101107978
93.39083	GeneID:101105488
9.082508	GeneID:101118698
10.29982	GeneID:443498
8.234067	GeneID:101103933
0.219761	GeneID:101114458
24.30006	GeneID:443465
35.44397	GeneID:443139
10.79874	GeneID:101105618
11.05636	GeneID:101114003
11.91093	GeneID:101113712
2066.716	GeneID:100101228
0.121733	GeneID:101109898
4.183127	GeneID:101117856
4.391413	GeneID:101104876
2.453854	GeneID:101115529
4.295958	GeneID:101106537
396.5688	GeneID:101115859
176.5428	GeneID:443504
11.59578	GeneID:100135689
700.1181	GeneID:101111240
492.6344	GeneID:101114105
23.05144	GeneID:101103447
2.681401	GeneID:101104798
7.872309	GeneID:101113540
6.080275	GeneID:101116563
0.352731	GeneID:101111889
1.611189	GeneID:101122944
8.216374	GeneID:101119643
0.01249	GeneID:101107051
0.023355	GeneID:101121788
27.36698	GeneID:101104868
0.167306	GeneID:101106863
0.01853	GeneID:106990131
0.188838	GeneID:101122536
0.144841	GeneID:101117152
4.253901	GeneID:101108968
0	GeneID:105611329
0.010059	GeneID:101105778
0.106409	GeneID:101112006
80.06652	GeneID:101120648
16.04236	GeneID:101103198
0.994561	GeneID:101120979
0.758809	GeneID:101105193
1.134406	GeneID:101103300
198.328	GeneID:101113980
51.3219	GeneID:101109897
12.15381	GeneID:101112350
52.07308	GeneID:101105624
3.771842	GeneID:101114443
52.27016	GeneID:101115414

21.3902	GeneID:101105380
13.47778	GeneID:101115792
29.64016	GeneID:101120475
37.64146	GeneID:101115469
0.754621	GeneID:101103994
32.09488	GeneID:101121432
0.988108	GeneID:101108899
15.98152	GeneID:101120836
25.33115	GeneID:101116744
9.068187	GeneID:101114767
1.457569	GeneID:101122638
2.284053	GeneID:101106626
32.52813	GeneID:780479
19.58297	GeneID:101114163
14.66273	GeneID:101120517
66.3155	GeneID:101117367
51.58585	GeneID:443547
15.8381	GeneID:101122407
27.67596	GeneID:101121578
0.012693	GeneID:101113051
7.068484	GeneID:101116893
6.205342	GeneID:101123511
12.37545	GeneID:101118911
26.50754	GeneID:101103177
2.720957	GeneID:101104895
11.68676	GeneID:101115625
15.19936	GeneID:100913164
4.866964	GeneID:101117635
14.67285	GeneID:101123545
14.79466	GeneID:101115739
13.68425	GeneID:101123359
55.24986	GeneID:101110514
1.767136	GeneID:101113401
330.2548	GeneID:101118968
11.43078	GeneID:101112176
127.8086	GeneID:100270711
53.51239	GeneID:101114218
53.88179	GeneID:101119523
20.4914	GeneID:101116114
4.475814	GeneID:101121673
6.775983	GeneID:101110042
6.828445	GeneID:101107358
26.93354	GeneID:101117193
32.24628	GeneID:101108921
20.00815	GeneID:101118316
39.51881	GeneID:101117233
39.38532	GeneID:101108187
11.03678	GeneID:101117434
25.15444	GeneID:101113414
174.6406	GeneID:106990093
147.7431	GeneID:101114675
100.0212	GeneID:100137071
68.98197	GeneID:101112875
124.731	GeneID:101112694
98.87749	GeneID:101122181
299.552	GeneID:101118881
82.47654	GeneID:101108947
213.6123	GeneID:101111798

258.8038	GeneID:101115801
124.4324	GeneID:101115686
12.59362	GeneID:101116236
243.5226	GeneID:101111219
107.4906	GeneID:101102715
251.9661	GeneID:101121881
339.1903	GeneID:105601858
267.5642	GeneID:100037661
77.9811	GeneID:101121437
188.9885	GeneID:101107685
53.12901	GeneID:101112730
25.52638	GeneID:101103218
5.736623	GeneID:101114394
37.01543	GeneID:101111426
105.9454	GeneID:101110115
10.4033	GeneID:105615363
13.43146	GeneID:101116175
115.7912	GeneID:494441
380.9411	GeneID:101123232
18.60174	GeneID:101105220
188.6228	GeneID:101110642
124.0774	GeneID:101104786
308.9577	GeneID:100307039
3.848464	GeneID:101116949
34.63299	GeneID:101106067
66.26856	GeneID:100302338
17.11216	GeneID:101113469
5.311801	GeneID:101118238
18.41245	GeneID:101104921
0.155441	GeneID:101110841
16.29606	GeneID:100307040
0.109382	GeneID:101107668
0.005839	GeneID:101119719
0.285671	GeneID:101107912
20.3857	GeneID:101106441
15.92512	GeneID:101117192
2.330662	GeneID:101104676
3.655421	GeneID:101121321
47.03434	GeneID:101104338
0.158809	GeneID:114110558
0.058277	GeneID:106991003
9.850668	GeneID:101113988
12.04388	GeneID:101122180
12.91979	GeneID:101114794
6.610528	GeneID:101109445
12.13481	GeneID:101101983
0.162939	GeneID:101110708
3.675729	GeneID:101114771
7.601836	GeneID:101114633
16.00724	GeneID:101107217
3.894737	GeneID:101104337
6.255593	GeneID:101114814
5.73398	GeneID:101123360
11.16111	GeneID:101105164
14.67758	GeneID:101111152
32.34306	GeneID:101120903
75.23513	GeneID:101118496
21.51955	GeneID:101107798

35.26745	GeneID:101120572
62.20999	GeneID:101110801
0.473081	GeneID:101116613
177.3382	GeneID:101106896
66.78689	GeneID:101113417
4.131798	GeneID:101108431
27.88345	GeneID:101118274
49.87432	GeneID:101108515
9.694625	GeneID:101115680
12.94879	GeneID:101107058
6.913404	GeneID:101115183
3.863754	GeneID:101119754
22.4671	GeneID:101117858
6.437656	GeneID:101113703
0.179796	GeneID:443149
0.0195	GeneID:443148
3.361179	GeneID:101121040
19.603	GeneID:101109679
61.39238	GeneID:101117529
55.83345	GeneID:101122607
28.30861	GeneID:101101927
50.81643	GeneID:101122789
21.64957	GeneID:101123475
22.20481	GeneID:101123302
13.05814	GeneID:101104926
6.299645	GeneID:101117671
12.67081	GeneID:101109178
32.84259	GeneID:101109247
3.612448	GeneID:101106603
4.543954	GeneID:101111068
25.49168	GeneID:101108377
3.276343	GeneID:101120697
4.154588	GeneID:101113218
8.230894	GeneID:101123002
0.769694	GeneID:101104083
3.819562	GeneID:101117881
6.498356	GeneID:101117612
10.79526	GeneID:101111136
8.834507	GeneID:101115950
1.507475	GeneID:101114197
1.298259	GeneID:101101879
398.7785	GeneID:101109432
102.4802	GeneID:101107656
23.53424	GeneID:101121114
5.134158	GeneID:101111315
0.088852	GeneID:101103146
3.027743	GeneID:101105042
0.794029	GeneID:105610628
0	GeneID:101117159
18.18261	GeneID:101104593
0	GeneID:101121349
17.27059	GeneID:101112420
49.10669	GeneID:101102837
1.990899	GeneID:780458
36.79556	GeneID:101110045
5.084342	GeneID:101119998
21.59455	GeneID:101123539
22.32562	GeneID:101112239

17.56888	GeneID:101121999
2.331824	GeneID:101115148
1.237385	GeneID:101105676
141.6727	GeneID:101118119
0.407464	GeneID:101109164
0.459036	GeneID:443086
0.326018	GeneID:101104109
20.12935	GeneID:101114870
2.959667	GeneID:101104113
1.66502	GeneID:101102318
3.77377	GeneID:101104304
0.176866	GeneID:101117763
11.03802	GeneID:101123048
10.35205	GeneID:101120171
7.826133	GeneID:101122795
2.506458	GeneID:101119767
3.889762	GeneID:101120026
0	GeneID:101108718
0	GeneID:101111726
64.79297	GeneID:101107055
0.010904	GeneID:101101885
15.58246	GeneID:101110915
3.864735	GeneID:101114471
11.27331	GeneID:101120555
1.06462	GeneID:101105824
5.509162	GeneID:101104845
2.596149	GeneID:101119006
0.040999	GeneID:105612430
13.66105	GeneID:101116041
40.45659	GeneID:101117939
2.804652	GeneID:101117114
0.094698	GeneID:101103189
14.79026	GeneID:101110136
8.193698	GeneID:101117449
45.10946	GeneID:443286
12.63375	GeneID:101122082
14.99317	GeneID:101120768
10.30667	GeneID:101120756
4.117186	GeneID:101106481
8.490523	GeneID:101122032
17.77558	GeneID:101122929
2.819024	GeneID:101112882
0.024783	GeneID:101120335
12.2542	GeneID:101113778
10.00275	GeneID:101121402
131.5054	GeneID:100302339
0.616937	GeneID:101107276
6.490841	GeneID:101109319
3.066073	GeneID:101105158
35.13298	GeneID:101115703
0.015765	GeneID:101115582
17.97714	GeneID:101110575
16.70856	GeneID:101118852
0.314447	GeneID:101107288
0.137928	GeneID:101122177
2.730953	GeneID:101114662
0.787956	GeneID:101122928
22.3473	GeneID:101106389

16.80726	GeneID:101120886
14.06592	GeneID:101120569
24.79429	GeneID:101111038
18.84645	GeneID:101106654
17.30731	GeneID:101120077
0	GeneID:101102684
0.27048	GeneID:101102494
20.79583	GeneID:101109125
27.76977	GeneID:101117189
19.21223	GeneID:101119060
4.886465	GeneID:101117262
0.570995	GeneID:101102322
0.04782	GeneID:443227
6.349433	GeneID:101116485
72.21616	GeneID:101104590
37.66578	GeneID:101110462
10.47048	GeneID:101117929
18.70911	GeneID:101104477
7.734226	GeneID:101122621
15.17734	GeneID:101102603
0.011436	GeneID:101109134
3.435314	GeneID:101123642
0	GeneID:101112468
0.149378	GeneID:101106238
10.88511	GeneID:101108142
6.698136	GeneID:101110506
4.591873	GeneID:101102484
0.11339	GeneID:101108988
44.9288	GeneID:780519
61.85226	GeneID:101103889
31.71182	GeneID:101123325
11.76997	GeneID:101121000
0.048155	GeneID:101105111
24.05844	GeneID:101116856
7.661455	GeneID:101116341
13.02944	GeneID:101110119
6.069178	GeneID:101109669
11.32373	GeneID:101114031
7.136945	GeneID:101117559
1.770161	GeneID:101108989
4.551064	GeneID:101111688
9.137242	GeneID:101108254
5.140592	GeneID:101103724
17.32638	GeneID:101102508
0.064478	GeneID:114113960
5.653583	GeneID:101110646
24.22012	GeneID:101107897
19.64153	GeneID:101108735
88.34061	GeneID:101116752
114.3207	GeneID:101118702
34.43251	GeneID:101102618
2.537228	GeneID:101121562
11.87446	GeneID:101116000
3.522215	GeneID:101102370
1.154343	GeneID:101117821
0.015958	GeneID:101106818
4.82938	GeneID:105614372
0	GeneID:101106856

0	GeneID:101103578
17.52787	GeneID:101121052
20.24248	GeneID:101110479
0.015815	GeneID:101103113
3.137512	GeneID:101117594
0	GeneID:101113731
20.88375	GeneID:101115940
3.764315	GeneID:101102746
0	GeneID:101117900
10.75823	GeneID:101109883
3.781147	GeneID:101117988
0.039311	GeneID:101106511
90.3478	GeneID:100145868
16.66849	GeneID:101122251
6.071688	GeneID:101115142
10.74265	GeneID:101108435
3.935313	GeneID:101123257
1.881468	GeneID:101114418
27.51968	GeneID:101113080
8.746366	GeneID:101110781
1.309424	GeneID:101117876
1.903048	GeneID:101105898
4.076989	GeneID:101116925
0	GeneID:106990176
6.448215	GeneID:443228
0.106689	GeneID:443066
78.16287	GeneID:101109714
23.4864	GeneID:101118043
5.253117	GeneID:101116615
0	GeneID:101109022
0.042892	GeneID:101102323
9.445973	GeneID:101120112
15.30318	GeneID:101111441
29.75749	GeneID:101118945
9.27441	GeneID:101112022
14.75564	GeneID:101111788
0	GeneID:114111390
55.53583	GeneID:101111800
69.81004	GeneID:101102591
53.96744	GeneID:101110082
0.957254	GeneID:101107011
14.63672	GeneID:101106714
8.935698	GeneID:101114600
32.95049	GeneID:101108154
8.202979	GeneID:101114860
5.757906	GeneID:101107456
1.172481	GeneID:101120832
14.22751	GeneID:101114986
35.94743	GeneID:443026
0.717332	GeneID:101123624
1.393493	GeneID:101119411
0.156478	GeneID:105608968
2.629283	GeneID:101110344
17.58957	GeneID:101115418
12.09209	GeneID:101119464
1.217048	GeneID:101110408
14.22492	GeneID:101117555
2.936541	GeneID:101102355

6.32147	GeneID:101113496
3.250416	GeneID:101102904
0.861674	GeneID:101101901
2.767214	GeneID:101123474
5.490371	GeneID:101117131
4.671812	GeneID:101102563
25.02592	GeneID:101118138
18.92497	GeneID:101118371
92.03015	GeneID:101121203
0.024653	GeneID:101115129
1.805721	GeneID:101117589
1.528182	GeneID:101104737
1.375899	GeneID:101120258
4.11999	GeneID:101123508
14.86473	GeneID:101107281
0	GeneID:114115413
18.88633	GeneID:101103266
0.259639	GeneID:101112248
2.843735	GeneID:101115304
0.238464	GeneID:101122728
12.04221	GeneID:101107440
18.67986	GeneID:101121014
6.09759	GeneID:101103200
12.2964	GeneID:101111176
24.15956	GeneID:101106022
8.72225	GeneID:101113907
5.024202	GeneID:101111131
20.54308	GeneID:101113810
13.33822	GeneID:101113610
12.19885	GeneID:101114760
8.283034	GeneID:101106397
5.64301	GeneID:101115548
11.44858	GeneID:101123655
14.22658	GeneID:101115396
19.26724	GeneID:101120279
19.46067	GeneID:101104834
18.76463	GeneID:101108309
21.5853	GeneID:101114877
18.35639	GeneID:101110867
0.026838	GeneID:101113394
2.990165	GeneID:101115079
7.399178	GeneID:101108814
0.486576	GeneID:101105259
0.676553	GeneID:101118806
15.91969	GeneID:100271658
0.136449	GeneID:101106335
16.24051	GeneID:101104765
21.51251	GeneID:101120745
154.9309	GeneID:101110420
0.015022	GeneID:101118624
55.40935	GeneID:780487
0.141966	GeneID:101106770
43.31401	GeneID:101108612
0.060184	GeneID:100307041
23.73842	GeneID:101105757
25.78063	GeneID:101106266
31.94692	GeneID:101110677
0	GeneID:101114699

36.58508	GeneID:101118918
0.318639	GeneID:101123649
4.460409	GeneID:100359363
0.426509	GeneID:106991990
41.33762	GeneID:101116673
1.471827	GeneID:101103356
2.160173	GeneID:101109108
4.357674	GeneID:101122044
10.18391	GeneID:101110081
0.801993	GeneID:100913166
192.291	GeneID:100137067
478.1038	GeneID:100270712
0	GeneID:101116051
12.87076	GeneID:101120755
5.800145	GeneID:101116783
13.54968	GeneID:101109049
5.442036	GeneID:101108615
1.958891	GeneID:101110466
47.71014	GeneID:101102657
26.61008	GeneID:101108967
7.469285	GeneID:101117757
3.817847	GeneID:101109216
9.219232	GeneID:101113379
10.99516	GeneID:101110017
0.012441	GeneID:101121759
3.906326	GeneID:101122506
7.082577	GeneID:101121642
36.92441	GeneID:101113606
2.768656	GeneID:101121206
75.03424	GeneID:101108149
13.03115	GeneID:101107721
1.21119	GeneID:105608840
2.442137	GeneID:105606445
4.524718	GeneID:101116811
0.702777	GeneID:101109642
20.25524	GeneID:101112224
10.57287	GeneID:101109013
8.842381	GeneID:101118802
3.664762	GeneID:101111259
3.432909	GeneID:101119201
5.955372	GeneID:101113968
7.641515	GeneID:101115971
7.158129	GeneID:101117820
11.92525	GeneID:101108108
12.16176	GeneID:101107673
0.515681	GeneID:101119402
1.034941	GeneID:101122416
6.081857	GeneID:101108827
9.387633	GeneID:101106405
41.56049	GeneID:101122713
68.62663	GeneID:101110580
0.088883	GeneID:101114355
1.068716	GeneID:101120127
0.206684	GeneID:101123371
0	GeneID:101111352
0.193223	GeneID:114111226
8.873721	GeneID:101112047
15.02519	GeneID:101108058

0.00805	GeneID:101122366
3.828013	GeneID:101122848
3.932885	GeneID:101118358
6.371026	GeneID:101107784
5.81556	GeneID:101119609
3.335445	GeneID:101115854
7.946092	GeneID:101117643
2.703413	GeneID:101120919
0.008031	GeneID:101114789
1.382423	GeneID:101120730
8.949301	GeneID:101105280
4.038483	GeneID:101118896
4.650458	GeneID:101119353
7.097942	GeneID:101122842
1.721611	GeneID:101117915
2.67295	GeneID:101105415
44.40331	GeneID:101113293
0.039827	GeneID:101104244
0.019528	GeneID:101121873
0.11089	GeneID:101110928
0.168948	GeneID:101104228
0.21223	GeneID:101116962
21.53439	GeneID:101118417
1.253562	GeneID:101115189
6.741878	GeneID:101102261
19.35048	GeneID:101104965
2.180875	GeneID:101111393
1.28514	GeneID:101111912
0	GeneID:101104236
0	GeneID:101116375
4.994549	GeneID:101117439
1.577515	GeneID:101116415
3.028083	GeneID:101116019
6.342658	GeneID:101122278
6.307681	GeneID:101111148
14.20268	GeneID:101112055
2.811294	GeneID:101114470
13.67962	GeneID:101119166
0.085924	GeneID:101107540
0.223793	GeneID:114113957
3.538817	GeneID:101108221
0.657693	GeneID:101119248
0.490412	GeneID:101114735
0.183293	GeneID:101123047
0.17013	GeneID:101121264
3.103223	GeneID:101103470
0.734852	GeneID:101118178
0.044023	GeneID:101118203
0.018786	GeneID:101111054
2.104158	GeneID:101116128
5.416867	GeneID:101117807
12.931	GeneID:101122209
8.122543	GeneID:101101892
4.191443	GeneID:101102560
4.688182	GeneID:101123014
7.872578	GeneID:101105706
0	GeneID:101116458
0	GeneID:114112803

6.600839	GeneID:101105213
6.079048	GeneID:101108764
11.93905	GeneID:101120593
15.3034	GeneID:101116448
4.205421	GeneID:101110197
133.2537	GeneID:101105431
19.34704	GeneID:101102648
3.115887	GeneID:101117991
0.514353	GeneID:101104529
0.127343	GeneID:101109104
0.231127	GeneID:101121722
1.867568	GeneID:101123227
4.574704	GeneID:101117225
11.63764	GeneID:101107636
0.675699	GeneID:101110322
0.626118	GeneID:101112285
0.739225	GeneID:114108829
2.586259	GeneID:101107713
0.354735	GeneID:101121929
0.068698	GeneID:101109098
0.649285	GeneID:101106753
1.008366	GeneID:101107005
0.365792	GeneID:101114681
2.079198	GeneID:101108396
12.52137	GeneID:101109320
16.61968	GeneID:101118436
6.221046	GeneID:114115388
13.25942	GeneID:101108363
0.024904	GeneID:101119352
0	GeneID:101112779
261.6733	GeneID:101113302
50.47354	GeneID:101116725
2.58437	GeneID:101107576
0.115779	GeneID:101119956
24.70998	GeneID:101103469
0	GeneID:101108169
18.21006	GeneID:101105543
0.178336	GeneID:101102630
17.77541	GeneID:101106220
32.3581	GeneID:101122507
12.5612	GeneID:101103327
27.71169	GeneID:101119621
7.110324	GeneID:101104621
11.44926	GeneID:101111018
10.10208	GeneID:101114371
0.010121	GeneID:101123263
0	GeneID:101115812
0	GeneID:101112903
0.123319	GeneID:101117271
0.053958	GeneID:101120774
0.029534	GeneID:101111188
0.831054	GeneID:101116244
8.136448	GeneID:101105983
6.710608	GeneID:101102260
0.714972	GeneID:101121926
1.561722	GeneID:101113028
4.09856	GeneID:101115214
0.035128	GeneID:114116357

18.62442	GeneID:101108170
28.89715	GeneID:101119841
8.284852	GeneID:101102582
0.100898	GeneID:101106710
3.002277	GeneID:101102139
39.53429	GeneID:106991420
30.40163	GeneID:101116996
131.7002	GeneID:101105319
8.760356	GeneID:101102154
6.600148	GeneID:101110902
14.98617	GeneID:101123249
16.99275	GeneID:101112268
17.46358	GeneID:101103162
6.391781	GeneID:101112577
0	GeneID:101104678
14.43165	GeneID:101123542
12.99607	GeneID:101105253
3.74856	GeneID:101110960
7.025899	GeneID:101118223
2.734561	GeneID:101111995
0.422444	GeneID:101123188
2.645007	GeneID:101112700
4.261957	GeneID:101123503
5.623287	GeneID:101121980
1.03497	GeneID:101119858
5.153834	GeneID:114108621
4.087867	GeneID:101103510
4.173334	GeneID:101119628
3.270999	GeneID:101106113
0.328038	GeneID:101116440
29.00523	GeneID:101106492
7.246353	GeneID:101119501
55.18419	GeneID:101109003
35.7804	GeneID:101110152
4.724689	GeneID:101123259
7.890187	GeneID:101120705
11.88427	GeneID:101105073
53.08204	GeneID:101119961
7.453011	GeneID:100144426
0.166663	GeneID:100135696
44.66018	GeneID:100170327
11.10129	GeneID:101103314
4.087806	GeneID:101120053
11.07744	GeneID:101116708
1.25848	GeneID:101123663
6.512884	GeneID:101123059
17.37058	GeneID:101116884
5.276607	GeneID:101106258
0.006645	GeneID:101107744
2.897053	GeneID:101123237
0	GeneID:101114084
772.841	GeneID:101106472
2.286099	GeneID:101104245
3.406697	GeneID:101118886
11.46219	GeneID:101112918
96.16946	GeneID:101117097
1.570349	GeneID:101109631
0	GeneID:101117931

189.9603	GeneID:100037704
26.0643	GeneID:101110183
42.72855	GeneID:101112927
5.253374	GeneID:101118347
27.92165	GeneID:101109620
0.196766	GeneID:101104991
0.51342	GeneID:101103811
43.77454	GeneID:101113198
12.89775	GeneID:101103943
5.565145	GeneID:101116937
5.673412	GeneID:101110231
0	GeneID:101118523
4.513082	GeneID:114114075
5.097596	GeneID:101114741
0.023506	GeneID:101104365
12.22951	GeneID:101106771
1.345175	GeneID:101108448
4.760763	GeneID:101104957
1.098306	GeneID:101113704
6.357913	GeneID:101101999
0.023336	GeneID:100169936
0.651237	GeneID:101123561
33.29293	GeneID:100169937
2.567815	GeneID:101104499
42.77671	GeneID:101106171
2.957108	GeneID:101102932
1.358432	GeneID:101111735
0.453754	GeneID:101107163
23.6624	GeneID:101107682
0.349089	GeneID:101101855
0.03488	GeneID:101115941
0.727427	GeneID:114117985
0.710267	GeneID:101112079
11.2808	GeneID:100188916
12.24432	GeneID:101102339
0.141602	GeneID:101104291
16.5616	GeneID:101113718
15.08236	GeneID:101103583
28.77133	GeneID:101102927
6.500479	GeneID:101120415
9.97052	GeneID:101104521
16.70386	GeneID:100142676
6.813236	GeneID:101106799
11.04548	GeneID:101118956
0.127905	GeneID:101119965
15.27465	GeneID:101117330
0	GeneID:101103406
1.961894	GeneID:101116349
0.112644	GeneID:101109929
0.15676	GeneID:101104391
7.520581	GeneID:101110912
0.071738	GeneID:106991352
3.632899	GeneID:101123179
4.65351	GeneID:101114895
0.203904	GeneID:101122449
0.797851	GeneID:101109950
3.233872	GeneID:101103731
11.16011	GeneID:101118684

8.199513	GeneID:101119879
25.01499	GeneID:101107741
5.580931	GeneID:101107136
11.98976	GeneID:101123603
28.76367	GeneID:101122758
5.145358	GeneID:101113450
21.30792	GeneID:114113846
7.772202	GeneID:101104688
15.82214	GeneID:106990146
2.819802	GeneID:101106768
0	GeneID:101113948
2.058383	GeneID:100144427
14.9819	GeneID:443204
28.38456	GeneID:101107871
4.670802	GeneID:101118213
3.339924	GeneID:100192431
2.489278	GeneID:100192432
12.82609	GeneID:100192433
31.85059	GeneID:100144766
3.598411	GeneID:101110837
14.32609	GeneID:101114903
9.292201	GeneID:101107351
3.246072	GeneID:101123405
2.17742	GeneID:101113690
0.141183	GeneID:101113473
0.168299	GeneID:114117574
0.03034	GeneID:101112874
0	GeneID:101112622
0.068514	GeneID:101112371
0.037355	GeneID:101123087
0.021289	GeneID:101107325
0.305176	GeneID:101106984
39.80885	GeneID:101119109
69.01915	GeneID:101119093
13.583	GeneID:101106360
10.8783	GeneID:101112813
4.85578	GeneID:101109734
4.639102	GeneID:101111818
7.718264	GeneID:101115248
26.50688	GeneID:101113330
22.24704	GeneID:101113243
13.13616	GeneID:114115668
6.29864	GeneID:100307042
5.809603	GeneID:101116052
0	GeneID:101114435
0	GeneID:101106949
0	GeneID:101119717
13.29857	GeneID:101117180
28.60517	GeneID:101115796
2.149909	GeneID:101115330
2.661117	GeneID:101107049
0	GeneID:101113641
0	GeneID:101113391
11.27073	GeneID:101110461
15.21904	GeneID:101121927
0	GeneID:101123206
0.121301	GeneID:101119926
0.015374	GeneID:780516

1.273592	GeneID:101105201
0	GeneID:101102090
0.29233	GeneID:101102336
2.891769	GeneID:101117050
1.662652	GeneID:101103192
1.259973	GeneID:101107664
2.33882	GeneID:101105233
4.363609	GeneID:101108865
2.942376	GeneID:101116817
1.012725	GeneID:101109353
1.306566	GeneID:443074
4.442261	GeneID:101122038
2.008081	GeneID:100302340
2.019689	GeneID:101101956
0.09759	GeneID:101113249
0.285949	GeneID:101104968
0	GeneID:101118502
2.68188	GeneID:780460
0	GeneID:101115744
16.00887	GeneID:443306
27.78536	GeneID:101108510
6.372193	GeneID:101114608
0	GeneID:101112475
0	GeneID:101116269
0	GeneID:101116004
11.50327	GeneID:100642180
0	GeneID:101116719
12.13959	GeneID:443095
0.36405	GeneID:101116844
50.61074	GeneID:101106941
0	GeneID:114110258
55.71336	GeneID:780507
6.72364	GeneID:101115039
12.73244	GeneID:101112552
27.94882	GeneID:443548
24.99838	GeneID:554324
0.110689	GeneID:101120660
1.89856	GeneID:101105659
0.306297	GeneID:101123664
1.977711	GeneID:101104022
0.541653	GeneID:101118665
48.45632	GeneID:101118266
1.993757	GeneID:101107075
53.94627	GeneID:100302102
0.028699	GeneID:101110161
18.32014	GeneID:101123666
0.691993	GeneID:100307043
33.70339	GeneID:100307044
10.32563	GeneID:101122510
5.503862	GeneID:101117026
1.052569	GeneID:101106018
1.944498	GeneID:101107464
21.91238	GeneID:101115334
0	GeneID:101122684
7.600771	GeneID:101115568
47.57447	GeneID:101110717
2.140131	GeneID:101103594
3.833056	GeneID:101113245

0	GeneID:105607892
1.187652	GeneID:101105473
2.588143	GeneID:101107186
1.035835	GeneID:101114346
2.651105	GeneID:101110338
12.14799	GeneID:101107834
25.76413	GeneID:101115126
52.30339	GeneID:101118583
4.548543	GeneID:101120413
24.66588	GeneID:101108768
6.051947	GeneID:101118724
5.782989	GeneID:101114387
7.619272	GeneID:101108933
15.68244	GeneID:101103798
5.477771	GeneID:101103228
13.36668	GeneID:101108805
192.2388	GeneID:101105938
6.041914	GeneID:101108378
20.27751	GeneID:101101847
71.60842	GeneID:101109649
93.63773	GeneID:101114010
8.913191	GeneID:101118565
0.020133	GeneID:101116010
16.41051	GeneID:101123607
82.67006	GeneID:101108482
41.78275	GeneID:101120487
6.252922	GeneID:101113368
2.189556	GeneID:101117098
4.108674	GeneID:101117989
9.563154	GeneID:101114227
5.387105	GeneID:101121947
17.01882	GeneID:105613042
4.644102	GeneID:101115412
38.16282	GeneID:101110857
115.7081	GeneID:101120390
31.26958	GeneID:101123330
4.016488	GeneID:101104655
39.32106	GeneID:101105287
32.17371	GeneID:101108401
0.99954	GeneID:101122315
17.71491	GeneID:101117848
49.56791	GeneID:101111731
4.302238	GeneID:443088
0.256788	GeneID:101112331
7.805318	GeneID:780525
2.168853	GeneID:101117247
4.845929	GeneID:101103169
2.523011	GeneID:105604619
1.543726	GeneID:101116790
21.02736	GeneID:101105906
8.050493	GeneID:101101987
2.347726	GeneID:101119232
17.39316	GeneID:101116803
11.05197	GeneID:101122937
0.237576	GeneID:101105089
32.15509	GeneID:101104842
2.544821	GeneID:101105344
8.761604	GeneID:101113713

14.32224	GeneID:101111037
16.22226	GeneID:101117478
0	GeneID:101117222
37.4051	GeneID:100216462
0.910783	GeneID:101108403
10.09997	GeneID:101108669
18.48329	GeneID:101115265
15.45977	GeneID:101106379
20.44451	GeneID:101120014
4.48173	GeneID:101110947
2.579858	GeneID:105616536
0.108749	GeneID:101109112
11.23219	GeneID:101115134
8.428471	GeneID:101114406
3.622933	GeneID:101118933
0.521785	GeneID:101104971
0.102329	GeneID:101122147
0.004498	GeneID:105610579
6.932676	GeneID:101123298
85.77821	GeneID:100302086
7.748412	GeneID:101110335
5.460553	GeneID:101103125
0	GeneID:101121199
5.501016	GeneID:101121452
1.057426	GeneID:101120775
13.69985	GeneID:101114664
3.01342	GeneID:443218
0.148381	GeneID:101117101
0.433323	GeneID:101111137
63.44305	GeneID:101122588
4.944665	GeneID:101106037
0	GeneID:101103003
0.015299	GeneID:101119414
0	GeneID:101114446
34.70992	GeneID:101102186
0.615541	GeneID:101103500
1.448089	GeneID:114118686
0.543573	GeneID:101114157
0.095478	GeneID:101102030
19.11382	GeneID:101104562
0.059422	GeneID:106990838
0.297528	GeneID:101103258
0.14168	GeneID:114109537
0.105228	GeneID:101123074
0.024838	GeneID:101115972
0.101453	GeneID:101104271
0.059991	GeneID:114110269
1.446121	GeneID:101104229
0.469734	GeneID:101117859
19.90211	GeneID:101123457
11.50837	GeneID:101104696
5.069213	GeneID:101110295
12.09255	GeneID:105612388
0.162975	GeneID:101103750
0	GeneID:106990809
2.423138	GeneID:101113491
156.5061	GeneID:101105170
5.966305	GeneID:101106923

8.412787	GeneID:101114708
0	GeneID:101123411
17.2249	GeneID:101107877
38.70269	GeneID:100913151
7.079833	GeneID:101103040
1.099721	GeneID:101123434
4.689466	GeneID:101114648
1.618631	GeneID:101110051
0.086996	GeneID:100240742
4.701563	GeneID:101108524
0.468201	GeneID:114109529
0.027452	GeneID:101114901
0	GeneID:101115184
5.060184	GeneID:101114222
2.662135	GeneID:101121924
1.361955	GeneID:101112153
2.161113	GeneID:101101964
4.210506	GeneID:105601872
1.555583	GeneID:105609583
2.669159	GeneID:101108269
0.86997	GeneID:101121399
0.747754	GeneID:101101900
0	GeneID:101104095
30.95794	GeneID:101117645
0.102957	GeneID:101106946
0	GeneID:105606080
4.427498	GeneID:101119441
5.883249	GeneID:101115892
9.585251	GeneID:101105366
0.078091	GeneID:101111053
37.26008	GeneID:101106265
0.11672	GeneID:101111523
12.97222	GeneID:101122481
0.741664	GeneID:101106908
0	GeneID:101115935
0.027809	GeneID:101115608
0.02344	GeneID:101119442
22.93531	GeneID:101111238
2.219473	GeneID:101123190
10.70926	GeneID:101102468
0.356862	GeneID:101106889
12.41434	GeneID:101102983
1.339361	GeneID:101118419
0.765755	GeneID:105616092
23.50274	GeneID:101108186
0	GeneID:101118036
0	GeneID:101105562
0.046199	GeneID:101112564
1.181842	GeneID:101118501
1.121362	GeneID:101110540
0.358097	GeneID:101106849
9.405143	GeneID:443323
150.6858	GeneID:101118330
10.83416	GeneID:101109272
0.049889	GeneID:101123036
0	GeneID:101105281
0.015341	GeneID:101106741
1.718815	GeneID:101102297

1507.583	GeneID:443355
225.5788	GeneID:101103834
14.4294	GeneID:100125353
13.50333	GeneID:101113204
9.297659	GeneID:101103824
23.79906	GeneID:101123163
9.324672	GeneID:101123458
23.06098	GeneID:101119449
14.40733	GeneID:101118364
2.209096	GeneID:101114636
9.459518	GeneID:101107165
31.1201	GeneID:101104126
20.41661	GeneID:101113117
20.8107	GeneID:780454
107.2529	GeneID:101104799
11.19905	GeneID:101108248
6.548749	GeneID:101107844
2.322081	GeneID:101108628
0.014062	GeneID:114114121
5.359297	GeneID:101111372
2.78058	GeneID:101108852
4.601518	GeneID:101107413
16.21933	GeneID:101104706
18.91646	GeneID:101119406
4.602651	GeneID:101115476
0	GeneID:114118392
54.57464	GeneID:101106615
0	GeneID:101118499
19.78382	GeneID:101105940
133.7659	GeneID:443411
0.285587	GeneID:114117761
1.587357	GeneID:101105278
0.008148	GeneID:101120795
19.32565	GeneID:101104278
33.77237	GeneID:101109363
28.16598	GeneID:101119777
12.13371	GeneID:101112309
50.48789	GeneID:101118991
7.00558	GeneID:780481
4.24577	GeneID:780482
13.38664	GeneID:101101932
1.511256	GeneID:101118762
21.67922	GeneID:101122197
28.77367	GeneID:101103546
2.011335	GeneID:114117561
0.159685	GeneID:101114236
13.22132	GeneID:101117072
17.48934	GeneID:101121790
6.496969	GeneID:101114906
25.61312	GeneID:101106523
16.69043	GeneID:101107022
0.013131	GeneID:100037689
4.133635	GeneID:101113421
30.99848	GeneID:443211
6.552413	GeneID:101121692
5.808137	GeneID:101112165
4.683027	GeneID:101115743
1.050237	GeneID:101103296

167.9559	GeneID:101118285
32.03021	GeneID:101117406
113.8923	GeneID:101118469
6.536837	GeneID:101110007
0.113333	GeneID:101103544
16.36688	GeneID:443478
6.99325	GeneID:443276
4.549051	GeneID:101111329
0.412526	GeneID:101117759
0.127621	GeneID:101121265
0.298417	GeneID:101113565
0.075919	GeneID:101121776
0	GeneID:101115159
0.17579	GeneID:101122358
0.058443	GeneID:100913155
0.005003	GeneID:101114905
0	GeneID:101122539
1.915802	GeneID:101106150
0.167488	GeneID:101121012
0.007176	GeneID:101118526
0.032282	GeneID:101108521
67.01722	GeneID:101106625
0.029203	GeneID:101113305
0.224224	GeneID:101114365
0.017029	GeneID:101110852
0.200185	GeneID:101104110
0.007943	GeneID:101117393
18.1036	GeneID:101115240
17.20218	GeneID:101120573
3.974341	GeneID:100885762
10.35799	GeneID:101104145
0.00872	GeneID:101110627
20.45481	GeneID:101106688
0.039615	GeneID:100037696
0.017703	GeneID:101112599
0.154455	GeneID:101121227
0.204565	GeneID:101104193
3.528916	GeneID:101106737
3.376739	GeneID:101118106
5.352811	GeneID:101120144
7.133741	GeneID:101119657
4.389154	GeneID:101108754
10.41836	GeneID:101119196
3.486204	GeneID:101107214
11.28958	GeneID:101109846
5.418969	GeneID:101109536
16.72355	GeneID:101105153
1.951735	GeneID:101103047
0.376136	GeneID:101104386
3.40285	GeneID:101113458
5.919305	GeneID:101117847
1.61707	GeneID:101109586
1.993783	GeneID:101122548
12.77679	GeneID:101102616
7.770568	GeneID:101102875
0.183476	GeneID:101113990
0.978029	GeneID:101114859
4.63442	GeneID:101111708

0	GeneID:101117195
0.046149	GeneID:101104903
0.026825	GeneID:101123170
0.024198	GeneID:101118117
0.010563	GeneID:101119389
6.985489	GeneID:101120986
34.31181	GeneID:101114955
11.14758	GeneID:101121287
34.50476	GeneID:101111759
1.585581	GeneID:101117423
0.034517	GeneID:101103773
442.1695	GeneID:443005
0.069718	GeneID:101103587
0.795466	GeneID:101110362
16.99263	GeneID:101105988
19.46704	GeneID:101119047
4.578646	GeneID:101118155
0.076804	GeneID:101109955
1.266636	GeneID:101104385
75.92125	GeneID:101101977
9.125072	GeneID:101121731
21.12735	GeneID:101114683
0.725011	GeneID:101112173
3.34497	GeneID:101107225
0.016942	GeneID:101112583
3.947915	GeneID:101105994
35.82869	GeneID:101120928
10.14034	GeneID:101111467
1.682252	GeneID:101108770
1.745523	GeneID:101120361
4.046752	GeneID:101112349;GeneID:114108736
0	GeneID:101119230
0.136068	GeneID:100820747
2.287192	GeneID:101113282
43.93404	GeneID:780483
0.008835	GeneID:100820749
0	GeneID:100820750
3.781004	GeneID:100820751
14.58876	GeneID:105609643
17.97463	GeneID:101105075
11.66998	GeneID:101106983
4.388446	GeneID:101102338
10.8175	GeneID:105602991
4.952934	GeneID:101102104
47.38713	GeneID:100302318
55.31243	GeneID:100174904
6.915515	GeneID:101117379
17.25577	GeneID:101113987
24.23009	GeneID:101120124
13.18851	GeneID:101107150
2.789311	GeneID:101112039
6.101917	GeneID:101120875
0	GeneID:101113844
0.107299	GeneID:101103768
0	GeneID:101102644
1.232797	GeneID:101105979
4.841595	GeneID:101102469
3.287667	GeneID:101109438

6.087122	GeneID:101108824
13.29656	GeneID:101122190
2.839556	GeneID:101117834
0	GeneID:443179
0.183627	GeneID:101115976
3.462607	GeneID:101112987
8.766894	GeneID:101110240
0.085578	GeneID:101117412
0.024761	GeneID:101112303
18.40237	GeneID:101114822
7.699526	GeneID:101117899
18.8852	GeneID:101117194
1.124757	GeneID:101101959
3.278927	GeneID:101121316
0.089511	GeneID:101114706
4.364285	GeneID:101104496
0.068168	GeneID:101119068
0.080937	GeneID:101120459
0.796775	GeneID:105613513
10.55585	GeneID:101120772
0.427524	GeneID:101123530
0.343697	GeneID:101108759
0	GeneID:101105842
3.774404	GeneID:101108971
22.56021	GeneID:101117209
1.662079	GeneID:101107353
0.336137	GeneID:101115678
1.021638	GeneID:101104900
0.248856	GeneID:100913168
0.057403	GeneID:101115426
0	GeneID:101116499
0.020191	GeneID:101108925
0.123265	GeneID:106990980
0.647695	GeneID:100217402
74.66918	GeneID:100302074
244.7788	GeneID:100302068
0.150531	GeneID:101117619
1.371859	GeneID:101103993
0.240021	GeneID:101101866
17.5545	GeneID:101114996
3.768946	GeneID:101108948
1.078995	GeneID:101105361
3.637764	GeneID:101109979
6.514285	GeneID:101122894
2.762835	GeneID:114116889
6.107124	GeneID:101110233
3.080776	GeneID:101119387
6.867092	GeneID:101113785
6.558195	GeneID:101121147
1.595444	GeneID:101123280
17.67164	GeneID:101115424
0.056901	GeneID:443220
9.027557	GeneID:101114838
0.231259	GeneID:101116880
0	GeneID:101112798
15.45944	GeneID:101119786
12.93252	GeneID:101111659
6.233336	GeneID:101104352

5.509069	GeneID:101115644
7.911829	GeneID:101116643
9.993757	GeneID:101120570
14.69891	GeneID:101120125
13.62463	GeneID:101119877
2.892441	GeneID:101109876
0.039421	GeneID:101111478
0	GeneID:101111387
9.808761	GeneID:101123025
17.57565	GeneID:101117133
12.5174	GeneID:101110428
58.36379	GeneID:114116642
26.94921	GeneID:101102474
14.27899	GeneID:443129
35.22881	GeneID:101118706
0.011515	GeneID:101111826
22.10981	GeneID:101110774
12.36678	GeneID:101114414
4.635189	GeneID:101112423
9.104433	GeneID:101102912
0.638169	GeneID:101113872
17.48868	GeneID:443282
0	GeneID:443329
1.249441	GeneID:101122535
60.49077	GeneID:101110024
10.78452	GeneID:443333
0	GeneID:100101237
0.312868	GeneID:443511
0.392753	GeneID:443238
7.562437	GeneID:101111644
26.02845	GeneID:101111114
6.836897	GeneID:101102611
19.7219	GeneID:101105449
0.036378	GeneID:101112051
0	GeneID:101111797
1.351277	GeneID:114114577
0	GeneID:101102063
2.40082	GeneID:101114015
21.67032	GeneID:101104248
4.176488	GeneID:101106192
1.439756	GeneID:101103005
2.549239	GeneID:101105096
5.766875	GeneID:101116959
31.51069	GeneID:101122352
2.409202	GeneID:101102349
0.209625	GeneID:101117930
0.017174	GeneID:101114380
12.48979	GeneID:101122040
8.292999	GeneID:101121803
537.8499	GeneID:101122284
0	GeneID:101113598
0.021649	GeneID:101119228
3.011062	GeneID:101107643
6.019613	GeneID:101115082
0.011307	GeneID:101117710
3.622798	GeneID:101104806
174.9256	GeneID:443345
7.976605	GeneID:101107396

5.276342	GeneID:101107144
11.36229	GeneID:101106891
60.57108	GeneID:101118368
0.085828	GeneID:101115130
2.590898	GeneID:101116241
0.04178	GeneID:101122355
0.520716	GeneID:105604875
0.04469	GeneID:101103323
0.107504	GeneID:101108501
0.878252	GeneID:101118587
4.550531	GeneID:101107065
10.81357	GeneID:101119016
0.048984	GeneID:100101239
6.065401	GeneID:101106236
3.794634	GeneID:101115764
0	GeneID:101115988
12.27847	GeneID:101116138
9.134335	GeneID:101106592
0.118376	GeneID:101117476
3.337013	GeneID:101105662
9.520652	GeneID:101119123
20.04584	GeneID:101116167
6.027545	GeneID:101120651
5.307954	GeneID:101114169
6.713293	GeneID:101119637
2.803004	GeneID:101115287
1.62448	GeneID:101121758
0	GeneID:101123622
8.59404	GeneID:101106830
0.419624	GeneID:101117210
3.224942	GeneID:101102282
0.424153	GeneID:101111939
4.744422	GeneID:101103436
5.335471	GeneID:101103279
35.13116	GeneID:101102520
0.047266	GeneID:541609
0.144367	GeneID:100174983
0	GeneID:101119614
0	GeneID:101120810
0.01505	GeneID:101109484
83.19428	GeneID:100147797
46.25211	GeneID:100135692
70.26976	GeneID:101107402
13.2055	GeneID:101120526
5.110499	GeneID:101122583
0.14616	GeneID:101107269
0.049957	GeneID:101114727
11.10094	GeneID:101109744
34.60532	GeneID:101112212
123.246	GeneID:101113276
0	GeneID:101115809
35.18187	GeneID:443239
121.6587	GeneID:101102999
0.258403	GeneID:101108685
165.2947	GeneID:101109387
0	GeneID:101123590
0.121144	GeneID:100170329
0.677956	GeneID:101111913

35.5587	GeneID:101103778
43.08801	GeneID:101105496
34.612	GeneID:101115868
13.77824	GeneID:101101931
3.070212	GeneID:101123099
3.922967	GeneID:101117866
15.99101	GeneID:101116913
8.040783	GeneID:101114894
3.424249	GeneID:101104064
0.046555	GeneID:105605804
0.027979	GeneID:101104882
23.49084	GeneID:101117456
28.18175	GeneID:101117823
8.757059	GeneID:101102764
1.34575	GeneID:101102594
25.8562	GeneID:101114524
17.96426	GeneID:101111850
19.32224	GeneID:101119215
14.75084	GeneID:101111070
31.33896	GeneID:101117003
2.100713	GeneID:101121067
9.632233	GeneID:101118952
8.487554	GeneID:101116393
69.36568	GeneID:100302341
68.69637	GeneID:101103693
2.542762	GeneID:101105461
7.867498	GeneID:101112295
0.010714	GeneID:101121976
0	GeneID:100233166
0	GeneID:101115534
1.977083	GeneID:106991715
123.8477	GeneID:101104166
5.578826	GeneID:114108861
78.69352	GeneID:101102864
1.240362	GeneID:101110493
12.80725	GeneID:101108976
4.709138	GeneID:101106267
3.604803	GeneID:101110735
24.54645	GeneID:114112926
83.12974	GeneID:100192421
39.72246	GeneID:101115838
1.021895	GeneID:101112183
0.035142	GeneID:106991180
6.46152	GeneID:101106689
0.199053	GeneID:101114066
97.25259	GeneID:101114927
105.2969	GeneID:101110631
2.12855	GeneID:105611474
0.655386	GeneID:101116593
0.572121	GeneID:101114097
1.697115	GeneID:105608415
27.28778	GeneID:101107395
20.27712	GeneID:101114824
35.02786	GeneID:101110004
26.82528	GeneID:101108370
3.771863	GeneID:100125622
31.58934	GeneID:101111134
25.07805	GeneID:101111422

12.61525	GeneID:101108912
9.739966	GeneID:101120577
4.527436	GeneID:101115460
25.58303	GeneID:101122348
26.61094	GeneID:101108446
0	GeneID:101111690
0.093408	GeneID:101112753
0	GeneID:443413
31.91495	GeneID:101110316
7.154492	GeneID:101107341
35.26864	GeneID:101108475
8.565852	GeneID:101112088
26.69465	GeneID:101109394
15.53323	GeneID:101114646
60.35777	GeneID:101116535
33.87719	GeneID:101116877
12.35593	GeneID:101110468
53.87134	GeneID:101122321
12.3703	GeneID:101115000
24.41471	GeneID:101115313
13.09535	GeneID:101107318
0	GeneID:101107979
9.75947	GeneID:101119200
8.439058	GeneID:101120286
10.84912	GeneID:101115656
13.62135	GeneID:101103071
2.180592	GeneID:101103494
11.99109	GeneID:101117540
42.31036	GeneID:101117138
17.36832	GeneID:101112332
18.25432	GeneID:101119834
12.03698	GeneID:101118747
0	GeneID:101111158
44.84638	GeneID:443093
2.408382	GeneID:101109891
0	GeneID:101119177
0.12512	GeneID:101103118
0	GeneID:101123066
0.943153	GeneID:101110729
22.1422	GeneID:101112655
5.973574	GeneID:101110422
13.9052	GeneID:101120972
25.97833	GeneID:101120117
0.408051	GeneID:101116221
1.250925	GeneID:101119394
20.37156	GeneID:101117217
5.179112	GeneID:101117528
5.859346	GeneID:101122094
3.547609	GeneID:101103251
7.022617	GeneID:101112322
6.513133	GeneID:101109595
7.135173	GeneID:101105704
14.18845	GeneID:101115975
0.211307	GeneID:101109073
22.4403	GeneID:101111125
45.97634	GeneID:101122478
19.39436	GeneID:101104709
0.015217	GeneID:101122658

81.21929	GeneID:101119285
11.11094	GeneID:101109518
0.031669	GeneID:101106341
1.608622	GeneID:101106600
4.774501	GeneID:101115143
1.97423	GeneID:101119894
6.309732	GeneID:101104692
2.659901	GeneID:443090
0	GeneID:114110561
0.381136	GeneID:101119430
7.596096	GeneID:101112228
67.61746	GeneID:101112413
0.120884	GeneID:101115549
10.69124	GeneID:101105472
1.217078	GeneID:101119000
2.476405	GeneID:101106904
10.25253	GeneID:101120549
14.47166	GeneID:101119805
8.156868	GeneID:101114759
11.67017	GeneID:101104273
28.44032	GeneID:101109959
2.192012	GeneID:101116208
10.72781	GeneID:101102382
21.85127	GeneID:101104824
0	GeneID:101114624
0.266435	GeneID:101105692
0.107163	GeneID:105613058
0.830658	GeneID:101103102
8.655243	GeneID:101117727
0.209171	GeneID:101116655
0.026597	GeneID:101118916
0.380388	GeneID:101114515
0.200965	GeneID:101119373
0.619962	GeneID:101114925
0.075109	GeneID:101106559
0	GeneID:105607633
0	GeneID:101108132
10.23167	GeneID:101122042
6.879973	GeneID:101110651
0.510048	GeneID:101107652
4.833961	GeneID:105606440
0.135974	GeneID:101106191
5.500427	GeneID:101119099
2.272908	GeneID:101117126
0	GeneID:101109088
0	GeneID:106990949
0.64919	GeneID:101118323
0.551932	GeneID:101122079
0.183771	GeneID:101119371
0.911099	GeneID:101107100
0.041552	GeneID:101123135
0.073158	GeneID:101111202
5.297198	GeneID:101106020
0.017759	GeneID:101103963
0.569687	GeneID:101111463
1.500088	GeneID:101111955
0.131555	GeneID:101118366
0.77858	GeneID:101109948

0	GeneID:101103715
0.024992	GeneID:114117307
2.709147	GeneID:114109426
0	GeneID:101112960
0	GeneID:101115547
0.028624	GeneID:101119331
0.275327	GeneID:101102762
1.742805	GeneID:105605440
0.260371	GeneID:101103941
2.631717	GeneID:101107437
0.014903	GeneID:101114457
0.198725	GeneID:101113876
0.025103	GeneID:101123617
0	GeneID:101115723
0.042355	GeneID:101121499
0	GeneID:101106639
0.600109	GeneID:105607760
3.072205	GeneID:101105759
0.113181	GeneID:101118361
0.743269	GeneID:101111492
1.357164	GeneID:105601862
0.067302	GeneID:101105390
0.178659	GeneID:101118815
0.018421	GeneID:101119970
0.270249	GeneID:101111360
34.33174	GeneID:101117208
0.081302	GeneID:101121739
0.222198	GeneID:101123017
1.670976	GeneID:101110748
11.97988	GeneID:101112533
6.307322	GeneID:101105426
567.7446	GeneID:101110492
0.792429	GeneID:101105750
0.328621	GeneID:101115601
0.189245	GeneID:101117844
21.37619	GeneID:101112147
26.91382	GeneID:101119059
7.241223	GeneID:101116818
18.91923	GeneID:101122644
3.516072	GeneID:105603758
1.467222	GeneID:106991334
54.40467	GeneID:101109822
72.19241	GeneID:100820742
107.5404	GeneID:101110596
121.8858	GeneID:101104397
106.4194	GeneID:101113149
0.020802	GeneID:100913163
5.139945	GeneID:101115673
17.9542	GeneID:101116602
6.017371	GeneID:101122883
0.458877	GeneID:101118986
2.391013	GeneID:101116013
1.674281	GeneID:101115544
19.72315	GeneID:101108384
8.343982	GeneID:101111359
0.904831	GeneID:101107589
3.943237	GeneID:101113577
5.279208	GeneID:101111275

0.755088	GeneID:101105217
21.71545	GeneID:101109893
7.46708	GeneID:101104589
0.006785	GeneID:101120397
0.571641	GeneID:101121478
18.96865	GeneID:101102396
87.88143	GeneID:101103057
11.10871	GeneID:101102496
8.905084	GeneID:101117909
4.19325	GeneID:101108191
0.687793	GeneID:443271
0	GeneID:443272
0.976797	GeneID:443273
0.153439	GeneID:443274
0.228131	GeneID:101114332
0.011763	GeneID:101117078
0.00774	GeneID:101106134
0.009436	GeneID:101107319
0.028422	GeneID:101122701
0.595638	GeneID:101114065
0.46578	GeneID:101110444
0.227994	GeneID:101103501
2.643269	GeneID:443281
0.108061	GeneID:101106989
0.006929	GeneID:101118179
0.020686	GeneID:101102915
0.40108	GeneID:101105943
0.031826	GeneID:101120547
0.239296	GeneID:101112396
12.08834	GeneID:101114619
1.009131	GeneID:101115022
0.25256	GeneID:101109395
13.90828	GeneID:101113568
0.116941	GeneID:105606083
20.06739	GeneID:101108265
3.434038	GeneID:101117366
6.226753	GeneID:101122439
3.280322	GeneID:101107151
6.029809	GeneID:101107019
0	GeneID:101106563
0	GeneID:443101
0.068157	GeneID:101116870
0	GeneID:101113057
0.056647	GeneID:101119427
0.007261	GeneID:443069
0	GeneID:443292
0.458397	GeneID:443520
0.235764	GeneID:101108742
25.60329	GeneID:101115470
0.034687	GeneID:443332
35.2325	GeneID:100037672
10.162	GeneID:101119471
0.294103	GeneID:100142675
12.30863	GeneID:101122864
18.1067	GeneID:101119652
3.520747	GeneID:101122298
0	GeneID:101111364
2.21227	GeneID:101103230

0	GeneID:101119760
0.140971	GeneID:114118895
201.3961	GeneID:101105583
0.030935	GeneID:101105086
0.075277	GeneID:101103902
1.759065	GeneID:101117254
3.611624	GeneID:101109266
0.025722	GeneID:101109690
0	GeneID:101120720
35.72758	GeneID:101102753
12.41571	GeneID:100169938
13.0809	GeneID:101121297
685.7081	GeneID:100579169
27.79993	GeneID:101110724
5.552392	GeneID:101110482
6.872149	GeneID:101114416
11.46823	GeneID:101114544
25.53456	GeneID:100141301
5.151075	GeneID:443054
1.751673	GeneID:101103813
20.51526	GeneID:101114517
99.48951	GeneID:100037663
0.042291	GeneID:101115496
4.210378	GeneID:101120874
6.235449	GeneID:101113936
0	GeneID:101115371
4.293493	GeneID:101122761
7.99311	GeneID:100568291
0	GeneID:101105897
31.99572	GeneID:101114960
21.51986	GeneID:101119003
5.285042	GeneID:101109136
19.38905	GeneID:101105304
30.42206	GeneID:101106073
9.368981	GeneID:101110854
18.08563	GeneID:101119683
9.767774	GeneID:101116678
4.742559	GeneID:101102343
63.35021	GeneID:101108496
93.19897	GeneID:101113297
3.303361	GeneID:101114076
14.70754	GeneID:101113863
10.65601	GeneID:101109667
9.847526	GeneID:101116974
8.390566	GeneID:101120220
5.388052	GeneID:101119386
10.02737	GeneID:101112549
10.56543	GeneID:101110600
9.66018	GeneID:101122185
14.04922	GeneID:101112643
16.34655	GeneID:101115579
5.68388	GeneID:101112132
31.47256	GeneID:101112499
16.84023	GeneID:101117061
2.463255	GeneID:101122565
2.714451	GeneID:101110583
0.030499	GeneID:101119458
0.506373	GeneID:101108346

0	GeneID:101108609
0.165196	GeneID:100125619
11.90399	GeneID:101112677
2.821904	GeneID:101110000
0.076793	GeneID:101104339
10.54446	GeneID:443165
0.008443	GeneID:101120932
0	GeneID:101112033
6.009649	GeneID:101120330
30.26425	GeneID:101122598
3.84676	GeneID:101115004
2.625573	GeneID:101108802
0.482845	GeneID:105602314
5.630026	GeneID:101110670
8.185738	GeneID:101104350
21.1823	GeneID:101103289
6.04129	GeneID:101115765
17.75532	GeneID:100885767
10.47327	GeneID:101108772
5.732147	GeneID:101103083
0.234066	GeneID:101116862
0.081641	GeneID:101117363
0.465272	GeneID:101108227
135.1771	GeneID:443235
722.5717	GeneID:100135447
9.339311	GeneID:101114124
0.155429	GeneID:101115197
6.954294	GeneID:101113133
2.737575	GeneID:443484
28.83353	GeneID:101120135
40.01357	GeneID:101120835
1.675515	GeneID:101102210
8.891019	GeneID:101121941
11.51208	GeneID:101119009
111.459	GeneID:101122862
100.5157	GeneID:100192316
42.45797	GeneID:101109685
21.97025	GeneID:101113118
0.489337	GeneID:101111416
1.441188	GeneID:101119036
0	GeneID:100500730
0.016132	GeneID:443353
2.279479	GeneID:101120384
0.780194	GeneID:101103643
0.027805	GeneID:101108980
1.131245	GeneID:101102761
0	GeneID:101105570
6.692394	GeneID:101114808
21.40444	GeneID:101120491
0.066211	GeneID:101102585
1.693923	GeneID:101110341
6.714853	GeneID:101120264
1.975723	GeneID:105610737
29.73369	GeneID:101114588
3.997213	GeneID:101111849
14.53582	GeneID:101118615
2.706099	GeneID:101110940
11.09497	GeneID:101102395

9.408415	GeneID:101116420
5.005869	GeneID:101123274
12.33109	GeneID:101102128
11.77604	GeneID:101114439
0	GeneID:101113331
0.423036	GeneID:101112135
0.450141	GeneID:100049064
2.265828	GeneID:101118185
57.39203	GeneID:101103467
0.337869	GeneID:101119758
10.91059	GeneID:101102014
8.73296	GeneID:101121918
5.330268	GeneID:101102055
1.584095	GeneID:101113167
4.158829	GeneID:101110196
0	GeneID:101115314
1.258013	GeneID:101108487
0.109738	GeneID:101106647
0.113975	GeneID:101117337
0.087412	GeneID:443167
0.019438	GeneID:443181
0.490499	GeneID:101107544
69.25325	GeneID:100820752
7.920291	GeneID:101115701
9.24669	GeneID:101110455
42.46204	GeneID:101107708
32.72951	GeneID:101107092
3.872822	GeneID:101108012
30.43959	GeneID:101113686
7.851086	GeneID:101109245
9.099778	GeneID:101107515
4.227874	GeneID:101110121
2.55738	GeneID:100642178
0.452908	GeneID:101108052
8.880065	GeneID:101118619
22.19232	GeneID:101106548
83.28323	GeneID:101109237
0.039448	GeneID:101110713
13.7711	GeneID:101114610
19.67285	GeneID:101106886
6.021505	GeneID:101113642
1.877732	GeneID:101106823
5.223185	GeneID:101117069
58.06498	GeneID:101101945
0.198422	GeneID:101122331
9.758752	GeneID:101116117
8.827608	GeneID:101112412
0.160151	GeneID:101104156
4.93134	GeneID:101115909
3.775277	GeneID:101122344
17.10128	GeneID:101113083
0	GeneID:101111564
30.40137	GeneID:101109953
72.88565	GeneID:101114199
19.92775	GeneID:101122796
43.93172	GeneID:100135435
2.572425	GeneID:101103687
8.084818	GeneID:101110558

4.702883	GeneID:101114978
0.108685	GeneID:101119205
1.584241	GeneID:101119960
10.18165	GeneID:101121656
2.639301	GeneID:101108210
6.257478	GeneID:101106217
1.312225	GeneID:101118875
0	GeneID:101111517
4.25088	GeneID:101117777
0.0138	GeneID:101103212
3.210827	GeneID:101114910
0.013381	GeneID:101102695
0.078042	GeneID:101109223
0.134531	GeneID:101112557
0.433418	GeneID:101113655
82.09807	GeneID:101118648
6.252997	GeneID:101110595
9.234598	GeneID:101102534
9.921629	GeneID:101118611
24.21483	GeneID:101103532
1.997889	GeneID:101119562
0.816201	GeneID:101103321
52.60433	GeneID:101121537
16.77151	GeneID:101117411
37.73359	GeneID:101106560
23.25619	GeneID:101117179
22.18152	GeneID:105609730
0	GeneID:101103410
0.18464	GeneID:105601964
0.60603	GeneID:101121950
0.318242	GeneID:101103514
11.68964	GeneID:100145856
38.75663	GeneID:101111906
2.239184	GeneID:101110345
23.40335	GeneID:101118287
0.318038	GeneID:101118541
11.49504	GeneID:101111557
1.384678	GeneID:100271925
20.16124	GeneID:101119343
6.933854	GeneID:101105539
0.064213	GeneID:101123510
0	GeneID:101108617
0.608438	GeneID:443075
0.450268	GeneID:101121424
6.942399	GeneID:101110421
19.96814	GeneID:101116491
10.27432	GeneID:101102037
1.154076	GeneID:101104416
0.326067	GeneID:101117452
2.106018	GeneID:101104445
0.634286	GeneID:101111396
0.612122	GeneID:101117368
0.639281	GeneID:101102325
0	GeneID:101120927
0.132608	GeneID:105613643
30.19858	GeneID:101113492
18.44636	GeneID:101111161
2.90438	GeneID:105616456

0.256573	GeneID:114118892
6.595408	GeneID:101123478
31.42317	GeneID:443519
11.63043	GeneID:101122066
0.946912	GeneID:100135687
7.217274	GeneID:101117970
0.560827	GeneID:101116490
69.87674	GeneID:101117073
30.99028	GeneID:101102941
7.041495	GeneID:101104395
17.02751	GeneID:101107728
86.76803	GeneID:101104062
11.25508	GeneID:101112539
5.064063	GeneID:101122605
9.535002	GeneID:101117042
22.05715	GeneID:101123327
13.90356	GeneID:101121386
4.041006	GeneID:101111092
33.92729	GeneID:101123406
0.359149	GeneID:101118810
5.628244	GeneID:101117041
3.254969	GeneID:101115835
5.567515	GeneID:101101994
0.533227	GeneID:101101858
4.673695	GeneID:101111929
0	GeneID:101114997
32.48601	GeneID:100036759
2.169871	GeneID:101107690
0.67941	GeneID:101119209
1.663597	GeneID:101107841
6.20677	GeneID:101109588
6.534	GeneID:100192429
10.0519	GeneID:101122911
1.397505	GeneID:114117346
27.72422	GeneID:101111384
4.844018	GeneID:101116706
10.90411	GeneID:100528012
10.29857	GeneID:101112768
1.643946	GeneID:101109289
0.288038	GeneID:101101882
14.46164	GeneID:101104609
30.02967	GeneID:101116309
114.8091	GeneID:101114820
211.5301	GeneID:101112071
100.5356	GeneID:101120892
20.4235	GeneID:101107173
14.83901	GeneID:101119699
0	GeneID:101113541
65.82227	GeneID:101108919
158.6309	GeneID:101122979
0.124997	GeneID:101111590
94.61076	GeneID:114112892
150.2472	GeneID:101115771
29.23881	GeneID:101112400
8.196551	GeneID:101107313
4.814697	GeneID:101108552
9.27777	GeneID:101122520
6.262018	GeneID:101120401

4.274048	GeneID:443128
12.07608	GeneID:101120910
37.36615	GeneID:101102279
0	GeneID:106991217
0.034141	GeneID:101103109
0	GeneID:105606181
0.098986	GeneID:443207
0	GeneID:443198
0.068925	GeneID:100534656
0	GeneID:101109713
5.317376	GeneID:101102876
35.4178	GeneID:101120328
212.9077	GeneID:101120819
232.0027	GeneID:101107187
121.6087	GeneID:101105552
151.8165	GeneID:101103808
90.82747	GeneID:101109972
138.5969	GeneID:100294646
72.25115	GeneID:101114612
101.1662	GeneID:101118755
120.7539	GeneID:101122518
42.13659	GeneID:101106495
34.18201	GeneID:101104959
165.026	GeneID:101117560
149.6602	GeneID:101113093
10.60542	GeneID:101119633
114.1715	GeneID:101104061
49.03757	GeneID:101121577
136.4444	GeneID:101107118
63.8173	GeneID:101122300
59.02662	GeneID:101113371
2.093143	GeneID:101114573
1.968916	GeneID:443063
18.14955	GeneID:101106114
9.035968	GeneID:101106936
12.86751	GeneID:101121520
4.112753	GeneID:101104698
5.756628	GeneID:101106005
19.76922	GeneID:101123347
241.2245	GeneID:101118190
0.032208	GeneID:101108094
0	GeneID:101109914
1.406541	GeneID:101115275
0	GeneID:101105239
0	GeneID:101114267
0.02425	GeneID:101114012
0.464897	GeneID:101106161
1.11254	GeneID:101115026
1.407534	GeneID:101114773
0.039839	GeneID:101114518
0.574961	GeneID:114108616
1.594645	GeneID:105606599
0.052037	GeneID:101105652
2.736459	GeneID:101121362
1.923992	GeneID:101121109
0.317235	GeneID:101115382
2.603875	GeneID:101120592
6.48085	GeneID:101115137

0.54215	GeneID:101120346
0.164971	GeneID:101114883
0.25464	GeneID:101122425
0.100413	GeneID:101108379
0.234716	GeneID:101122680
139.9189	GeneID:101108645
1.74562	GeneID:101108122
2.264601	GeneID:101121669
22.93736	GeneID:443349
10.2457	GeneID:101122179
4.245944	GeneID:443360
0	GeneID:101106056
1.213256	GeneID:101106824
0.372055	GeneID:101104380
0	GeneID:101104641
0.741991	GeneID:101106307
2.911142	GeneID:101103879
5.282686	GeneID:101104131
44.07416	GeneID:101105636
0.718426	GeneID:101110911
10.15891	GeneID:100187549
0.876739	GeneID:101119857
0	GeneID:101122814
0.132858	GeneID:101119084
5.557371	GeneID:101112958
1.608527	GeneID:101116145
1.972605	GeneID:101117692
54.44782	GeneID:100188975
4.38805	GeneID:101110375
5.045186	GeneID:101122241
3.281199	GeneID:101118387
7.550385	GeneID:101119945
1.924495	GeneID:101105705
0.45588	GeneID:101118612
0	GeneID:101117872
122.6443	GeneID:443241
33.95898	GeneID:101104357
0	GeneID:101103619
0.025059	GeneID:101113808
0.227232	GeneID:101104597
2.298221	GeneID:114112801
3.073184	GeneID:101121717
0.062806	GeneID:443190
0	GeneID:101121700
0.331534	GeneID:101118153
0.213794	GeneID:105606344
9.661979	GeneID:101107346
5.28031	GeneID:101117803
1.777033	GeneID:101104909
7.444619	GeneID:101116370
0.03857	GeneID:101105173
0.061009	GeneID:101119692
0.021851	GeneID:101107448
24.09756	GeneID:105604608
22.30172	GeneID:105611800
6.400733	GeneID:101110034
0.099036	GeneID:101110949
67.56798	GeneID:101122048

18.05309	GeneID:105604590
10.98296	GeneID:101106463
10.0387	GeneID:443409
0.71636	GeneID:101109443
0.869787	GeneID:443530
0.344714	GeneID:101110778
71.84161	GeneID:101119880
4.161321	GeneID:101122787
6.033465	GeneID:101110086
4.487387	GeneID:101122359
10.922	GeneID:101123575
5.894193	GeneID:100913161
0.131463	GeneID:101111157
31.28355	GeneID:101114519
8.572789	GeneID:101110013
26.9924	GeneID:101116612
0	GeneID:100190978
5.341601	GeneID:101105716
6.112743	GeneID:101102168
23.80082	GeneID:101110995
22.7867	GeneID:101109368
8.908165	GeneID:101121861
0.575125	GeneID:101106734
2.061578	GeneID:101110783
0.353374	GeneID:101105087
0.260055	GeneID:101117244
544.8354	GeneID:100127209
441.7839	GeneID:101117797
219.8195	GeneID:101111703
2.898046	GeneID:101120879
7.778014	GeneID:101121442
12.44083	GeneID:101118151
25.41736	GeneID:101108674
31.17854	GeneID:100913152
2.202762	GeneID:101117117
32.98877	GeneID:101110063
39.81181	GeneID:101122782
21.51568	GeneID:101122219
195.1138	GeneID:780447
0.849104	GeneID:101107064
376.5806	GeneID:101112844
70.20105	GeneID:101112054
804.2783	GeneID:101115754
5.537718	GeneID:101120452
2.027473	GeneID:101107895
0.047956	GeneID:101118384
11.98918	GeneID:101104335
0.350043	GeneID:114113264
143.1091	GeneID:101108164
0	GeneID:101121786
1.891663	GeneID:101114835
9.2502	GeneID:101121446
53.31167	GeneID:101122254
7.914148	GeneID:101122677
25.5894	GeneID:101110772
26.78676	GeneID:101114318
27.19124	GeneID:101104556
1.499587	GeneID:101122389

3.020641	GeneID:101111375
0.420388	GeneID:101122172
0	GeneID:101114783
0.268838	GeneID:101121647
0.031647	GeneID:443018
1.885769	GeneID:101106827
0	GeneID:101108550
0.02694	GeneID:101110536
0.053032	GeneID:101117928
0	GeneID:101104894
0.167572	GeneID:101122994
128.7155	GeneID:101101854
11.42002	GeneID:101120228
15.63045	GeneID:101119395
2.309541	GeneID:101119533
10.21054	GeneID:100216476
5.683797	GeneID:101114080
7.735974	GeneID:101113059
22.85935	GeneID:101119368
2.193712	GeneID:101104785
13.01561	GeneID:101121207
8.993592	GeneID:443154
2.744535	GeneID:101121461
0.012529	GeneID:101106519
0.008958	GeneID:101115477
8.093984	GeneID:101107657
1.137959	GeneID:101122555
2.100153	GeneID:101112773
13.14067	GeneID:101105848
27.46135	GeneID:101111231
15.63545	GeneID:101117317
29.56027	GeneID:101109674
29.85689	GeneID:101122877
4.142444	GeneID:101122106
0	GeneID:101120495
13.69117	GeneID:101121340
0.221083	GeneID:101111524
9.793842	GeneID:443045
1.296399	GeneID:101113326
0	GeneID:101116476
0.094246	GeneID:101116726
4.886819	GeneID:101107734
3.375759	GeneID:101113762
5.31596	GeneID:101107478
13.86605	GeneID:101117687
1.08291	GeneID:101113567
4.947036	GeneID:101102203
74.10605	GeneID:101111650
51.77945	GeneID:101115522
84.71439	GeneID:100415775
48.75644	GeneID:101122061
21.90687	GeneID:101104194
59.59186	GeneID:443257
31.10504	GeneID:101102843
27.83386	GeneID:101120868
53.32831	GeneID:101104080
48.11515	GeneID:101110910
5.352681	GeneID:101115944

1.418431	GeneID:100196903
21.66467	GeneID:101116540
3.104953	GeneID:101105916
43.2283	GeneID:101103471
92.43625	GeneID:101108867
13.77557	GeneID:101114073
19.26671	GeneID:101120181
3.40569	GeneID:101105148
0.676057	GeneID:101110140
22.25571	GeneID:101105641
33.62361	GeneID:100534647
8.611385	GeneID:101108064
0.833756	GeneID:101110473
2.385854	GeneID:101103357
15.11826	GeneID:101106828
7.285936	GeneID:101106234
7.452881	GeneID:101102194
7.305875	GeneID:101101941
4.95761	GeneID:101121899
14.22601	GeneID:101102441
0.152799	GeneID:101122399
0.858392	GeneID:101104441
9.18712	GeneID:443126
5.557303	GeneID:443363
0.01809	GeneID:101104211
0.142793	GeneID:443396
26.42893	GeneID:101112907
40.81941	GeneID:101122484
0	GeneID:101111520
1.939694	GeneID:101119890
18.19567	GeneID:101112558
15.1752	GeneID:101115914
4.633559	GeneID:101123504
3.499749	GeneID:101109504
4.41625	GeneID:101118681
26.16694	GeneID:101103579
8.374453	GeneID:101120796
9.721943	GeneID:100145882
1.664709	GeneID:101111886
14.26506	GeneID:101102172
4.045001	GeneID:101107376
11.90278	GeneID:101111421
4.832908	GeneID:101110029
5.134143	GeneID:101106299
4.759864	GeneID:101104093
6.293272	GeneID:101118114
16.12545	GeneID:101121748
0.526381	GeneID:101111458
1.904015	GeneID:101111715
3.935228	GeneID:443318
24.81584	GeneID:443515
8.108388	GeneID:443325
0.013255	GeneID:101113253
8.003832	GeneID:101115925
0.198772	GeneID:101110498
12.84359	GeneID:443500
1.737626	GeneID:101111599
0.628237	GeneID:443132

13.36545	GeneID:443469
80.01699	GeneID:100286737
66.40936	GeneID:443470
70.10568	GeneID:443133
55.81697	GeneID:100187551
178.3075	GeneID:100270713
0.093111	GeneID:101105881
4.887757	GeneID:101108594
0	GeneID:101109031
4.133979	GeneID:101114988
4.109934	GeneID:101117329
0.878266	GeneID:101121878
0.044587	GeneID:101111779
1.378171	GeneID:101116950
0.164393	GeneID:101104446
0.036203	GeneID:101106668
0	GeneID:101121384
0	GeneID:101110700
2.665445	GeneID:101110899
1.678211	GeneID:101123596
1.259016	GeneID:101104452
11.17756	GeneID:101102283
3.346642	GeneID:101116526
0.211281	GeneID:101119518
0	GeneID:101113658
87.50596	GeneID:101119211
5.035868	GeneID:101123198
7.213652	GeneID:780484
2.7408	GeneID:101116923
4.100522	GeneID:101107332
1.321887	GeneID:101107435
1.348958	GeneID:101103788
0.5605	GeneID:101104255
0.766195	GeneID:101104142
4.44535	GeneID:101113292
0	GeneID:103611088
0.70141	GeneID:443342
1.879528	GeneID:101120946
5.818069	GeneID:105609264
0.333325	GeneID:114118064
9.043258	GeneID:101120724
0	GeneID:443064
0.028624	GeneID:443472
0.142013	GeneID:101110054
0.041289	GeneID:100820736
0.0218	GeneID:664707
53.34603	GeneID:443518
0.060442	GeneID:101108025
0.405831	GeneID:443056
3.623214	GeneID:101107377
1.614856	GeneID:101116938
0	GeneID:101103931
0.661691	GeneID:101119984
0	GeneID:101107039
1.255693	GeneID:114116619
0.022178	GeneID:101104186
17.02633	GeneID:101116821
0.392883	GeneID:101105858

8.959941	GeneID:101123416
1.496402	GeneID:101109490
6.463787	GeneID:101123669
16.79224	GeneID:443206
0.084185	GeneID:101102552
0.303023	GeneID:101109262
0.054374	GeneID:101112663
1.384928	GeneID:443404
0.374778	GeneID:443539
103.0212	GeneID:101111084
15.74553	GeneID:101103054
59.62076	GeneID:101103308
0.005775	GeneID:101117137
0.053421	GeneID:101101957
0.891397	GeneID:101109529
8.529067	GeneID:101102800
19.78015	GeneID:780505
0.048899	GeneID:443401
0	GeneID:101112916
17.08747	GeneID:101112653
43.57915	GeneID:101113034
0.065847	GeneID:100820753
1.022759	GeneID:101118168
0.279055	GeneID:101121781
0.091746	GeneID:100462692
0.09722	GeneID:101103528
0	GeneID:101113424
0.642283	GeneID:100499506
0	GeneID:101107268
0.126747	GeneID:101122495
26.21803	GeneID:101102232
1.733782	GeneID:443435
1.410383	GeneID:101107088
10.54814	GeneID:101103538
0.057776	GeneID:101113274
23.19648	GeneID:101116705
1.240958	GeneID:101113261
0.027587	GeneID:101105996
1.39217	GeneID:101106514
17.35861	GeneID:101111354
4.236464	GeneID:101113991
0	GeneID:101122781
3.551722	GeneID:105602079
4.043548	GeneID:101109063
0	GeneID:443350
0.114696	GeneID:101111837
0.051164	GeneID:443406
11.21226	GeneID:101103369
33.98383	GeneID:101113174
1.385212	GeneID:443341
3.335204	GeneID:101111126
0	GeneID:101105494
1.940987	GeneID:101112275
0.401157	GeneID:101110204
83.86557	GeneID:101106476
21.618	GeneID:101118785
46.25311	GeneID:100913157
32.3754	GeneID:101109322

22.54293	GeneID:101120571
19.77975	GeneID:101119503
3.814385	GeneID:101113581
26.08731	GeneID:101105896
18.00811	GeneID:101117969
15.67376	GeneID:101104207
7.009357	GeneID:101119912
23.27061	GeneID:101105968
4.132671	GeneID:101118403
30.01541	GeneID:101109546
19.48468	GeneID:101104144
59.02904	GeneID:101107823
0.852692	GeneID:101109327
0	GeneID:101115747
9.436318	GeneID:114118014
2.313503	GeneID:105608914
6.341744	GeneID:101108243
0.098805	GeneID:101107802
3.586567	GeneID:101110982
8.748108	GeneID:101103676
14.41877	GeneID:101116998
3.335289	GeneID:101108806
5.92889	GeneID:101102722
33.07306	GeneID:101111956
7.467637	GeneID:101121736
0.330169	GeneID:101118082
2.869273	GeneID:443524
14.91929	GeneID:101119286
0	GeneID:101121588
19.55524	GeneID:101110478
7.968911	GeneID:101116942
3.164396	GeneID:101107251
7.567321	GeneID:101112063
6.758337	GeneID:101106673
15.65029	GeneID:101110106
2.496062	GeneID:101115188
4.44298	GeneID:101117296
8.043381	GeneID:101110908
8.093014	GeneID:101109605
1.510478	GeneID:101112848
35.3533	GeneID:101110014
12.97333	GeneID:101119358
2.584035	GeneID:101112941
2.499847	GeneID:101115530
6.73263	GeneID:101122650
0.056457	GeneID:101103674
15.55627	GeneID:101115806
14.10927	GeneID:101121707
0	GeneID:114110121
0.73878	GeneID:101116598
23.84338	GeneID:101106422
11.77099	GeneID:101110569
0	GeneID:554252
0	GeneID:101113287
0	GeneID:101114333
0	GeneID:105609016
0.020529	GeneID:101104274
7.221496	GeneID:443431

0.005501	GeneID:101108266
0.910415	GeneID:101111635
0.138485	GeneID:101116947
5.94921	GeneID:101114160
20.72268	GeneID:101102286
32.91025	GeneID:101104768
12.28592	GeneID:101104238
9.469196	GeneID:101112801
5.324528	GeneID:101121097
6.007857	GeneID:101107292
19.26458	GeneID:101106729
4.698064	GeneID:101110460
5.355077	GeneID:101112174
14.36109	GeneID:101119914
7.971629	GeneID:101117951
20.26889	GeneID:101109456
6.796243	GeneID:101116454
0.789627	GeneID:101122726
4.185276	GeneID:101122000
15.99896	GeneID:101102341
33.32653	GeneID:101109567
0	GeneID:101118907
0.542882	GeneID:101107028
3.306843	GeneID:101120714
8.121158	GeneID:101106794
13.20877	GeneID:101113708
8.152196	GeneID:101120166
64.67819	GeneID:101109716
55.98942	GeneID:101105184
14.06687	GeneID:101108038
19.55021	GeneID:101113164
2.672684	GeneID:101122737
3.156355	GeneID:101107928
1.903332	GeneID:105613386
0.00681	GeneID:101103100
4.074623	GeneID:101110893
8.126047	GeneID:114108637
0.684938	GeneID:101113102
3.794731	GeneID:101111855
0.026774	GeneID:101115577
0	GeneID:101116353
0.250364	GeneID:101119097
0.005329	GeneID:101112486
0.304827	GeneID:101118754
0.049573	GeneID:101118657
0.114594	GeneID:101111020
80.83826	GeneID:101104610
14.44178	GeneID:101105506
1.22377	GeneID:101108447
6.173781	GeneID:105606122
3.711282	GeneID:101109678
0.004268	GeneID:101114514
0.090239	GeneID:101105744
7.383544	GeneID:101121405
3.736303	GeneID:101111638
1.712127	GeneID:101122222
2.441052	GeneID:101115273
4.511813	GeneID:100216438

7.810528	GeneID:101122309
12.7099	GeneID:443136
15.5117	GeneID:443082
10.93247	GeneID:101119664
34.76026	GeneID:101113906
19.0742	GeneID:101109807
10.87463	GeneID:780485
0.521592	GeneID:101111234
3.142249	GeneID:101105408
72.00739	GeneID:443083
1.27455	GeneID:101103683
1.607647	GeneID:101105432
8.844928	GeneID:101116656
14.15519	GeneID:101107040
3.776628	GeneID:101102293
11.30806	GeneID:106991367
0.104484	GeneID:101112456
25.70126	GeneID:101108256
30.83546	GeneID:101102588
7.759462	GeneID:101118126
9.40668	GeneID:101111127
7.771406	GeneID:101118378
2.934917	GeneID:101118892
32.99512	GeneID:101114490
5.622226	GeneID:105606505
43.21975	GeneID:101103093
2.123992	GeneID:443057
1.460218	GeneID:101116513
13.76559	GeneID:101107241
0	GeneID:541604
0	GeneID:101103270
10.96171	GeneID:101110878
0.013597	GeneID:101111321
45.05471	GeneID:101105349
0.014579	GeneID:101111103
29.48764	GeneID:101102483
11.89958	GeneID:101120012
48.71017	GeneID:101102921
0	GeneID:101103802
9.215662	GeneID:101109704
21.41356	GeneID:101118041
27.2447	GeneID:101110080
2.397719	GeneID:101119126
7.054724	GeneID:101114307
0.882749	GeneID:101117377
3.564246	GeneID:101105162
10.23898	GeneID:101119848
0.457806	GeneID:101115719
12.51121	GeneID:101118038
2.658143	GeneID:443499
7.123717	GeneID:443140
48.22012	GeneID:101113656
19.4674	GeneID:101115445
3.248277	GeneID:101117689
2.659705	GeneID:101109832
0.40384	GeneID:101105872
1.554247	GeneID:101110688
0.973824	GeneID:641296

1.249824	GeneID:443217
18.99915	GeneID:443143
0.550845	GeneID:101106739
140.2003	GeneID:443141
21.8931	GeneID:101116297
0.065401	GeneID:101106491
3.429391	GeneID:494433
1.416356	GeneID:100423047
4.082026	GeneID:101108702
29.76758	GeneID:101119919
44.27271	GeneID:101120887
5.115147	GeneID:443209
2.084251	GeneID:101109956
3.121384	GeneID:101107517
0	GeneID:101114796
0	GeneID:101109217
3.096783	GeneID:101108959
15.78747	GeneID:101114543
2.414046	GeneID:101112899
0.093188	GeneID:101122151;GeneID:100125357
1503.1	GeneID:100174988
82.47362	GeneID:100174906
21.4745	GeneID:101121956
3.549776	GeneID:101115161
0.81307	GeneID:101115627
19.02509	GeneID:101113512
15.74562	GeneID:101120515
2.930449	GeneID:101108864
20.13884	GeneID:114109674
0.097819	GeneID:101109875
39.25277	GeneID:101120052
3.574782	GeneID:101115746
5.138441	GeneID:101112636
3.626785	GeneID:101112279
8.055584	GeneID:101120620
13.22395	GeneID:101108644
11.57112	GeneID:101113856
69.30473	GeneID:101123427
39.42633	GeneID:101108935
16.46451	GeneID:101109331
0	GeneID:101123564
2.435481	GeneID:100302705
0	GeneID:101113263
6.129702	GeneID:101122026
10.2782	GeneID:101119334
13.5661	GeneID:101103318
3.663819	GeneID:101118166
45.76518	GeneID:780486
22.43871	GeneID:105605912
18.72727	GeneID:101102095
68.76659	GeneID:101110716
8.575983	GeneID:101113909
1.177122	GeneID:101112816
0.087147	GeneID:101113239
0	GeneID:101115029
0.444679	GeneID:101108708
7.497222	GeneID:101118411
4.998908	GeneID:101117375

3.670386	GeneID:101121705
11.09714	GeneID:101113542
4.72157	GeneID:101113233
3.221856	GeneID:101116590
12.40696	GeneID:101121603
2.247245	GeneID:101108494
0	GeneID:101112344
7.319359	GeneID:101102487
13.7639	GeneID:101123089
6.792735	GeneID:101103319
3.612873	GeneID:101103656
5.871858	GeneID:101116914
20.8011	GeneID:101104116
28.89723	GeneID:101108836
24.78946	GeneID:101105900
9.727712	GeneID:101110355
0.395966	GeneID:101117255
2.59314	GeneID:101105592
0.10414	GeneID:101121939
27.33983	GeneID:101111302
24.72175	GeneID:101110020
7.354042	GeneID:101122885
0	GeneID:101108116
27.77892	GeneID:101102365
43.62422	GeneID:443219
93.61979	GeneID:443117
40.86376	GeneID:443102
196.6336	GeneID:101119486
1871.707	GeneID:100294614
8.488149	GeneID:100141297
31.66217	GeneID:101109511
22.4059	GeneID:101117071
2.292669	GeneID:101104065
1.364512	GeneID:101106979
13.5015	GeneID:101121022
4.383949	GeneID:101122579
21.28613	GeneID:101106671
9.106169	GeneID:101111870
250.3982	GeneID:101118293
10.81364	GeneID:101120935
11.55687	GeneID:101109985
22.19402	GeneID:101115756
19.53387	GeneID:101109495
13.88932	GeneID:101121826
5.922657	GeneID:101123266
25.37312	GeneID:101110071
18.90407	GeneID:101104546
24.01846	GeneID:101122604
2.819823	GeneID:101112580
0	GeneID:101113378
7.451067	GeneID:101103087
4.059623	GeneID:101123043
1.17252	GeneID:101112689
2.407488	GeneID:105612179
0.845508	GeneID:101107230
0	GeneID:101110418
19.94328	GeneID:101120743
1.735537	GeneID:101115566

3.089491	GeneID:101118557
0.795076	GeneID:101122871
3.37249	GeneID:101118139
22.71073	GeneID:101122775
0.09794	GeneID:105614925
0	GeneID:101111758
0.036166	GeneID:101112012
0.880675	GeneID:101105466
0.012257	GeneID:101118217
0.104049	GeneID:101111448
1.082167	GeneID:105608581
0.016998	GeneID:101109038
0.316532	GeneID:101110988
1.437109	GeneID:101122538
0.459305	GeneID:101120425
0.063338	GeneID:101121535
0.044067	GeneID:101106337
0	GeneID:101113880
0.064118	GeneID:101122852
0.088295	GeneID:101116251
4.514652	GeneID:101105210
0.375315	GeneID:101106661
0.329655	GeneID:101122690
1.921187	GeneID:101115919
1.204551	GeneID:101119094
0	GeneID:101119863
1.498717	GeneID:101106359
0.597086	GeneID:101120989
0	GeneID:101114087
0	GeneID:101103556
0.012479	GeneID:101123485
0.273836	GeneID:101107566
0.103742	GeneID:101115443
1.45627	GeneID:101102663
0.083277	GeneID:101120348
0.055733	GeneID:101106315
0.061347	GeneID:101118435
0	GeneID:101110253
0	GeneID:101106850
0	GeneID:101102319
0.232122	GeneID:101120717
1.439721	GeneID:101105527
0.856942	GeneID:101103859
4.029193	GeneID:101114598
0.010833	GeneID:101101984
0.09705	GeneID:101117892
0.33749	GeneID:101114135
0.073867	GeneID:101123388
0.136984	GeneID:101106197
4.232348	GeneID:101117981
0.011541	GeneID:101114792
0.697723	GeneID:101114538
0.059497	GeneID:101115095
0.019167	GeneID:101104736
0	GeneID:101116101
3.655013	GeneID:101118008
0.430851	GeneID:101102032
7.023205	GeneID:101115679

0.253584	GeneID:101113681
0	GeneID:101114768
0.19231	GeneID:114115663
0.262416	GeneID:101106869
0	GeneID:101105697
0.068908	GeneID:101121389
4.639239	GeneID:101111729
0.093188	GeneID:100141302
0.325995	GeneID:101116360
2.64781	GeneID:101103020
5.995543	GeneID:101110698
13.94698	GeneID:101108703
0.02295	GeneID:101122116
3.305807	GeneID:100270685
4.884169	GeneID:100302624
0.047338	GeneID:100620053
0.104029	GeneID:101109757
5.337715	GeneID:100620050
0.593695	GeneID:101112056
0.286501	GeneID:101105660
0.622213	GeneID:101112614
0.126079	GeneID:101108597
1.013427	GeneID:101119596
0.002987	GeneID:101118630
0.042011	GeneID:101120673
0.110972	GeneID:101122655
0.201218	GeneID:100174987
0.385249	GeneID:101118623
0.017487	GeneID:101106338
0.528816	GeneID:101101966
0.197025	GeneID:101118349
0.199021	GeneID:101115980
0.45831	GeneID:101110209
0	GeneID:101117169
0	GeneID:101117399
0.457561	GeneID:101105154
16.12386	GeneID:101117127
38.1795	GeneID:101114309
16.45527	GeneID:101119573
20.30014	GeneID:101102499
4.450717	GeneID:101118408
3.607777	GeneID:101116522
11.3546	GeneID:101111479
0.024191	GeneID:101110839
4.108536	GeneID:101106839
1.690127	GeneID:101104044
0.009477	GeneID:101113002
20.95825	GeneID:101122963
36.81128	GeneID:101120959
8.778384	GeneID:101109401
13.99339	GeneID:101120594
5.5425	GeneID:101111108
23.00991	GeneID:101116367
4.517339	GeneID:114108613
3.129553	GeneID:101110559
0.02574	GeneID:101119822
14.81283	GeneID:101111427
7.582082	GeneID:101114030

35.42544	GeneID:101122047
43.1263	GeneID:101116624
18.19681	GeneID:101121418
7.940243	GeneID:101114250
42.23518	GeneID:101121409
8.183698	GeneID:101122986
20.71803	GeneID:101108003
8.306825	GeneID:101103759
15.66759	GeneID:101106756
18.55378	GeneID:101111280
17.53922	GeneID:101111840
12.6056	GeneID:101110232
4.366818	GeneID:101117992
11.73926	GeneID:101115023
35.73837	GeneID:101110351
28.48366	GeneID:101121317
13.45693	GeneID:101103543
7.188094	GeneID:101119656
10.36805	GeneID:101111627
1.340623	GeneID:101108800
19.89646	GeneID:443087
16.8057	GeneID:101119868
13.5335	GeneID:101113845
0.739689	GeneID:101121343
12.02565	GeneID:101112715
0	GeneID:101123050
23.03638	GeneID:101121220
43.37545	GeneID:101117825
0.034454	GeneID:101105784
1.453281	GeneID:101119650
1.91715	GeneID:101113479
11.3334	GeneID:101112733
39.80063	GeneID:101109446
25.85962	GeneID:105612224
11.69305	GeneID:101105928
9.302417	GeneID:101108655
0.381542	GeneID:101118313
12.65526	GeneID:101109922
14.87014	GeneID:101119330
4.969768	GeneID:101119677
5.354776	GeneID:101104418
3.103989	GeneID:101120464
3.54626	GeneID:101104412
6.181095	GeneID:105603997
1.239579	GeneID:101107863
0.291322	GeneID:101116307
0.503713	GeneID:101111991
32.21735	GeneID:101108740
2.4755	GeneID:101102944
5.011569	GeneID:101121299
3.631877	GeneID:101112644
21.66528	GeneID:101108135
0	GeneID:101106573
0.80222	GeneID:101108572
5.581552	GeneID:101111874
7.246704	GeneID:101105016
0.234273	GeneID:105603599
2.546592	GeneID:101103105

0.267206	GeneID:101112642
1.345971	GeneID:101115087
6.555498	GeneID:101105225
3.567556	GeneID:101109533
0.4078	GeneID:101102174
2.64818	GeneID:106991493
3.465546	GeneID:101117110
3.108408	GeneID:101123397
1.750611	GeneID:101110929
0.602034	GeneID:101110732
0.368566	GeneID:101110481
5.444946	GeneID:101119701
33.06961	GeneID:101115774
9.871812	GeneID:101102946
0.205908	GeneID:101119620
7.922165	GeneID:101112010
6.534572	GeneID:101109076
2.00387	GeneID:101107720
2.649584	GeneID:101121554
2.158806	GeneID:101111986
0.074431	GeneID:101101877
2.271315	GeneID:101117696
2.659103	GeneID:101118461
0.044342	GeneID:101106188
0.627915	GeneID:101103691
12.71292	GeneID:101115220
13.35074	GeneID:101107066
66.12704	GeneID:101107535
9.620593	GeneID:101109352
4.739445	GeneID:101122564
18.95394	GeneID:101119971
0.573625	GeneID:101108506
9.89725	GeneID:101120211
5.169123	GeneID:101114023
1.646652	GeneID:101123527
0.057638	GeneID:101116060
3.349647	GeneID:106991730
0.56332	GeneID:101106609
1.887618	GeneID:101118416
10.93277	GeneID:101107307
0.013746	GeneID:101117942
5.814078	GeneID:101117203
9.615355	GeneID:101122522
8.908467	GeneID:101104261
3.042174	GeneID:101109434
4.627689	GeneID:101123442
0.098638	GeneID:101119383
60.62978	GeneID:100037703
0.095357	GeneID:101113663
0.041082	GeneID:101106209
3.513123	GeneID:101103926
0.588255	GeneID:101113963
21.84549	GeneID:101102827
8.058016	GeneID:101113367
0.957806	GeneID:101107533
10.2333	GeneID:101103342
4.609931	GeneID:101115052
12.02989	GeneID:101113647

0.009643	GeneID:101117442
0.312061	GeneID:101114734
0.051967	GeneID:101107719
0.767938	GeneID:100144753
4.132646	GeneID:780504
15.5596	GeneID:443371
5.950914	GeneID:101102003
0.136252	GeneID:101110249
0.058074	GeneID:101109707
27.96325	GeneID:101102677
4.691288	GeneID:101104701
65.19715	GeneID:101116082
6.152438	GeneID:101113441
0.150085	GeneID:101122437
32.30489	GeneID:101104073
6.376446	GeneID:101117833
1.654285	GeneID:101122799
11.9492	GeneID:101110272
0.052795	GeneID:105607536
0.765474	GeneID:101115319
7.60097	GeneID:105607079
0.018482	GeneID:101108444
6.814504	GeneID:101118186
17.0057	GeneID:101111966
51.75849	GeneID:100302636
33.44068	GeneID:101123641
39.69702	GeneID:101111475
5.970152	GeneID:101117396
0.262588	GeneID:101114934
9.395518	GeneID:101104718
2.473079	GeneID:101123469
12.64458	GeneID:101106423
43.17044	GeneID:101120247
29.84807	GeneID:101112265
7.400422	GeneID:101105271
0.262851	GeneID:101110125
3.721155	GeneID:101110350
1.16022	GeneID:101106208
0.538632	GeneID:101101852
0	GeneID:101121433
7.863993	GeneID:101120177
8.523256	GeneID:101104167
1.494773	GeneID:101106406
1.126352	GeneID:101117636
2.518459	GeneID:101114417
2.744736	GeneID:101108068
15.46428	GeneID:101123077
7.09447	GeneID:101120953
7.321432	GeneID:101113256
12.40453	GeneID:101120765
2.90957	GeneID:101119851
11.62991	GeneID:101102691
6.016551	GeneID:101121124
1.069281	GeneID:101108387
4.829858	GeneID:101105190
4.181185	GeneID:101104975
0.588543	GeneID:101107091
0.007517	GeneID:101109583

0.014151	GeneID:101114565
0.383929	GeneID:101105257
1.072813	GeneID:101115711
0.031952	GeneID:101106663
0.032319	GeneID:101106624
4.835378	GeneID:101103833
0.49107	GeneID:101107608
2.443004	GeneID:101108287
0.165127	GeneID:101119589
0.009157	GeneID:101120303
4.902953	GeneID:101111011
1.93274	GeneID:101112731
0.36975	GeneID:101102950
4.648251	GeneID:101109089
2.816274	GeneID:101122109
11.94802	GeneID:101105389
0.035835	GeneID:100048991
278.4946	GeneID:101112250
11.10348	GeneID:101113783
19.85756	GeneID:101113003
5.071578	GeneID:101120106
0.409315	GeneID:101114802
36.48031	GeneID:101112504
27.35703	GeneID:101113520
156.0635	GeneID:101115562
0.98132	GeneID:101112755
0.919122	GeneID:101114298
0.051586	GeneID:101116376
0.112103	GeneID:101121250
0	GeneID:105612570
2.240588	GeneID:101110585
1.738134	GeneID:101121026
6.245045	GeneID:101101916
7.355892	GeneID:101104086
11.92067	GeneID:101115617
5.764992	GeneID:101120741
21.2898	GeneID:101121766
34.38304	GeneID:101122308
12.13985	GeneID:101121812
4.365852	GeneID:101121289
0	GeneID:101103851
0	GeneID:101110807
0.048492	GeneID:101112781
2.699752	GeneID:101114616
5.0822	GeneID:101118407
13.76763	GeneID:105608916
2.724959	GeneID:101120018
15.24433	GeneID:101113813
41.37533	GeneID:101106952
8.784269	GeneID:101117855
35.47845	GeneID:101105546
0.747523	GeneID:101104498
20.38936	GeneID:101117141
0	GeneID:101114078
40.91169	GeneID:101118967
23.56638	GeneID:101121142
7.695623	GeneID:101102417
19.04715	GeneID:101114005

2.571039	GeneID:101108383
1.070457	GeneID:101108063
42.2407	GeneID:101108468
42.38701	GeneID:101120442
1.288324	GeneID:101119273
12.14671	GeneID:101114104
10.65485	GeneID:101110497
15.06165	GeneID:101122012
237.4549	GeneID:101109430
274.064	GeneID:101106346
0.060703	GeneID:101106606
1129.092	GeneID:100303604
15.14329	GeneID:101108117
173.3243	GeneID:101117946
1.371517	GeneID:101109688
1439.86	GeneID:443415
0.014312	GeneID:101111109
0.118574	GeneID:101106091
108.1671	GeneID:101111379
0.04459	GeneID:101110156
3052.59	GeneID:443079
137.48	GeneID:101110427
1278.834	GeneID:100141298
404.1491	GeneID:443080
0.070828	GeneID:101111010
197.4238	GeneID:100526788
540.268	GeneID:100141296
22.76125	GeneID:101117175
116.2052	GeneID:100526786
0.139498	GeneID:101108902
54.60787	GeneID:100526785
1898.626	GeneID:100303605
389.0304	GeneID:101112304
30.53157	GeneID:101112716
1839.591	GeneID:101112045
58.34505	GeneID:101110490
70.27921	GeneID:101109161
1.025149	GeneID:101108376
200.9263	GeneID:101108641
61.30151	GeneID:101110495
102.8928	GeneID:101113319
114.4326	GeneID:100526789
51.02887	GeneID:100526798
0.368078	GeneID:101107880
1784.882	GeneID:100294612
1487.826	GeneID:100294613
32.3584	GeneID:105611408
175.9393	GeneID:100301913
110.0456	GeneID:443414
1617.775	GeneID:100499198
93.36501	GeneID:101116525
5.074951	GeneID:101112044
534.6605	GeneID:101107125
0.006663	GeneID:101110179
7.481808	GeneID:101113710
45.37023	GeneID:101119479
44.59158	GeneID:101108222
1.263002	GeneID:101113821

13.35484	GeneID:101119265
0.279217	GeneID:101101867
2.906512	GeneID:101110032
2.05042	GeneID:101121015
3.579623	GeneID:101102239
0.730932	GeneID:101104848
8.08761	GeneID:101102895
4.353148	GeneID:101119314
6.49764	GeneID:101109625
7.077009	GeneID:101101990
15.35336	GeneID:101104760
27.35714	GeneID:101114853
0.262497	GeneID:101109350
6.42424	GeneID:101119118
0.634269	GeneID:101118515
0	GeneID:443386
0.035808	GeneID:101102692
26.01981	GeneID:101117424
15.30243	GeneID:101120460
22.2747	GeneID:101107969
10.93629	GeneID:101121444
39.98412	GeneID:101105743
17.1448	GeneID:101105176
35.75989	GeneID:101105588
10.11916	GeneID:100216328
13.85281	GeneID:101122429
189.8742	GeneID:101119403
34.05502	GeneID:101113307
0.307058	GeneID:101103444
0	GeneID:101107458
34.96466	GeneID:101119502
42.11569	GeneID:101122907
42.69574	GeneID:101110413
27.57851	GeneID:101106991
49.60163	GeneID:101111239
33.10683	GeneID:101102292
10.42432	GeneID:101108262
1.145076	GeneID:101120057
230.9362	GeneID:101120750
404.9804	GeneID:101103549
35.19401	GeneID:101107107
32.32289	GeneID:101121575
5.220842	GeneID:101120155
6.522466	GeneID:101117447
35.68513	GeneID:101112568
1.676617	GeneID:101120524
5.260251	GeneID:101118601
10.87055	GeneID:101112750
12.34616	GeneID:101114526
13.54637	GeneID:101116230
7.877084	GeneID:101112134
4.808826	GeneID:101119512
32.3512	GeneID:101112288
47.59815	GeneID:101121869
1.806722	GeneID:101110725
3.138541	GeneID:101114839
9.802624	GeneID:101122867
8.390106	GeneID:101117941

2.758189	GeneID:101107386
72.06725	GeneID:101103634
0	GeneID:114109055
0.095966	GeneID:101104595
30.75562	GeneID:101110348
0	GeneID:101113186
0.095316	GeneID:101118683
1.305492	GeneID:101113154
0	GeneID:101123084
11.80392	GeneID:100302343
2.301142	GeneID:100216439
6.016757	GeneID:101103884
14.69762	GeneID:101107755
2.608908	GeneID:101110182
0	GeneID:101120313
0	GeneID:101106513
2.810353	GeneID:101121411
0.038217	GeneID:101106762
2.976036	GeneID:101112525
0.892752	GeneID:101104320
14.02283	GeneID:101112579
2.591709	GeneID:101105277
0.012328	GeneID:101104042
[truncated: 369,361 more chars]
