# Supplementary figures and images for: Transcriptome and proteome combined analysis of wool fiber diameter regulation mechanism
Source: Anim Biosci. 2025 Sep 30;39(2):250378. doi: 10.5713/ab.25.0378 (PMC12877398; doi:10.5713/ab.25.0378)

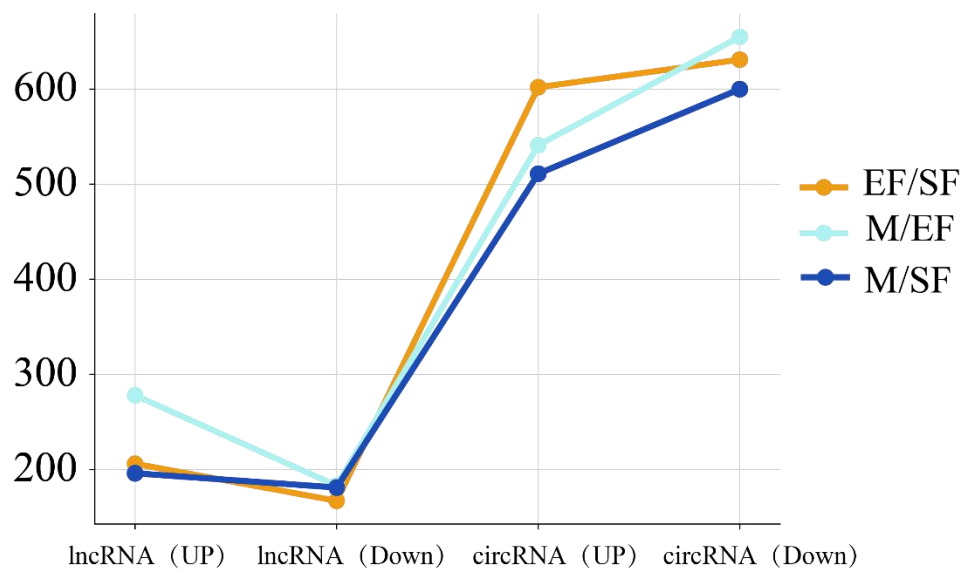

Supplementary 5. Summary of differential circRNA and differential lncRNA

Supplement: Supplementary file 4 [file ab-25-0378-Supplementary-5.pdf]
