# Supplementary material for: Transcriptome and proteome combined analysis of wool fiber diameter regulation mechanism
Source: Anim Biosci. 2025 Sep 30;39(2):250378. doi: 10.5713/ab.25.0378 (PMC12877398; doi:10.5713/ab.25.0378)
Supplement: Supplementary file 7 [file ab-25-0378-Supplementary-8.pdf]

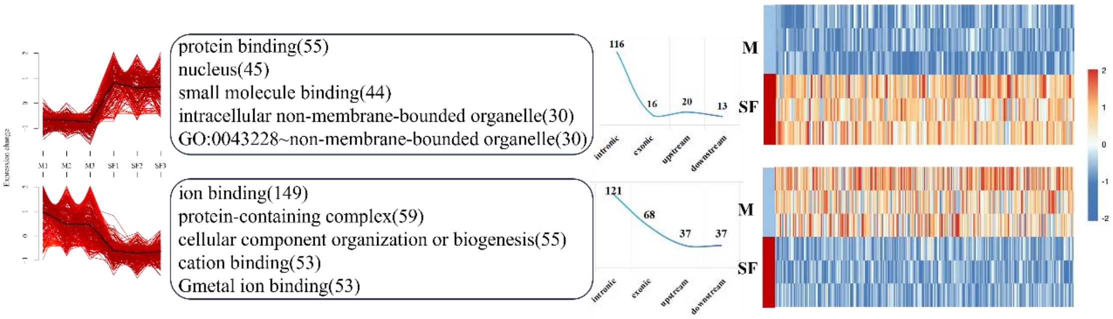

Supplement 8. Analysis of differential lncRNA in M/SF group, Above is cluster 1, below is cluster 2. From left to right are the expression patterns of DECs in cluster 1 and cluster 2 in each sample, DECs functional analysis, DECs location distribution, and DECs expression heat maps in the main pathways.
